# The application of machine learning methods to the prediction of novel ligands for ROR*γ*/ROR*γ*T receptors

**DOI:** 10.1016/j.csbj.2023.10.021

**Published:** 2023-10-29

**Authors:** Rafał A. Bachorz, Joanna Pastwińska, Damian Nowak, Kaja Karaś, Iwona Karwaciak, Marcin Ratajewski

**Affiliations:** Institute of Medical Biology, Polish Academy of Sciences, Lodowa 106, Łódź, 93-232, Poland

**Keywords:** Machine learning, Nuclear receptors, QSAR, Virtual screening, ROR*γ*

## Abstract

In this work, we developed and applied a computational procedure for creating and validating predictive models capable of estimating the biological activity of ligands. The combination of modern machine learning methods, experimental data, and the appropriate setup of molecular descriptors led to a set of well-performing models. We thoroughly inspected both the methodological space and various possibilities for creating a chemical feature space. The resulting models were applied to the virtual screening of the ZINC20 database to identify new, biologically active ligands of ROR*γ* receptors, which are a subfamily of nuclear receptors. Based on the known ligands of ROR*γ*, we selected candidates and calculate their predicted activities with the best-performing models. We chose two candidates that were experimentally verified. One of these candidates was confirmed to induce the biological activity of the ROR*γ* receptors, which we consider proof of the efficacy of the proposed methodology.

## Highlights

• Application of Machine Learning to the virtual screening of nuclear receptors ligands was applied.
• Publicly available ZINC20 data base was screened.
• Biologically active ROR*γ*/ROR*γ*T agonist was found.
• Presented approach is applicable for searching of novel active compounds targeting other proteins.

## Graphical abstract

**Figure:**
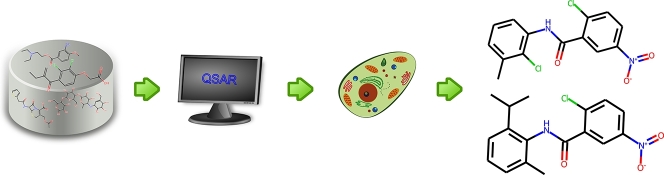

## 1 Introduction

Nuclear receptors are a superfamily of transcription factors whose activity is regulated by the binding of a specific ligand [1]. In the human genome, 48 genes encode nuclear receptors that are implicated in various physiological processes, including development, differentiation, reproduction and homeostasis [1], [2]. One of these nuclear receptor genes, RORC, encodes two isoform proteins named ROR*γ* and ROR*γ*T under alternative promoters [3], [4]. The two isoforms exhibit divergence in their N-terminal regions, spanning 21 amino acids, and they also display distinct tissue distributions and functions. The longer ROR*γ* variant is broadly expressed and is implicated in the regulation of the circadian cycle and metabolism of lipids and glucose [5], [6], [7]. Conversely, the shorter ROR*γ*T isoform is confined to Th17 lymphocytes, where it directly regulates the differentiation of these lymphocytes from naïve CD4+ cells Crome et al. [8]. These Th17 cells, known for their production of interleukin 17 (IL-17A/F), form a critical part of the adaptive immune system, contributing to the defense against pathogens (e.g., *Staphylococcus aureus*, *Bacillus anthracis*, and *Candida albicans*) primarily at mucosal barriers [9], [10], [11]. However, dysregulation leading to the overactivation of Th17 lymphocytes has been linked to tissue damage in certain autoimmune diseases, e.g., multiple sclerosis [12], psoriasis [13], and rheumatoid arthritis [14]. It has been determined that several small molecules that bind the ligand-binding domain (LBD) of ROR*γ*T protect against autoimmune diseases in mouse models, similar to RORC knockdown in mice [15]. Thus, the molecules acting as inverse agonists of ROR*γ*T, inhibiting the activity of this transcription factor, and consequently, diminishing the pathological function of Th17 cells in autoimmune disorders [16], [17], [18] are under intense investigation [19], [20]. Upon binding of an inverse agonist, a conformational change occurs in the ROR*γ*T LBD that displaces the coactivators and recruits corepressors [7], [18], [21]. As a result, ROR*γ*T can no longer bind the ROR response element (RORE) [16] within the regulatory regions of its target genes. However, some inverse agonists may also bind previously unknown allosteric binding pockets, causing a conformational change in the ROR*γ*T LBD, that prevent the binding of coactivators [22]. Within this study, we propose a set of carefully developed QSAR models that can predict the biological activity of small molecules in the context of the ROR*γ* and ROR*γ*T receptors. We employed six commonly applied machine learning methods, i.e.: extreme gradient boosting [23] as implemented in the XGBoost library [24], multilayer perceptron [25], [26] as implemented in the TensorFlow library [27], support vector machines [28], [29], [30], random forest [31], [32], ridge [33], [34] and bagging as implemented in the scikit-learn library [35]. These methods differ significantly in the level of sophistication and thus in overall performance. Within the current study, we created a generic Python application that can be applied for any QSPR/QSAR problem. This application will become freely available in the near future, and an in-depth description will be provided in a follow-up article. The application can easily be applied to cross-check the current study and to pursue further investigation. Due to the level of abstraction, the code can easily be extended to alternative approaches, at the levels of both molecular feature creation and predictive analytics techniques. The presented approach, which is based on cheminformatics and machine learning methods, allows us to perform efficient in silico analysis of the properties of a given molecule with respect to the ligand-binding domain of the ROR*γ* and ROR*γ*T receptors and to determine the probability of their interaction followed by an expected biological effect. This approach provides a tool for much faster prediction of potential ligands and increases the efficiency of drug discovery compared to tedious and time-consuming screening performed in a laboratory. This methodology enables the analysis of potential interactions of various molecules with any proteins acting as receptors. Interestingly, the potential specificity of a given compound toward multiple proteins can also be examined, which may be crucial for the investigation of drug-drug interactions or the prediction of potential side effects of a new drug candidate. The efficiency of the method has been experimentally proven. Developed machine learning models have been applied for the virtual screening of the ZINC20 database, and two unknown ROR*γ* and ROR*γ*T ligands have been identified thus far.

## 2 Materials and methods

### 2.1 In-silico techniques

#### 2.1.1 Experimental data

**Fig. 1:**
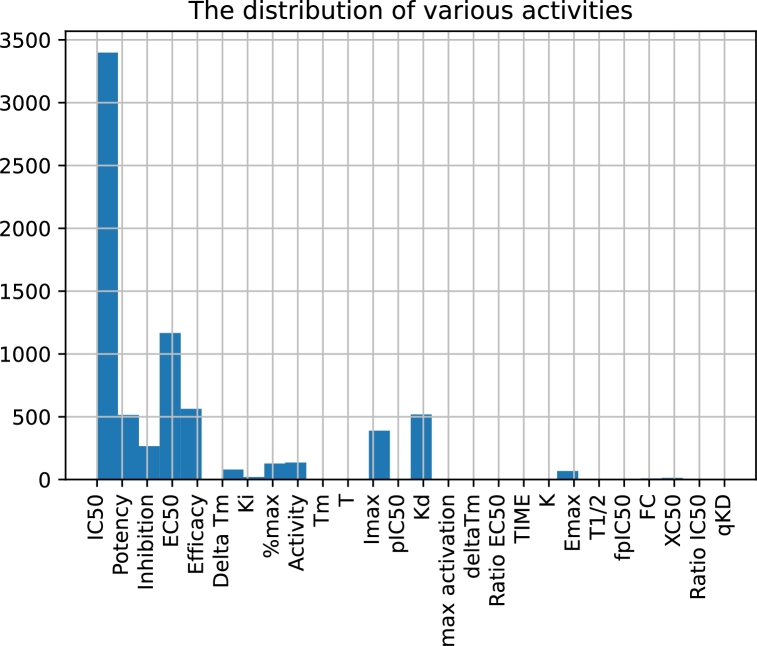
**The distribution of experimental data.** The distribution of different experimental values (activities), expressed as IC50, EC50, efficacy, inhibition, etc. values, for the considered ROR*γ* ligands. The data were obtained from the ChEMBL 3.0 database [36].

The data were extracted from the ChEMBL database, version 3.0 [36]. The ChEMBL database is a manually curated, commonly available repository of bioactive molecules with drug-like properties. Currently, the database contains information on more than 2 million molecules and almost 20 million activity measurements in the context of almost 15 thousand targets. The ChEMBL database is a valuable source of experimental data that provides the fundamental bases for many QSAR investigations. Within the current study, we extracted 7307 activities related to the *Homo sapiens* ROR*γ* receptor from this database. These entries reflect various measured quantities, such as IC50, EC50, efficacy, and inhibition (Fig. 1). After extracting the entries related to the IC50 values and removing the rows that did not contain credible “standard value” and “standard unit” fields, 3246 measurements of activity remained. It is quite common to have more than one activity entry for certain molecules; thus, after aggregating the entries per molecule, the effective number of species was 2472. For the majority of the collected molecules, there is only a single IC50 entry, but in certain cases, the number of measurements reaches 15. Sometimes the information is confusing, i.e., the IC50 values for a given molecule span a broad range. To cope with this problem, we excluded entries for which the standard deviation of available measurements exceeded the threshold value of 100 nM. For the remaining cases with multiple IC50 entries, we implemented two strategies: either we chose the arithmetic mean or the median value. We have also tried not remove the observations; in this case, all activity measurements were taken into account, and the mean values were used as a final aggregate. For the regression models we applied logarithmic scaling, and the target quantity for the regression problems was chosen as the negative of natural logarithm of IC50. For the classification problems, on the other hand, we split the species into “active” and “inactive” groups with the threshold value being arbitrarily chosen at the level of 1000 nM.

#### 2.1.2 Molecular feature creation

Within this study, we have proposed molecular representation in the form of molecular fingerprints and molecular descriptors. The initial form of the molecule is expected to be provided as SMILES code [37]. Within the implemented transformation pipeline, the SMILES codes can be converted to a fingerprint representation, and currently, all the methods of fingerprint calculation available in the RDKit library [38] can be applied. In particular, the RDKit-specific fingerprints inspired by the public descriptions of the daylight fingerprint [39], the atom pair [40], the topological torsion [41], the Morgan fingerprints, [42] and MACCS keys [43] can be applied to create the molecular representation of the considered species. On the other hand, the molecular representation can be further augmented by extending the space with selected molecular descriptors. To achieve this step, we incorporated the capabilities available in the Mordred software [44], which is an open-source library providing an implementation of 1825 various molecular descriptors. Within this study, we selected possible ways of molecular representation creation. There were attempts to apply molecular fingerprints with certain internal parametrization, i.e., the length of bit representation and path size. On the other hand, the classical QED descriptors have been applied to the molecular descriptor space [45]. There were also attempts to combine both molecular descriptors and fingerprint representations. These efforts aimed to determine the best performing molecular representation that could be considered a good practice for these and future efforts. It is worth mentioning that molecular features representation limited to molecular fingerprints only, especially when considering short vector lengths, can be insufficient for QSAR/QSPR studies.

#### 2.1.3 Machine learning training

Each predictive methodology involves a certain number of hyperparameters. The particular choice of hyperparameters has a great impact on the overall predictive model capabilities; therefore, it is crucial to determine them systematically. Here, we have applied the dedicated framework Hyperopt [46], which implements the heuristics of the tree of Parzen estimators [47]. This efficient tool supports searching the space of hyperparameters. Within the current study, we have defined the hyperparameter space as follows, depending on the methodology:

• XGBoost: n_estimators (number of gradient boosting trees), max_depth (maximum tree depth), gamma (minimum loss reduction required to make further split on a leaf), rel_alpha (L1 regularization term), reg_lambda (L2 regularization term), min_child_weight (minimum sum of instance weight needed in a child), colsample_bytree (subsample ratio of columns when constructing each tree), eta (learning rate), subsample (subsample ratio of the training instance)
• Multilayer perceptron: MLP architecture (predefined architectures with a certain depth and number of neurons on each layer), activation function (rectified linear unit, scaled rectified linear unit, exponential linear unit, hyperbolic tangent), batch size, initial learning rate, minimum learning rate, regularization weight, optimizer, dropout rate
• Random forest: n_estimators (number of trees), max_depth (maximum depth of tree)
• Bagging: n_estimators (the number of base estimators in the ensemble), max_features (the number of features drawn to train each base estimator), max_samples (the number of samples drawn to train each base estimator)
• Support vector machines: C (regularization parameter), kernel (linear, polynomial, radial basis function, sigmoid), shrinking (binary flag determining the usage of shrinking strategy)
• Ridge: alpha (constant that multiplies the L2 term, controlling regularization strength), solver type (singular value decomposition, Cholesky decomposition, conjugate gradient solver, least-square, stochastic average gradient, L-BFGS-B algorithm)

Each methodology has been carefully investigated in the context of the regression and classification predictive capabilities. By applying the well-established method of hyperparameter optimization mentioned above, the impact of their random (or default) selection has been largely mitigated. Thus, the final models exploited the majority of their predictive capabilities.

#### 2.1.4 The ZINC20 chemical space search and applicability domain

The final, best performing models were applied to search the ZINC20 database [48]. We selected all known ligands of ROR*γ* receptors as good similarity-search candidates. The search space was spanned by approximately 880 million compounds available in the ZINC20 database. For all these species, we calculated the 1024-bit Morgan fingerprints with the chemfp library [49]. In the next step, we calculated the Tanimoto similarity coefficients between the known ROR*γ* ligands and ZINC20 members. We have assumed here that a potential candidate must be at least 70% similar to a known ROR*γ* ligand. This approach resulted in fewer than 2000 compounds for which the predictive models were applied. To estimate the applicability domain of our models, we designed the chemical space as two T-distributed stochastic neighbors embedding (t-SNE) [50] components calculated on the basis of the 1024-bit molecular fingerprint. Within this space, all the training points and the ZINC20 candidates are shown.

#### 2.1.5 Molecular docking calculation

To qualitatively and quantitatively assess the interaction between the two hit molecules and the ROR*γ* receptor, we performed molecular docking calculations. We applied Smina software [51], and the structure of the receptor was downloaded from the PDB database under the identifier 4WPF [52]. The geometry of the receptor was prepared in Chimera 1.16 software [53] and reflects the agonistic conformation of the ROR*γ* receptor.

### 2.2 Experimental techniques

#### 2.2.1 Reagents

2-Chloro-N-(2-isopropyl-6-methylphenyl)-5-nitrobenzamide (cat. AMB2552929) and 2-chloro-N-(2-chloro-3-methylphenyl)-5-nitrobenzamide (cat. AMB3369132) were purchased from Ambinter (Orléans, France).

#### 2.2.2 Cell culture

The HepG2 cell line (human hepatocellular carcinoma) was purchased from American Type Culture Collection (ATCC, Manassas, VA, United States) and cultured in Dulbecco's modified Eagle's medium (DMEM) supplemented with 10% fetal bovine serum (PAN Biotech GmbH, Aidenbach, Germany). The reporter cell lines: HepG2-ROR*γ* and NHRTOX-HepG2, which were previously described [54], [55] were cultured in DMEM supplemented with 10% fetal bovine serum and 50 μg/ml hygromycin B.

#### 2.2.3 Cell viability

Cell viability after treatment with the analyzed compounds was determined using the CellTiter-Glo® Luminescent Cell Viability Assay from Promega (Fitchburg, WI, USA) according to the manufacturer's instructions. Luminescence was measured using Infinite® 200 PRO (Tecan, Männedorf, Switzerland).

#### 2.2.4 Transient transfection and luciferase measurements

The reporter HepG2-ROR*γ* cells were seeded on 96-well white plates at a density of 15,000 cells per well. Twenty-four hours later, the cells were transfected with control CMV-XL5 or an expression vector encoding the ROR*γ* transcription factor, ROR*α* (Origene, Rockville, MD, USA) or ROR*β* [56] (a kind gift from Dr. R. Schuele, Freiburg, Germany) transcription factors and pCMV6-SEAP (a gift from Dr. S. Schlatter, Zurich) using TurboFect Transfection Reagent (Thermo Fisher Scientific, Waltham, MA, USA). The next day, the cells were treated with the analyzed compound, and after 24 h, they were lysed and subjected to luciferase measurements, which were determined using an Infinite® 200 PRO (Tecan Group, Männedorf, Switzerland). As a substrate for luciferase, D-luciferin from Cayman Chemical Company (Ann Arbor, MI, USA), was selected. The secreted alkaline phosphatase activity was spectrophotometrically determined at 405 nm and was utilized as a control of transfection efficiency.

#### 2.2.5 CD4+ cell isolation and Th17 polarization

Naïve CD4+ lymphocytes were isolated using CD4 M-pluriBeads® anti-hu (pluriSelect Life Science, Leipzig, Germany) from buffy coats of healthy, anonymous donors. Buffy coats were purchased from the Regional Center for Blood Donation and Blood Treatment, Łódź, Poland, as waste material. To obtain fully differentiated Th17 lymphocytes from naïve CD4+ cells, we used a protocol that was previously described by Wilson et al. [57]. Briefly, freshly isolated cells were cultured in RPMI 1640 medium (PAN-Biotech, Aidenbach, Germany) containing 1% human AB serum, 50 ng/mL human IL-1b, 30 ng/mL human IL-6, 10 ng/mL human IL-23, 10 ng/mL human TGF-*β* and beads coated with anti-CD2, anti-CD3, and anti-CD28 antibodies (T-Cell Activation/Expansion Kit from Miltenyi Biotec, Bergisch Gladbach, Germany) for 5 days in the presence of increasing concentrations of the analyzed compounds. Cytokines were purchased from PeproTech (Rocky Hill, NJ, USA).

#### 2.2.6 Gene expression analysis

First, total RNA from cells treated with the analyzed compounds was isolated using TRI Reagent (Molecular Research Center, Cincinnati, OH, USA). Second, 5 μg of RNA was reverse transcribed with a Maxima First Strand cDNA Synthesis Kit for RT-quantitative PCR (Thermo Fisher Scientific, Waltham, MA, USA). Quantitative PCR was conducted using SYBR Green I Master Mix on a Roche LightCycler 480 system (Basel, Switzerland). The following primer pairs were employed in this study: *IL17A*, 5′-AAACAACGATGACTCCTGGG-3′ (forward) and 5′-CTTGTCCTCAGAATTTGGGC-3′ (reverse), as previously described [4]; *IL17F*, 5′-CTTTCTGAGTGAGGCGGC-3′ (forward) and 5′-TGGGAACGGAATTCATGG-3′ (reverse), as previously described [58]; *G6PC*, 5′-TCCATACTGGTGGGTTTTGG-3′ (forward) and 5′-GAGGAAAATGAGCAGCAAGG-3′ (reverse), as described in [54], *CYP3A4*, 5′-TTCAGCAAGAAGAACAAGGACAA-3′ (forward) and 5′-GGTTGAAGAAGTCCTCCTAAGC-3′ (reverse) described in [59]. The PCR conditions were as follows: initial denaturation at 95 ^∘^C for 5 min, followed by 45 cycles of 95 ^∘^C for 10 s, 60 ^∘^C for 10 s, and 72 ^∘^C for 20 s. The expression of the cognate gene was standardized against the expression of the three housekeeping genes *HPRT1*, *HMBS* and *RPL13A*, as previously described by Vandesompele et al. [60].

#### 2.2.7 Statistics

The results obtained from cell lines were analyzed using one-way ANOVA followed by Tukey's post hoc-test. The results from primary human cells were analyzed with Friedman repeated-measures ANOVA on ranks followed by Fisher's LSD method for all pairwise multiple comparisons. A p<0.05 value was considered statistically significant. Statistical analysis was performed using SigmaStat ver. 4.0 (Systat Software Inc., San Jose, CA, USA).

## 3 Results and discussion

### 3.1 In-silico results

For each of the methodologies, we have prepared a family of predictive models solving the classification and regression problem. The former models predict the activity of the molecule expressed as a binary value depending on the IC50 value. When the IC50 value is below the threshold value, here set to be 1000 nM, then the molecule is considered active; otherwise, it is considered inactive. For some methodologies, the returned value is only an active/not-active category; for other methodologies, the logit value is provided. The latter is the result of sigmoid transformation and might be considered as the probability that the molecule will fall into the “active” category. The probability value, in addition to the information about the activity, also delivers information about the confidence of the model. Ideally, if the model worked perfectly on a nonconfusing dataset, the predictions would be 0 and 1 for the nonactive species and active species, respectively. However, in real-world applications, when both the methodology and data are imperfect, the prediction is the value from the (0,1) interval. Therefore, the confidence of the classifier can be modeled as the difference between a certain prediction and a value of 0.5, which reflects the model uncertainty.

Within each methodology, we considered multiple variants of the predictive models. All results are collected in Table 1, Table 2, Table 3, Table 4, Table 5, Table 6 for the random forest, support vector machine, XGBoost, ridge, multilayer perceptron and bagging classifiers, respectively. The results for the models that solve the regression problems are shown in Table 7, Table 8, Table 9, Table 10, Table 11, Table 12, in the same order of methodologies. The meaning of a particular variant of the method is encoded in the column “Predictive model variant”. The encoding appearing in this column contains 5 comma-separated components with the following meaning:

• Methodology acronyms: “RF”, “SVM”, “XGBoost”, “Ridge”, “MLP” and “Bagging” for random forest, support vector machines, XGBoost, ridge, multilayer perceptron and bagging
• Molecular features components: “MD”, “FP”, and “FPMD”. In the case of an “MD” entry (molecular descriptors), the feature space is spanned by:
  **–** Eight molecular descriptors: molecular weight, octanol/water partition coefficient, number of hydrogen bond donors, number of hydrogen bond acceptors, molecular polar surface area, number of rotatable bonds, number of aromatic rings and molar refractivity,
  **–** Three drug-likeness indices: QED [45], Lipinski “rule-of-five” [61], [62], and Ghose filter. [63]
  If “FP” is present (Fingerprint), then the circular Morgan fingerprint [42] with a length of 1024 is calculated. If both entries are present, i.e., “FPMD”, then the molecular feature space was spanned both by the molecular descriptors and fingerprints.
• Principal component analysis (PCA) features. In the case of molecular fingerprints (“FP”), we have provided a PCA-transformed version with a differing number of principal components. This approach results in four additional variants of the predictive models accounting for 128, 256, 512 and 1024 principal components denoted as “PCA128”, “PCA256”, “PCA512” and “PCA1024”, respectively. The PCA components were sorted according to the variance gain; thus, by extending the PCA space, we incorporated additional components with decreasing variance gain. The base variant, i.e., the variant that avoids the PCA transformation is noted as “NoPCA”.
• Raw data aggregation strategy. Raw biological data are ambiguous for some compounds. Thus, multiple IC50 entries are generated for the same molecule. However, for the final predictive model creation, a single value is expected. For some compounds, the experimental value spread was significant. In extreme cases, the same compound could be concluded to be active based on certain experimental values, i.e., the IC50 values were well below 1000 nM, and inactive based on experimental values, i.e., the IC50 values were well above 1000 nM. Therefore, we introduced the standard deviation as a measure of experimental value spread and a threshold value of 100 nM above which the compound was disregarded. The remaining multiple entries were aggregated as the mean or median. Three possibilities exist:
  **–** “median100”: the threshold value of 100 nM and the median as an aggregation function,
  **–** “mean100”: the threshold value of 100 nM and the arithmetic mean as an aggregation function,
  **–** “meanNoLim”: no standard deviation threshold and arithmetic mean as an aggregation function.
• The dataset used for calculating the quality measures. Here we have two options:
  **–** “test”: the test set of 10% of the data extracted prior to the training (unseen data),
  **–** “train”: the training set (provided in the supplementary material).

**Table 1:** The results obtained for the classifiers based on the random forest method. The presented results are for the randomly chosen hold-out set of the compounds.

Predictive model variant	precision	recall	accuracy	f1	rocauc	ap	mcc
RF,MD,NoPCA,median100,test	0.8688	0.9456	0.8700	0.9055	0.8346	0.8573	0.7046
RF,MD,NoPCA,mean100,test	0.8688	0.9456	0.8700	0.9055	0.8346	0.8573	0.7046
RF,MD,NoPCA,meanNoLim,test	0.8667	0.9235	0.8387	0.8942	0.7617	0.8568	0.5616
RF,FP,NoPCA,median100,test	0.8931	0.9660	0.9013	0.9281	0.8712	0.8851	0.7778
RF,FP,NoPCA,mean100,test	0.8931	0.9660	0.9013	0.9281	0.8712	0.8851	0.7778
RF,FP,NoPCA,meanNoLim,test	0.8889	0.9180	0.8548	0.9032	0.7975	0.8765	0.6145
RF,FPMD,PCA128,median100,test	0.8563	0.9728	0.8744	0.9108	0.8285	0.8509	0.7181
RF,FPMD,PCA128,mean100,test	0.8563	0.9728	0.8744	0.9108	0.8285	0.8509	0.7181
RF,FPMD,PCA128,meanNoLim,test	0.8776	0.9399	0.8589	0.9077	0.7853	0.8692	0.6165
RF,FPMD,PCA256,median100,test	0.8606	0.9660	0.8744	0.9103	0.8317	0.8538	0.7167
RF,FPMD,PCA256,mean100,test	0.8606	0.9660	0.8744	0.9103	0.8317	0.8538	0.7167
RF,FPMD,PCA256,meanNoLim,test	0.8719	0.9672	0.8710	0.9171	0.7836	0.8675	0.6473
RF,FPMD,PCA512,median100,test	0.8650	0.9592	0.8744	0.9097	0.8349	0.8566	0.7158
RF,FPMD,PCA512,mean100,test	0.8650	0.9592	0.8744	0.9097	0.8349	0.8566	0.7158
RF,FPMD,PCA512,meanNoLim,test	0.8627	0.9617	0.8589	0.9096	0.7655	0.8580	0.6112
RF,FPMD,PCA1024,median100,test	0.8913	0.8367	0.8251	0.8632	0.8197	0.8534	0.6240
RF,FPMD,PCA1024,mean100,test	0.8913	0.8367	0.8251	0.8632	0.8197	0.8534	0.6240
RF,FPMD,PCA1024,meanNoLim,test	0.8824	0.9016	0.8387	0.8919	0.7816	0.8681	0.5751

**Table 2:** The results obtained for the classifiers based on the support vector machines method. These results are for the randomly chosen hold-out set of the species.

Predictive model variant	precision	recall	accuracy	f1	rocauc	ap	mcc
SVM,MD,Scaling,NoPCA,median100,test	0.8589	0.9524	0.8655	0.9032	0.8249	0.8494	0.6945
SVM,MD,Scaling,NoPCA,mean100,test	0.8589	0.9524	0.8655	0.9032	0.8249	0.8494	0.6945
SVM,MD,Scaling,NoPCA,meanNoLim,test	0.8537	0.9563	0.8468	0.9021	0.7474	0.8486	0.5747
SVM,FP,NoPCA,median100,test	0.9020	0.9388	0.8924	0.9200	0.8707	0.8871	0.7572
SVM,FP,NoPCA,mean100,test	0.9020	0.9388	0.8924	0.9200	0.8707	0.8871	0.7572
SVM,FP,NoPCA,meanNoLim,test	0.8953	0.9344	0.8710	0.9144	0.8134	0.8850	0.6551
SVM,FPMD,Scaling,PCA128,median100,test	0.9085	0.9456	0.9013	0.9267	0.8807	0.8949	0.7776
SVM,FPMD,Scaling,PCA128,mean100,test	0.9085	0.9456	0.9013	0.9267	0.8807	0.8949	0.7776
SVM,FPMD,Scaling,PCA128,meanNoLim,test	0.8832	0.9508	0.8710	0.9158	0.7985	0.8761	0.6496
SVM,FPMD,Scaling,PCA256,median100,test	0.9150	0.9524	0.9103	0.9333	0.8907	0.9028	0.7980
SVM,FPMD,Scaling,PCA256,mean100,test	0.9150	0.9524	0.9103	0.9333	0.8907	0.9028	0.7980
SVM,FPMD,Scaling,PCA256,meanNoLim,test	0.8866	0.9399	0.8669	0.9125	0.8007	0.8777	0.6409
SVM,FPMD,Scaling,PCA512,median100,test	0.9150	0.9524	0.9103	0.9333	0.8907	0.9028	0.7980
SVM,FPMD,Scaling,PCA512,mean100,test	0.9150	0.9524	0.9103	0.9333	0.8907	0.9028	0.7980
SVM,FPMD,Scaling,PCA512,meanNoLim,test	0.8947	0.9290	0.8669	0.9115	0.8106	0.8836	0.6455
SVM,FPMD,Scaling,PCA1024,median100,test	0.9211	0.9524	0.9148	0.9365	0.8972	0.9086	0.8084
SVM,FPMD,Scaling,PCA1024,mean100,test	0.9211	0.9524	0.9148	0.9365	0.8972	0.9086	0.8084
SVM,FPMD,Scaling,PCA1024,meanNoLim,test	0.8821	0.9399	0.8629	0.9101	0.7930	0.8734	0.6287

**Table 3:** The results obtained for the classifiers based on the XGBoost method. These results are for the randomly chosen hold-out set of the species.

Predictive model variant	precision	recall	accuracy	f1	rocauc	ap	mcc
XGBoost,MD,NoPCA,median100,test	0.8562	0.8912	0.8296	0.8733	0.8008	0.8348	0.6145
XGBoost,MD,NoPCA,mean100,test	0.8562	0.8912	0.8296	0.8733	0.8008	0.8348	0.6145
XGBoost,MD,NoPCA,meanNoLim,test	0.8601	0.9071	0.8226	0.8830	0.7459	0.8488	0.5205
XGBoost,FP,NoPCA,median100,test	0.9267	0.9456	0.9148	0.9360	0.9004	0.9121	0.8089
XGBoost,FP,NoPCA,mean100,test	0.9267	0.9456	0.9148	0.9360	0.9004	0.9121	0.8089
XGBoost,FP,NoPCA,meanNoLim,test	0.9011	0.8962	0.8508	0.8986	0.8096	0.8842	0.6162
XGBoost,FPMD,PCA128,median100,test	0.8812	0.9592	0.8879	0.9186	0.8546	0.8722	0.7466
XGBoost,FPMD,PCA128,mean100,test	0.8812	0.9592	0.8879	0.9186	0.8546	0.8722	0.7466
XGBoost,FPMD,PCA128,meanNoLim0,test	0.8838	0.9563	0.8750	0.9186	0.8012	0.8775	0.6604
XGBoost,FPMD,PCA256,median100,test	0.8696	0.9524	0.8744	0.9091	0.8380	0.8595	0.7152
XGBoost,FPMD,PCA256,mean100,test	0.8696	0.9524	0.8744	0.9091	0.8380	0.8595	0.7152
XGBoost,FPMD,PCA256,meanNoLim0,test	0.8844	0.9617	0.8790	0.9215	0.8040	0.8788	0.6714
XGBoost,FPMD,PCA512,median100,test	0.8519	0.9388	0.8520	0.8932	0.8115	0.8401	0.6624
XGBoost,FPMD,PCA512,mean100,test	0.8519	0.9388	0.8520	0.8932	0.8115	0.8401	0.6624
XGBoost,FPMD,PCA512,meanNoLim0,test	0.8693	0.9454	0.8548	0.9058	0.7727	0.8622	0.6023
XGBoost,FPMD,PCA1024,median100,test	0.8662	0.9252	0.8565	0.8947	0.8244	0.8507	0.6737
XGBoost,FPMD,PCA1024,mean100,test	0.8662	0.9252	0.8565	0.8947	0.8244	0.8507	0.6737
XGBoost,FPMD,PCA1024,meanNoLim,test	0.8698	0.9126	0.8347	0.8907	0.7640	0.8583	0.5553

**Table 4:** The results obtained for the classifiers based on the ridge method. These results are for the randomly chosen hold-out set of the species.

Predictive model variant	precision	recall	accuracy	f1	rocauc	ap	mcc
Ridge,MD,Scaling,NoPCA,median100,test	0.8193	0.9252	0.8161	0.8690	0.7652	0.8073	0.5764
Ridge,MD,Scaling,NoPCA,mean100,test	0.8193	0.9252	0.8161	0.8690	0.7652	0.8073	0.5764
Ridge,MD,Scaling,NoPCA,meanNoLim,test	0.8301	0.9344	0.8105	0.8792	0.6980	0.8241	0.4643
Ridge,FP,NoPCA,median100,test	0.8750	0.9048	0.8520	0.8896	0.8274	0.8544	0.6662
Ridge,FP,NoPCA,mean100,test	0.8750	0.9048	0.8520	0.8896	0.8274	0.8544	0.6662
Ridge,FP,NoPCA,meanNoLim,test	0.8989	0.9235	0.8669	0.9111	0.8156	0.8866	0.6482
Ridge,FPMD,Scaling,PCA128,median100,test	0.8671	0.9320	0.8610	0.8984	0.8278	0.8529	0.6838
Ridge,FPMD,Scaling,PCA128,mean100,test	0.8671	0.9320	0.8610	0.8984	0.8278	0.8529	0.6838
Ridge,FPMD,Scaling,PCA128,meanNoLim,test	0.8912	0.9399	0.8710	0.9149	0.8084	0.8820	0.6529
Ridge,FPMD,Scaling,PCA256,median100,test	0.8671	0.9320	0.8610	0.8984	0.8278	0.8529	0.6838
Ridge,FPMD,Scaling,PCA256,mean100,test	0.8671	0.9320	0.8610	0.8984	0.8278	0.8529	0.6838
Ridge,FPMD,Scaling,PCA256,meanNoLim,test	0.8895	0.9235	0.8589	0.9062	0.8002	0.8779	0.6238
Ridge,FPMD,Scaling,PCA512,median100,test	0.8947	0.9252	0.8789	0.9097	0.8573	0.8771	0.7271
Ridge,FPMD,Scaling,PCA512,mean100,test	0.8947	0.9252	0.8789	0.9097	0.8573	0.8771	0.7271
Ridge,FPMD,Scaling,PCA512,meanNoLim,test	0.8942	0.9235	0.8629	0.9086	0.8079	0.8822	0.6360
Ridge,FPMD,Scaling,PCA1024,median100,test	0.8816	0.9116	0.8610	0.8963	0.8374	0.8619	0.6865
Ridge,FPMD,Scaling,PCA1024,mean100,test	0.8816	0.9116	0.8610	0.8963	0.8374	0.8619	0.6865
Ridge,FPMD,Scaling,PCA1024,meanNoLim,test	0.8930	0.9126	0.8548	0.9027	0.8024	0.8795	0.6177

**Table 5:** The results obtained for the classifiers based on the multilayer perceptron architecture. These results are for the randomly chosen hold-out set of the species.

Predictive model variant	precision	recall	accuracy	f1	rocauc	ap	mcc
MLP,MD,Scaling,NoPCA,median100,test	0.8516	0.8980	0.8296	0.8742	0.7977	0.8320	0.6129
MLP,MD,Scaling,NoPCA,mean100,test	0.8509	0.9320	0.8475	0.8896	0.8081	0.8379	0.6519
MLP,MD,Scaling,NoPCA,meanNoLim,test	0.8469	0.9071	0.8105	0.8760	0.7228	0.8368	0.4814
MLP,FPMD,Scaling,PCA128,median100,test	0.9110	0.9048	0.8789	0.9078	0.8669	0.8870	0.7314
MLP,FPMD,Scaling,PCA128,mean100,test	0.9020	0.9388	0.8924	0.9200	0.8707	0.8871	0.7572
MLP,FPMD,Scaling,PCA128,meanNoLim,test	0.8817	0.8962	0.8347	0.8889	0.7789	0.8668	0.5664
MLP,FPMD,Scaling,PCA256,median100,test	0.8993	0.9116	0.8744	0.9054	0.8571	0.8781	0.7189
MLP,FPMD,Scaling,PCA256,mean100,test	0.8993	0.9116	0.8744	0.9054	0.8571	0.8781	0.7189
MLP,FPMD,Scaling,PCA256,meanNoLim,test	0.9111	0.8962	0.8589	0.9036	0.8250	0.8931	0.6408
MLP,FPMD,Scaling,PCA512,median100,test	0.9034	0.8912	0.8655	0.8973	0.8535	0.8769	0.7026
MLP,FPMD,Scaling,PCA512,mean100,test	0.8944	0.8639	0.8430	0.8789	0.8333	0.8624	0.6569
MLP,FPMD,Scaling,PCA512,meanNoLim,test	0.8776	0.9399	0.8589	0.9077	0.7853	0.8692	0.6165
MLP,FPMD,Scaling,PCA1024,median100,test	0.8889	0.9252	0.8744	0.9067	0.8507	0.8717	0.7165
MLP,FPMD,Scaling,PCA1024,mean100,test	0.8993	0.9116	0.8744	0.9054	0.8571	0.8781	0.7189
MLP,FPMD,Scaling,PCA1024,meanNoLim,test	0.9011	0.8962	0.8508	0.8986	0.8096	0.8842	0.6162

**Table 6:** The results obtained for the classifiers based on the bagging method. These results are for the randomly chosen hold-out set of the species.

Predictive model variant	precision	recall	accuracy	f1	rocauc	ap	mcc
Bagging,MD,NoPCA,median100,test	0.8473	0.7551	0.7489	0.7986	0.7460	0.8013	0.4736
Bagging,MD,NoPCA,mean100,test	0.8473	0.7551	0.7489	0.7986	0.7460	0.8013	0.4736
Bagging,MD,NoPCA,meanNoLim,test	0.8824	0.7377	0.7339	0.8036	0.7304	0.8445	0.4168
Bagging,FP,NoPCA,median100,test	0.9037	0.8299	0.8296	0.8652	0.8294	0.8621	0.6389
Bagging,FP,NoPCA,mean100,test	0.9037	0.8299	0.8296	0.8652	0.8294	0.8621	0.6389
Bagging,FP,NoPCA,meanNoLim,test	0.9085	0.7596	0.7661	0.8274	0.7721	0.8675	0.4923
Bagging,FPMD,PCA128,median100,test	0.8657	0.7891	0.7803	0.8256	0.7761	0.8221	0.5345
Bagging,FPMD,PCA128,mean100,test	0.8657	0.7891	0.7803	0.8256	0.7761	0.8221	0.5345
Bagging,FPMD,PCA128,meanNoLim,test	0.8741	0.6448	0.6694	0.7421	0.6916	0.8257	0.3384
Bagging,FPMD,PCA256,median100,test	0.8346	0.7211	0.7220	0.7737	0.7224	0.7857	0.4258
Bagging,FPMD,PCA256,mean100,test	0.8346	0.7211	0.7220	0.7737	0.7224	0.7857	0.4258
Bagging,FPMD,PCA256,meanNoLim,test	0.8881	0.6940	0.7097	0.7791	0.7239	0.8421	0.3986
Bagging,FPMD,PCA512,median100,test	0.8581	0.8639	0.8161	0.8610	0.7938	0.8310	0.5895
Bagging,FPMD,PCA512,mean100,test	0.8581	0.8639	0.8161	0.8610	0.7938	0.8310	0.5895
Bagging,FPMD,PCA512,meanNoLim,test	0.8646	0.9071	0.8266	0.8853	0.7536	0.8528	0.5334

**Table 7:** The results obtained for the regressors based on the random forest method. The Presented results are for the randomly chosen hold-out set of the species.

Predictive model variant	r2	mse	msle	mae	mape
RF,MD,NoPCA,median100,test	0.7312	0.3929	0.0081	0.4511	0.0770
RF,MD,NoPCA,mean100,test	0.6575	0.4961	0.0101	0.5176	0.0879
RF,MD,NoPCA,meanNoLim,test	0.5485	0.4807	0.0088	0.5342	0.0847
RF,FP,NoPCA,median100,test	0.7423	0.3767	0.0075	0.4605	0.0776
RF,FP,NoPCA,mean100,test	0.7289	0.3927	0.0078	0.4651	0.0785
RF,FP,NoPCA,meanNoLim,test	0.6195	0.4051	0.0075	0.4669	0.0744
RF,FPMD,PCA128,median100,test	0.6822	0.4646	0.0096	0.5033	0.0863
RF,FPMD,PCA128,mean100,test	0.6737	0.4726	0.0099	0.5087	0.0878
RF,FPMD,PCA128,meanNoLim,test	0.5884	0.4383	0.0082	0.5043	0.0810
RF,FPMD,PCA256,median100,test	0.6628	0.4930	0.0103	0.5212	0.0898
RF,FPMD,PCA256,mean100,test	0.6618	0.4898	0.0102	0.5219	0.0900
RF,FPMD,PCA256,meanNoLim,test	0.5798	0.4474	0.0085	0.5094	0.0821
RF,FPMD,PCA512,median100,test	0.6613	0.4951	0.0103	0.5294	0.0911
RF,FPMD,PCA512,mean100,test	0.6680	0.4809	0.0102	0.5190	0.0900
RF,FPMD,PCA512,meanNoLim,test	0.5623	0.4661	0.0089	0.5225	0.0845
RF,FPMD,PCA1024,median100,test	0.6356	0.5327	0.0113	0.5654	0.0978
RF,FPMD,PCA1024,mean100,test	0.6479	0.5100	0.0109	0.5558	0.0964
RF,FPMD,PCA1024,meanNoLim,test	0.5355	0.4946	0.0093	0.5476	0.0879

**Table 8:** The results obtained for the regressors based on the support vector machines method. The presented results are for the randomly chosen hold-out set of the species.

Predictive model variant	r2	mse	msle	mae	mape
SVM,MD,Scaling,NoPCA,median100,test	0.6198	0.5558	0.0116	0.5652	0.0969
SVM,MD,Scaling,NoPCA,mean100,test	0.6209	0.5491	0.0115	0.5596	0.0961
SVM,MD,Scaling,NoPCA,meanNoLim,test	0.5190	0.5122	0.0095	0.5746	0.0915
SVM,FP,NoPCA,median100,test	0.7549	0.3583	0.0070	0.4558	0.0760
SVM,FP,NoPCA,mean100,test	0.7579	0.3507	0.0069	0.4492	0.0751
SVM,FP,NoPCA,meanNoLim,test	0.5960	0.4301	0.0078	0.4584	0.0728
SVM,FPMD,Scaling,PCA128,median100,test	0.7866	0.3119	0.0060	0.4203	0.0685
SVM,FPMD,Scaling,PCA128,mean100,test	0.7865	0.3093	0.0060	0.4162	0.0680
SVM,FPMD,Scaling,PCA128,meanNoLim,test	0.6455	0.3774	0.0069	0.4285	0.0676
SVM,FPMD,Scaling,PCA256,median100,test	0.7730	0.3319	0.0064	0.4300	0.0703
SVM,FPMD,Scaling,PCA256,mean100,test	0.7760	0.3244	0.0063	0.4231	0.0694
SVM,FPMD,Scaling,PCA256,meanNoLim,test	0.6489	0.3738	0.0069	0.4241	0.0675
SVM,FPMD,Scaling,PCA512,median100,test	0.7635	0.3458	0.0068	0.4415	0.0733
SVM,FPMD,Scaling,PCA512,mean100,test	0.7653	0.3400	0.0067	0.4367	0.0727
SVM,FPMD,Scaling,PCA512,meanNoLim,test	0.6500	0.3727	0.0068	0.4246	0.0677
SVM,FPMD,Scaling,PCA1024,median100,test	0.7593	0.3518	0.0070	0.4469	0.0750
SVM,FPMD,Scaling,PCA1024,mean100,test	0.7598	0.3479	0.0070	0.4431	0.0745
SVM,FPMD,Scaling,PCA1024,meanNoLim,test	0.6487	0.3741	0.0068	0.4287	0.0682

**Table 9:** The results obtained for the regressors based on the XGBoost method. The presented results are for the randomly chosen hold-out set of the species.

Predictive model variant	r2	mse	msle	mae	mape
XGBoost,MD,NoPCA,median100,test	0.6520	0.5087	0.0105	0.5271	0.0902
XGBoost,MD,NoPCA,mean100,test	0.6407	0.5205	0.0109	0.5270	0.0905
XGBoost,MD,NoPCA,meanNoLim,test	0.5250	0.5058	0.0092	0.5467	0.0866
XGBoost,FP,NoPCA,median100,test	0.7307	0.3936	0.0076	0.4821	0.0795
XGBoost,FP,NoPCA,mean100,test	0.7429	0.3724	0.0072	0.4663	0.0767
XGBoost,FP,NoPCA,meanNoLim,test	0.5772	0.4502	0.0082	0.4928	0.0776
XGBoost,FPMD,PCA128,median100,test	0.6784	0.4701	0.0097	0.5238	0.0898
XGBoost,FPMD,PCA128,mean100,test	0.7136	0.4148	0.0086	0.4845	0.0831
XGBoost,FPMD,PCA128,meanNoLim,test	0.5705	0.4574	0.0086	0.5080	0.0817
XGBoost,FPMD,PCA256,median100,test	0.6802	0.4675	0.0097	0.5204	0.0890
XGBoost,FPMD,PCA256,mean100,test	0.6897	0.4495	0.0094	0.5060	0.0871
XGBoost,FPMD,PCA256,meanNoLim,test	0.5998	0.4261	0.0080	0.4897	0.0789
XGBoost,FPMD,PCA512,median100,test	0.6746	0.4757	0.0098	0.5032	0.0856
XGBoost,FPMD,PCA512,mean100,test	0.6816	0.4613	0.0095	0.5095	0.0869
XGBoost,FPMD,PCA512,meanNoLim,test	0.5919	0.4345	0.0082	0.5036	0.0810
XGBoost,FPMD,PCA1024,median100,test	0.6527	0.5077	0.0103	0.5462	0.0921
XGBoost,FPMD,PCA1024,mean100,test	0.6823	0.4602	0.0094	0.5100	0.0862
XGBoost,FPMD,PCA1024,meanNoLim,test	0.5789	0.4484	0.0082	0.5164	0.0821

**Table 10:** The results obtained for the regressors based on the ridge method. The presented results are for the randomly chosen hold-out set of the species.

Predictive model variant	r2	mse	msle	mae	mape
Ridge,FPMD,Scaling,NoPCA,median100,test	0.6839	0.4621	0.0094	0.5342	0.0891
Ridge,FPMD,Scaling,NoPCA,mean100,test	0.6840	0.4577	0.0093	0.5292	0.0884
Ridge,FPMD,Scaling,NoPCA,meanNoLim,test	0.5415	0.4882	0.0091	0.5335	0.0848
Ridge,FP,NoPCA,median100,test	0.6673	0.4864	0.0100	0.5454	0.0909
Ridge,FP,NoPCA,mean100,test	0.6682	0.4806	0.0099	0.5392	0.0900
Ridge,FP,NoPCA,meanNoLim,test	0.5199	0.5112	0.0095	0.5409	0.0860
Ridge,FPMD,Scaling,PCA128,median100,test	0.7079	0.4269	0.0085	0.5224	0.0866
Ridge,FPMD,Scaling,PCA128,mean100,test	0.7110	0.4187	0.0084	0.5175	0.0860
Ridge,FPMD,Scaling,PCA128,meanNoLim,test	0.4960	0.5366	0.0099	0.5705	0.0906
Ridge,FPMD,Scaling,PCA256,median100,test	0.7244	0.4029	0.0080	0.5143	0.0850
Ridge,FPMD,Scaling,PCA256,mean100,test	0.7268	0.3958	0.0079	0.5096	0.0844
Ridge,FPMD,Scaling,PCA256,meanNoLim,test	0.5563	0.4725	0.0088	0.5443	0.0865
Ridge,FPMD,Scaling,PCA512,median100,test	0.6996	0.4392	0.0087	0.5156	0.0852
Ridge,FPMD,Scaling,PCA512,mean100,test	0.7001	0.4344	0.0086	0.5110	0.0846
Ridge,FPMD,Scaling,PCA512,meanNoLim,test	0.5609	0.4676	0.0086	0.5287	0.0835
Ridge,FPMD,Scaling,PCA1024,median100,test	0.6833	0.4630	0.0094	0.5347	0.0892
Ridge,FPMD,Scaling,PCA1024,mean100,test	0.6836	0.4583	0.0093	0.5295	0.0884
Ridge,FPMD,Scaling,PCA1024,meanNoLim,test	0.5407	0.4890	0.0091	0.5339	0.0848

**Table 11:** The results obtained for the regressors based on the multilayer perceptron architecture. The presented results are for the randomly chosen hold-out set of the species.

Predictive model variant	r2	mse	msle	mae	mape
MLP,MD,Scaling,NoPCA,median100,test	0.6311	0.5393	0.0107	0.5629	0.0938
MLP,MD,Scaling,NoPCA,mean100,test	0.6473	0.5109	0.0097	0.5497	0.0888
MLP,MD,Scaling,NoPCA,meanNoLim,test	0.5110	0.5207	0.0095	0.5575	0.0875
MLP,FPMD,Scaling,PCA128,median100,test	0.7395	0.3809	0.0074	0.4849	0.0801
MLP,FPMD,Scaling,PCA128,mean100,test	0.7467	0.3669	0.0074	0.4839	0.0811
MLP,FPMD,Scaling,PCA128,meanNoLim,test	0.5626	0.4657	0.0084	0.5212	0.0810
MLP,FPMD,Scaling,PCA256,median100,test	0.7031	0.4340	0.0082	0.5216	0.0847
MLP,FPMD,Scaling,PCA256,mean100,test	0.7259	0.3970	0.0076	0.4881	0.0804
MLP,FPMD,Scaling,PCA256,meanNoLim,test	0.5637	0.4645	0.0086	0.5201	0.0821
MLP,FPMD,Scaling,PCA512,median100,test	0.7416	0.3777	0.0070	0.4845	0.0773
MLP,FPMD,Scaling,PCA512,mean100,test	0.7324	0.3877	0.0075	0.4709	0.0781
MLP,FPMD,Scaling,PCA512,meanNoLim,test	0.5456	0.4838	0.0087	0.5347	0.0824
MLP,FPMD,Scaling,PCA1024,median100,test	0.7287	0.3966	0.0076	0.5032	0.0831
MLP,FPMD,Scaling,PCA1024,mean100,test	0.7175	0.4092	0.0079	0.5025	0.0822
MLP,FPMD,Scaling,PCA1024,meanNoLim,test	0.5190	0.5121	0.0094	0.5519	0.0866

**Table 12:** The results obtained for the regressors based on the bagging method. The presented results are for the randomly chosen hold-out set of the species.

Predictive model variant	r2	mse	msle	mae	mape
Bagging,MD,Scaling,NoPCA,median100,test	0.6442	0.5202	0.0107	0.5348	0.0909
Bagging,MD,Scaling,NoPCA,mean100,test	0.6343	0.5298	0.0109	0.5384	0.0918
Bagging,MD,Scaling,NoPCA,meanNoLim,test	0.5431	0.4865	0.0090	0.5450	0.0867
Bagging,FP,NoPCA,median100,test	0.7296	0.3953	0.0078	0.4664	0.0783
Bagging,FP,NoPCA,mean100,test	0.7328	0.3870	0.0076	0.4621	0.0774
Bagging,FP,NoPCA,meanNoLim,test	0.6037	0.4219	0.0078	0.4714	0.0754
Bagging,FPMD,PCA128,median100,test	0.6657	0.4887	0.0102	0.5256	0.0905
Bagging,FPMD,PCA128,mean100,test	0.6796	0.4641	0.0098	0.5084	0.0880
Bagging,FPMD,PCA128,meanNoLim,test	0.6015	0.4244	0.0080	0.4952	0.0796
Bagging,FPMD,PCA256,median100,test	0.6618	0.4943	0.0103	0.5289	0.0913
Bagging,FPMD,PCA256,mean100,test	0.6555	0.4990	0.0103	0.5232	0.0899
Bagging,FPMD,PCA256,meanNoLim,test	0.5706	0.4572	0.0086	0.5213	0.0840
Bagging,FPMD,PCA512,median100,test	0.6431	0.5217	0.0109	0.5344	0.0924
Bagging,FPMD,PCA512,mean100,test	0.6516	0.5046	0.0107	0.5355	0.0930
Bagging,FPMD,PCA512,meanNoLim,test	0.5594	0.4691	0.0089	0.5283	0.0854

Each row in the tables is related to a carefully determined predictive model with optimized hyperparameters according to a certain strategy. The hyperparameter space was defined according to the nature of the methodology; the section Machine Learning training provides details. The optimization of the hyperparameter space was carried out with the hyperopt module [46], the number of evaluations of the goal function was set to 200, and the optimization goal function was chosen to maximize the precision value or to minimize the mean square error (MSE) for the classifiers and regressors, respectively. The goal function was calculated in the 5-fold cross-validation scheme to avoid the impact of a particular split on the resulting quality measures. Prior to the training, 10% of the data were extracted and later applied for a final quality estimation. For each predictive model, exactly the same splitting strategy was applied; therefore, the quality measures presented in the tables refer to the same molecules.

#### 3.1.1 Classifiers

**Fig. 2:**
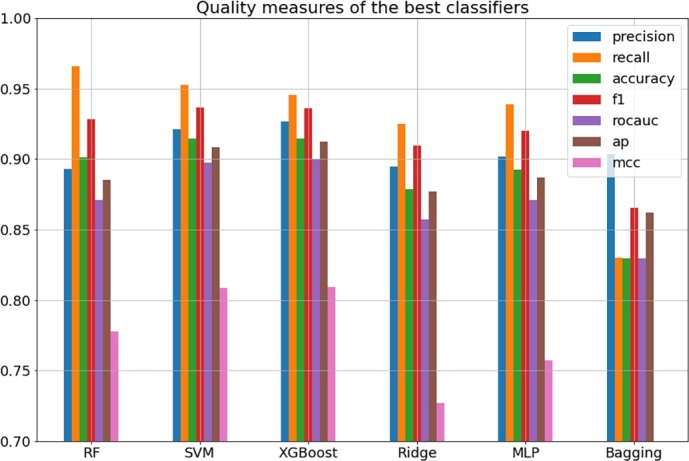
**The best classifiers.** Bar plot showing the best classification predictive models. The quality of the models is expressed as the basic binary classifier quality measure, i.e. precision, recall, accuracy, f1-score, rocauc, average precision and Matthew coefficient.

**Fig. 3:**
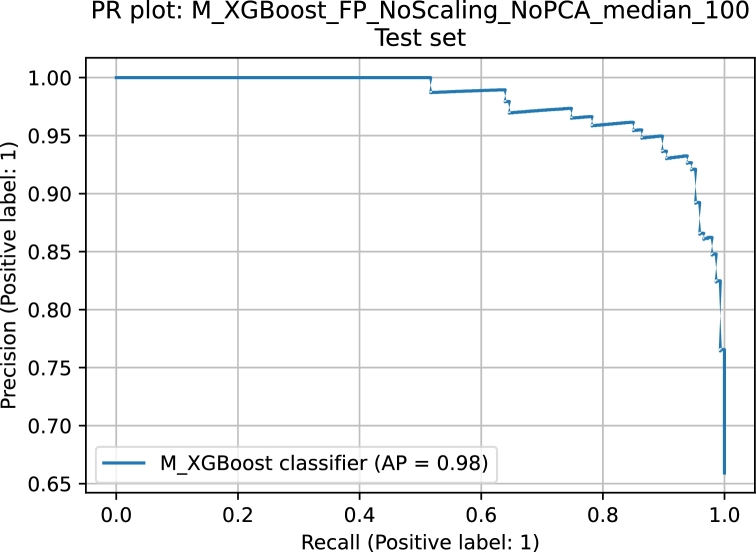
**Precision vs. recall plot of the best performing classifier.** The test was performed with the dataset that was excluded prior to training thus, it reflects the objective measure of the predictive capabilities of the model.

The quality of each classifier is summarized by seven quality measures: precision, recall, accuracy, F1-score, area under the receiver operator curve (ROCAUC), average precision (AP) and Matthew correlation coefficient. All detailed results are presented in Table 1, Table 2, Table 3, Table 4, Table 5, Table 6. Each methodology was considered in 18 different scenarios, as explained above. The exception here is the MLP method, for which we did not consider the “NoPCA” variants of the cases involving the molecular fingerprints due to poor performance of the MLP architectures with sparse data. The predictive capabilities differ depending on the methodology itself and the particular variant of this methodology. Table 13 summarizes the best classifiers from each methodology; these results are shown graphically in Fig. 2. Our ultimate goal was to create efficient models capable of predicting biologically active compounds. The efficiency here simultaneously means high precision and recall, since we want to avoid false-positives and false-negatives. Among all considered classifiers, SVM and XGBoost have exceptional performance. Both are characterized by high values of precision and recall, but from the perspective of ROCAUC (area under receiver operating curve) and AP (average precision), the XGBoost model seems to perform slightly better. The precision-recall plot of this model is presented in Fig. 3. The analogous plots of remaining considered models as well as the confusion matrices and ROC curves are provided in the supplementary information.

**Table 13:** The best-performing classifiers.

Predictive model variant	precision	recall	accuracy	f1	rocauc	ap	mcc
RF,FP,NoPCA,median100,test	0.8931	0.9660	0.9013	0.9281	0.8712	0.8851	0.7778
SVM,FPMD,Scaling,PCA1024,mean100,test	0.9211	0.9524	0.9148	0.9365	0.8972	0.9086	0.8084
XGBoost,FP,NoPCA,median100,test	0.9267	0.9456	0.9148	0.9360	0.9004	0.9121	0.8089
Ridge,FPMD,Scaling,PCA512,median100,test	0.8947	0.9252	0.8789	0.9097	0.8573	0.8771	0.7271
MLP,FPMD,Scaling,PCA128,mean100,test	0.9020	0.9388	0.8924	0.9200	0.8707	0.8871	0.7572
Bagging,FP,NoPCA,median100,test	0.9037	0.8299	0.8296	0.8652	0.8294	0.8621	0.6389

#### 3.1.2 Regressors

**Fig. 4:**
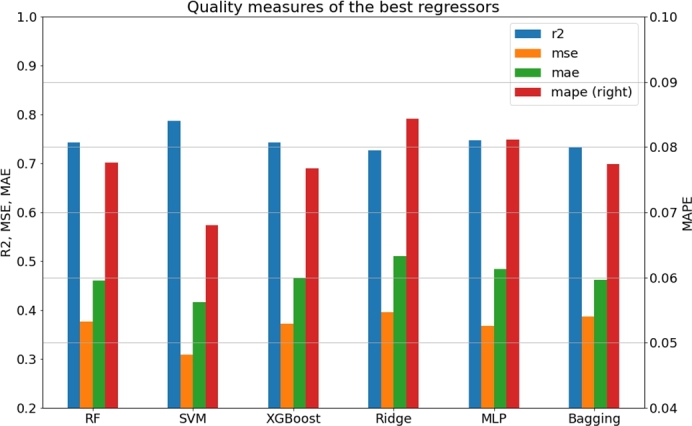
**The best classifiers.** Bar plot showing the best regression predictive models. The quality of the models is expressed in terms of the *R*^2^ coefficient (R2), mean squared error (MSE), mean absolute error (MAE) and mean absolute percentage error (MAPE). The latter is reflected on the right vertical axis.

**Fig. 5:**
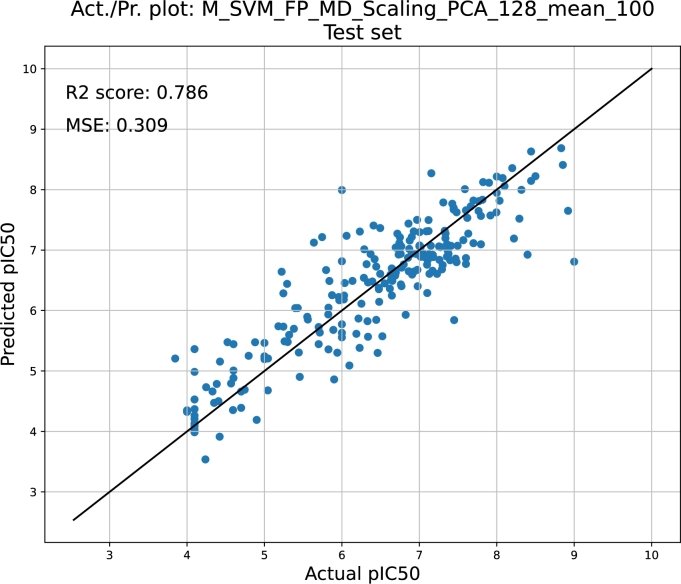
**Plot showing the prediction of the pIC50 values vs. the experimental values.** The test was performed with the dataset that was excluded prior to training thus, it reflects the objective measure of the predictive capabilities of the model.

The quality estimation of the regression predictive models was established based on the R^2^ coefficient, mean squared error (MSE), mean squared logarithmic error (MSLE), mean absolute error (MAE) and mean average percentage error (MAPE). The results are presented in Table 7, Table 8, Table 9, Table 10, Table 11, Table 12. Similar to classifiers, the quality of the regressors depends on the methodology and chosen strategy. The best performing models are summarized in Table 14 and Fig. 5. It can be clearly concluded that the best model was obtained with the SVM method and is characterized by the lowest MSE value (below 0.3 log units) and the highest R^2^ coefficient. In Fig. 4, we present the plot of the predicted pIC50 versus the actual values. The analogous plots of the remaining regression predictive models obtained for both the training and test sets are provided in the supplementary information.

**Table 14:** The best-performing regressors.

Predictive model variant	r2	mse	msle	mae	mape
RF,FP,NoPCA,median100,test	0.7423	0.3767	0.0075	0.4605	0.0776
SVM,FPMD,Scaling,PCA128,mean100,test	0.7865	0.3093	0.0060	0.4162	0.0680
XGBoost,FP,NoPCA,mean100,test	0.7429	0.3724	0.0072	0.4663	0.0767
Ridge,FPMD,Scaling,PCA256,mean100,test	0.7268	0.3958	0.0079	0.5096	0.0844
MLP,FPMD,Scaling,PCA128,mean100,test	0.7467	0.3669	0.0074	0.4839	0.0811
Bagging,FP,NoPCA,mean100,test	0.7328	0.3870	0.0076	0.4621	0.0774

#### 3.1.3 ZINC20 search

**Fig. 6:**
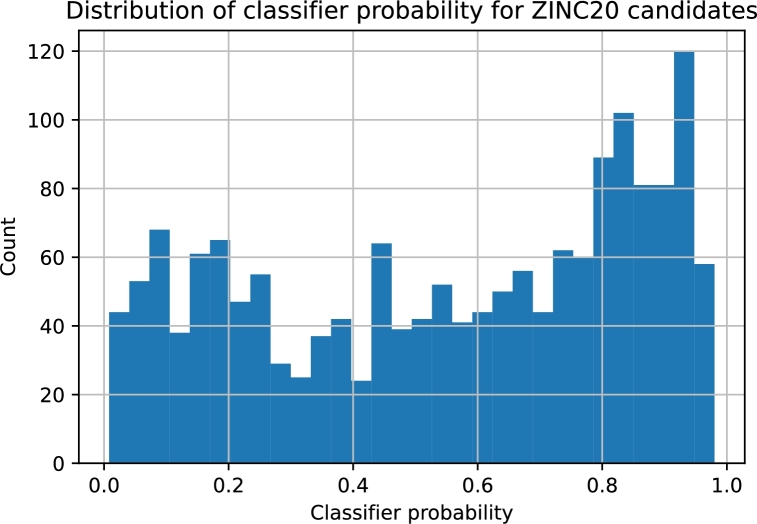
**Distribution of classifier probabilities of the ZINC20 candidates.** Histogram of the XGBoost classifier predictions of the ZINC20 candidate species. The distribution is approximately uniform with a slight bias toward the high probabilities.

**Fig. 7:**
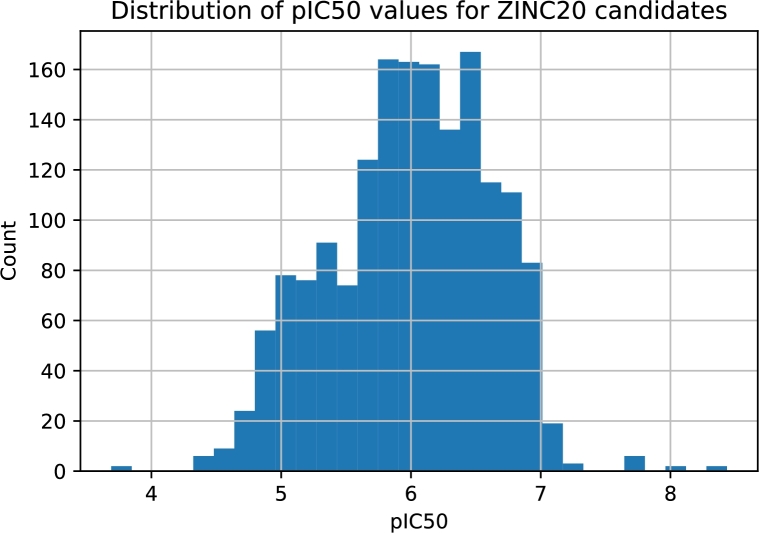
**Distribution of predicted pIC50 values of ZINC20 candidates.** The histogram of predicted pIC50 values based on the SVM regression predictive model. The shape of the distribution resembles a Gaussian bell-like shape with the center ca. at pIC50=6 with rapidly vanishing tails.

**Fig. 8:**
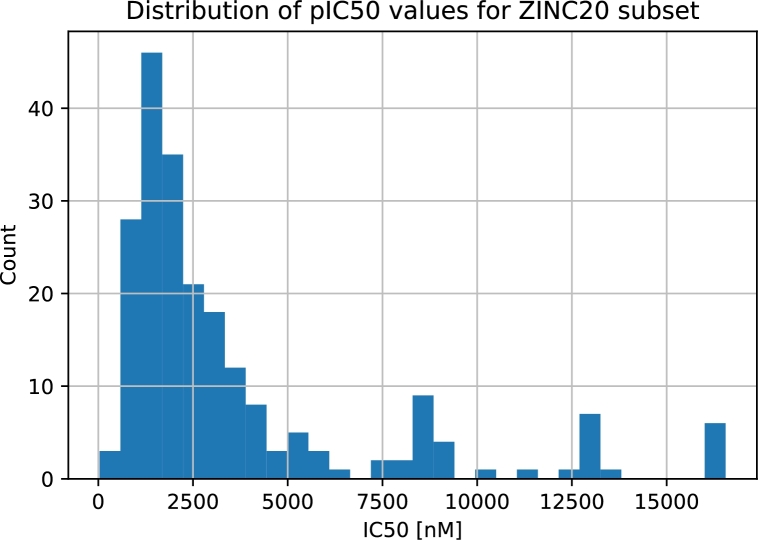
**The pIC50 values of the ZINC20 subset.** The distribution of predicted IC50 values of ZINC20 candidates for which the classifier probability was above 90%. The majority of the most promising candidates are focused in the area below 5000 nM. This part of the distribution resembles skewed Gaussian distribution.

**Fig. 9:**
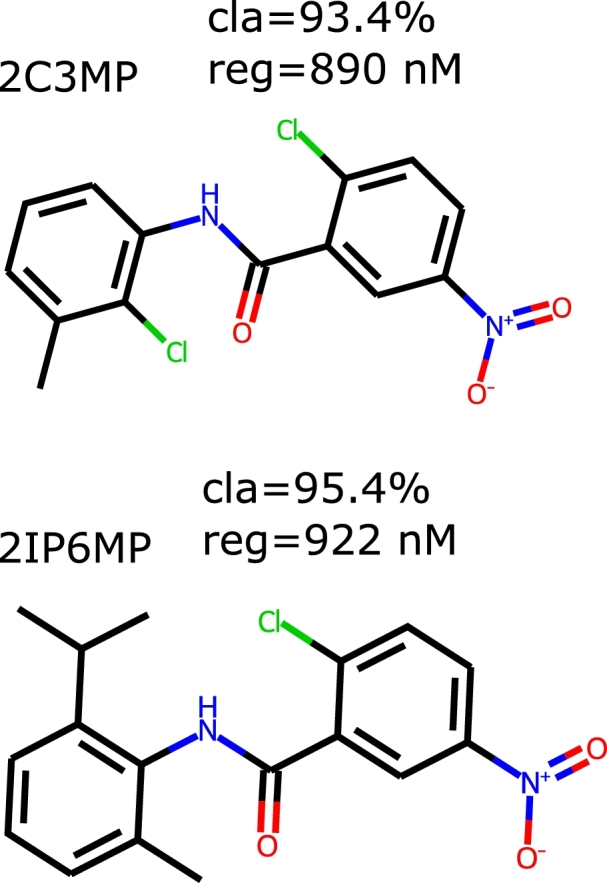
**The chosen compounds.** The chemical structures of compounds that were chosen for further experimental verification. Together with the chemical structures, predictions of biological activity are provided.

**Fig. 10:**
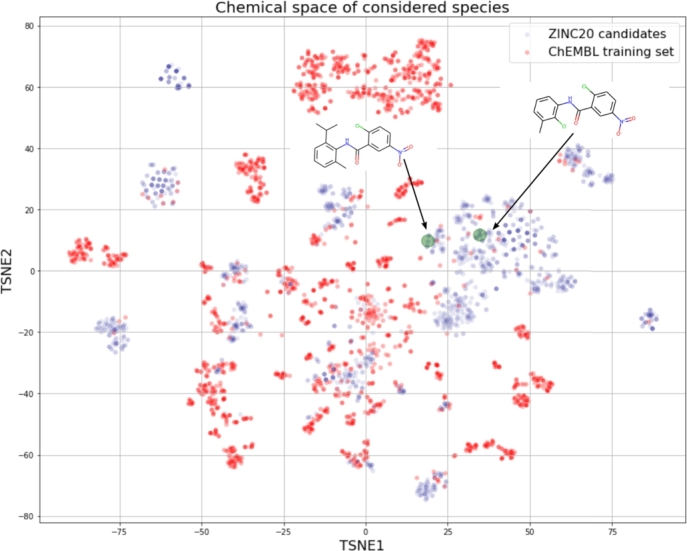
**The area of applicability of the models.** The red points reflect the molecules contained in the training set, and the violet points are related to the ZINC20 candidates. The green circles illustrate the region of chemical space with two candidates chosen for further experimental study.

All known ROR*γ* ligands were compared with the ZINC20 database, and the most similar species were retrieved. ZINC20 virtual screening led to 1673 compounds. For each of these compounds, we applied the leading predictive models, i.e., those for which the best quality measures were achieved (the section In-silico results provides details). Among these molecules, 220 were characterized by a classifier probability above 0.9. The majority of the predicted IC50 values for the ZINC20 candidates varied in the range of 35 nM-16.5 μM. There were three predictions above 50 μM, but for clarity, they were removed. Interestingly, the distribution of the classifier probabilities resembles a uniform distribution, whereas the predicted pIC50 values form a Gaussian-like distribution (Fig. 6 and Fig. 7). We focused our attention on species for which the classifier probabilities exceeded 0.9. Within this set, 80% of the regressor predictions are below 5.0 μM; however, the remaining 20% exceed this value (Fig. 8). This finding clearly shows an inconsistency between the classifier prediction and the regressor prediction. From these molecules, based on the predictions, commercial availability and price, we selected two molecules for further experimental validation (Fig. 9). Both compounds are characterized by a high probability of biological activity and a relatively low IC50 value. These molecules were also carefully inspected against the area of applicability of the QSAR models. Based on the proposed protocol (the section The ZINC20 chemical space search and applicability domain provides technical details), we created a visualization of the training set and these molecules within the chemical space (Fig. 10). For both, there are training representatives in the nearest neighborhood; therefore, we concluded that they are within the area of applicability of the developed models. Interestingly, among the molecules resulting from the ZINC20 database screening, we identified one species, TMP920, for which experimental data were available [16], even though it was not yet contained in the ChEMBL database. As a result, this molecule was not present in the training data utilized in this study. However, the regressor predicted an IC50 value of 37 nM, whereas the measured value was 30 nM [16]. This excellent agreement is an additional presumption supporting the results and reinforcing the methodology proposed here. The entire list of 1673 compounds considered within the virtual screening discussed here, together with the predicted biological activities, is available in the Supplementary Information.

#### 3.1.4 Molecular docking

**Fig. 11:**
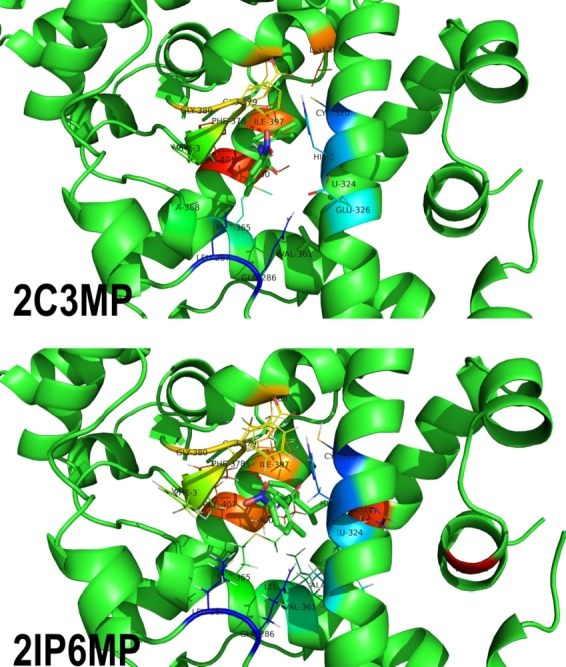
**The final poses of chosen compounds.** Both compounds were docked to the same ROR*γ* receptor structure (PDB ID: 4WPF), reflecting the agonistic conformation of this protein.

The two experimentally verified compounds were docked to the agonistic conformation of the ROR*γ* receptor (see section 2.1.5 for further details related to the applied methodology). The binding energy of both of them amounts to -8.6 kcal/mol. The final poses are presented in Fig. 11. In the case of both ligands, one hydrogen bond was formed between the oxygen of the nitro group of the ligand and the hydroxyl group of glutamic acid (GLU-379). The remaining ligand-protein interactions are of nonpolar nature and are formed deeper in the hydrophobic part of a binding pocket. They are mainly related to the aromatic interaction between the phenyl ring of the ligand and the phenyl rings of surrounding amino acids, mainly PHE-387, PHE-388, and HIS-323. Based on these results, it is difficult to explain noticeable differences in the biological activity of 2C3MP and 2IP6MP compounds. Most likely, subtle differences in the protein-ligand aromatic interactions in the hydrophobic cavity lead to different strengths of the HIS-479 - TYR-502 hydrogen bond, which is a key factor determining the conformation of the receptor and thus its biological activity [52].

### 3.2 Experimental results

**Fig. 12:**
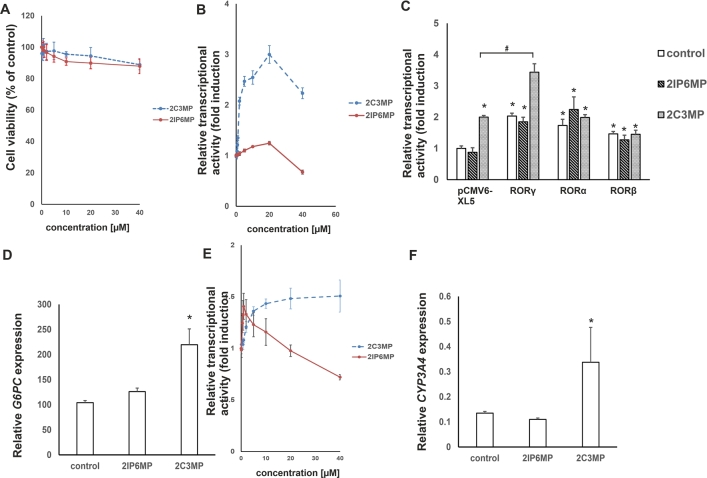
**The biological effects of 2-chloro-N-(2-isopropyl-6-methylphenyl)-5-nitrobenzamid (2IP6MP) and 2-chloro-N-(2-chloro-3-methylphenyl)-5-nitrobenzamide (2C3MP) on ROR***γ***-expressing HepG2 cells.** A) Results of the viability of HepG2 cells treated with increasing concentrations of 2IP6MP and 2C3MP for 24 h; mean ± SD, n = 3. B) The effect of increasing concentrations of 2IP6MP and 2C3MP on ROR*γ*-HepG2 reporter cell line. Mean ± SD, n = 5. C). 2C3MP (20 μM) but not 2IP6MP (20 μM) potentiates the effect of human ROR*γ* in the ROR*γ*-HepG2 reporter cell line. Mean ± SD, n = 5; ⁎ denotes statistically significant difference at p<0.05 compare to the control treatment; # denotes a statistically significant difference at p<0.05 compare to the pCMV6-XL5 and 2C3MP treated cells. D) The effect of the 2C3MP (20 μM) and 2IP6MP (20 μM) on the expression of ROR*γ*-dependent gene, G6PC in human HepG2 cell line, determined by quantitative RT-PCR; mean ± SD, n = 4, * denotes statistically significant difference at p<0.05 compare to the control treatment. E) The effect of increasing concentrations of 2IP6MP and 2C3MP on the nhrtox-HepG2 reporter cell line. Mean ± SD, n = 5. F) Effect of 2C3MP (40 μM) and 2IP6MP (1 μM) on the expression of the PXR-dependent gene CYP3A4 in the human HepG2 cell line, as determined by quantitative RT–PCR; mean ± SD and n = 3; * denotes a statistically significant difference at p<0.05 compared to the control treatment.

In the first stage of experimental validation of the compounds identified in the computer-based environment, we focused on the assessment of their cytotoxicity against HepG2 cells that exclusively express the ROR*γ* isoform [4]. Interestingly, we were able to confirm that these compounds did not exert significant toxicity after 24 h at concentrations up to 40 μM (Fig. 12A). We decided to use our reporter cell line to show whether both considered compounds can influence ROR*γ*-dependent transcription. As shown in Fig. 12B, 2-chloro-N-(2-chloro-3-methylphenyl)-5-nitrobenzamide (2C3MP) induced the activity of the reporter in a concentration-dependent manner, while 2-chloro-N-(2-isopropyl-6-methylphenyl)-5-nitrobenzamide (2IP6MP) did not. Furthermore, the ectopic overexpression of ROR*γ* but not ROR*α* and ROR*β* resulted in a significantly higher response to 2-chloro-N-(2-chloro-3-methylphenyl)-5-nitrobenzamide (2C3MP) of the reporter and confirmed the involvement of this protein in the biological action of the considered compound (Fig. 12C). Interestingly, we did not observe similar results for 2-chloro-N-(2-isopropyl-6-methylphenyl)-5-nitrobenzamid (2IP6MP) (Fig. 12C). Next, we decided to check whether 2-chloro-N-(2-isopropyl-6-methylphenyl)-5-nitrobenzamide and 2-chloro-N-(2-chloro-3-methylphenyl)-5-nitrobenzamide influence the expression of the ROR*γ*-dependent gene *G6PC* [64]. Quantitative PCR was performed, which indicated that 2-chloro-N-(2-chloro-3-methylphenyl)-5-nitrobenzamide induced (2.1-fold induction) the expression of G6PC mRNA, while 2-chloro-N-(2-isopropyl-6-methylphenyl)-5-nitrobenzamide was not effective (only 1.2-fold induction of G6PC expression) (Fig. 12D). Activation of the PXR nuclear receptor, which subsequently triggers the activation of the CYP3A4 cytochrome, can result in drug-drug interactions [65], [66]. This effect has been associated with the reduced efficacy observed in clinical trials of certain ROR*γ*T modulators [19]. Previous research has demonstrated that benzamides activate PXR [67]. This motivated us to investigate whether the identified compounds induce PXR-dependent transcription. To achieve this, we employed the nhrtox-hepg2 cell line, as previously described [55]. As illustrated in Fig. 12, 2IP6MP caused a 1.4-fold increase in reporter activity at a concentration of 1 μM, while the second compound, 2C3MP, induced a 1.5-fold increase at 40 μM. Real-time RT-PCR analysis revealed that at these concentrations, 2IP6MP showed no significant effect, whereas 2C3MP led to a 2.5-fold increase in CYP3A4 mRNA levels (Fig. 12).

**Fig. 13:**
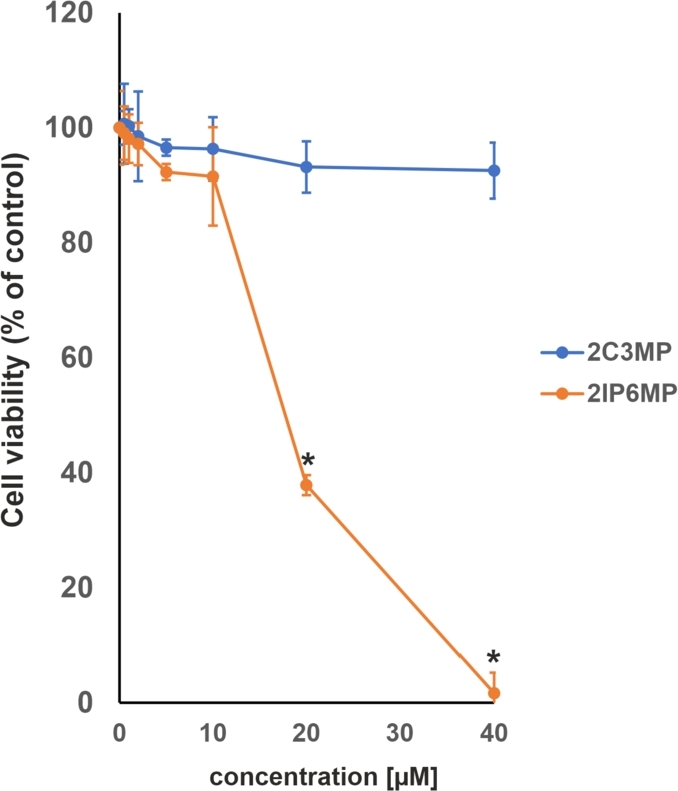
**Results of the cell viability of human Th17 cells treated with 2IP6MP and 2C3MP.** Human CD4+ cells were isolated from buffy coats of healthy, anonymous donors and treated with increasing concentrations of 2IP6MP and 2C3MP upon Th17 for 5 days. After that time cell viability was determined using a CellTiter-Glo Luminescent Cell Viability Assay. The results are shown as the mean ± SD, n = 3 (three different donors); * denotes a statistically significant difference at p<0.05.

**Fig. 14:**
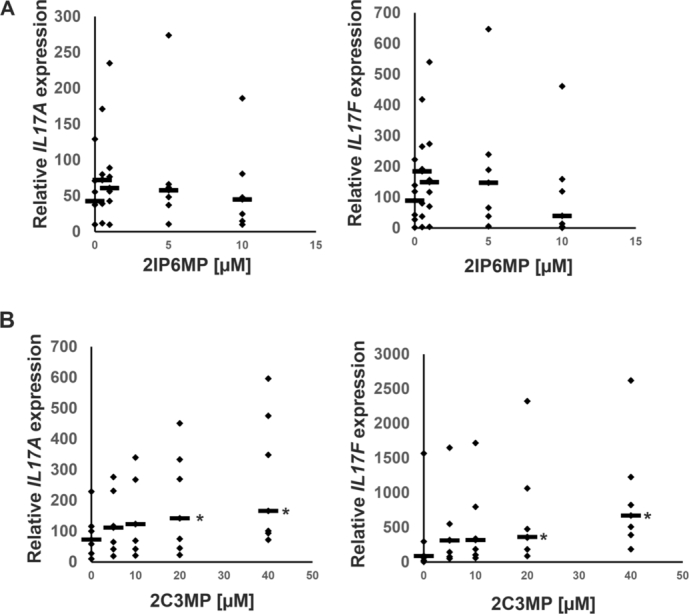
**The impact of chosen compounds on gene expression.** The effect of 2IP6MP (A) and 2C3MP (B) on the expression of the ROR*γ*T-dependent genes: IL17A and IL17F in human Th17 cells. Primary CD4+ cells were isolated from buffy coats of healthy, anonymous donors and treated with increasing concentrations of 2IP6MP and 2C3MP upon Th17 for 5 days. The expression of cognate genes was determined by quantitative RT-PCR and the results are shown as dot plots with median values (bars) from seven independent donors (n=7); ⁎ indicates a statistically significant difference at p<0.05.

Next, we decided to perform a similar analysis using primary human Th17 cells that express a shorter ROR*γ*T isoform that regulates the expression of *IL17A* and *IL17F* [8]. Cytotoxicity assays indicated that 2-chloro-N-(2-chloro-3-methylphenyl)-5-nitrobenzamide up to 10 μM and 2-chloro-N-(2-chloro-3-methylphenyl)-5-nitrobenzamide up to 40 μM are not cytotoxic to these cells (Fig. 13). Analysis of the expression of signature genes that are regulated in Th17 cells by ROR*γ*T revealed that 2-chloro-N-(2-chloro-3-methylphenyl)-5-nitrobenzamide induced the expression of *IL17A* and *IL17F* in a concentration-dependent manner (Fig. 14B), while the second considered structure was not active (Fig. 14A), confirming the results obtained from HepG2 cells.

### 3.3 Discussion

The identification of new ROR*γ*/ROR*γ*T ligands may have many potential applications in the future that seem to be overlooked by the world of science today. Due to the participation of the ROR*γ* isoform in the regulation of G6PC, agonists can be employed, e.g., in the treatment of nonalcoholic fatty liver disease to improve the functions of liver cells [68]. In particular, the compound that we identified shows very low cytotoxicity (Fig. 12A). Th17 lymphocytes, which are implicated in cancers [69], [70], may promote the tumor microenvironment in some types of cancers, e.g., breast cancer [71]. However, Th17 lymphocytes can also participate in tumor eradication by producing large amounts of cytokines as well as stimulating infiltration of the microenvironment by dendritic cells, cytotoxic T lymphocytes or natural killer cells [72], [73], [74]. Thus, 2-chloro-N-(2-chloro-3-methylphenyl)-5-nitrobenzamide, because of its low cytotoxicity, may potentially be employed in some anticancer regimens to increase not only the antitumor properties of Th17 lymphocytes but also the efficacy of the immune system. In recent years, Th17 lymphocytes have also attracted attention as an interesting component for adoptive cell therapy [75], [76], [77], [78], in which cells are obtained from the patient, multiplied outside the body, and then reintroduced in large numbers into the bloodstream to destroy the tumor. The use of ROR*γ*T ligands (including the newly identified 2-chloro-N-(2-chloro-3-methylphenyl)-5-nitrobenzamide) has the potential to promote ex vivo cell proliferation and to improve Th17 cell phenotypes. Recently, Lai et al. showed that the improvement in the generation of Th17 lymphocytes is beneficial in adoptive cell therapy in a murine model [79]. Nevertheless, owing to potential interactions with the PXR/CYP3A4 signaling pathway, the prospective utilization of the identified compound or other benzamide derivatives should be approached with caution and necessitates further investigation. It is not possible to rule out the possibility that our findings may limit the potential use of such compounds in vivo.

Successful experimental validation has proven that the developed predictive models are correct. The combination of open source resources, including the experimental data retrieved from the ChEMBL database as well as all applied libraries of the Python ecosystem, led to an efficient and robust computational protocol in the form of a Python program. This tool covers 6 commonly available machine learning methodologies and a set of auxiliary functionalities supporting data retrieval, cleaning and final processing. The workflows are managed by dedicated configuration files that allow for large-scale calculations.

In summary, our cheminformatics approaches that used machine learning techniques allowed us to identify a new ligand of ROR*γ*/ROR*γ*T receptors but with already known nitrobenzamide, [80] or a general benzamide scaffold [81], [82], [83], [84], [85]. It should be noted that our approach was aimed at identifying ligands for ROR*γ*/ROR*γ*T receptors, not specifically agonists or inverse agonists, which are sometimes distinguished by only one functional group [86] and can be utilized for any protein target whose activity is regulated by small molecular weight ligands.

### 3.4 Conclusions and future effort

Within the current study, we have introduced an efficient methodology that allows the creation of well-performing QSAR models based on commonly available machine learning methods. We have carefully examined 6 methodologies, and within each of them, we considered selected alternative approaches oriented on the experimental raw data processing and the way the chemical features were created. Among all considered predictive models, we selected the best performing classifier and regressor, which were later applied to the preselected compounds. The selection was oriented on the ZINC20 database containing ca. 880 million commercially available compounds. First, based on the molecular similarity between known ROR*γ* ligands and ZINC20 species, we selected ca. 1700 candidates, which were at least 70% similar to biologically active ROR*γ* ligands in terms of the Tanimoto coefficient based on Morgan fingerprints. Second, these species were inspected with the best performing regressor and classifier, which led to a shortened list of candidates. Last, we have taken into account the commercial aspects and selected 2 species for final experimental validation, which showed that, despite the high similarity, only one of the two selected compounds demonstrated activity toward the ROR*γ*/ROR*γ*T receptors.

The percentage of success, in this case, is very high and indicates that the use of our methodology based on machine learning in conjunction with the experiment significantly improves the search for substances with the expected activity against various proteins.

As a potential avenue for future efforts, we observe the development of generative approaches combined with multicriteria optimization techniques for molecular optimization. The two species, selected and experimentally verified within the current study, might be considered good starting structures for further research. In addition to biological activity, we also intend to take into account other objectives, such as solubility, toxicity, and organic synthesis difficulty. As a result, we will obtain the Pareto-optimal solutions, and similar to the current study, some of these solutions will be experimentally verified.

## 4 Abbreviations

CYP3A4, cytochrome P450 3A4; G6PC, glucose-6-phosphatase catalytic subunit; HPRT1, hypoxanthine phosphoribosyltransferase 1; HMBS, hydroxymethylbilane synthase; PXR, pregnane X receptor; RORC, RAR related orphan receptor C; ROR*γ*, nuclear receptor ROR-gamma isoform 1; ROR*γ*T, nuclear receptor ROR-gamma isoform 2; RORE, ROR response element; RPL13A, ribosomal protein L13a; Th17, T helper 17 cell.

## Funding

This work was supported by the 10.13039/501100004281National Science Centre project number: 2019/33/B/NZ7/00795.

## CRediT authorship contribution statement

**Rafał A. Bachorz:** Conceptualization, Methodology, Software, Validation, Formal analysis, Investigation, Writing. **Joanna Pastwińska:** Methodology, Investigation. **Damian Nowak:** Investigation. **Kaja Karaś:** Investigation. **Iwona Karwaciak:** Investigation. **Marcin Ratajewski:** Conceptualization, Methodology, Validation, Formal analysis, Investigation, Writing, Funding acquisition.

## Declaration of Competing Interest

The authors declare that they have no known competing financial interests or personal relationships that could have appeared to influence the work reported in this paper.

## Data Availability

The data of this study are available from the corresponding author upon reasonable request.

## Appendix A Supplementary material

Supplementary material The plots reflecting the quality of all considered regression and classification predictive models. Supplementary material

Supplementary information The list of all 1673 compounds resulting from similarity search of ZINC20 database. Both the classifier and the regressor predictions of biological activity of best performing models are provided. Supplementary information
