# Supplementary material for: The application of machine learning methods to the prediction of novel ligands for RORγ/RORγT receptors
Source: Comput Struct Biotechnol J. 2023 Oct 29;21:5491–505. doi: 10.1016/j.csbj.2023.10.021 (PMC10663739; doi:10.1016/j.csbj.2023.10.021)
Supplement: Supplementary material — The plots reflecting the quality of all considered regression and classification predictive models. [file mmc1.pdf]

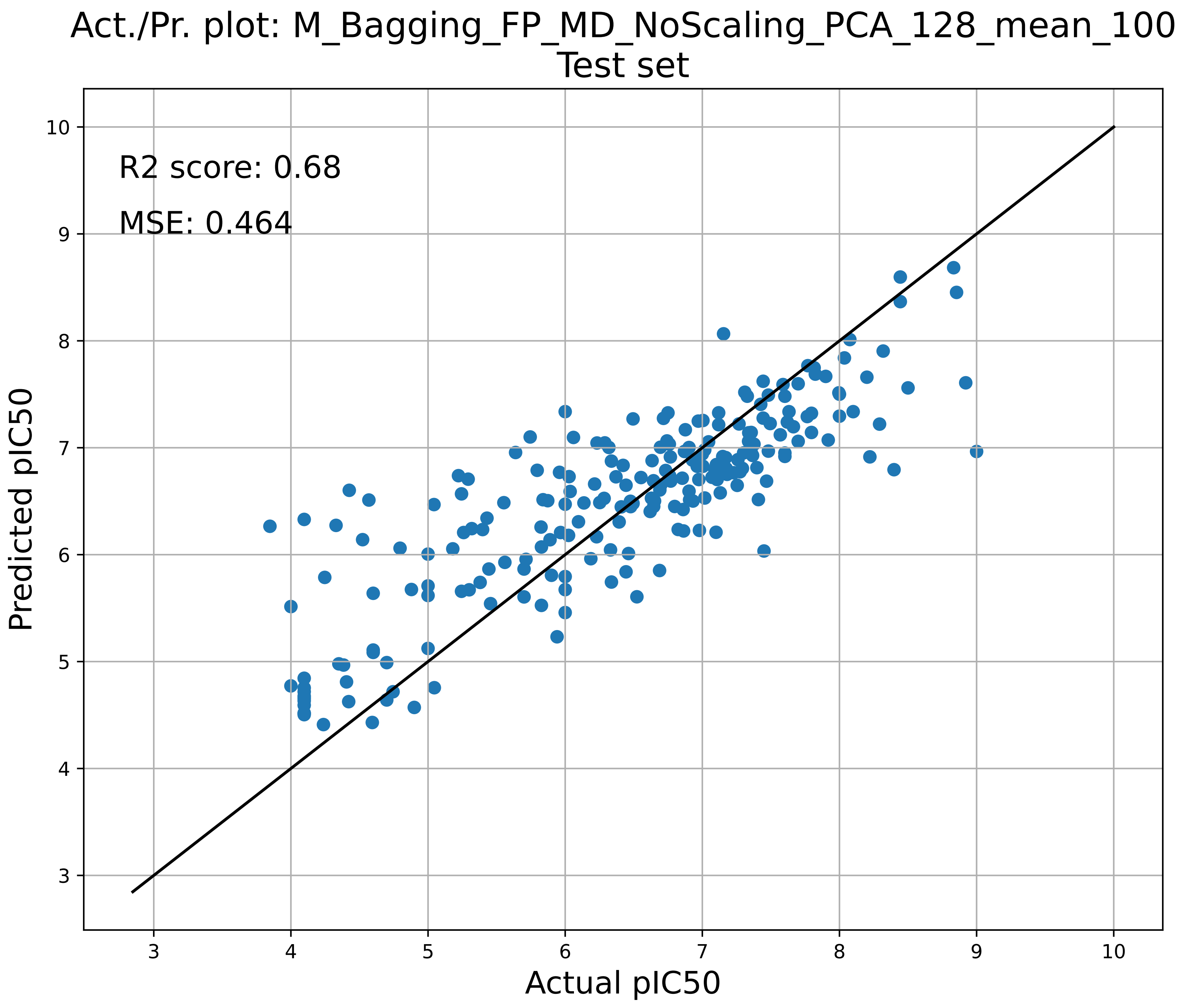

Act./Pr. plot: M_Bagging_FP_MD_NoScaling_PCA_128_mean_100
Test set
R2 score: 0.68
MSE: 0.464
Predicted pIC50
Actual pIC50
3
4
5
6
7
8
9
10

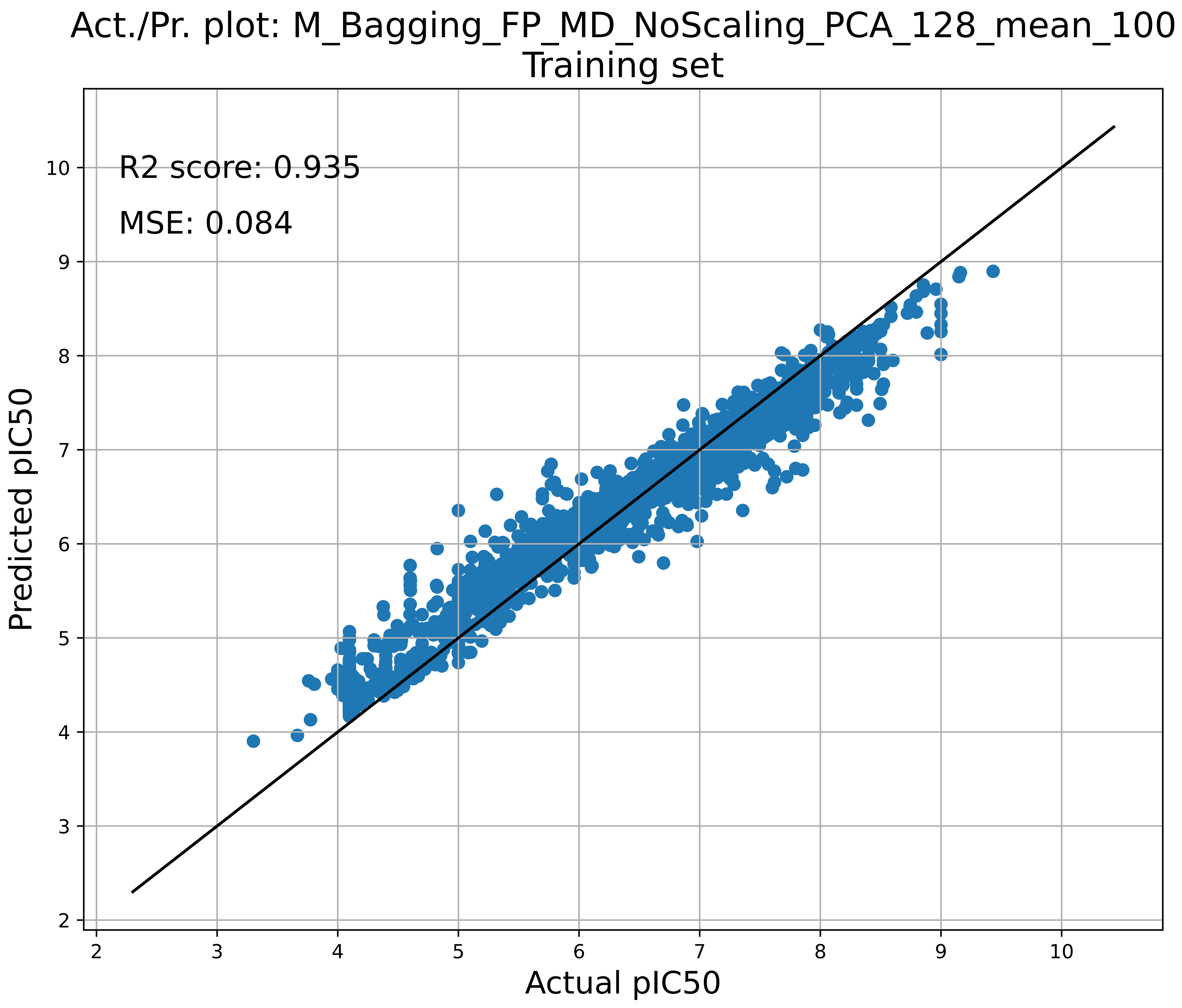
Act./Pr. plot: M_Bagging_FP_MD_NoScaling_PCA_128_mean_100
Training set
R2 score: 0.935
MSE: 0.084
Predicted pIC50
Actual pIC50
2
3
4
5
6
7
8
9
10

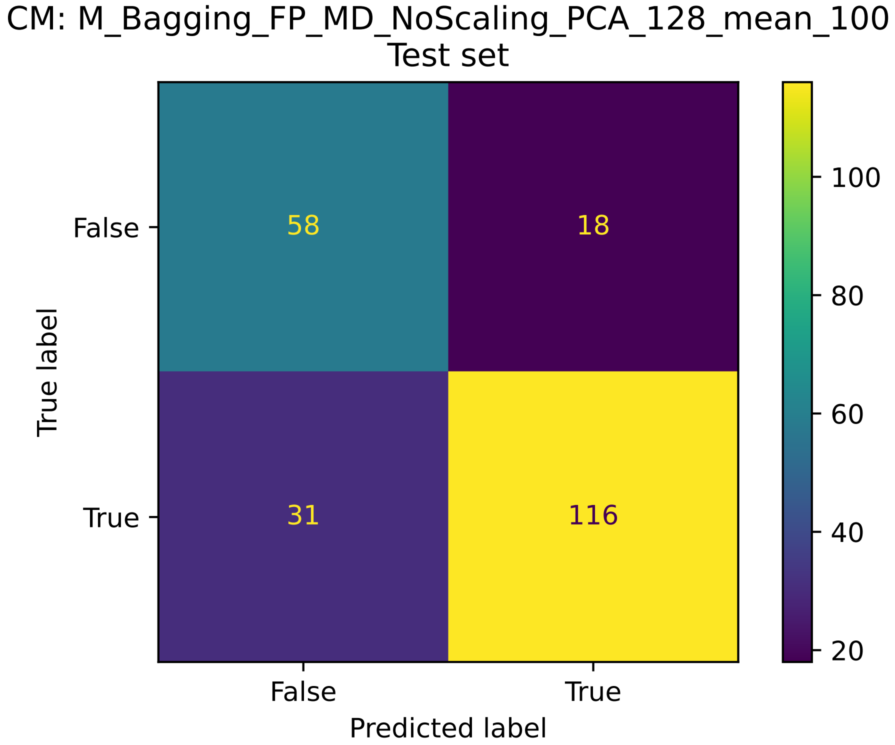
CM: M_Bagging_FP_MD_NoScaling_PCA_128_mean_100
Test set
True label
False
True
58
18
31
116
Predicted label
False
True
100
80
60
40
20

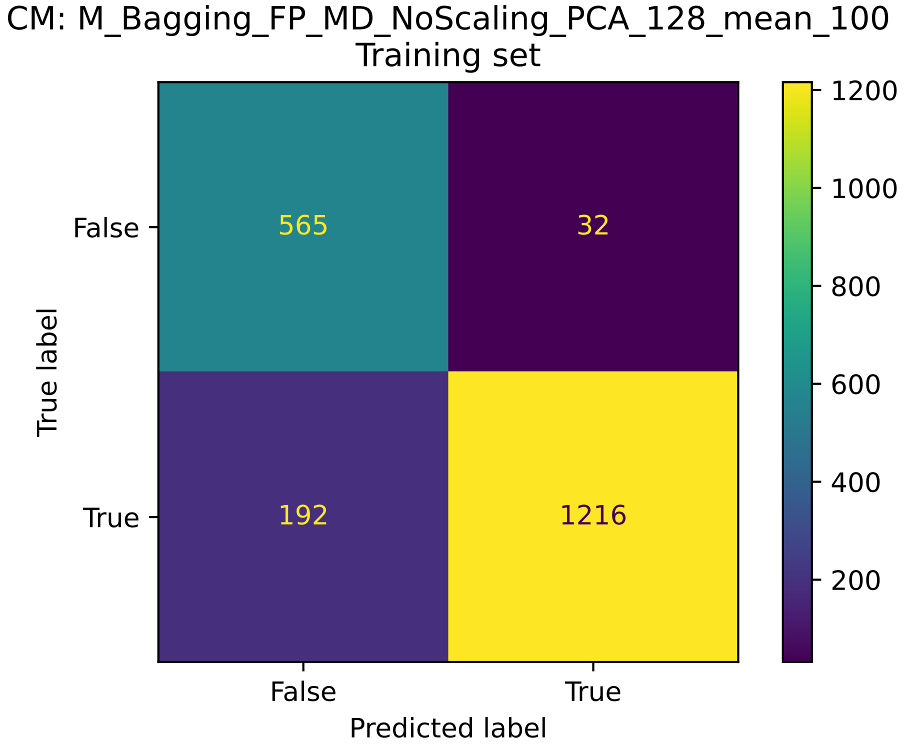
CM: M_Bagging_FP_MD_NoScaling_PCA_128_mean_100
Training set
True label
Predicted label
False
True
False
True
565
32
192
1216
1200
1000
800
600
400
200

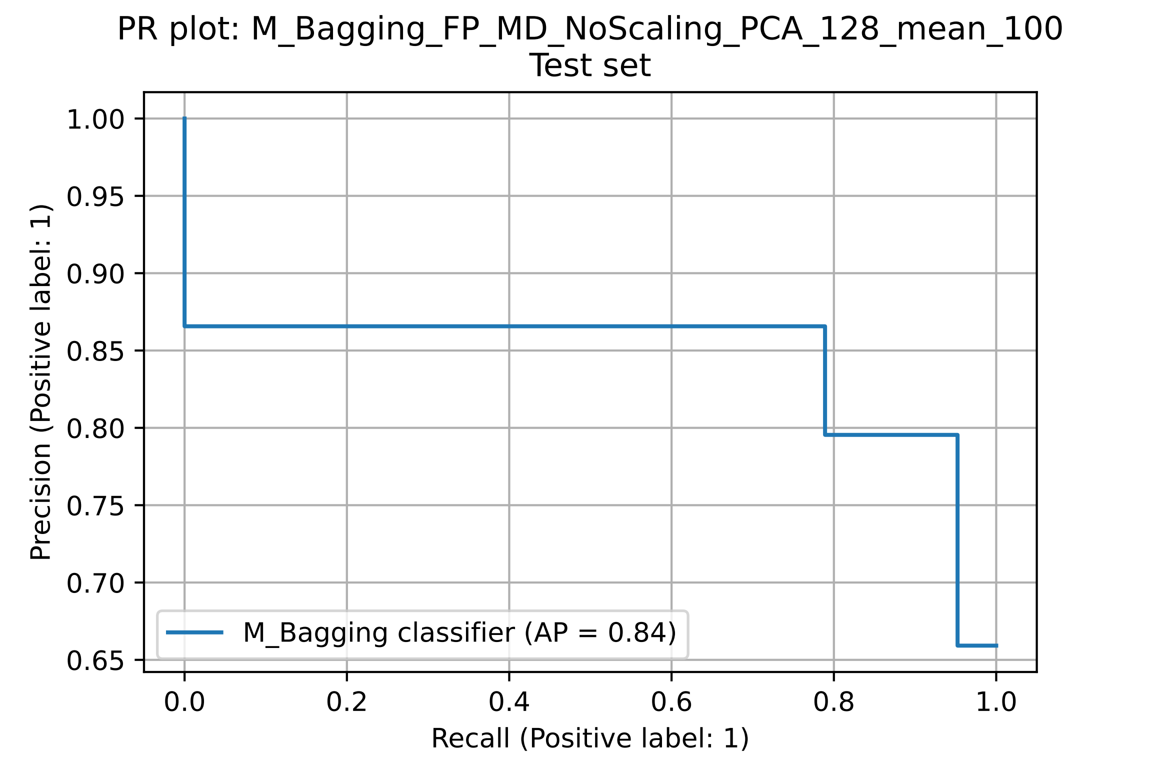
PR plot: M_Bagging_FP_MD_NoScaling_PCA_128_mean_100
Test set
Precision (Positive label: 1)
Recall (Positive label: 1)
1.00
0.95
0.90
0.85
0.80
0.75
0.70
0.65
0.0
0.2
0.4
0.6
0.8
1.0
M_Bagging classifier (AP = 0.84)

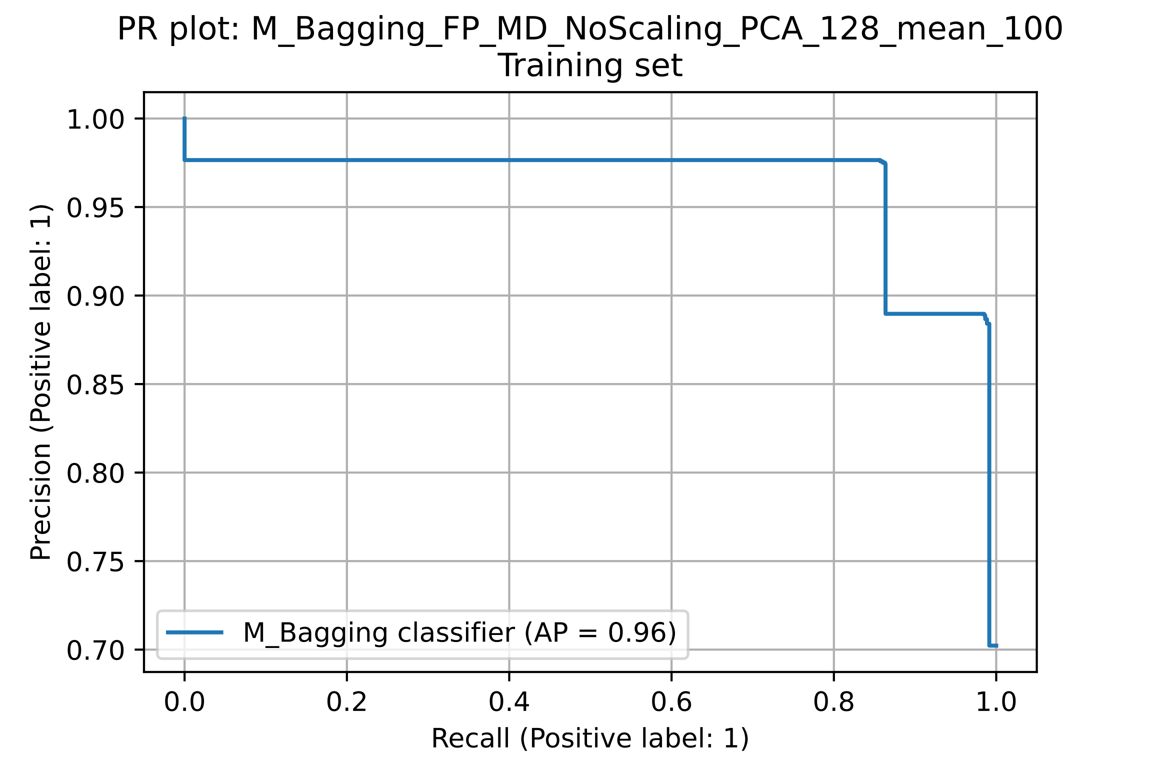

PR plot: M_Bagging_FP_MD_NoScaling_PCA_128_mean_100
Training set
Precision (Positive label: 1)
Recall (Positive label: 1)
1.00
0.95
0.90
0.85
0.80
0.75
0.70
0.0
0.2
0.4
0.6
0.8
1.0
M_Bagging classifier (AP = 0.96)

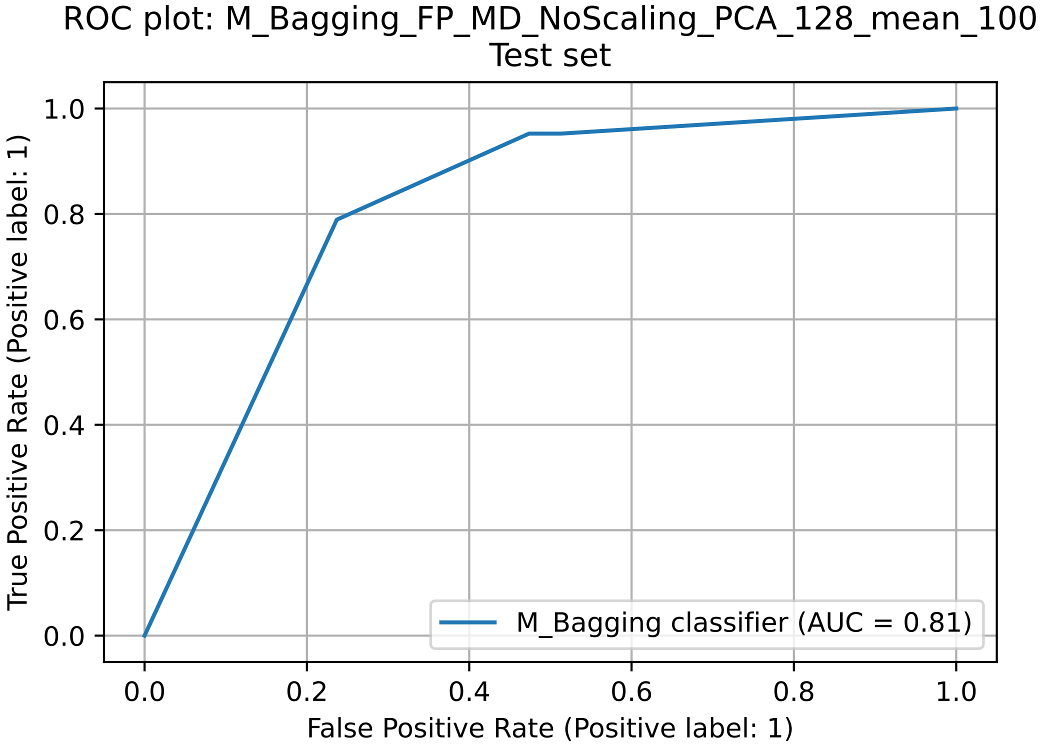
ROC plot: M_Bagging_FP_MD_NoScaling_PCA_128_mean_100
Test set
True Positive Rate (Positive label: 1)
False Positive Rate (Positive label: 1)
0.0
0.2
0.4
0.6
0.8
1.0
M_Bagging classifier (AUC = 0.81)

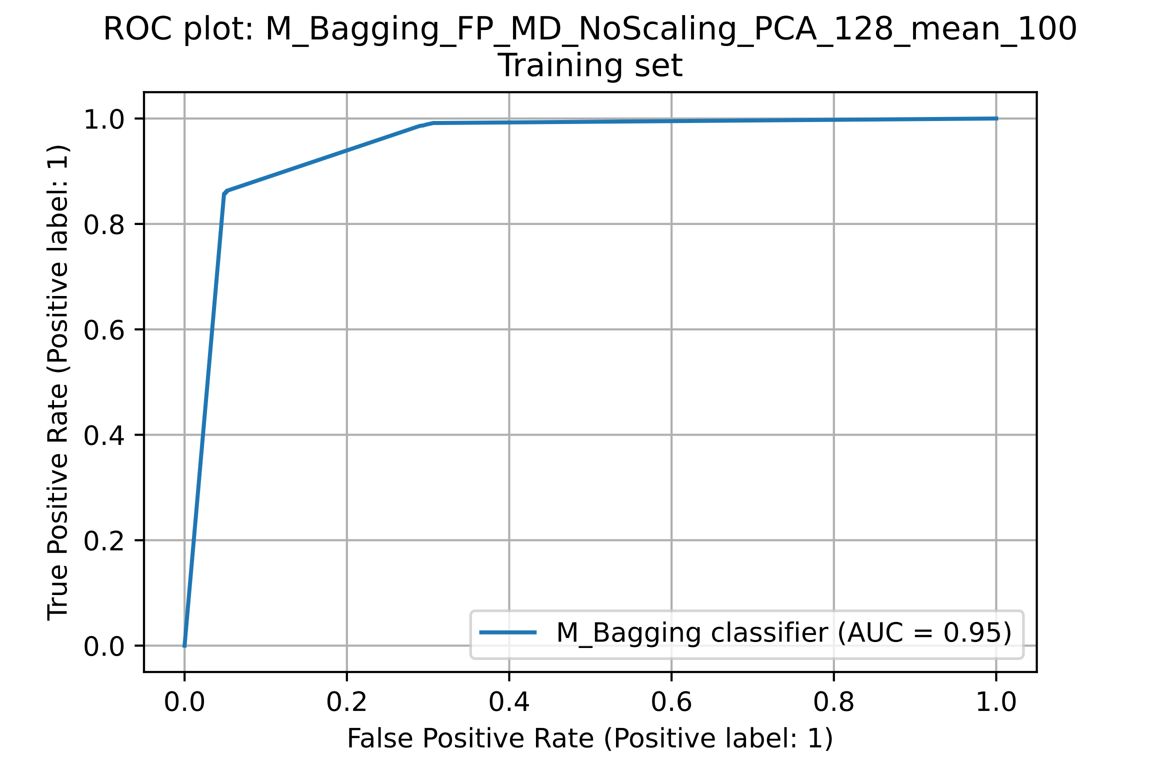

ROC plot: M_Bagging_FP_MD_NoScaling_PCA_128_mean_100
Training set
True Positive Rate (Positive label: 1)
False Positive Rate (Positive label: 1)
M_Bagging classifier (AUC = 0.95)
0.0
0.2
0.4
0.6
0.8
1.0

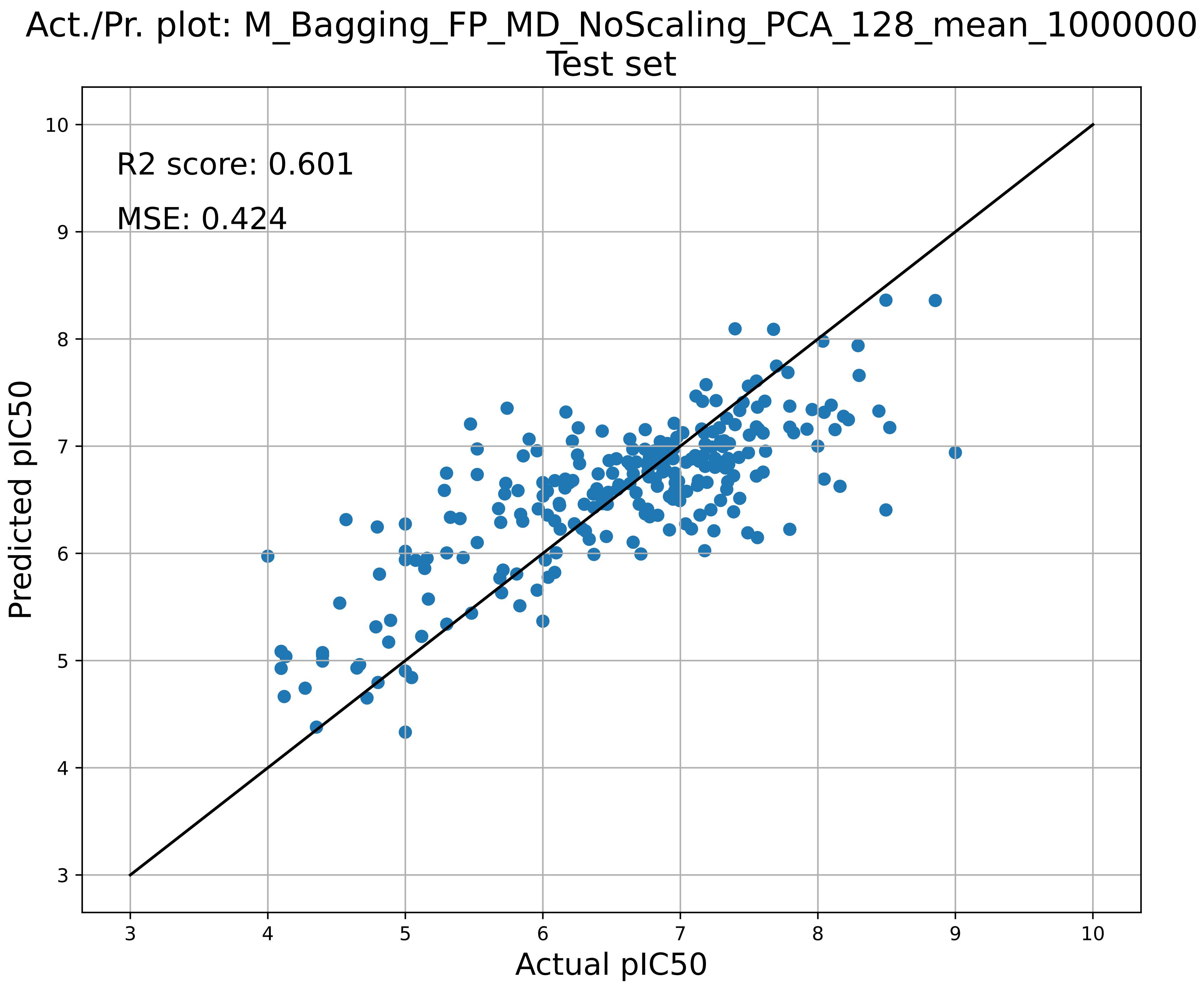

Act./Pr. plot: M_Bagging_FP_MD_NoScaling_PCA_128_mean_1000000
Test set
R2 score: 0.601
MSE: 0.424
Predicted pIC50
Actual pIC50
3
4
5
6
7
8
9
10

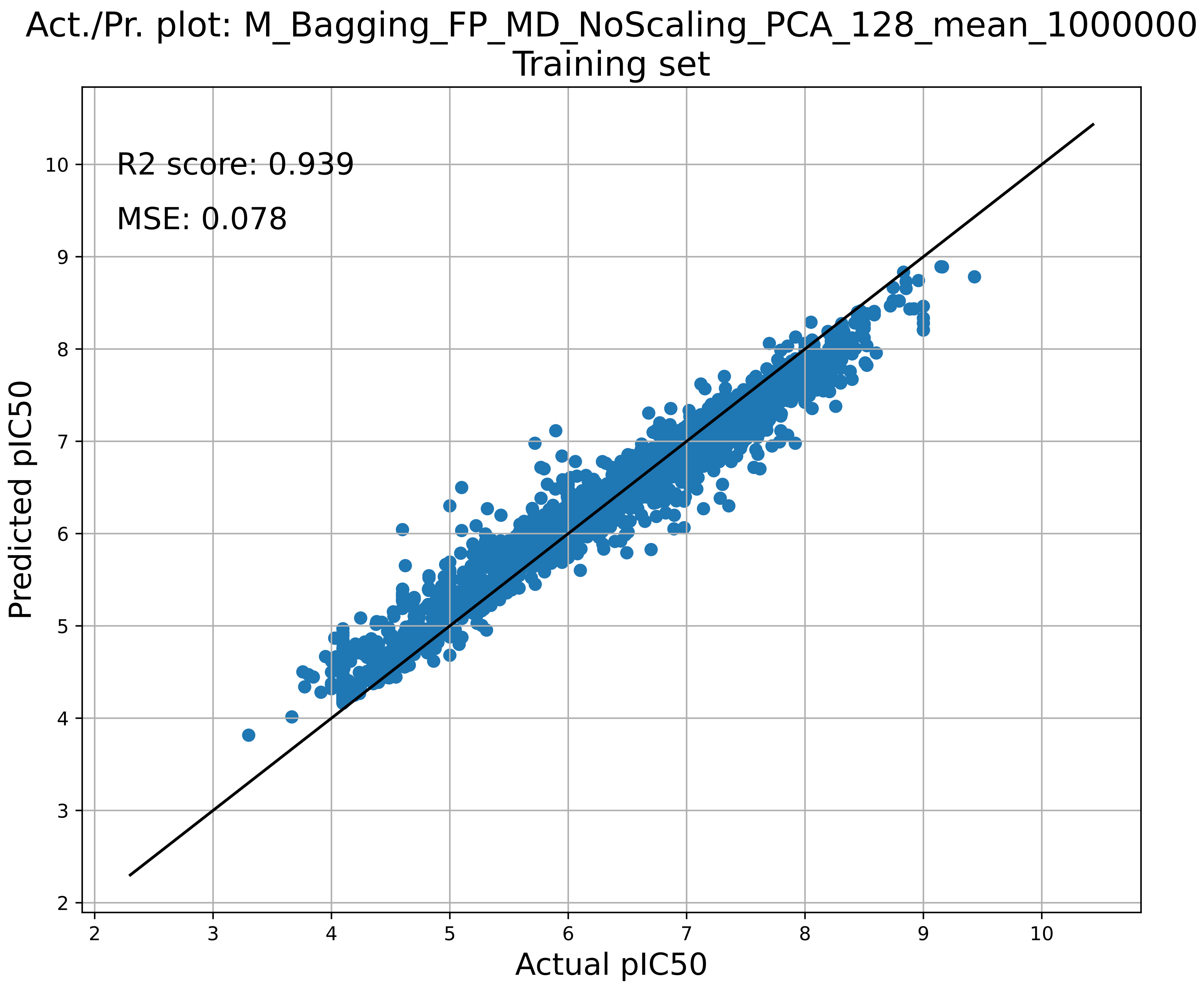

Act./Pr. plot: M_Bagging_FP_MD_NoScaling_PCA_128_mean_1000000
Training set
R2 score: 0.939
MSE: 0.078
Predicted pIC50
Actual pIC50
2
3
4
5
6
7
8
9
10

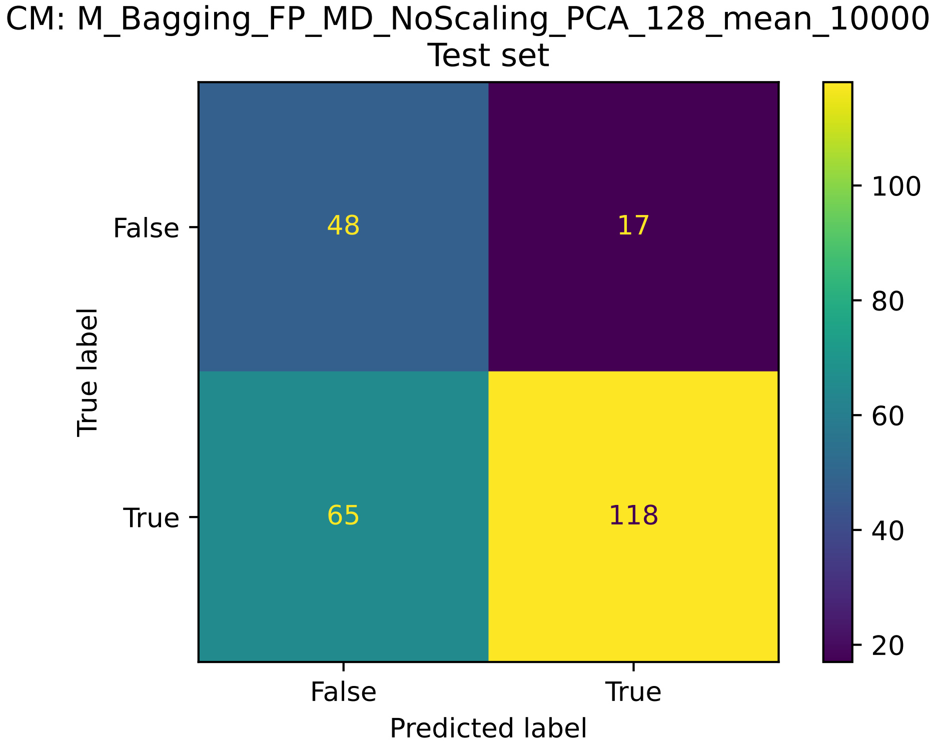
CM: M_Bagging_FP_MD_NoScaling_PCA_128_mean_1000000
Test set
48
17
65
118
False
True
True label
False
True
Predicted label
100
80
60
40
20

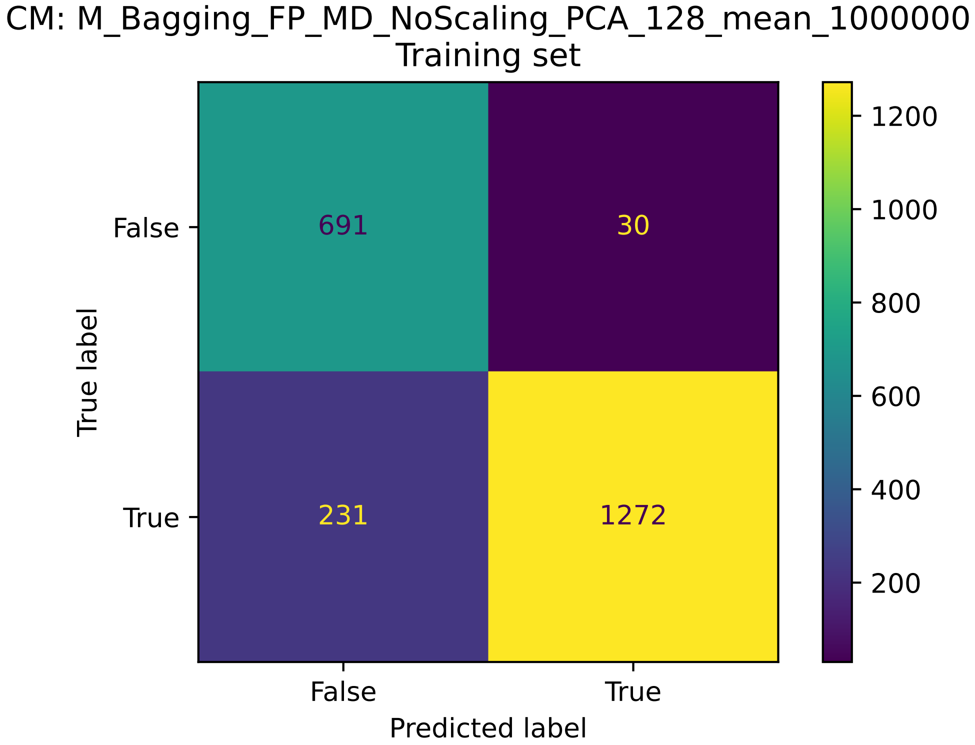
CM: M_Bagging_FP_MD_NoScaling_PCA_128_mean_1000000
Training set
691
30
231
1272
False
True
True label
False
True
Predicted label
1200
1000
800
600
400
200

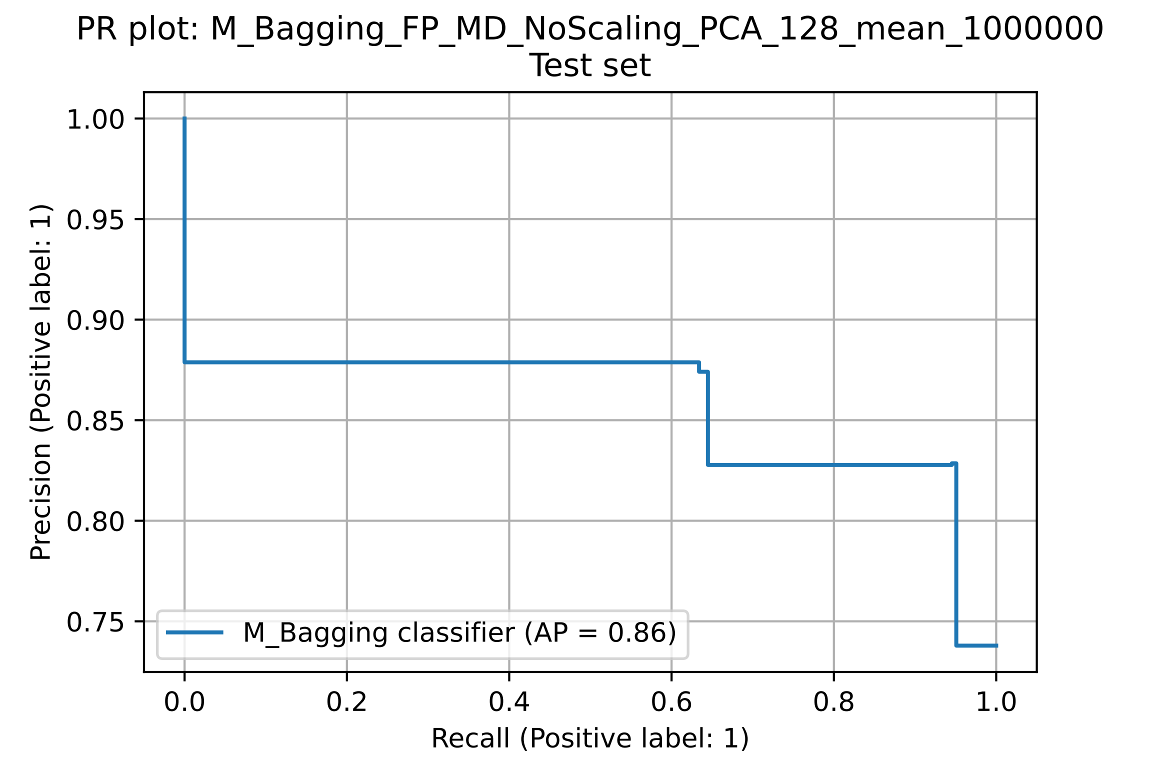

PR plot: M_Bagging_FP_MD_NoScaling_PCA_128_mean_1000000
Test set
Precision (Positive label: 1)
Recall (Positive label: 1)
1.00
0.95
0.90
0.85
0.80
0.75
0.0
0.2
0.4
0.6
0.8
1.0
M_Bagging classifier (AP = 0.86)

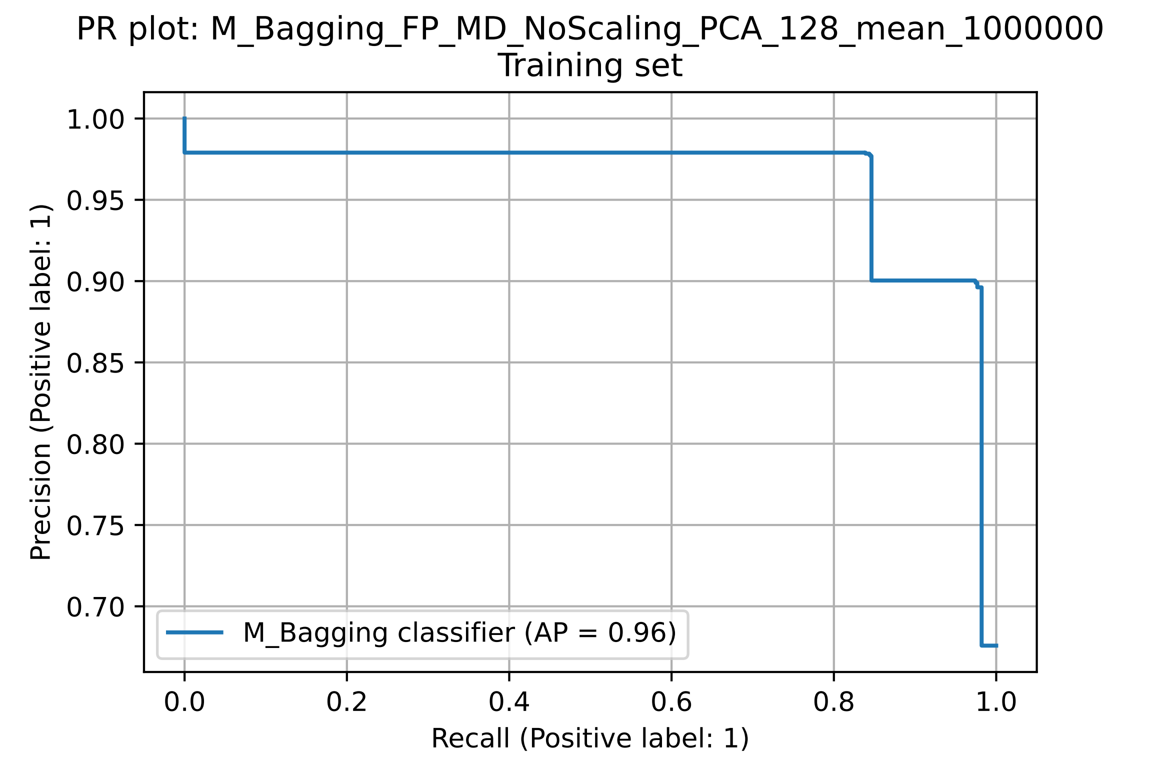

PR plot: M_Bagging_FP_MD_NoScaling_PCA_128_mean_1000000
Training set
1.00
0.95
0.90
0.85
0.80
0.75
0.70
Precision (Positive label: 1)
0.0
0.2
0.4
0.6
0.8
1.0
Recall (Positive label: 1)
M_Bagging classifier (AP = 0.96)

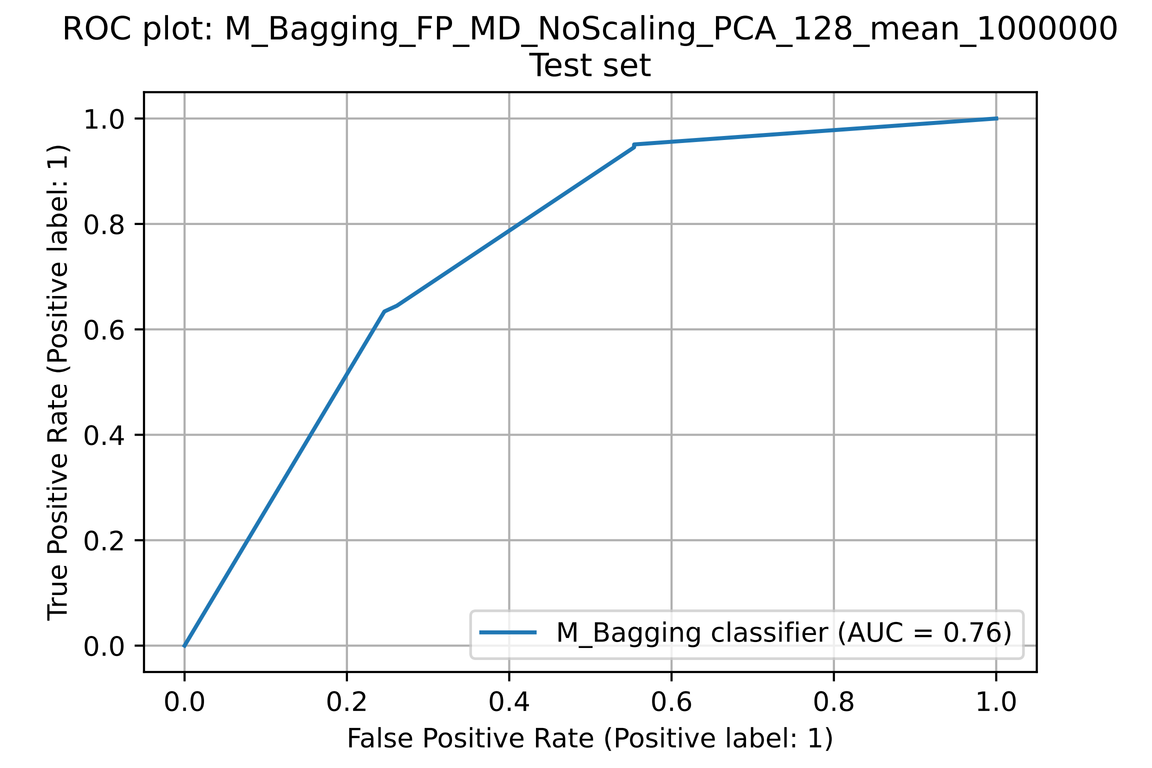
ROC plot: M_Bagging_FP_MD_NoScaling_PCA_128_mean_1000000
Test set
True Positive Rate (Positive label: 1)
False Positive Rate (Positive label: 1)
0.0
0.2
0.4
0.6
0.8
1.0
M_Bagging classifier (AUC = 0.76)

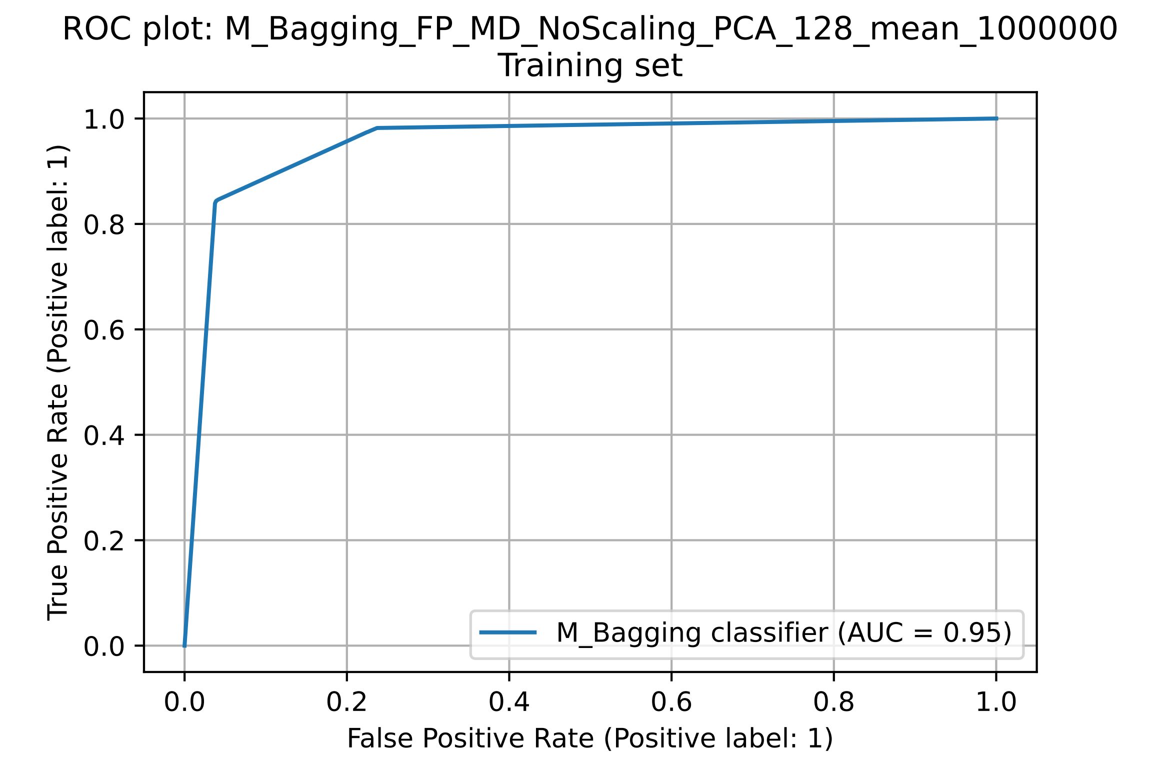
ROC plot: M_Bagging_FP_MD_NoScaling_PCA_128_mean_1000000
Training set
True Positive Rate (Positive label: 1)
False Positive Rate (Positive label: 1)
0.0
0.2
0.4
0.6
0.8
1.0
M_Bagging classifier (AUC = 0.95)

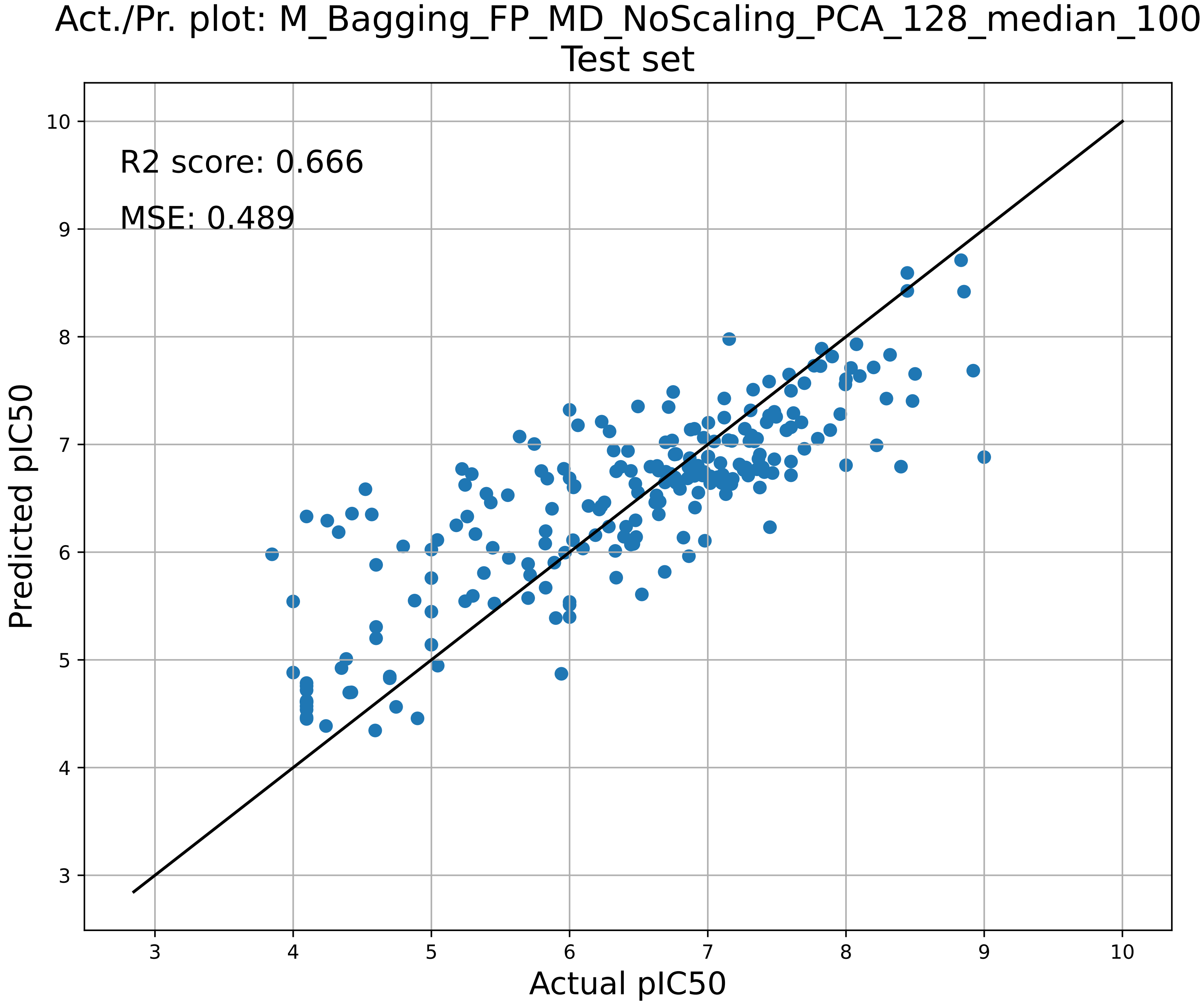
Act./Pr. plot: M_Bagging_FP_MD_NoScaling_PCA_128_median_100
Test set
R2 score: 0.666
MSE: 0.489
Predicted pIC50
Actual pIC50
3
4
5
6
7
8
9
10

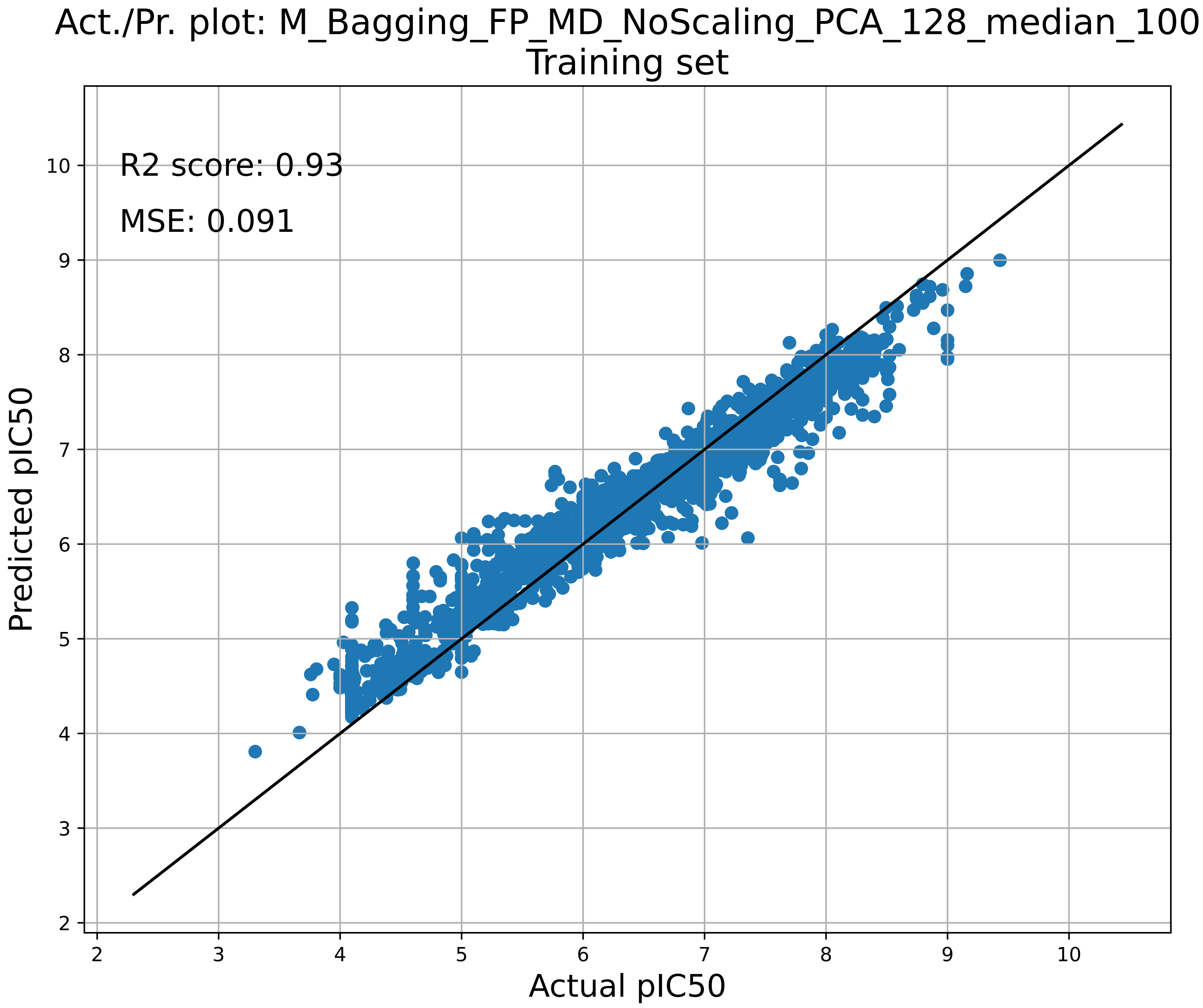

Act./Pr. plot: M_Bagging_FP_MD_NoScaling_PCA_128_median_100
Training set
R2 score: 0.93
MSE: 0.091
Predicted pIC50
Actual pIC50
2
3
4
5
6
7
8
9
10

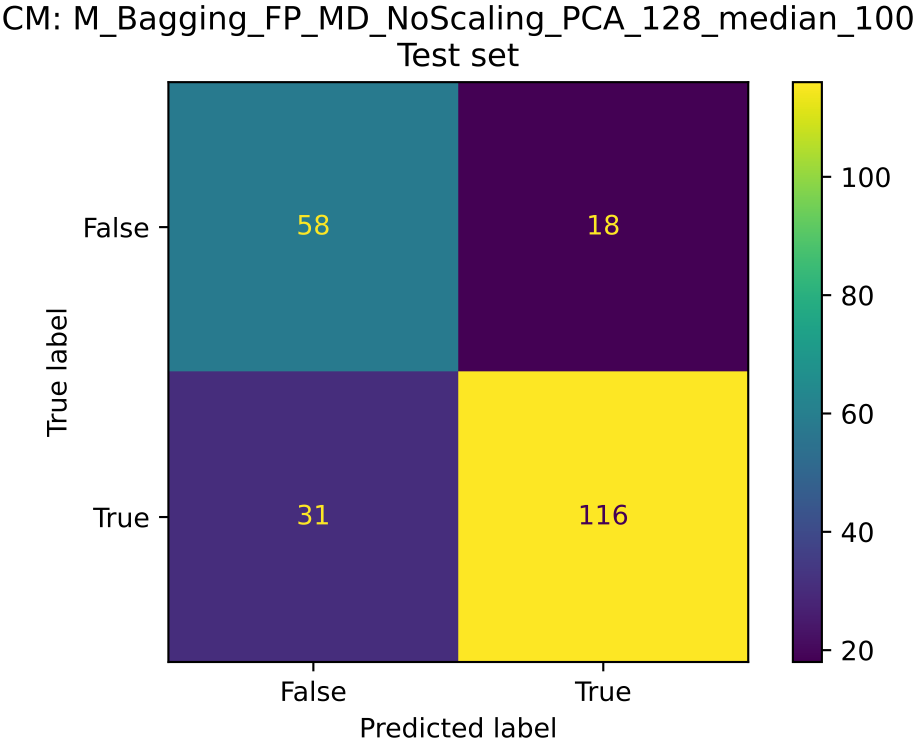

CM: M_Bagging_FP_MD_NoScaling_PCA_128_median_100
Test set
True label
False
True
58
18
31
116
Predicted label
False
True
100
80
60
40
20

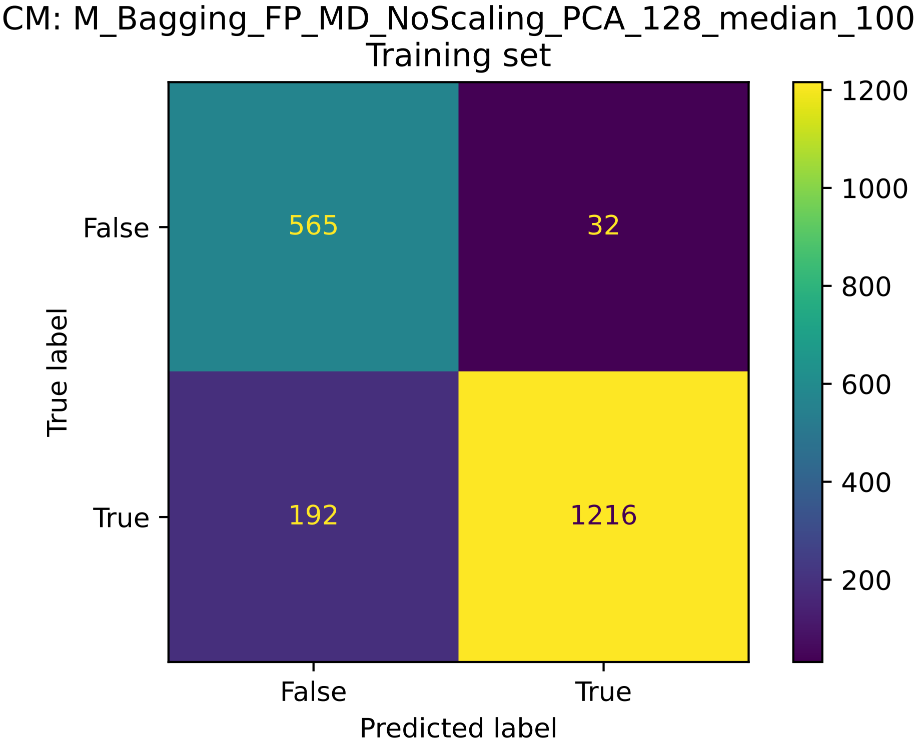
CM: M_Bagging_FP_MD_NoScaling_PCA_128_median_100
Training set
True label
False
True
565
32
192
1216
Predicted label
False
True
1200
1000
800
600
400
200

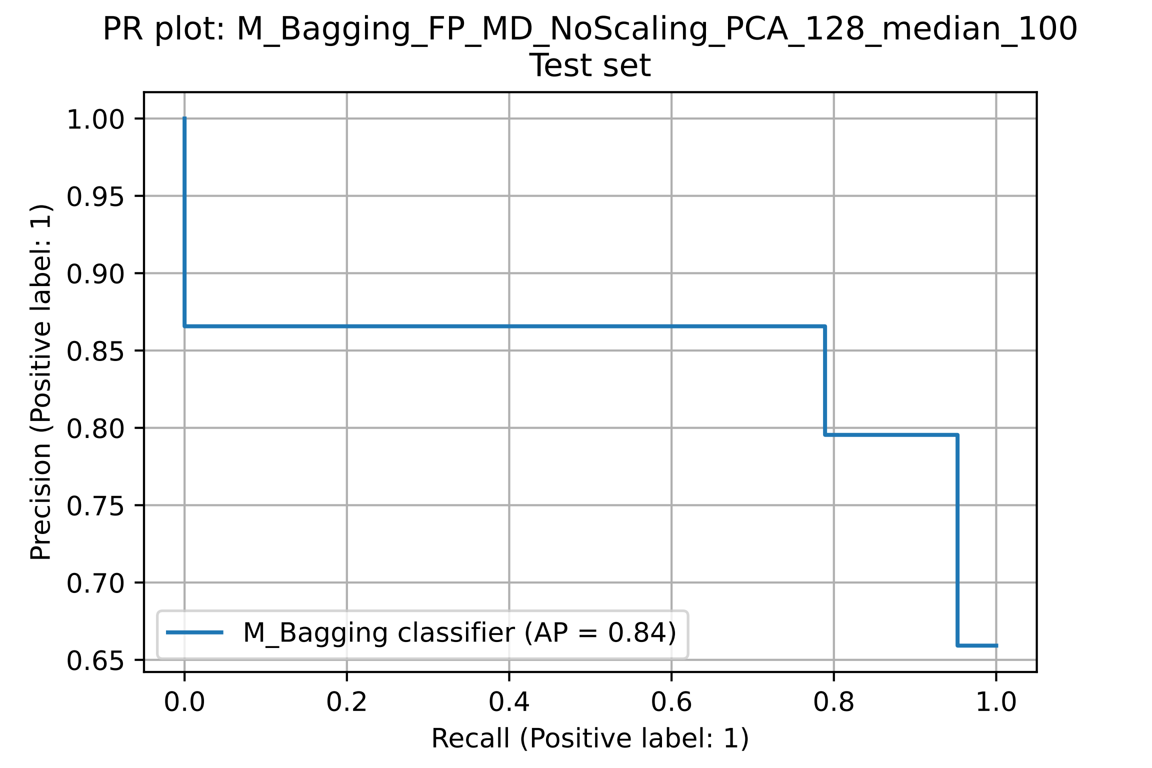
PR plot: M_Bagging_FP_MD_NoScaling_PCA_128_median_100
Test set
1.00
0.95
0.90
0.85
0.80
0.75
0.70
0.65
Precision (Positive label: 1)
0.0
0.2
0.4
0.6
0.8
1.0
Recall (Positive label: 1)
M_Bagging classifier (AP = 0.84)

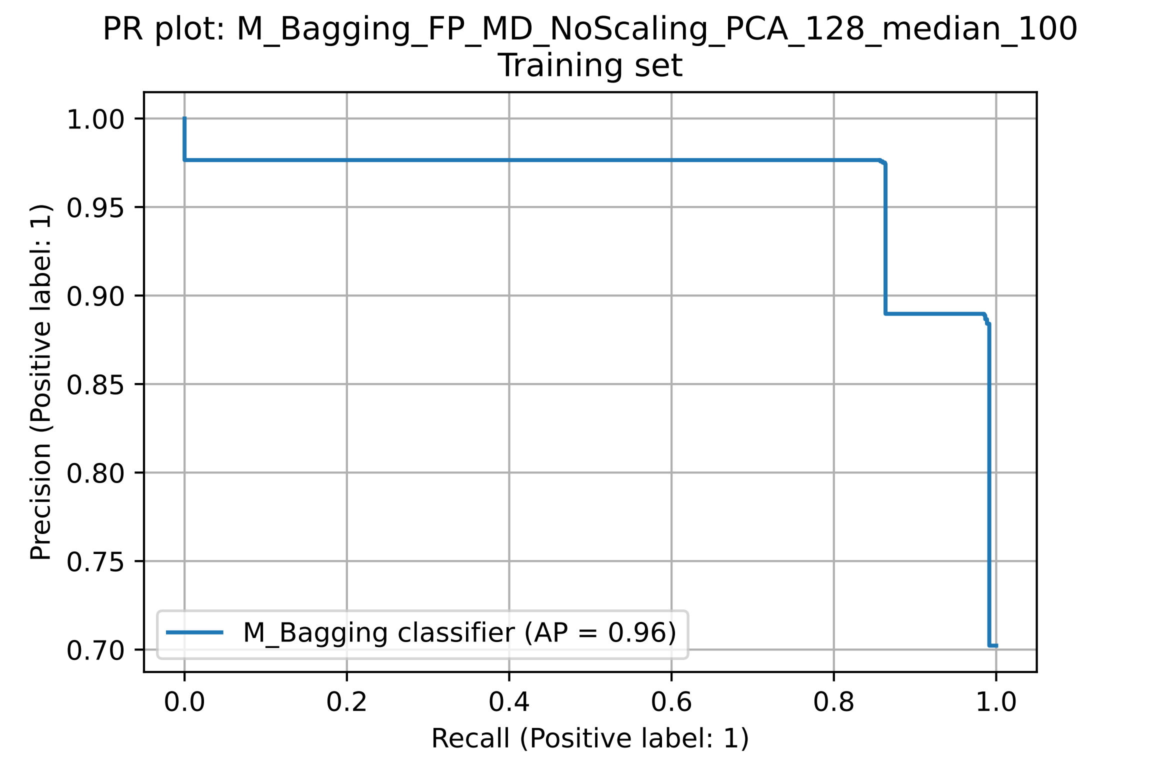
PR plot: M_Bagging_FP_MD_NoScaling_PCA_128_median_100
Training set
Precision (Positive label: 1)
Recall (Positive label: 1)
1.00
0.95
0.90
0.85
0.80
0.75
0.70
0.0
0.2
0.4
0.6
0.8
1.0
M_Bagging classifier (AP = 0.96)

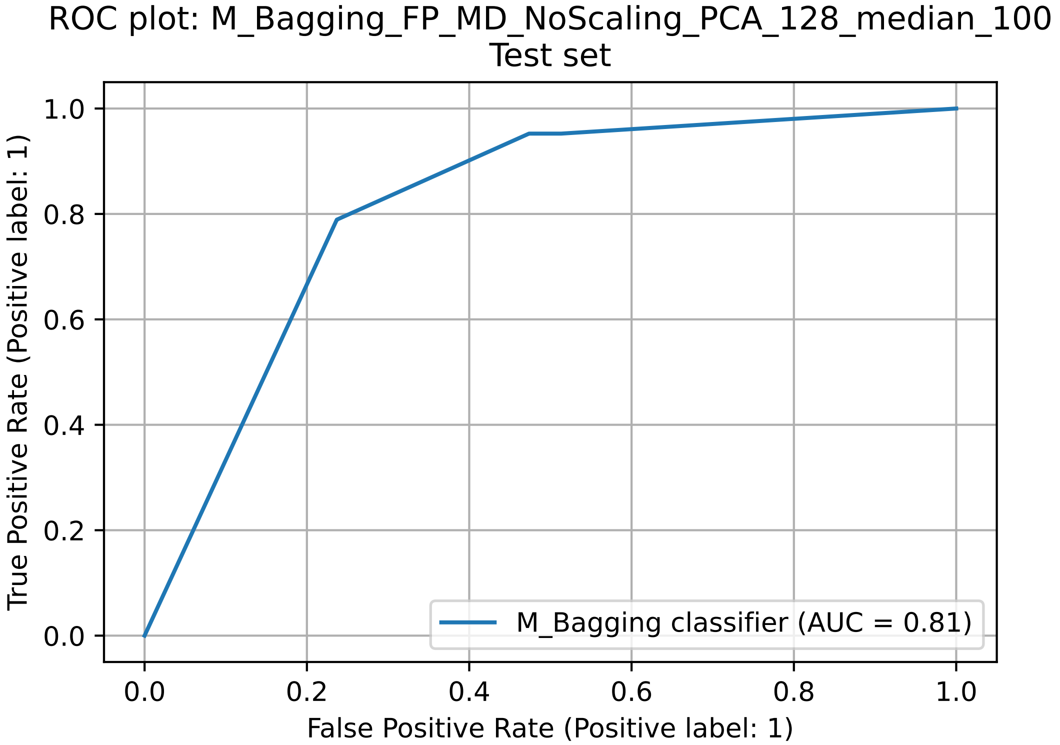

ROC plot: M_Bagging_FP_MD_NoScaling_PCA_128_median_100
Test set
True Positive Rate (Positive label: 1)
False Positive Rate (Positive label: 1)
0.0
0.2
0.4
0.6
0.8
1.0
M_Bagging classifier (AUC = 0.81)

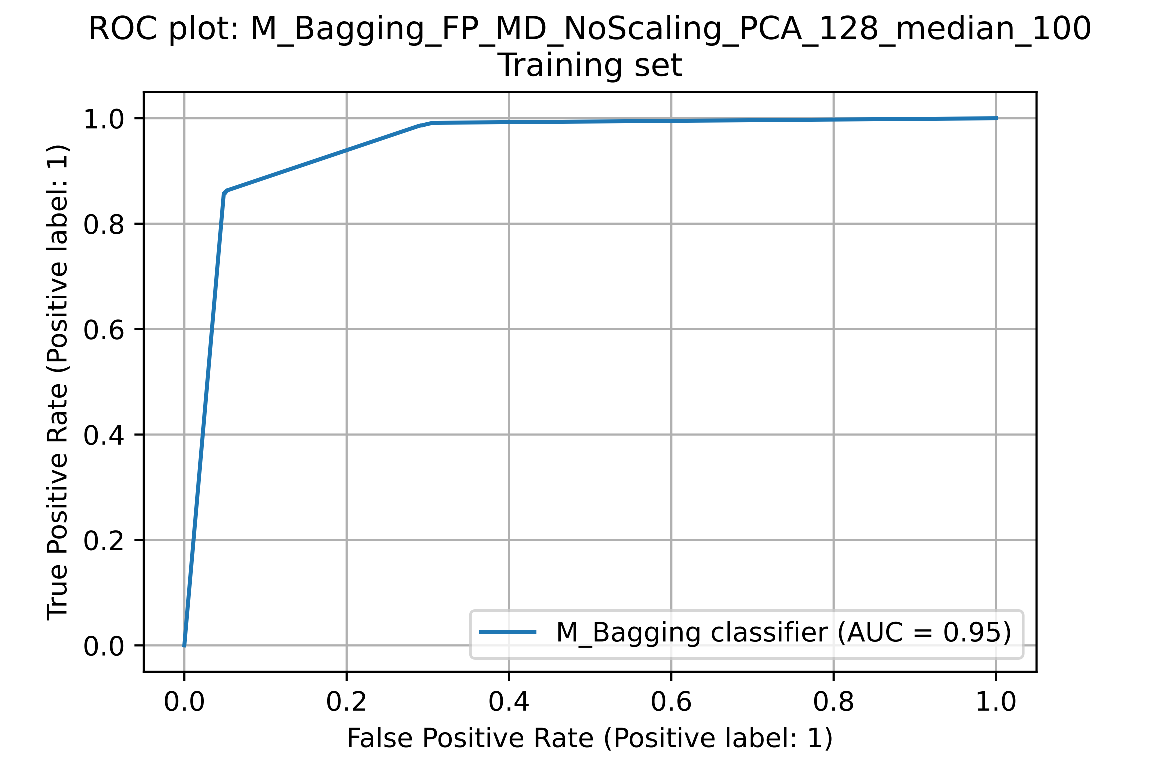

ROC plot: M_Bagging_FP_MD_NoScaling_PCA_128_median_100
Training set
True Positive Rate (Positive label: 1)
False Positive Rate (Positive label: 1)
0.0
0.2
0.4
0.6
0.8
1.0
M_Bagging classifier (AUC = 0.95)

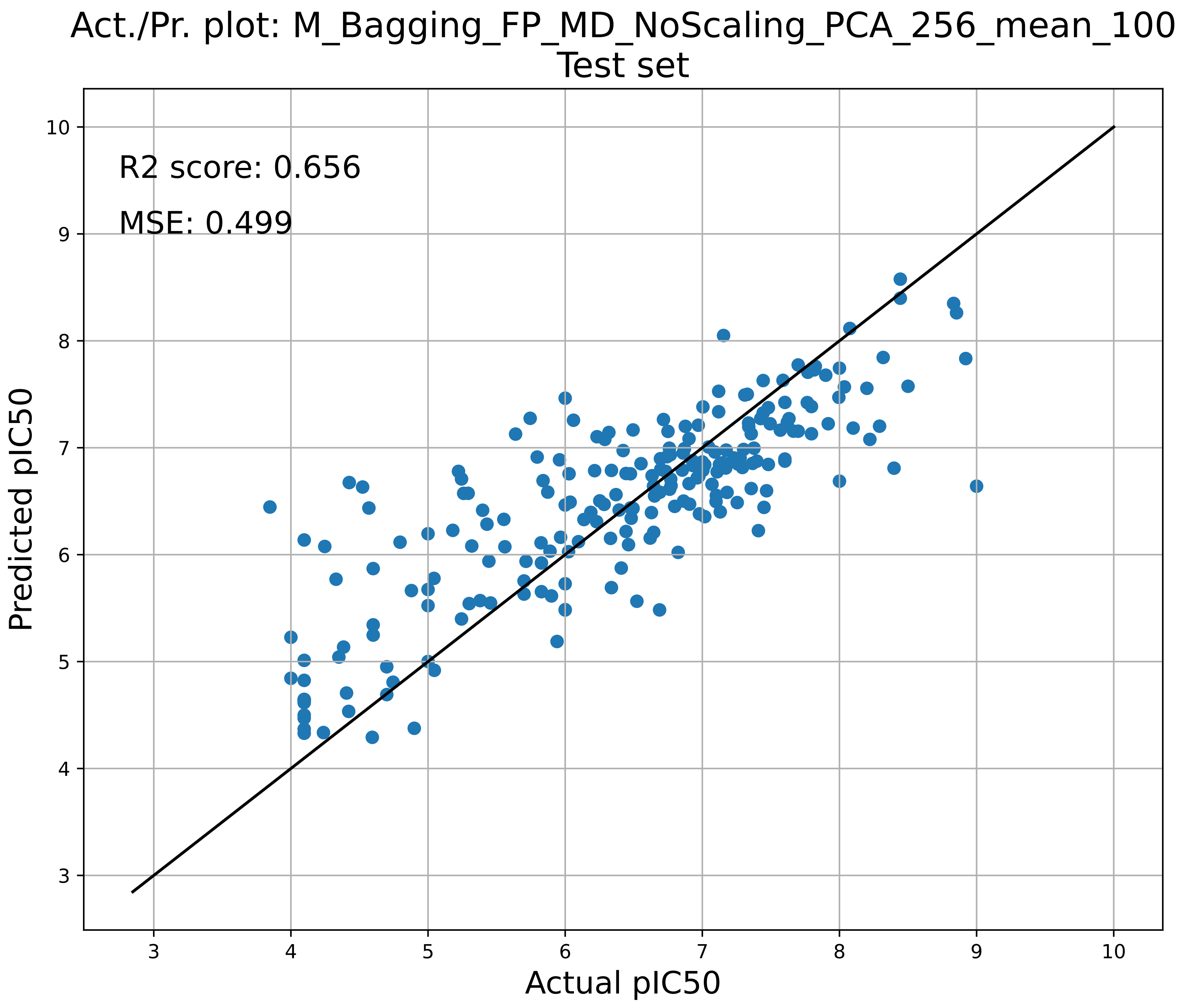
Act./Pr. plot: M_Bagging_FP_MD_NoScaling_PCA_256_mean_100
Test set
R2 score: 0.656
MSE: 0.499
Predicted pIC50
Actual pIC50
3
4
5
6
7
8
9
10

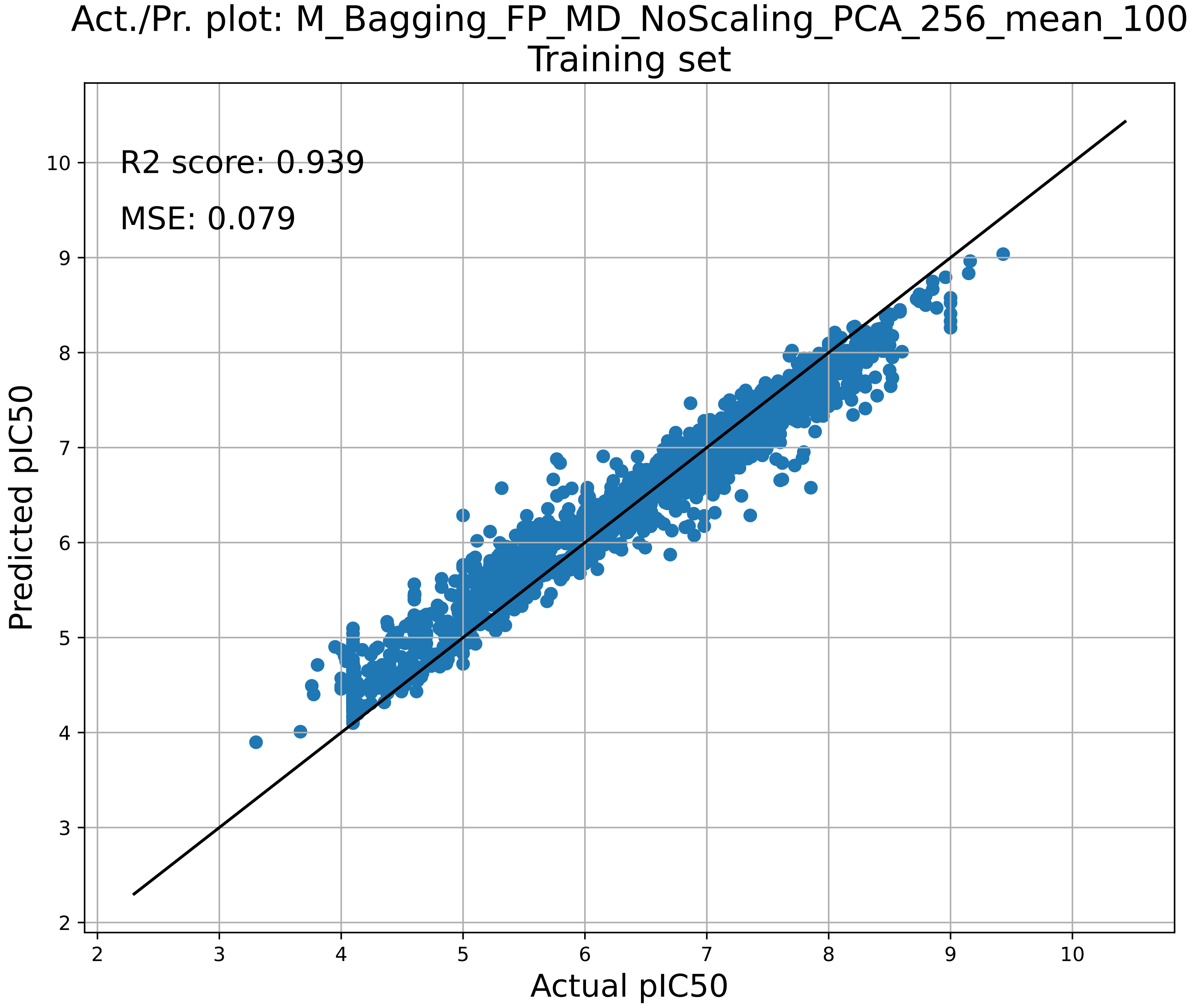
Act./Pr. plot: M_Bagging_FP_MD_NoScaling_PCA_256_mean_100
Training set
R2 score: 0.939
MSE: 0.079
Predicted pIC50
Actual pIC50
2
3
4
5
6
7
8
9
10

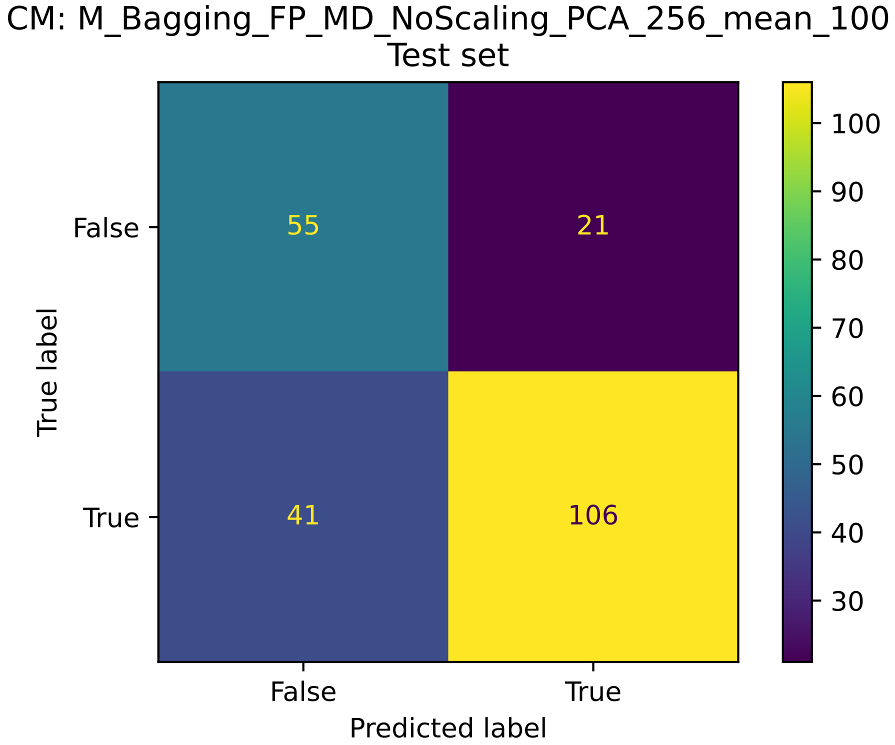
CM: M_Bagging_FP_MD_NoScaling_PCA_256_mean_100
Test set
True label
False
True
55
21
41
106
Predicted label
False
True
100
90
80
70
60
50
40
30

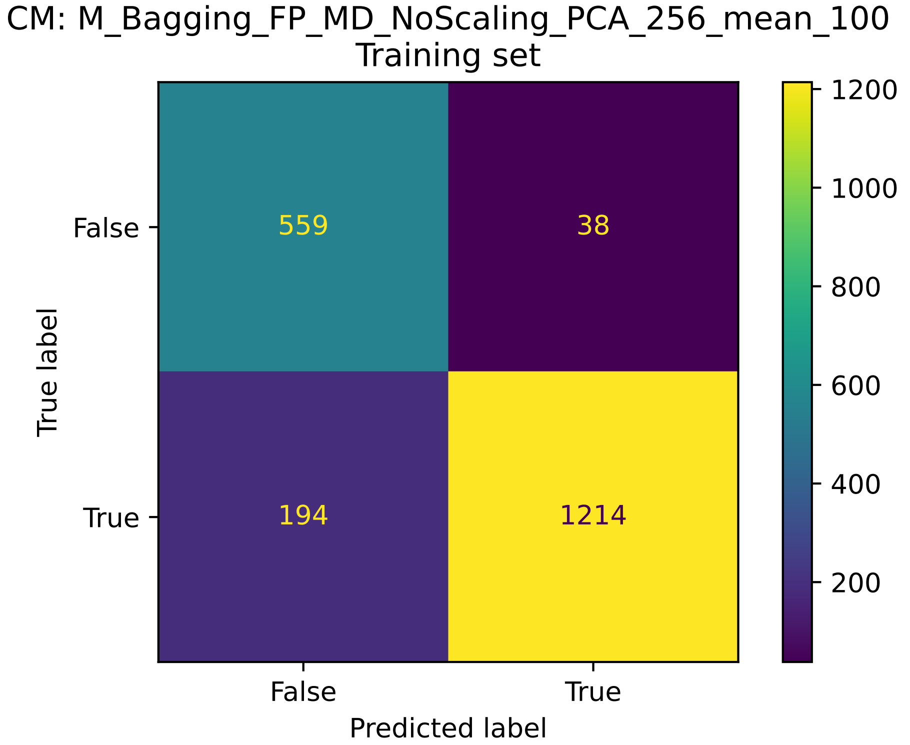
CM: M_Bagging_FP_MD_NoScaling_PCA_256_mean_100
Training set
559
38
194
1214
False
True
True label
False
True
Predicted label
1200
1000
800
600
400
200

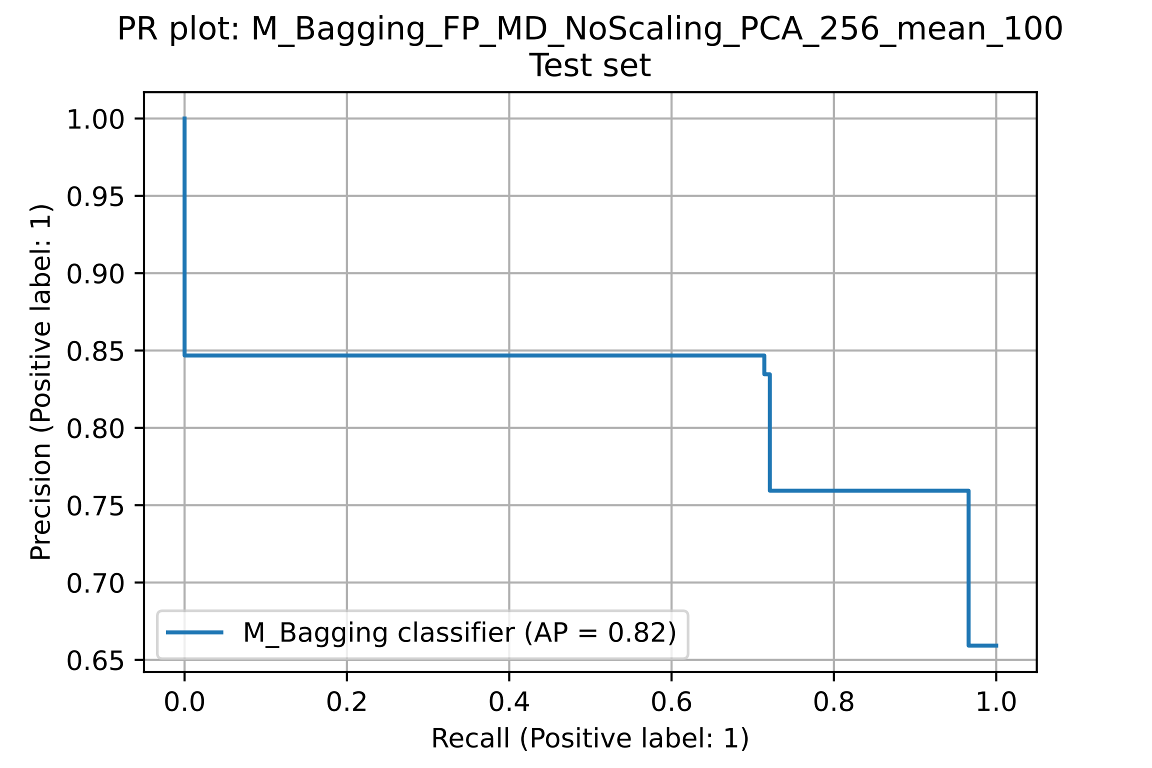
PR plot: M_Bagging_FP_MD_NoScaling_PCA_256_mean_100
Test set
1.00
0.95
0.90
0.85
0.80
0.75
0.70
0.65
Precision (Positive label: 1)
0.0
0.2
0.4
0.6
0.8
1.0
Recall (Positive label: 1)
M_Bagging classifier (AP = 0.82)

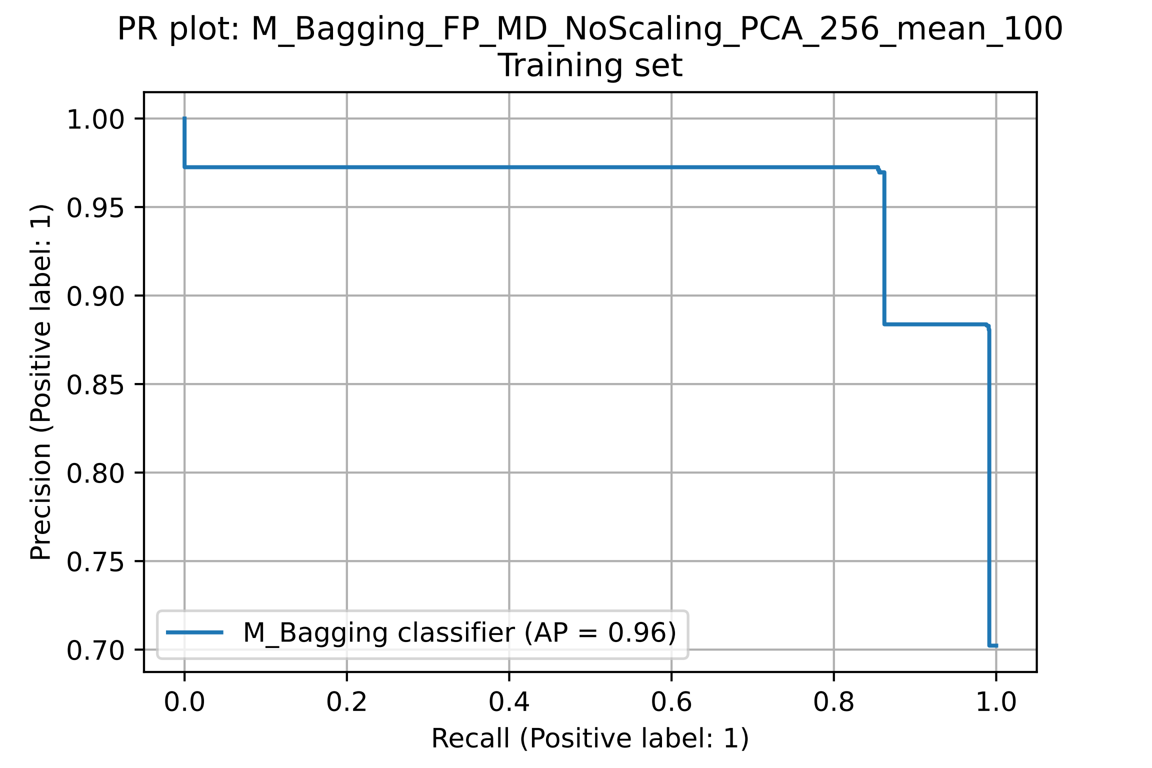
PR plot: M_Bagging_FP_MD_NoScaling_PCA_256_mean_100
Training set
1.00
0.95
0.90
0.85
0.80
0.75
0.70
Precision (Positive label: 1)
0.0
0.2
0.4
0.6
0.8
1.0
Recall (Positive label: 1)
M_Bagging classifier (AP = 0.96)

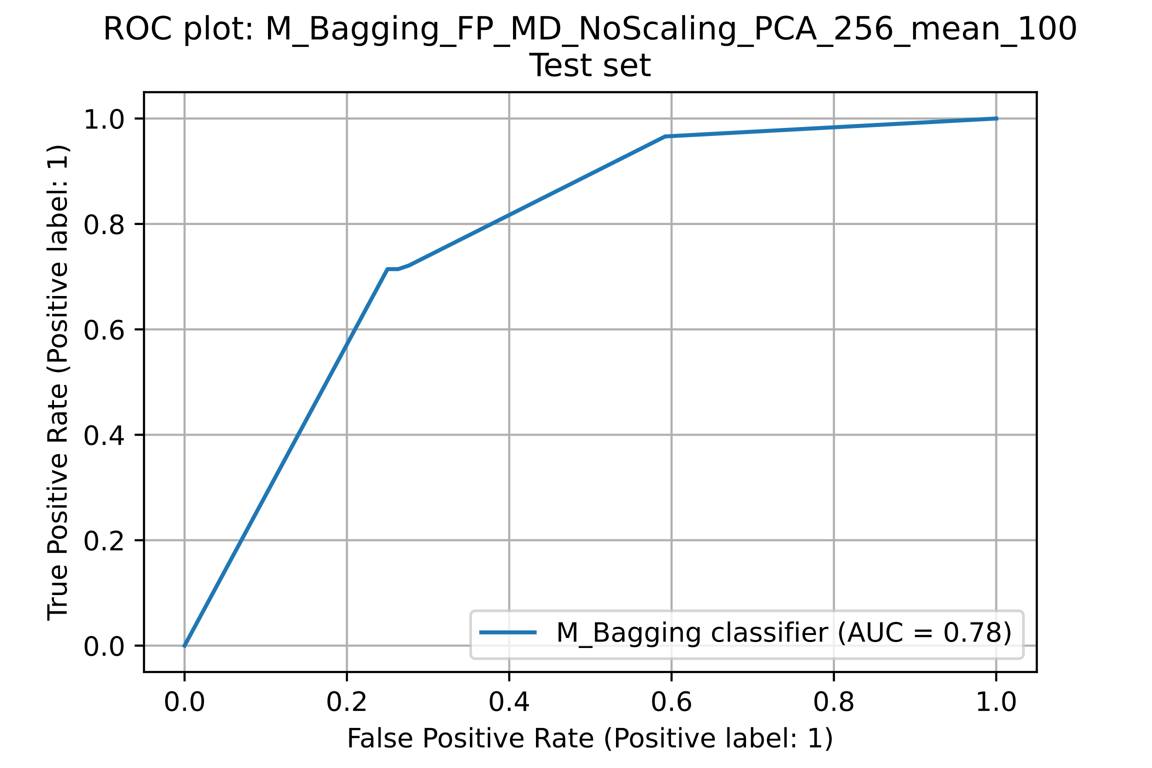
ROC plot: M_Bagging_FP_MD_NoScaling_PCA_256_mean_100
Test set
True Positive Rate (Positive label: 1)
False Positive Rate (Positive label: 1)
0.0
0.2
0.4
0.6
0.8
1.0
0.0
0.2
0.4
0.6
0.8
1.0
M_Bagging classifier (AUC = 0.78)

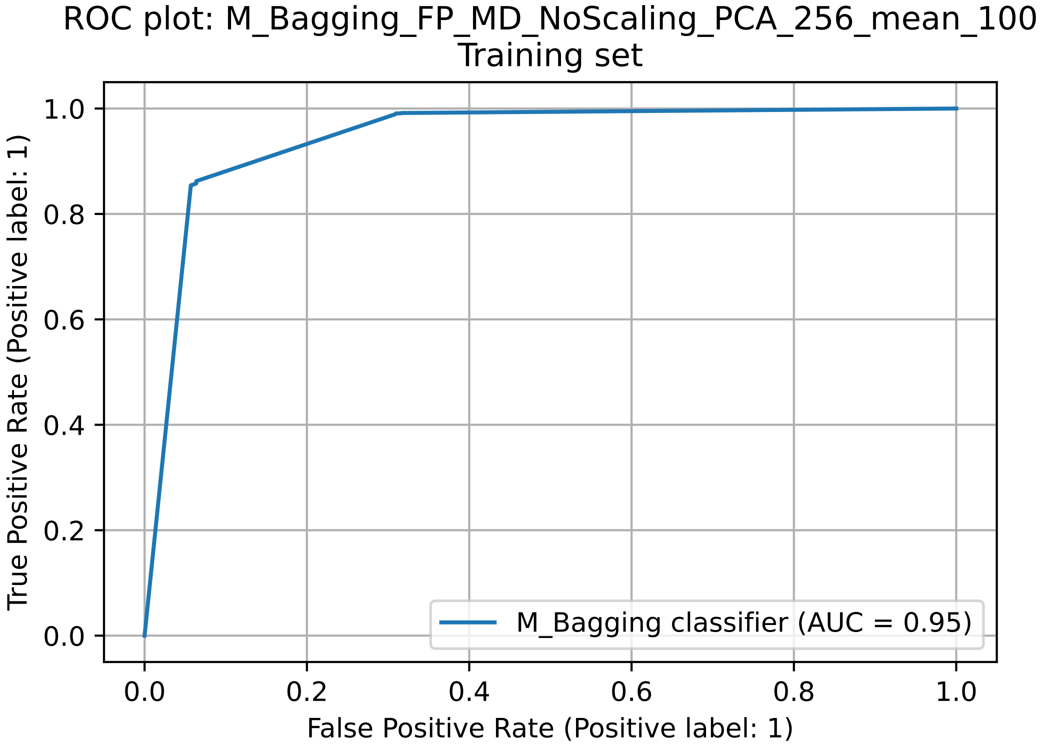
ROC plot: M_Bagging_FP_MD_NoScaling_PCA_256_mean_100
Training set
True Positive Rate (Positive label: 1)
False Positive Rate (Positive label: 1)
0.0
0.2
0.4
0.6
0.8
1.0
M_Bagging classifier (AUC = 0.95)

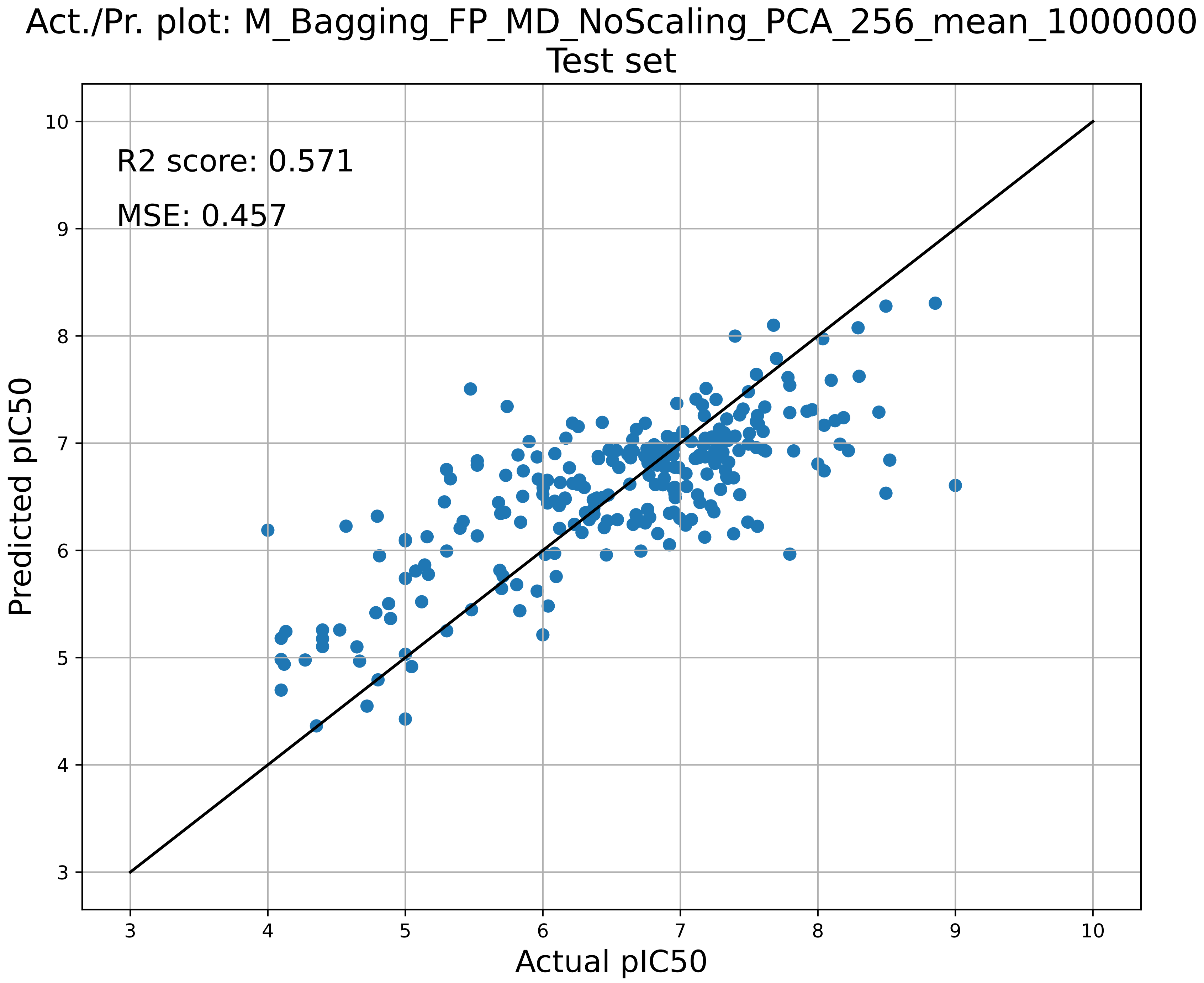

Act./Pr. plot: M_Bagging_FP_MD_NoScaling_PCA_256_mean_1000000
Test set
R2 score: 0.571
MSE: 0.457
Predicted pIC50
Actual pIC50
3
4
5
6
7
8
9
10

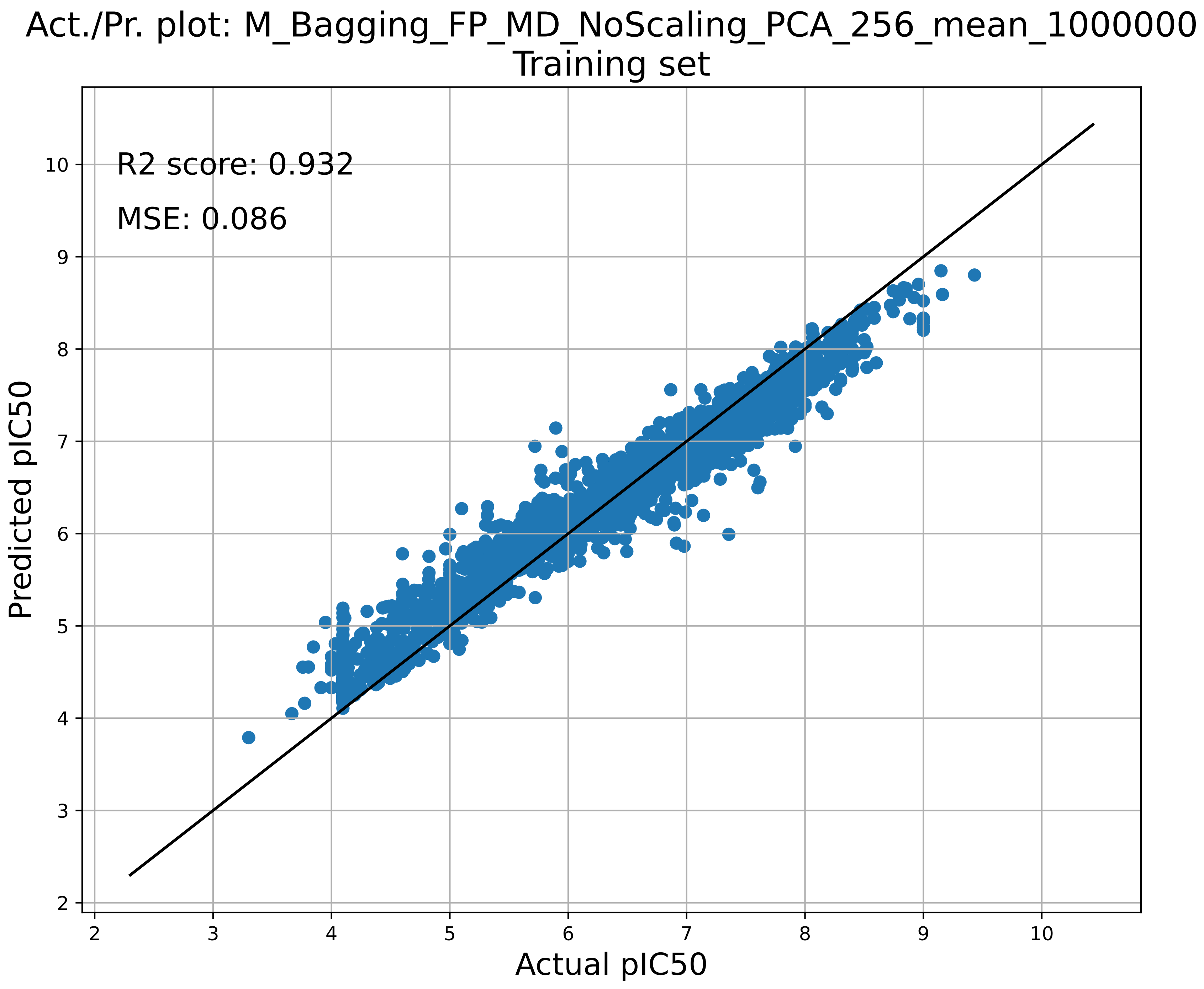
Act./Pr. plot: M_Bagging_FP_MD_NoScaling_PCA_256_mean_1000000
Training set
R2 score: 0.932
MSE: 0.086
Predicted pIC50
Actual pIC50
2
3
4
5
6
7
8
9
10

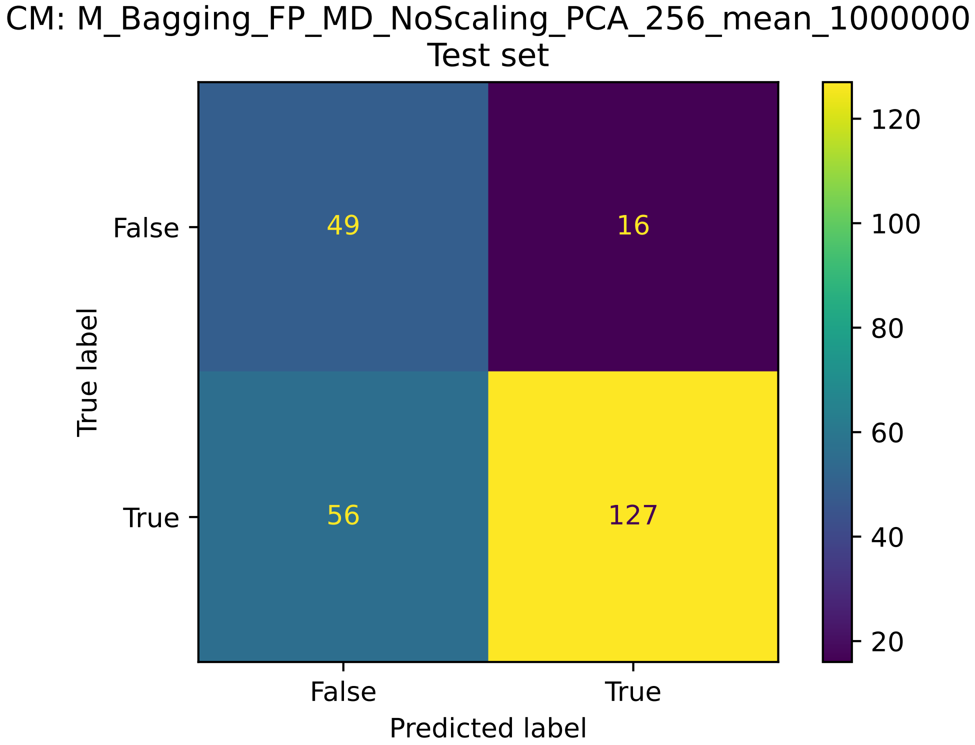
CM: M_Bagging_FP_MD_NoScaling_PCA_256_mean_1000000
Test set
True label
Predicted label
False
True
False
True
49
16
56
127
20
40
60
80
100
120

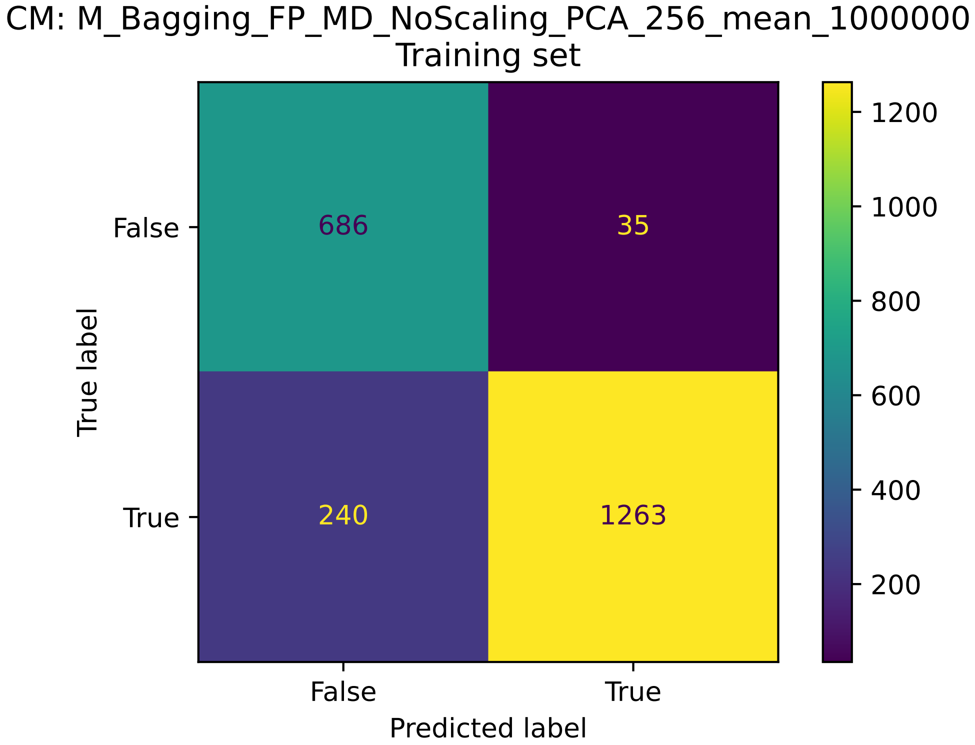

CM: M_Bagging_FP_MD_NoScaling_PCA_256_mean_1000000
Training set
686
35
240
1263
False
True
True label
False
True
Predicted label
1200
1000
800
600
400
200

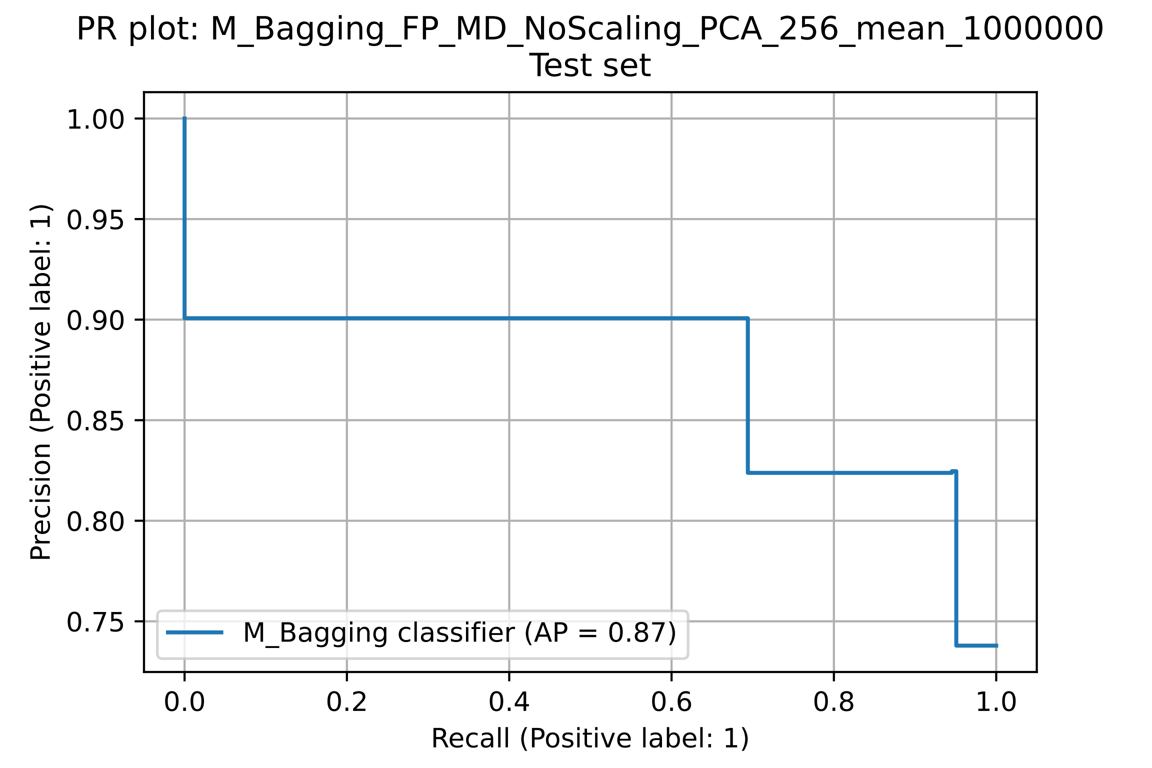
PR plot: M_Bagging_FP_MD_NoScaling_PCA_256_mean_1000000
Test set
Precision (Positive label: 1)
Recall (Positive label: 1)
1.00
0.95
0.90
0.85
0.80
0.75
0.0
0.2
0.4
0.6
0.8
1.0
M_Bagging classifier (AP = 0.87)

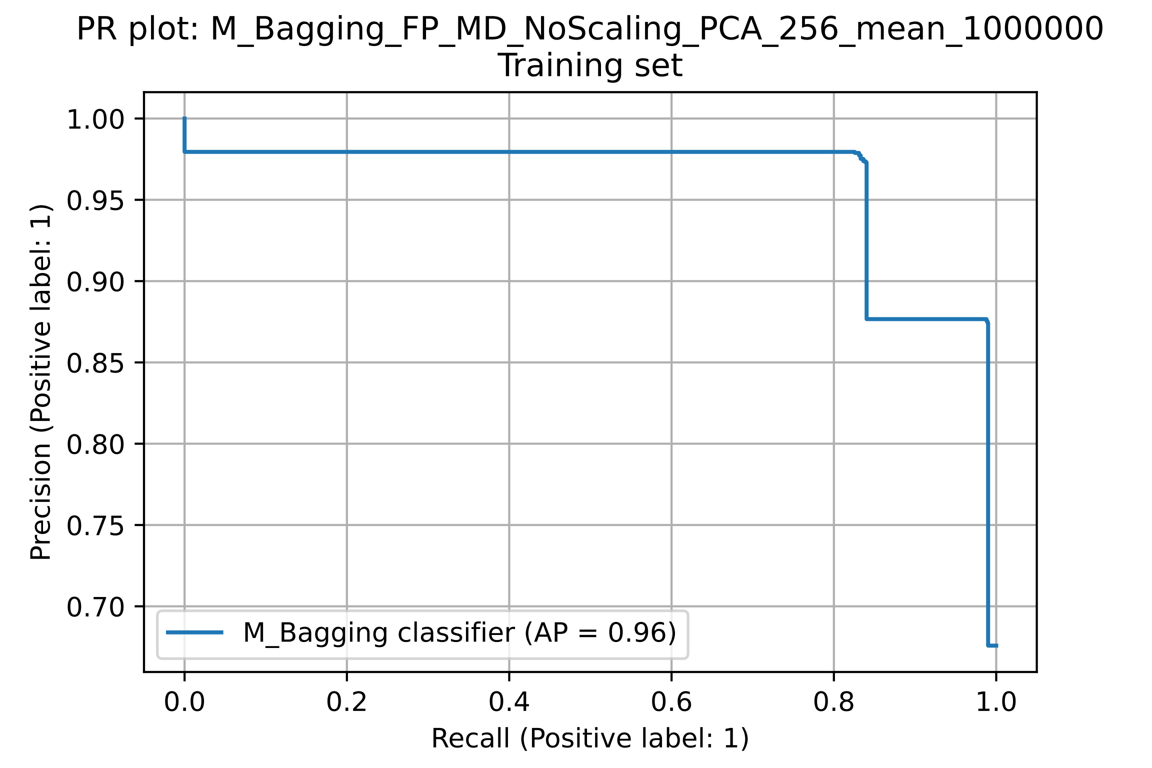
PR plot: M_Bagging_FP_MD_NoScaling_PCA_256_mean_1000000
Training set
Precision (Positive label: 1)
Recall (Positive label: 1)
1.00
0.95
0.90
0.85
0.80
0.75
0.70
0.0
0.2
0.4
0.6
0.8
1.0
M_Bagging classifier (AP = 0.96)

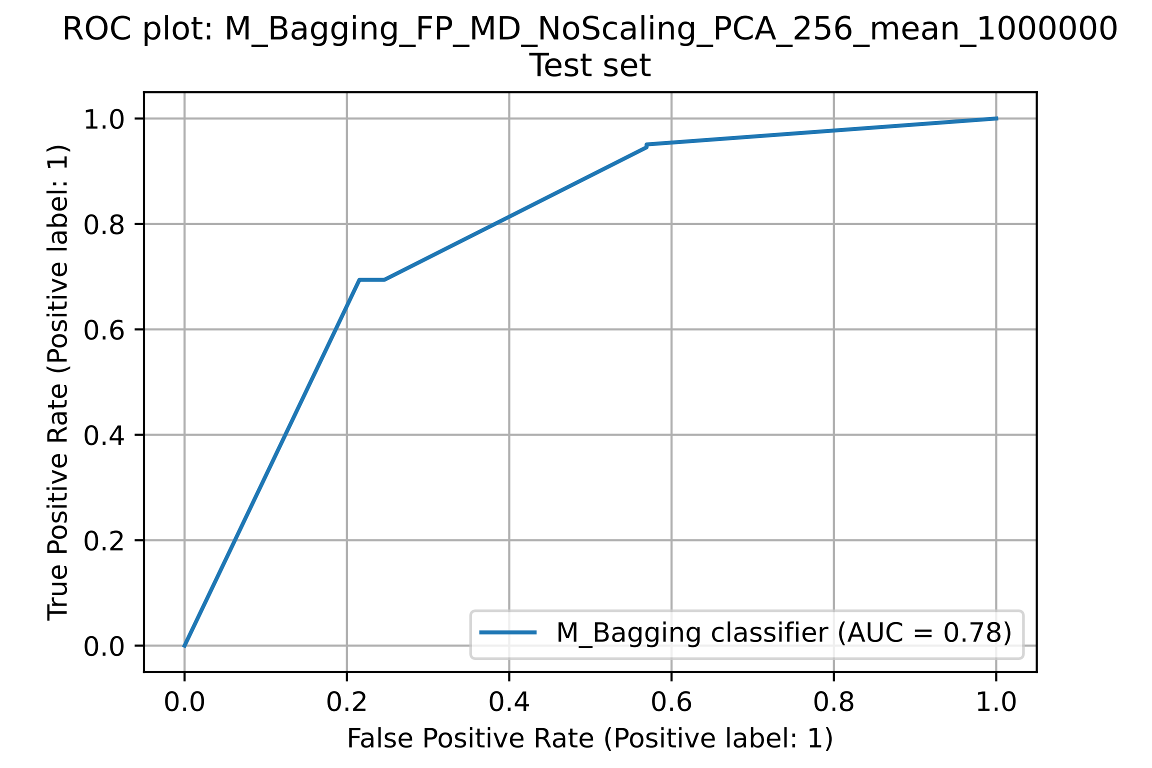
ROC plot: M_Bagging_FP_MD_NoScaling_PCA_256_mean_1000000
Test set
True Positive Rate (Positive label: 1)
False Positive Rate (Positive label: 1)
0.0
0.2
0.4
0.6
0.8
1.0
M_Bagging classifier (AUC = 0.78)

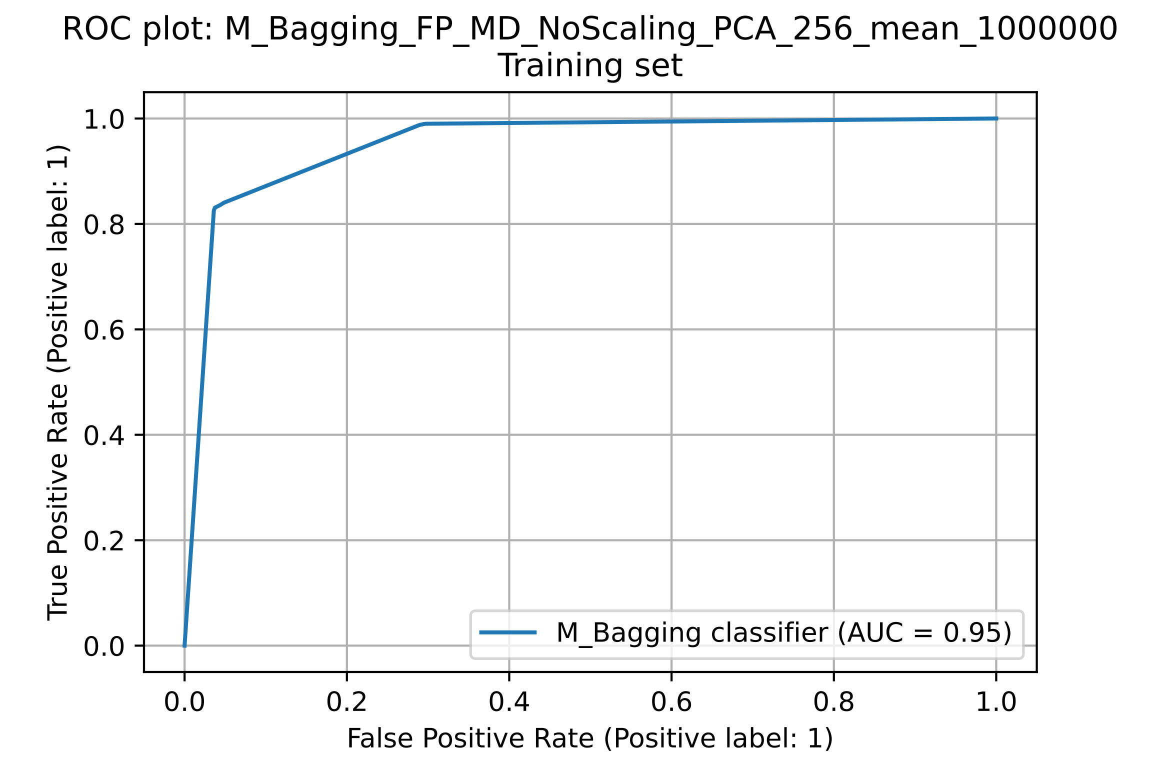
ROC plot: M_Bagging_FP_MD_NoScaling_PCA_256_mean_1000000
Training set
True Positive Rate (Positive label: 1)
False Positive Rate (Positive label: 1)
0.0
0.2
0.4
0.6
0.8
1.0
M_Bagging classifier (AUC = 0.95)

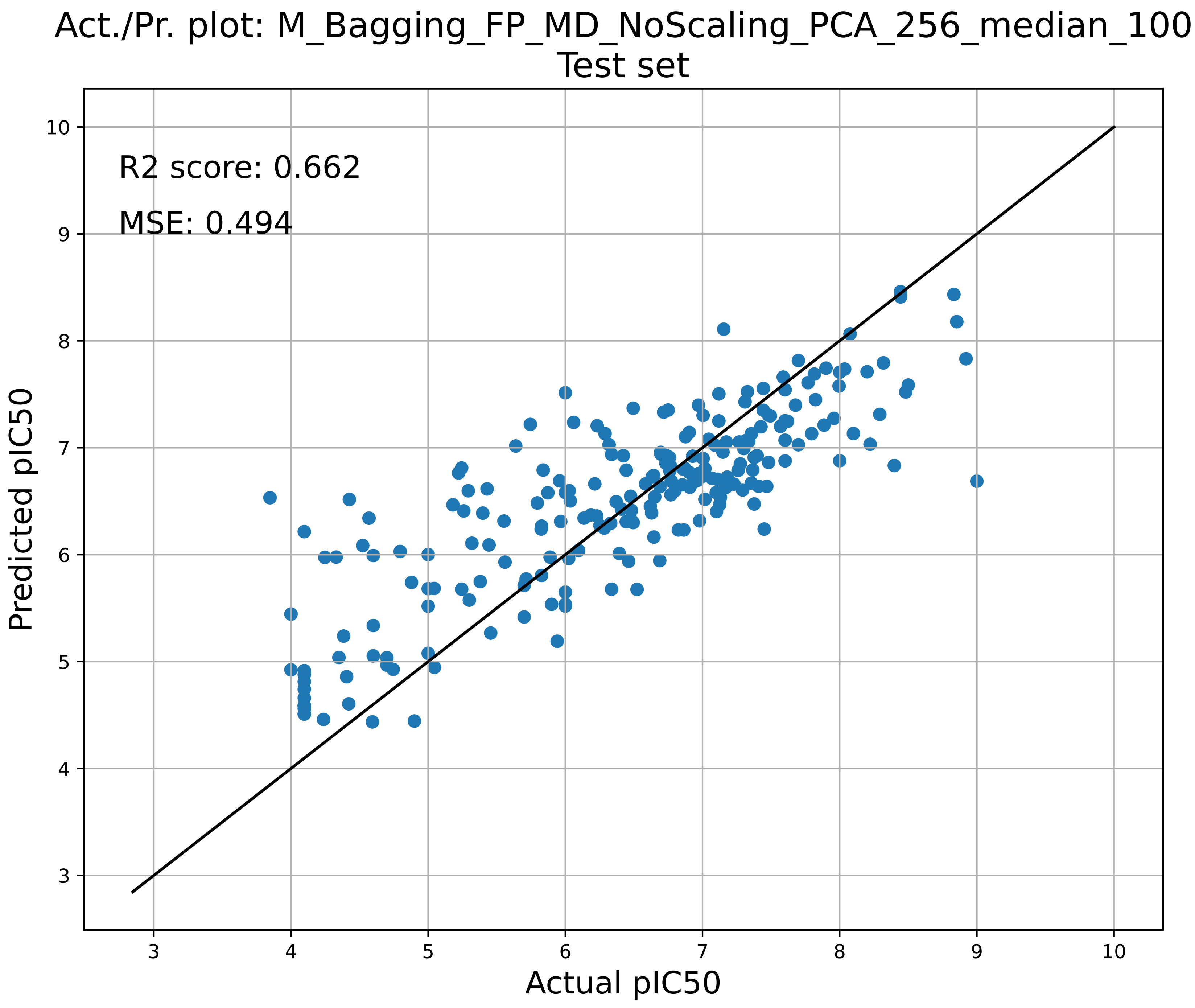

Act./Pr. plot: M_Bagging_FP_MD_NoScaling_PCA_256_median_100
Test set
R2 score: 0.662
MSE: 0.494
Predicted pIC50
Actual pIC50
3
4
5
6
7
8
9
10

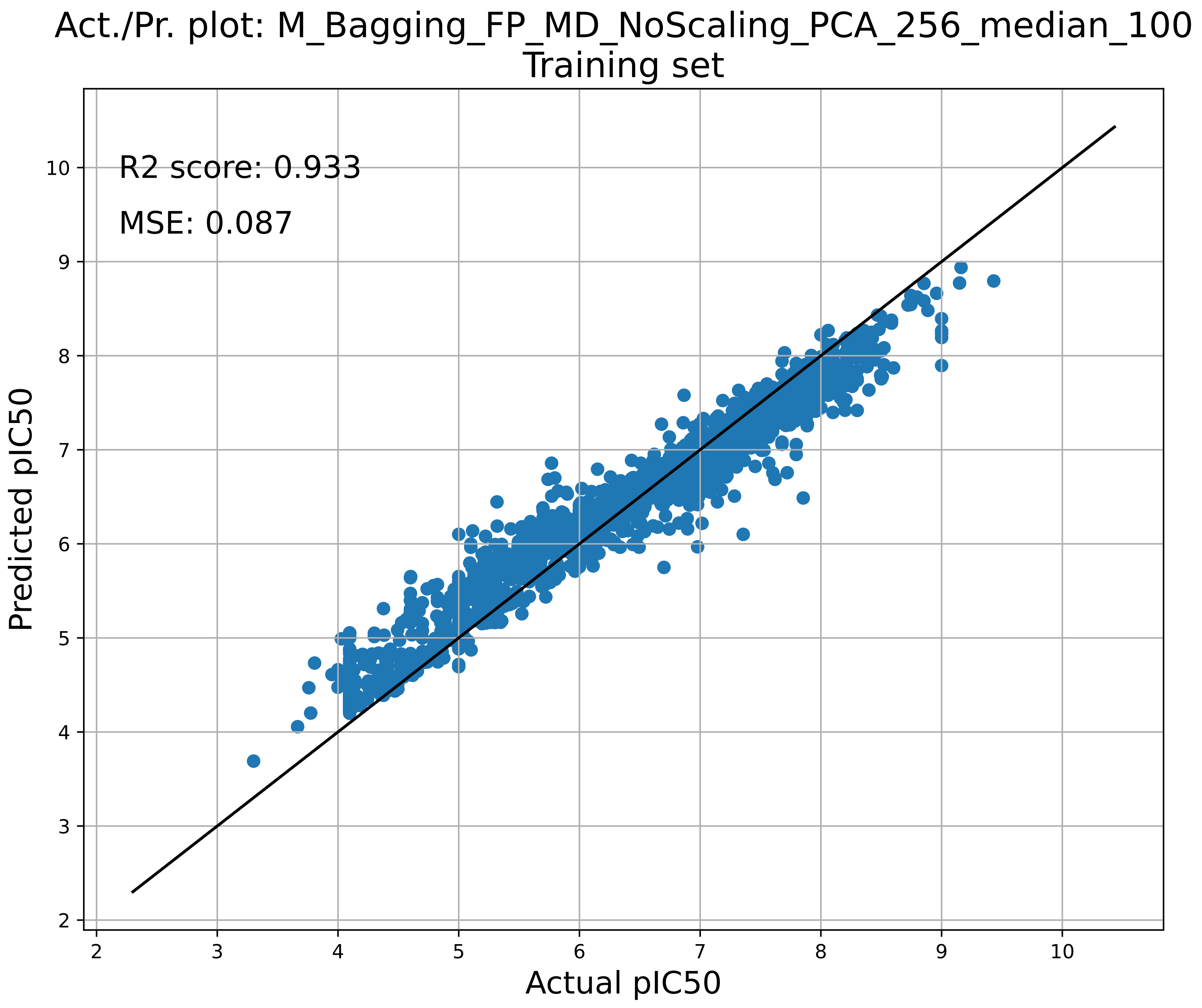
Act./Pr. plot: M_Bagging_FP_MD_NoScaling_PCA_256_median_100
Training set
R2 score: 0.933
MSE: 0.087
Predicted pIC50
Actual pIC50
2
3
4
5
6
7
8
9
10

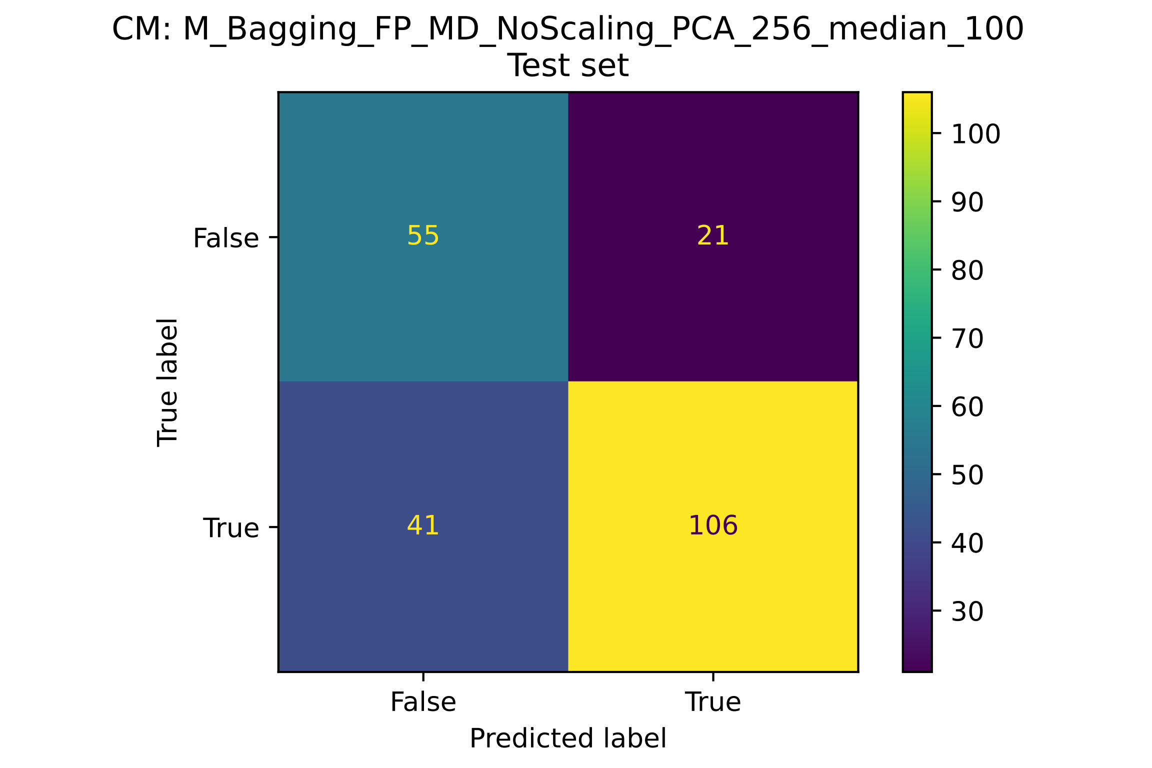
CM: M_Bagging_FP_MD_NoScaling_PCA_256_median_100
Test set
55
21
41
106
False
True
True label
False
True
Predicted label
100
90
80
70
60
50
40
30

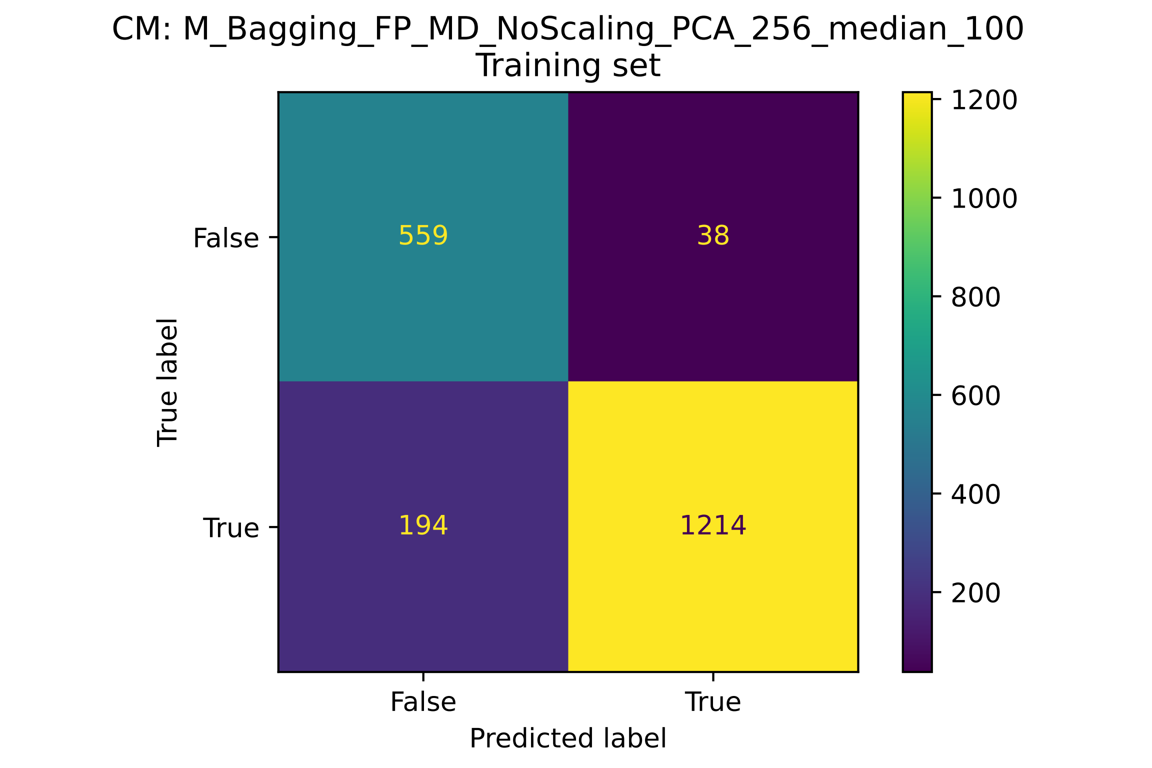
CM: M_Bagging_FP_MD_NoScaling_PCA_256_median_100
Training set
559
38
194
1214
False
True
True label
False
True
Predicted label
1200
1000
800
600
400
200

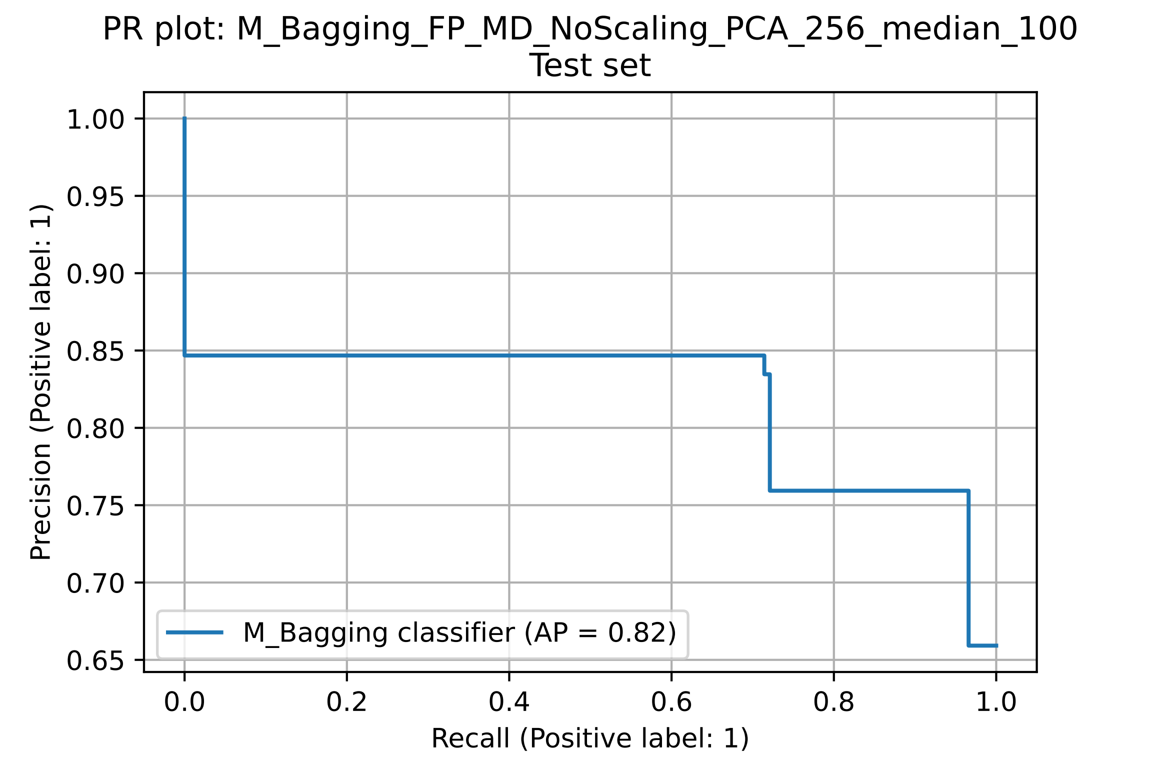
PR plot: M_Bagging_FP_MD_NoScaling_PCA_256_median_100
Test set
Precision (Positive label: 1)
Recall (Positive label: 1)
1.00
0.95
0.90
0.85
0.80
0.75
0.70
0.65
0.0
0.2
0.4
0.6
0.8
1.0
M_Bagging classifier (AP = 0.82)

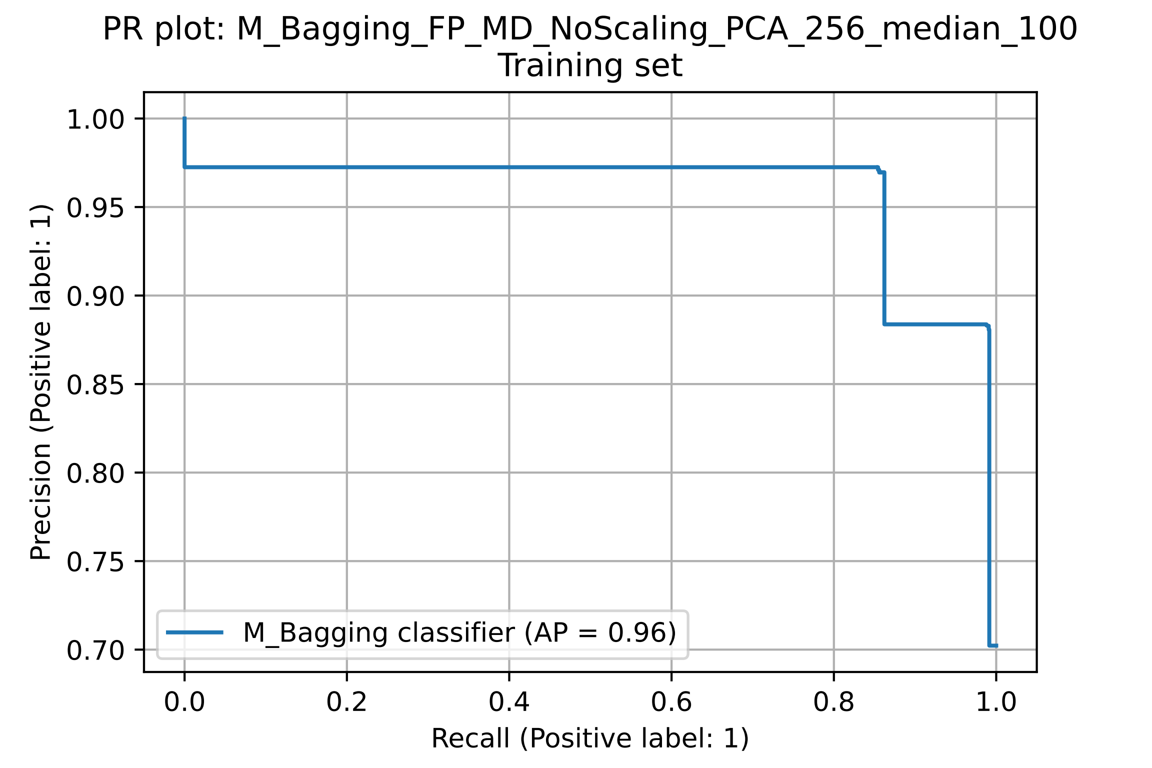

PR plot: M_Bagging_FP_MD_NoScaling_PCA_256_median_100
Training set
Precision (Positive label: 1)
Recall (Positive label: 1)
1.00
0.95
0.90
0.85
0.80
0.75
0.70
0.0
0.2
0.4
0.6
0.8
1.0
M_Bagging classifier (AP = 0.96)

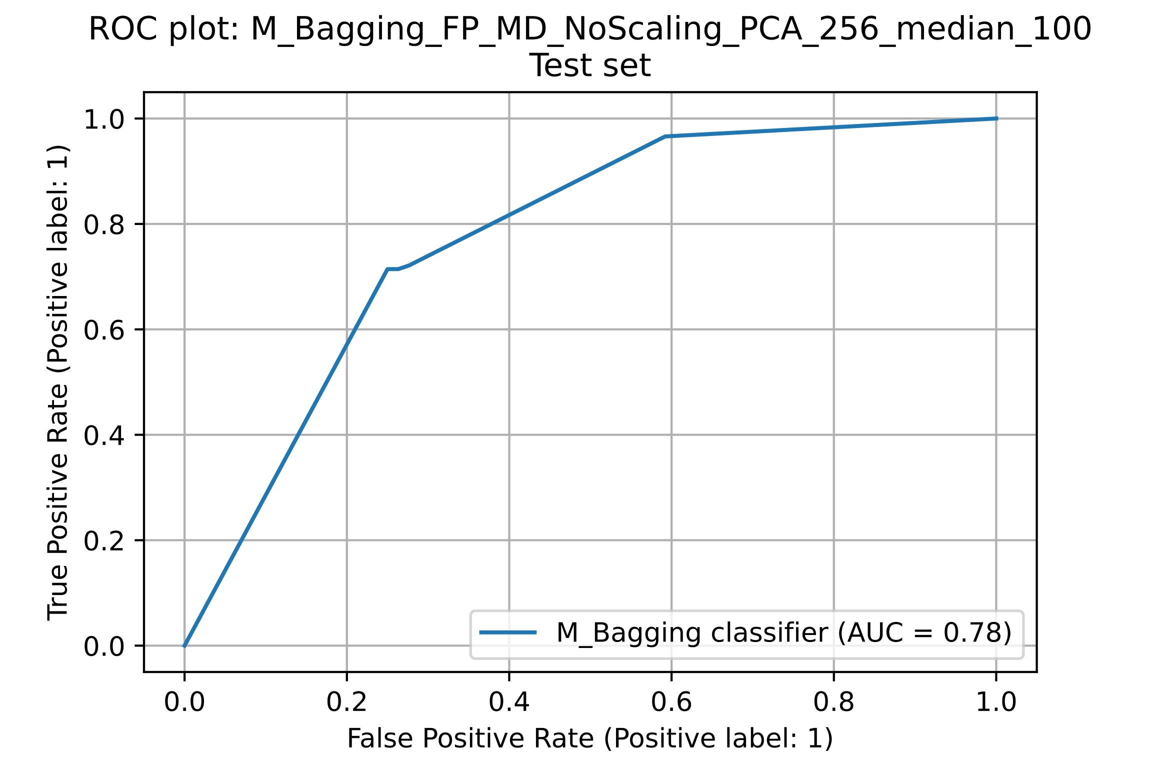
ROC plot: M_Bagging_FP_MD_NoScaling_PCA_256_median_100
Test set
True Positive Rate (Positive label: 1)
False Positive Rate (Positive label: 1)
0.0
0.2
0.4
0.6
0.8
1.0
M_Bagging classifier (AUC = 0.78)

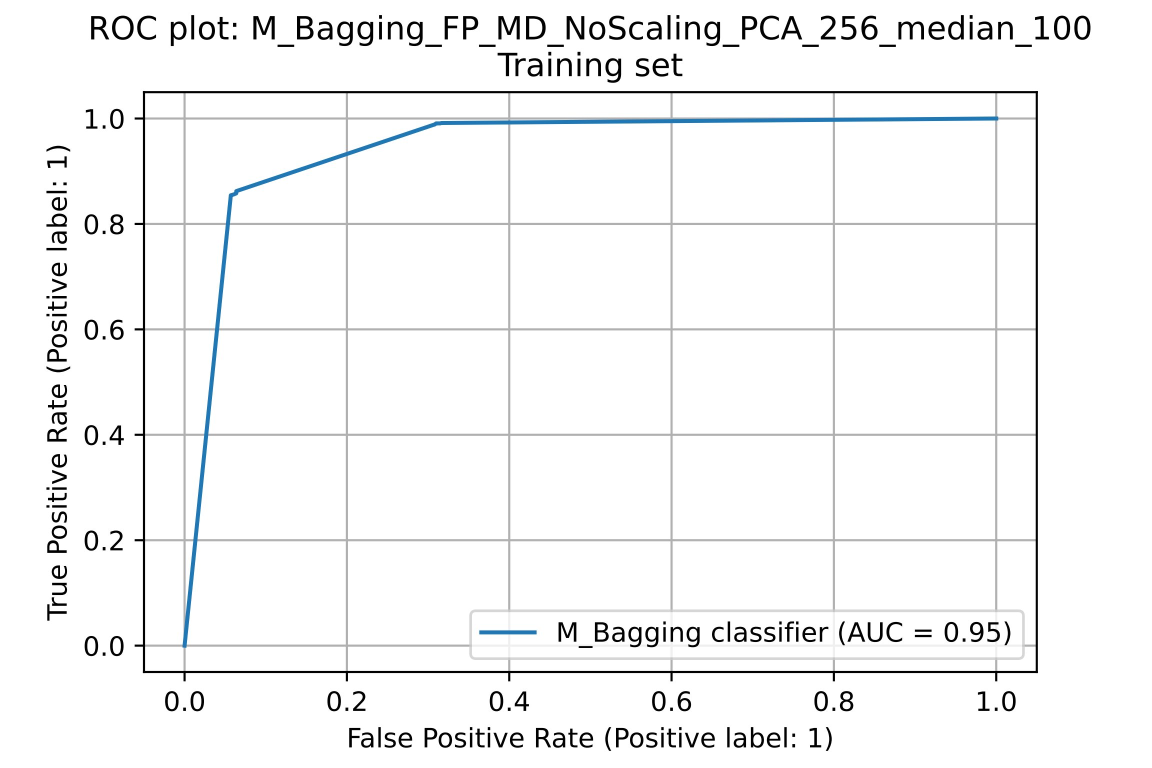
ROC plot: M_Bagging_FP_MD_NoScaling_PCA_256_median_100
Training set
True Positive Rate (Positive label: 1)
False Positive Rate (Positive label: 1)
0.0
0.2
0.4
0.6
0.8
1.0
M_Bagging classifier (AUC = 0.95)

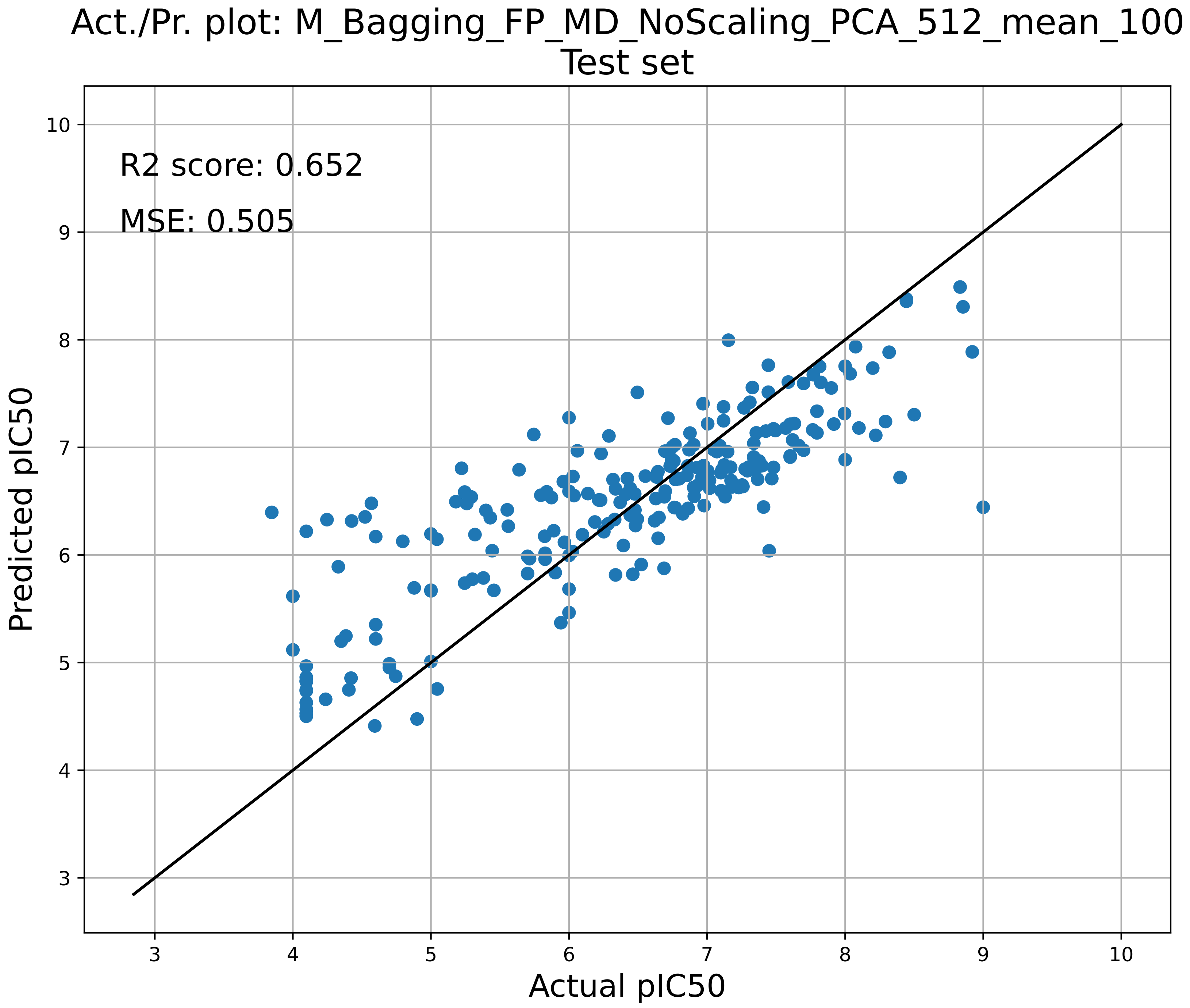
Act./Pr. plot: M_Bagging_FP_MD_NoScaling_PCA_512_mean_100
Test set
R2 score: 0.652
MSE: 0.505
Predicted pIC50
Actual pIC50
3
4
5
6
7
8
9
10
3
4
5
6
7
8
9
10

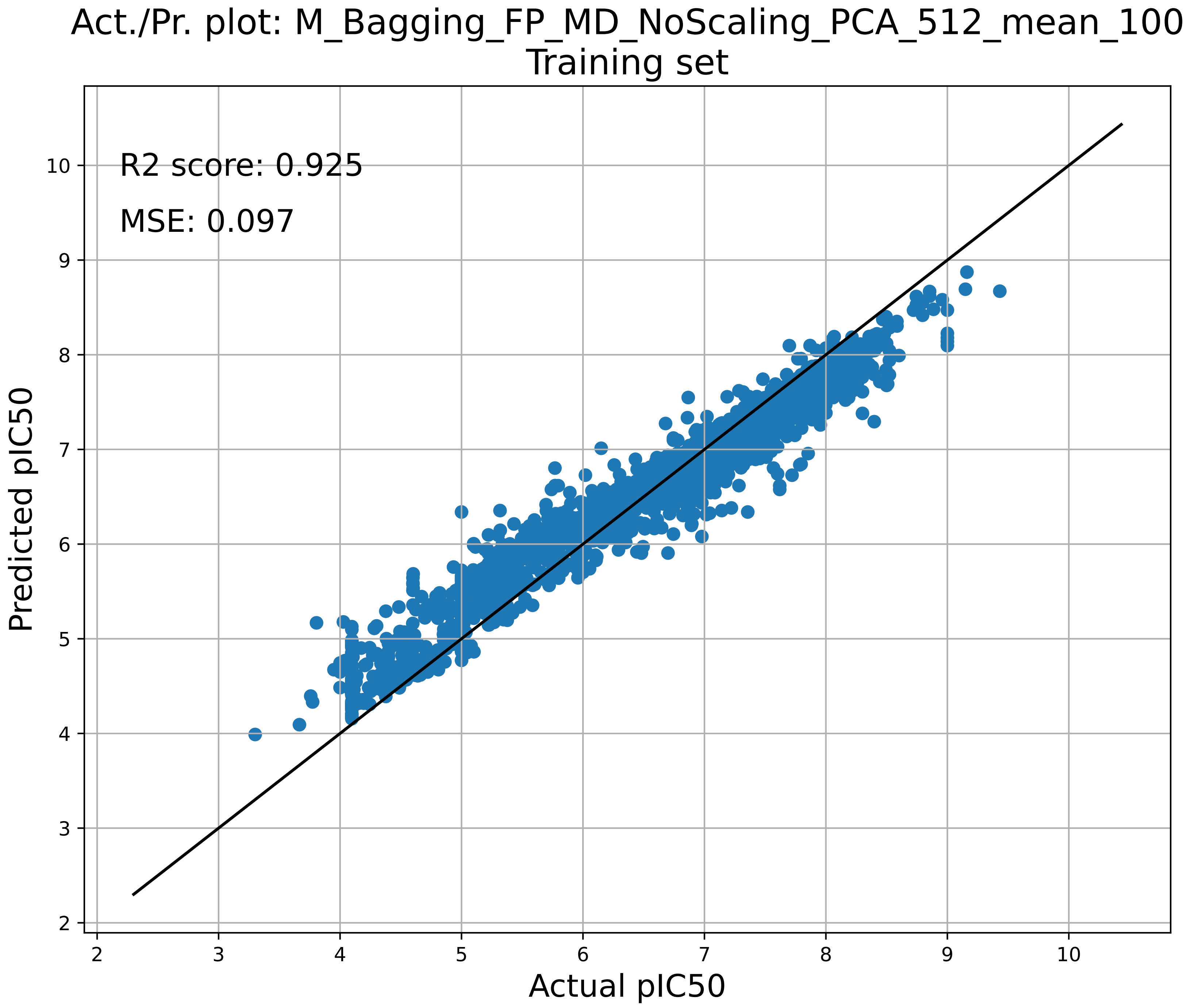

Act./Pr. plot: M_Bagging_FP_MD_NoScaling_PCA_512_mean_100
Training set
R2 score: 0.925
MSE: 0.097
Predicted pIC50
Actual pIC50
2
3
4
5
6
7
8
9
10

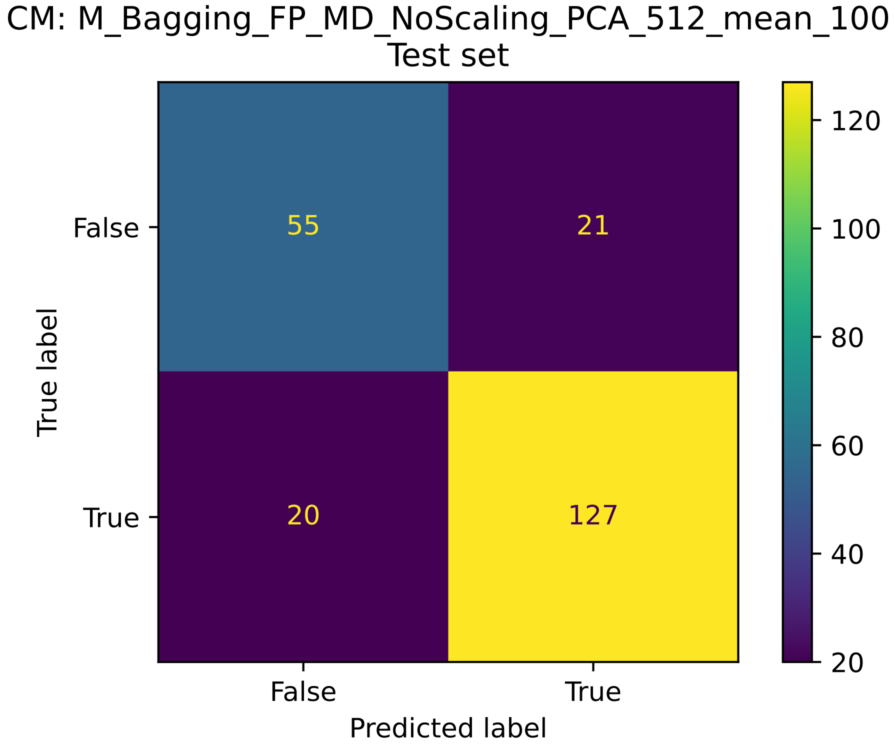
CM: M_Bagging_FP_MD_NoScaling_PCA_512_mean_100
Test set
True label
False
True
55
21
20
127
Predicted label
False
True
120
100
80
60
40
20

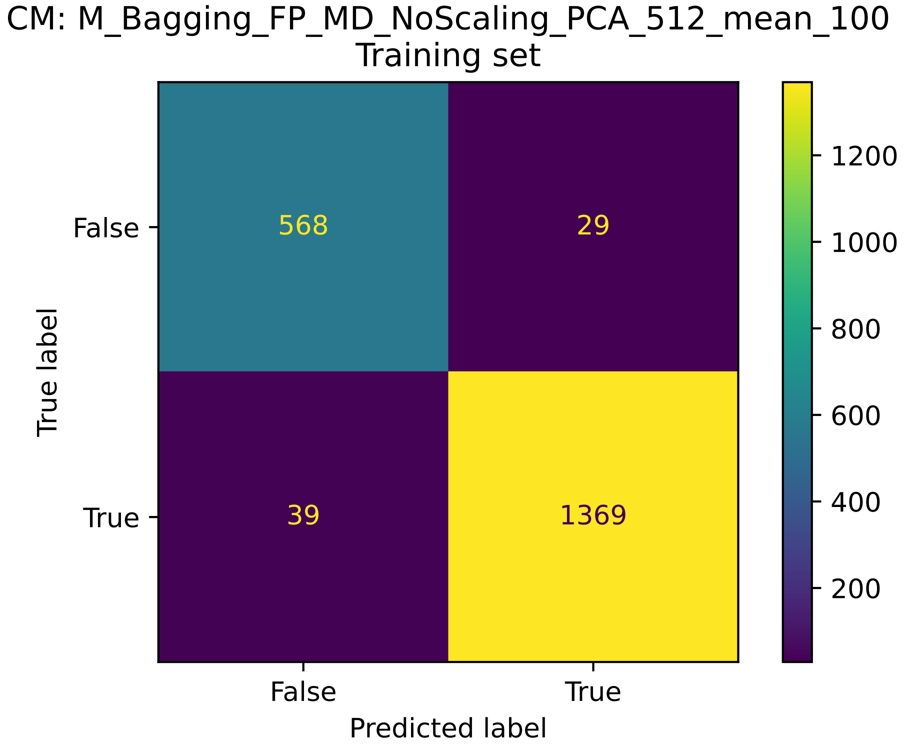
CM: M_Bagging_FP_MD_NoScaling_PCA_512_mean_100
Training set
True label
False
True
568
29
39
1369
Predicted label
False
True
1200
1000
800
600
400
200

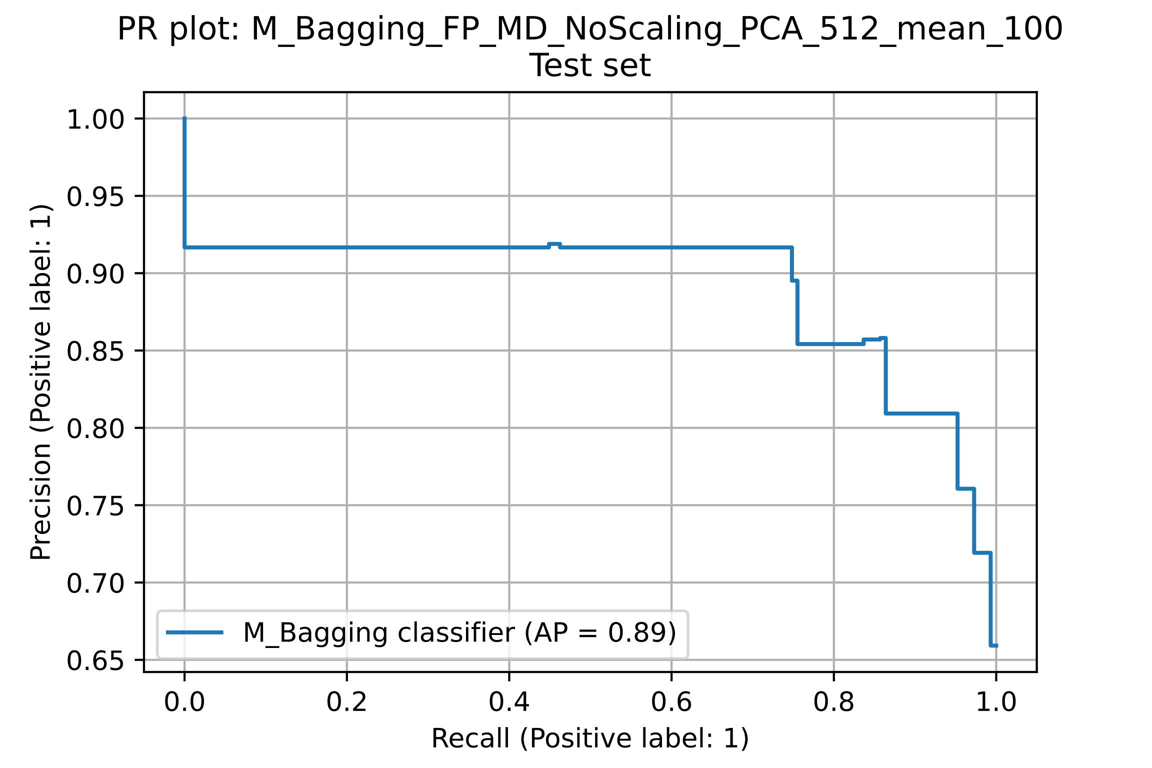
PR plot: M_Bagging_FP_MD_NoScaling_PCA_512_mean_100
Test set
Precision (Positive label: 1)
Recall (Positive label: 1)
1.00
0.95
0.90
0.85
0.80
0.75
0.70
0.65
0.0
0.2
0.4
0.6
0.8
1.0
M_Bagging classifier (AP = 0.89)

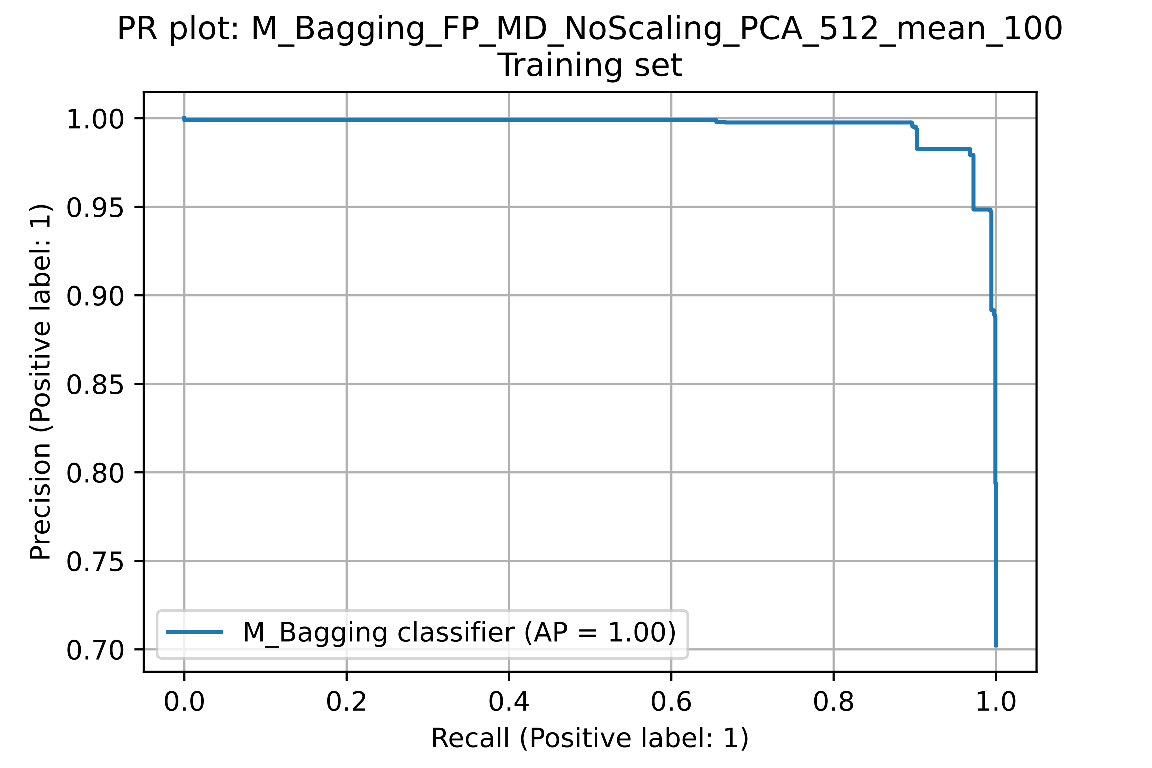
PR plot: M_Bagging_FP_MD_NoScaling_PCA_512_mean_100
Training set
Precision (Positive label: 1)
Recall (Positive label: 1)
M_Bagging classifier (AP = 1.00)
1.00
0.95
0.90
0.85
0.80
0.75
0.70
0.0
0.2
0.4
0.6
0.8
1.0

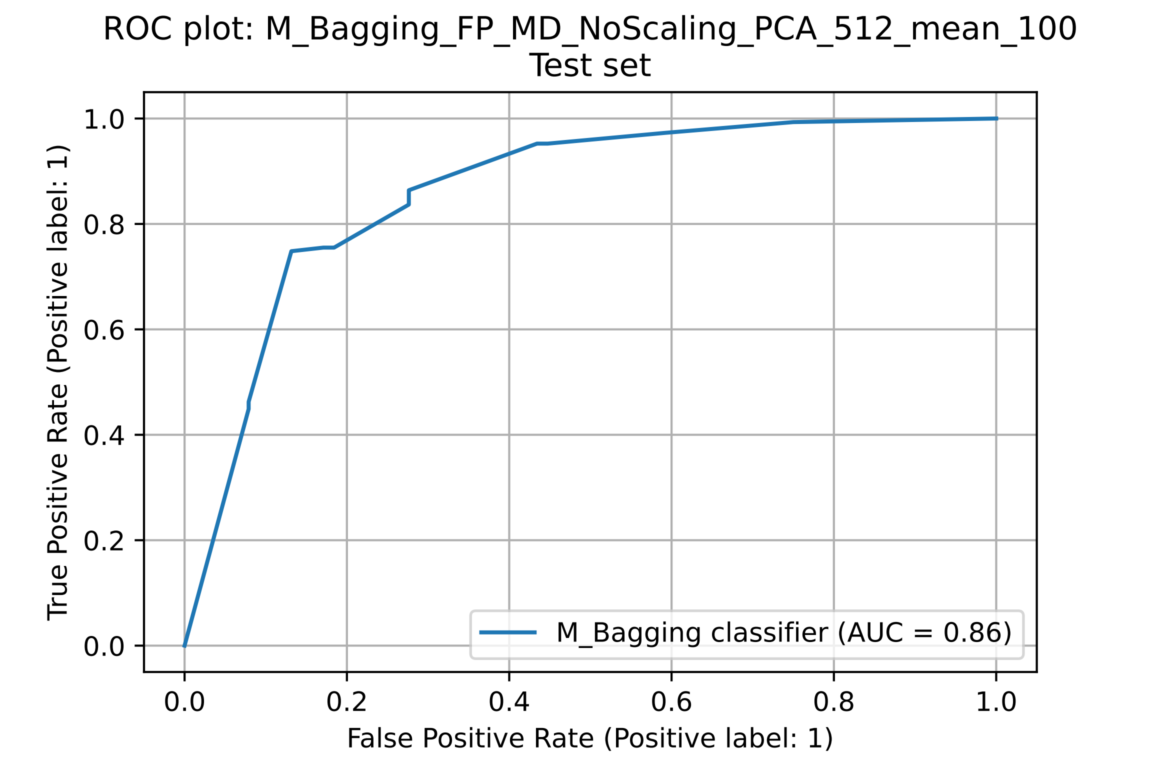

ROC plot: M_Bagging_FP_MD_NoScaling_PCA_512_mean_100
Test set
True Positive Rate (Positive label: 1)
False Positive Rate (Positive label: 1)
0.0
0.2
0.4
0.6
0.8
1.0
M_Bagging classifier (AUC = 0.86)

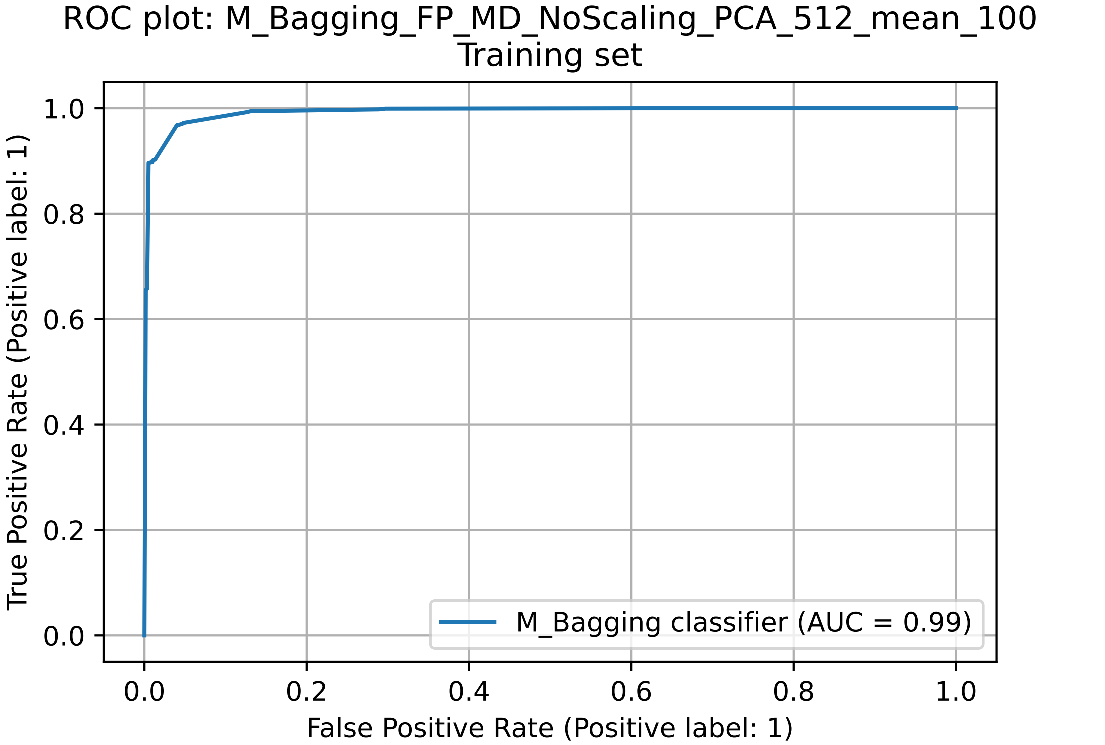

ROC plot: M_Bagging_FP_MD_NoScaling_PCA_512_mean_100
Training set
1.0
0.8
0.6
0.4
0.2
0.0
True Positive Rate (Positive label: 1)
0.0
0.2
0.4
0.6
0.8
1.0
False Positive Rate (Positive label: 1)
M_Bagging classifier (AUC = 0.99)

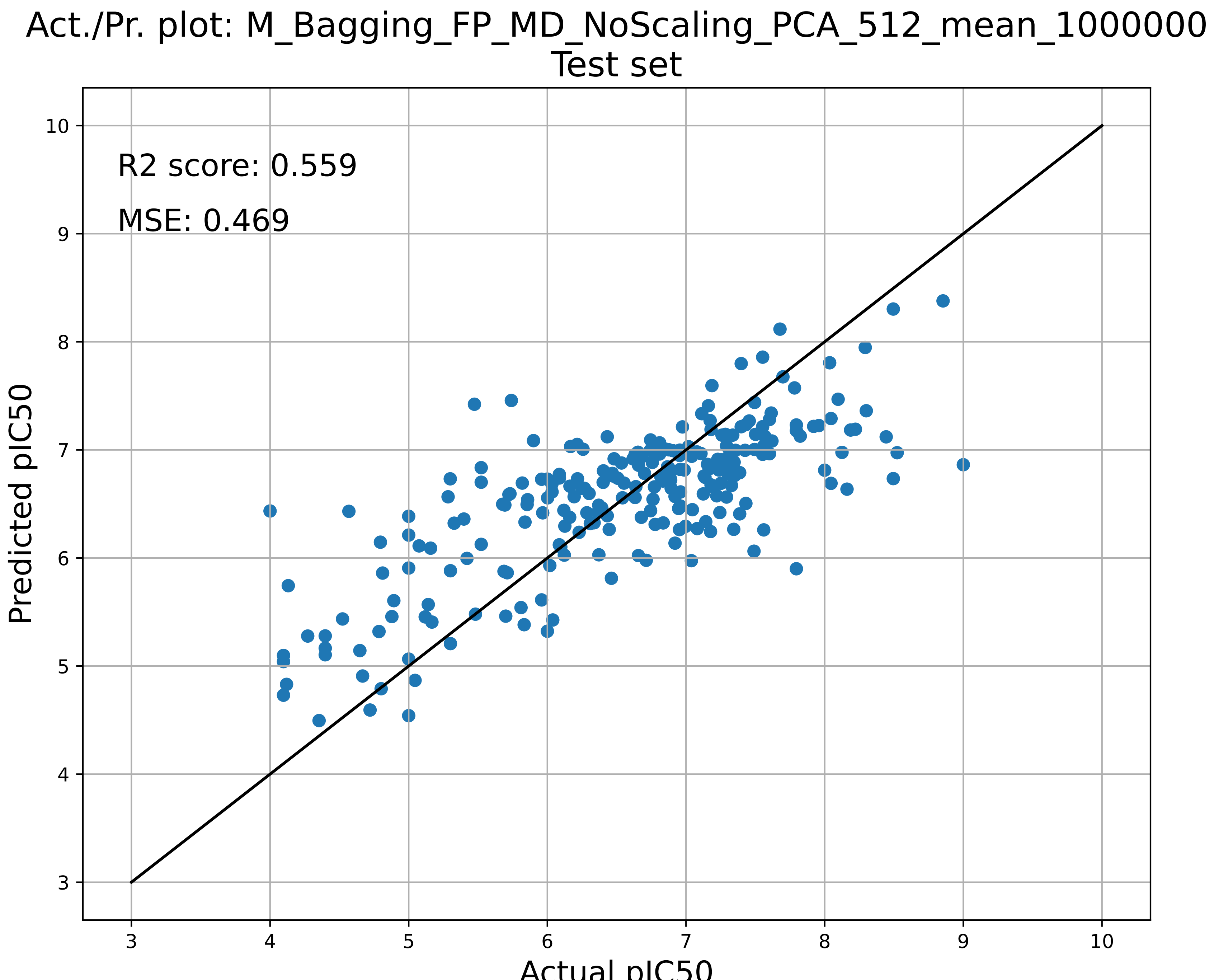

Act./Pr. plot: M_Bagging_FP_MD_NoScaling_PCA_512_mean_1000000
Test set
R2 score: 0.559
MSE: 0.469
Predicted pIC50
Actual pIC50
3
4
5
6
7
8
9
10

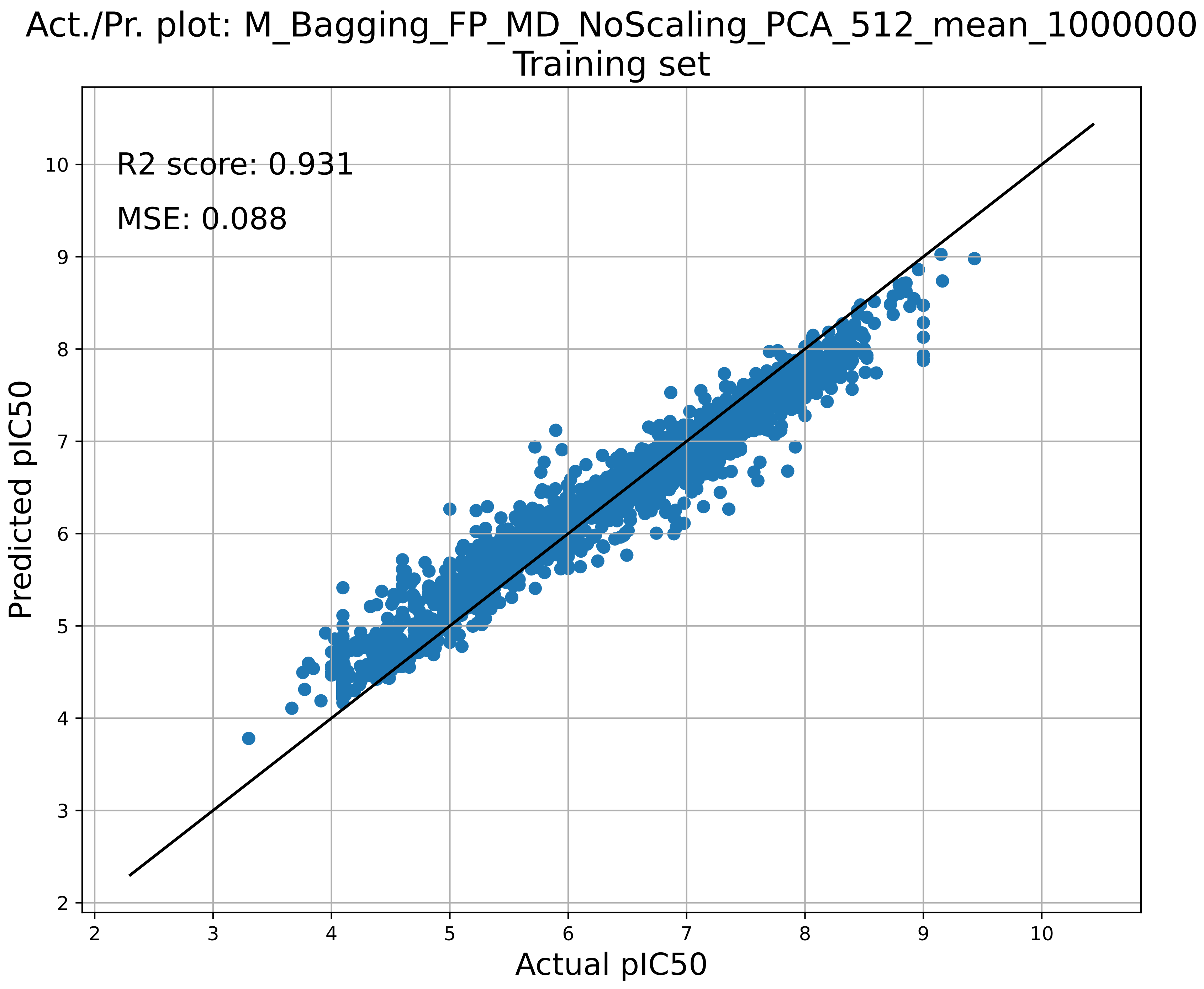

Act./Pr. plot: M_Bagging_FP_MD_NoScaling_PCA_512_mean_1000000
Training set
R2 score: 0.931
MSE: 0.088
Predicted pIC50
Actual pIC50
2
3
4
5
6
7
8
9
10

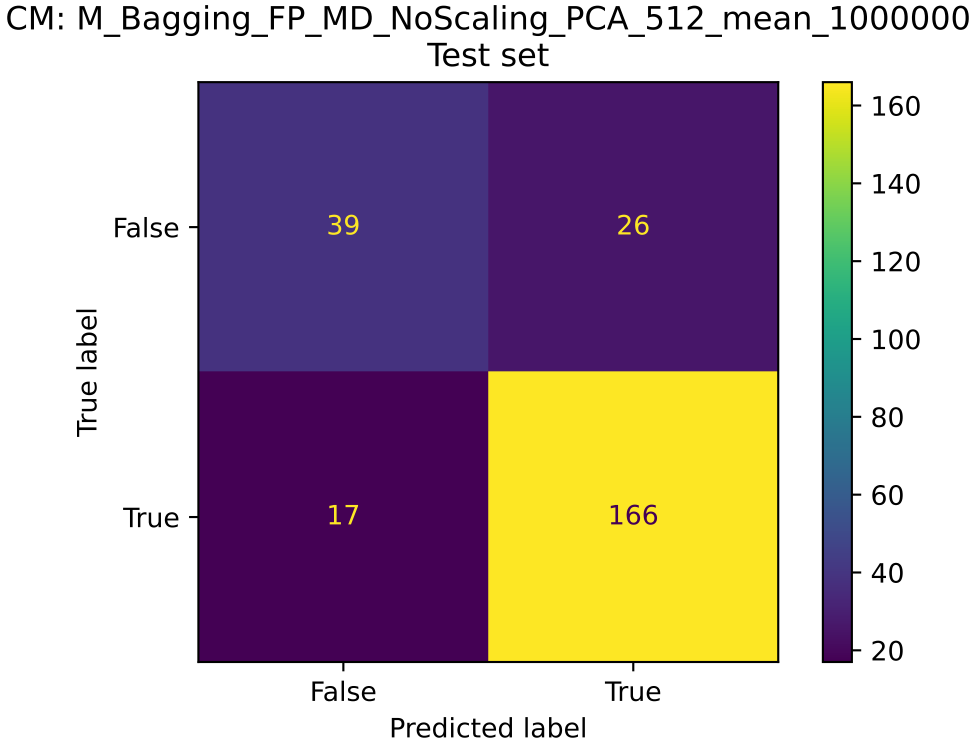
CM: M_Bagging_FP_MD_NoScaling_PCA_512_mean_1000000
Test set
39
26
17
166
False
True
True label
False
True
Predicted label
160
140
120
100
80
60
40
20

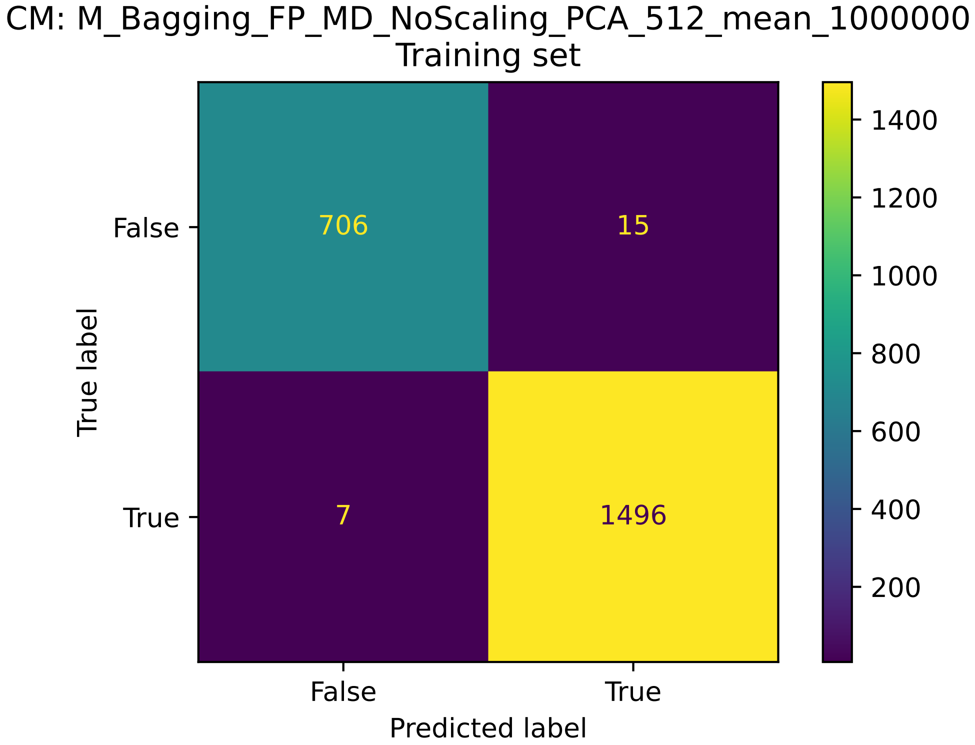

CM: M_Bagging_FP_MD_NoScaling_PCA_512_mean_1000000
Training set
706
15
7
1496
False
True
True label
False
True
Predicted label
1400
1200
1000
800
600
400
200

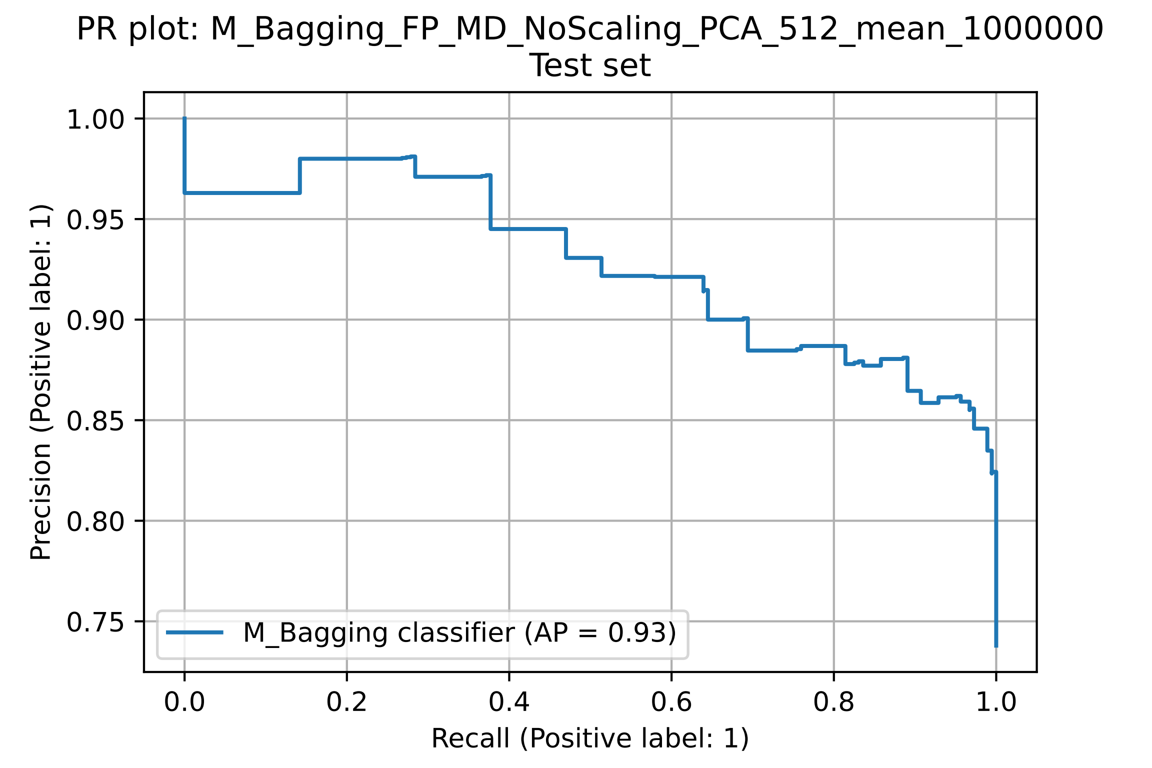
PR plot: M_Bagging_FP_MD_NoScaling_PCA_512_mean_1000000
Test set
Precision (Positive label: 1)
Recall (Positive label: 1)
1.00
0.95
0.90
0.85
0.80
0.75
0.0
0.2
0.4
0.6
0.8
1.0
M_Bagging classifier (AP = 0.93)

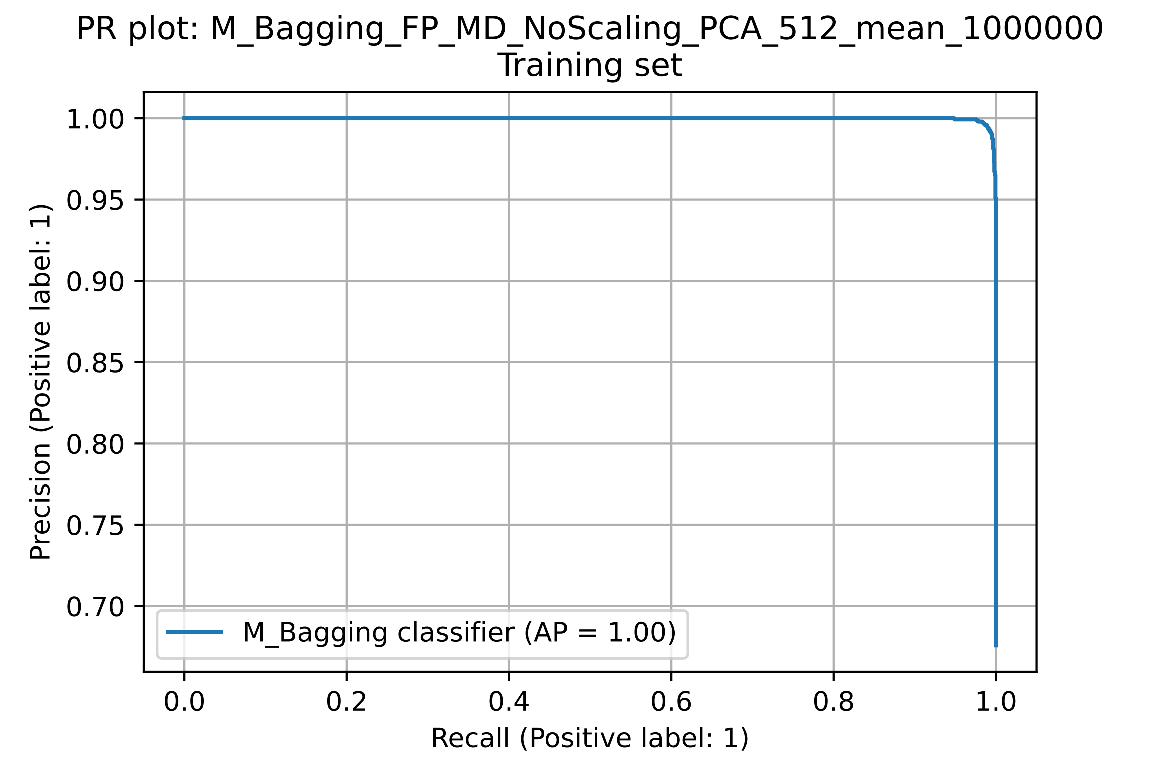

PR plot: M_Bagging_FP_MD_NoScaling_PCA_512_mean_1000000
Training set
Precision (Positive label: 1)
Recall (Positive label: 1)
1.00
0.95
0.90
0.85
0.80
0.75
0.70
0.0
0.2
0.4
0.6
0.8
1.0
M_Bagging classifier (AP = 1.00)

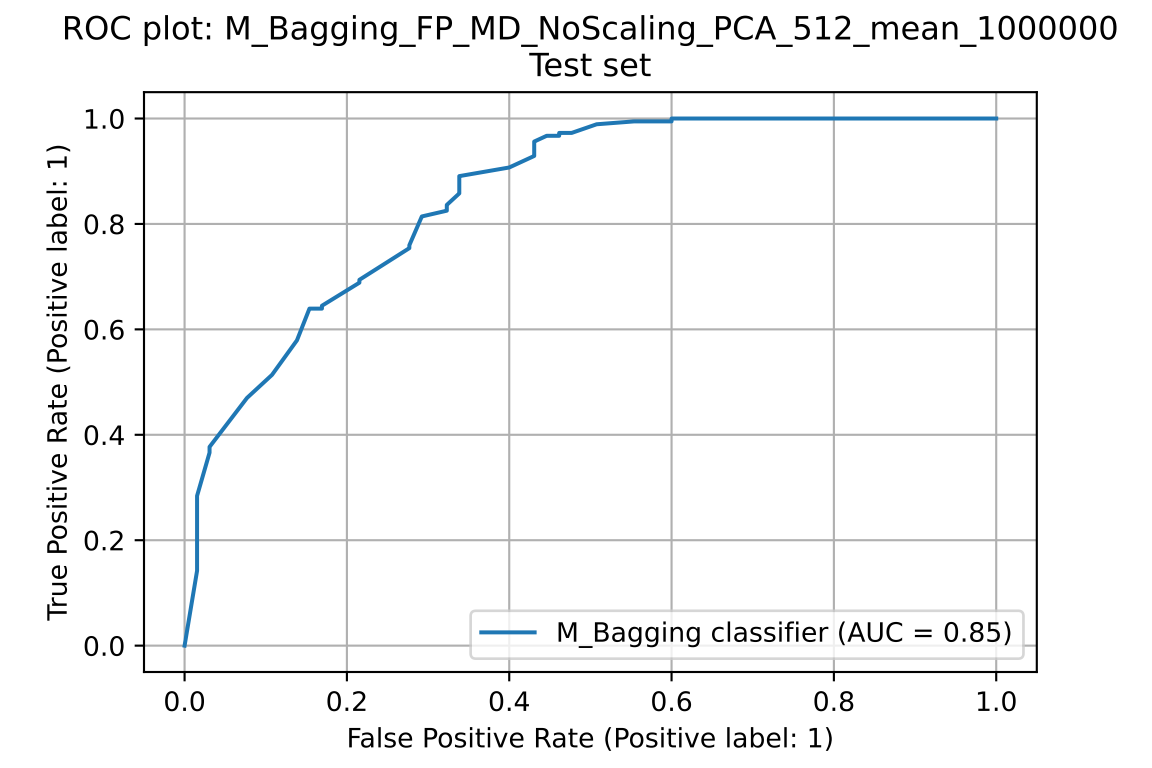

ROC plot: M_Bagging_FP_MD_NoScaling_PCA_512_mean_1000000
Test set
True Positive Rate (Positive label: 1)
False Positive Rate (Positive label: 1)
0.0
0.2
0.4
0.6
0.8
1.0
M_Bagging classifier (AUC = 0.85)

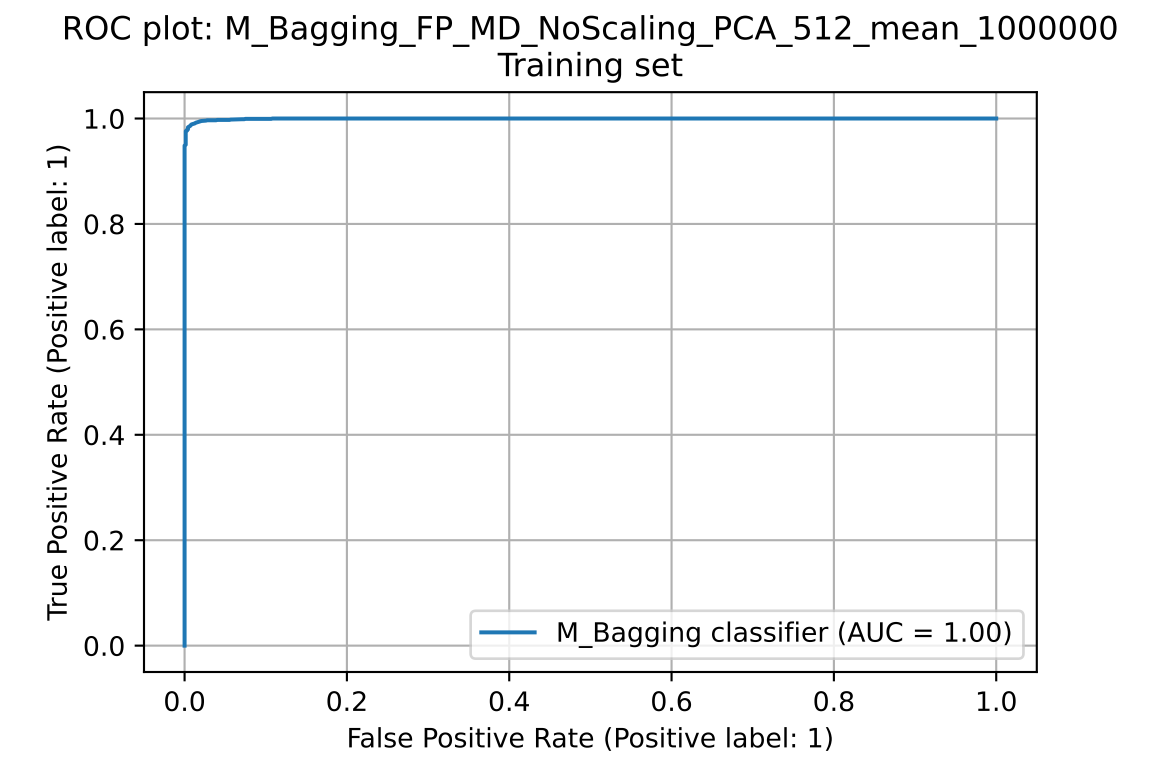
ROC plot: M_Bagging_FP_MD_NoScaling_PCA_512_mean_1000000
Training set
True Positive Rate (Positive label: 1)
False Positive Rate (Positive label: 1)
0.0
0.2
0.4
0.6
0.8
1.0
M_Bagging classifier (AUC = 1.00)

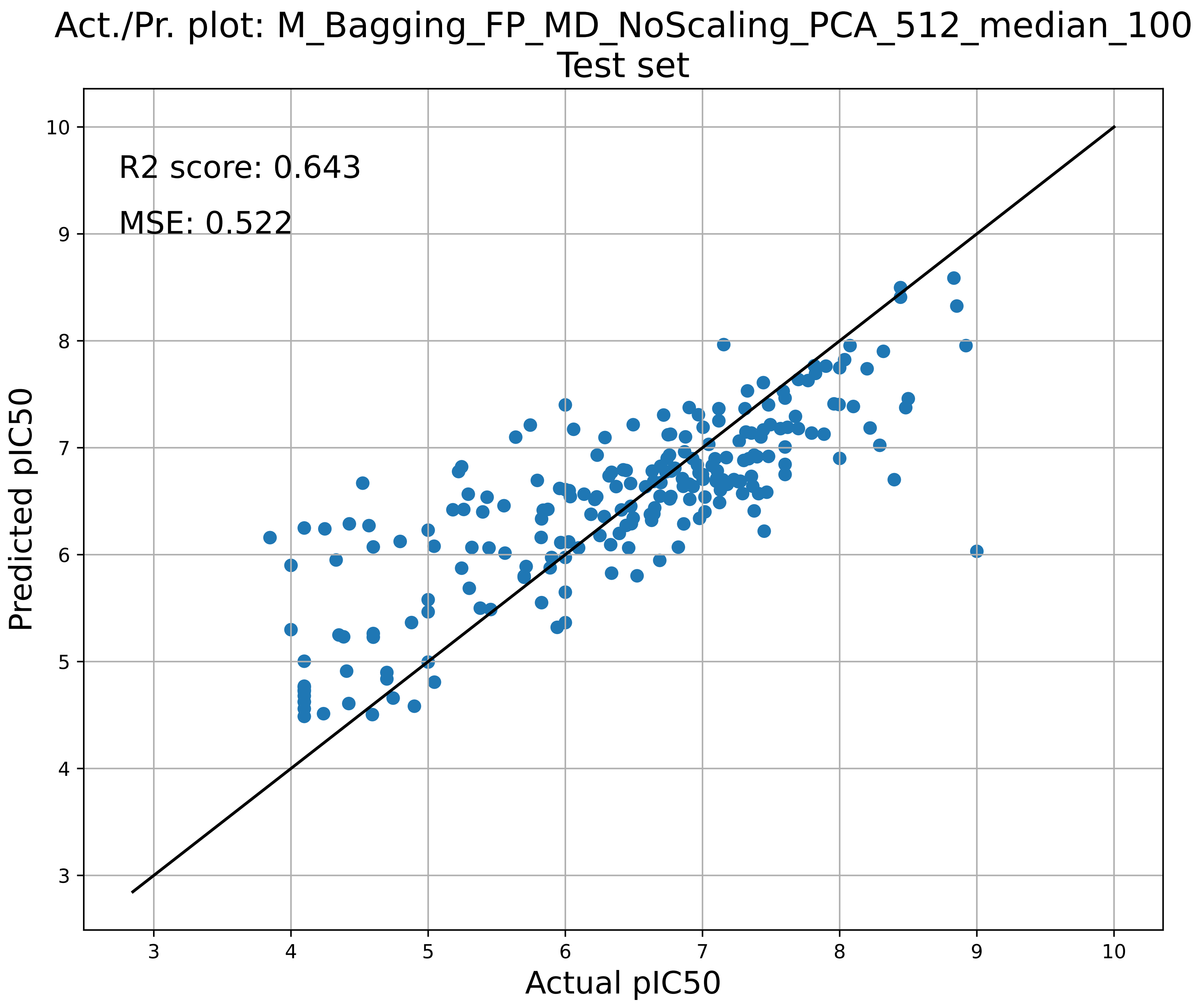

Act./Pr. plot: M_Bagging_FP_MD_NoScaling_PCA_512_median_100
Test set
R2 score: 0.643
MSE: 0.522
Predicted pIC50
Actual pIC50
3
4
5
6
7
8
9
10

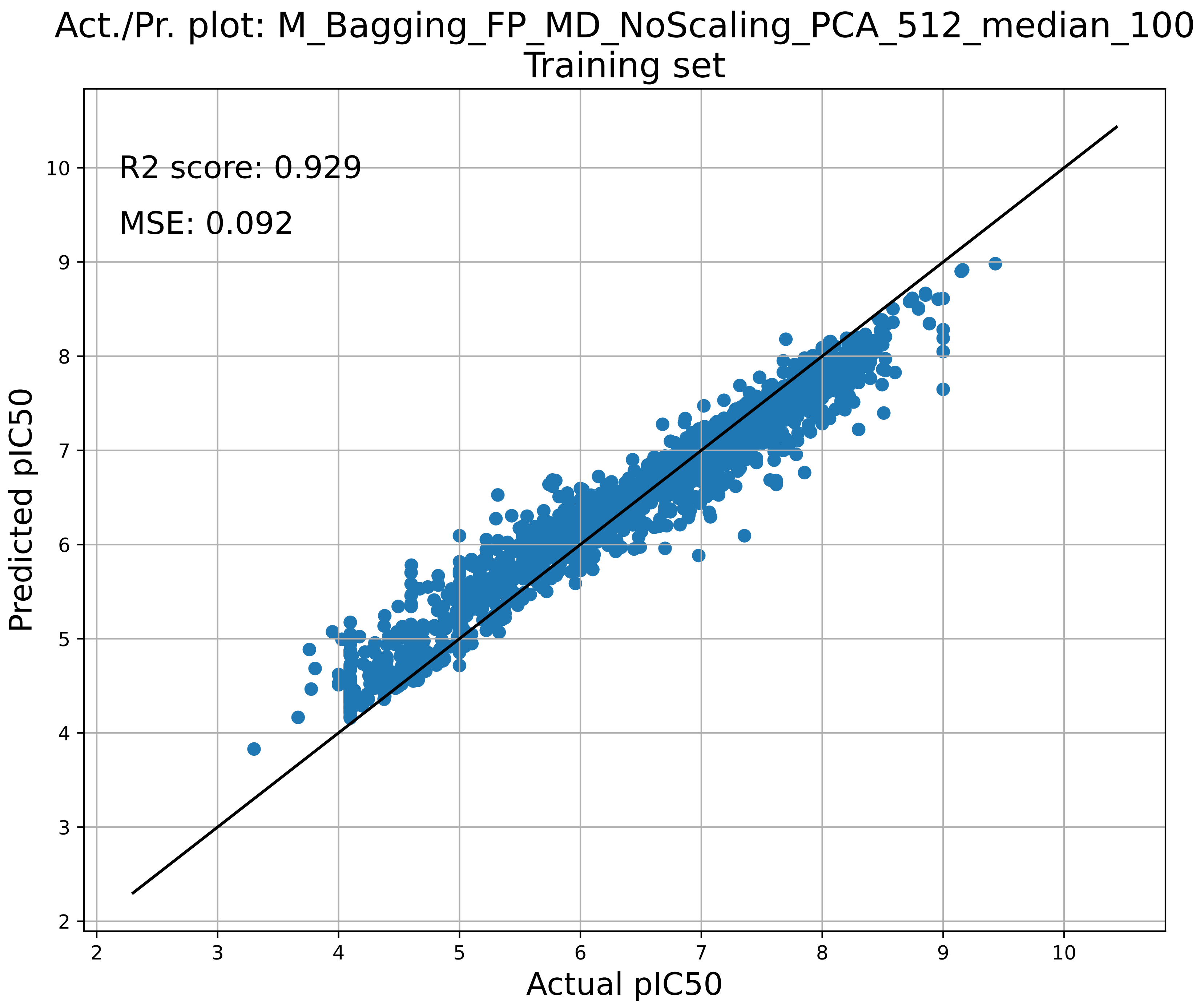

Act./Pr. plot: M_Bagging_FP_MD_NoScaling_PCA_512_median_100
Training set
R2 score: 0.929
MSE: 0.092
Predicted pIC50
Actual pIC50
2
3
4
5
6
7
8
9
10

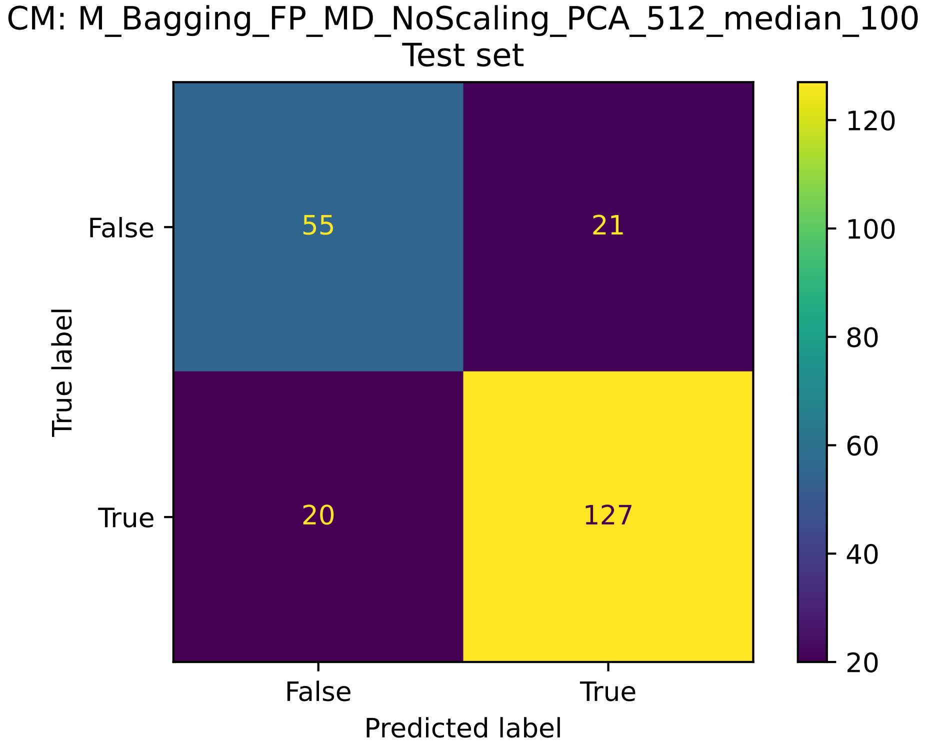
CM: M_Bagging_FP_MD_NoScaling_PCA_512_median_100
Test set
True label
False
True
55
21
20
127
Predicted label
False
True
120
100
80
60
40
20

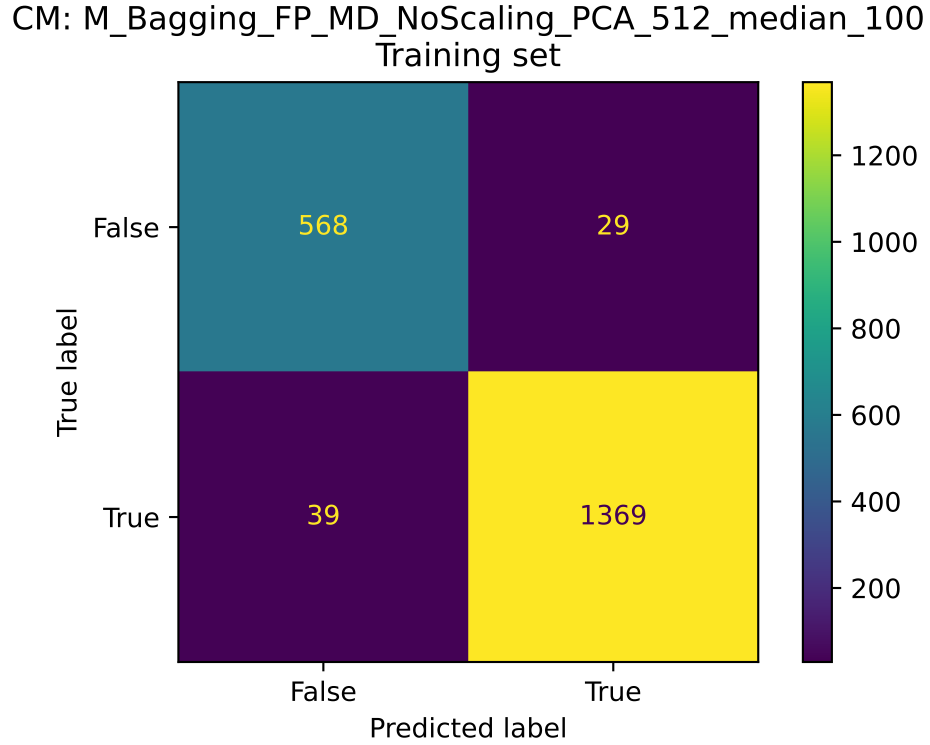
CM: M_Bagging_FP_MD_NoScaling_PCA_512_median_100
Training set
568
29
39
1369
False
True
True label
False
True
Predicted label
1200
1000
800
600
400
200

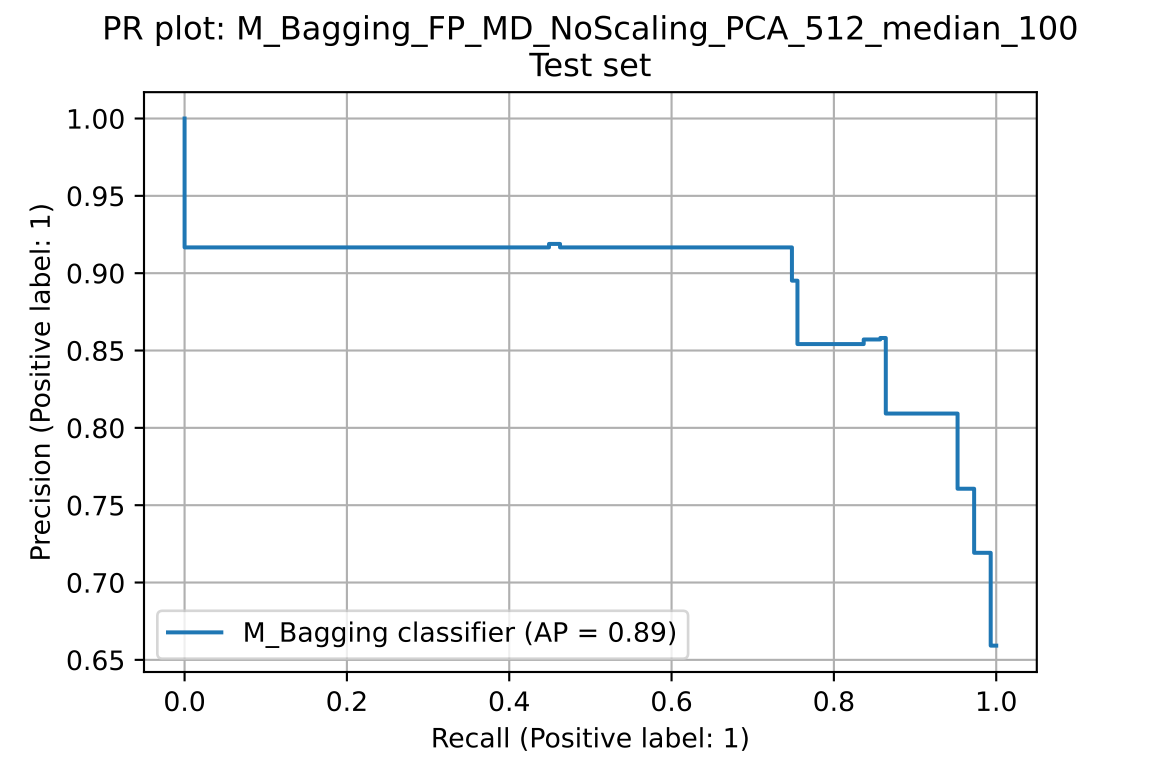
PR plot: M_Bagging_FP_MD_NoScaling_PCA_512_median_100
Test set
Precision (Positive label: 1)
Recall (Positive label: 1)
1.00
0.95
0.90
0.85
0.80
0.75
0.70
0.65
0.0
0.2
0.4
0.6
0.8
1.0
M_Bagging classifier (AP = 0.89)

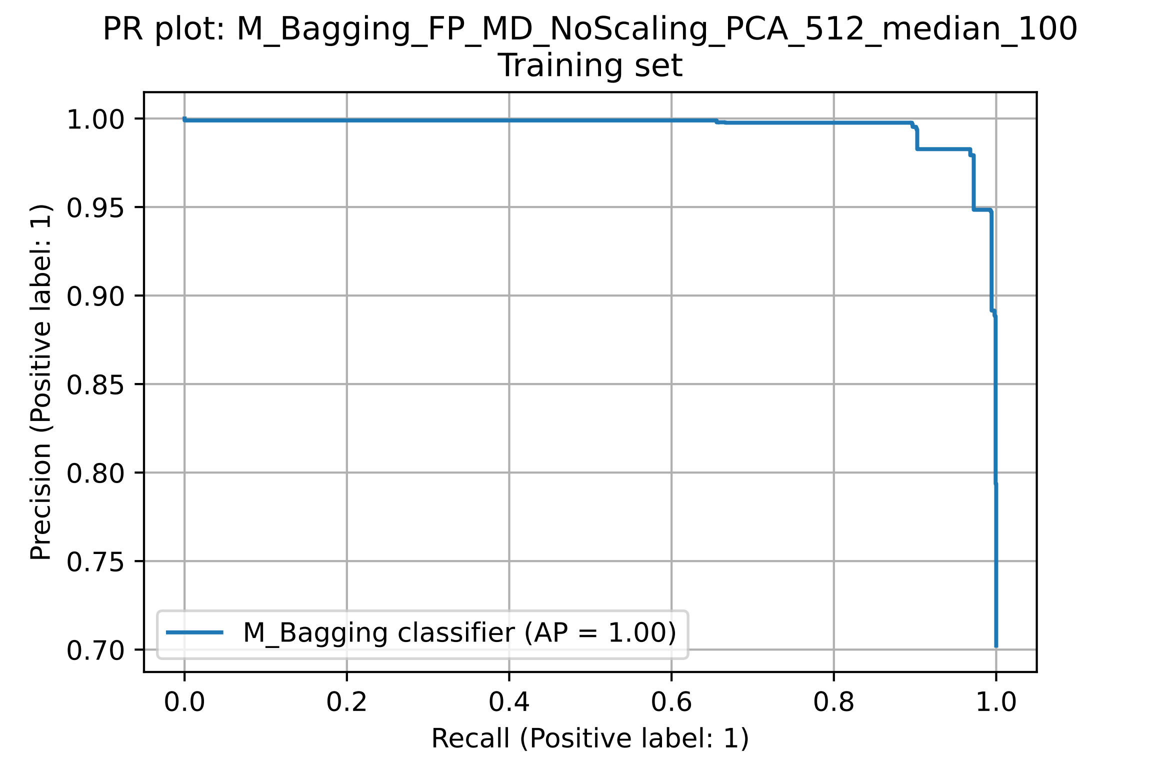
PR plot: M_Bagging_FP_MD_NoScaling_PCA_512_median_100
Training set
Precision (Positive label: 1)
Recall (Positive label: 1)
1.00
0.95
0.90
0.85
0.80
0.75
0.70
0.0
0.2
0.4
0.6
0.8
1.0
M_Bagging classifier (AP = 1.00)

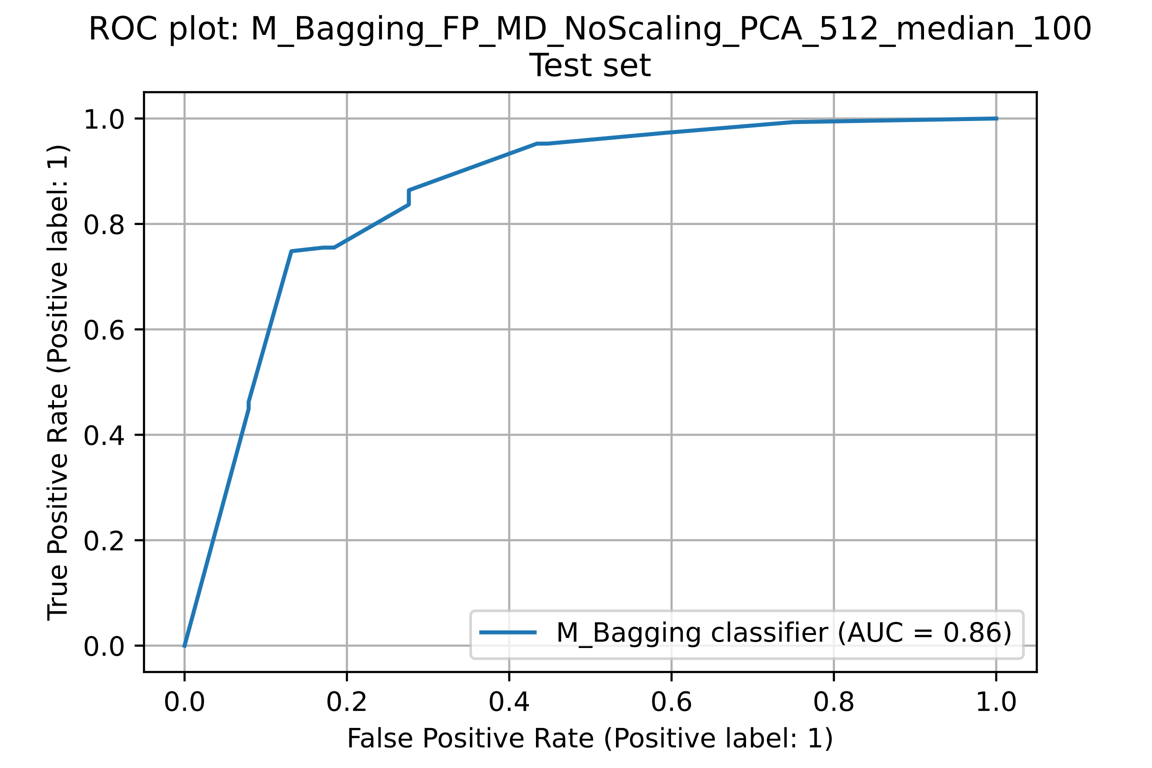
ROC plot: M_Bagging_FP_MD_NoScaling_PCA_512_median_100
Test set
True Positive Rate (Positive label: 1)
False Positive Rate (Positive label: 1)
0.0
0.2
0.4
0.6
0.8
1.0
M_Bagging classifier (AUC = 0.86)

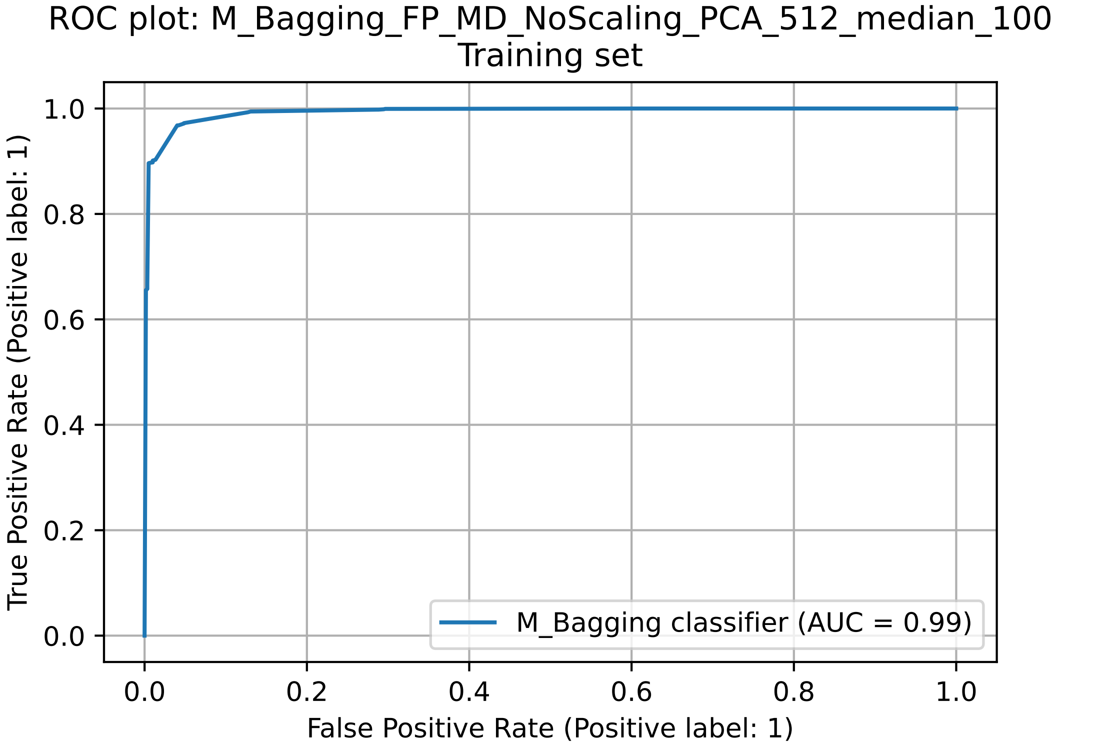
ROC plot: M_Bagging_FP_MD_NoScaling_PCA_512_median_100
Training set
True Positive Rate (Positive label: 1)
False Positive Rate (Positive label: 1)
0.0
0.2
0.4
0.6
0.8
1.0
M_Bagging classifier (AUC = 0.99)

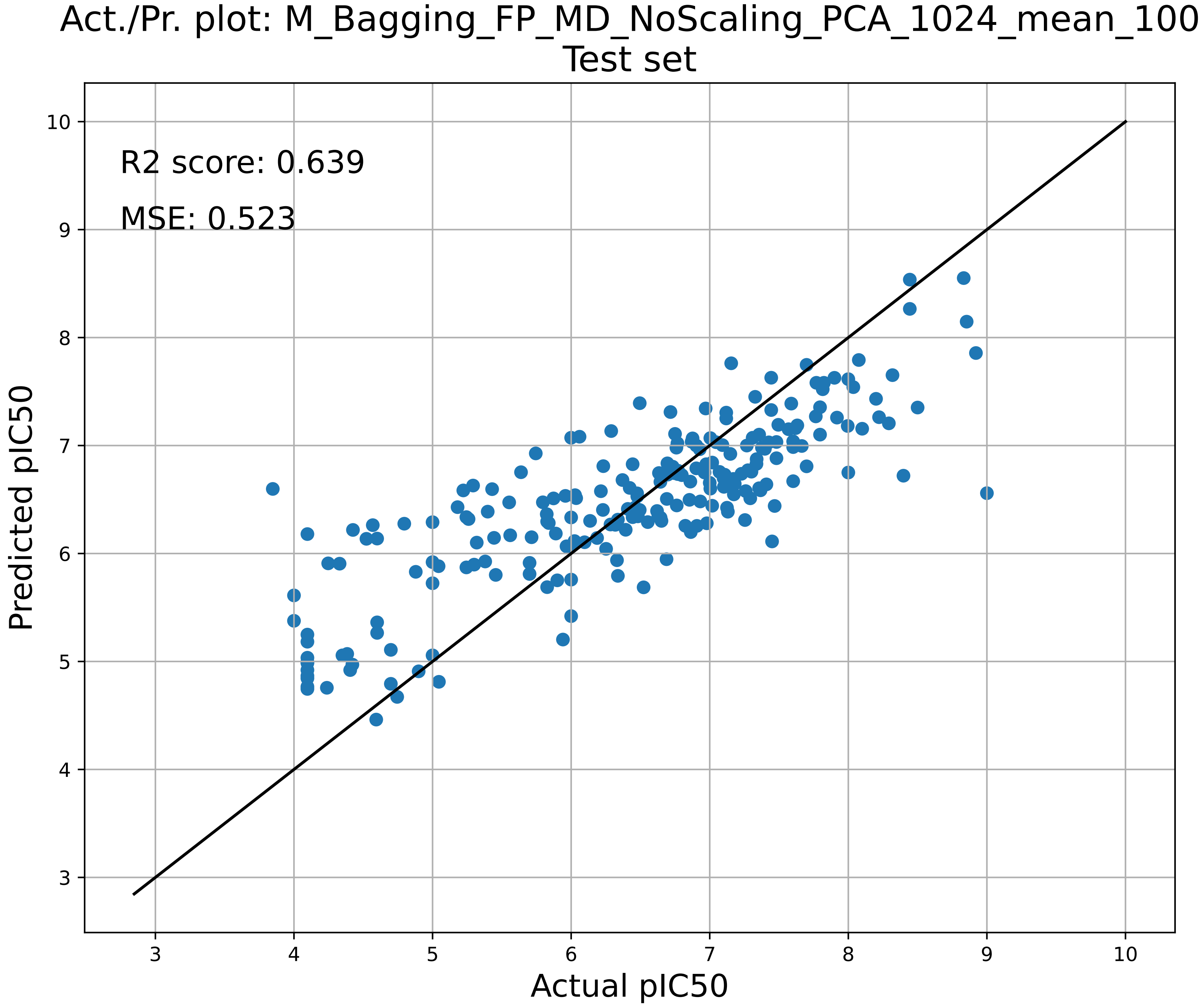

Act./Pr. plot: M_Bagging_FP_MD_NoScaling_PCA_1024_mean_100
Test set
R2 score: 0.639
MSE: 0.523
Predicted pIC50
Actual pIC50
3
4
5
6
7
8
9
10

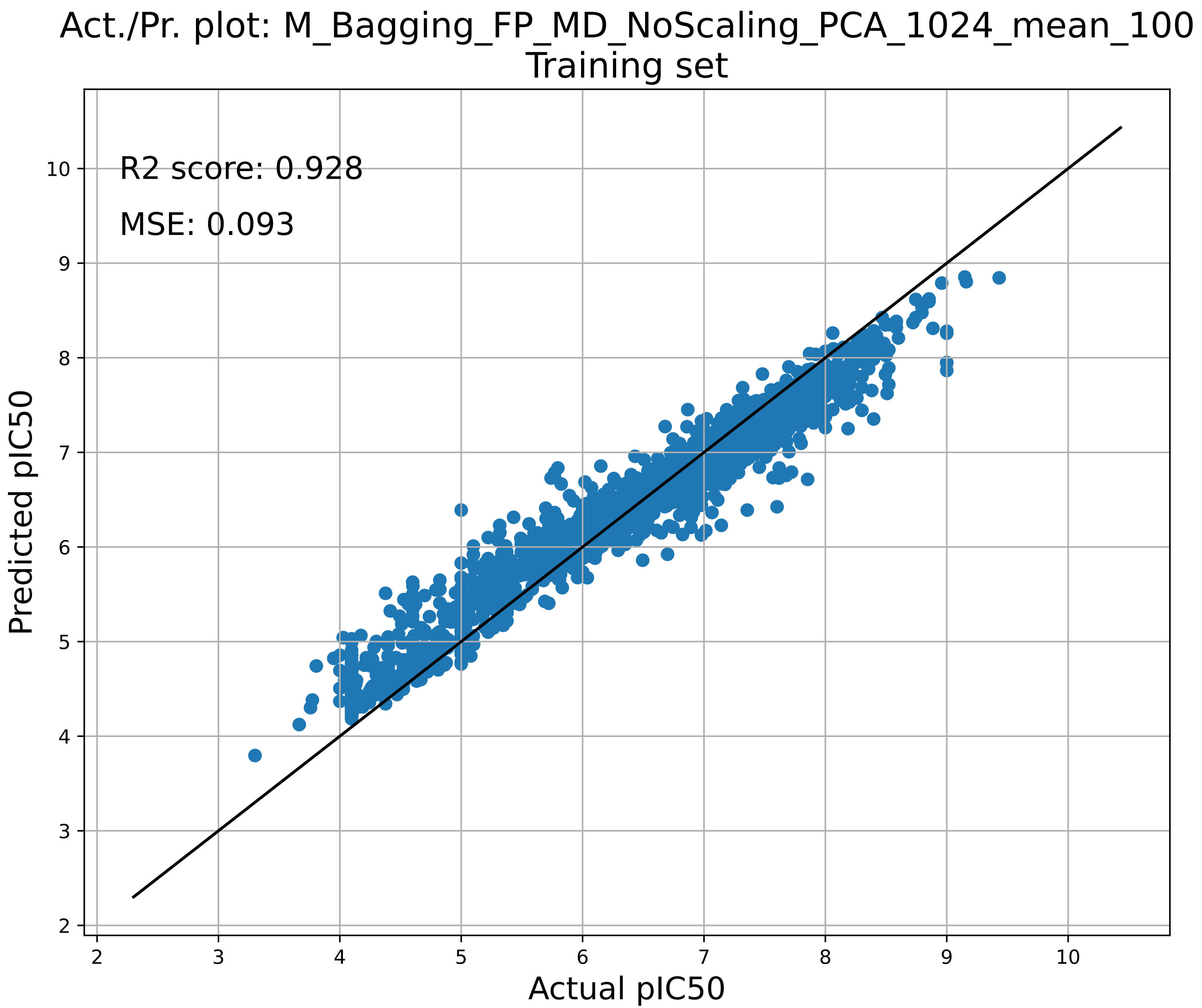
Act./Pr. plot: M_Bagging_FP_MD_NoScaling_PCA_1024_mean_100
Training set
R2 score: 0.928
MSE: 0.093
Predicted pIC50
Actual pIC50
2
3
4
5
6
7
8
9
10

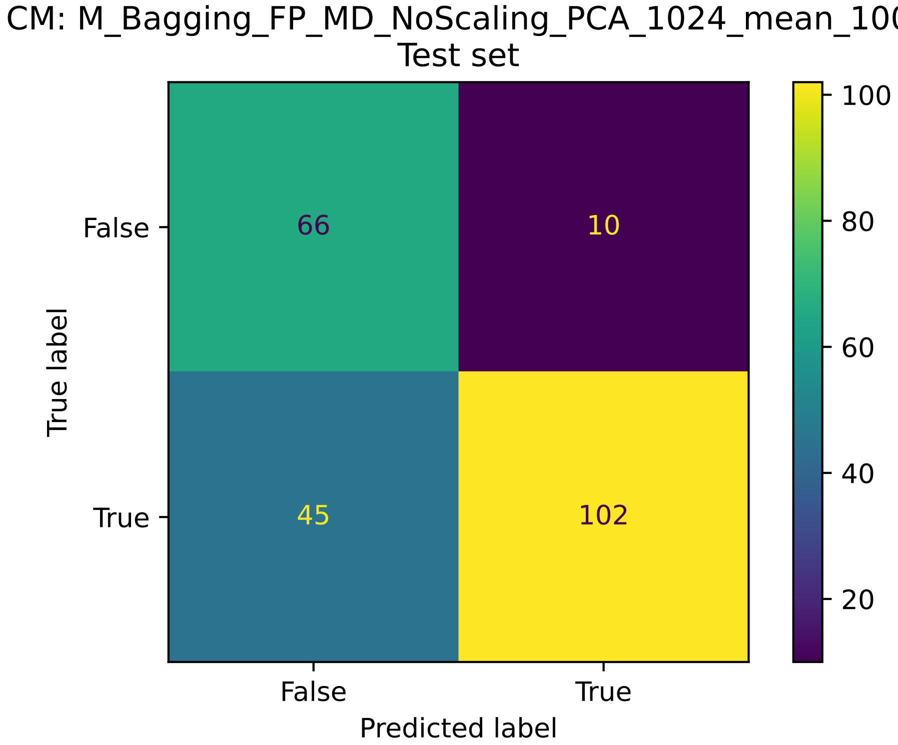
CM: M_Bagging_FP_MD_NoScaling_PCA_1024_mean_100
Test set
True label
False
True
Predicted label
False
True
66
10
45
102
100
80
60
40
20

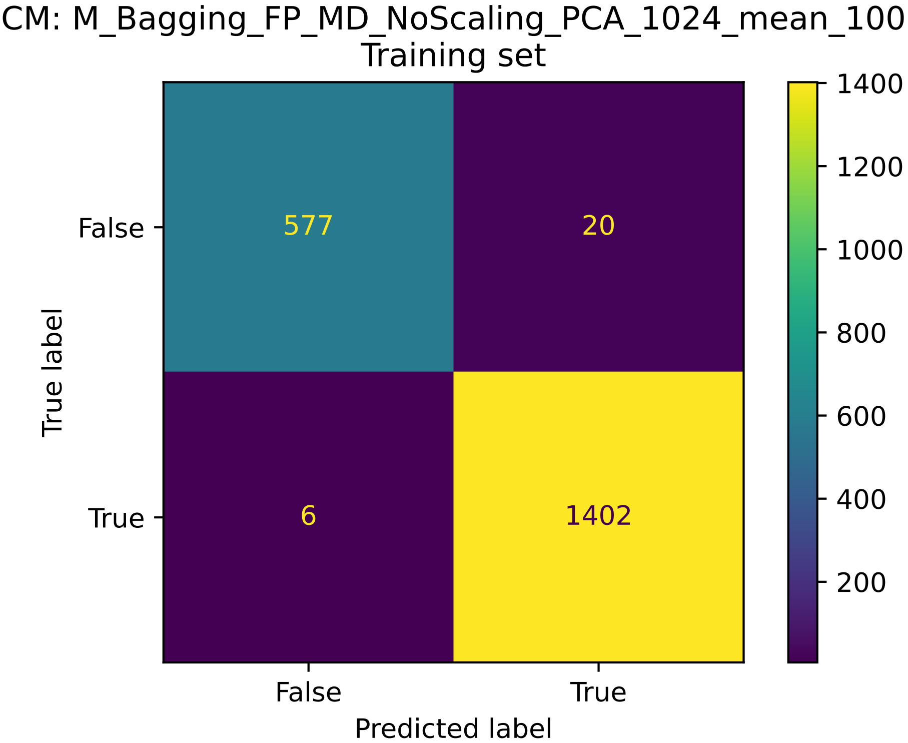
CM: M_Bagging_FP_MD_NoScaling_PCA_1024_mean_100
Training set
True label
False
True
577
20
6
1402
Predicted label
False
True
1400
1200
1000
800
600
400
200

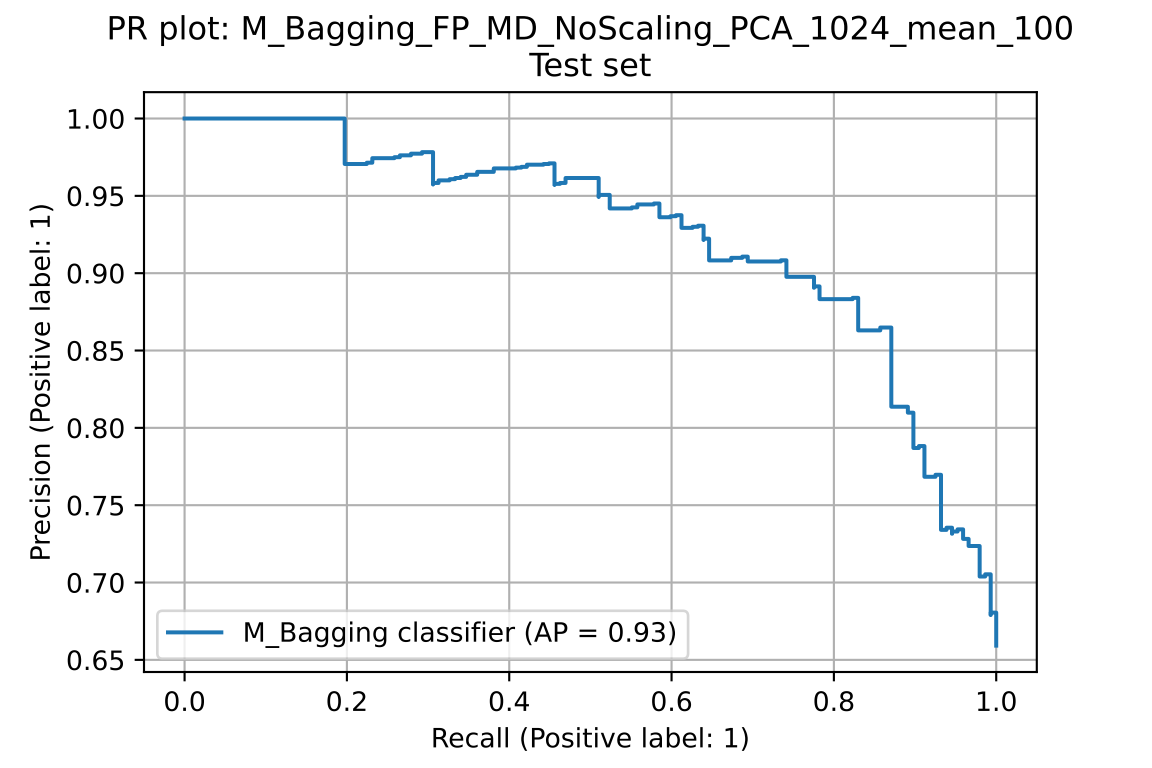
PR plot: M_Bagging_FP_MD_NoScaling_PCA_1024_mean_100
Test set
1.00
0.95
0.90
0.85
0.80
0.75
0.70
0.65
Precision (Positive label: 1)
0.0
0.2
0.4
0.6
0.8
1.0
Recall (Positive label: 1)
M_Bagging classifier (AP = 0.93)

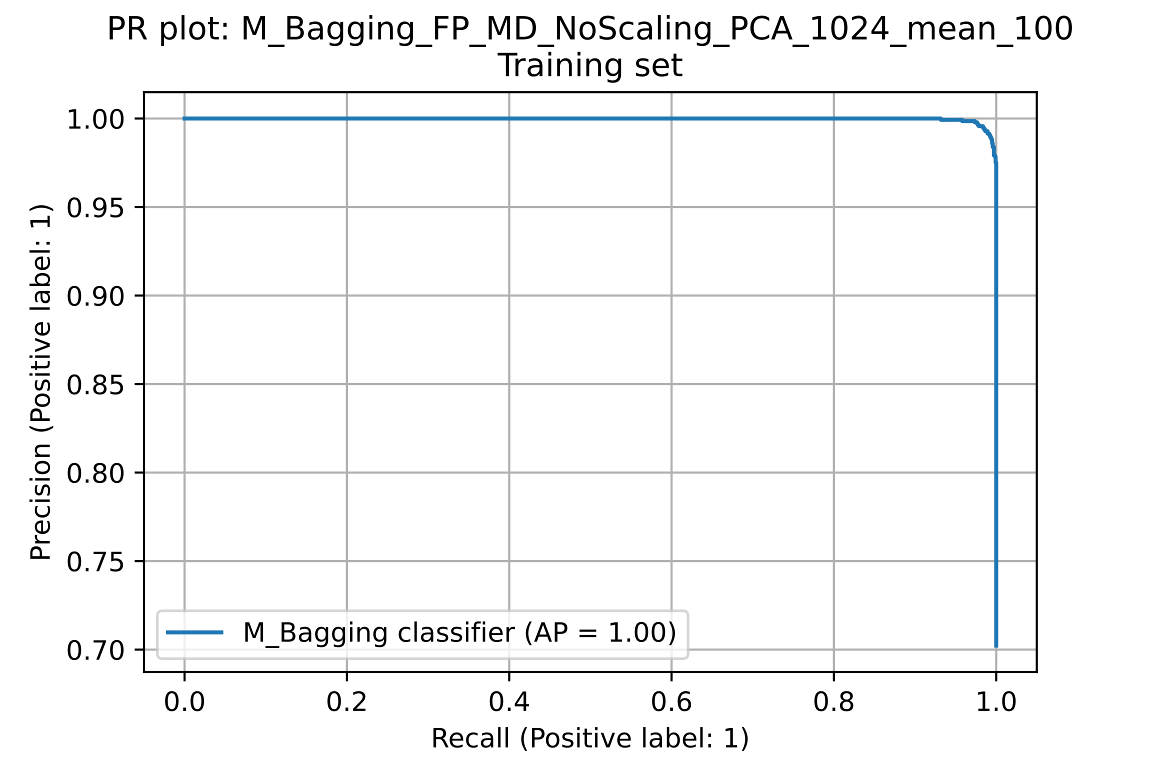
PR plot: M_Bagging_FP_MD_NoScaling_PCA_1024_mean_100
Training set
Precision (Positive label: 1)
Recall (Positive label: 1)
1.00
0.95
0.90
0.85
0.80
0.75
0.70
0.0
0.2
0.4
0.6
0.8
1.0
M_Bagging classifier (AP = 1.00)

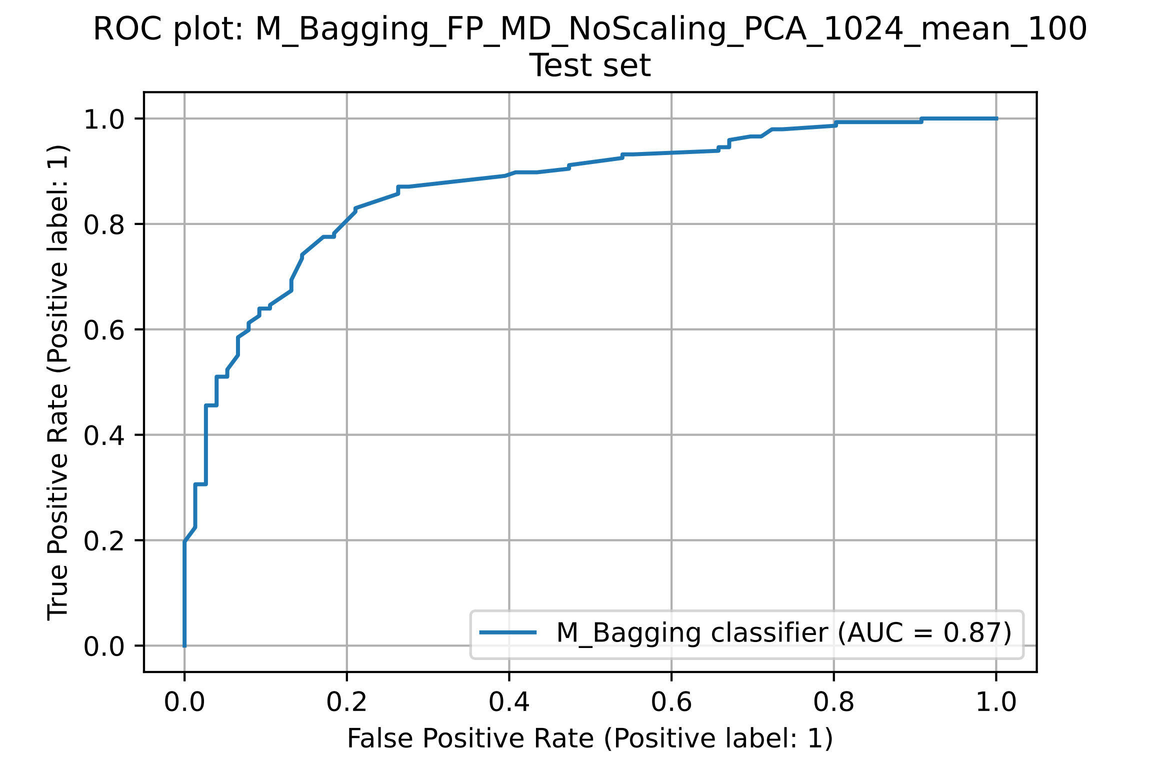
ROC plot: M_Bagging_FP_MD_NoScaling_PCA_1024_mean_100
Test set
True Positive Rate (Positive label: 1)
False Positive Rate (Positive label: 1)
0.0
0.2
0.4
0.6
0.8
1.0
M_Bagging classifier (AUC = 0.87)

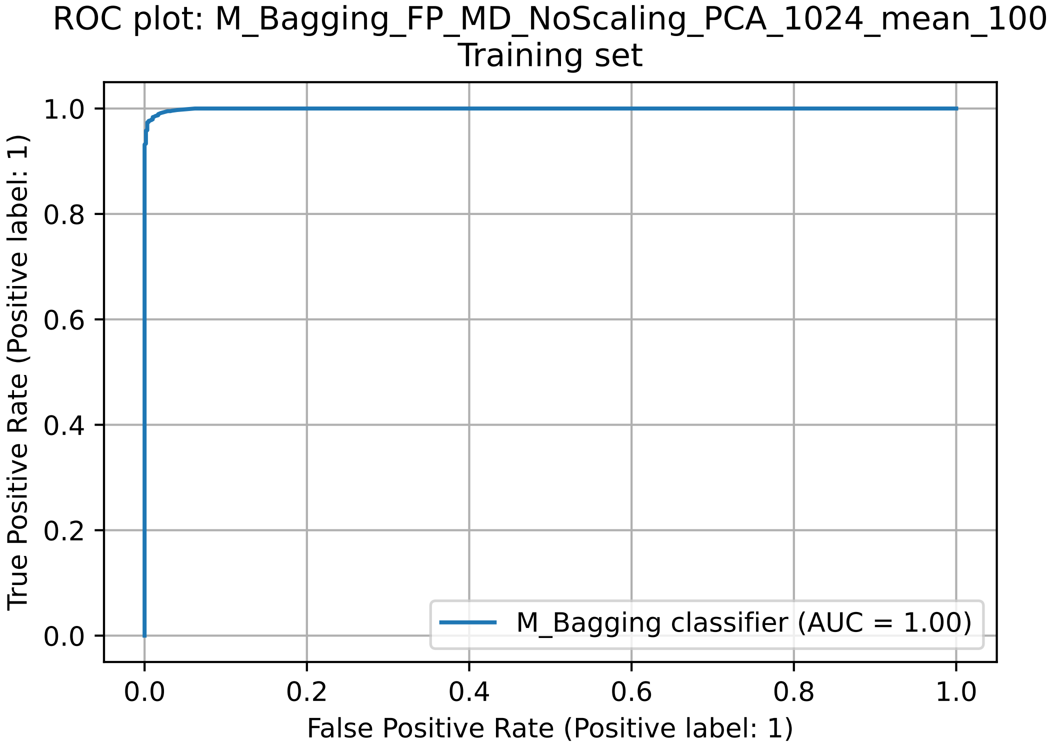
ROC plot: M_Bagging_FP_MD_NoScaling_PCA_1024_mean_100
Training set
True Positive Rate (Positive label: 1)
False Positive Rate (Positive label: 1)
0.0
0.2
0.4
0.6
0.8
1.0
M_Bagging classifier (AUC = 1.00)

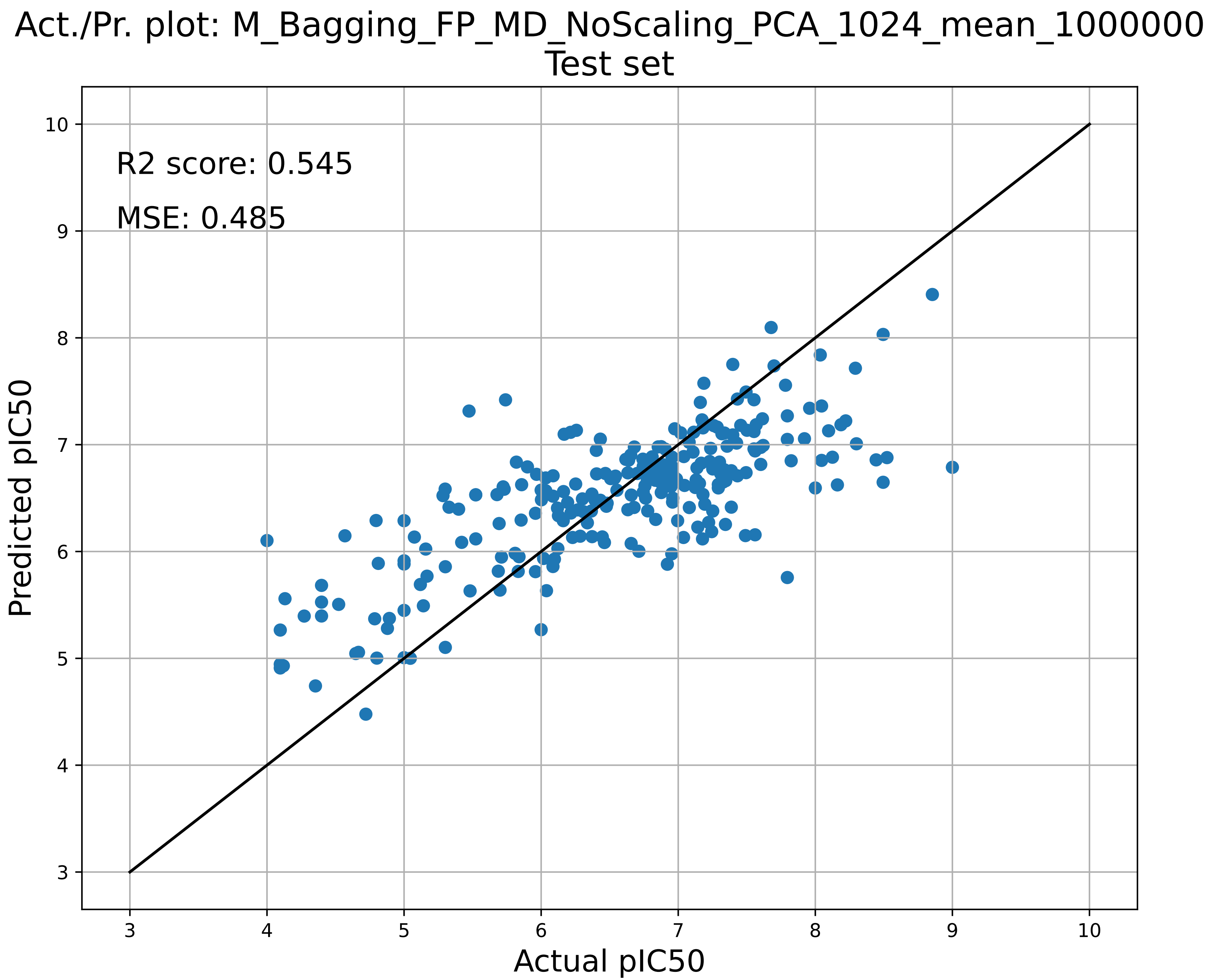
Act./Pr. plot: M_Bagging_FP_MD_NoScaling_PCA_1024_mean_1000000
Test set
R2 score: 0.545
MSE: 0.485
Predicted pIC50
Actual pIC50
3
4
5
6
7
8
9
10

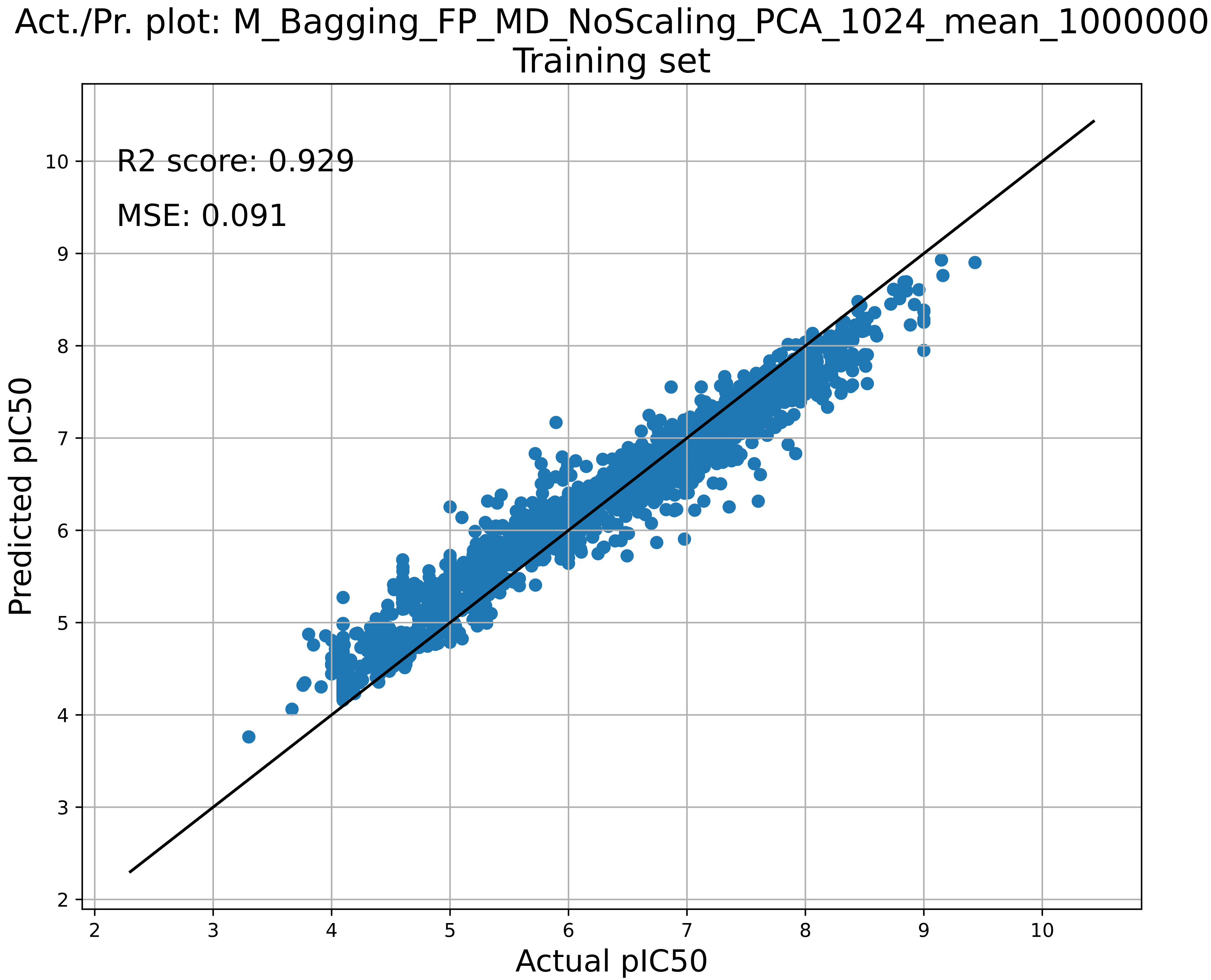

Act./Pr. plot: M_Bagging_FP_MD_NoScaling_PCA_1024_mean_1000000
Training set
R2 score: 0.929
MSE: 0.091
Predicted pIC50
Actual pIC50
2
3
4
5
6
7
8
9
10

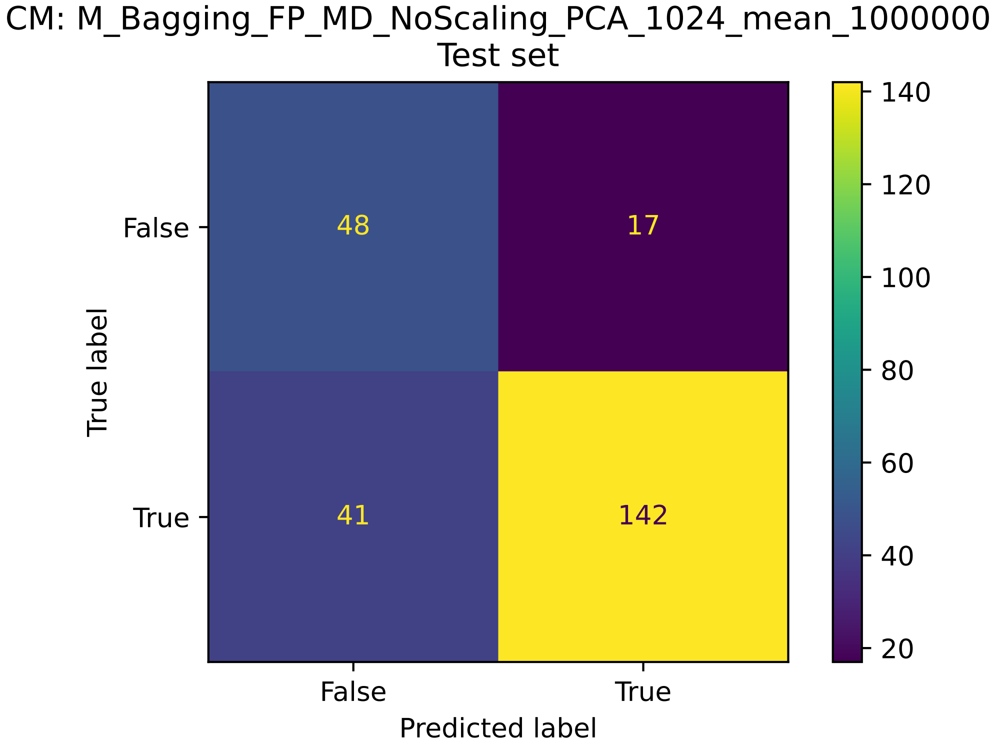

CM: M_Bagging_FP_MD_NoScaling_PCA_1024_mean_1000000
Test set
True label
False
True
48
17
41
142
False
True
Predicted label
140
120
100
80
60
40
20

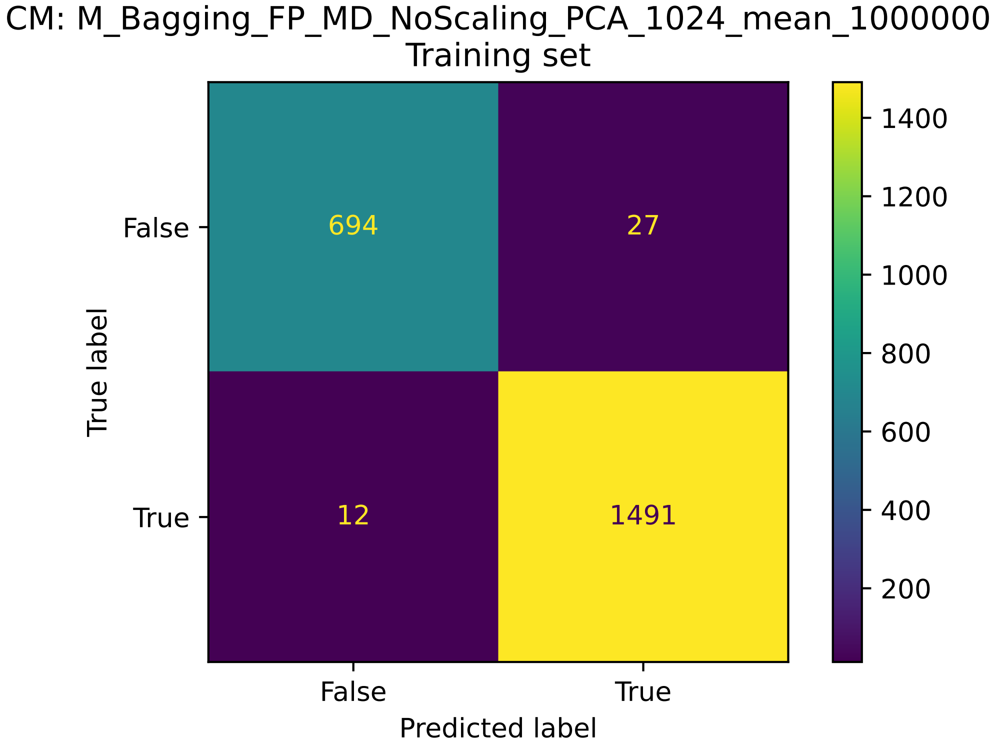
CM: M_Bagging_FP_MD_NoScaling_PCA_1024_mean_1000000
Training set
True label
Predicted label
False
True
False
True
694
27
12
1491
1400
1200
1000
800
600
400
200

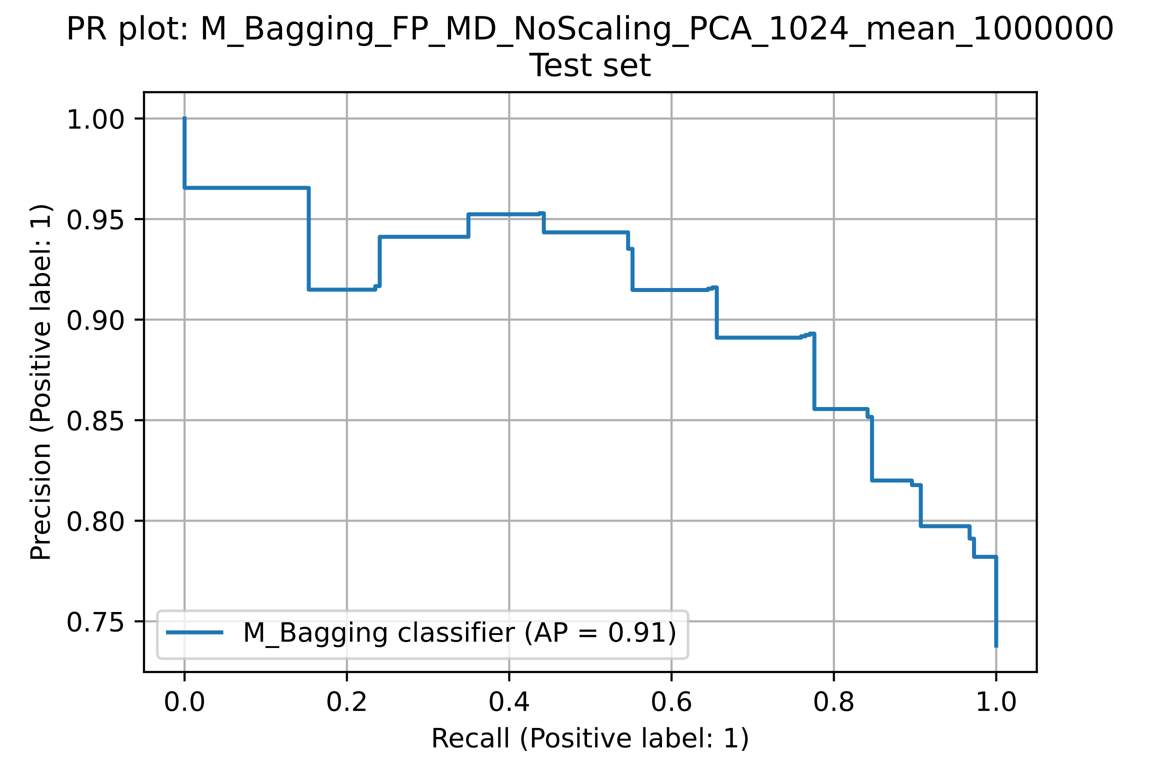
PR plot: M_Bagging_FP_MD_NoScaling_PCA_1024_mean_1000000
Test set
Precision (Positive label: 1)
Recall (Positive label: 1)
1.00
0.95
0.90
0.85
0.80
0.75
0.0
0.2
0.4
0.6
0.8
1.0
M_Bagging classifier (AP = 0.91)

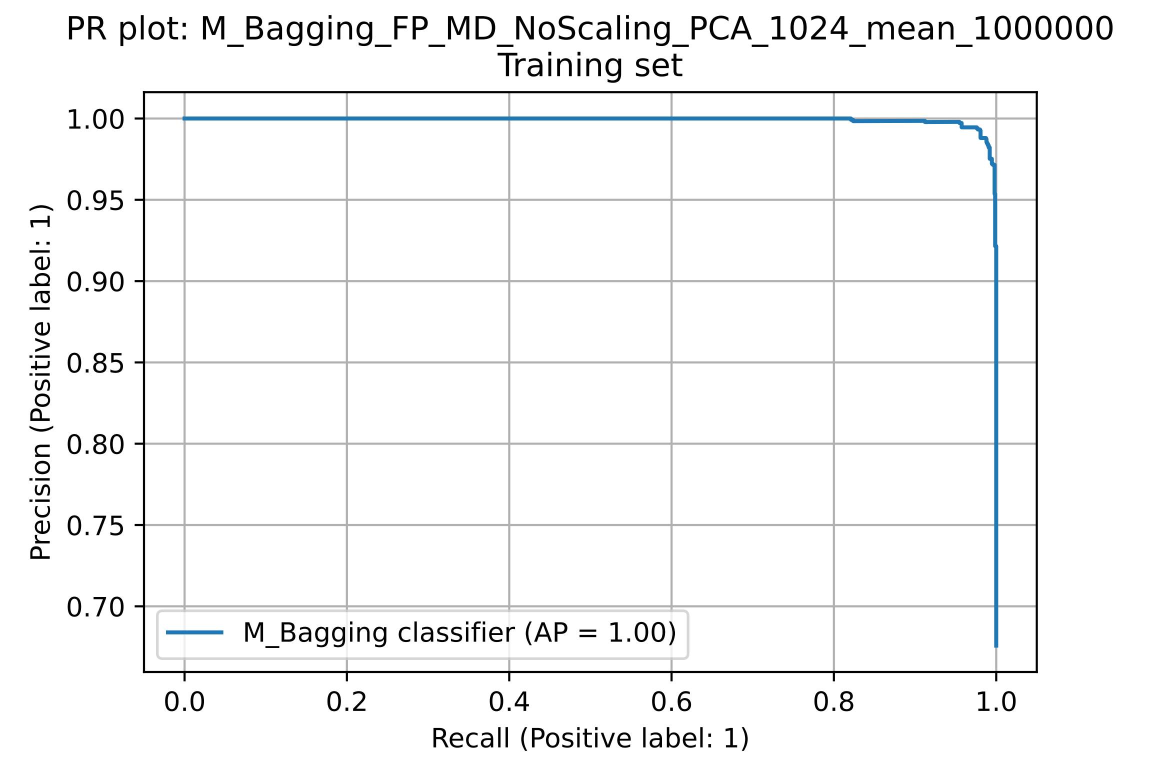
PR plot: M_Bagging_FP_MD_NoScaling_PCA_1024_mean_1000000
Training set
Precision (Positive label: 1)
Recall (Positive label: 1)
1.00
0.95
0.90
0.85
0.80
0.75
0.70
0.0
0.2
0.4
0.6
0.8
1.0
M_Bagging classifier (AP = 1.00)

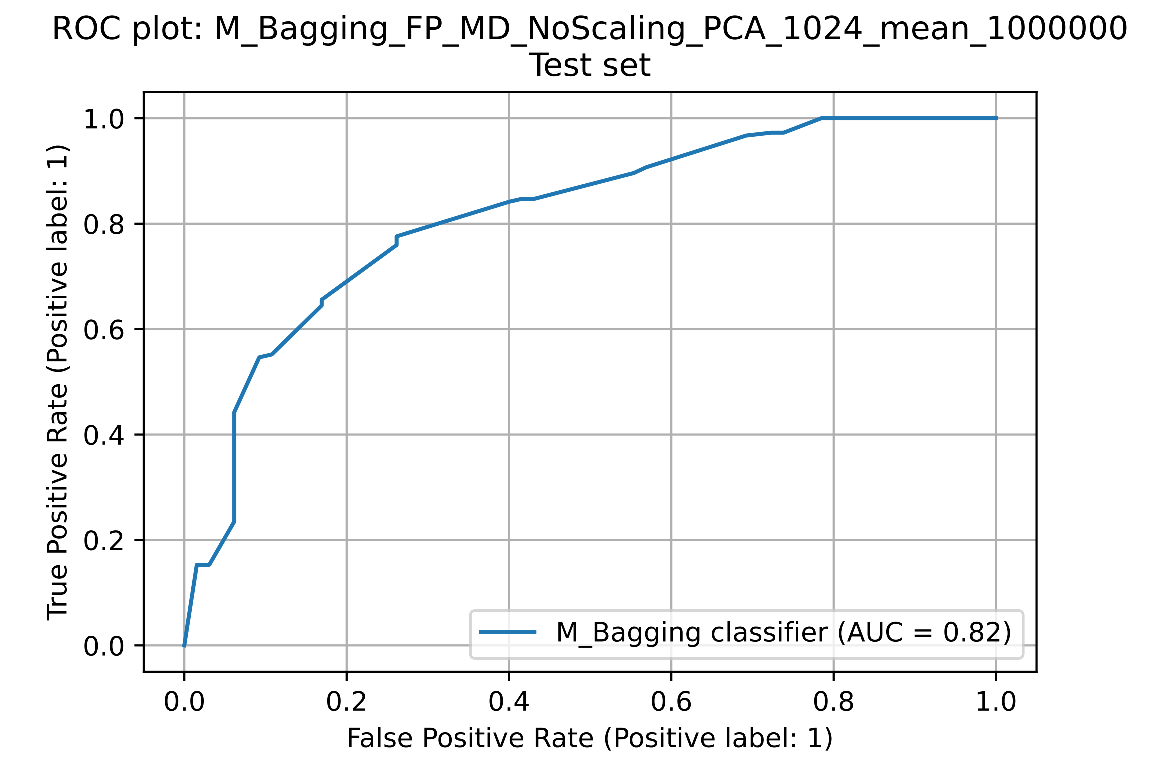

ROC plot: M_Bagging_FP_MD_NoScaling_PCA_1024_mean_1000000
Test set
True Positive Rate (Positive label: 1)
False Positive Rate (Positive label: 1)
0.0
0.2
0.4
0.6
0.8
1.0
M_Bagging classifier (AUC = 0.82)

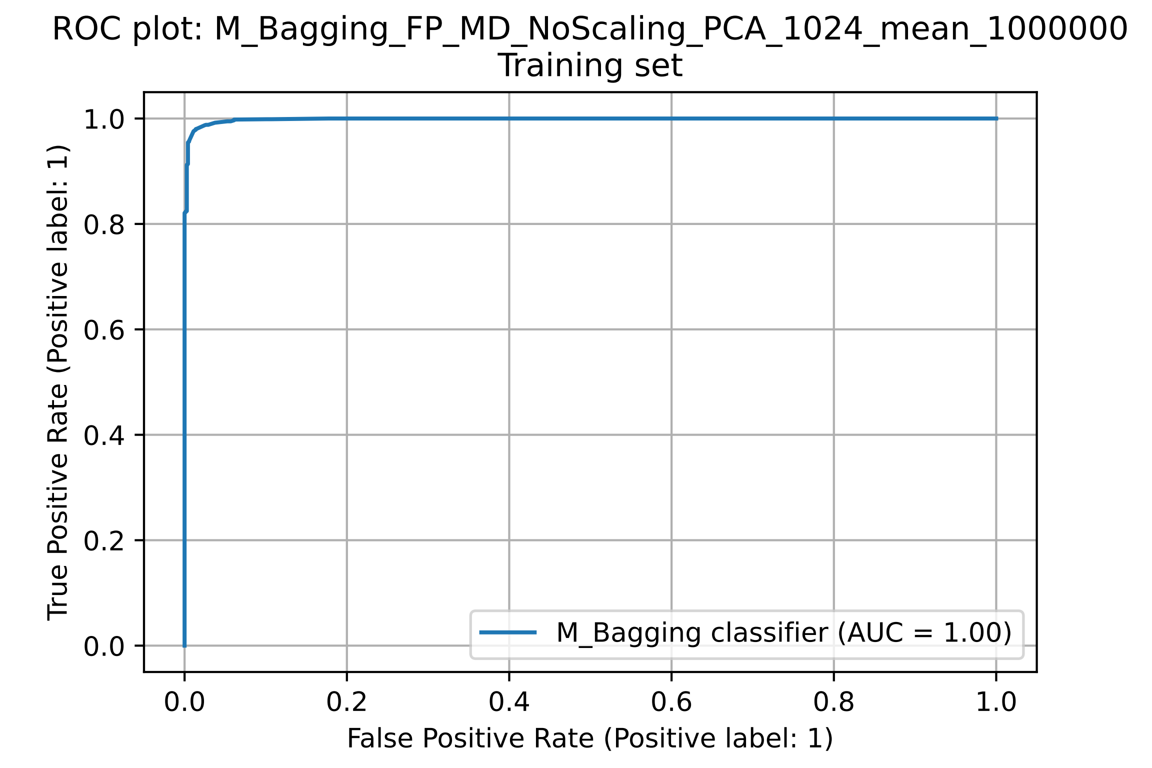
ROC plot: M_Bagging_FP_MD_NoScaling_PCA_1024_mean_1000000
Training set
True Positive Rate (Positive label: 1)
False Positive Rate (Positive label: 1)
0.0
0.2
0.4
0.6
0.8
1.0
M_Bagging classifier (AUC = 1.00)

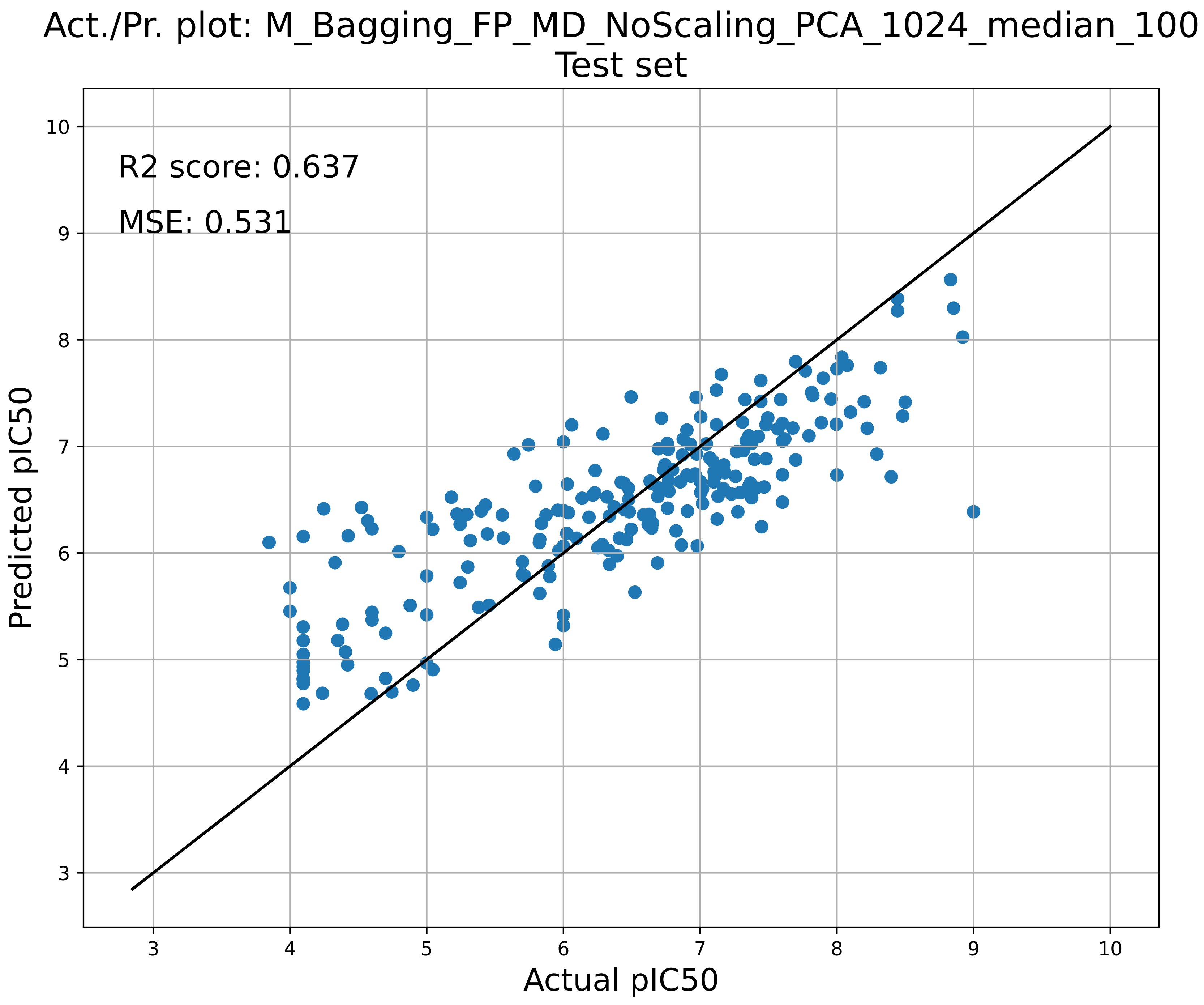

Act./Pr. plot: M_Bagging_FP_MD_NoScaling_PCA_1024_median_100
Test set
R2 score: 0.637
MSE: 0.531
Predicted pIC50
Actual pIC50
3
4
5
6
7
8
9
10

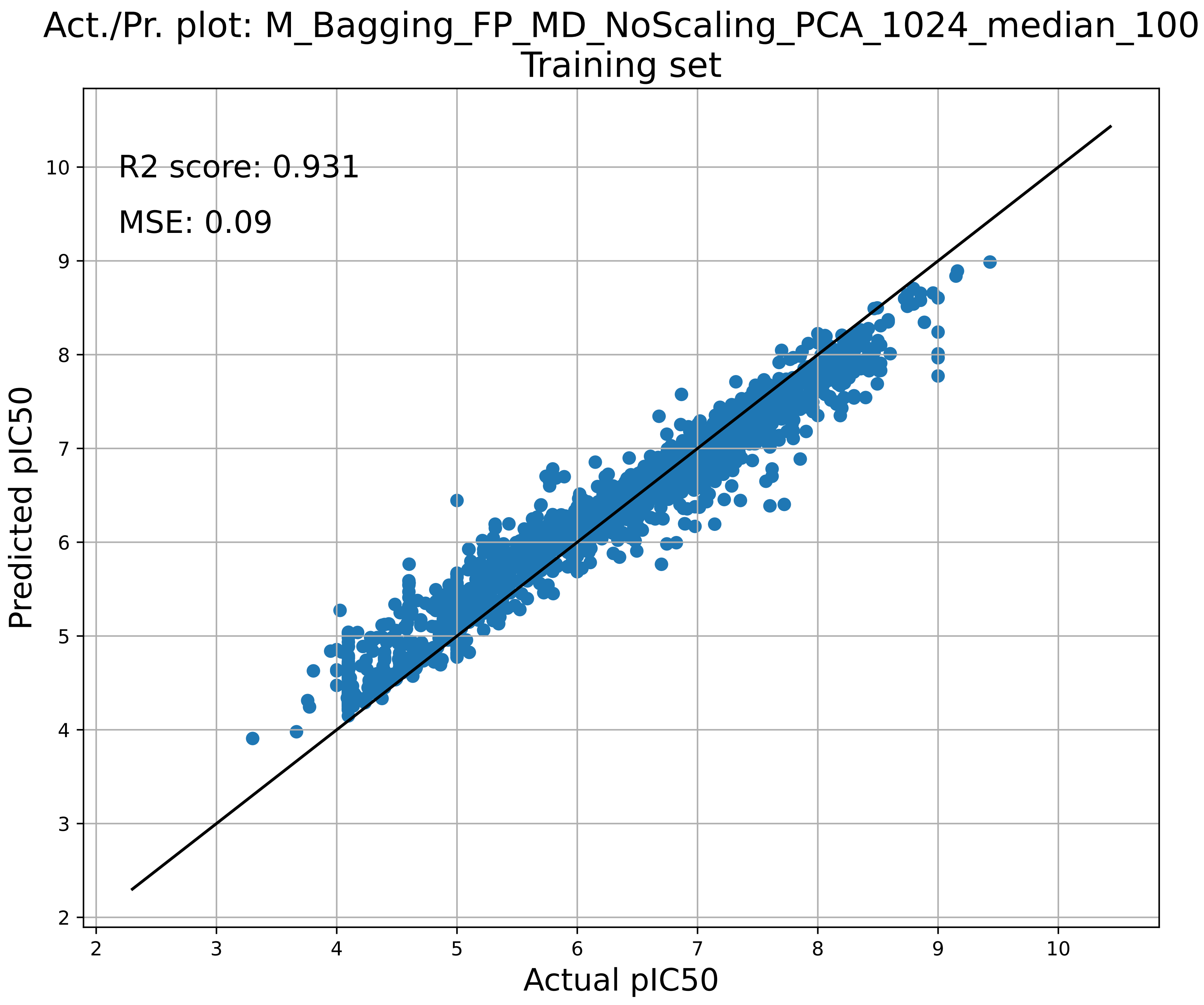
Act./Pr. plot: M_Bagging_FP_MD_NoScaling_PCA_1024_median_100
Training set
R2 score: 0.931
MSE: 0.09
Predicted pIC50
Actual pIC50
2
3
4
5
6
7
8
9
10

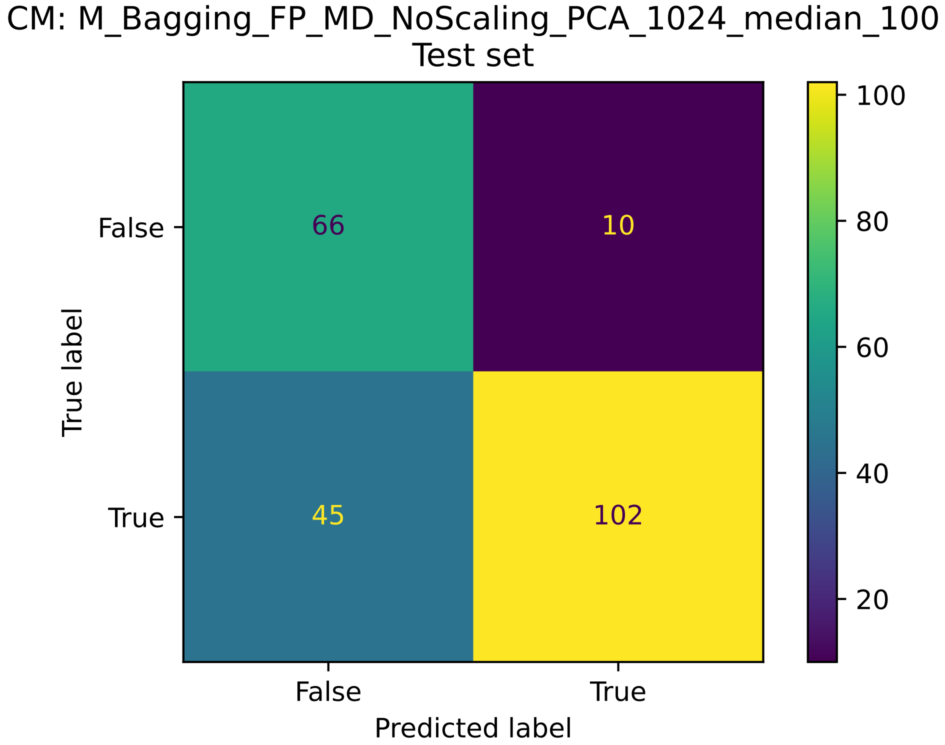

CM: M_Bagging_FP_MD_NoScaling_PCA_1024_median_100
Test set
True label
Predicted label
False
True
False
True
66
10
45
102
100
80
60
40
20

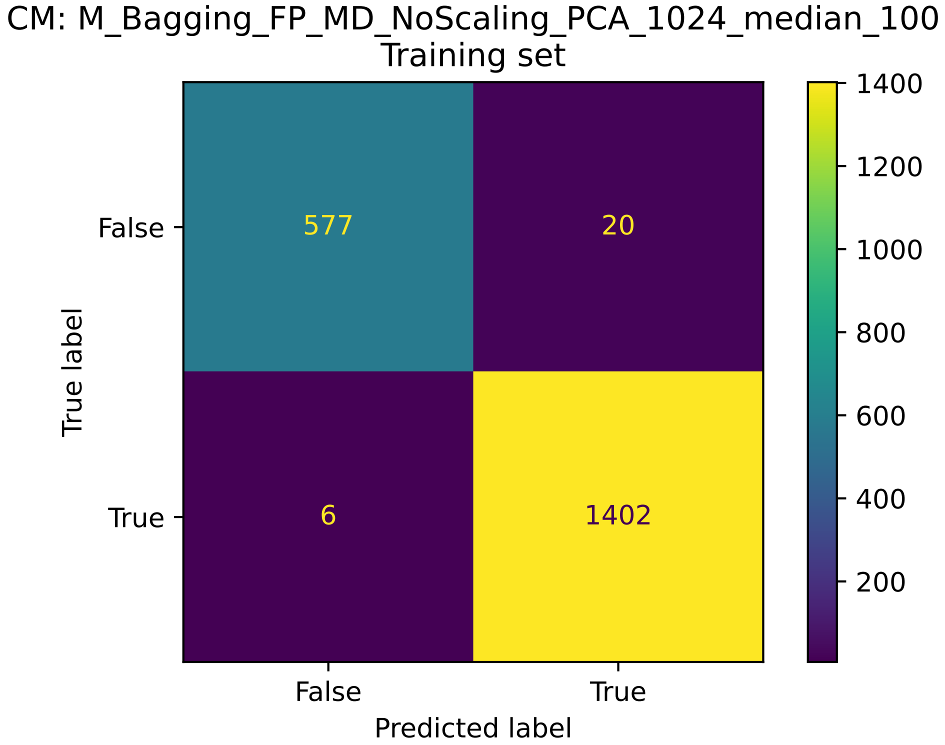

CM: M_Bagging_FP_MD_NoScaling_PCA_1024_median_100
Training set
True label
False
True
577
20
6
1402
Predicted label
False
True
1400
1200
1000
800
600
400
200

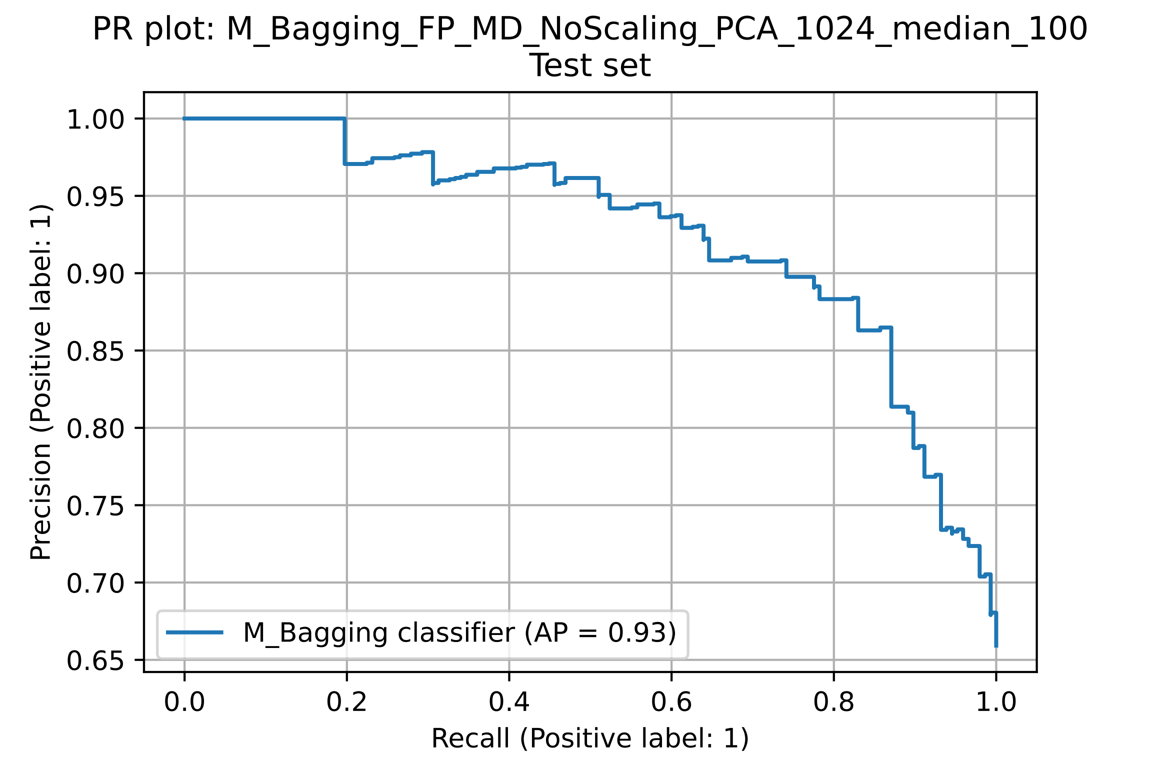

PR plot: M_Bagging_FP_MD_NoScaling_PCA_1024_median_100
Test set
Precision (Positive label: 1)
Recall (Positive label: 1)
M_Bagging classifier (AP = 0.93)
1.00
0.95
0.90
0.85
0.80
0.75
0.70
0.65
0.0
0.2
0.4
0.6
0.8
1.0

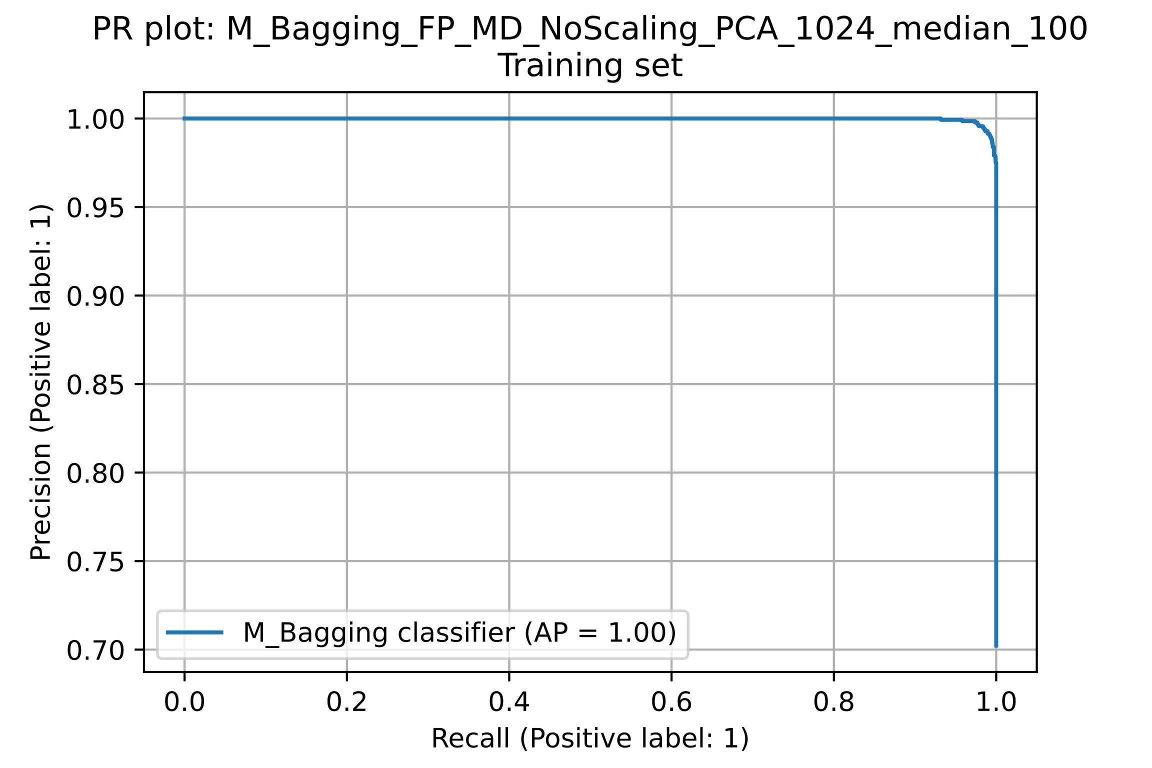
PR plot: M_Bagging_FP_MD_NoScaling_PCA_1024_median_100
Training set
Precision (Positive label: 1)
Recall (Positive label: 1)
1.00
0.95
0.90
0.85
0.80
0.75
0.70
0.0
0.2
0.4
0.6
0.8
1.0
M_Bagging classifier (AP = 1.00)

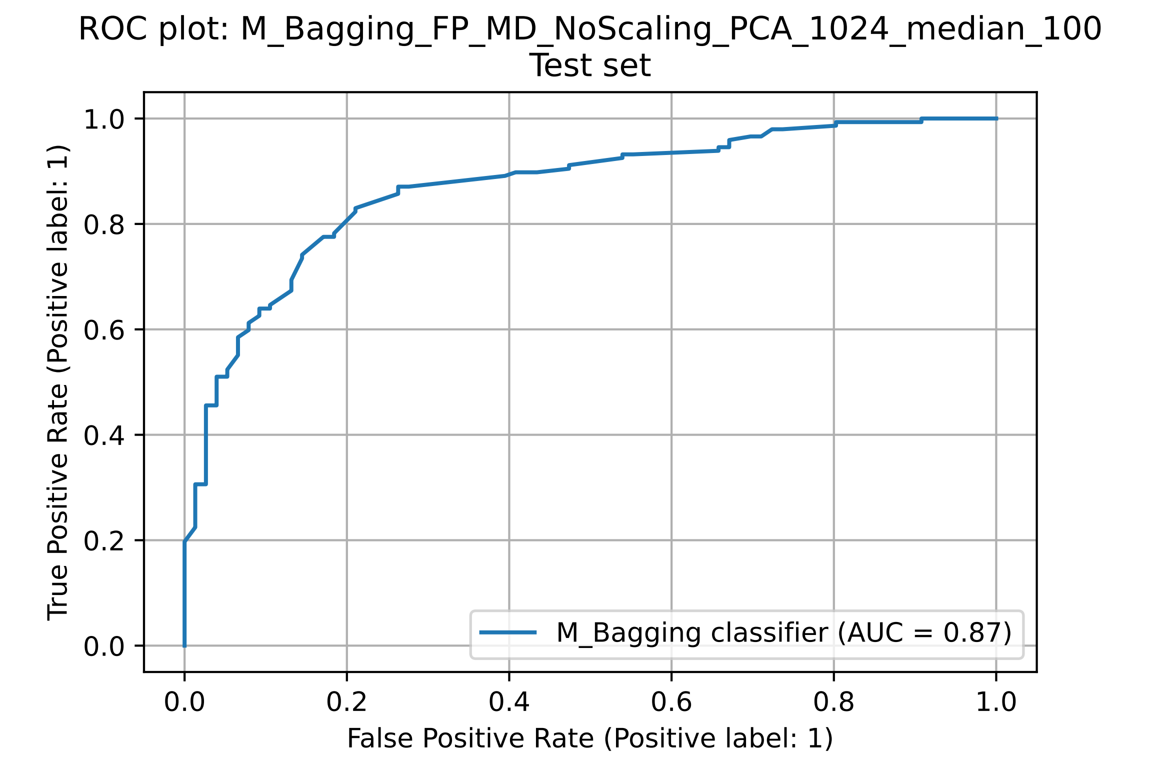
ROC plot: M_Bagging_FP_MD_NoScaling_PCA_1024_median_100
Test set
True Positive Rate (Positive label: 1)
False Positive Rate (Positive label: 1)
0.0
0.2
0.4
0.6
0.8
1.0
M_Bagging classifier (AUC = 0.87)

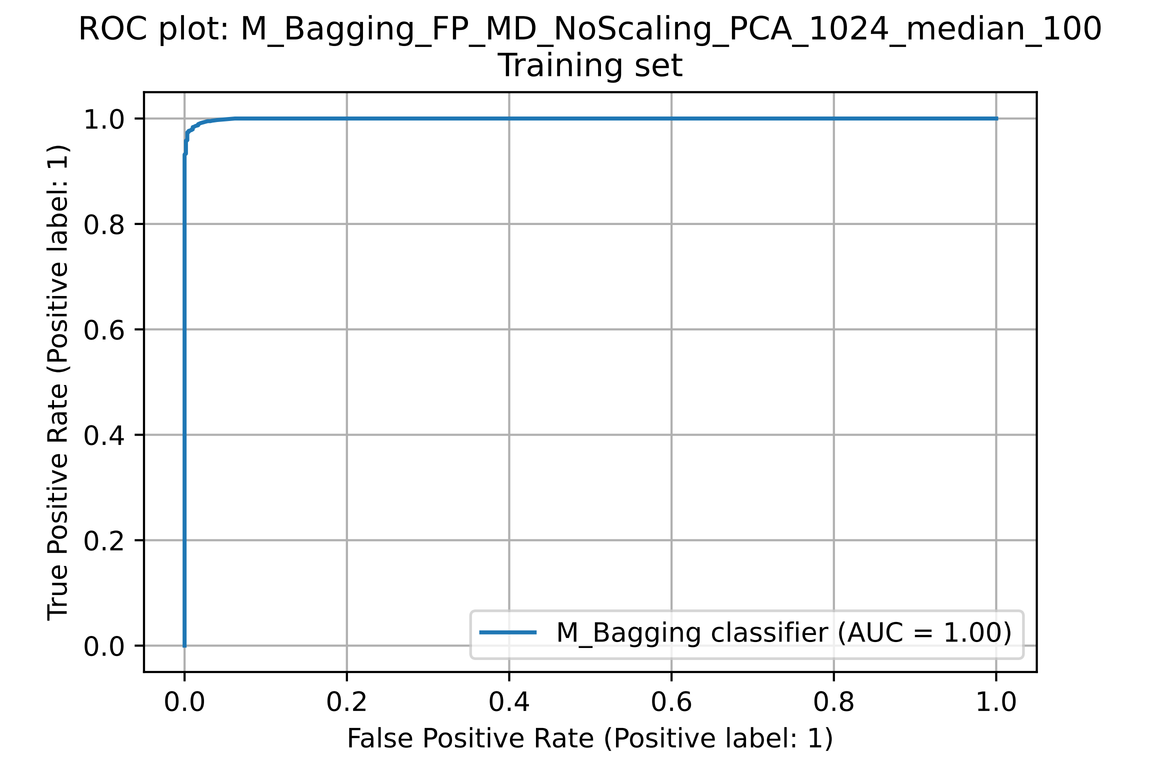
ROC plot: M_Bagging_FP_MD_NoScaling_PCA_1024_median_100
Training set
True Positive Rate (Positive label: 1)
False Positive Rate (Positive label: 1)
0.0
0.2
0.4
0.6
0.8
1.0
M_Bagging classifier (AUC = 1.00)

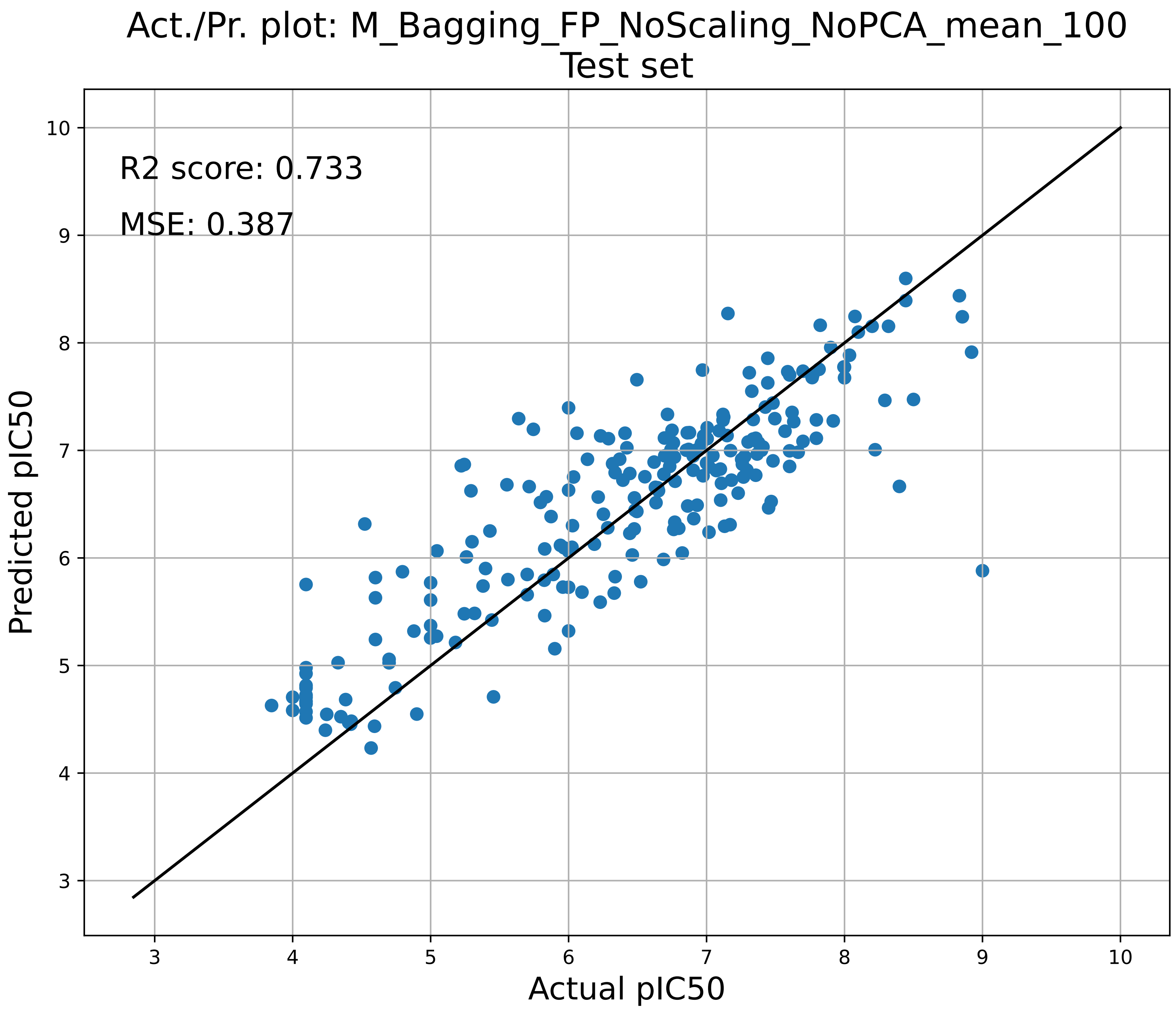
Act./Pr. plot: M_Bagging_FP_NoScaling_NoPCA_mean_100
Test set
R2 score: 0.733
MSE: 0.387
Predicted pIC50
Actual pIC50
3
4
5
6
7
8
9
10

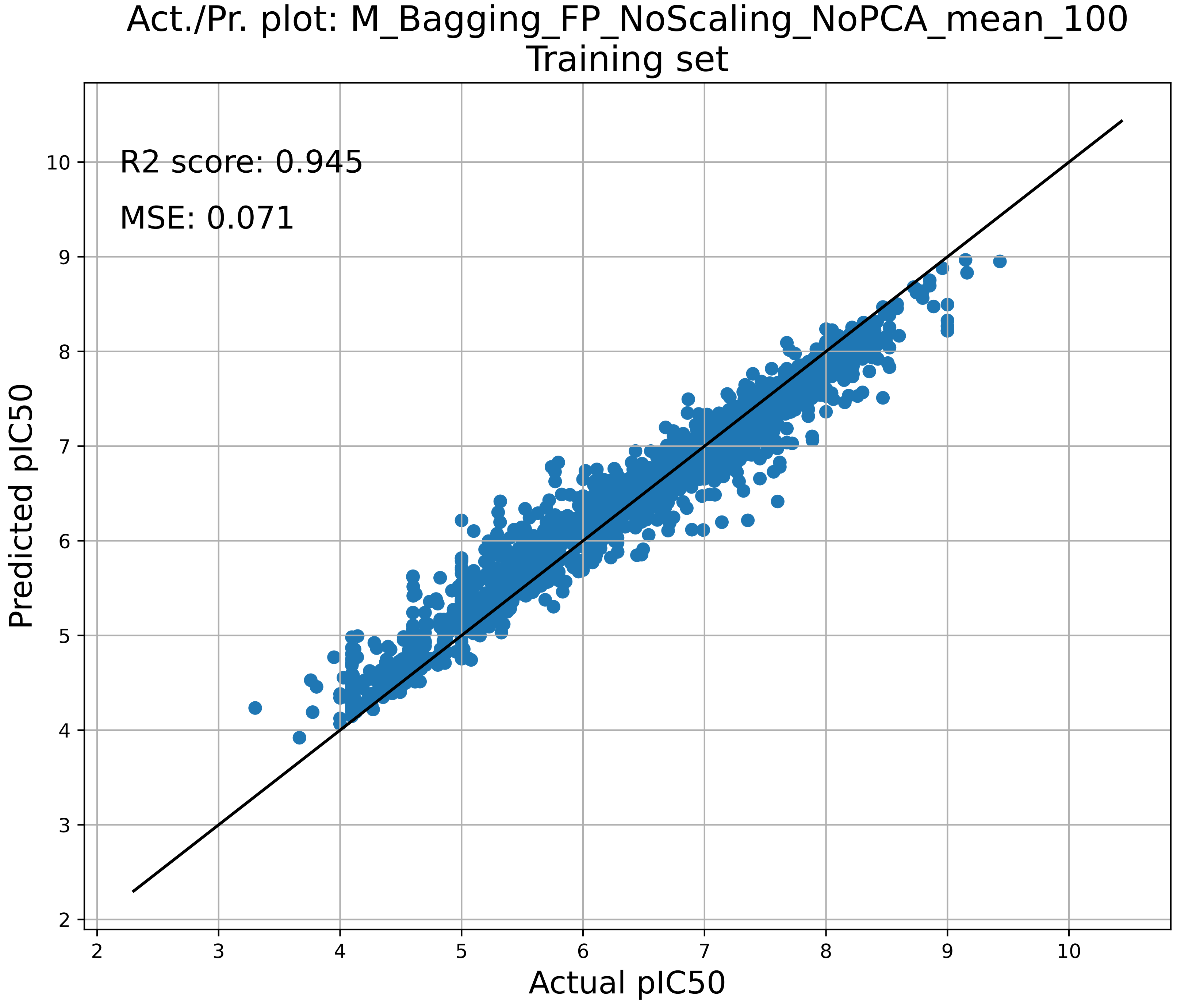
Act./Pr. plot: M_Bagging_FP_NoScaling_NoPCA_mean_100
Training set
R2 score: 0.945
MSE: 0.071
Predicted pIC50
Actual pIC50
2
3
4
5
6
7
8
9
10

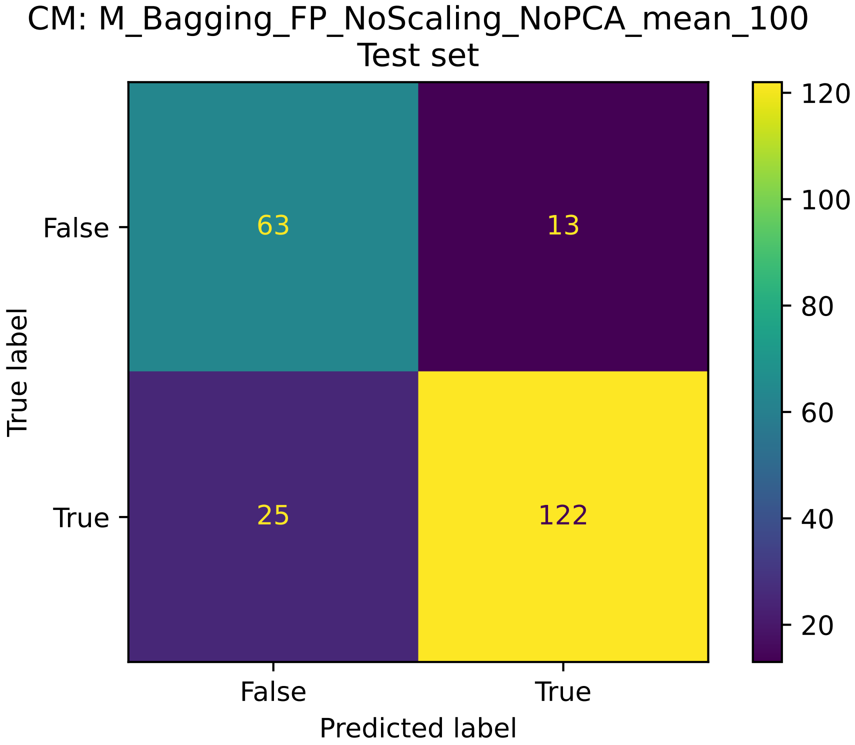
CM: M_Bagging_FP_NoScaling_NoPCA_mean_100
Test set
63
13
25
122
False
True
True label
False
True
Predicted label
120
100
80
60
40
20

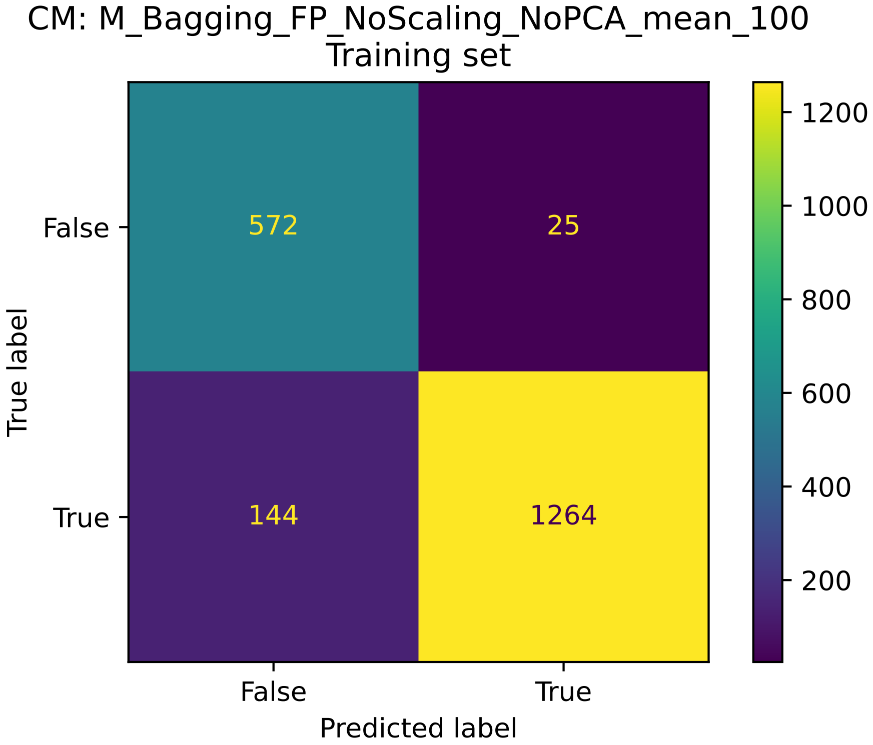
CM: M_Bagging_FP_NoScaling_NoPCA_mean_100
Training set
True label
False
True
572
25
144
1264
Predicted label
False
True
1200
1000
800
600
400
200

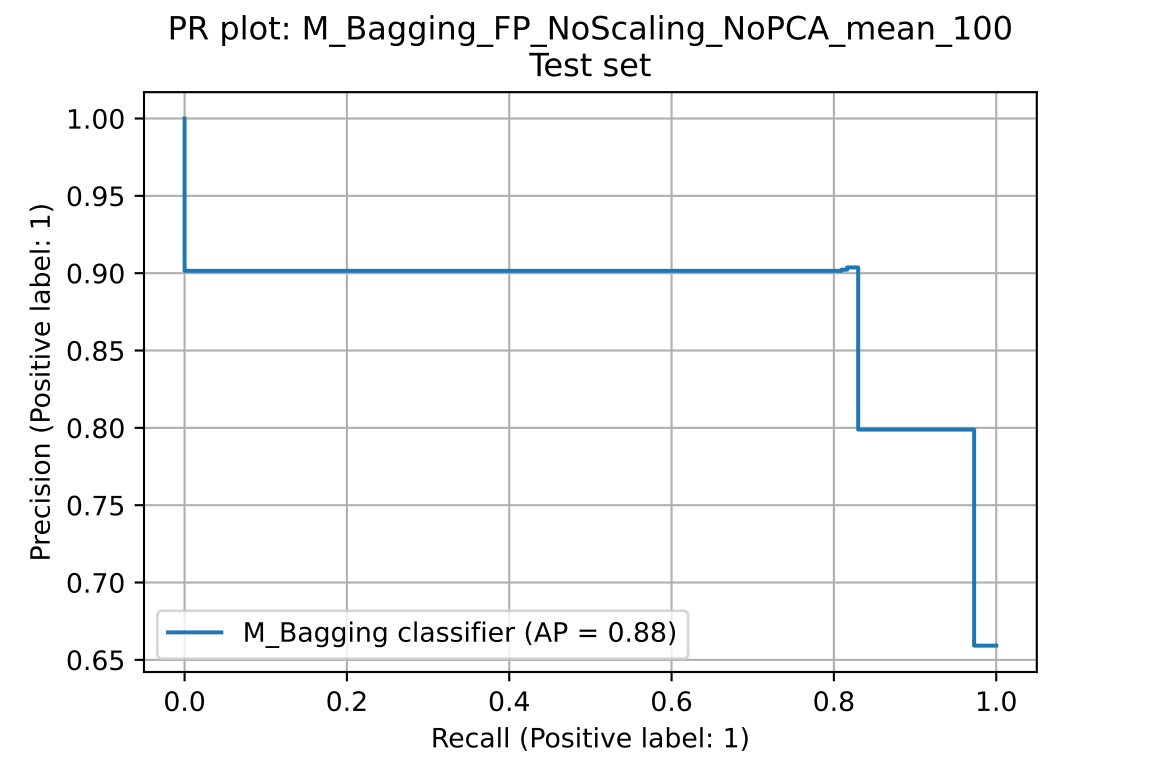

PR plot: M_Bagging_FP_NoScaling_NoPCA_mean_100
Test set
Precision (Positive label: 1)
Recall (Positive label: 1)
1.00
0.95
0.90
0.85
0.80
0.75
0.70
0.65
0.0
0.2
0.4
0.6
0.8
1.0
M_Bagging classifier (AP = 0.88)

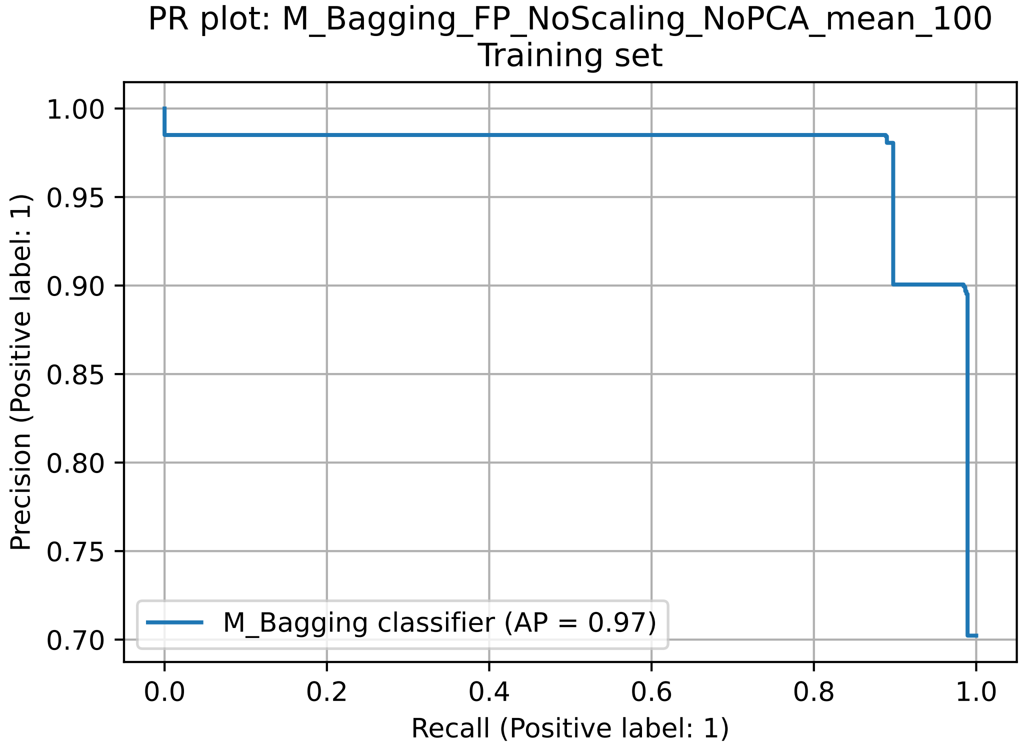
PR plot: M_Bagging_FP_NoScaling_NoPCA_mean_100
Training set
1.00
0.95
0.90
0.85
0.80
0.75
0.70
Precision (Positive label: 1)
0.0
0.2
0.4
0.6
0.8
1.0
Recall (Positive label: 1)
M_Bagging classifier (AP = 0.97)

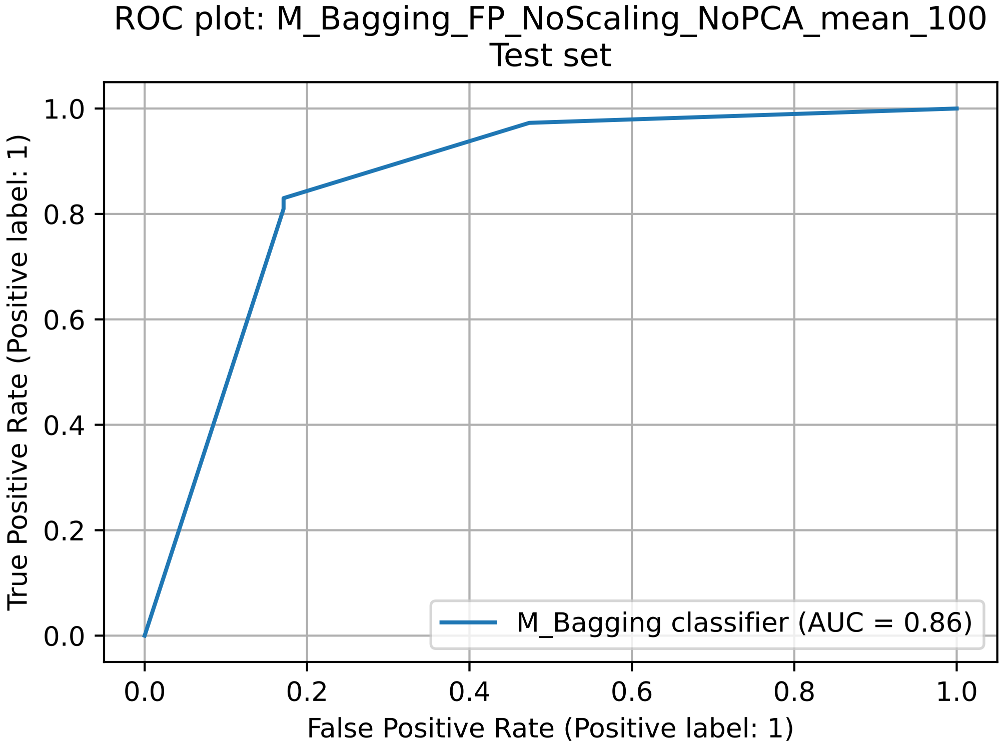

ROC plot: M_Bagging_FP_NoScaling_NoPCA_mean_100
Test set
True Positive Rate (Positive label: 1)
False Positive Rate (Positive label: 1)
0.0
0.2
0.4
0.6
0.8
1.0
M_Bagging classifier (AUC = 0.86)

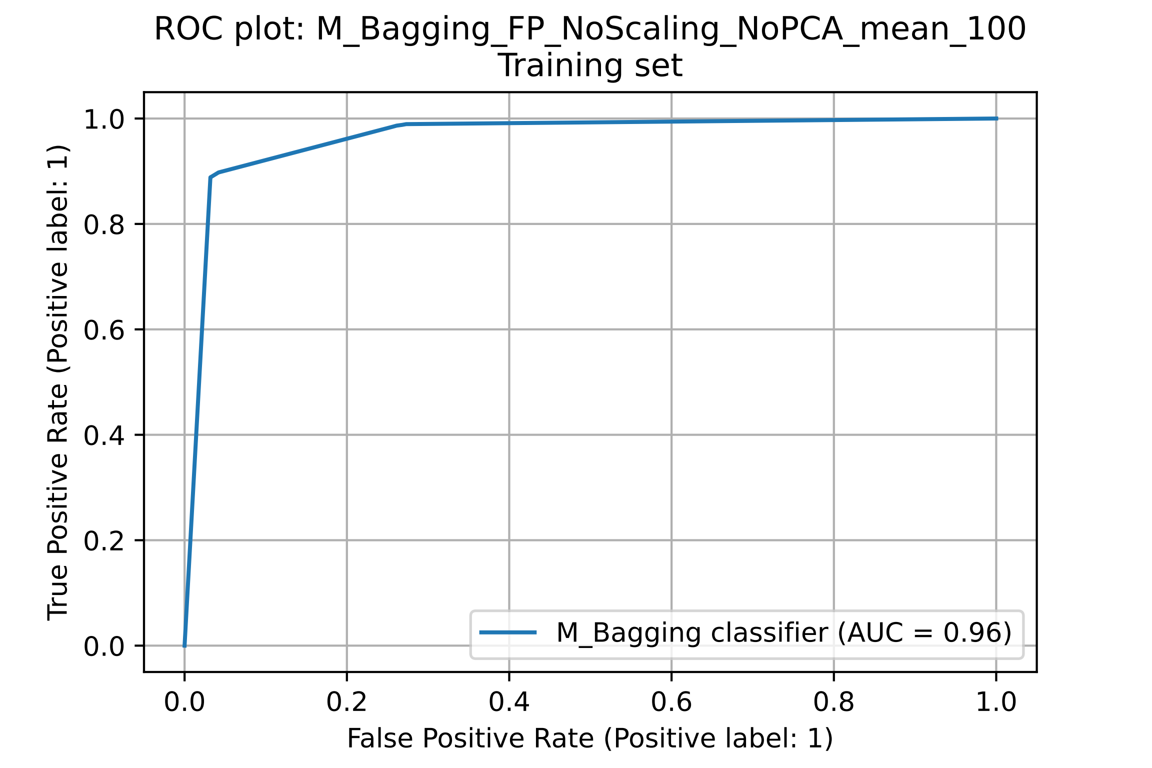
ROC plot: M_Bagging_FP_NoScaling_NoPCA_mean_100
Training set
True Positive Rate (Positive label: 1)
False Positive Rate (Positive label: 1)
0.0
0.2
0.4
0.6
0.8
1.0
0.0
0.2
0.4
0.6
0.8
1.0
M_Bagging classifier (AUC = 0.96)

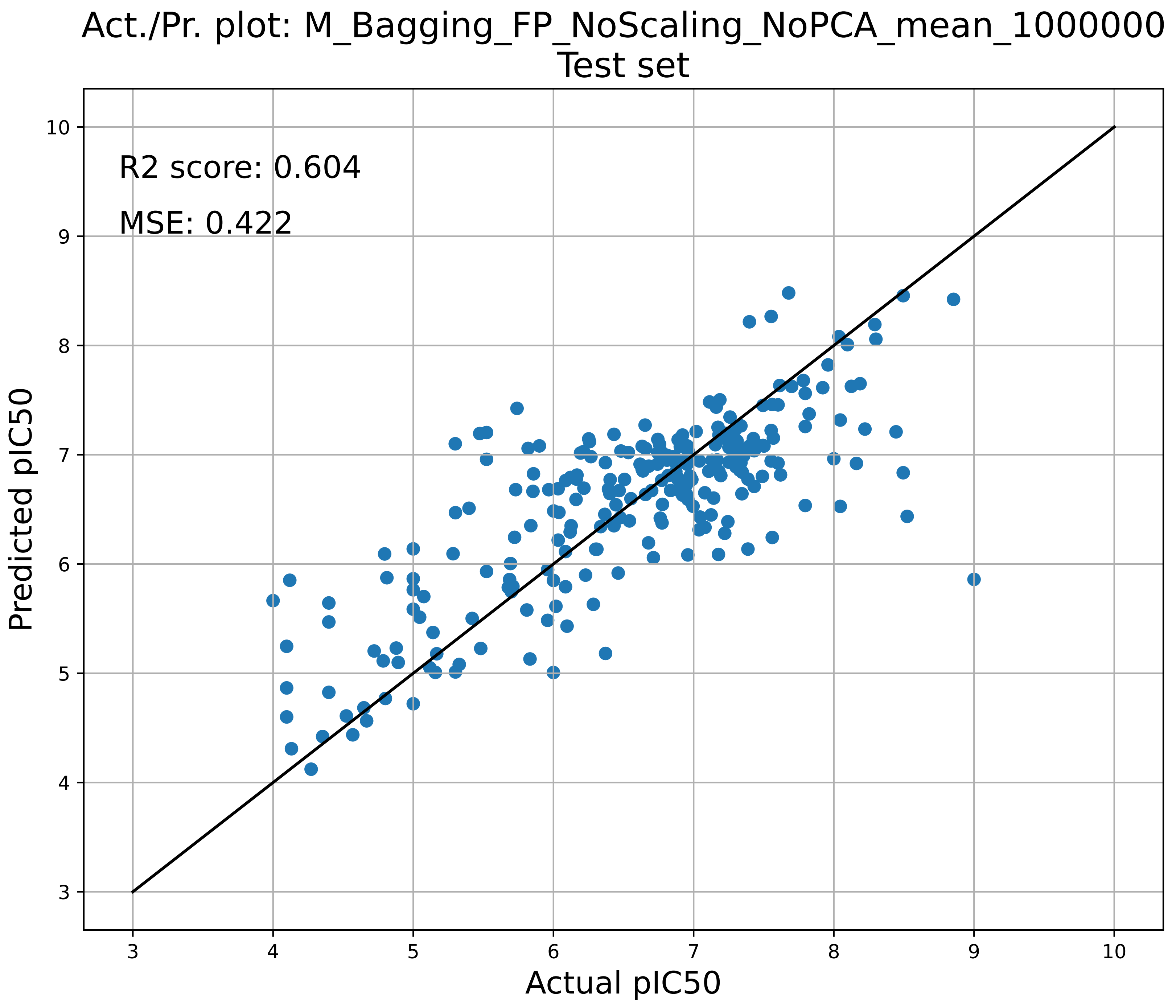

Act./Pr. plot: M_Bagging_FP_NoScaling_NoPCA_mean_1000000
Test set
R2 score: 0.604
MSE: 0.422
Predicted pIC50
Actual pIC50
3
4
5
6
7
8
9
10

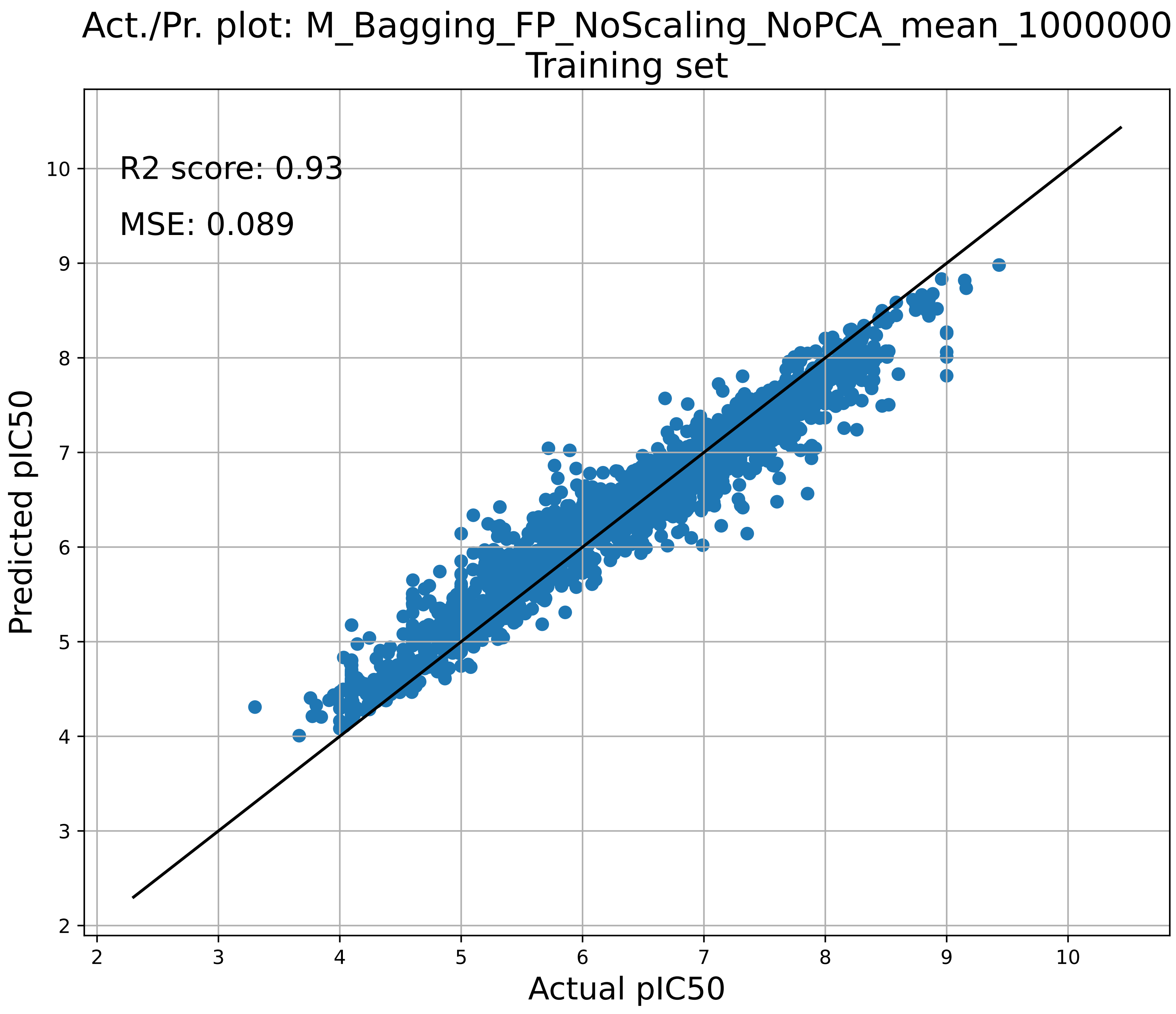
Act./Pr. plot: M_Bagging_FP_NoScaling_NoPCA_mean_1000000
Training set
R2 score: 0.93
MSE: 0.089
Predicted pIC50
Actual pIC50
2
3
4
5
6
7
8
9
10

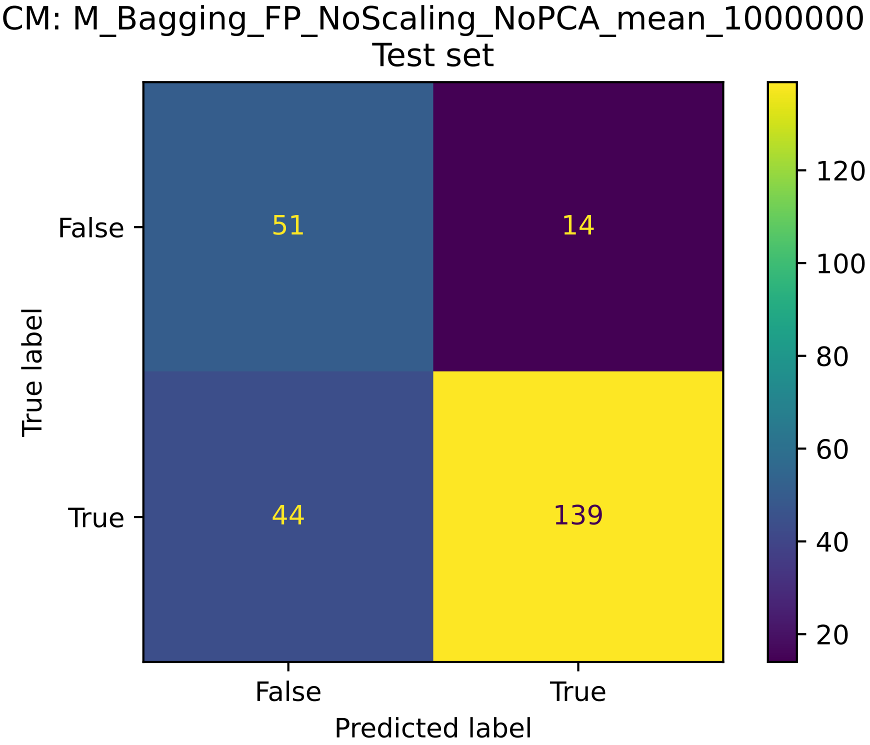
CM: M_Bagging_FP_NoScaling_NoPCA_mean_1000000
Test set
True label
False
True
51
14
44
139
False
True
Predicted label
20
40
60
80
100
120

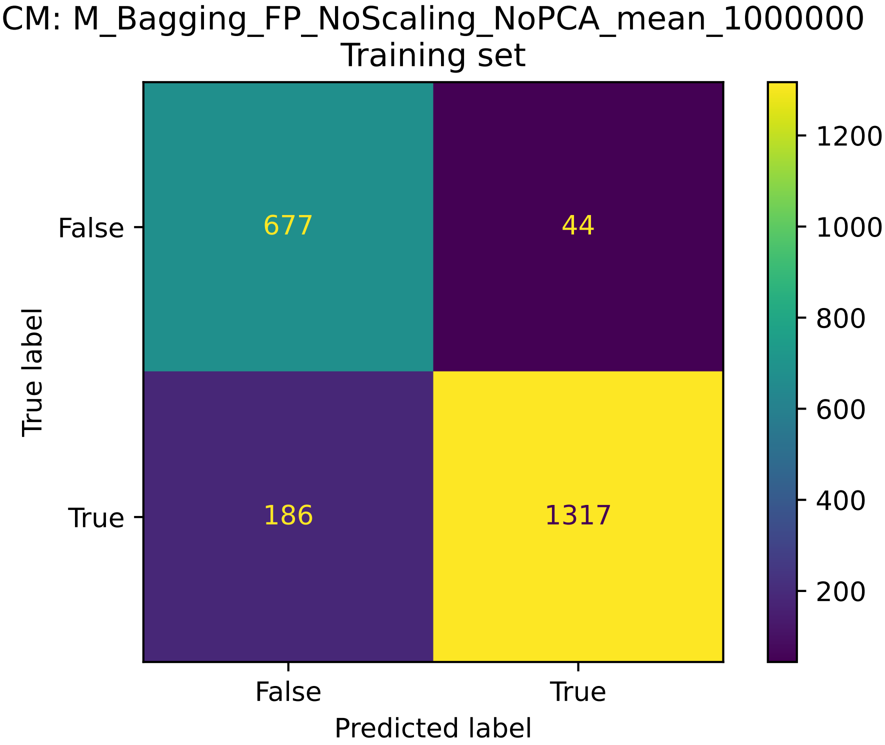
CM: M_Bagging_FP_NoScaling_NoPCA_mean_1000000
Training set
True label
False
True
677
44
186
1317
Predicted label
False
True
1200
1000
800
600
400
200

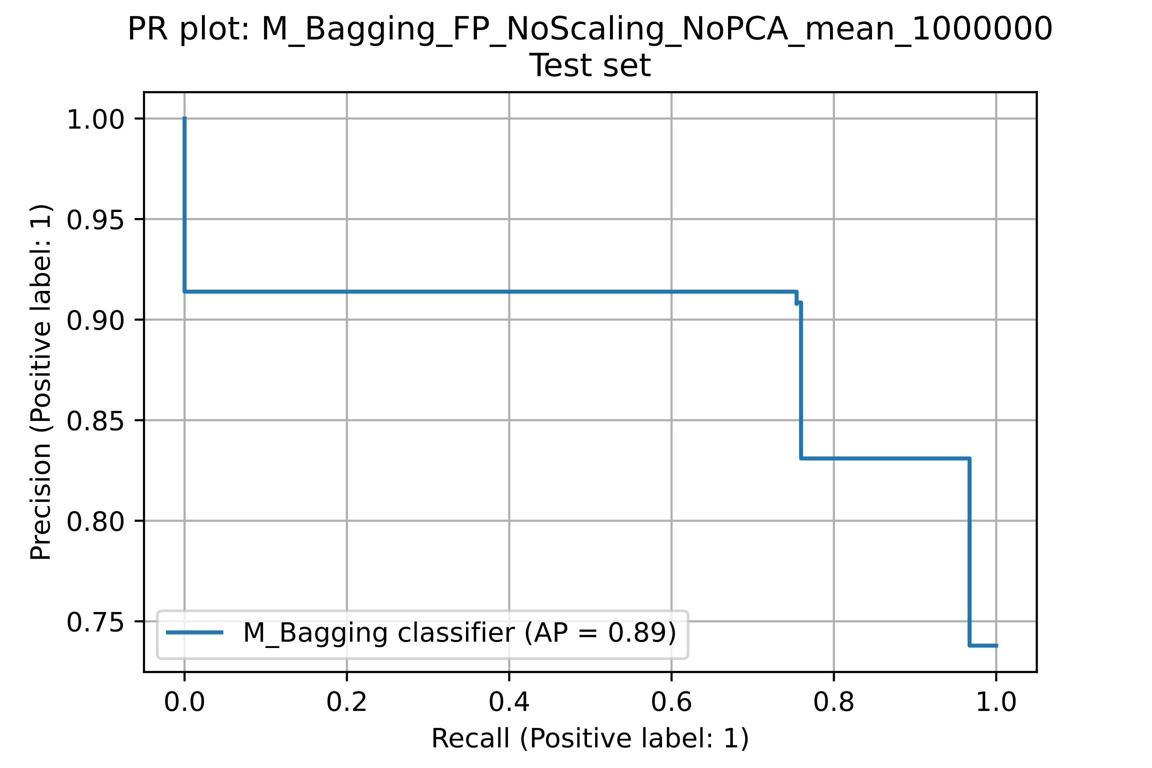

PR plot: M_Bagging_FP_NoScaling_NoPCA_mean_1000000
Test set
Precision (Positive label: 1)
Recall (Positive label: 1)
1.00
0.95
0.90
0.85
0.80
0.75
0.0
0.2
0.4
0.6
0.8
1.0
M_Bagging classifier (AP = 0.89)

PR plot: M_Bagging_FP_NoScaling_NoPCA_mean_1000000
Training set

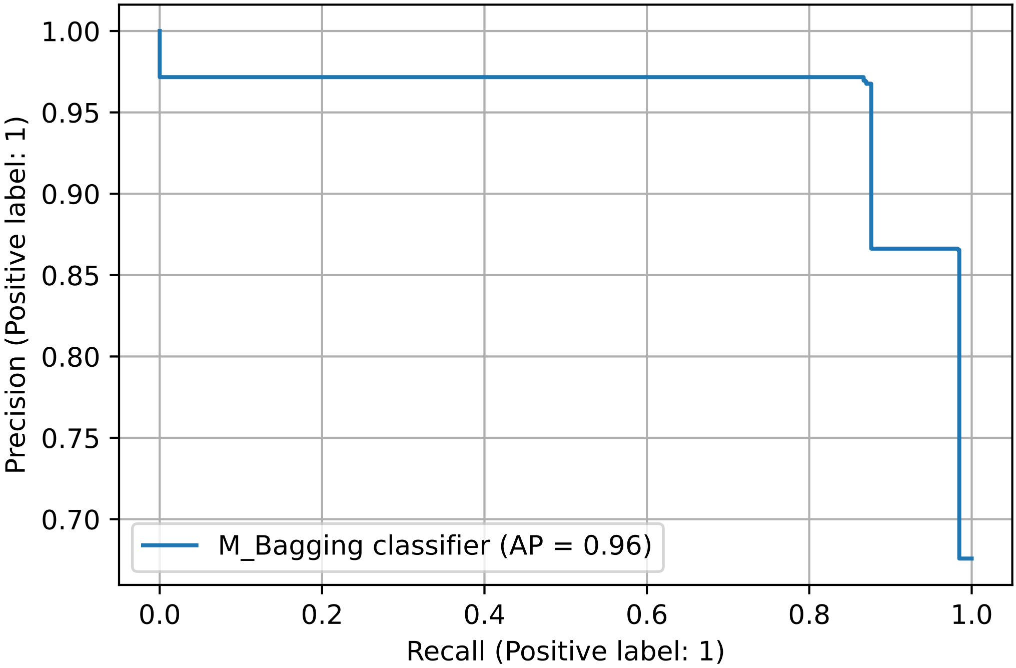

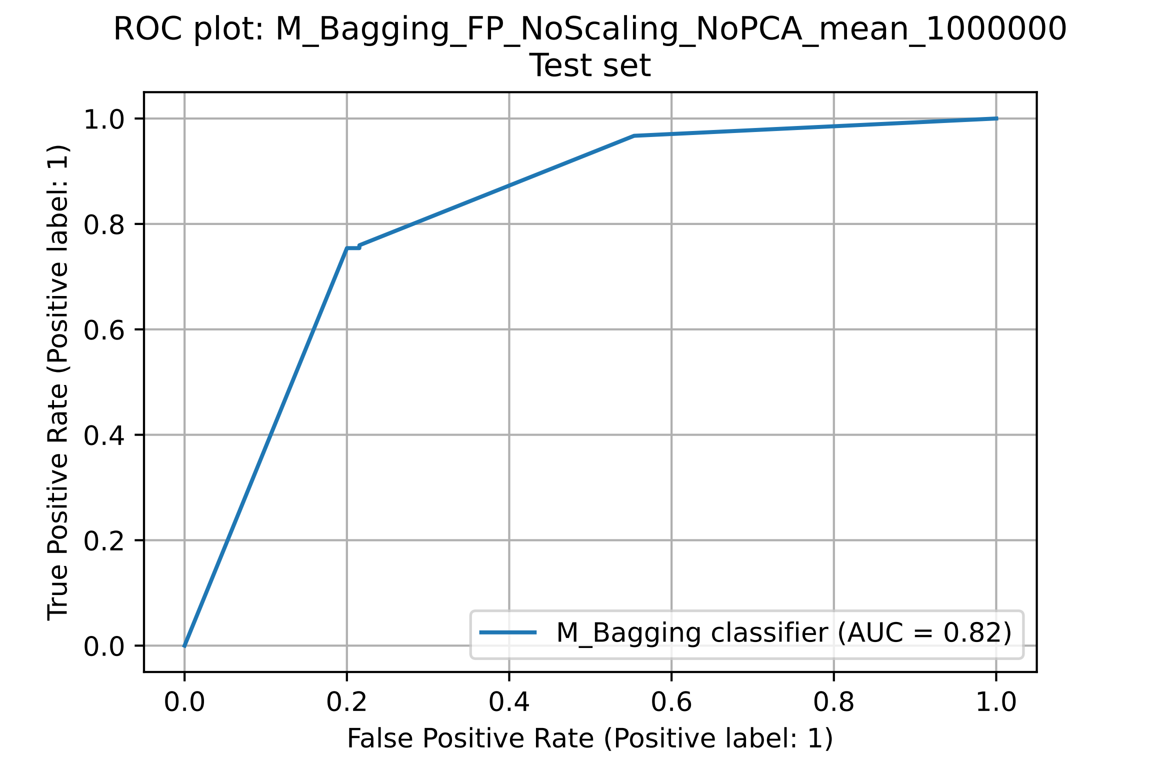

ROC plot: M_Bagging_FP_NoScaling_NoPCA_mean_1000000
Test set
True Positive Rate (Positive label: 1)
False Positive Rate (Positive label: 1)
0.0
0.2
0.4
0.6
0.8
1.0
M_Bagging classifier (AUC = 0.82)

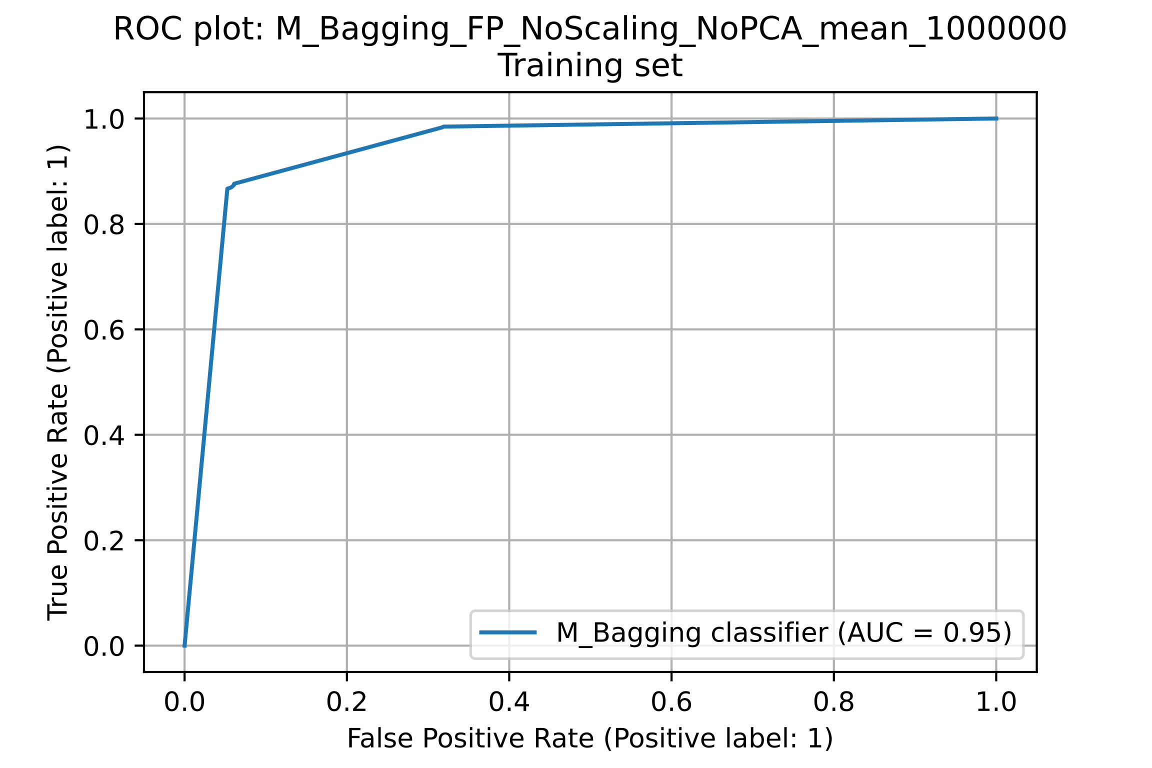
ROC plot: M_Bagging_FP_NoScaling_NoPCA_mean_1000000
Training set
True Positive Rate (Positive label: 1)
False Positive Rate (Positive label: 1)
0.0
0.2
0.4
0.6
0.8
1.0
M_Bagging classifier (AUC = 0.95)

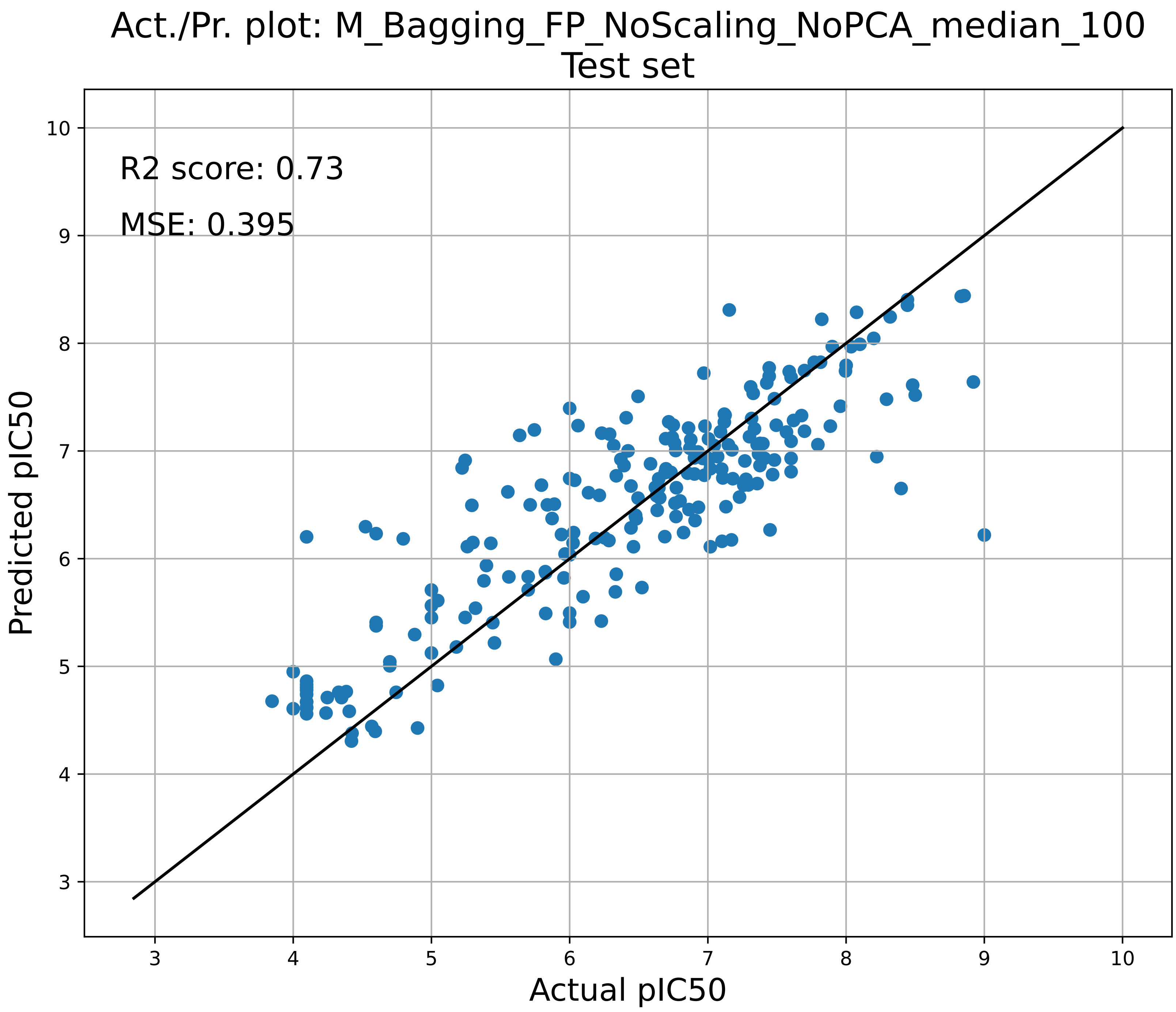
Act./Pr. plot: M_Bagging_FP_NoScaling_NoPCA_median_100
Test set
R2 score: 0.73
MSE: 0.395
Predicted pIC50
Actual pIC50
3
4
5
6
7
8
9
10

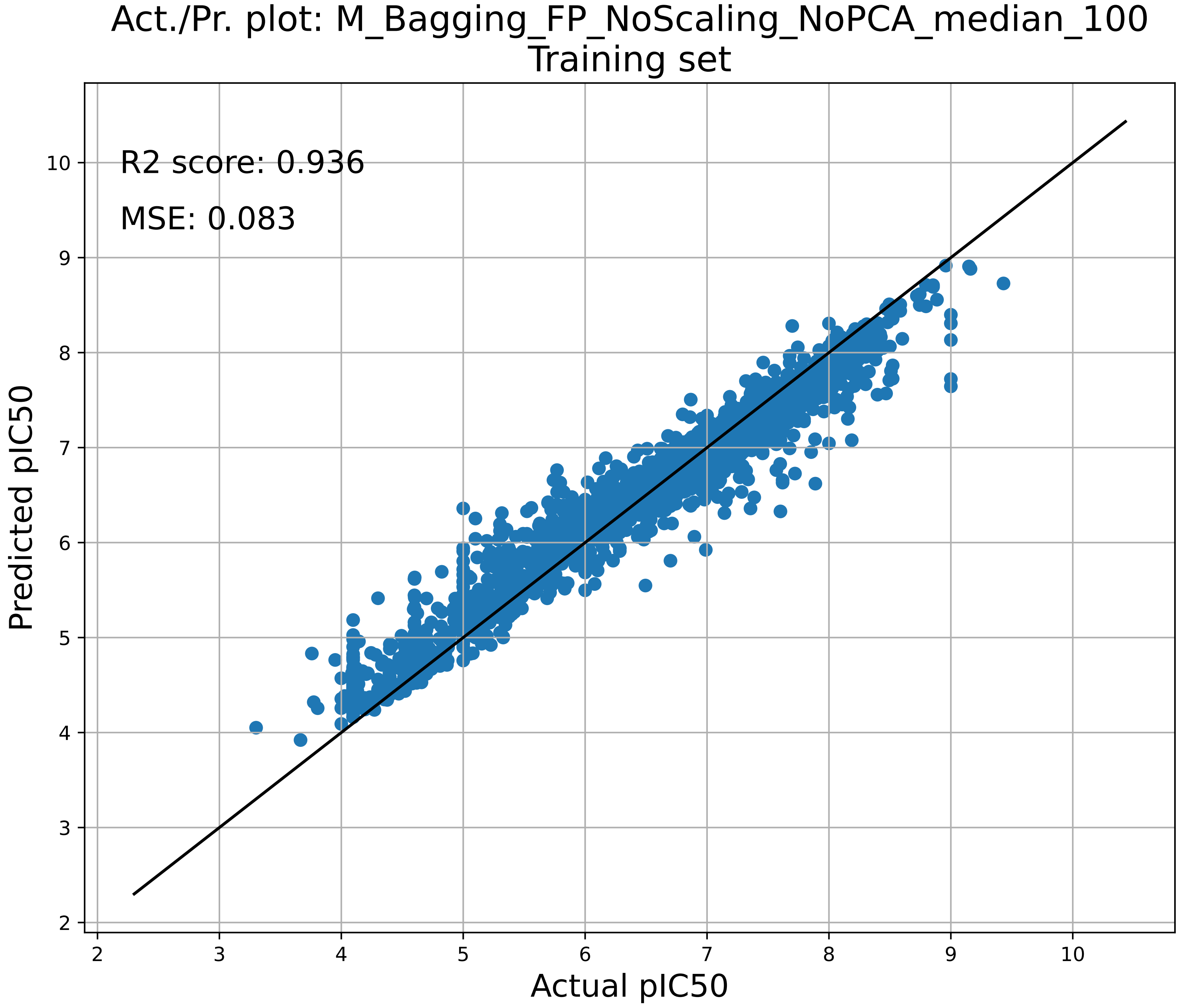

Act./Pr. plot: M_Bagging_FP_NoScaling_NoPCA_median_100
Training set
R2 score: 0.936
MSE: 0.083
Predicted pIC50
Actual pIC50
2
3
4
5
6
7
8
9
10

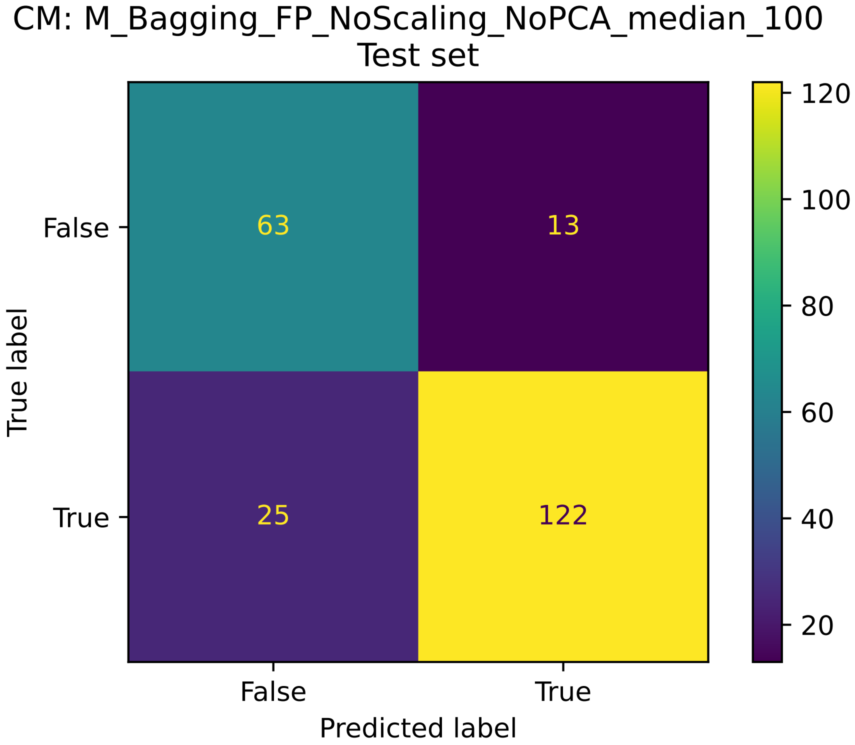
CM: M_Bagging_FP_NoScaling_NoPCA_median_100
Test set
True label
False
True
63
13
25
122
Predicted label
False
True
120
100
80
60
40
20

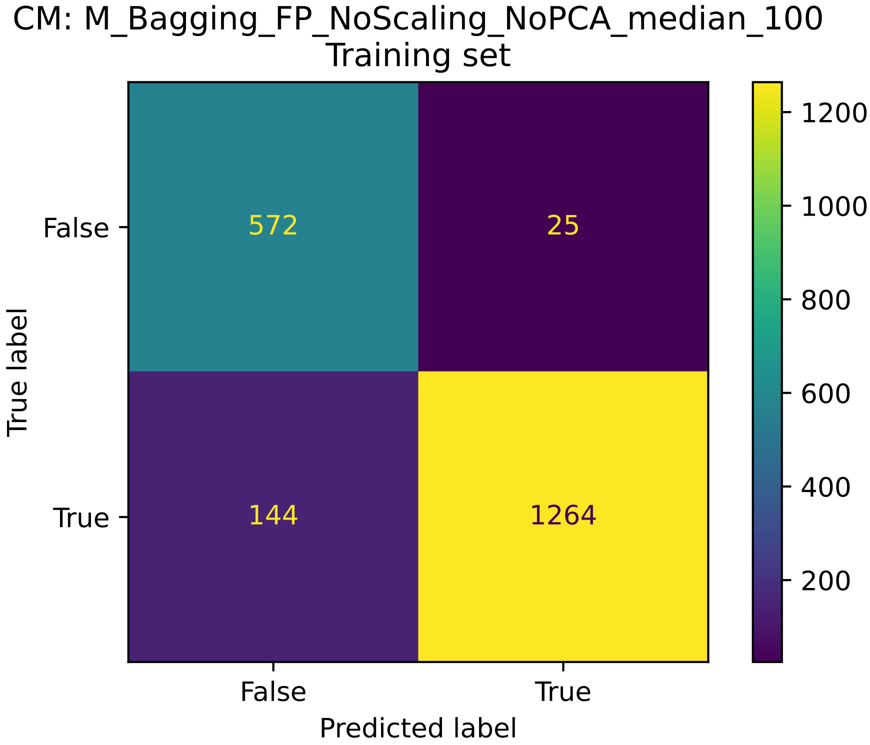
CM: M_Bagging_FP_NoScaling_NoPCA_median_100
Training set
572
25
144
1264
False
True
False
True
True label
Predicted label
1200
1000
800
600
400
200

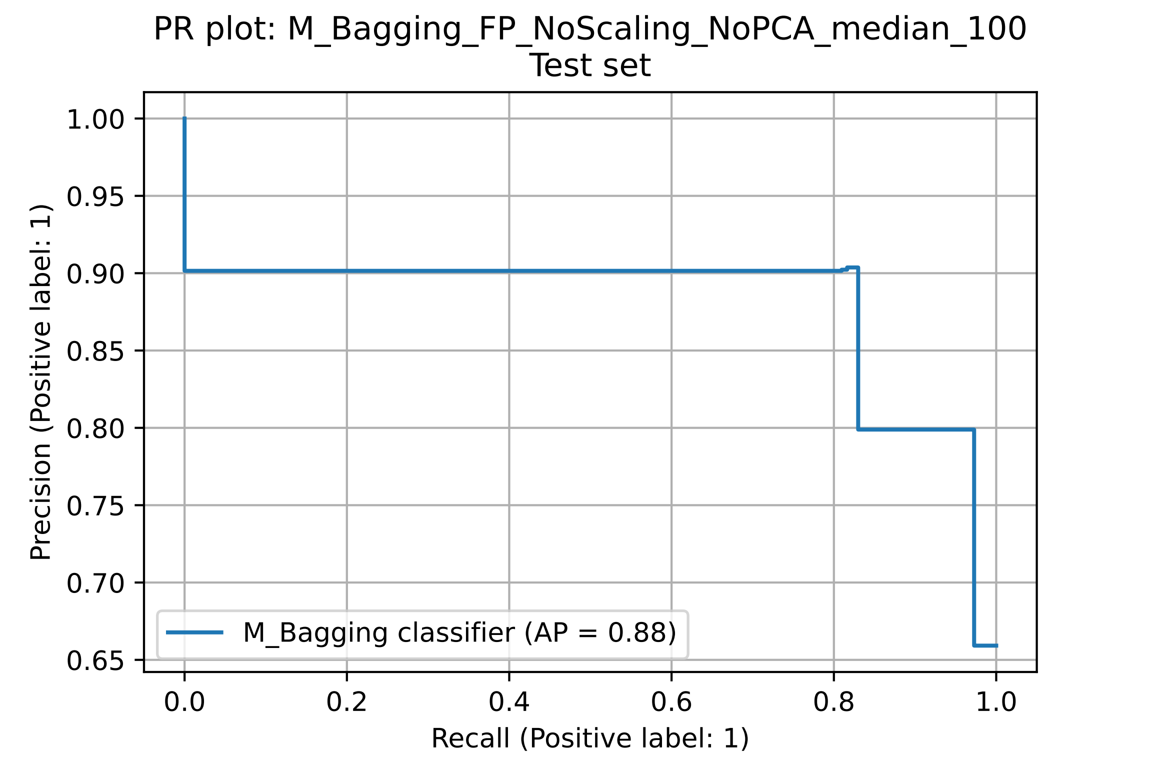

PR plot: M_Bagging_FP_NoScaling_NoPCA_median_100
Test set
Precision (Positive label: 1)
Recall (Positive label: 1)
1.00
0.95
0.90
0.85
0.80
0.75
0.70
0.65
0.0
0.2
0.4
0.6
0.8
1.0
M_Bagging classifier (AP = 0.88)

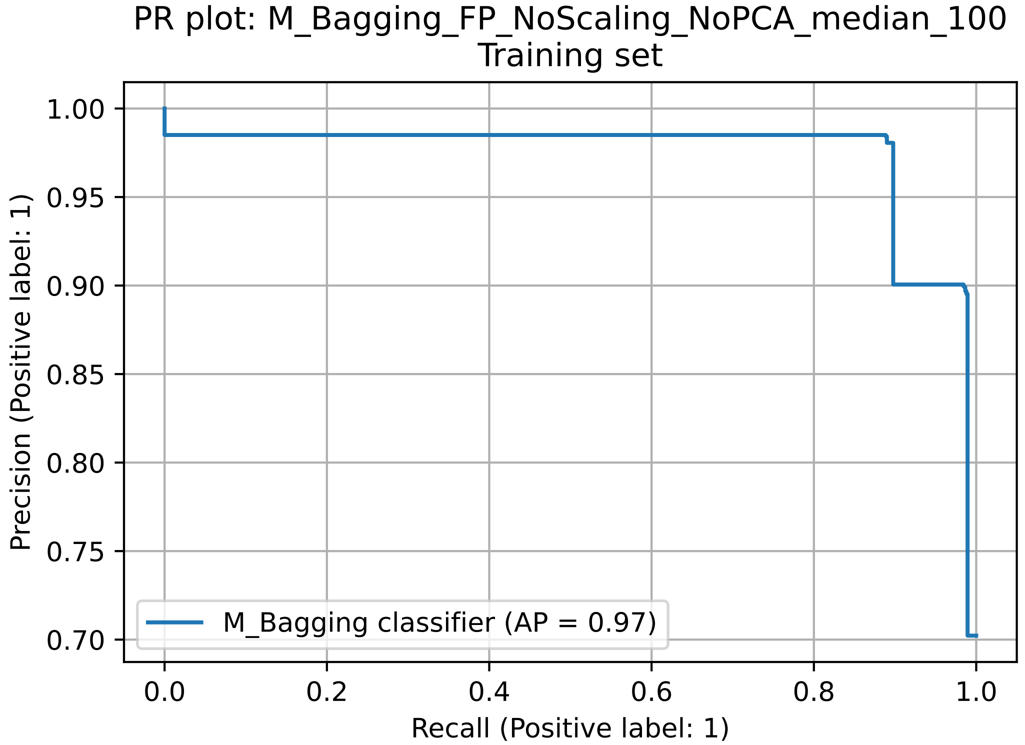
PR plot: M_Bagging_FP_NoScaling_NoPCA_median_100
Training set
Precision (Positive label: 1)
Recall (Positive label: 1)
1.00
0.95
0.90
0.85
0.80
0.75
0.70
0.0
0.2
0.4
0.6
0.8
1.0
M_Bagging classifier (AP = 0.97)

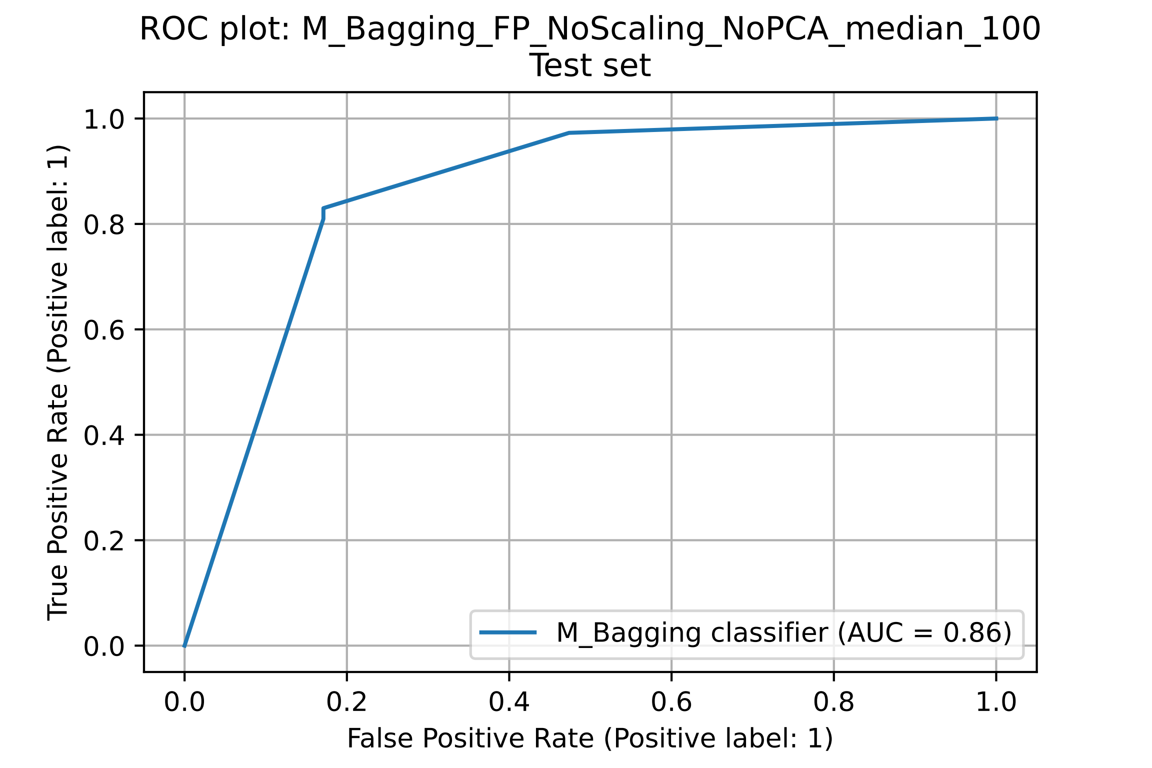

ROC plot: M_Bagging_FP_NoScaling_NoPCA_median_100
Test set
True Positive Rate (Positive label: 1)
False Positive Rate (Positive label: 1)
0.0
0.2
0.4
0.6
0.8
1.0
M_Bagging classifier (AUC = 0.86)

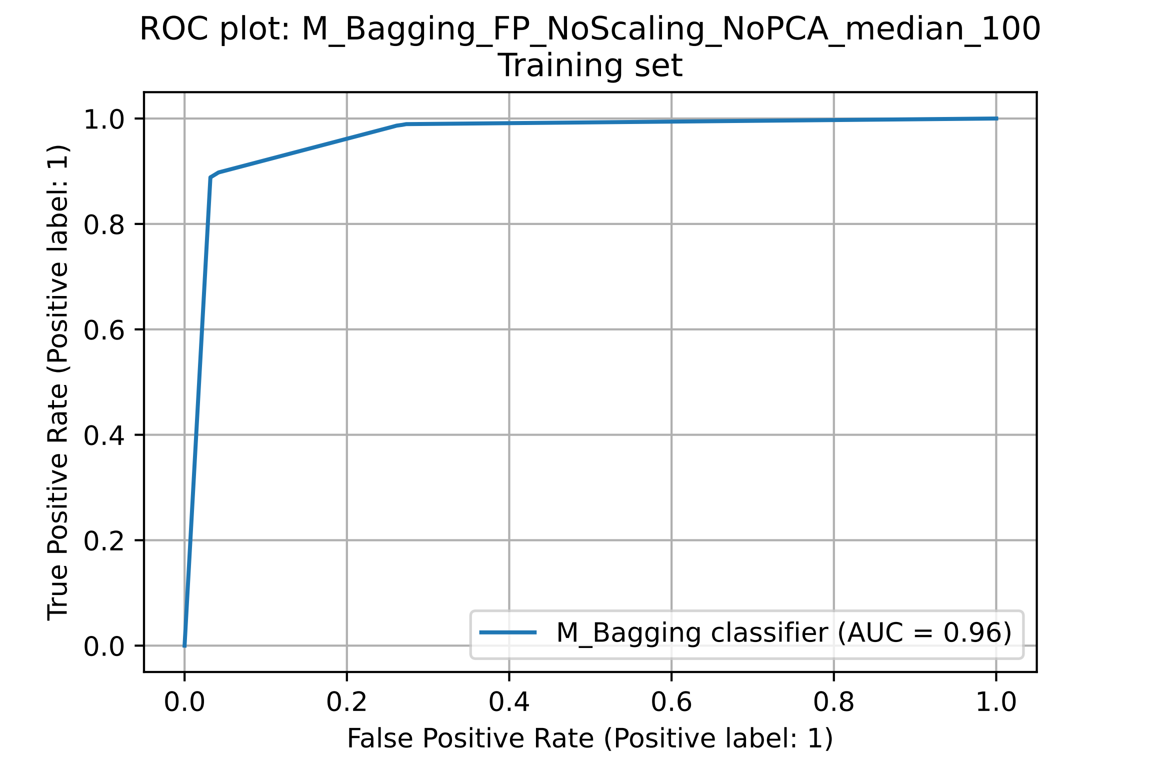
ROC plot: M_Bagging_FP_NoScaling_NoPCA_median_100
Training set
True Positive Rate (Positive label: 1)
False Positive Rate (Positive label: 1)
0.0
0.2
0.4
0.6
0.8
1.0
M_Bagging classifier (AUC = 0.96)

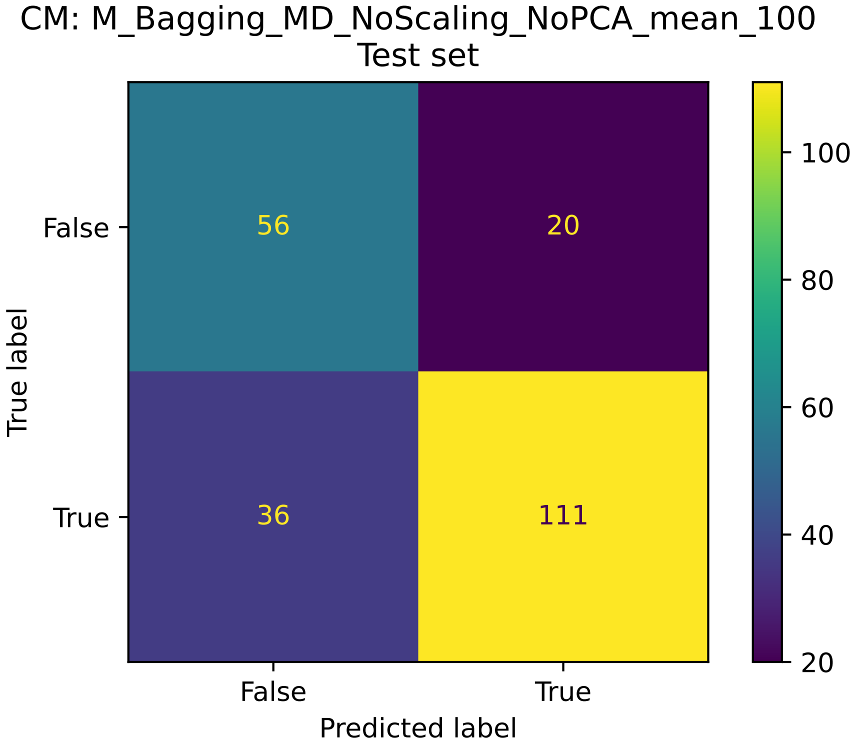

CM: M_Bagging_MD_NoScaling_NoPCA_mean_100
Test set
True label
False
True
56
20
36
111
Predicted label
False
True
100
80
60
40
20

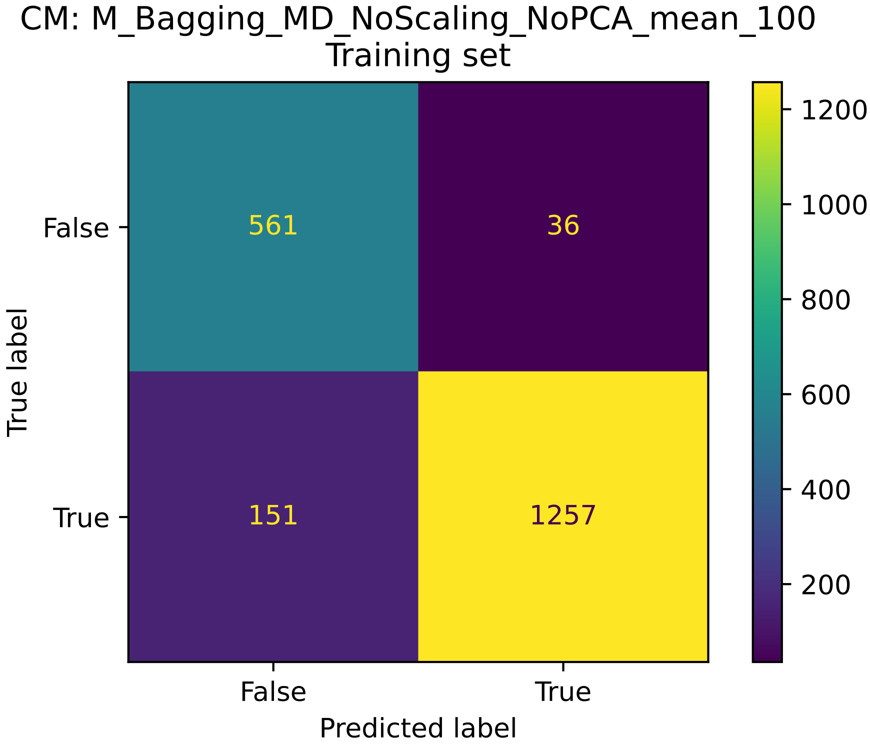

CM: M_Bagging_MD_NoScaling_NoPCA_mean_100
Training set
561
36
151
1257
False
True
True label
False
True
Predicted label
1200
1000
800
600
400
200

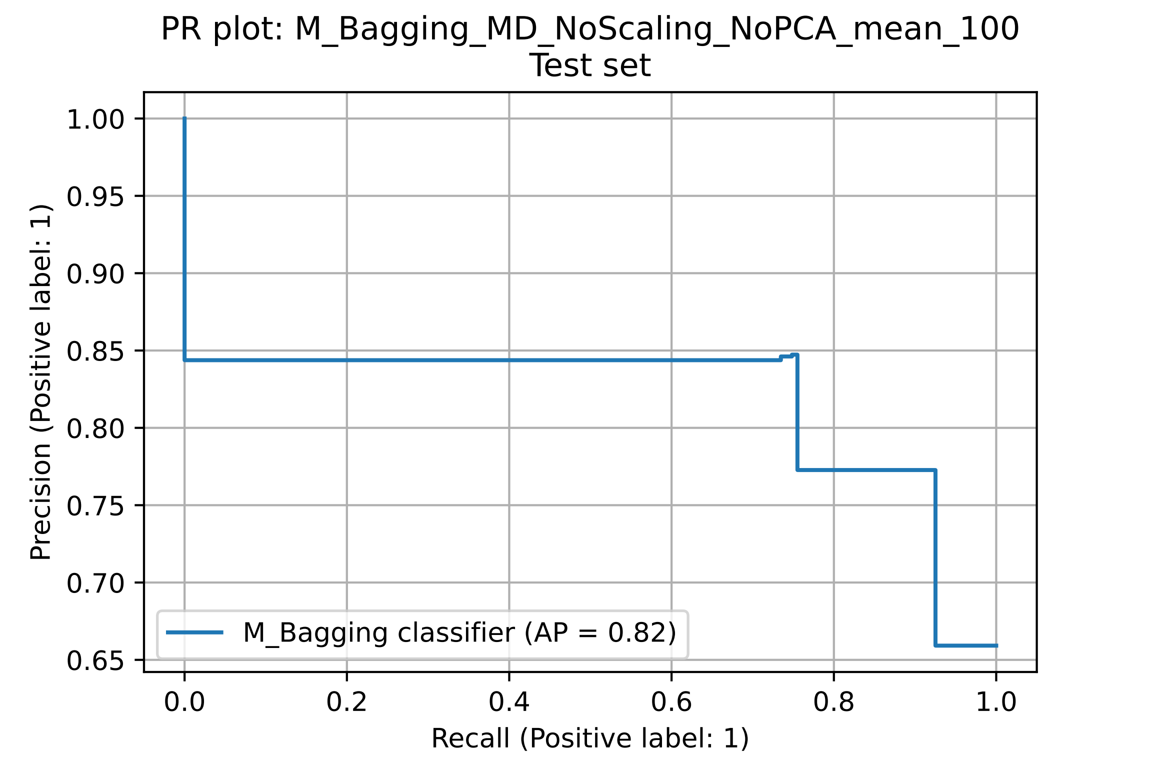
PR plot: M_Bagging_MD_NoScaling_NoPCA_mean_100
Test set
Precision (Positive label: 1)
Recall (Positive label: 1)
1.00
0.95
0.90
0.85
0.80
0.75
0.70
0.65
0.0
0.2
0.4
0.6
0.8
1.0
M_Bagging classifier (AP = 0.82)

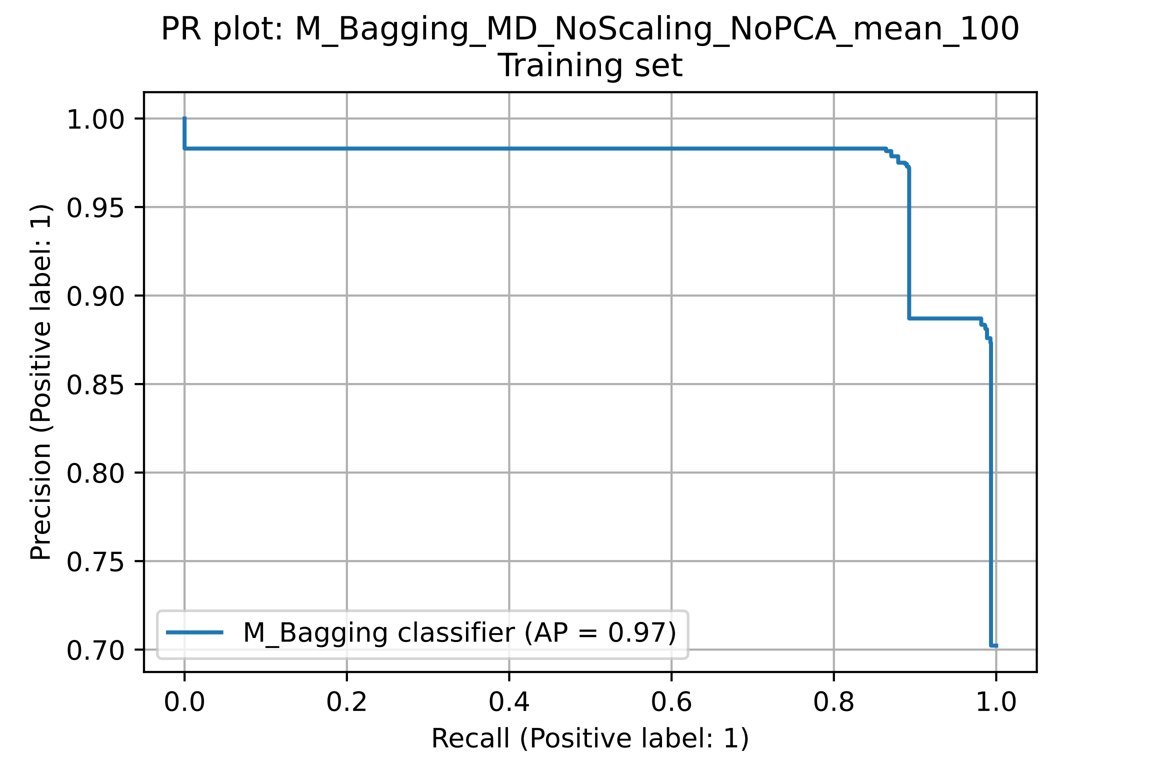
PR plot: M_Bagging_MD_NoScaling_NoPCA_mean_100
Training set
Precision (Positive label: 1)
Recall (Positive label: 1)
1.00
0.95
0.90
0.85
0.80
0.75
0.70
0.0
0.2
0.4
0.6
0.8
1.0
M_Bagging classifier (AP = 0.97)

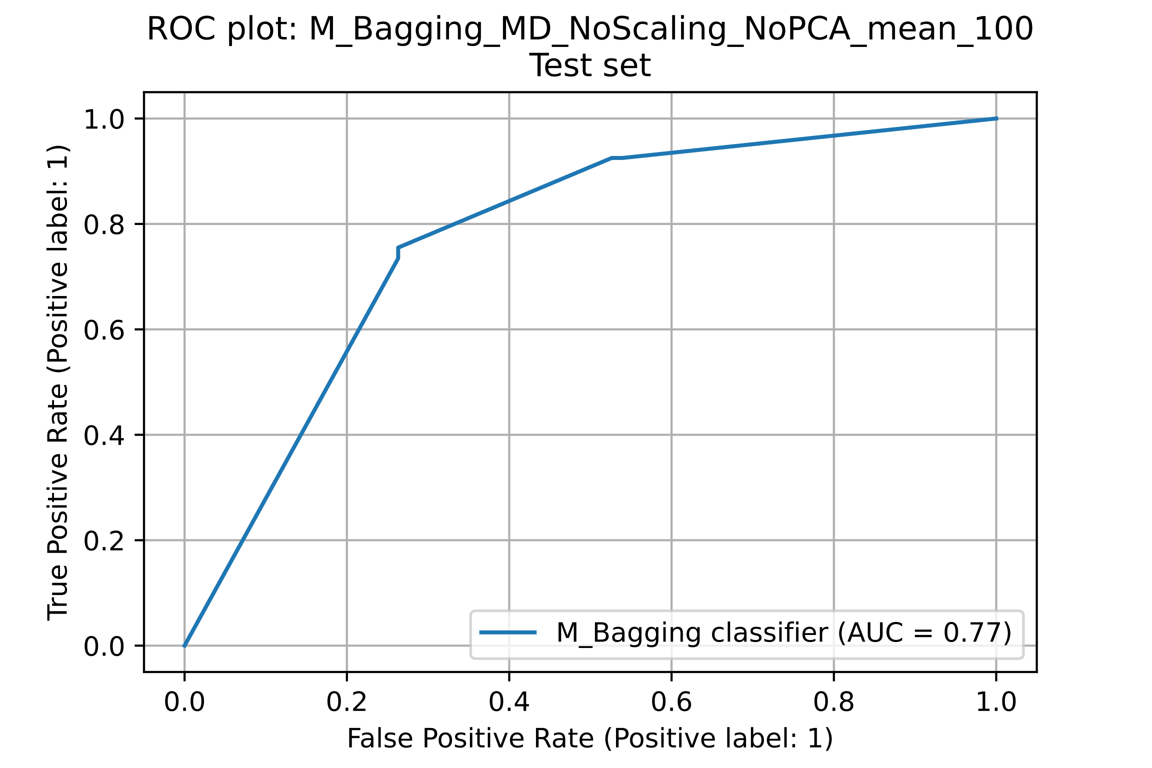

ROC plot: M_Bagging_MD_NoScaling_NoPCA_mean_100
Test set
1.0
0.8
0.6
0.4
0.2
0.0
True Positive Rate (Positive label: 1)
0.0
0.2
0.4
0.6
0.8
1.0
False Positive Rate (Positive label: 1)
M_Bagging classifier (AUC = 0.77)

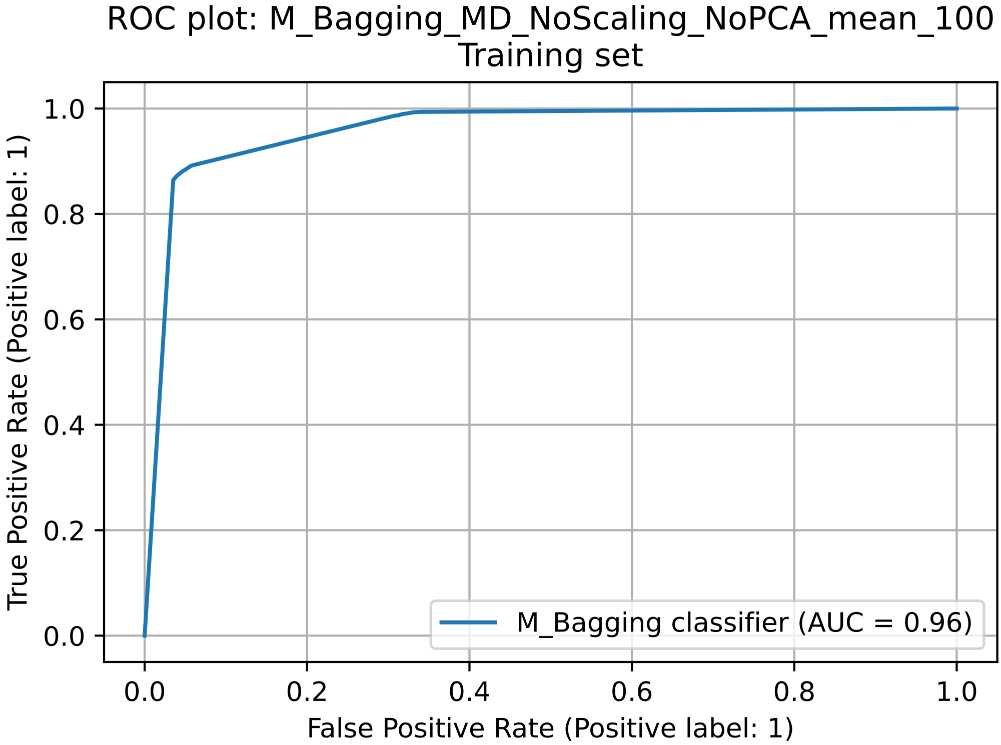
ROC plot: M_Bagging_MD_NoScaling_NoPCA_mean_100
Training set
True Positive Rate (Positive label: 1)
False Positive Rate (Positive label: 1)
0.0
0.2
0.4
0.6
0.8
1.0
M_Bagging classifier (AUC = 0.96)

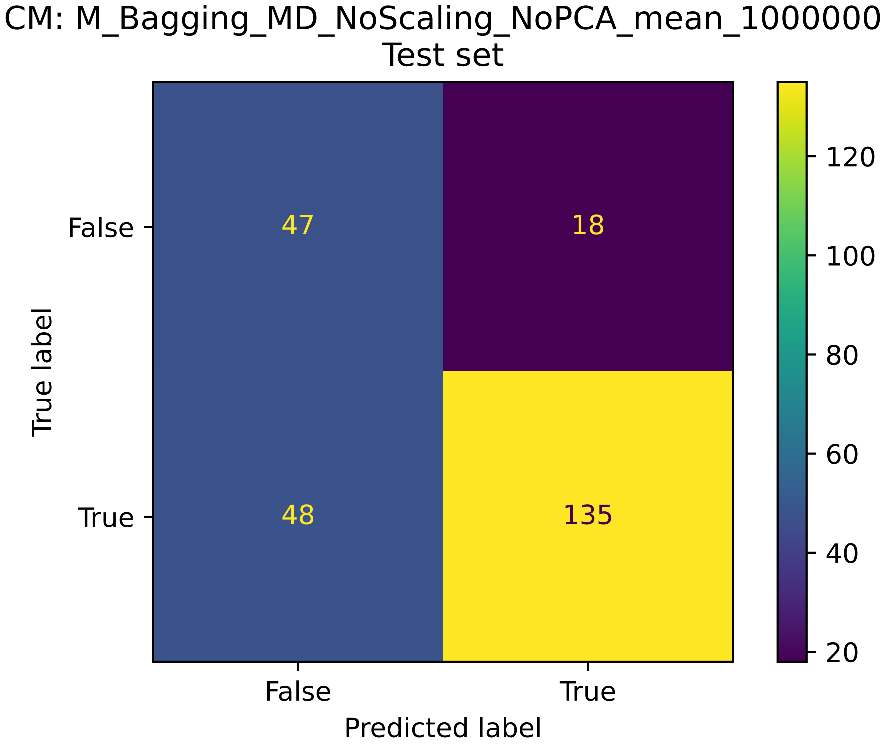

CM: M_Bagging_MD_NoScaling_NoPCA_mean_1000000
Test set
47
18
48
135
False
True
True label
False
True
Predicted label
120
100
80
60
40
20

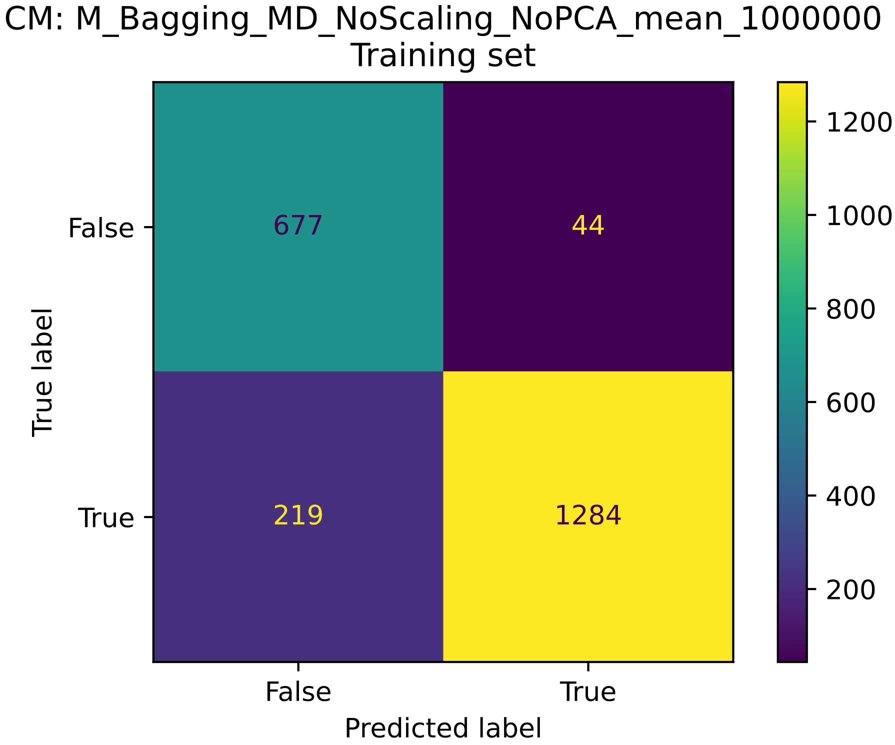

CM: M_Bagging_MD_NoScaling_NoPCA_mean_1000000
Training set
677
44
219
1284
False
True
True label
False
True
Predicted label
1200
1000
800
600
400
200

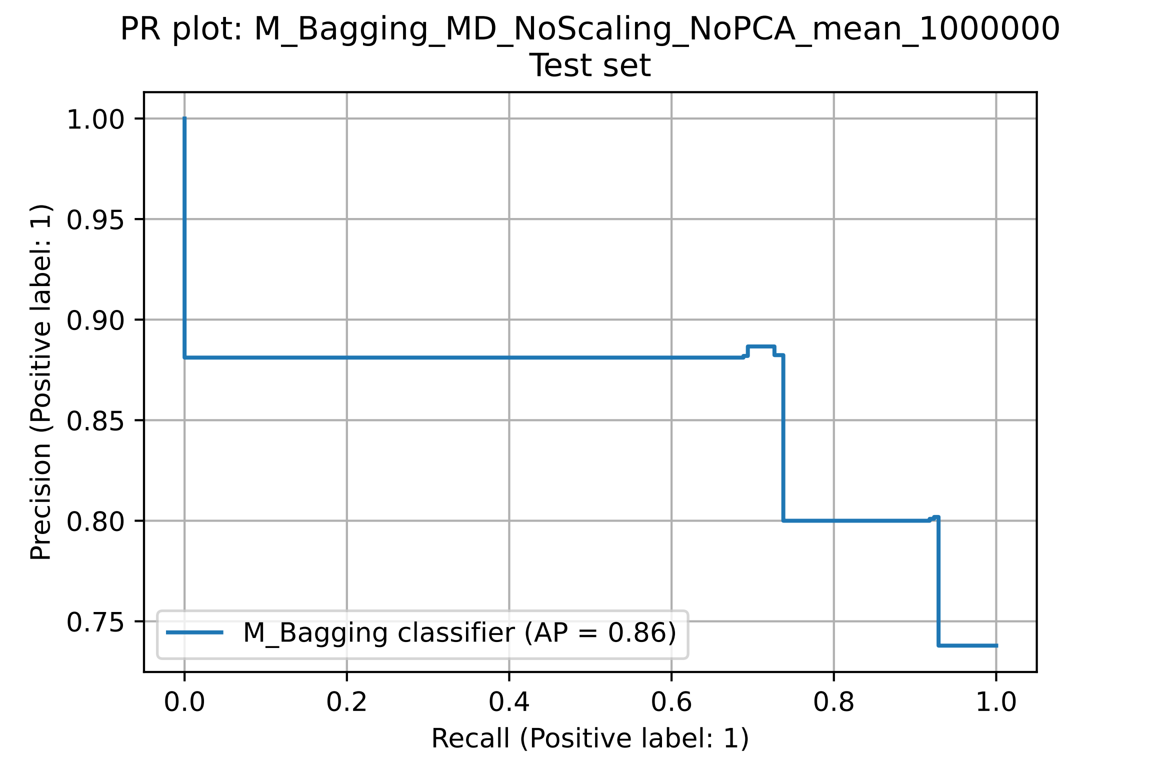

PR plot: M_Bagging_MD_NoScaling_NoPCA_mean_1000000
Test set
Precision (Positive label: 1)
Recall (Positive label: 1)
1.00
0.95
0.90
0.85
0.80
0.75
0.0
0.2
0.4
0.6
0.8
1.0
M_Bagging classifier (AP = 0.86)

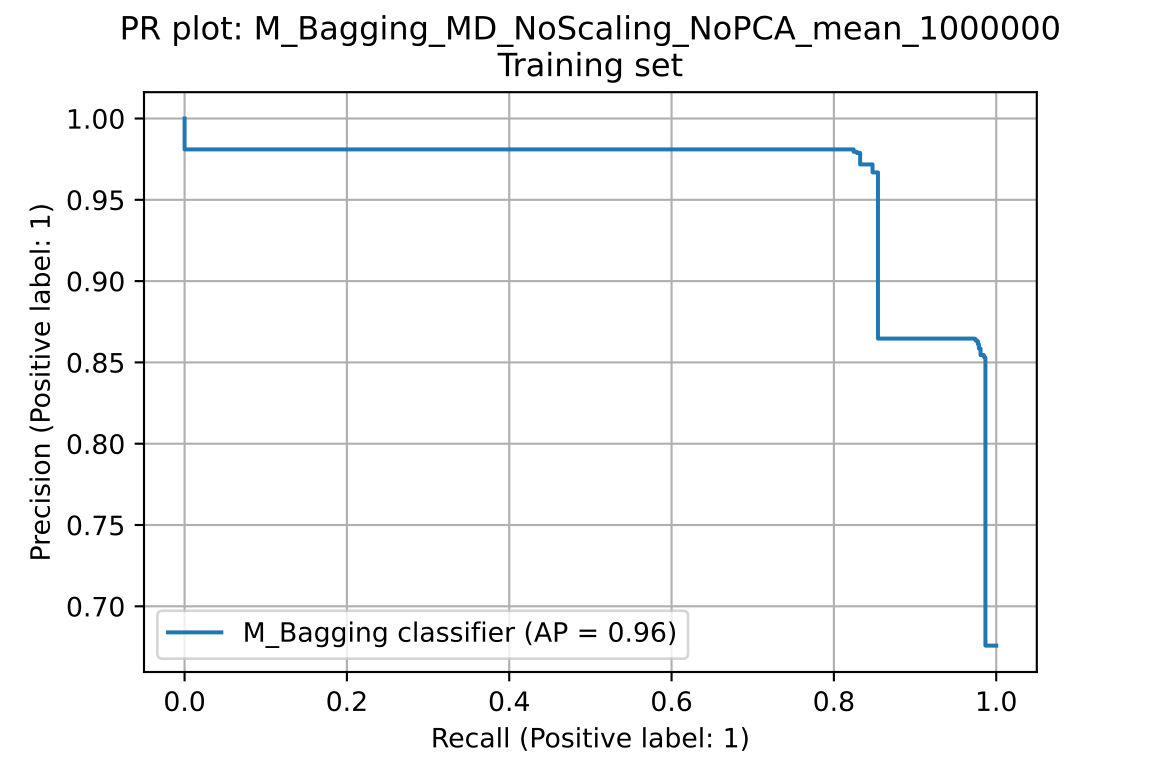

PR plot: M_Bagging_MD_NoScaling_NoPCA_mean_1000000
Training set
1.00
0.95
0.90
0.85
0.80
0.75
0.70
Precision (Positive label: 1)
0.0
0.2
0.4
0.6
0.8
1.0
Recall (Positive label: 1)
M_Bagging classifier (AP = 0.96)

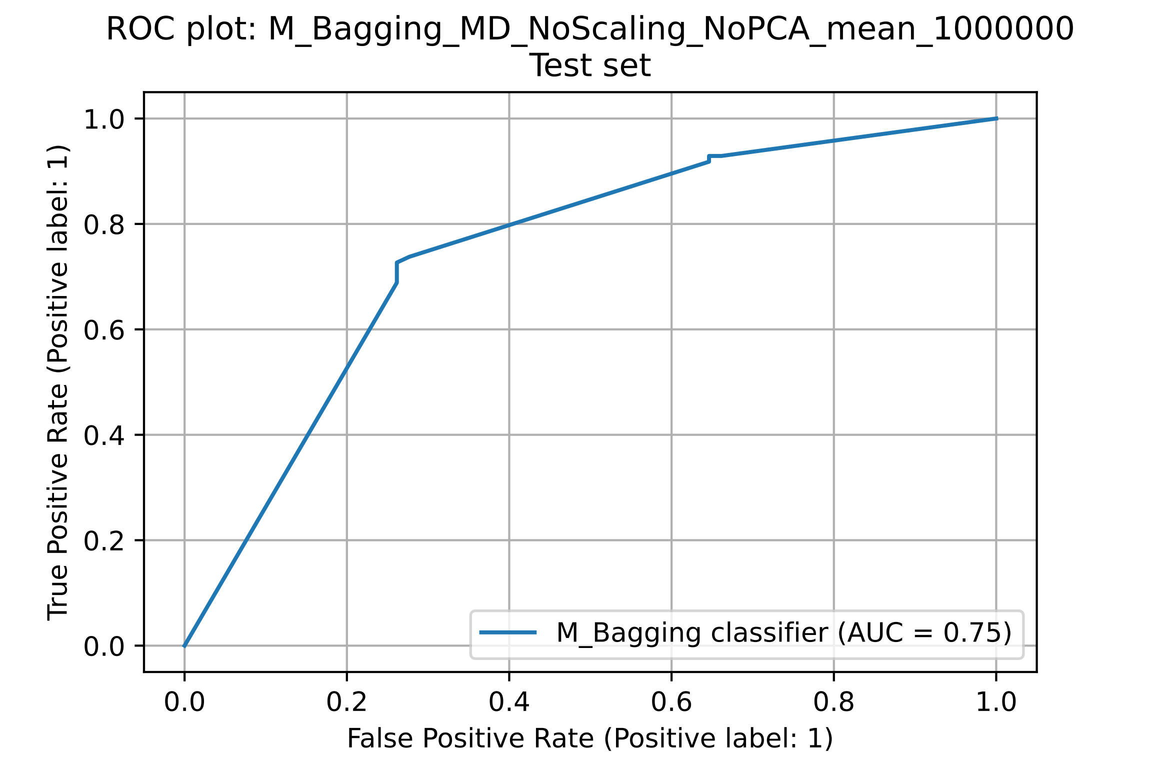
ROC plot: M_Bagging_MD_NoScaling_NoPCA_mean_1000000
Test set
True Positive Rate (Positive label: 1)
False Positive Rate (Positive label: 1)
0.0
0.2
0.4
0.6
0.8
1.0
M_Bagging classifier (AUC = 0.75)

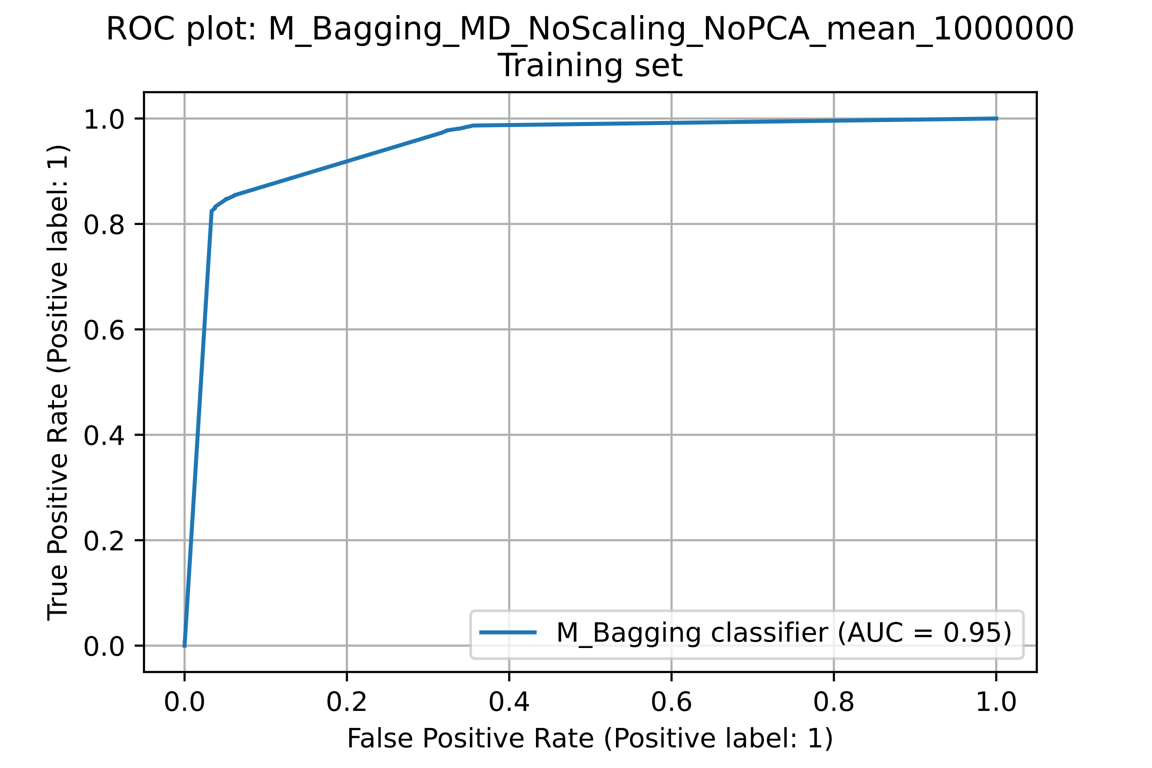
ROC plot: M_Bagging_MD_NoScaling_NoPCA_mean_1000000
Training set
True Positive Rate (Positive label: 1)
False Positive Rate (Positive label: 1)
0.0
0.2
0.4
0.6
0.8
1.0
M_Bagging classifier (AUC = 0.95)

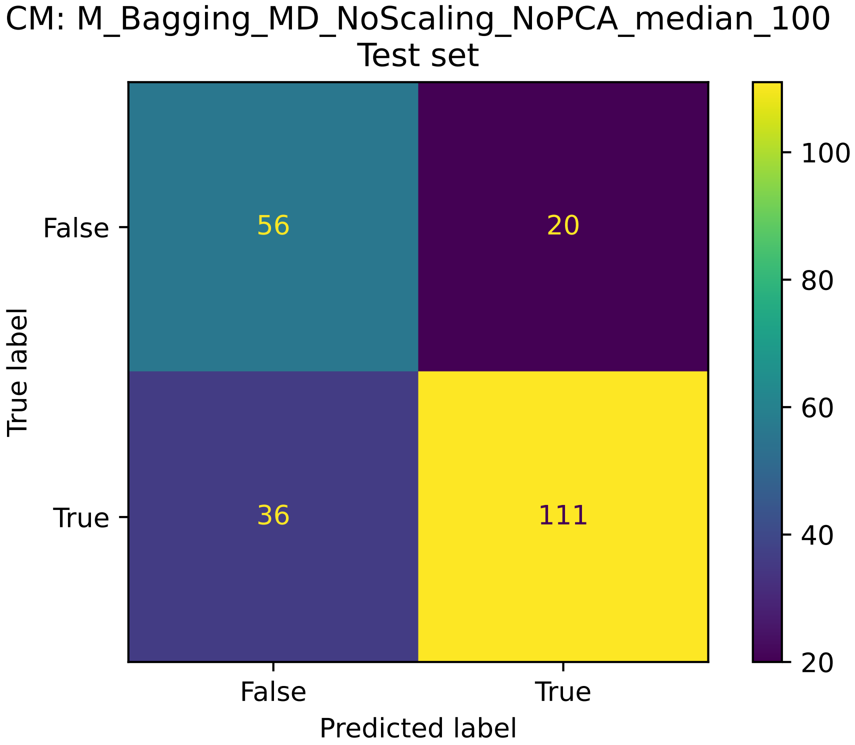

CM: M_Bagging_MD_NoScaling_NoPCA_median_100
Test set
True label
False
True
56
20
36
111
Predicted label
False
True
100
80
60
40
20

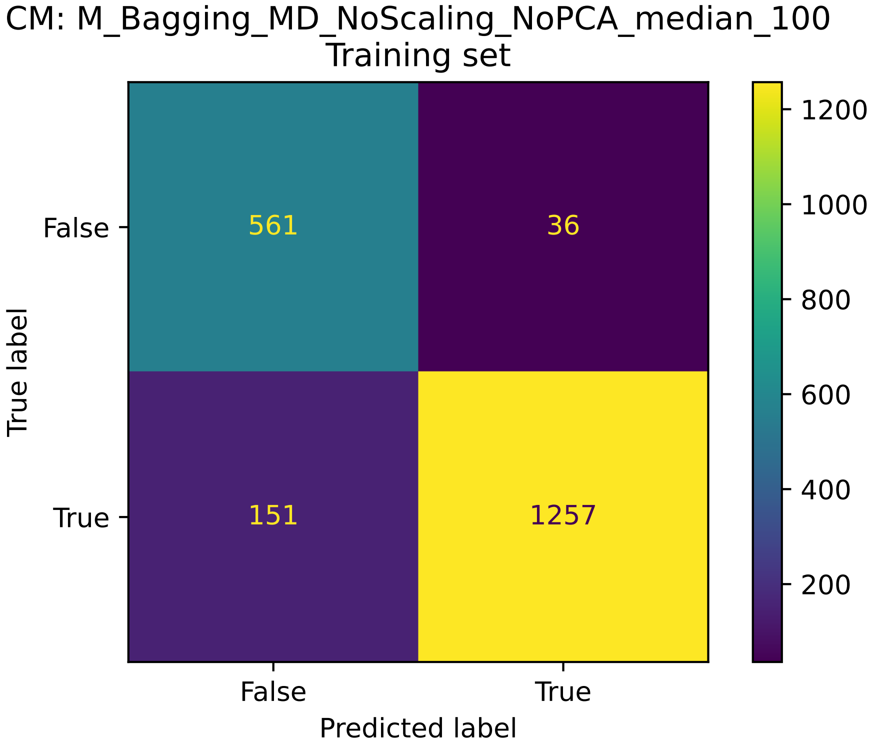
CM: M_Bagging_MD_NoScaling_NoPCA_median_100
Training set
True label
False
True
561
36
151
1257
Predicted label
False
True
1200
1000
800
600
400
200

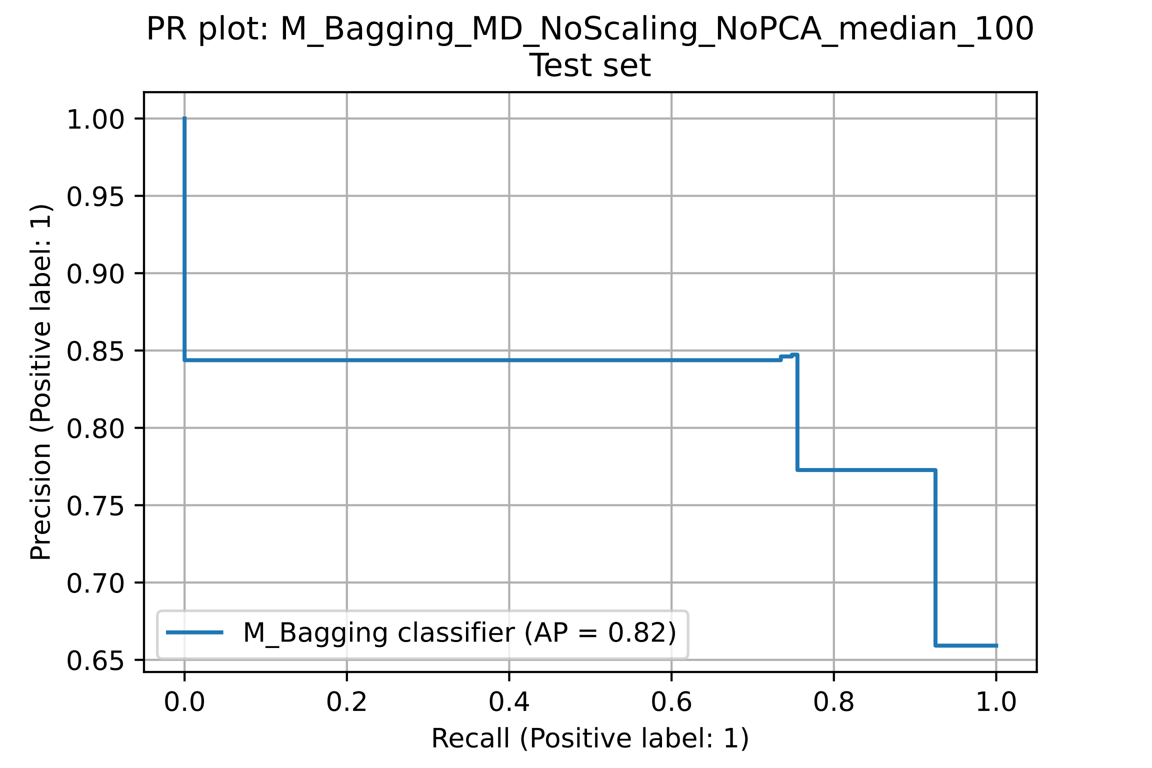
PR plot: M_Bagging_MD_NoScaling_NoPCA_median_100
Test set
1.00
0.95
0.90
0.85
0.80
0.75
0.70
0.65
Precision (Positive label: 1)
0.0
0.2
0.4
0.6
0.8
1.0
Recall (Positive label: 1)
M_Bagging classifier (AP = 0.82)

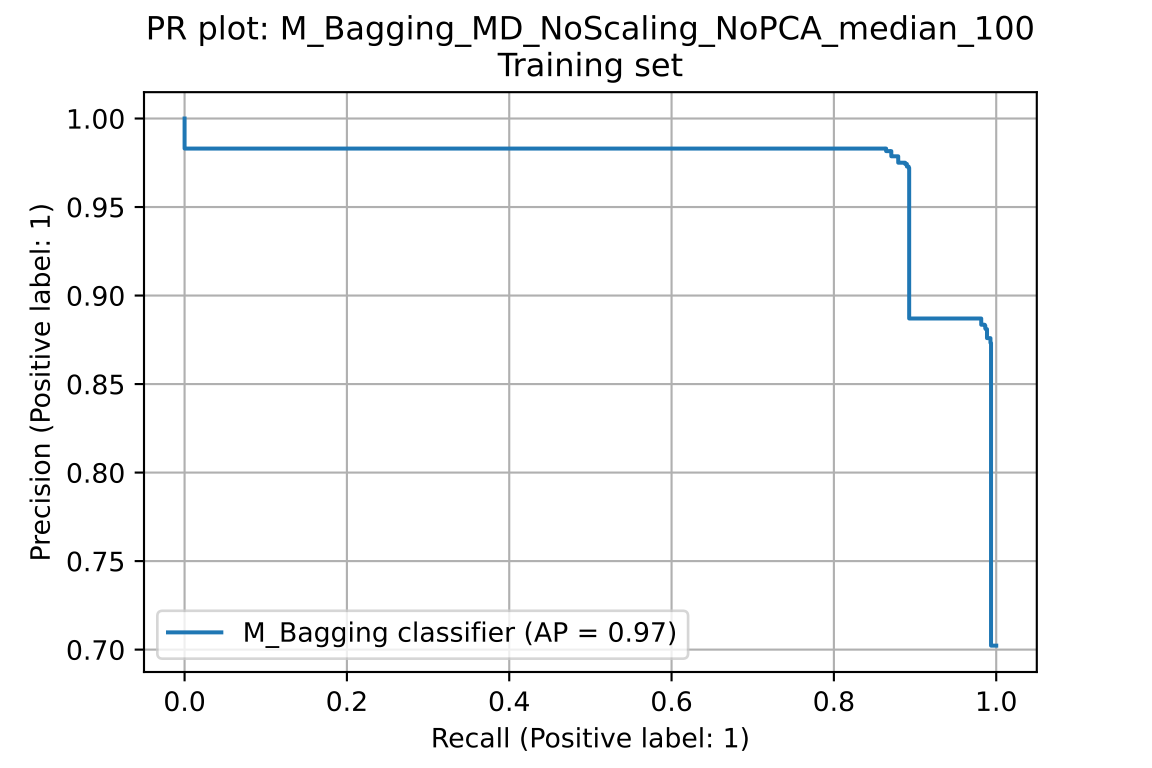
PR plot: M_Bagging_MD_NoScaling_NoPCA_median_100
Training set
1.00
0.95
0.90
0.85
0.80
0.75
0.70
Precision (Positive label: 1)
0.0
0.2
0.4
0.6
0.8
1.0
Recall (Positive label: 1)
M_Bagging classifier (AP = 0.97)

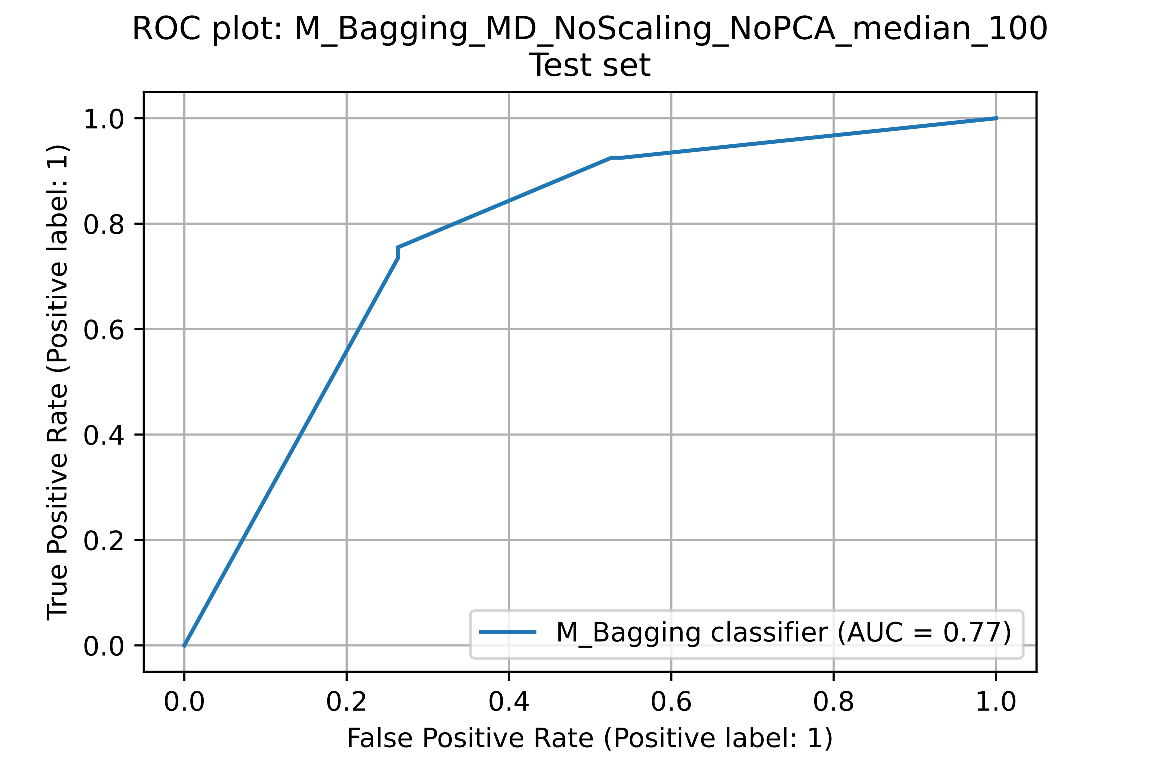
ROC plot: M_Bagging_MD_NoScaling_NoPCA_median_100
Test set
True Positive Rate (Positive label: 1)
False Positive Rate (Positive label: 1)
0.0
0.2
0.4
0.6
0.8
1.0
M_Bagging classifier (AUC = 0.77)

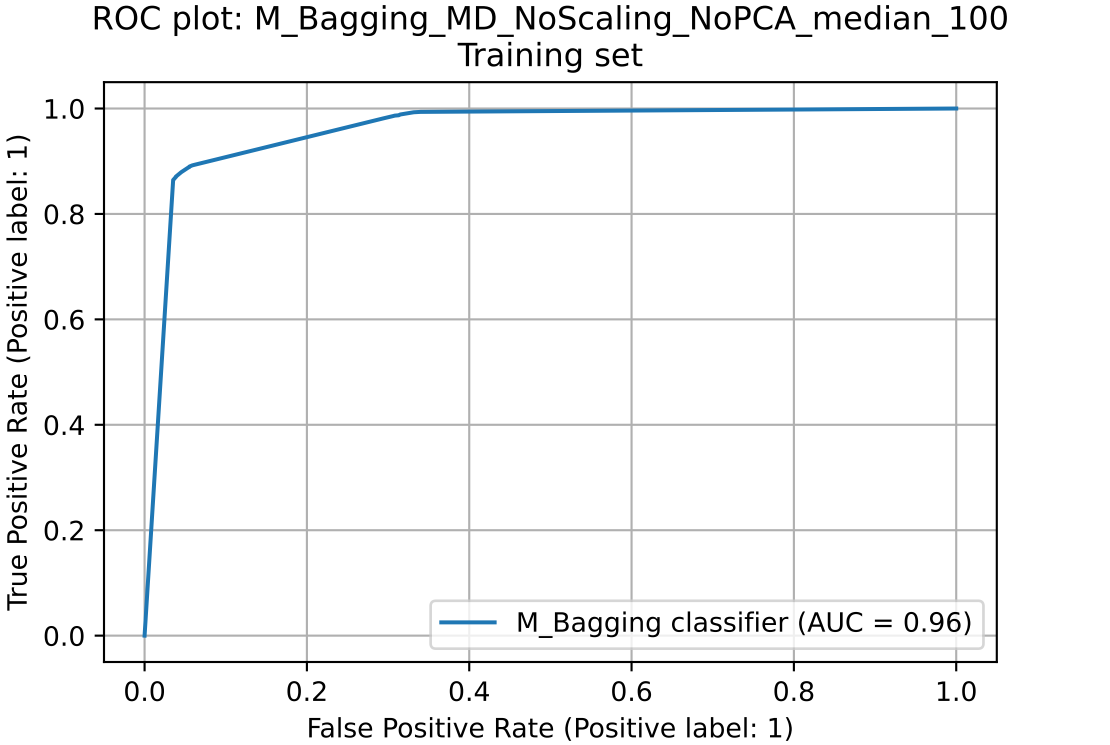
ROC plot: M_Bagging_MD_NoScaling_NoPCA_median_100
Training set
True Positive Rate (Positive label: 1)
False Positive Rate (Positive label: 1)
0.0
0.2
0.4
0.6
0.8
1.0
M_Bagging classifier (AUC = 0.96)

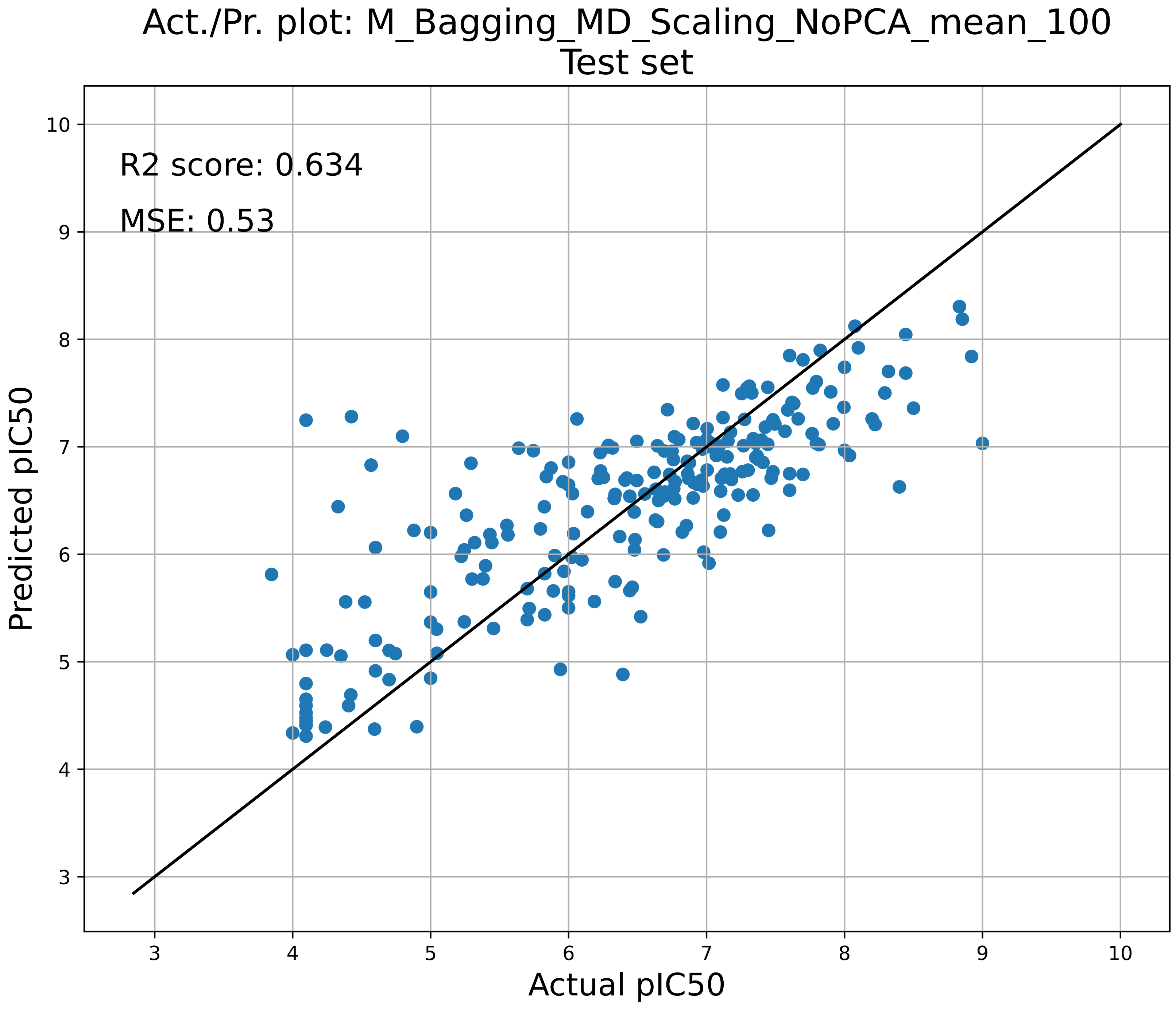

Act./Pr. plot: M_Bagging_MD_Scaling_NoPCA_mean_100
Test set
R2 score: 0.634
MSE: 0.53
Predicted pIC50
Actual pIC50
3
4
5
6
7
8
9
10

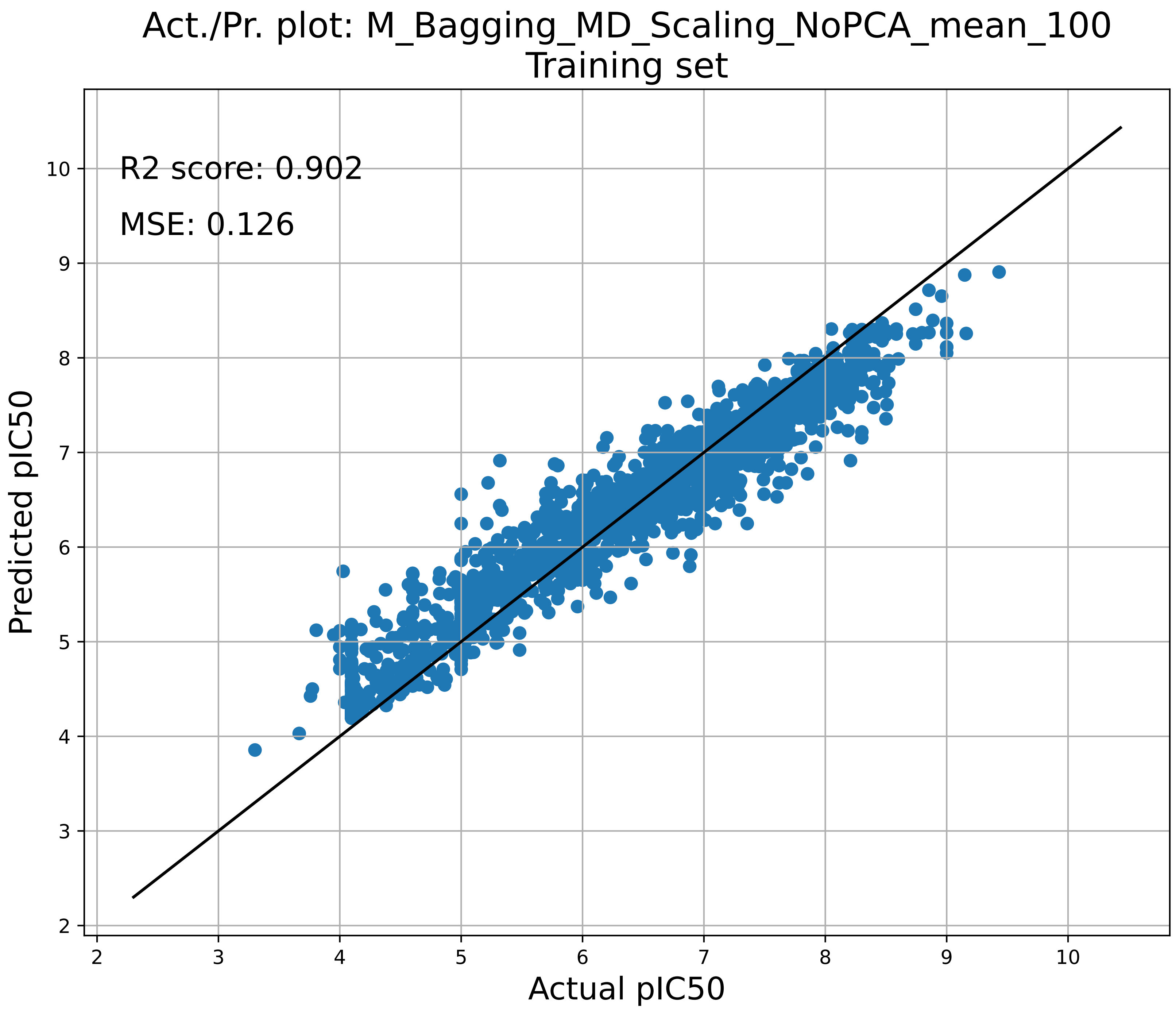
Act./Pr. plot: M_Bagging_MD_Scaling_NoPCA_mean_100
Training set
R2 score: 0.902
MSE: 0.126
Predicted pIC50
Actual pIC50
2
3
4
5
6
7
8
9
10

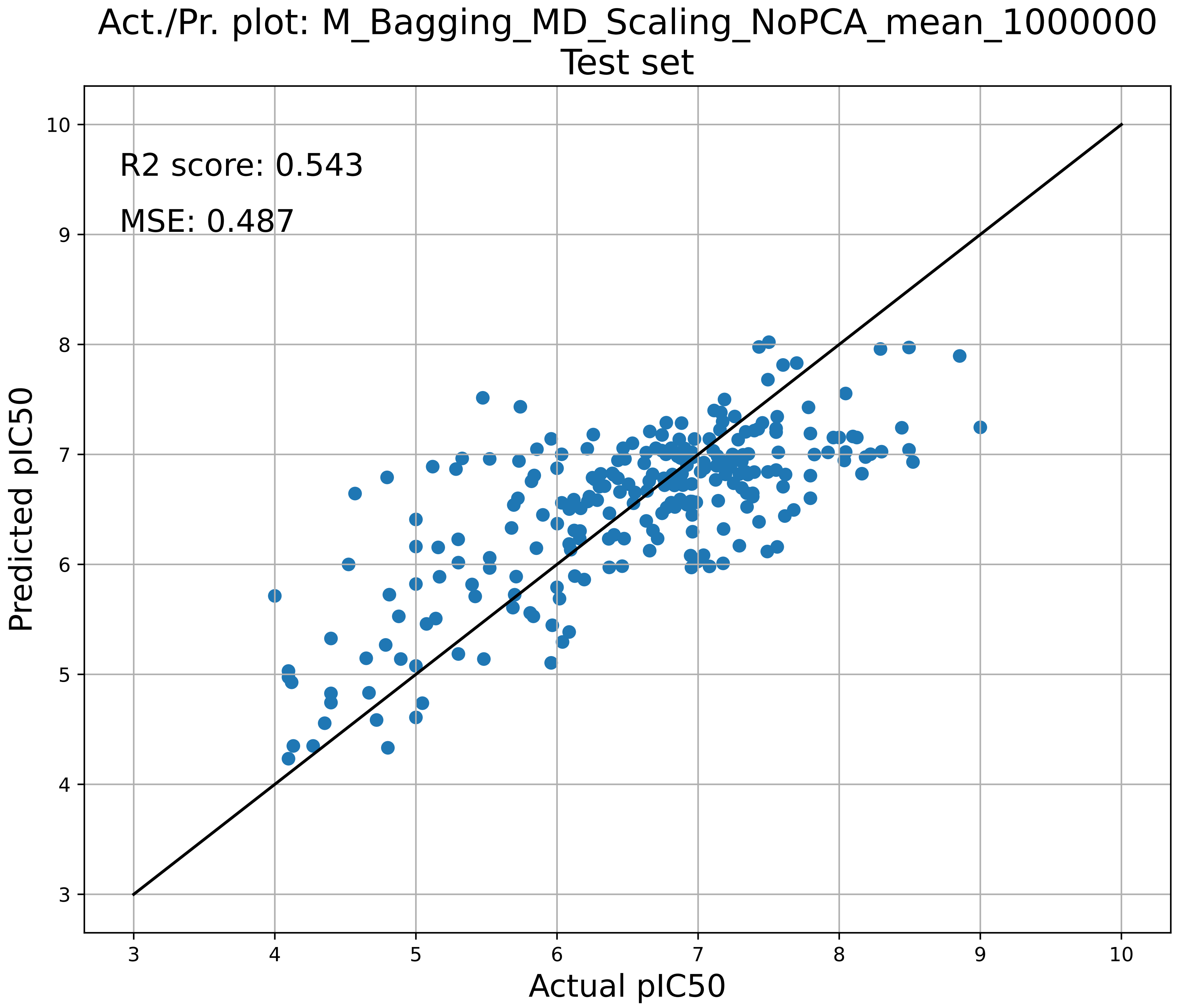
Act./Pr. plot: M_Bagging_MD_Scaling_NoPCA_mean_1000000
Test set
R2 score: 0.543
MSE: 0.487
Predicted pIC50
Actual pIC50
3
4
5
6
7
8
9
10
3
4
5
6
7
8
9
10

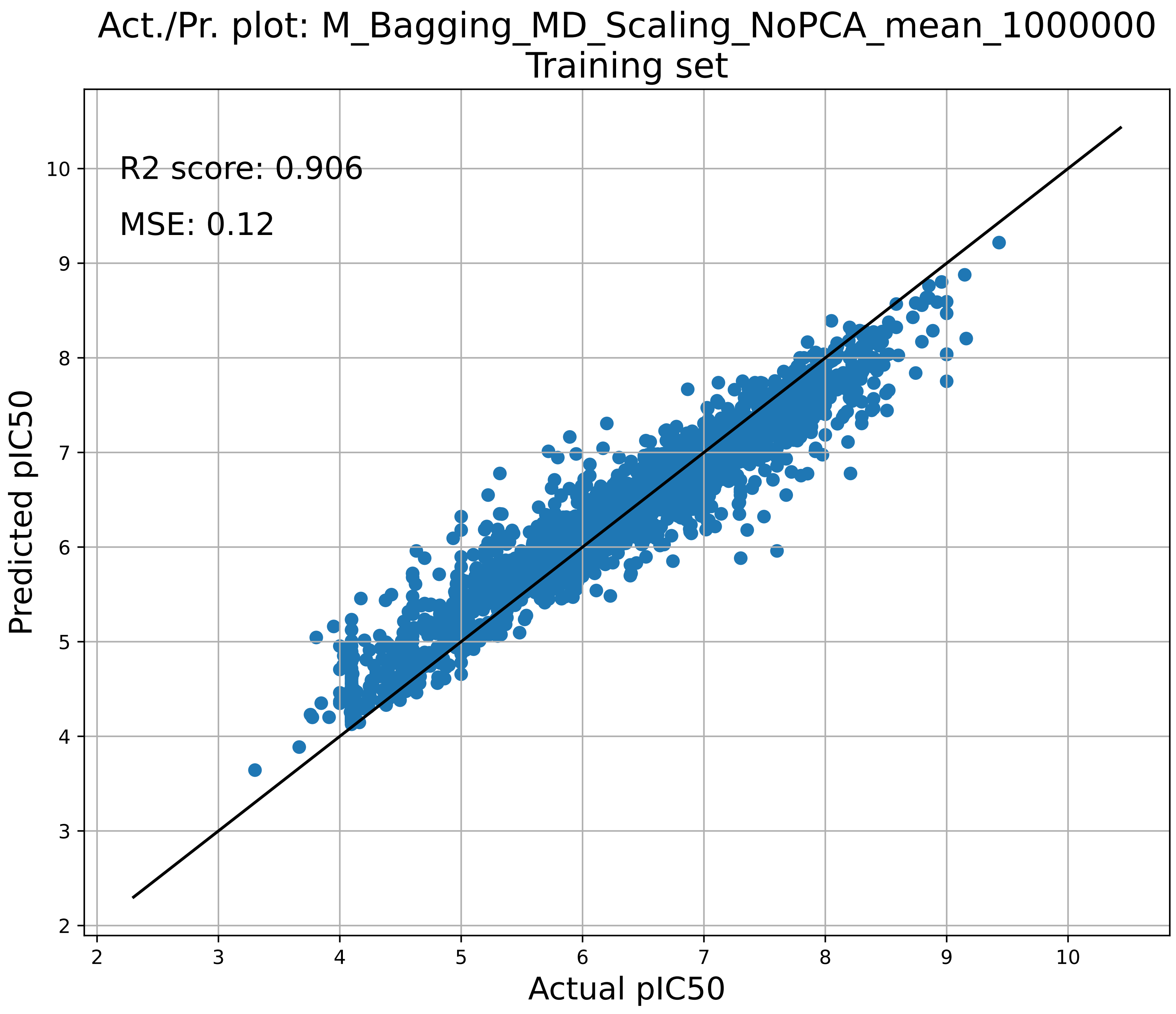

Act./Pr. plot: M_Bagging_MD_Scaling_NoPCA_mean_1000000
Training set
R2 score: 0.906
MSE: 0.12
Predicted pIC50
Actual pIC50
2
3
4
5
6
7
8
9
10

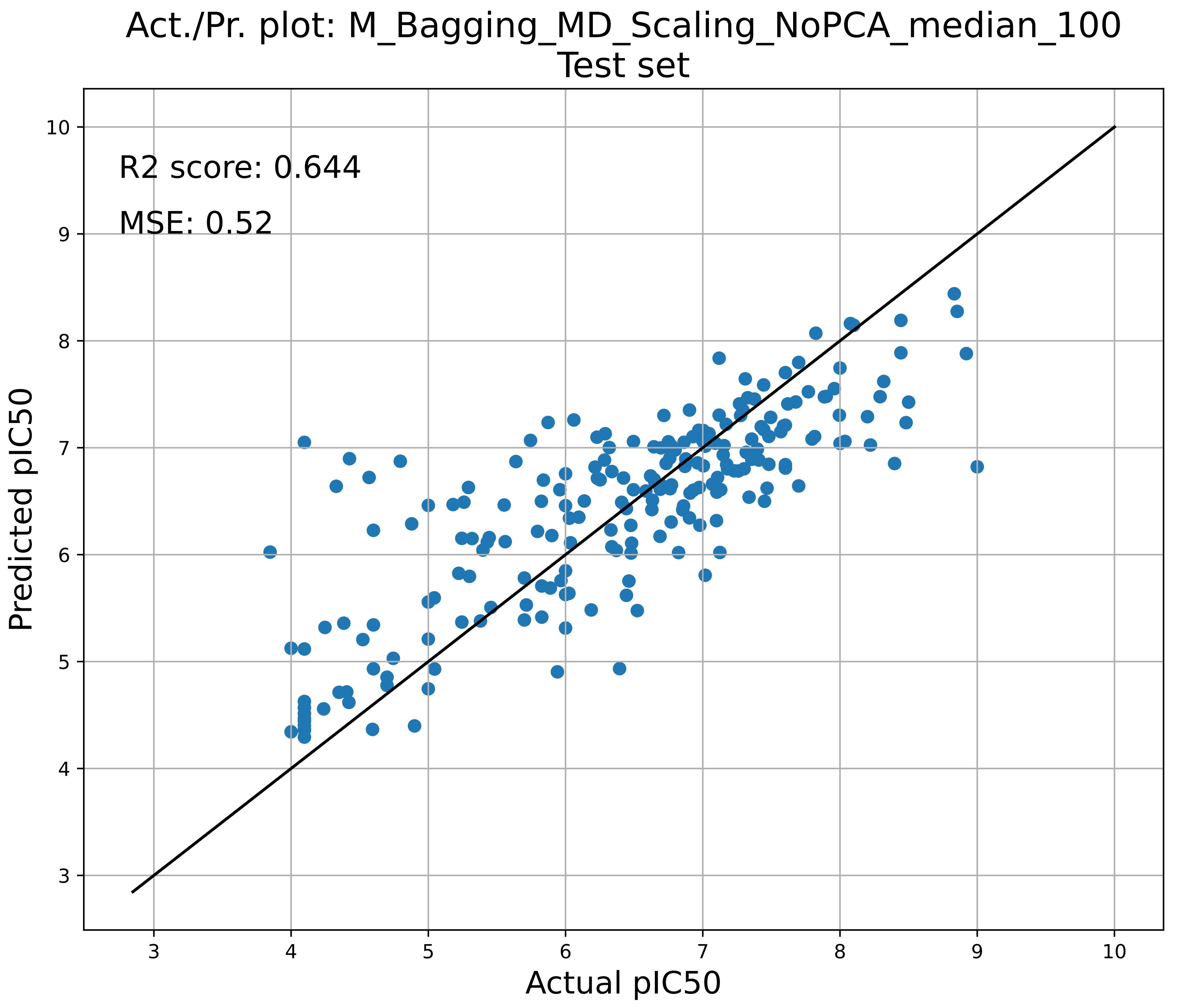

Act./Pr. plot: M_Bagging_MD_Scaling_NoPCA_median_100
Test set
R2 score: 0.644
MSE: 0.52
Predicted pIC50
Actual pIC50
3
4
5
6
7
8
9
10

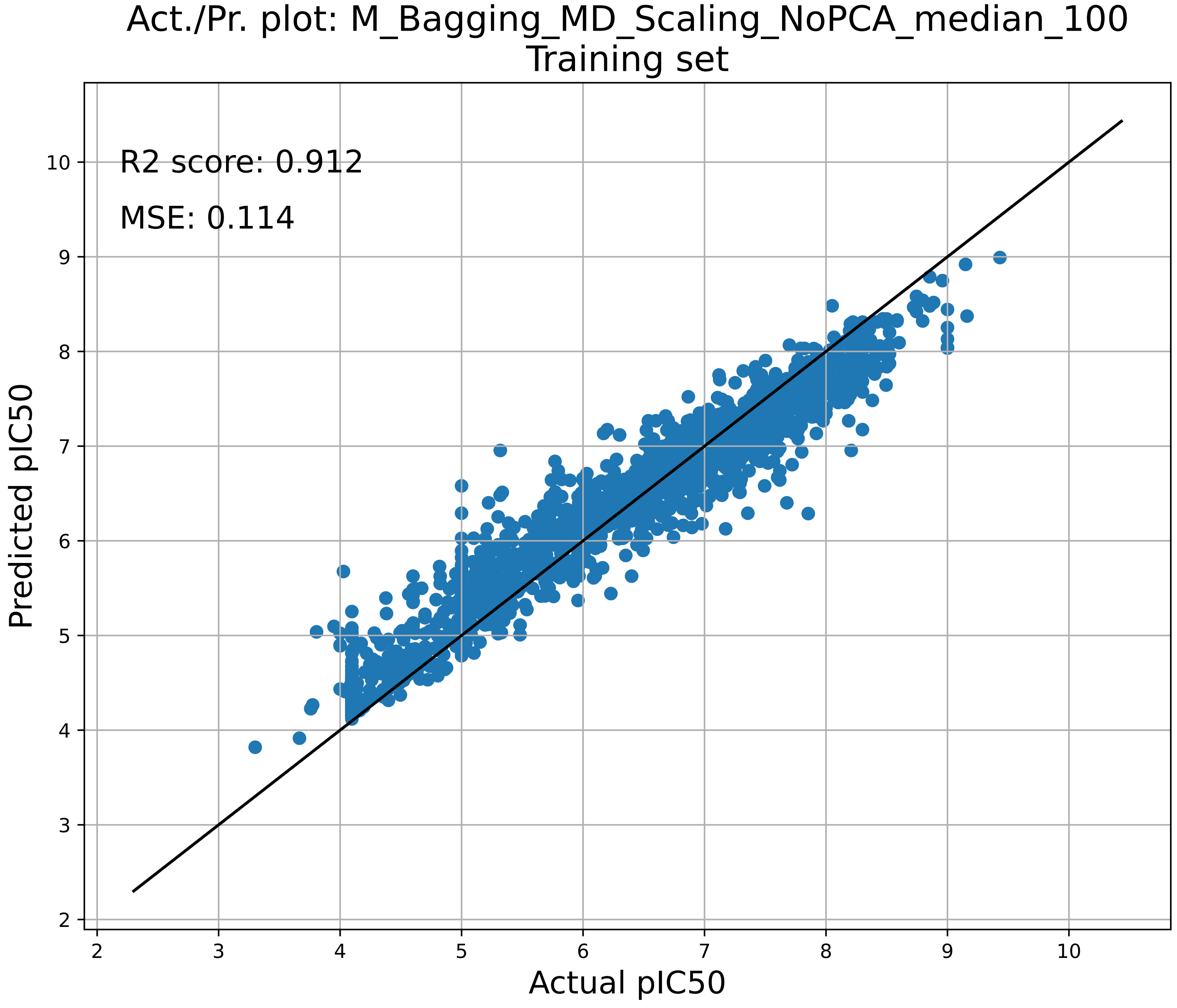
Act./Pr. plot: M_Bagging_MD_Scaling_NoPCA_median_100
Training set
R2 score: 0.912
MSE: 0.114
Predicted pIC50
Actual pIC50
2
3
4
5
6
7
8
9
10

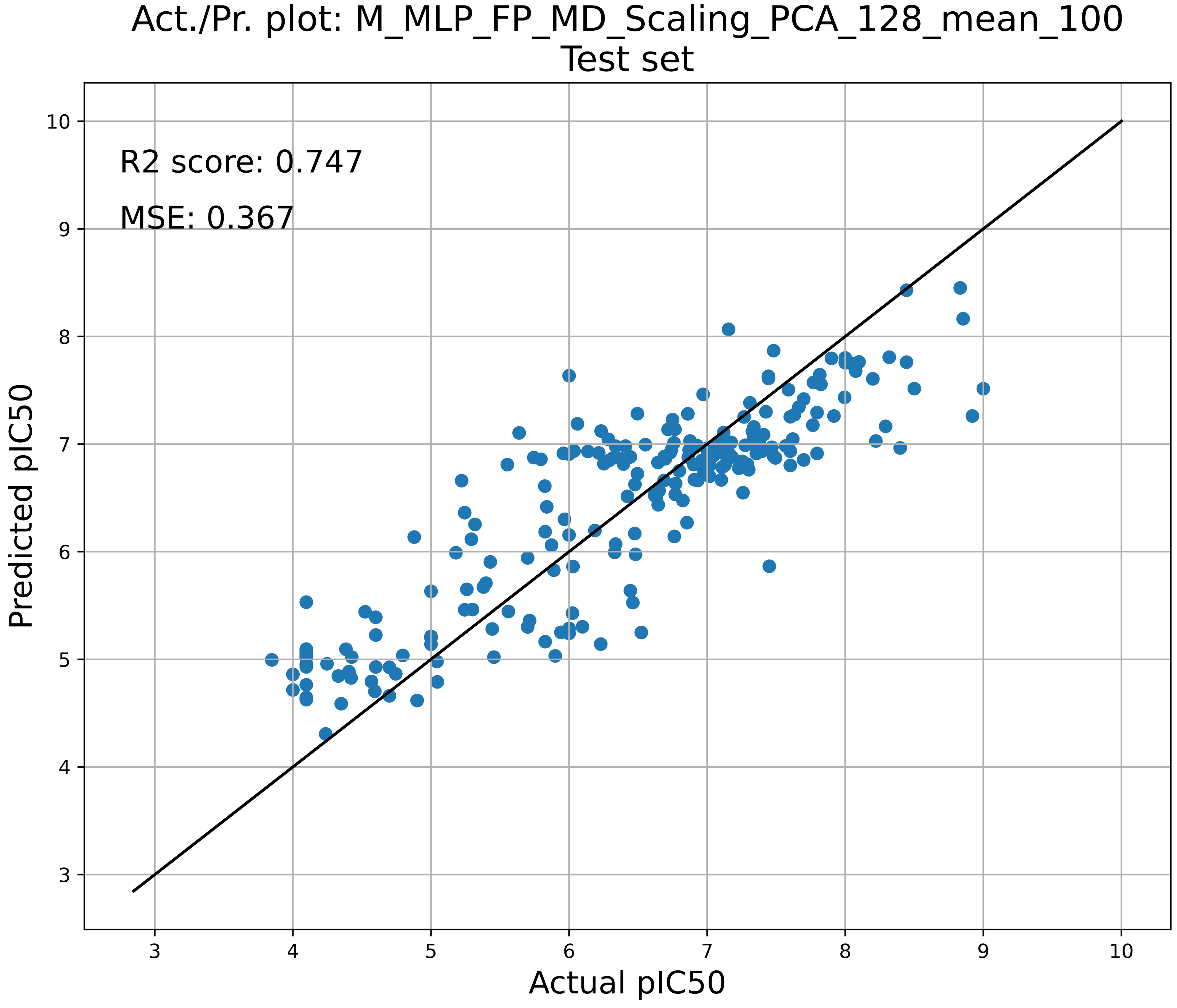
Act./Pr. plot: M_MLP_FP_MD_Scaling_PCA_128_mean_100
Test set
R2 score: 0.747
MSE: 0.367
Predicted pIC50
Actual pIC50
3
4
5
6
7
8
9
10

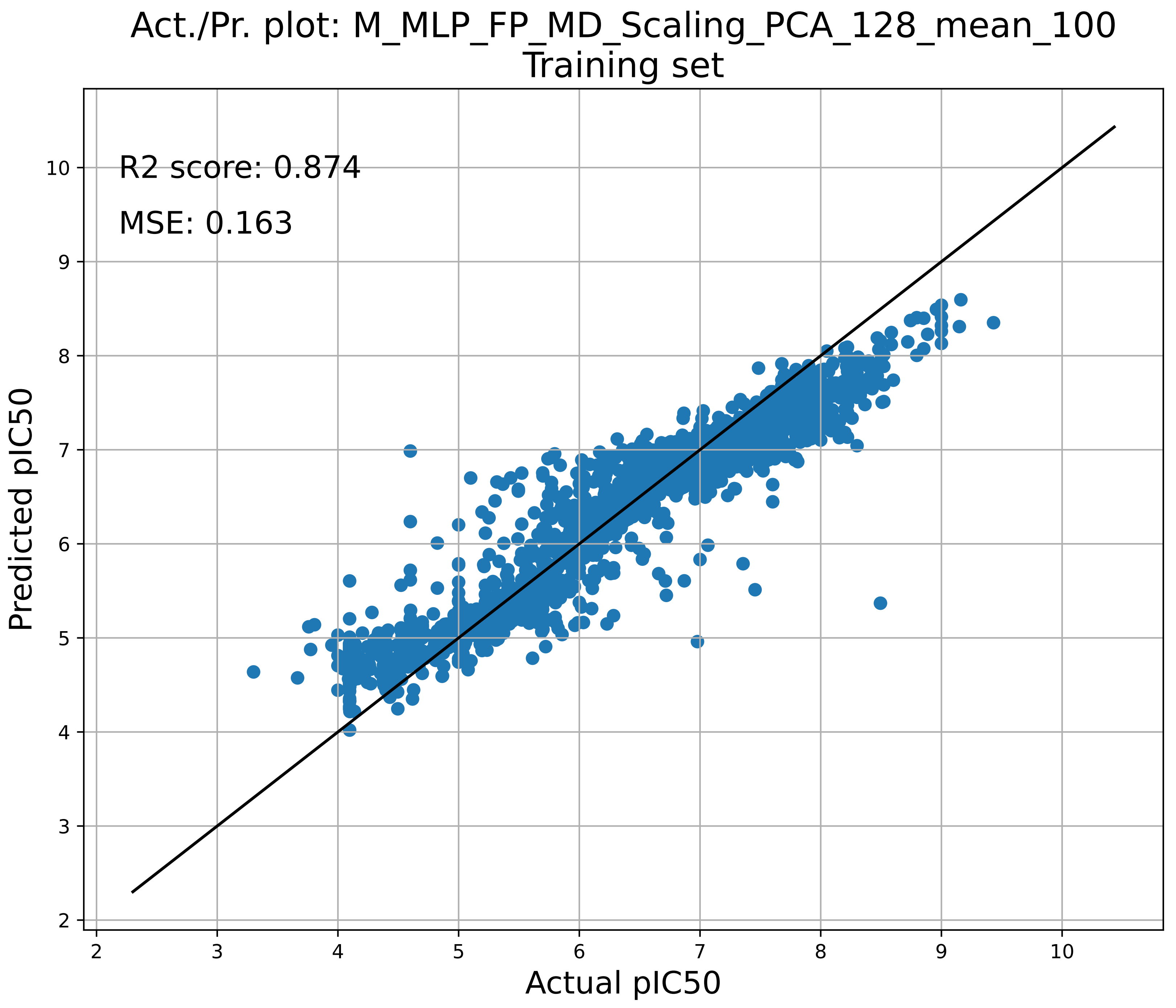

Act./Pr. plot: M_MLP_FP_MD_Scaling_PCA_128_mean_100
Training set
R2 score: 0.874
MSE: 0.163
Predicted pIC50
Actual pIC50
2
3
4
5
6
7
8
9
10

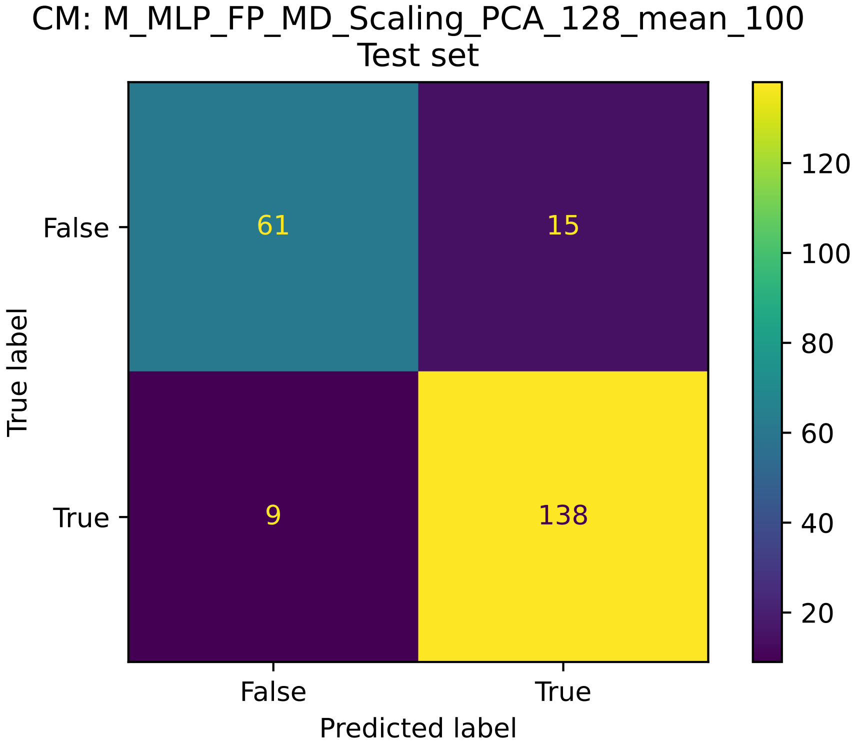
CM: M_MLP_FP_MD_Scaling_PCA_128_mean_100
Test set
True label
False
True
61
15
9
138
False
True
Predicted label
120
100
80
60
40
20

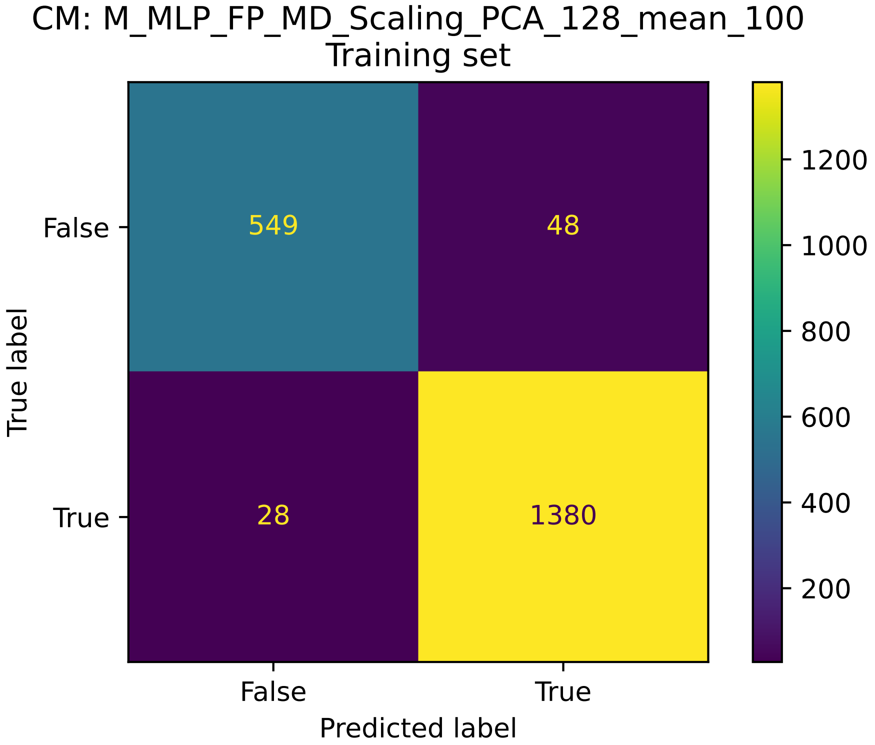
CM: M_MLP_FP_MD_Scaling_PCA_128_mean_100
Training set
True label
Predicted label
False
True
False
True
549
48
28
1380
1200
1000
800
600
400
200

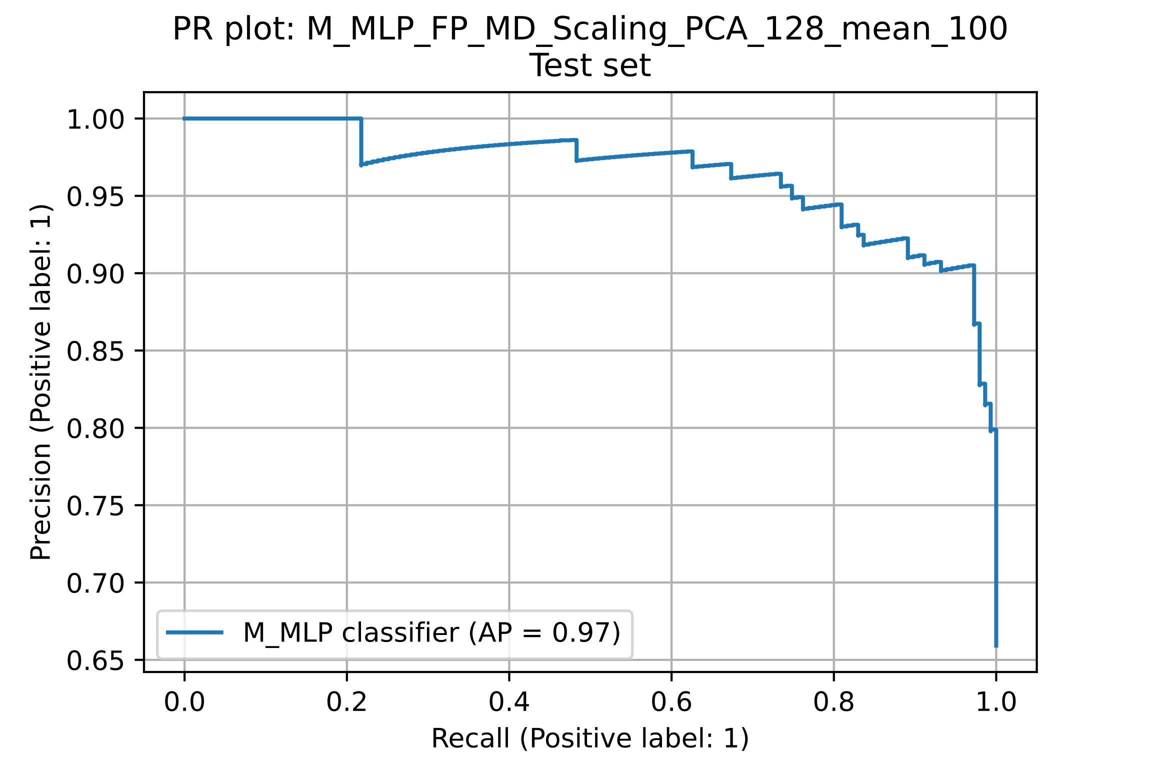

PR plot: M_MLP_FP_MD_Scaling_PCA_128_mean_100
Test set
Precision (Positive label: 1)
Recall (Positive label: 1)
1.00
0.95
0.90
0.85
0.80
0.75
0.70
0.65
0.0
0.2
0.4
0.6
0.8
1.0
M_MLP classifier (AP = 0.97)

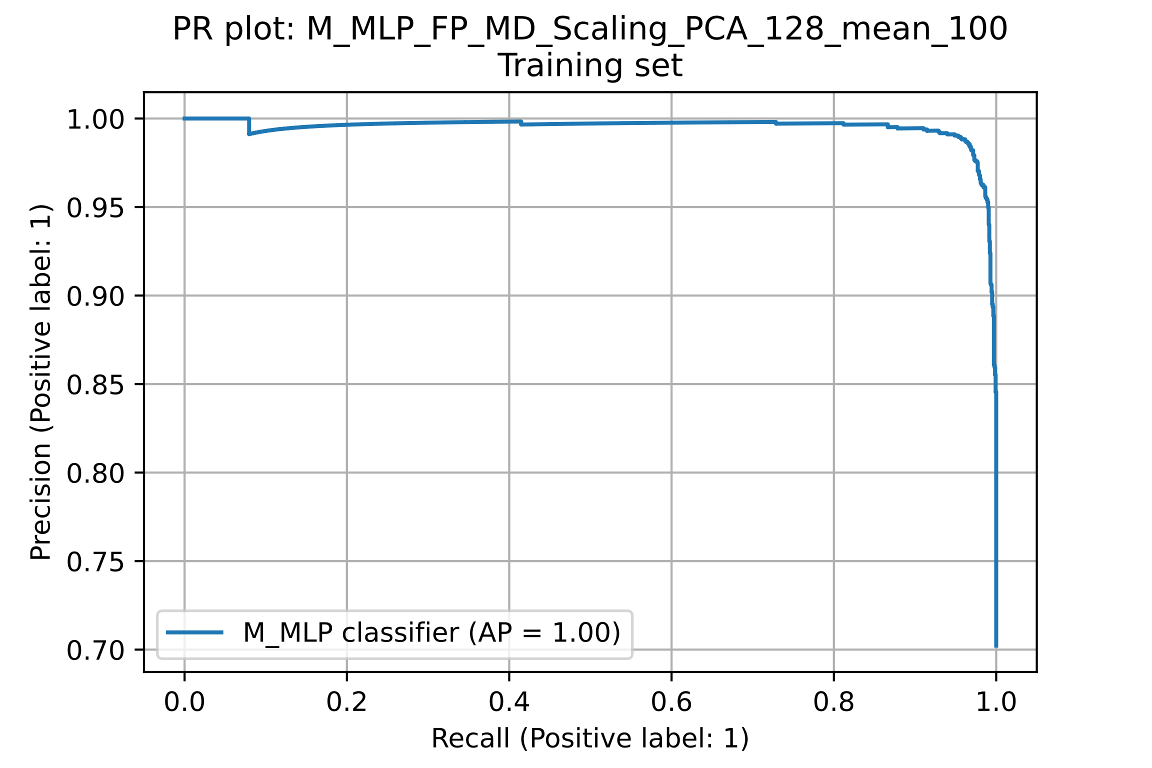
PR plot: M_MLP_FP_MD_Scaling_PCA_128_mean_100
Training set
Precision (Positive label: 1)
Recall (Positive label: 1)
1.00
0.95
0.90
0.85
0.80
0.75
0.70
0.0
0.2
0.4
0.6
0.8
1.0
M_MLP classifier (AP = 1.00)

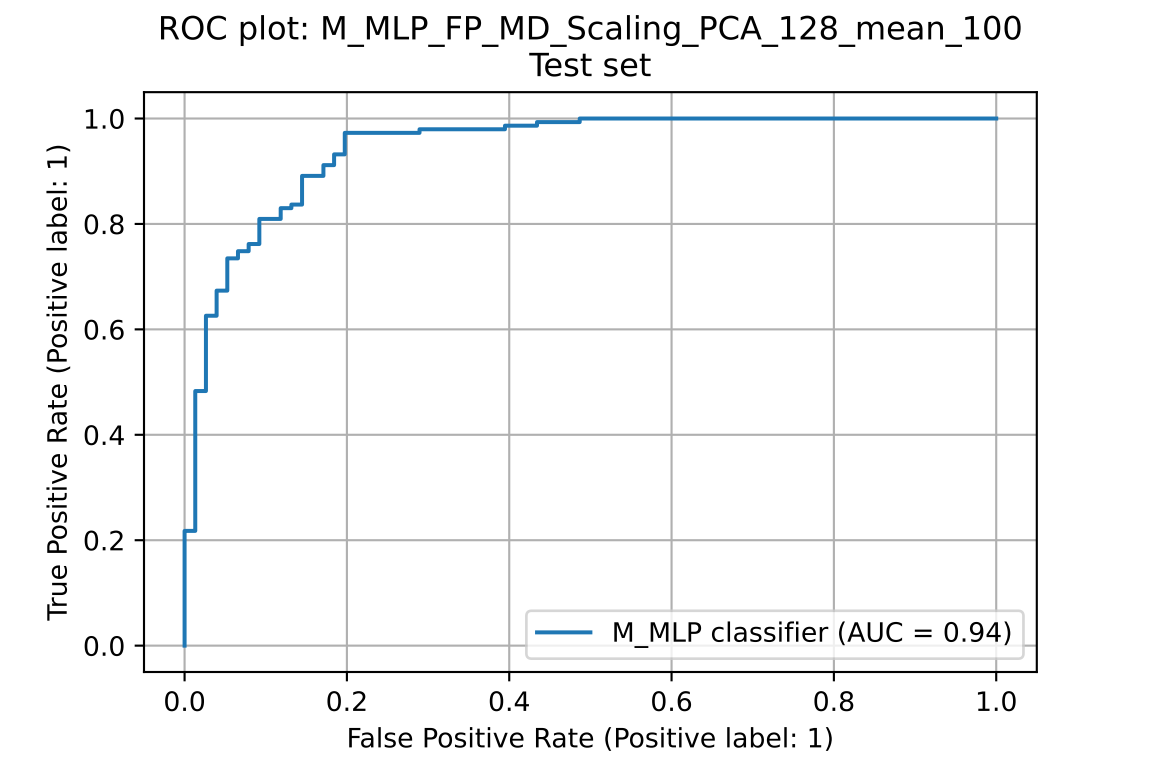
ROC plot: M_MLP_FP_MD_Scaling_PCA_128_mean_100
Test set
True Positive Rate (Positive label: 1)
False Positive Rate (Positive label: 1)
0.0
0.2
0.4
0.6
0.8
1.0
M_MLP classifier (AUC = 0.94)

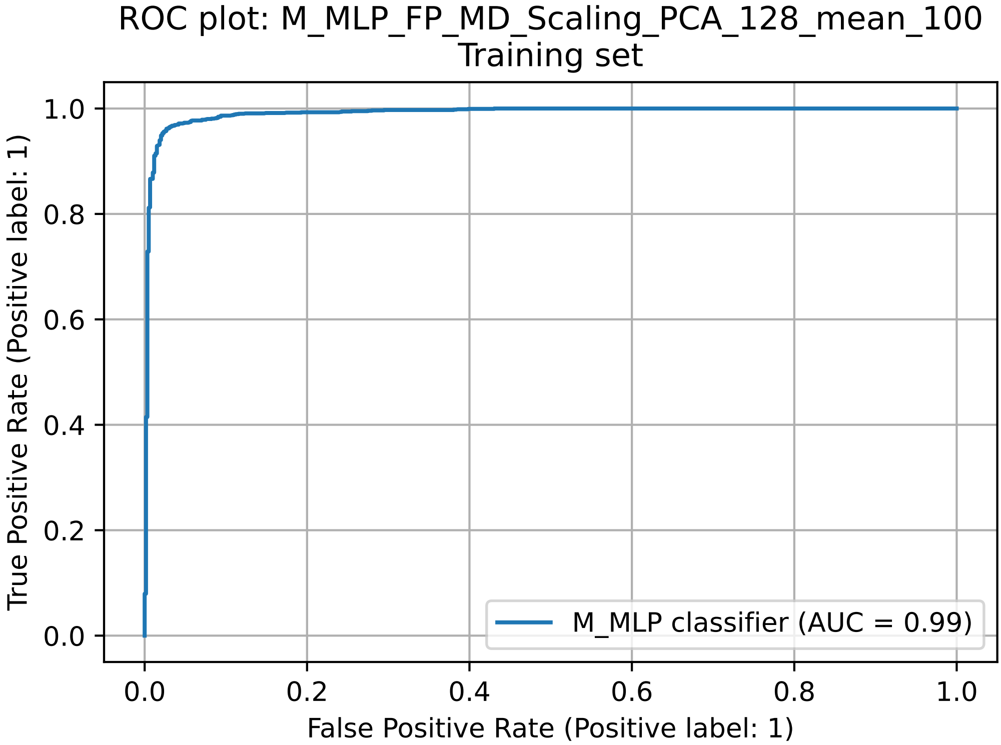
ROC plot: M_MLP_FP_MD_Scaling_PCA_128_mean_100
Training set
True Positive Rate (Positive label: 1)
False Positive Rate (Positive label: 1)
0.0
0.2
0.4
0.6
0.8
1.0
M_MLP classifier (AUC = 0.99)

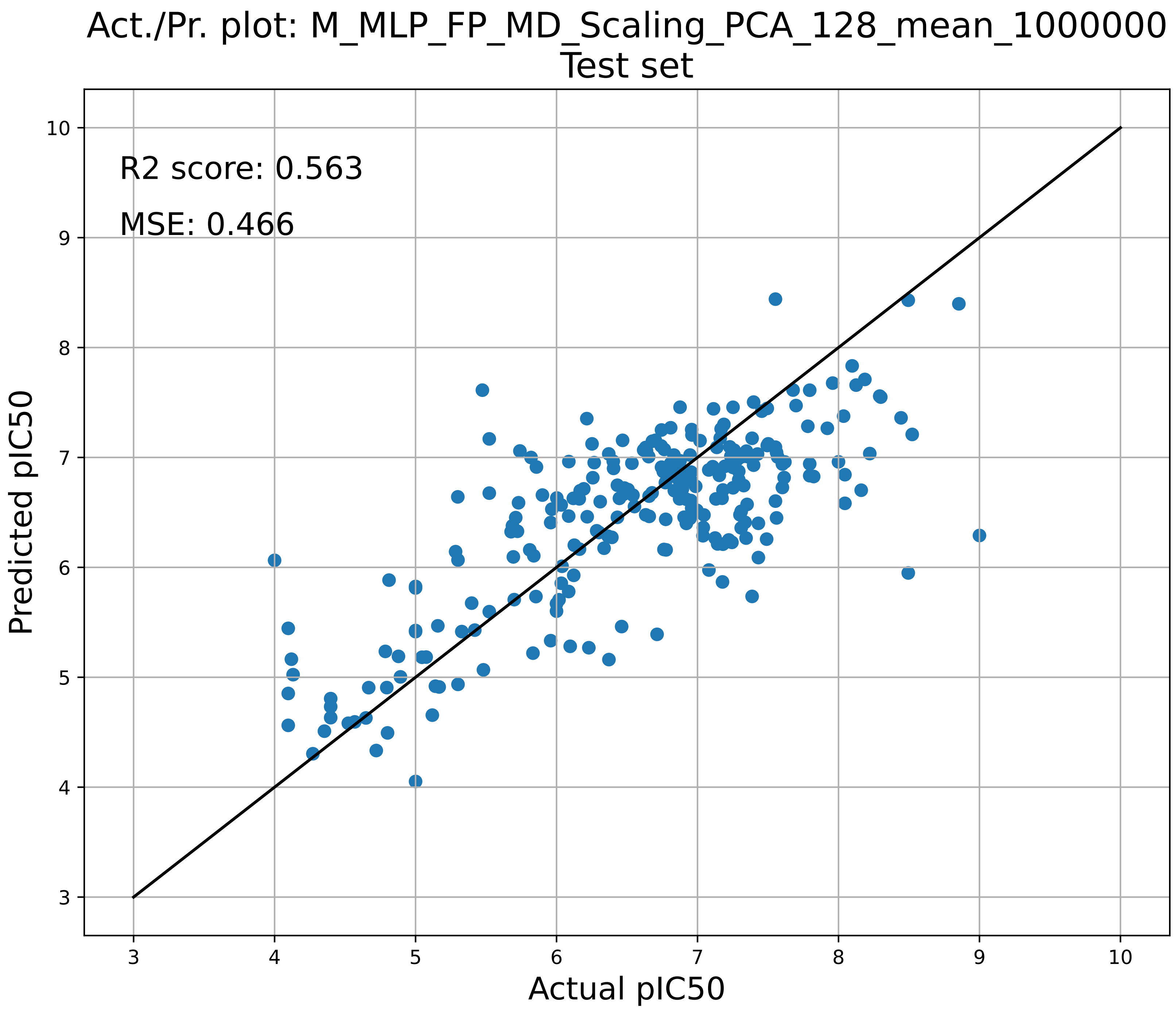
Act./Pr. plot: M_MLP_FP_MD_Scaling_PCA_128_mean_1000000
Test set
R2 score: 0.563
MSE: 0.466
Predicted pIC50
Actual pIC50
3
4
5
6
7
8
9
10

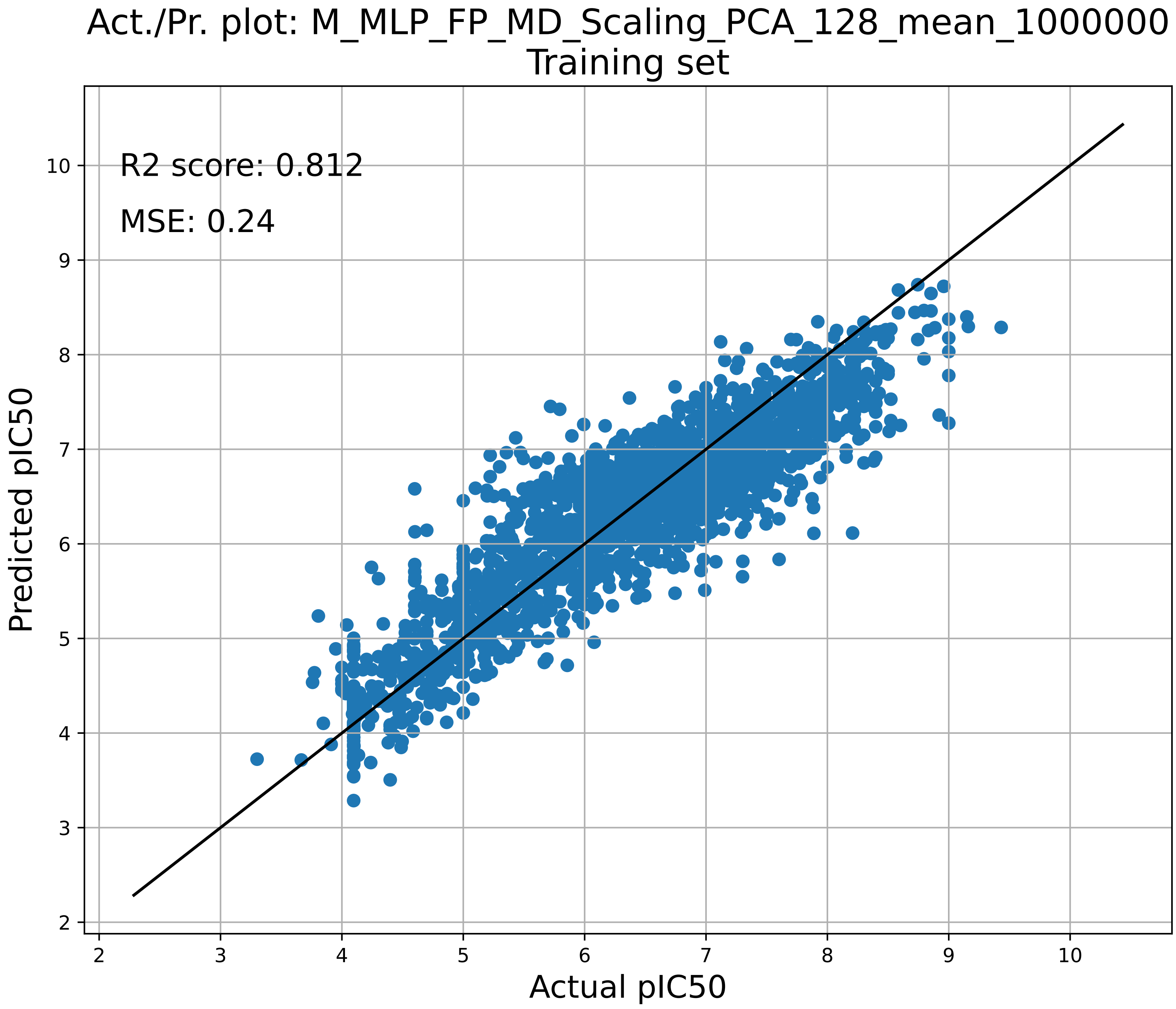
Act./Pr. plot: M_MLP_FP_MD_Scaling_PCA_128_mean_1000000
Training set
R2 score: 0.812
MSE: 0.24
Predicted pIC50
Actual pIC50
2
3
4
5
6
7
8
9
10

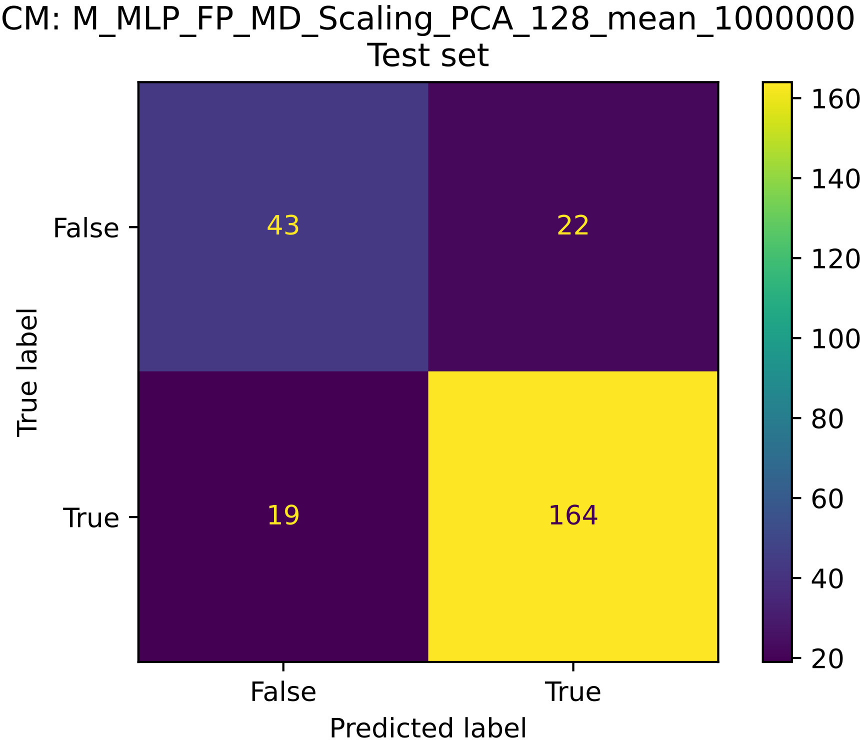
CM: M_MLP_FP_MD_Scaling_PCA_128_mean_1000000
Test set
True label
False
True
43
22
19
164
False
True
Predicted label
160
140
120
100
80
60
40
20

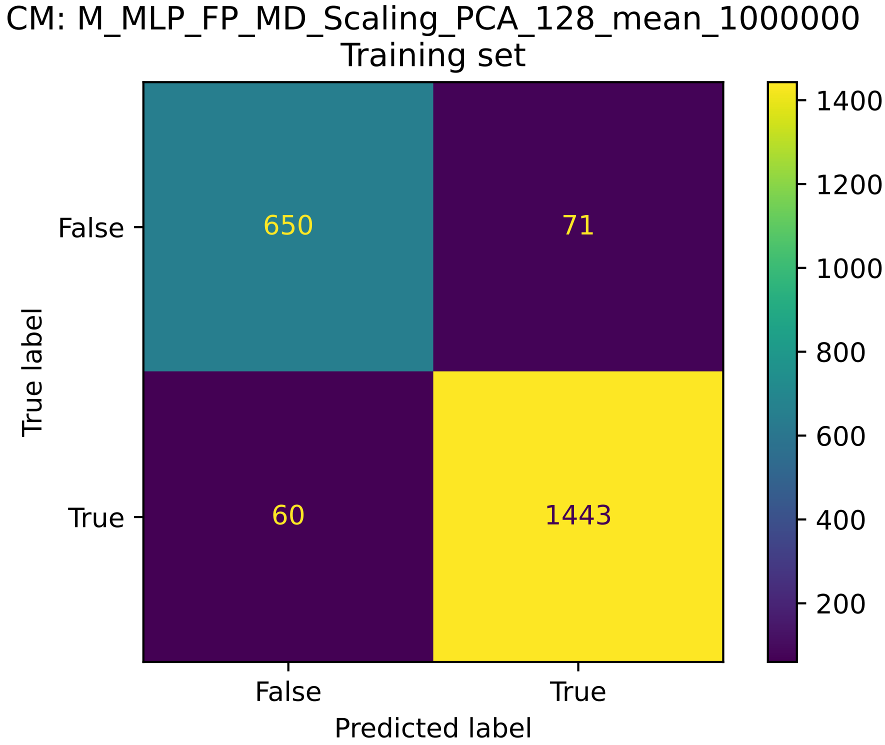
CM: M_MLP_FP_MD_Scaling_PCA_128_mean_1000000
Training set
650
71
60
1443
False
True
False
True
True label
Predicted label
1400
1200
1000
800
600
400
200

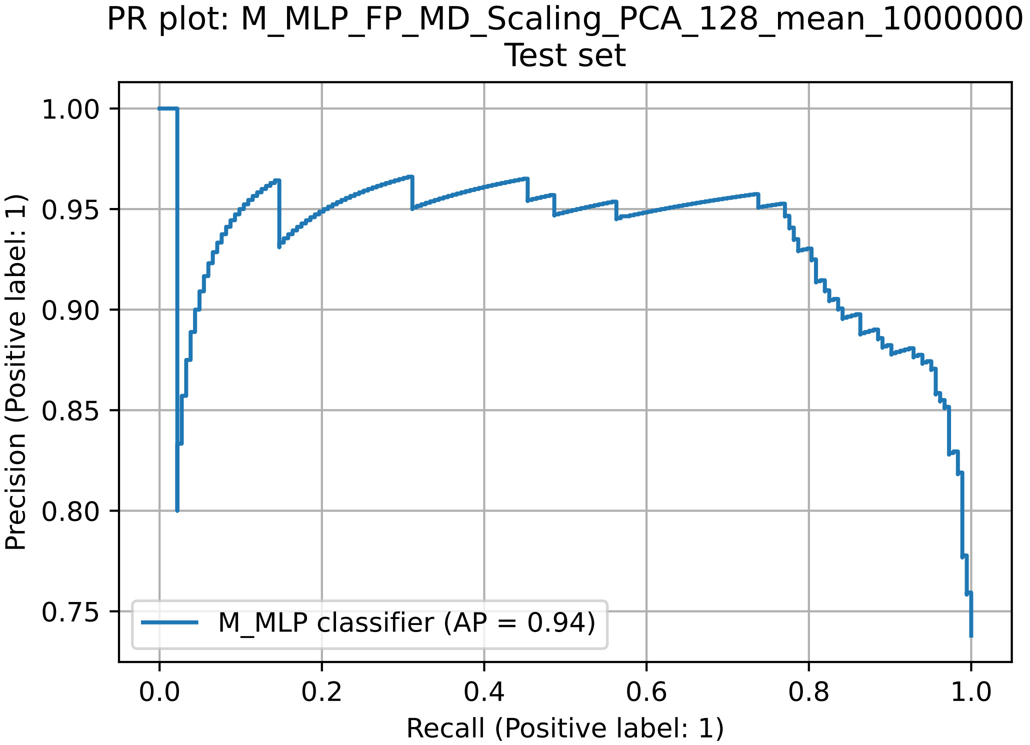

PR plot: M_MLP_FP_MD_Scaling_PCA_128_mean_1000000
Test set
Precision (Positive label: 1)
Recall (Positive label: 1)
1.00
0.95
0.90
0.85
0.80
0.75
0.0
0.2
0.4
0.6
0.8
1.0
M_MLP classifier (AP = 0.94)

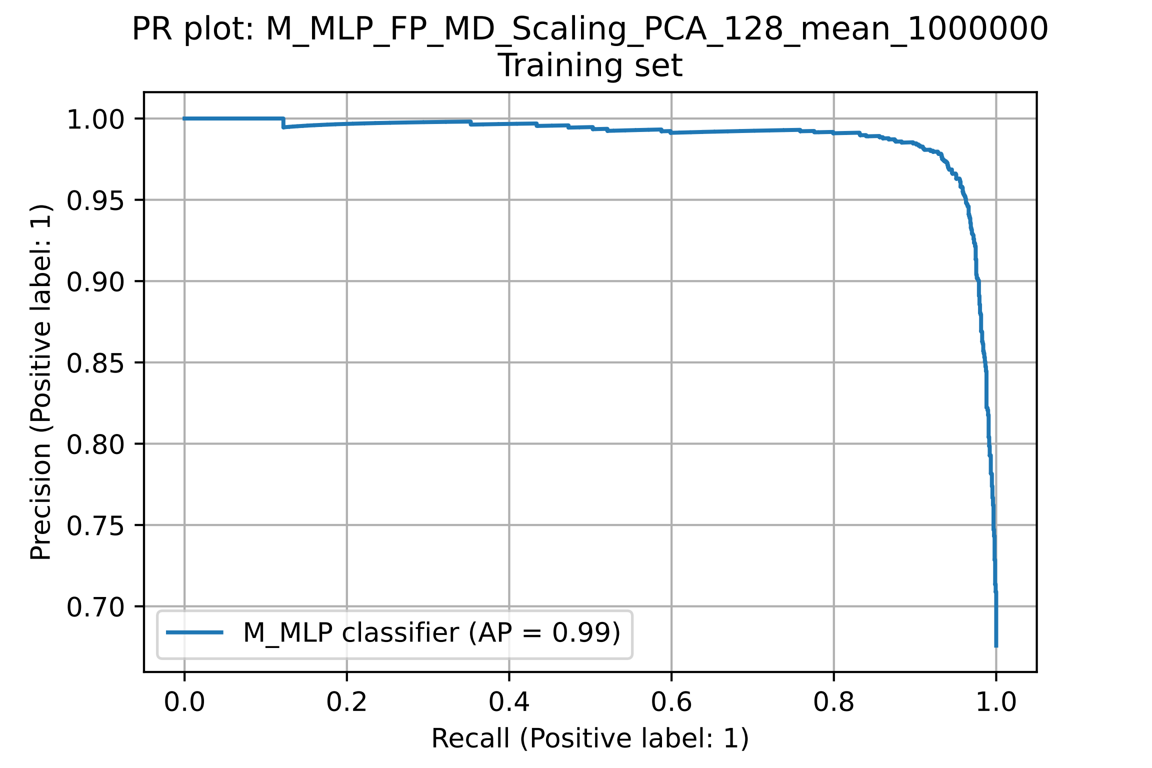

PR plot: M_MLP_FP_MD_Scaling_PCA_128_mean_1000000
Training set
Precision (Positive label: 1)
Recall (Positive label: 1)
M_MLP classifier (AP = 0.99)
1.00
0.95
0.90
0.85
0.80
0.75
0.70
0.0
0.2
0.4
0.6
0.8
1.0

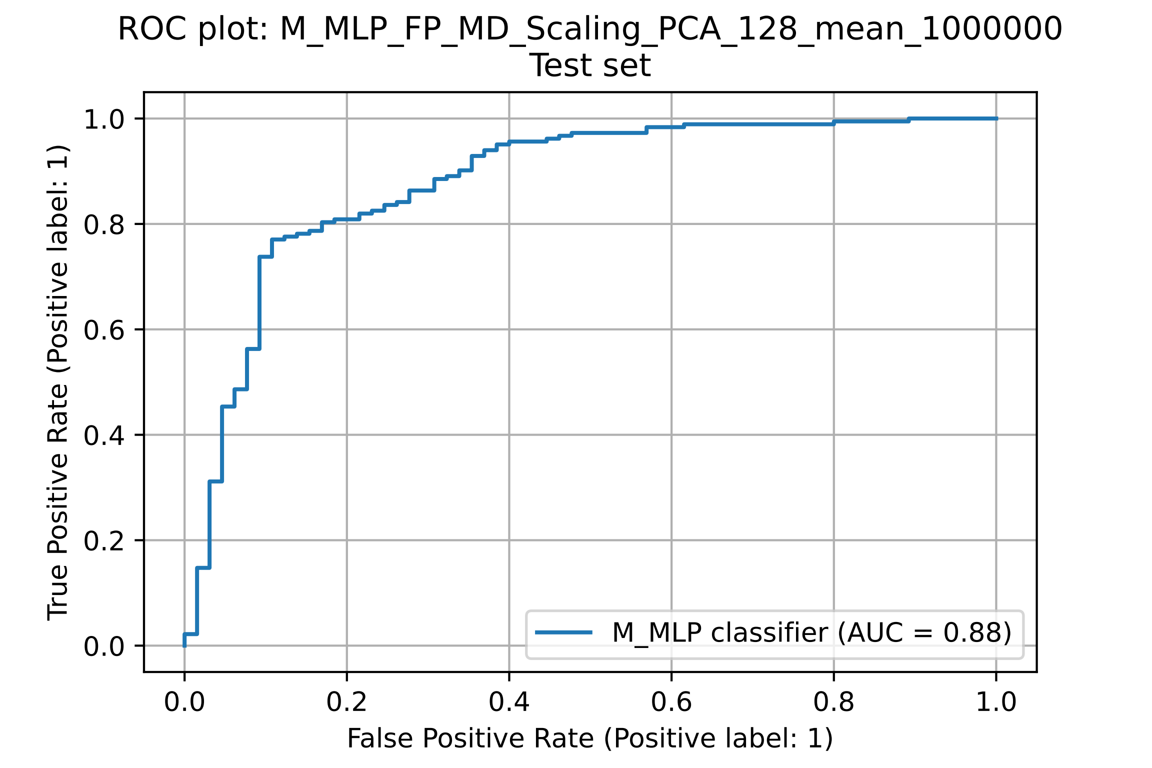

ROC plot: M_MLP_FP_MD_Scaling_PCA_128_mean_1000000
Test set
True Positive Rate (Positive label: 1)
False Positive Rate (Positive label: 1)
0.0
0.2
0.4
0.6
0.8
1.0
M_MLP classifier (AUC = 0.88)

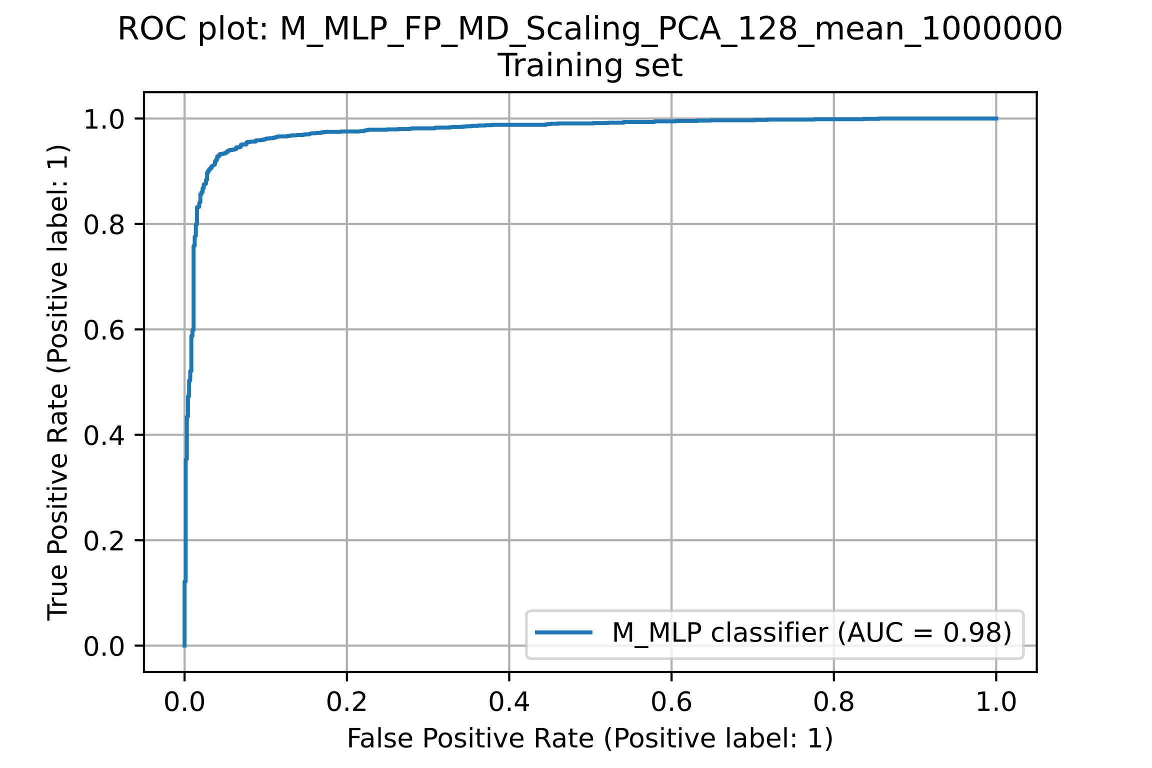
ROC plot: M_MLP_FP_MD_Scaling_PCA_128_mean_1000000
Training set
True Positive Rate (Positive label: 1)
False Positive Rate (Positive label: 1)
0.0
0.2
0.4
0.6
0.8
1.0
M_MLP classifier (AUC = 0.98)

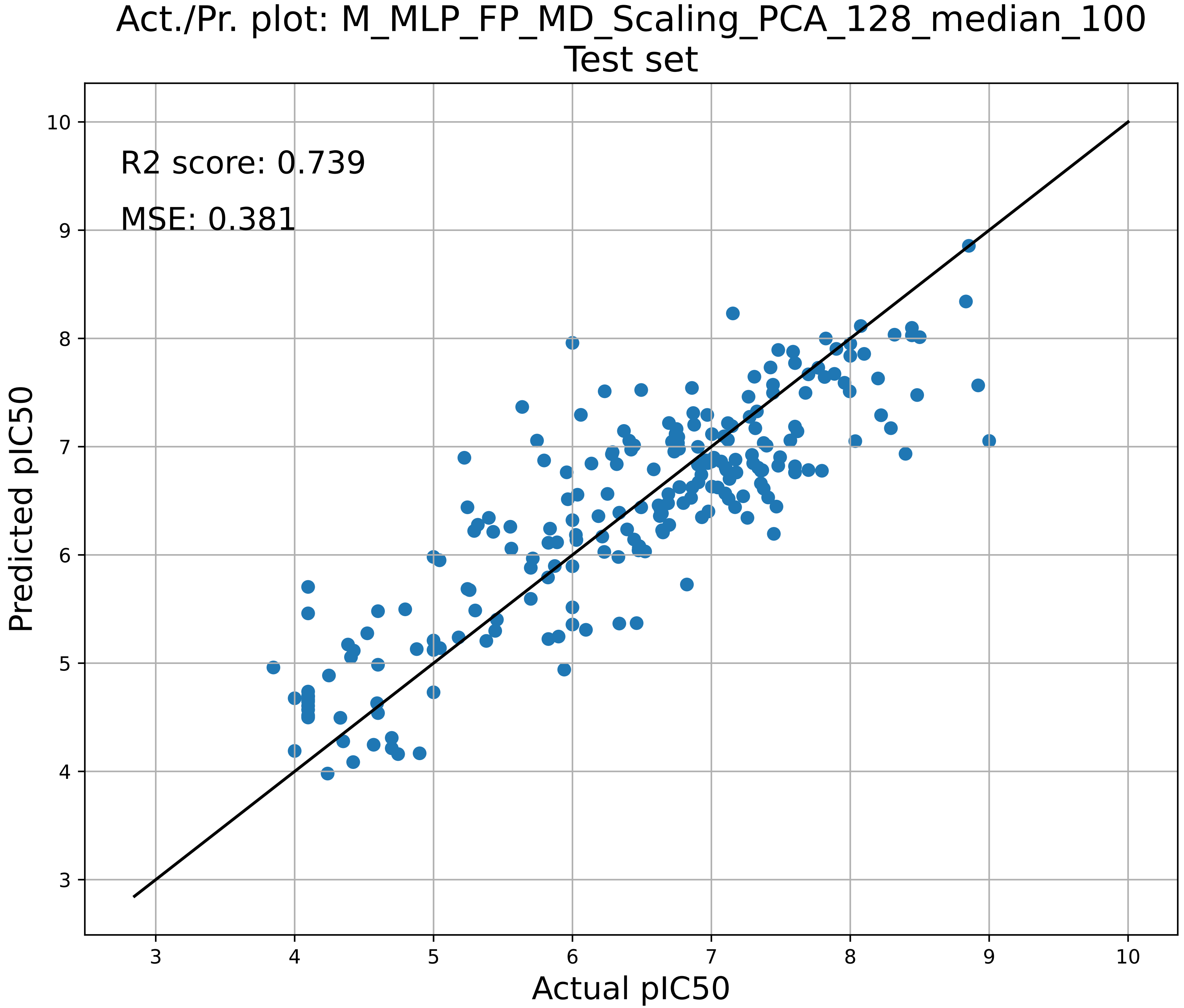

Act./Pr. plot: M_MLP_FP_MD_Scaling_PCA_128_median_100
Test set
R2 score: 0.739
MSE: 0.381
Predicted pIC50
Actual pIC50
10
9
8
7
6
5
4
3
3
4
5
6
7
8
9
10

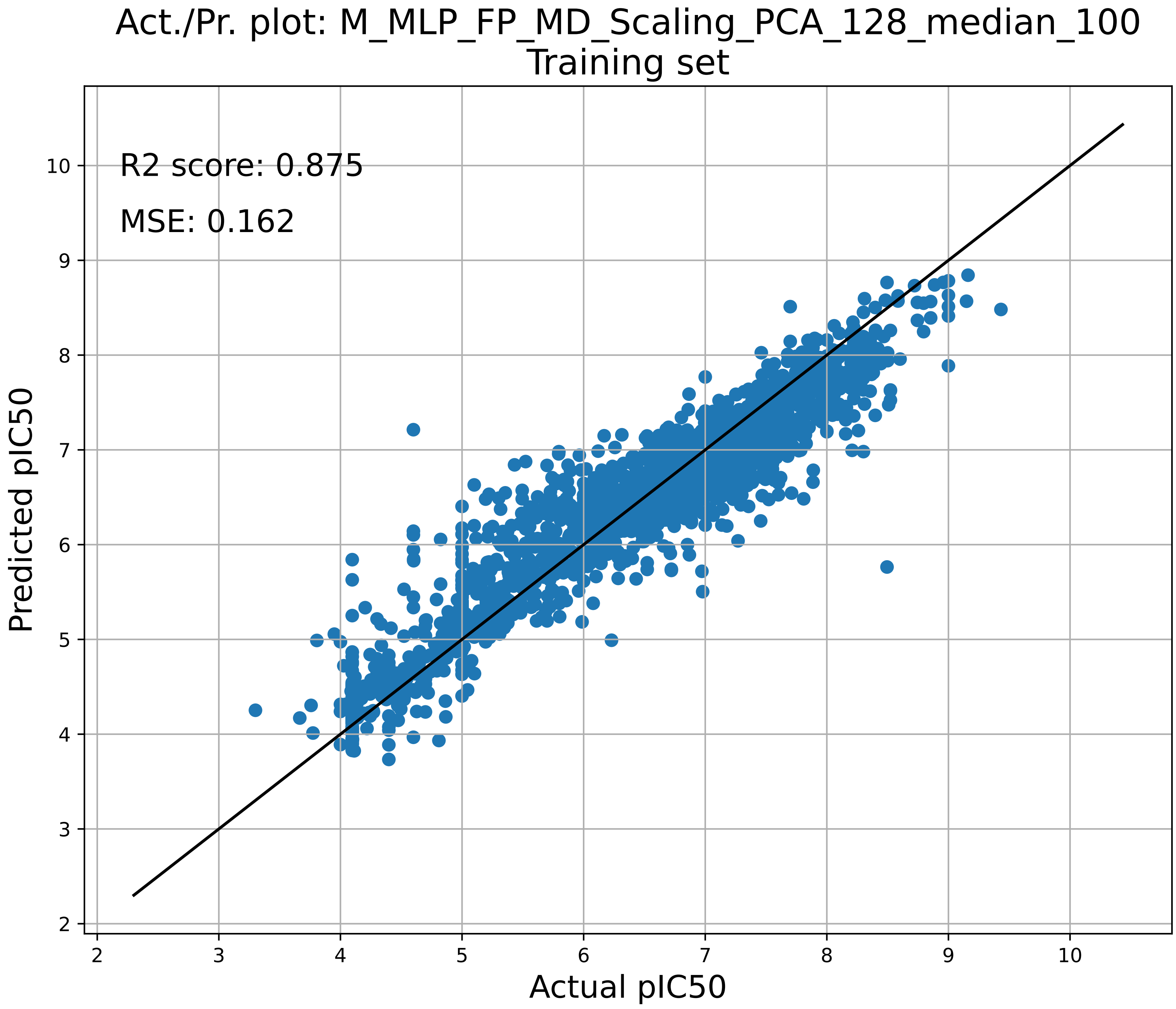

Act./Pr. plot: M_MLP_FP_MD_Scaling_PCA_128_median_100
Training set
R2 score: 0.875
MSE: 0.162
Predicted pIC50
Actual pIC50
2
3
4
5
6
7
8
9
10

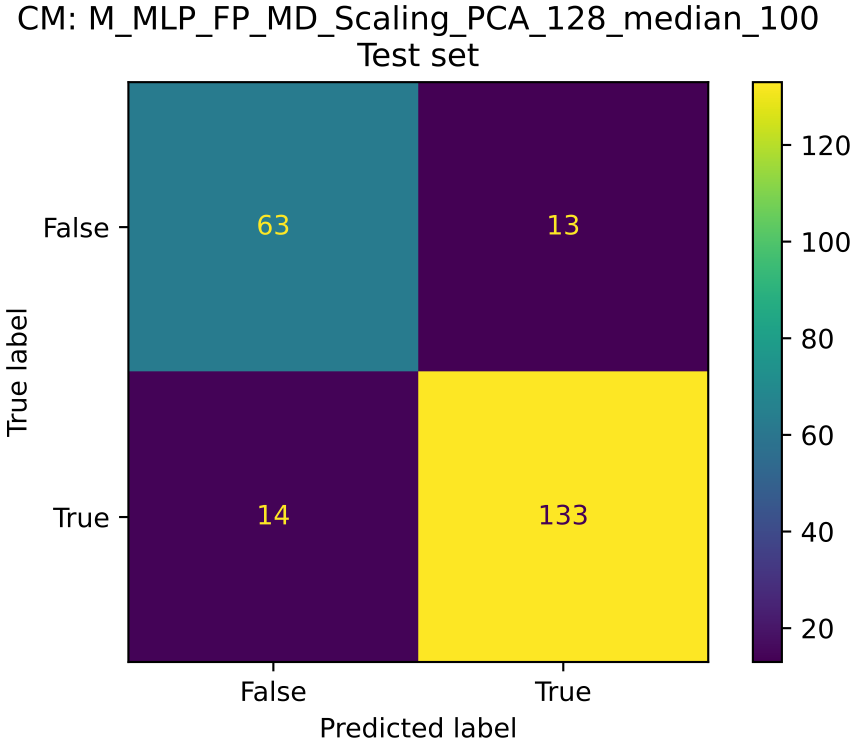
CM: M_MLP_FP_MD_Scaling_PCA_128_median_100
Test set
True label
False
True
63
13
14
133
Predicted label
False
True
120
100
80
60
40
20

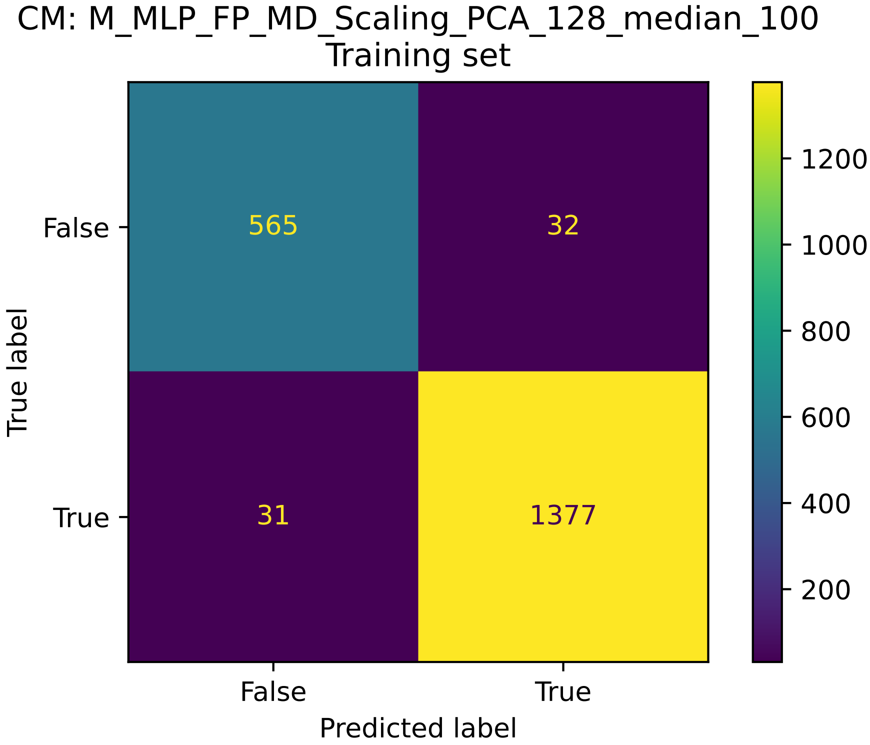
CM: M_MLP_FP_MD_Scaling_PCA_128_median_100
Training set
True label
False
True
565
32
31
1377
Predicted label
False
True
1200
1000
800
600
400
200

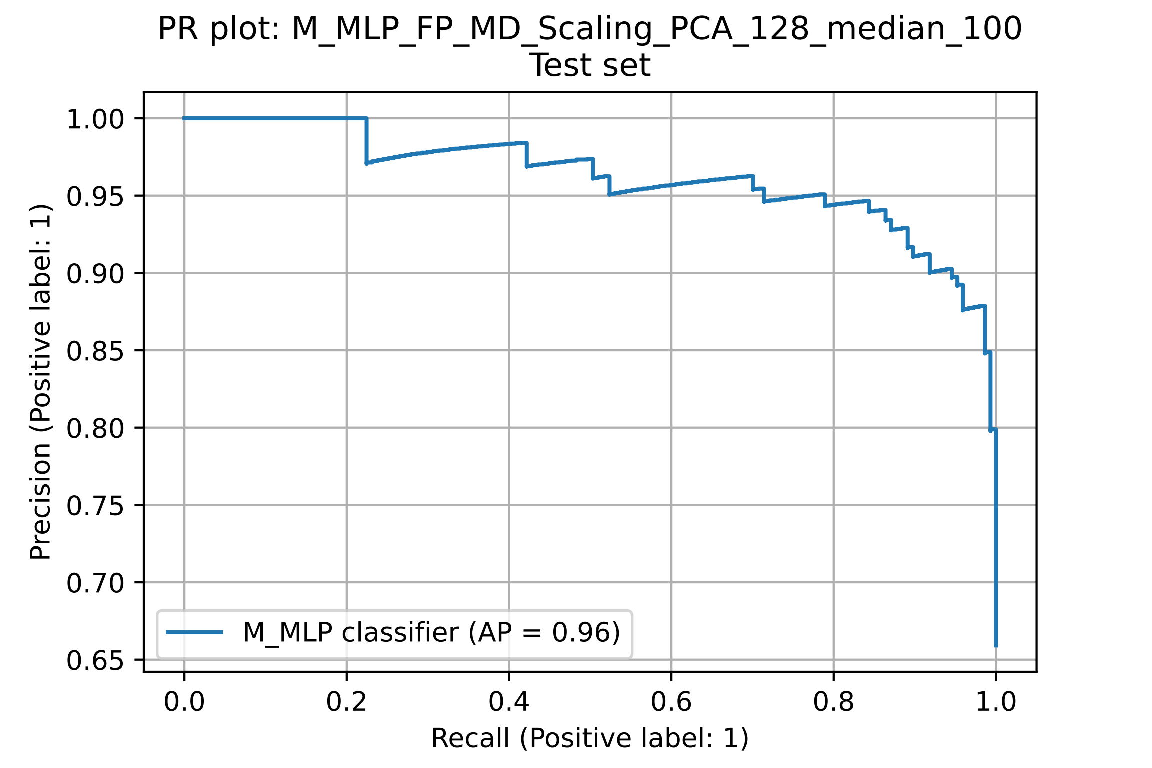
PR plot: M_MLP_FP_MD_Scaling_PCA_128_median_100
Test set
1.00
0.95
0.90
0.85
0.80
0.75
0.70
0.65
Precision (Positive label: 1)
0.0
0.2
0.4
0.6
0.8
1.0
Recall (Positive label: 1)
M_MLP classifier (AP = 0.96)

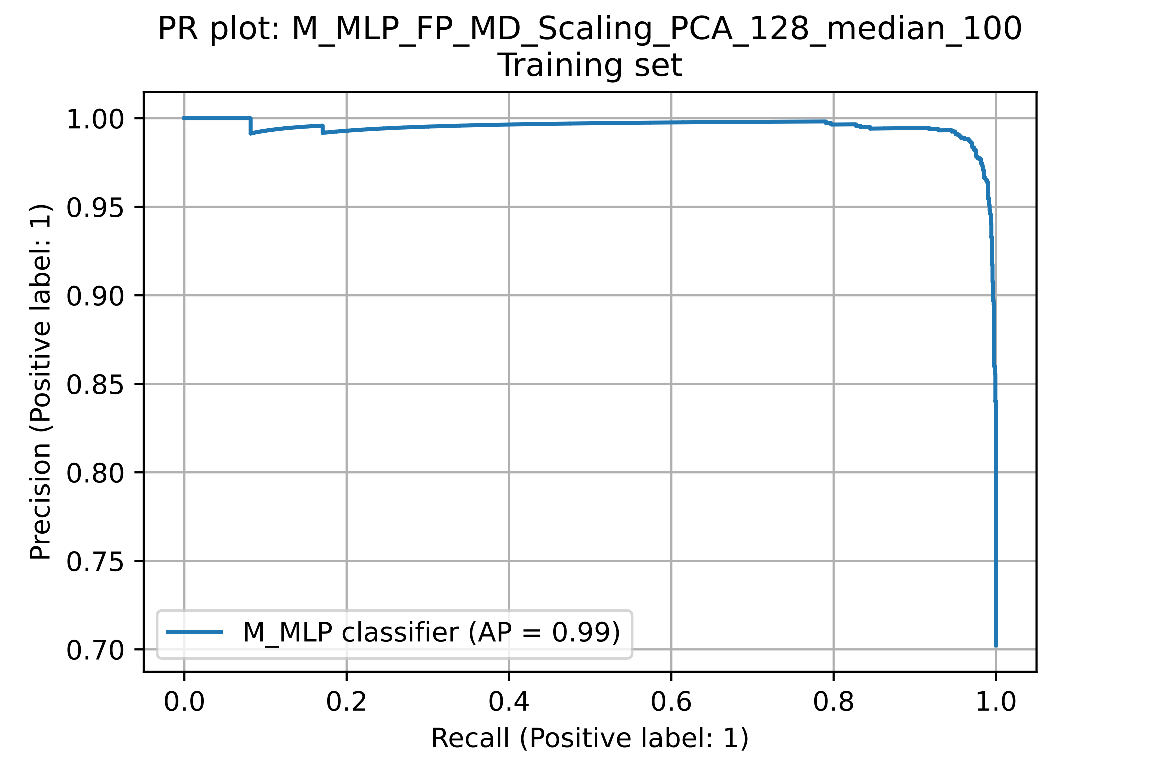

PR plot: M_MLP_FP_MD_Scaling_PCA_128_median_100
Training set
Precision (Positive label: 1)
Recall (Positive label: 1)
M_MLP classifier (AP = 0.99)
1.00
0.95
0.90
0.85
0.80
0.75
0.70
0.0
0.2
0.4
0.6
0.8
1.0

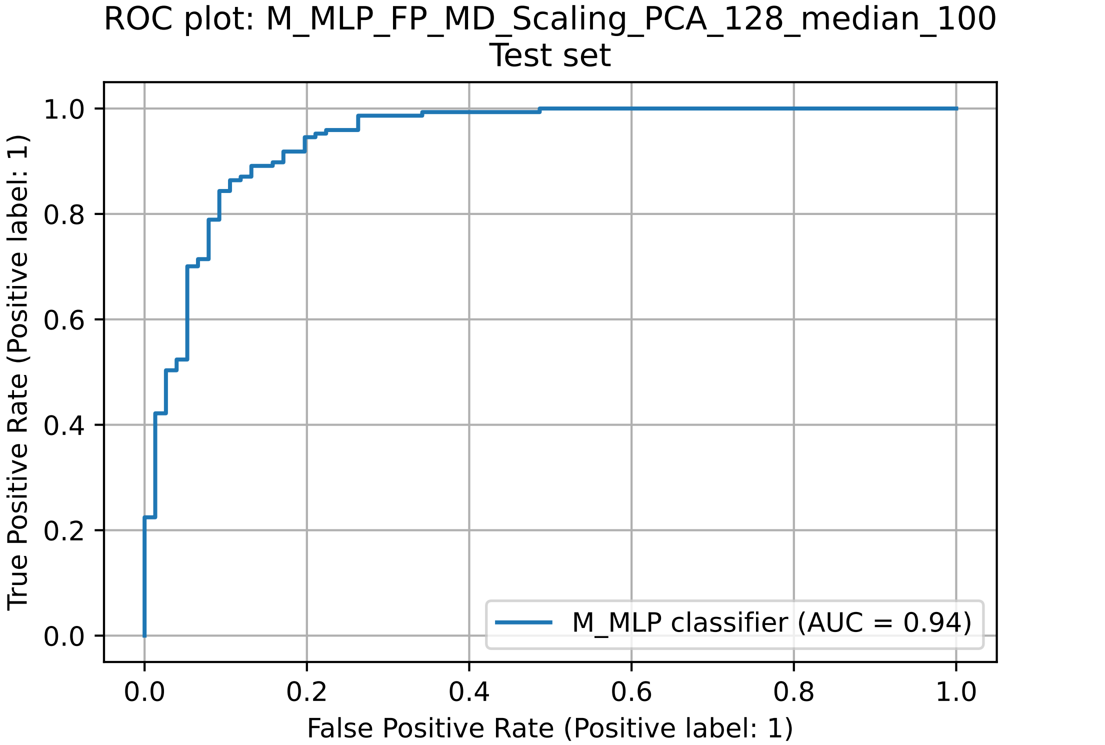
ROC plot: M_MLP_FP_MD_Scaling_PCA_128_median_100
Test set
True Positive Rate (Positive label: 1)
False Positive Rate (Positive label: 1)
0.0
0.2
0.4
0.6
0.8
1.0
M_MLP classifier (AUC = 0.94)

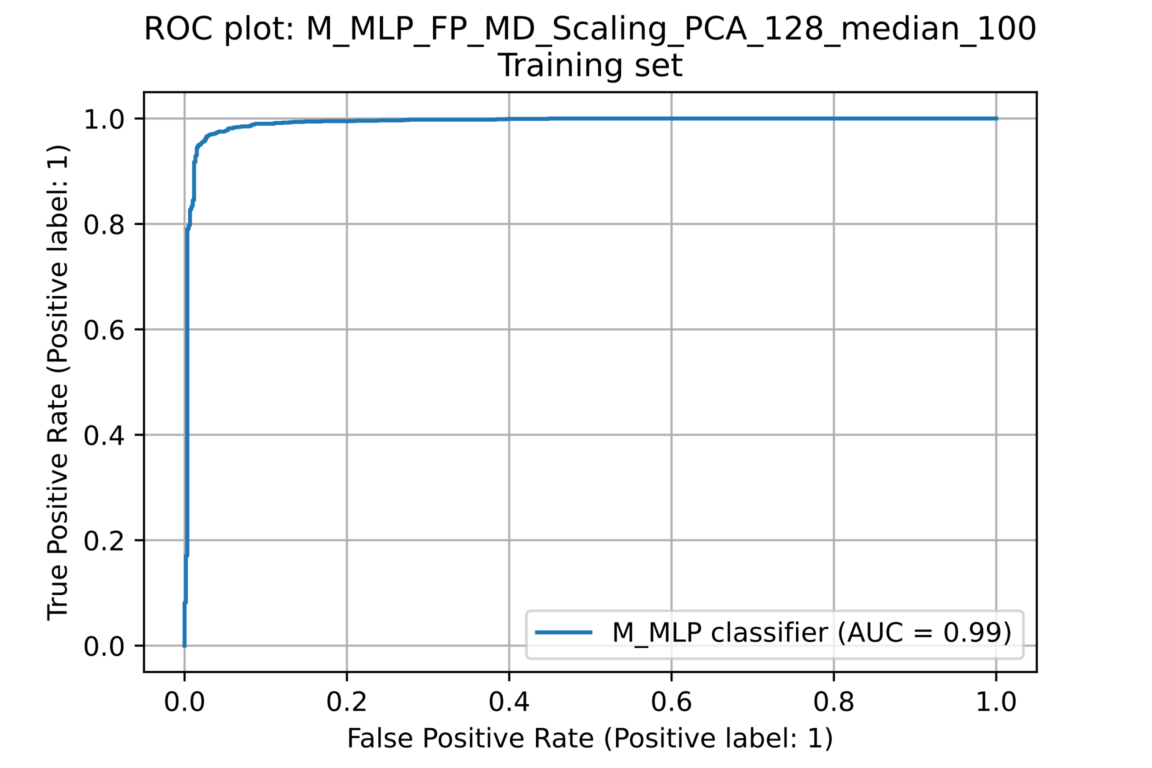

ROC plot: M_MLP_FP_MD_Scaling_PCA_128_median_100
Training set
True Positive Rate (Positive label: 1)
False Positive Rate (Positive label: 1)
0.0
0.2
0.4
0.6
0.8
1.0
M_MLP classifier (AUC = 0.99)

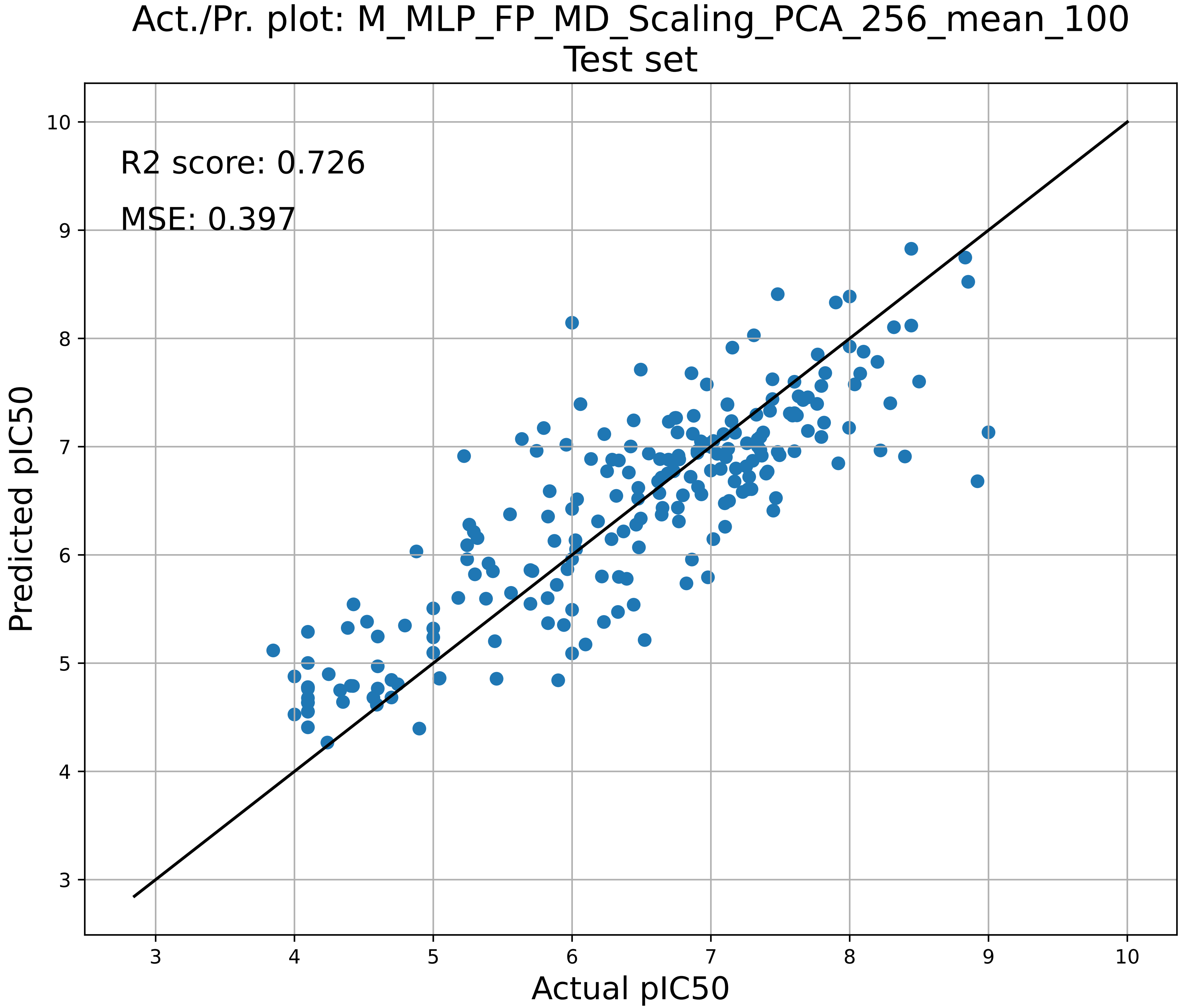
Act./Pr. plot: M_MLP_FP_MD_Scaling_PCA_256_mean_100
Test set
R2 score: 0.726
MSE: 0.397
Predicted pIC50
Actual pIC50
3
4
5
6
7
8
9
10

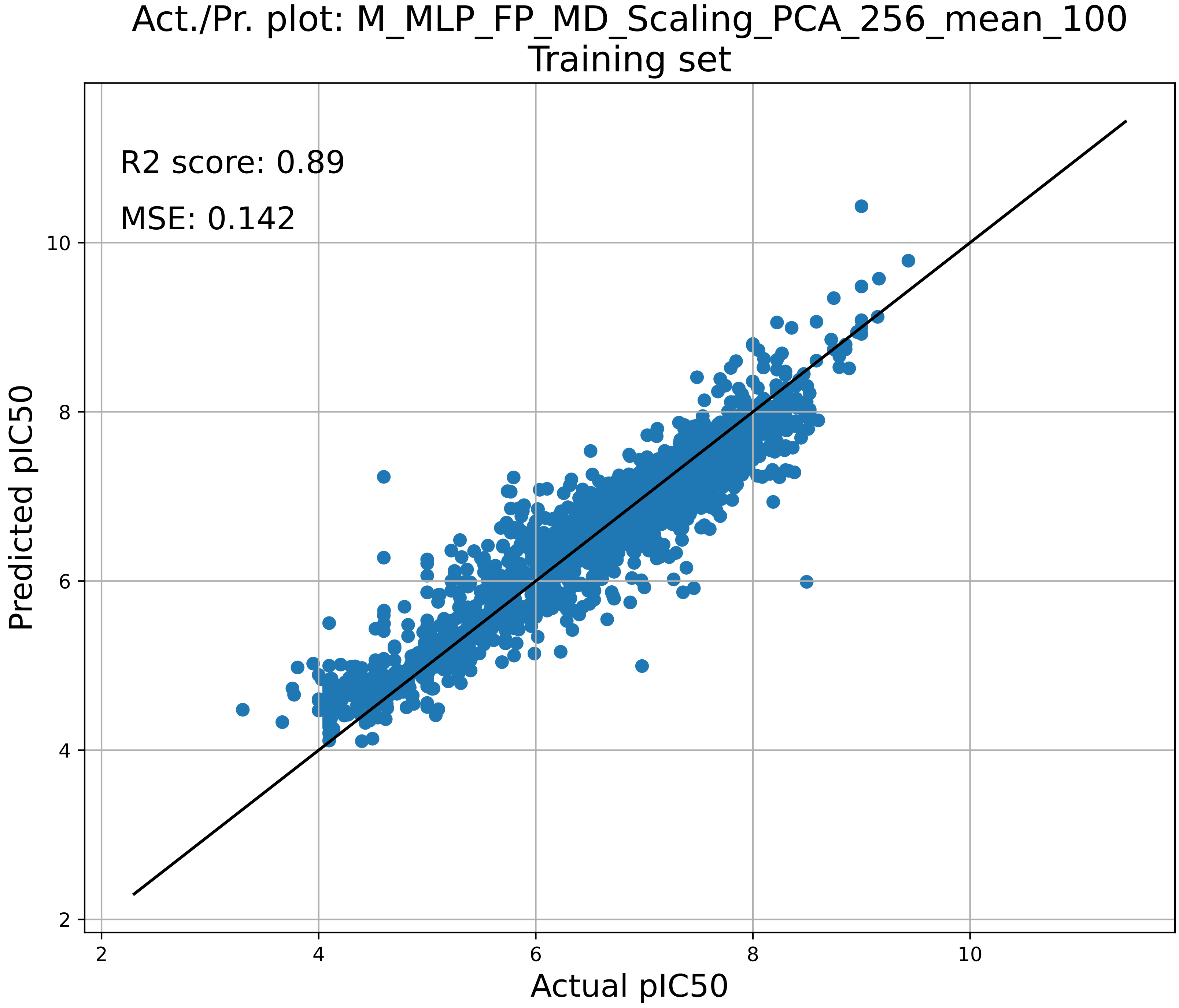

Act./Pr. plot: M_MLP_FP_MD_Scaling_PCA_256_mean_100
Training set
R2 score: 0.89
MSE: 0.142
Predicted pIC50
Actual pIC50
2
4
6
8
10
2
4
6
8
10

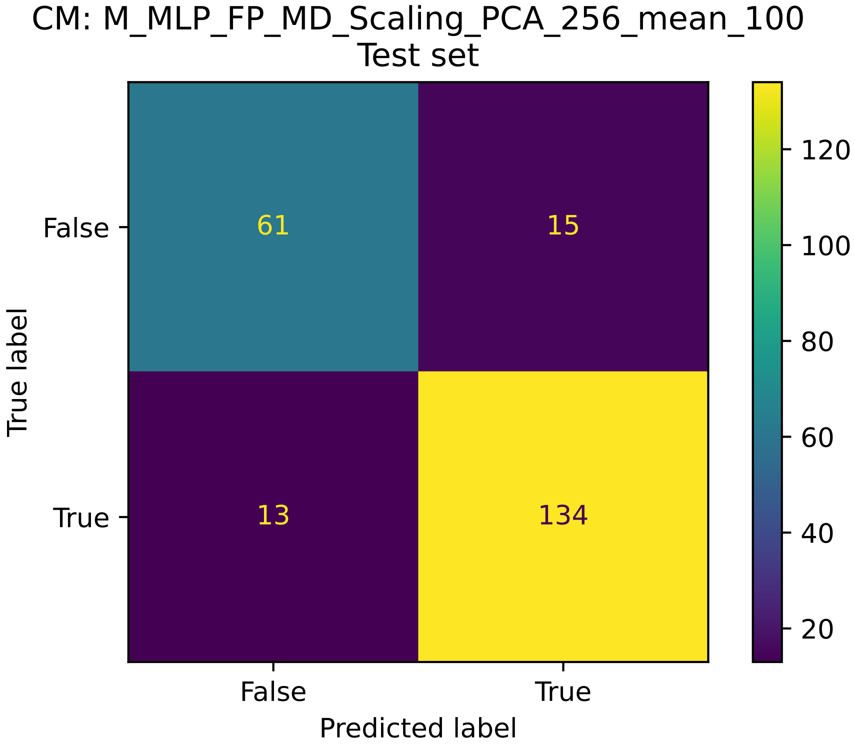
CM: M_MLP_FP_MD_Scaling_PCA_256_mean_100
Test set
True label
False
True
61
15
13
134
Predicted label
False
True
120
100
80
60
40
20

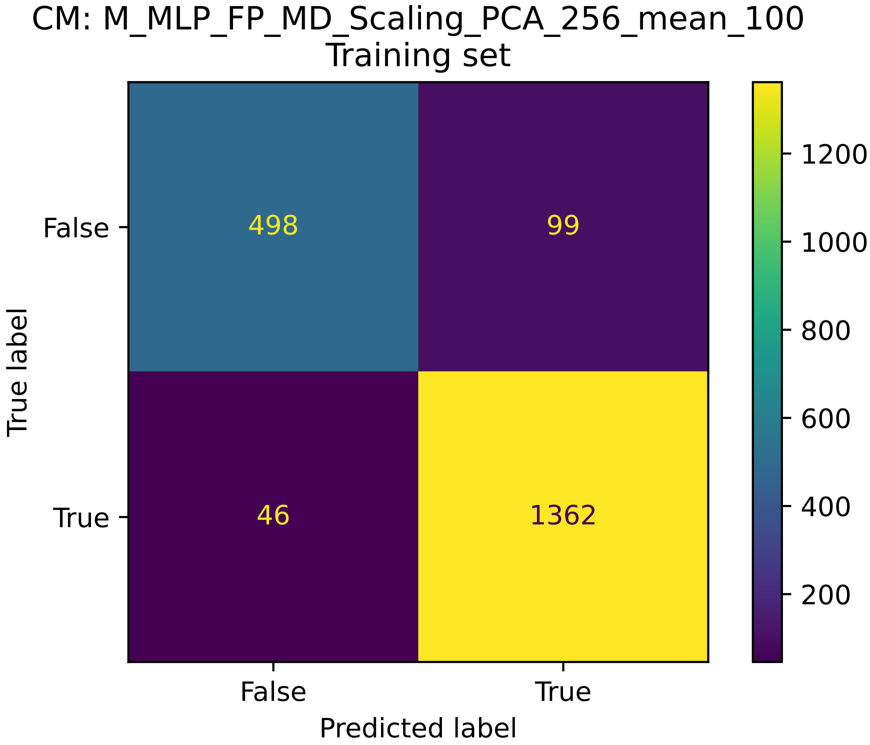
CM: M_MLP_FP_MD_Scaling_PCA_256_mean_100
Training set
True label
Predicted label
False
True
False
True
498
99
46
1362
1200
1000
800
600
400
200

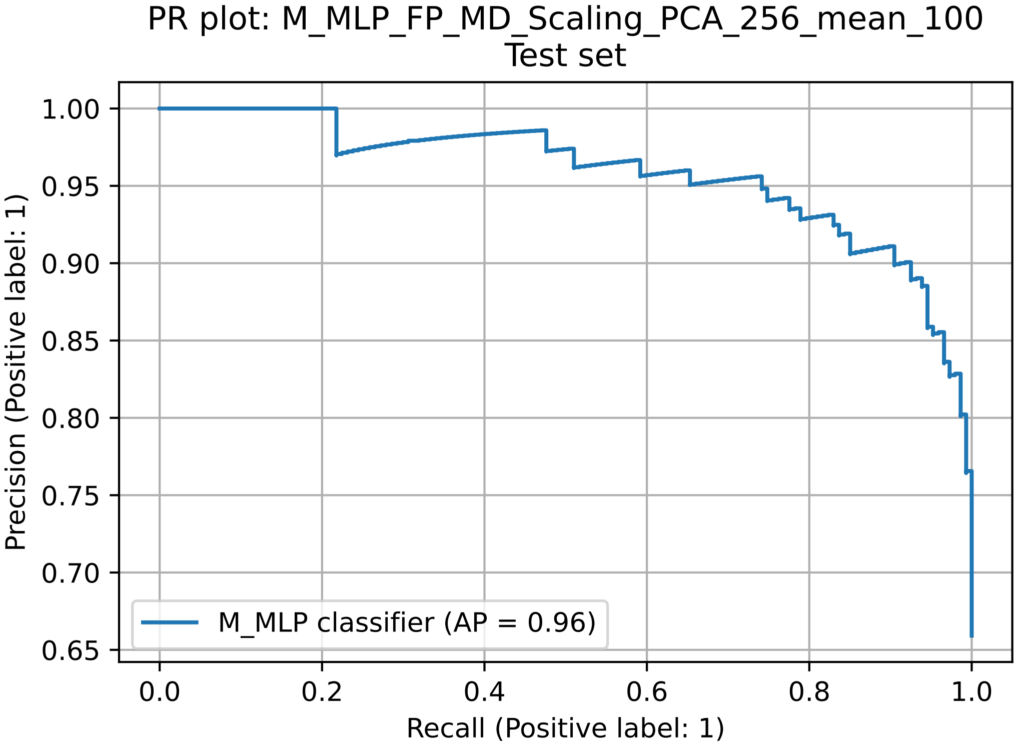
PR plot: M_MLP_FP_MD_Scaling_PCA_256_mean_100
Test set
Precision (Positive label: 1)
Recall (Positive label: 1)
1.00
0.95
0.90
0.85
0.80
0.75
0.70
0.65
0.0
0.2
0.4
0.6
0.8
1.0
M_MLP classifier (AP = 0.96)

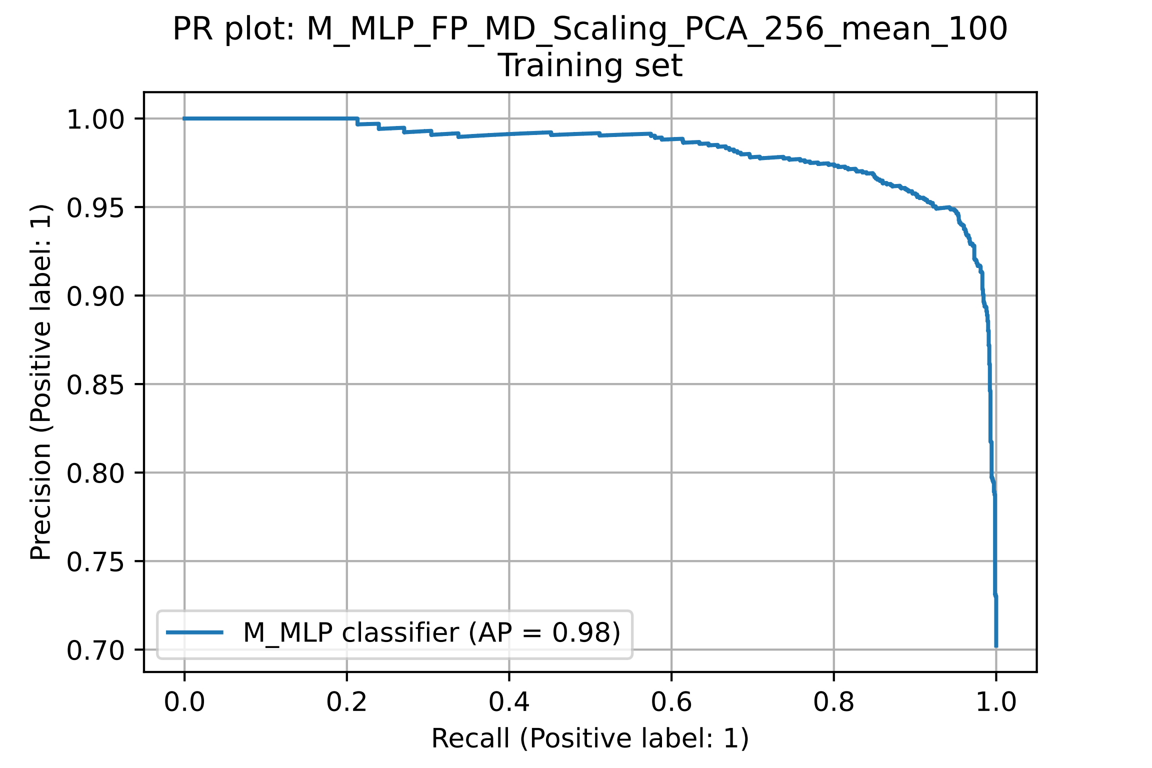
PR plot: M_MLP_FP_MD_Scaling_PCA_256_mean_100
Training set
1.00
0.95
0.90
0.85
0.80
0.75
0.70
Precision (Positive label: 1)
0.0
0.2
0.4
0.6
0.8
1.0
Recall (Positive label: 1)
M_MLP classifier (AP = 0.98)

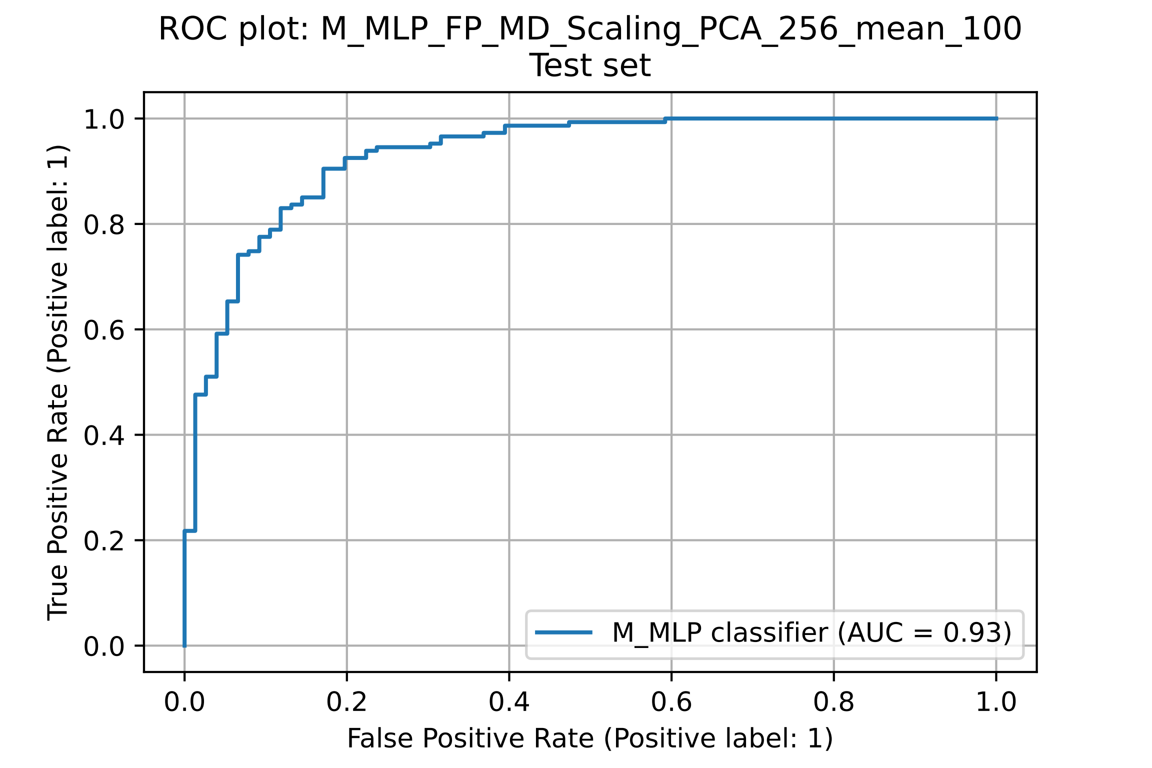
ROC plot: M_MLP_FP_MD_Scaling_PCA_256_mean_100
Test set
True Positive Rate (Positive label: 1)
False Positive Rate (Positive label: 1)
0.0
0.2
0.4
0.6
0.8
1.0
M_MLP classifier (AUC = 0.93)

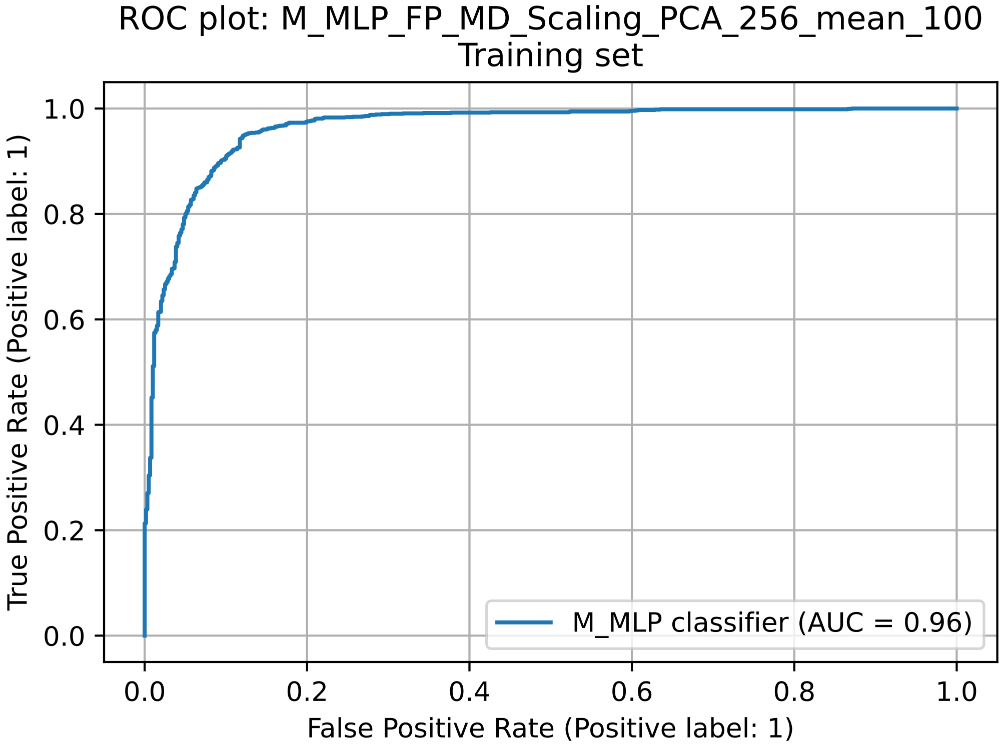

ROC plot: M_MLP_FP_MD_Scaling_PCA_256_mean_100
Training set
True Positive Rate (Positive label: 1)
False Positive Rate (Positive label: 1)
0.0
0.2
0.4
0.6
0.8
1.0
M_MLP classifier (AUC = 0.96)

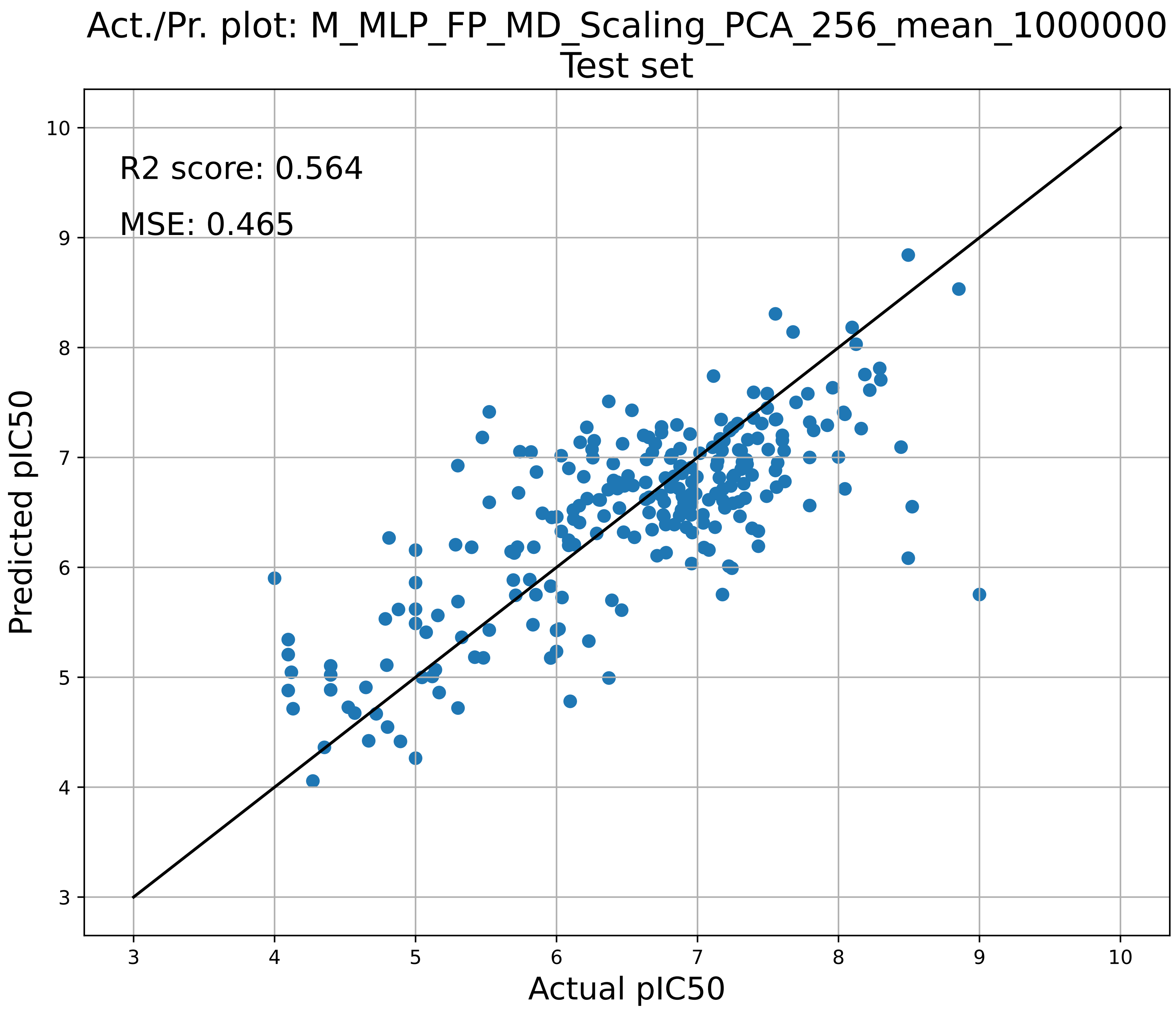

Act./Pr. plot: M_MLP_FP_MD_Scaling_PCA_256_mean_1000000
Test set
R2 score: 0.564
MSE: 0.465
Predicted pIC50
Actual pIC50
3
4
5
6
7
8
9
10

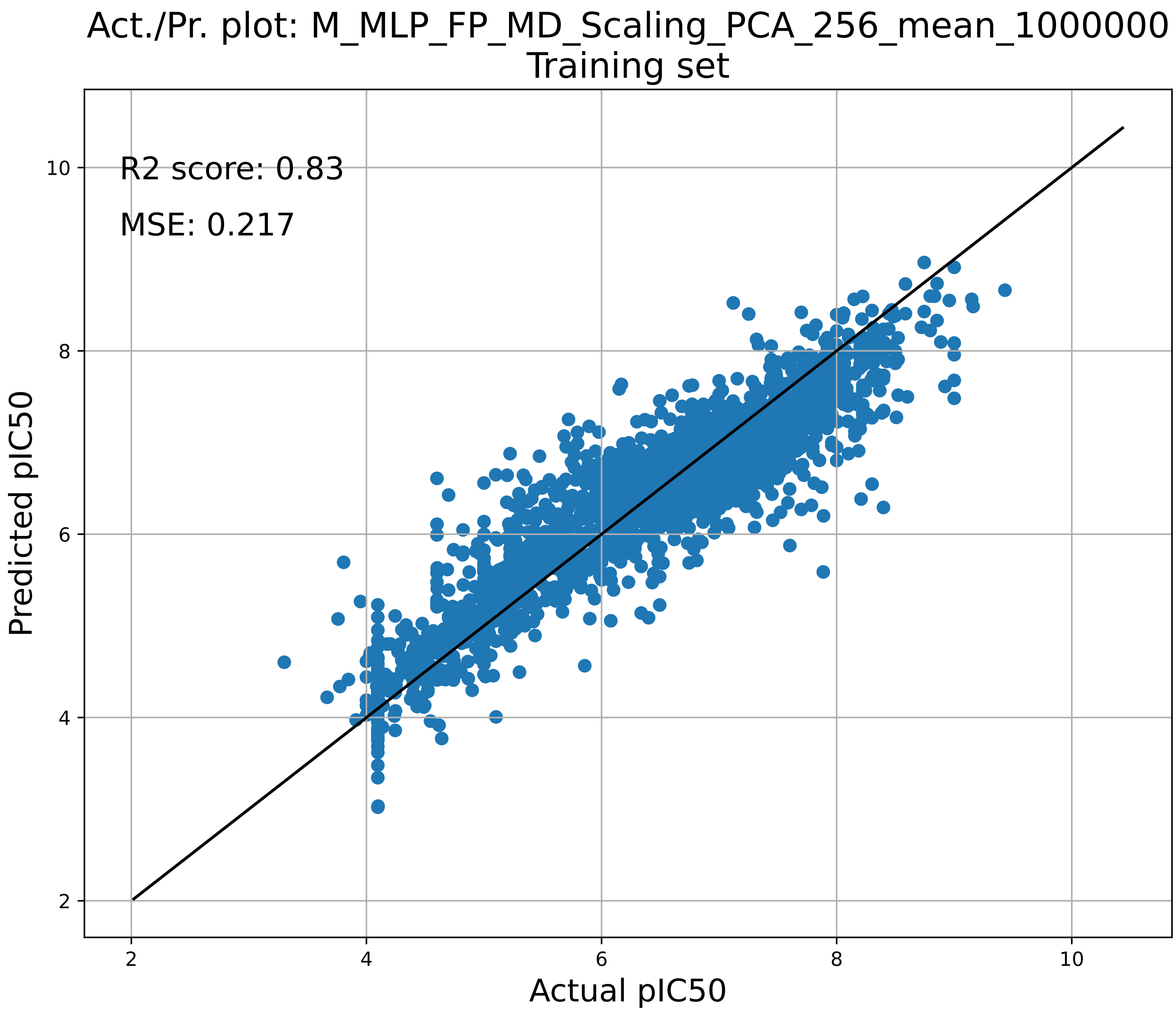
Act./Pr. plot: M_MLP_FP_MD_Scaling_PCA_256_mean_1000000
Training set
R2 score: 0.83
MSE: 0.217
Predicted pIC50
Actual pIC50
2
4
6
8
10
2
4
6
8
10

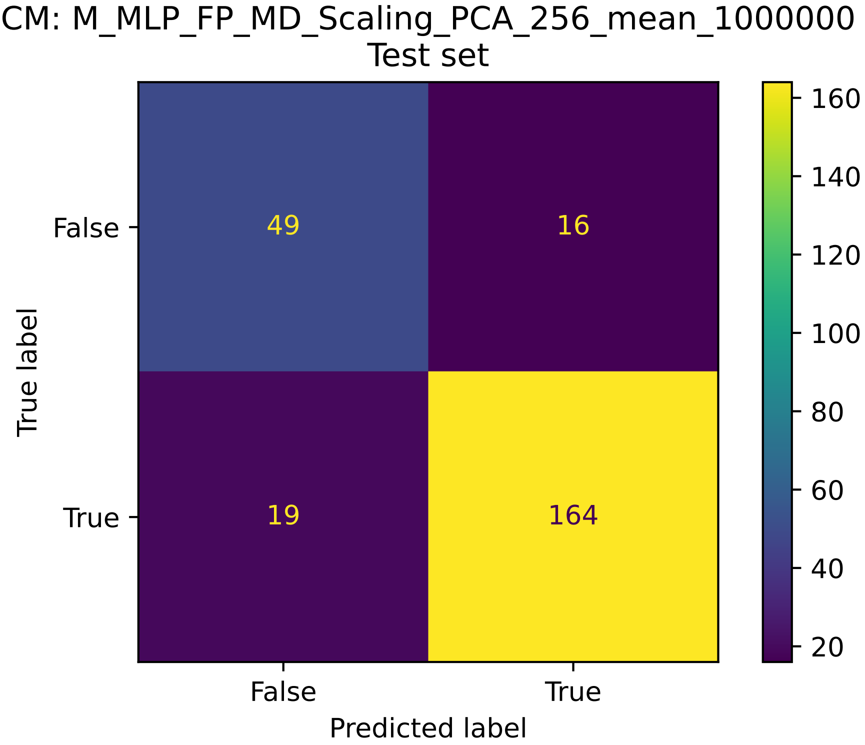
CM: M_MLP_FP_MD_Scaling_PCA_256_mean_1000000
Test set
True label
False
True
49
16
19
164
False
True
Predicted label
160
140
120
100
80
60
40
20

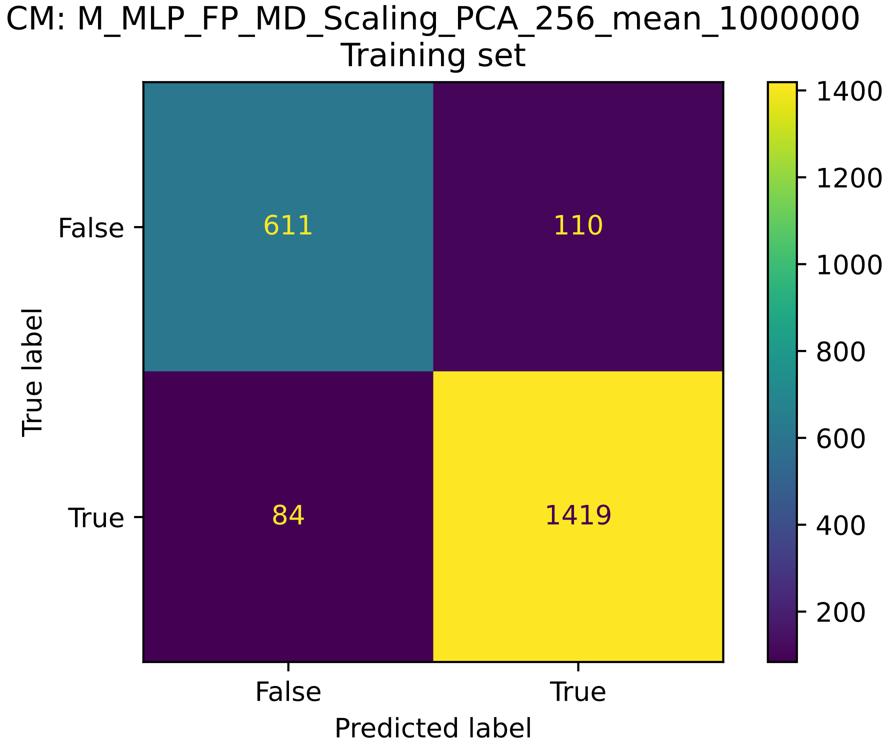
CM: M_MLP_FP_MD_Scaling_PCA_256_mean_1000000
Training set
611
110
84
1419
False
True
False
True
True label
Predicted label
1400
1200
1000
800
600
400
200

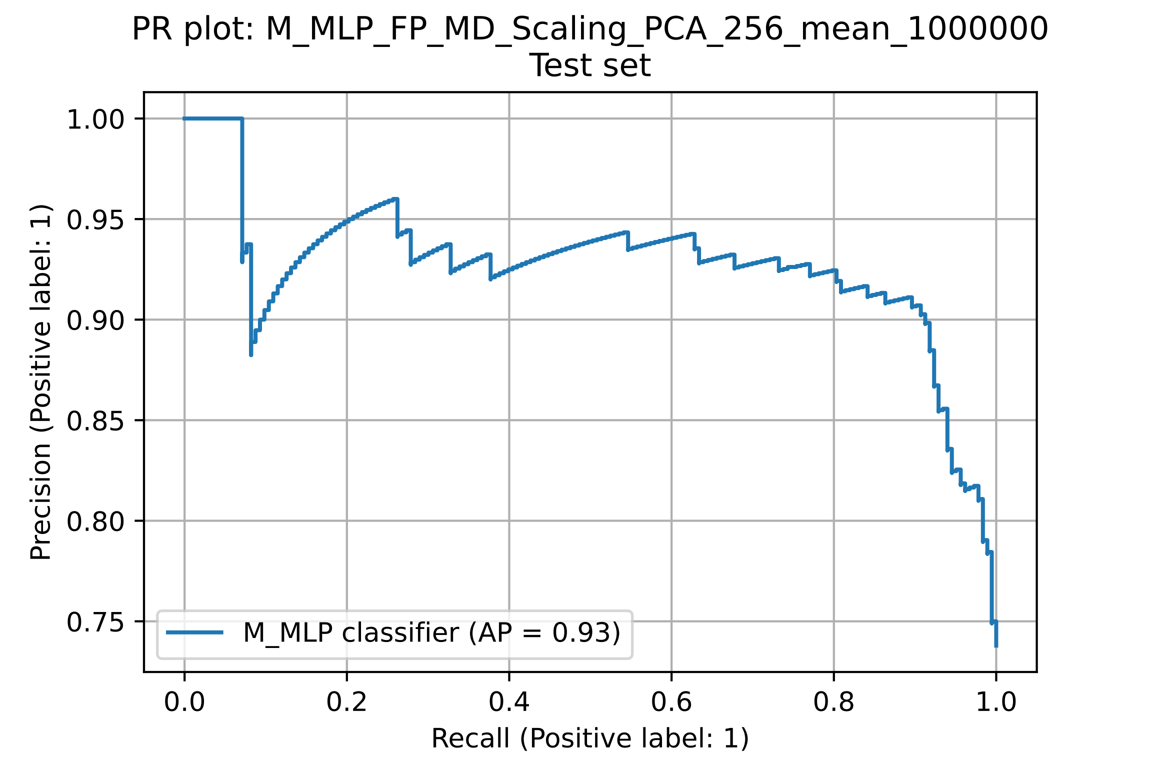
PR plot: M_MLP_FP_MD_Scaling_PCA_256_mean_1000000
Test set
Precision (Positive label: 1)
Recall (Positive label: 1)
1.00
0.95
0.90
0.85
0.80
0.75
0.0
0.2
0.4
0.6
0.8
1.0
M_MLP classifier (AP = 0.93)

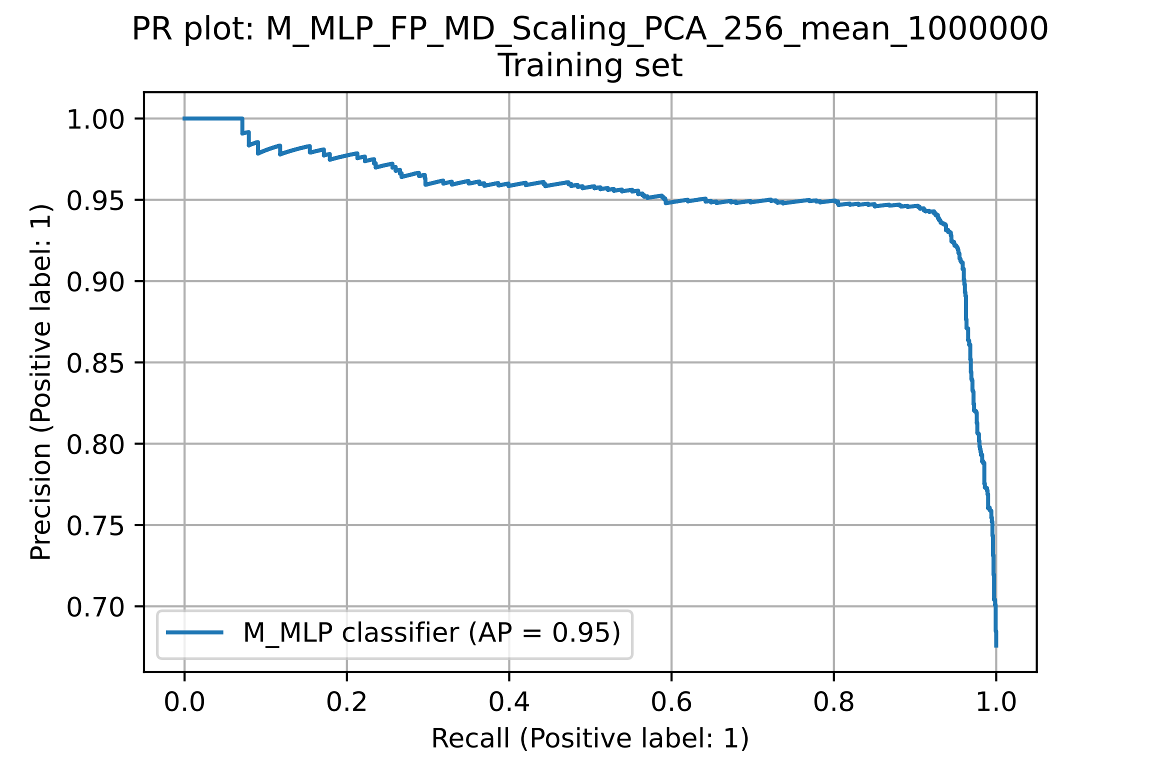

PR plot: M_MLP_FP_MD_Scaling_PCA_256_mean_1000000
Training set
Precision (Positive label: 1)
Recall (Positive label: 1)
1.00
0.95
0.90
0.85
0.80
0.75
0.70
0.0
0.2
0.4
0.6
0.8
1.0
M_MLP classifier (AP = 0.95)

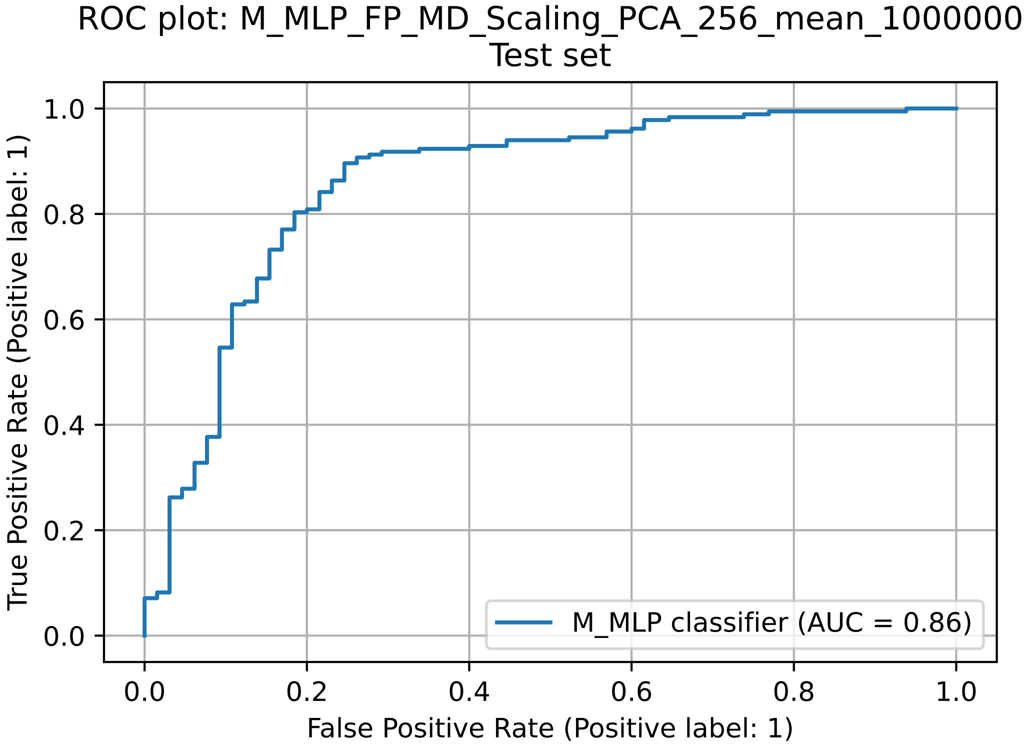

ROC plot: M_MLP_FP_MD_Scaling_PCA_256_mean_1000000
Test set
True Positive Rate (Positive label: 1)
False Positive Rate (Positive label: 1)
0.0
0.2
0.4
0.6
0.8
1.0
M_MLP classifier (AUC = 0.86)

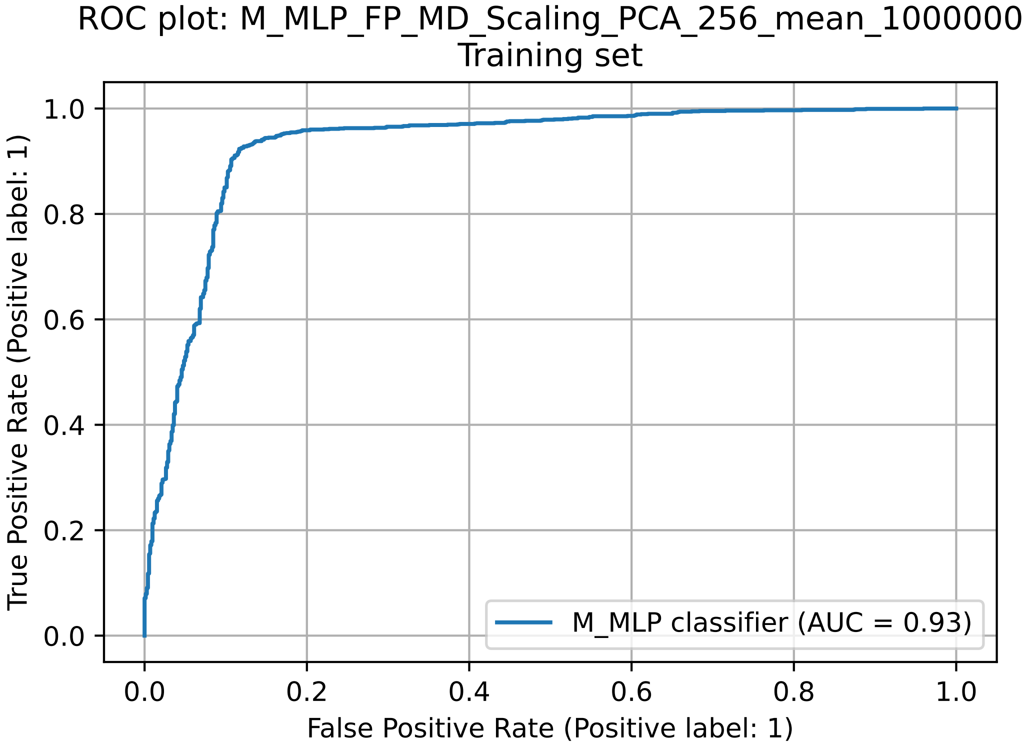
ROC plot: M_MLP_FP_MD_Scaling_PCA_256_mean_1000000
Training set
True Positive Rate (Positive label: 1)
False Positive Rate (Positive label: 1)
0.0
0.2
0.4
0.6
0.8
1.0
M_MLP classifier (AUC = 0.93)

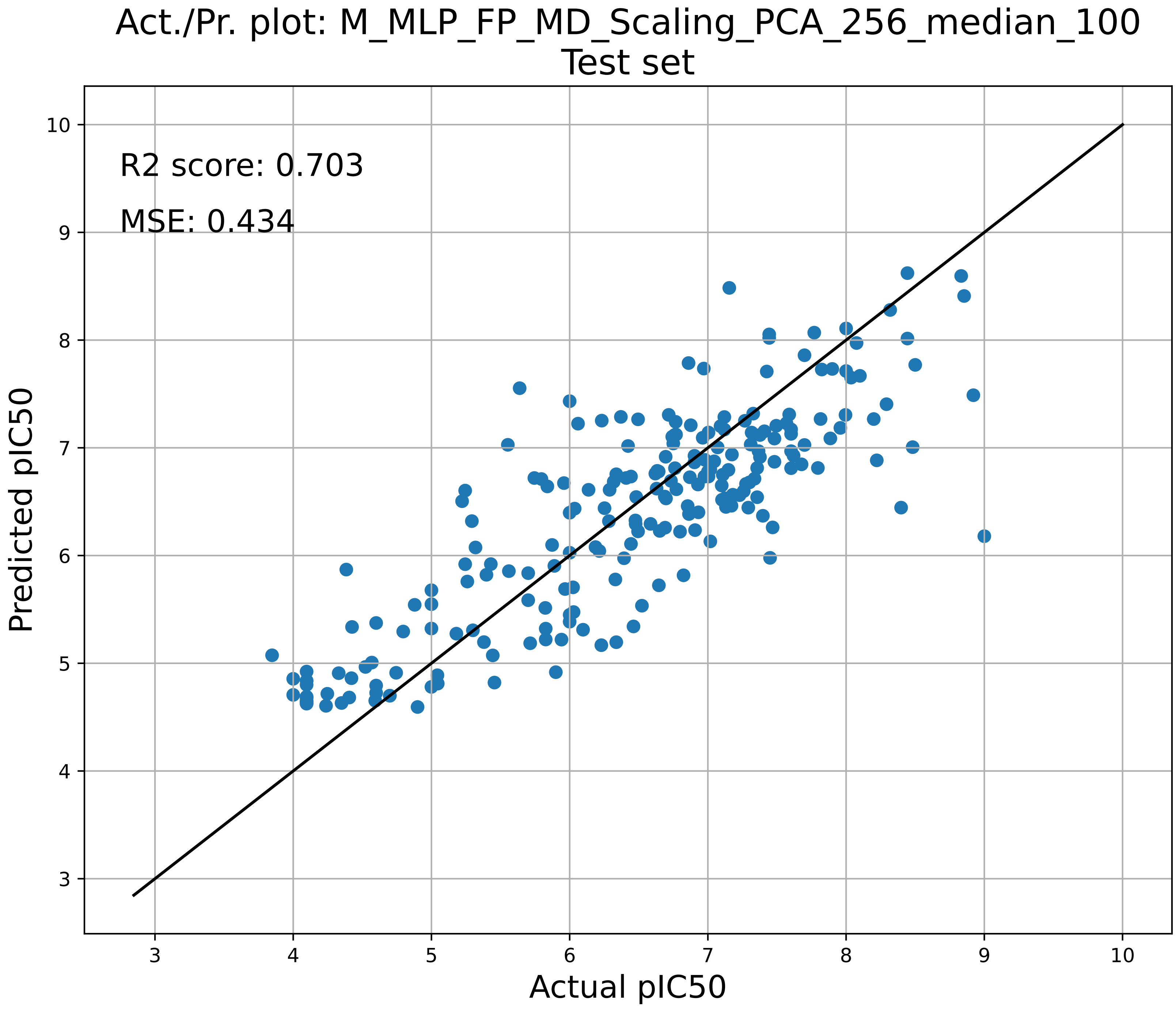

Act./Pr. plot: M_MLP_FP_MD_Scaling_PCA_256_median_100
Test set
R2 score: 0.703
MSE: 0.434
Predicted pIC50
Actual pIC50
3
4
5
6
7
8
9
10

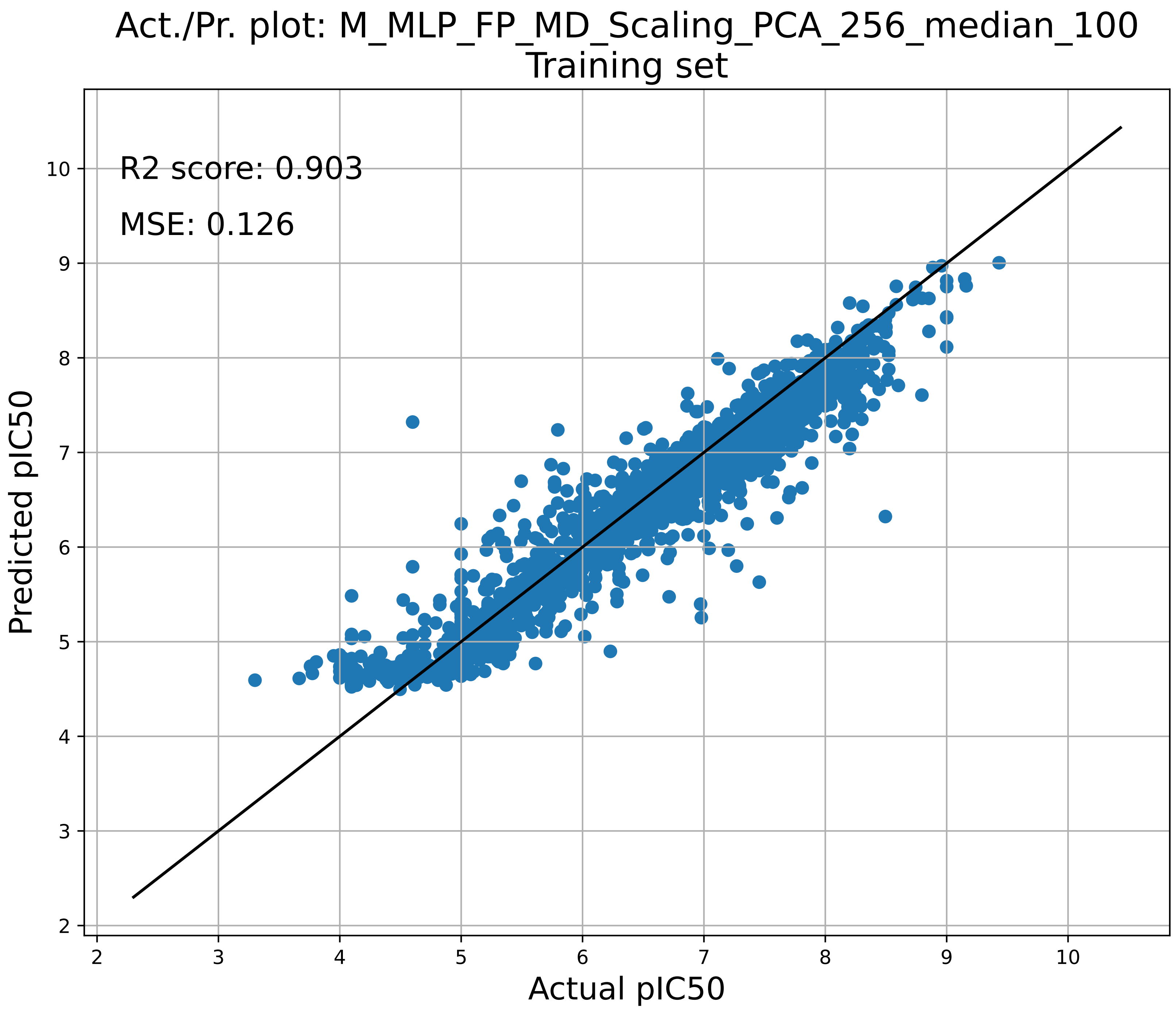

Act./Pr. plot: M_MLP_FP_MD_Scaling_PCA_256_median_100
Training set
R2 score: 0.903
MSE: 0.126
Predicted pIC50
Actual pIC50
2
3
4
5
6
7
8
9
10

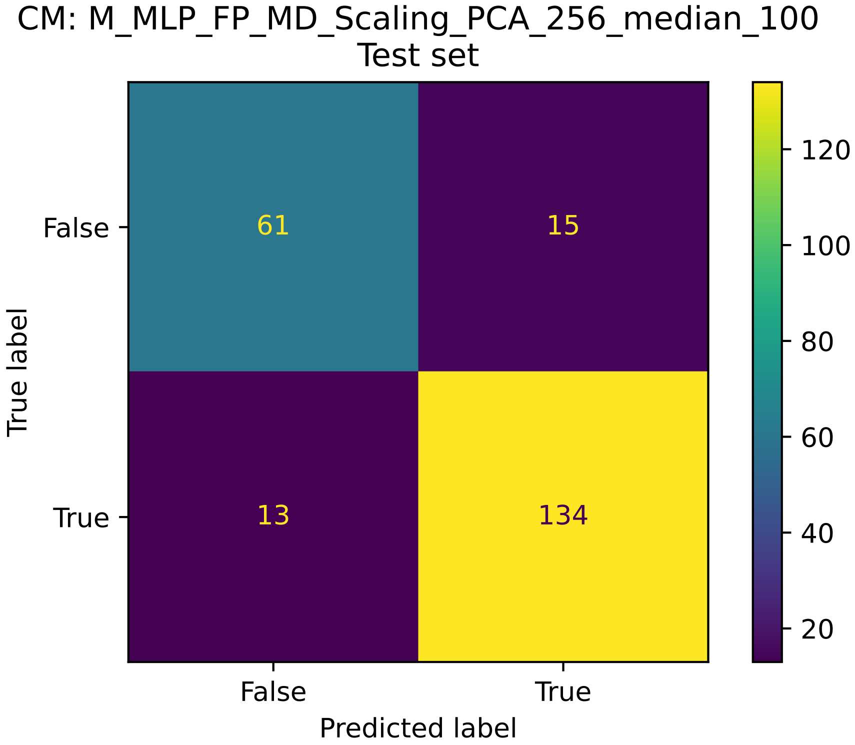
CM: M_MLP_FP_MD_Scaling_PCA_256_median_100
Test set
True label
Predicted label
False
True
False
True
61
15
13
134
120
100
80
60
40
20

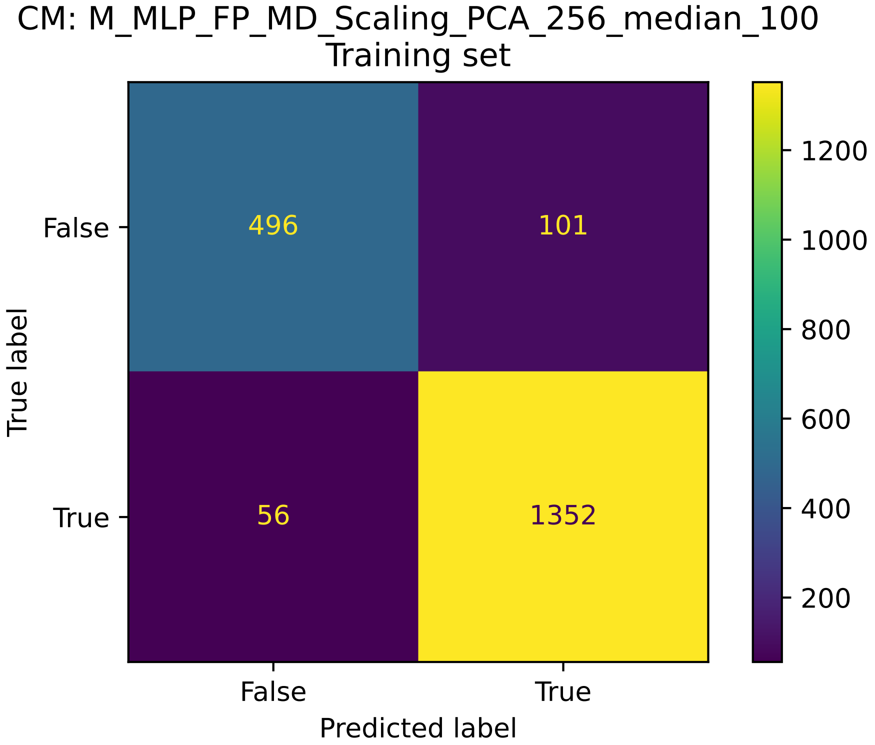

CM: M_MLP_FP_MD_Scaling_PCA_256_median_100
Training set
True label
False
True
496
101
56
1352
Predicted label
1200
1000
800
600
400
200

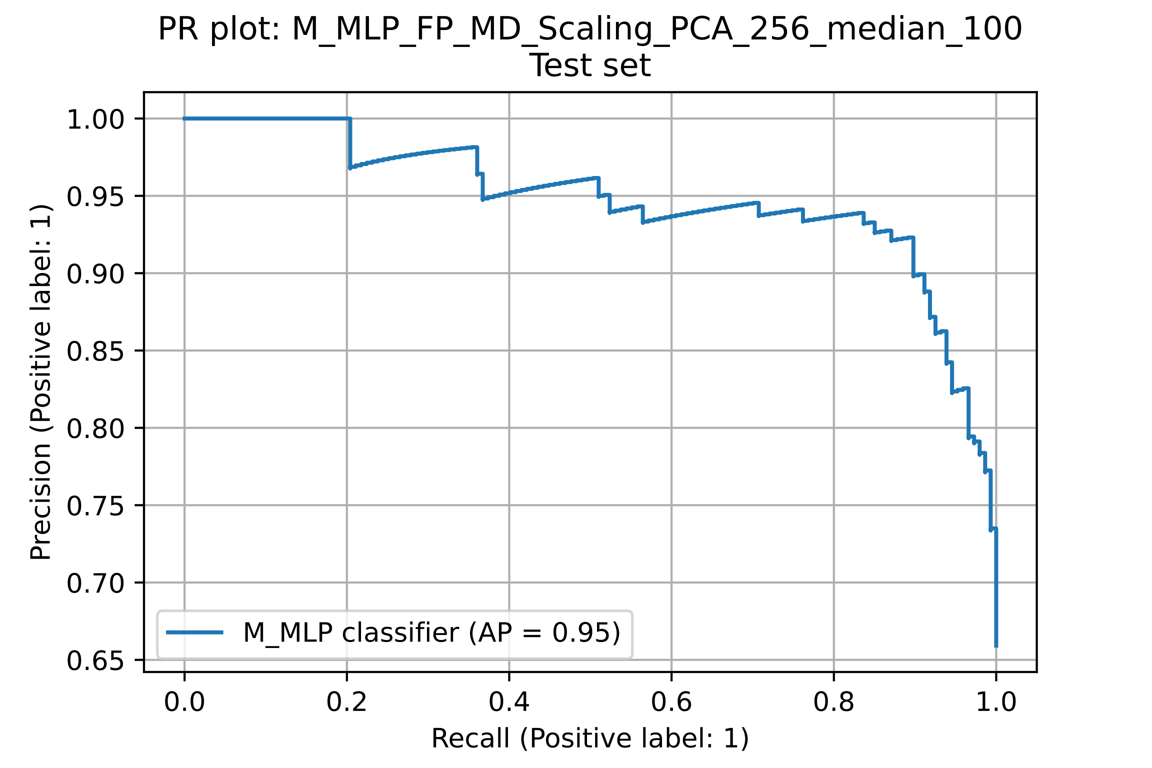
PR plot: M_MLP_FP_MD_Scaling_PCA_256_median_100
Test set
Precision (Positive label: 1)
1.00
0.95
0.90
0.85
0.80
0.75
0.70
0.65
0.0
0.2
0.4
0.6
0.8
1.0
Recall (Positive label: 1)
M_MLP classifier (AP = 0.95)

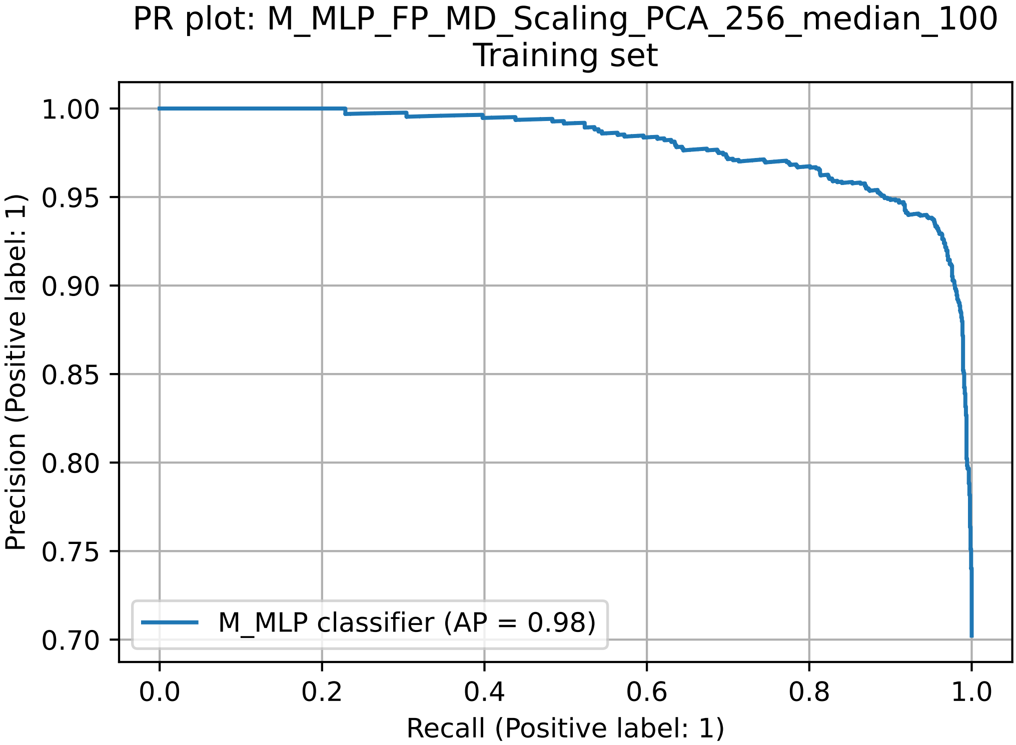
PR plot: M_MLP_FP_MD_Scaling_PCA_256_median_100
Training set
Precision (Positive label: 1)
Recall (Positive label: 1)
M_MLP classifier (AP = 0.98)
1.00
0.95
0.90
0.85
0.80
0.75
0.70
0.0
0.2
0.4
0.6
0.8
1.0

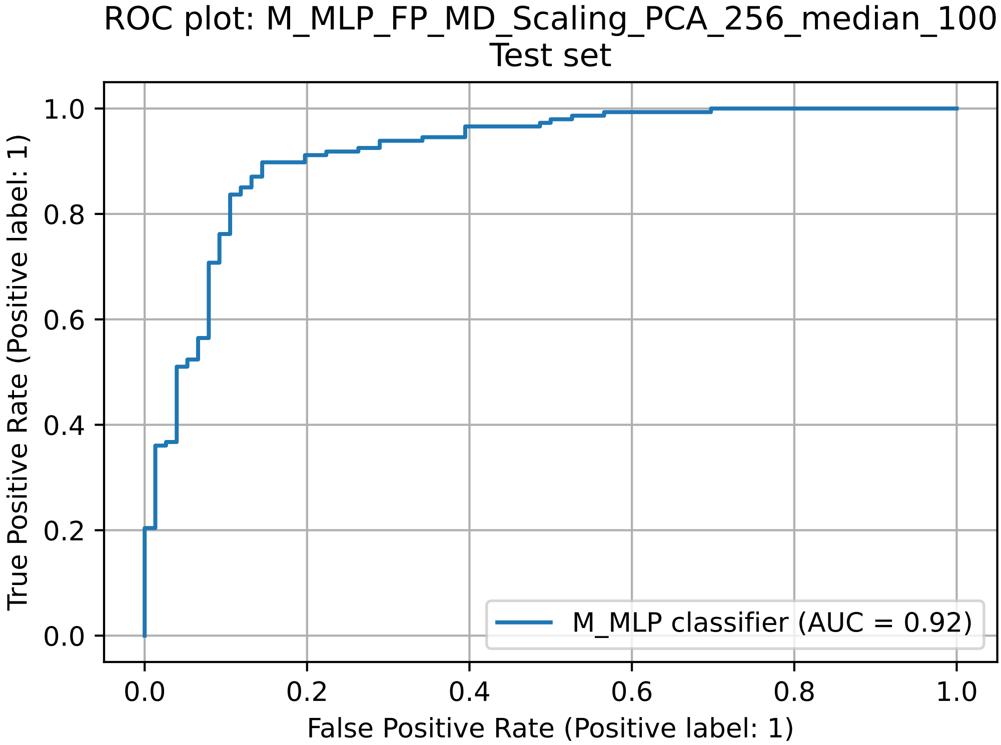
ROC plot: M_MLP_FP_MD_Scaling_PCA_256_median_100
Test set
True Positive Rate (Positive label: 1)
False Positive Rate (Positive label: 1)
0.0
0.2
0.4
0.6
0.8
1.0
M_MLP classifier (AUC = 0.92)

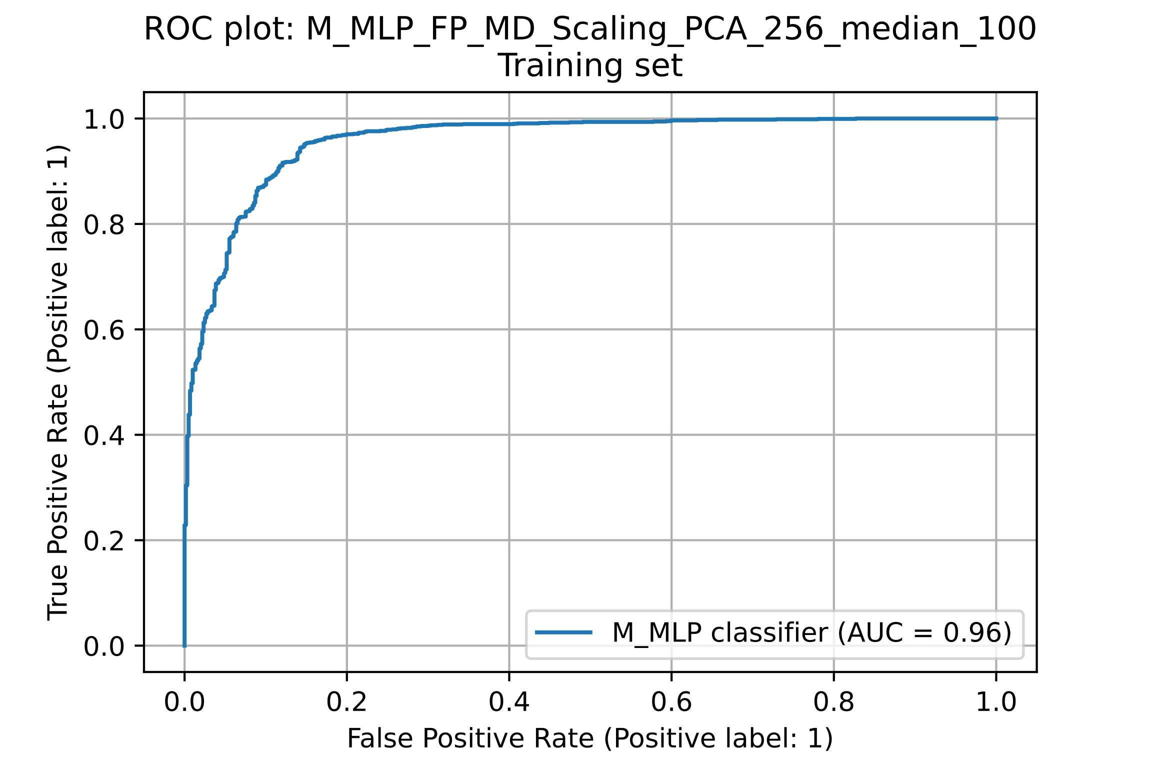
ROC plot: M_MLP_FP_MD_Scaling_PCA_256_median_100
Training set
True Positive Rate (Positive label: 1)
False Positive Rate (Positive label: 1)
0.0
0.2
0.4
0.6
0.8
1.0
M_MLP classifier (AUC = 0.96)

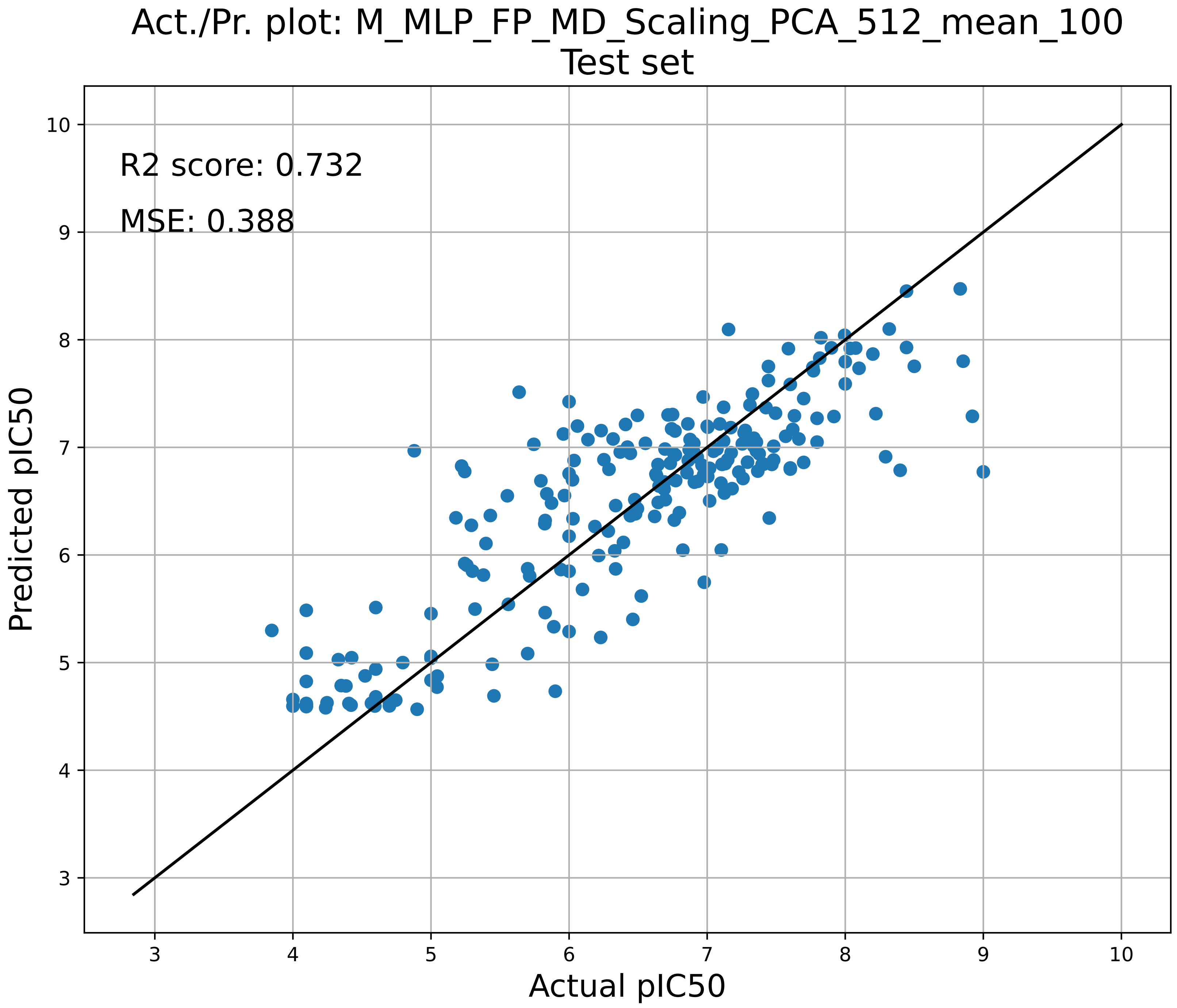
Act./Pr. plot: M_MLP_FP_MD_Scaling_PCA_512_mean_100
Test set
R2 score: 0.732
MSE: 0.388
Predicted pIC50
Actual pIC50
3
4
5
6
7
8
9
10

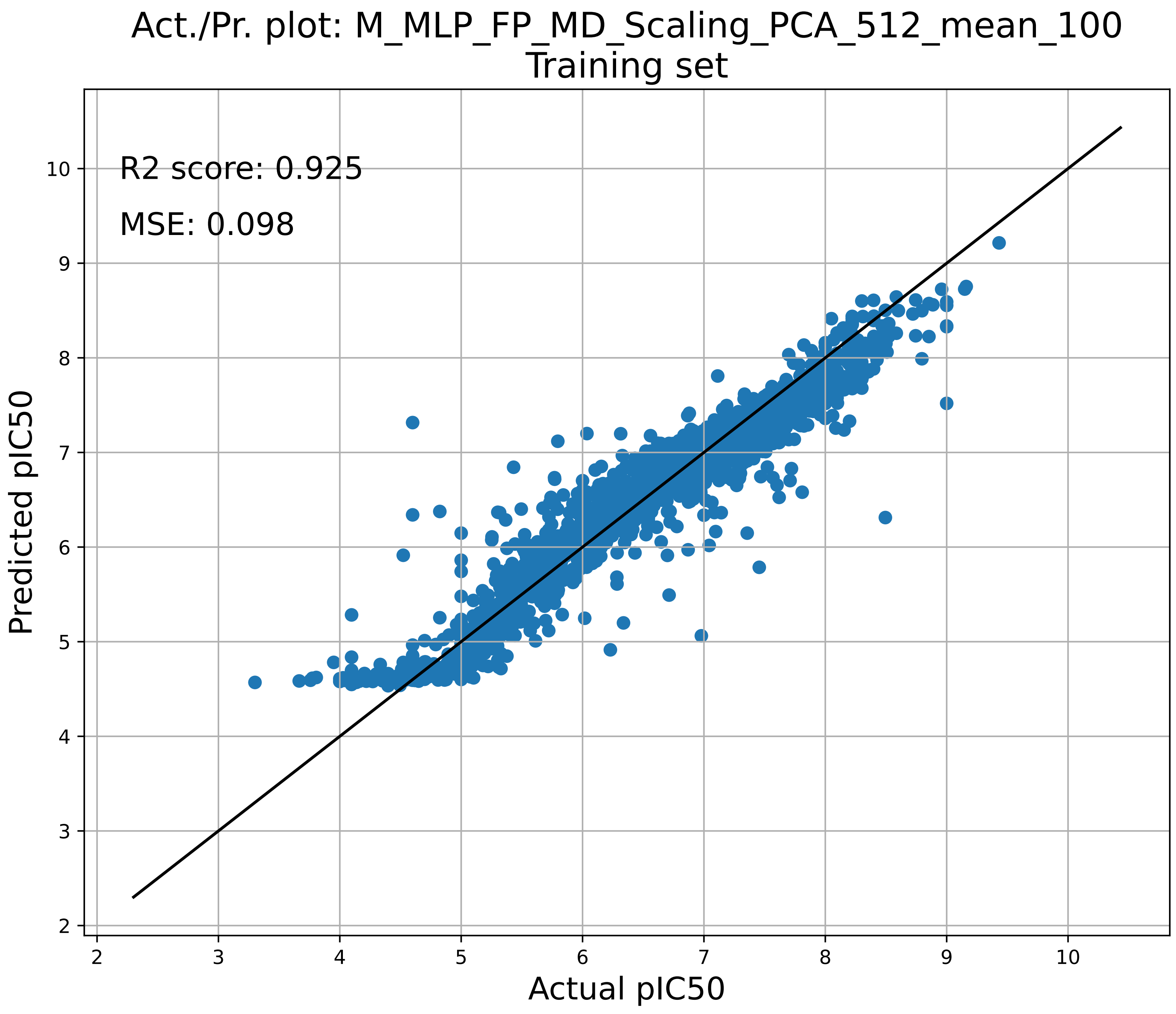

Act./Pr. plot: M_MLP_FP_MD_Scaling_PCA_512_mean_100
Training set
R2 score: 0.925
MSE: 0.098
Predicted pIC50
Actual pIC50
2
3
4
5
6
7
8
9
10

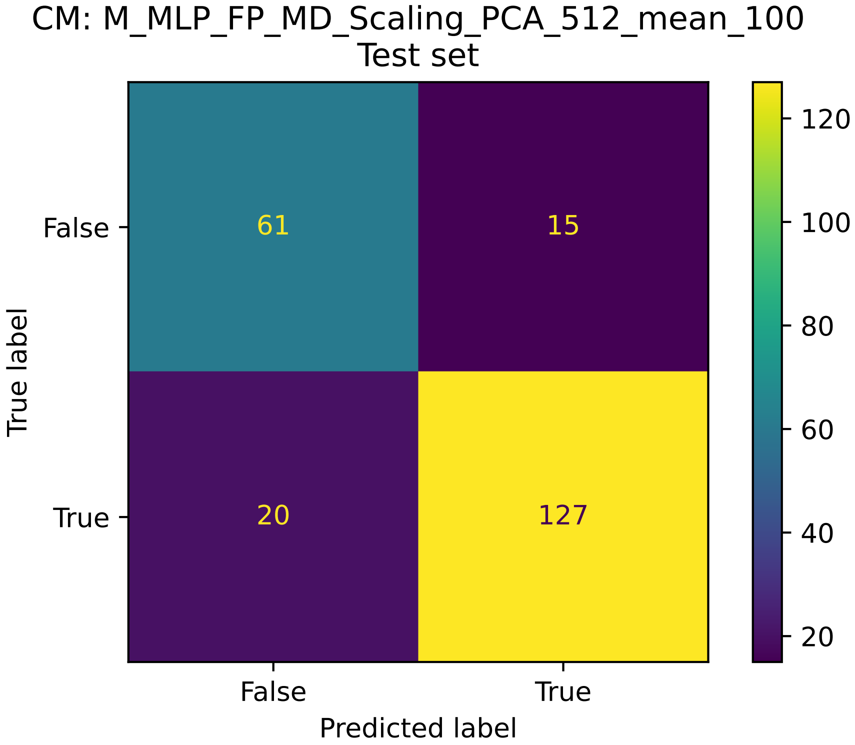
CM: M_MLP_FP_MD_Scaling_PCA_512_mean_100
Test set
61
15
20
127
False
True
True label
False
True
Predicted label
120
100
80
60
40
20

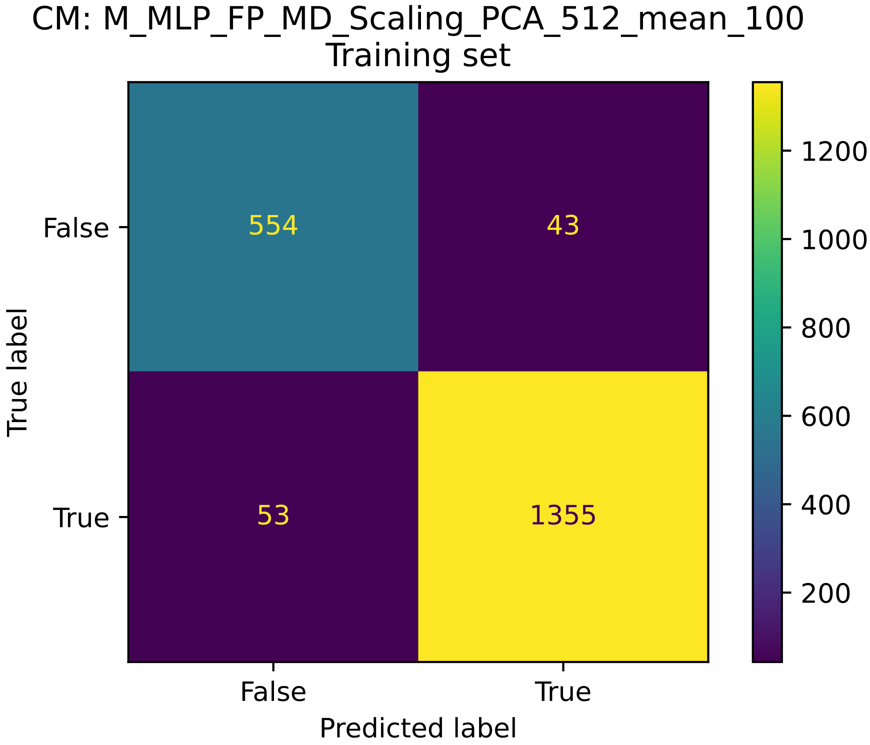

CM: M_MLP_FP_MD_Scaling_PCA_512_mean_100
Training set
554
43
53
1355
False
True
True label
False
True
Predicted label
1200
1000
800
600
400
200

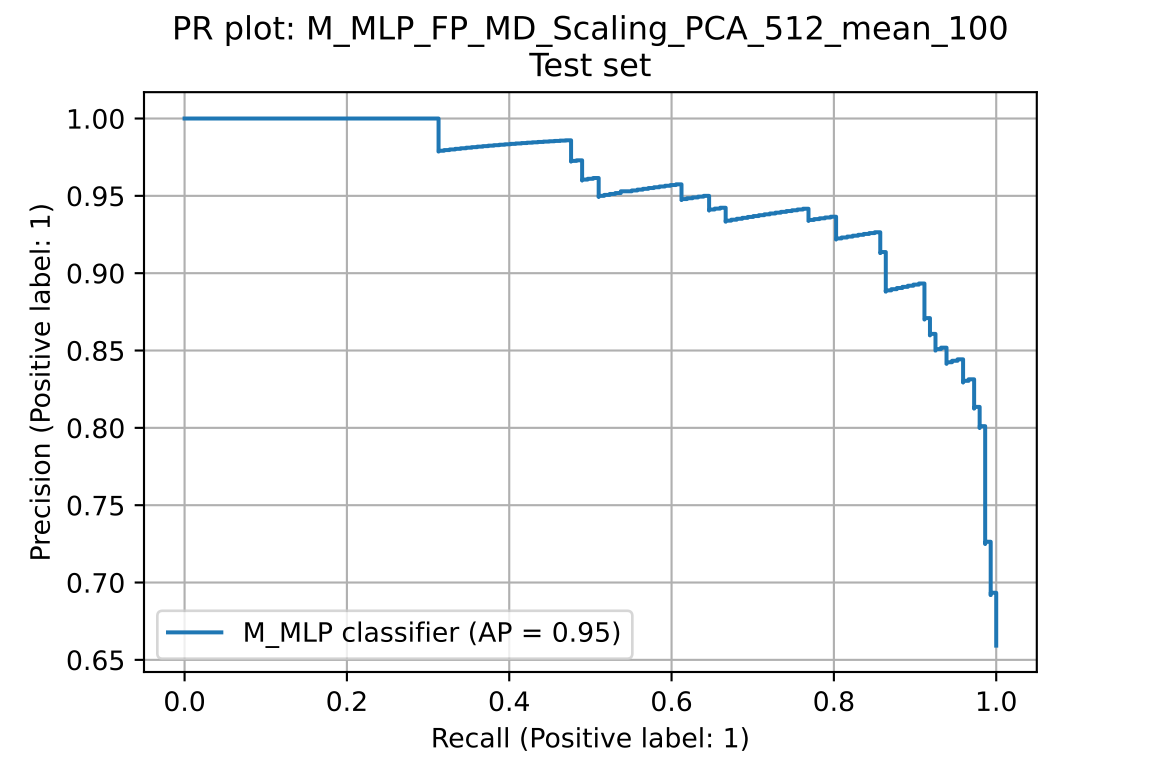

PR plot: M_MLP_FP_MD_Scaling_PCA_512_mean_100
Test set
Precision (Positive label: 1)
Recall (Positive label: 1)
1.00
0.95
0.90
0.85
0.80
0.75
0.70
0.65
0.0
0.2
0.4
0.6
0.8
1.0
M_MLP classifier (AP = 0.95)

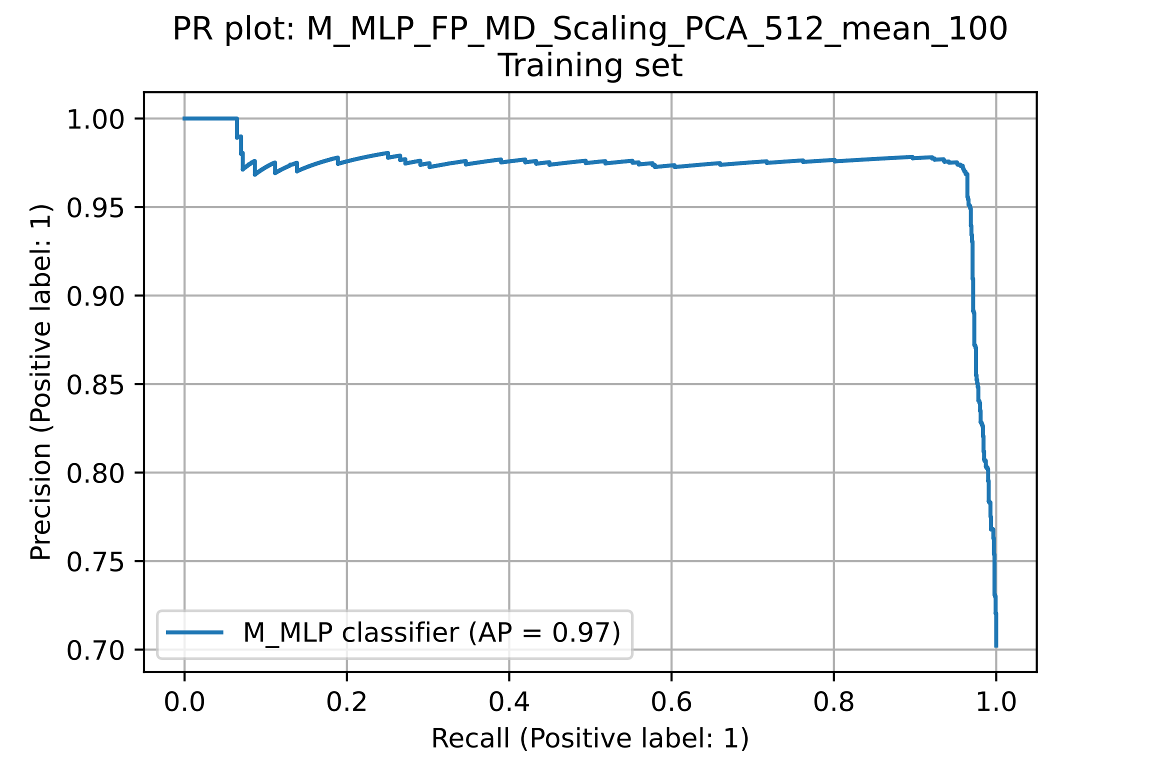
PR plot: M_MLP_FP_MD_Scaling_PCA_512_mean_100
Training set
Precision (Positive label: 1)
1.00
0.95
0.90
0.85
0.80
0.75
0.70
0.0
0.2
0.4
0.6
0.8
1.0
Recall (Positive label: 1)
M_MLP classifier (AP = 0.97)

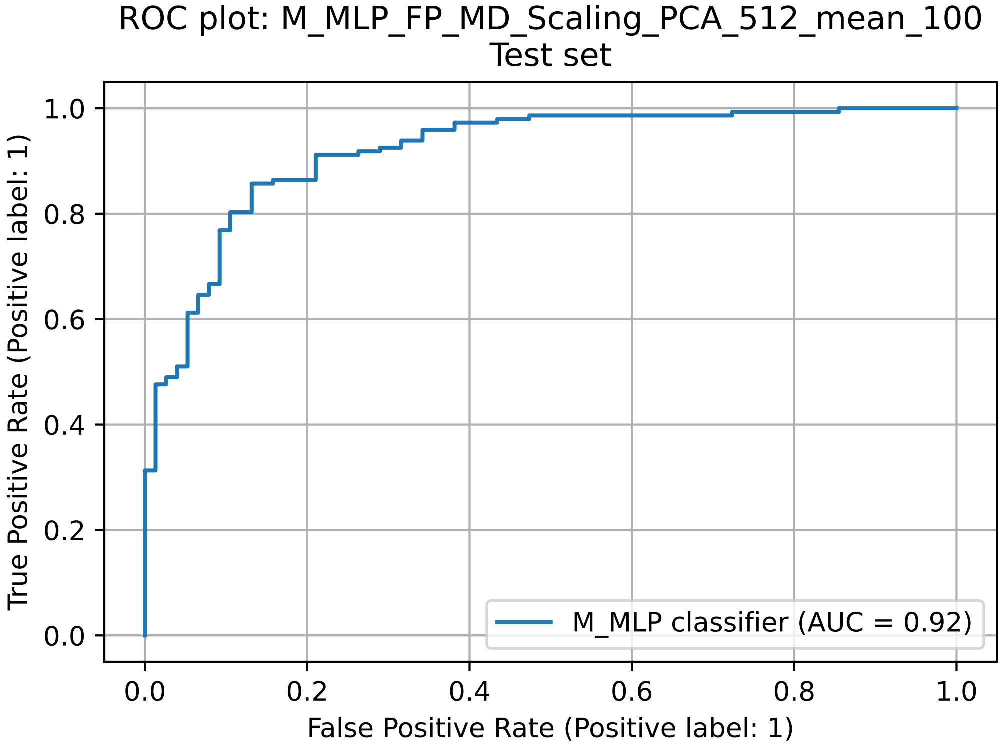
ROC plot: M_MLP_FP_MD_Scaling_PCA_512_mean_100
Test set
True Positive Rate (Positive label: 1)
False Positive Rate (Positive label: 1)
0.0
0.2
0.4
0.6
0.8
1.0
M_MLP classifier (AUC = 0.92)

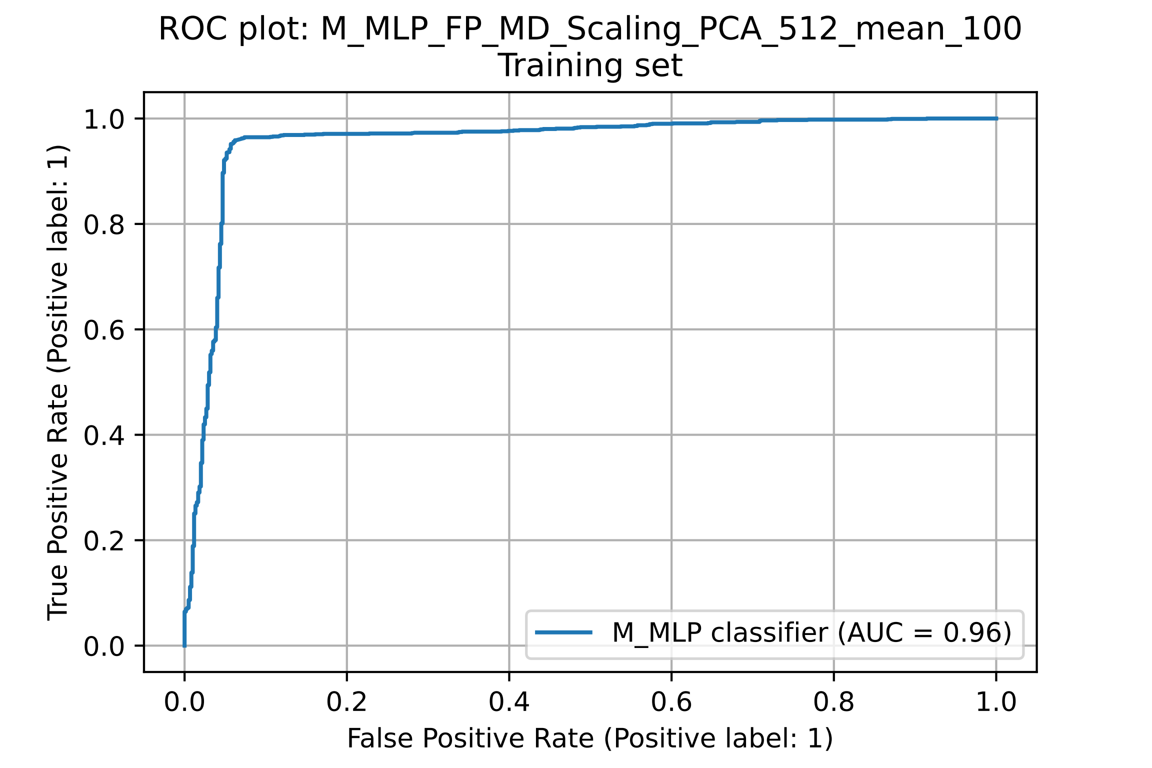

ROC plot: M_MLP_FP_MD_Scaling_PCA_512_mean_100
Training set
True Positive Rate (Positive label: 1)
False Positive Rate (Positive label: 1)
0.0
0.2
0.4
0.6
0.8
1.0
M_MLP classifier (AUC = 0.96)

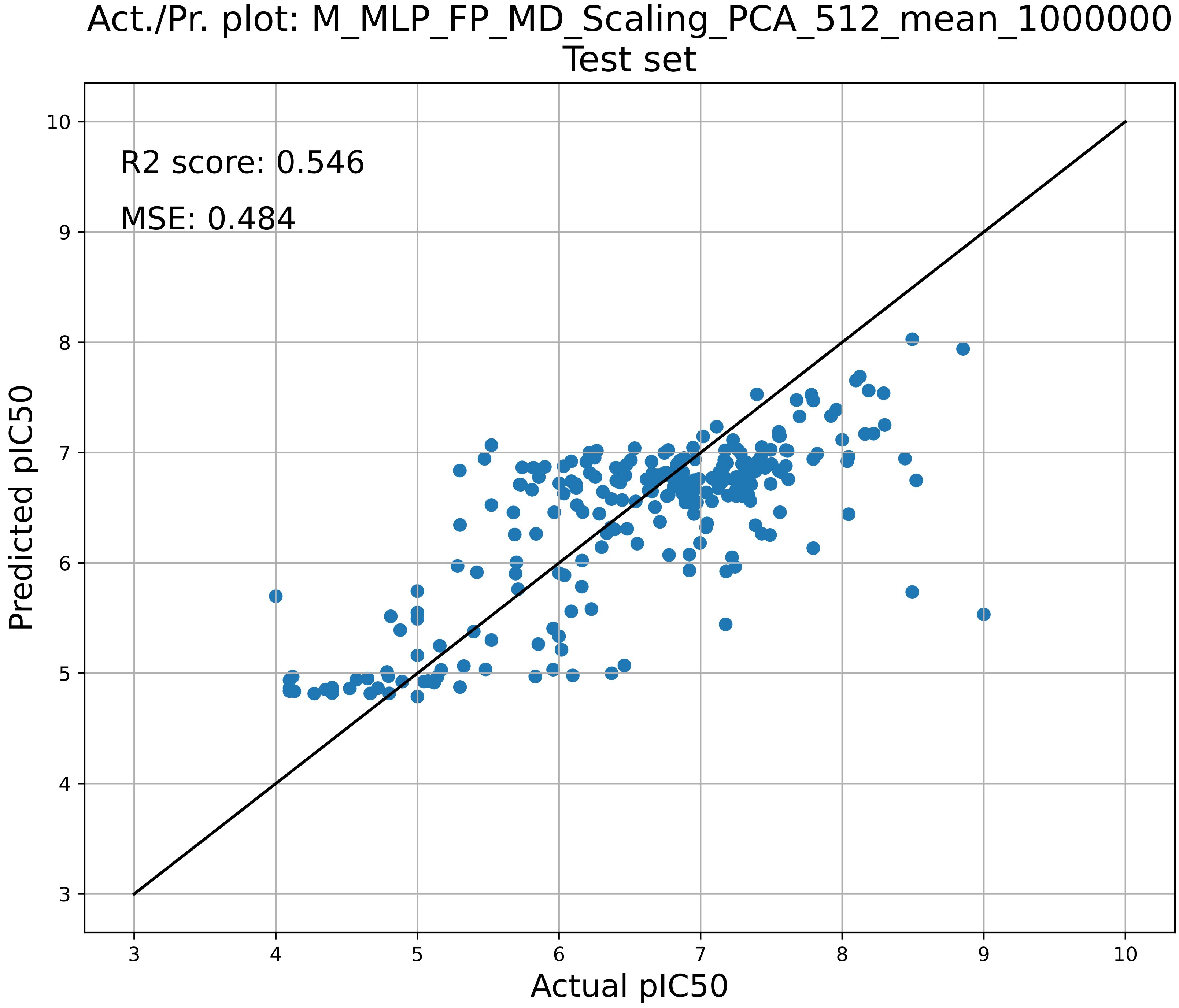

Act./Pr. plot: M_MLP_FP_MD_Scaling_PCA_512_mean_1000000
Test set
R2 score: 0.546
MSE: 0.484
Predicted pIC50
Actual pIC50
3
4
5
6
7
8
9
10

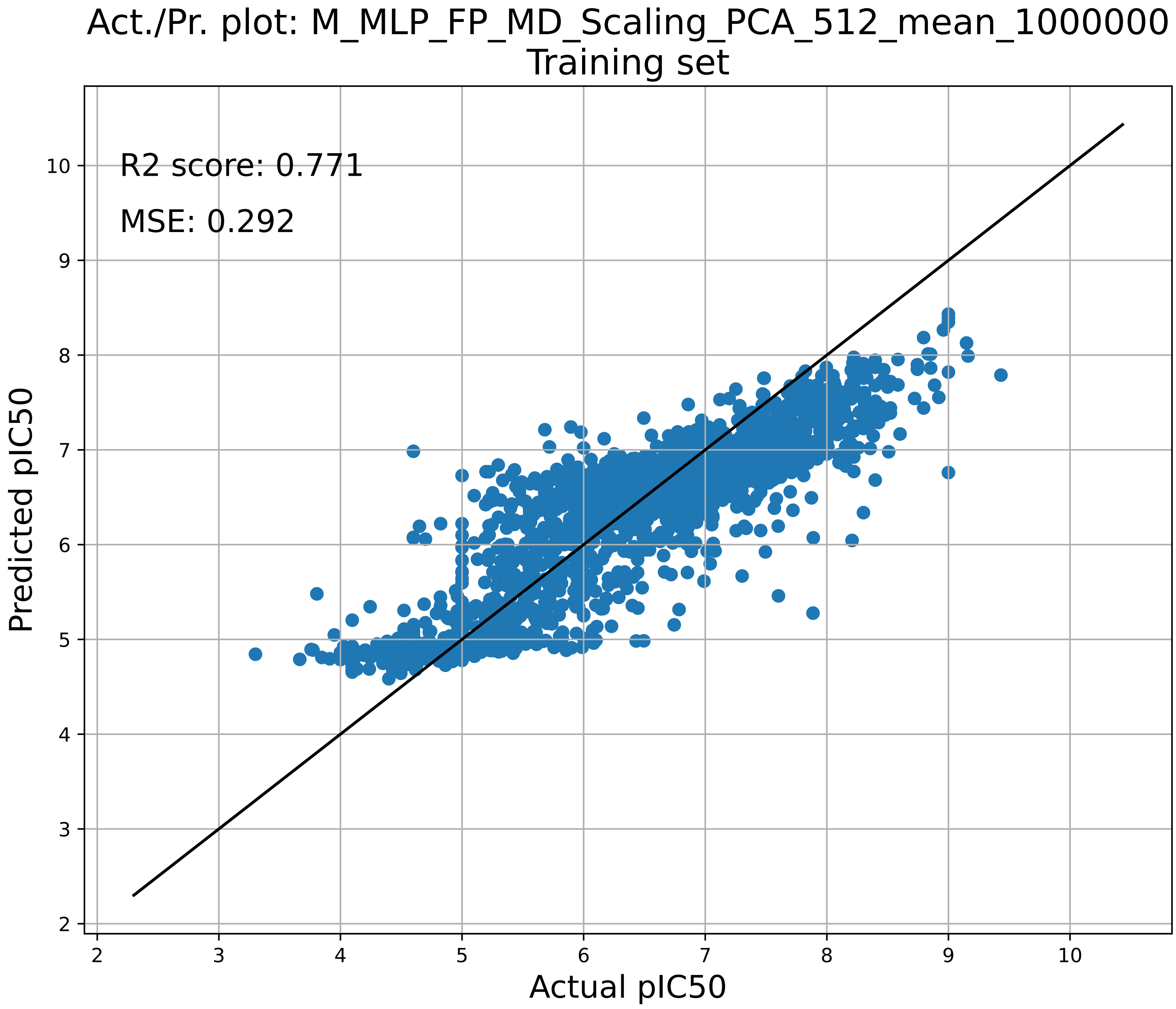

Act./Pr. plot: M_MLP_FP_MD_Scaling_PCA_512_mean_1000000
Training set
R2 score: 0.771
MSE: 0.292
Predicted pIC50
Actual pIC50
2
3
4
5
6
7
8
9
10

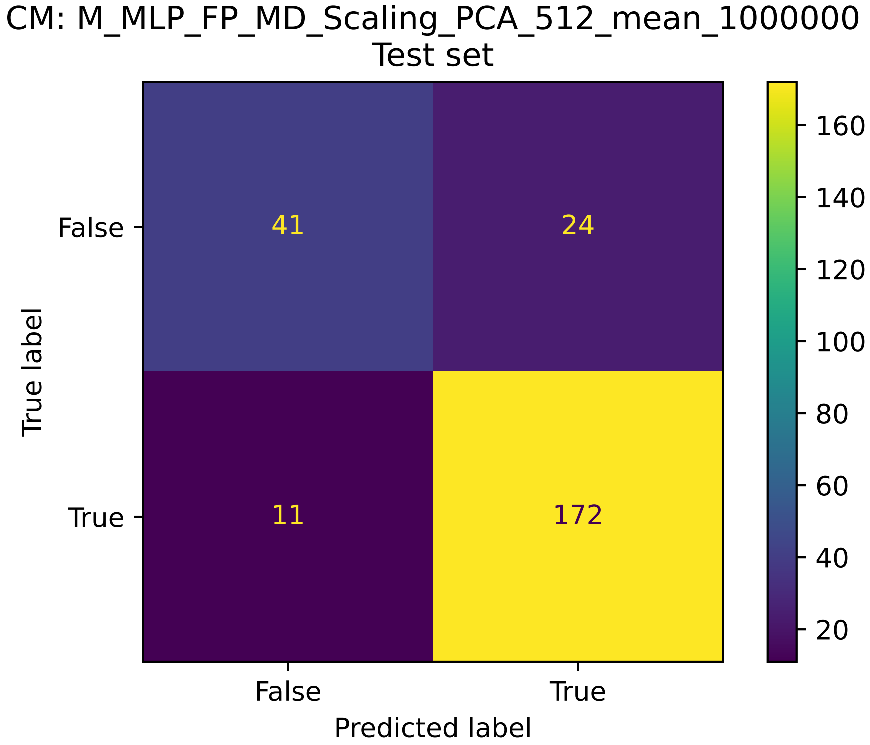
CM: M_MLP_FP_MD_Scaling_PCA_512_mean_1000000
Test set
True label
False
True
41
24
11
172
Predicted label
False
True
20
40
60
80
100
120
140
160

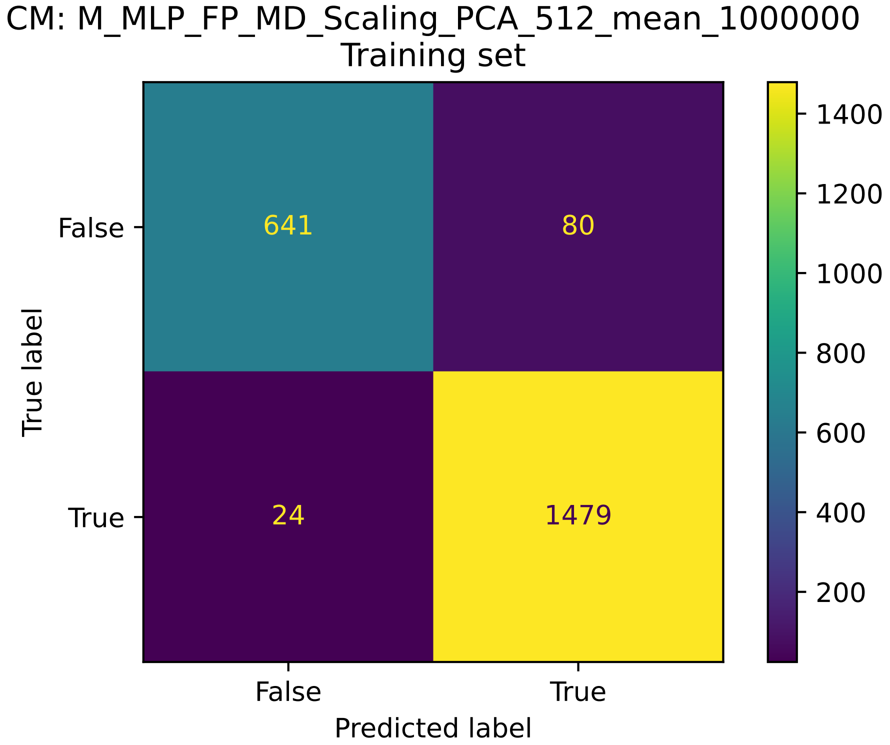

CM: M_MLP_FP_MD_Scaling_PCA_512_mean_1000000
Training set
641
80
24
1479
False
True
True label
False
True
Predicted label
1400
1200
1000
800
600
400
200

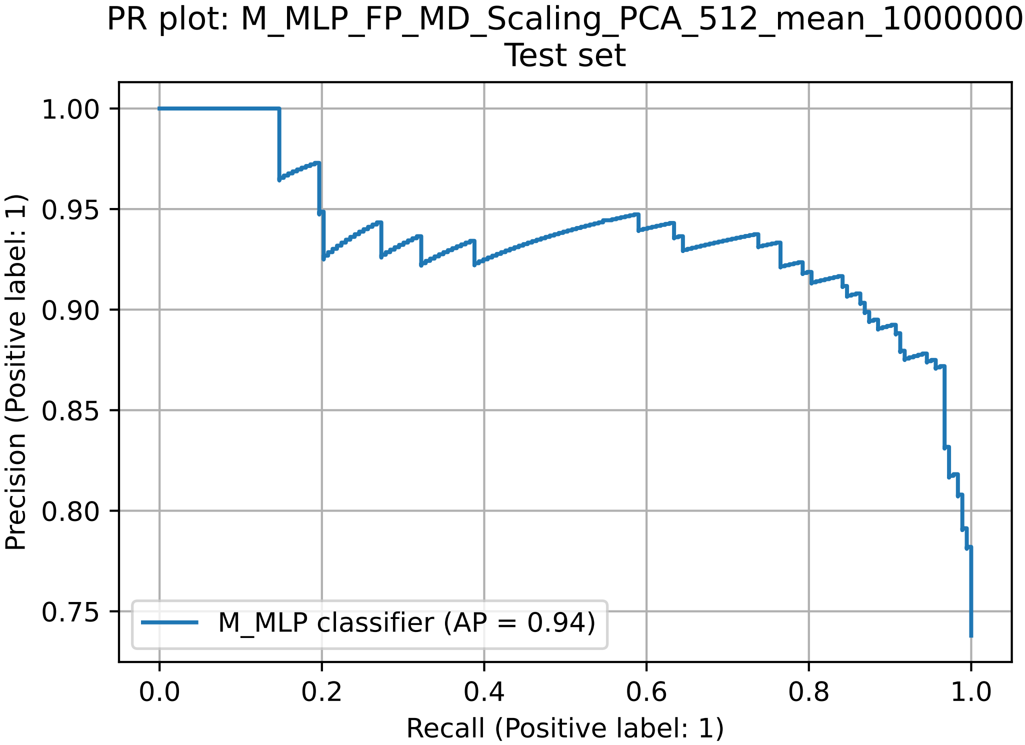

PR plot: M_MLP_FP_MD_Scaling_PCA_512_mean_1000000
Test set
Precision (Positive label: 1)
Recall (Positive label: 1)
1.00
0.95
0.90
0.85
0.80
0.75
0.0
0.2
0.4
0.6
0.8
1.0
M_MLP classifier (AP = 0.94)

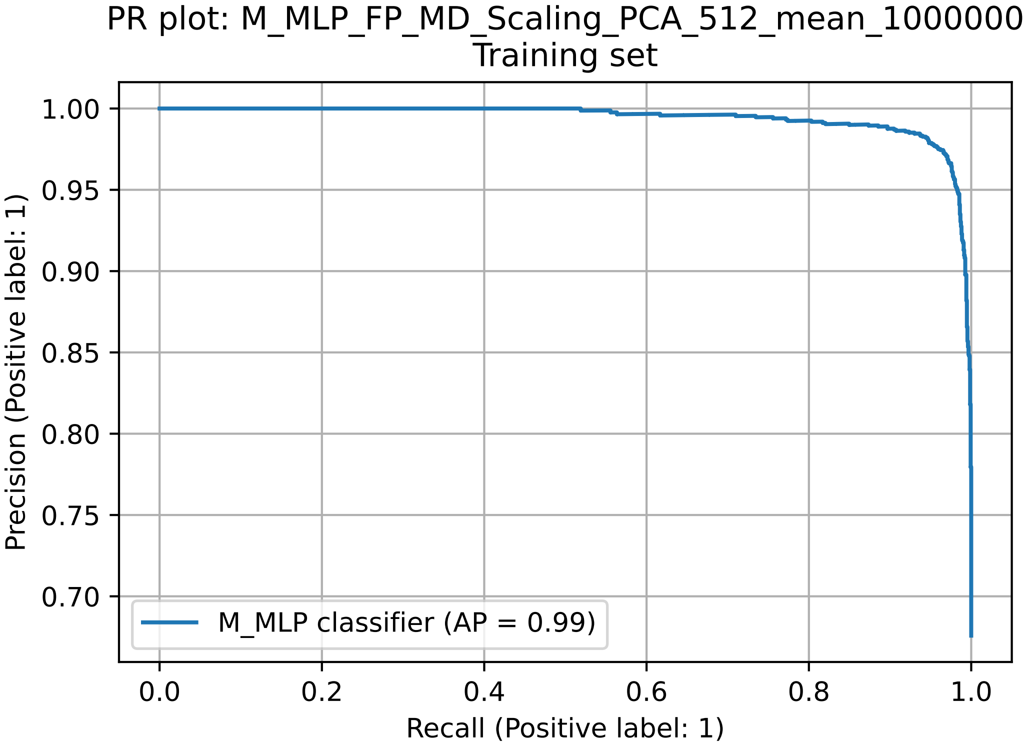

PR plot: M_MLP_FP_MD_Scaling_PCA_512_mean_1000000
Training set
Precision (Positive label: 1)
Recall (Positive label: 1)
1.00
0.95
0.90
0.85
0.80
0.75
0.70
0.0
0.2
0.4
0.6
0.8
1.0
M_MLP classifier (AP = 0.99)

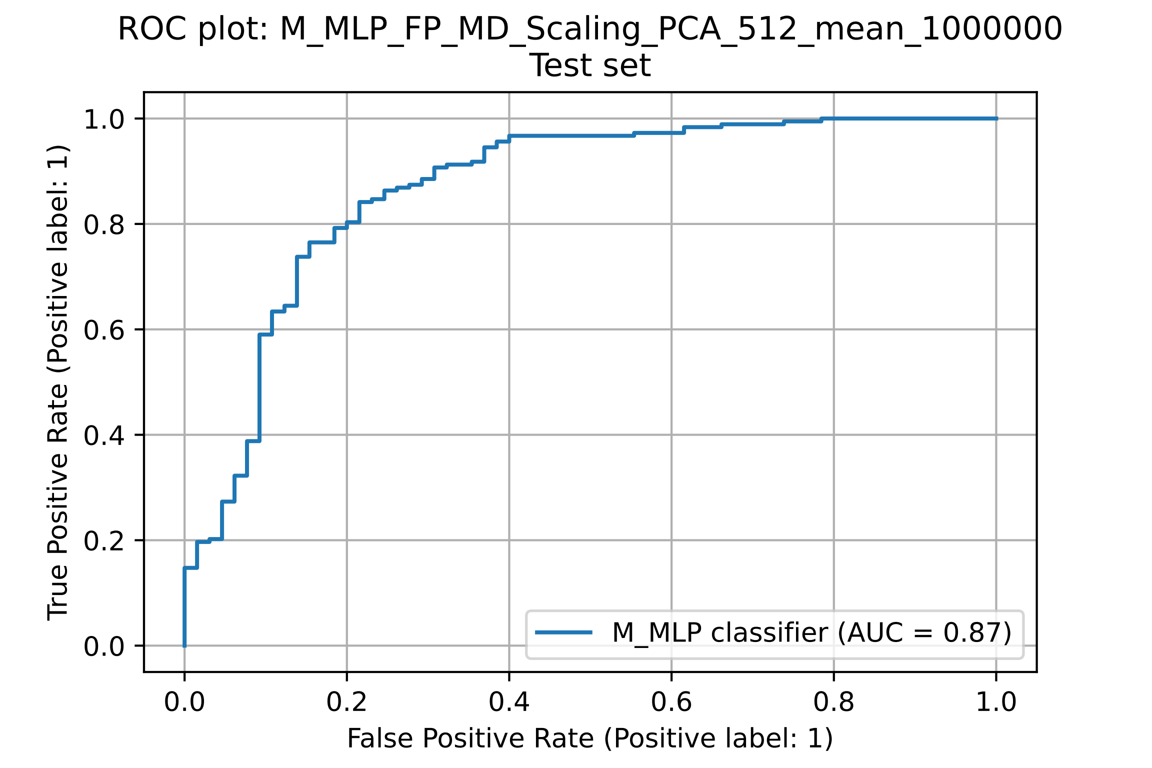
ROC plot: M_MLP_FP_MD_Scaling_PCA_512_mean_1000000
Test set
True Positive Rate (Positive label: 1)
False Positive Rate (Positive label: 1)
0.0
0.2
0.4
0.6
0.8
1.0
M_MLP classifier (AUC = 0.87)

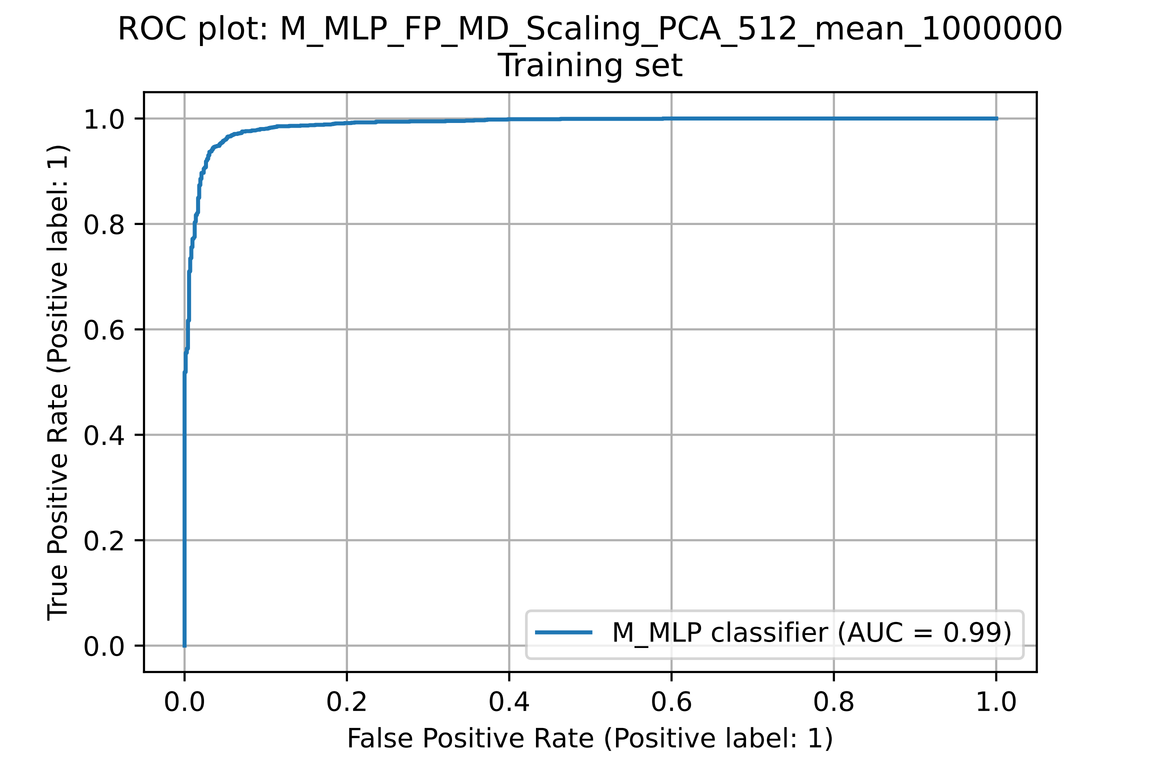
ROC plot: M_MLP_FP_MD_Scaling_PCA_512_mean_1000000
Training set
True Positive Rate (Positive label: 1)
False Positive Rate (Positive label: 1)
0.0
0.2
0.4
0.6
0.8
1.0
M_MLP classifier (AUC = 0.99)

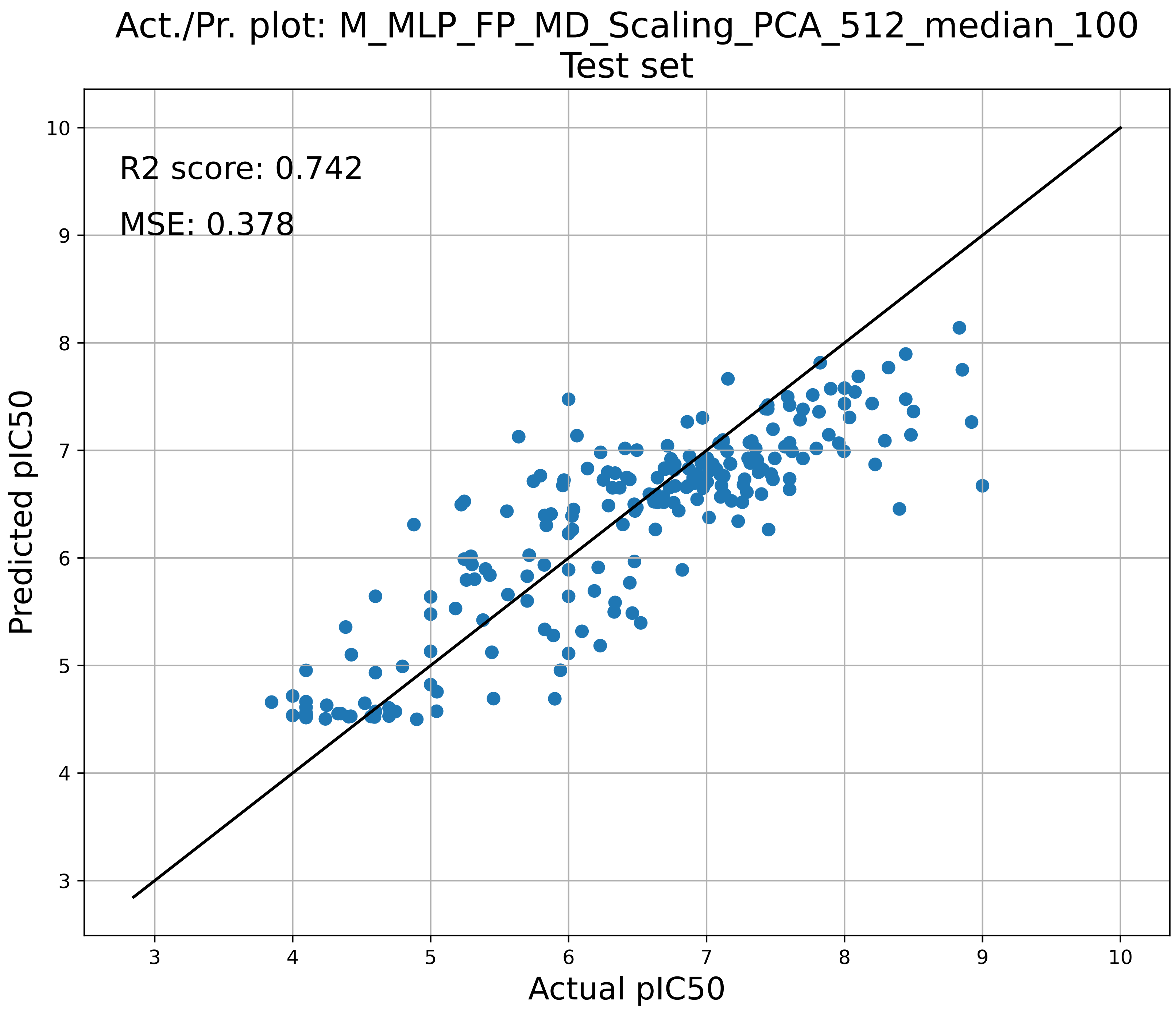

Act./Pr. plot: M_MLP_FP_MD_Scaling_PCA_512_median_100
Test set
R2 score: 0.742
MSE: 0.378
Predicted pIC50
Actual pIC50
3
4
5
6
7
8
9
10

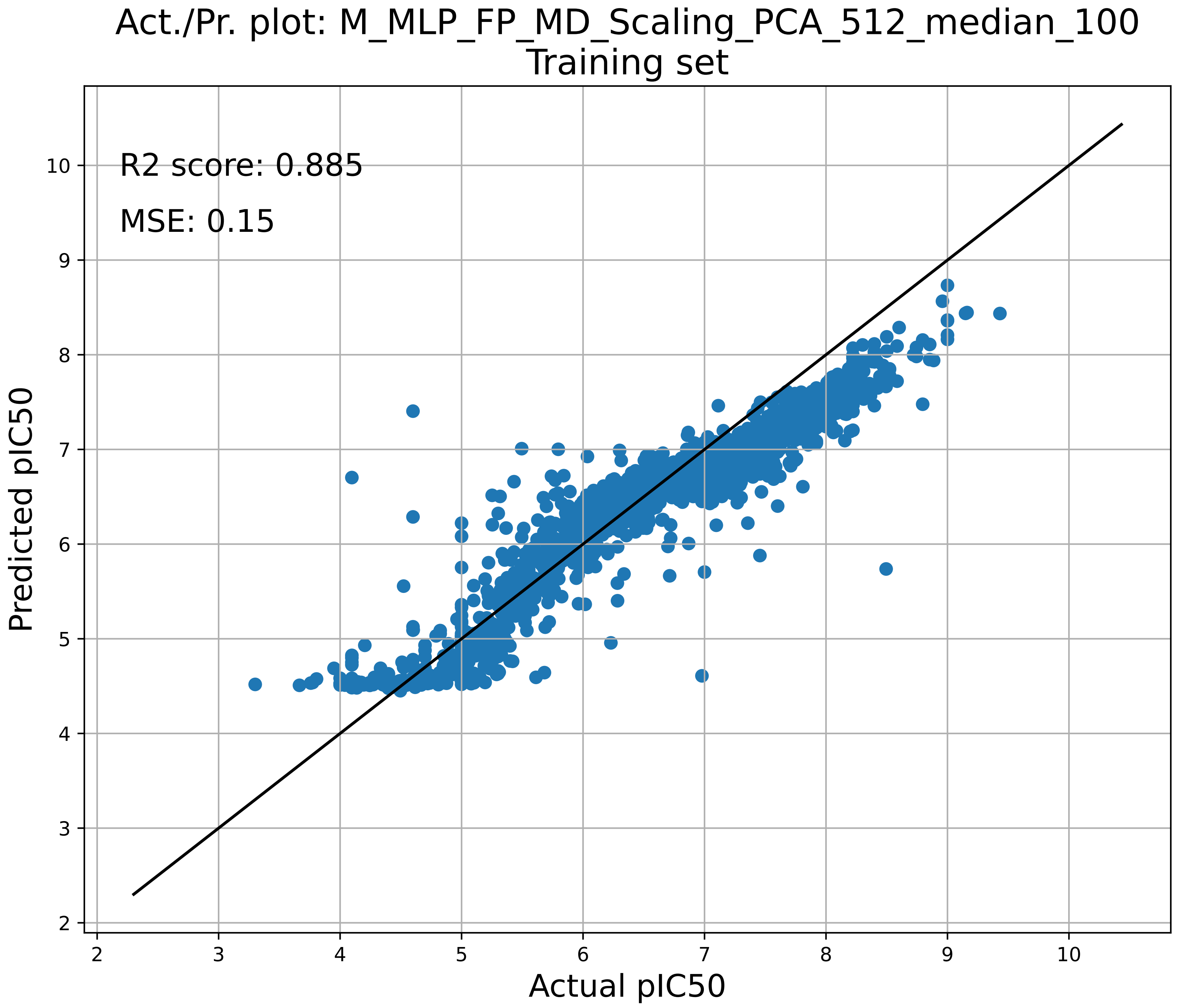

Act./Pr. plot: M_MLP_FP_MD_Scaling_PCA_512_median_100
Training set
R2 score: 0.885
MSE: 0.15
Predicted pIC50
Actual pIC50
2
3
4
5
6
7
8
9
10

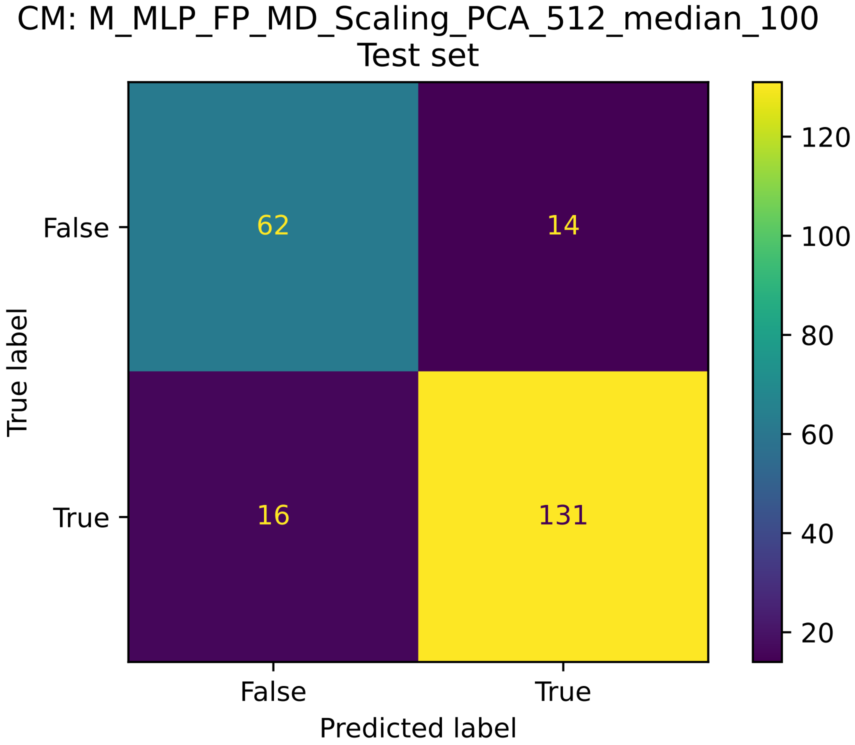
CM: M_MLP_FP_MD_Scaling_PCA_512_median_100
Test set
62
14
16
131
False
True
True label
False
True
Predicted label
120
100
80
60
40
20

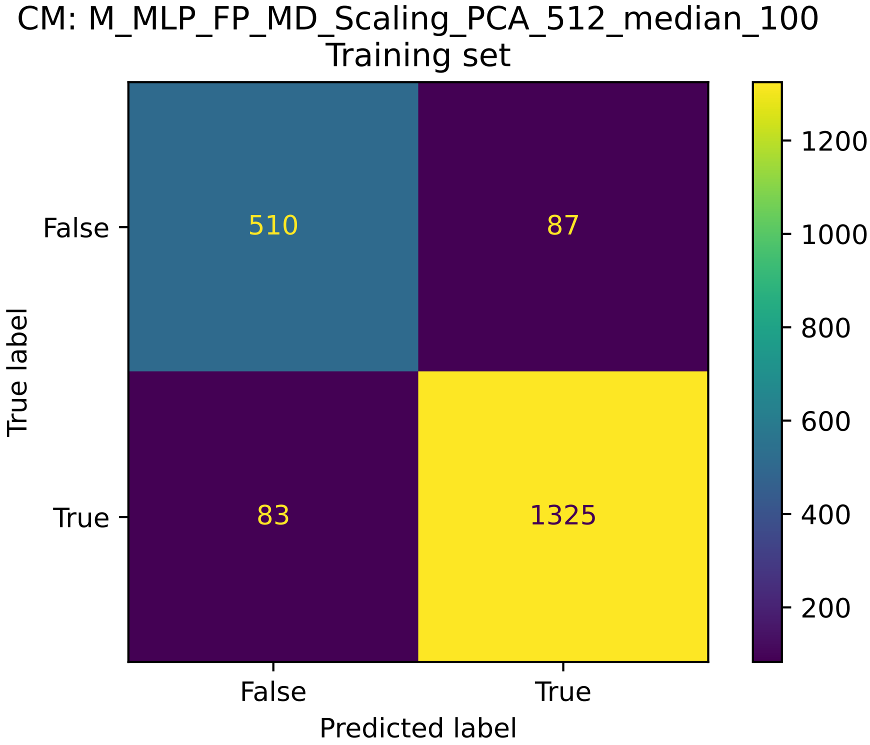
CM: M_MLP_FP_MD_Scaling_PCA_512_median_100
Training set
510
87
83
1325
False
True
True label
False
True
Predicted label
1200
1000
800
600
400
200

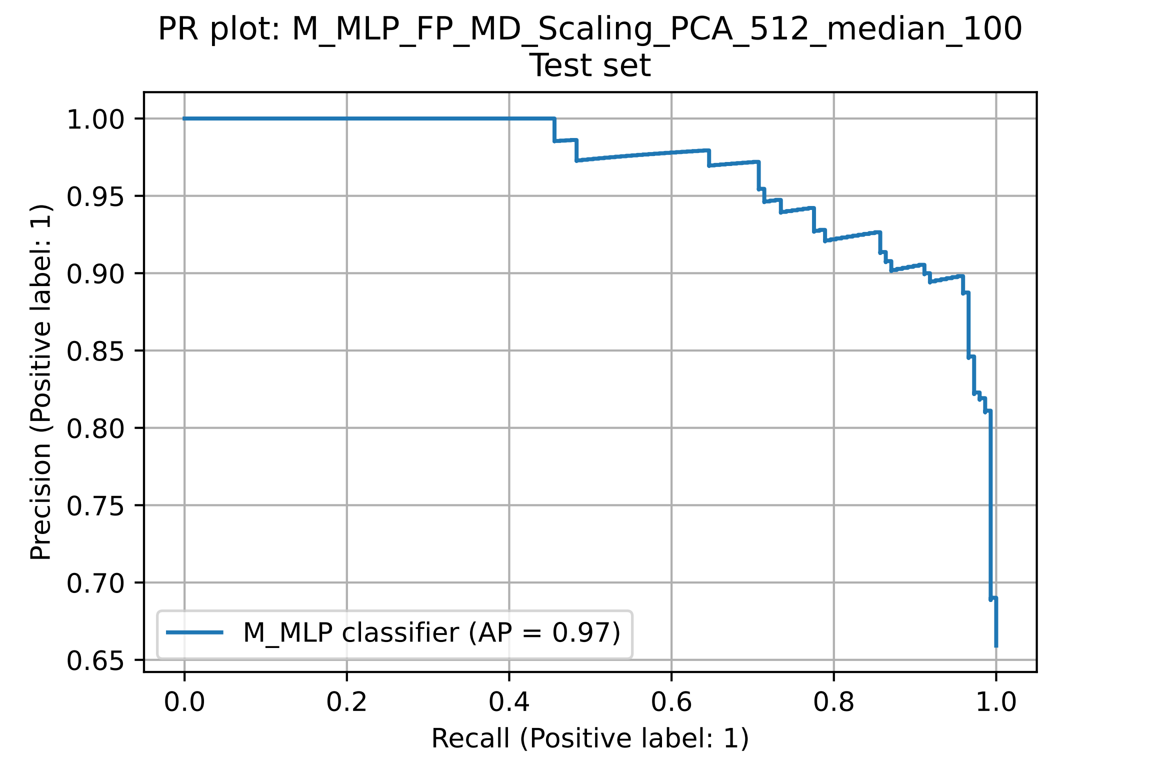
PR plot: M_MLP_FP_MD_Scaling_PCA_512_median_100
Test set
Precision (Positive label: 1)
Recall (Positive label: 1)
1.00
0.95
0.90
0.85
0.80
0.75
0.70
0.65
0.0
0.2
0.4
0.6
0.8
1.0
M_MLP classifier (AP = 0.97)

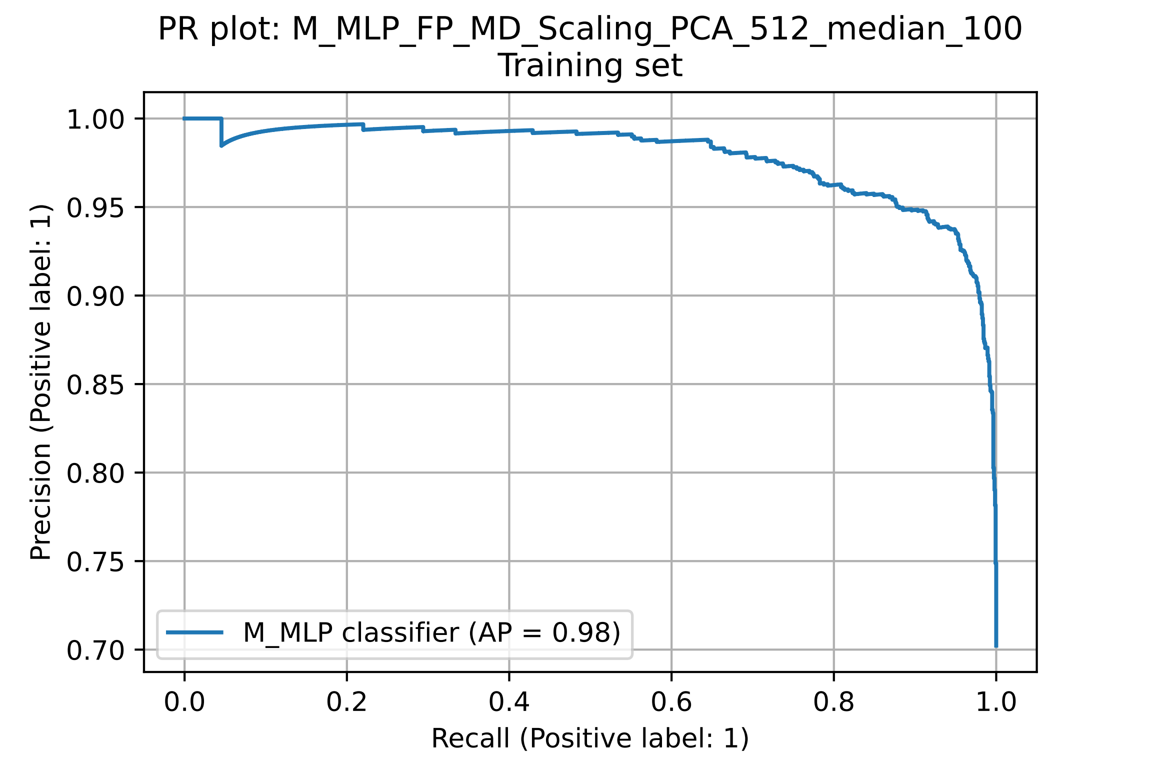

PR plot: M_MLP_FP_MD_Scaling_PCA_512_median_100
Training set
Precision (Positive label: 1)
Recall (Positive label: 1)
1.00
0.95
0.90
0.85
0.80
0.75
0.70
0.0
0.2
0.4
0.6
0.8
1.0
M_MLP classifier (AP = 0.98)

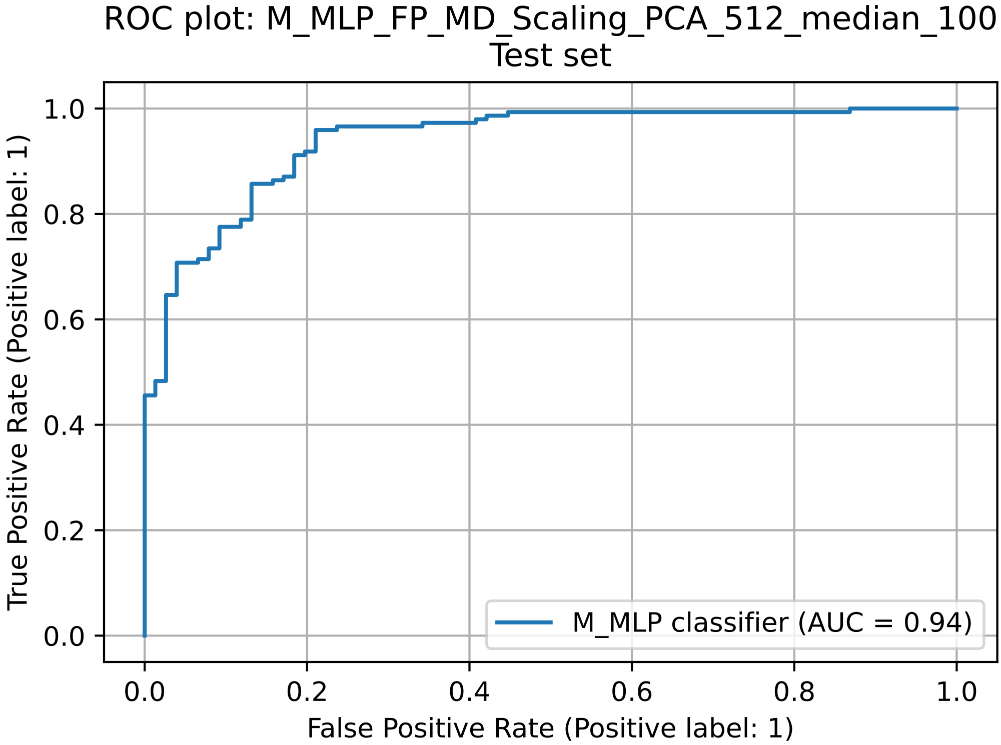
ROC plot: M_MLP_FP_MD_Scaling_PCA_512_median_100
Test set
True Positive Rate (Positive label: 1)
False Positive Rate (Positive label: 1)
0.0
0.2
0.4
0.6
0.8
1.0
M_MLP classifier (AUC = 0.94)

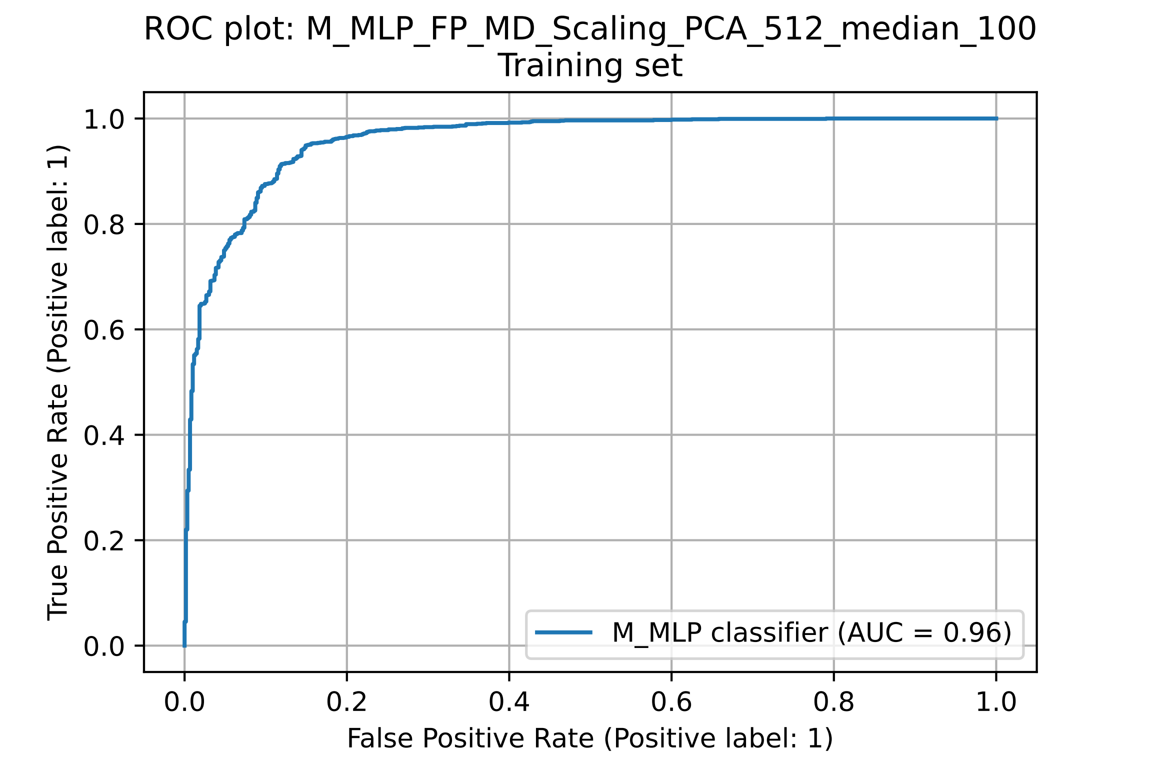

ROC plot: M_MLP_FP_MD_Scaling_PCA_512_median_100
Training set
True Positive Rate (Positive label: 1)
False Positive Rate (Positive label: 1)
0.0
0.2
0.4
0.6
0.8
1.0
M_MLP classifier (AUC = 0.96)

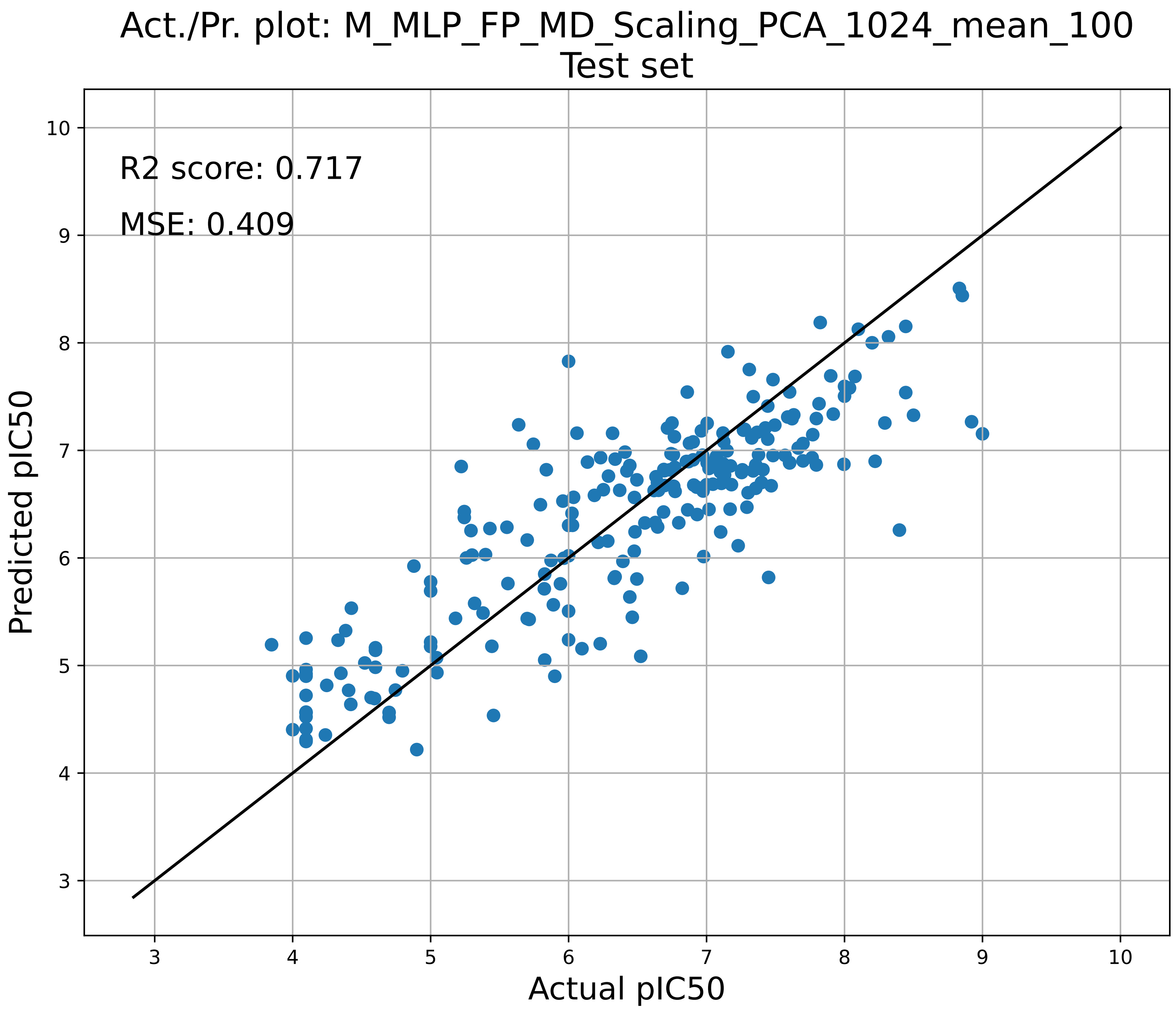
Act./Pr. plot: M_MLP_FP_MD_Scaling_PCA_1024_mean_100
Test set
R2 score: 0.717
MSE: 0.409
Predicted pIC50
Actual pIC50
3
4
5
6
7
8
9
10

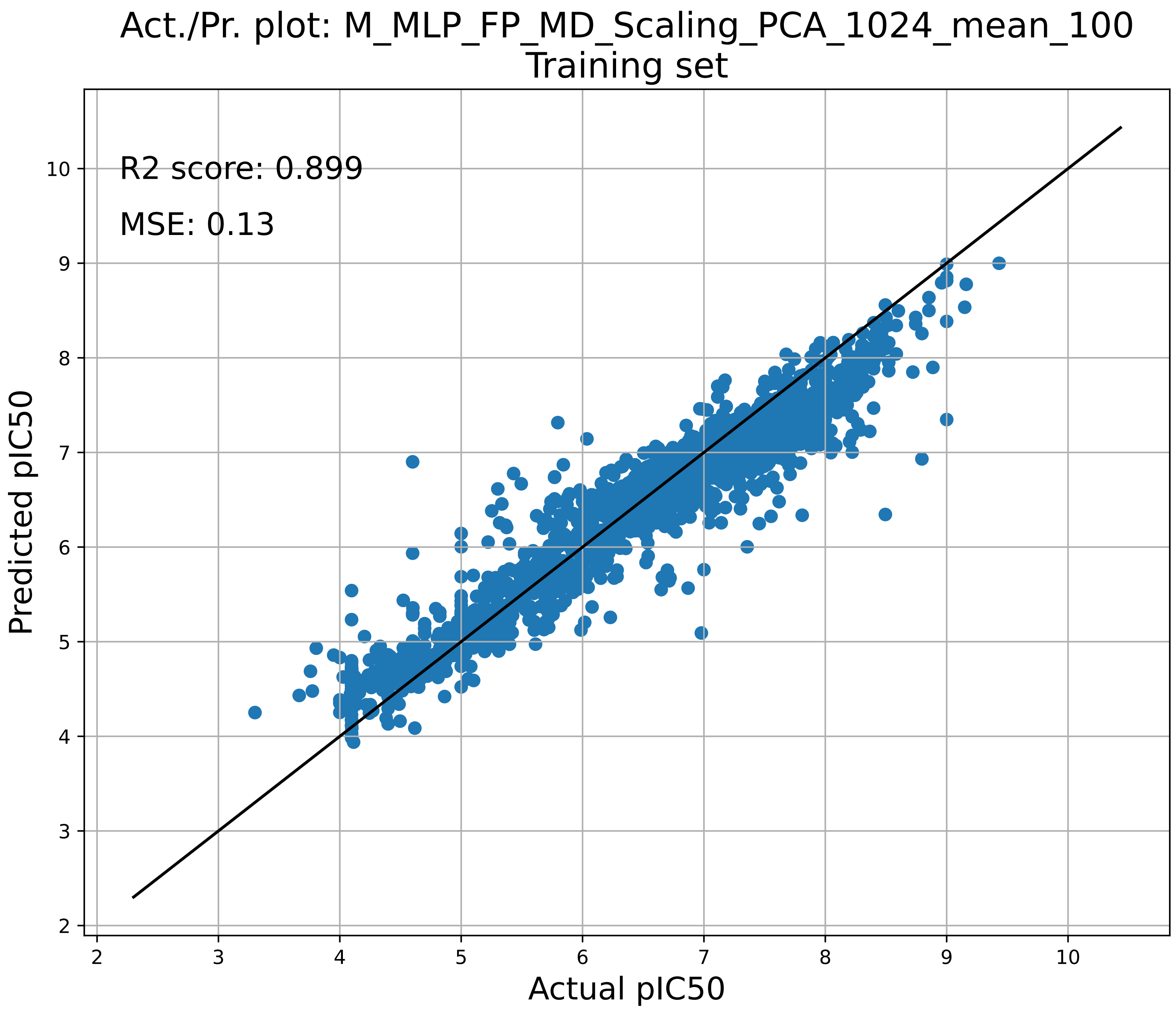
Act./Pr. plot: M_MLP_FP_MD_Scaling_PCA_1024_mean_100
Training set
R2 score: 0.899
MSE: 0.13
Predicted pIC50
Actual pIC50
2
3
4
5
6
7
8
9
10

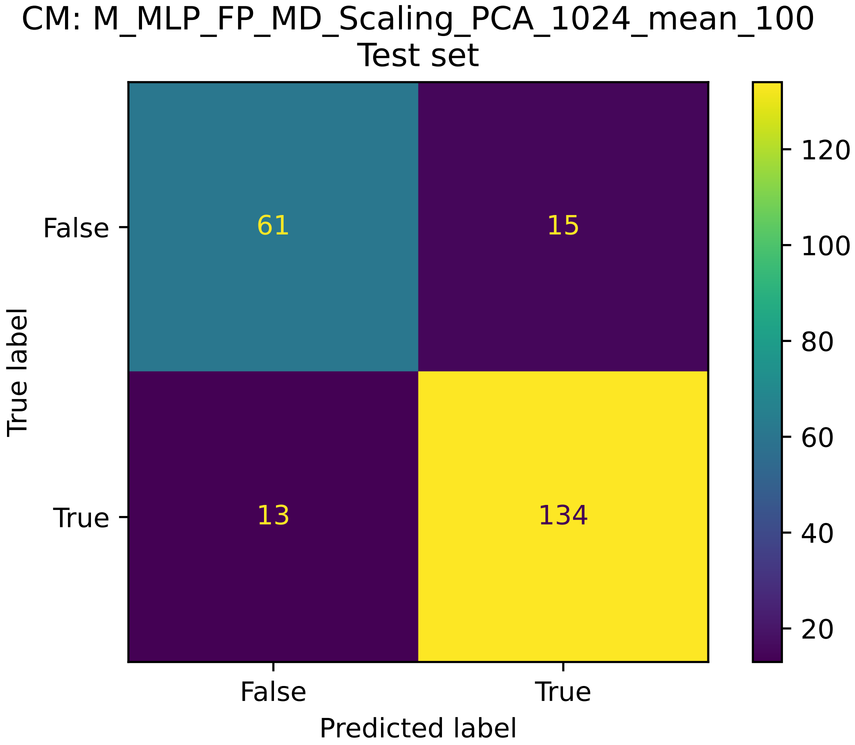
CM: M_MLP_FP_MD_Scaling_PCA_1024_mean_100
Test set
True label
False
True
61
15
13
134
Predicted label
False
True
20
40
60
80
100
120

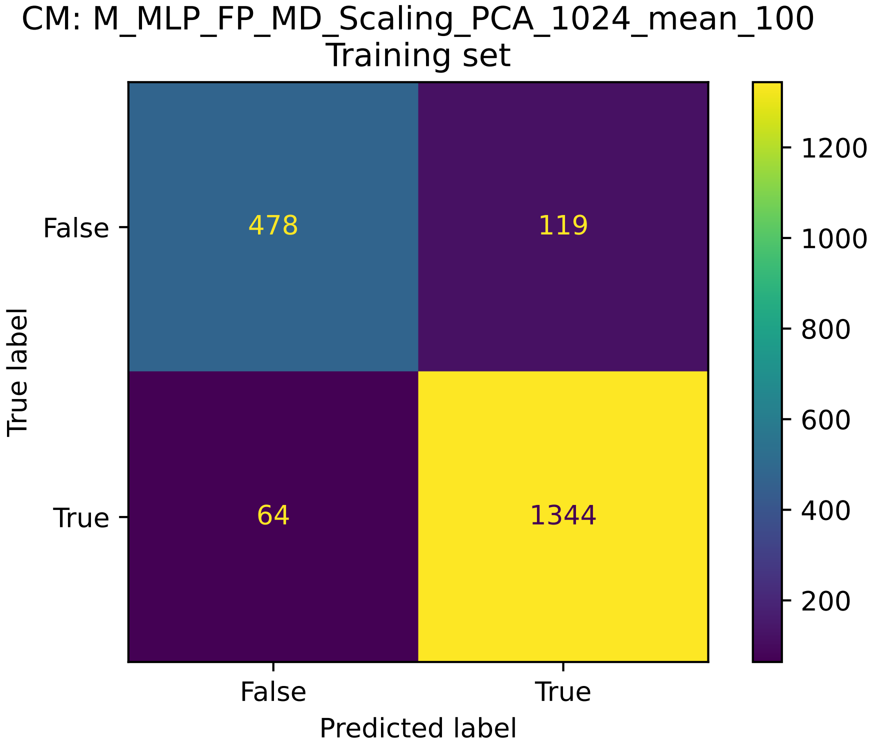
CM: M_MLP_FP_MD_Scaling_PCA_1024_mean_100
Training set
True label
Predicted label
False
True
False
True
478
119
64
1344
1200
1000
800
600
400
200

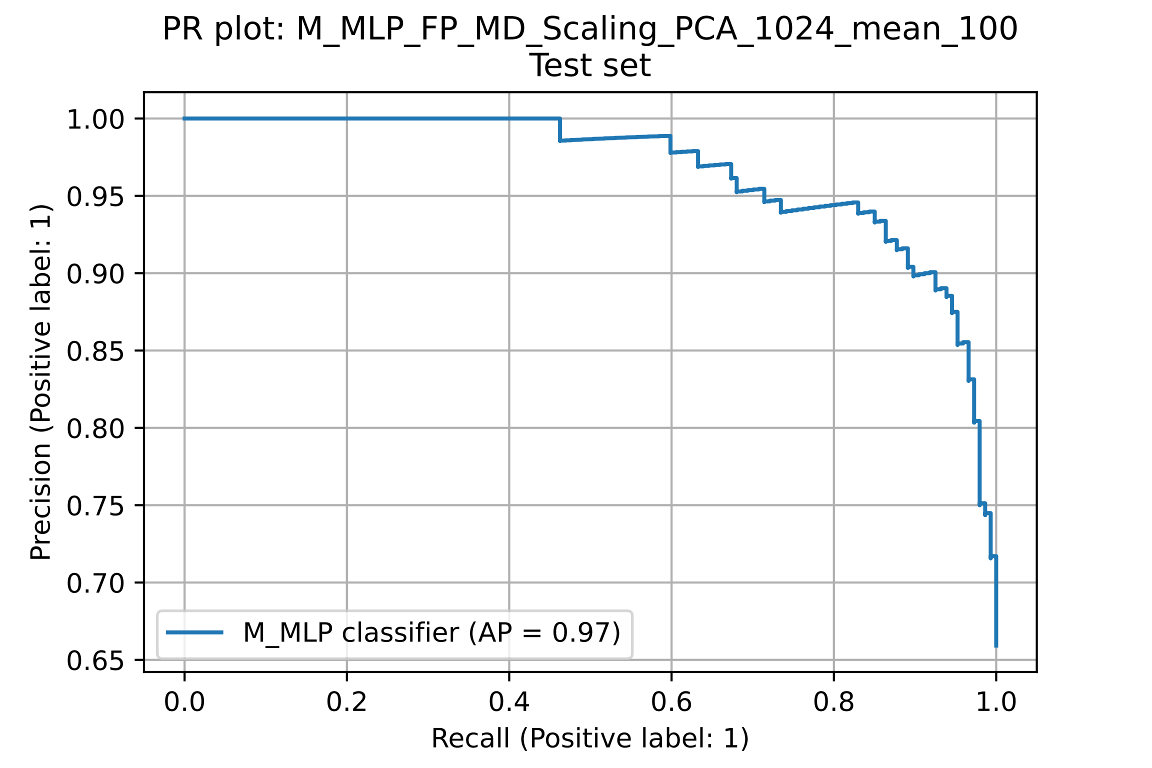
PR plot: M_MLP_FP_MD_Scaling_PCA_1024_mean_100
Test set
Precision (Positive label: 1)
Recall (Positive label: 1)
1.00
0.95
0.90
0.85
0.80
0.75
0.70
0.65
0.0
0.2
0.4
0.6
0.8
1.0
M_MLP classifier (AP = 0.97)

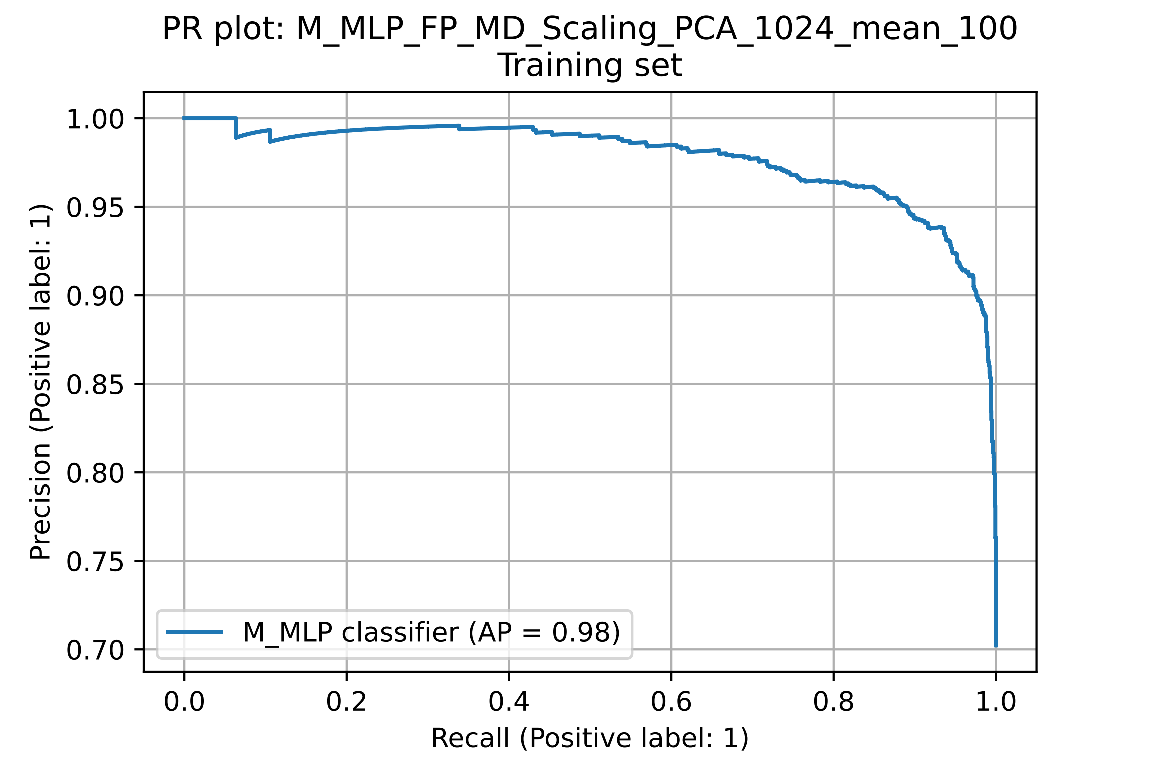
PR plot: M_MLP_FP_MD_Scaling_PCA_1024_mean_100
Training set
Precision (Positive label: 1)
Recall (Positive label: 1)
M_MLP classifier (AP = 0.98)
1.00
0.95
0.90
0.85
0.80
0.75
0.70
0.0
0.2
0.4
0.6
0.8
1.0

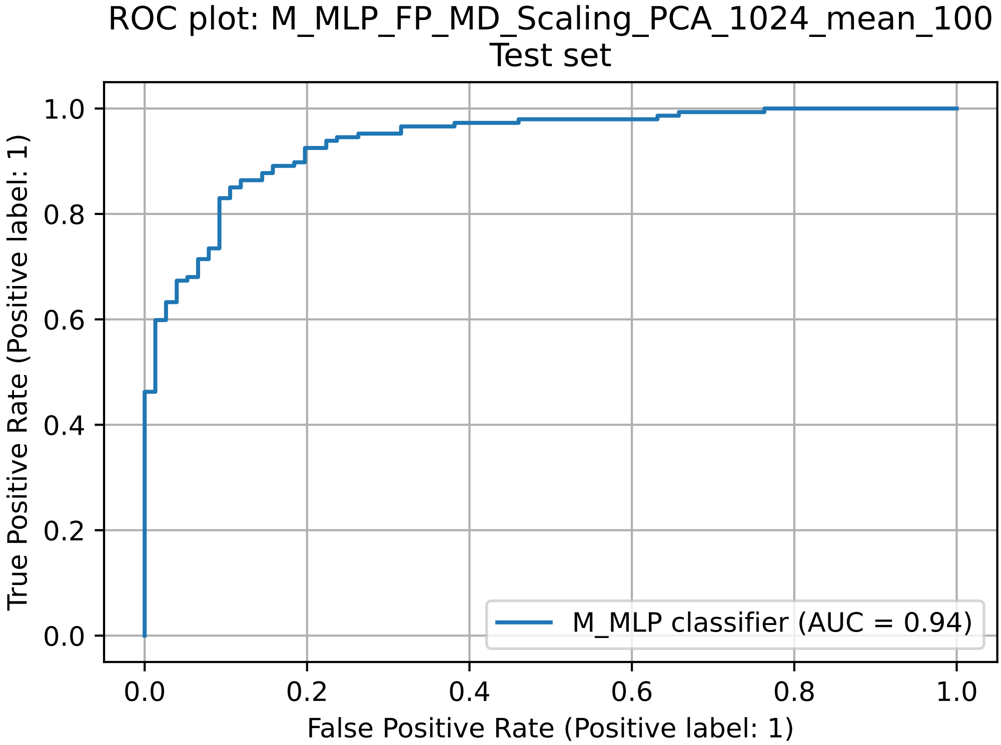
ROC plot: M_MLP_FP_MD_Scaling_PCA_1024_mean_100
Test set
True Positive Rate (Positive label: 1)
False Positive Rate (Positive label: 1)
0.0
0.2
0.4
0.6
0.8
1.0
M_MLP classifier (AUC = 0.94)

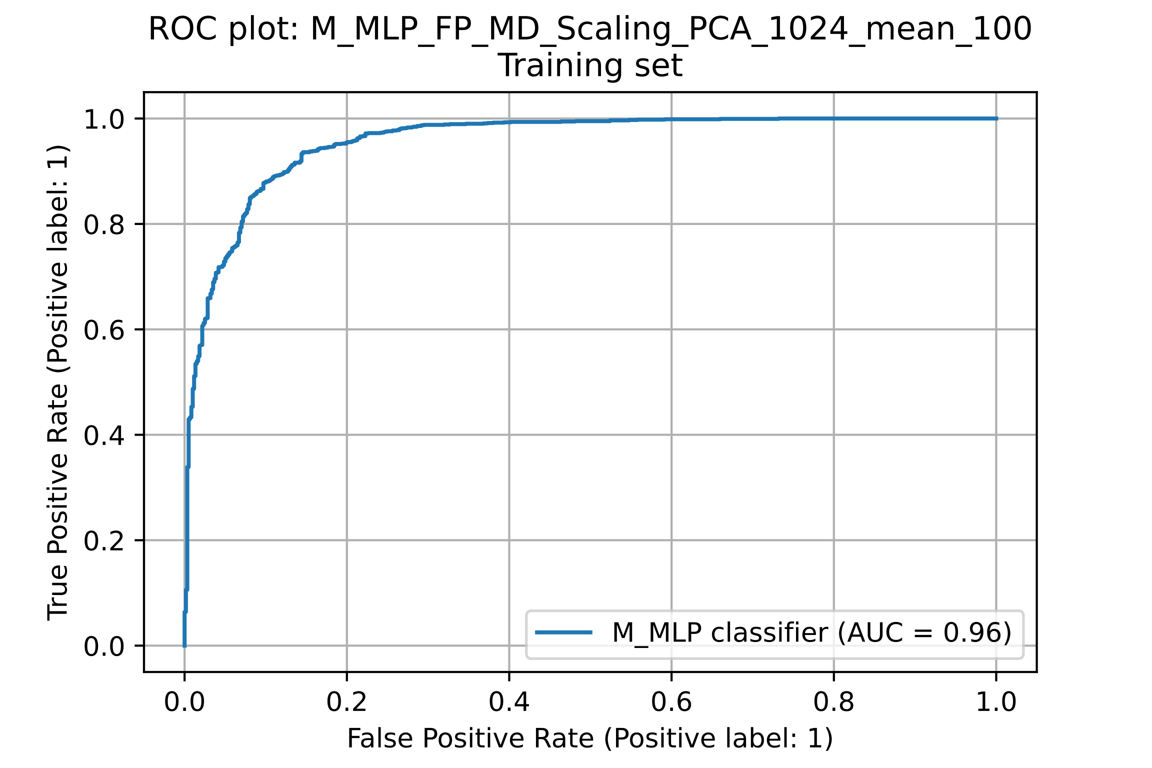
ROC plot: M_MLP_FP_MD_Scaling_PCA_1024_mean_100
Training set
True Positive Rate (Positive label: 1)
False Positive Rate (Positive label: 1)
0.0
0.2
0.4
0.6
0.8
1.0
M_MLP classifier (AUC = 0.96)

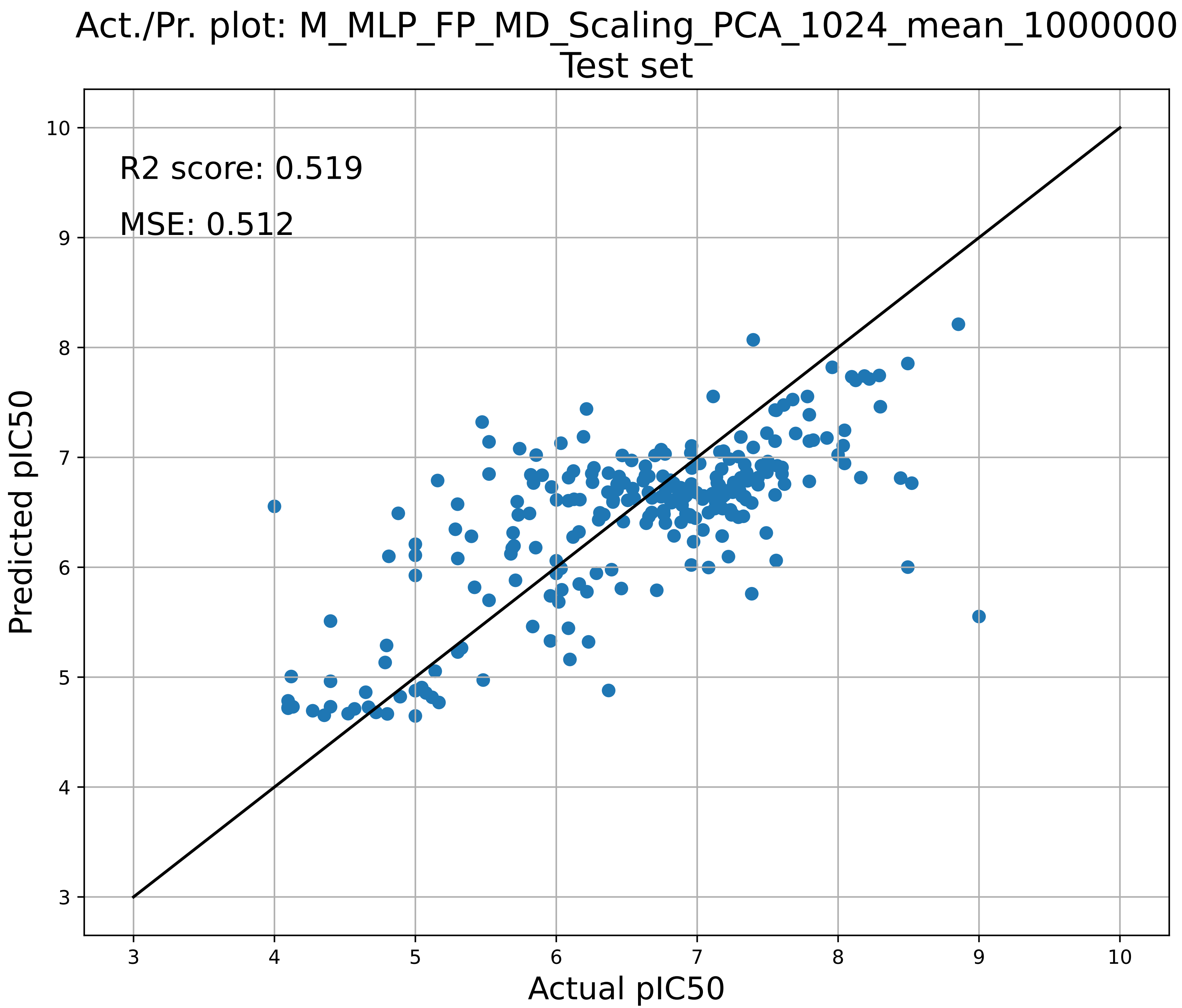
Act./Pr. plot: M_MLP_FP_MD_Scaling_PCA_1024_mean_1000000
Test set
R2 score: 0.519
MSE: 0.512
Predicted pIC50
Actual pIC50
3
4
5
6
7
8
9
10

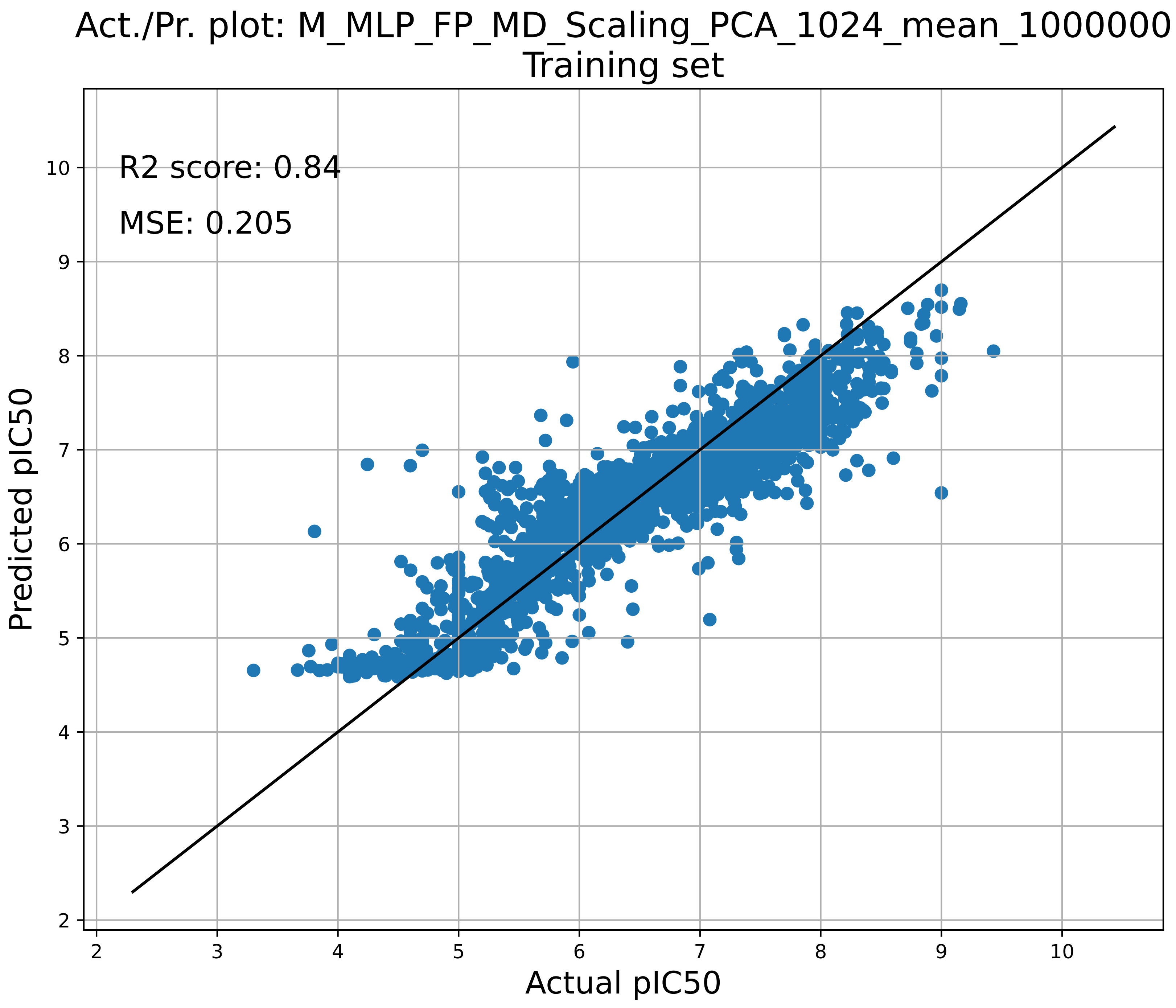

Act./Pr. plot: M_MLP_FP_MD_Scaling_PCA_1024_mean_1000000
Training set
R2 score: 0.84
MSE: 0.205
Predicted pIC50
Actual pIC50
2
3
4
5
6
7
8
9
10

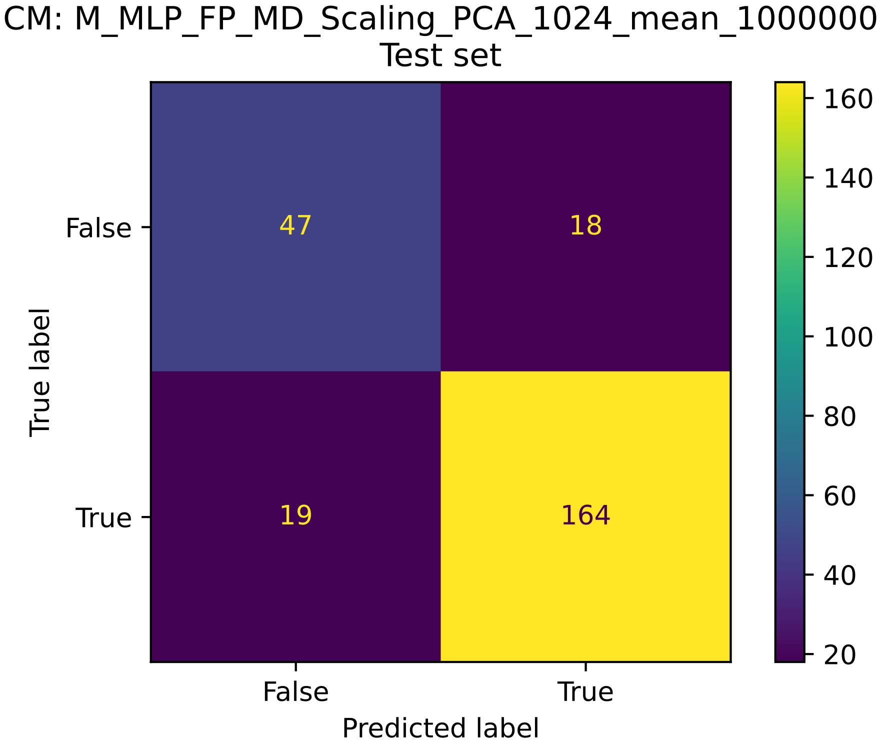
CM: M_MLP_FP_MD_Scaling_PCA_1024_mean_1000000
Test set
True label
Predicted label
False
True
False
True
47
18
19
164
160
140
120
100
80
60
40
20

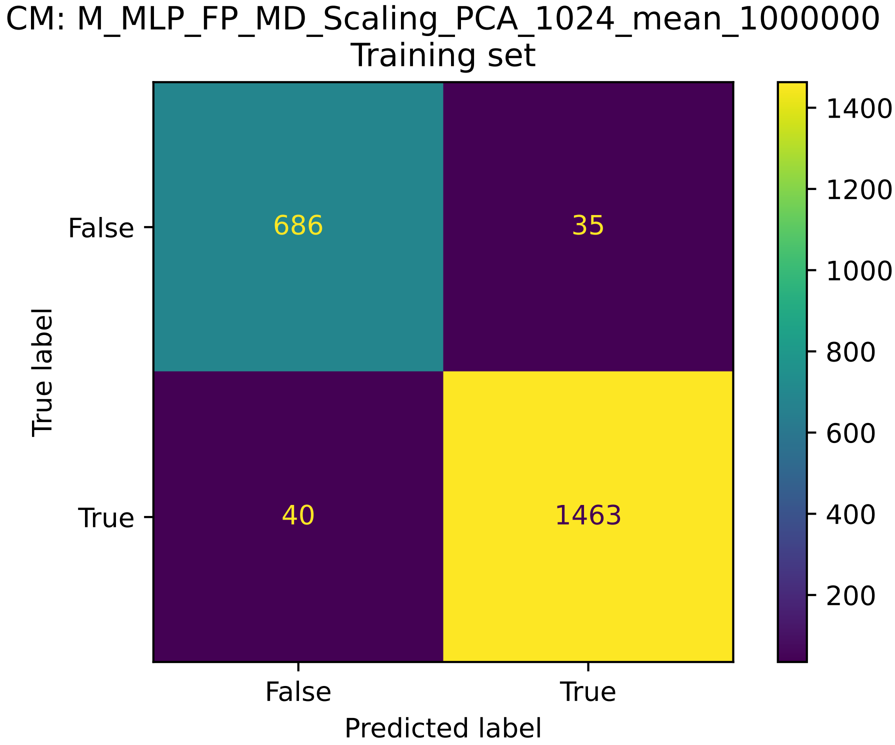

CM: M_MLP_FP_MD_Scaling_PCA_1024_mean_1000000
Training set
True label
Predicted label
False
True
False
True
686
35
40
1463
1400
1200
1000
800
600
400
200

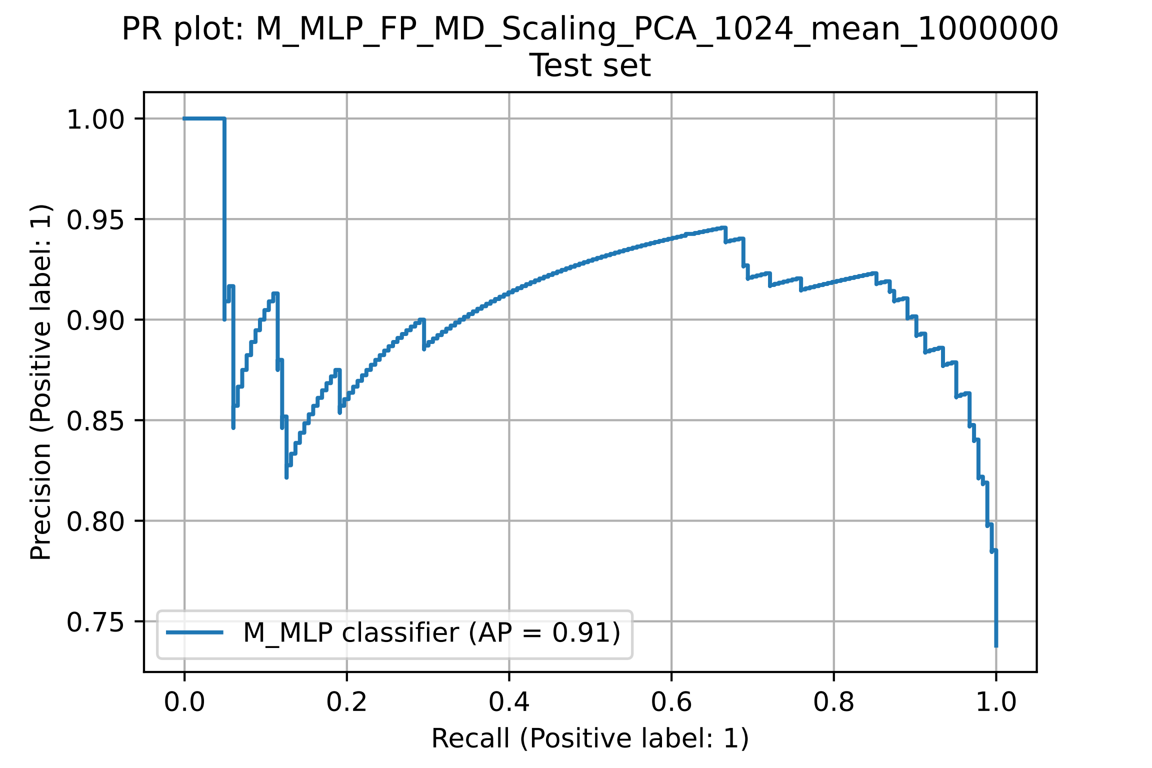

PR plot: M_MLP_FP_MD_Scaling_PCA_1024_mean_1000000
Test set
1.00
0.95
0.90
0.85
0.80
0.75
Precision (Positive label: 1)
0.0
0.2
0.4
0.6
0.8
1.0
Recall (Positive label: 1)
M_MLP classifier (AP = 0.91)

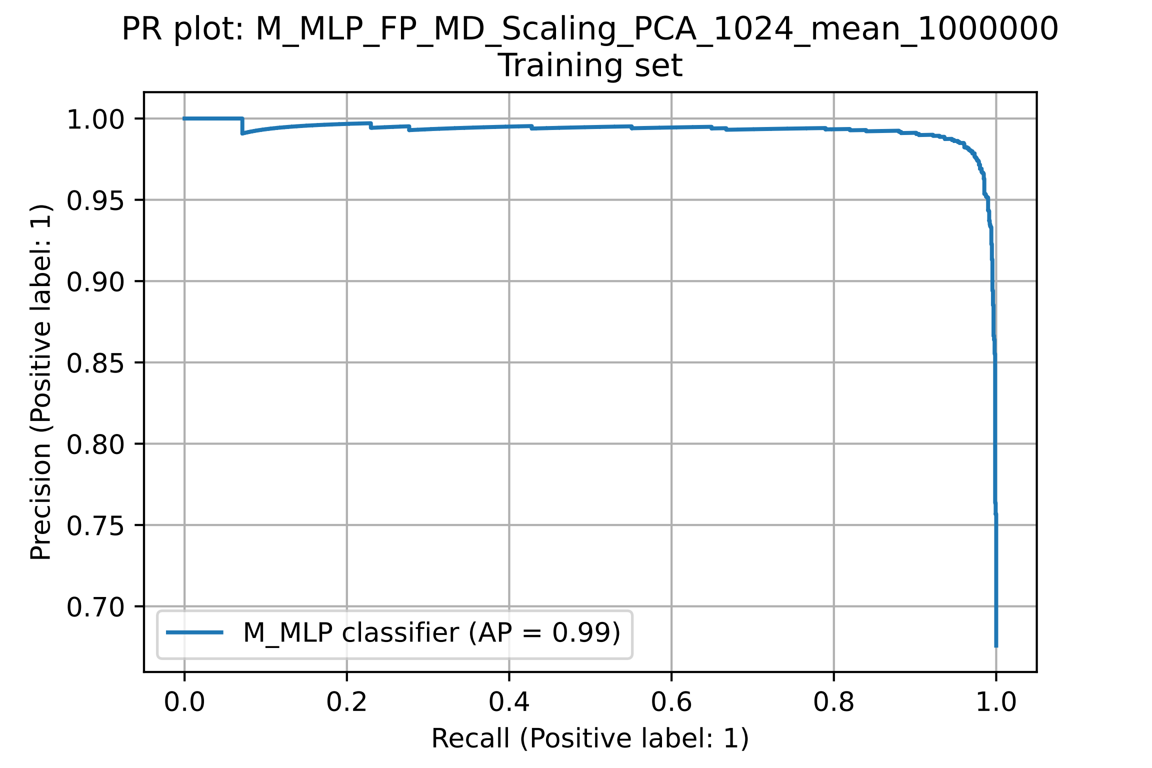

PR plot: M_MLP_FP_MD_Scaling_PCA_1024_mean_1000000
Training set
1.00
0.95
0.90
0.85
0.80
0.75
0.70
Precision (Positive label: 1)
0.0
0.2
0.4
0.6
0.8
1.0
Recall (Positive label: 1)
M_MLP classifier (AP = 0.99)

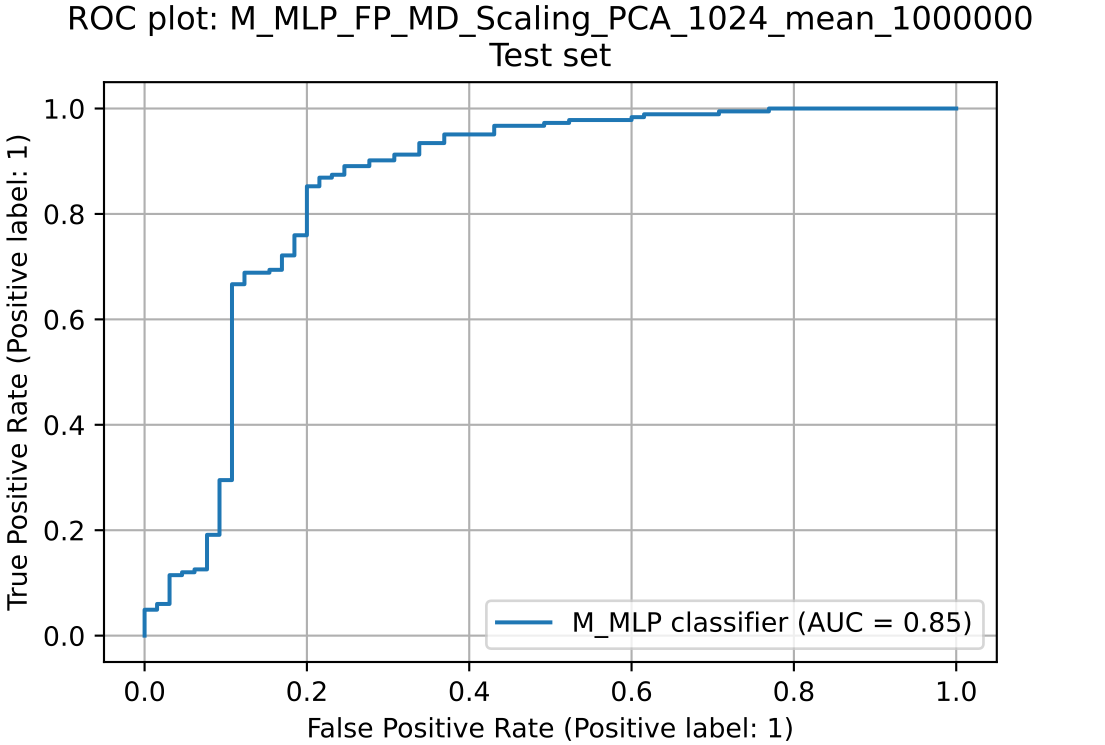
ROC plot: M_MLP_FP_MD_Scaling_PCA_1024_mean_1000000
Test set
True Positive Rate (Positive label: 1)
False Positive Rate (Positive label: 1)
0.0
0.2
0.4
0.6
0.8
1.0
M_MLP classifier (AUC = 0.85)

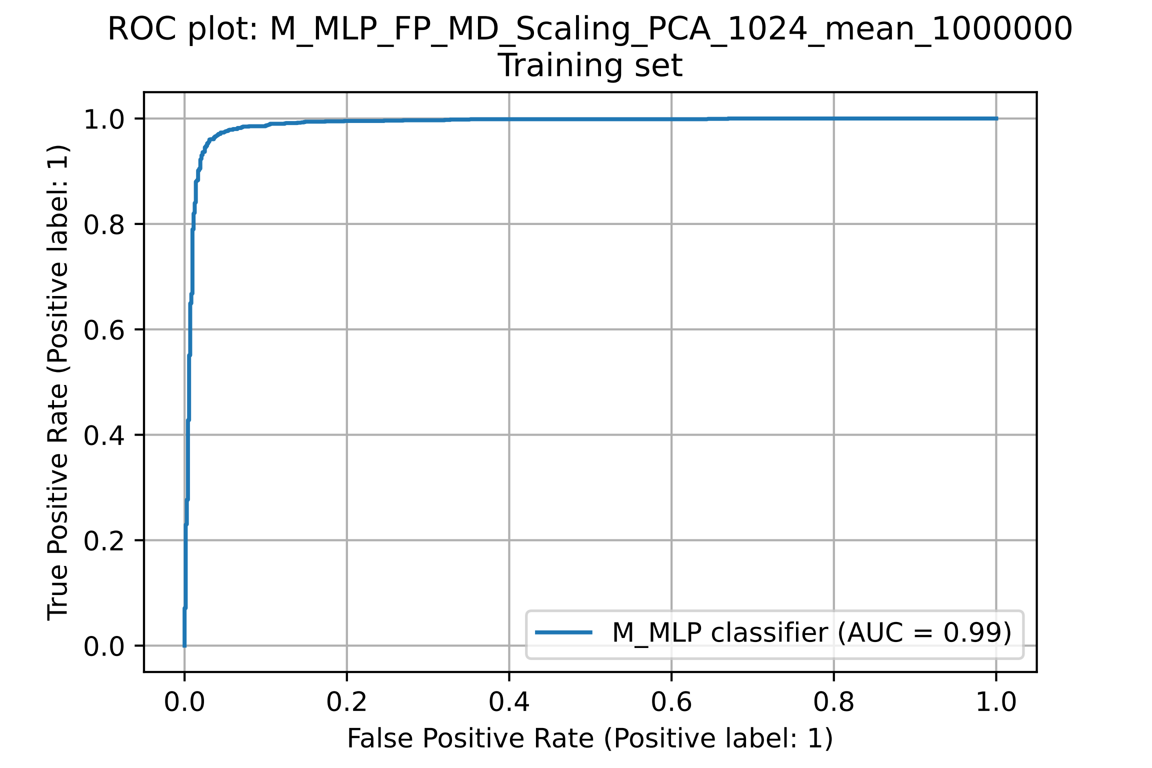
ROC plot: M_MLP_FP_MD_Scaling_PCA_1024_mean_1000000
Training set
True Positive Rate (Positive label: 1)
False Positive Rate (Positive label: 1)
0.0
0.2
0.4
0.6
0.8
1.0
M_MLP classifier (AUC = 0.99)

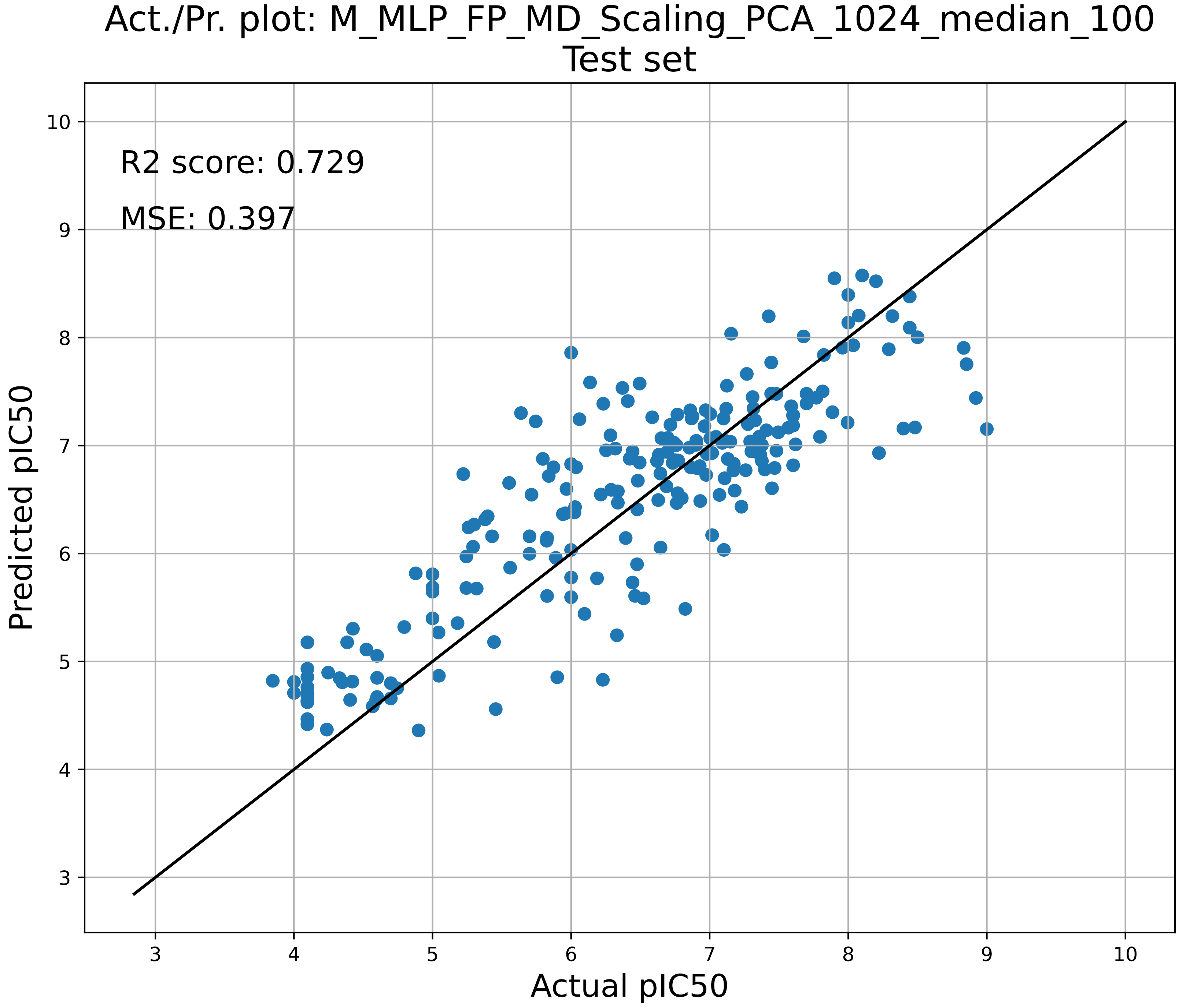
Act./Pr. plot: M_MLP_FP_MD_Scaling_PCA_1024_median_100
Test set
R2 score: 0.729
MSE: 0.397
Predicted pIC50
Actual pIC50
3
4
5
6
7
8
9
10

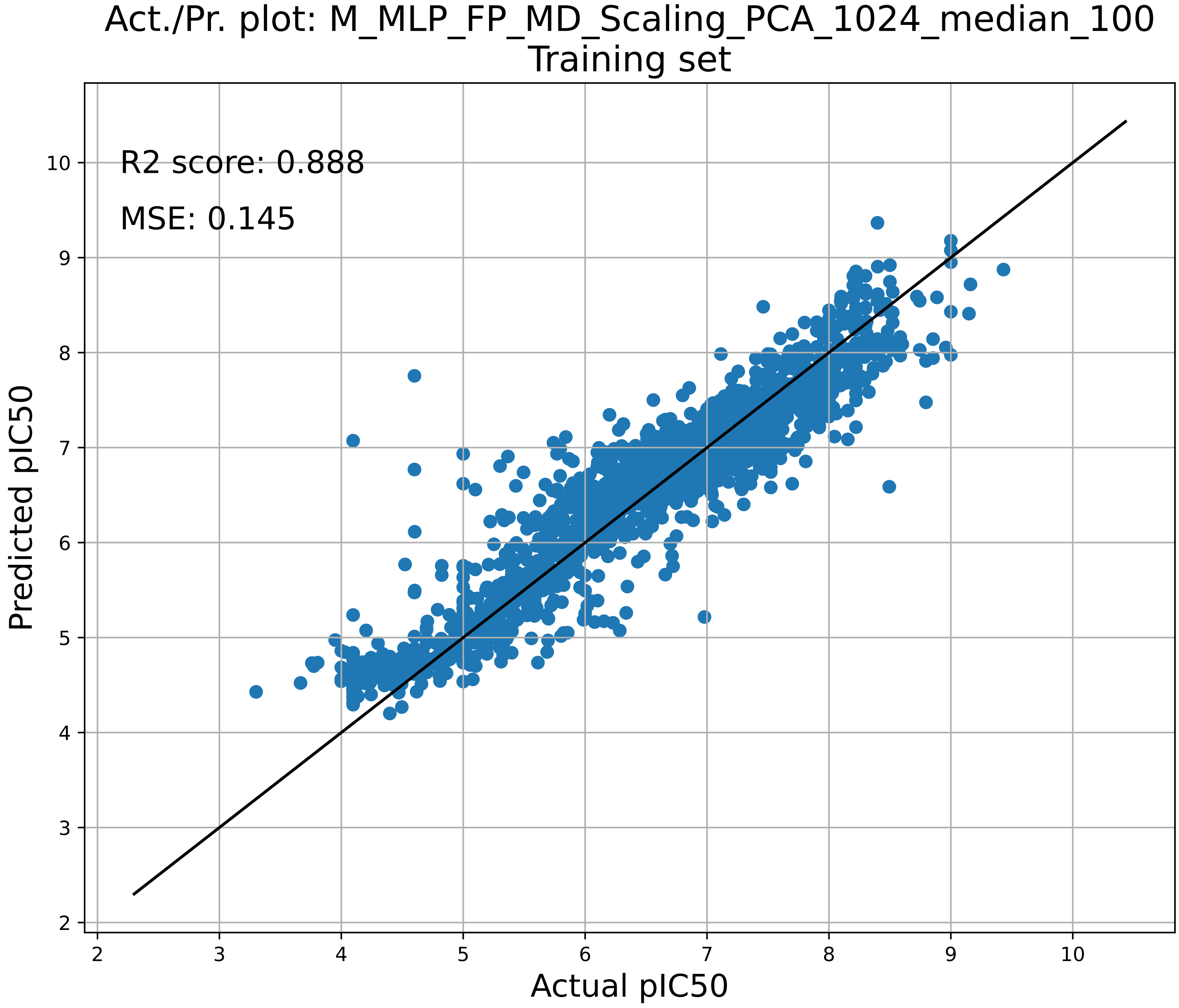

Act./Pr. plot: M_MLP_FP_MD_Scaling_PCA_1024_median_100
Training set
R2 score: 0.888
MSE: 0.145
Predicted pIC50
Actual pIC50
2
3
4
5
6
7
8
9
10

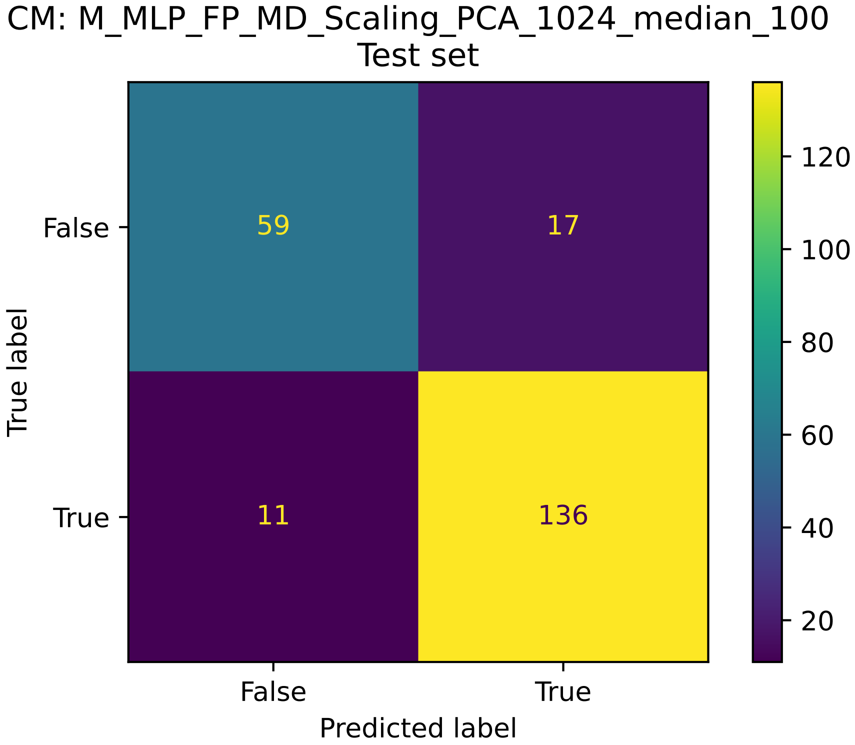
CM: M_MLP_FP_MD_Scaling_PCA_1024_median_100
Test set
True label
Predicted label
False
True
False
True
59
17
11
136
20
40
60
80
100
120

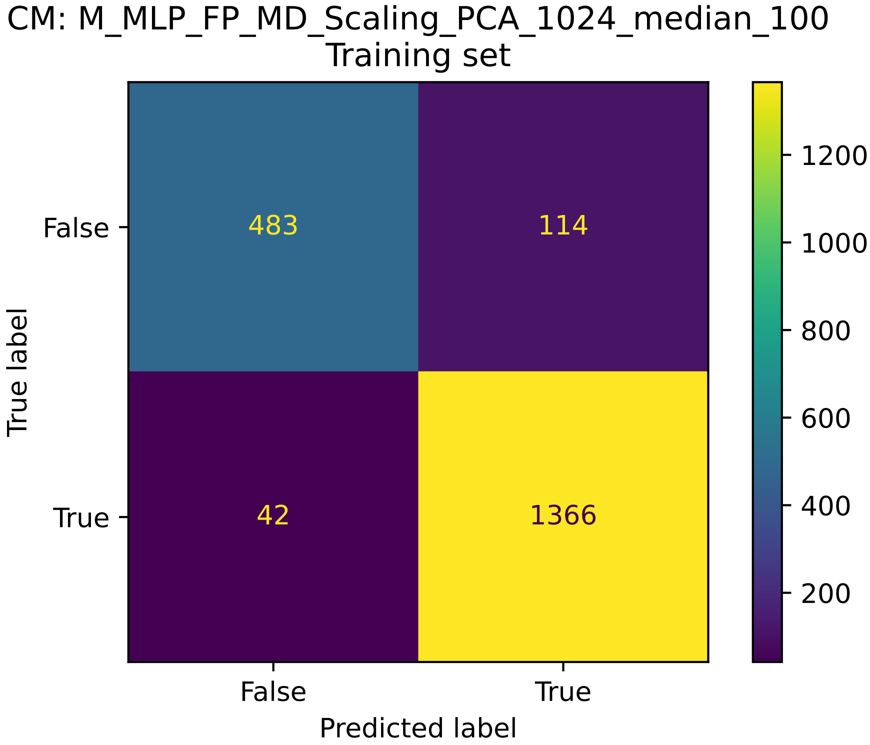
CM: M_MLP_FP_MD_Scaling_PCA_1024_median_100
Training set
483
114
42
1366
False
True
True label
False
True
Predicted label
1200
1000
800
600
400
200

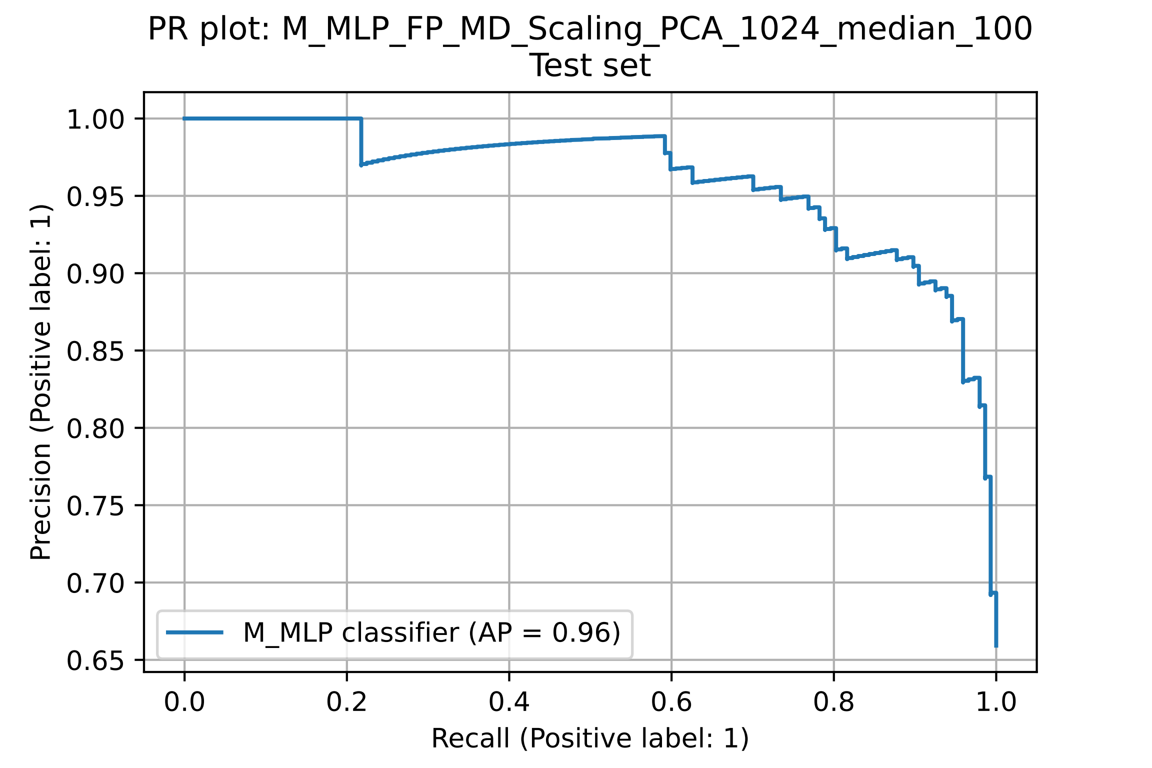
PR plot: M_MLP_FP_MD_Scaling_PCA_1024_median_100
Test set
Precision (Positive label: 1)
Recall (Positive label: 1)
1.00
0.95
0.90
0.85
0.80
0.75
0.70
0.65
0.0
0.2
0.4
0.6
0.8
1.0
M_MLP classifier (AP = 0.96)

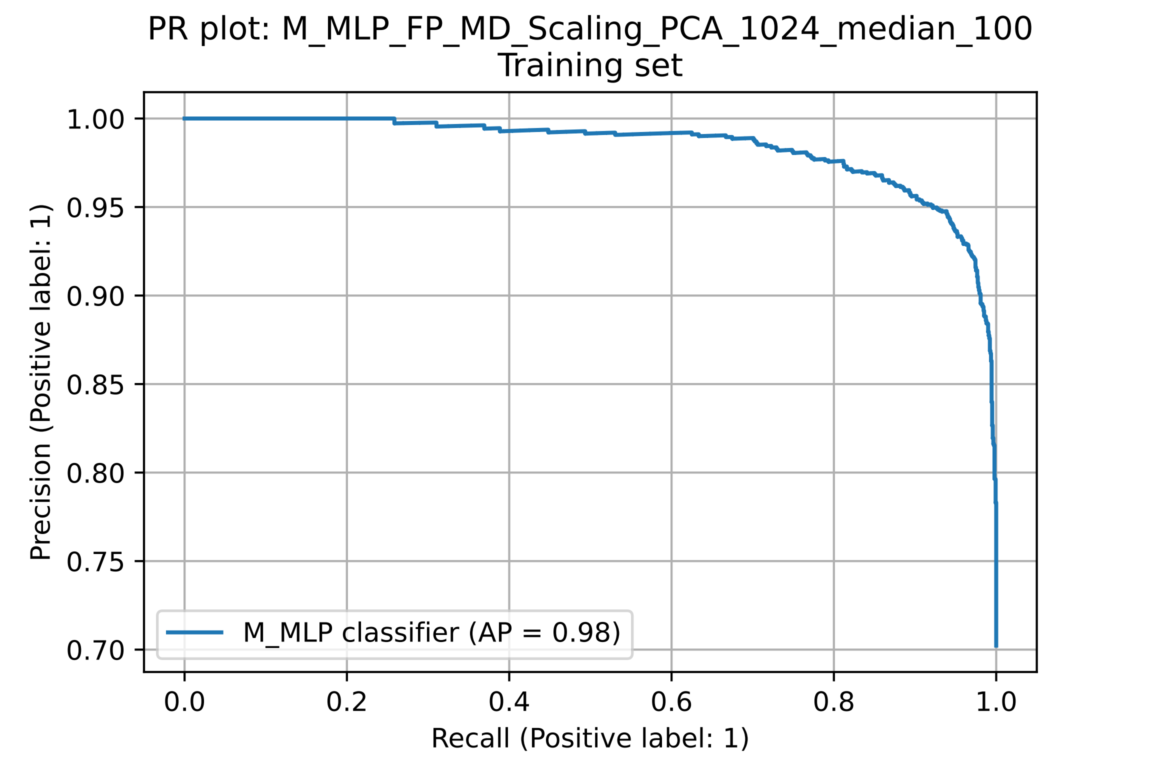
PR plot: M_MLP_FP_MD_Scaling_PCA_1024_median_100
Training set
Precision (Positive label: 1)
Recall (Positive label: 1)
M_MLP classifier (AP = 0.98)
1.00
0.95
0.90
0.85
0.80
0.75
0.70
0.0
0.2
0.4
0.6
0.8
1.0

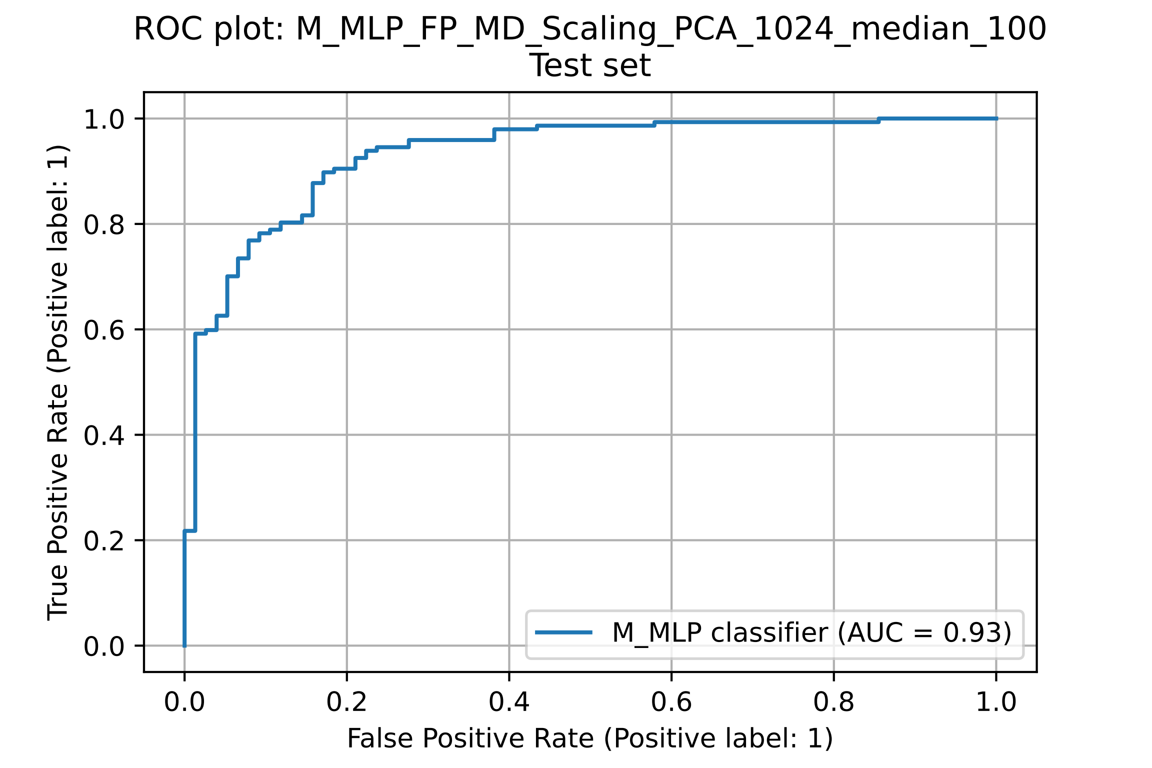
ROC plot: M_MLP_FP_MD_Scaling_PCA_1024_median_100
Test set
1.0
0.8
0.6
0.4
0.2
0.0
True Positive Rate (Positive label: 1)
0.0
0.2
0.4
0.6
0.8
1.0
False Positive Rate (Positive label: 1)
M_MLP classifier (AUC = 0.93)

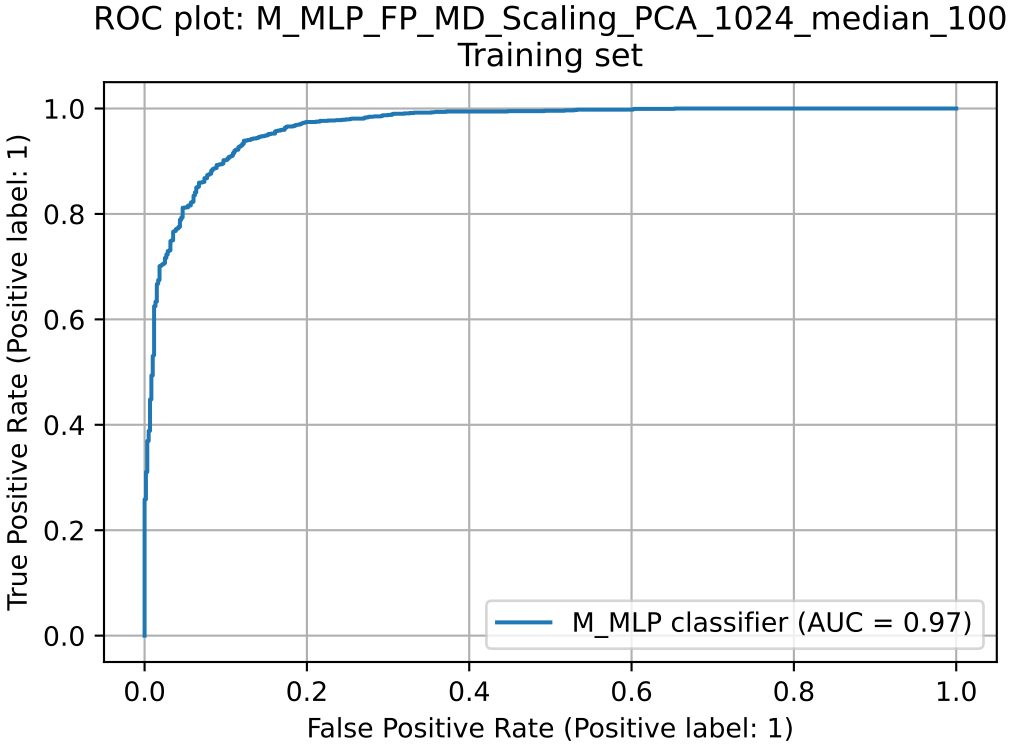
ROC plot: M_MLP_FP_MD_Scaling_PCA_1024_median_100
Training set
True Positive Rate (Positive label: 1)
False Positive Rate (Positive label: 1)
0.0
0.2
0.4
0.6
0.8
1.0
M_MLP classifier (AUC = 0.97)

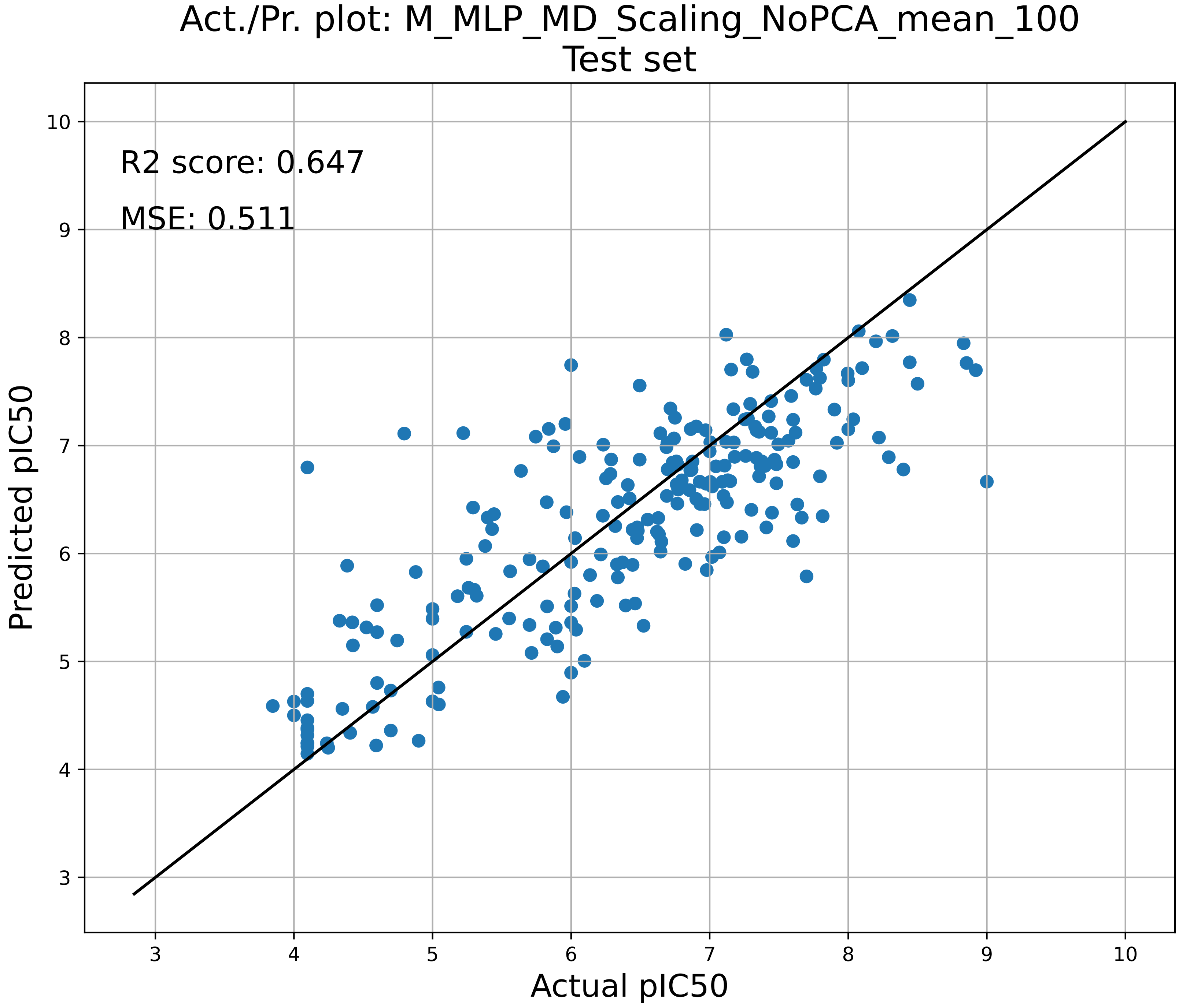
Act./Pr. plot: M_MLP_MD_Scaling_NoPCA_mean_100
Test set
R2 score: 0.647
MSE: 0.511
Predicted pIC50
Actual pIC50
3
4
5
6
7
8
9
10

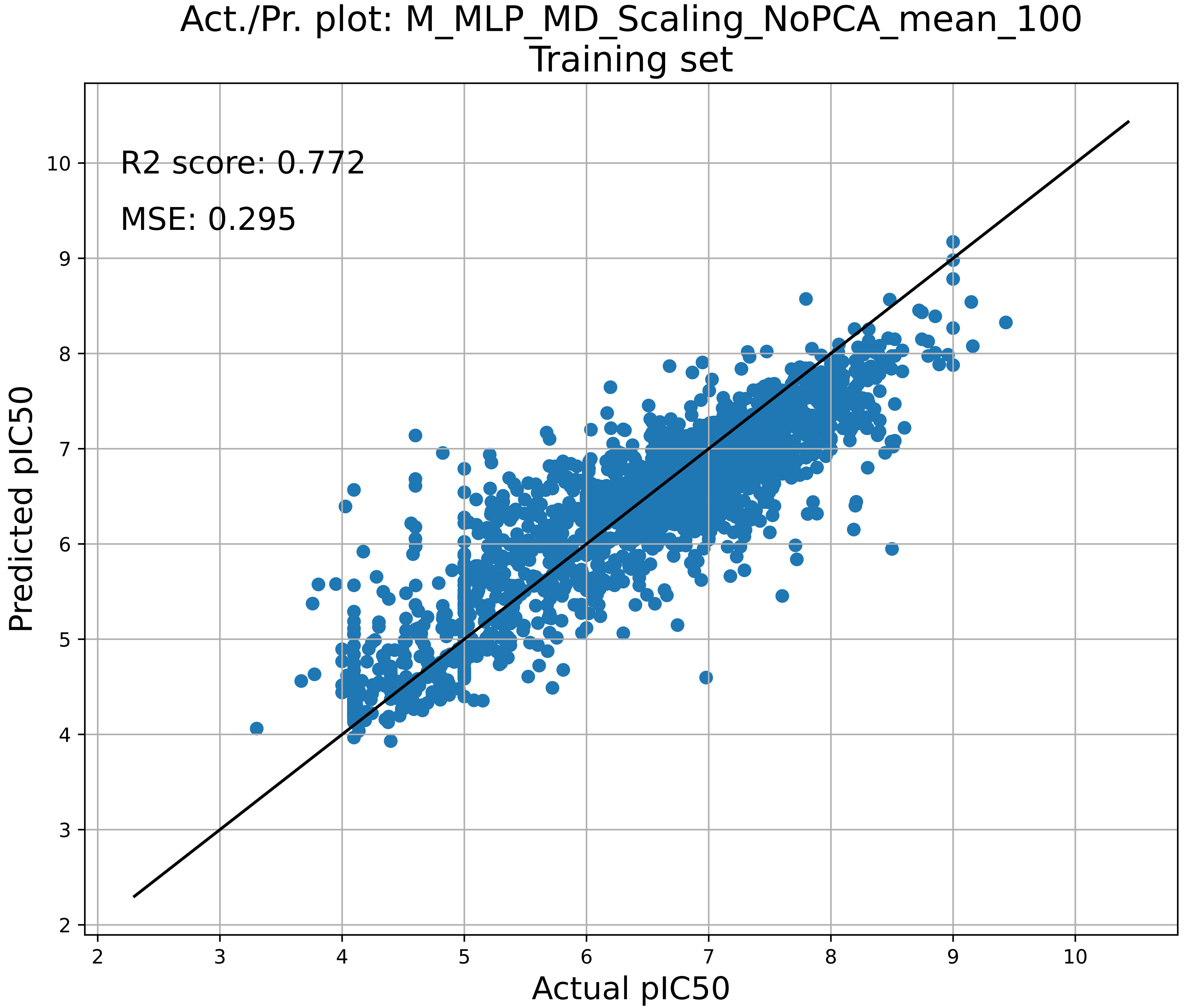

Act./Pr. plot: M_MLP_MD_Scaling_NoPCA_mean_100
Training set
R2 score: 0.772
MSE: 0.295
Predicted pIC50
Actual pIC50
2
3
4
5
6
7
8
9
10

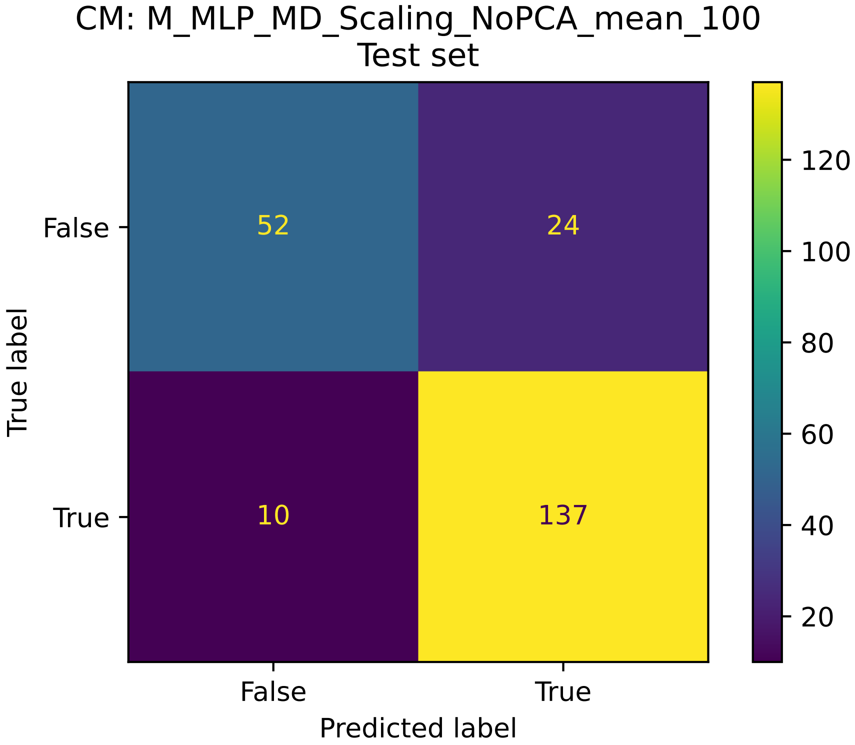

CM: M_MLP_MD_Scaling_NoPCA_mean_100
Test set
True label
False
True
52
24
10
137
Predicted label
False
True
120
100
80
60
40
20

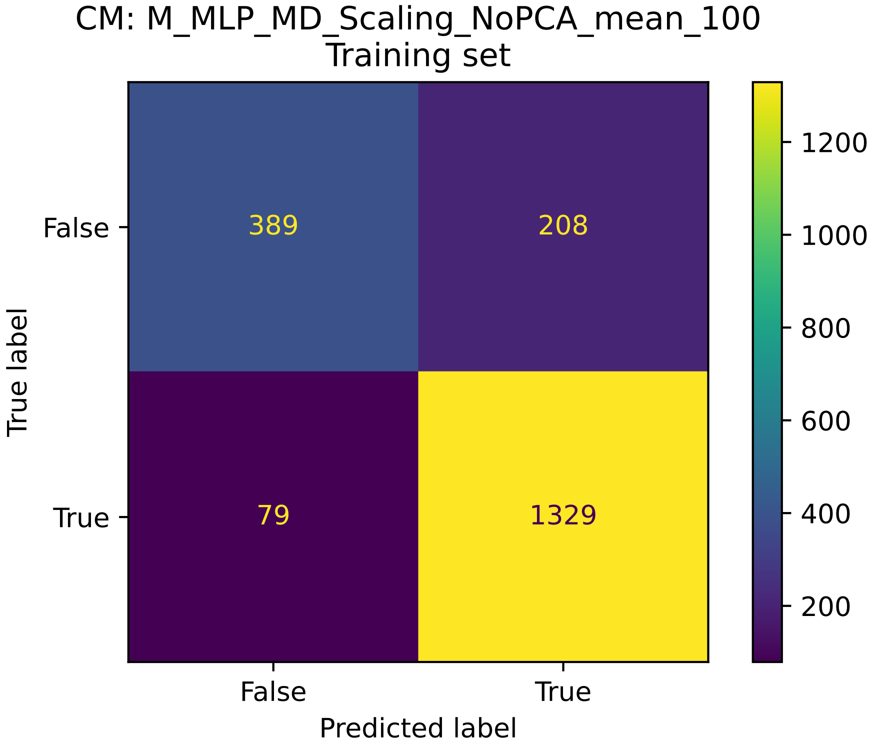
CM: M_MLP_MD_Scaling_NoPCA_mean_100
Training set
True label
Predicted label
False
True
False
True
389
208
79
1329
1200
1000
800
600
400
200

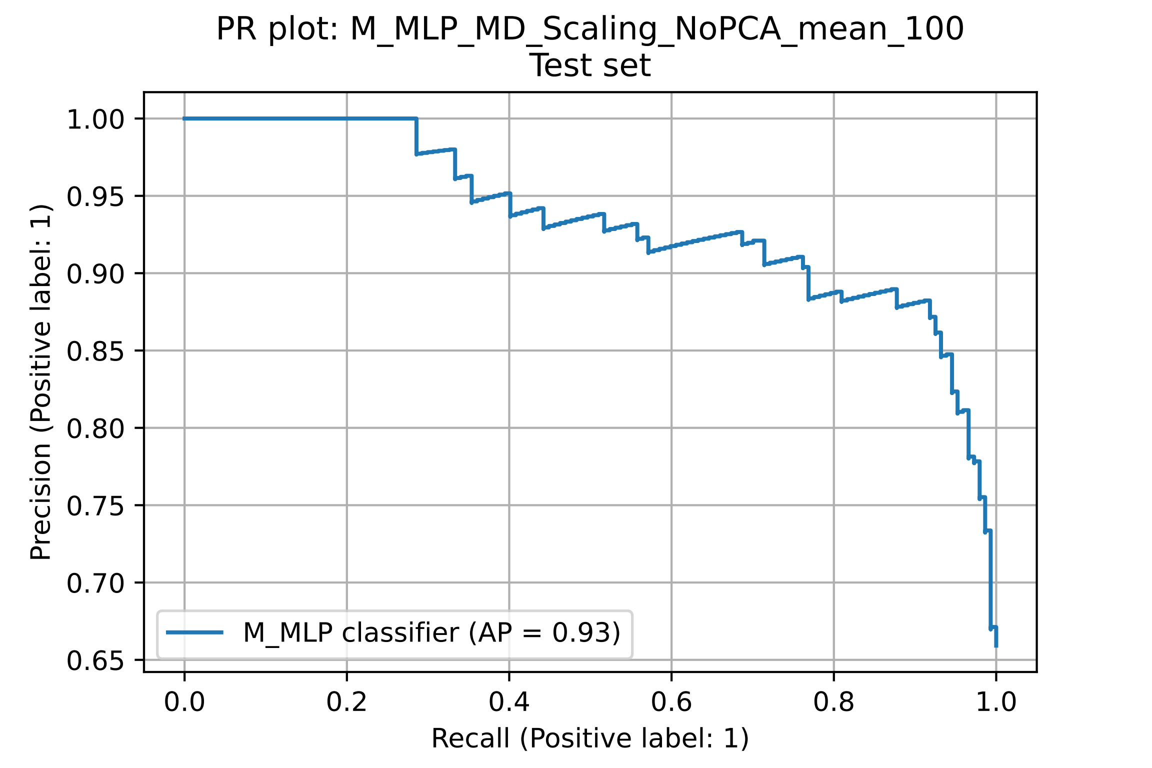
PR plot: M_MLP_MD_Scaling_NoPCA_mean_100
Test set
Precision (Positive label: 1)
Recall (Positive label: 1)
1.00
0.95
0.90
0.85
0.80
0.75
0.70
0.65
0.0
0.2
0.4
0.6
0.8
1.0
M_MLP classifier (AP = 0.93)

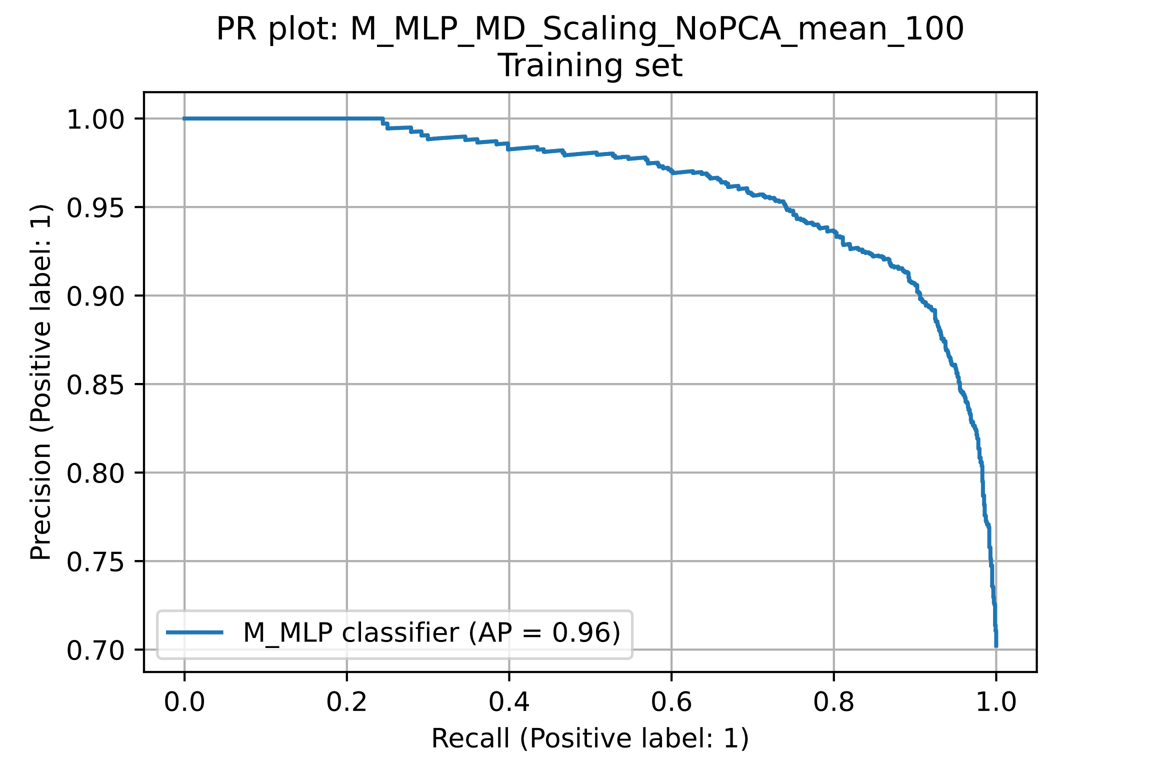
PR plot: M_MLP_MD_Scaling_NoPCA_mean_100
Training set
Precision (Positive label: 1)
Recall (Positive label: 1)
1.00
0.95
0.90
0.85
0.80
0.75
0.70
0.0
0.2
0.4
0.6
0.8
1.0
M_MLP classifier (AP = 0.96)

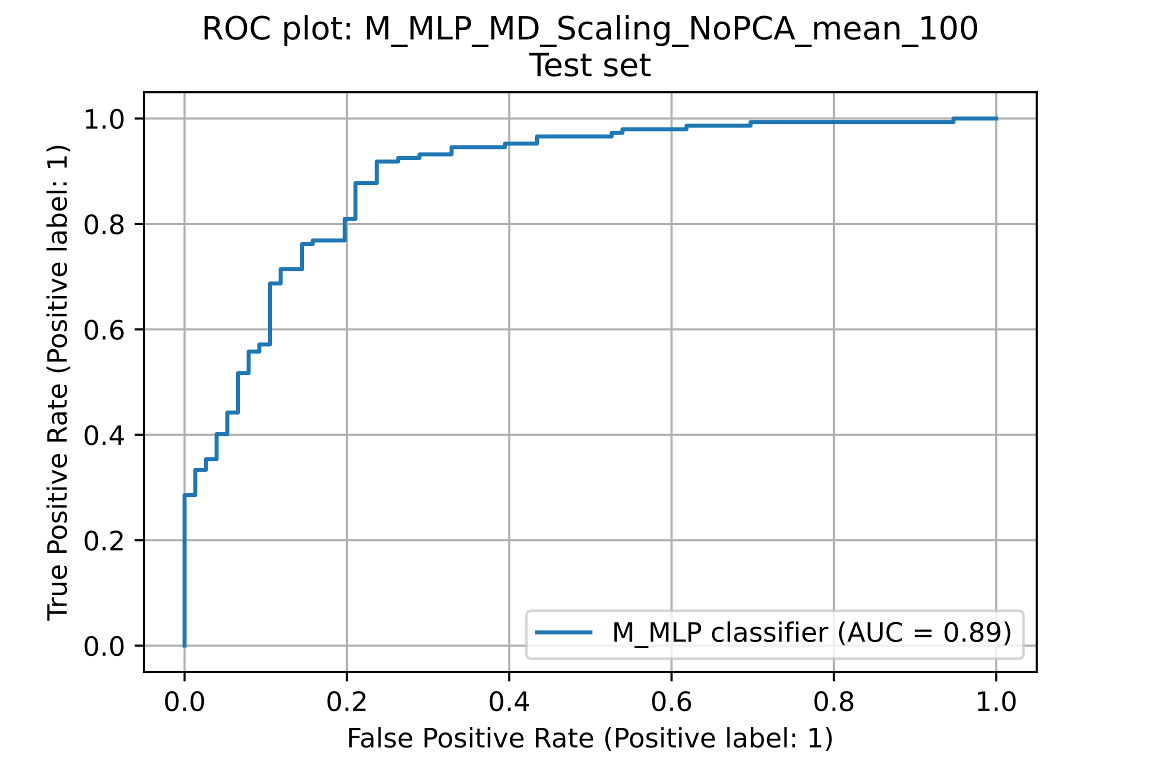

ROC plot: M_MLP_MD_Scaling_NoPCA_mean_100
Test set
True Positive Rate (Positive label: 1)
False Positive Rate (Positive label: 1)
0.0
0.2
0.4
0.6
0.8
1.0
M_MLP classifier (AUC = 0.89)

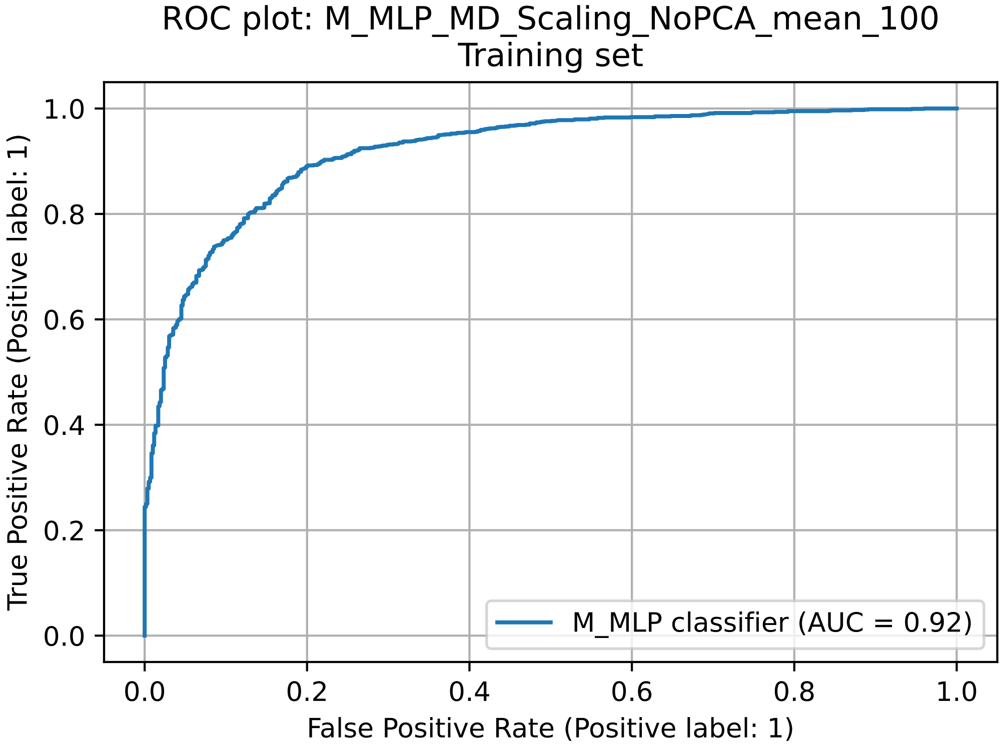
ROC plot: M_MLP_MD_Scaling_NoPCA_mean_100
Training set
True Positive Rate (Positive label: 1)
False Positive Rate (Positive label: 1)
M_MLP classifier (AUC = 0.92)
0.0
0.2
0.4
0.6
0.8
1.0

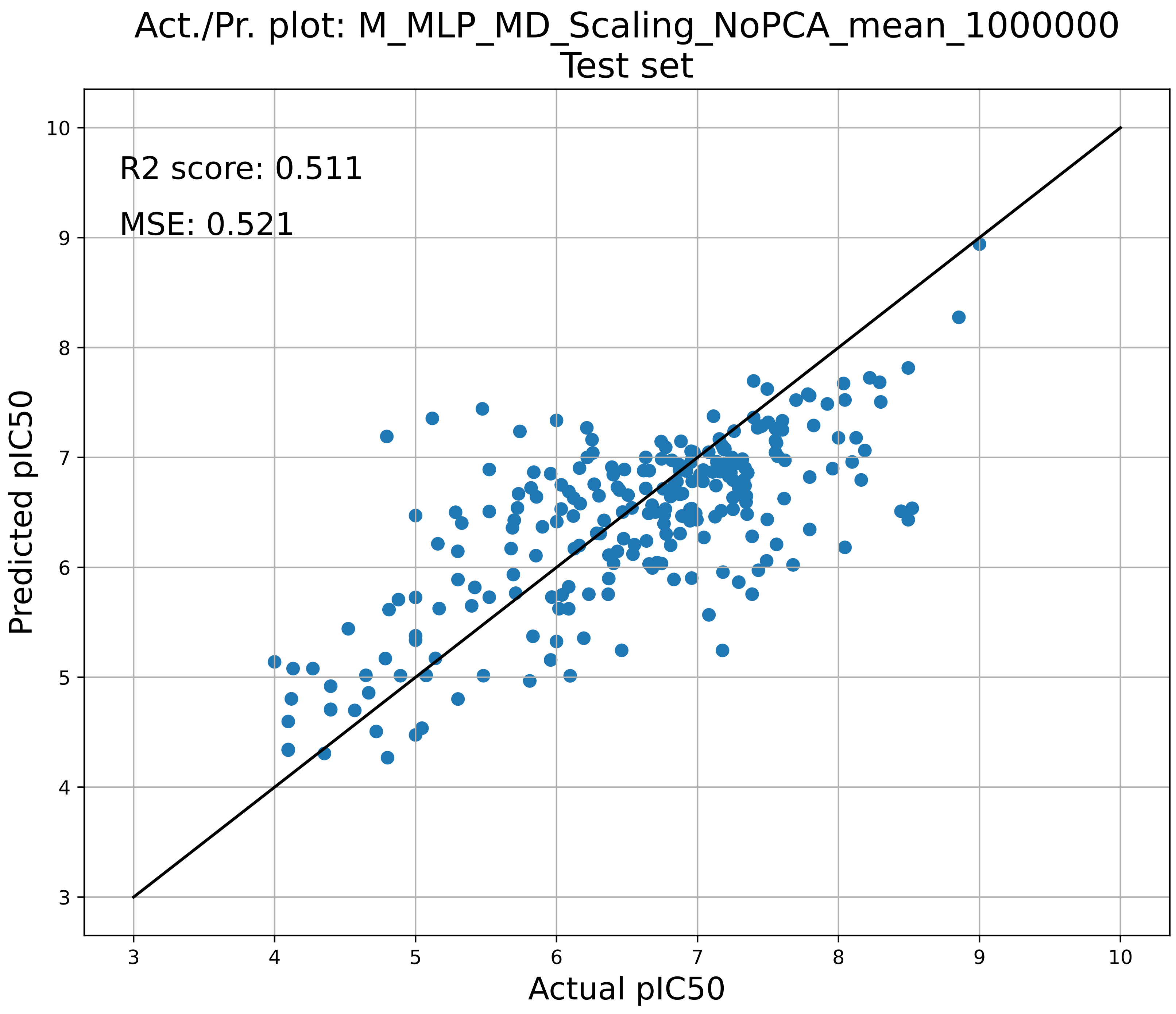

Act./Pr. plot: M_MLP_MD_Scaling_NoPCA_mean_1000000
Test set
R2 score: 0.511
MSE: 0.521
Predicted pIC50
Actual pIC50
3
4
5
6
7
8
9
10

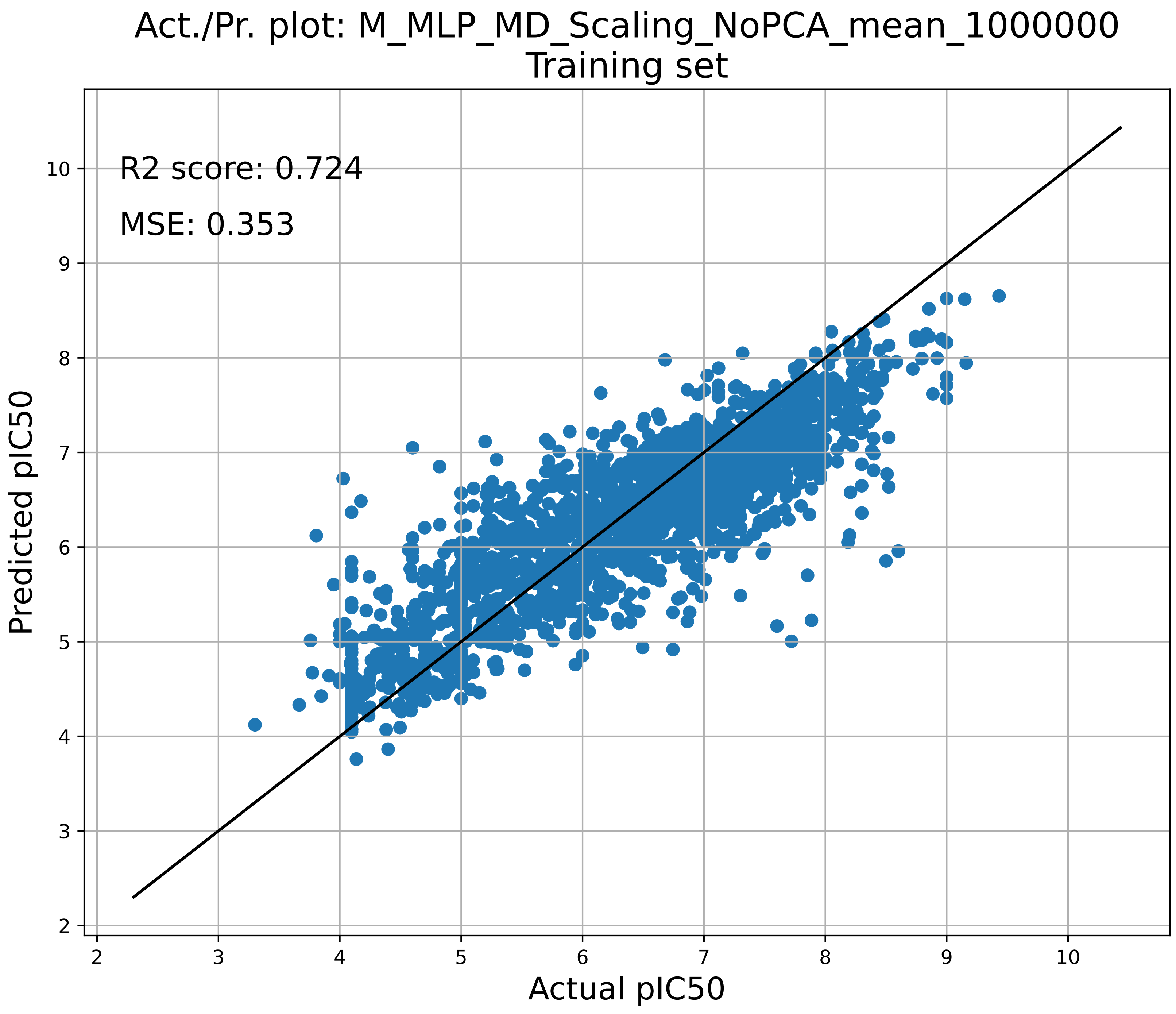
Act./Pr. plot: M_MLP_MD_Scaling_NoPCA_mean_1000000
Training set
R2 score: 0.724
MSE: 0.353
Predicted pIC50
Actual pIC50
2
3
4
5
6
7
8
9
10

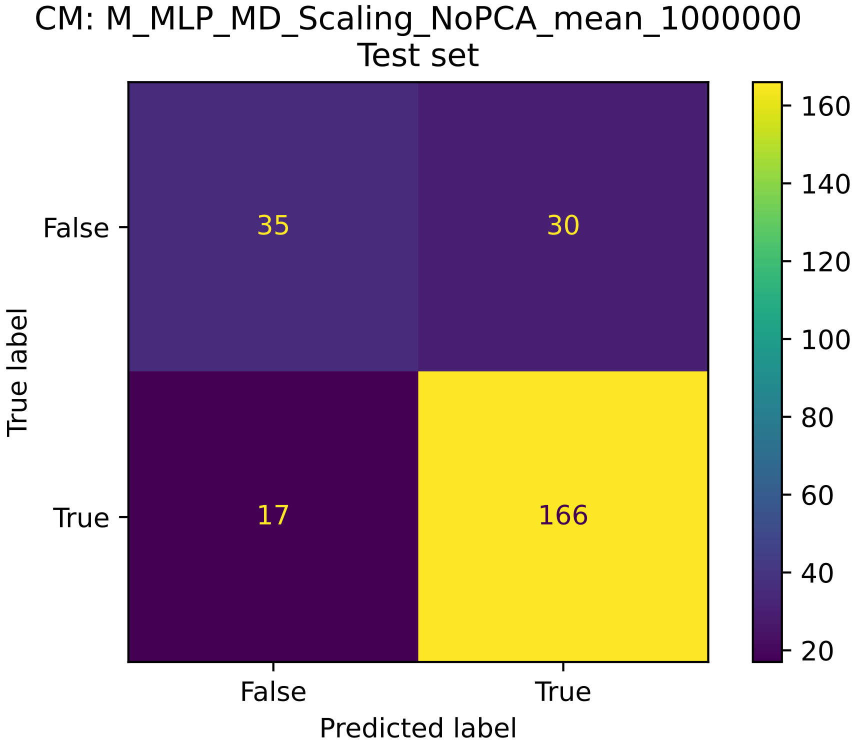
CM: M_MLP_MD_Scaling_NoPCA_mean_1000000
Test set
True label
False
True
35
30
17
166
Predicted label
160
140
120
100
80
60
40
20

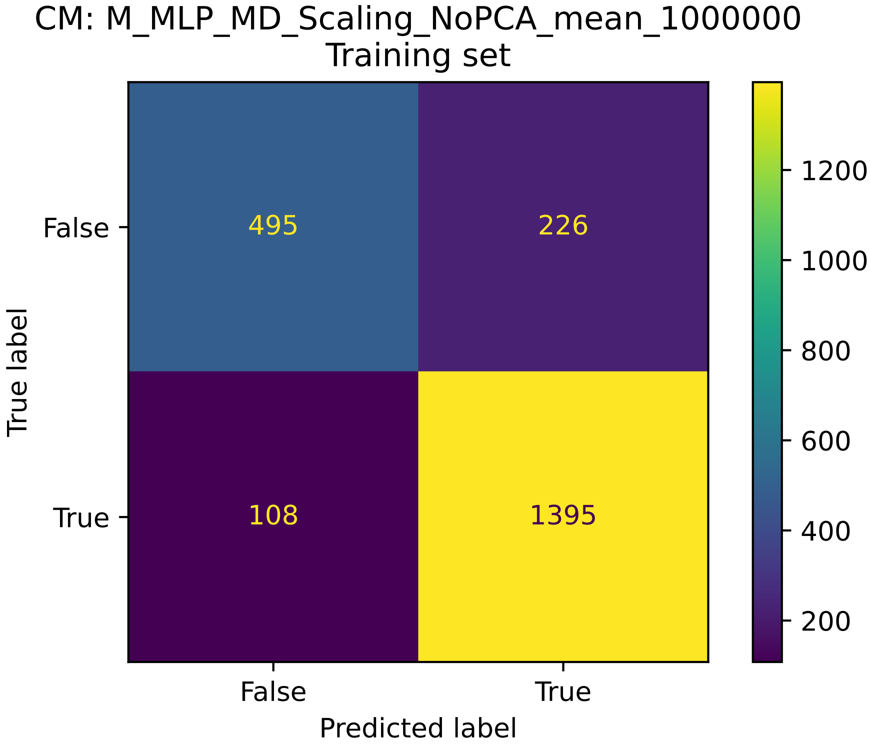

CM: M_MLP_MD_Scaling_NoPCA_mean_1000000
Training set
True label
False
True
495
226
108
1395
Predicted label
False
True
200
400
600
800
1000
1200

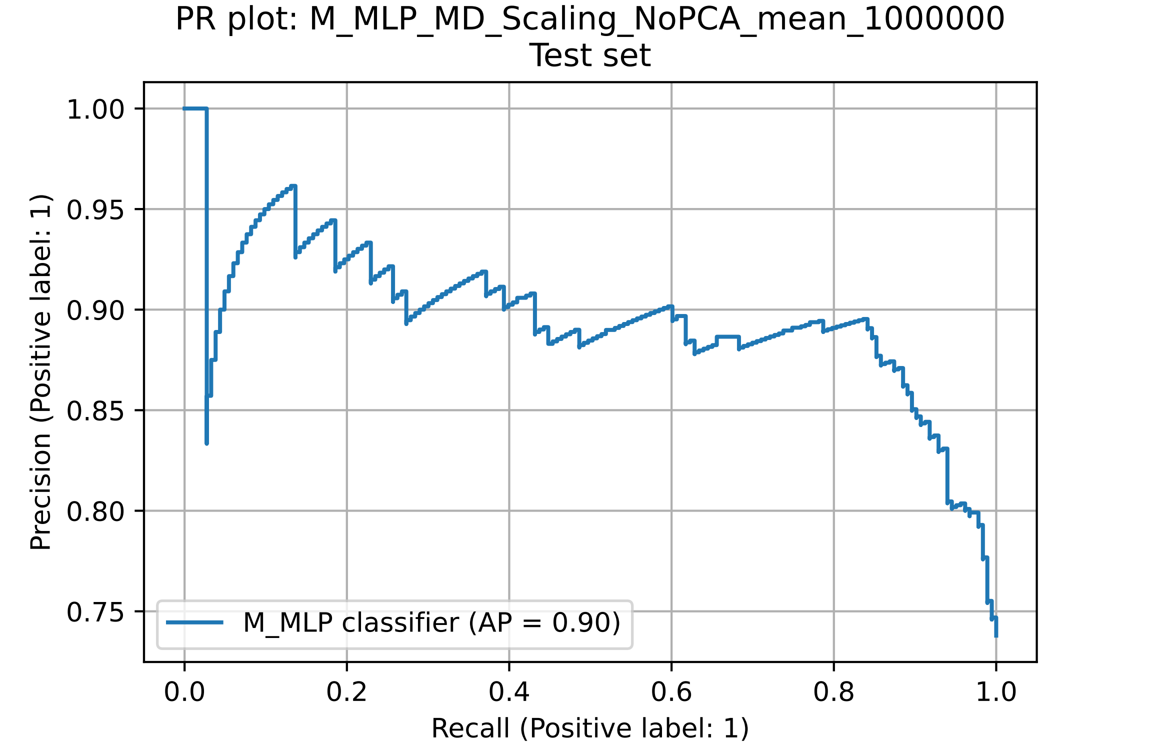
PR plot: M_MLP_MD_Scaling_NoPCA_mean_1000000
Test set
Precision (Positive label: 1)
Recall (Positive label: 1)
1.00
0.95
0.90
0.85
0.80
0.75
0.0
0.2
0.4
0.6
0.8
1.0
M_MLP classifier (AP = 0.90)

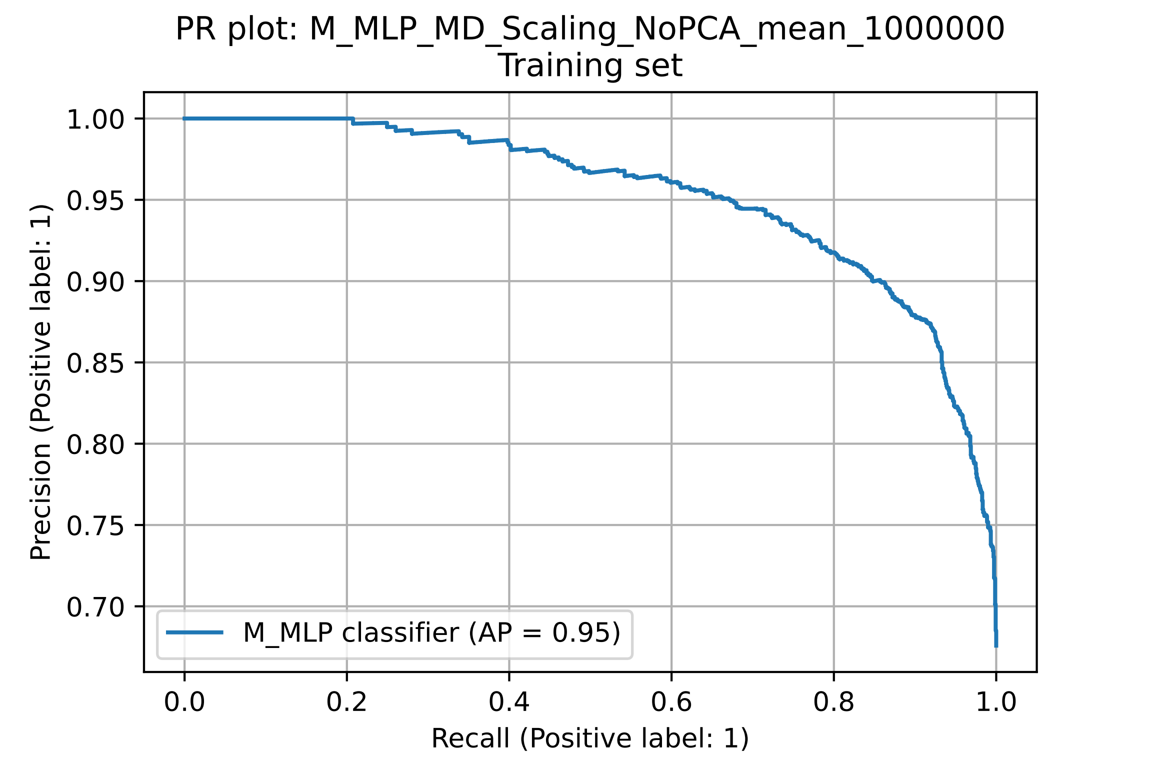
PR plot: M_MLP_MD_Scaling_NoPCA_mean_1000000
Training set
Precision (Positive label: 1)
Recall (Positive label: 1)
1.00
0.95
0.90
0.85
0.80
0.75
0.70
0.0
0.2
0.4
0.6
0.8
1.0
M_MLP classifier (AP = 0.95)

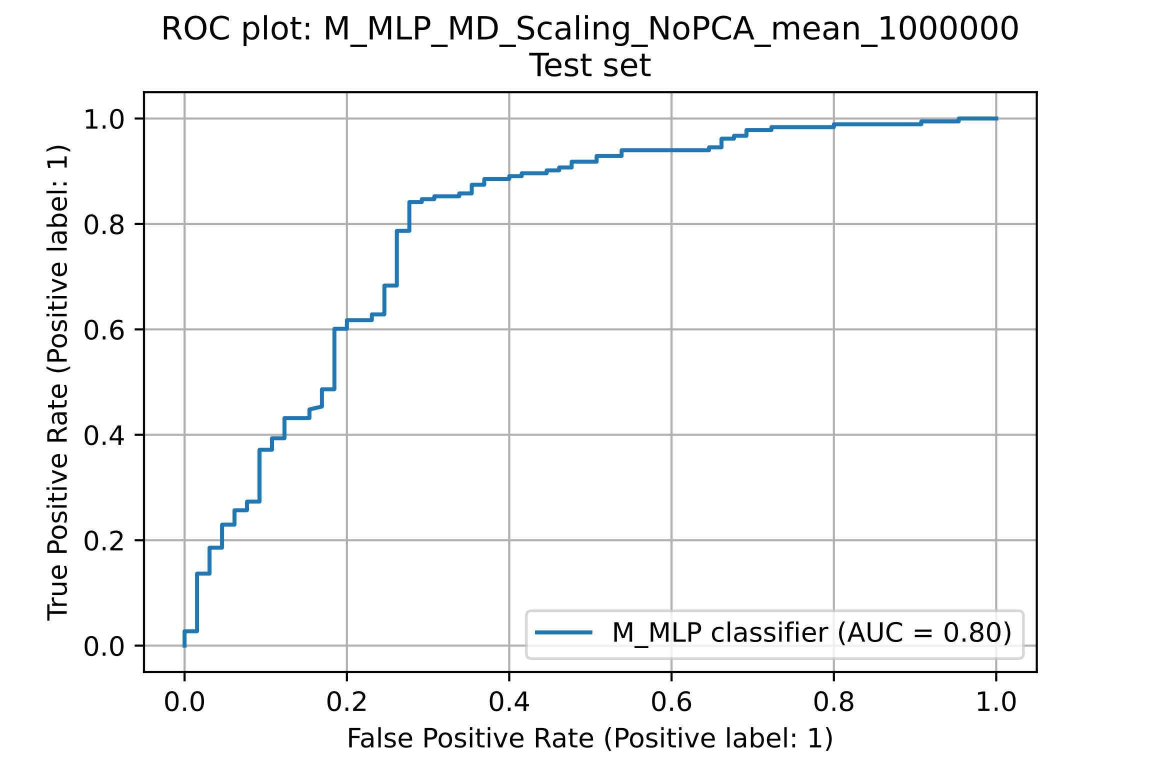
ROC plot: M_MLP_MD_Scaling_NoPCA_mean_1000000
Test set
True Positive Rate (Positive label: 1)
False Positive Rate (Positive label: 1)
0.0
0.2
0.4
0.6
0.8
1.0
M_MLP classifier (AUC = 0.80)

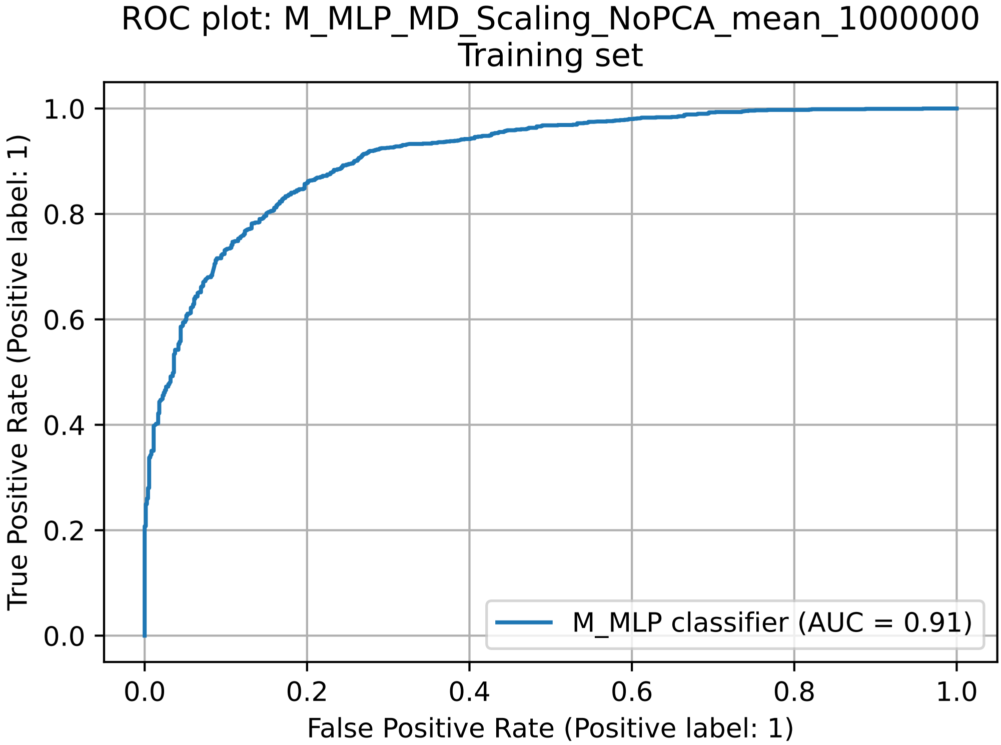

ROC plot: M_MLP_MD_Scaling_NoPCA_mean_1000000
Training set
True Positive Rate (Positive label: 1)
False Positive Rate (Positive label: 1)
0.0
0.2
0.4
0.6
0.8
1.0
M_MLP classifier (AUC = 0.91)

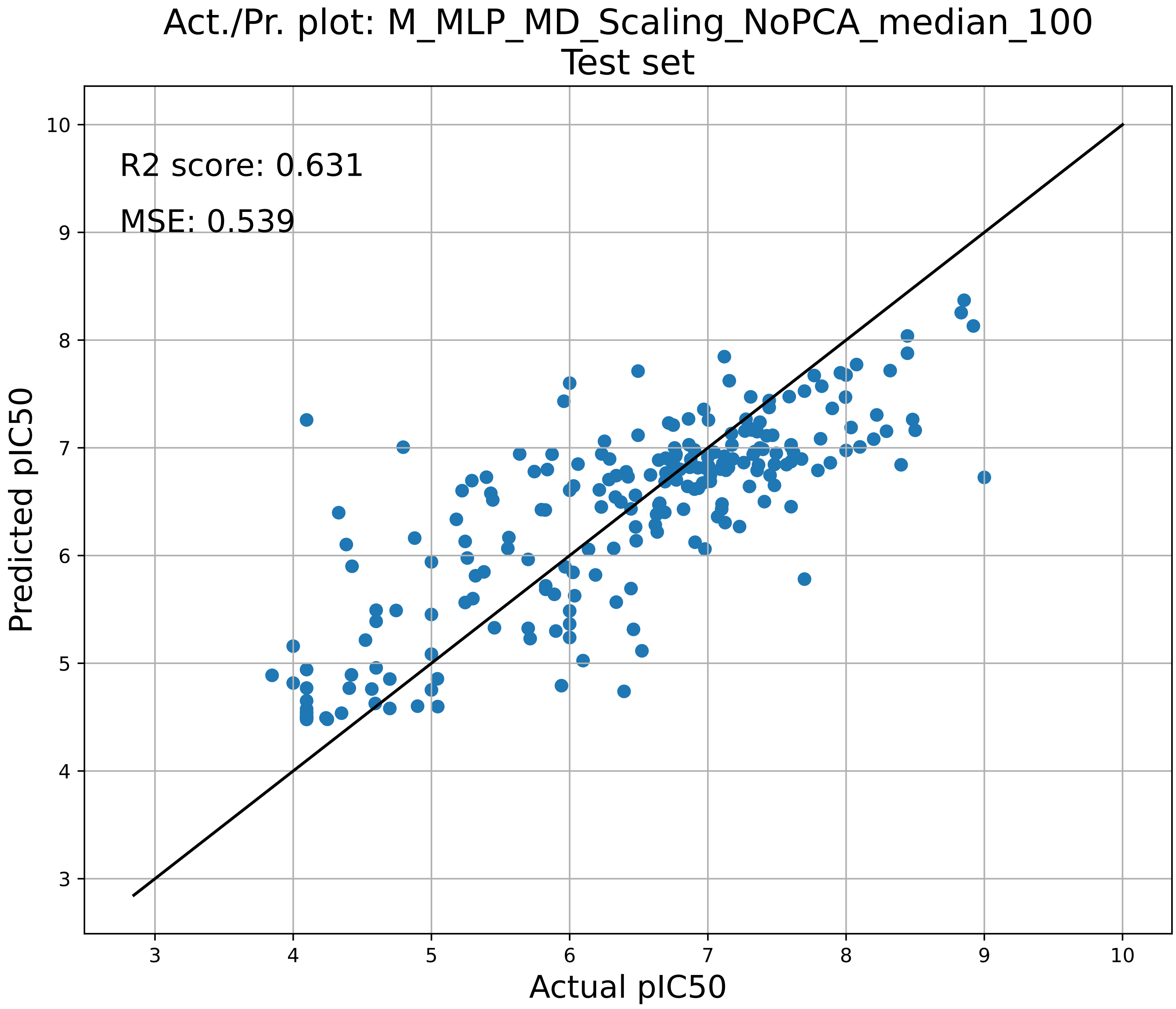

Act./Pr. plot: M_MLP_MD_Scaling_NoPCA_median_100
Test set
R2 score: 0.631
MSE: 0.539
Predicted pIC50
Actual pIC50
3
4
5
6
7
8
9
10

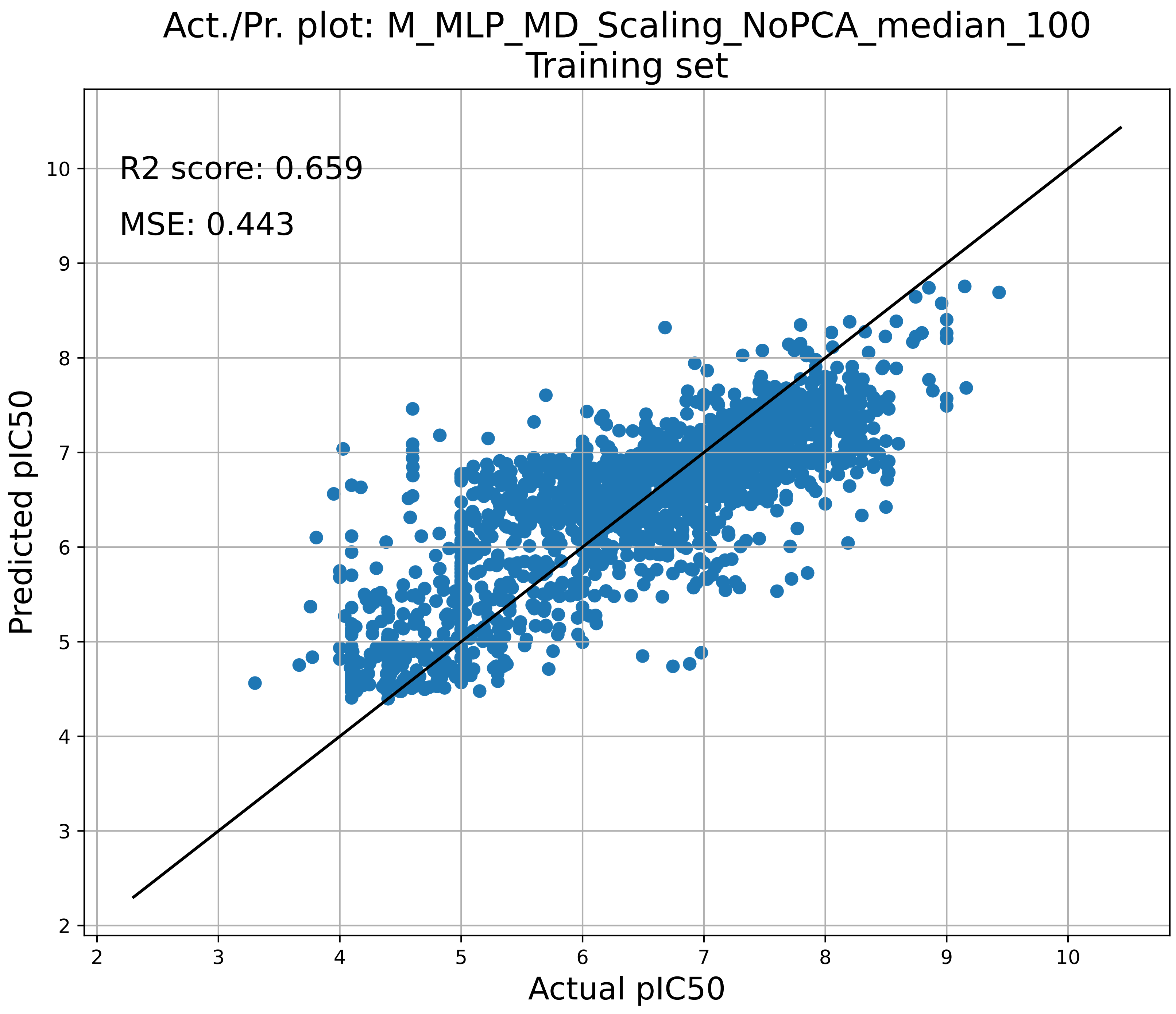

Act./Pr. plot: M_MLP_MD_Scaling_NoPCA_median_100
Training set
R2 score: 0.659
MSE: 0.443
Predicted pIC50
Actual pIC50
2
3
4
5
6
7
8
9
10

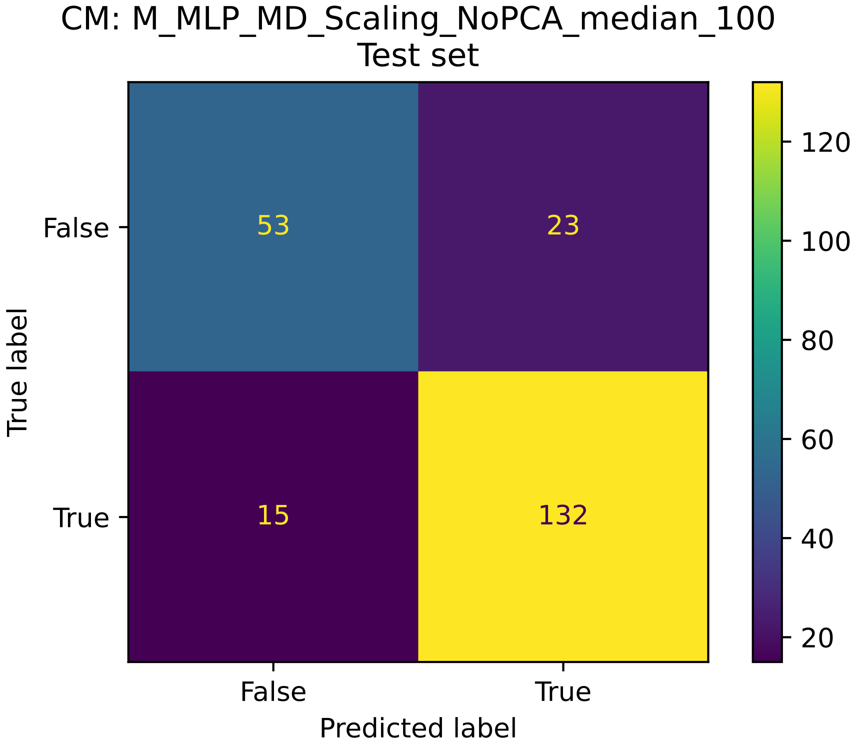

CM: M_MLP_MD_Scaling_NoPCA_median_100
Test set
True label
False
True
53
23
15
132
Predicted label
False
True
120
100
80
60
40
20

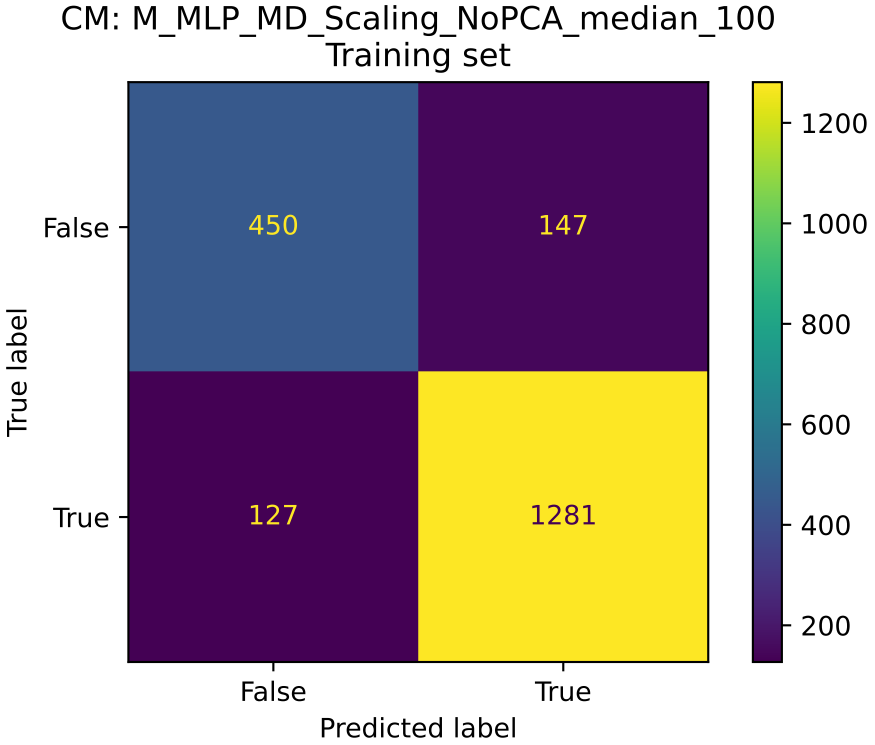
CM: M_MLP_MD_Scaling_NoPCA_median_100
Training set
450
147
127
1281
False
True
True label
False
True
Predicted label
1200
1000
800
600
400
200

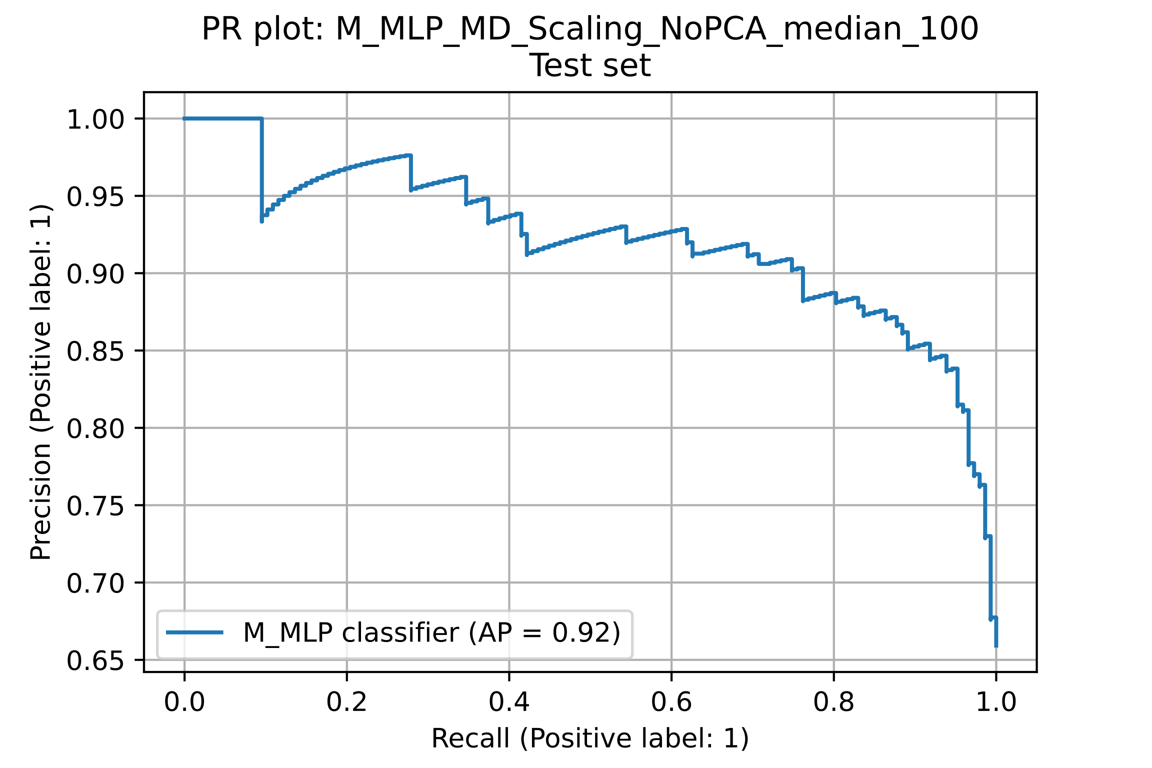

PR plot: M_MLP_MD_Scaling_NoPCA_median_100
Test set
Precision (Positive label: 1)
Recall (Positive label: 1)
1.00
0.95
0.90
0.85
0.80
0.75
0.70
0.65
0.0
0.2
0.4
0.6
0.8
1.0
M_MLP classifier (AP = 0.92)

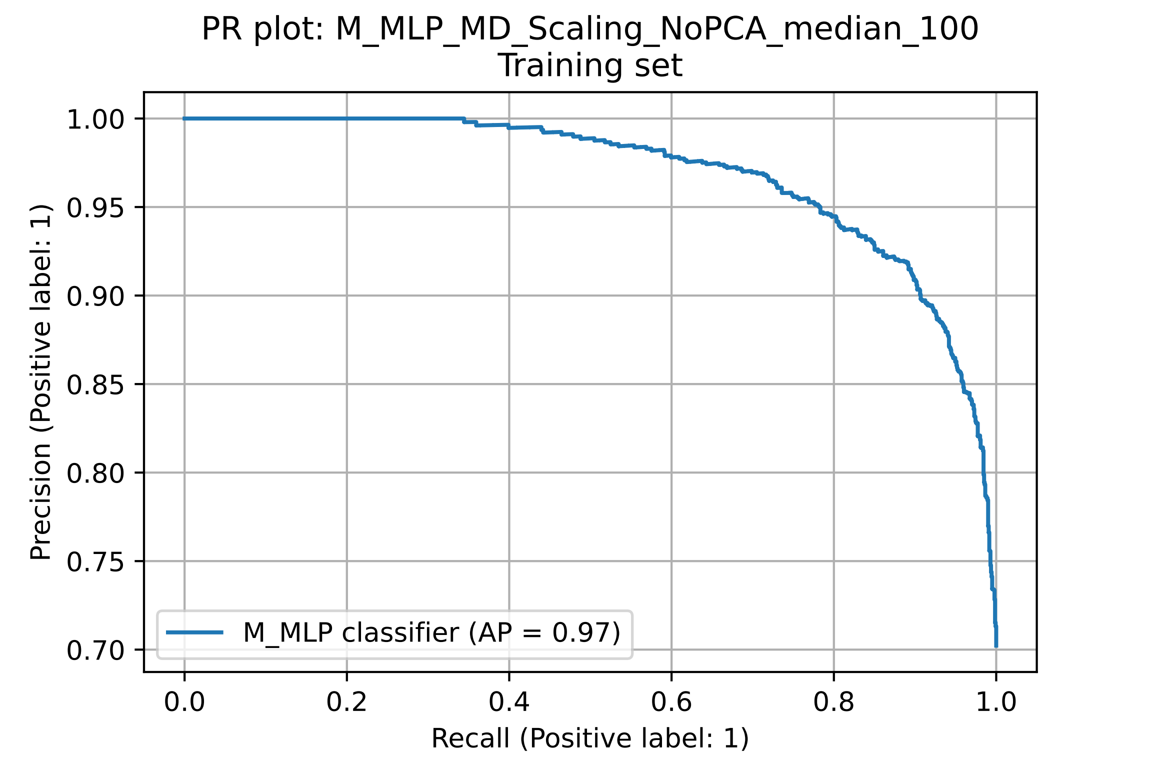

PR plot: M_MLP_MD_Scaling_NoPCA_median_100
Training set
Precision (Positive label: 1)
Recall (Positive label: 1)
1.00
0.95
0.90
0.85
0.80
0.75
0.70
0.0
0.2
0.4
0.6
0.8
1.0
M_MLP classifier (AP = 0.97)

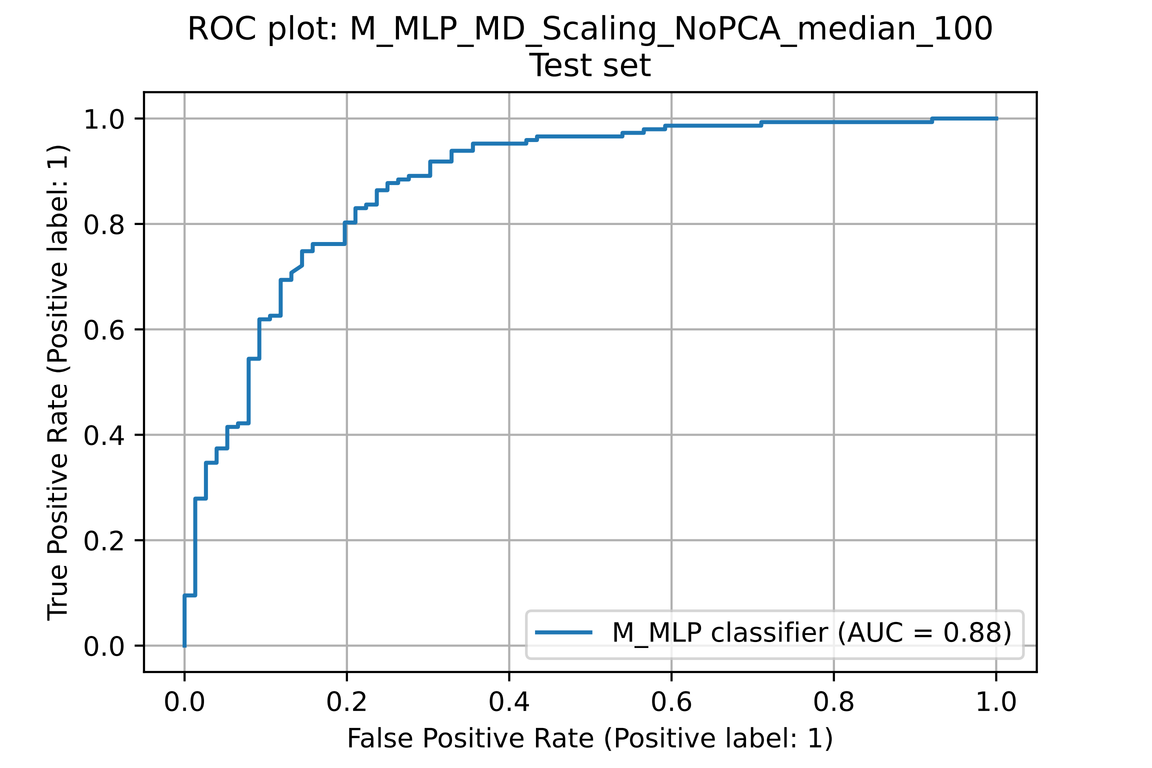
ROC plot: M_MLP_MD_Scaling_NoPCA_median_100
Test set
True Positive Rate (Positive label: 1)
False Positive Rate (Positive label: 1)
0.0
0.2
0.4
0.6
0.8
1.0
M_MLP classifier (AUC = 0.88)

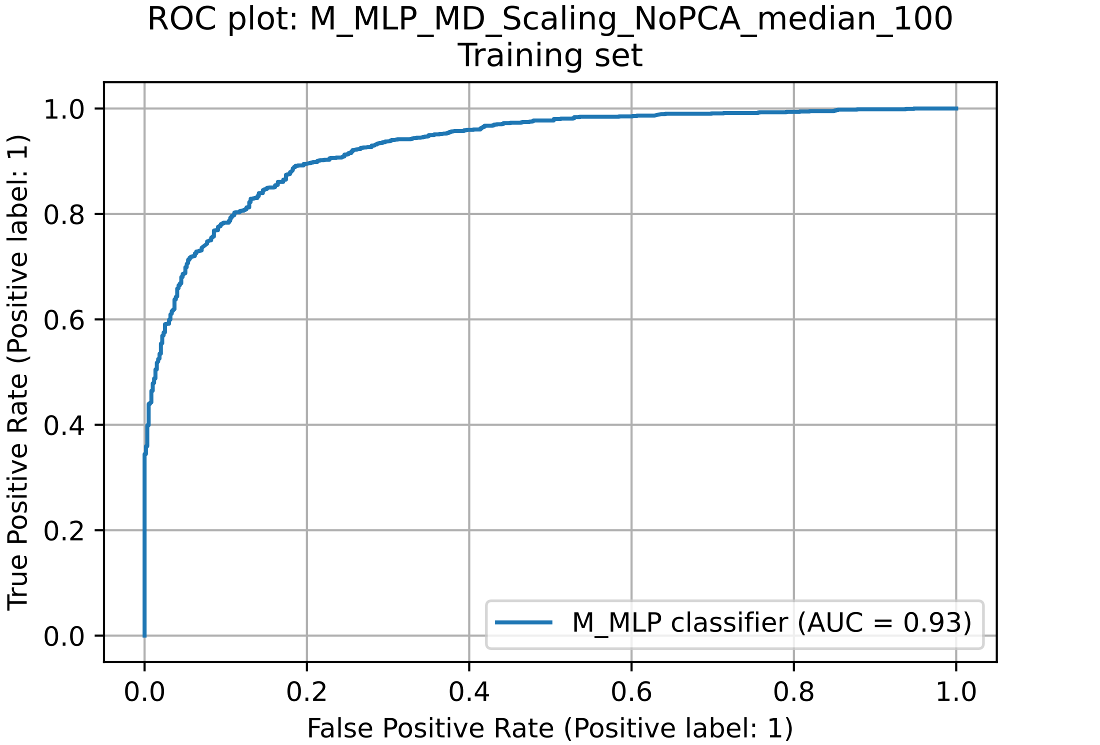
ROC plot: M_MLP_MD_Scaling_NoPCA_median_100
Training set
True Positive Rate (Positive label: 1)
False Positive Rate (Positive label: 1)
0.0
0.2
0.4
0.6
0.8
1.0
M_MLP classifier (AUC = 0.93)

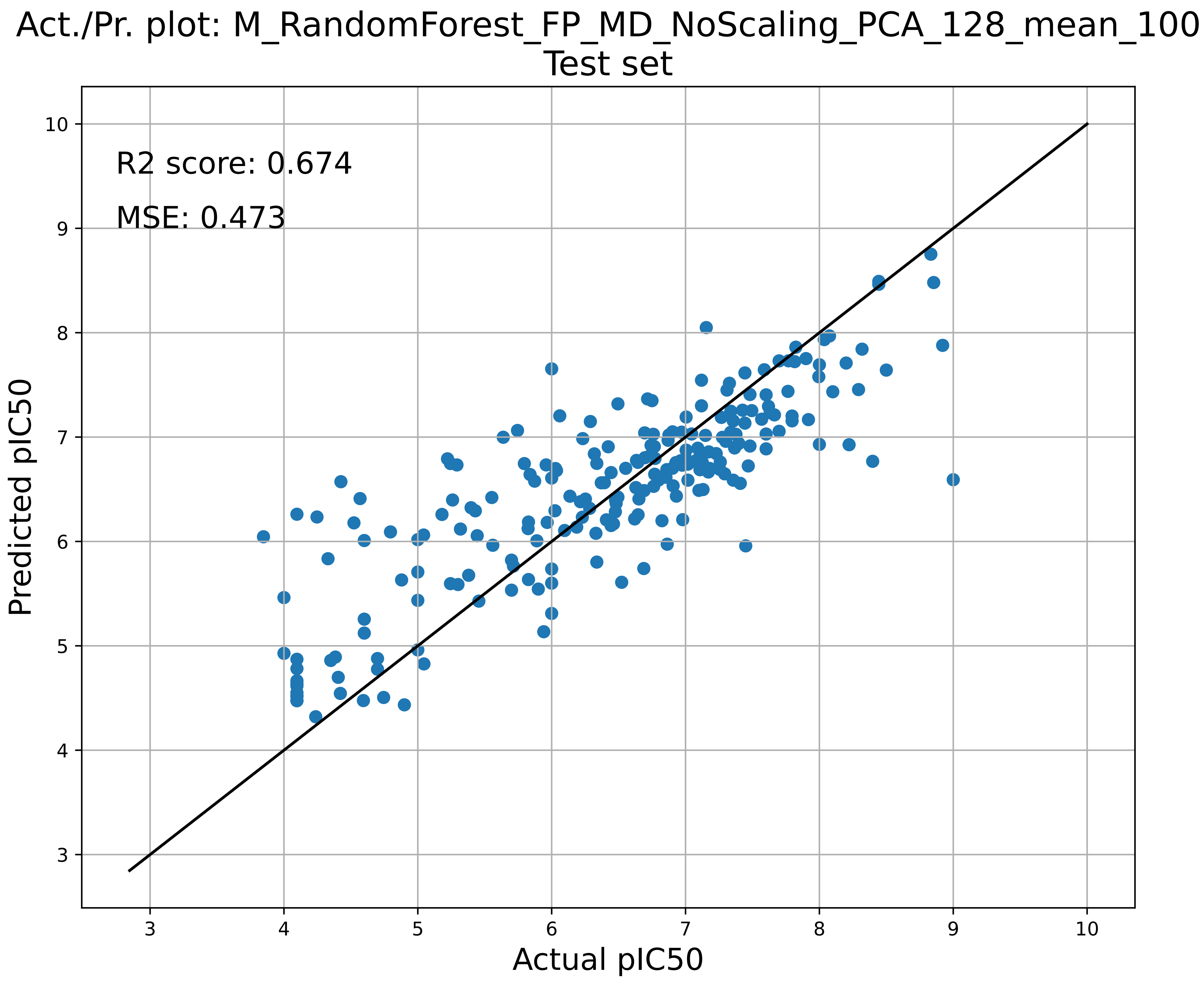
Act./Pr. plot: M_RandomForest_FP_MD_NoScaling_PCA_128_mean_100
Test set
R2 score: 0.674
MSE: 0.473
Predicted pIC50
Actual pIC50
3
4
5
6
7
8
9
10
3
4
5
6
7
8
9
10

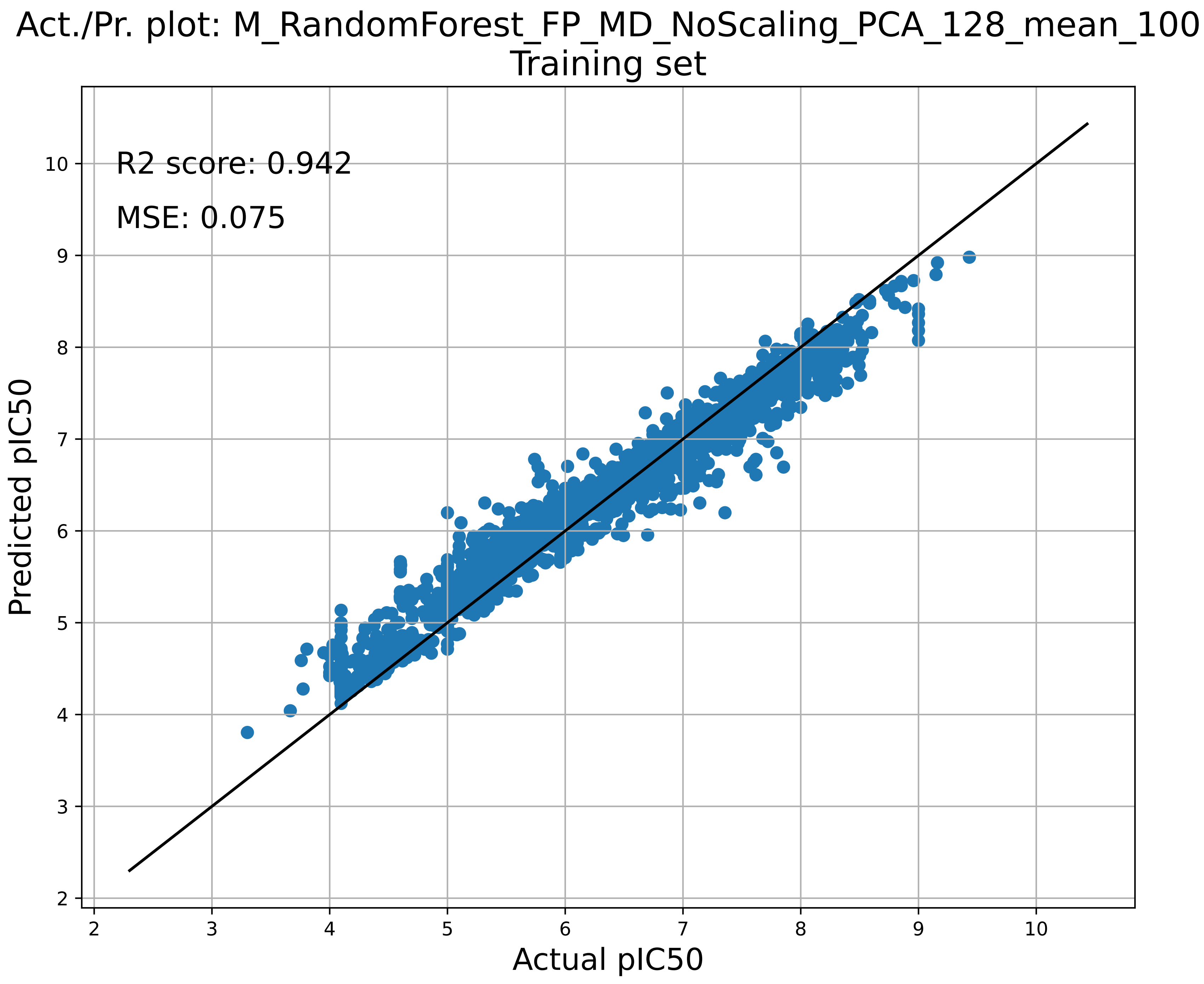

Act./Pr. plot: M_RandomForest_FP_MD_NoScaling_PCA_128_mean_100
Training set
R2 score: 0.942
MSE: 0.075
Predicted pIC50
Actual pIC50
2
3
4
5
6
7
8
9
10

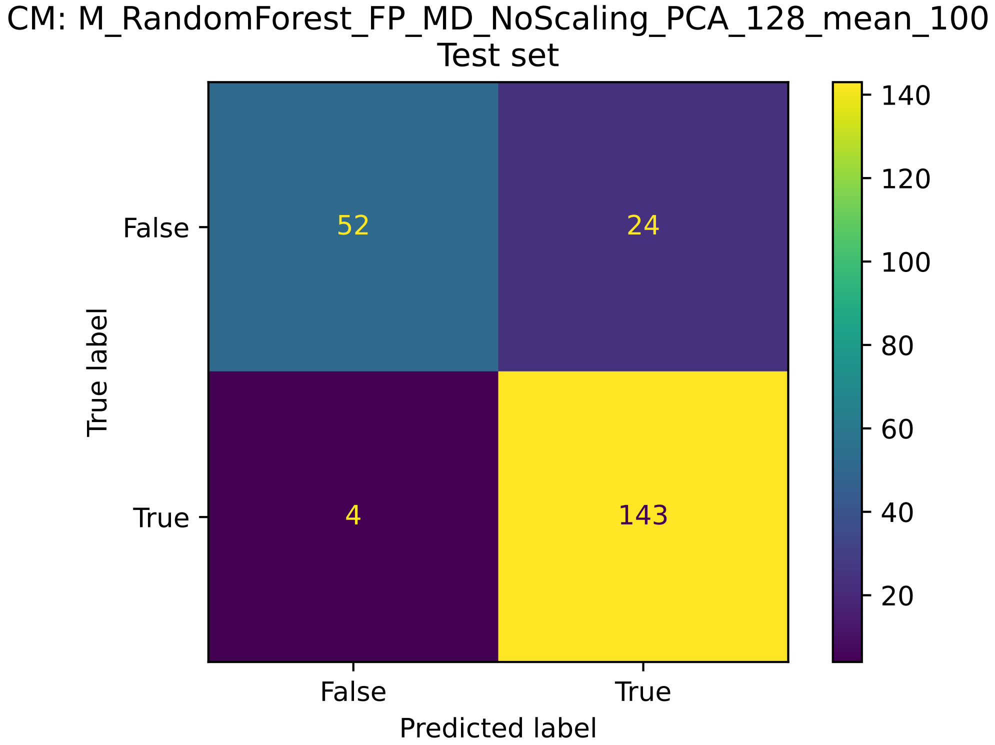
CM: M_RandomForest_FP_MD_NoScaling_PCA_128_mean_100
Test set
True label
Predicted label
False
True
False
True
52
24
4
143
140
120
100
80
60
40
20

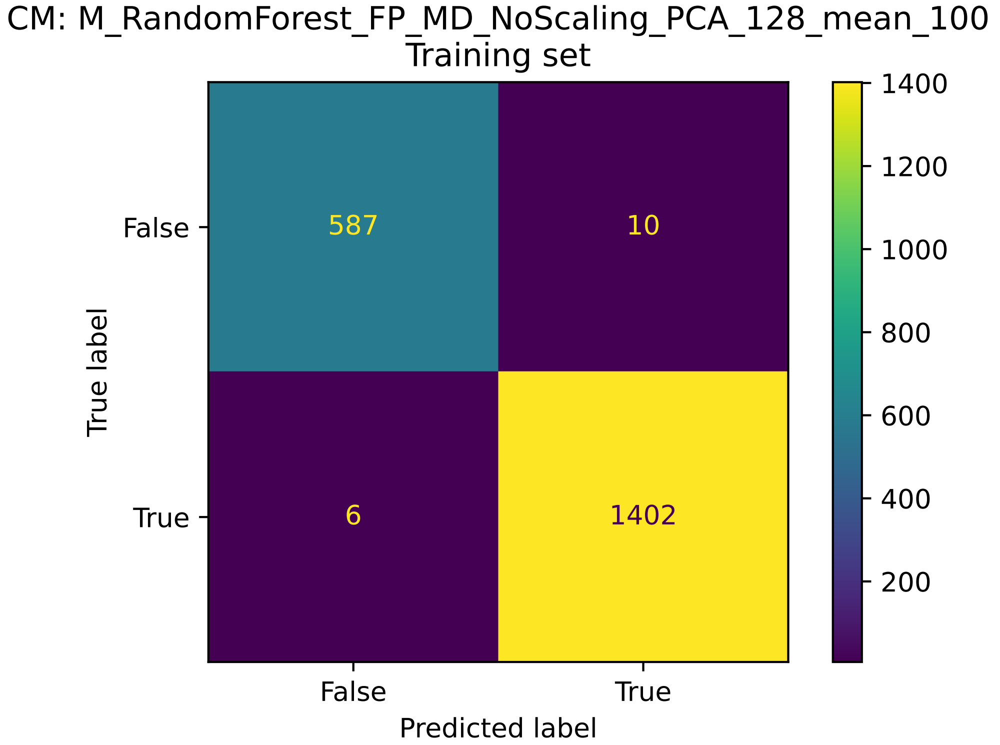
CM: M_RandomForest_FP_MD_NoScaling_PCA_128_mean_100
Training set
587
10
6
1402
False
True
True label
False
True
Predicted label
1400
1200
1000
800
600
400
200

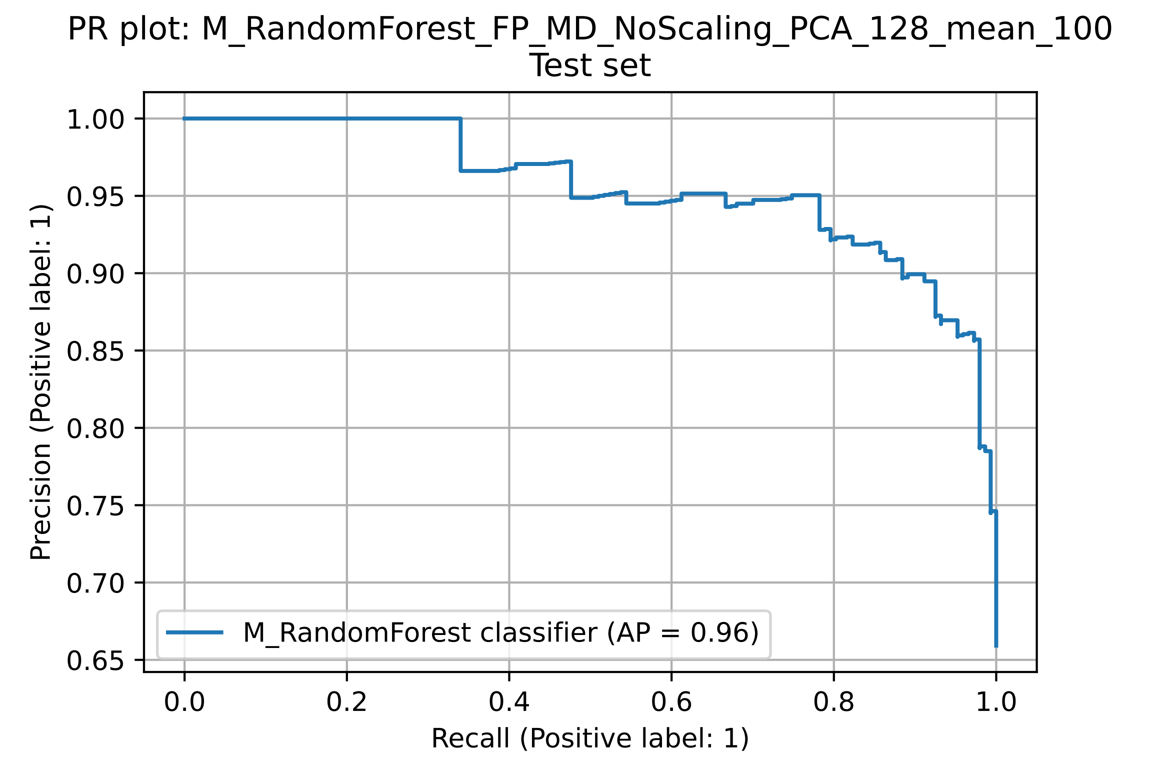
PR plot: M_RandomForest_FP_MD_NoScaling_PCA_128_mean_100
Test set
Precision (Positive label: 1)
Recall (Positive label: 1)
1.00
0.95
0.90
0.85
0.80
0.75
0.70
0.65
0.0
0.2
0.4
0.6
0.8
1.0
M_RandomForest classifier (AP = 0.96)

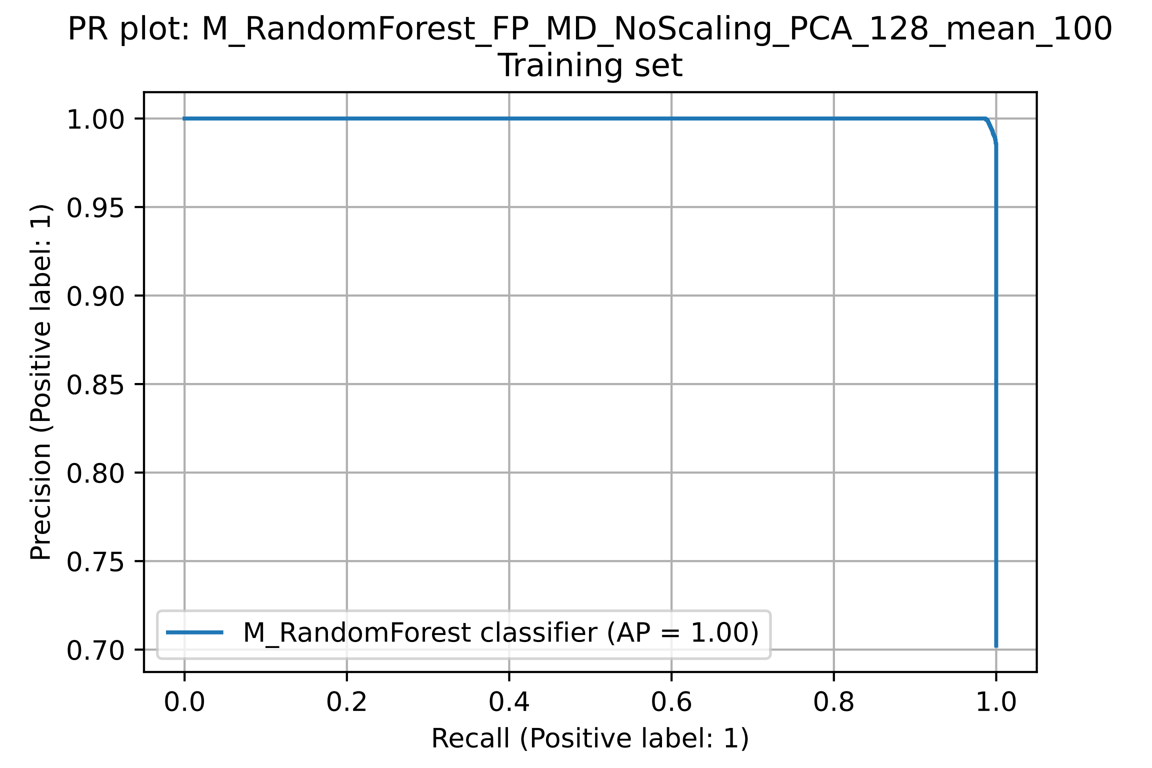

PR plot: M_RandomForest_FP_MD_NoScaling_PCA_128_mean_100
Training set
1.00
0.95
0.90
0.85
0.80
0.75
0.70
Precision (Positive label: 1)
0.0
0.2
0.4
0.6
0.8
1.0
Recall (Positive label: 1)
M_RandomForest classifier (AP = 1.00)

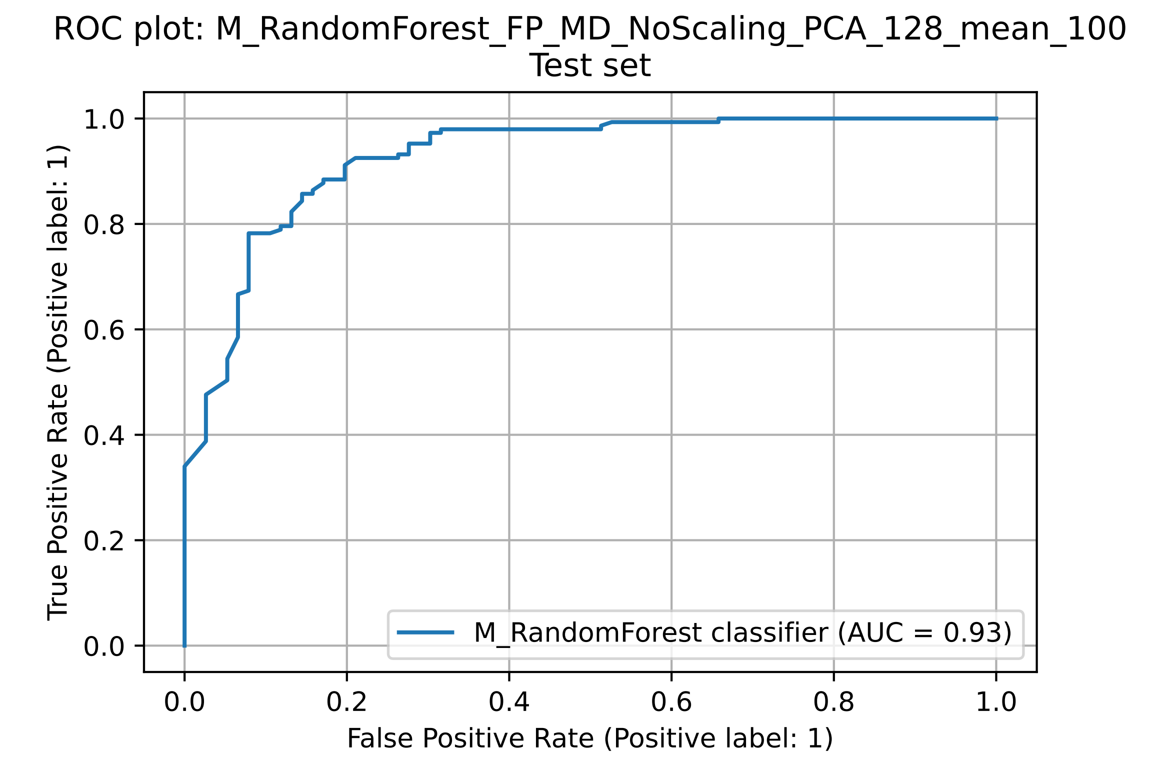
ROC plot: M_RandomForest_FP_MD_NoScaling_PCA_128_mean_100
Test set
True Positive Rate (Positive label: 1)
False Positive Rate (Positive label: 1)
0.0
0.2
0.4
0.6
0.8
1.0
M_RandomForest classifier (AUC = 0.93)

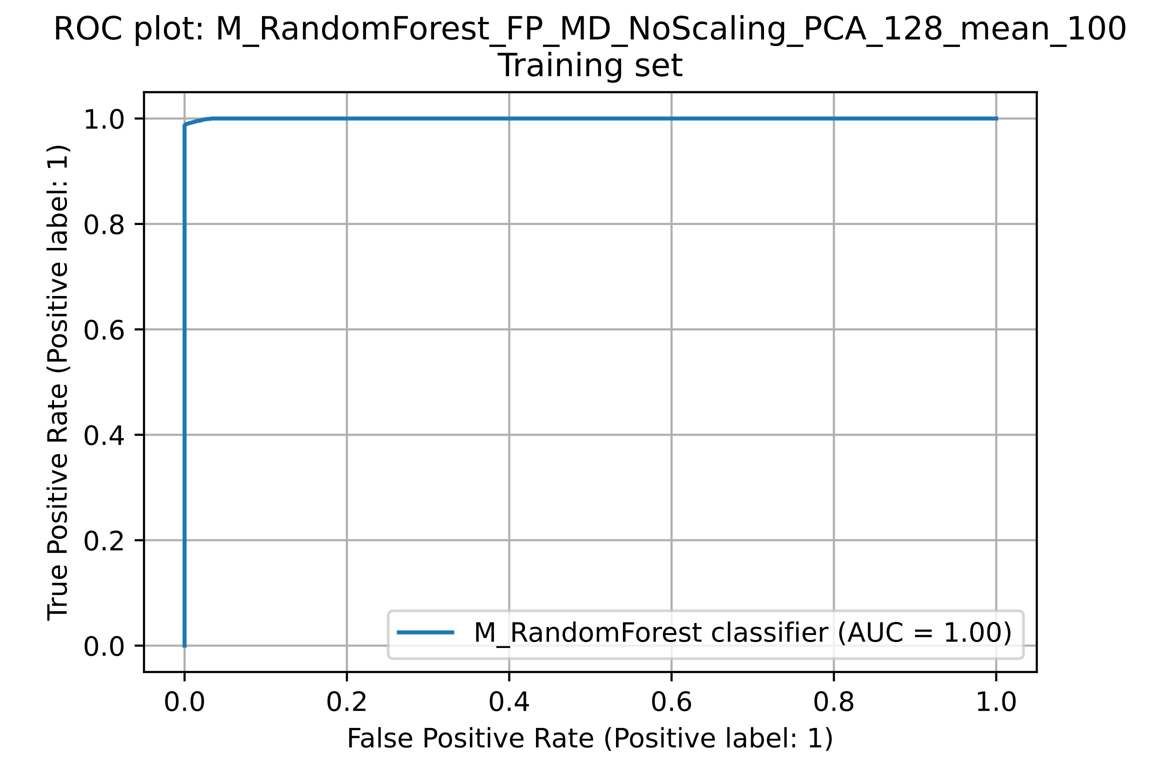

ROC plot: M_RandomForest_FP_MD_NoScaling_PCA_128_mean_100
Training set
True Positive Rate (Positive label: 1)
False Positive Rate (Positive label: 1)
0.0
0.2
0.4
0.6
0.8
1.0
M_RandomForest classifier (AUC = 1.00)

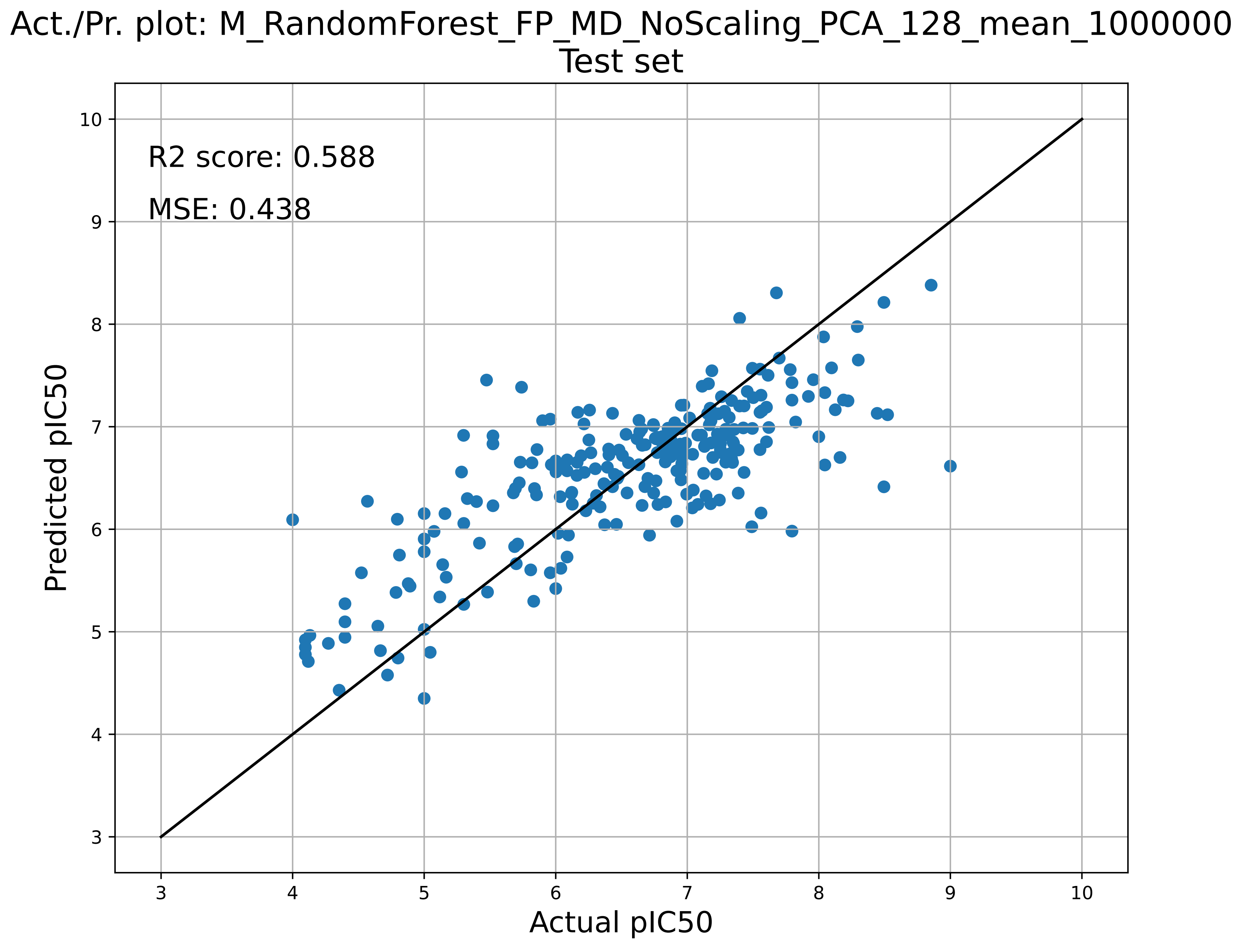

Act./Pr. plot: M_RandomForest_FP_MD_NoScaling_PCA_128_mean_1000000
Test set
R2 score: 0.588
MSE: 0.438
Predicted pIC50
Actual pIC50
3
4
5
6
7
8
9
10
3
4
5
6
7
8
9
10

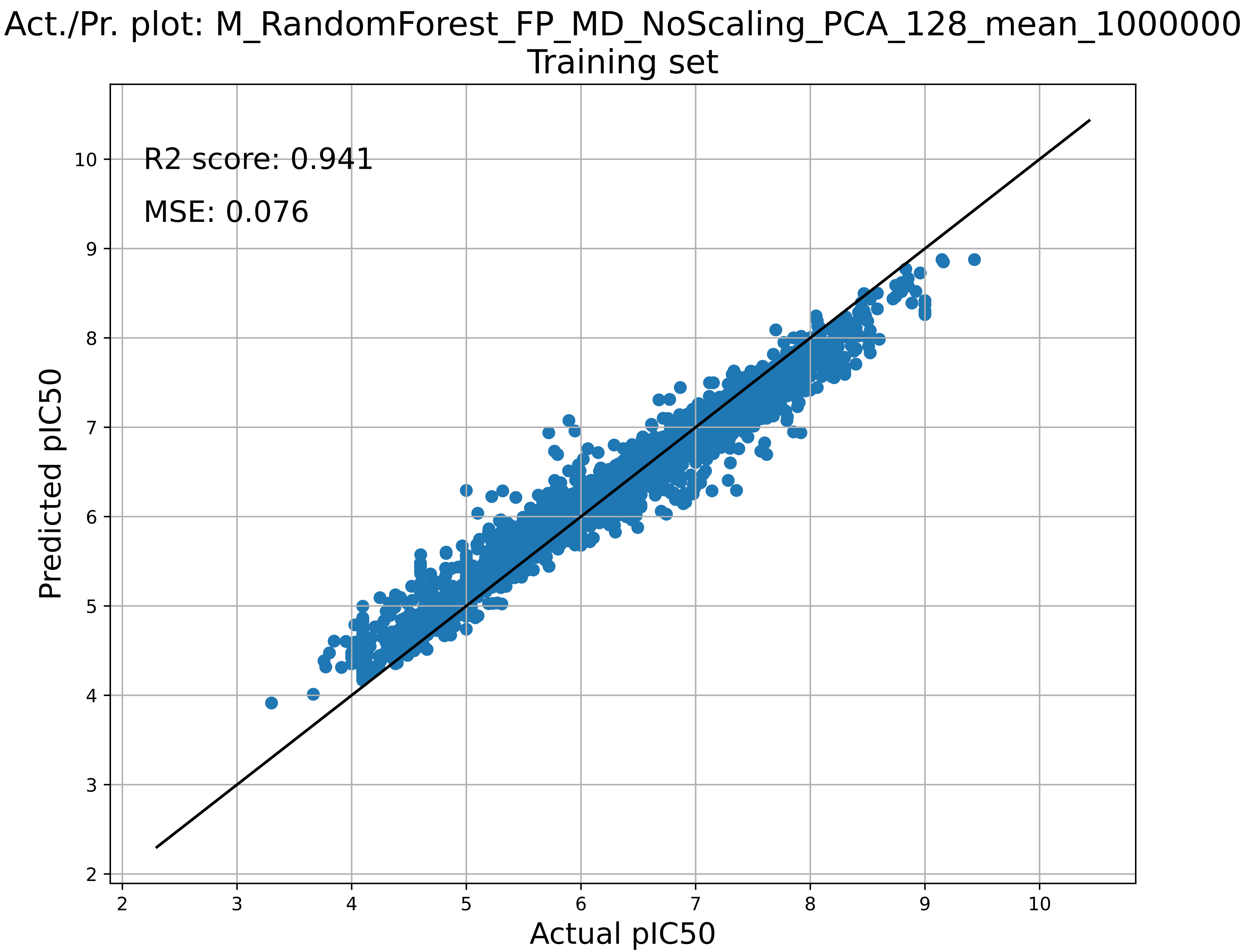

Act./Pr. plot: M_RandomForest_FP_MD_NoScaling_PCA_128_mean_1000000
Training set
R2 score: 0.941
MSE: 0.076
Predicted pIC50
Actual pIC50
2
3
4
5
6
7
8
9
10

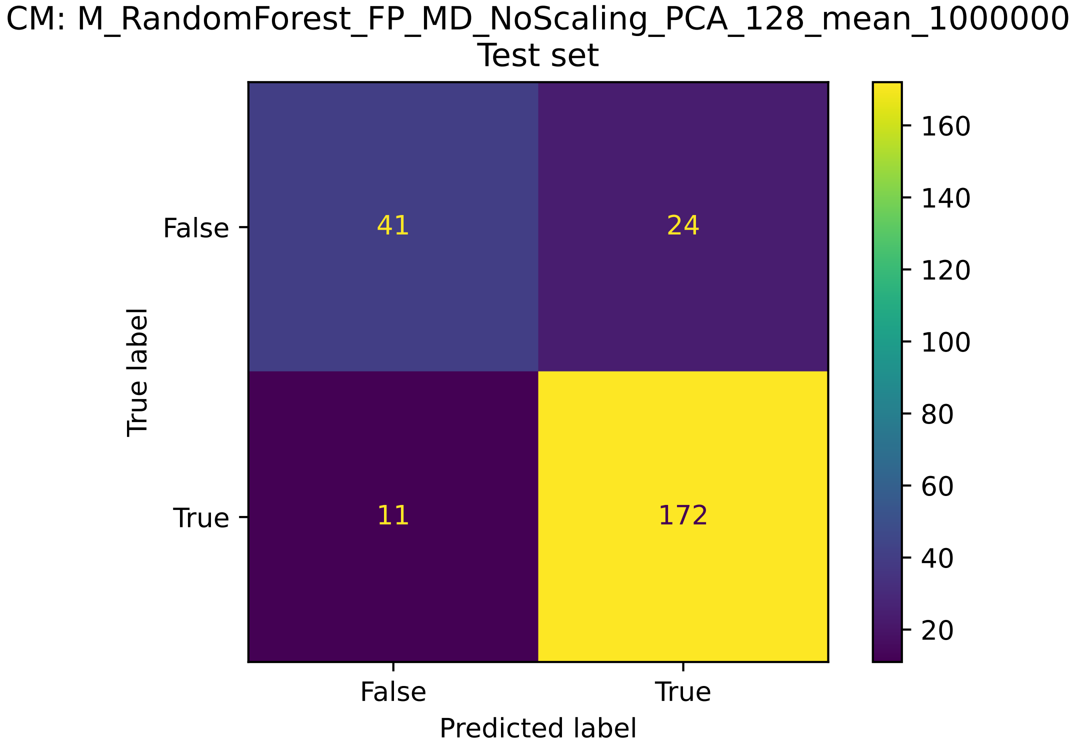
CM: M_RandomForest_FP_MD_NoScaling_PCA_128_mean_1000000
Test set
True label
Predicted label
False
True
False
True
41
24
11
172
160
140
120
100
80
60
40
20

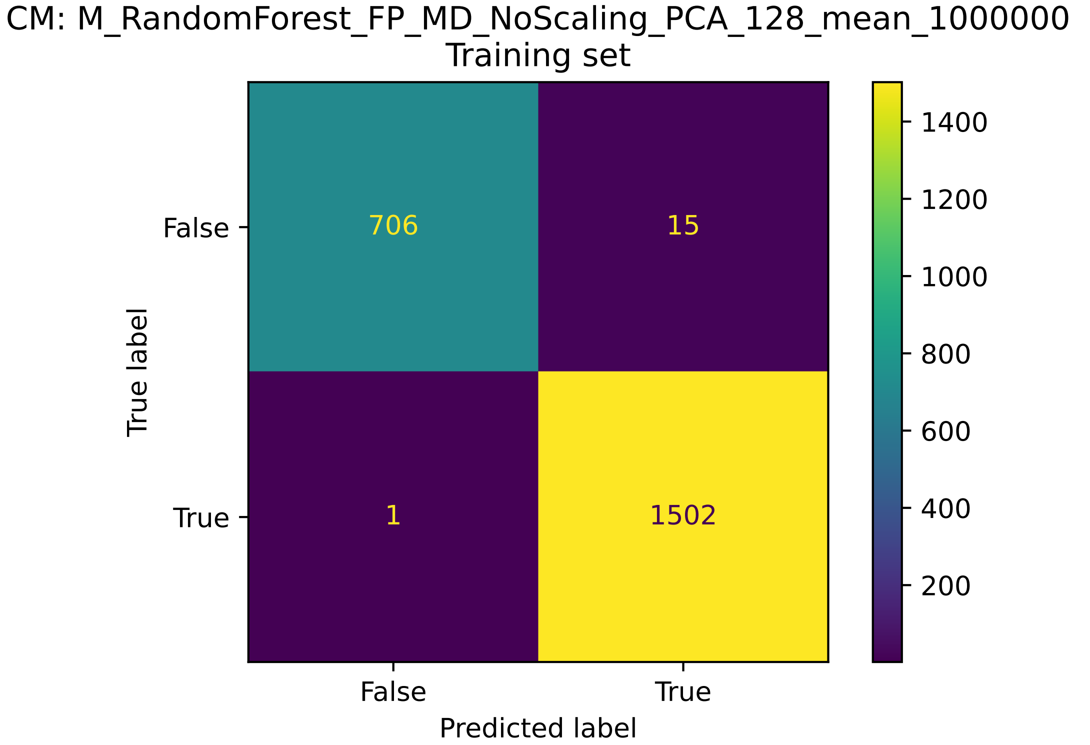
CM: M_RandomForest_FP_MD_NoScaling_PCA_128_mean_1000000
Training set
True label
Predicted label
False
True
False
True
706
15
1
1502
1400
1200
1000
800
600
400
200

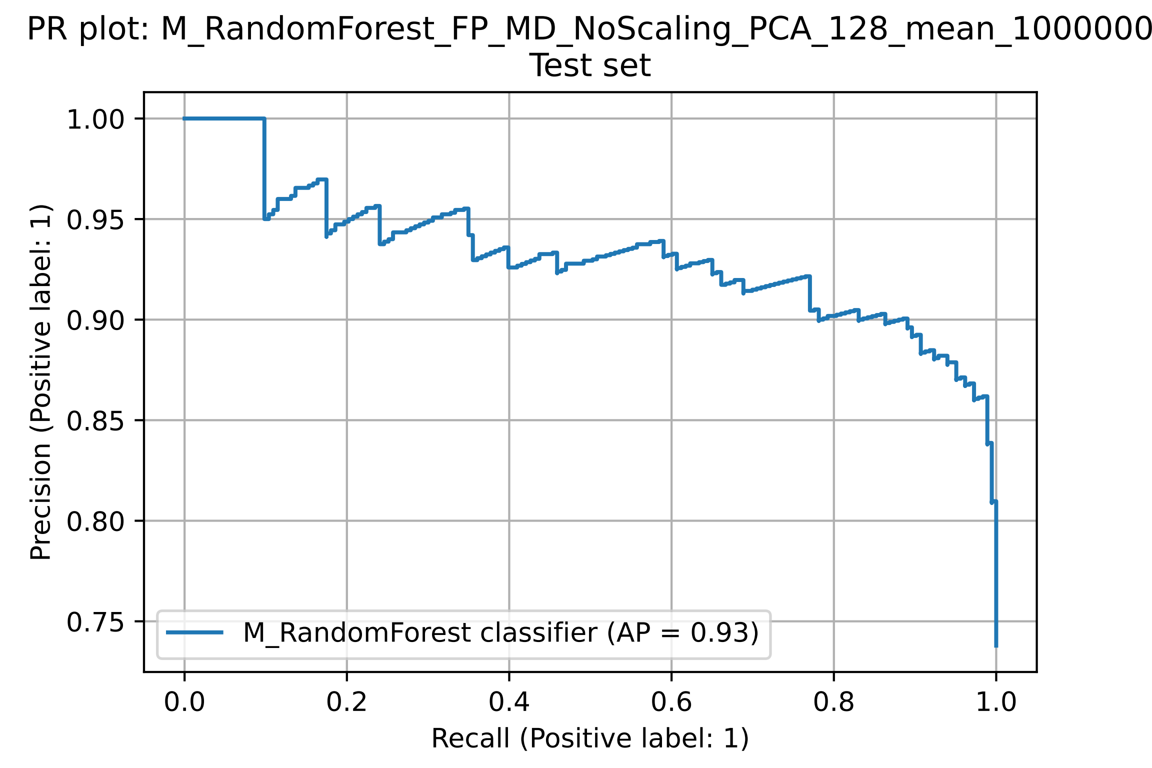
PR plot: M_RandomForest_FP_MD_NoScaling_PCA_128_mean_1000000
Test set
Precision (Positive label: 1)
Recall (Positive label: 1)
1.00
0.95
0.90
0.85
0.80
0.75
0.0
0.2
0.4
0.6
0.8
1.0
M_RandomForest classifier (AP = 0.93)

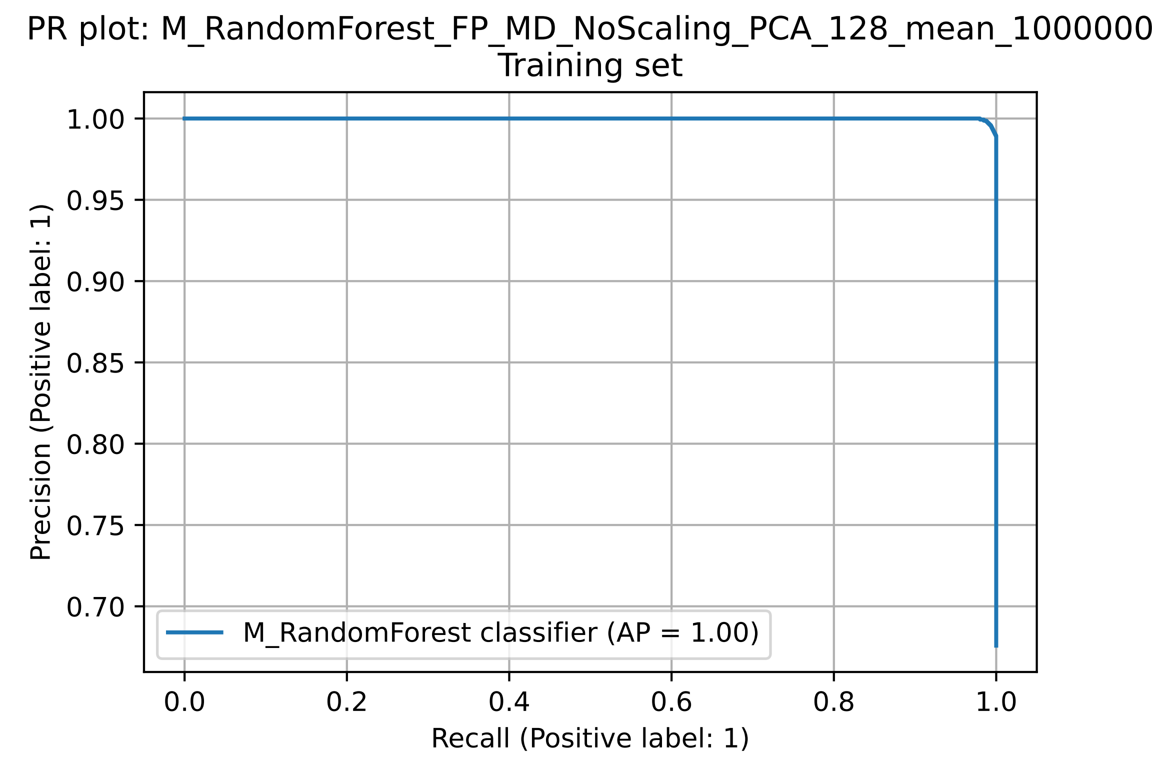
PR plot: M_RandomForest_FP_MD_NoScaling_PCA_128_mean_1000000
Training set
Precision (Positive label: 1)
Recall (Positive label: 1)
1.00
0.95
0.90
0.85
0.80
0.75
0.70
0.0
0.2
0.4
0.6
0.8
1.0
M_RandomForest classifier (AP = 1.00)

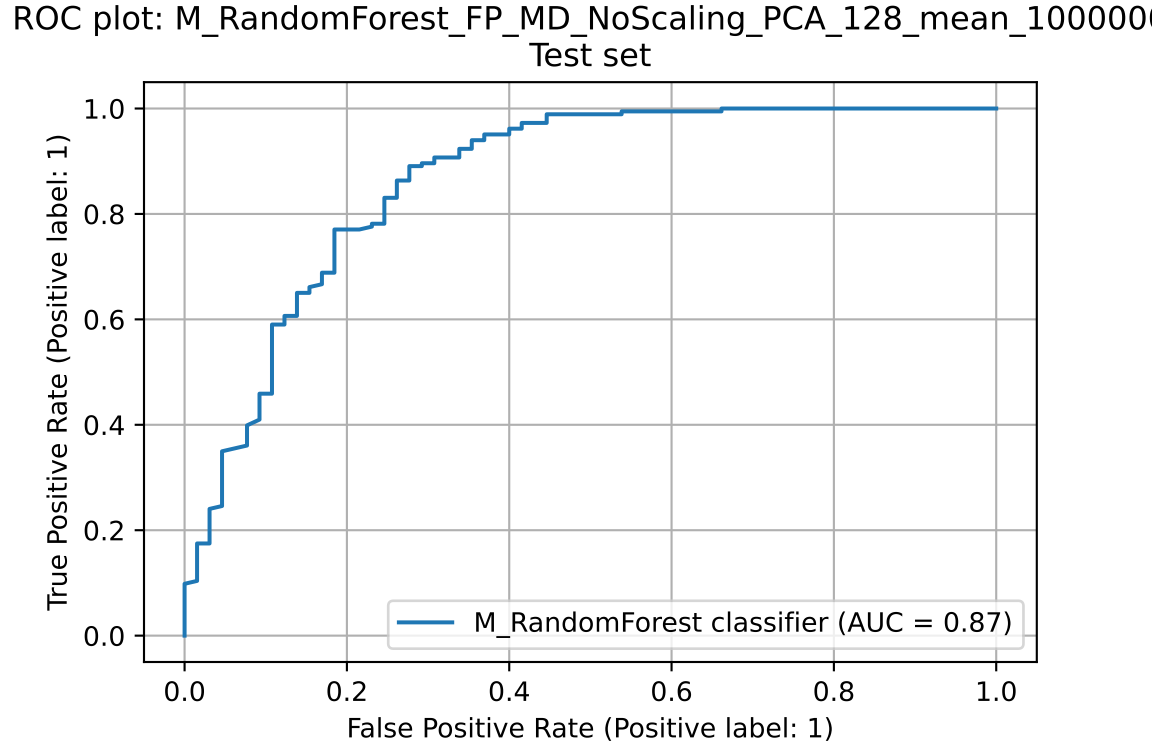
ROC plot: M_RandomForest_FP_MD_NoScaling_PCA_128_mean_100000
Test set
True Positive Rate (Positive label: 1)
False Positive Rate (Positive label: 1)
0.0
0.2
0.4
0.6
0.8
1.0
M_RandomForest classifier (AUC = 0.87)

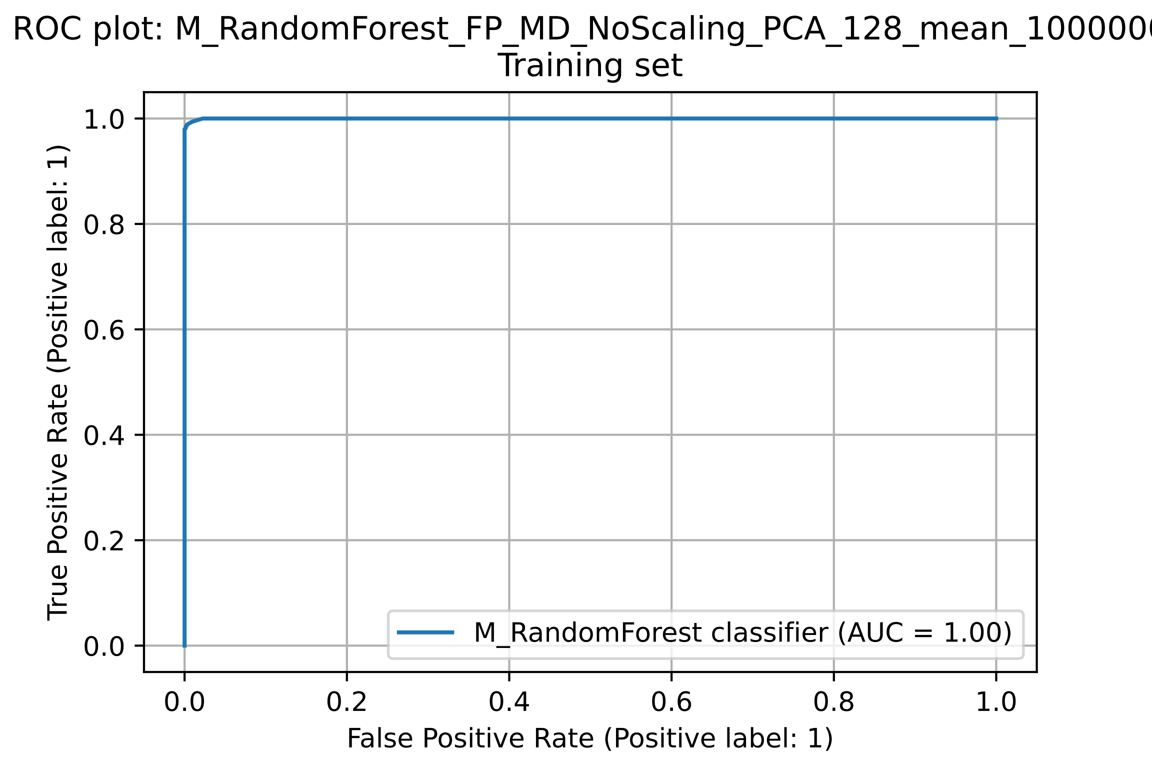
ROC plot: M_RandomForest_FP_MD_NoScaling_PCA_128_mean_100000
Training set
True Positive Rate (Positive label: 1)
False Positive Rate (Positive label: 1)
0.0
0.2
0.4
0.6
0.8
1.0
M_RandomForest classifier (AUC = 1.00)

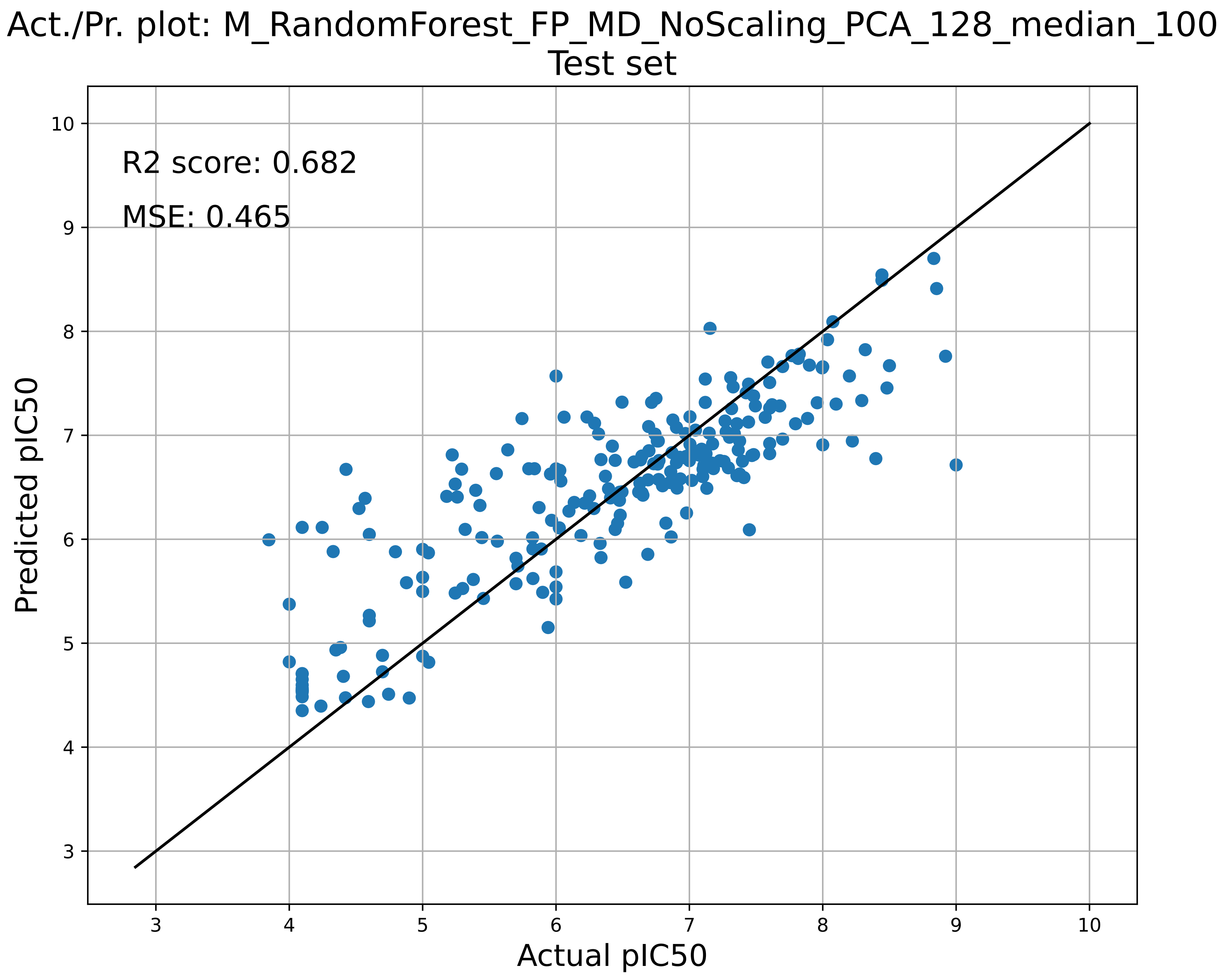

Act./Pr. plot: M_RandomForest_FP_MD_NoScaling_PCA_128_median_100
Test set
R2 score: 0.682
MSE: 0.465
Predicted pIC50
Actual pIC50
3
4
5
6
7
8
9
10

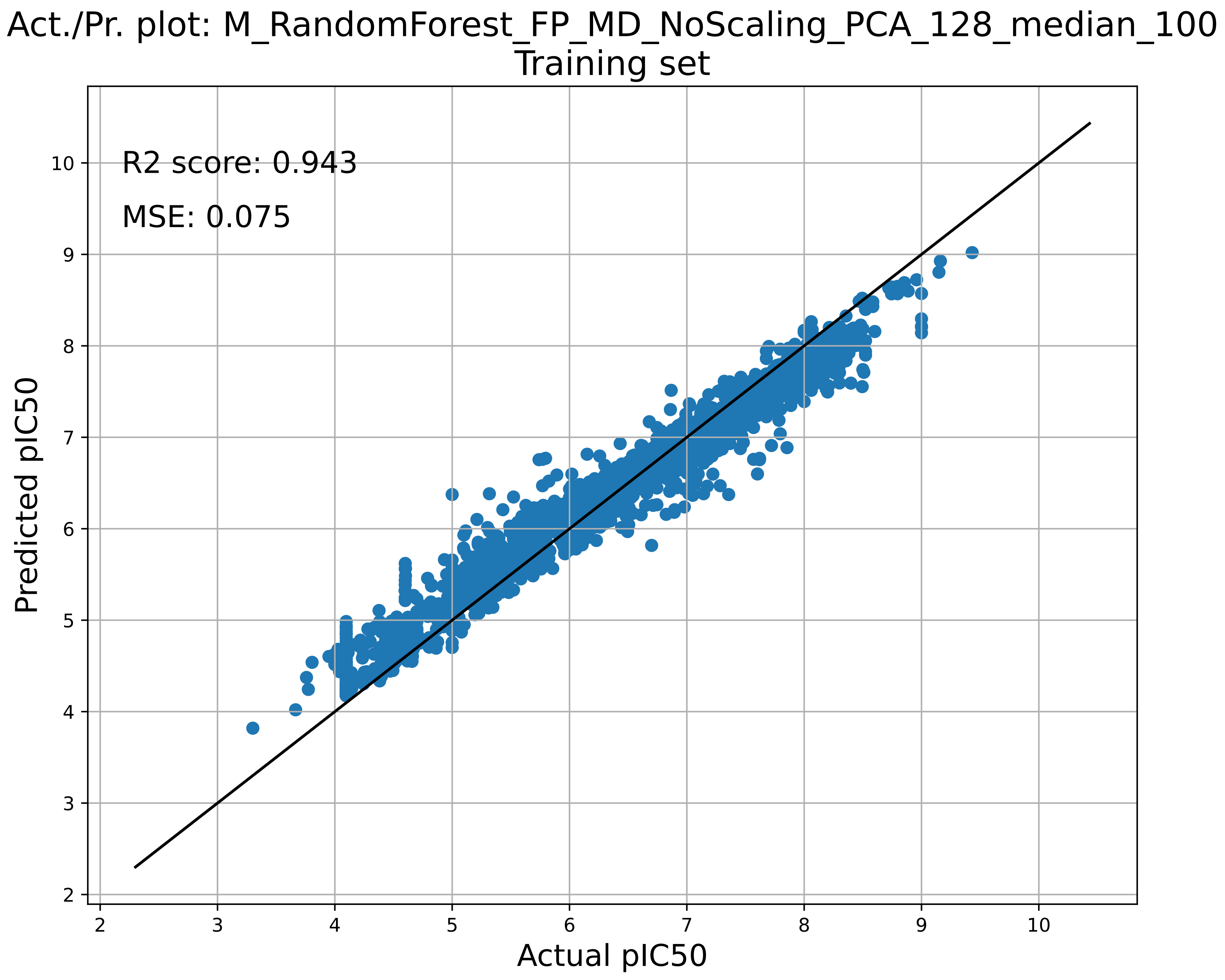

Act./Pr. plot: M_RandomForest_FP_MD_NoScaling_PCA_128_median_100
Training set
R2 score: 0.943
MSE: 0.075
Predicted pIC50
Actual pIC50
2
3
4
5
6
7
8
9
10

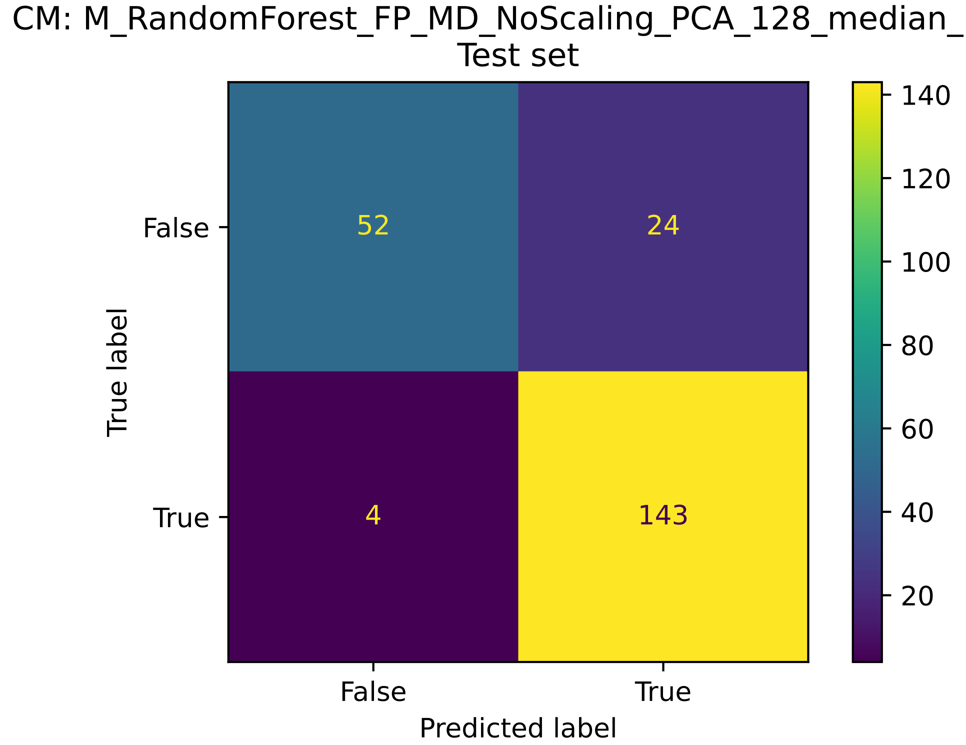

CM: M_RandomForest_FP_MD_NoScaling_PCA_128_median_100
Test set
52
24
4
143
False
True
False
True
True label
Predicted label
140
120
100
80
60
40
20

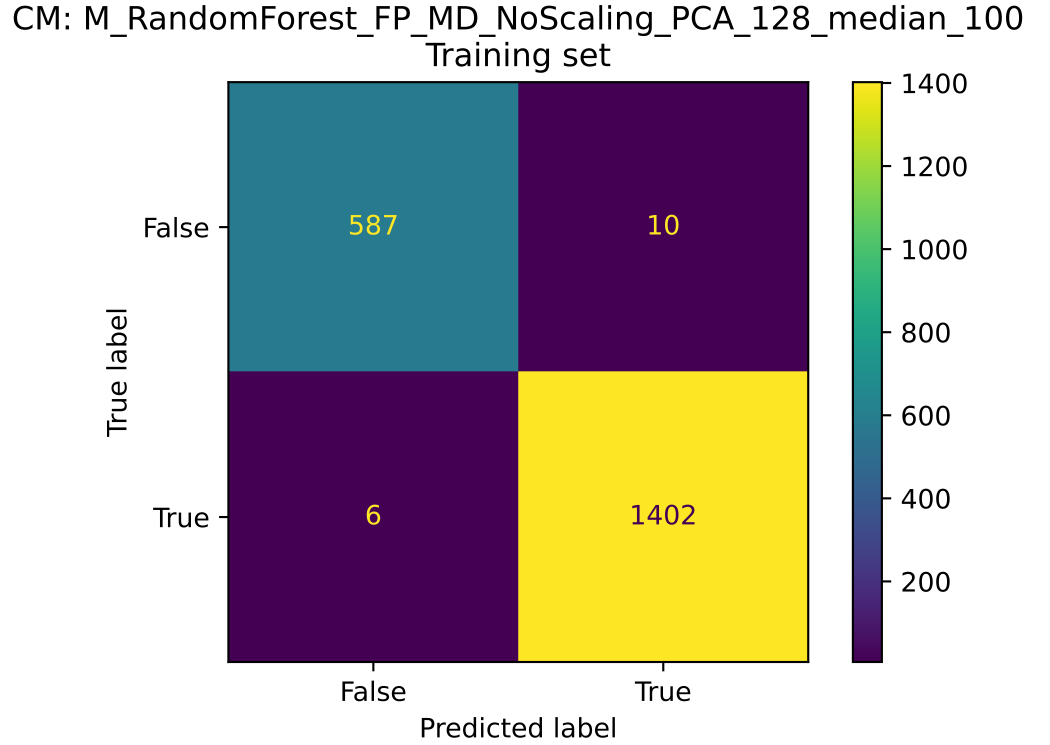
CM: M_RandomForest_FP_MD_NoScaling_PCA_128_median_100
Training set
587
10
6
1402
False
True
False
True
True label
Predicted label
1400
1200
1000
800
600
400
200

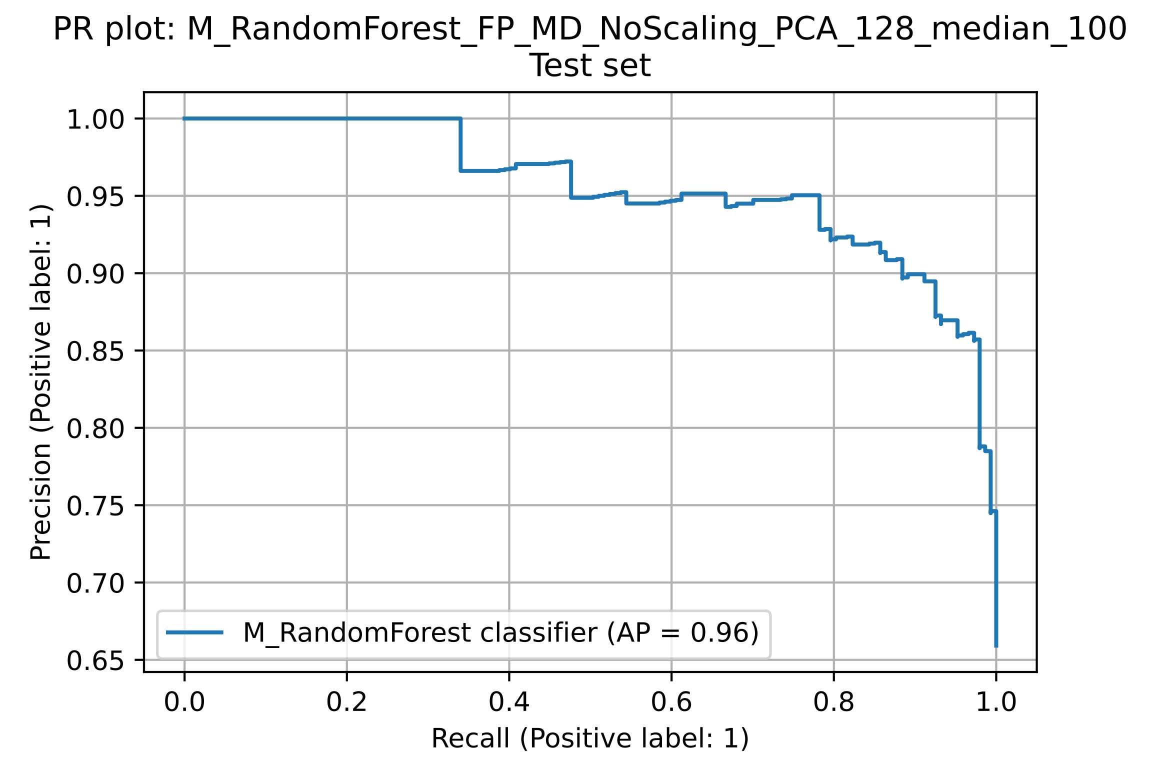
PR plot: M_RandomForest_FP_MD_NoScaling_PCA_128_median_100
Test set
Precision (Positive label: 1)
Recall (Positive label: 1)
1.00
0.95
0.90
0.85
0.80
0.75
0.70
0.65
0.0
0.2
0.4
0.6
0.8
1.0
M_RandomForest classifier (AP = 0.96)

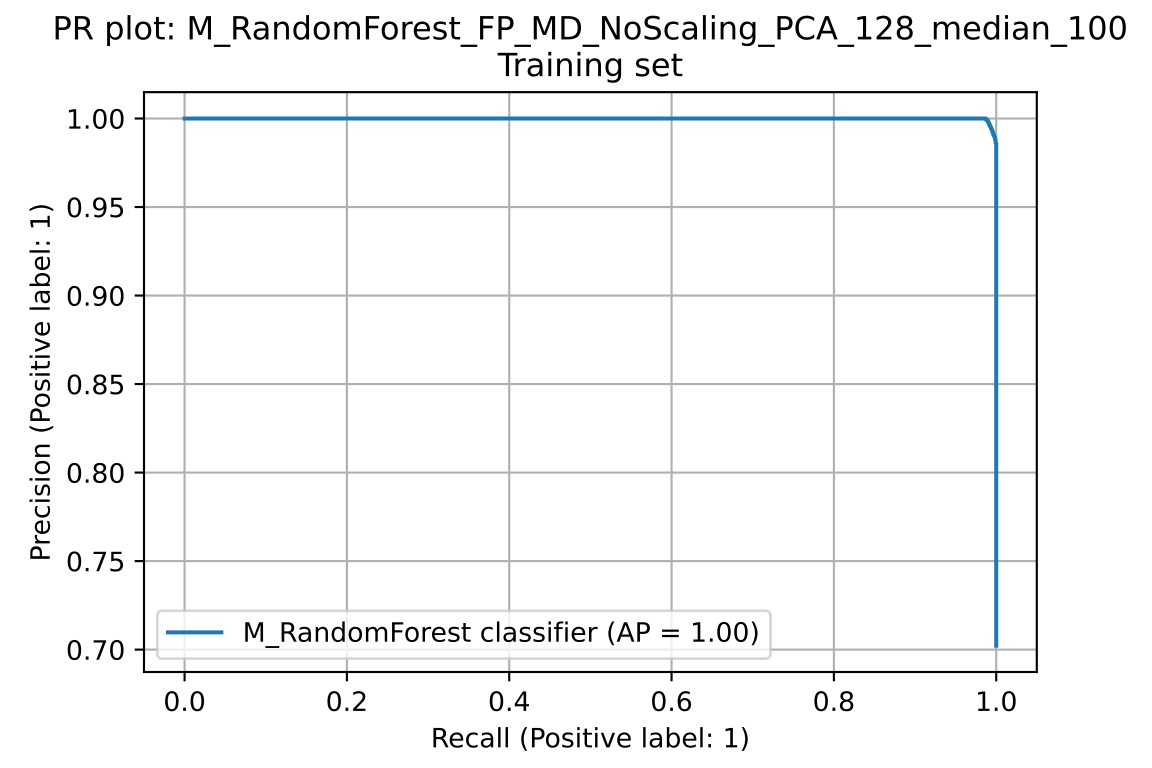
PR plot: M_RandomForest_FP_MD_NoScaling_PCA_128_median_100
Training set
1.00
0.95
0.90
0.85
0.80
0.75
0.70
Precision (Positive label: 1)
0.0
0.2
0.4
0.6
0.8
1.0
Recall (Positive label: 1)
M_RandomForest classifier (AP = 1.00)

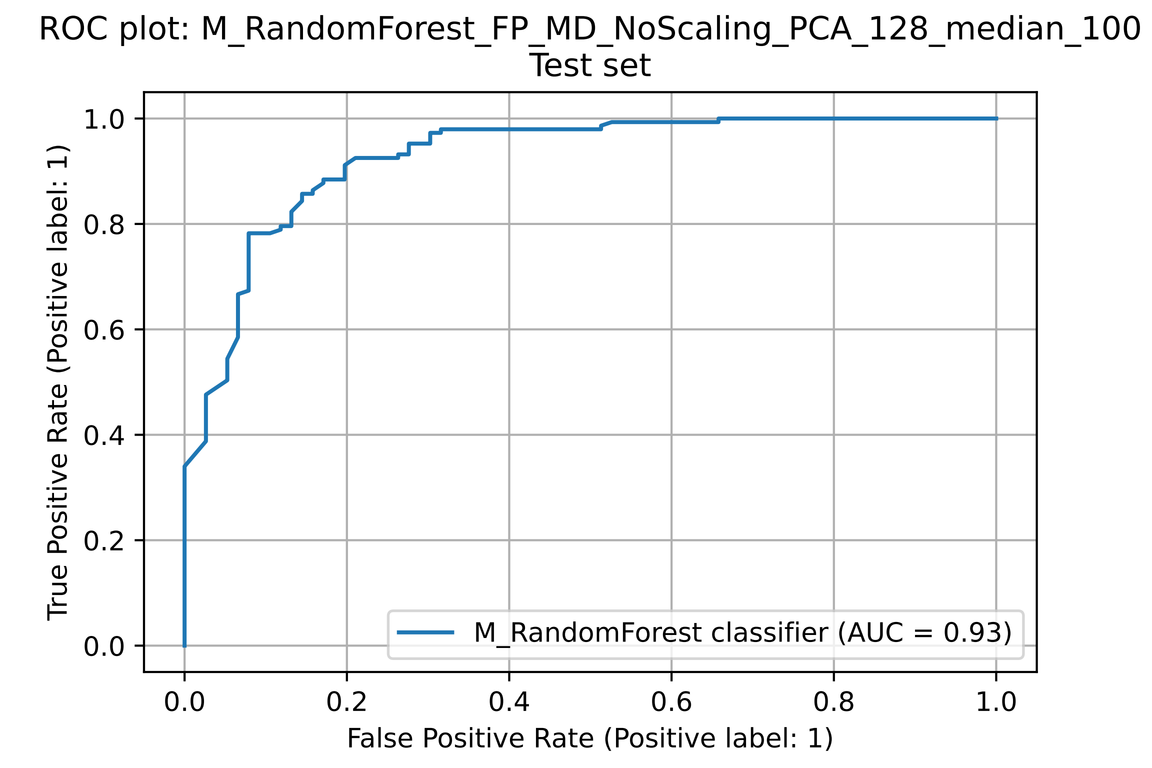
ROC plot: M_RandomForest_FP_MD_NoScaling_PCA_128_median_100
Test set
True Positive Rate (Positive label: 1)
False Positive Rate (Positive label: 1)
0.0
0.2
0.4
0.6
0.8
1.0
M_RandomForest classifier (AUC = 0.93)

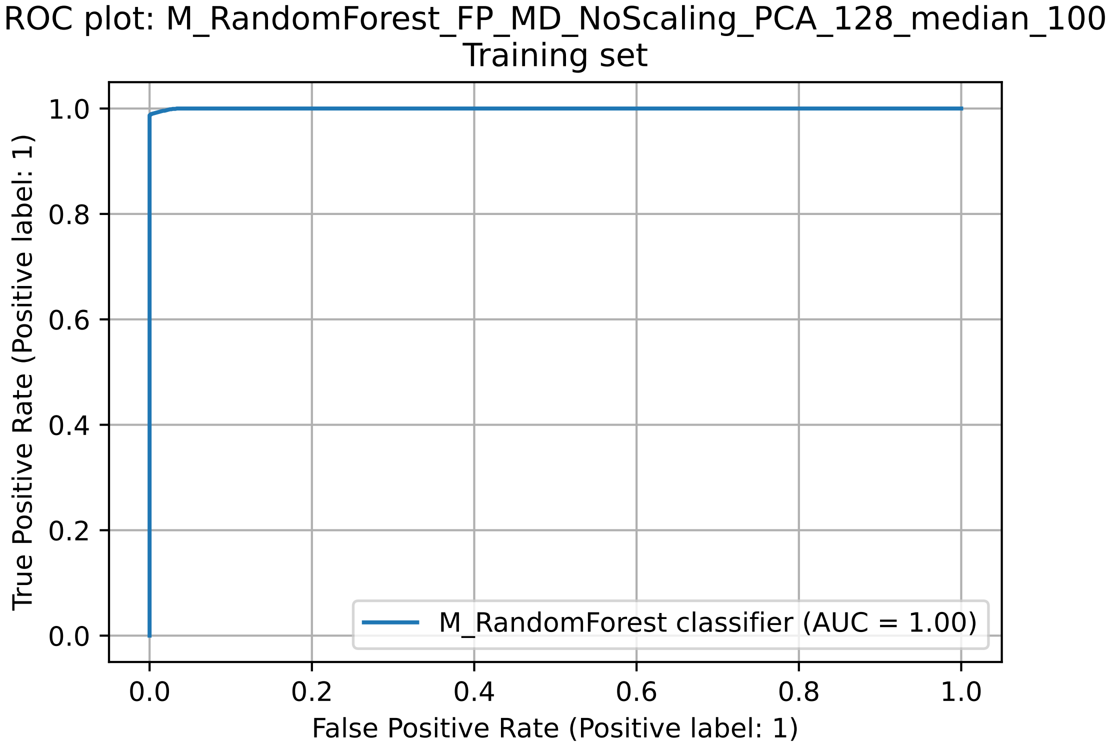
ROC plot: M_RandomForest_FP_MD_NoScaling_PCA_128_median_100
Training set
True Positive Rate (Positive label: 1)
False Positive Rate (Positive label: 1)
0.0
0.2
0.4
0.6
0.8
1.0
M_RandomForest classifier (AUC = 1.00)

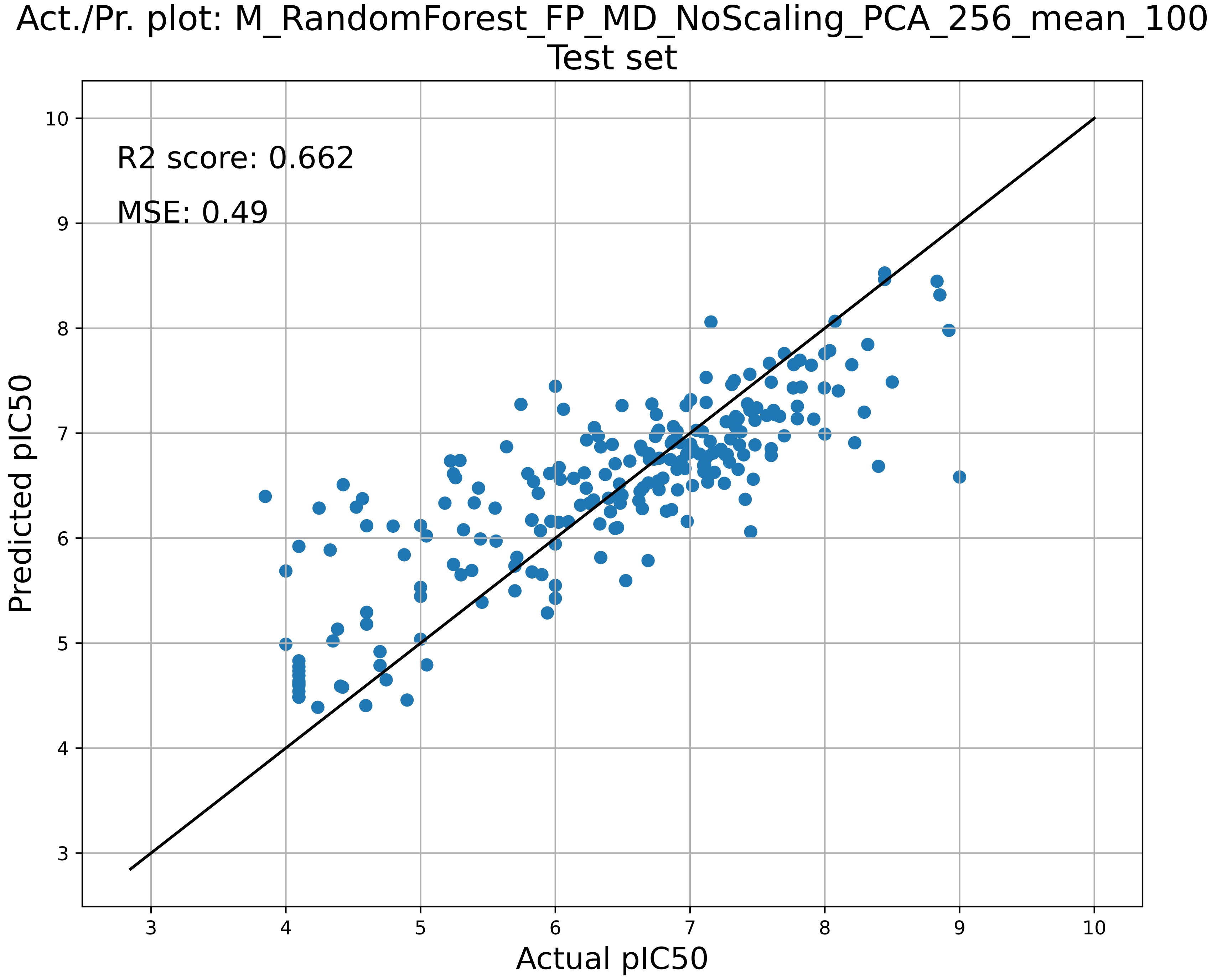

Act./Pr. plot: M_RandomForest_FP_MD_NoScaling_PCA_256_mean_100
Test set
R2 score: 0.662
MSE: 0.49
Predicted pIC50
Actual pIC50
3
4
5
6
7
8
9
10

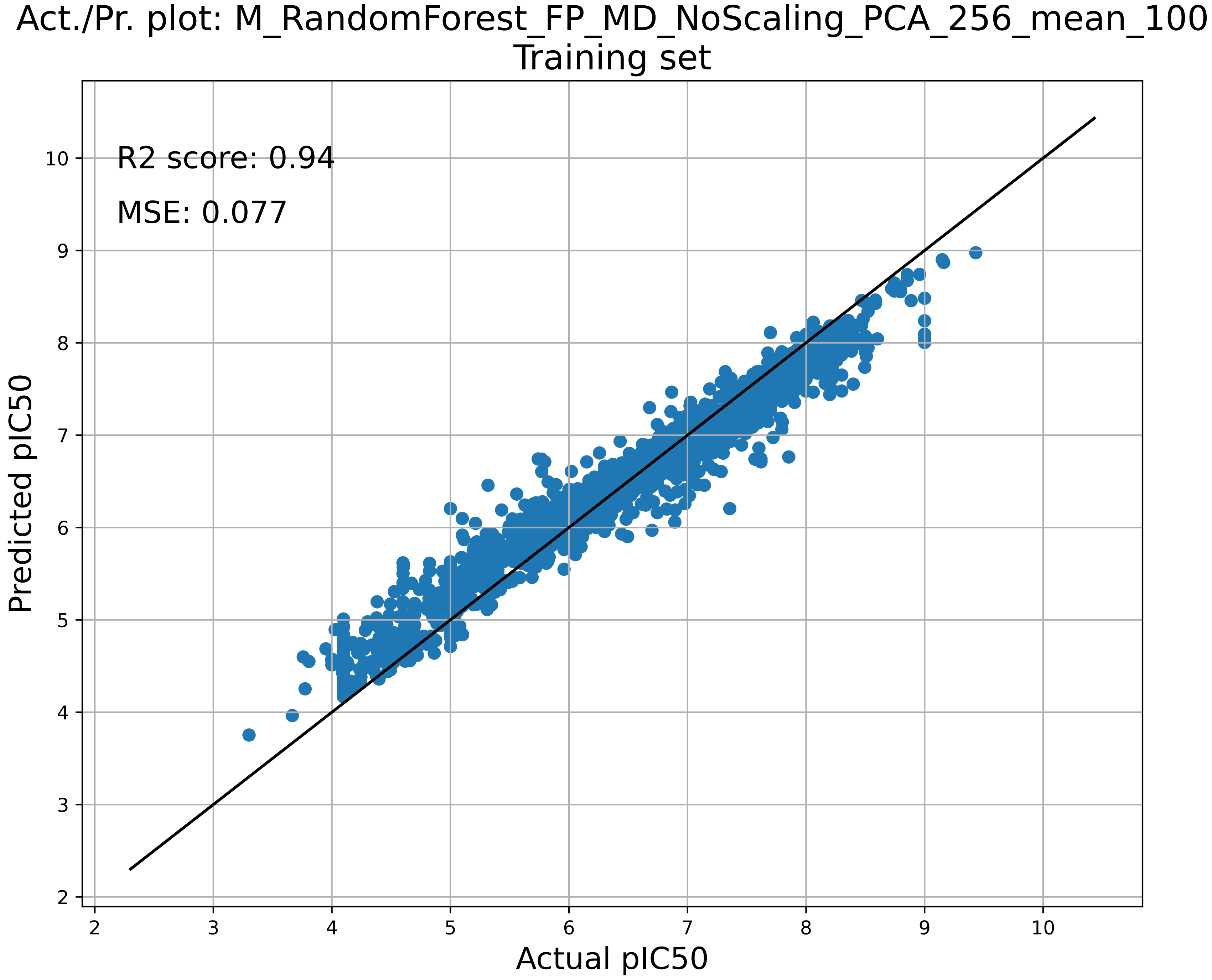

Act./Pr. plot: M_RandomForest_FP_MD_NoScaling_PCA_256_mean_100
Training set
R2 score: 0.94
MSE: 0.077
Predicted pIC50
Actual pIC50
2
3
4
5
6
7
8
9
10

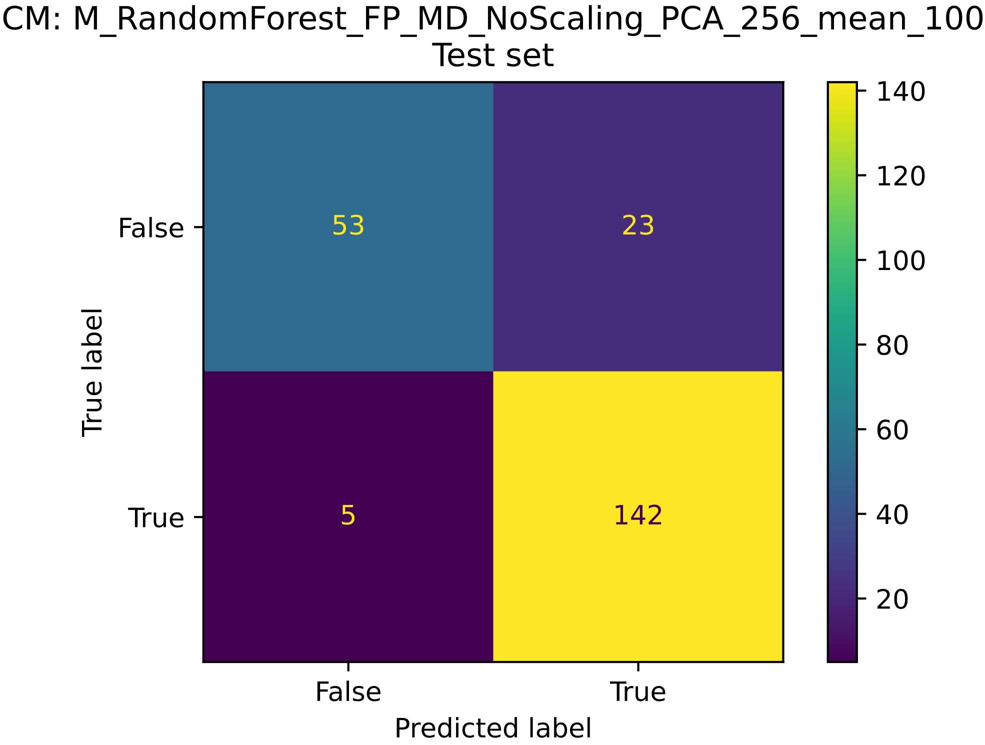
CM: M_RandomForest_FP_MD_NoScaling_PCA_256_mean_100
Test set
53
23
5
142
False
True
True label
False
True
Predicted label
140
120
100
80
60
40
20

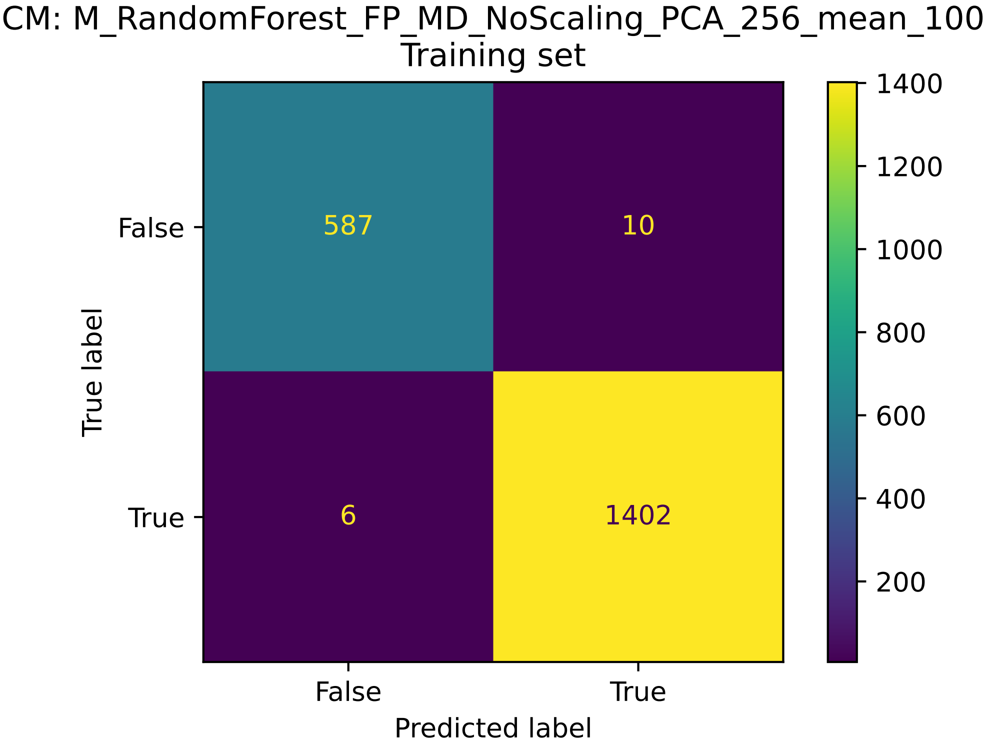
CM: M_RandomForest_FP_MD_NoScaling_PCA_256_mean_100
Training set
587
10
6
1402
False
True
True label
False
True
Predicted label
1400
1200
1000
800
600
400
200

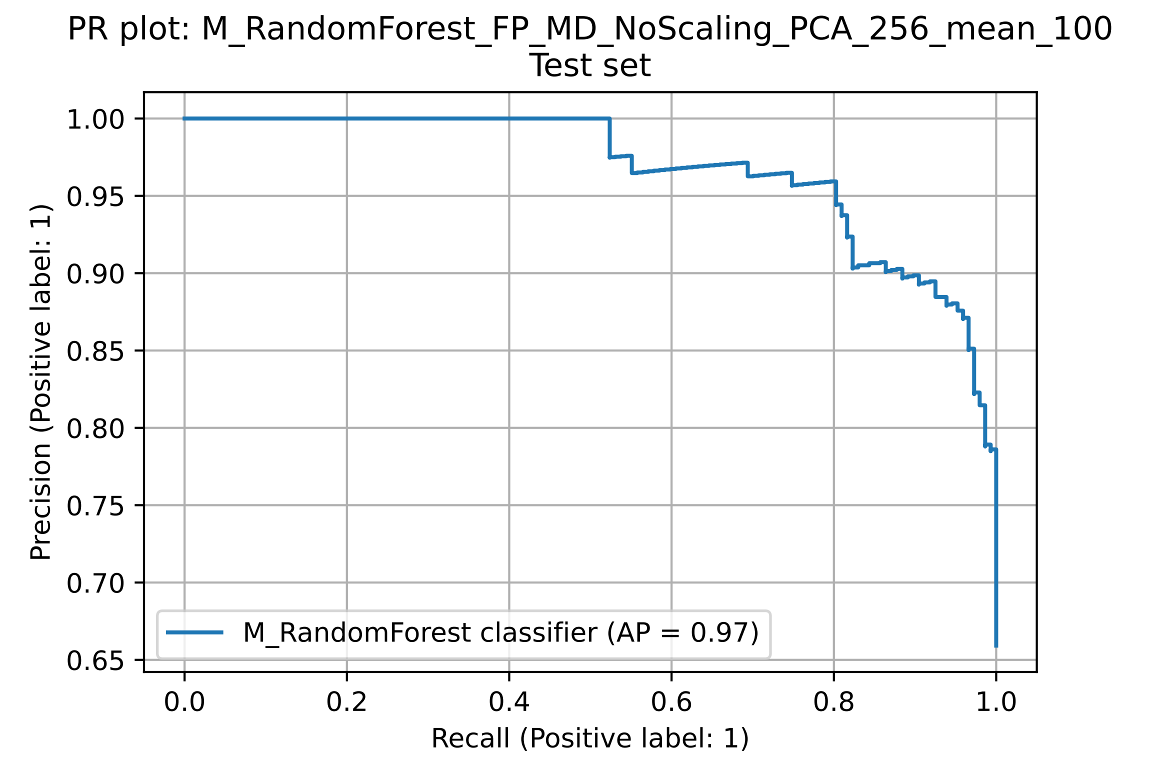
PR plot: M_RandomForest_FP_MD_NoScaling_PCA_256_mean_100
Test set
Precision (Positive label: 1)
Recall (Positive label: 1)
1.00
0.95
0.90
0.85
0.80
0.75
0.70
0.65
0.0
0.2
0.4
0.6
0.8
1.0
M_RandomForest classifier (AP = 0.97)

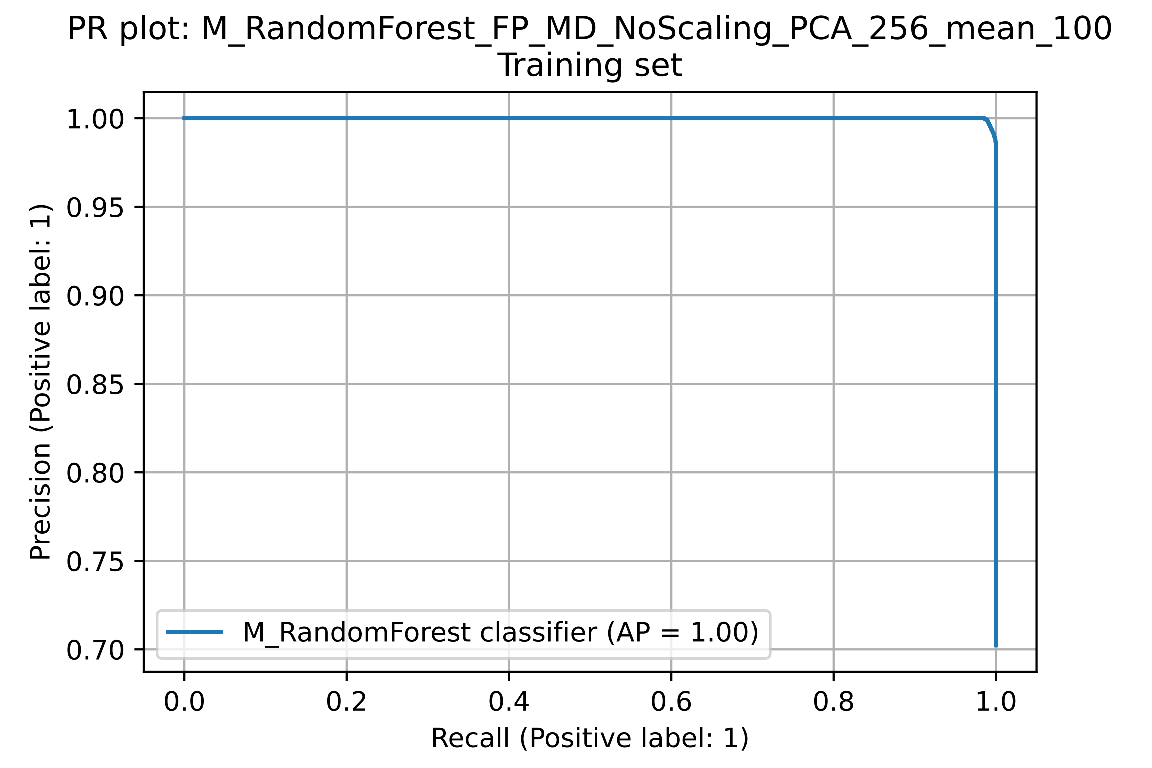
PR plot: M_RandomForest_FP_MD_NoScaling_PCA_256_mean_100
Training set
1.00
0.95
0.90
0.85
0.80
0.75
0.70
Precision (Positive label: 1)
0.0
0.2
0.4
0.6
0.8
1.0
Recall (Positive label: 1)
M_RandomForest classifier (AP = 1.00)

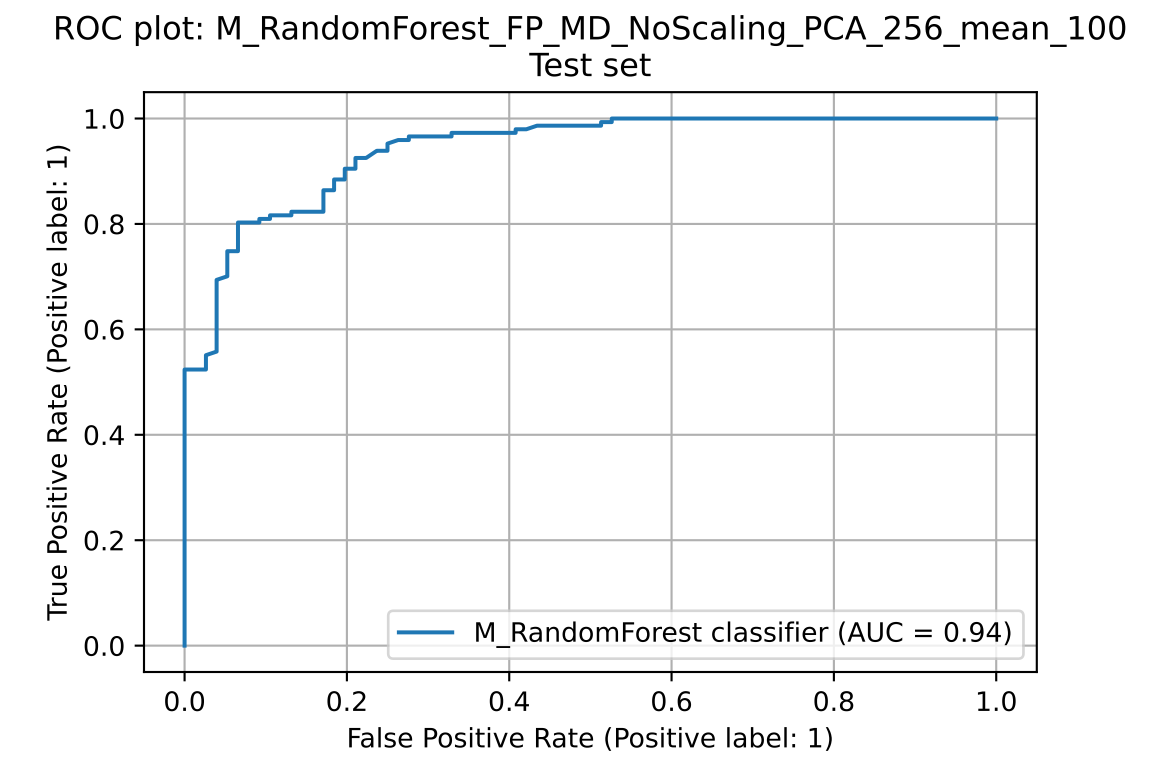

ROC plot: M_RandomForest_FP_MD_NoScaling_PCA_256_mean_100
Test set
True Positive Rate (Positive label: 1)
False Positive Rate (Positive label: 1)
0.0
0.2
0.4
0.6
0.8
1.0
M_RandomForest classifier (AUC = 0.94)

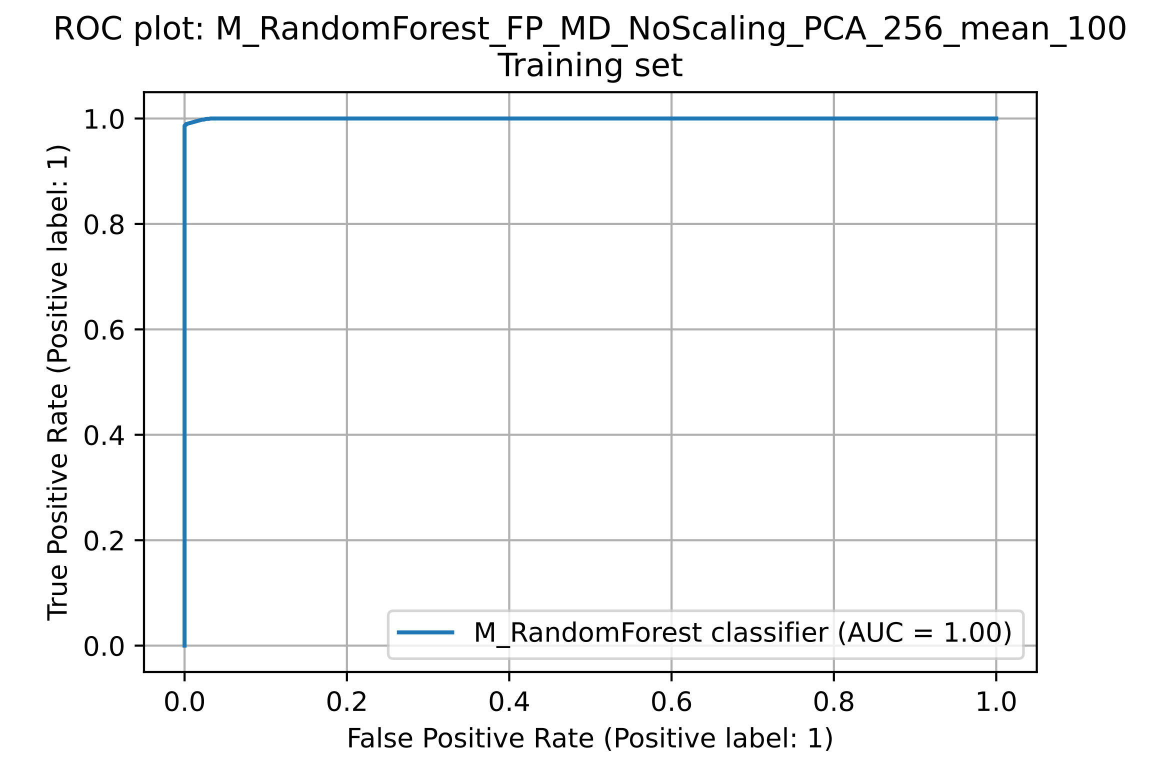
ROC plot: M_RandomForest_FP_MD_NoScaling_PCA_256_mean_100
Training set
True Positive Rate (Positive label: 1)
False Positive Rate (Positive label: 1)
0.0
0.2
0.4
0.6
0.8
1.0
M_RandomForest classifier (AUC = 1.00)

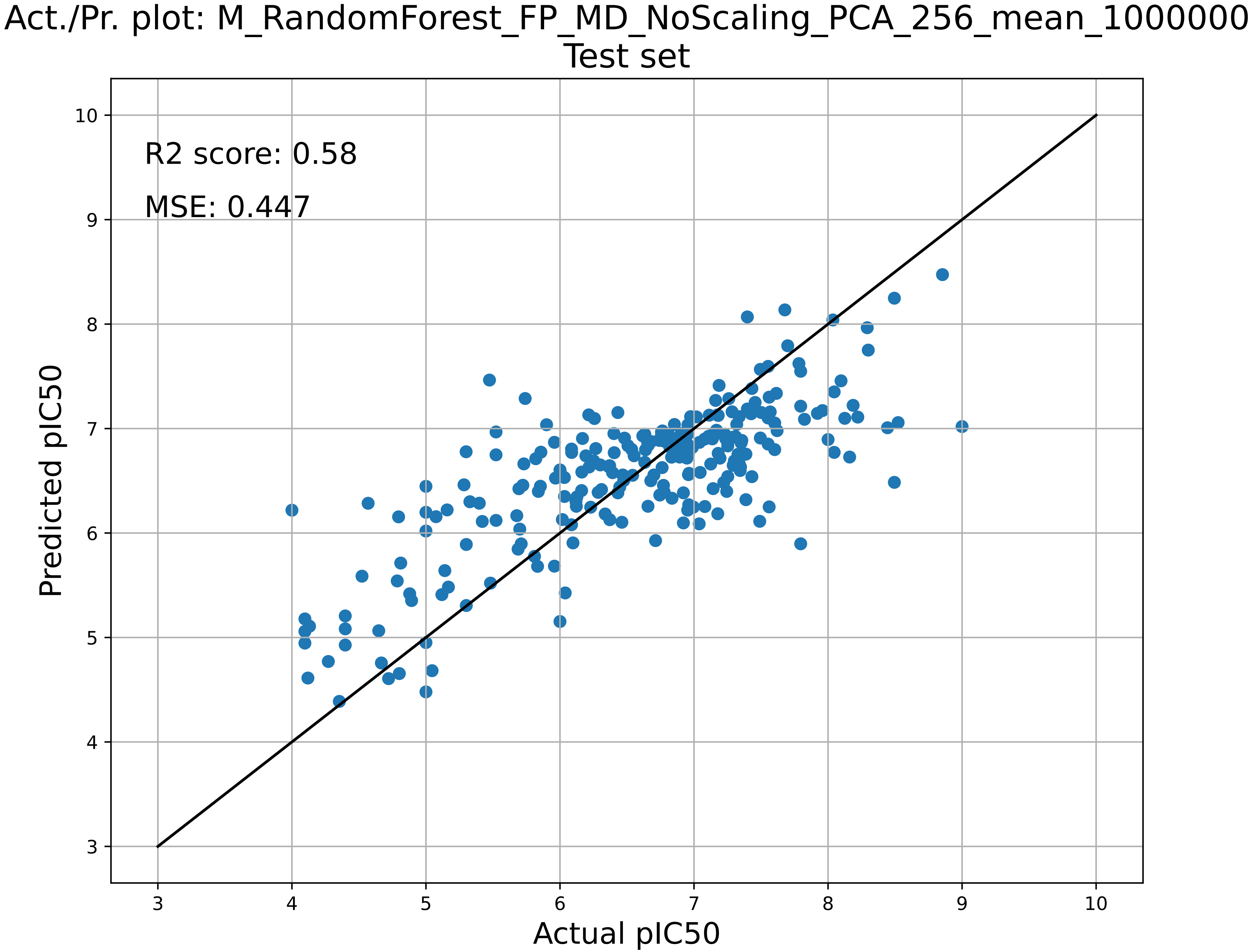

Act./Pr. plot: M_RandomForest_FP_MD_NoScaling_PCA_256_mean_1000000
Test set
R2 score: 0.58
MSE: 0.447
Predicted pIC50
Actual pIC50
3
4
5
6
7
8
9
10

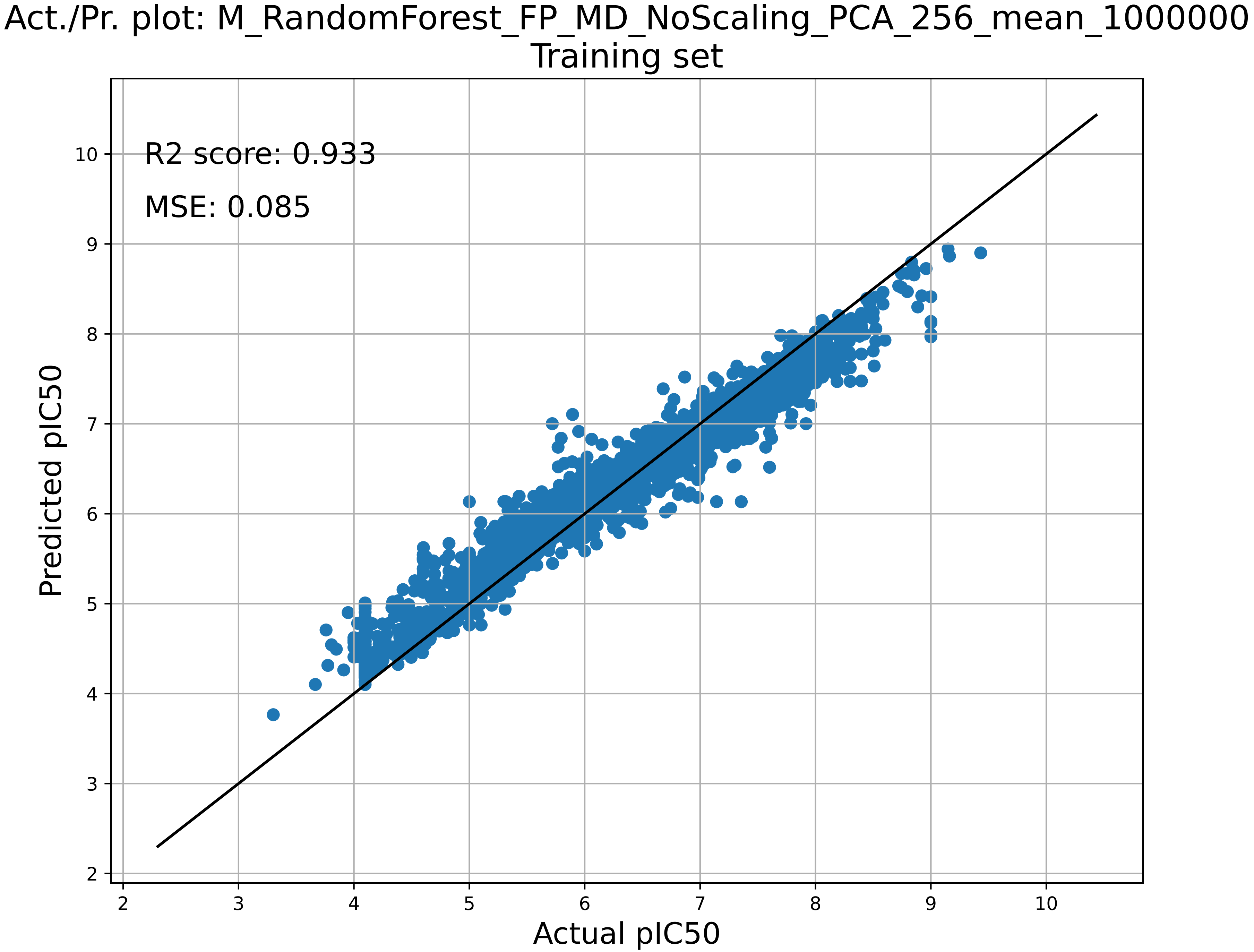
Act./Pr. plot: M_RandomForest_FP_MD_NoScaling_PCA_256_mean_1000000
Training set
R2 score: 0.933
MSE: 0.085
Predicted pIC50
Actual pIC50
2
3
4
5
6
7
8
9
10

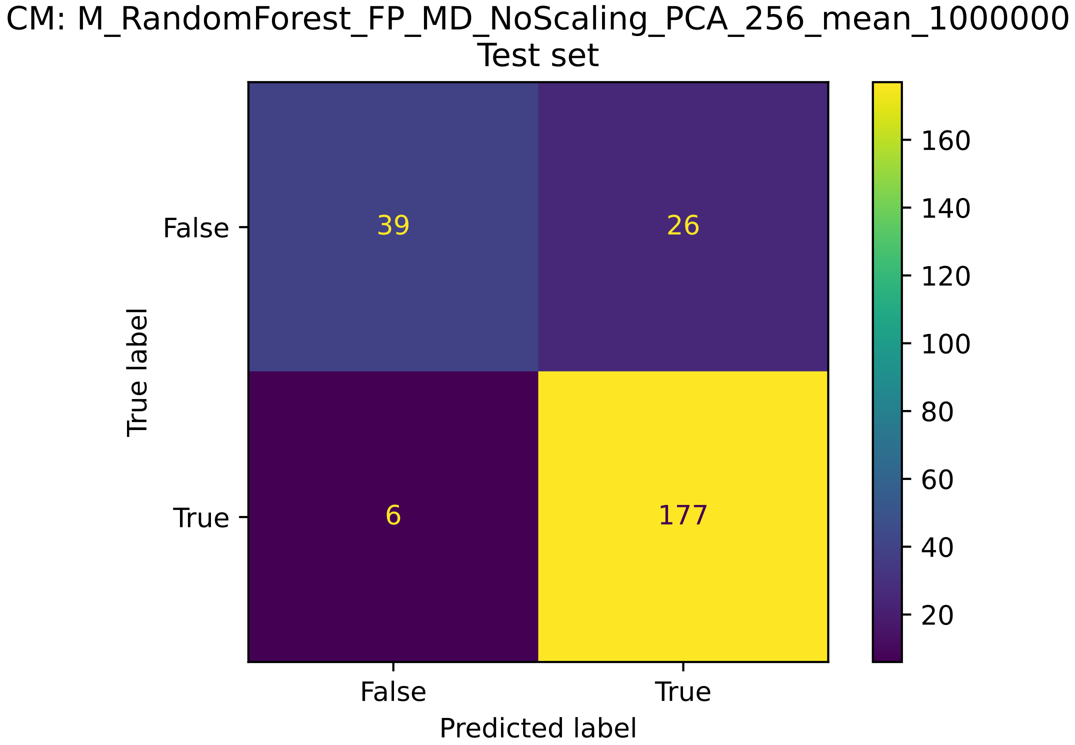
CM: M_RandomForest_FP_MD_NoScaling_PCA_256_mean_1000000
Test set
True label
False
True
39
26
6
177
Predicted label
False
True
160
140
120
100
80
60
40
20

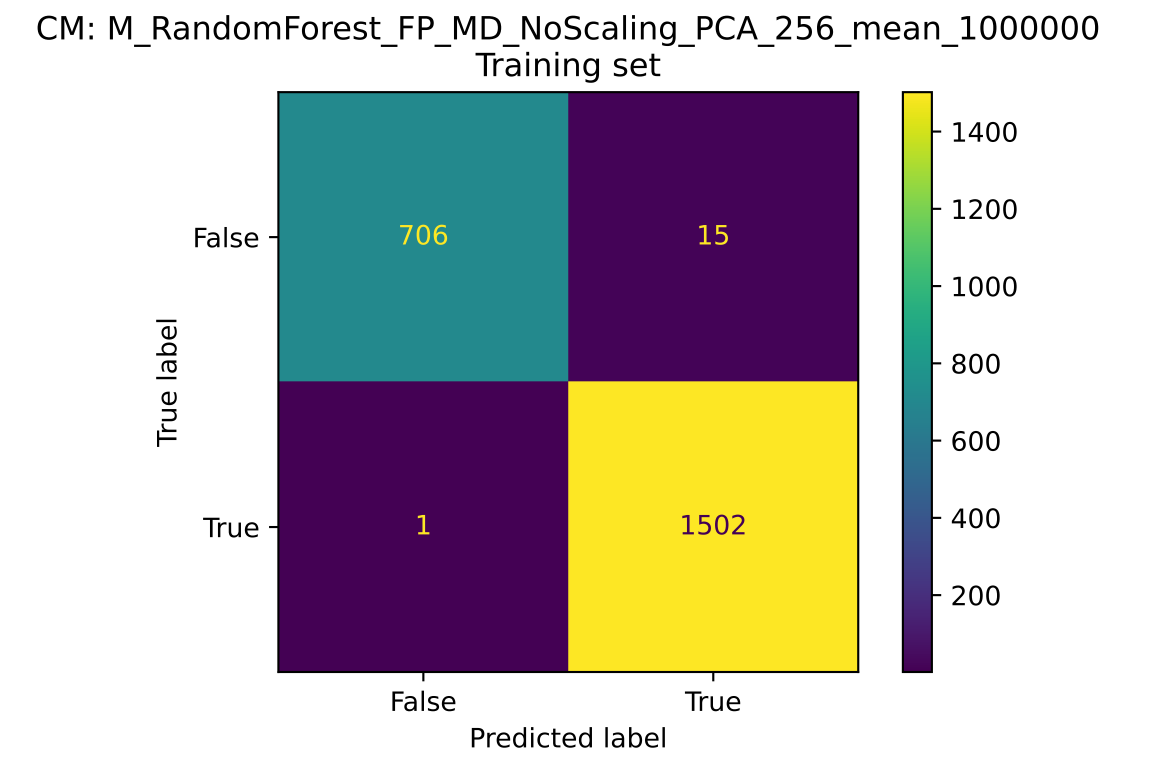

CM: M_RandomForest_FP_MD_NoScaling_PCA_256_mean_1000000
Training set
True label
Predicted label
False
True
False
True
706
15
1
1502
1400
1200
1000
800
600
400
200

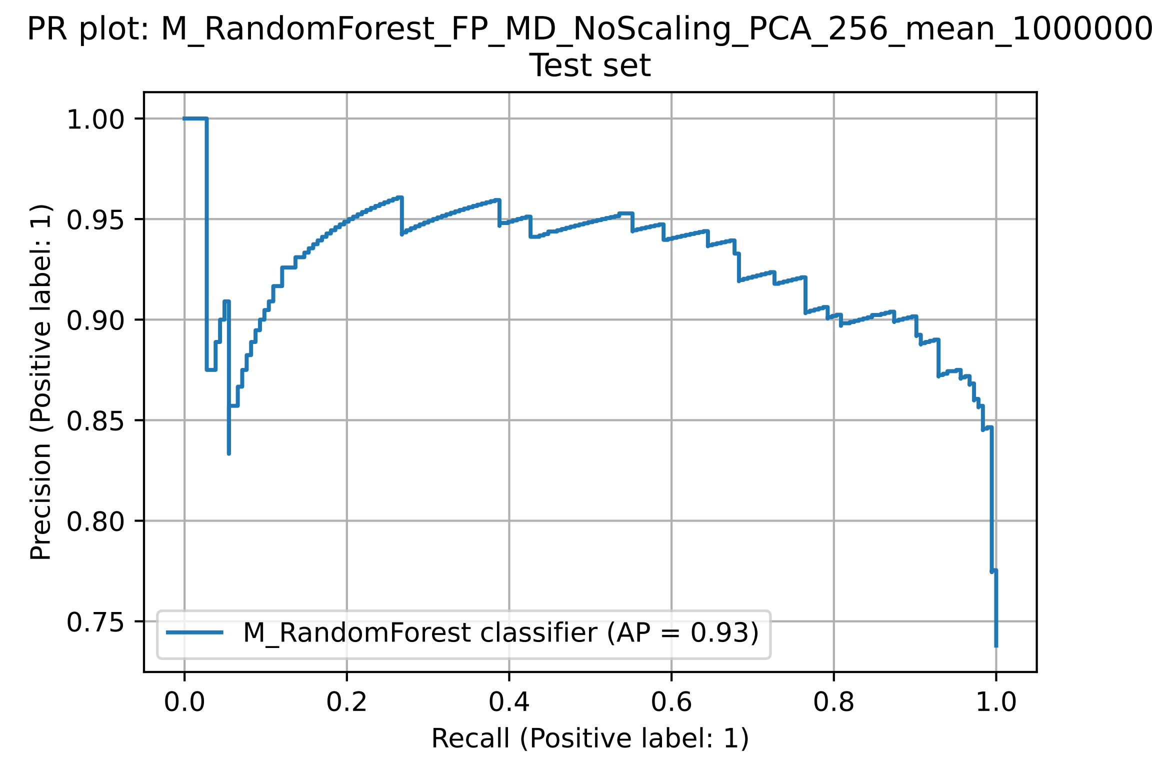
PR plot: M_RandomForest_FP_MD_NoScaling_PCA_256_mean_1000000
Test set
Precision (Positive label: 1)
Recall (Positive label: 1)
1.00
0.95
0.90
0.85
0.80
0.75
0.0
0.2
0.4
0.6
0.8
1.0
M_RandomForest classifier (AP = 0.93)

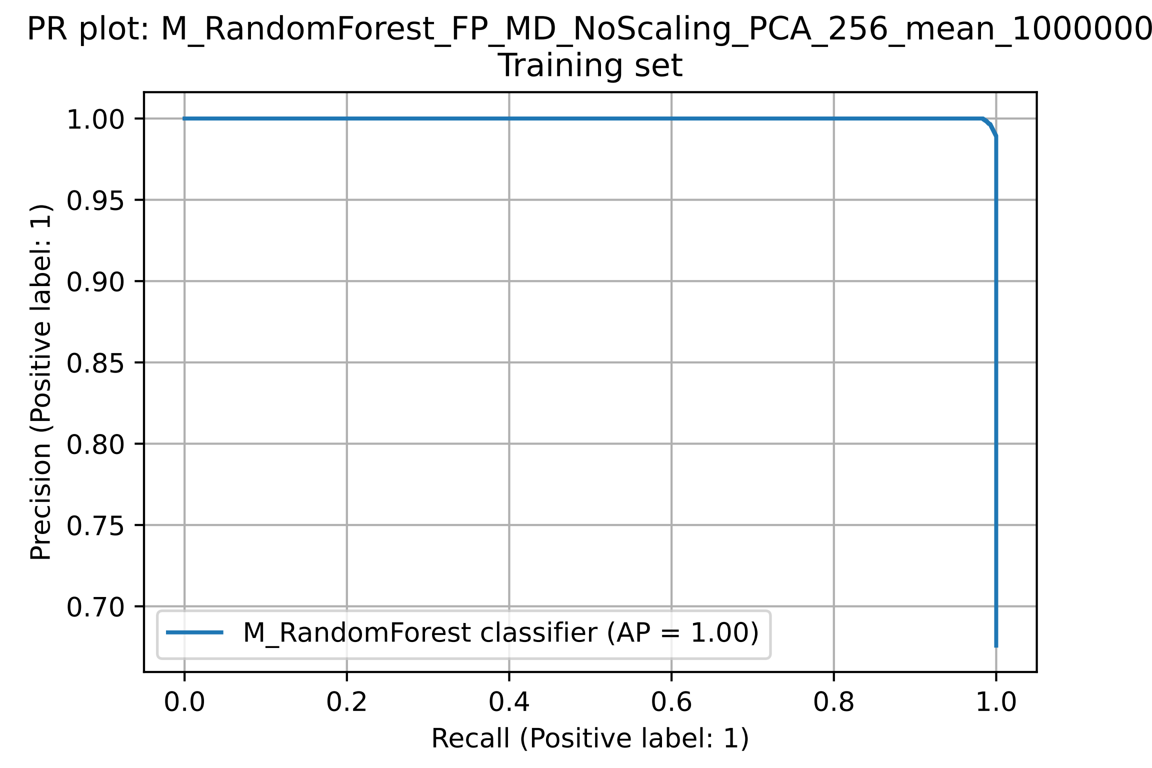
PR plot: M_RandomForest_FP_MD_NoScaling_PCA_256_mean_1000000
Training set
1.00
0.95
0.90
0.85
0.80
0.75
0.70
Precision (Positive label: 1)
0.0
0.2
0.4
0.6
0.8
1.0
Recall (Positive label: 1)
M_RandomForest classifier (AP = 1.00)

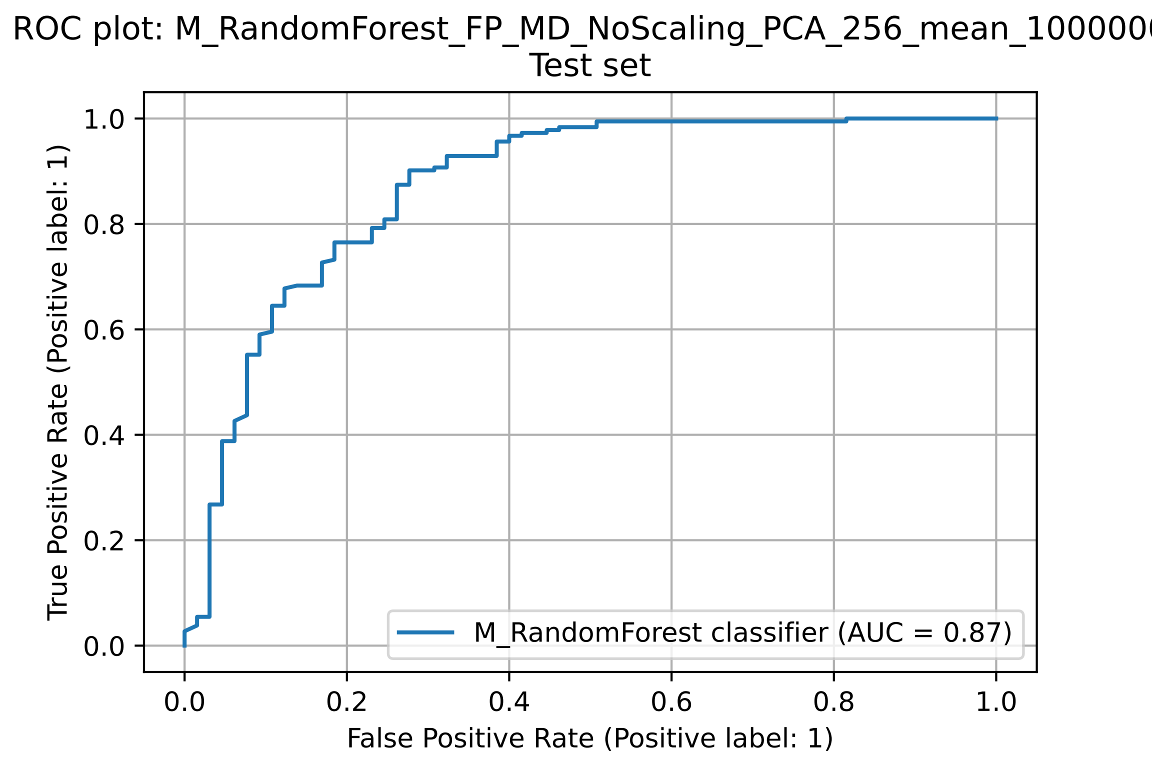
ROC plot: M_RandomForest_FP_MD_NoScaling_PCA_256_mean_100000
Test set
True Positive Rate (Positive label: 1)
False Positive Rate (Positive label: 1)
M_RandomForest classifier (AUC = 0.87)
0.0
0.2
0.4
0.6
0.8
1.0

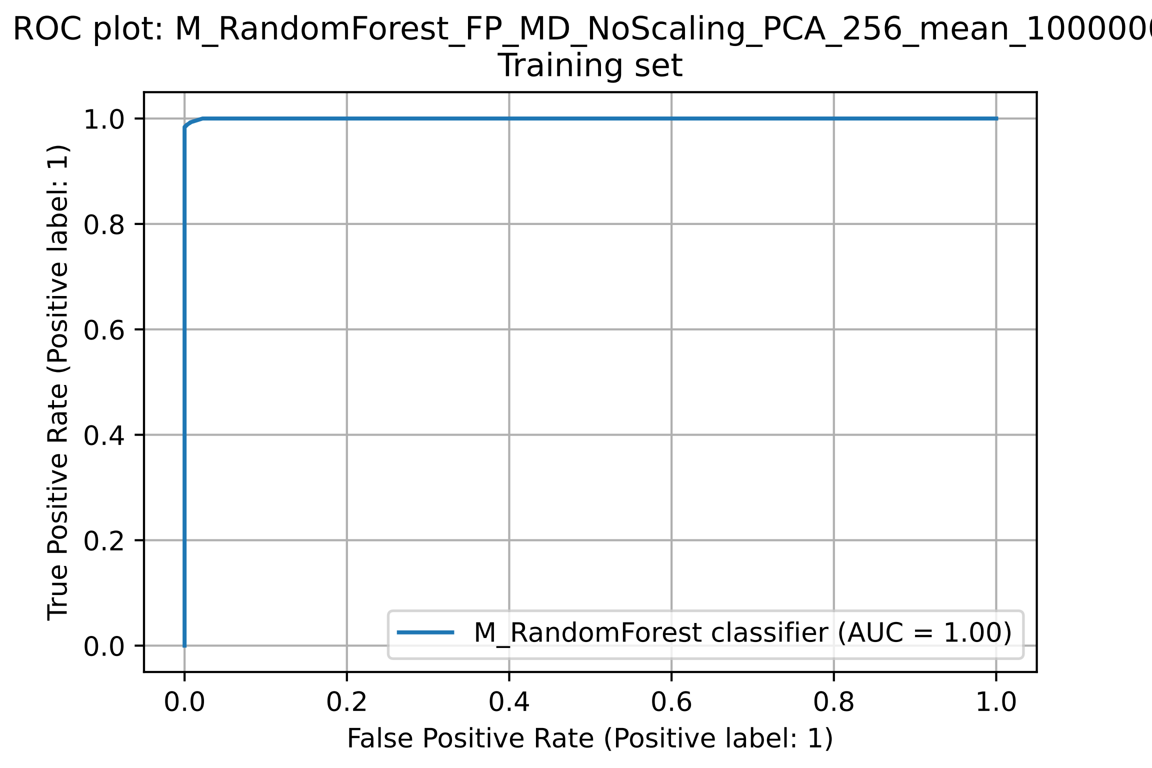

ROC plot: M_RandomForest_FP_MD_NoScaling_PCA_256_mean_100000
Training set
True Positive Rate (Positive label: 1)
False Positive Rate (Positive label: 1)
0.0
0.2
0.4
0.6
0.8
1.0
M_RandomForest classifier (AUC = 1.00)

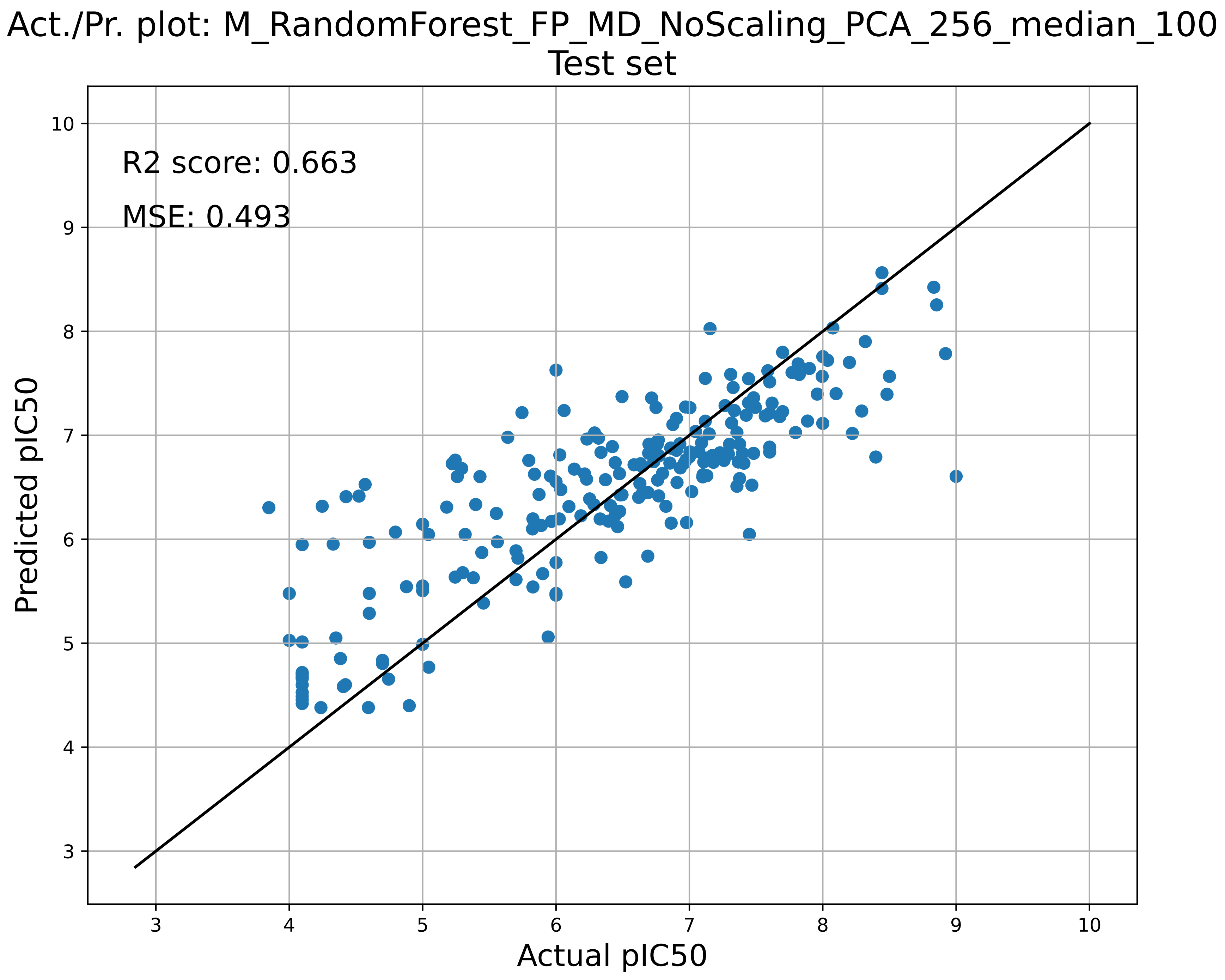
Act./Pr. plot: M_RandomForest_FP_MD_NoScaling_PCA_256_median_100
Test set
R2 score: 0.663
MSE: 0.493
Predicted pIC50
Actual pIC50
3
4
5
6
7
8
9
10

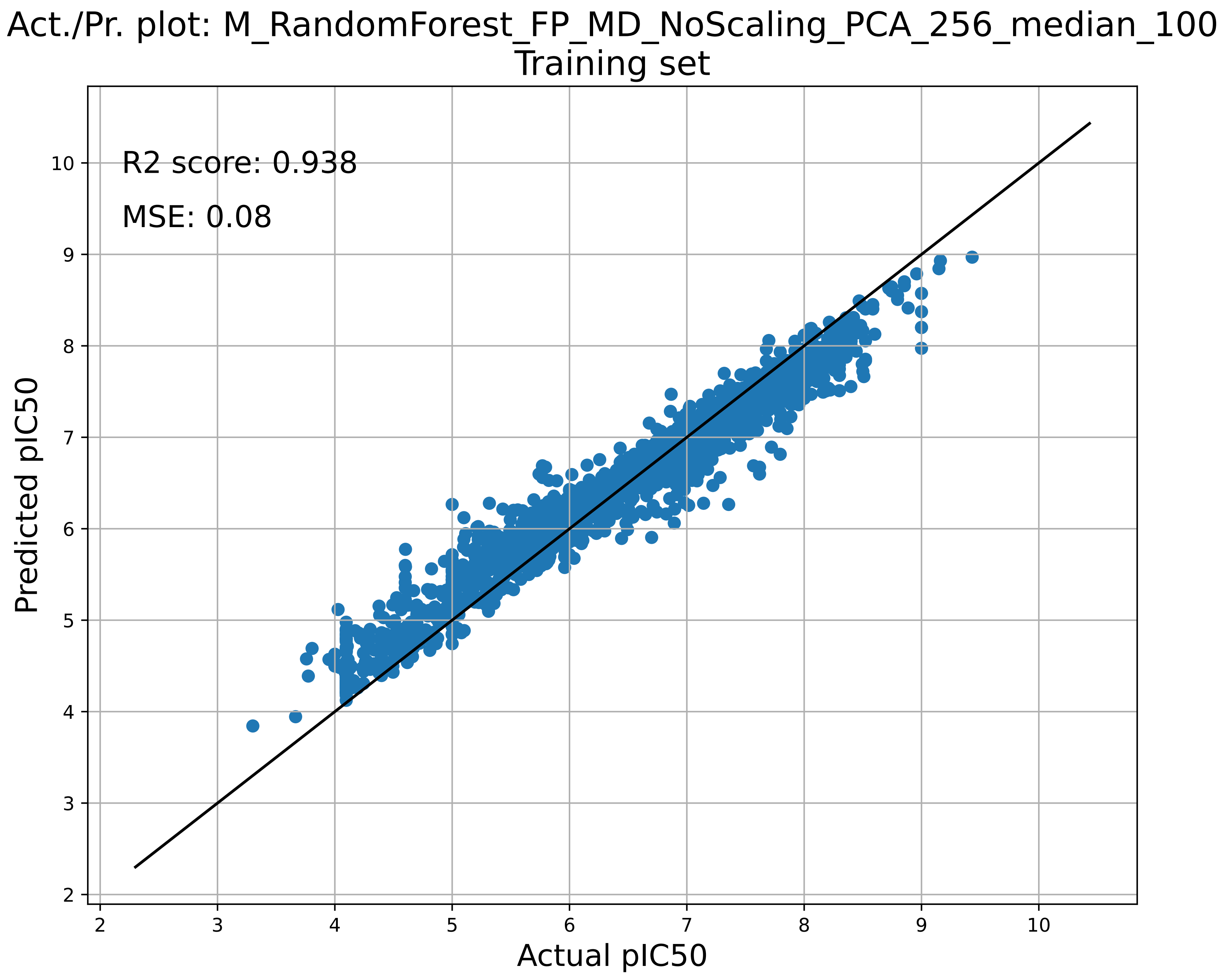
Act./Pr. plot: M_RandomForest_FP_MD_NoScaling_PCA_256_median_100
Training set
R2 score: 0.938
MSE: 0.08
Predicted pIC50
Actual pIC50
2
3
4
5
6
7
8
9
10

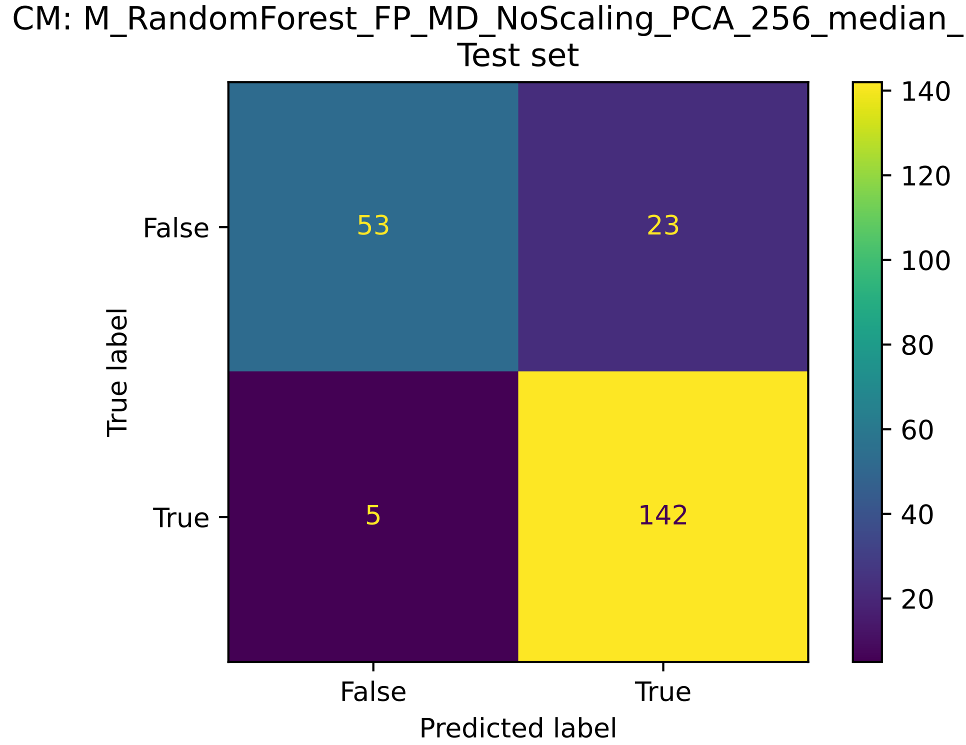
CM: M_RandomForest_FP_MD_NoScaling_PCA_256_median_100
Test set
53
23
5
142
False
True
True label
False
True
Predicted label
140
120
100
80
60
40
20

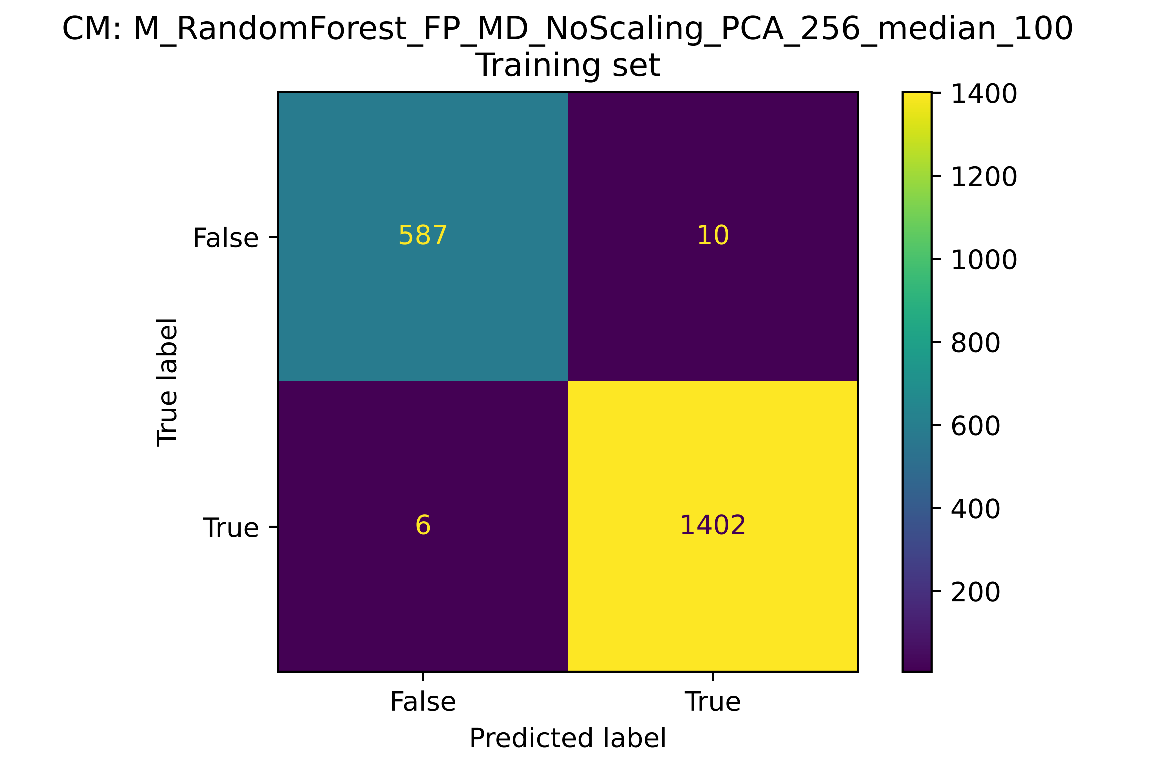
CM: M_RandomForest_FP_MD_NoScaling_PCA_256_median_100
Training set
True label
Predicted label
False
True
False
True
587
10
6
1402
1400
1200
1000
800
600
400
200

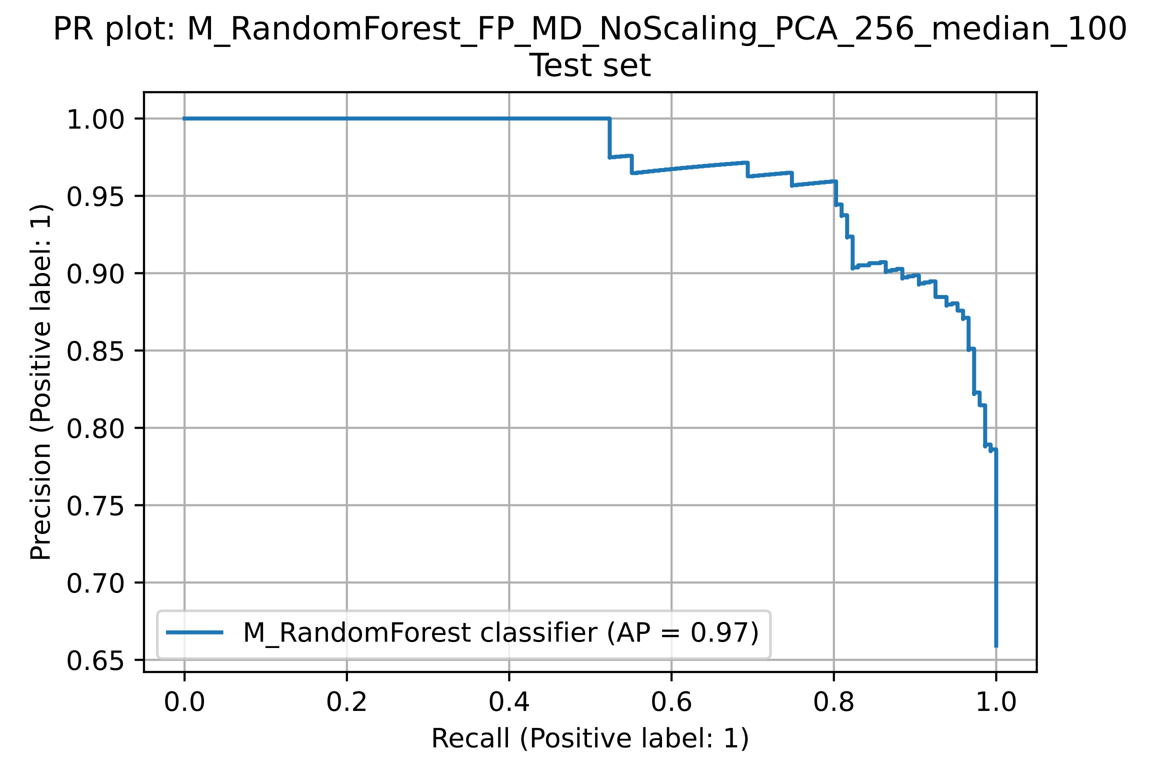
PR plot: M_RandomForest_FP_MD_NoScaling_PCA_256_median_100
Test set
Precision (Positive label: 1)
Recall (Positive label: 1)
1.00
0.95
0.90
0.85
0.80
0.75
0.70
0.65
0.0
0.2
0.4
0.6
0.8
1.0
M_RandomForest classifier (AP = 0.97)

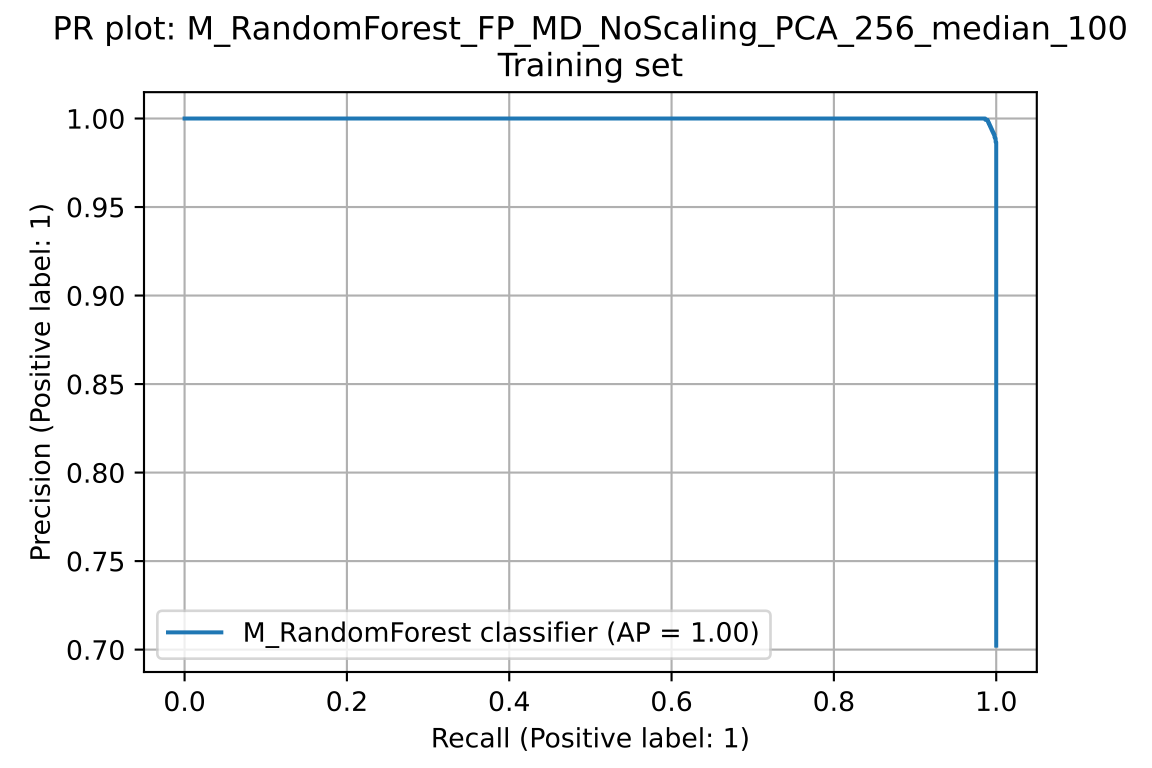
PR plot: M_RandomForest_FP_MD_NoScaling_PCA_256_median_100
Training set
Precision (Positive label: 1)
Recall (Positive label: 1)
1.00
0.95
0.90
0.85
0.80
0.75
0.70
0.0
0.2
0.4
0.6
0.8
1.0
M_RandomForest classifier (AP = 1.00)

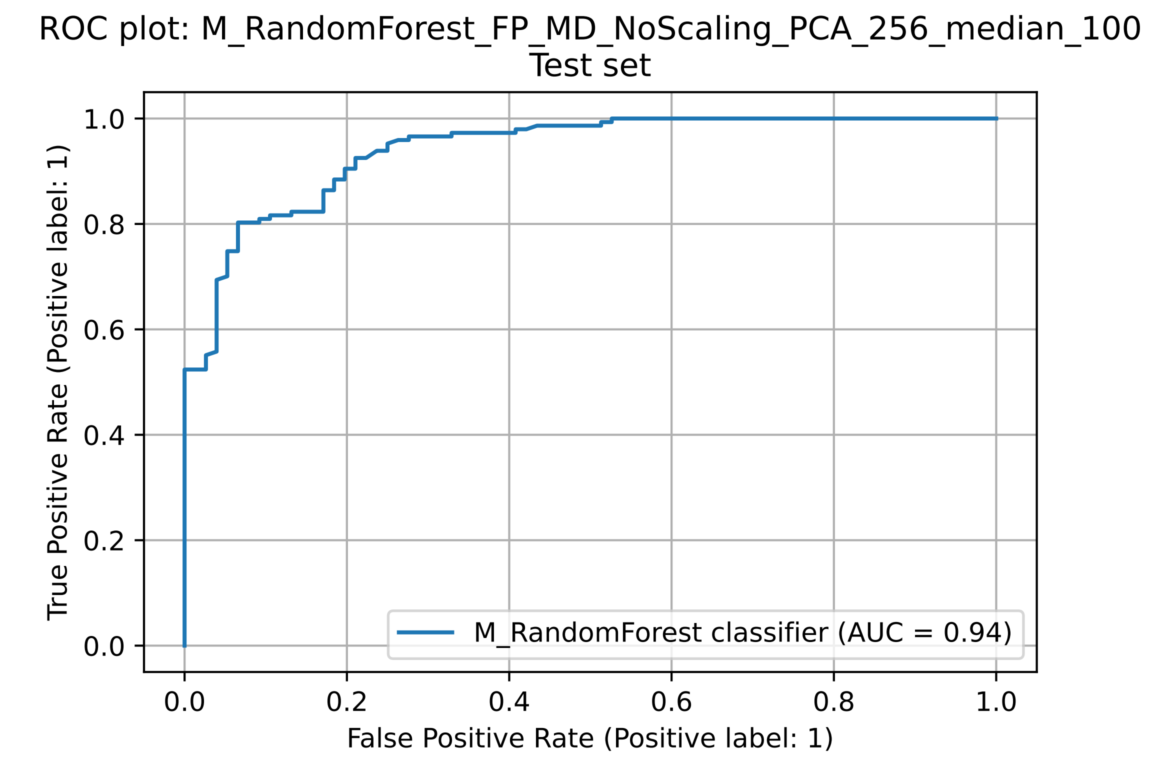

ROC plot: M_RandomForest_FP_MD_NoScaling_PCA_256_median_100
Test set
True Positive Rate (Positive label: 1)
False Positive Rate (Positive label: 1)
0.0
0.2
0.4
0.6
0.8
1.0
M_RandomForest classifier (AUC = 0.94)

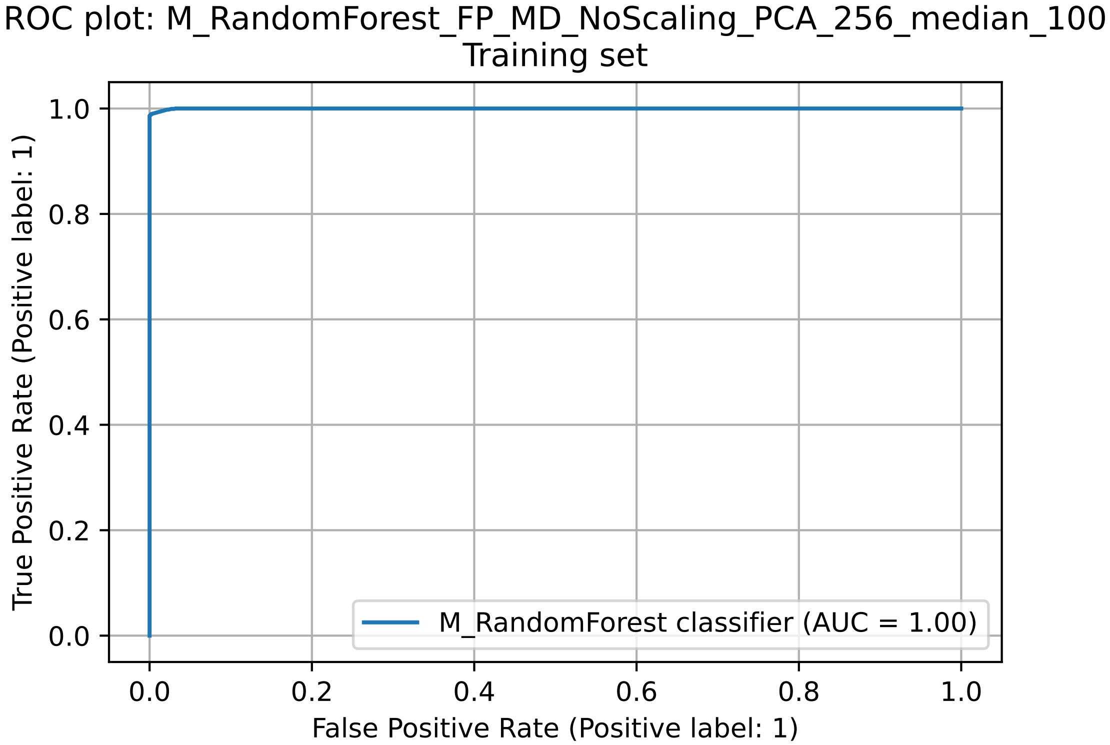

ROC plot: M_RandomForest_FP_MD_NoScaling_PCA_256_median_100
Training set
1.0
0.8
0.6
0.4
0.2
0.0
0.0
0.2
0.4
0.6
0.8
1.0
True Positive Rate (Positive label: 1)
False Positive Rate (Positive label: 1)
M_RandomForest classifier (AUC = 1.00)

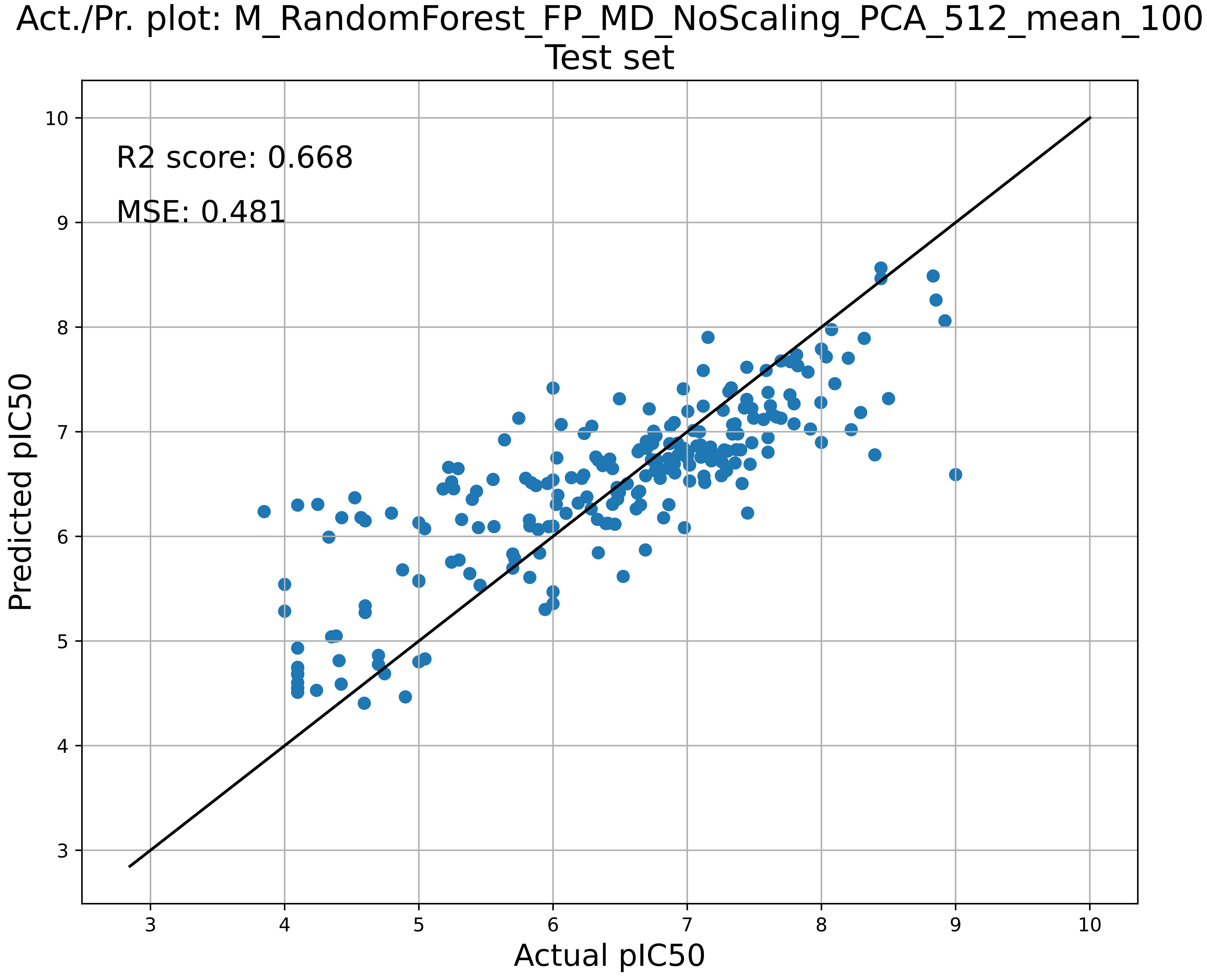
Act./Pr. plot: M_RandomForest_FP_MD_NoScaling_PCA_512_mean_100
Test set
R2 score: 0.668
MSE: 0.481
Predicted pIC50
Actual pIC50
3
4
5
6
7
8
9
10

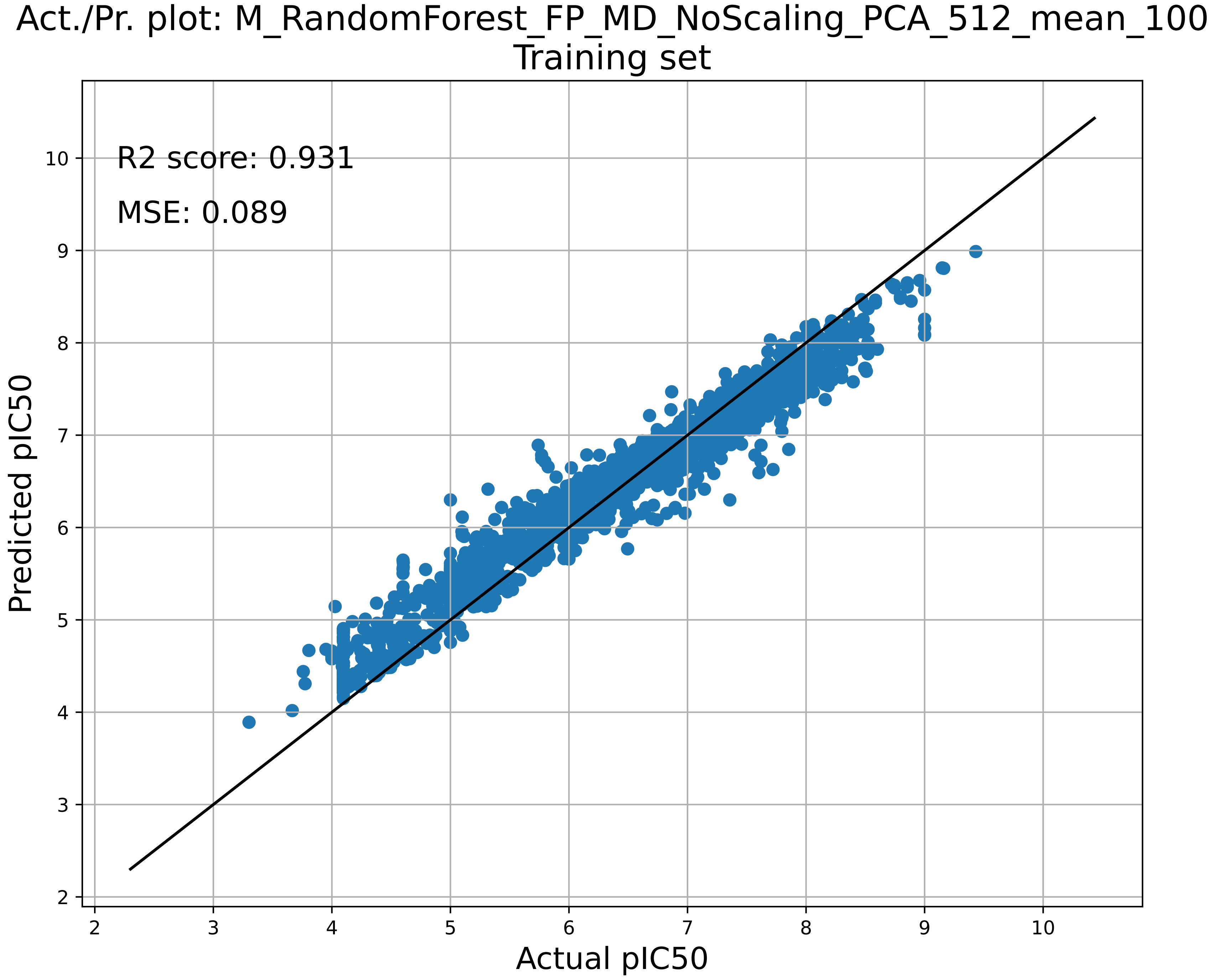

Act./Pr. plot: M_RandomForest_FP_MD_NoScaling_PCA_512_mean_100
Training set
R2 score: 0.931
MSE: 0.089
Predicted pIC50
Actual pIC50
2
3
4
5
6
7
8
9
10

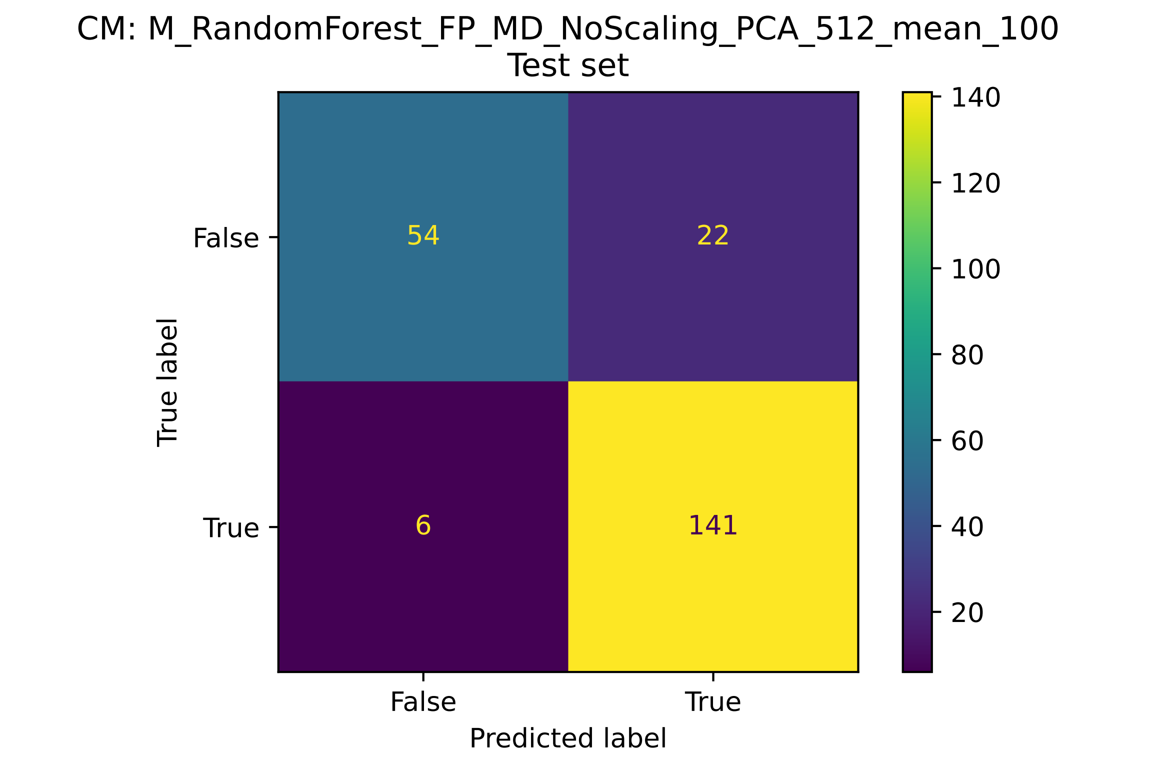

CM: M_RandomForest_FP_MD_NoScaling_PCA_512_mean_100
Test set
54
22
6
141
False
True
True label
False
True
Predicted label
140
120
100
80
60
40
20

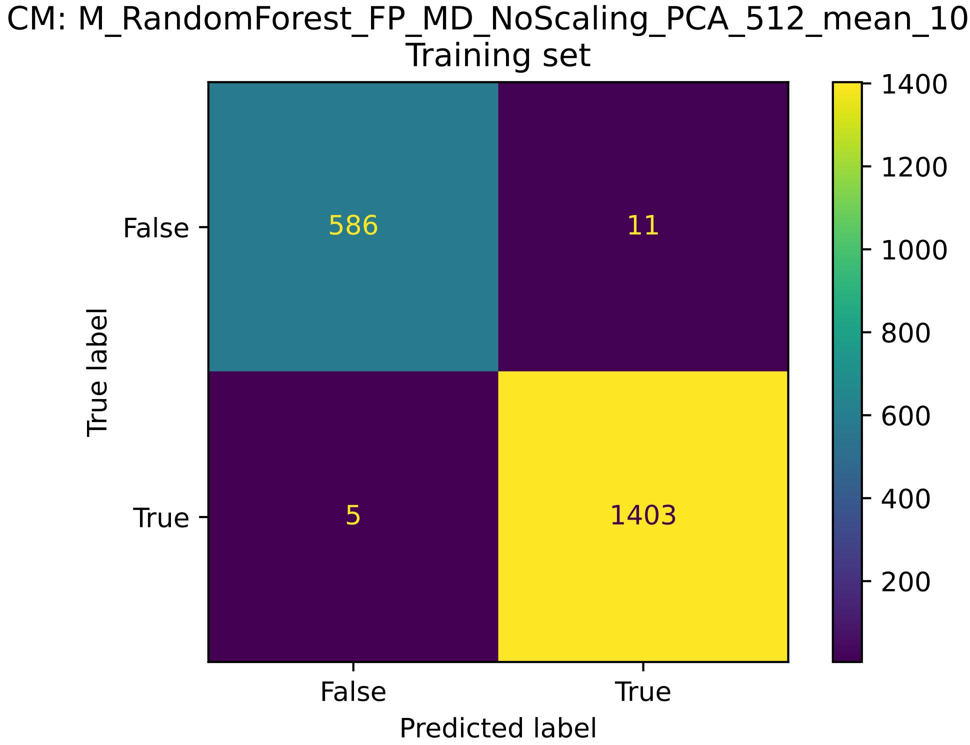

CM: M_RandomForest_FP_MD_NoScaling_PCA_512_mean_100
Training set
586
11
5
1403
False
True
True label
False
True
Predicted label
1400
1200
1000
800
600
400
200

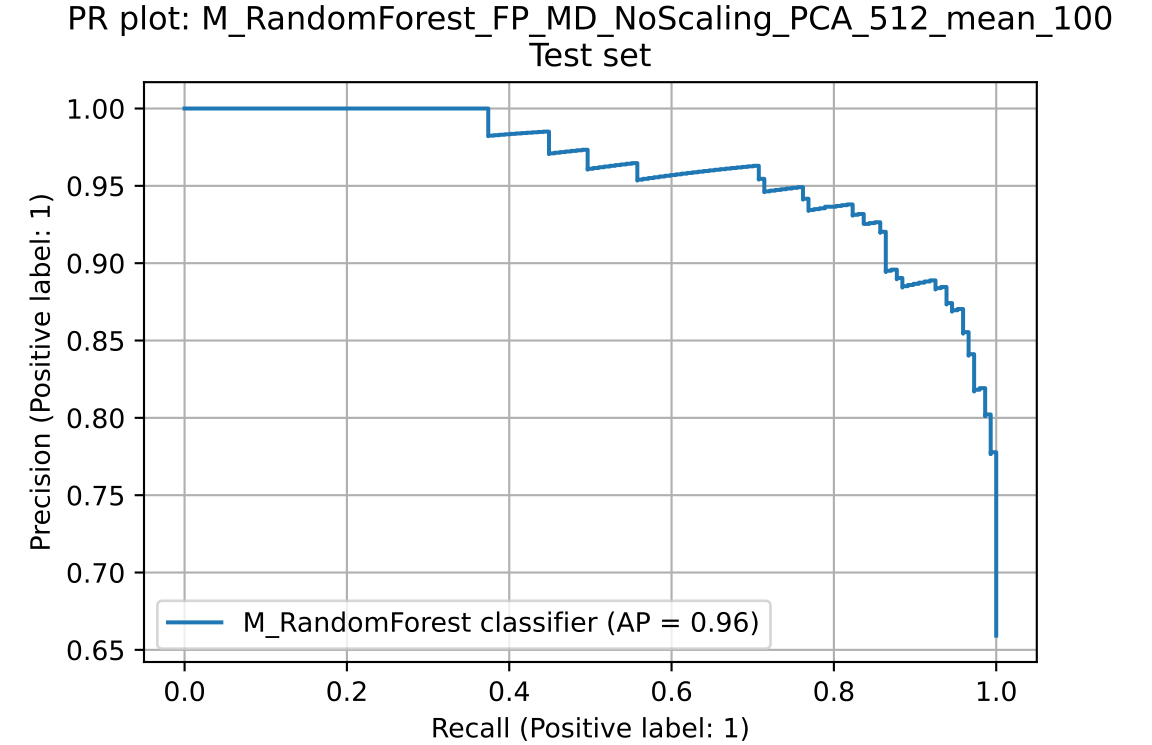
PR plot: M_RandomForest_FP_MD_NoScaling_PCA_512_mean_100
Test set
Precision (Positive label: 1)
1.00
0.95
0.90
0.85
0.80
0.75
0.70
0.65
0.0
0.2
0.4
0.6
0.8
1.0
Recall (Positive label: 1)
M_RandomForest classifier (AP = 0.96)

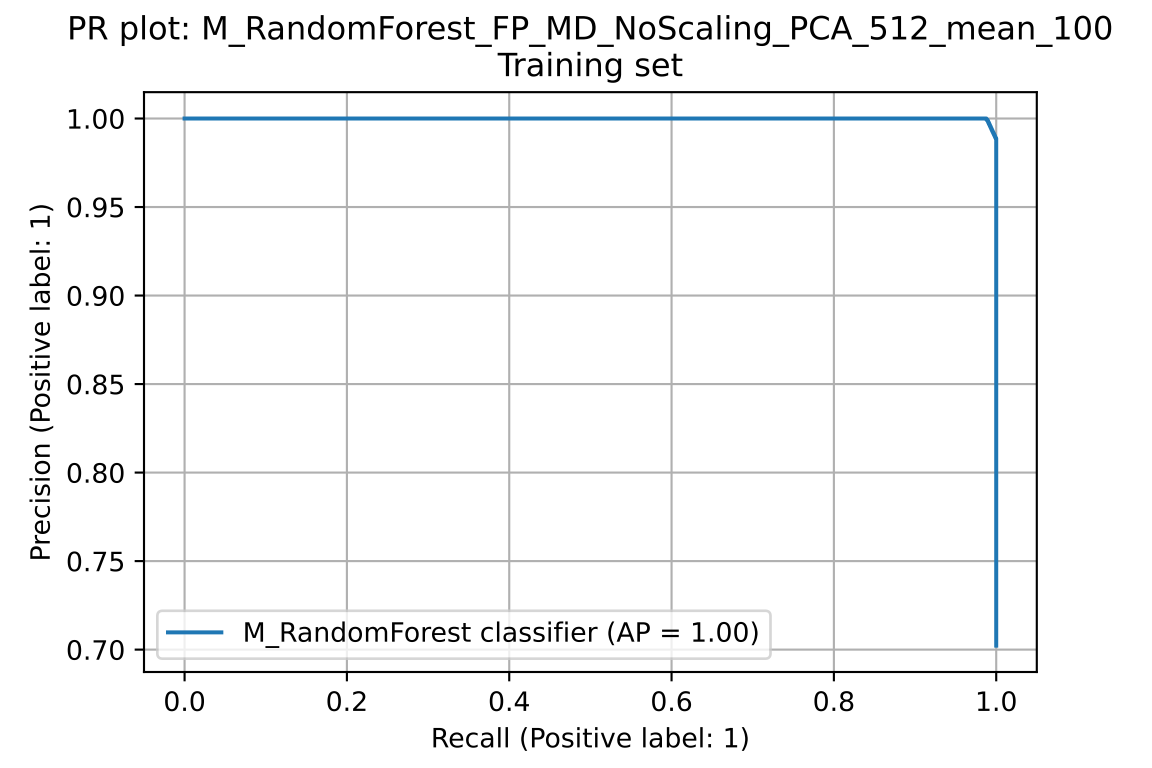
PR plot: M_RandomForest_FP_MD_NoScaling_PCA_512_mean_100
Training set
Precision (Positive label: 1)
Recall (Positive label: 1)
1.00
0.95
0.90
0.85
0.80
0.75
0.70
0.0
0.2
0.4
0.6
0.8
1.0
M_RandomForest classifier (AP = 1.00)

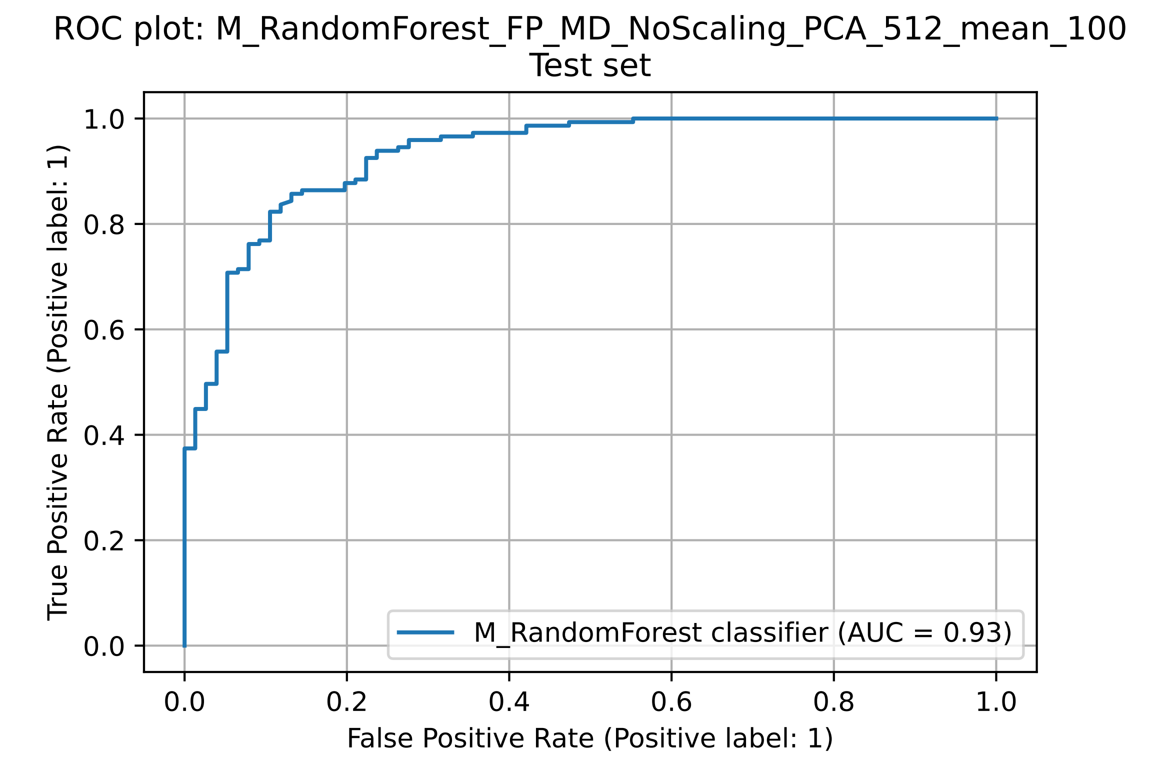

ROC plot: M_RandomForest_FP_MD_NoScaling_PCA_512_mean_100
Test set
True Positive Rate (Positive label: 1)
False Positive Rate (Positive label: 1)
0.0
0.2
0.4
0.6
0.8
1.0
M_RandomForest classifier (AUC = 0.93)

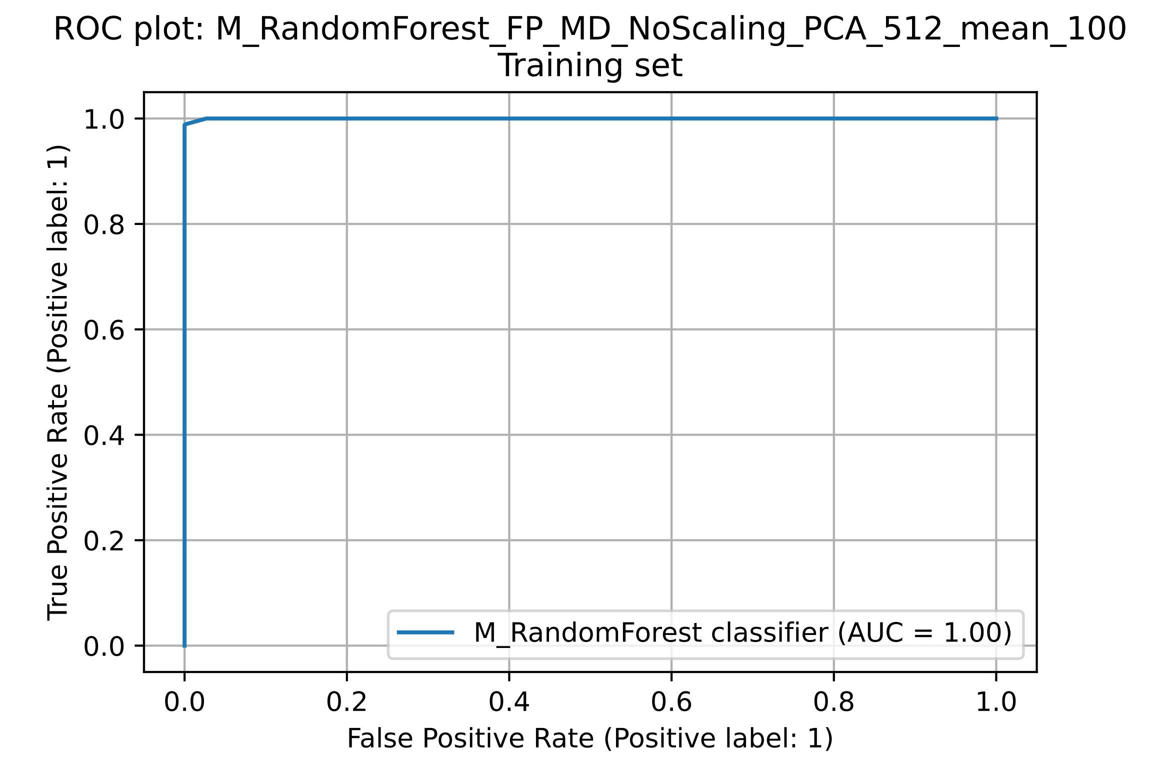
ROC plot: M_RandomForest_FP_MD_NoScaling_PCA_512_mean_100
Training set
True Positive Rate (Positive label: 1)
False Positive Rate (Positive label: 1)
0.0
0.2
0.4
0.6
0.8
1.0
M_RandomForest classifier (AUC = 1.00)

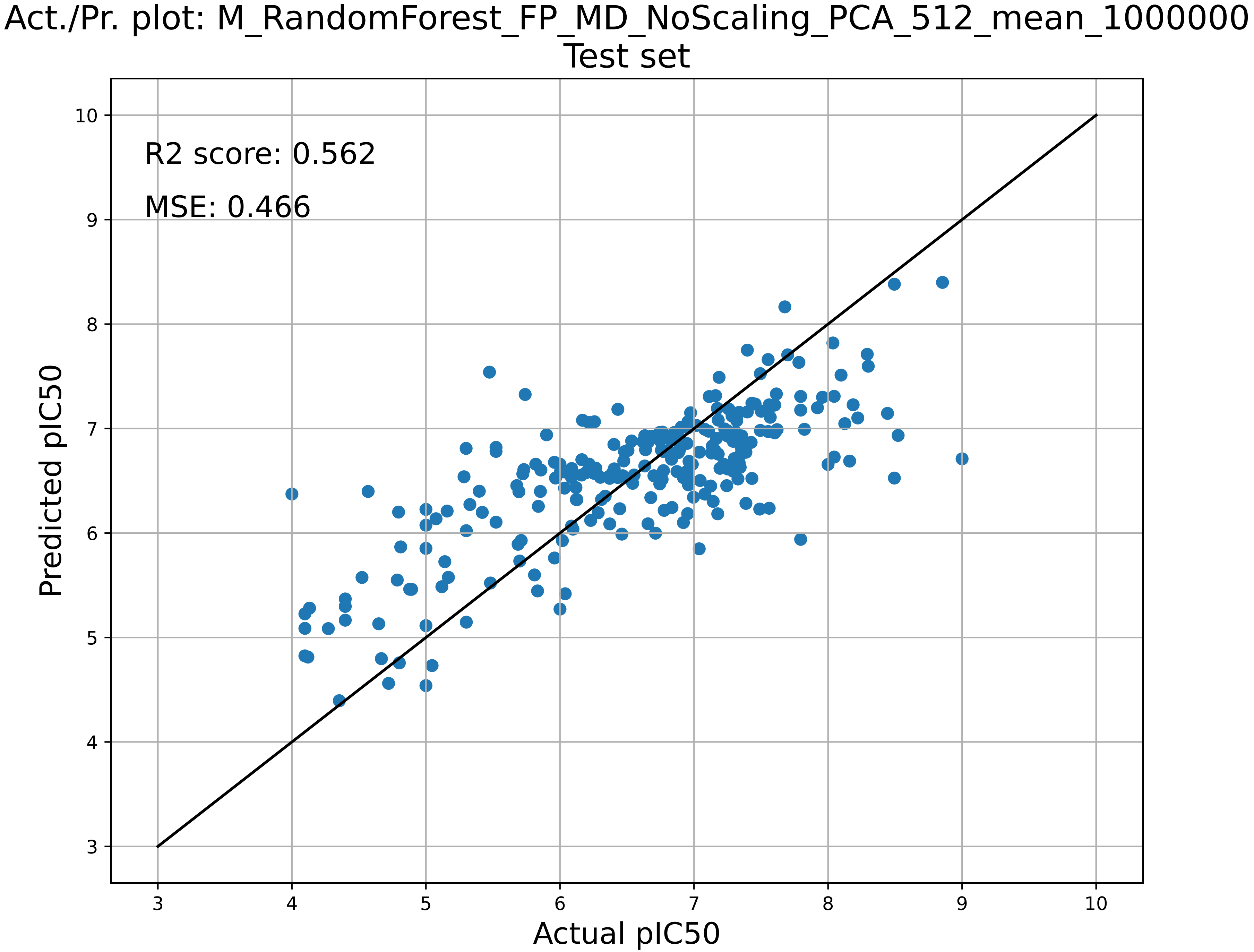

Act./Pr. plot: M_RandomForest_FP_MD_NoScaling_PCA_512_mean_1000000
Test set
R2 score: 0.562
MSE: 0.466
Predicted pIC50
Actual pIC50
10
9
8
7
6
5
4
3
3
4
5
6
7
8
9
10

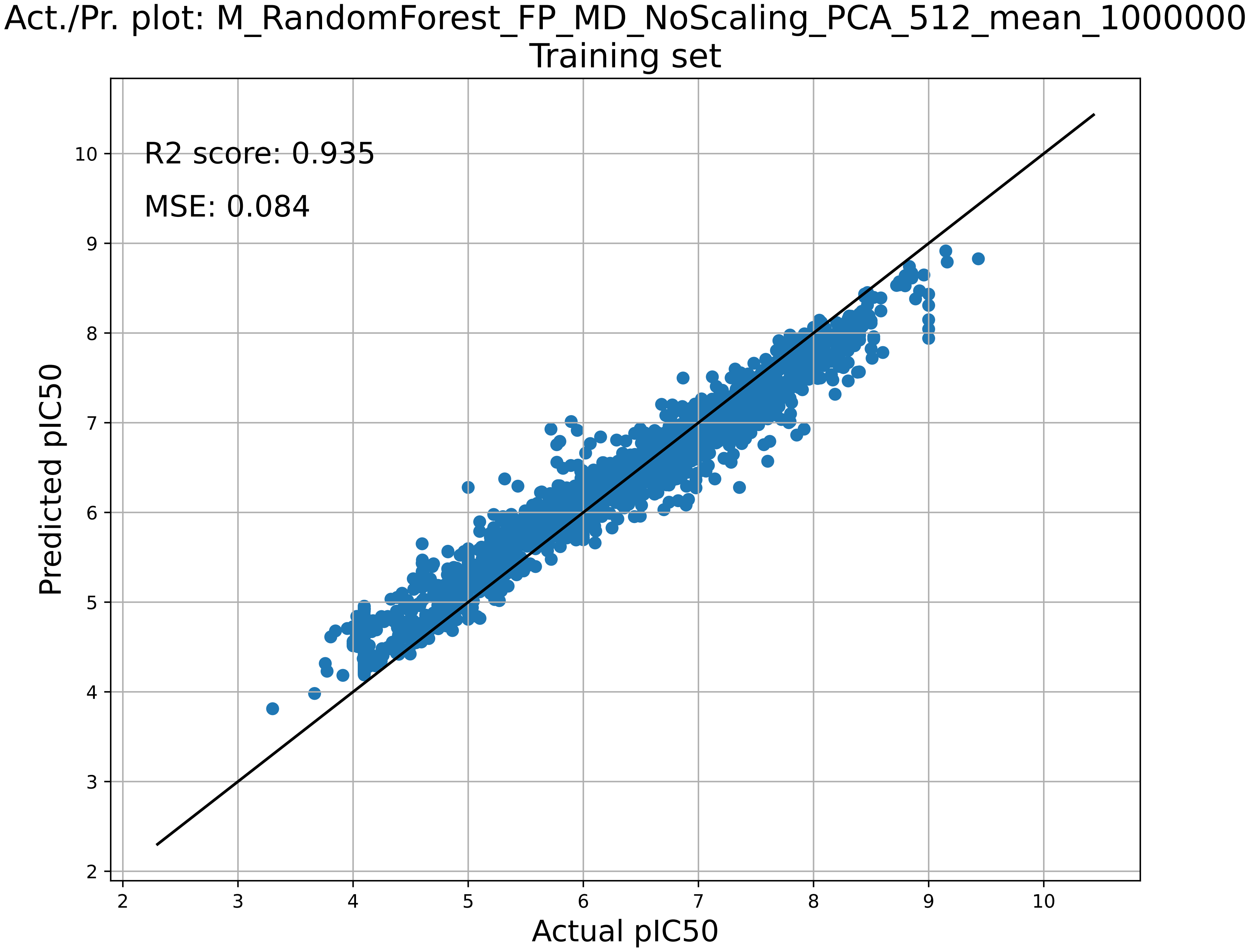
Act./Pr. plot: M_RandomForest_FP_MD_NoScaling_PCA_512_mean_1000000
Training set
R2 score: 0.935
MSE: 0.084
Predicted pIC50
Actual pIC50
2
3
4
5
6
7
8
9
10

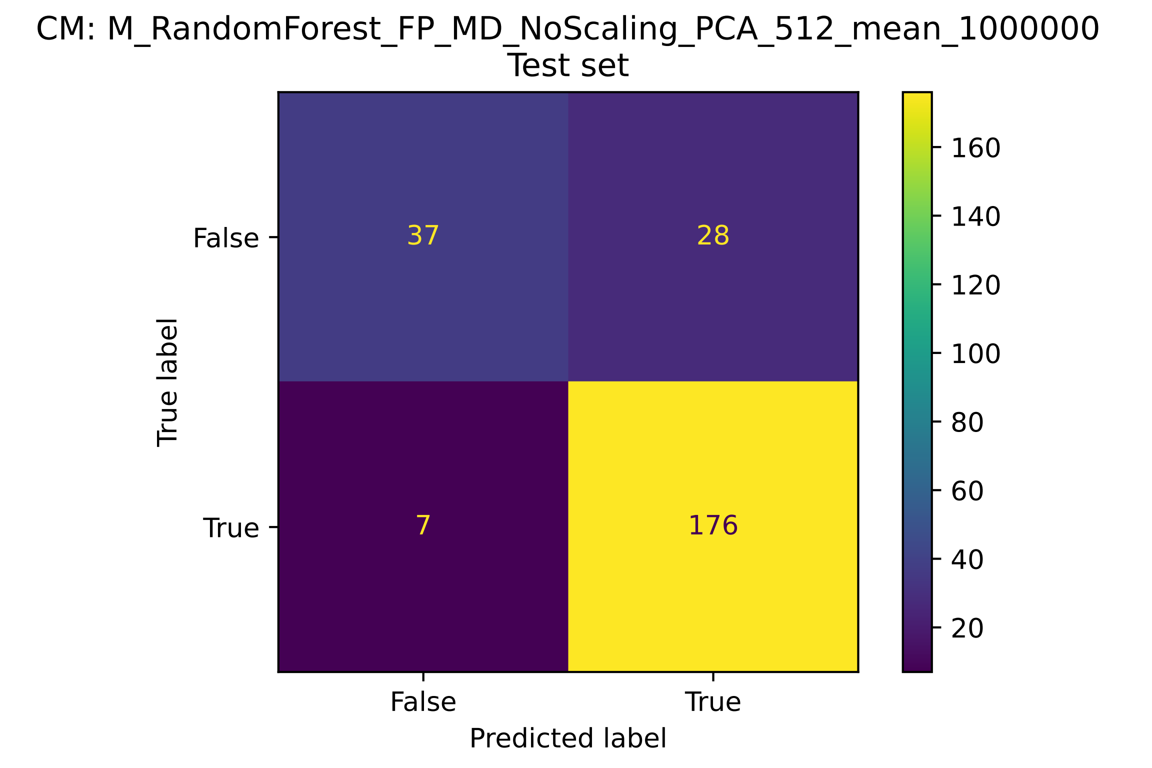
CM: M_RandomForest_FP_MD_NoScaling_PCA_512_mean_1000000
Test set
True label
False
True
37
28
7
176
Predicted label
False
True
160
140
120
100
80
60
40
20

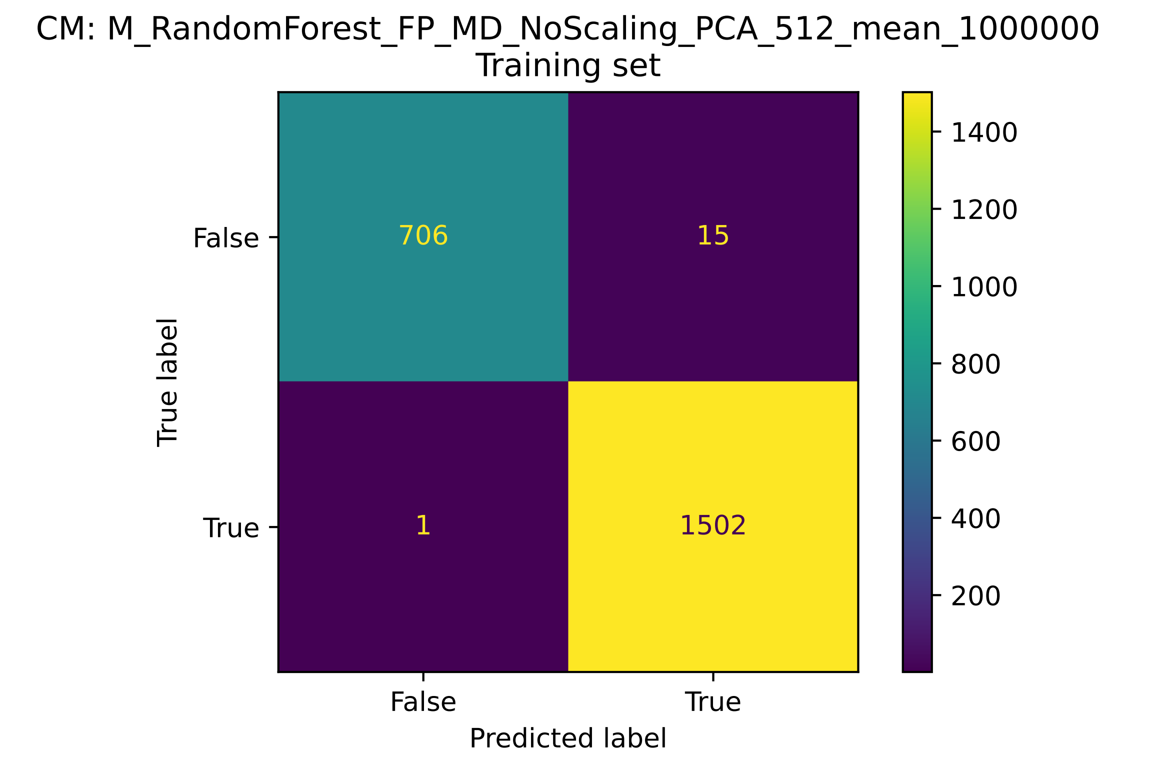
CM: M_RandomForest_FP_MD_NoScaling_PCA_512_mean_1000000
Training set
True label
False
True
706
15
1
1502
Predicted label
False
True
1400
1200
1000
800
600
400
200

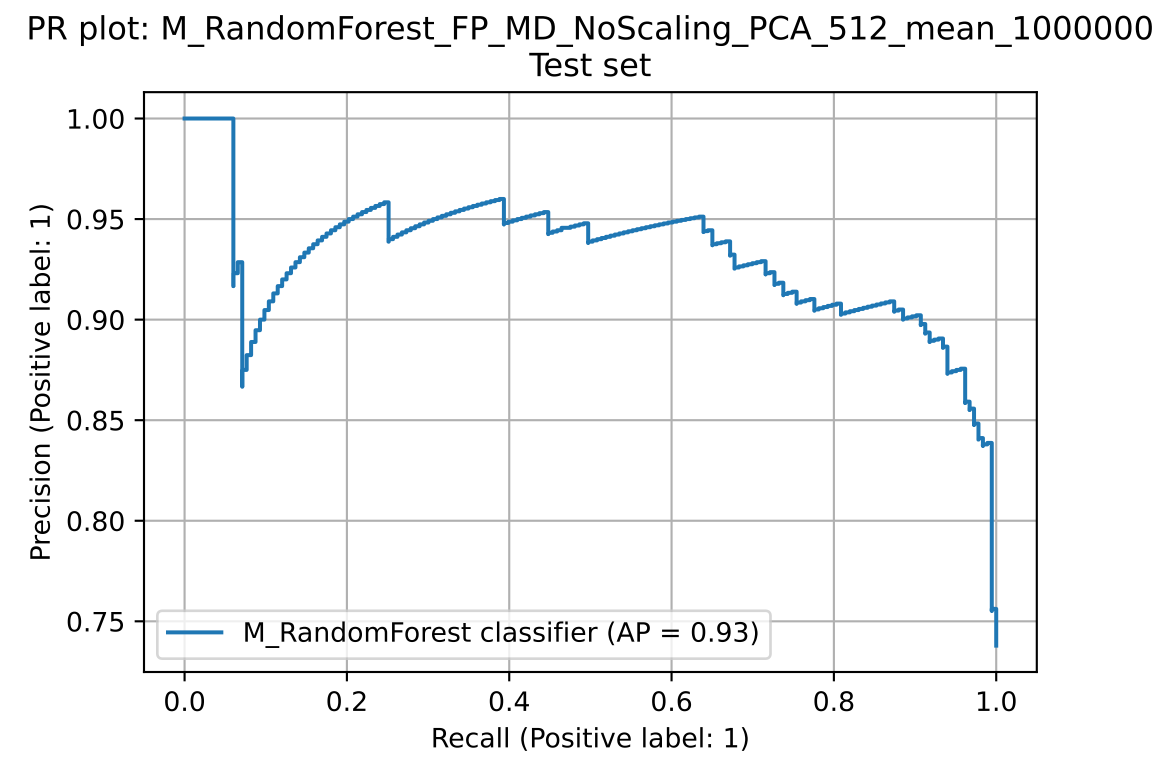

PR plot: M_RandomForest_FP_MD_NoScaling_PCA_512_mean_1000000
Test set
Precision (Positive label: 1)
Recall (Positive label: 1)
1.00
0.95
0.90
0.85
0.80
0.75
0.0
0.2
0.4
0.6
0.8
1.0
M_RandomForest classifier (AP = 0.93)

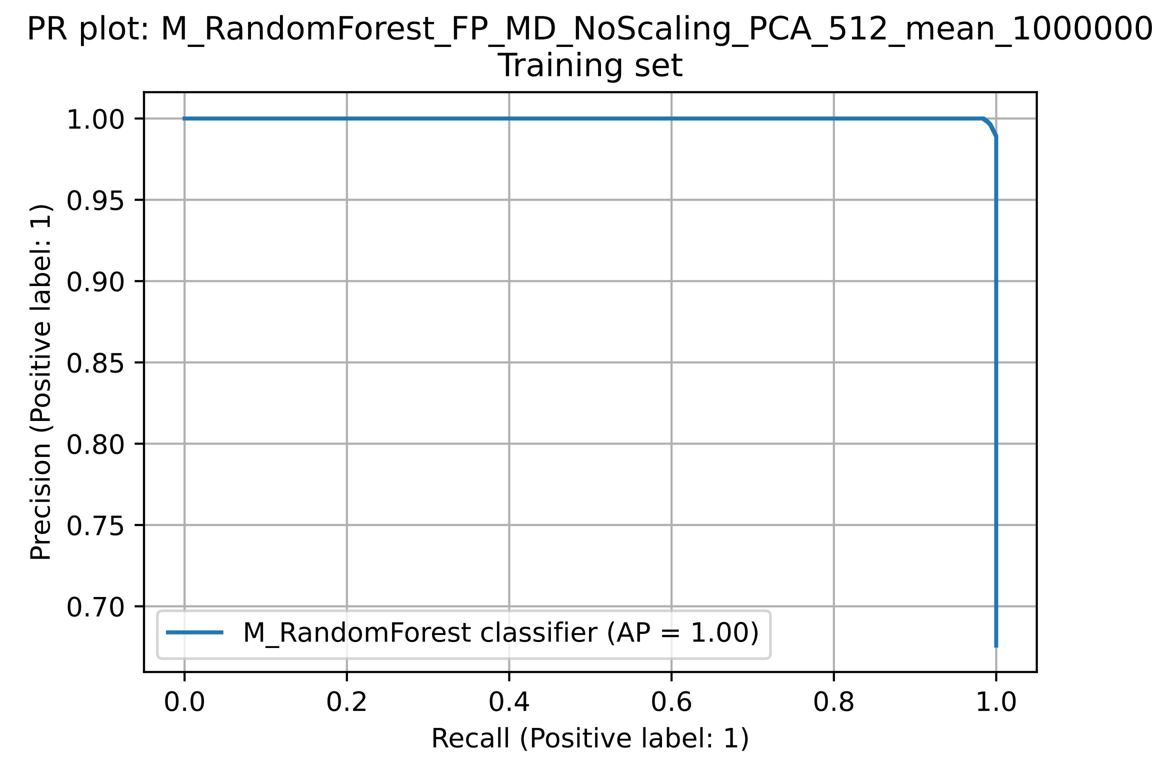

PR plot: M_RandomForest_FP_MD_NoScaling_PCA_512_mean_1000000
Training set
1.00
0.95
0.90
0.85
0.80
0.75
0.70
Precision (Positive label: 1)
0.0
0.2
0.4
0.6
0.8
1.0
Recall (Positive label: 1)
M_RandomForest classifier (AP = 1.00)

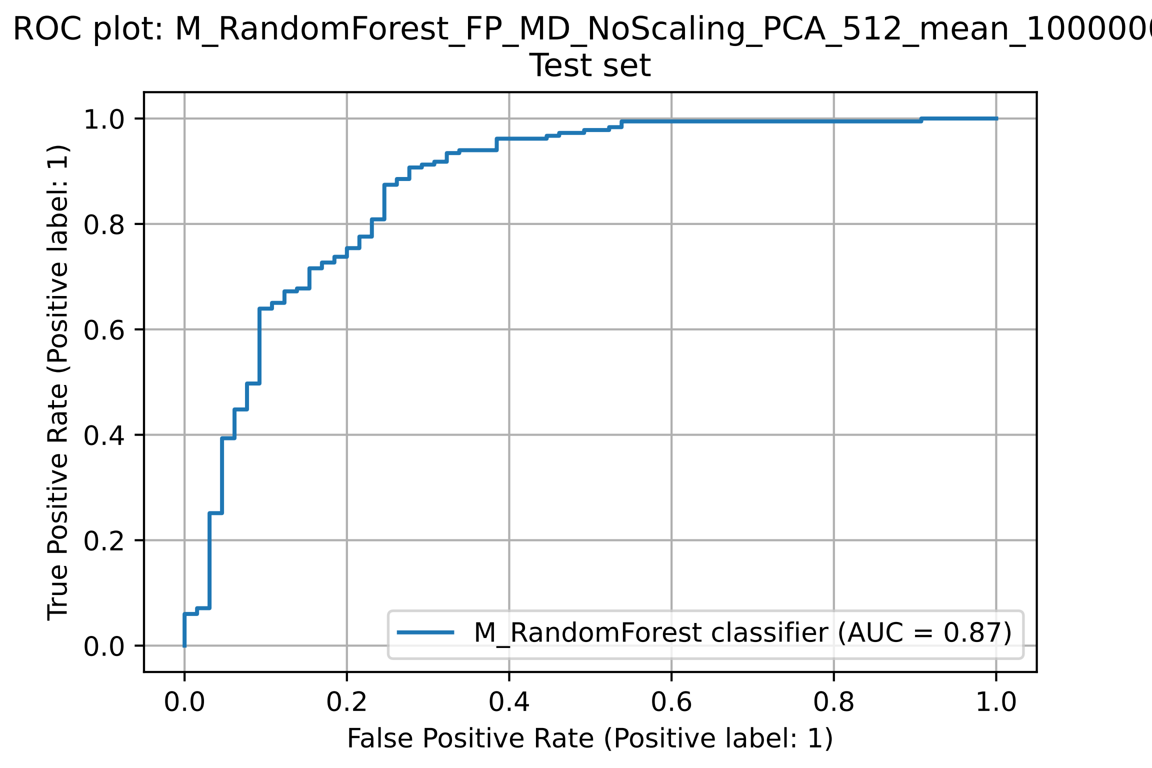
ROC plot: M_RandomForest_FP_MD_NoScaling_PCA_512_mean_100000
Test set
True Positive Rate (Positive label: 1)
False Positive Rate (Positive label: 1)
M_RandomForest classifier (AUC = 0.87)
0.0
0.2
0.4
0.6
0.8
1.0

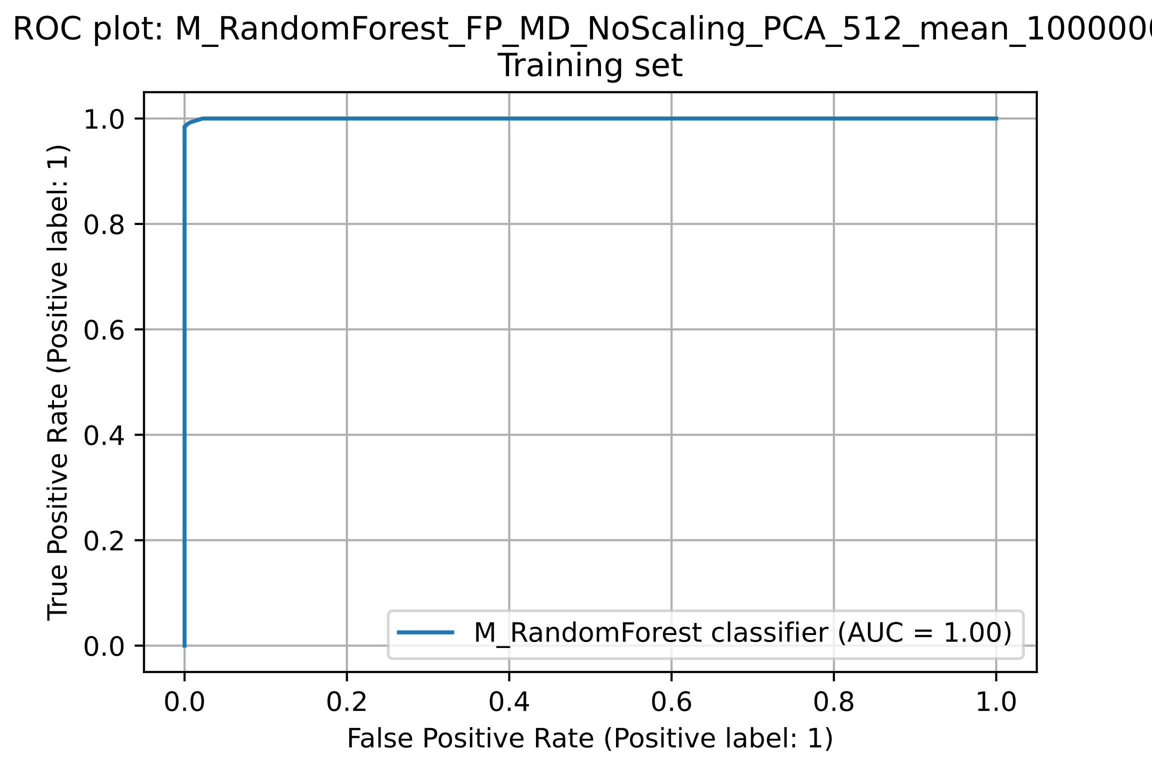
ROC plot: M_RandomForest_FP_MD_NoScaling_PCA_512_mean_100000
Training set
True Positive Rate (Positive label: 1)
False Positive Rate (Positive label: 1)
0.0
0.2
0.4
0.6
0.8
1.0
M_RandomForest classifier (AUC = 1.00)

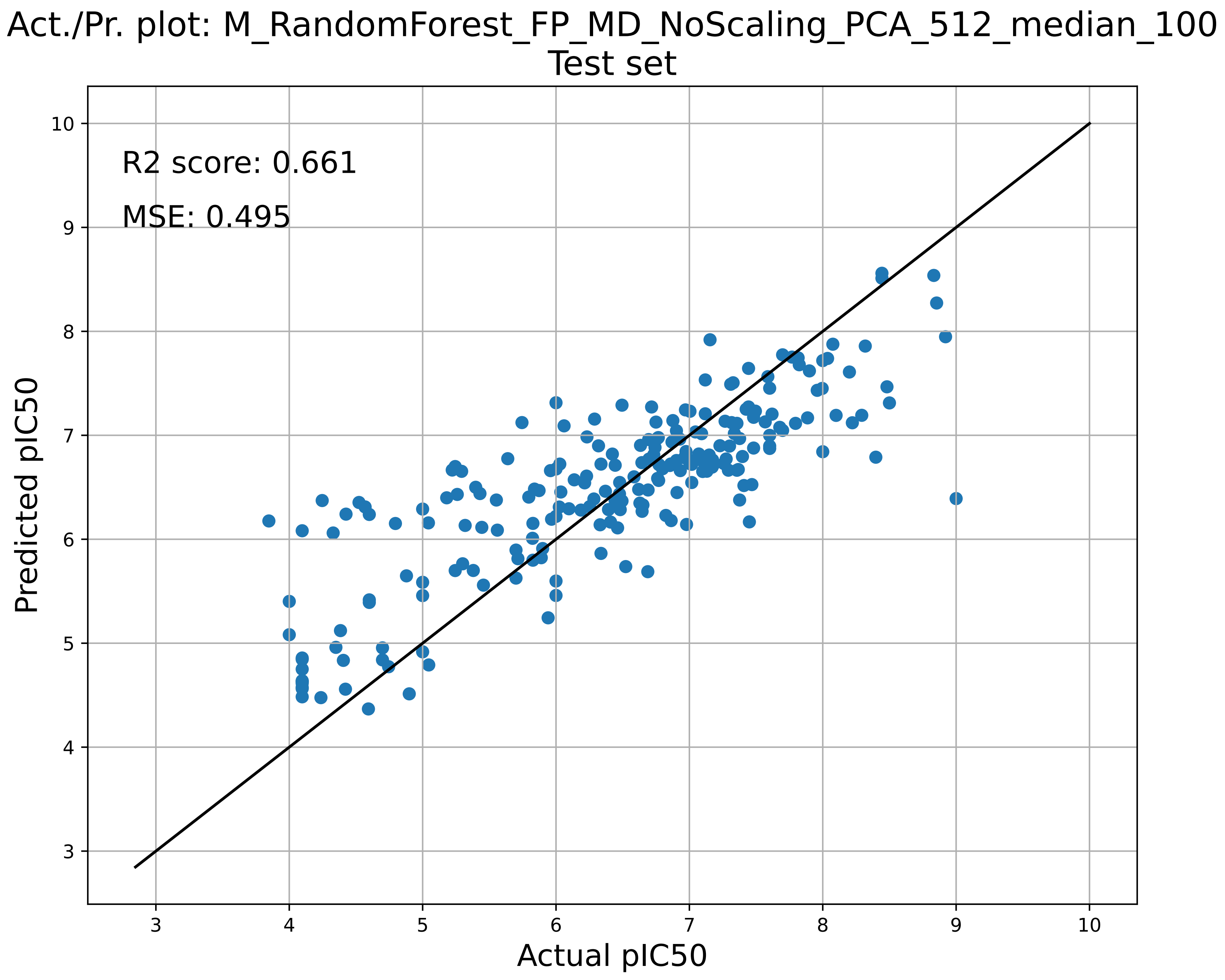

Act./Pr. plot: M_RandomForest_FP_MD_NoScaling_PCA_512_median_100
Test set
R2 score: 0.661
MSE: 0.495
Predicted pIC50
Actual pIC50
3
4
5
6
7
8
9
10

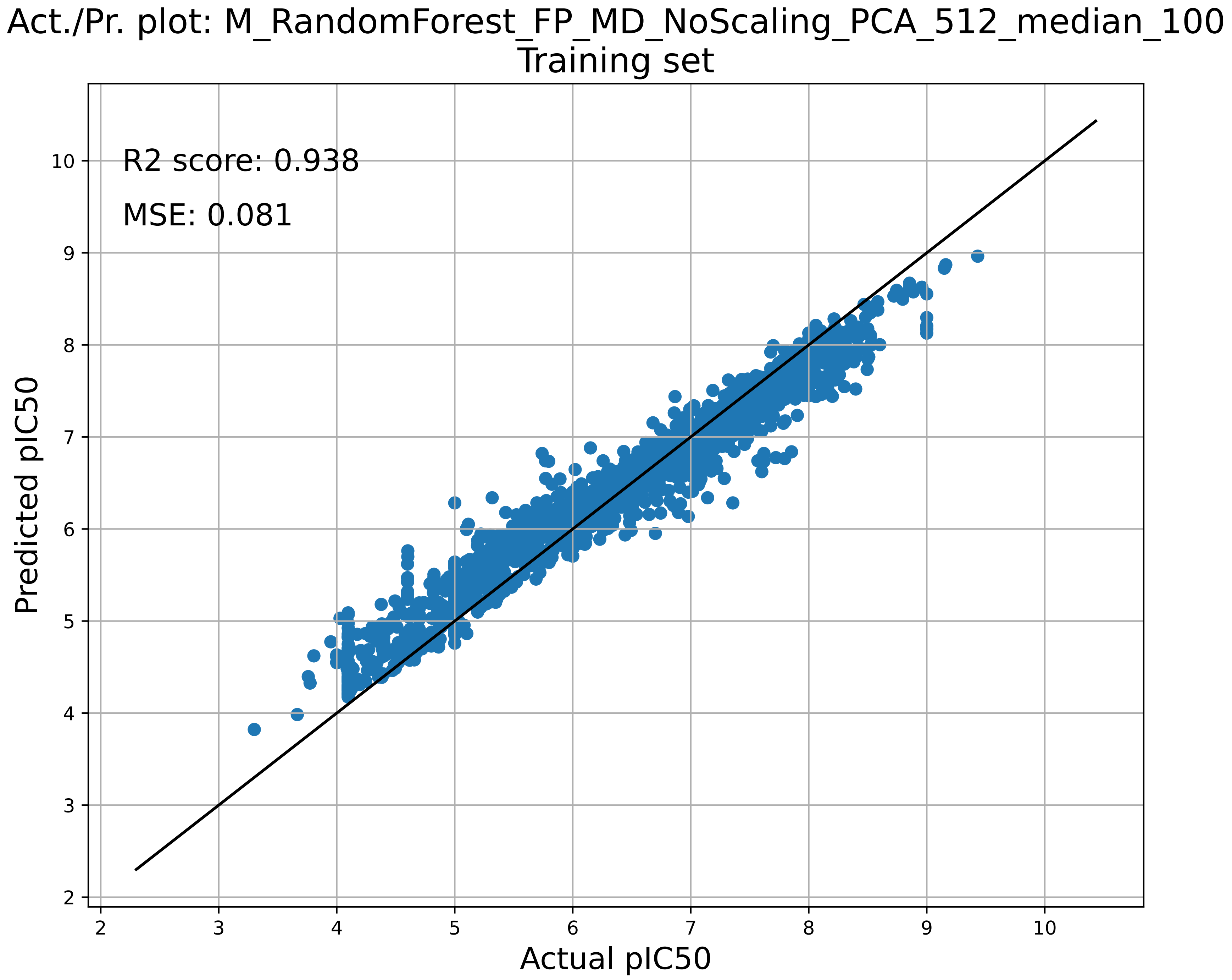

Act./Pr. plot: M_RandomForest_FP_MD_NoScaling_PCA_512_median_100
Training set
R2 score: 0.938
MSE: 0.081
Predicted pIC50
Actual pIC50
2
3
4
5
6
7
8
9
10

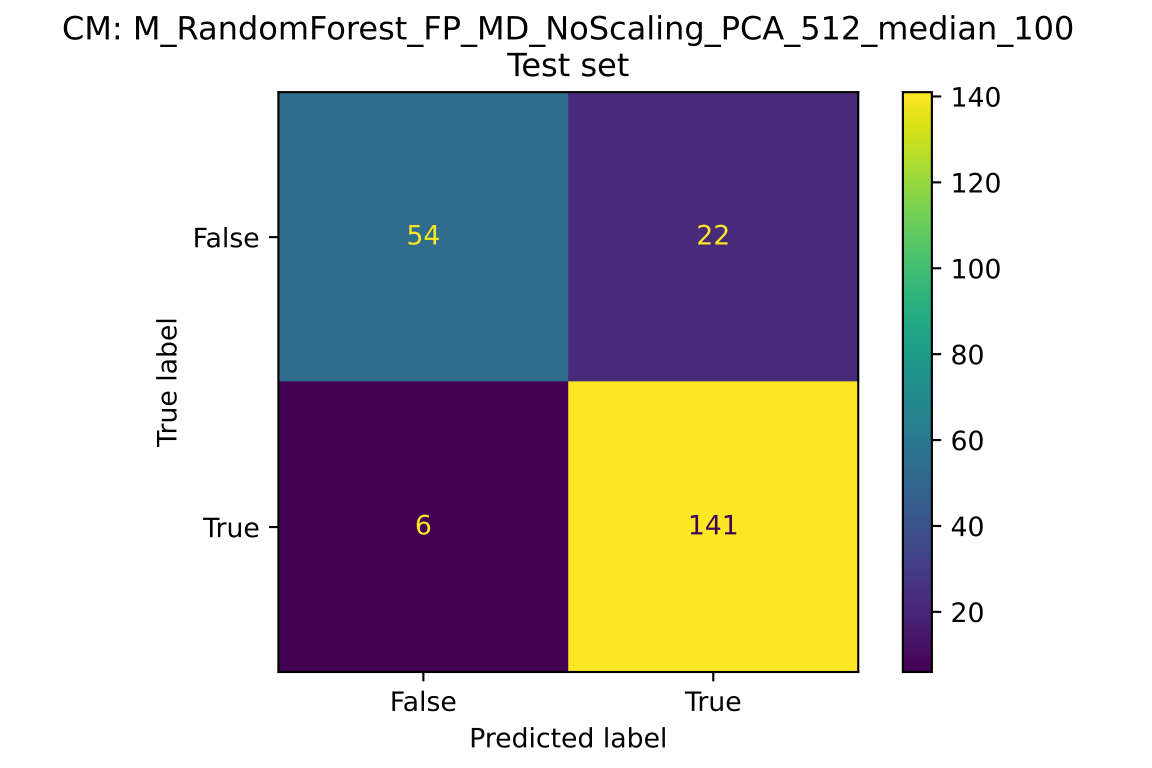

CM: M_RandomForest_FP_MD_NoScaling_PCA_512_median_100
Test set
54
22
6
141
False
True
True label
False
True
Predicted label
140
120
100
80
60
40
20

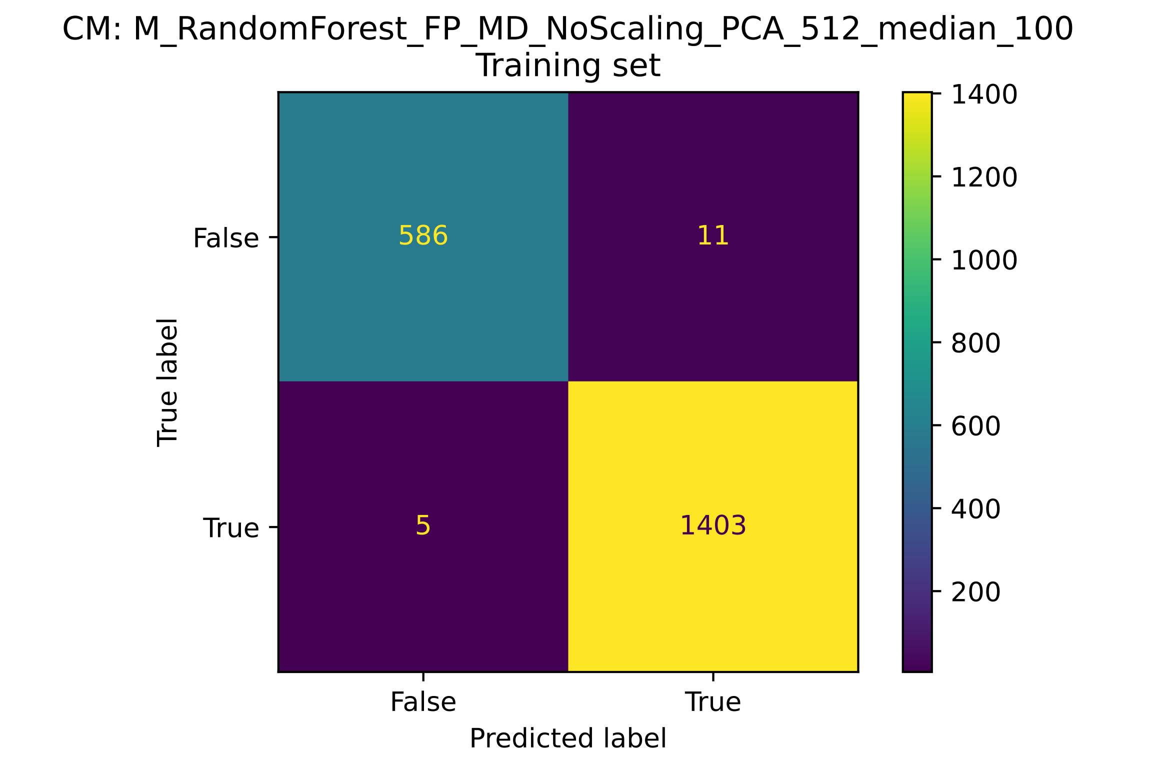
CM: M_RandomForest_FP_MD_NoScaling_PCA_512_median_100
Training set
586
11
5
1403
False
True
False
True
True label
Predicted label
1400
1200
1000
800
600
400
200

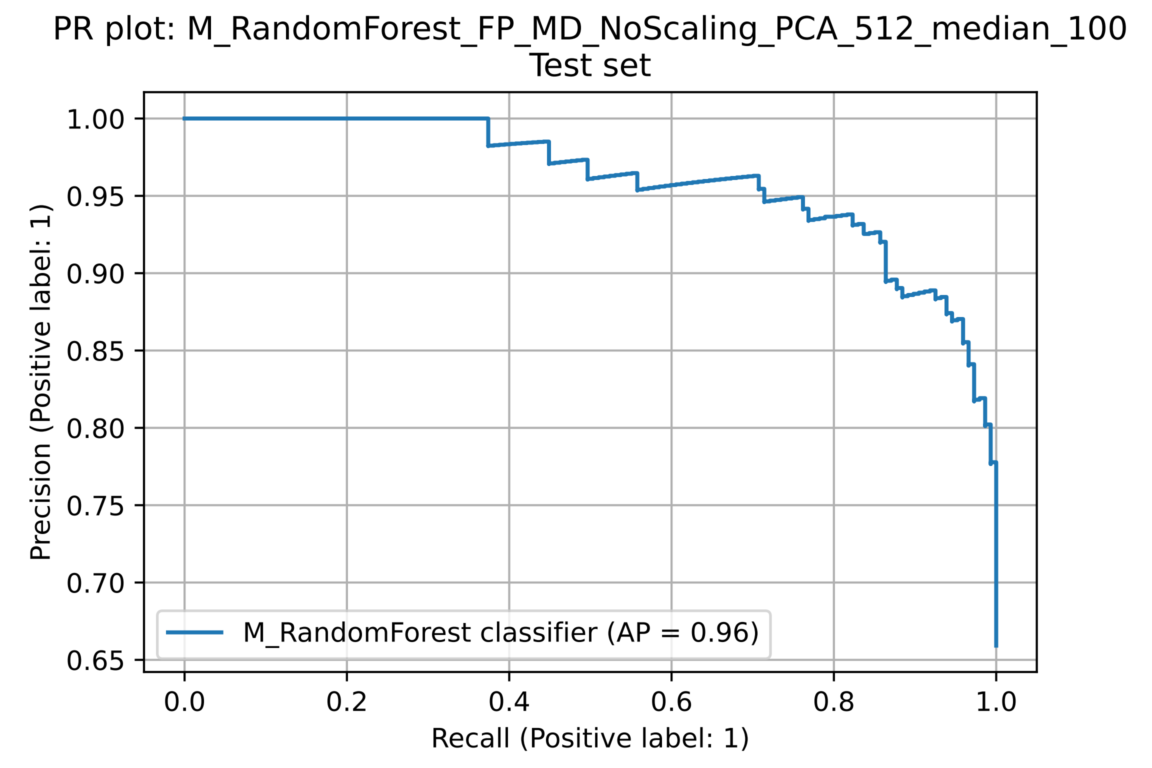

PR plot: M_RandomForest_FP_MD_NoScaling_PCA_512_median_100
Test set
Precision (Positive label: 1)
Recall (Positive label: 1)
1.00
0.95
0.90
0.85
0.80
0.75
0.70
0.65
0.0
0.2
0.4
0.6
0.8
1.0
M_RandomForest classifier (AP = 0.96)

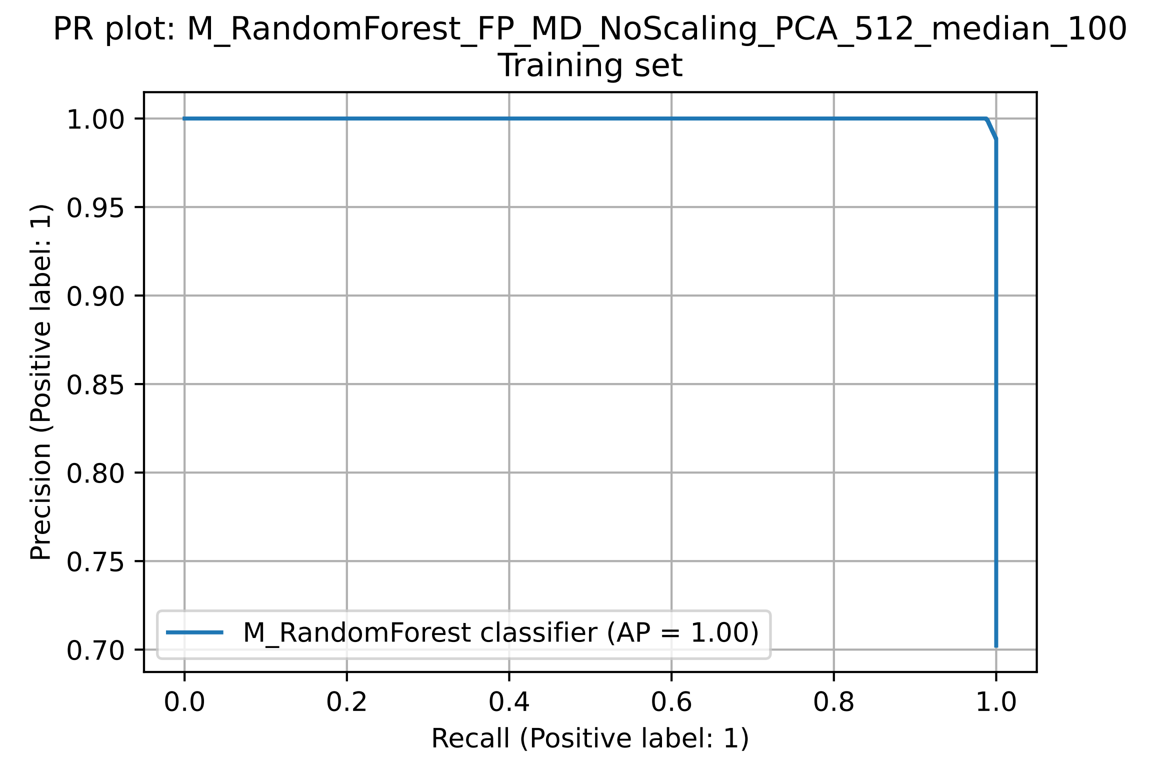
PR plot: M_RandomForest_FP_MD_NoScaling_PCA_512_median_100
Training set
1.00
0.95
0.90
0.85
0.80
0.75
0.70
Precision (Positive label: 1)
0.0
0.2
0.4
0.6
0.8
1.0
Recall (Positive label: 1)
M_RandomForest classifier (AP = 1.00)

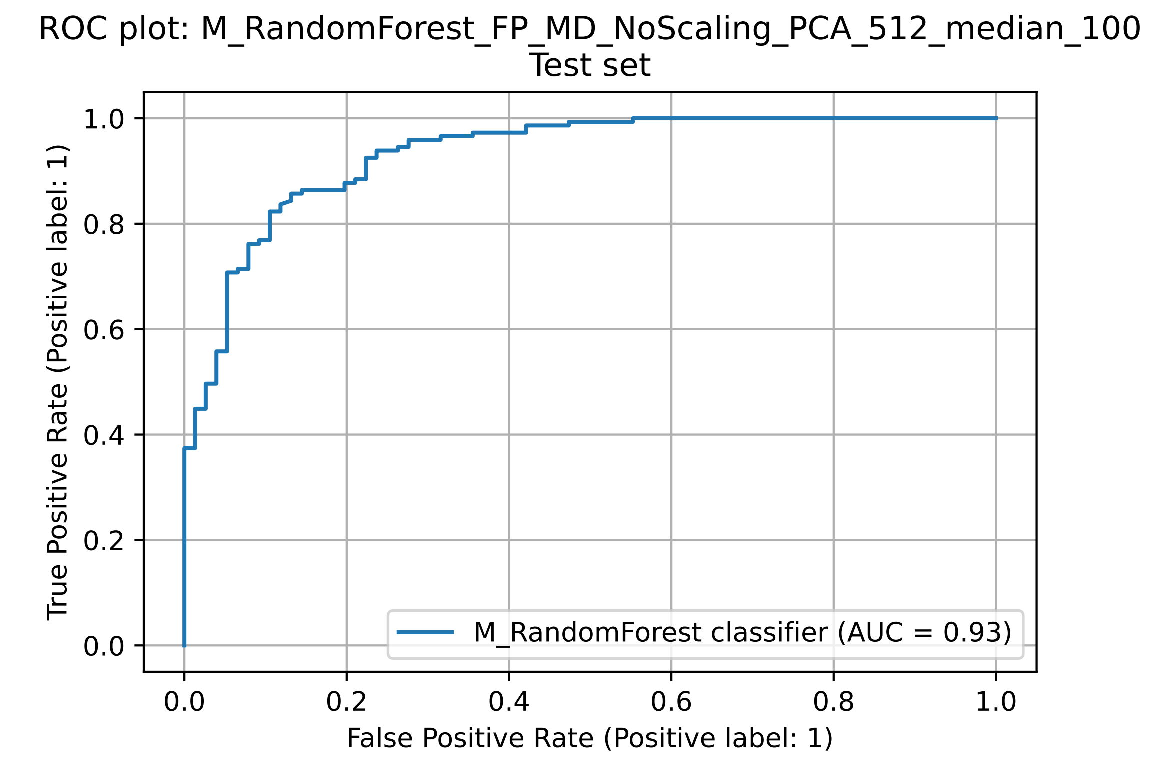

ROC plot: M_RandomForest_FP_MD_NoScaling_PCA_512_median_100
Test set
True Positive Rate (Positive label: 1)
False Positive Rate (Positive label: 1)
0.0
0.2
0.4
0.6
0.8
1.0
M_RandomForest classifier (AUC = 0.93)

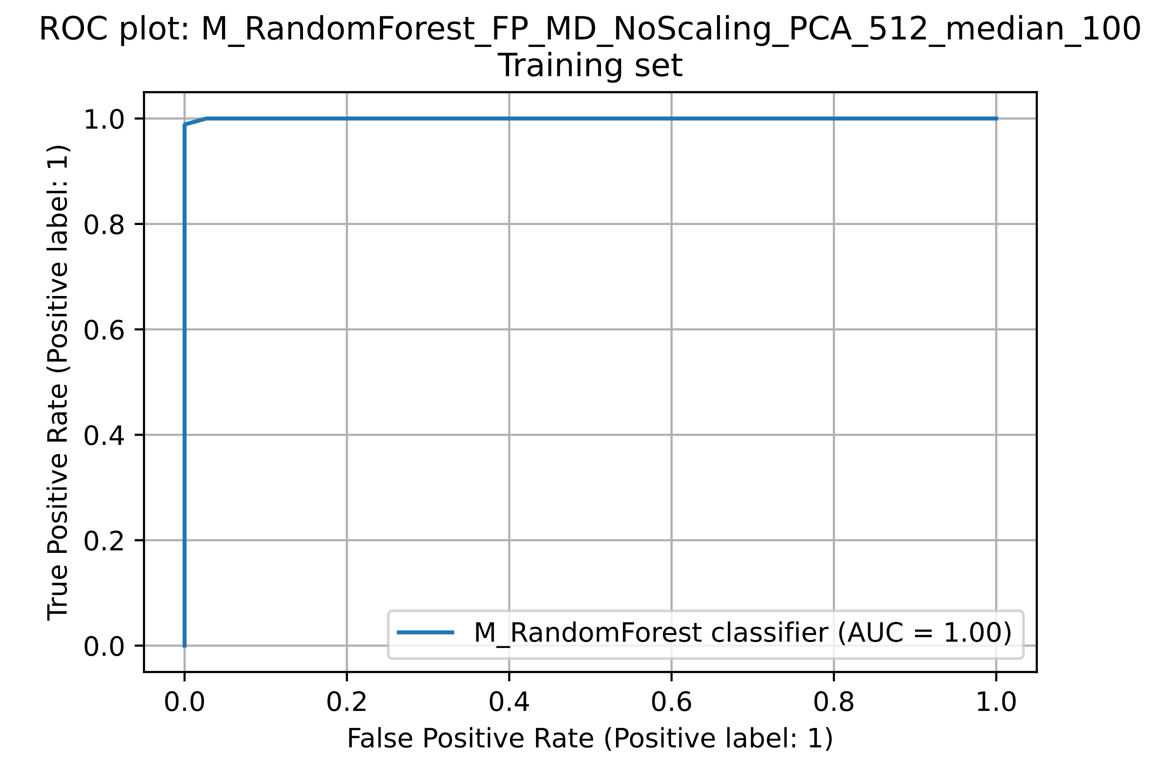
ROC plot: M_RandomForest_FP_MD_NoScaling_PCA_512_median_100
Training set
True Positive Rate (Positive label: 1)
False Positive Rate (Positive label: 1)
0.0
0.2
0.4
0.6
0.8
1.0
M_RandomForest classifier (AUC = 1.00)

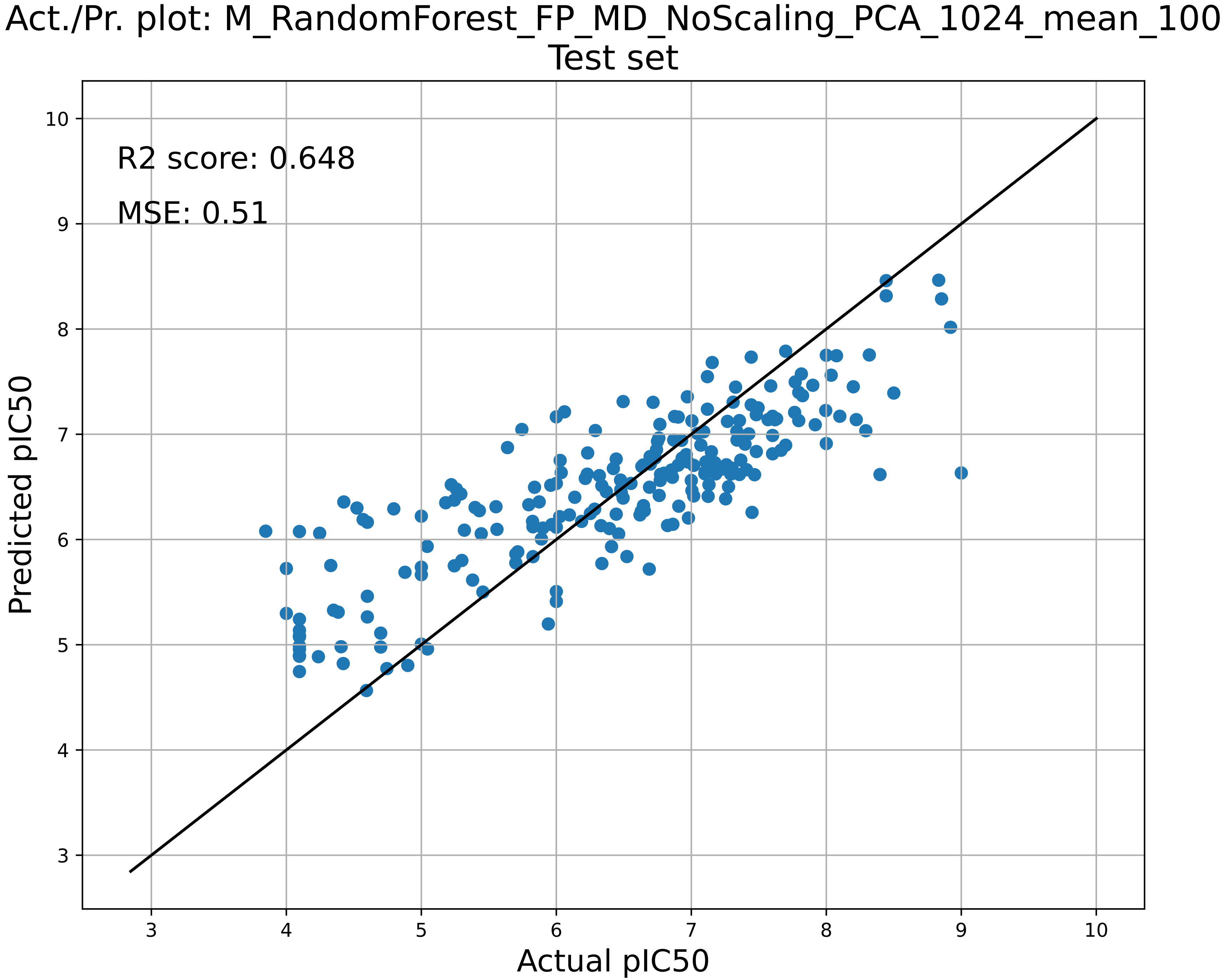
Act./Pr. plot: M_RandomForest_FP_MD_NoScaling_PCA_1024_mean_100
Test set
R2 score: 0.648
MSE: 0.51
Predicted pIC50
Actual pIC50
3
4
5
6
7
8
9
10

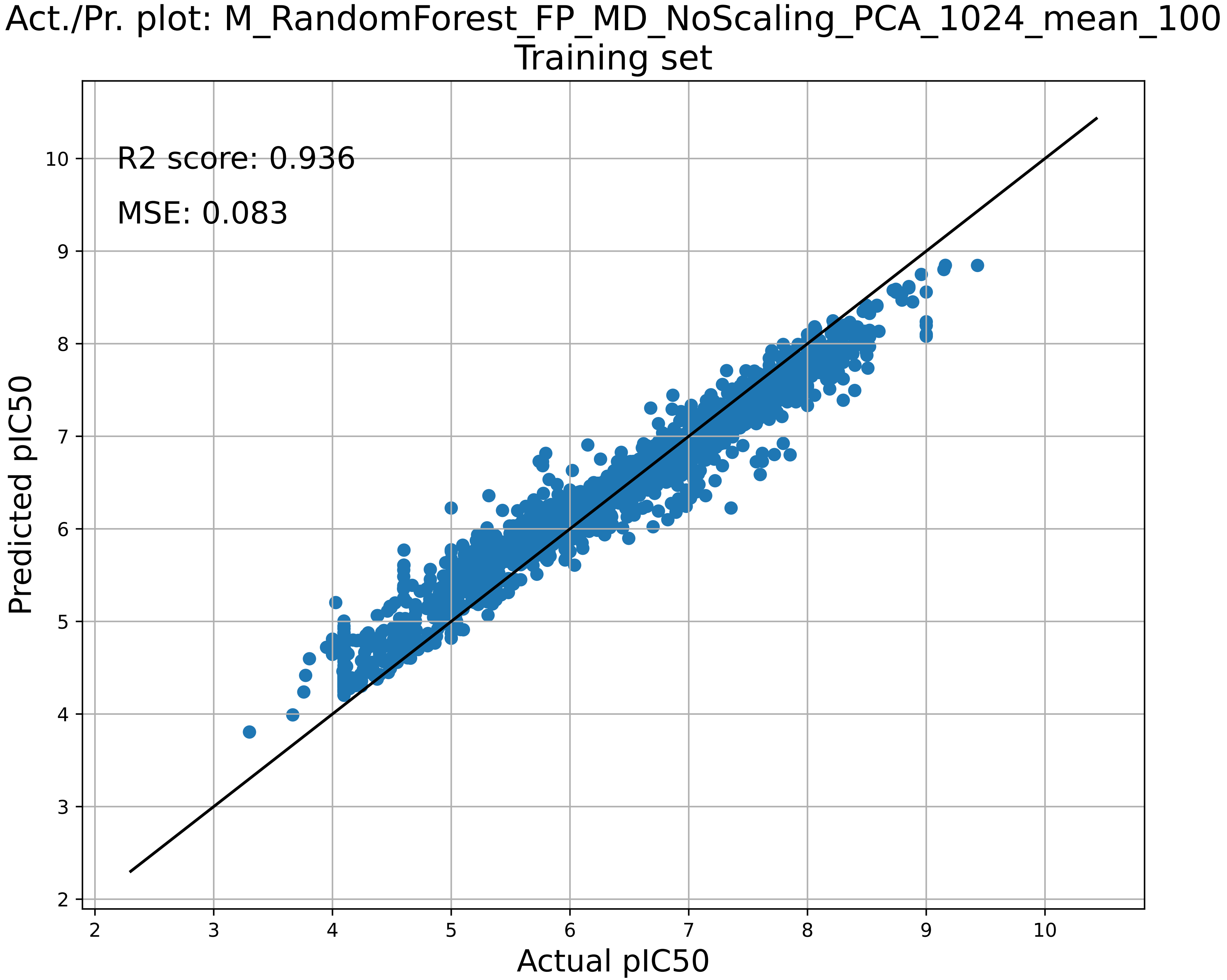

Act./Pr. plot: M_RandomForest_FP_MD_NoScaling_PCA_1024_mean_100
Training set
R2 score: 0.936
MSE: 0.083
Predicted pIC50
Actual pIC50
2
3
4
5
6
7
8
9
10

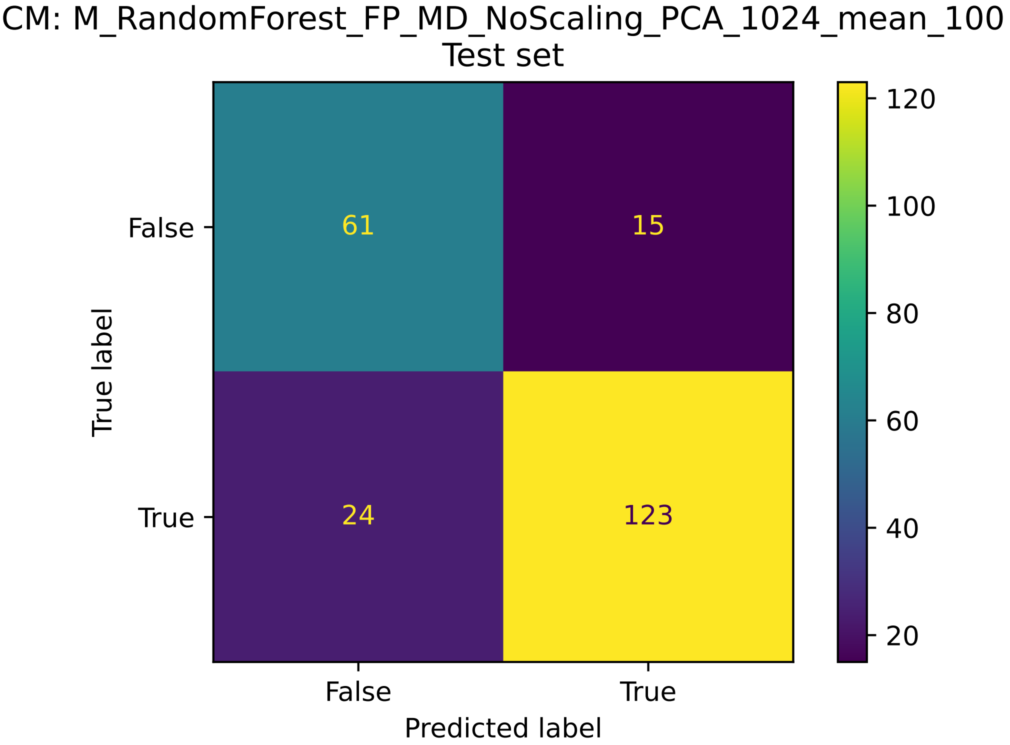
CM: M_RandomForest_FP_MD_NoScaling_PCA_1024_mean_100
Test set
True label
False
True
61
15
24
123
Predicted label
False
True
120
100
80
60
40
20

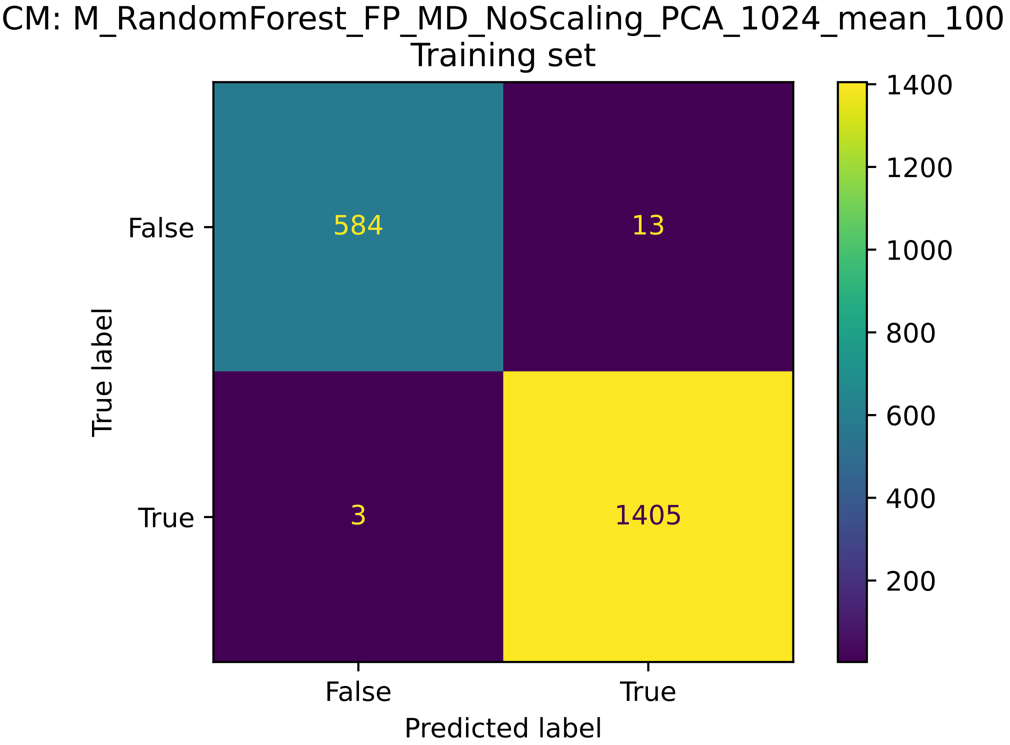
CM: M_RandomForest_FP_MD_NoScaling_PCA_1024_mean_100
Training set
True label
False
True
584
13
3
1405
False
True
Predicted label
1400
1200
1000
800
600
400
200

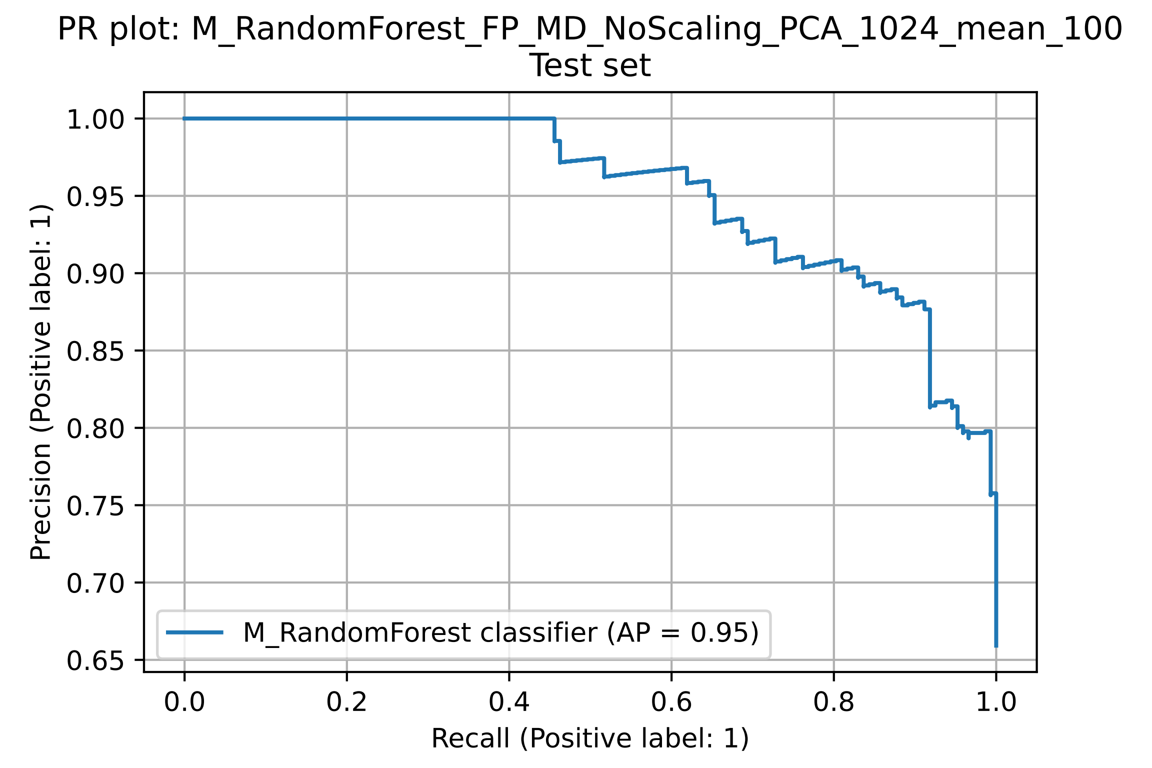
PR plot: M_RandomForest_FP_MD_NoScaling_PCA_1024_mean_100
Test set
1.00
0.95
0.90
0.85
0.80
0.75
0.70
0.65
Precision (Positive label: 1)
0.0
0.2
0.4
0.6
0.8
1.0
Recall (Positive label: 1)
M_RandomForest classifier (AP = 0.95)

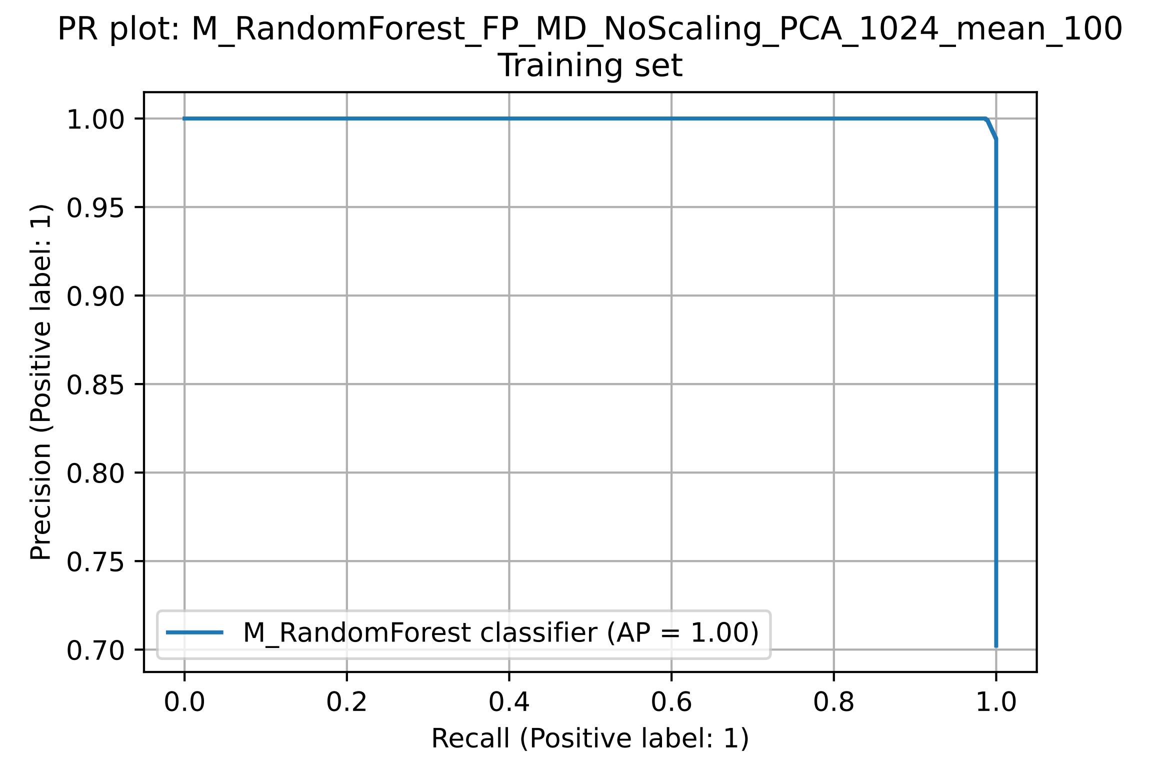

PR plot: M_RandomForest_FP_MD_NoScaling_PCA_1024_mean_100
Training set
Precision (Positive label: 1)
1.00
0.95
0.90
0.85
0.80
0.75
0.70
0.0
0.2
0.4
0.6
0.8
1.0
Recall (Positive label: 1)
M_RandomForest classifier (AP = 1.00)

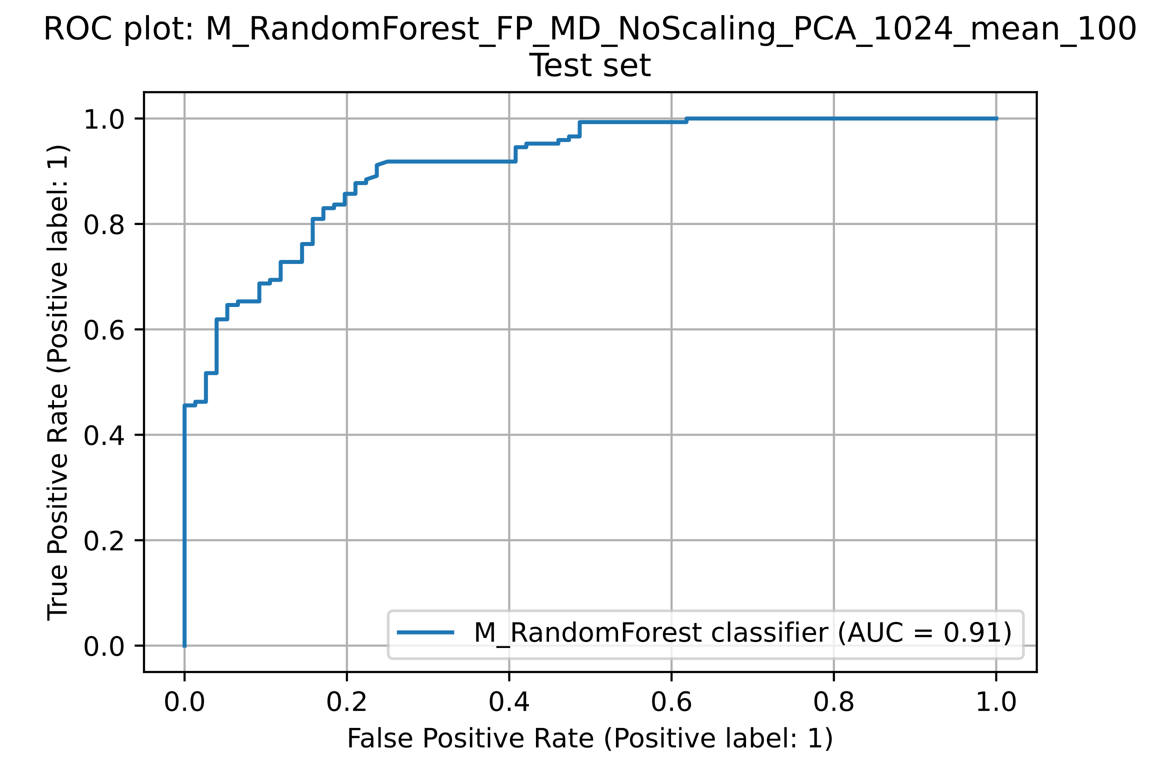
ROC plot: M_RandomForest_FP_MD_NoScaling_PCA_1024_mean_100
Test set
True Positive Rate (Positive label: 1)
False Positive Rate (Positive label: 1)
0.0
0.2
0.4
0.6
0.8
1.0
M_RandomForest classifier (AUC = 0.91)

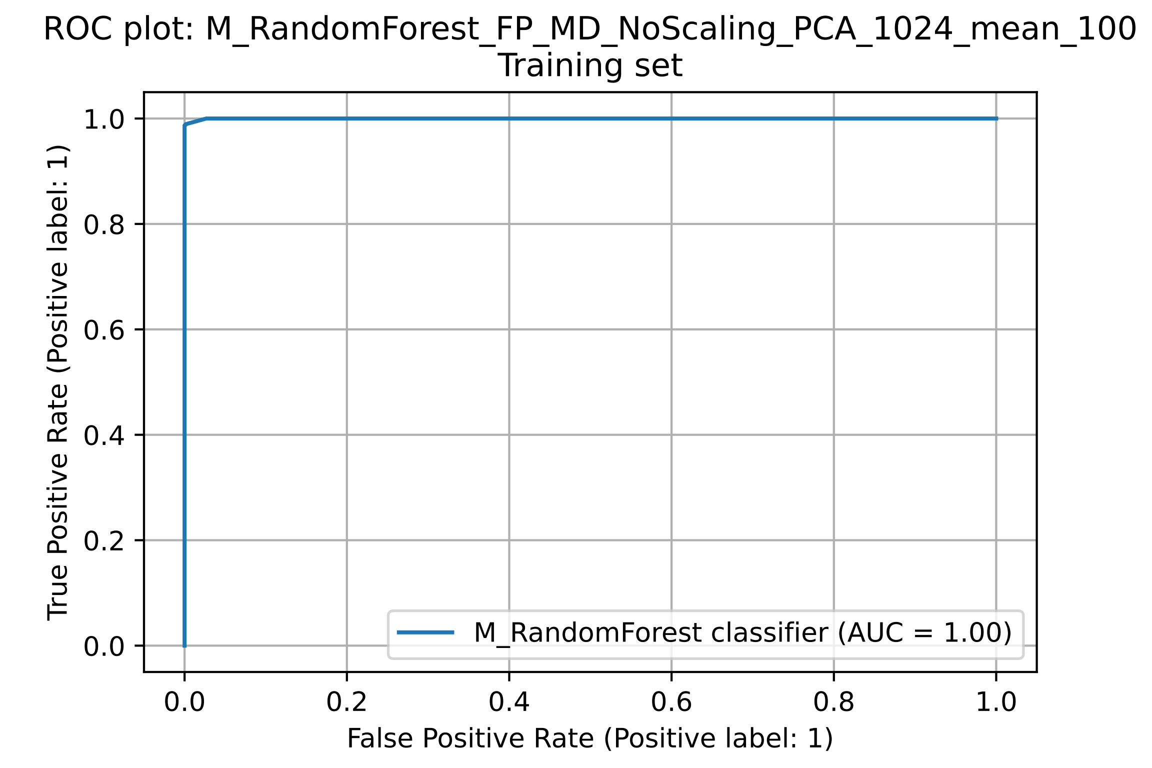
ROC plot: M_RandomForest_FP_MD_NoScaling_PCA_1024_mean_100
Training set
True Positive Rate (Positive label: 1)
1.0
0.8
0.6
0.4
0.2
0.0
0.0
0.2
0.4
0.6
0.8
1.0
False Positive Rate (Positive label: 1)
M_RandomForest classifier (AUC = 1.00)

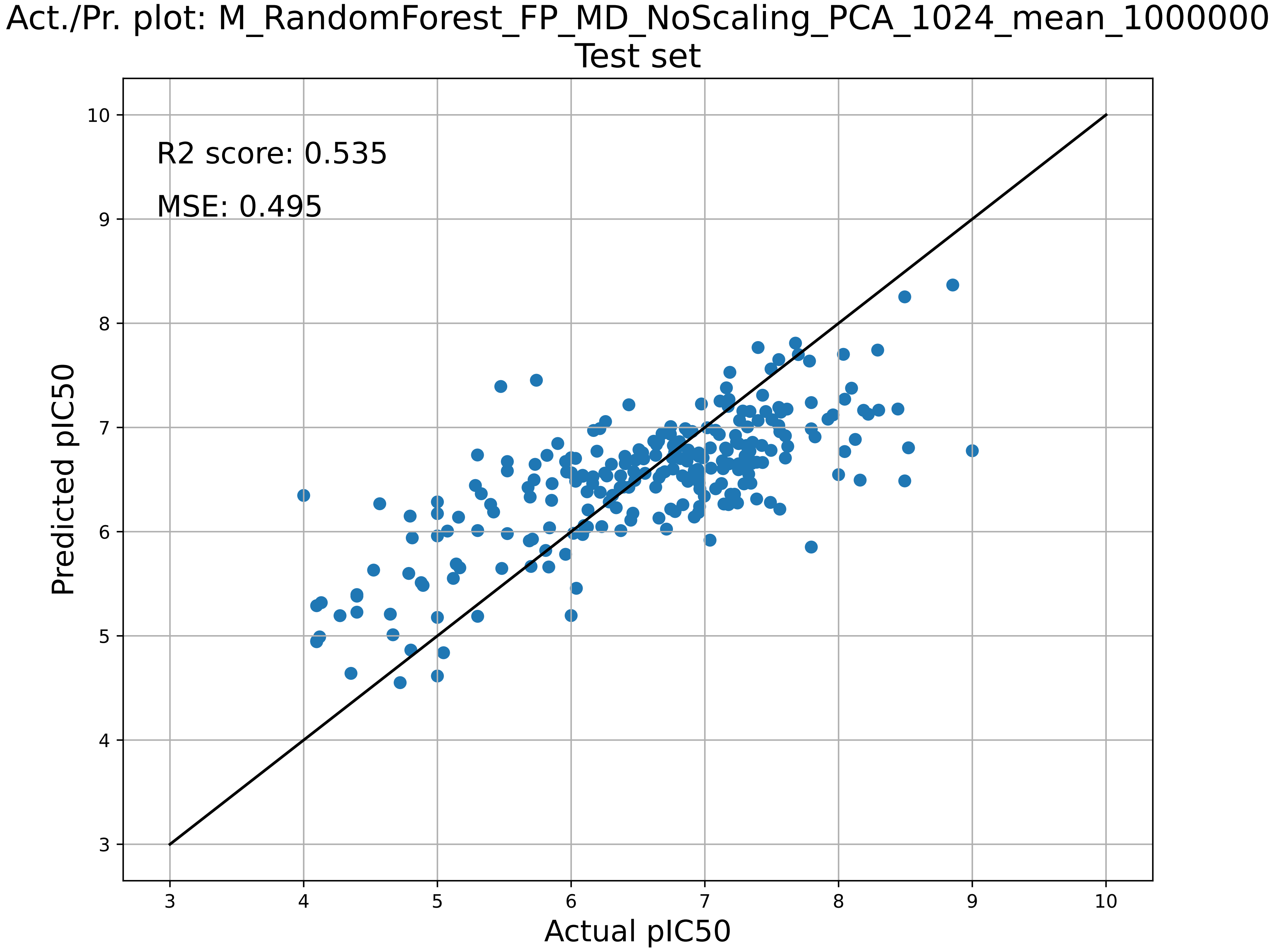
Act./Pr. plot: M_RandomForest_FP_MD_NoScaling_PCA_1024_mean_1000000
Test set
R2 score: 0.535
MSE: 0.495
Predicted pIC50
Actual pIC50
3
4
5
6
7
8
9
10

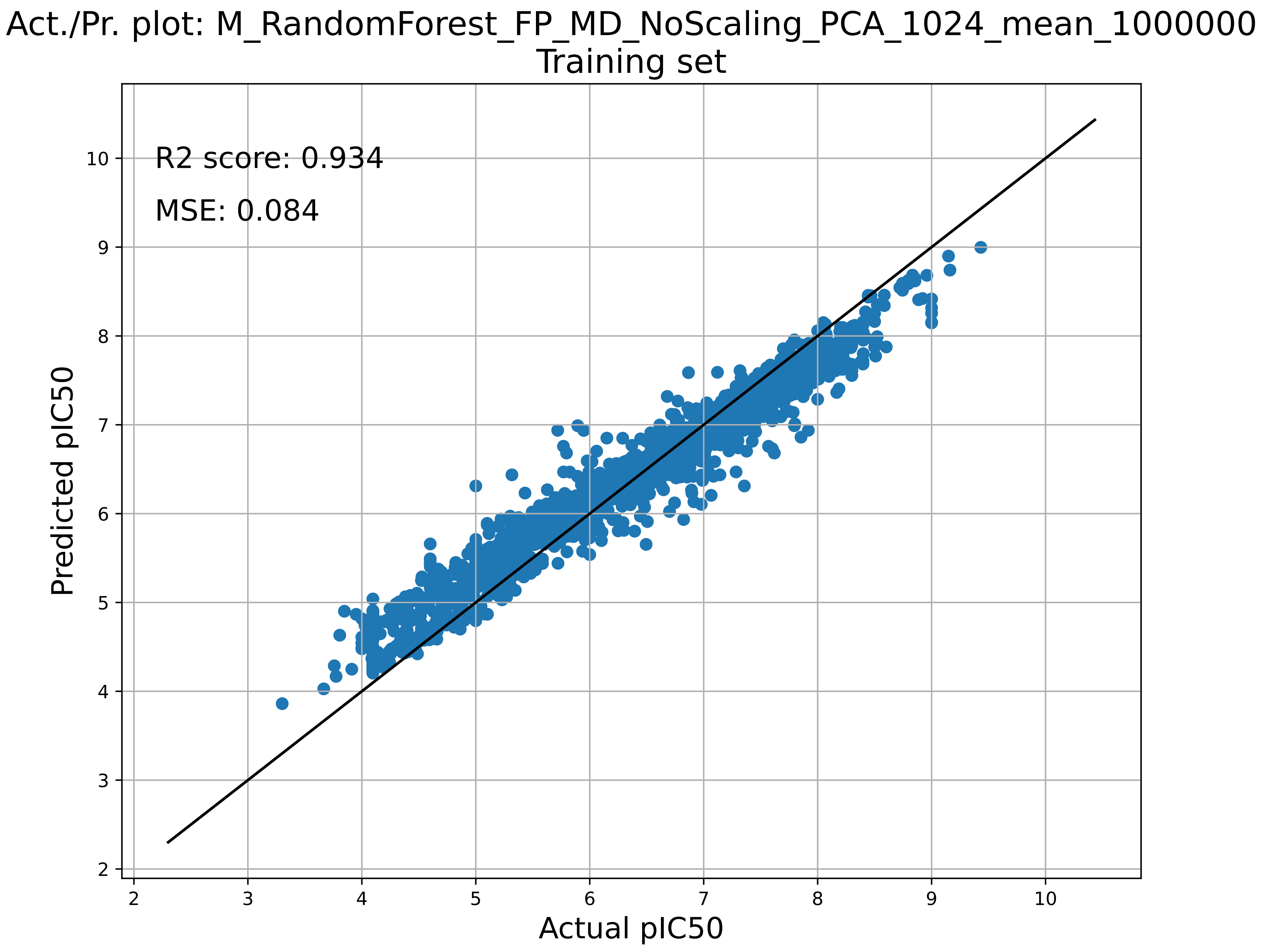
Act./Pr. plot: M_RandomForest_FP_MD_NoScaling_PCA_1024_mean_1000000
Training set
R2 score: 0.934
MSE: 0.084
Predicted pIC50
Actual pIC50
2
3
4
5
6
7
8
9
10

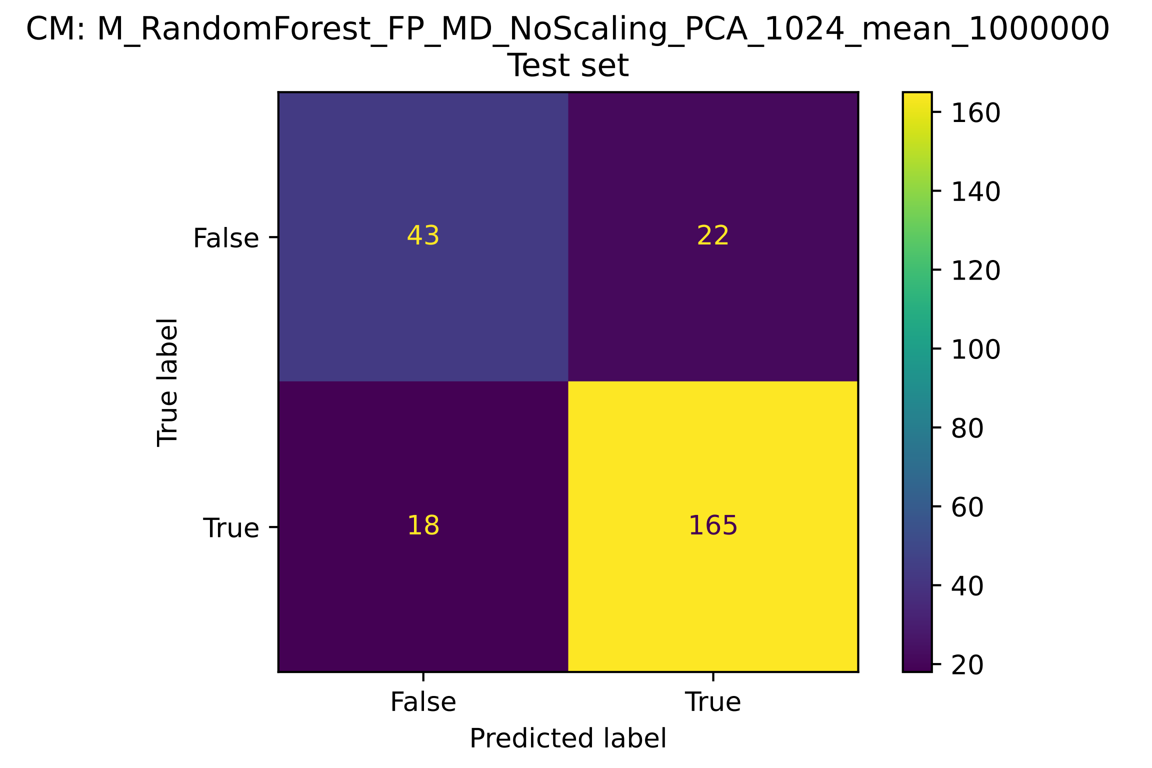

CM: M_RandomForest_FP_MD_NoScaling_PCA_1024_mean_1000000
Test set
True label
Predicted label
False
True
False
True
43
22
18
165
160
140
120
100
80
60
40
20

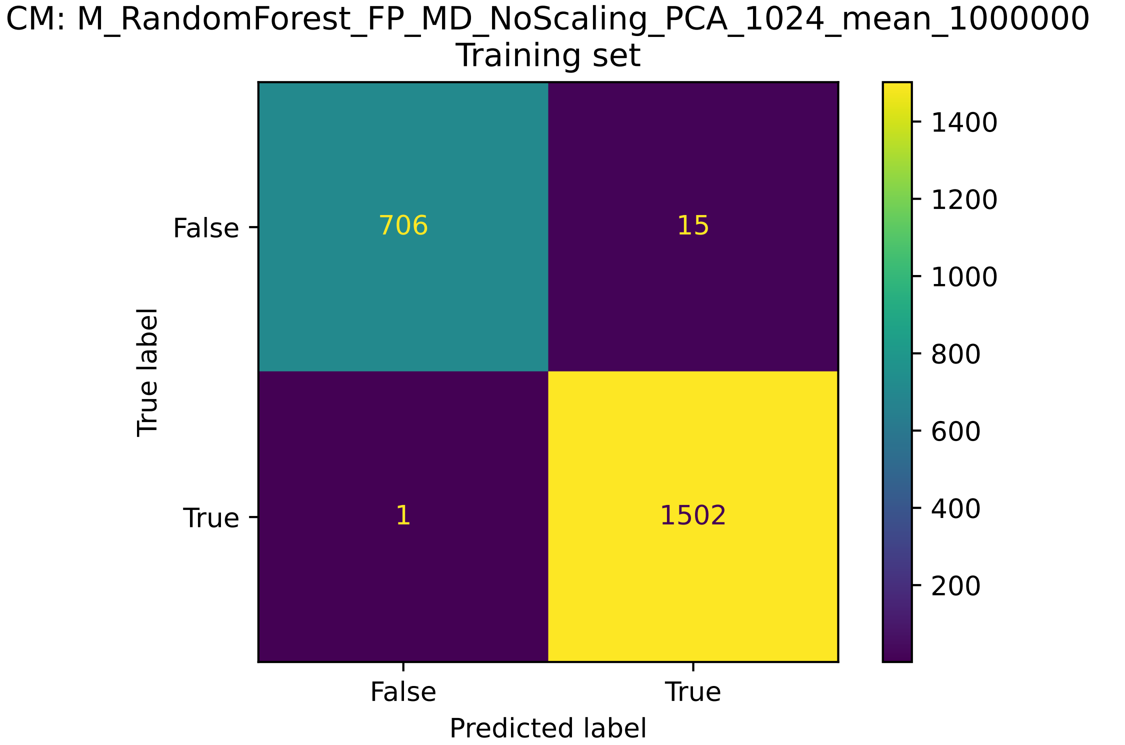
CM: M_RandomForest_FP_MD_NoScaling_PCA_1024_mean_1000000
Training set
706
15
1
1502
False
True
True label
False
True
Predicted label
1400
1200
1000
800
600
400
200

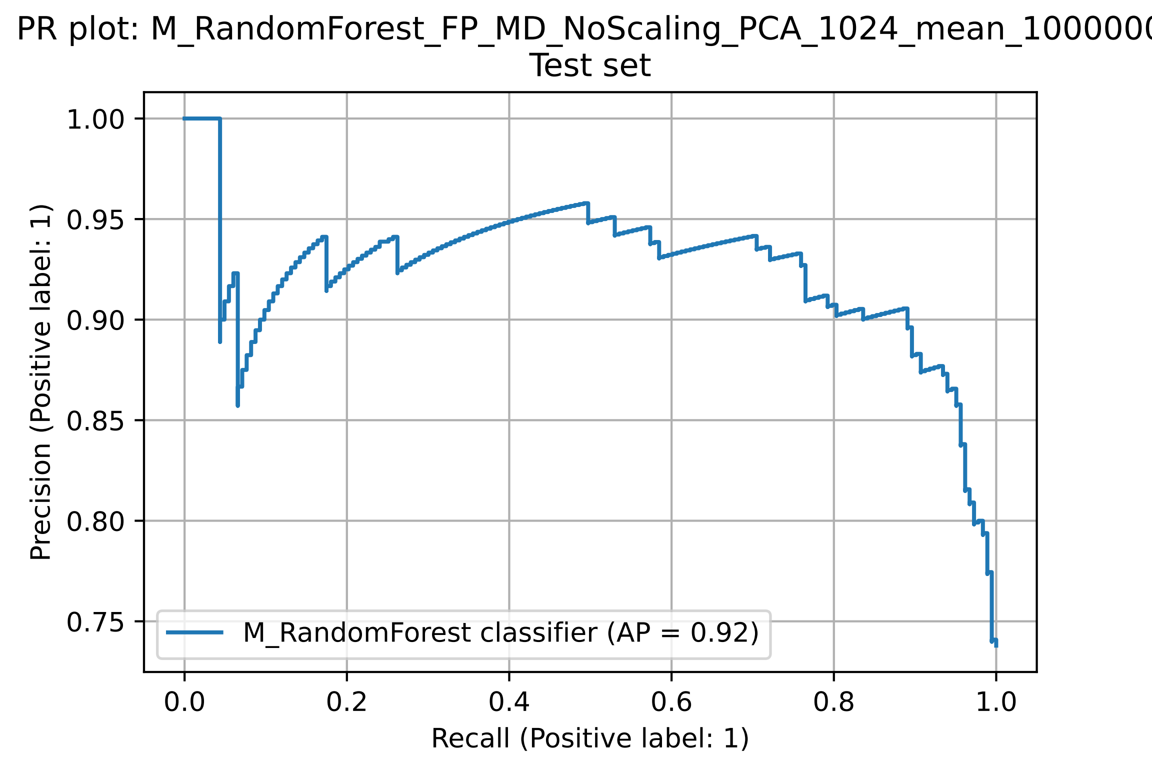

PR plot: M_RandomForest_FP_MD_NoScaling_PCA_1024_mean_1000000
Test set
1.00
0.95
0.90
0.85
0.80
0.75
Precision (Positive label: 1)
0.0
0.2
0.4
0.6
0.8
1.0
Recall (Positive label: 1)
M_RandomForest classifier (AP = 0.92)

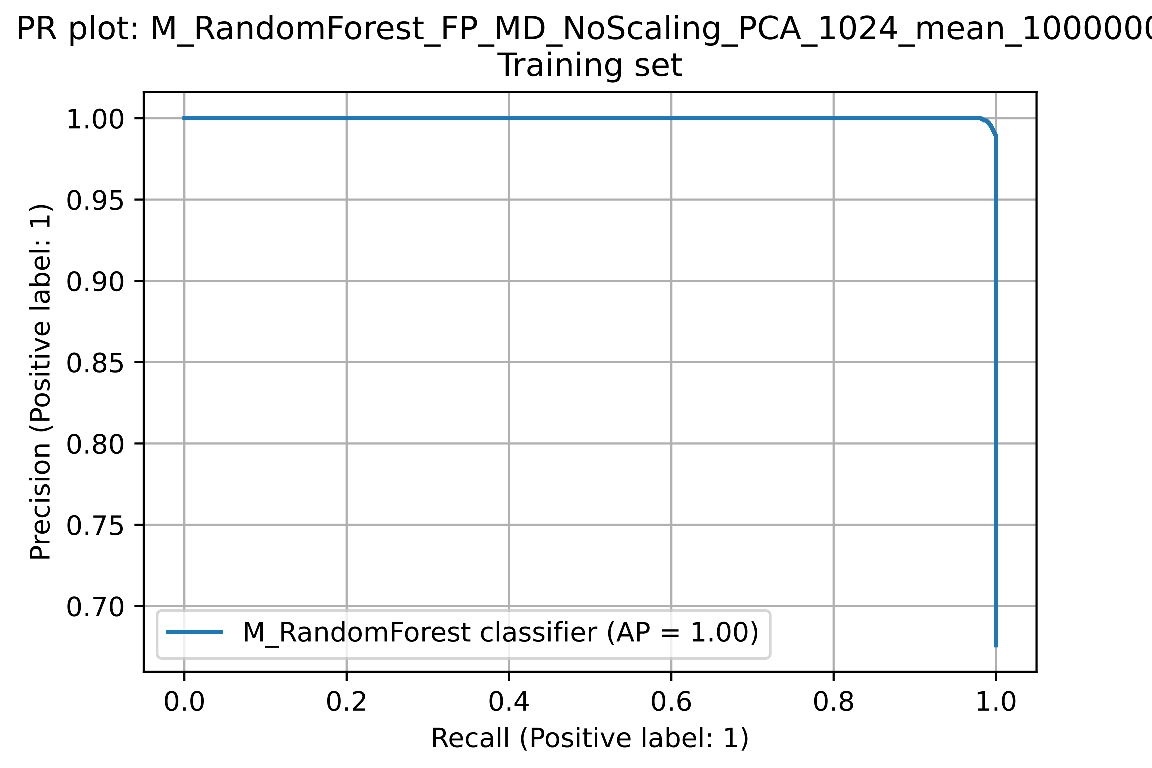
PR plot: M_RandomForest_FP_MD_NoScaling_PCA_1024_mean_100000
Training set
Precision (Positive label: 1)
1.00
0.95
0.90
0.85
0.80
0.75
0.70
Recall (Positive label: 1)
0.0
0.2
0.4
0.6
0.8
1.0
M_RandomForest classifier (AP = 1.00)

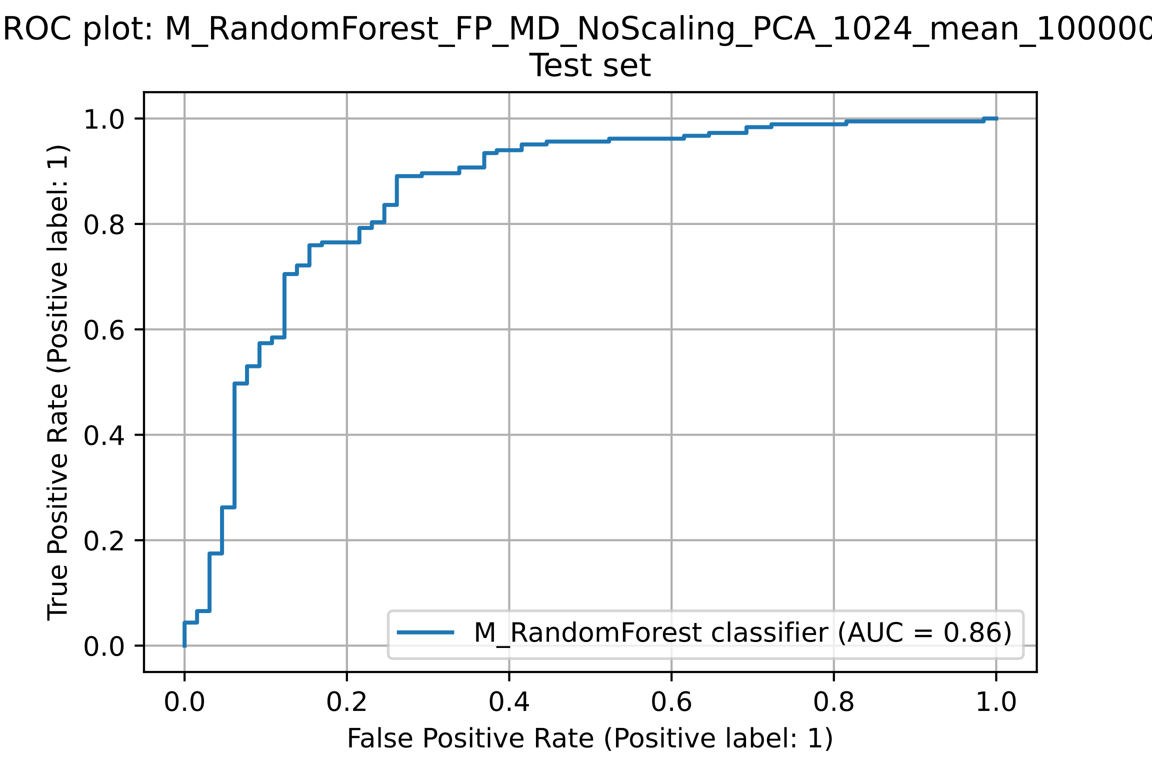
ROC plot: M_RandomForest_FP_MD_NoScaling_PCA_1024_mean_100000
Test set
True Positive Rate (Positive label: 1)
False Positive Rate (Positive label: 1)
0.0
0.2
0.4
0.6
0.8
1.0
M_RandomForest classifier (AUC = 0.86)

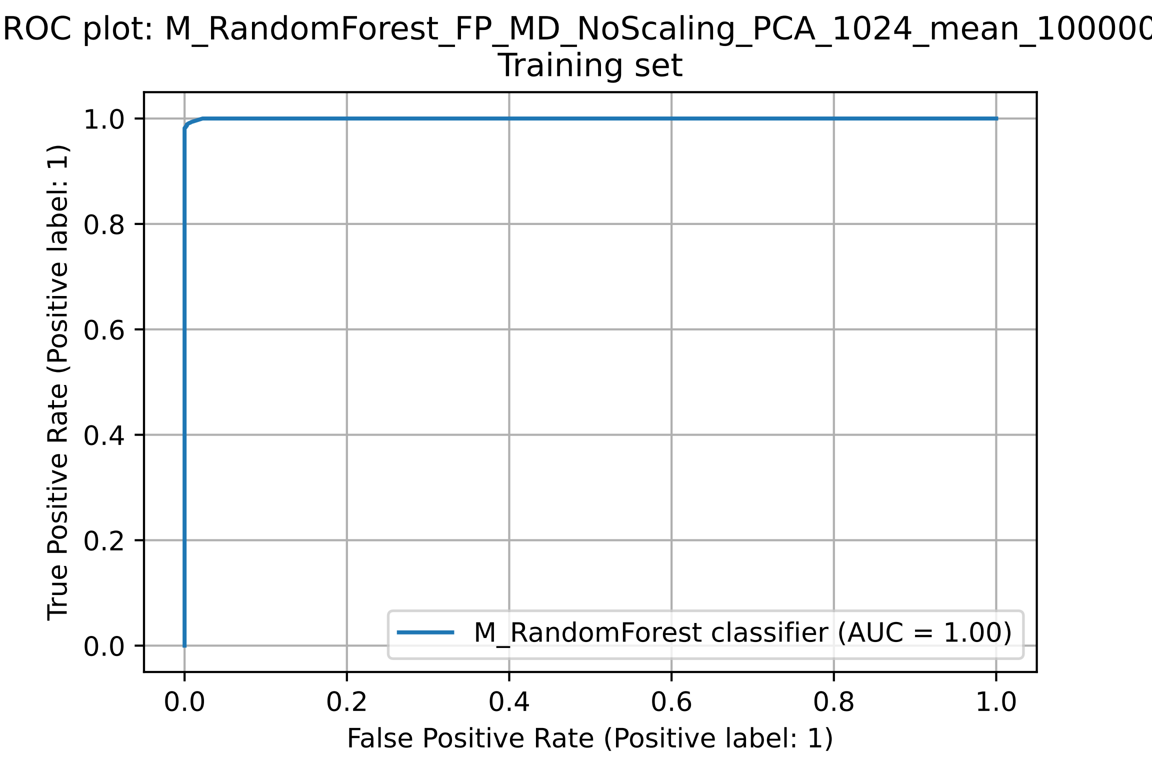

ROC plot: M_RandomForest_FP_MD_NoScaling_PCA_1024_mean_10000
Training set
True Positive Rate (Positive label: 1)
False Positive Rate (Positive label: 1)
0.0
0.2
0.4
0.6
0.8
1.0
M_RandomForest classifier (AUC = 1.00)

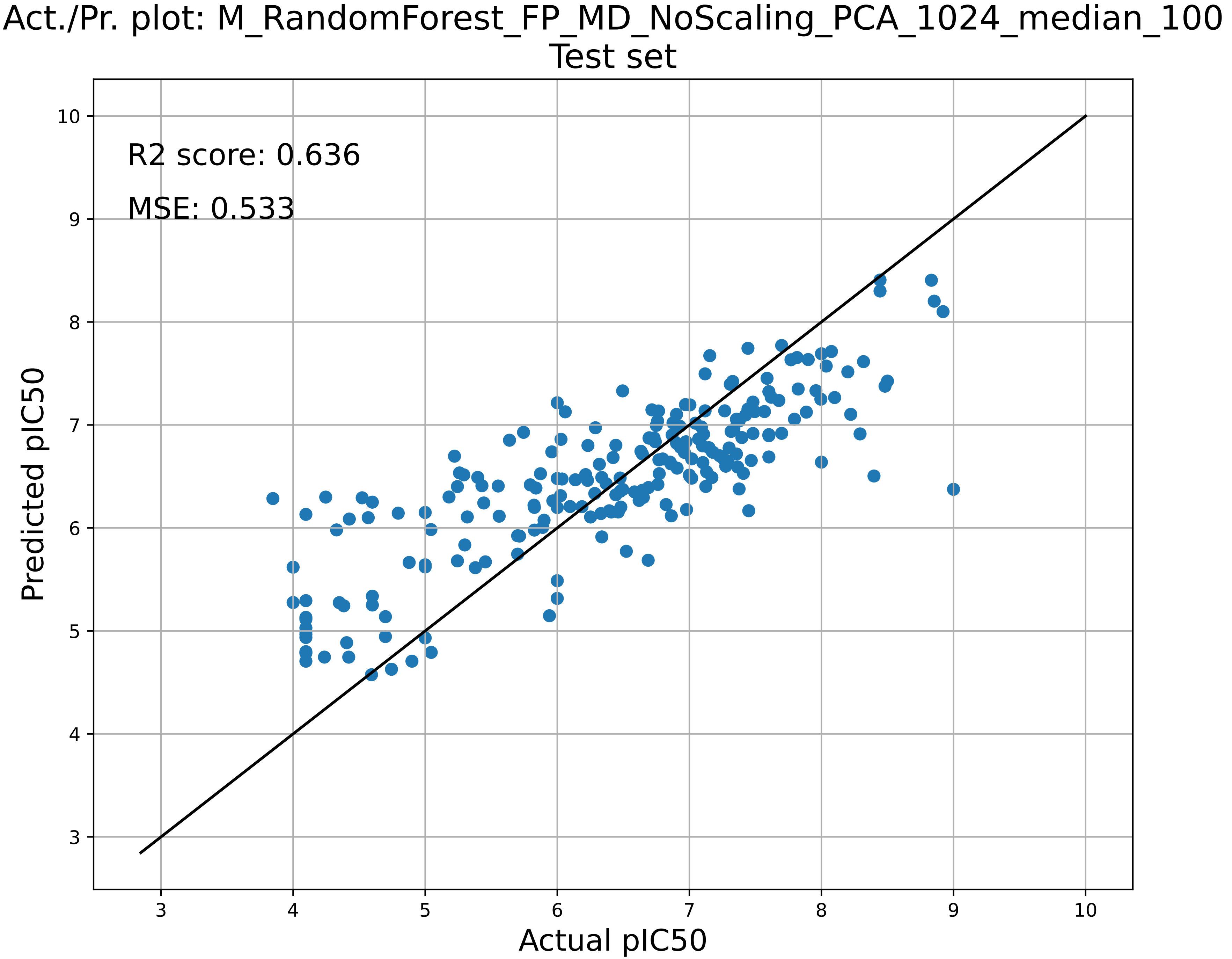
Act./Pr. plot: M_RandomForest_FP_MD_NoScaling_PCA_1024_median_100
Test set
R2 score: 0.636
MSE: 0.533
Predicted pIC50
Actual pIC50
3
4
5
6
7
8
9
10

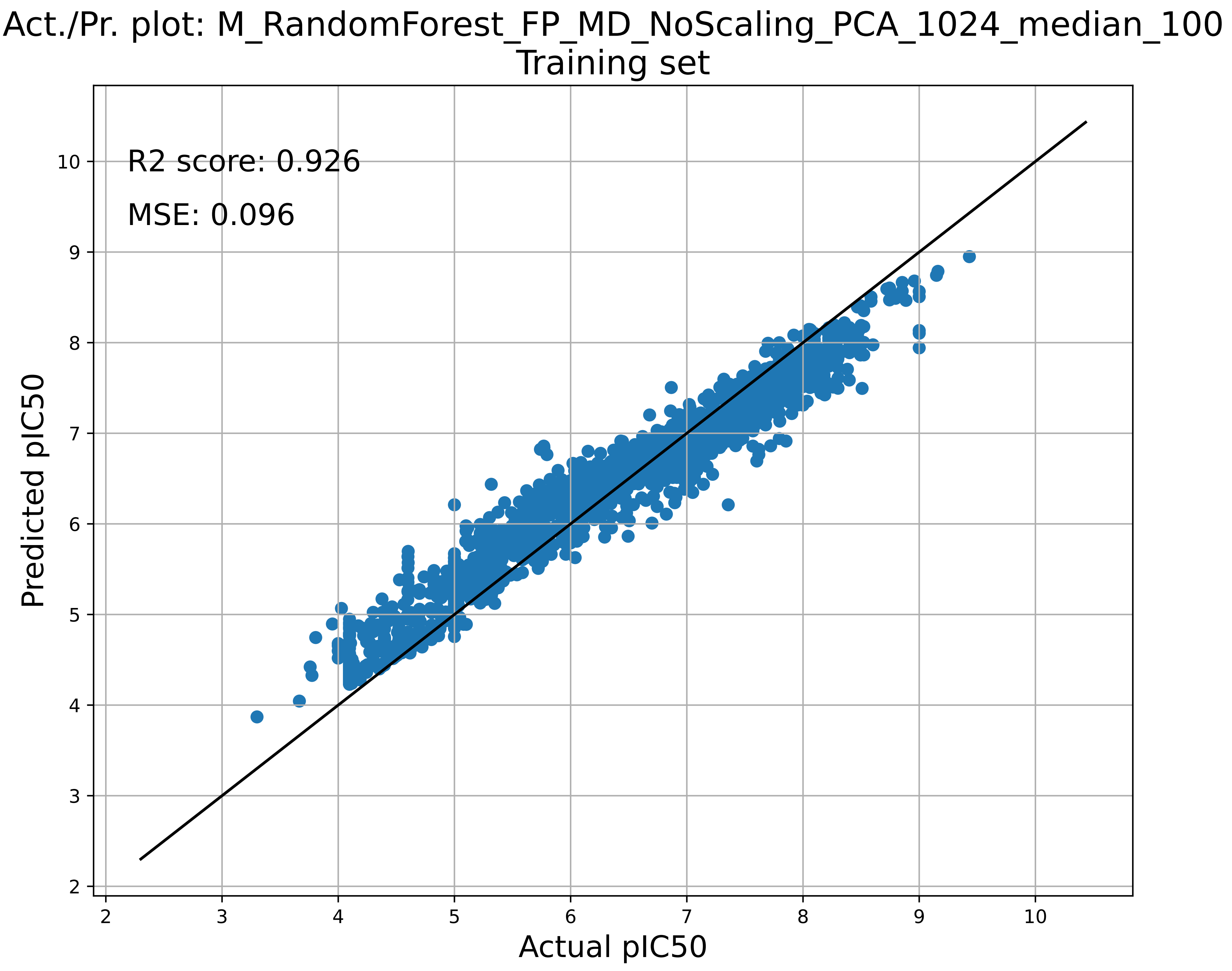
Act./Pr. plot: M_RandomForest_FP_MD_NoScaling_PCA_1024_median_100
Training set
R2 score: 0.926
MSE: 0.096
Predicted pIC50
Actual pIC50
2
3
4
5
6
7
8
9
10
2
3
4
5
6
7
8
9
10

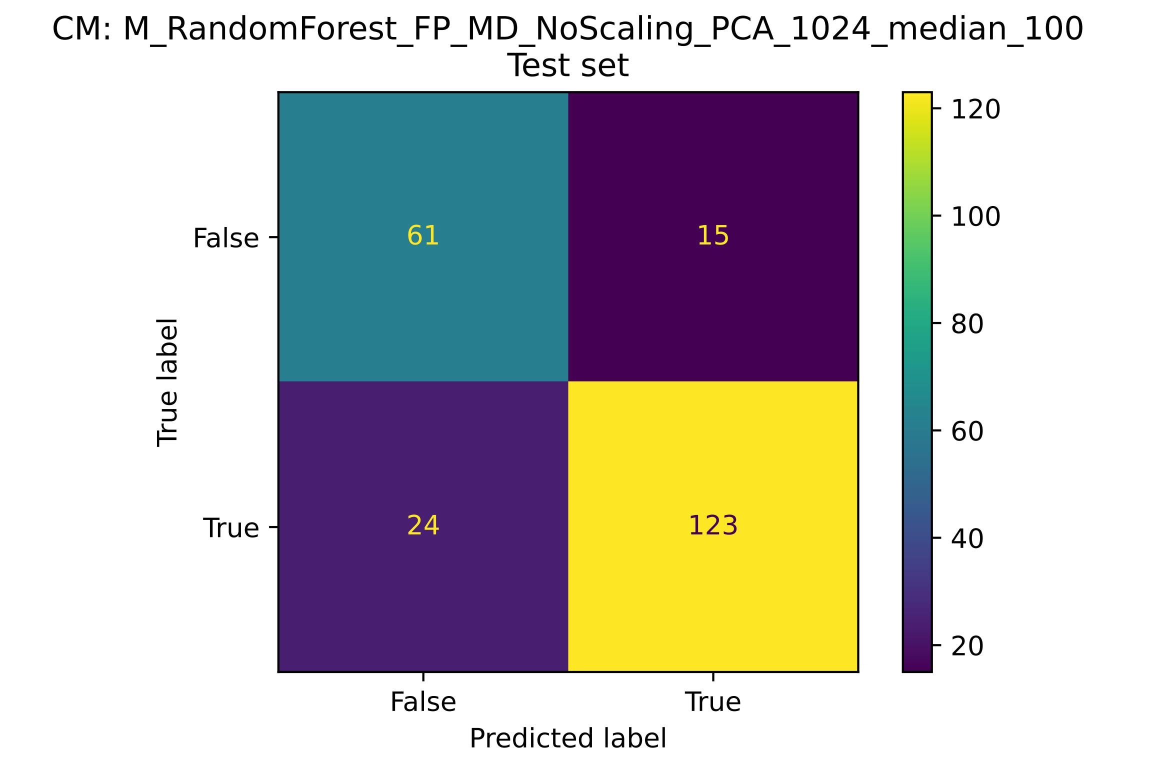
CM: M_RandomForest_FP_MD_NoScaling_PCA_1024_median_100
Test set
True label
False
True
61
15
24
123
Predicted label
False
True
120
100
80
60
40
20

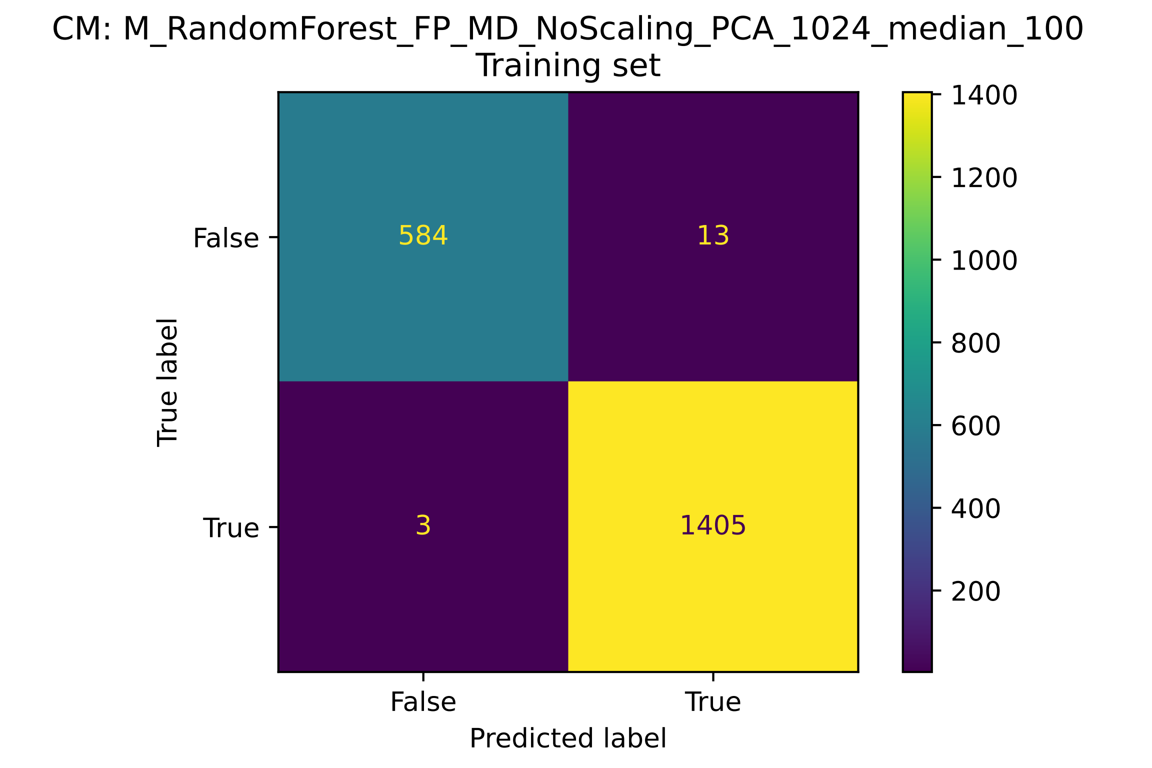

CM: M_RandomForest_FP_MD_NoScaling_PCA_1024_median_100
Training set
True label
Predicted label
False
True
False
True
584
13
3
1405
1400
1200
1000
800
600
400
200

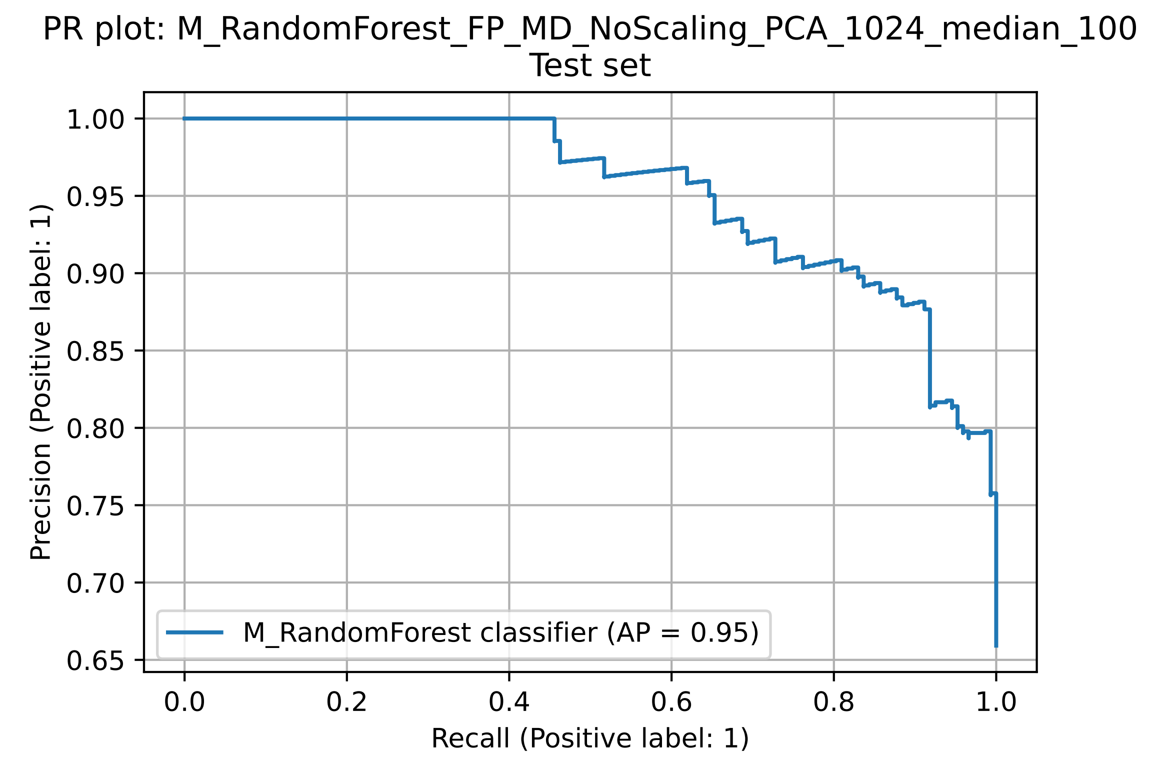

PR plot: M_RandomForest_FP_MD_NoScaling_PCA_1024_median_100
Test set
Precision (Positive label: 1)
Recall (Positive label: 1)
1.00
0.95
0.90
0.85
0.80
0.75
0.70
0.65
0.0
0.2
0.4
0.6
0.8
1.0
M_RandomForest classifier (AP = 0.95)

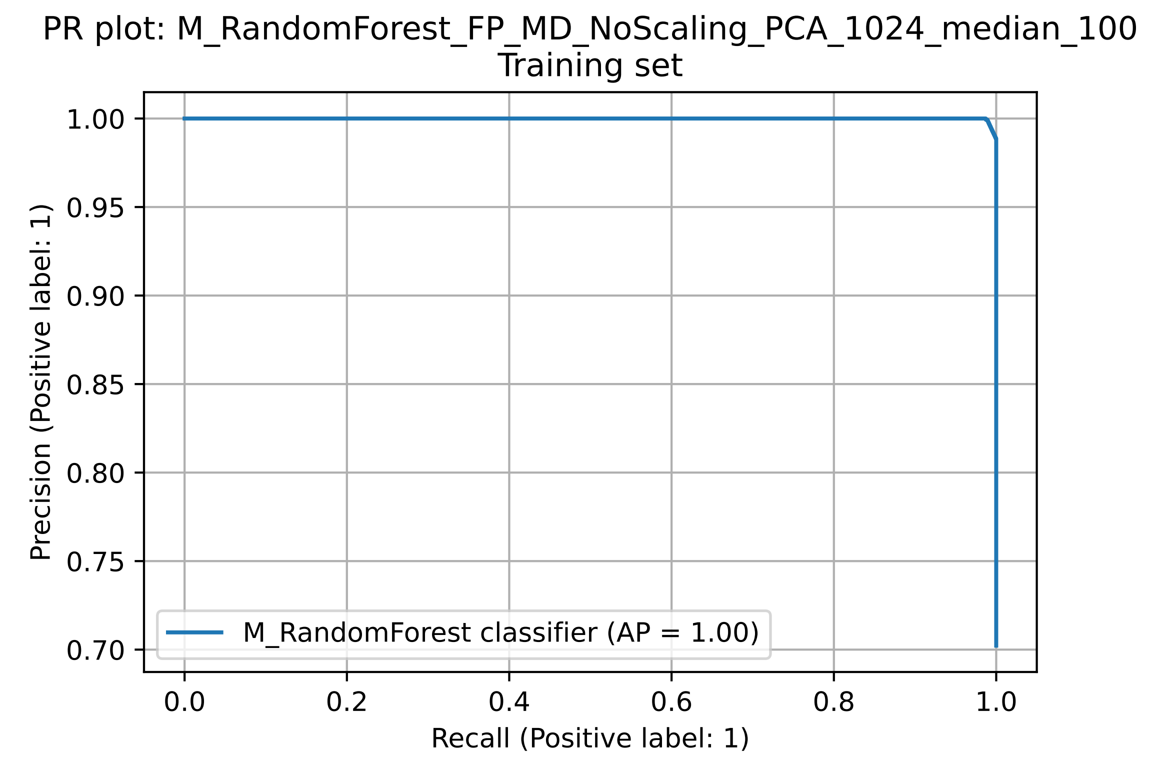
PR plot: M_RandomForest_FP_MD_NoScaling_PCA_1024_median_100
Training set
Precision (Positive label: 1)
1.00
0.95
0.90
0.85
0.80
0.75
0.70
Recall (Positive label: 1)
0.0
0.2
0.4
0.6
0.8
1.0
M_RandomForest classifier (AP = 1.00)

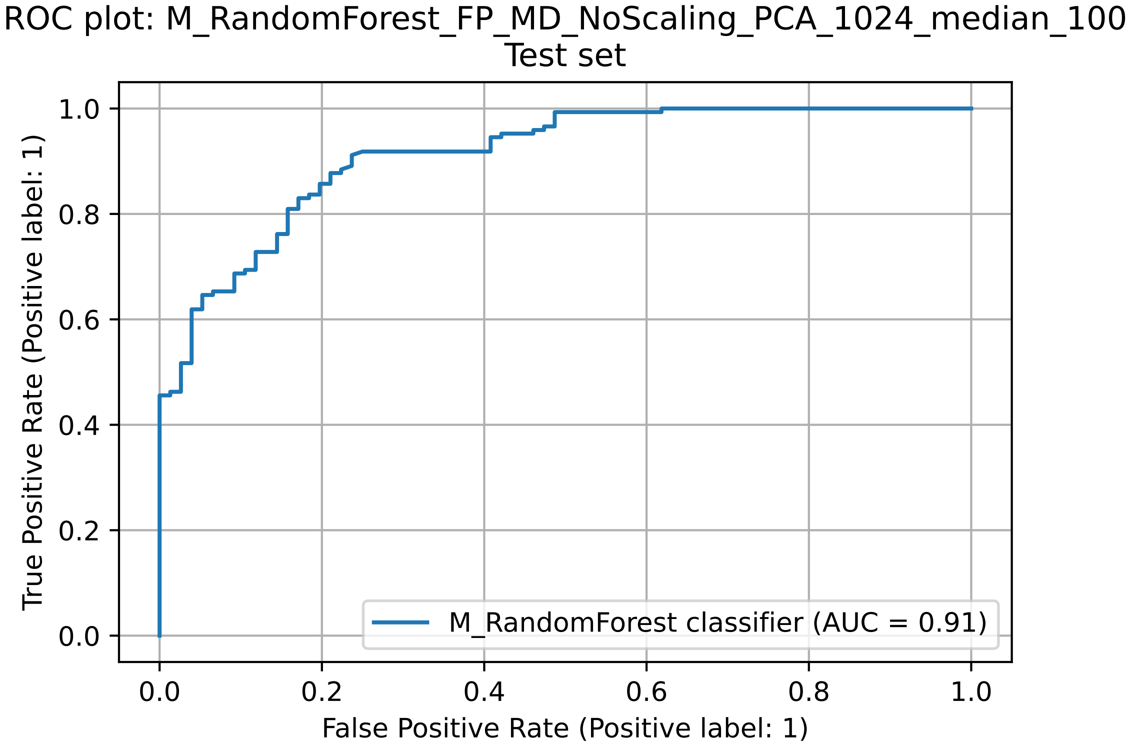
ROC plot: M_RandomForest_FP_MD_NoScaling_PCA_1024_median_100
Test set
True Positive Rate (Positive label: 1)
False Positive Rate (Positive label: 1)
0.0
0.2
0.4
0.6
0.8
1.0
M_RandomForest classifier (AUC = 0.91)

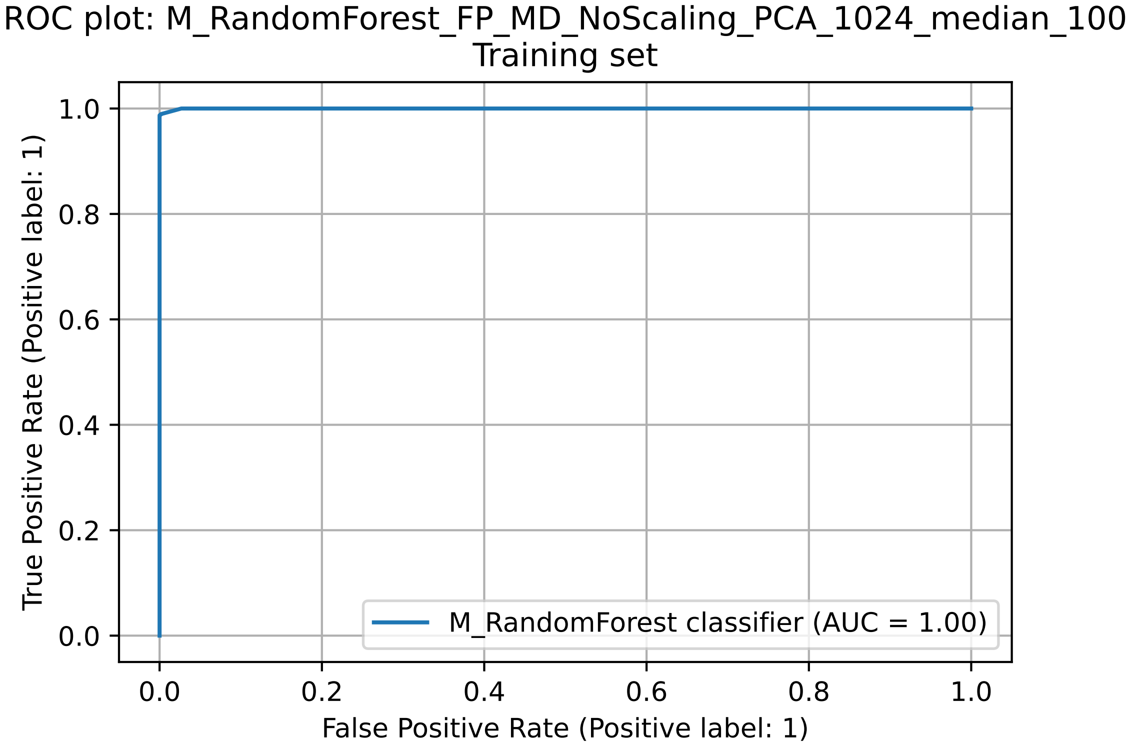
ROC plot: M_RandomForest_FP_MD_NoScaling_PCA_1024_median_100
Training set
True Positive Rate (Positive label: 1)
False Positive Rate (Positive label: 1)
0.0
0.2
0.4
0.6
0.8
1.0
M_RandomForest classifier (AUC = 1.00)

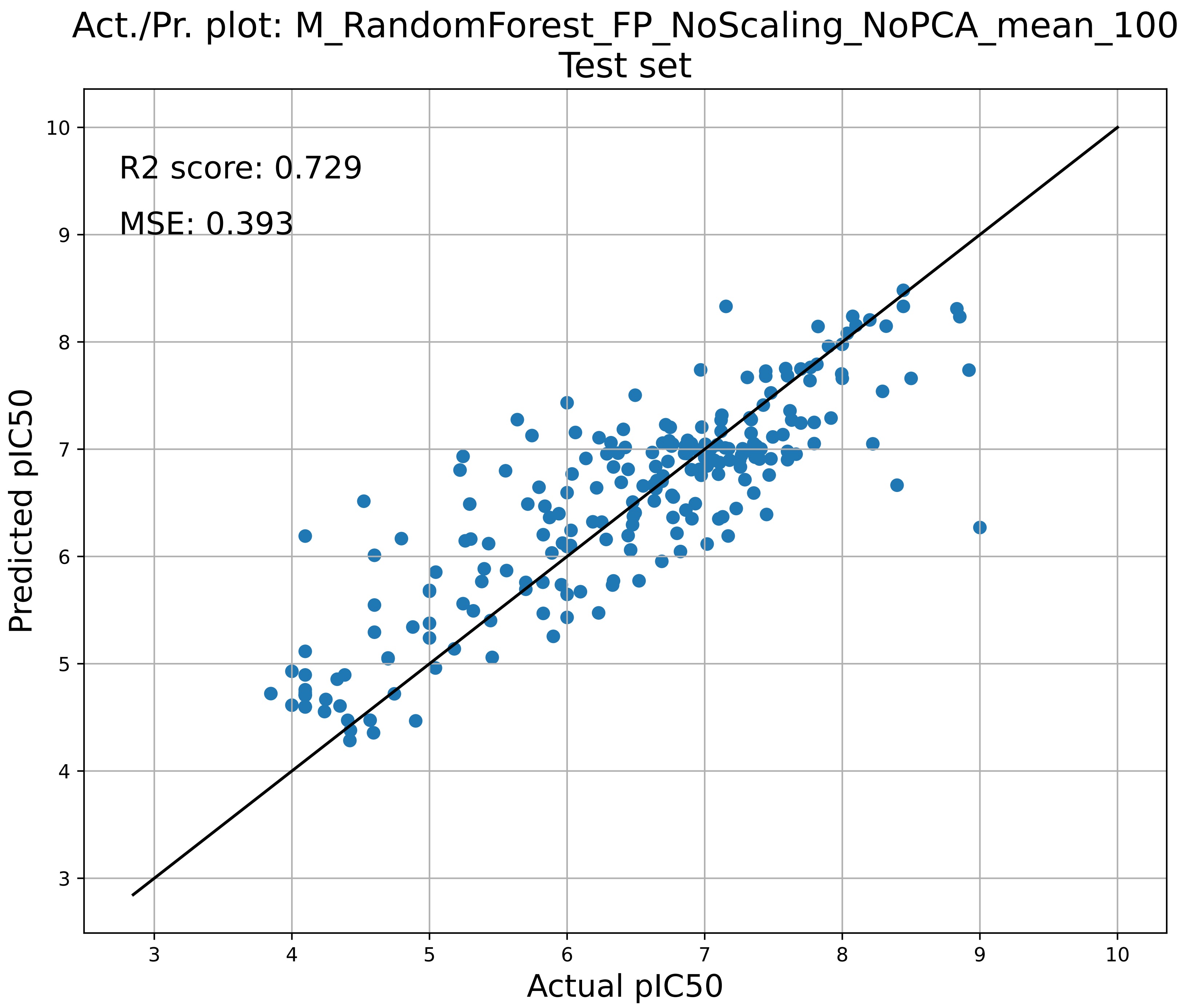

Act./Pr. plot: M_RandomForest_FP_NoScaling_NoPCA_mean_100
Test set
R2 score: 0.729
MSE: 0.393
Predicted pIC50
Actual pIC50
3
4
5
6
7
8
9
10

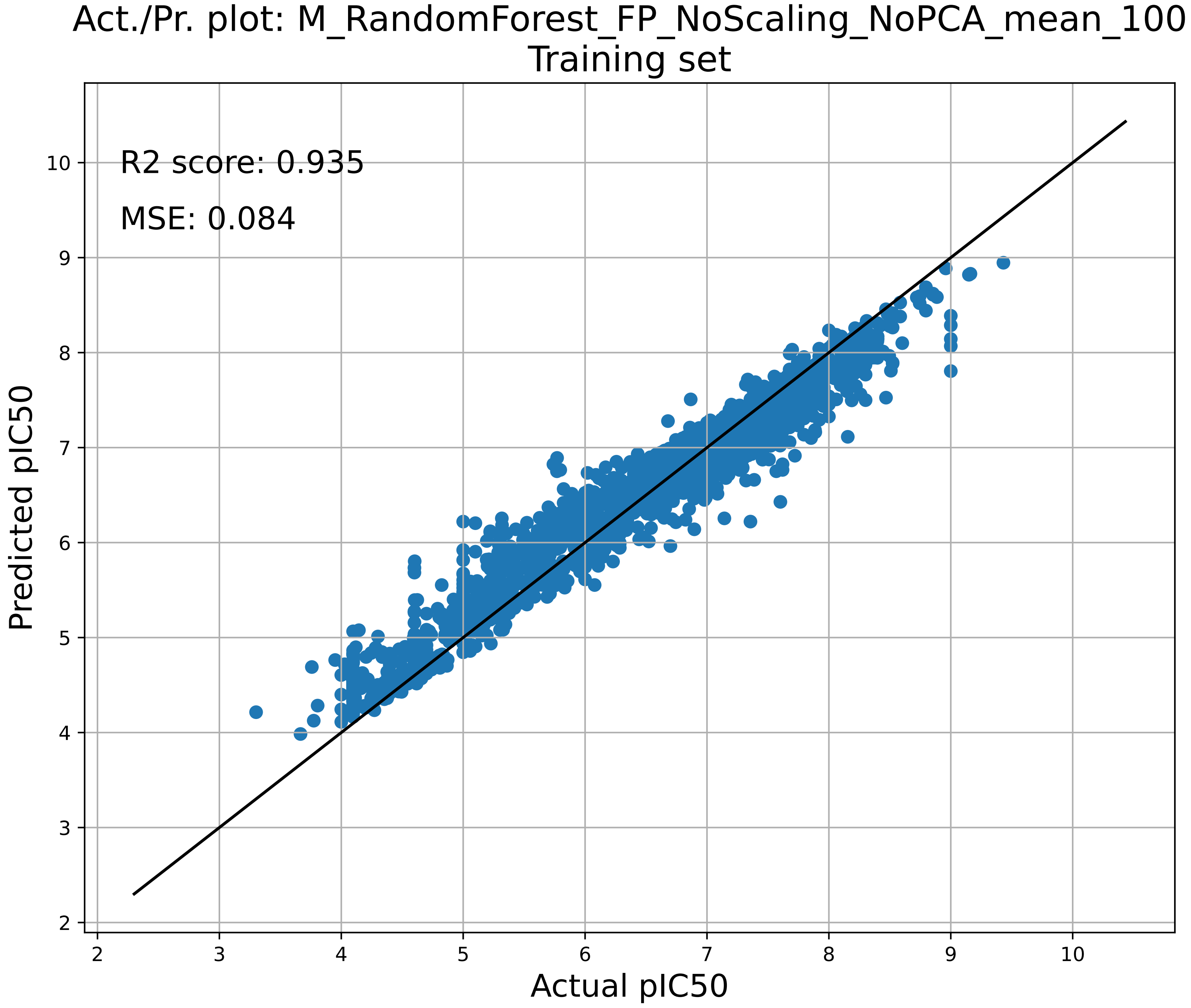

Act./Pr. plot: M_RandomForest_FP_NoScaling_NoPCA_mean_100
Training set
R2 score: 0.935
MSE: 0.084
Predicted pIC50
Actual pIC50
2
3
4
5
6
7
8
9
10

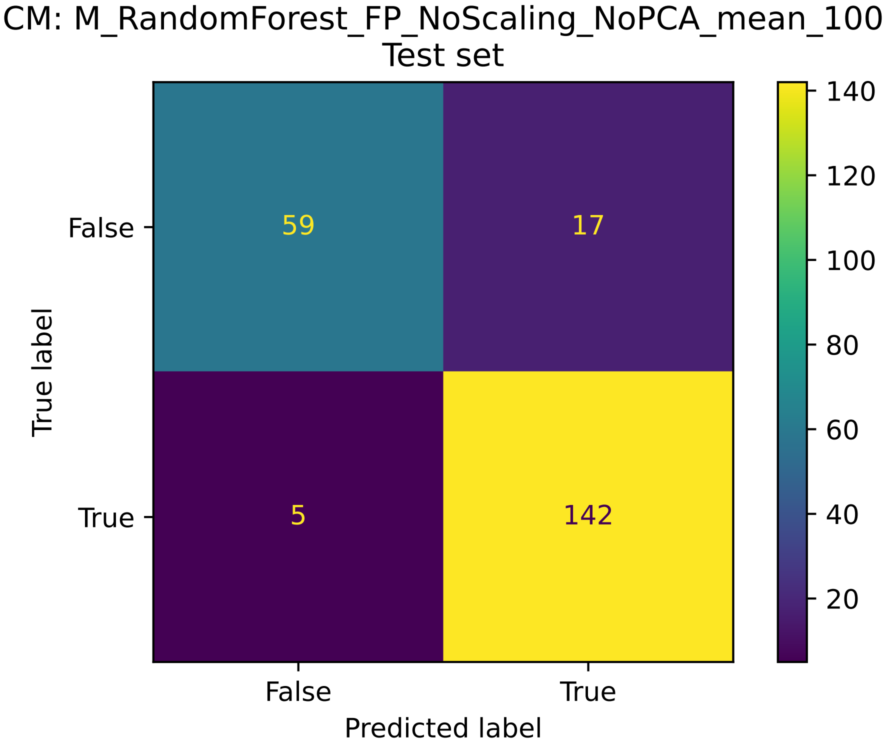
CM: M_RandomForest_FP_NoScaling_NoPCA_mean_100
Test set
59
17
5
142
False
True
True label
False
True
Predicted label
140
120
100
80
60
40
20

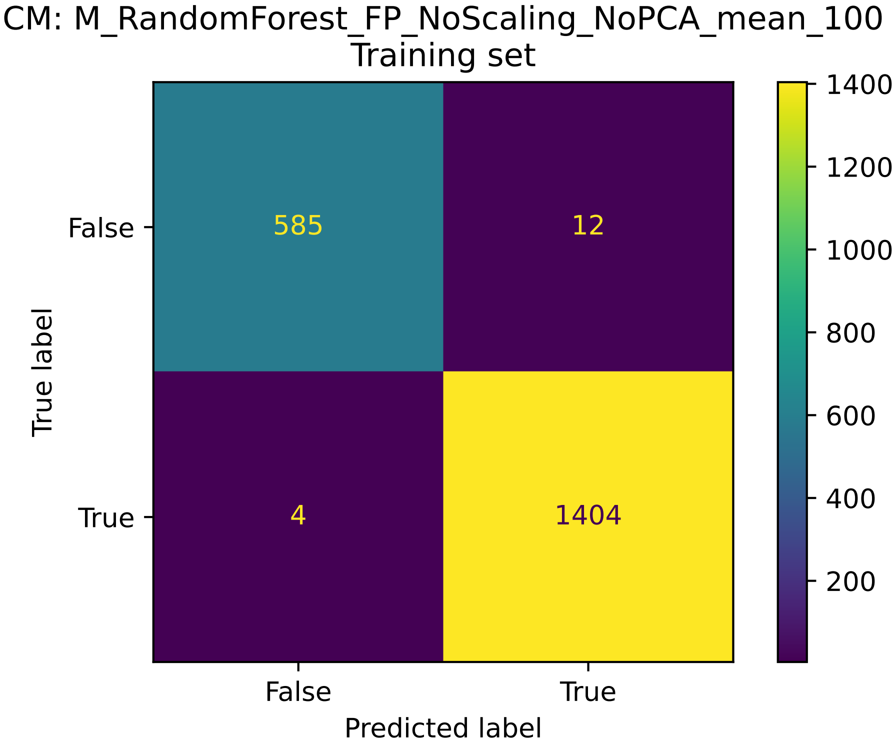

CM: M_RandomForest_FP_NoScaling_NoPCA_mean_100
Training set
585
12
4
1404
False
True
True label
False
True
Predicted label
1400
1200
1000
800
600
400
200

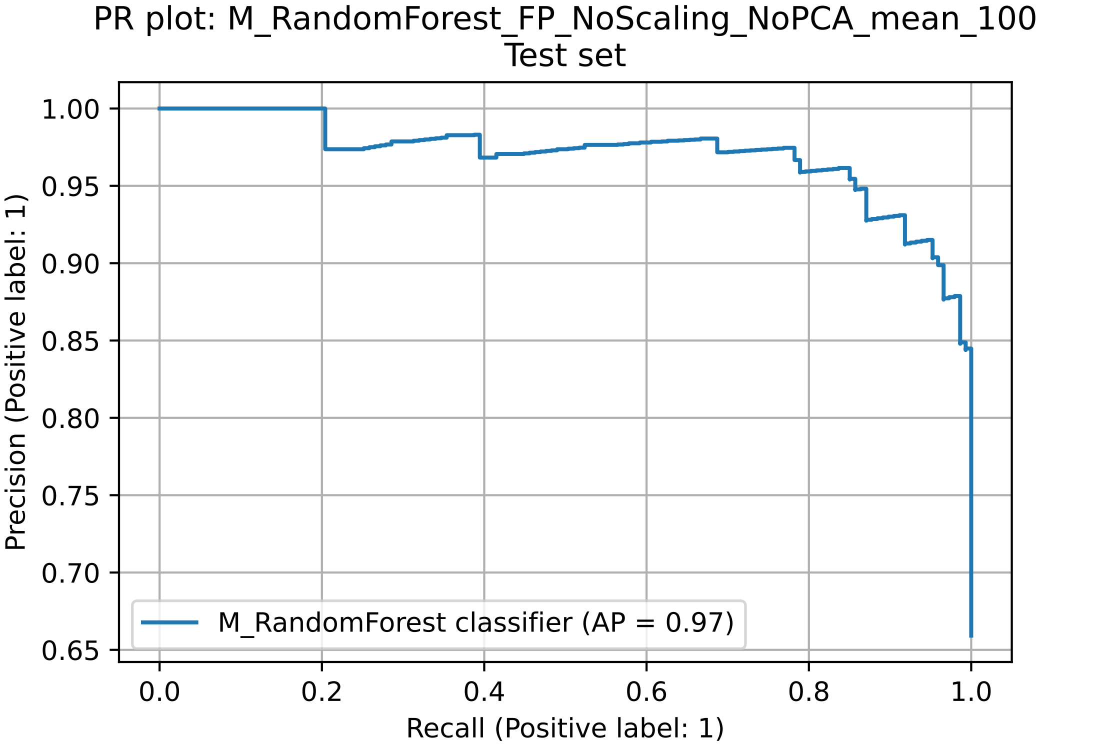
PR plot: M_RandomForest_FP_NoScaling_NoPCA_mean_100
Test set
Precision (Positive label: 1)
Recall (Positive label: 1)
1.00
0.95
0.90
0.85
0.80
0.75
0.70
0.65
0.0
0.2
0.4
0.6
0.8
1.0
M_RandomForest classifier (AP = 0.97)

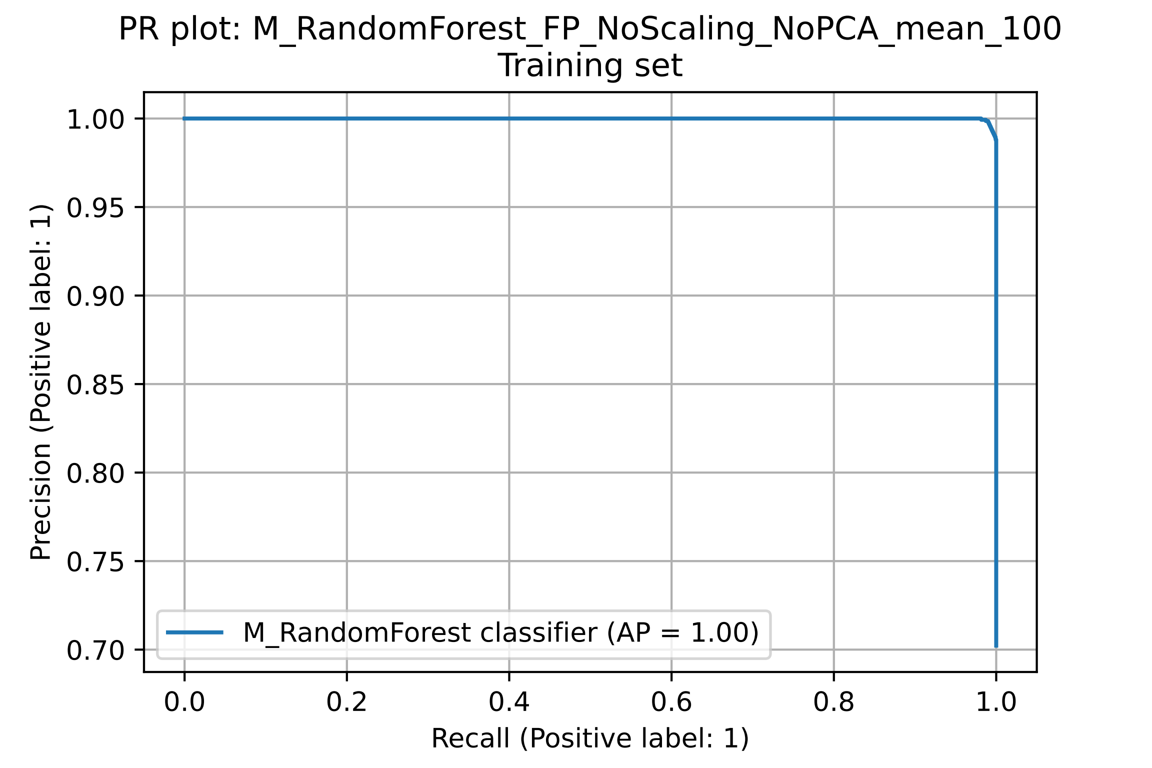
PR plot: M_RandomForest_FP_NoScaling_NoPCA_mean_100
Training set
Precision (Positive label: 1)
Recall (Positive label: 1)
M_RandomForest classifier (AP = 1.00)
1.00
0.95
0.90
0.85
0.80
0.75
0.70
0.0
0.2
0.4
0.6
0.8
1.0

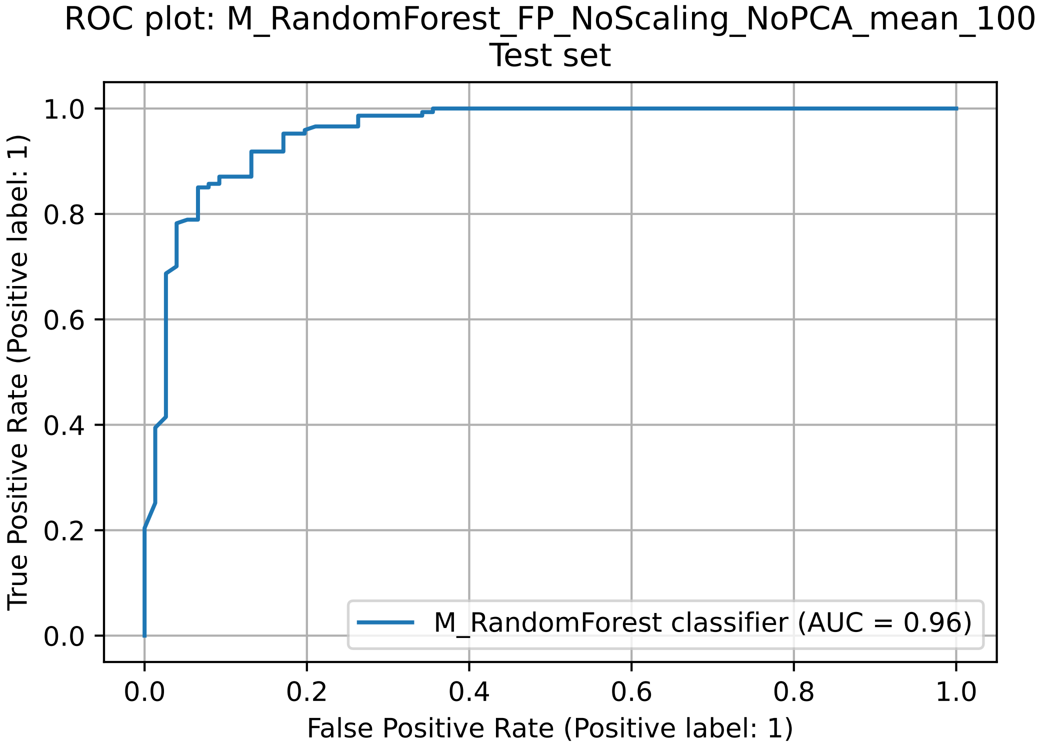
ROC plot: M_RandomForest_FP_NoScaling_NoPCA_mean_100
Test set
True Positive Rate (Positive label: 1)
False Positive Rate (Positive label: 1)
0.0
0.2
0.4
0.6
0.8
1.0
M_RandomForest classifier (AUC = 0.96)

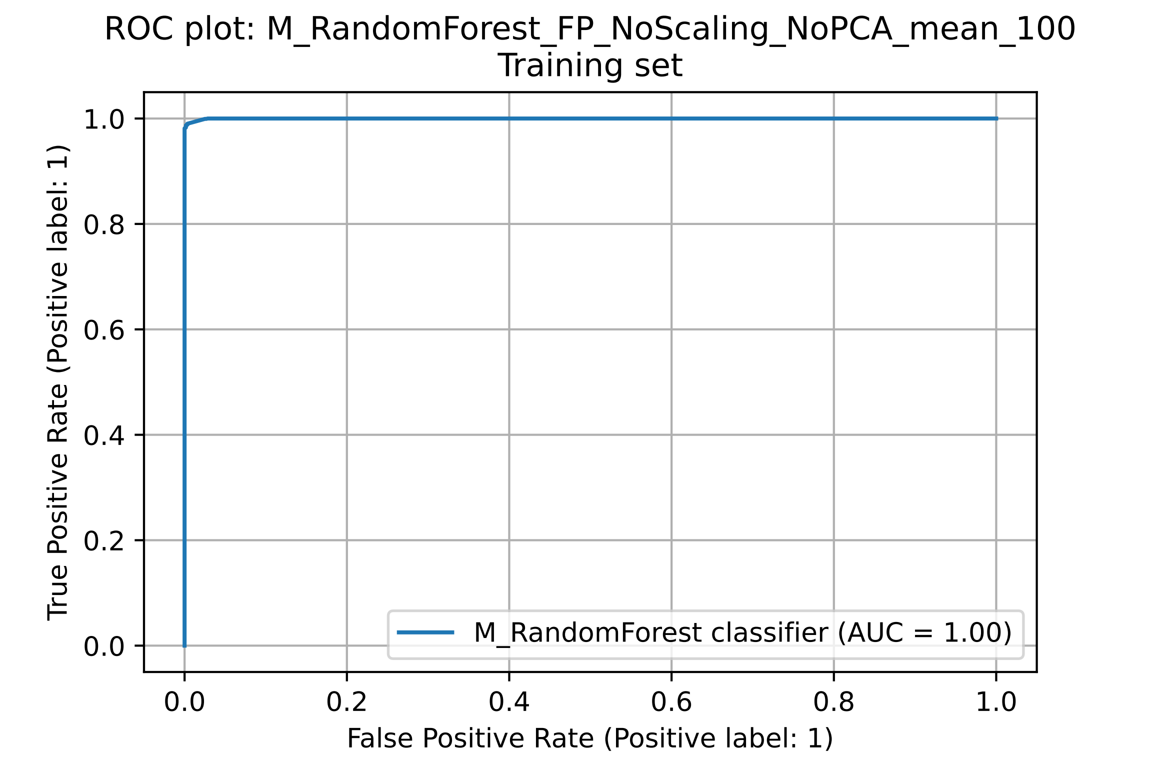
ROC plot: M_RandomForest_FP_NoScaling_NoPCA_mean_100
Training set
True Positive Rate (Positive label: 1)
False Positive Rate (Positive label: 1)
0.0
0.2
0.4
0.6
0.8
1.0
M_RandomForest classifier (AUC = 1.00)

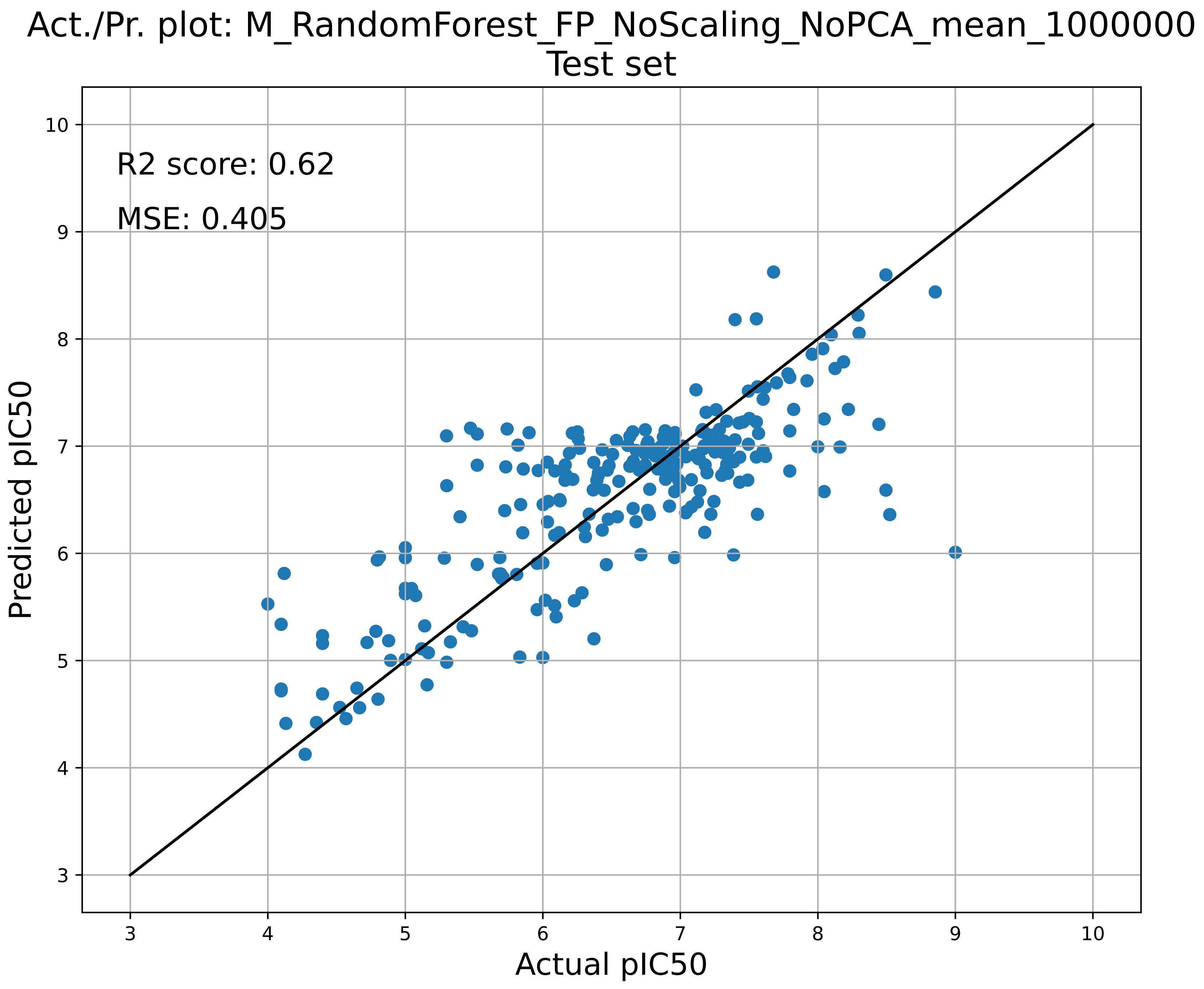

Act./Pr. plot: M_RandomForest_FP_NoScaling_NoPCA_mean_1000000
Test set
R2 score: 0.62
MSE: 0.405
Predicted pIC50
Actual pIC50
3
4
5
6
7
8
9
10

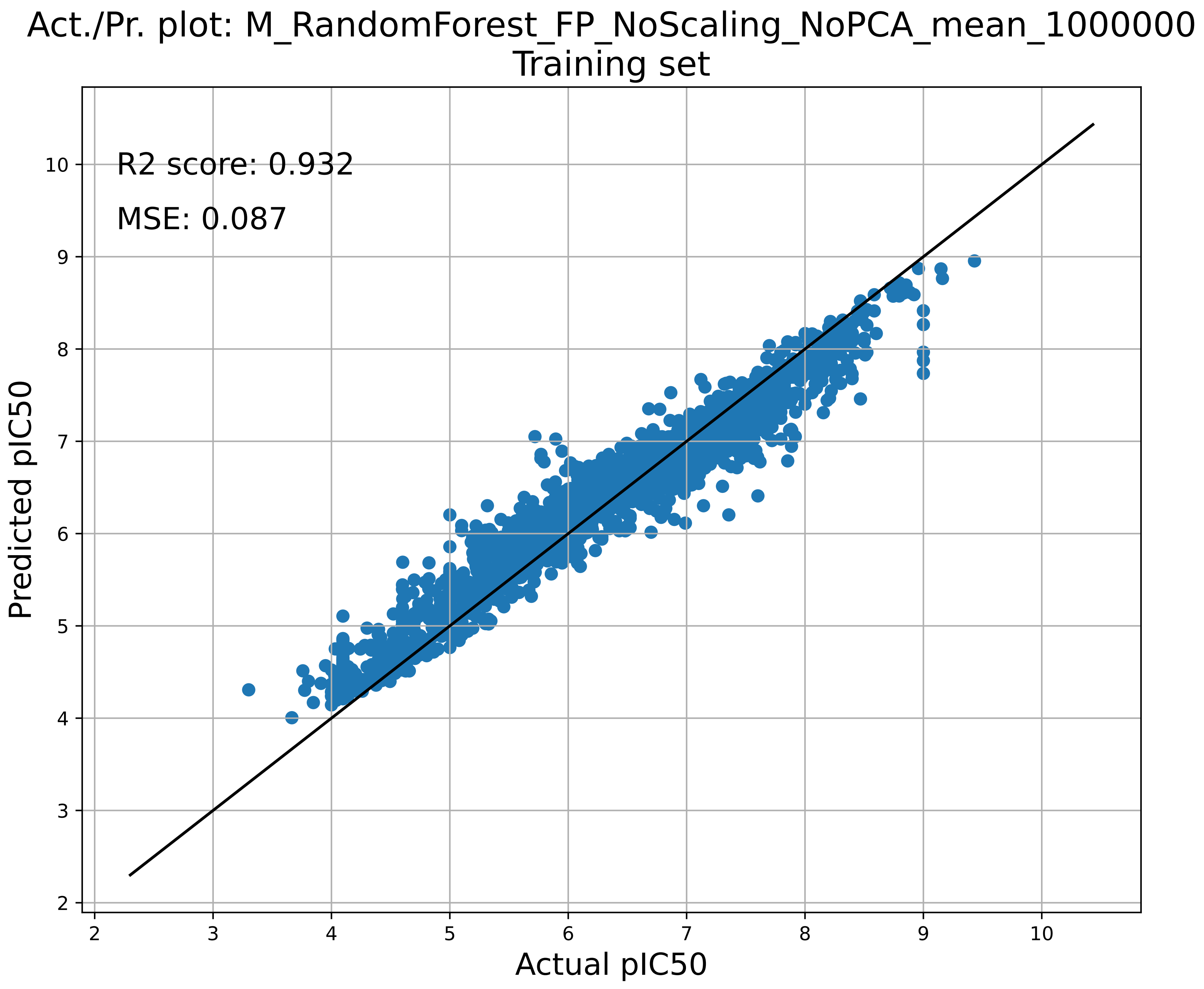

Act./Pr. plot: M_RandomForest_FP_NoScaling_NoPCA_mean_1000000
Training set
R2 score: 0.932
MSE: 0.087
Predicted pIC50
Actual pIC50
2
3
4
5
6
7
8
9
10

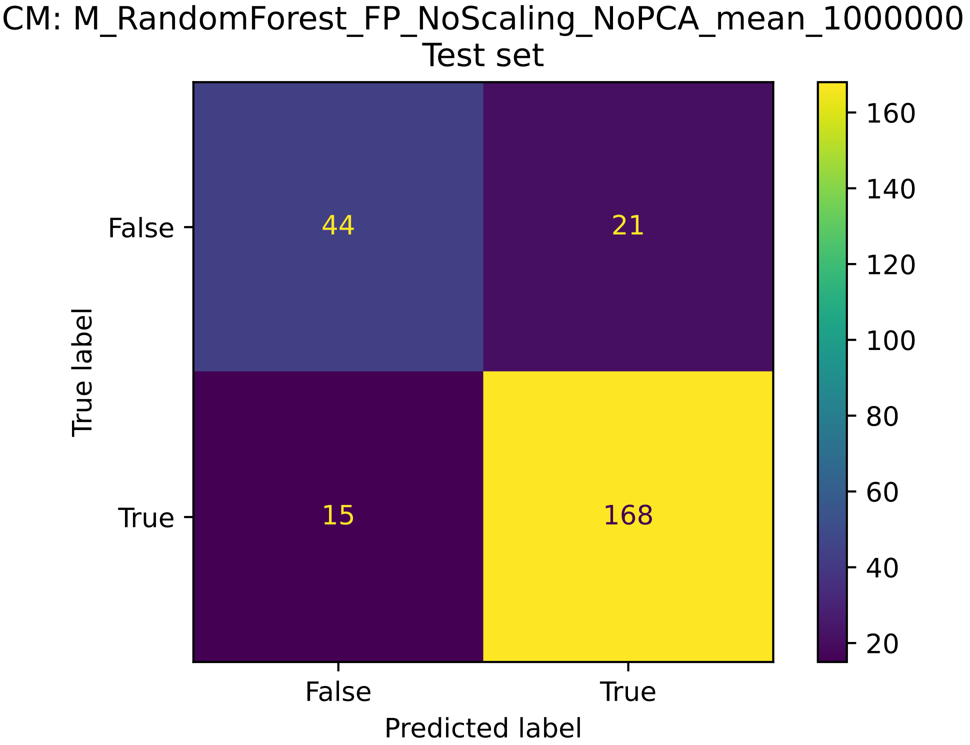
CM: M_RandomForest_FP_NoScaling_NoPCA_mean_1000000
Test set
True label
Predicted label
False
True
False
True
44
21
15
168
160
140
120
100
80
60
40
20

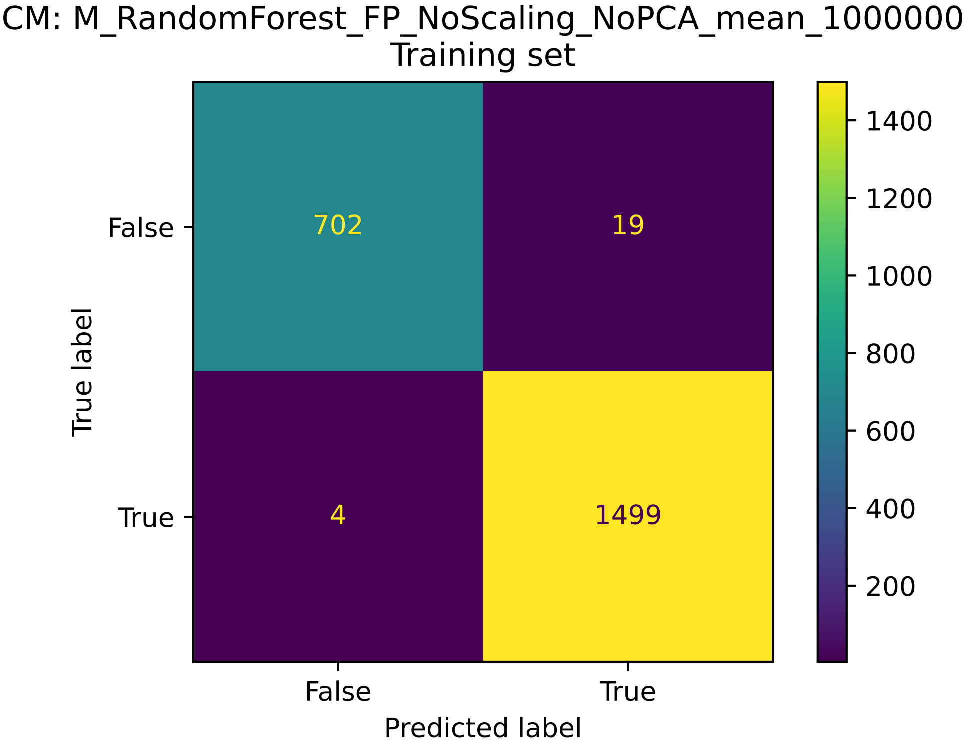
CM: M_RandomForest_FP_NoScaling_NoPCA_mean_1000000
Training set
True label
False
True
702
19
4
1499
Predicted label
200
400
600
800
1000
1200
1400

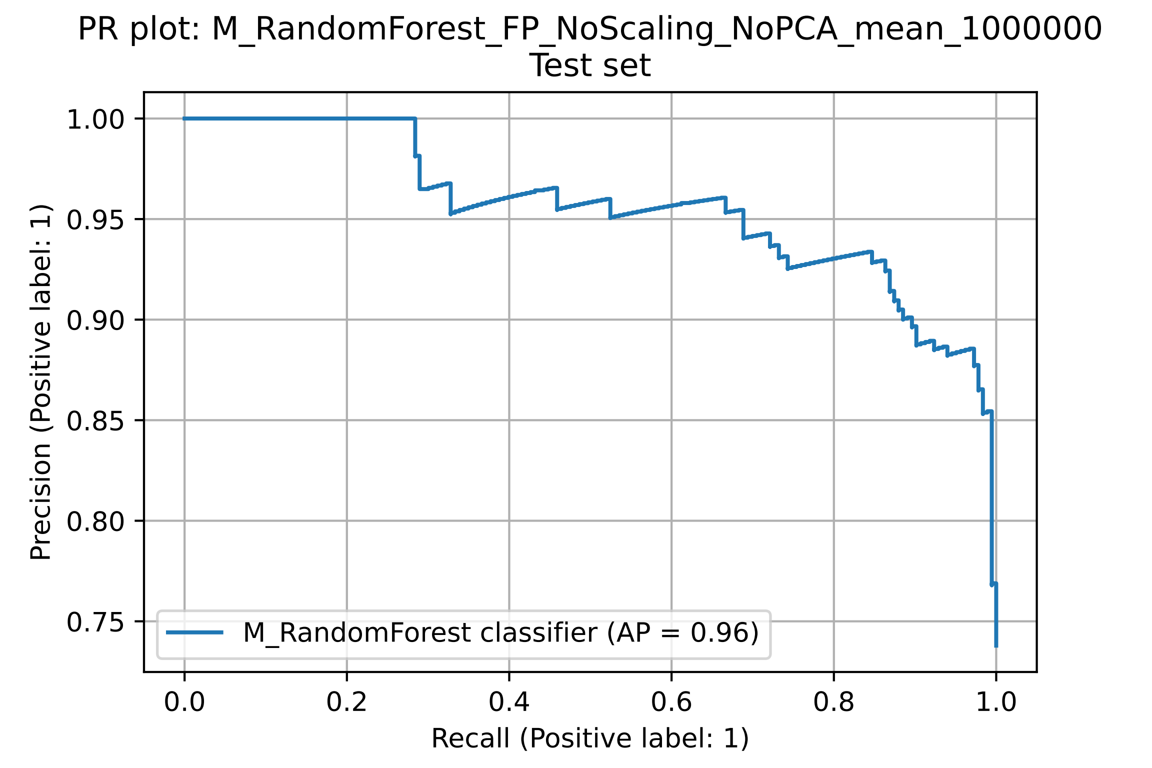
PR plot: M_RandomForest_FP_NoScaling_NoPCA_mean_1000000
Test set
Precision (Positive label: 1)
Recall (Positive label: 1)
1.00
0.95
0.90
0.85
0.80
0.75
0.0
0.2
0.4
0.6
0.8
1.0
M_RandomForest classifier (AP = 0.96)

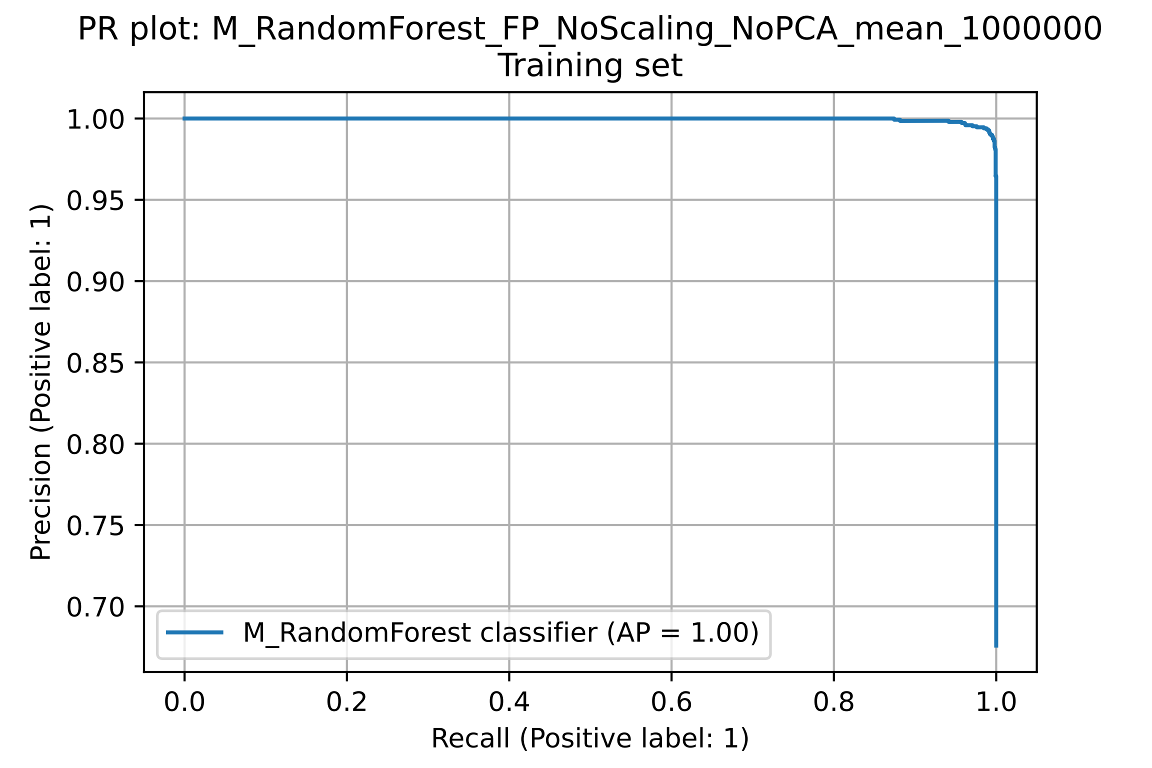

PR plot: M_RandomForest_FP_NoScaling_NoPCA_mean_1000000
Training set
Precision (Positive label: 1)
1.00
0.95
0.90
0.85
0.80
0.75
0.70
Recall (Positive label: 1)
0.0
0.2
0.4
0.6
0.8
1.0
M_RandomForest classifier (AP = 1.00)

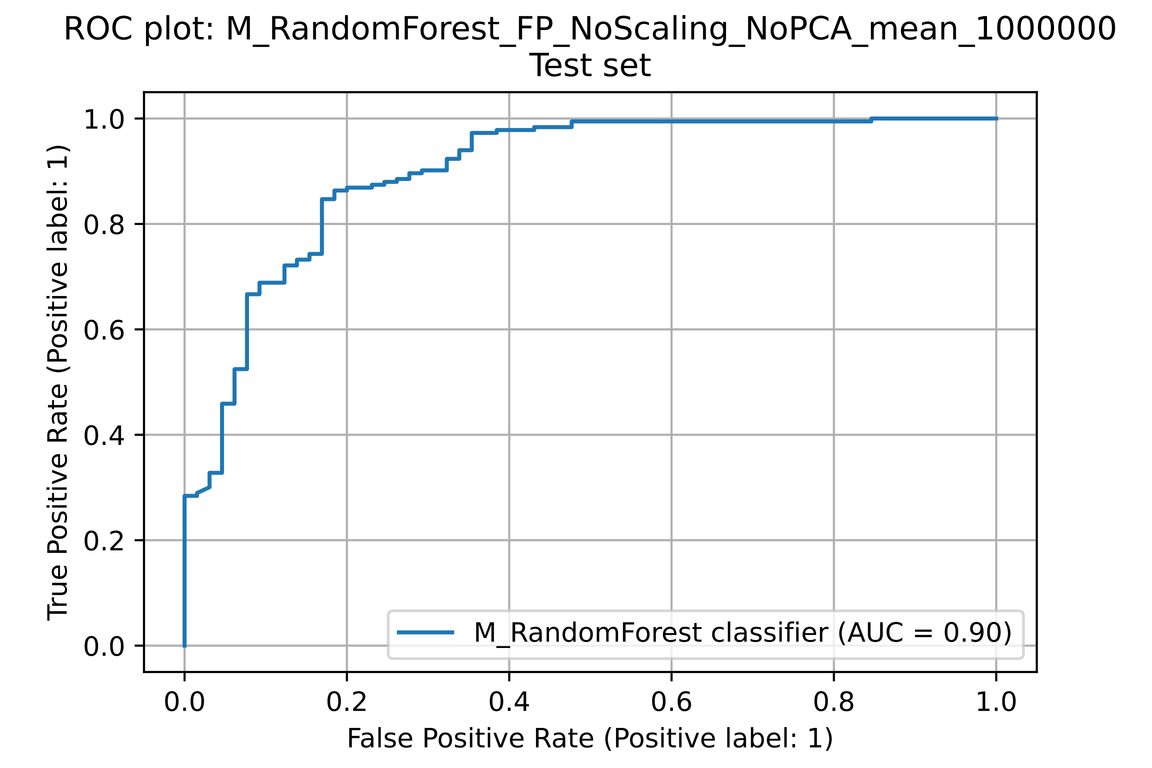
ROC plot: M_RandomForest_FP_NoScaling_NoPCA_mean_1000000
Test set
True Positive Rate (Positive label: 1)
False Positive Rate (Positive label: 1)
0.0
0.2
0.4
0.6
0.8
1.0
M_RandomForest classifier (AUC = 0.90)

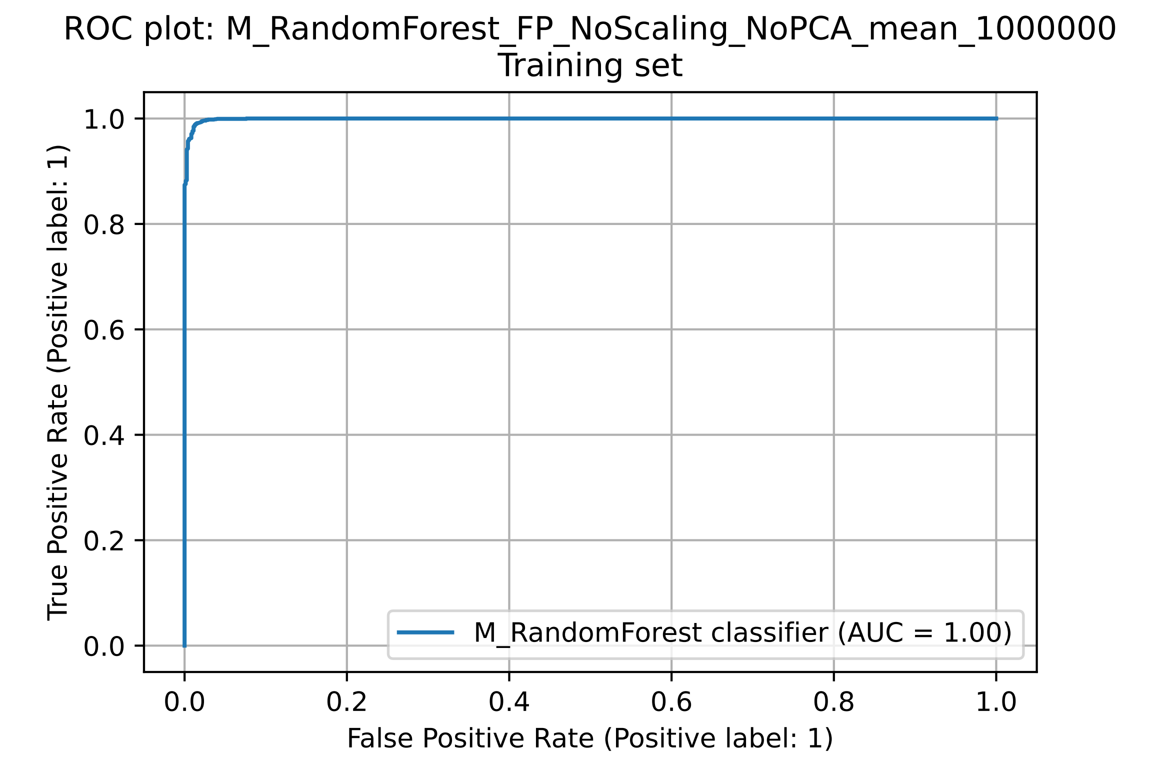

ROC plot: M_RandomForest_FP_NoScaling_NoPCA_mean_1000000
Training set
True Positive Rate (Positive label: 1)
False Positive Rate (Positive label: 1)
0.0
0.2
0.4
0.6
0.8
1.0
M_RandomForest classifier (AUC = 1.00)

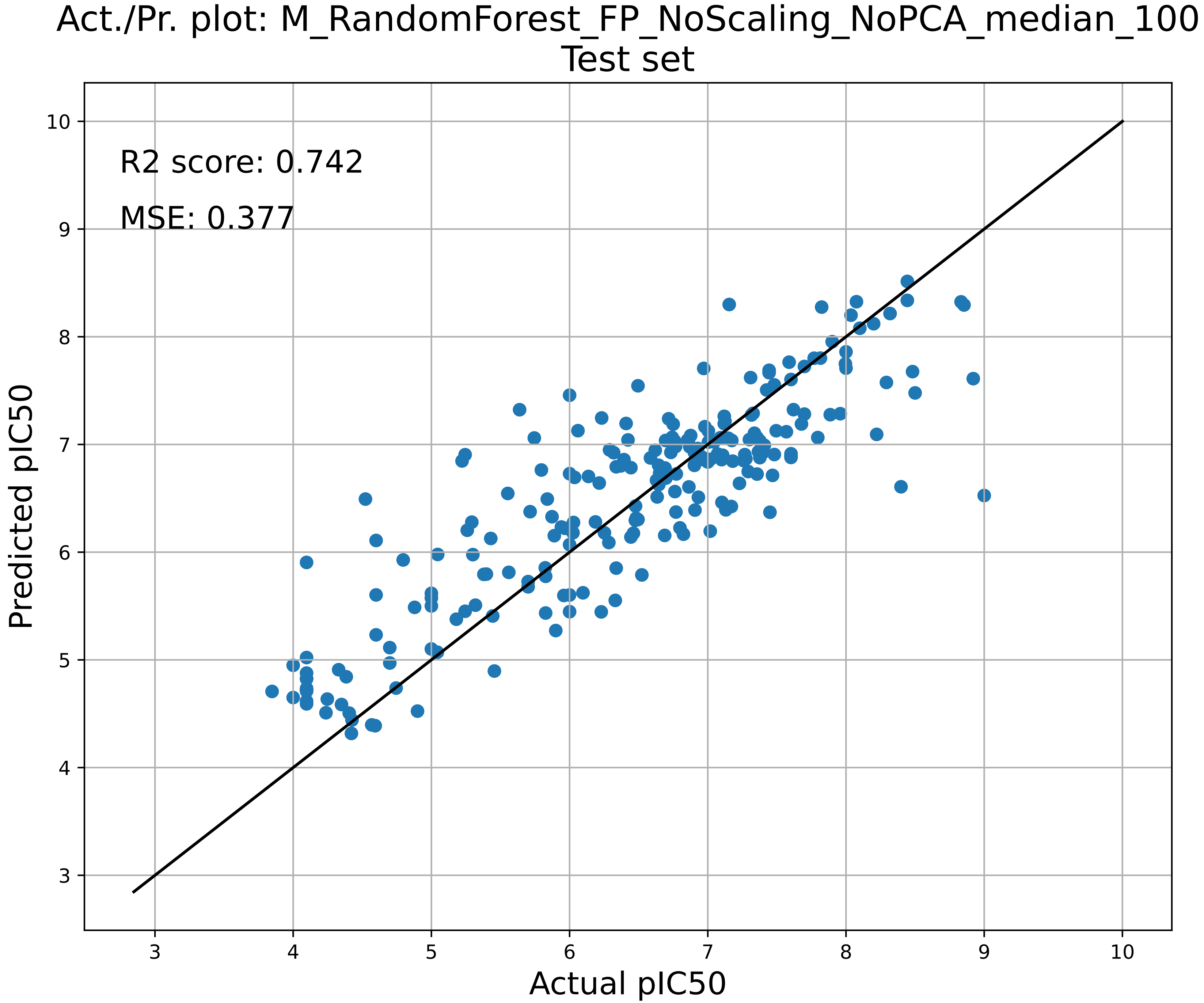

Act./Pr. plot: M_RandomForest_FP_NoScaling_NoPCA_median_100
Test set
R2 score: 0.742
MSE: 0.377
Predicted pIC50
Actual pIC50
3
4
5
6
7
8
9
10

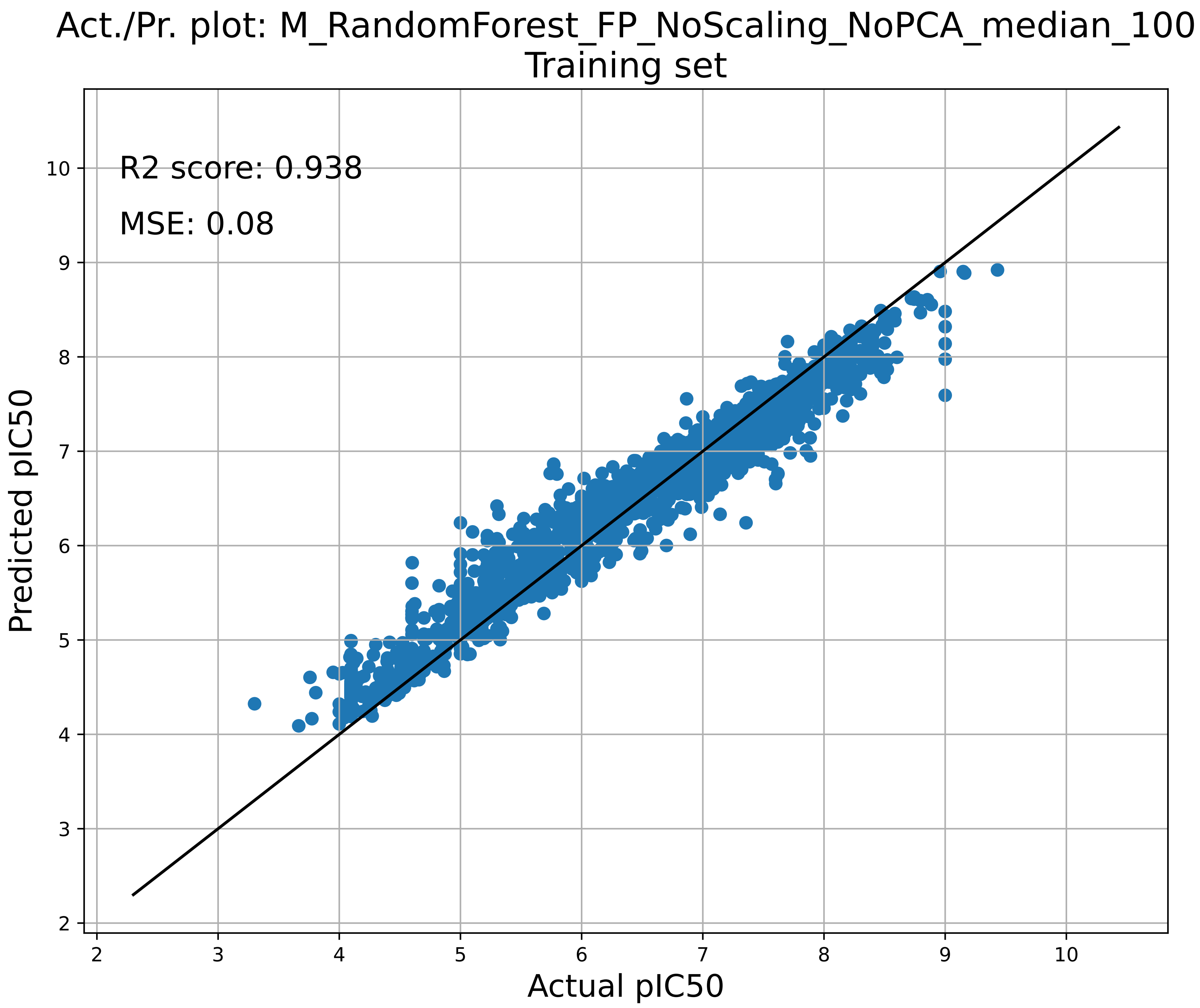
Act./Pr. plot: M_RandomForest_FP_NoScaling_NoPCA_median_100
Training set
R2 score: 0.938
MSE: 0.08
Predicted pIC50
Actual pIC50
2
3
4
5
6
7
8
9
10

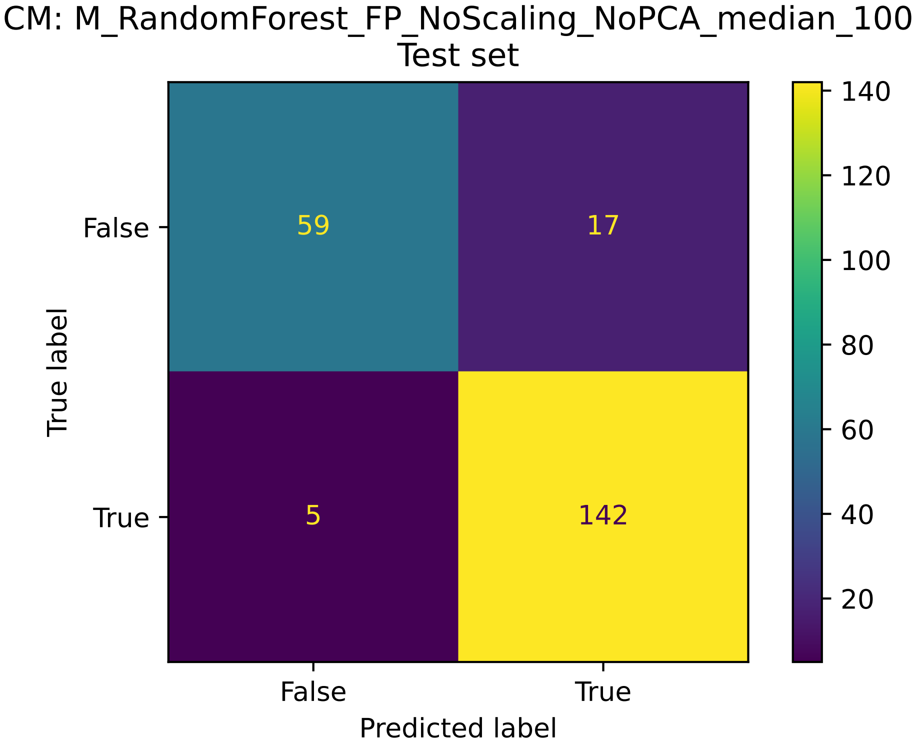
CM: M_RandomForest_FP_NoScaling_NoPCA_median_100
Test set
True label
False
True
Predicted label
False
True
59
17
5
142
20
40
60
80
100
120
140

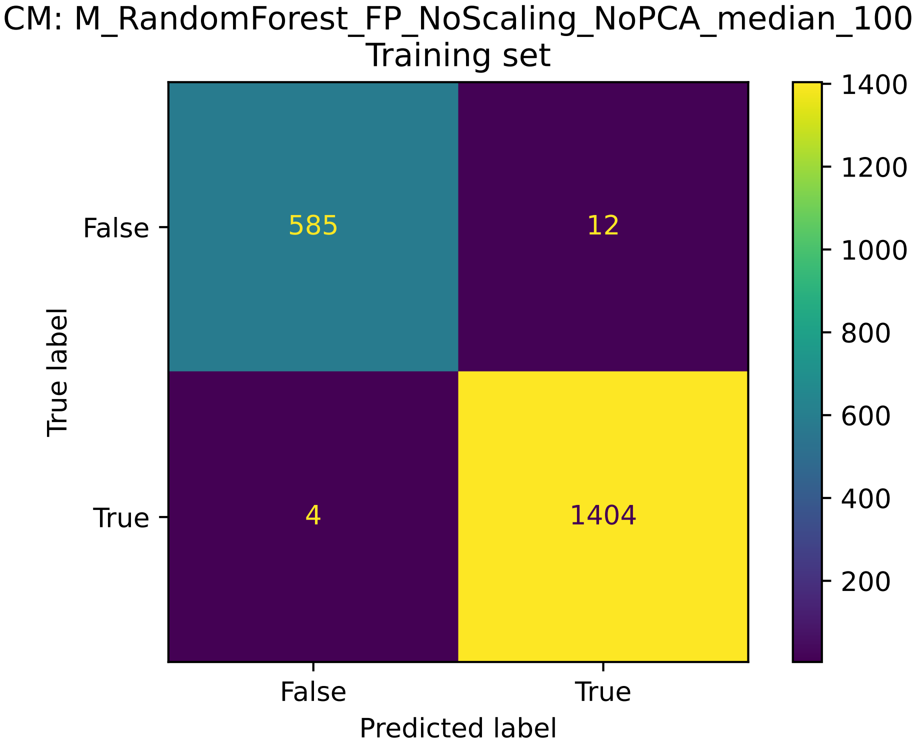
CM: M_RandomForest_FP_NoScaling_NoPCA_median_100
Training set
585
12
4
1404
False
True
True label
False
True
Predicted label
1400
1200
1000
800
600
400
200

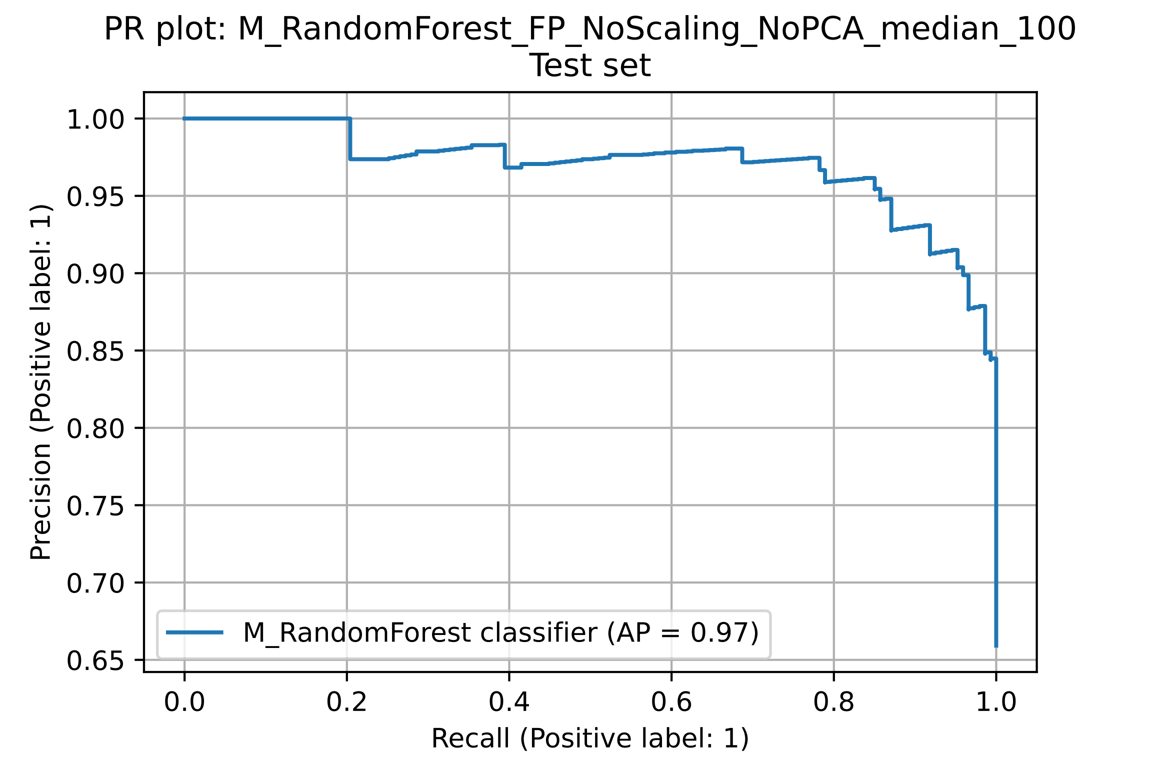
PR plot: M_RandomForest_FP_NoScaling_NoPCA_median_100
Test set
1.00
0.95
0.90
0.85
0.80
0.75
0.70
0.65
Precision (Positive label: 1)
0.0
0.2
0.4
0.6
0.8
1.0
Recall (Positive label: 1)
M_RandomForest classifier (AP = 0.97)

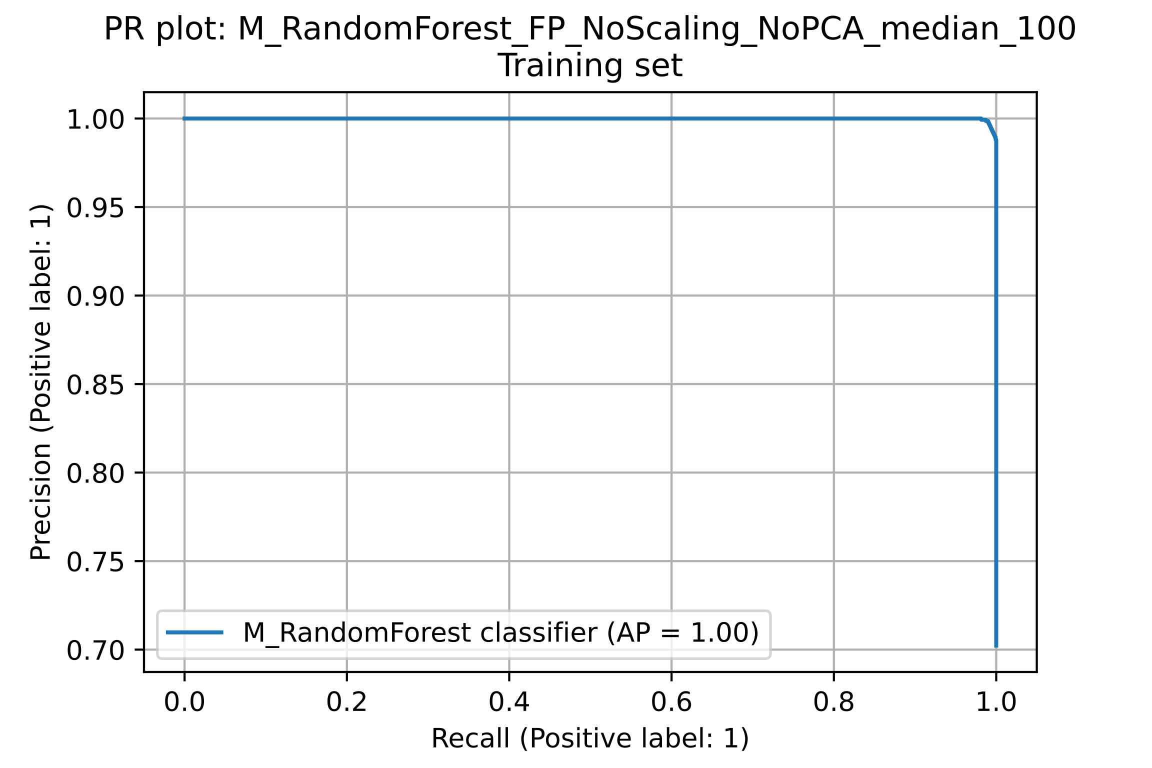
PR plot: M_RandomForest_FP_NoScaling_NoPCA_median_100
Training set
Precision (Positive label: 1)
1.00
0.95
0.90
0.85
0.80
0.75
0.70
0.0
0.2
0.4
0.6
0.8
1.0
Recall (Positive label: 1)
M_RandomForest classifier (AP = 1.00)

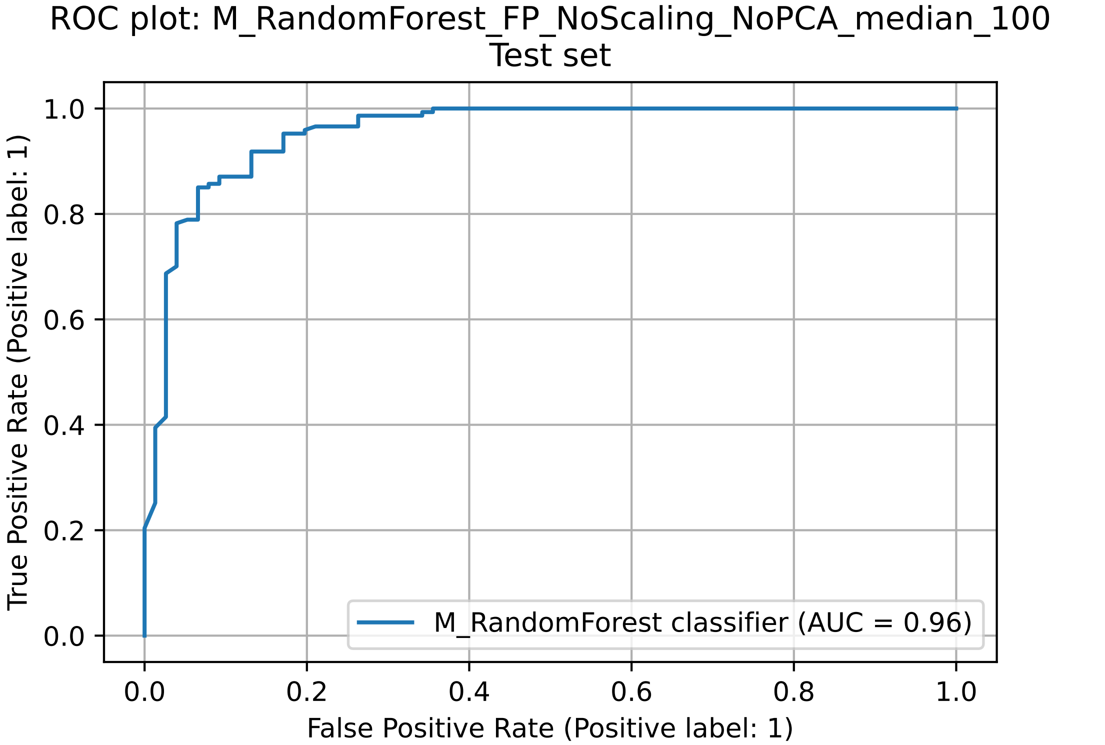
ROC plot: M_RandomForest_FP_NoScaling_NoPCA_median_100
Test set
True Positive Rate (Positive label: 1)
False Positive Rate (Positive label: 1)
0.0
0.2
0.4
0.6
0.8
1.0
M_RandomForest classifier (AUC = 0.96)

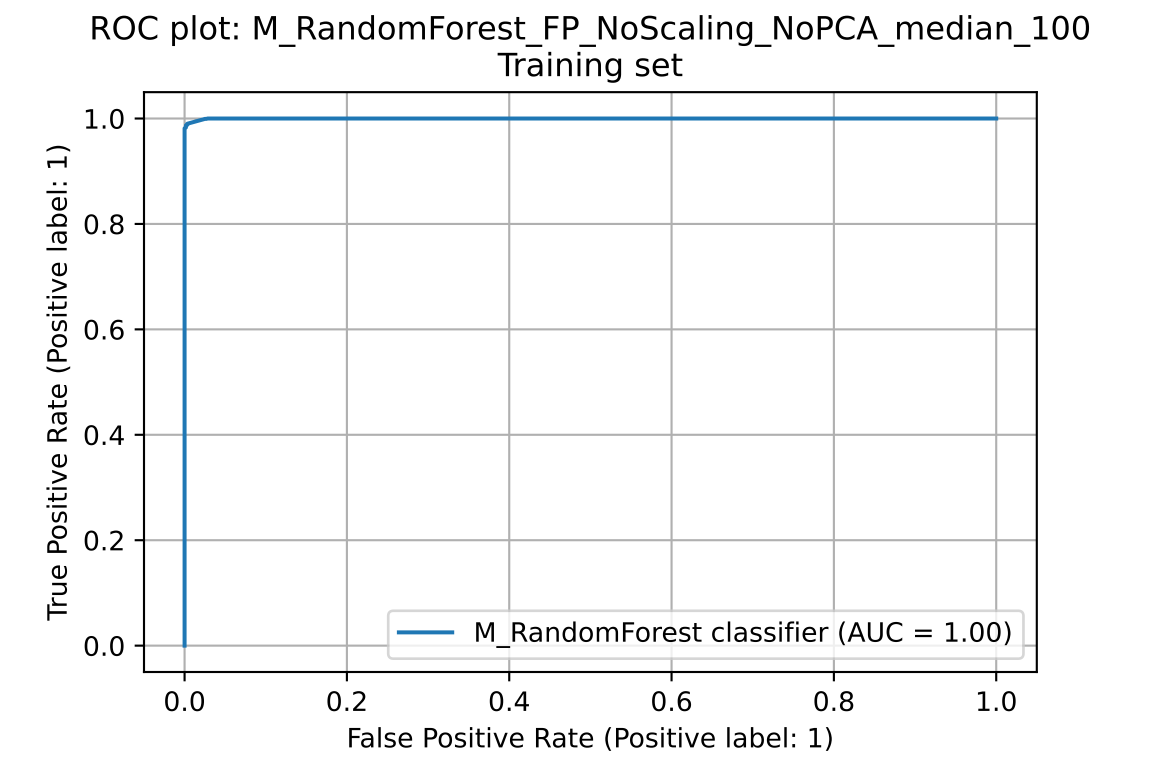
ROC plot: M_RandomForest_FP_NoScaling_NoPCA_median_100
Training set
True Positive Rate (Positive label: 1)
False Positive Rate (Positive label: 1)
0.0
0.2
0.4
0.6
0.8
1.0
M_RandomForest classifier (AUC = 1.00)

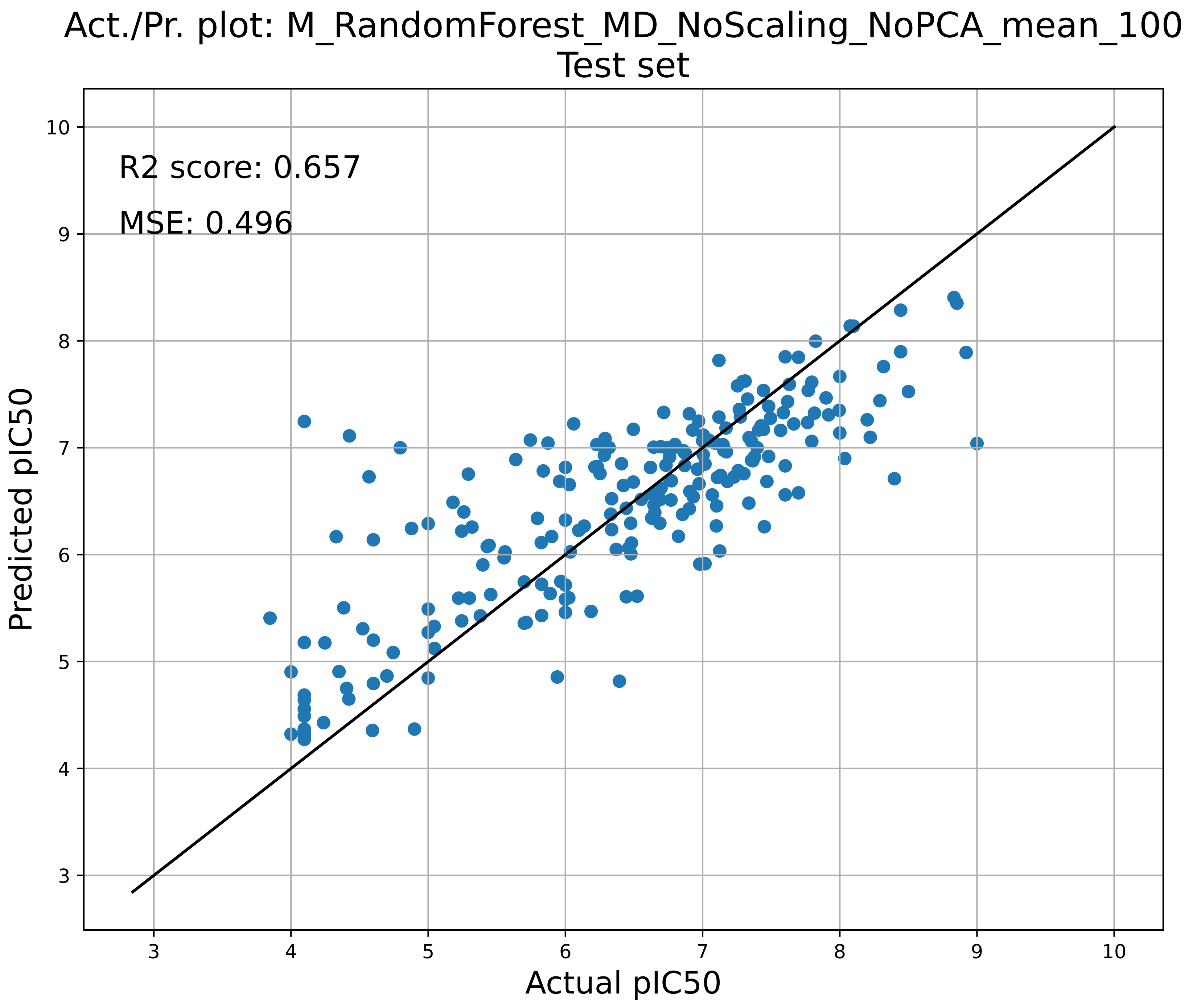

Act./Pr. plot: M_RandomForest_MD_NoScaling_NoPCA_mean_100
Test set
R2 score: 0.657
MSE: 0.496
Predicted pIC50
Actual pIC50
3
4
5
6
7
8
9
10

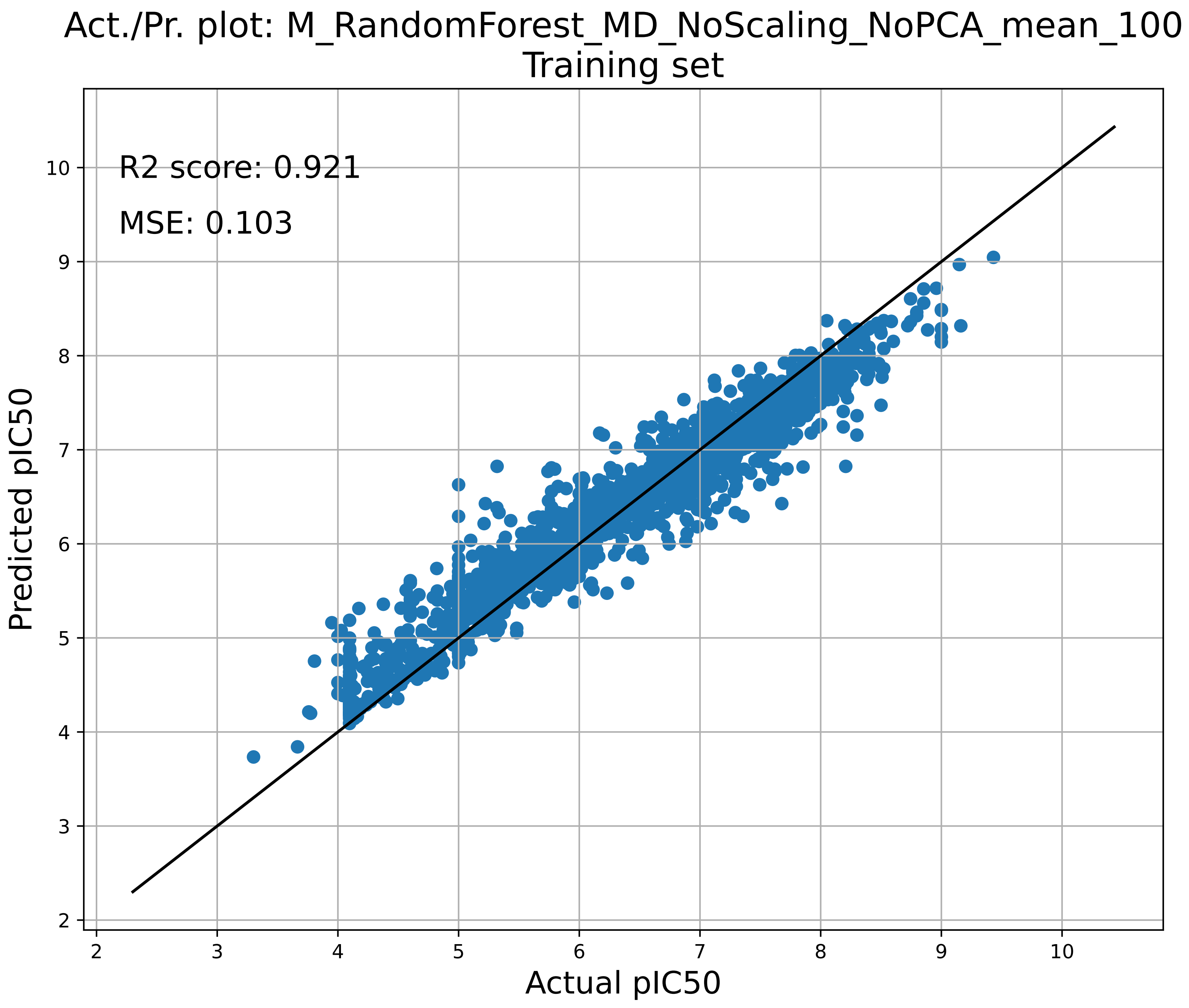
Act./Pr. plot: M_RandomForest_MD_NoScaling_NoPCA_mean_100
Training set
R2 score: 0.921
MSE: 0.103
Predicted pIC50
Actual pIC50
2
3
4
5
6
7
8
9
10

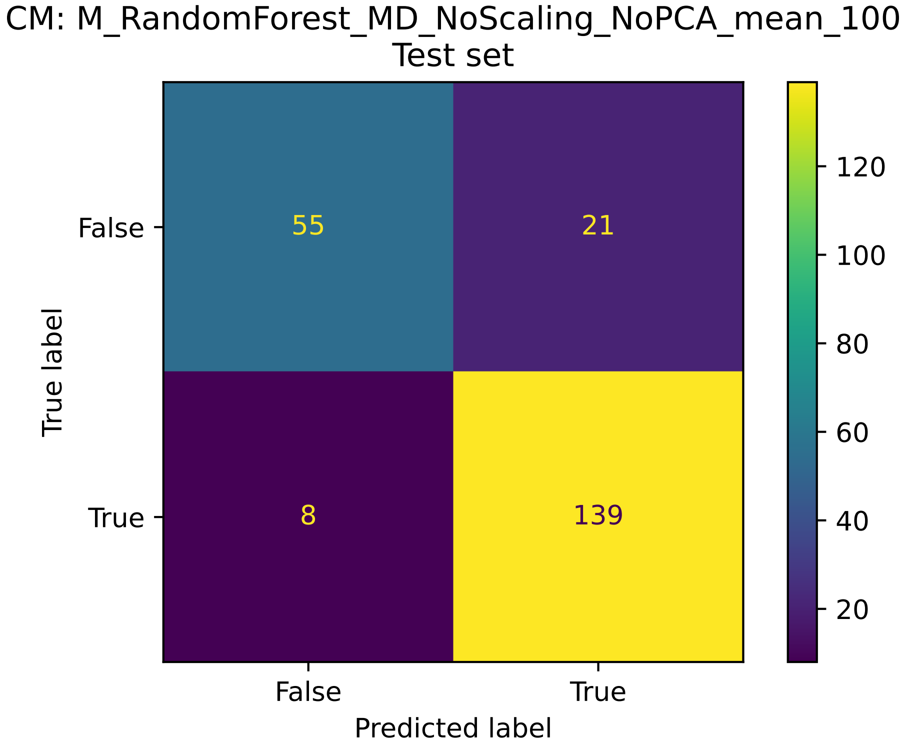
CM: M_RandomForest_MD_NoScaling_NoPCA_mean_100
Test set
True label
False
True
55
21
8
139
Predicted label
False
True
120
100
80
60
40
20

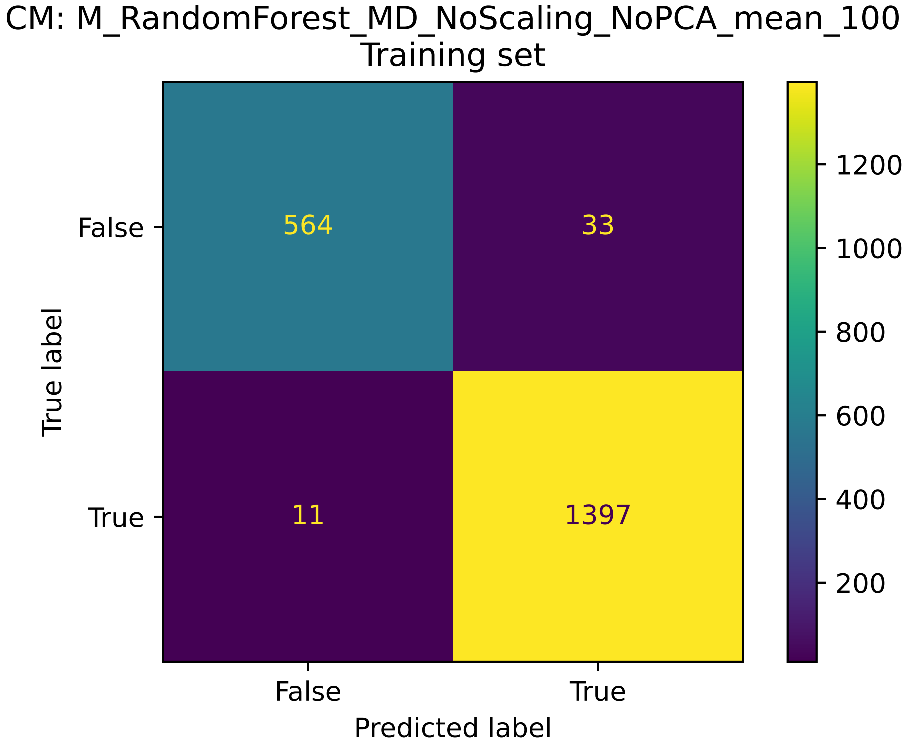

CM: M_RandomForest_MD_NoScaling_NoPCA_mean_100
Training set
True label
False
True
564
33
11
1397
Predicted label
1200
1000
800
600
400
200

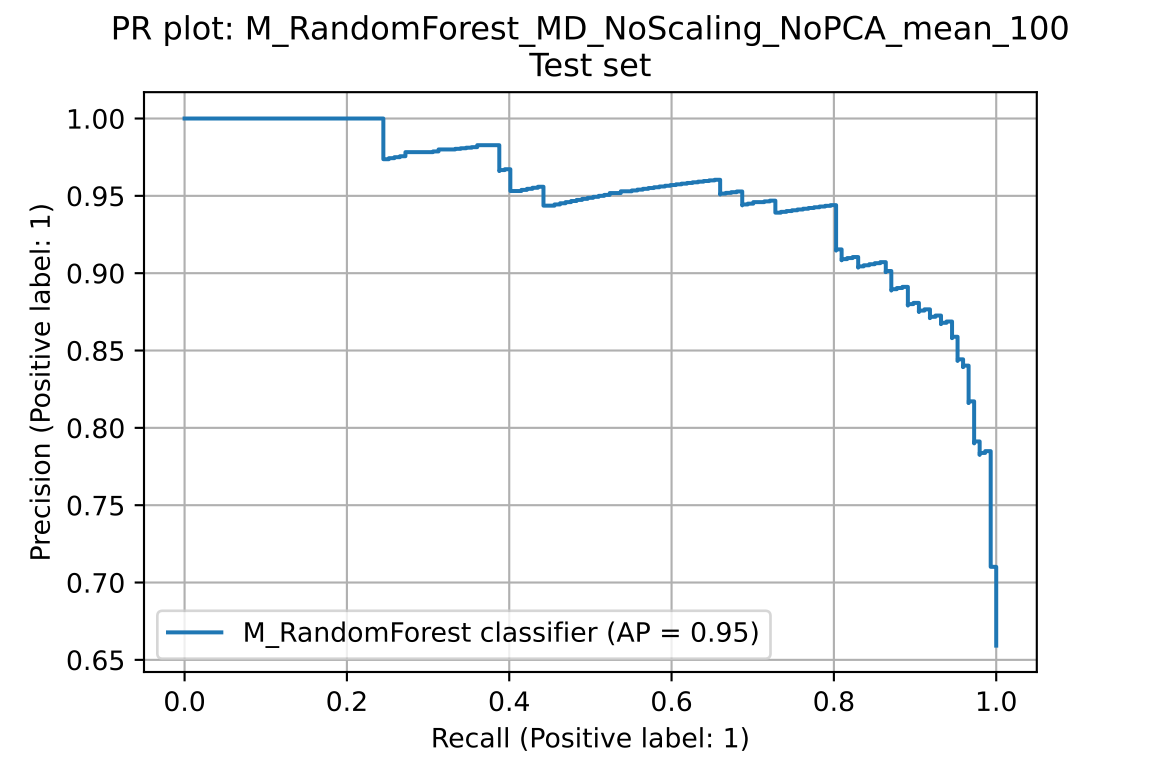
PR plot: M_RandomForest_MD_NoScaling_NoPCA_mean_100
Test set
Precision (Positive label: 1)
Recall (Positive label: 1)
1.00
0.95
0.90
0.85
0.80
0.75
0.70
0.65
0.0
0.2
0.4
0.6
0.8
1.0
M_RandomForest classifier (AP = 0.95)

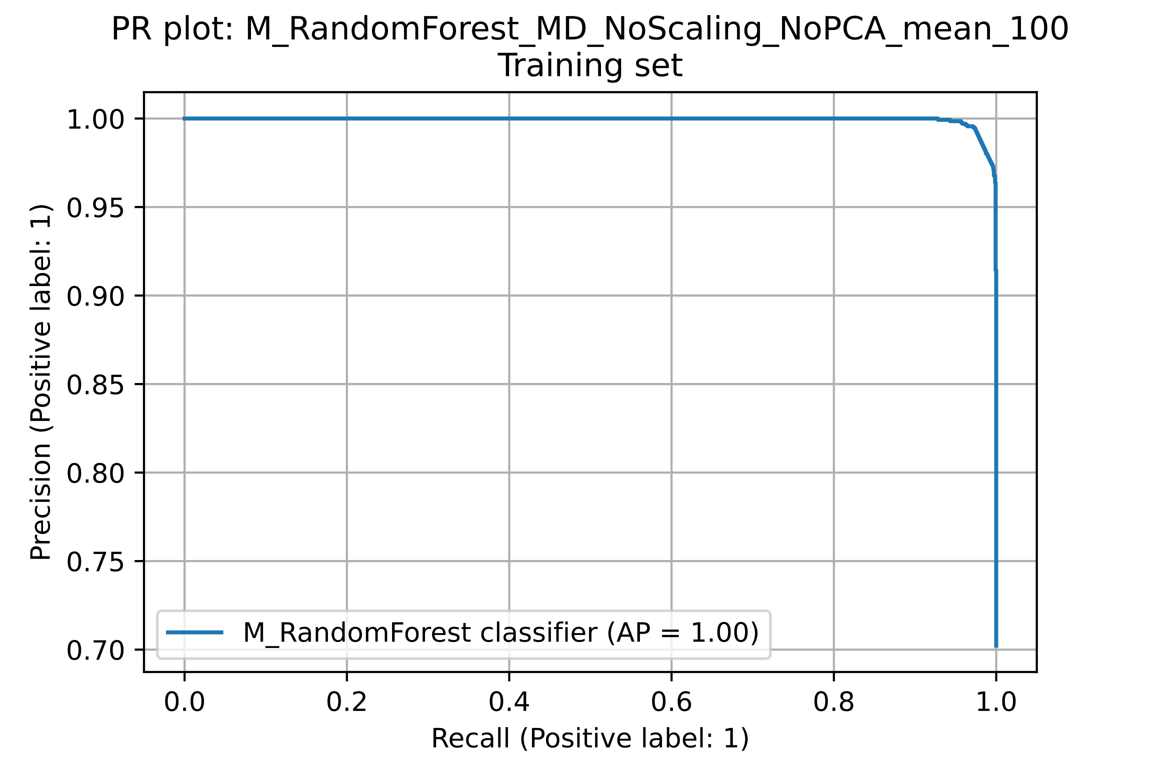

PR plot: M_RandomForest_MD_NoScaling_NoPCA_mean_100
Training set
1.00
0.95
0.90
0.85
0.80
0.75
0.70
Precision (Positive label: 1)
0.0
0.2
0.4
0.6
0.8
1.0
Recall (Positive label: 1)
M_RandomForest classifier (AP = 1.00)

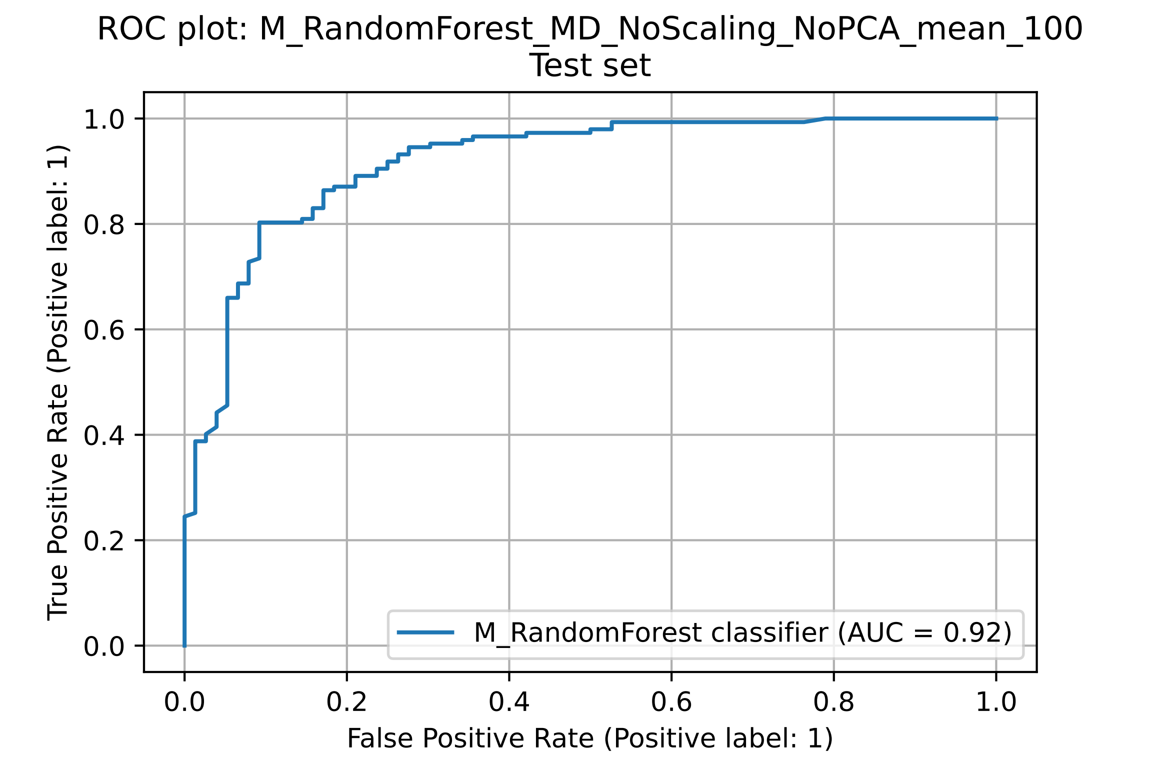
ROC plot: M_RandomForest_MD_NoScaling_NoPCA_mean_100
Test set
True Positive Rate (Positive label: 1)
False Positive Rate (Positive label: 1)
0.0
0.2
0.4
0.6
0.8
1.0
M_RandomForest classifier (AUC = 0.92)

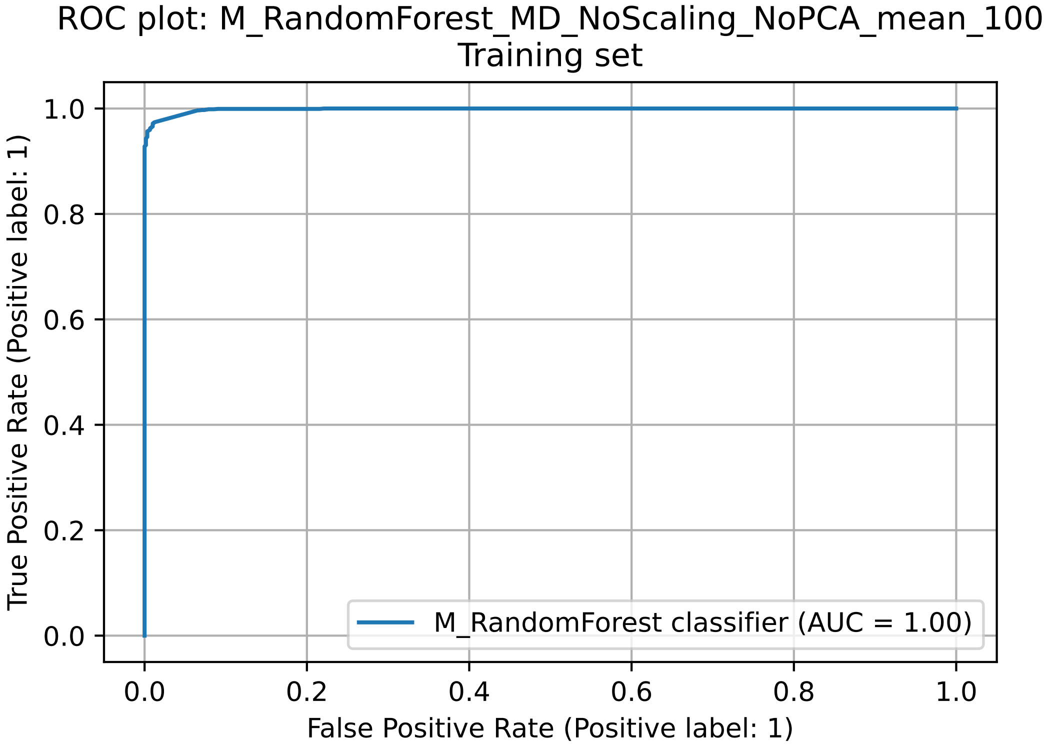
ROC plot: M_RandomForest_MD_NoScaling_NoPCA_mean_100
Training set
True Positive Rate (Positive label: 1)
False Positive Rate (Positive label: 1)
0.0
0.2
0.4
0.6
0.8
1.0
M_RandomForest classifier (AUC = 1.00)

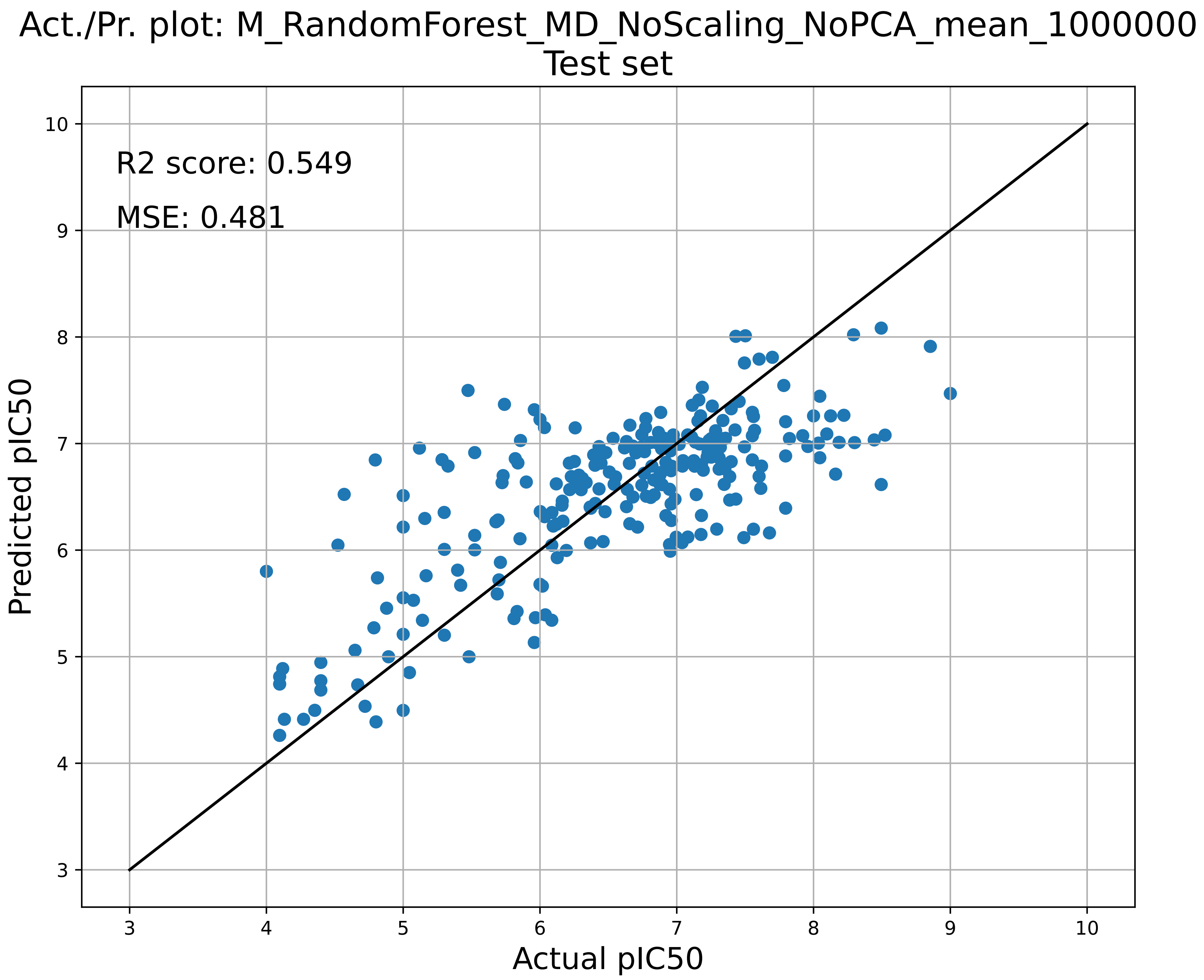
Act./Pr. plot: M_RandomForest_MD_NoScaling_NoPCA_mean_1000000
Test set
R2 score: 0.549
MSE: 0.481
Predicted pIC50
Actual pIC50
3
4
5
6
7
8
9
10

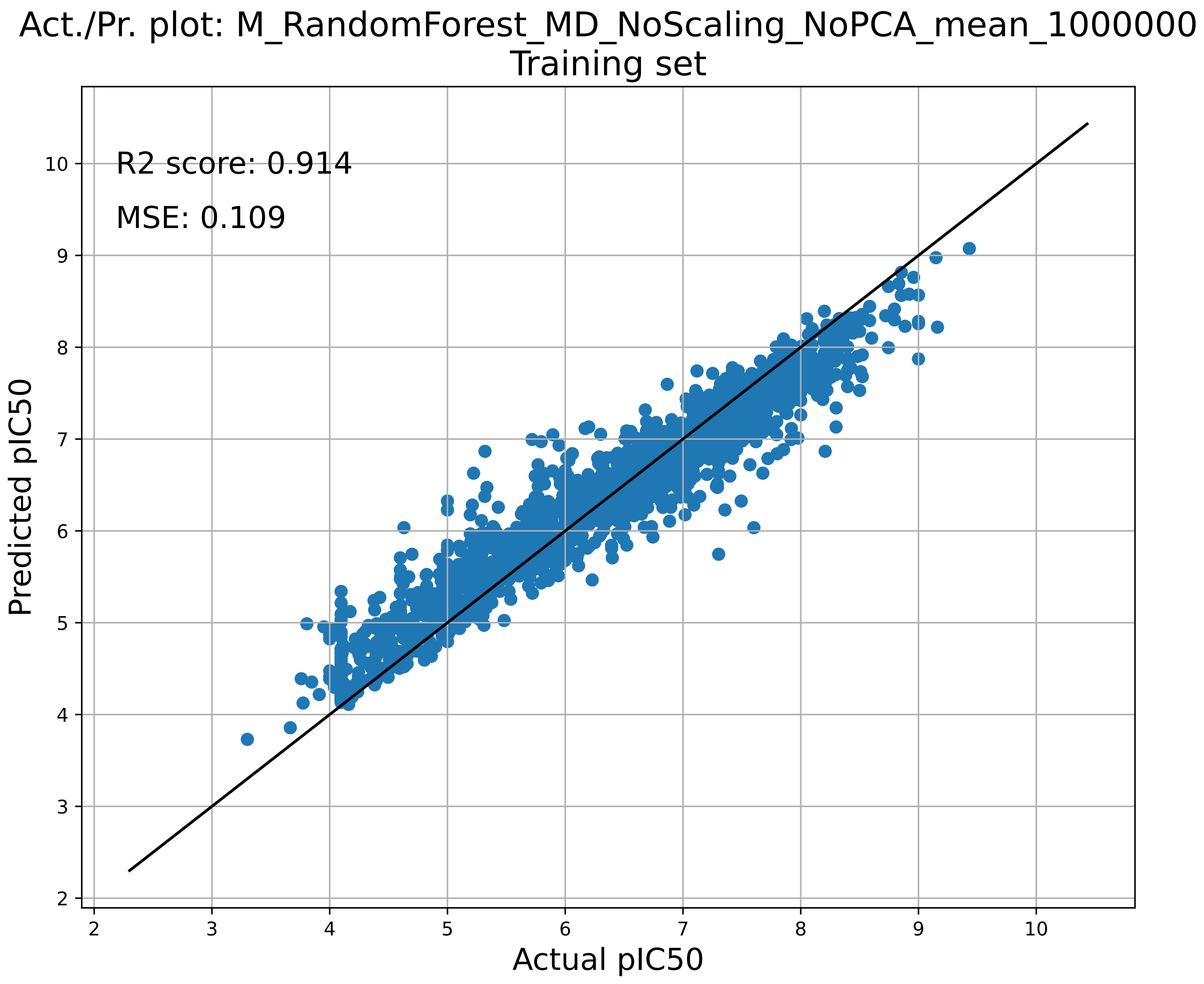

Act./Pr. plot: M_RandomForest_MD_NoScaling_NoPCA_mean_1000000
Training set
R2 score: 0.914
MSE: 0.109
Predicted pIC50
Actual pIC50
2
3
4
5
6
7
8
9
10

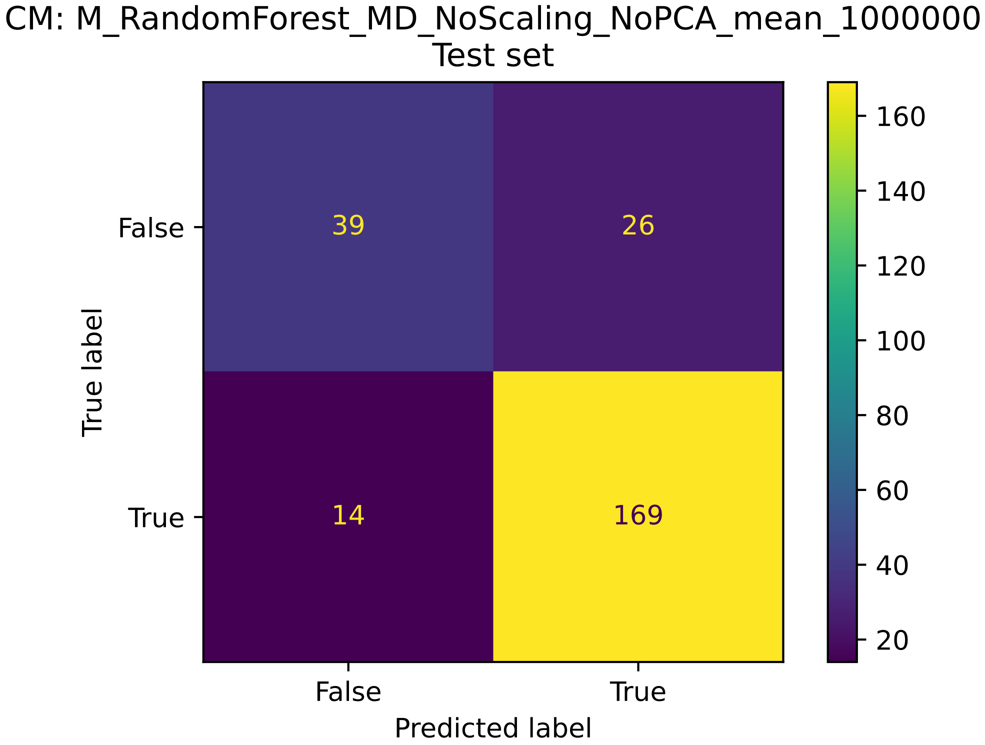
CM: M_RandomForest_MD_NoScaling_NoPCA_mean_1000000
Test set
True label
False
True
39
26
14
169
False
True
Predicted label
160
140
120
100
80
60
40
20

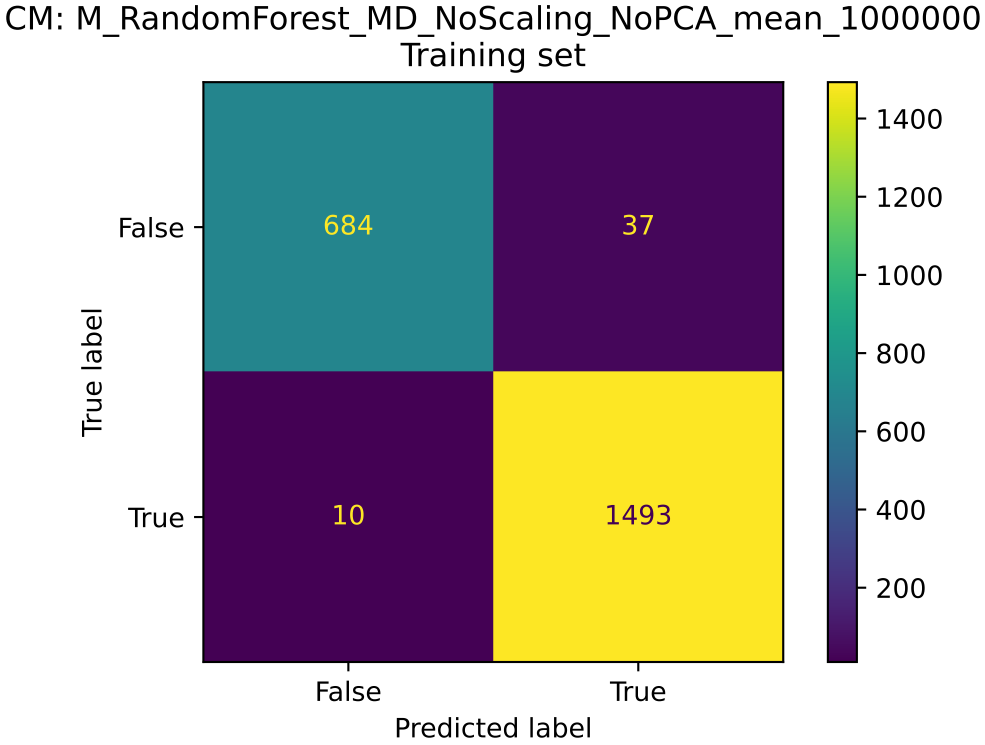
CM: M_RandomForest_MD_NoScaling_NoPCA_mean_1000000
Training set
True label
False
True
684
37
10
1493
False
True
Predicted label
1400
1200
1000
800
600
400
200

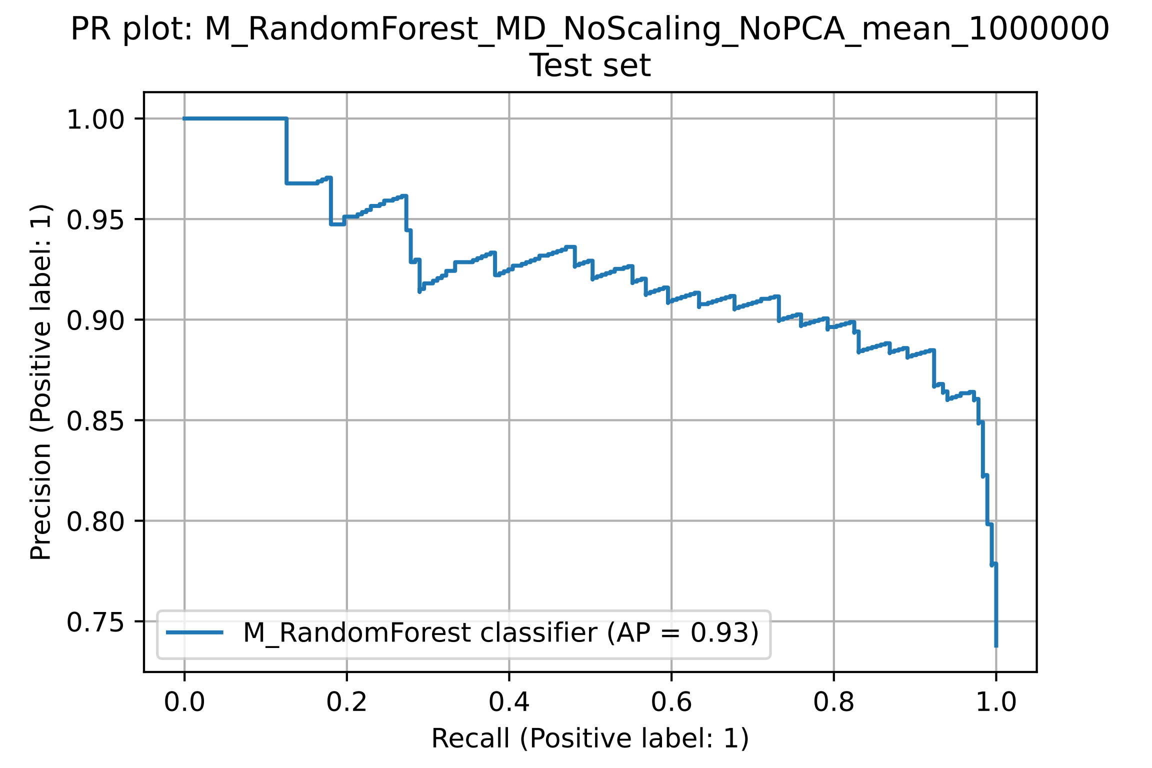
PR plot: M_RandomForest_MD_NoScaling_NoPCA_mean_1000000
Test set
Precision (Positive label: 1)
Recall (Positive label: 1)
1.00
0.95
0.90
0.85
0.80
0.75
0.0
0.2
0.4
0.6
0.8
1.0
M_RandomForest classifier (AP = 0.93)

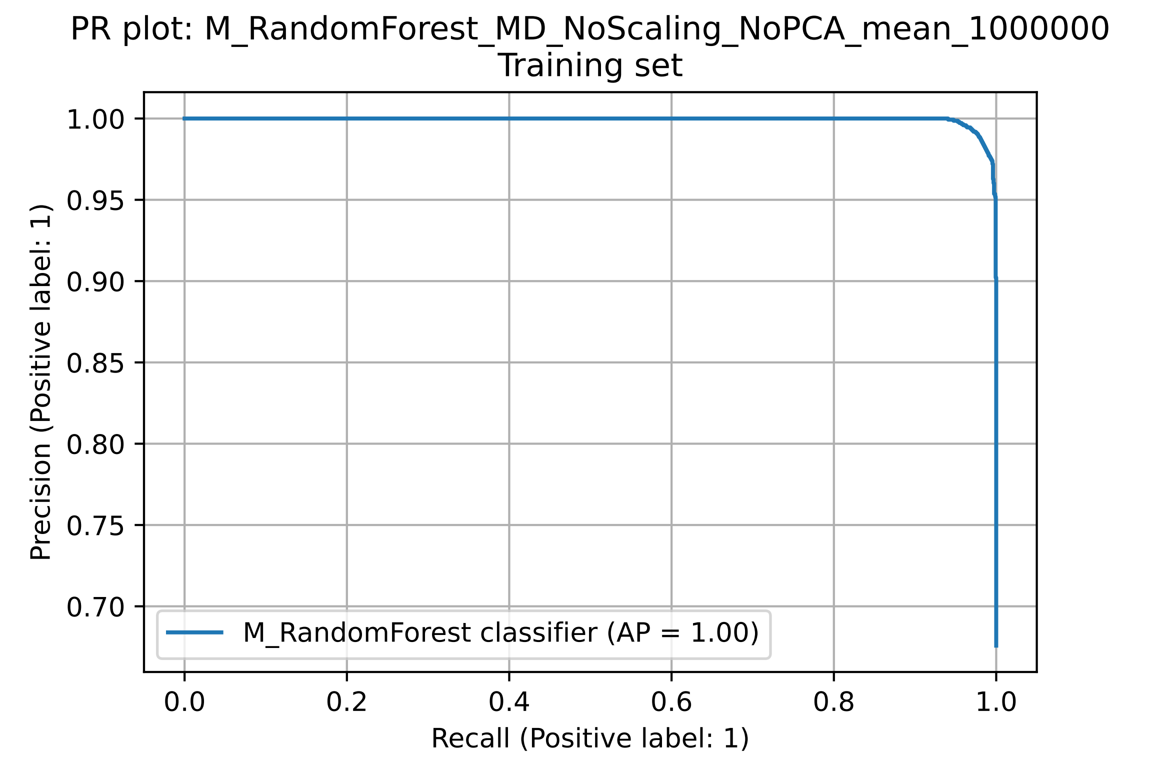
PR plot: M_RandomForest_MD_NoScaling_NoPCA_mean_1000000
Training set
Precision (Positive label: 1)
Recall (Positive label: 1)
1.00
0.95
0.90
0.85
0.80
0.75
0.70
0.0
0.2
0.4
0.6
0.8
1.0
M_RandomForest classifier (AP = 1.00)

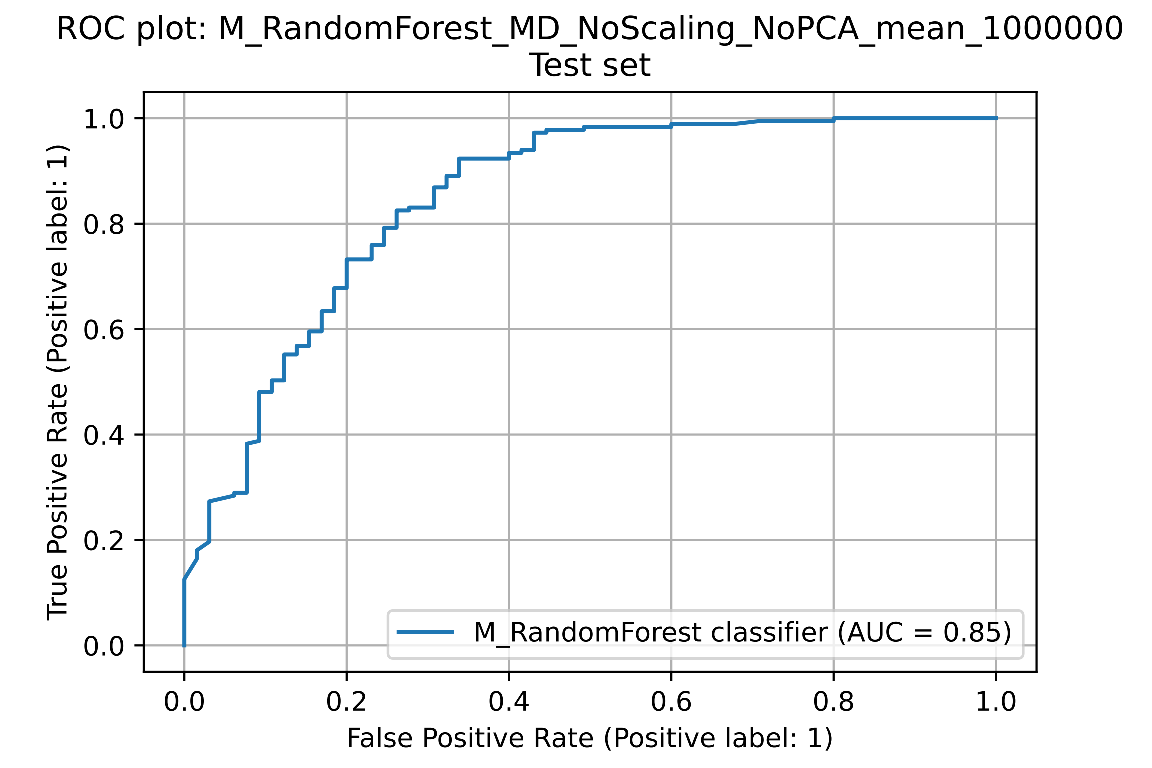
ROC plot: M_RandomForest_MD_NoScaling_NoPCA_mean_1000000
Test set
True Positive Rate (Positive label: 1)
False Positive Rate (Positive label: 1)
0.0
0.2
0.4
0.6
0.8
1.0
M_RandomForest classifier (AUC = 0.85)

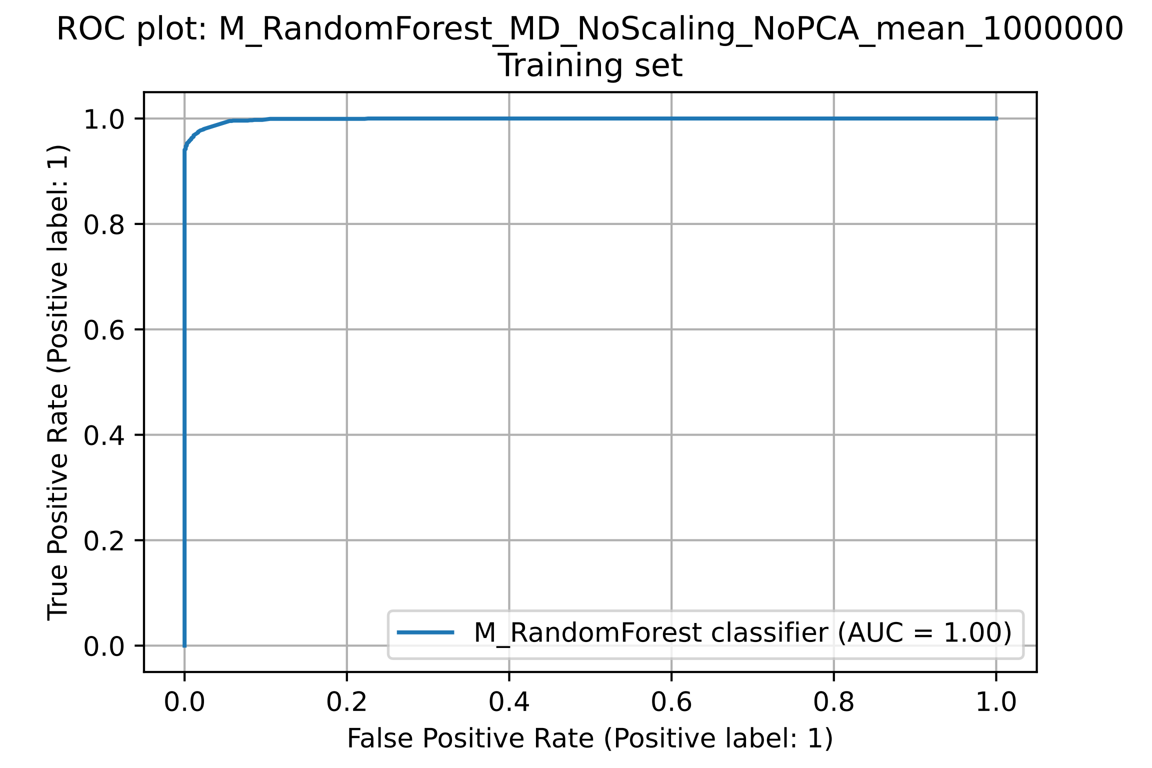
ROC plot: M_RandomForest_MD_NoScaling_NoPCA_mean_1000000
Training set
True Positive Rate (Positive label: 1)
False Positive Rate (Positive label: 1)
M_RandomForest classifier (AUC = 1.00)
0.0
0.2
0.4
0.6
0.8
1.0

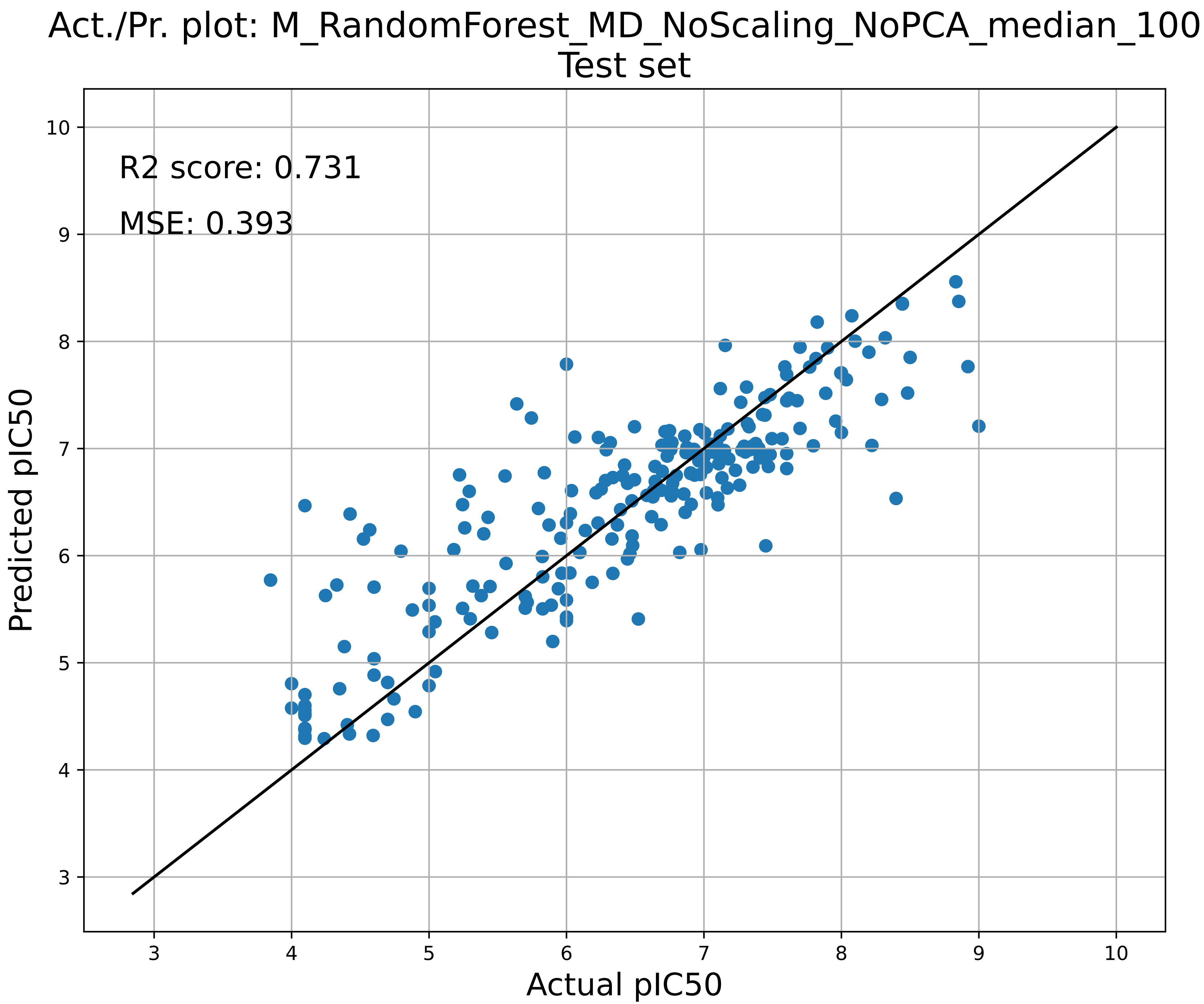

Act./Pr. plot: M_RandomForest_MD_NoScaling_NoPCA_median_100
Test set
R2 score: 0.731
MSE: 0.393
Predicted pIC50
Actual pIC50
3
4
5
6
7
8
9
10

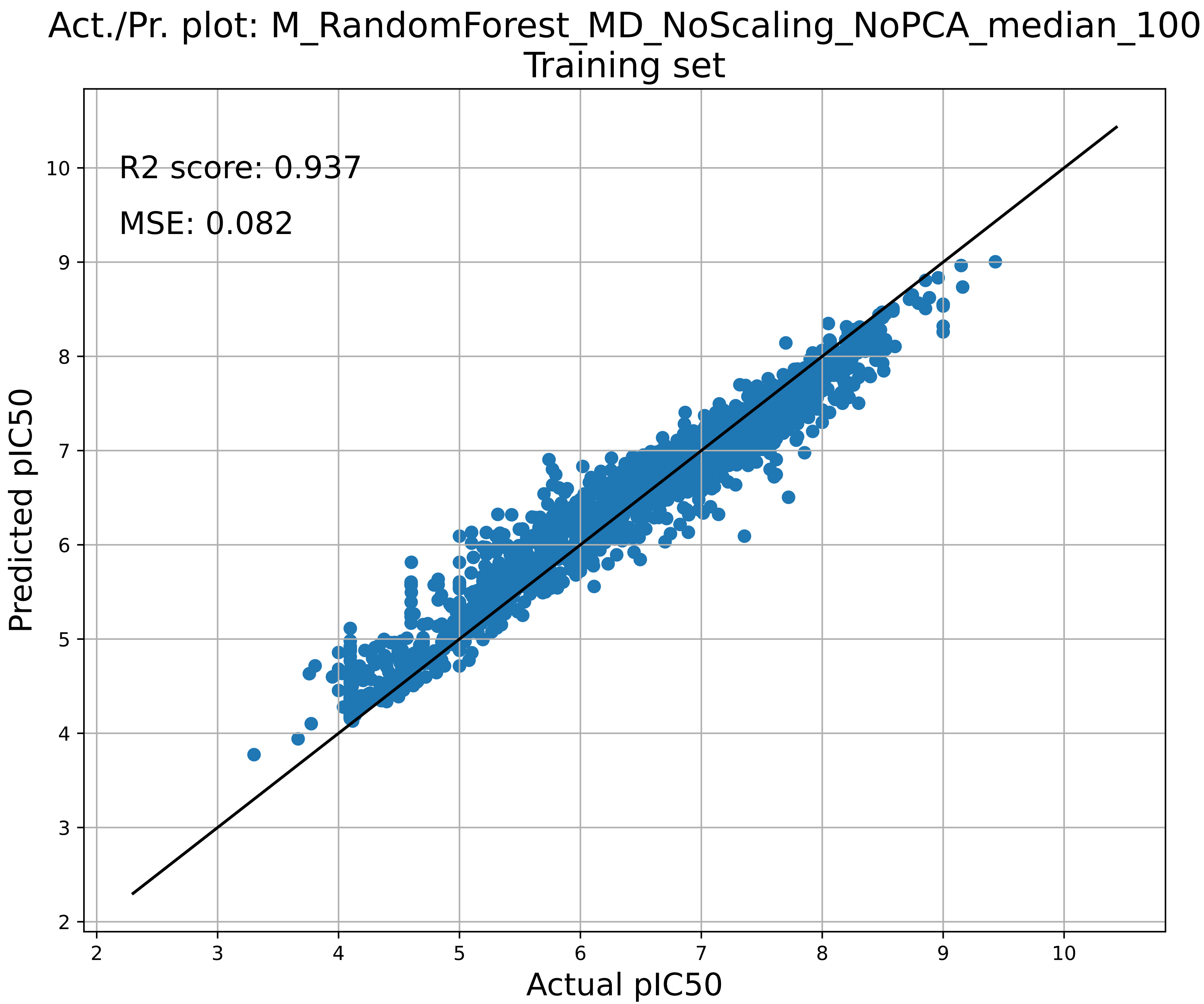
Act./Pr. plot: M_RandomForest_MD_NoScaling_NoPCA_median_100
Training set
R2 score: 0.937
MSE: 0.082
Predicted pIC50
Actual pIC50
2
3
4
5
6
7
8
9
10

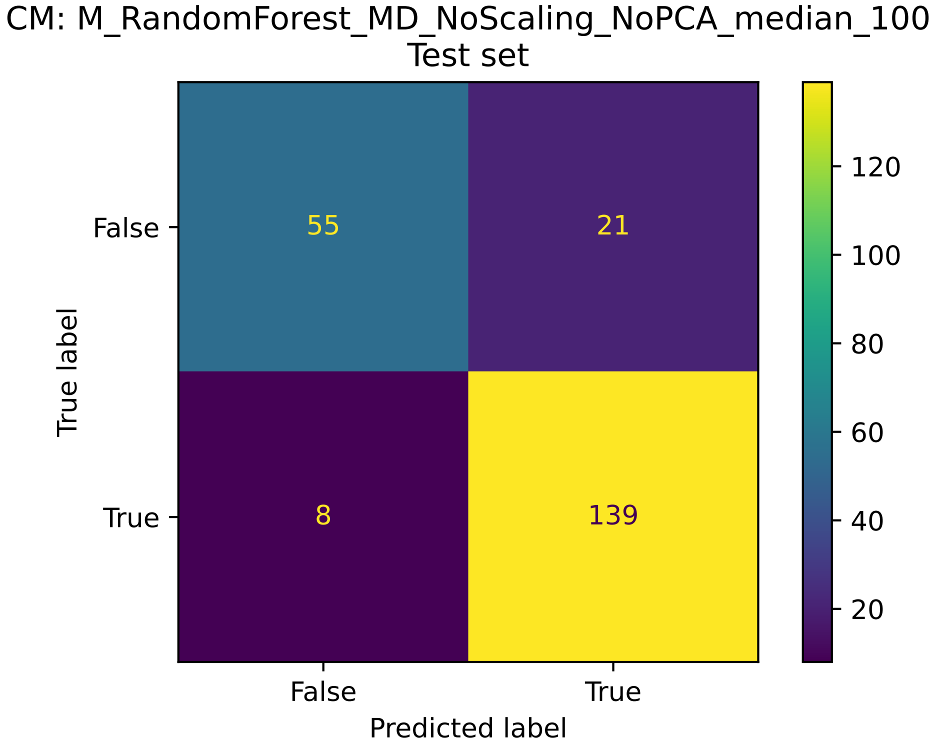

CM: M_RandomForest_MD_NoScaling_NoPCA_median_100
Test set
True label
Predicted label
False
True
False
True
55
21
8
139
20
40
60
80
100
120

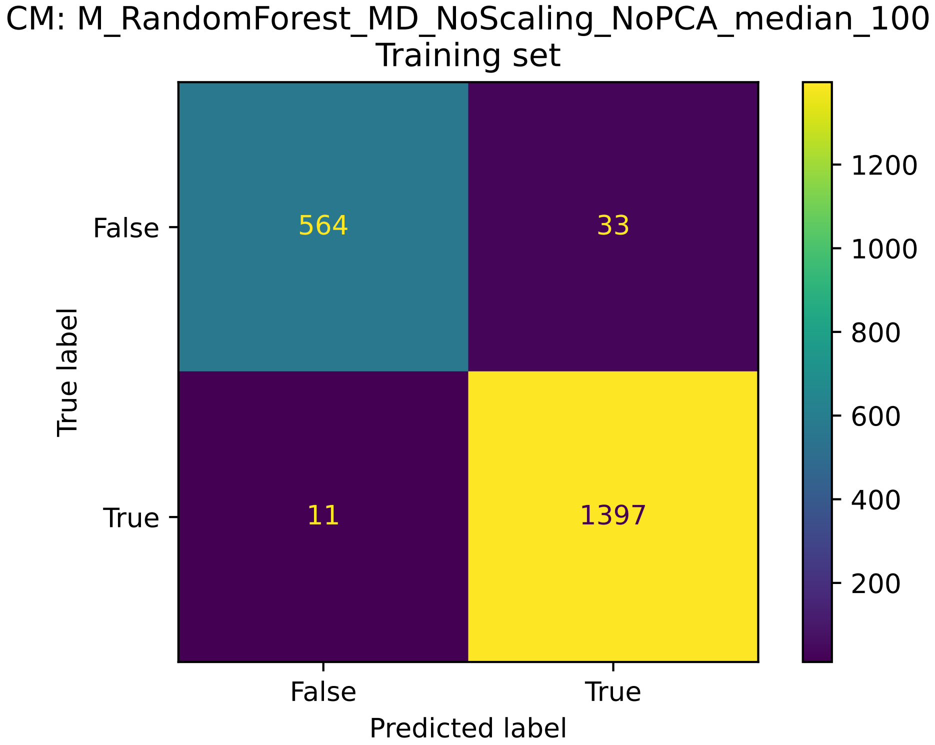
CM: M_RandomForest_MD_NoScaling_NoPCA_median_100
Training set
564
33
11
1397
False
True
True label
False
True
Predicted label
1200
1000
800
600
400
200

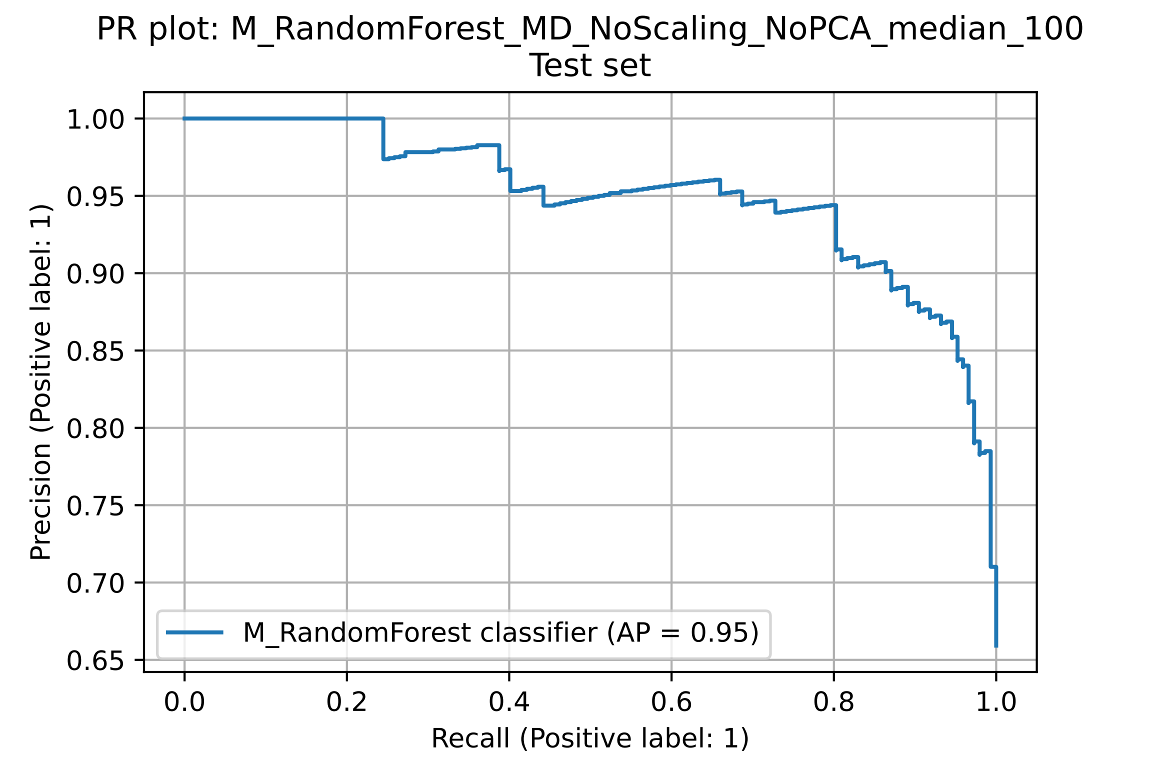
PR plot: M_RandomForest_MD_NoScaling_NoPCA_median_100
Test set
Precision (Positive label: 1)
Recall (Positive label: 1)
1.00
0.95
0.90
0.85
0.80
0.75
0.70
0.65
0.0
0.2
0.4
0.6
0.8
1.0
M_RandomForest classifier (AP = 0.95)

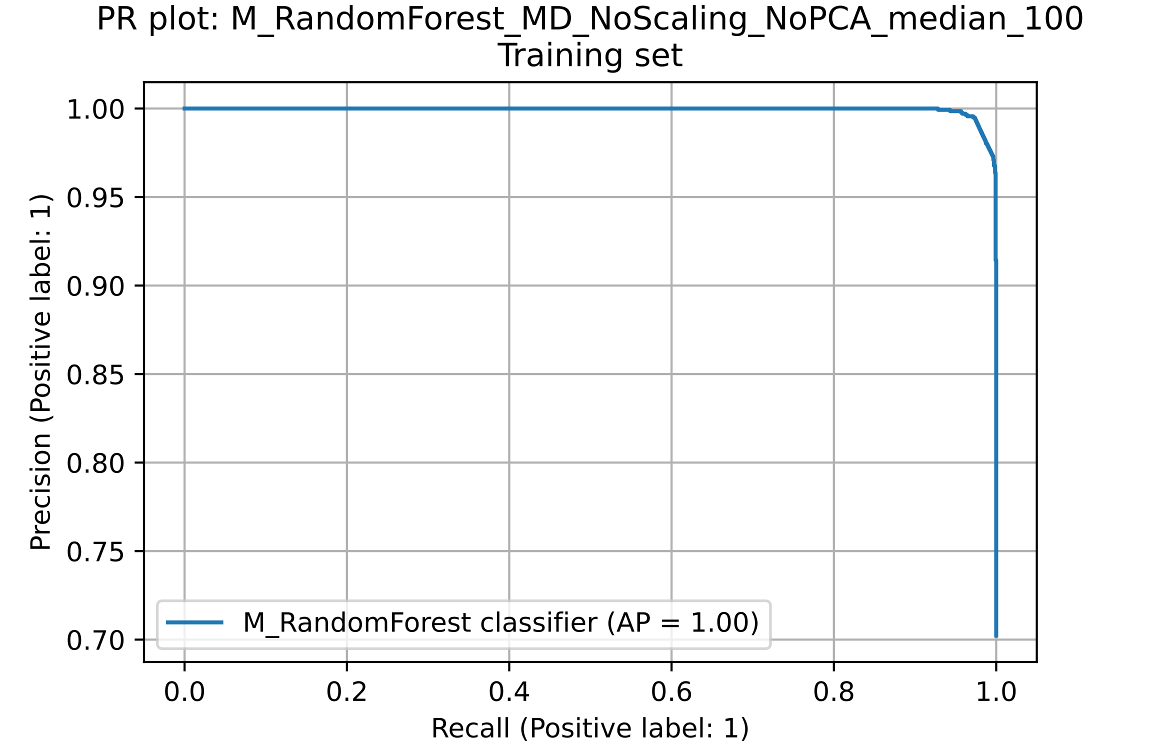
PR plot: M_RandomForest_MD_NoScaling_NoPCA_median_100
Training set
1.00
0.95
0.90
0.85
0.80
0.75
0.70
Precision (Positive label: 1)
0.0
0.2
0.4
0.6
0.8
1.0
Recall (Positive label: 1)
M_RandomForest classifier (AP = 1.00)

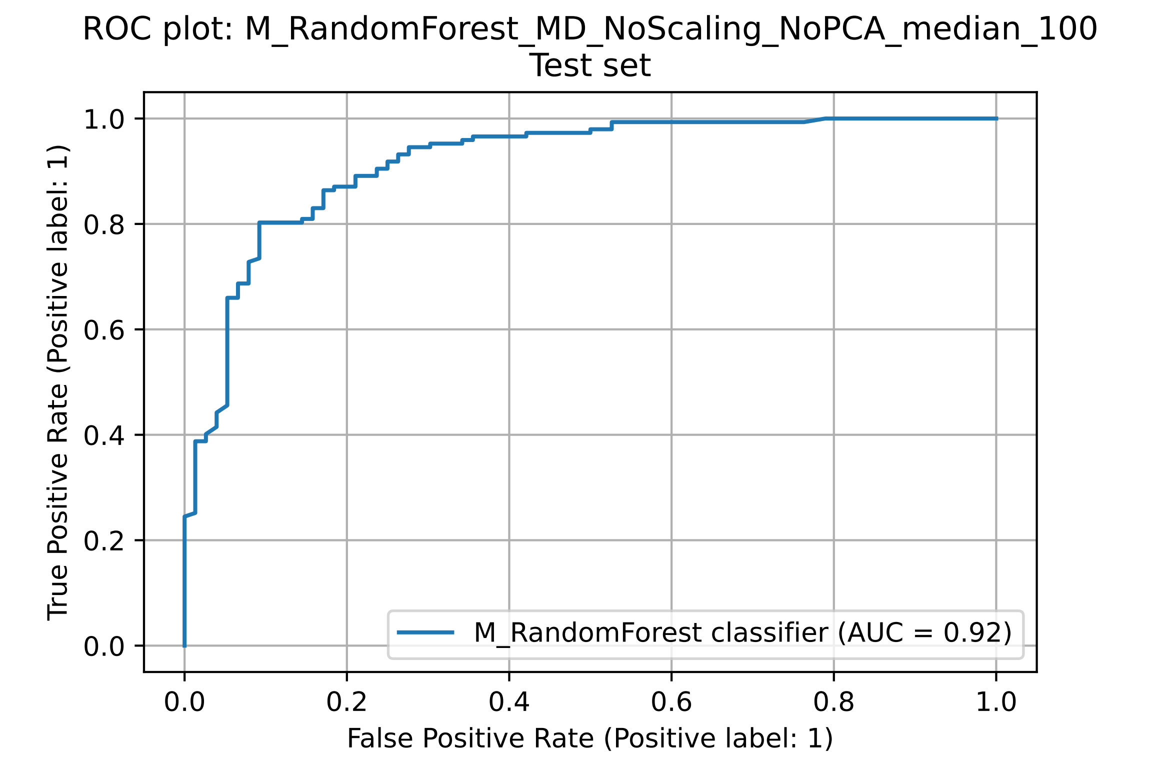
ROC plot: M_RandomForest_MD_NoScaling_NoPCA_median_100
Test set
True Positive Rate (Positive label: 1)
False Positive Rate (Positive label: 1)
0.0
0.2
0.4
0.6
0.8
1.0
M_RandomForest classifier (AUC = 0.92)

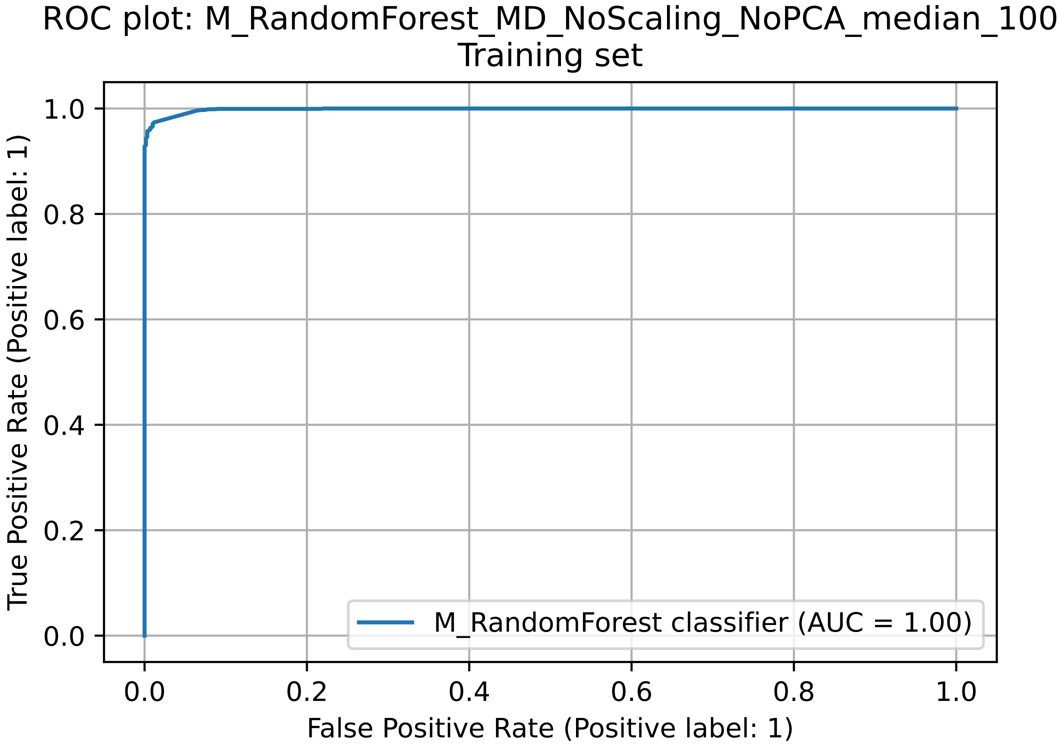
ROC plot: M_RandomForest_MD_NoScaling_NoPCA_median_100
Training set
True Positive Rate (Positive label: 1)
False Positive Rate (Positive label: 1)
0.0
0.2
0.4
0.6
0.8
1.0
M_RandomForest classifier (AUC = 1.00)

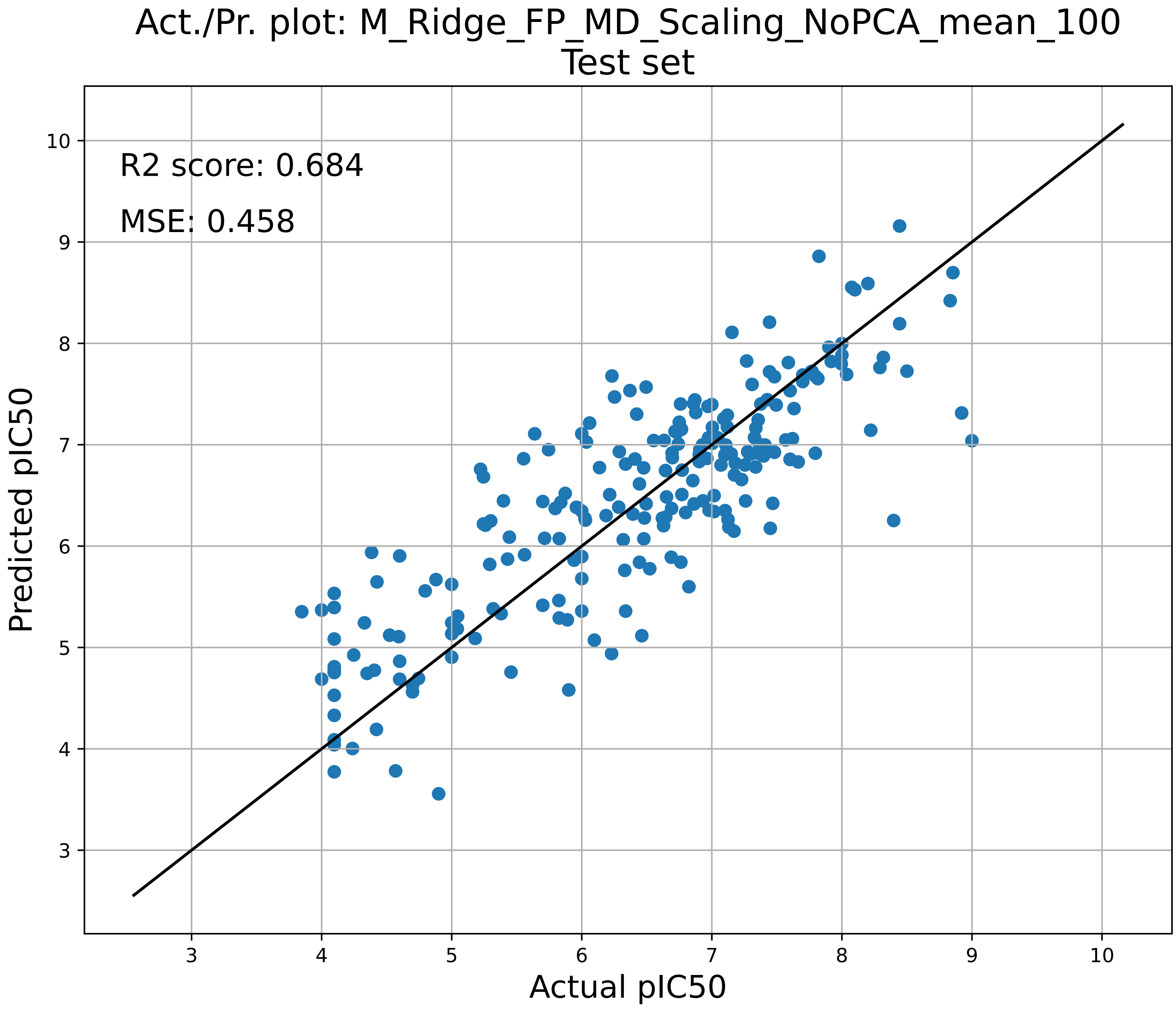
Act./Pr. plot: M_Ridge_FP_MD_Scaling_NoPCA_mean_100
Test set
R2 score: 0.684
MSE: 0.458
Predicted pIC50
Actual pIC50
3
4
5
6
7
8
9
10

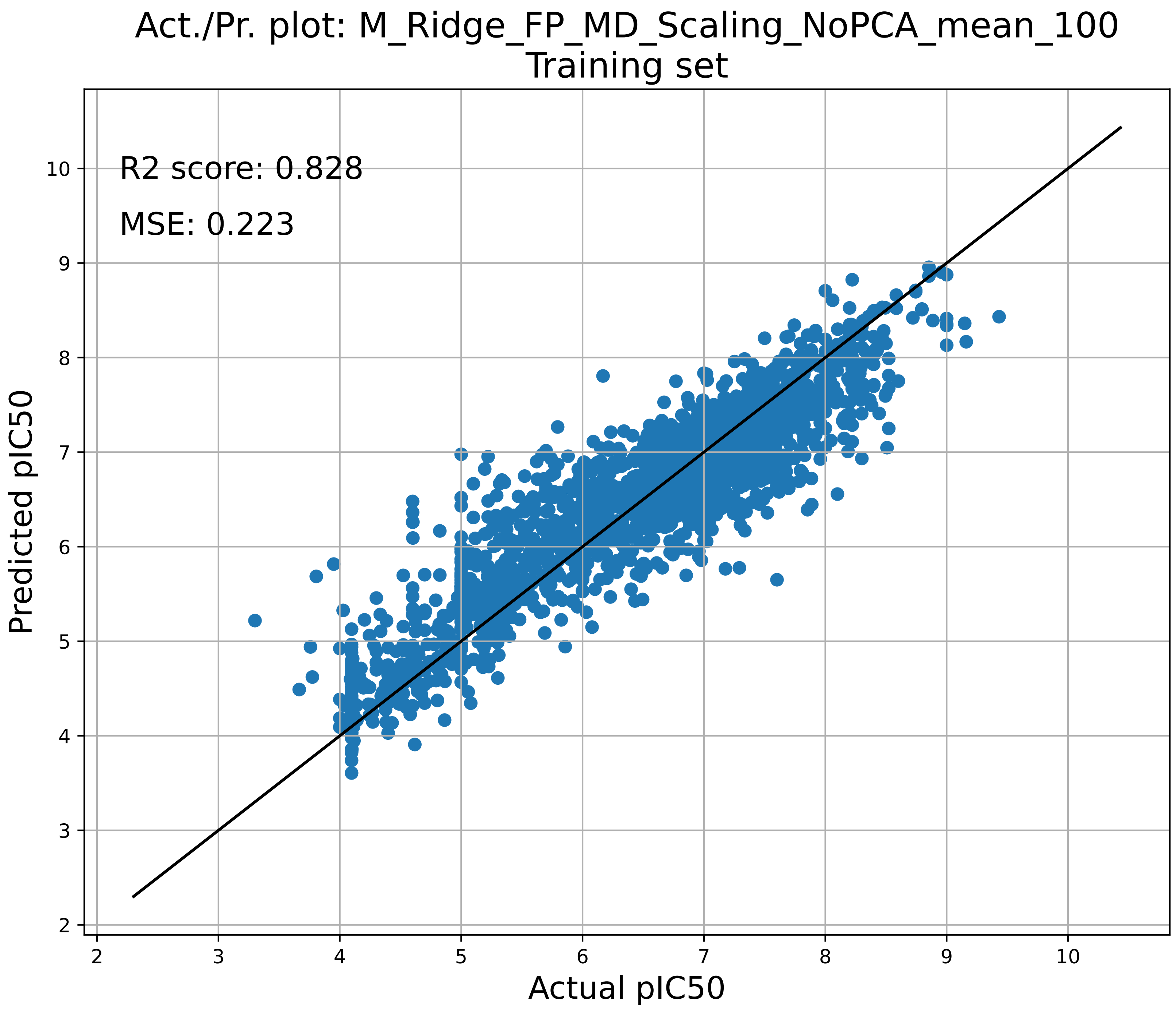
Act./Pr. plot: M_Ridge_FP_MD_Scaling_NoPCA_mean_100
Training set
R2 score: 0.828
MSE: 0.223
Predicted pIC50
Actual pIC50
2
3
4
5
6
7
8
9
10

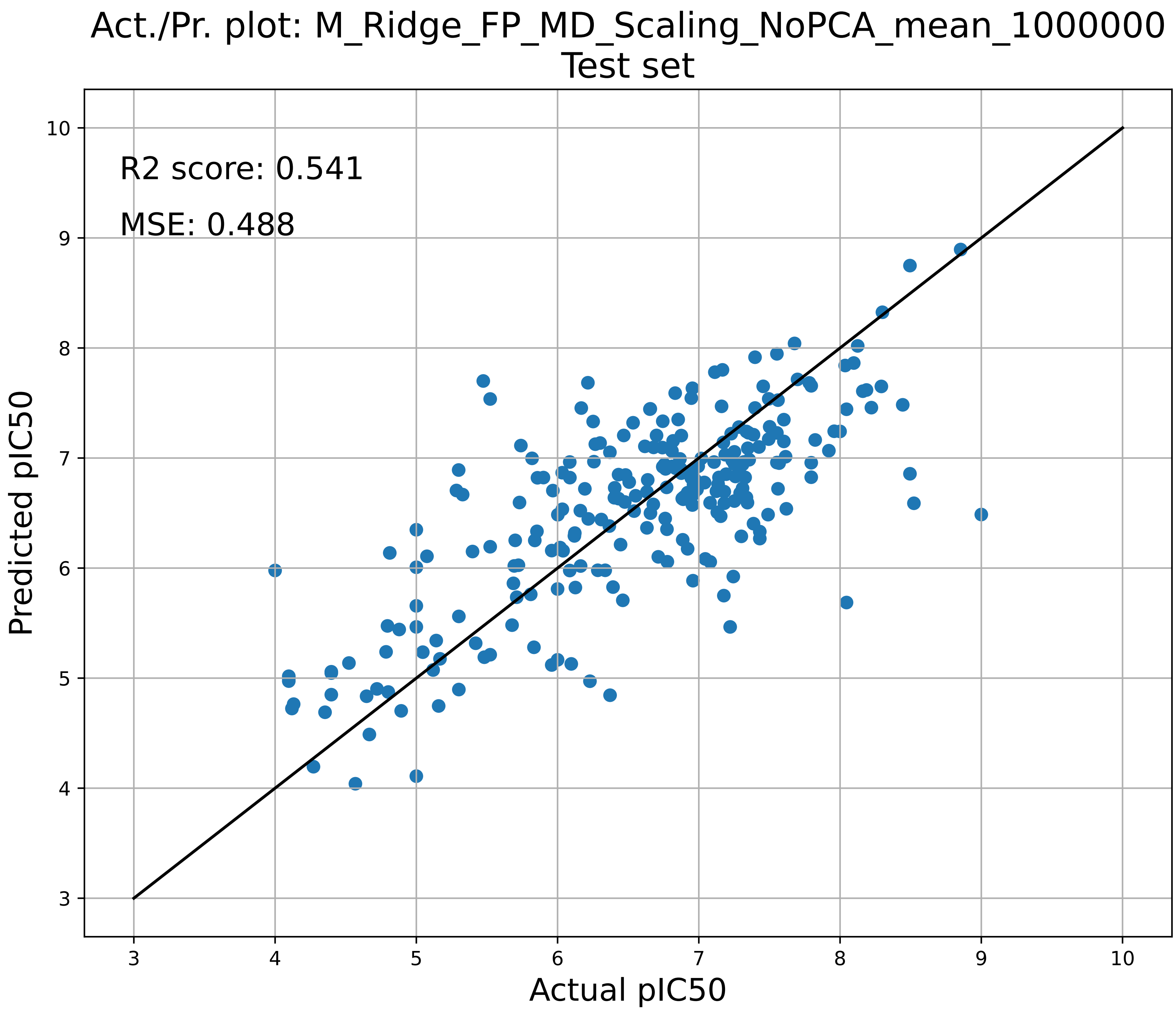

Act./Pr. plot: M_Ridge_FP_MD_Scaling_NoPCA_mean_1000000
Test set
R2 score: 0.541
MSE: 0.488
Predicted pIC50
Actual pIC50
3
4
5
6
7
8
9
10

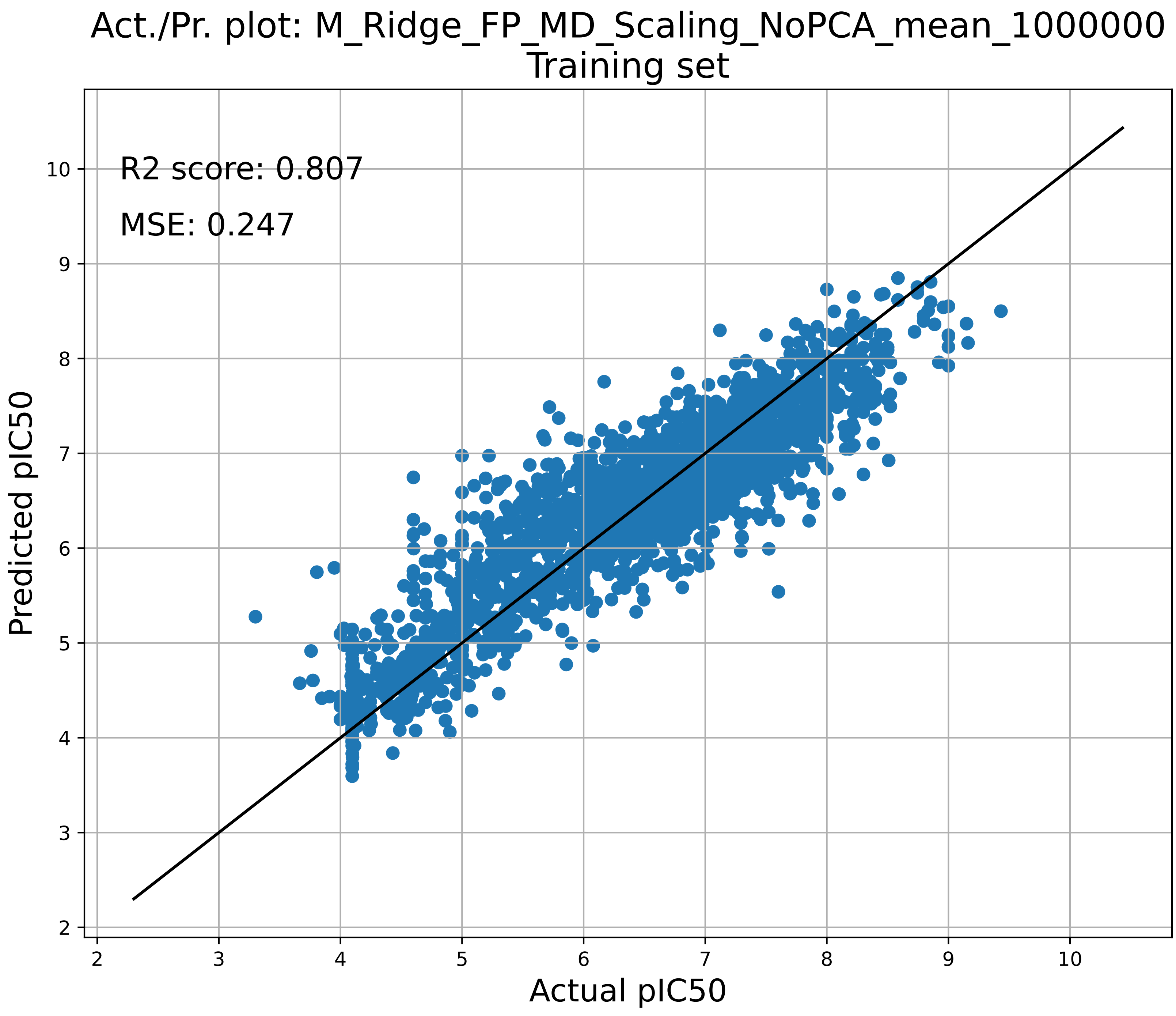

Act./Pr. plot: M_Ridge_FP_MD_Scaling_NoPCA_mean_1000000
Training set
R2 score: 0.807
MSE: 0.247
Predicted pIC50
Actual pIC50
2
3
4
5
6
7
8
9
10

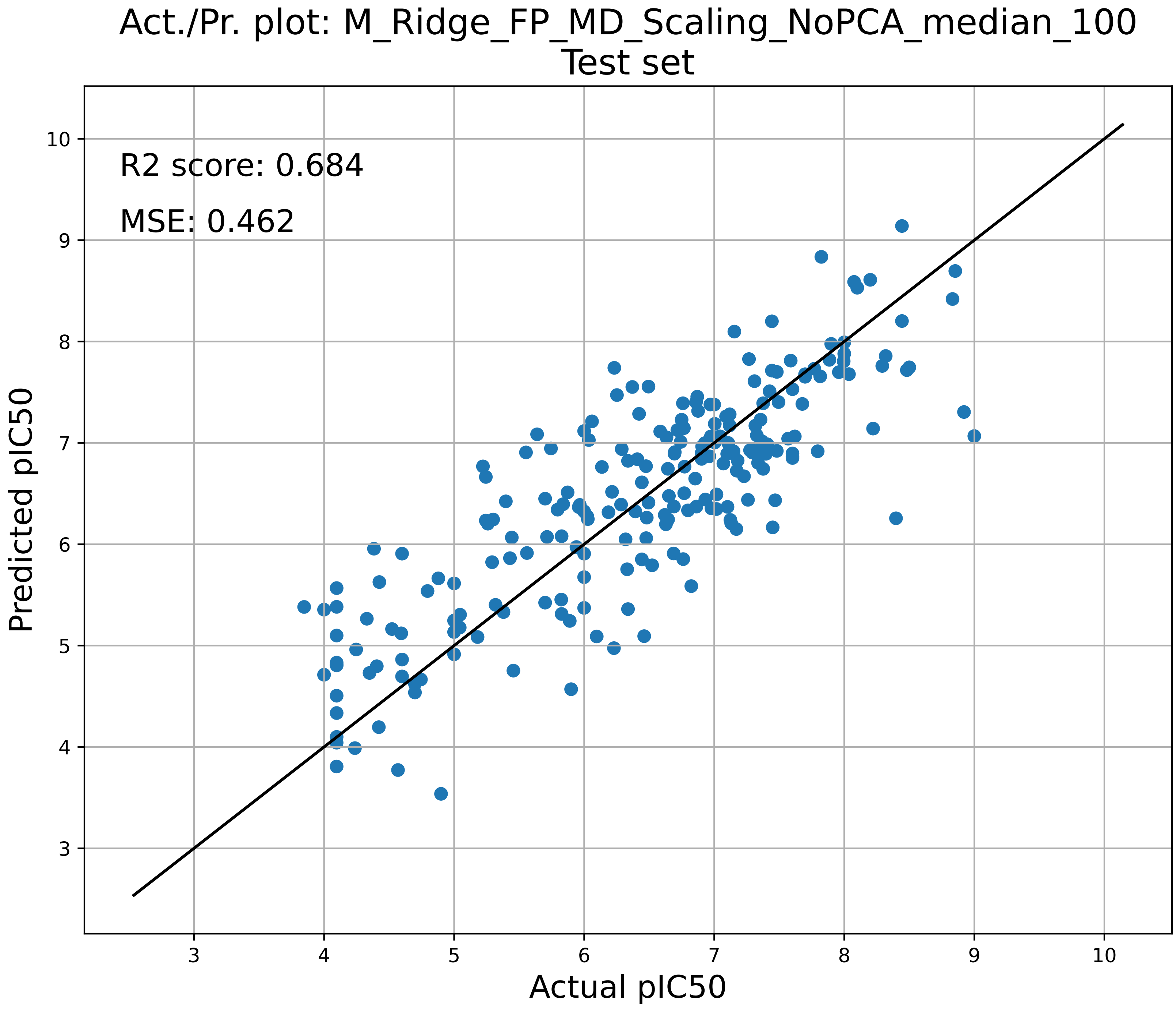
Act./Pr. plot: M_Ridge_FP_MD_Scaling_NoPCA_median_100
Test set
R2 score: 0.684
MSE: 0.462
Predicted pIC50
Actual pIC50
3
4
5
6
7
8
9
10

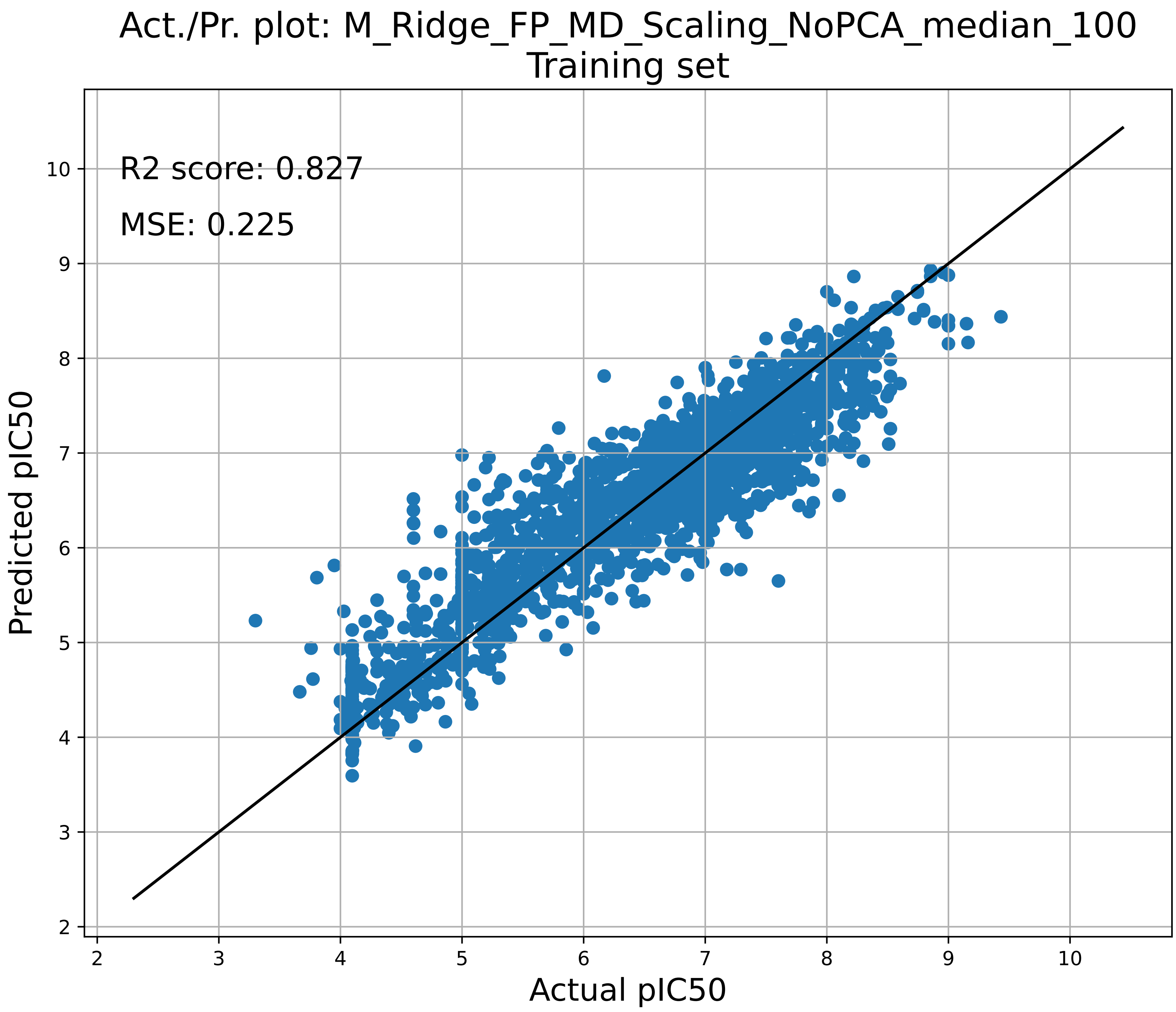
Act./Pr. plot: M_Ridge_FP_MD_Scaling_NoPCA_median_100
Training set
R2 score: 0.827
MSE: 0.225
Predicted pIC50
Actual pIC50
2
3
4
5
6
7
8
9
10

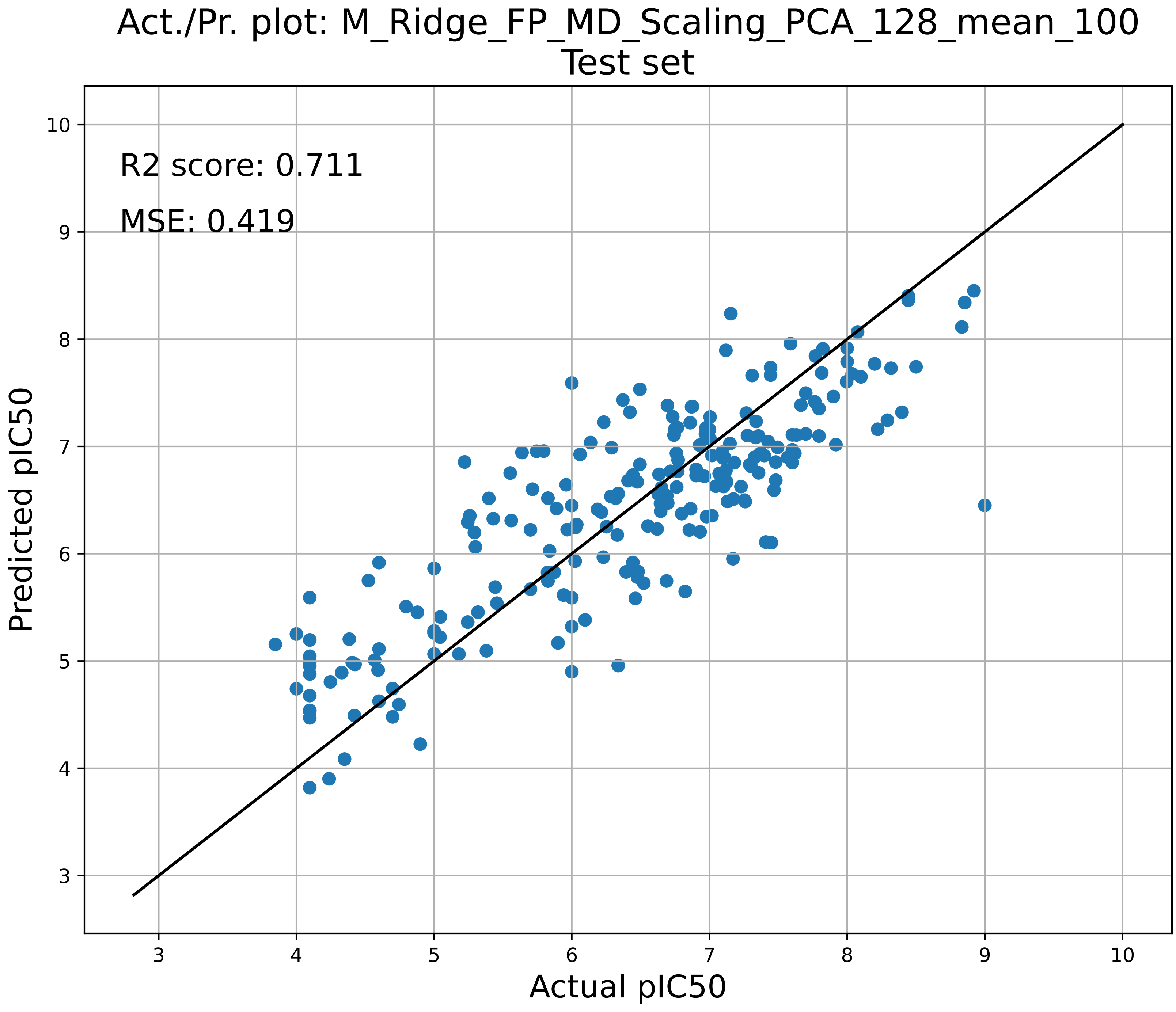
Act./Pr. plot: M_Ridge_FP_MD_Scaling_PCA_128_mean_100
Test set
R2 score: 0.711
MSE: 0.419
Predicted pIC50
Actual pIC50
3
4
5
6
7
8
9
10

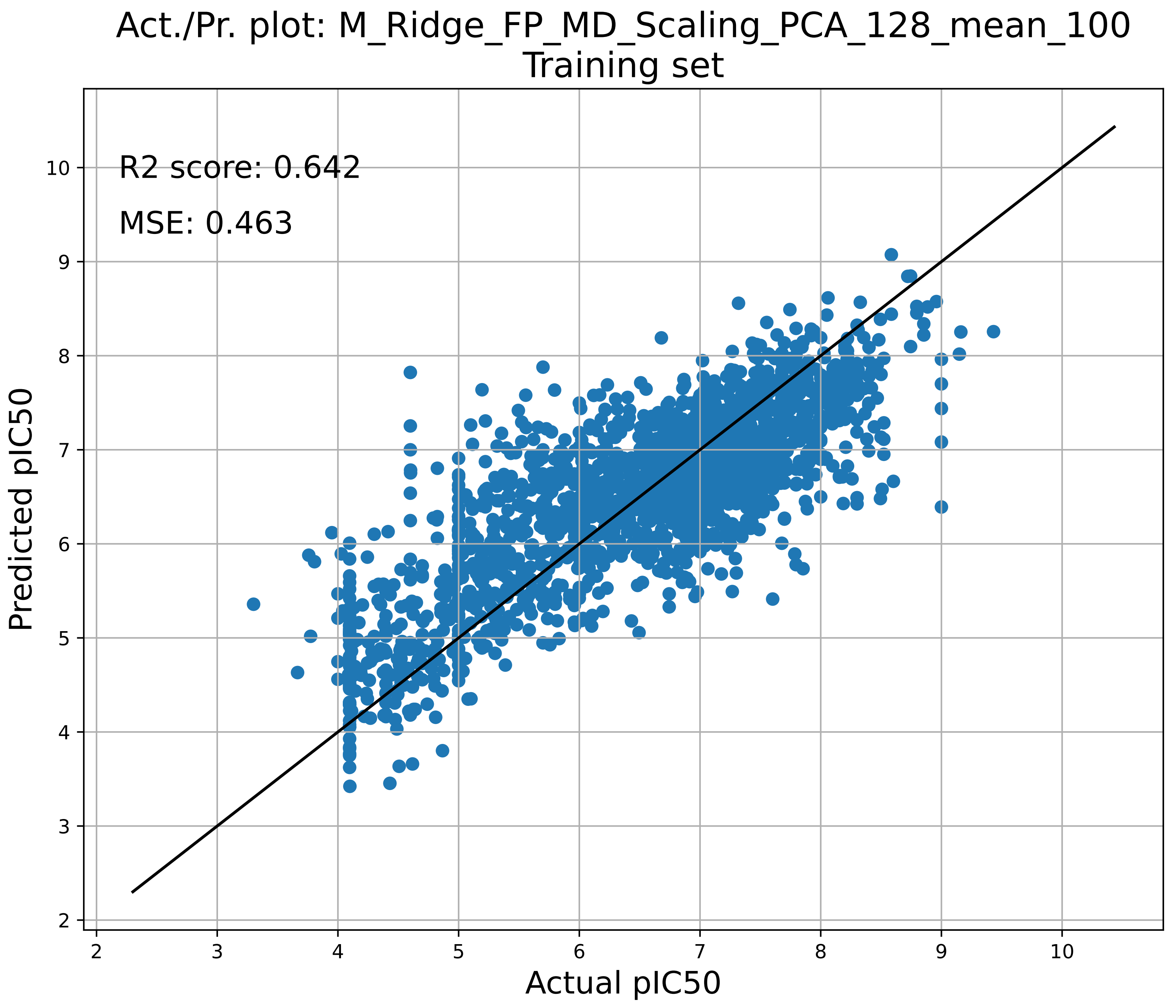

Act./Pr. plot: M_Ridge_FP_MD_Scaling_PCA_128_mean_100
Training set
R2 score: 0.642
MSE: 0.463
Predicted pIC50
Actual pIC50
2
3
4
5
6
7
8
9
10

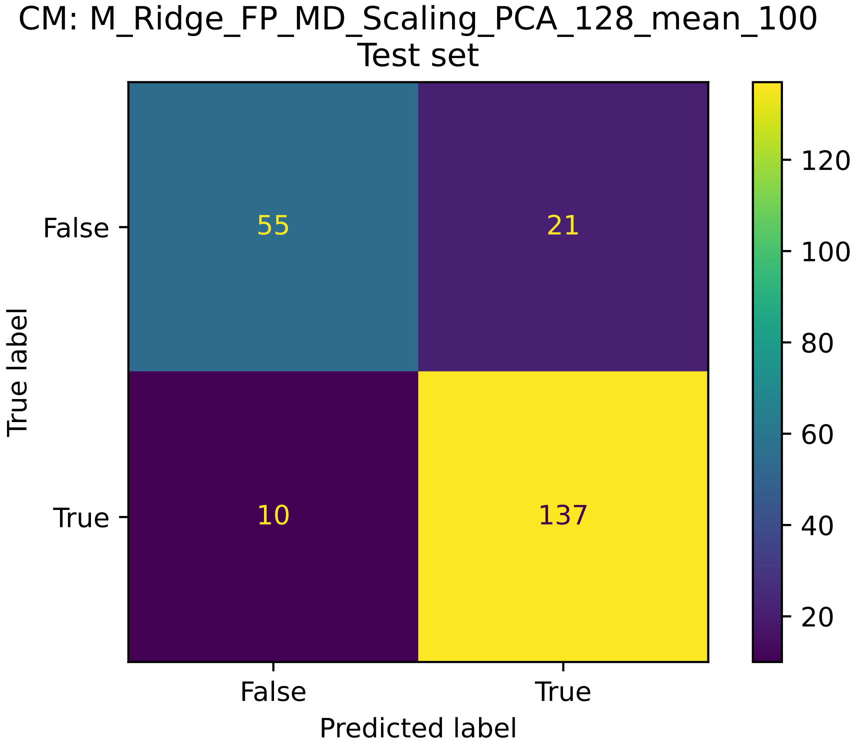
CM: M_Ridge_FP_MD_Scaling_PCA_128_mean_100
Test set
55
21
10
137
False
True
False
True
True label
Predicted label
20
40
60
80
100
120

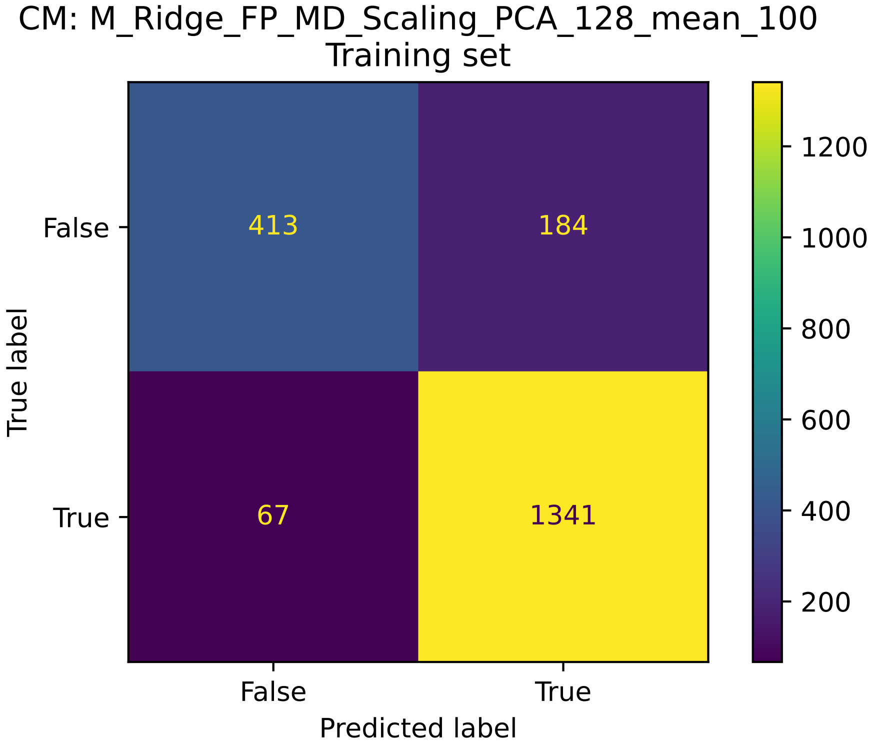
CM: M_Ridge_FP_MD_Scaling_PCA_128_mean_100
Training set
True label
False
True
413
184
67
1341
Predicted label
False
True
1200
1000
800
600
400
200

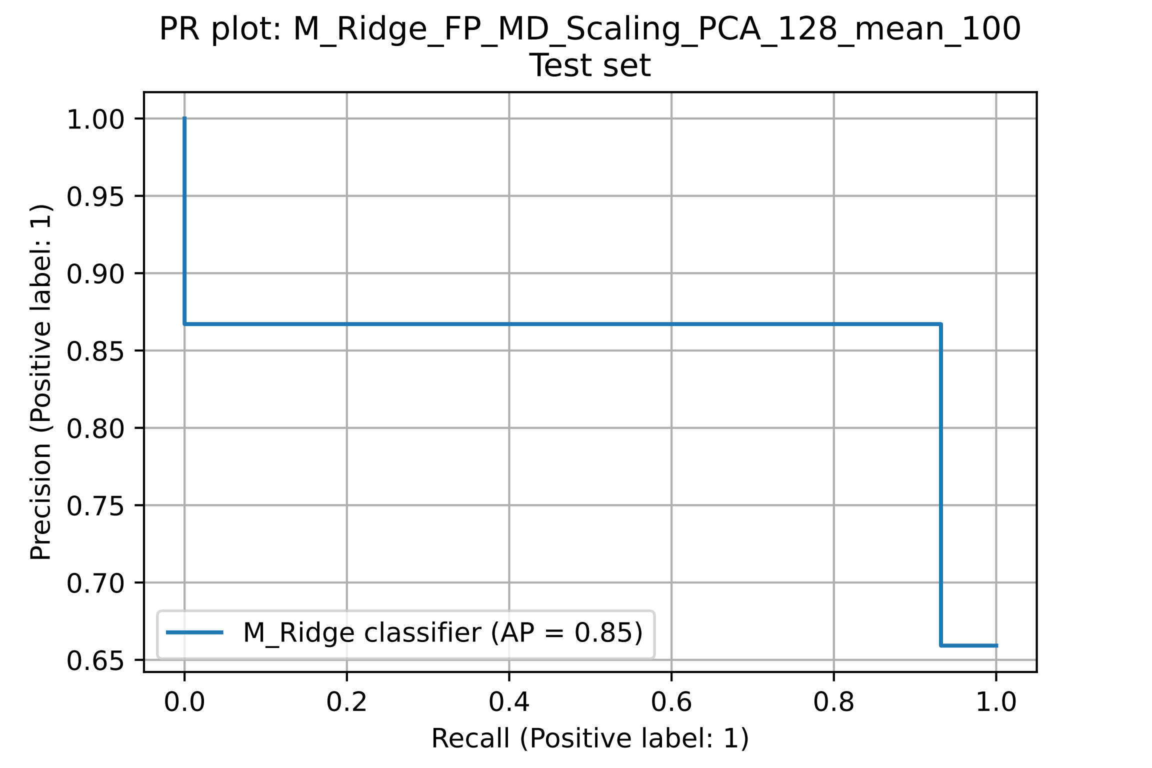

PR plot: M_Ridge_FP_MD_Scaling_PCA_128_mean_100
Test set
Precision (Positive label: 1)
Recall (Positive label: 1)
1.00
0.95
0.90
0.85
0.80
0.75
0.70
0.65
0.0
0.2
0.4
0.6
0.8
1.0
M_Ridge classifier (AP = 0.85)

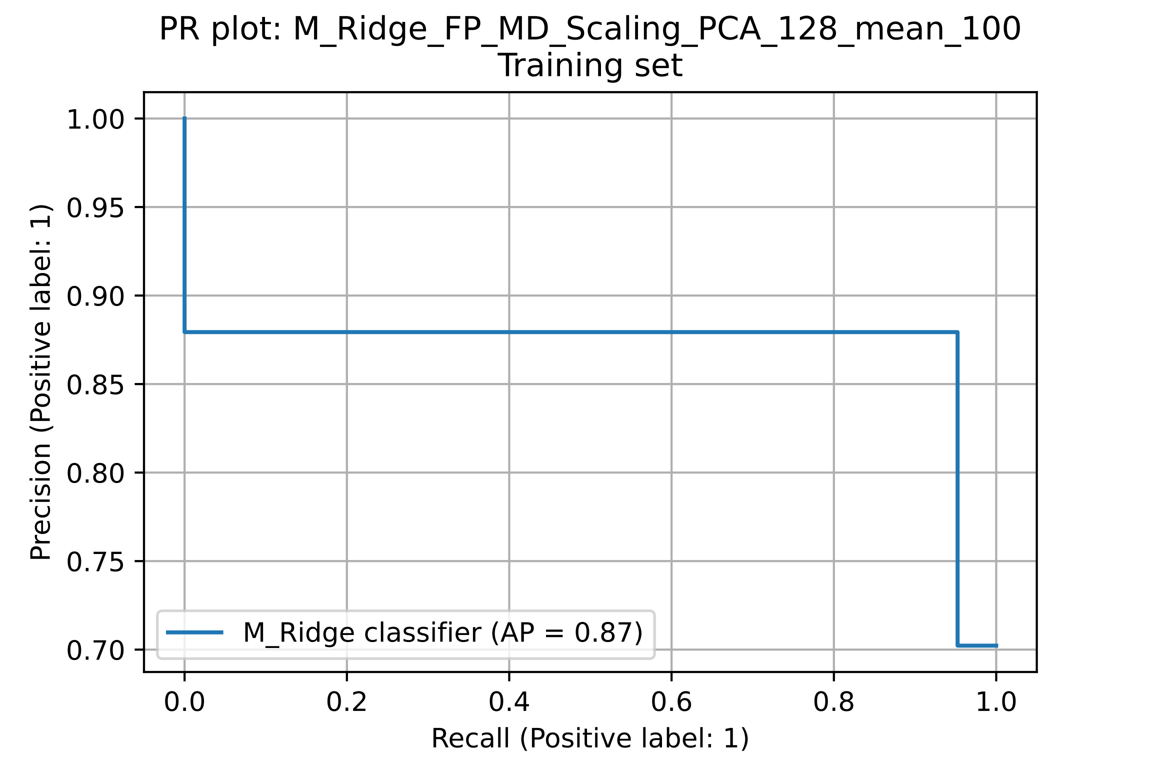
PR plot: M_Ridge_FP_MD_Scaling_PCA_128_mean_100
Training set
Precision (Positive label: 1)
Recall (Positive label: 1)
1.00
0.95
0.90
0.85
0.80
0.75
0.70
0.0
0.2
0.4
0.6
0.8
1.0
M_Ridge classifier (AP = 0.87)

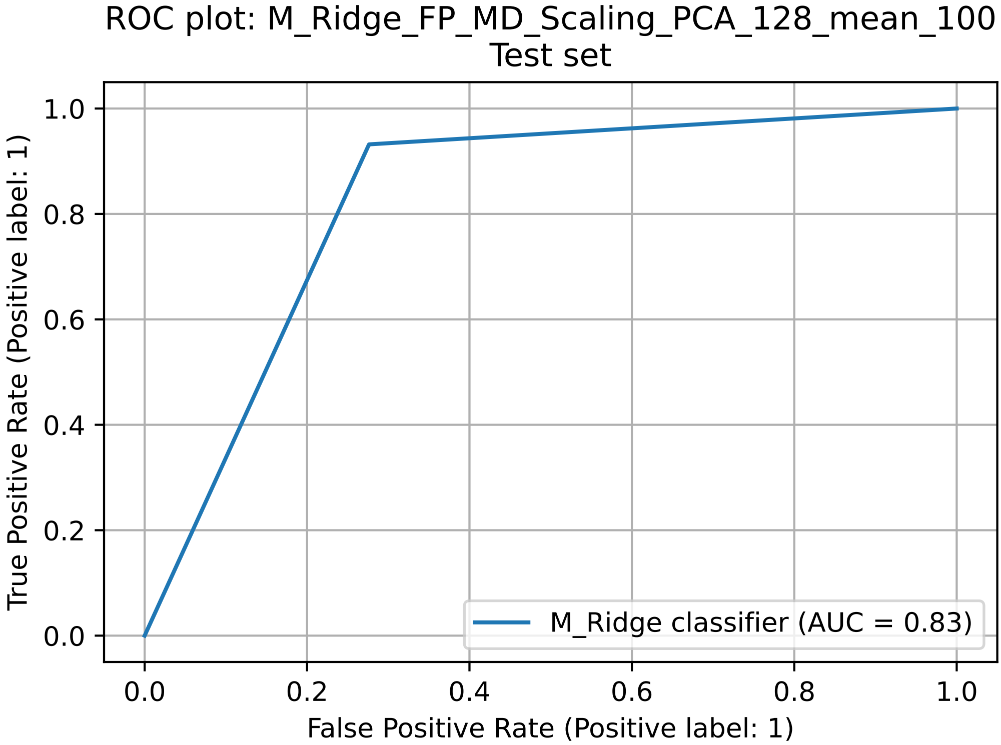
ROC plot: M_Ridge_FP_MD_Scaling_PCA_128_mean_100
Test set
True Positive Rate (Positive label: 1)
False Positive Rate (Positive label: 1)
0.0
0.2
0.4
0.6
0.8
1.0
M_Ridge classifier (AUC = 0.83)

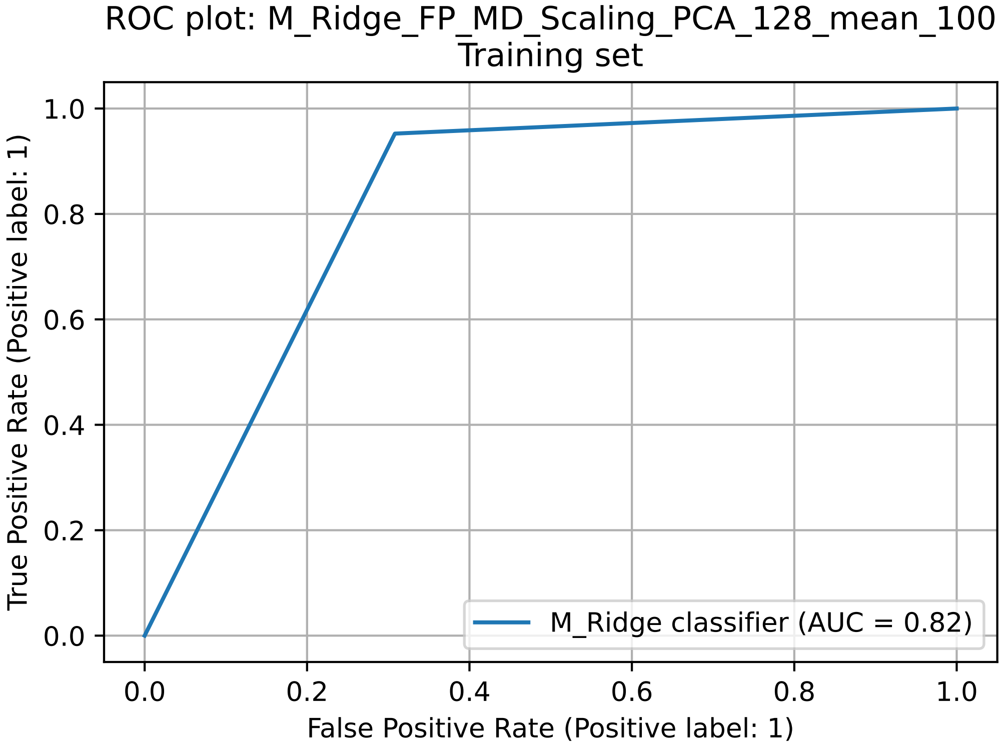

ROC plot: M_Ridge_FP_MD_Scaling_PCA_128_mean_100
Training set
True Positive Rate (Positive label: 1)
False Positive Rate (Positive label: 1)
0.0
0.2
0.4
0.6
0.8
1.0
M_Ridge classifier (AUC = 0.82)

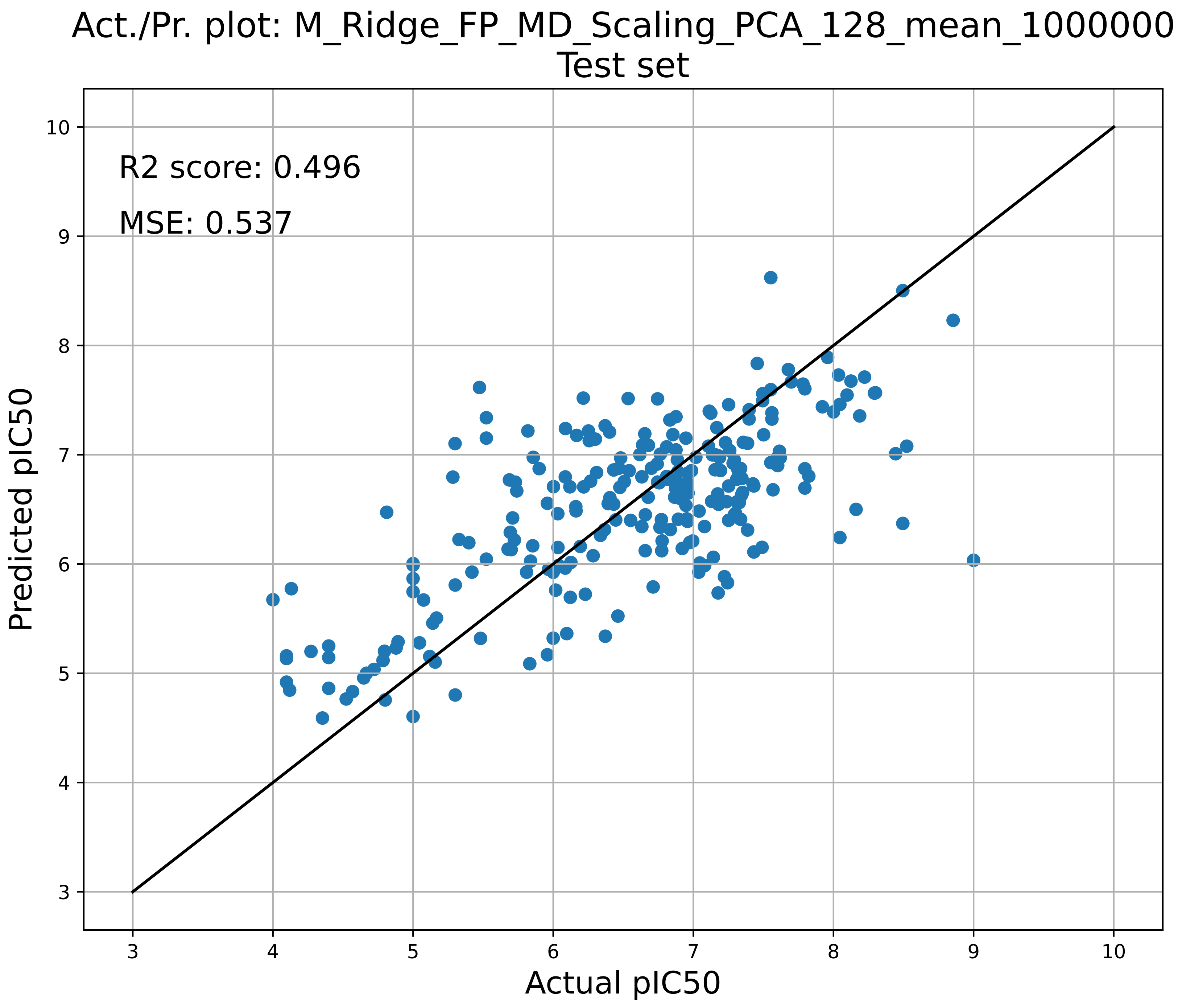

Act./Pr. plot: M_Ridge_FP_MD_Scaling_PCA_128_mean_1000000
Test set
R2 score: 0.496
MSE: 0.537
Predicted pIC50
Actual pIC50
3
4
5
6
7
8
9
10

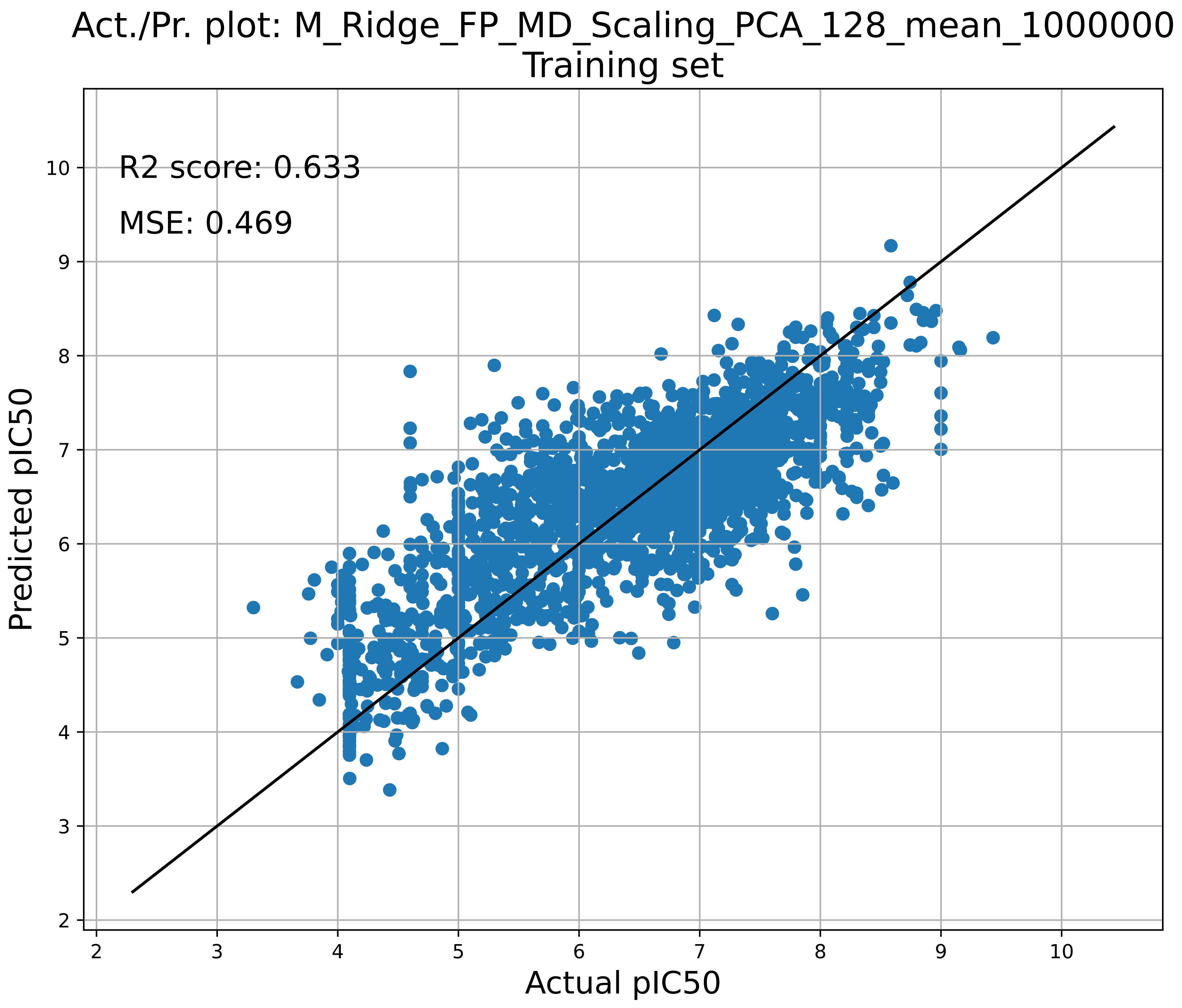

Act./Pr. plot: M_Ridge_FP_MD_Scaling_PCA_128_mean_1000000
Training set
R2 score: 0.633
MSE: 0.469
Predicted pIC50
Actual pIC50
2
3
4
5
6
7
8
9
10

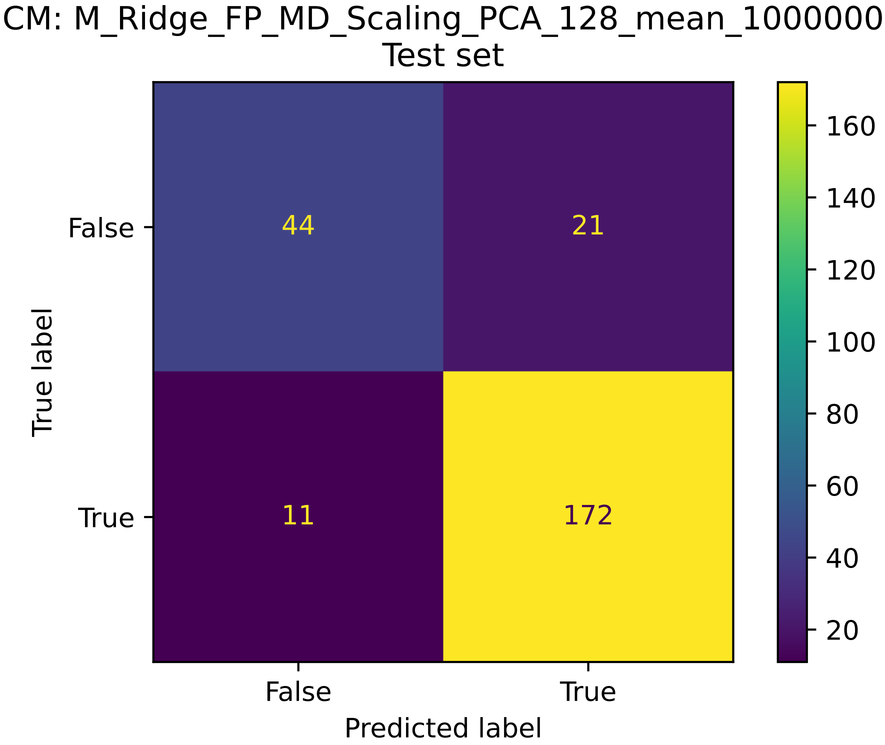
CM: M_Ridge_FP_MD_Scaling_PCA_128_mean_1000000
Test set
True label
Predicted label
False
True
False
True
44
21
11
172
20
40
60
80
100
120
140
160

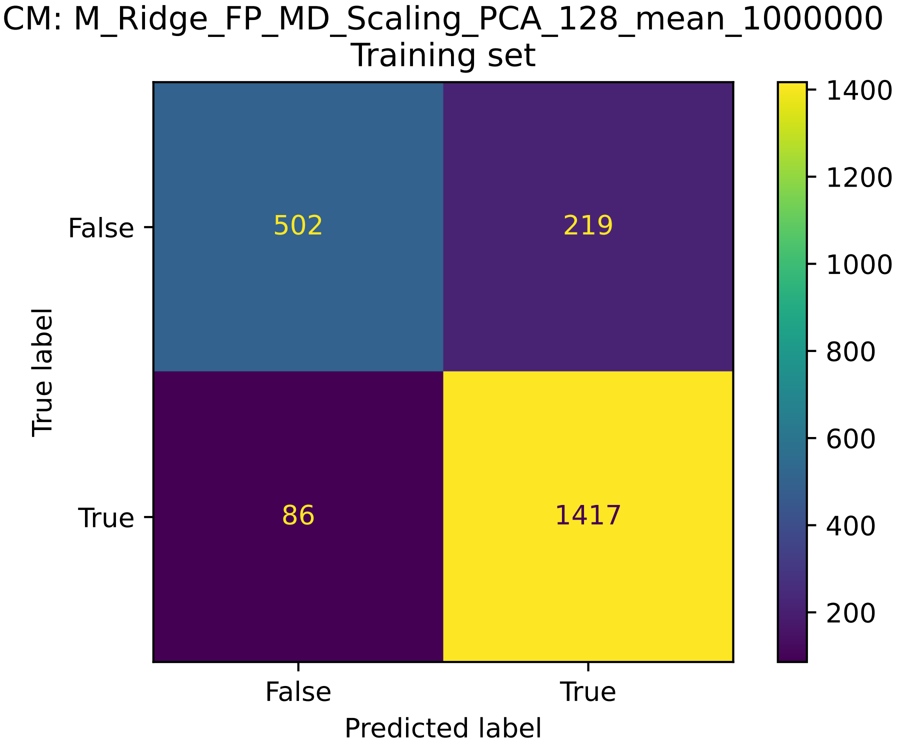
CM: M_Ridge_FP_MD_Scaling_PCA_128_mean_1000000
Training set
502
219
86
1417
False
True
True label
False
True
Predicted label
1400
1200
1000
800
600
400
200

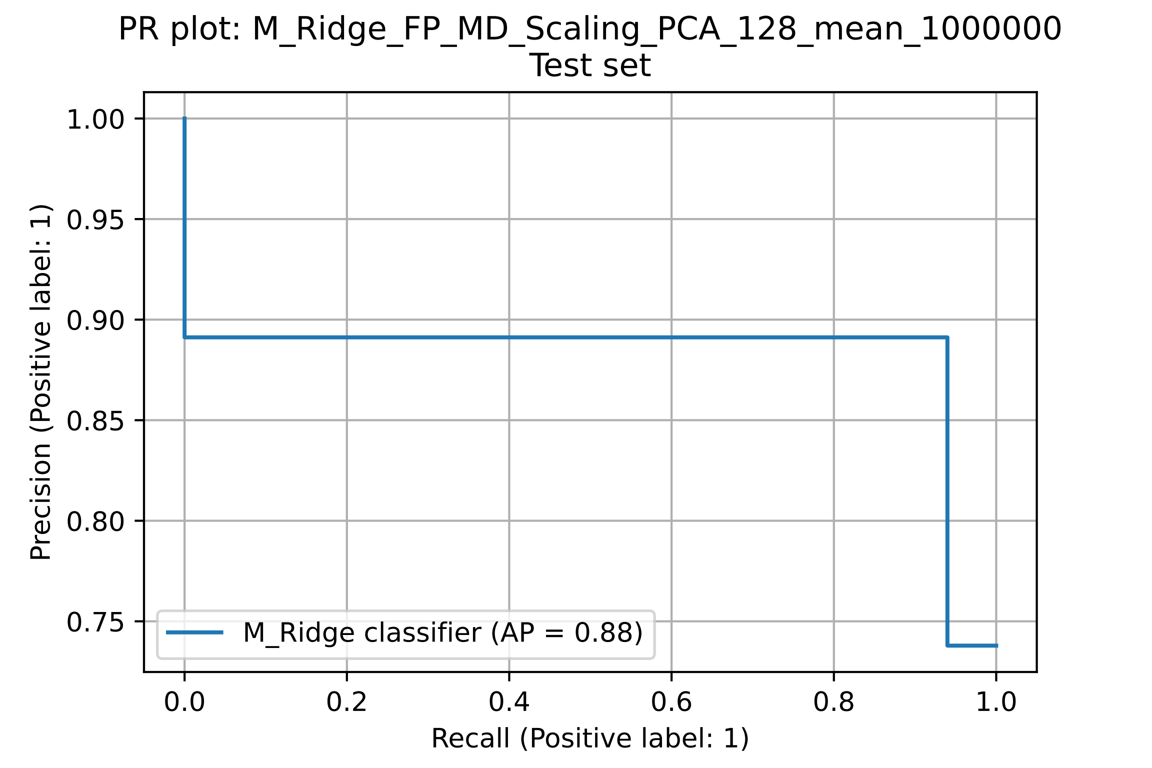
PR plot: M_Ridge_FP_MD_Scaling_PCA_128_mean_1000000
Test set
Precision (Positive label: 1)
Recall (Positive label: 1)
M_Ridge classifier (AP = 0.88)
1.00
0.95
0.90
0.85
0.80
0.75
0.0
0.2
0.4
0.6
0.8
1.0

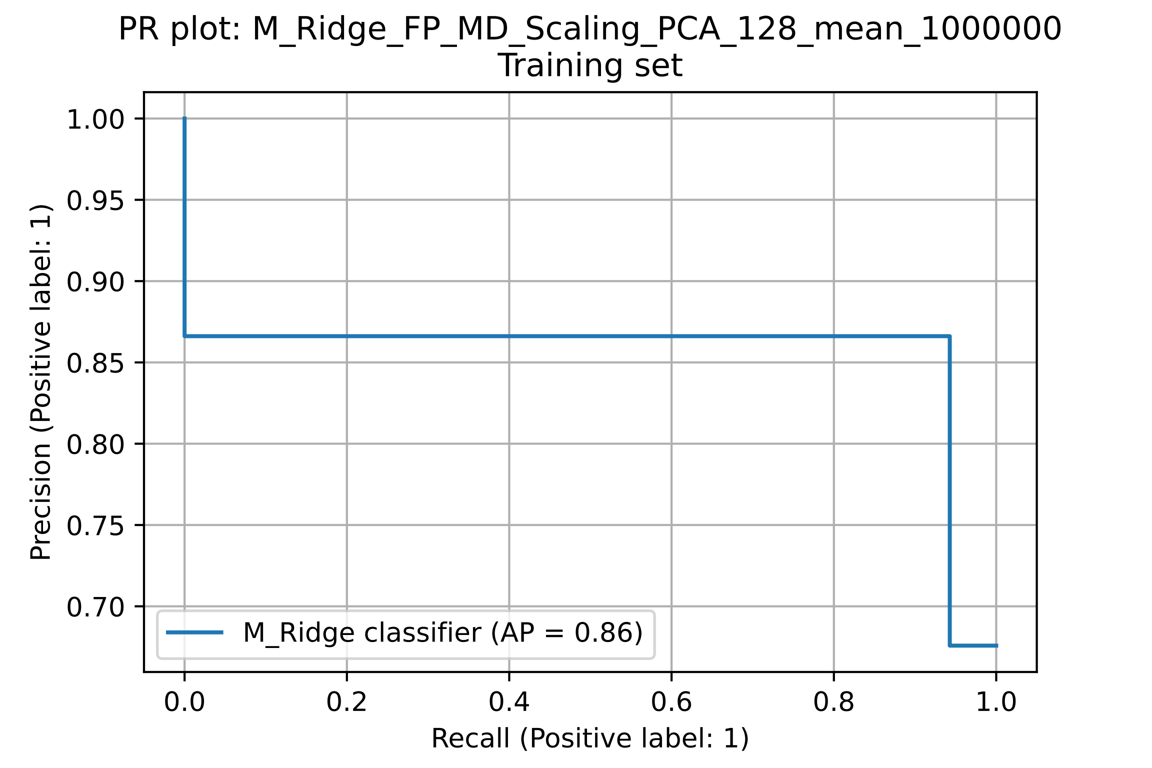
PR plot: M_Ridge_FP_MD_Scaling_PCA_128_mean_1000000
Training set
1.00
0.95
0.90
0.85
0.80
0.75
0.70
Precision (Positive label: 1)
0.0
0.2
0.4
0.6
0.8
1.0
Recall (Positive label: 1)
M_Ridge classifier (AP = 0.86)

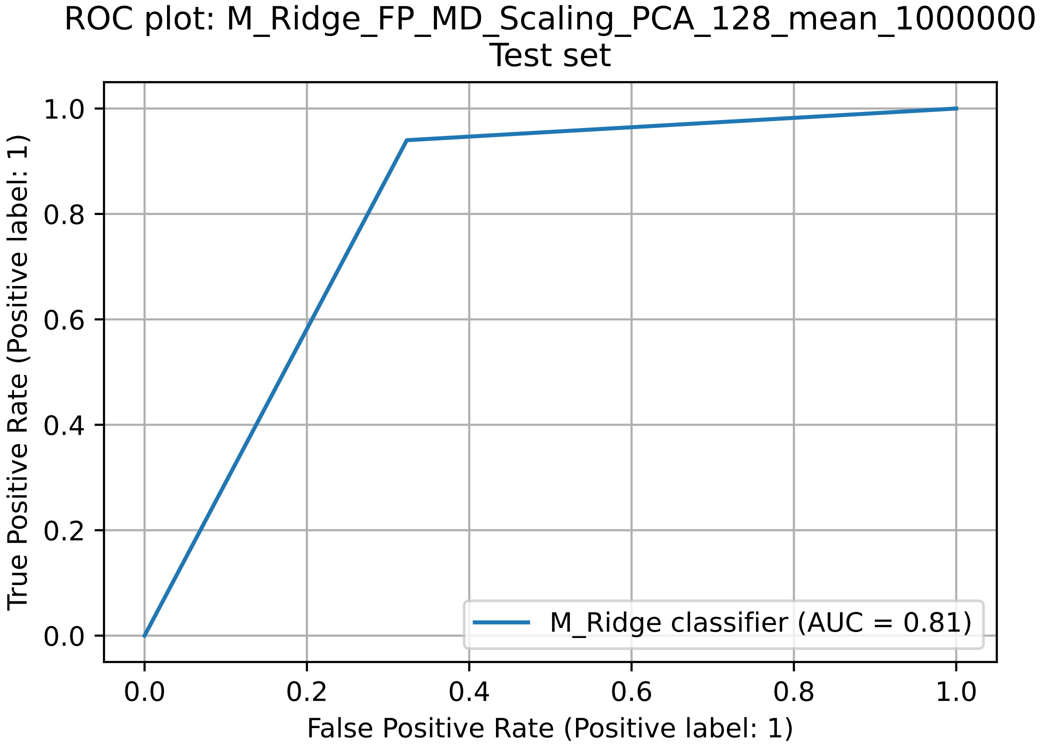
ROC plot: M_Ridge_FP_MD_Scaling_PCA_128_mean_1000000
Test set
True Positive Rate (Positive label: 1)
False Positive Rate (Positive label: 1)
M_Ridge classifier (AUC = 0.81)
0.0
0.2
0.4
0.6
0.8
1.0

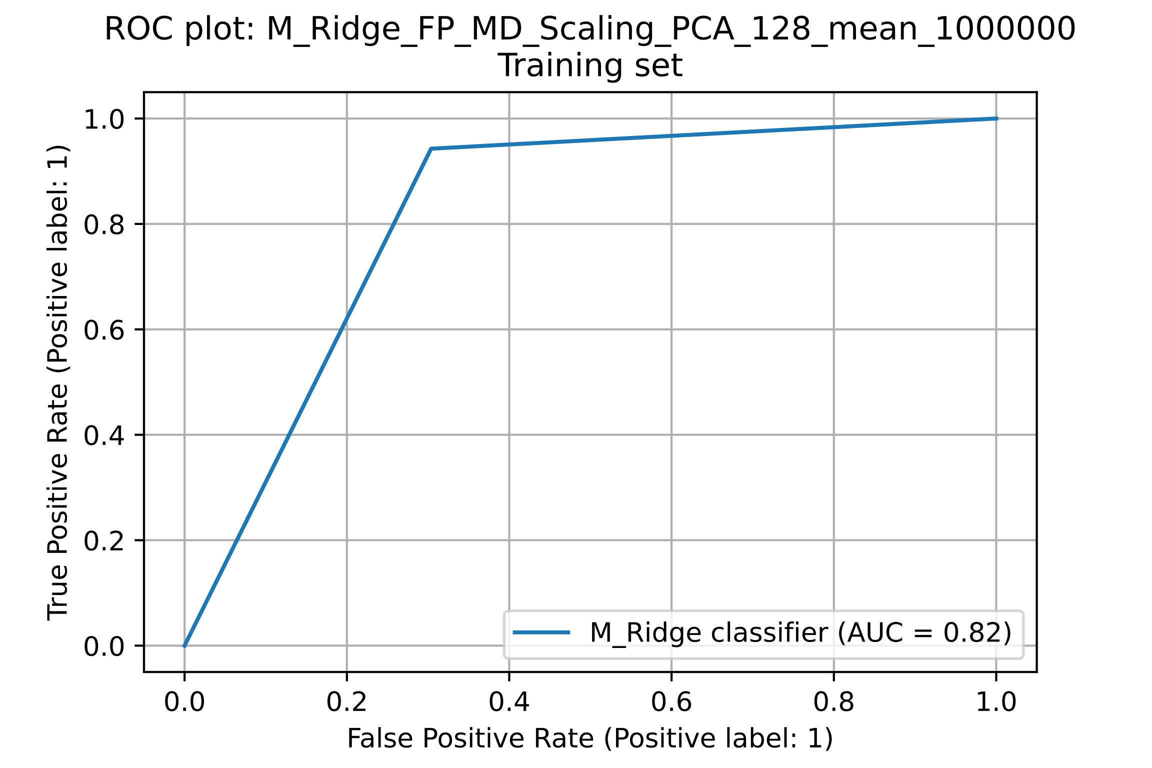

ROC plot: M_Ridge_FP_MD_Scaling_PCA_128_mean_1000000
Training set
1.0
0.8
0.6
0.4
0.2
0.0
True Positive Rate (Positive label: 1)
0.0
0.2
0.4
0.6
0.8
1.0
False Positive Rate (Positive label: 1)
M_Ridge classifier (AUC = 0.82)

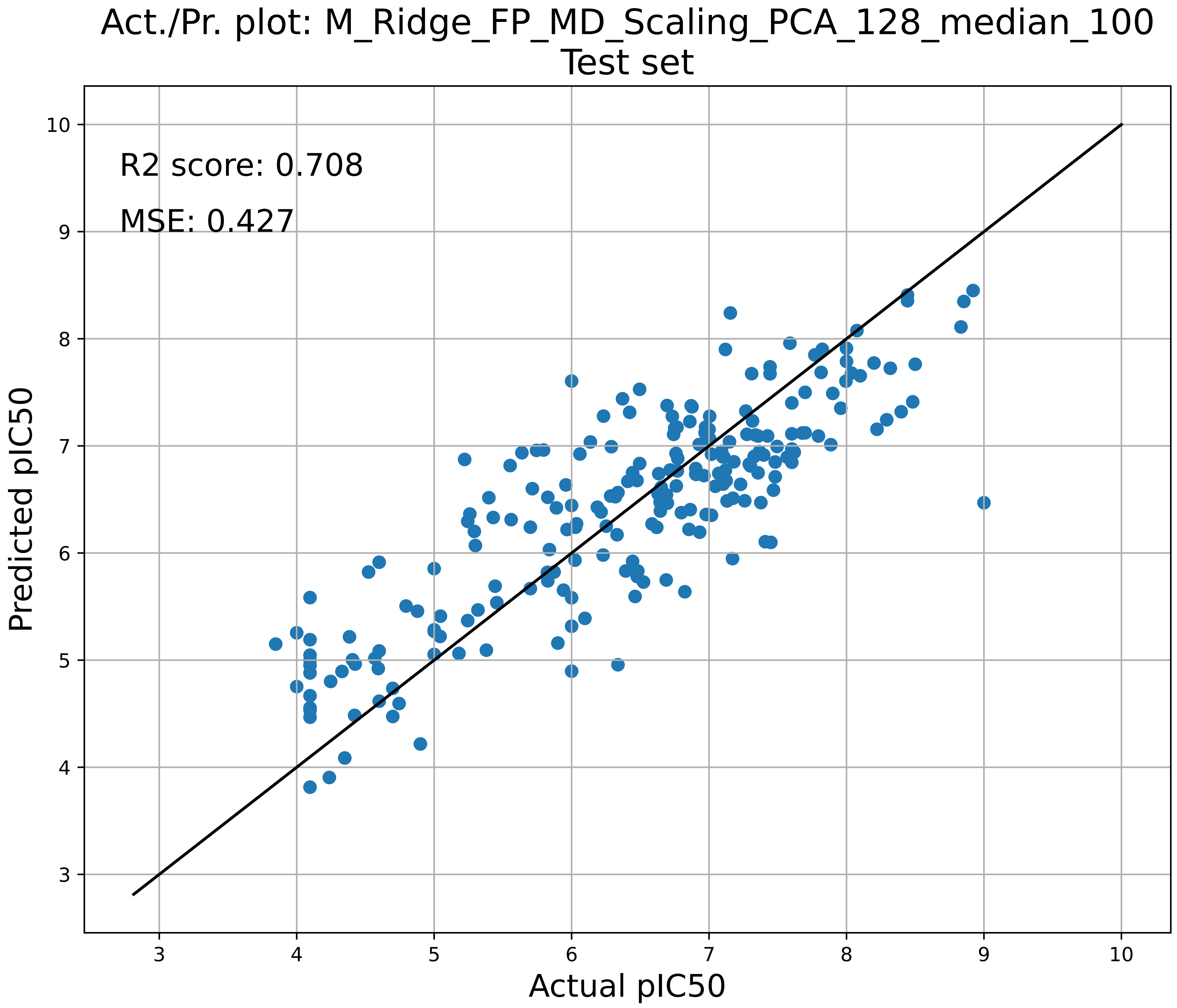

Act./Pr. plot: M_Ridge_FP_MD_Scaling_PCA_128_median_100
Test set
R2 score: 0.708
MSE: 0.427
Predicted pIC50
Actual pIC50
3
4
5
6
7
8
9
10

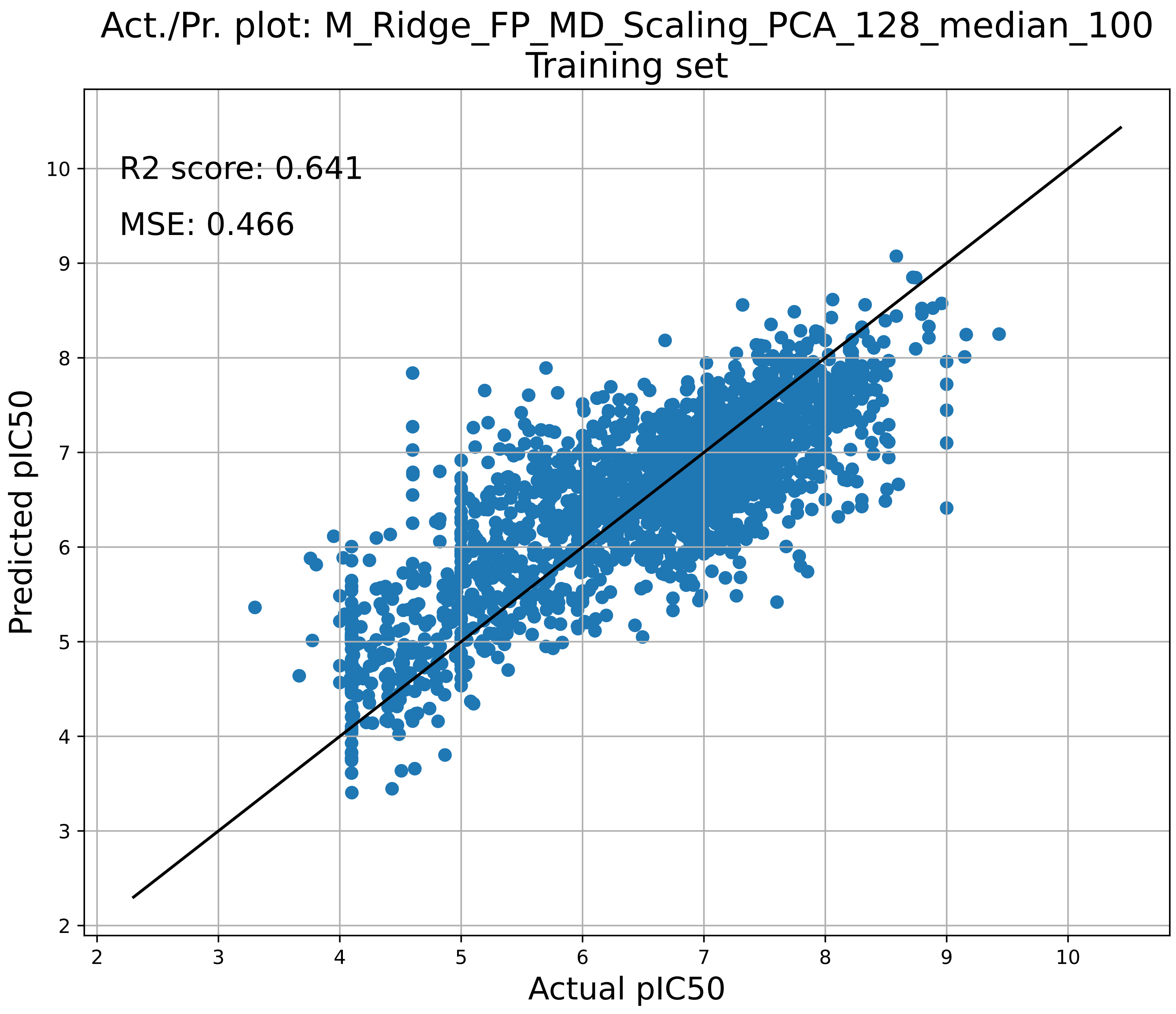

Act./Pr. plot: M_Ridge_FP_MD_Scaling_PCA_128_median_100
Training set
R2 score: 0.641
MSE: 0.466
Predicted pIC50
Actual pIC50
2
3
4
5
6
7
8
9
10

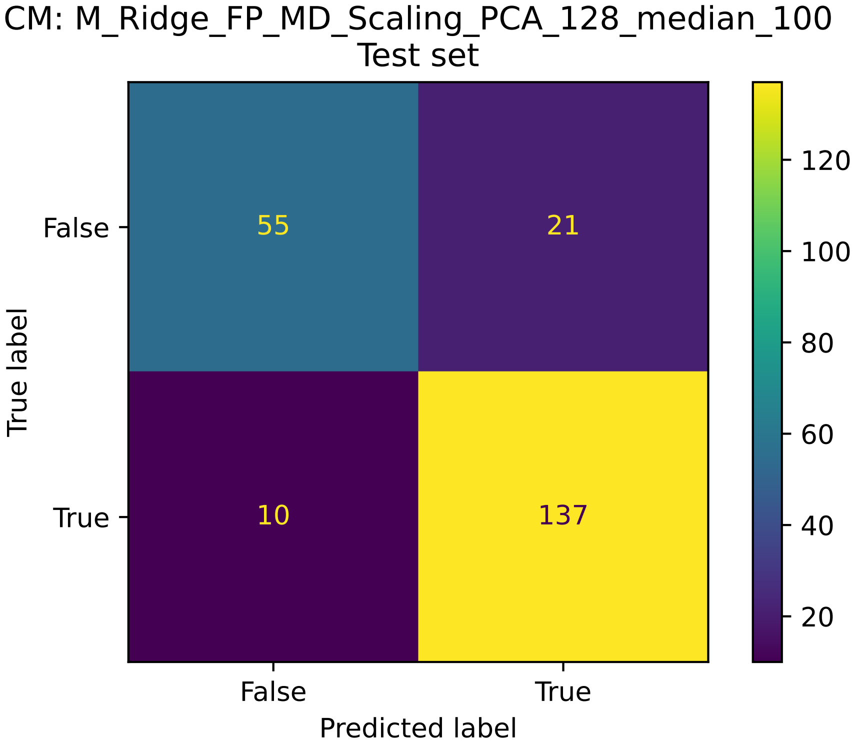
CM: M_Ridge_FP_MD_Scaling_PCA_128_median_100
Test set
55
21
10
137
False
True
True label
False
True
Predicted label
120
100
80
60
40
20

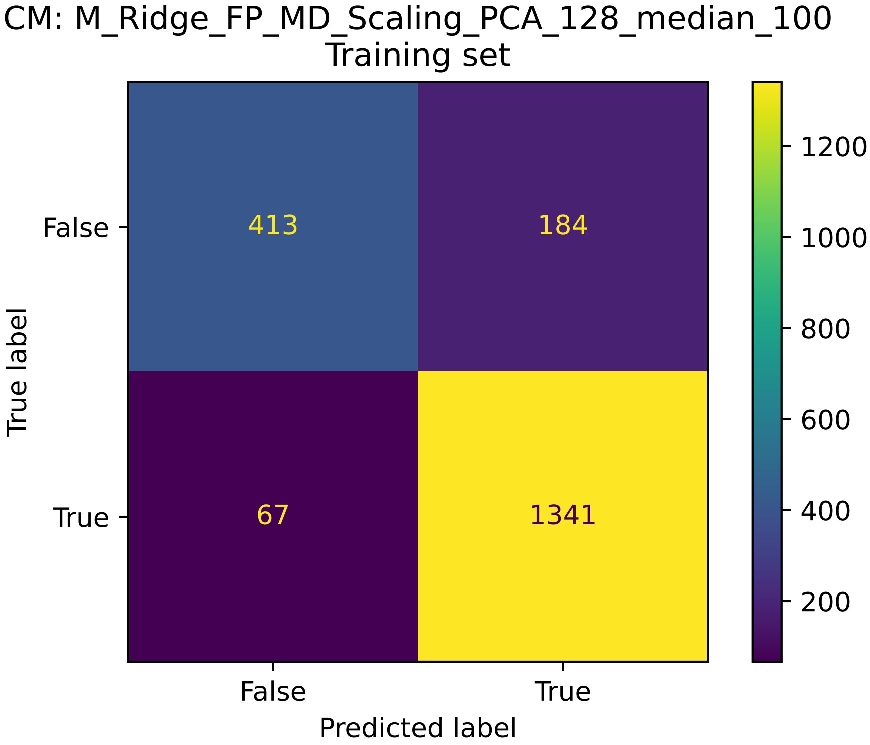
CM: M_Ridge_FP_MD_Scaling_PCA_128_median_100
Training set
413
184
67
1341
False
True
True label
False
True
Predicted label
200
400
600
800
1000
1200

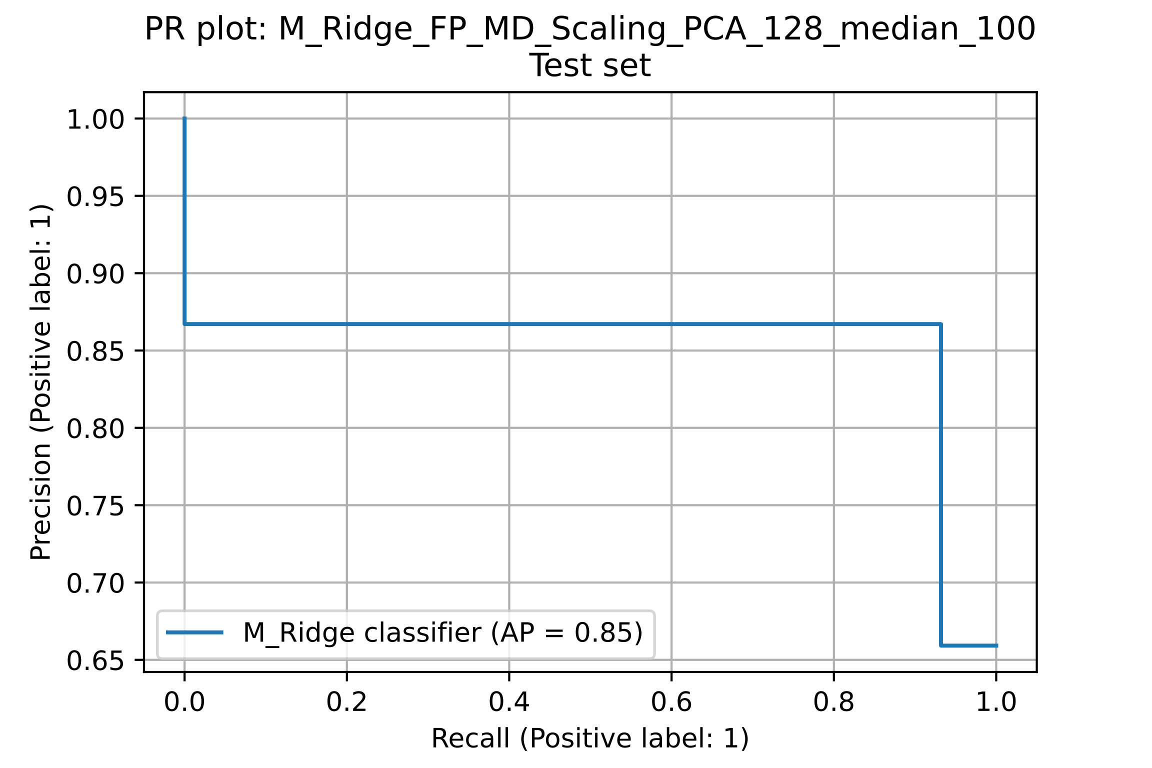

PR plot: M_Ridge_FP_MD_Scaling_PCA_128_median_100
Test set
Precision (Positive label: 1)
Recall (Positive label: 1)
1.00
0.95
0.90
0.85
0.80
0.75
0.70
0.65
0.0
0.2
0.4
0.6
0.8
1.0
M_Ridge classifier (AP = 0.85)

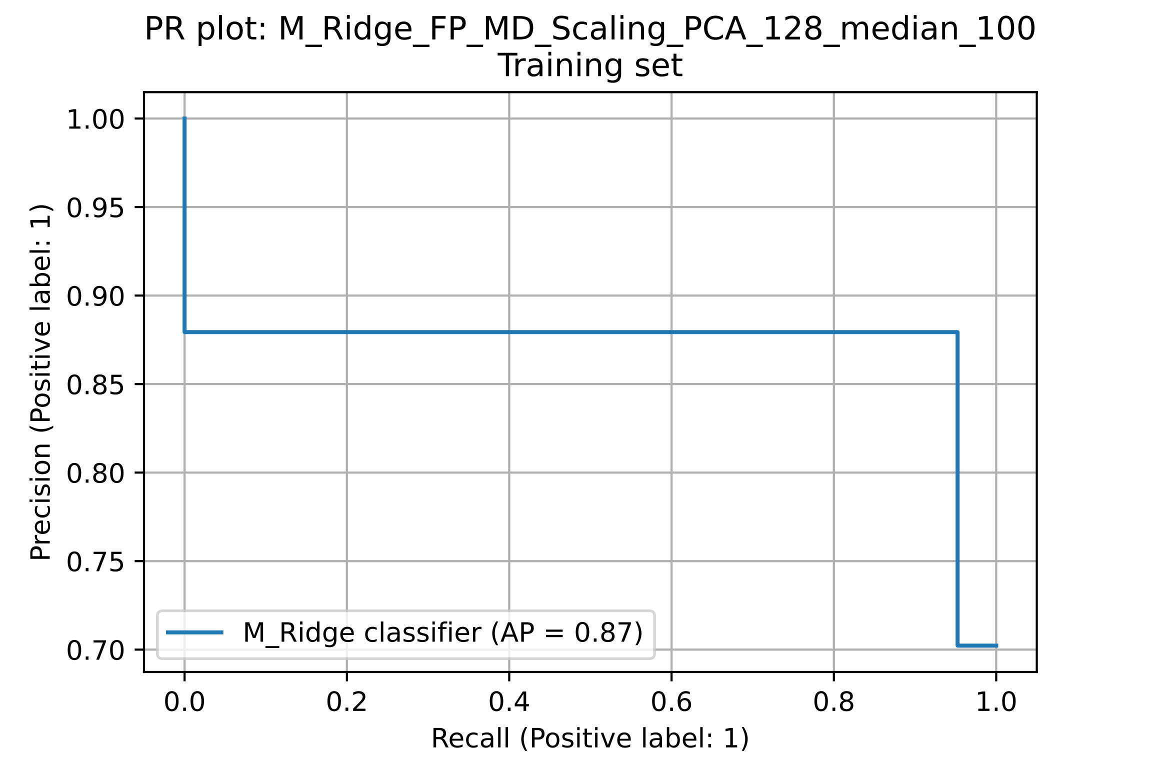

PR plot: M_Ridge_FP_MD_Scaling_PCA_128_median_100
Training set
Precision (Positive label: 1)
Recall (Positive label: 1)
1.00
0.95
0.90
0.85
0.80
0.75
0.70
0.0
0.2
0.4
0.6
0.8
1.0
M_Ridge classifier (AP = 0.87)

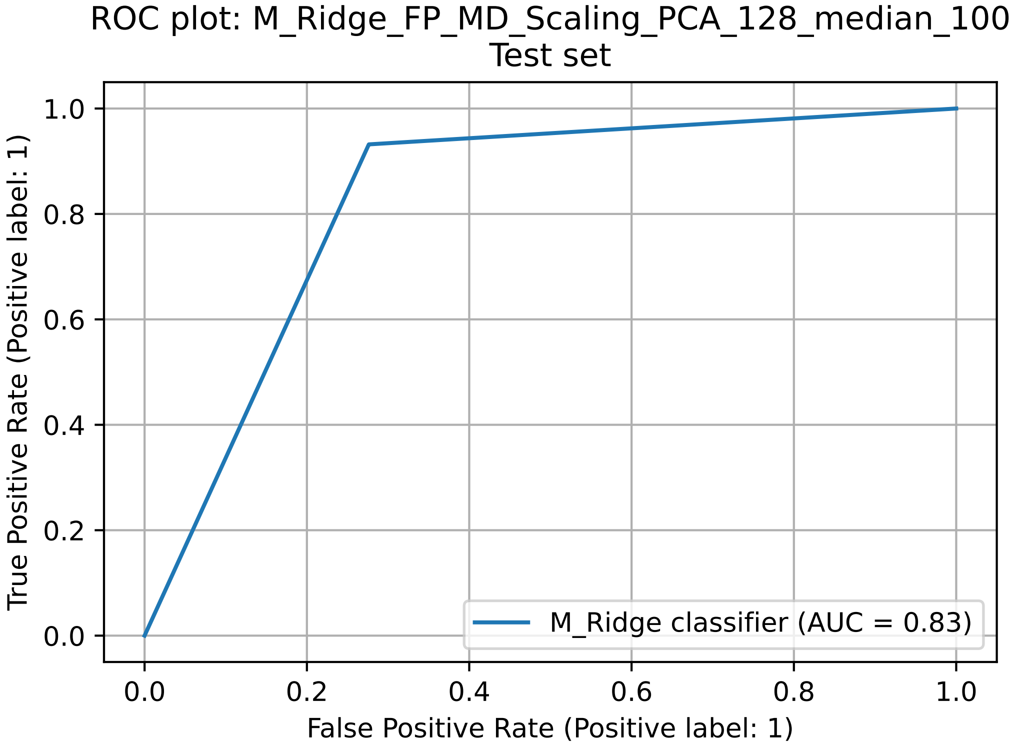

ROC plot: M_Ridge_FP_MD_Scaling_PCA_128_median_100
Test set
M_Ridge classifier (AUC = 0.83)
True Positive Rate (Positive label: 1)
False Positive Rate (Positive label: 1)
0.0
0.2
0.4
0.6
0.8
1.0

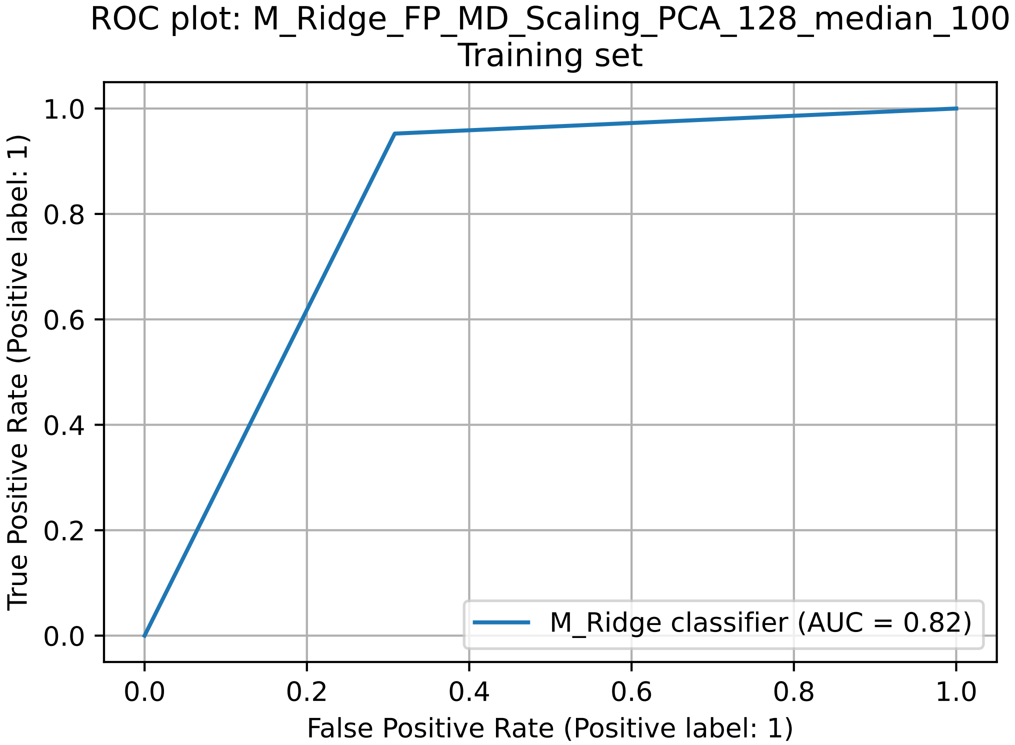
ROC plot: M_Ridge_FP_MD_Scaling_PCA_128_median_100
Training set
True Positive Rate (Positive label: 1)
False Positive Rate (Positive label: 1)
0.0
0.2
0.4
0.6
0.8
1.0
M_Ridge classifier (AUC = 0.82)

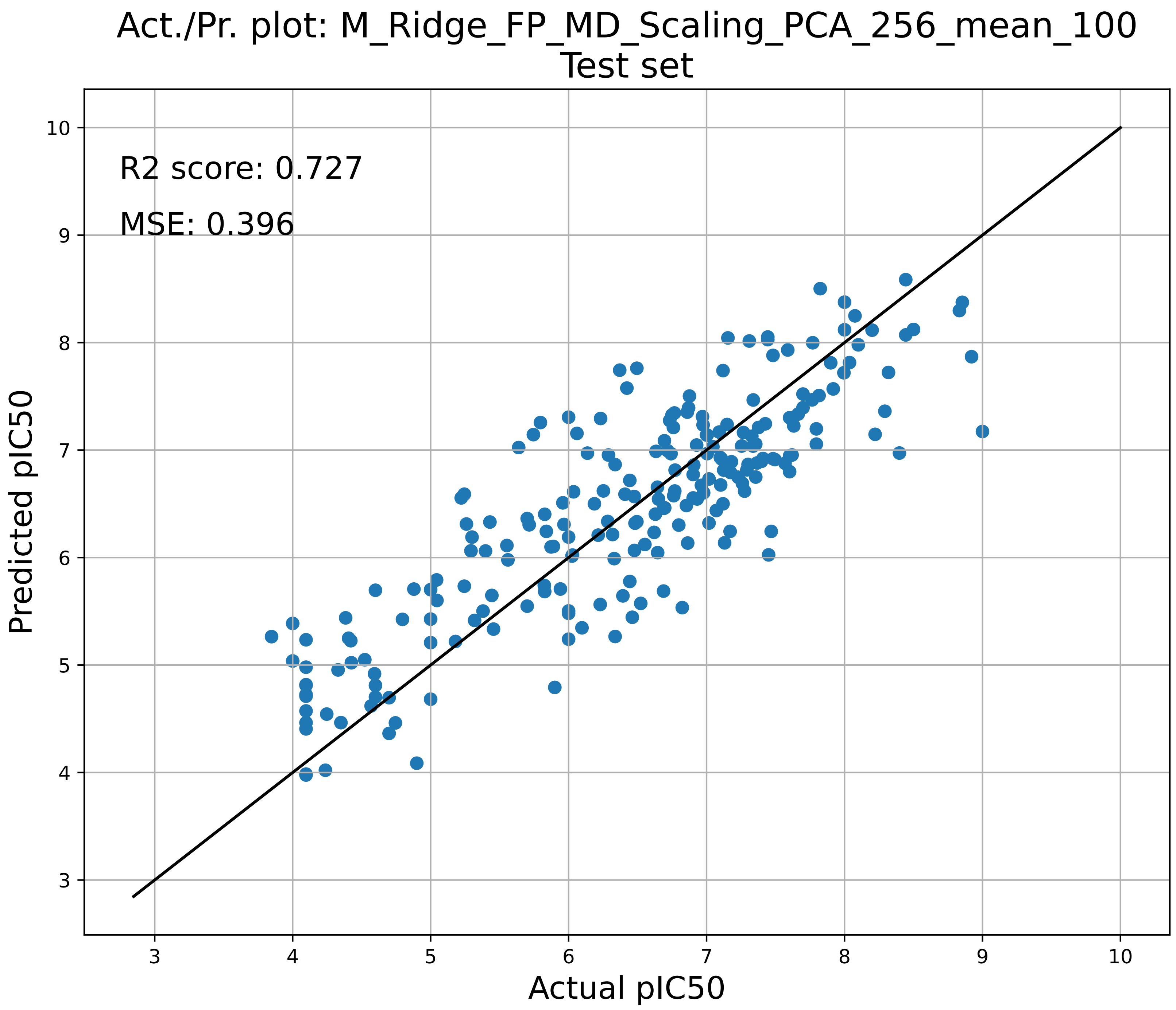
Act./Pr. plot: M_Ridge_FP_MD_Scaling_PCA_256_mean_100
Test set
R2 score: 0.727
MSE: 0.396
Predicted pIC50
Actual pIC50
3
4
5
6
7
8
9
10

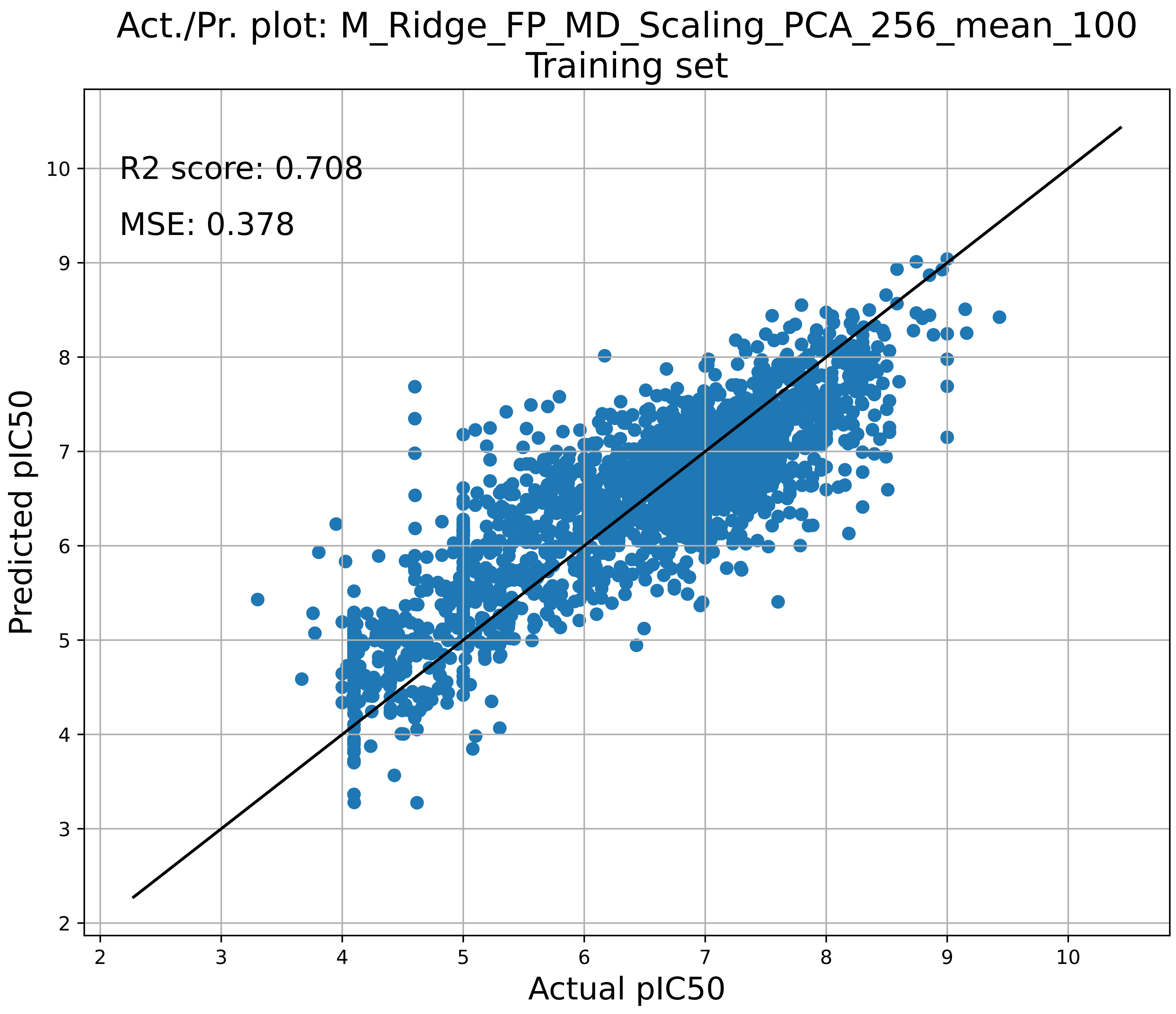

Act./Pr. plot: M_Ridge_FP_MD_Scaling_PCA_256_mean_100
Training set
R2 score: 0.708
MSE: 0.378
Predicted pIC50
Actual pIC50
2
3
4
5
6
7
8
9
10

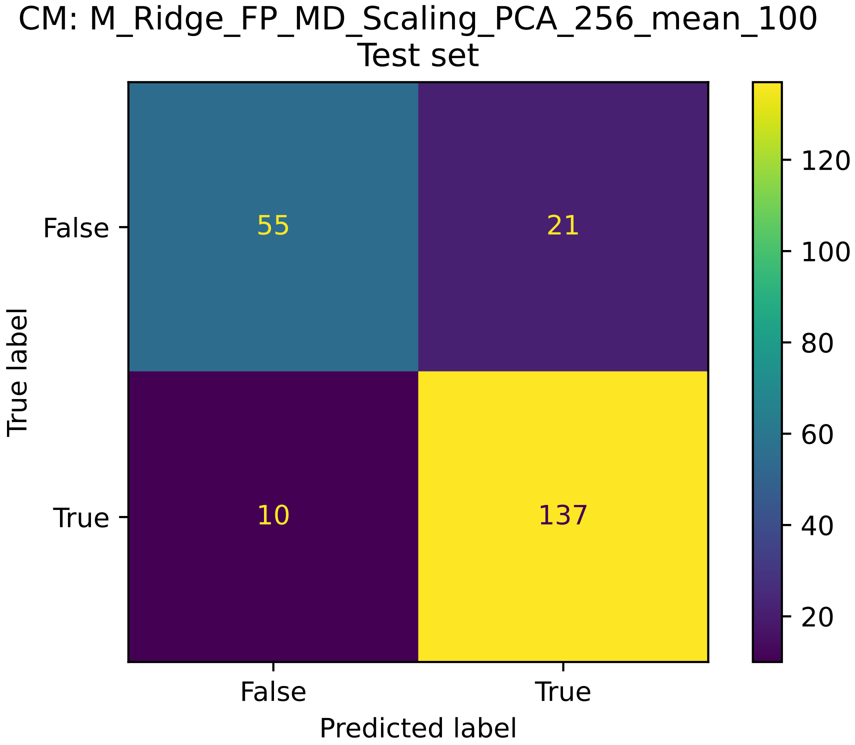

CM: M_Ridge_FP_MD_Scaling_PCA_256_mean_100
Test set
True label
False
True
55
21
10
137
Predicted label
False
True
20
40
60
80
100
120

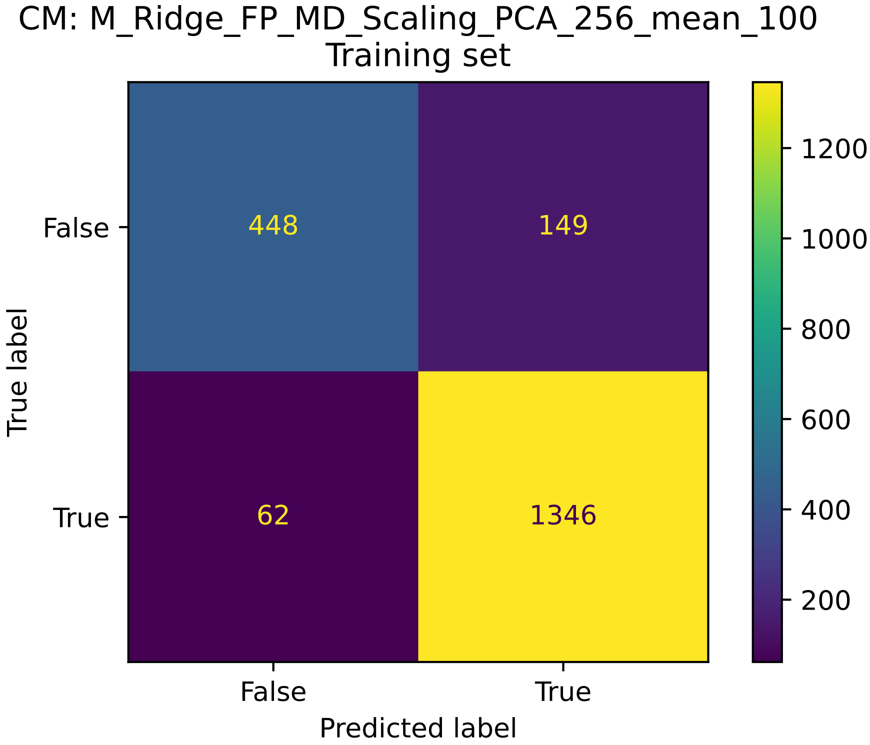

CM: M_Ridge_FP_MD_Scaling_PCA_256_mean_100
Training set
True label
False
True
448
149
62
1346
Predicted label
False
True
200
400
600
800
1000
1200

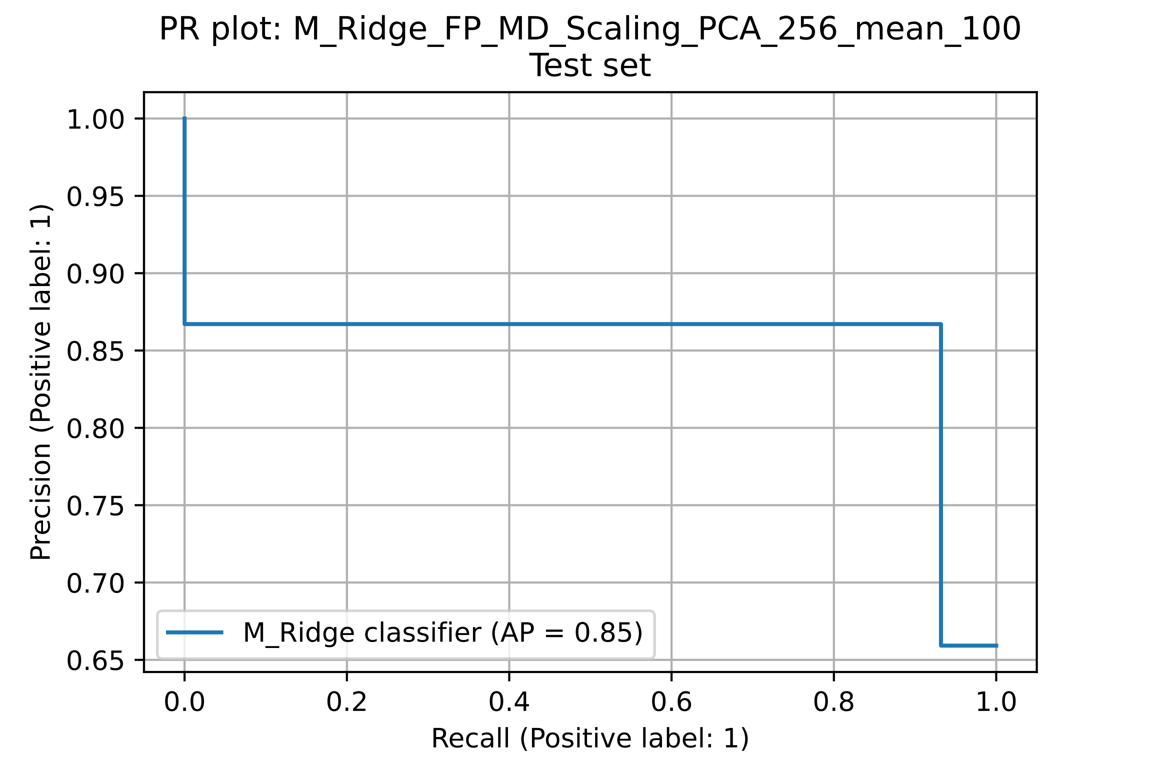

PR plot: M_Ridge_FP_MD_Scaling_PCA_256_mean_100
Test set
1.00
0.95
0.90
0.85
0.80
0.75
0.70
0.65
Precision (Positive label: 1)
0.0
0.2
0.4
0.6
0.8
1.0
Recall (Positive label: 1)
M_Ridge classifier (AP = 0.85)

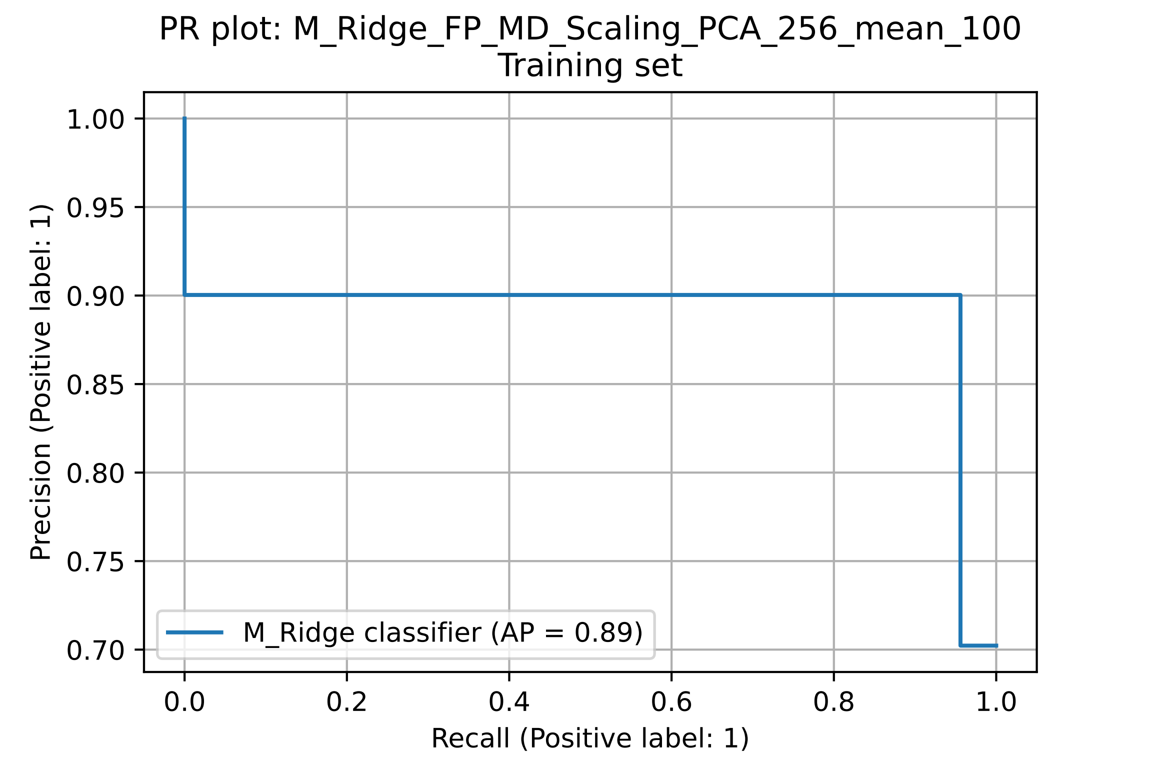

PR plot: M_Ridge_FP_MD_Scaling_PCA_256_mean_100
Training set
1.00
0.95
0.90
0.85
0.80
0.75
0.70
Precision (Positive label: 1)
0.0
0.2
0.4
0.6
0.8
1.0
Recall (Positive label: 1)
M_Ridge classifier (AP = 0.89)

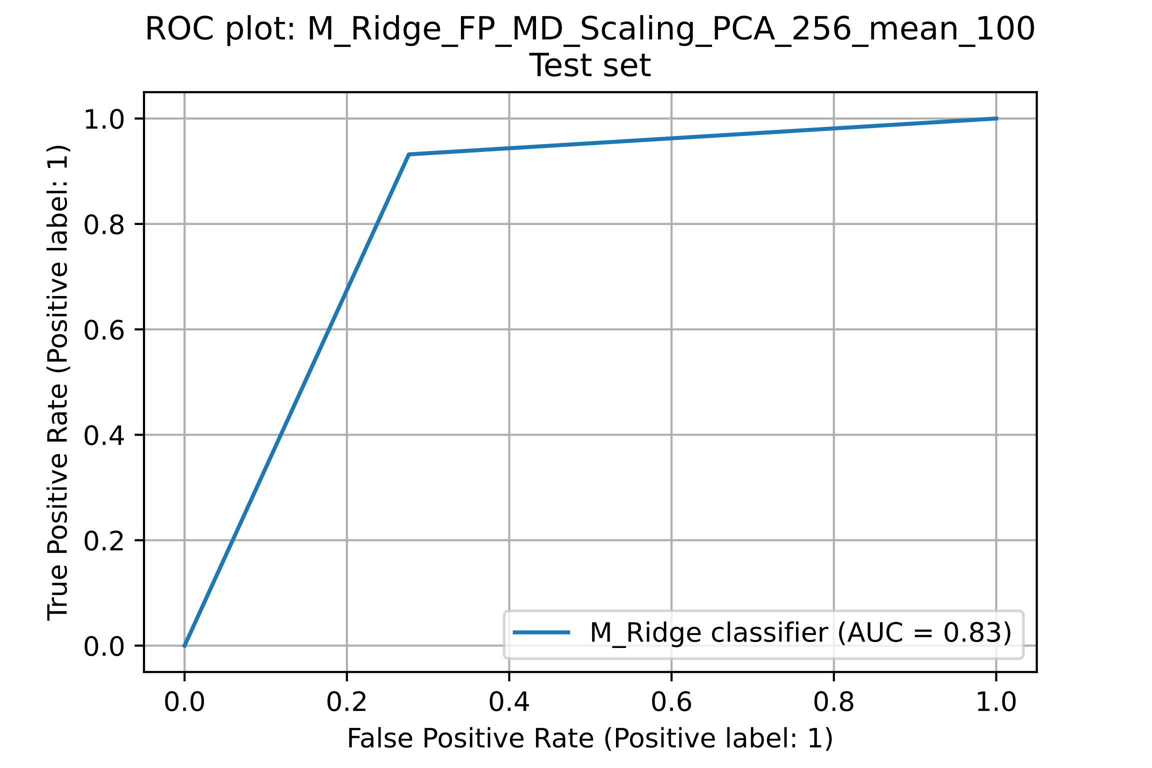

ROC plot: M_Ridge_FP_MD_Scaling_PCA_256_mean_100
Test set
True Positive Rate (Positive label: 1)
False Positive Rate (Positive label: 1)
0.0
0.2
0.4
0.6
0.8
1.0
M_Ridge classifier (AUC = 0.83)

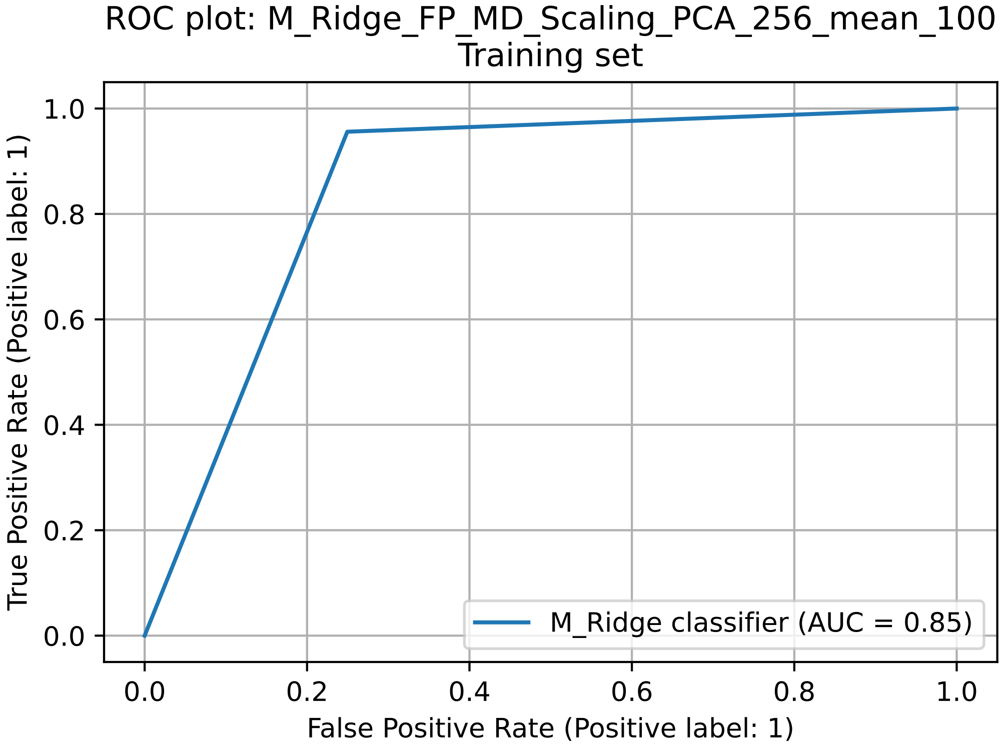
ROC plot: M_Ridge_FP_MD_Scaling_PCA_256_mean_100
Training set
True Positive Rate (Positive label: 1)
False Positive Rate (Positive label: 1)
0.0
0.2
0.4
0.6
0.8
1.0
M_Ridge classifier (AUC = 0.85)

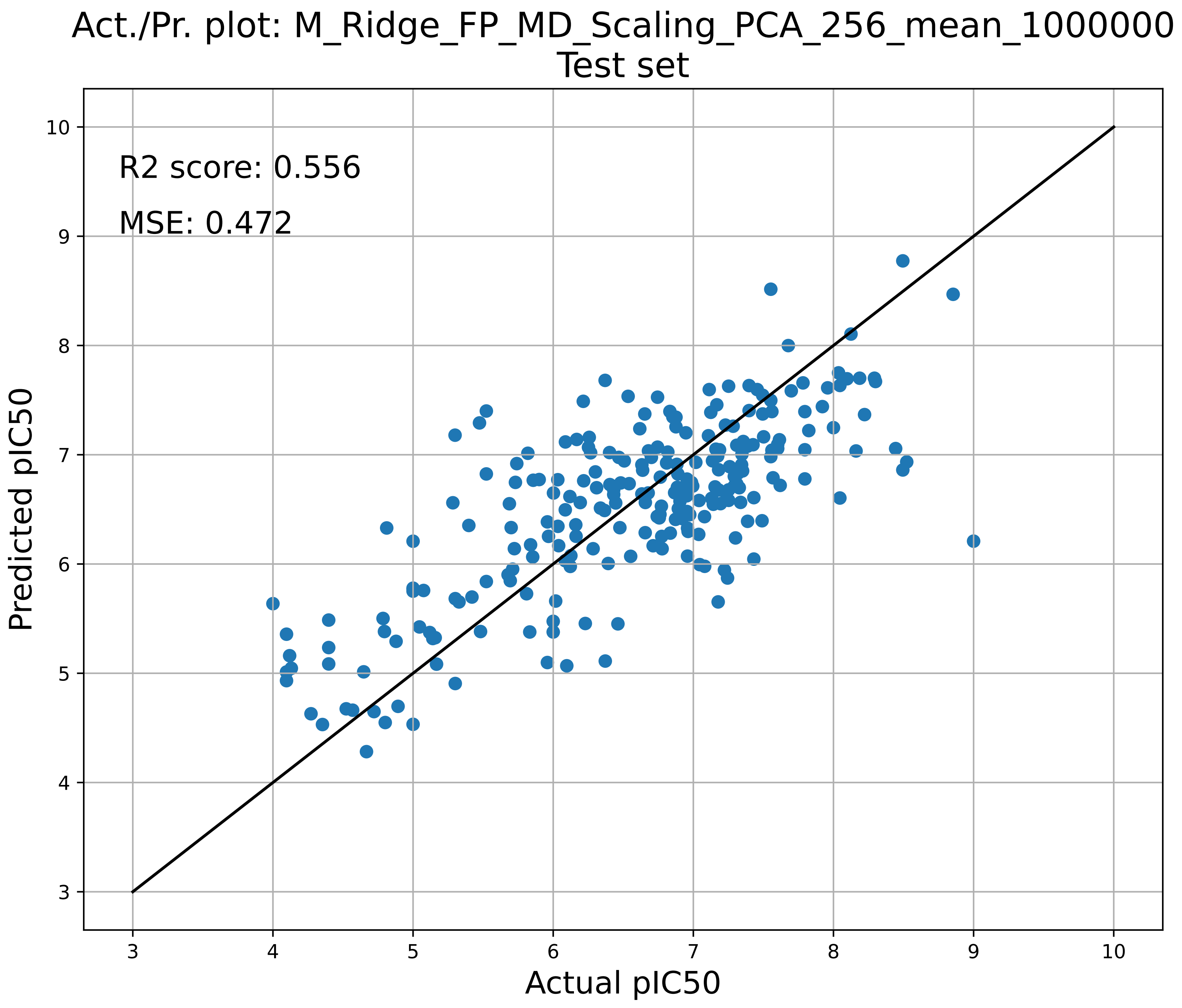

Act./Pr. plot: M_Ridge_FP_MD_Scaling_PCA_256_mean_1000000
Test set
R2 score: 0.556
MSE: 0.472
Predicted pIC50
Actual pIC50
3
4
5
6
7
8
9
10

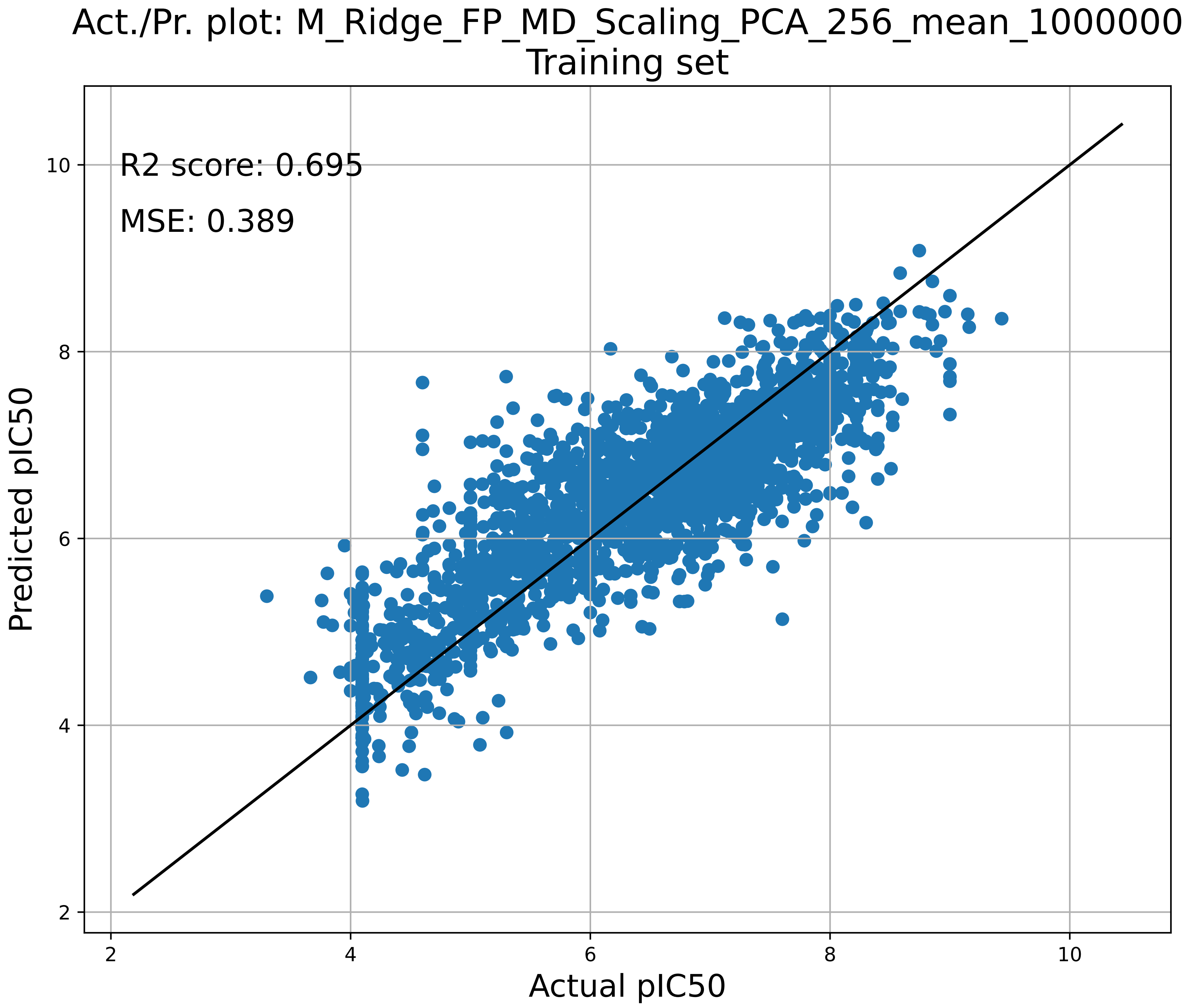

Act./Pr. plot: M_Ridge_FP_MD_Scaling_PCA_256_mean_1000000
Training set
R2 score: 0.695
MSE: 0.389
Predicted pIC50
Actual pIC50
2
4
6
8
10

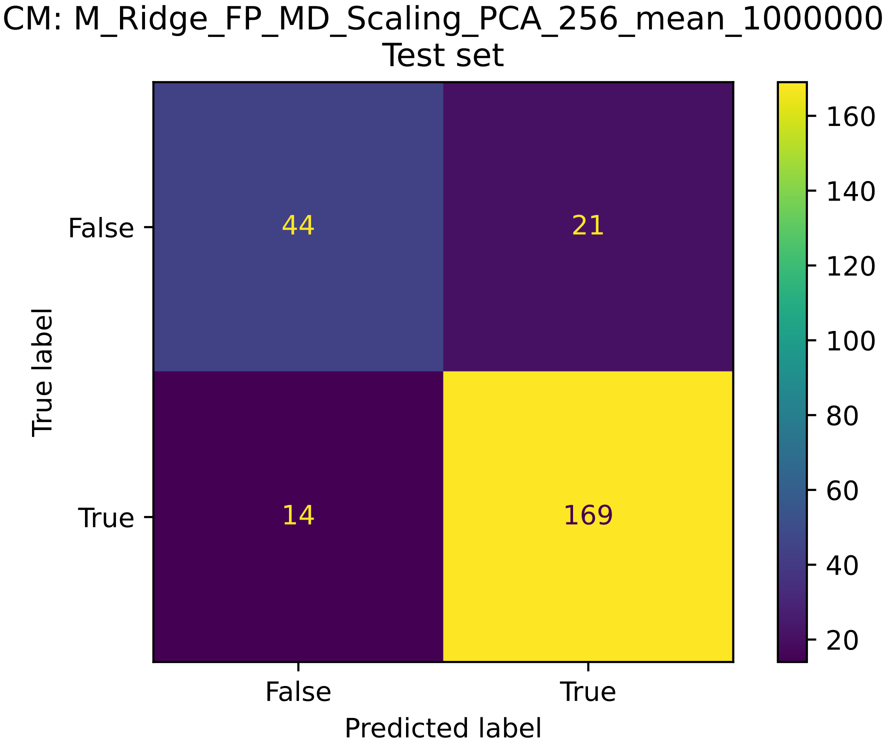
CM: M_Ridge_FP_MD_Scaling_PCA_256_mean_1000000
Test set
True label
False
True
44
21
14
169
Predicted label
False
True
160
140
120
100
80
60
40
20

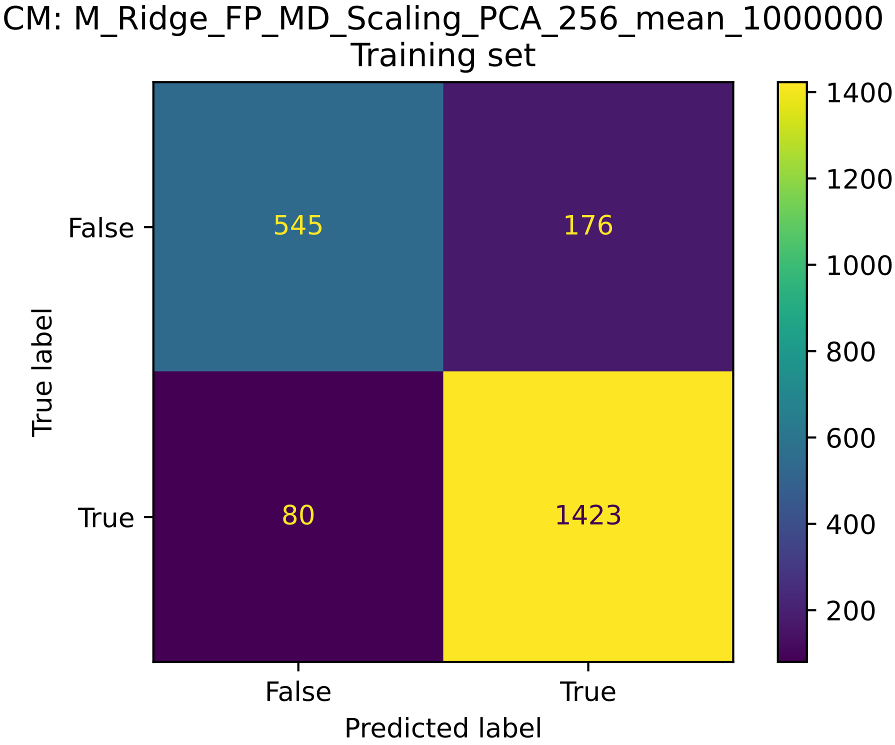
CM: M_Ridge_FP_MD_Scaling_PCA_256_mean_1000000
Training set
545
176
80
1423
False
True
True label
False
True
Predicted label
1400
1200
1000
800
600
400
200

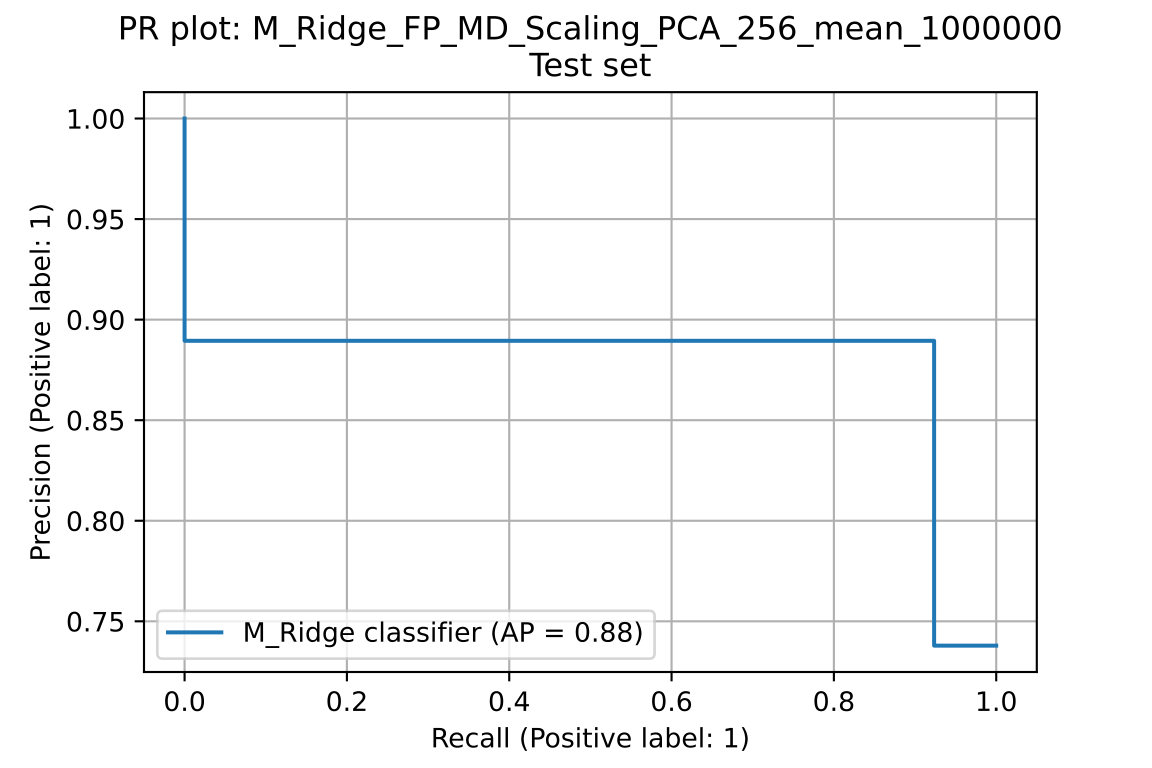

PR plot: M_Ridge_FP_MD_Scaling_PCA_256_mean_1000000
Test set
Precision (Positive label: 1)
Recall (Positive label: 1)
1.00
0.95
0.90
0.85
0.80
0.75
0.0
0.2
0.4
0.6
0.8
1.0
M_Ridge classifier (AP = 0.88)

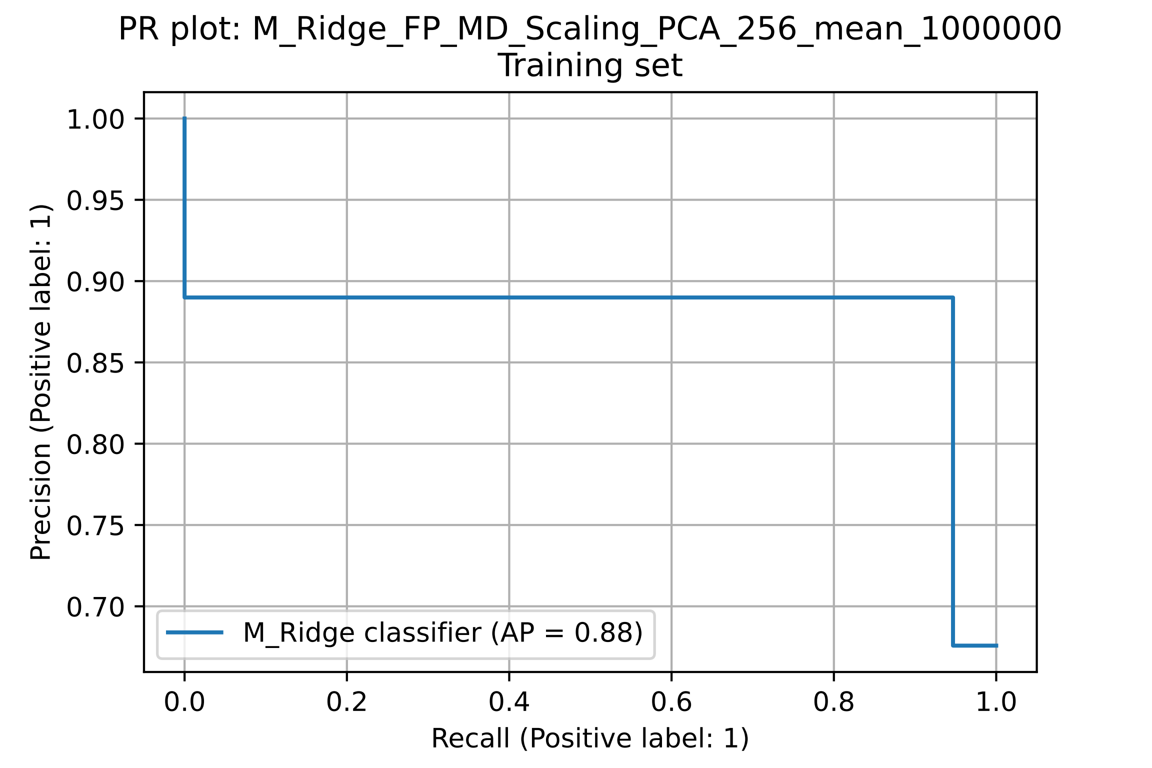

PR plot: M_Ridge_FP_MD_Scaling_PCA_256_mean_1000000
Training set
1.00
0.95
0.90
0.85
0.80
0.75
0.70
Precision (Positive label: 1)
0.0
0.2
0.4
0.6
0.8
1.0
Recall (Positive label: 1)
M_Ridge classifier (AP = 0.88)

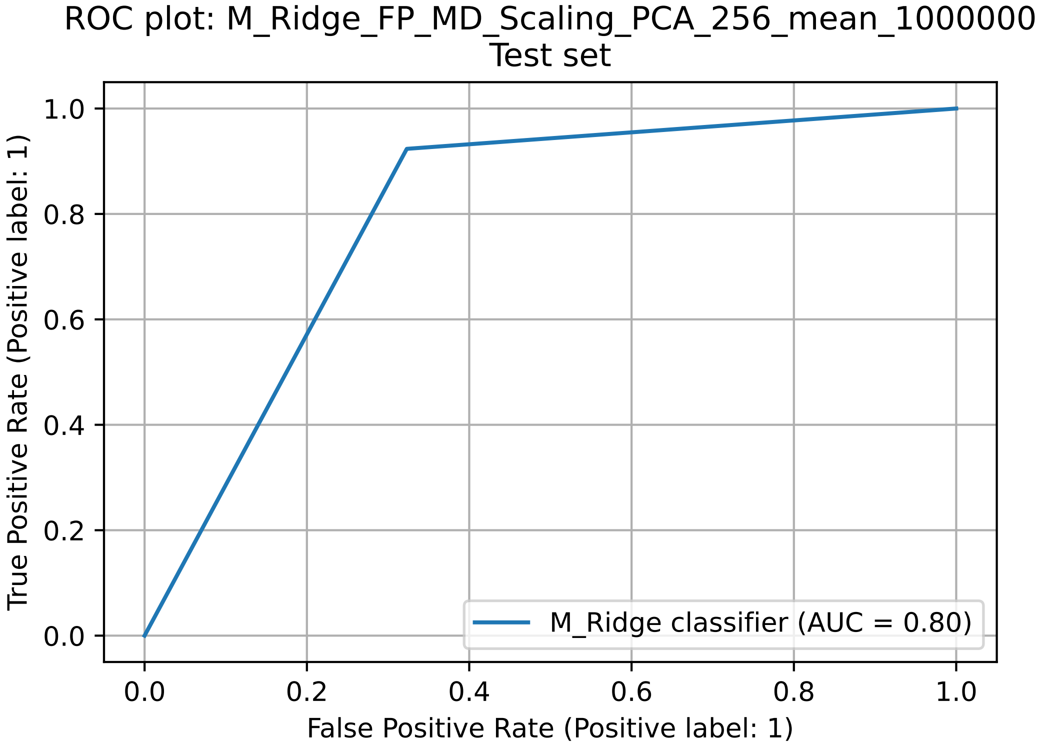

ROC plot: M_Ridge_FP_MD_Scaling_PCA_256_mean_1000000
Test set
True Positive Rate (Positive label: 1)
False Positive Rate (Positive label: 1)
M_Ridge classifier (AUC = 0.80)
0.0
0.2
0.4
0.6
0.8
1.0

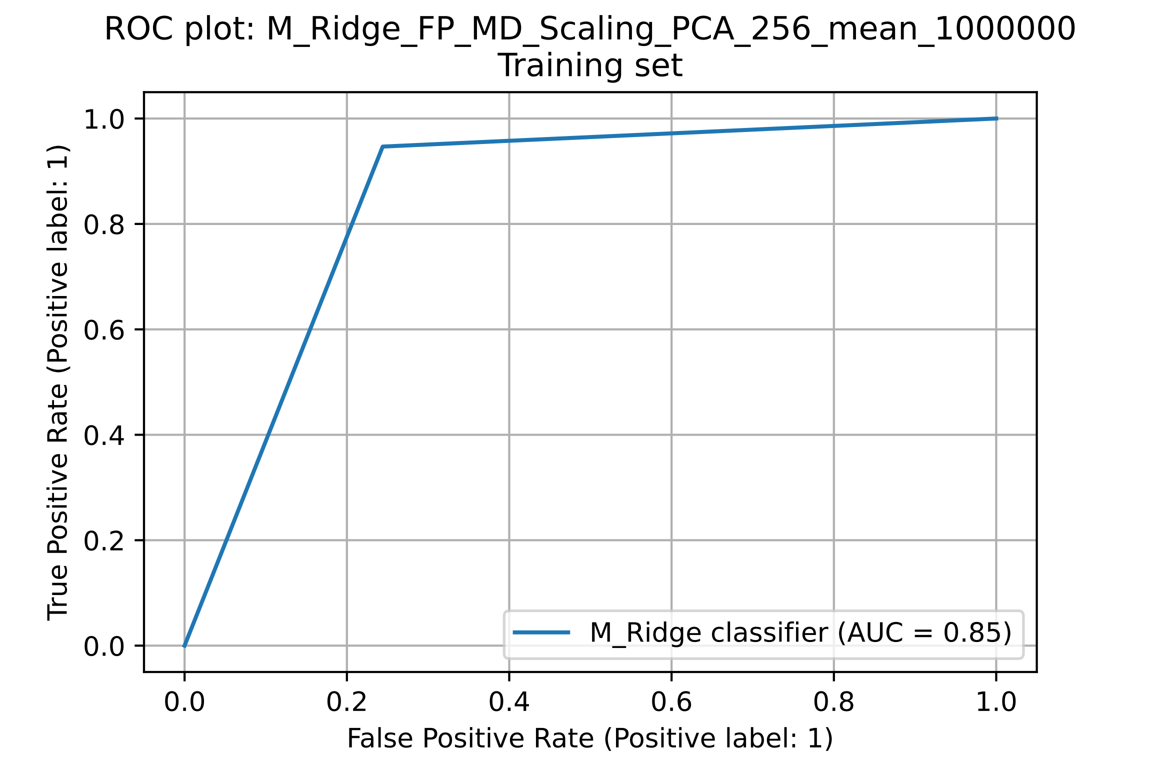
ROC plot: M_Ridge_FP_MD_Scaling_PCA_256_mean_1000000
Training set
True Positive Rate (Positive label: 1)
False Positive Rate (Positive label: 1)
0.0
0.2
0.4
0.6
0.8
1.0
M_Ridge classifier (AUC = 0.85)

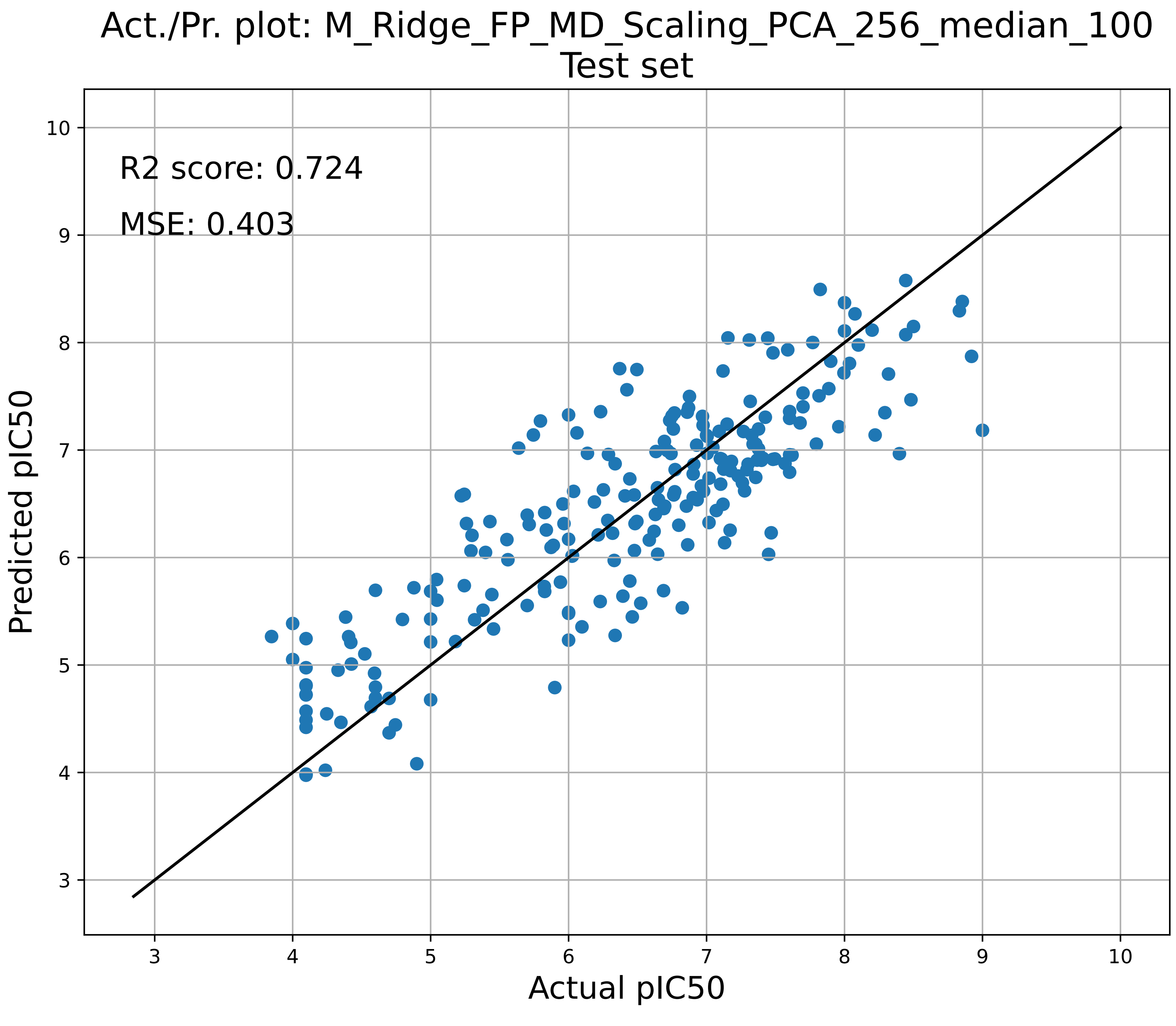
Act./Pr. plot: M_Ridge_FP_MD_Scaling_PCA_256_median_100
Test set
R2 score: 0.724
MSE: 0.403
Predicted pIC50
Actual pIC50
3
4
5
6
7
8
9
10

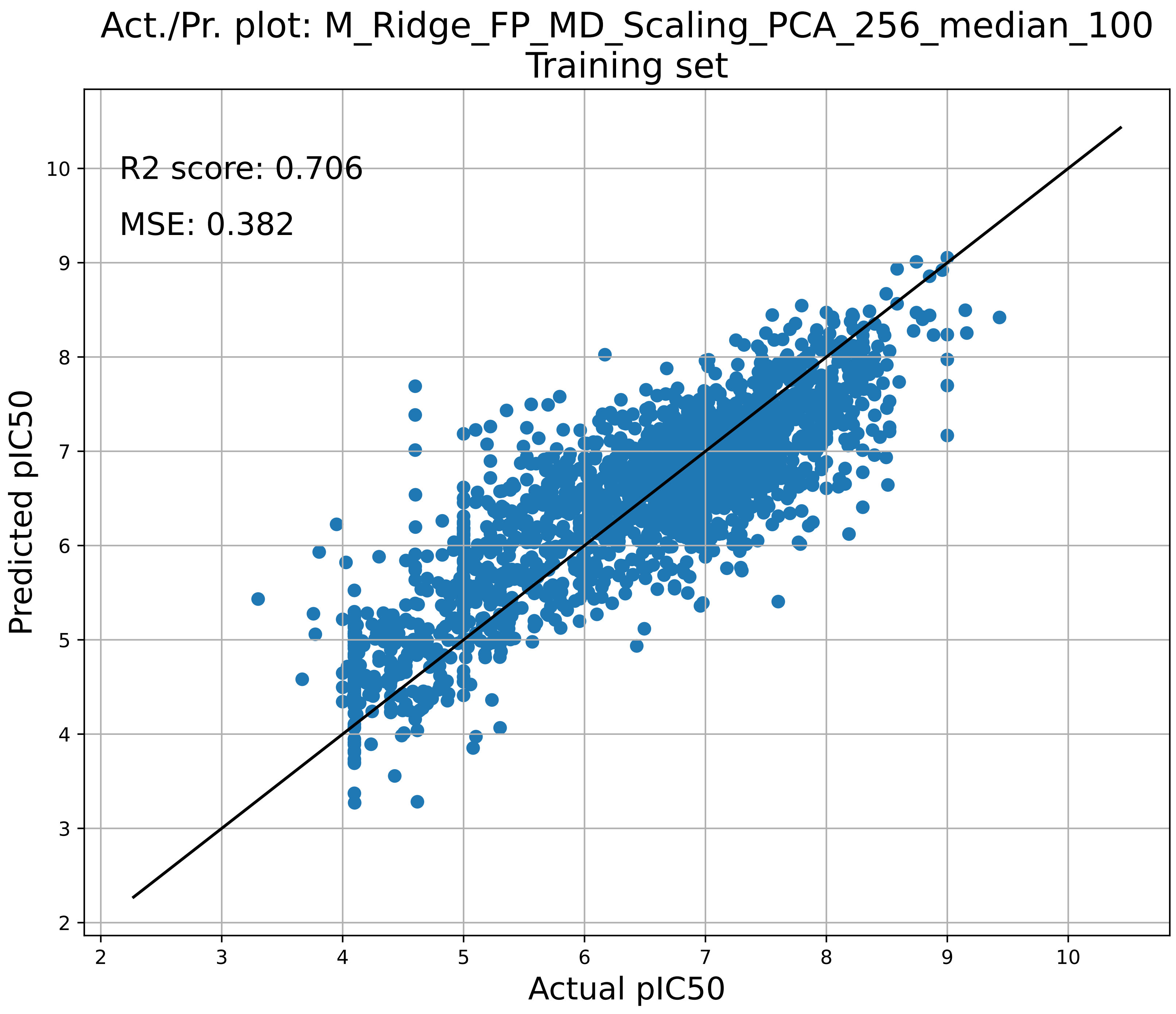

Act./Pr. plot: M_Ridge_FP_MD_Scaling_PCA_256_median_100
Training set
R2 score: 0.706
MSE: 0.382
Predicted pIC50
Actual pIC50
2
3
4
5
6
7
8
9
10

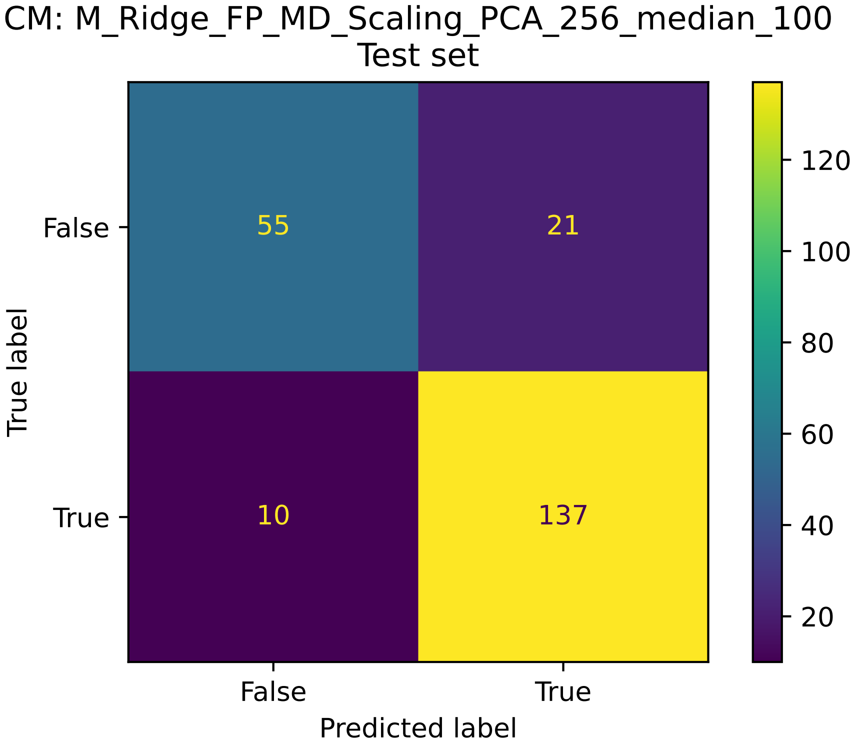
CM: M_Ridge_FP_MD_Scaling_PCA_256_median_100
Test set
True label
False
True
55
21
10
137
Predicted label
False
True
120
100
80
60
40
20

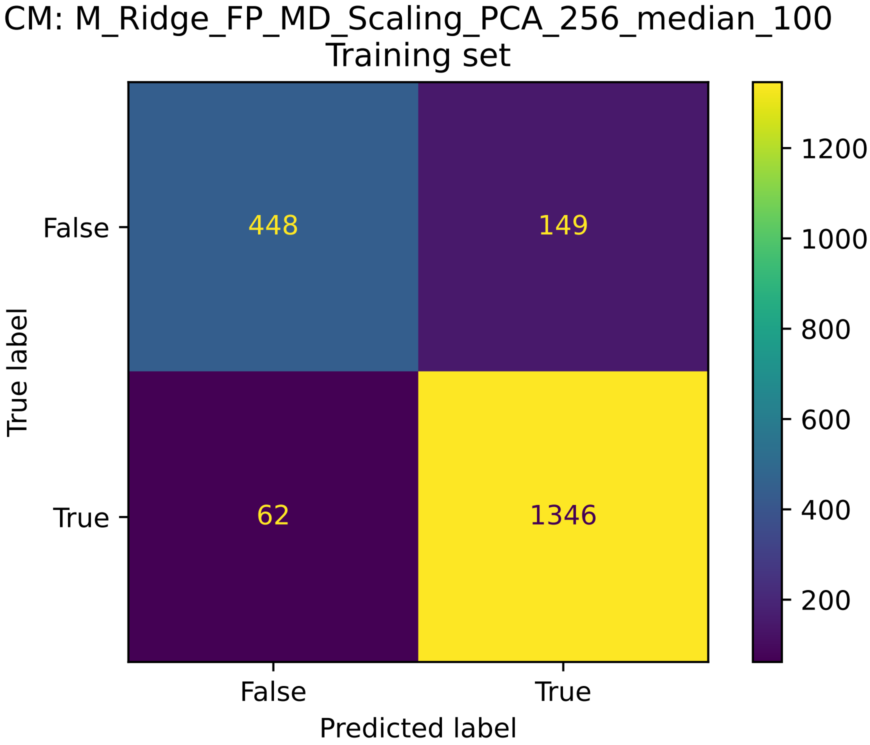
CM: M_Ridge_FP_MD_Scaling_PCA_256_median_100
Training set
True label
False
True
448
149
62
1346
Predicted label
False
True
1200
1000
800
600
400
200

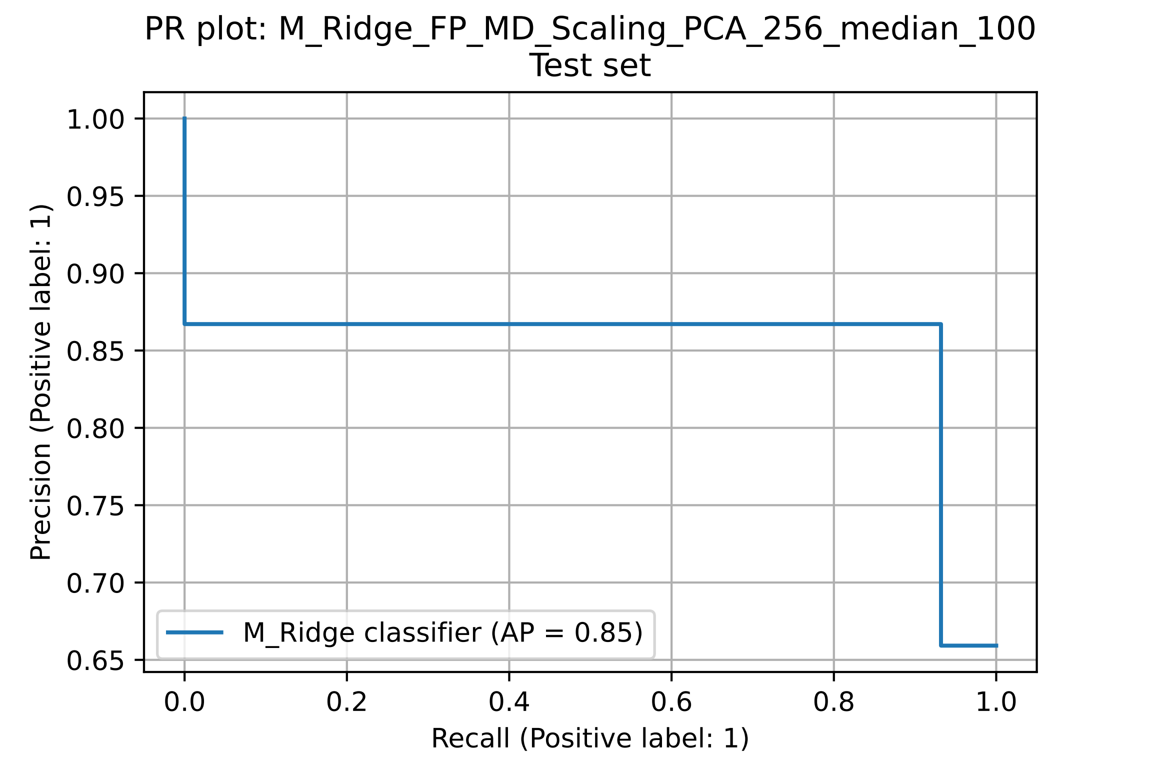
PR plot: M_Ridge_FP_MD_Scaling_PCA_256_median_100
Test set
1.00
0.95
0.90
0.85
0.80
0.75
0.70
0.65
Precision (Positive label: 1)
0.0
0.2
0.4
0.6
0.8
1.0
Recall (Positive label: 1)
M_Ridge classifier (AP = 0.85)

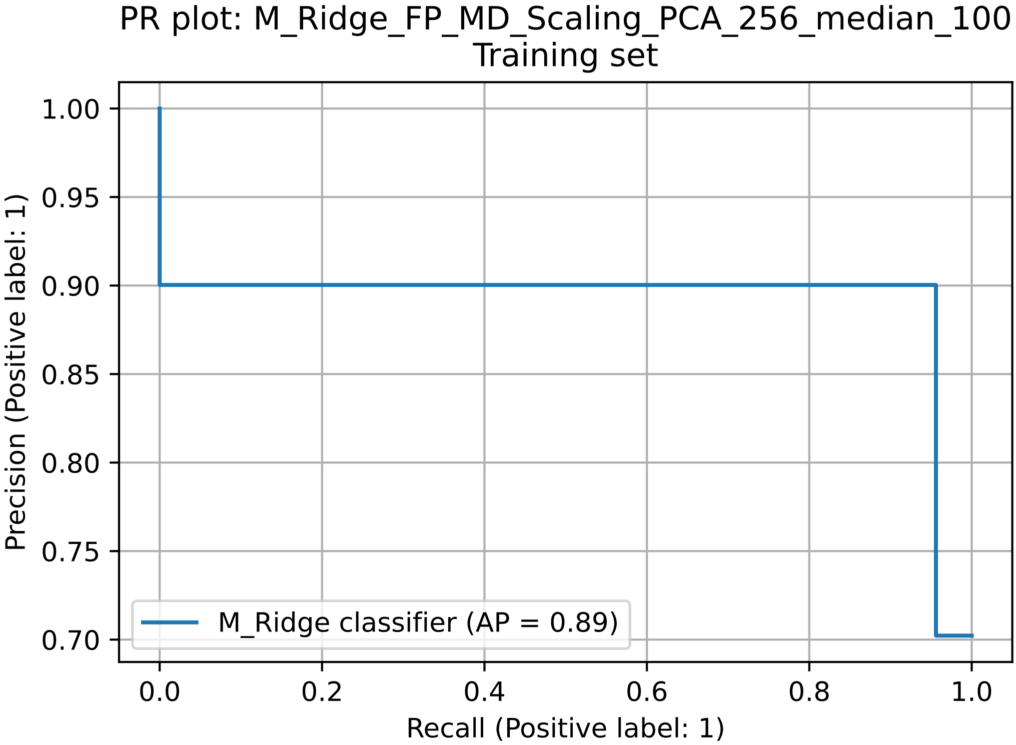
PR plot: M_Ridge_FP_MD_Scaling_PCA_256_median_100
Training set
1.00
0.95
0.90
0.85
0.80
0.75
0.70
Precision (Positive label: 1)
0.0
0.2
0.4
0.6
0.8
1.0
Recall (Positive label: 1)
M_Ridge classifier (AP = 0.89)

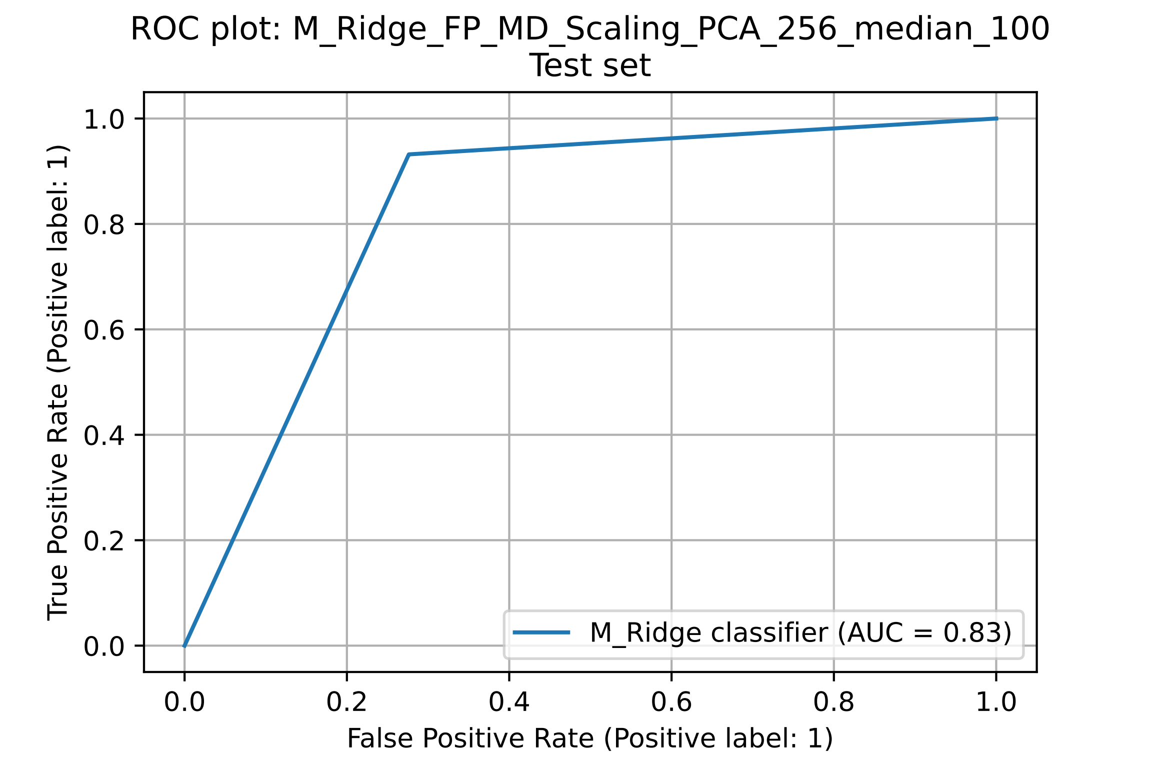

ROC plot: M_Ridge_FP_MD_Scaling_PCA_256_median_100
Test set
True Positive Rate (Positive label: 1)
False Positive Rate (Positive label: 1)
0.0
0.2
0.4
0.6
0.8
1.0
M_Ridge classifier (AUC = 0.83)

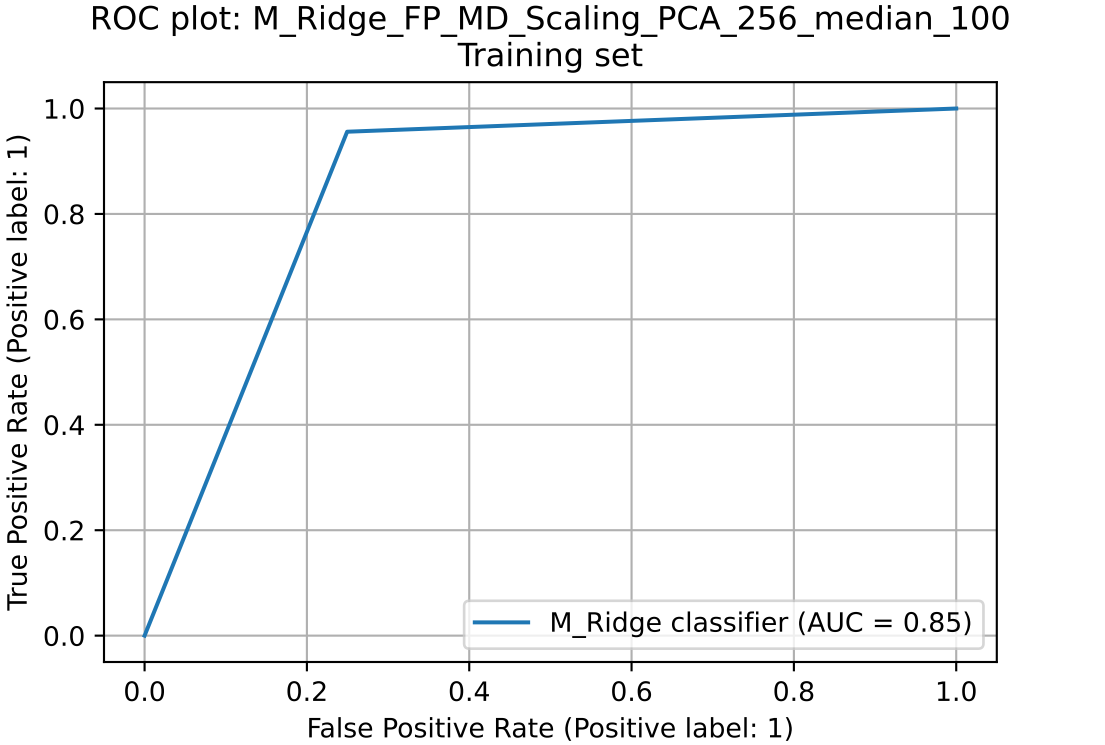
ROC plot: M_Ridge_FP_MD_Scaling_PCA_256_median_100
Training set
True Positive Rate (Positive label: 1)
False Positive Rate (Positive label: 1)
0.0
0.2
0.4
0.6
0.8
1.0
M_Ridge classifier (AUC = 0.85)

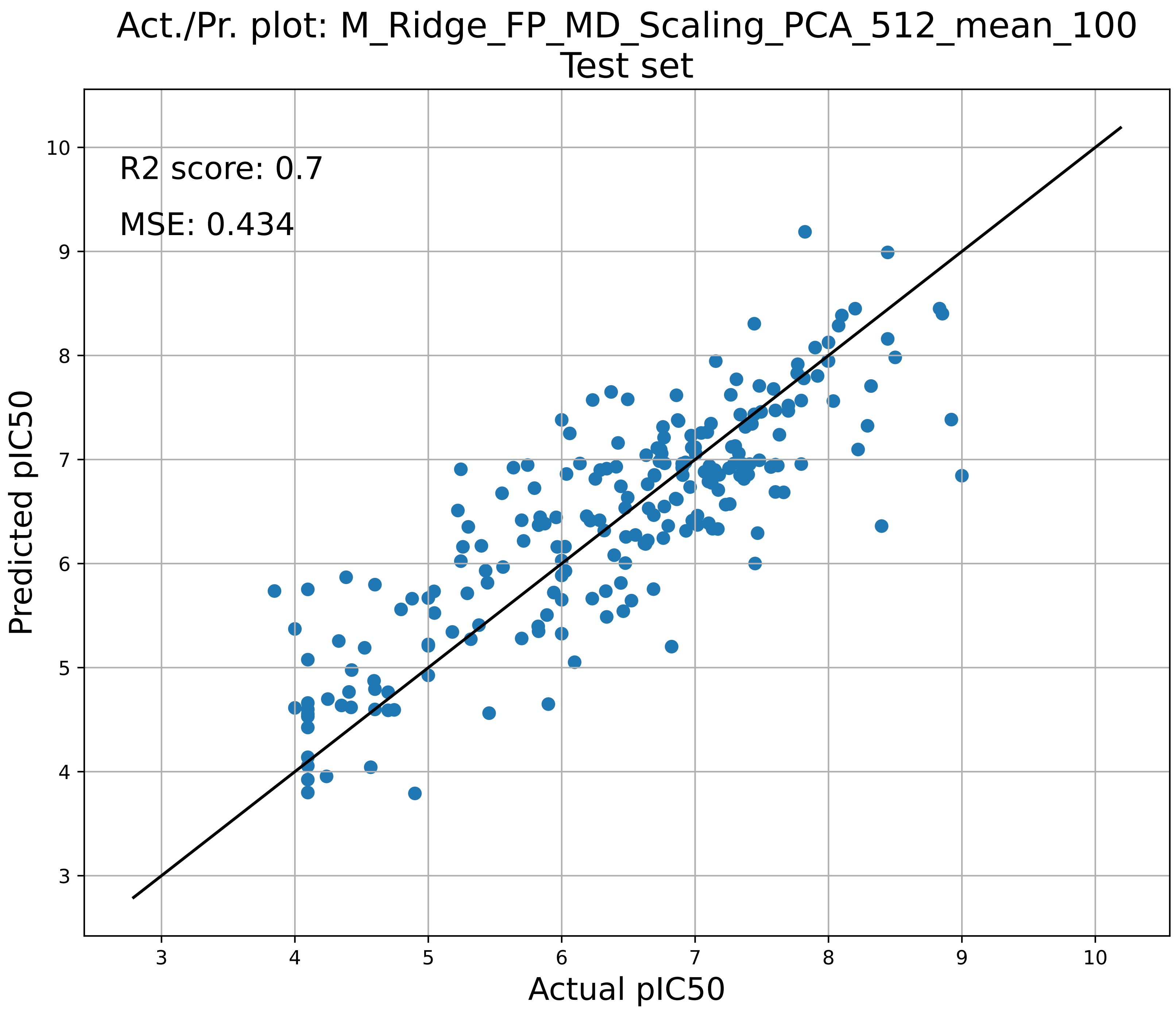
Act./Pr. plot: M_Ridge_FP_MD_Scaling_PCA_512_mean_100
Test set
R2 score: 0.7
MSE: 0.434
Predicted pIC50
Actual pIC50
3
4
5
6
7
8
9
10

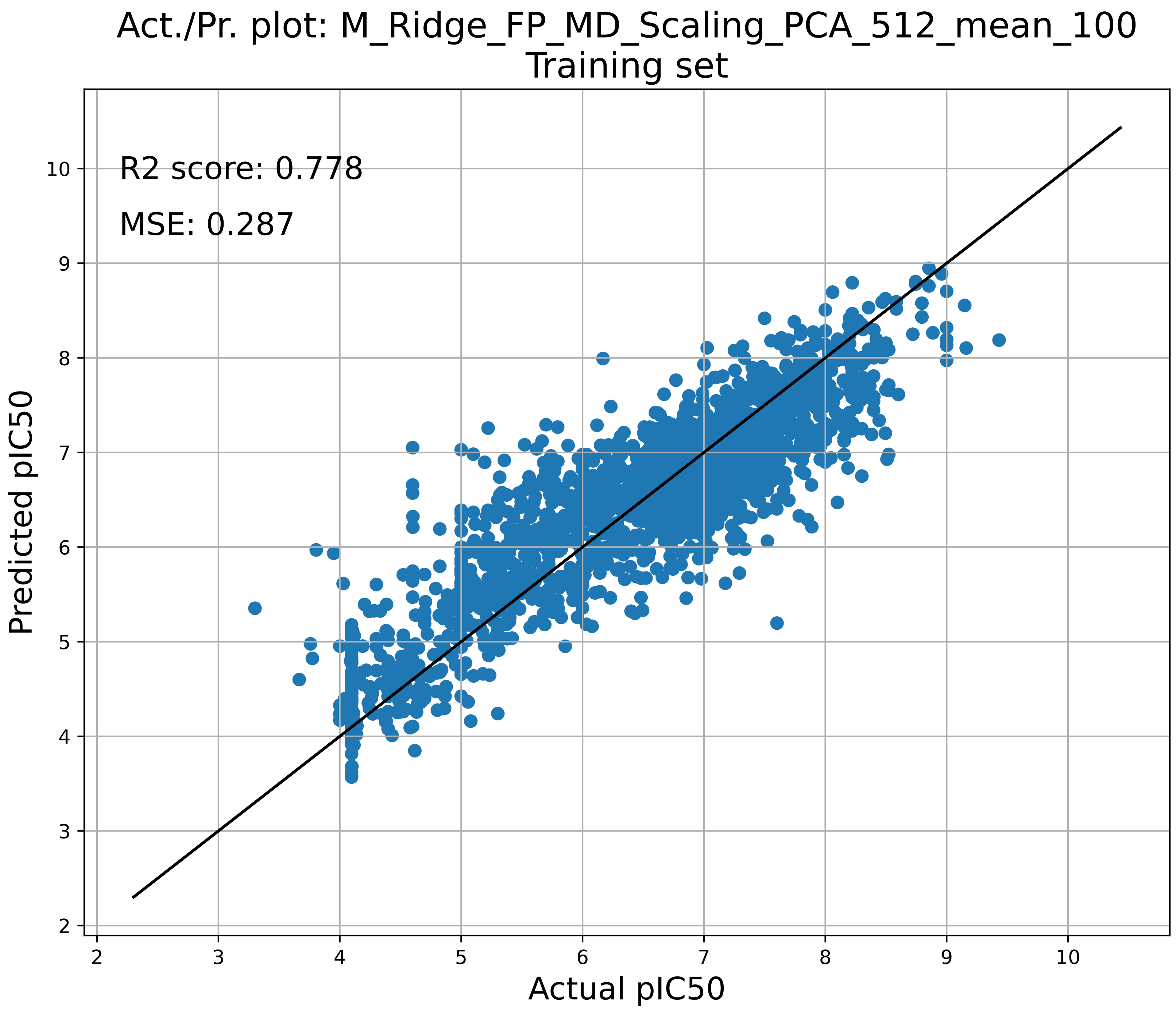

Act./Pr. plot: M_Ridge_FP_MD_Scaling_PCA_512_mean_100
Training set
R2 score: 0.778
MSE: 0.287
Predicted pIC50
Actual pIC50
2
3
4
5
6
7
8
9
10

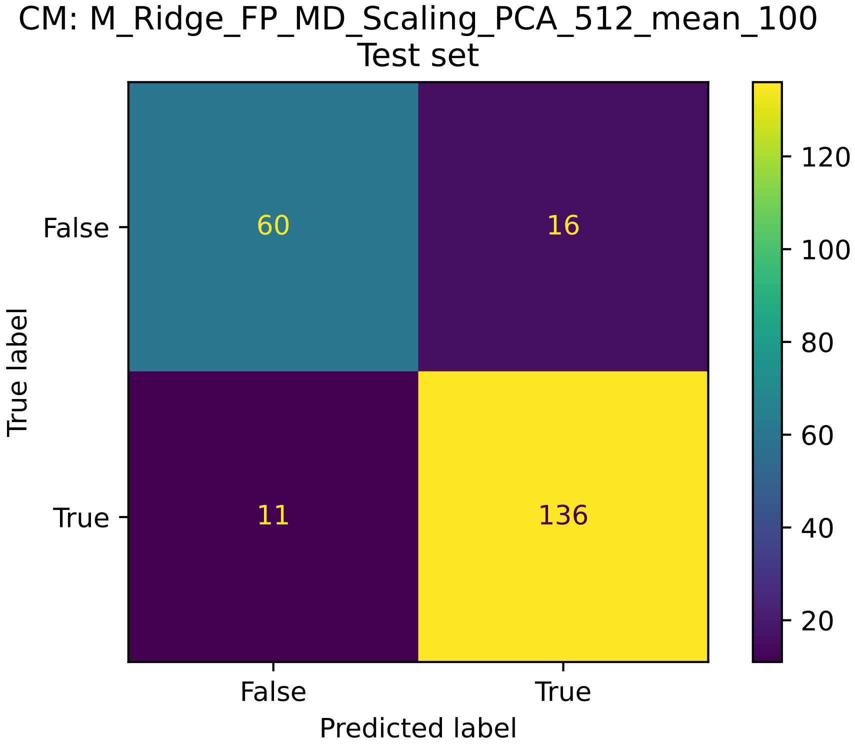

CM: M_Ridge_FP_MD_Scaling_PCA_512_mean_100
Test set
False
True
60
16
11
136
True label
False
True
Predicted label
120
100
80
60
40
20

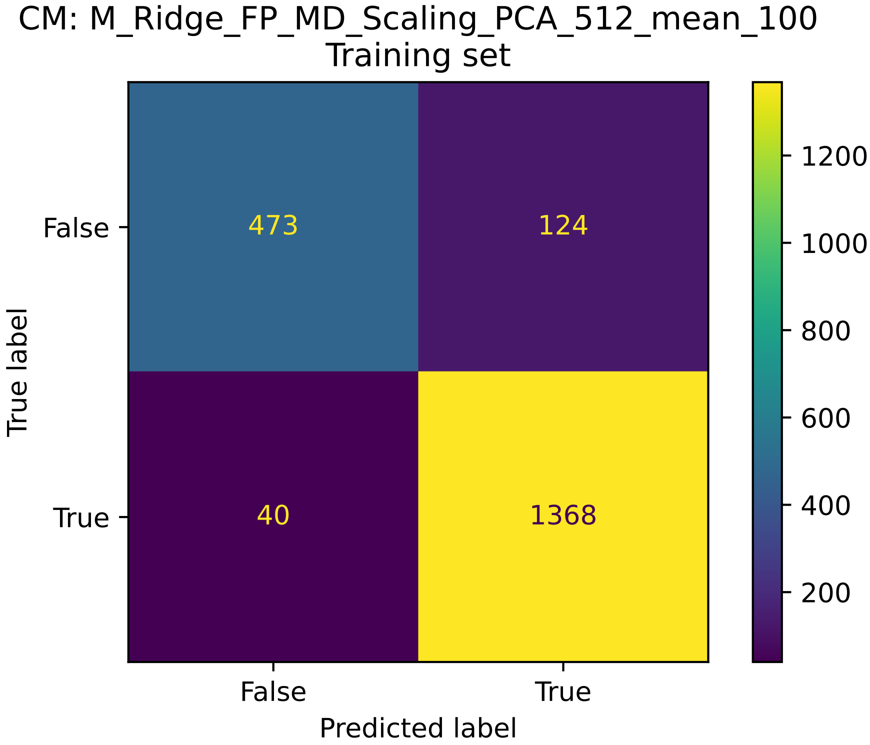
CM: M_Ridge_FP_MD_Scaling_PCA_512_mean_100
Training set
473
124
40
1368
False
True
True label
False
True
Predicted label
200
400
600
800
1000
1200

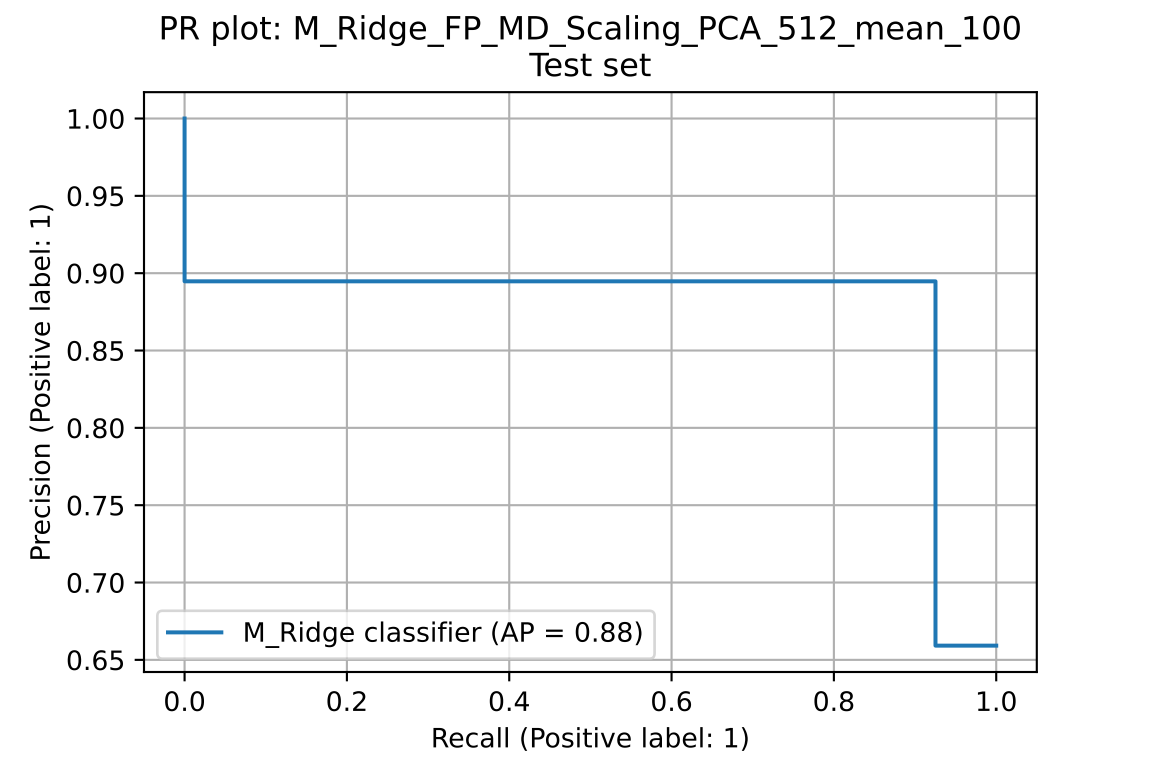
PR plot: M_Ridge_FP_MD_Scaling_PCA_512_mean_100
Test set
Precision (Positive label: 1)
Recall (Positive label: 1)
1.00
0.95
0.90
0.85
0.80
0.75
0.70
0.65
0.0
0.2
0.4
0.6
0.8
1.0
M_Ridge classifier (AP = 0.88)

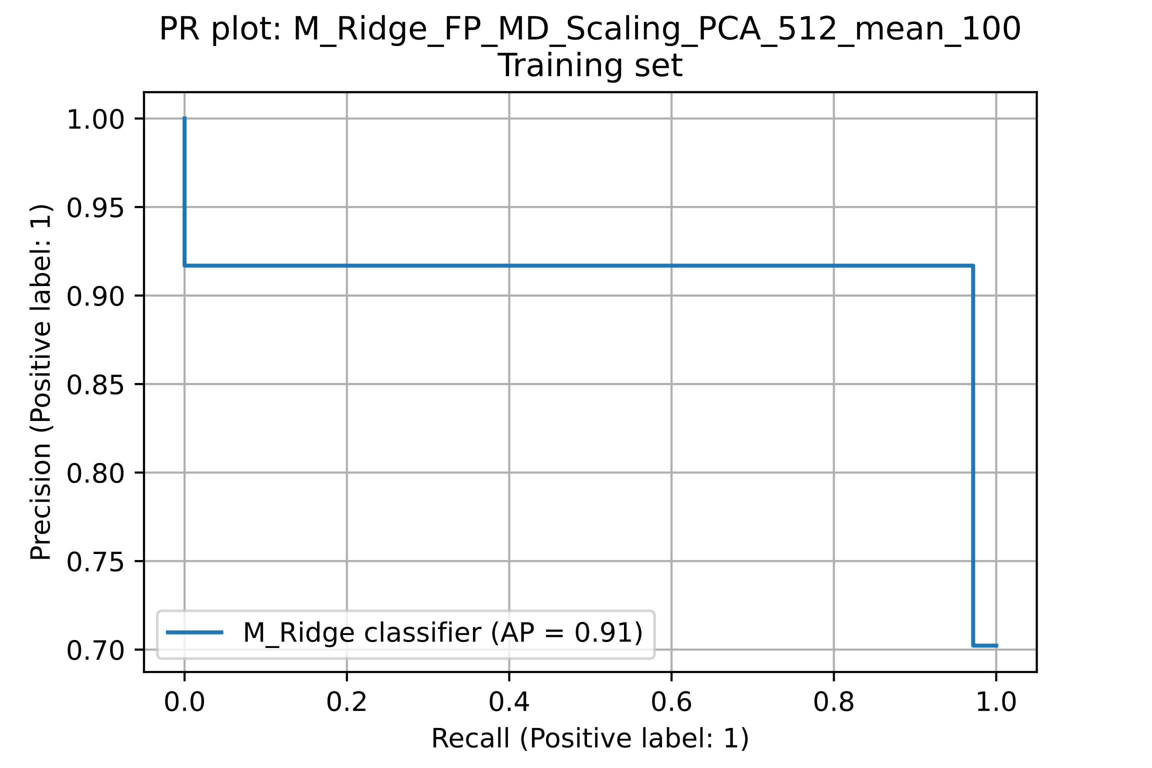
PR plot: M_Ridge_FP_MD_Scaling_PCA_512_mean_100
Training set
Precision (Positive label: 1)
Recall (Positive label: 1)
1.00
0.95
0.90
0.85
0.80
0.75
0.70
0.0
0.2
0.4
0.6
0.8
1.0
M_Ridge classifier (AP = 0.91)

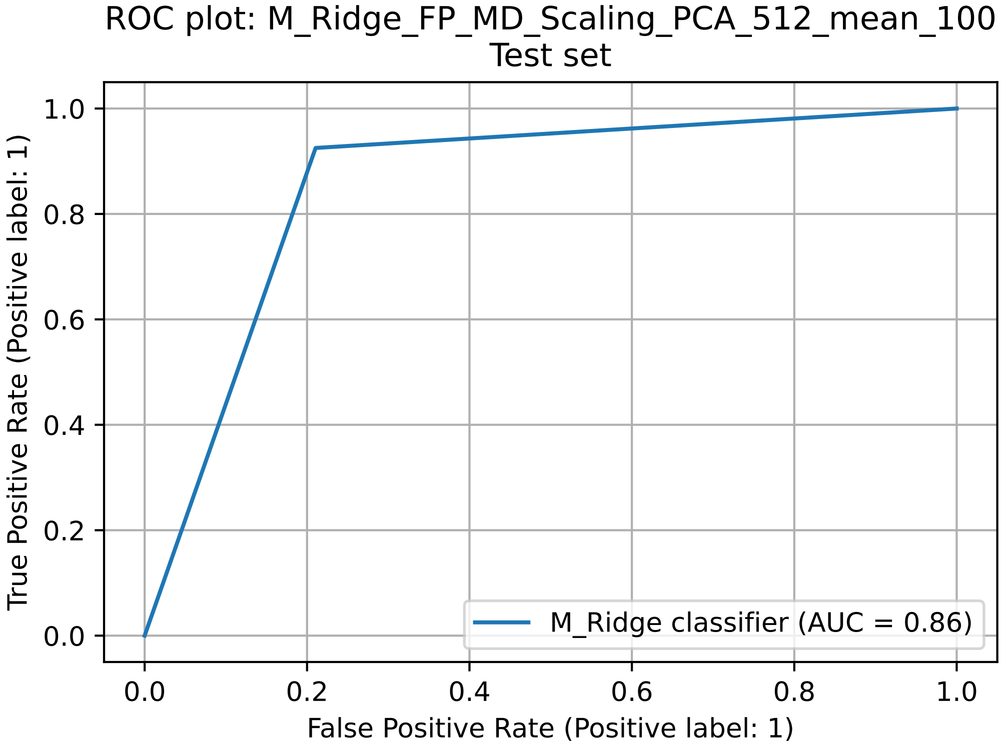

ROC plot: M_Ridge_FP_MD_Scaling_PCA_512_mean_100
Test set
True Positive Rate (Positive label: 1)
False Positive Rate (Positive label: 1)
0.0
0.2
0.4
0.6
0.8
1.0
M_Ridge classifier (AUC = 0.86)

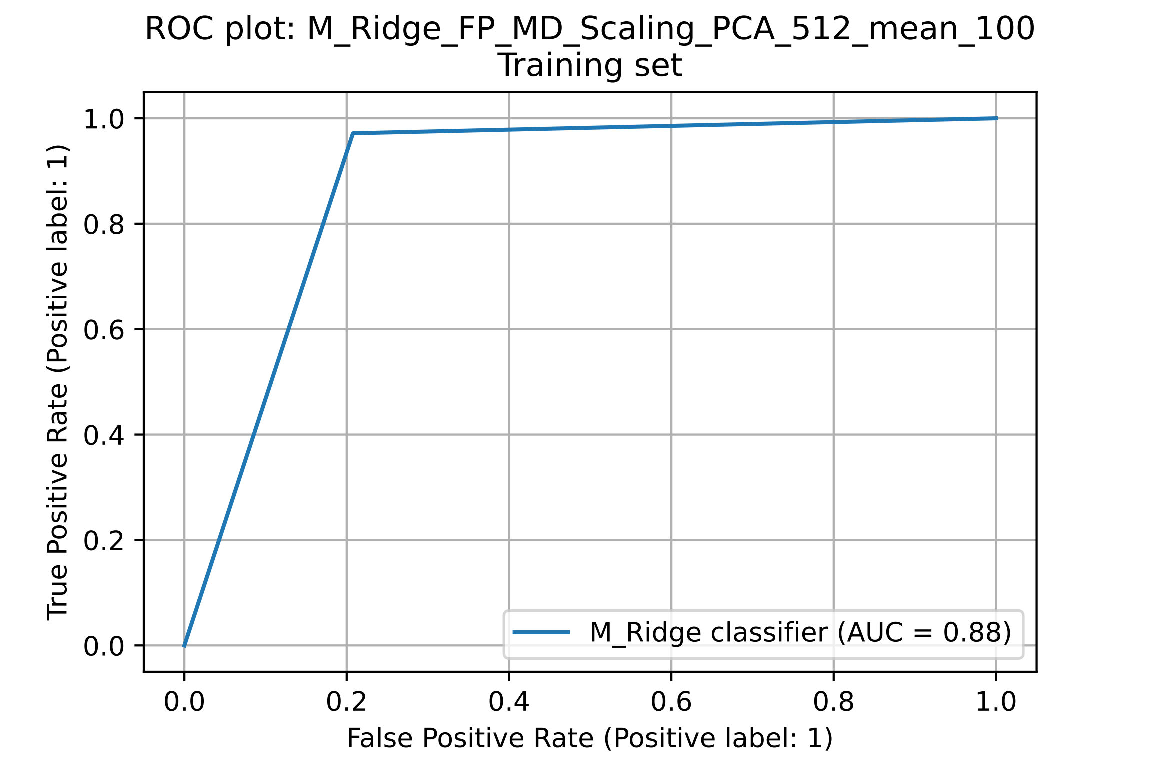

ROC plot: M_Ridge_FP_MD_Scaling_PCA_512_mean_100
Training set
True Positive Rate (Positive label: 1)
False Positive Rate (Positive label: 1)
0.0
0.2
0.4
0.6
0.8
1.0
M_Ridge classifier (AUC = 0.88)

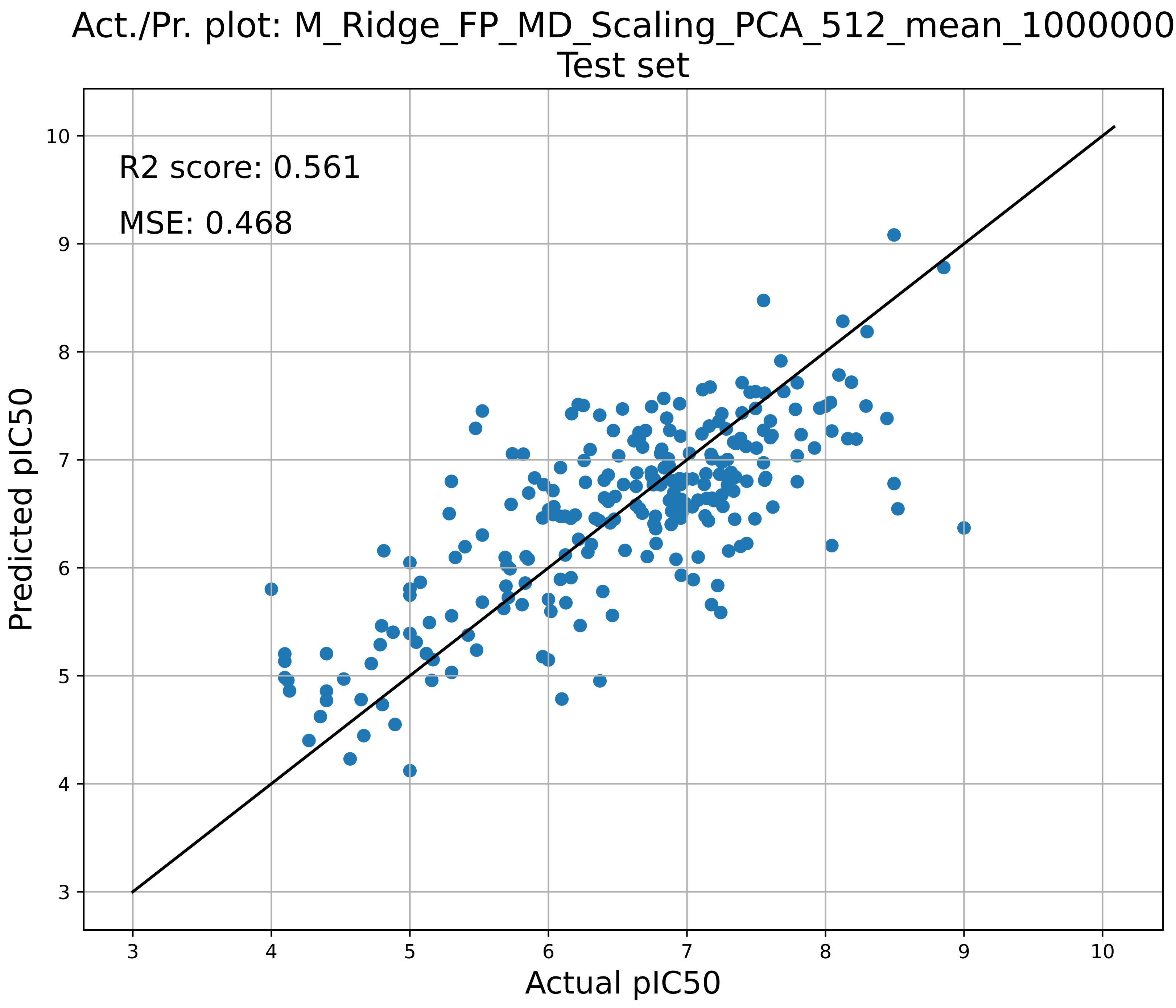

Act./Pr. plot: M_Ridge_FP_MD_Scaling_PCA_512_mean_1000000
Test set
R2 score: 0.561
MSE: 0.468
Predicted pIC50
Actual pIC50
3
4
5
6
7
8
9
10

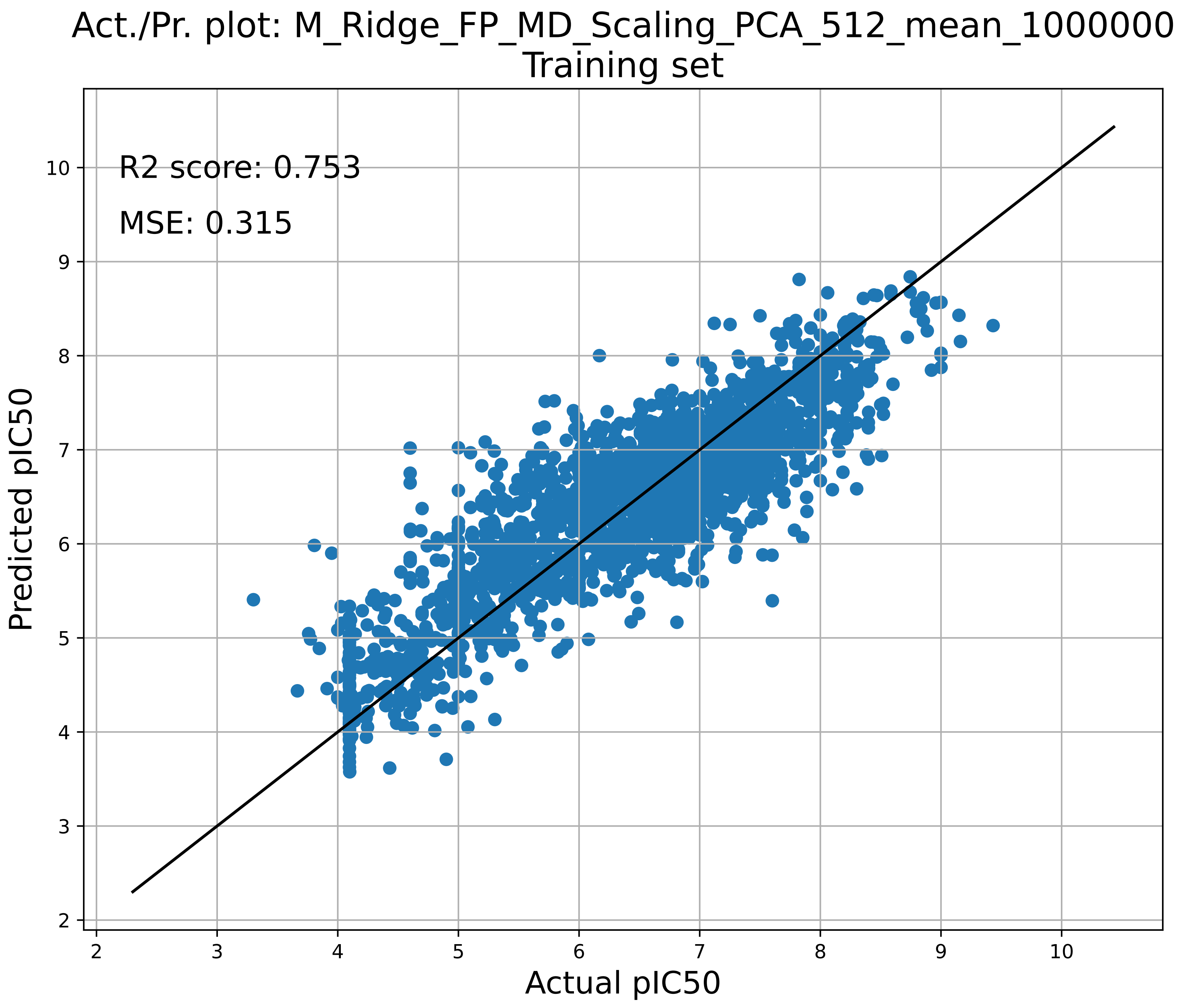

Act./Pr. plot: M_Ridge_FP_MD_Scaling_PCA_512_mean_1000000
Training set
R2 score: 0.753
MSE: 0.315
Predicted pIC50
Actual pIC50
2
3
4
5
6
7
8
9
10

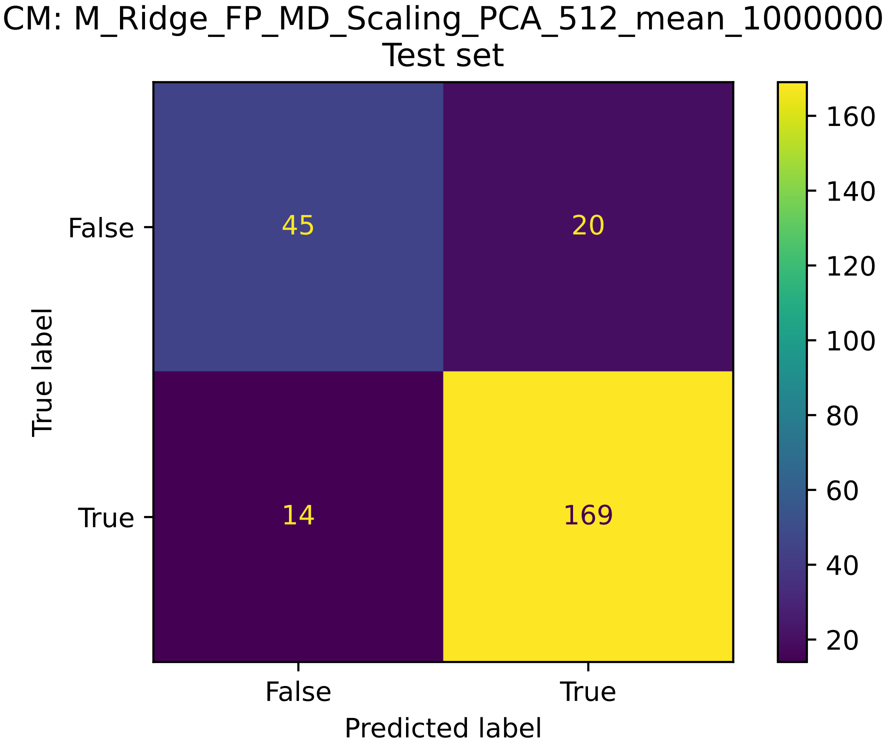
CM: M_Ridge_FP_MD_Scaling_PCA_512_mean_1000000
Test set
True label
False
True
45
20
14
169
False
True
Predicted label
160
140
120
100
80
60
40
20

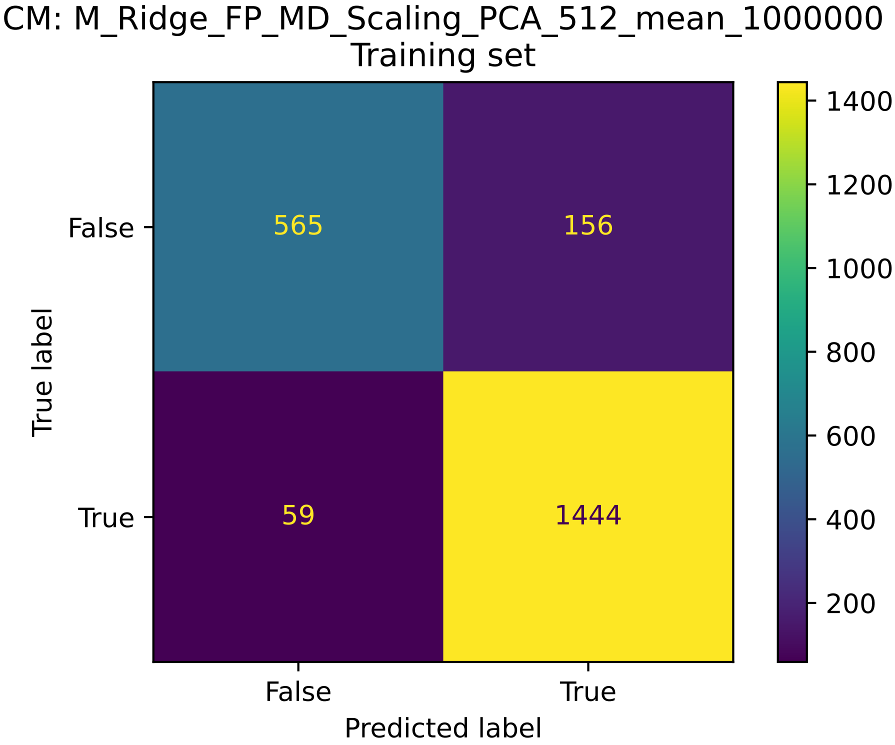
CM: M_Ridge_FP_MD_Scaling_PCA_512_mean_1000000
Training set
True label
False
True
565
156
59
1444
Predicted label
False
True
1400
1200
1000
800
600
400
200

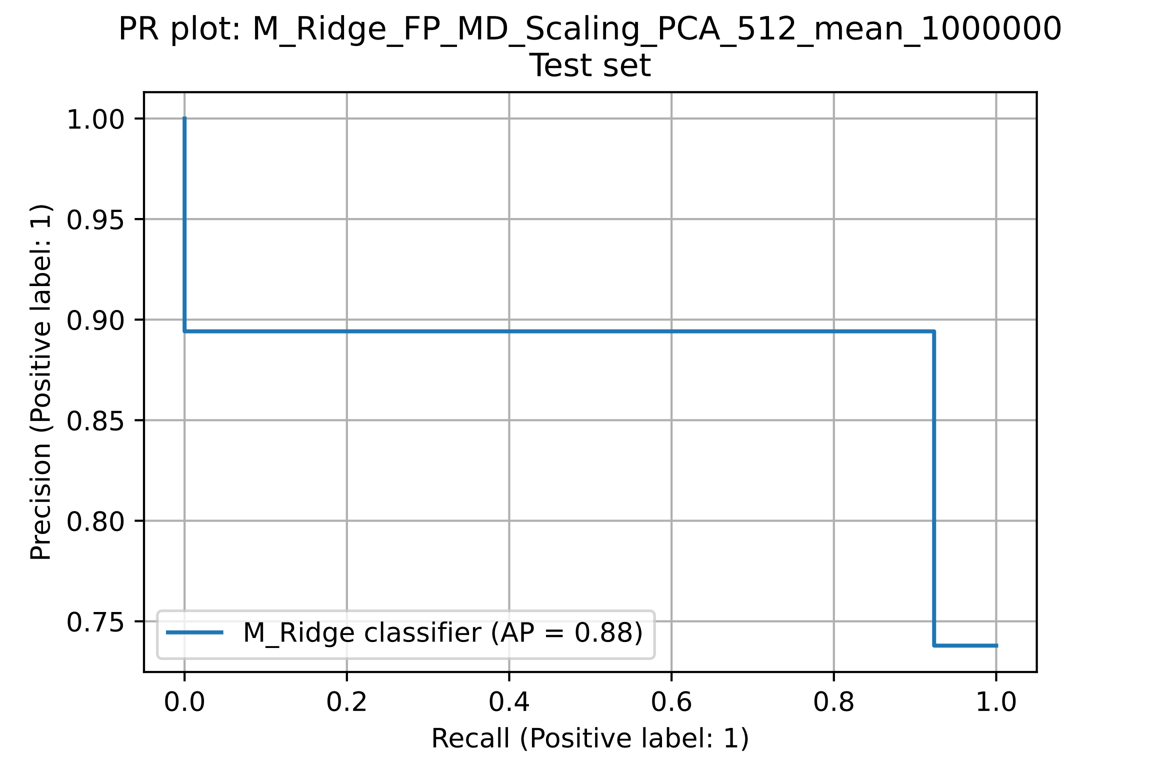

PR plot: M_Ridge_FP_MD_Scaling_PCA_512_mean_1000000
Test set
1.00
0.95
0.90
0.85
0.80
0.75
Precision (Positive label: 1)
0.0
0.2
0.4
0.6
0.8
1.0
Recall (Positive label: 1)
M_Ridge classifier (AP = 0.88)

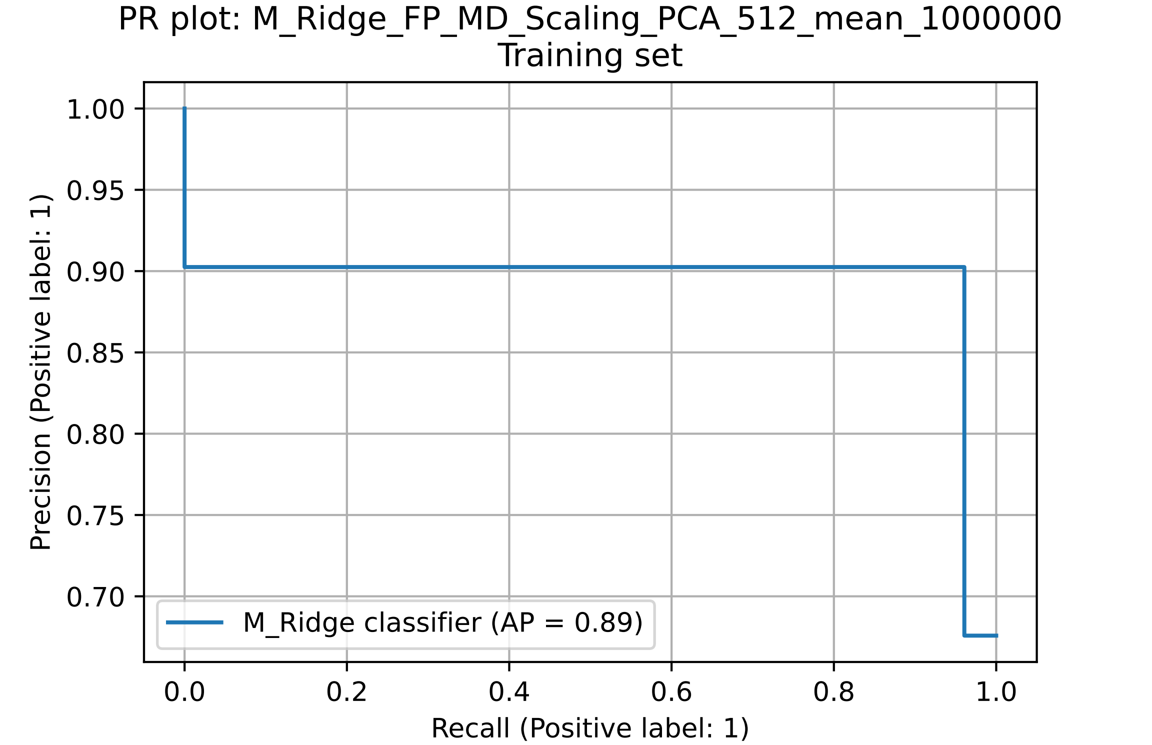
PR plot: M_Ridge_FP_MD_Scaling_PCA_512_mean_1000000
Training set
1.00
0.95
0.90
0.85
0.80
0.75
0.70
Precision (Positive label: 1)
0.0
0.2
0.4
0.6
0.8
1.0
Recall (Positive label: 1)
M_Ridge classifier (AP = 0.89)

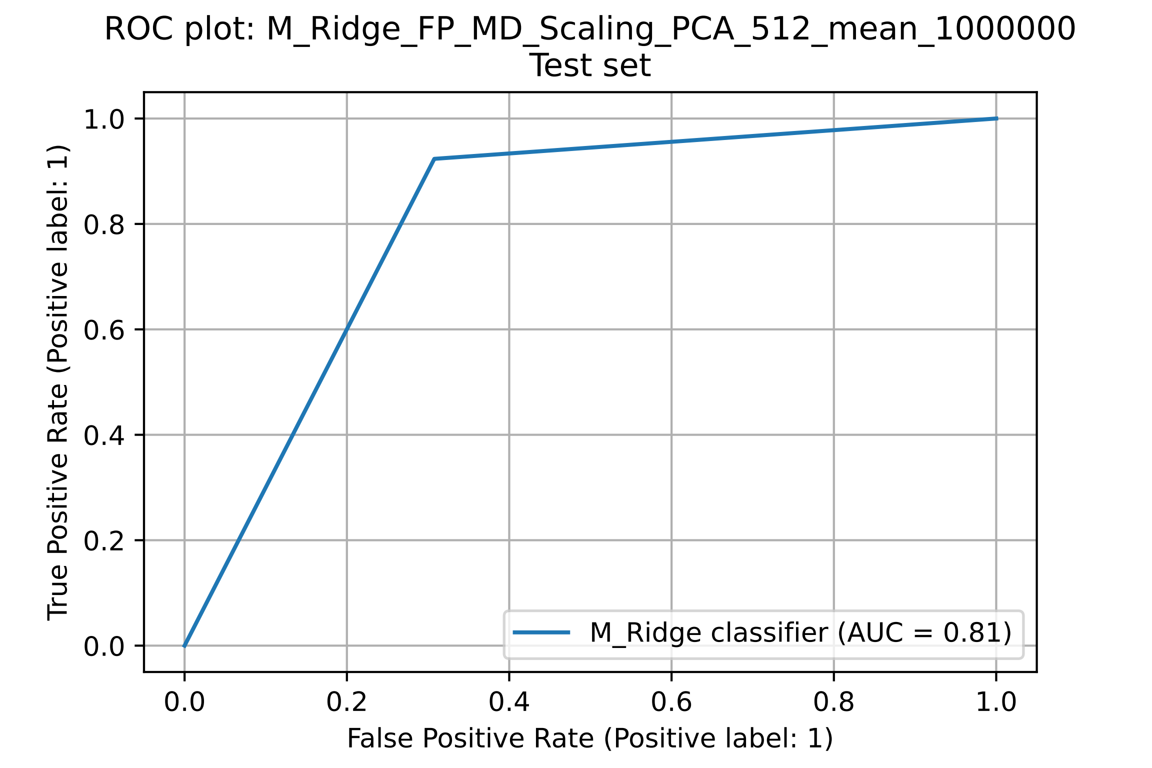

ROC plot: M_Ridge_FP_MD_Scaling_PCA_512_mean_1000000
Test set
True Positive Rate (Positive label: 1)
False Positive Rate (Positive label: 1)
0.0
0.2
0.4
0.6
0.8
1.0
M_Ridge classifier (AUC = 0.81)

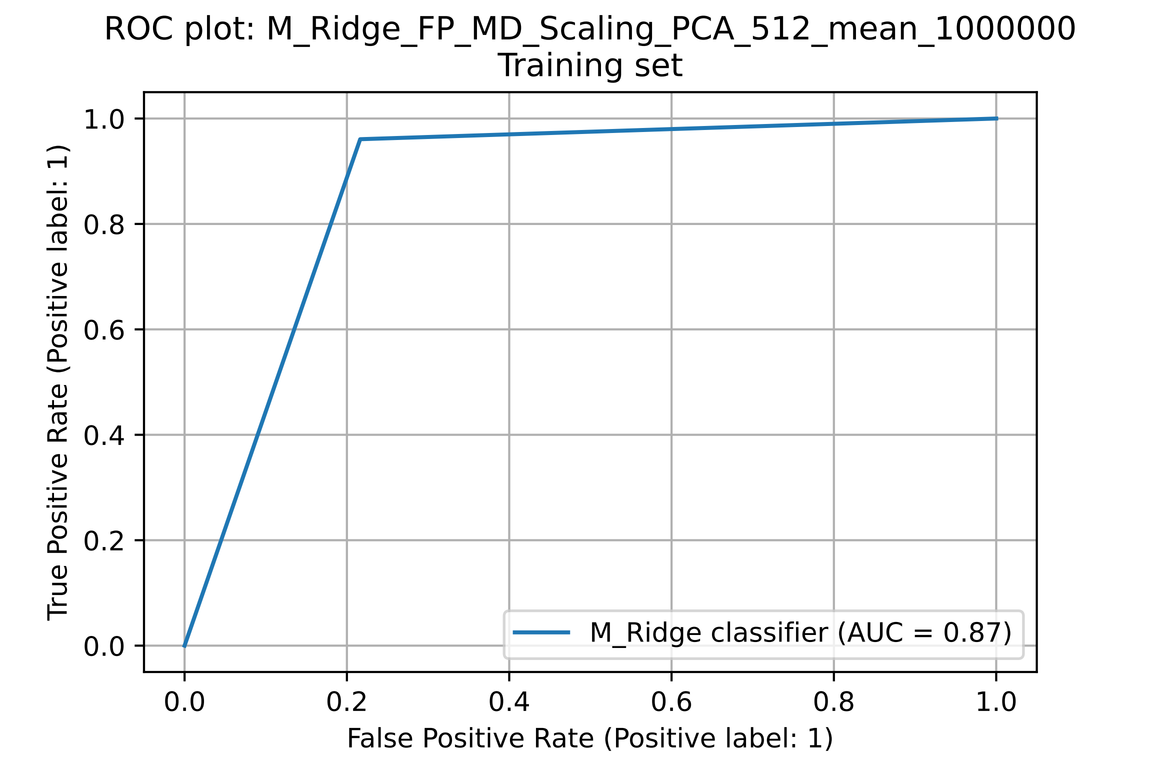
ROC plot: M_Ridge_FP_MD_Scaling_PCA_512_mean_1000000
Training set
True Positive Rate (Positive label: 1)
False Positive Rate (Positive label: 1)
0.0
0.2
0.4
0.6
0.8
1.0
M_Ridge classifier (AUC = 0.87)

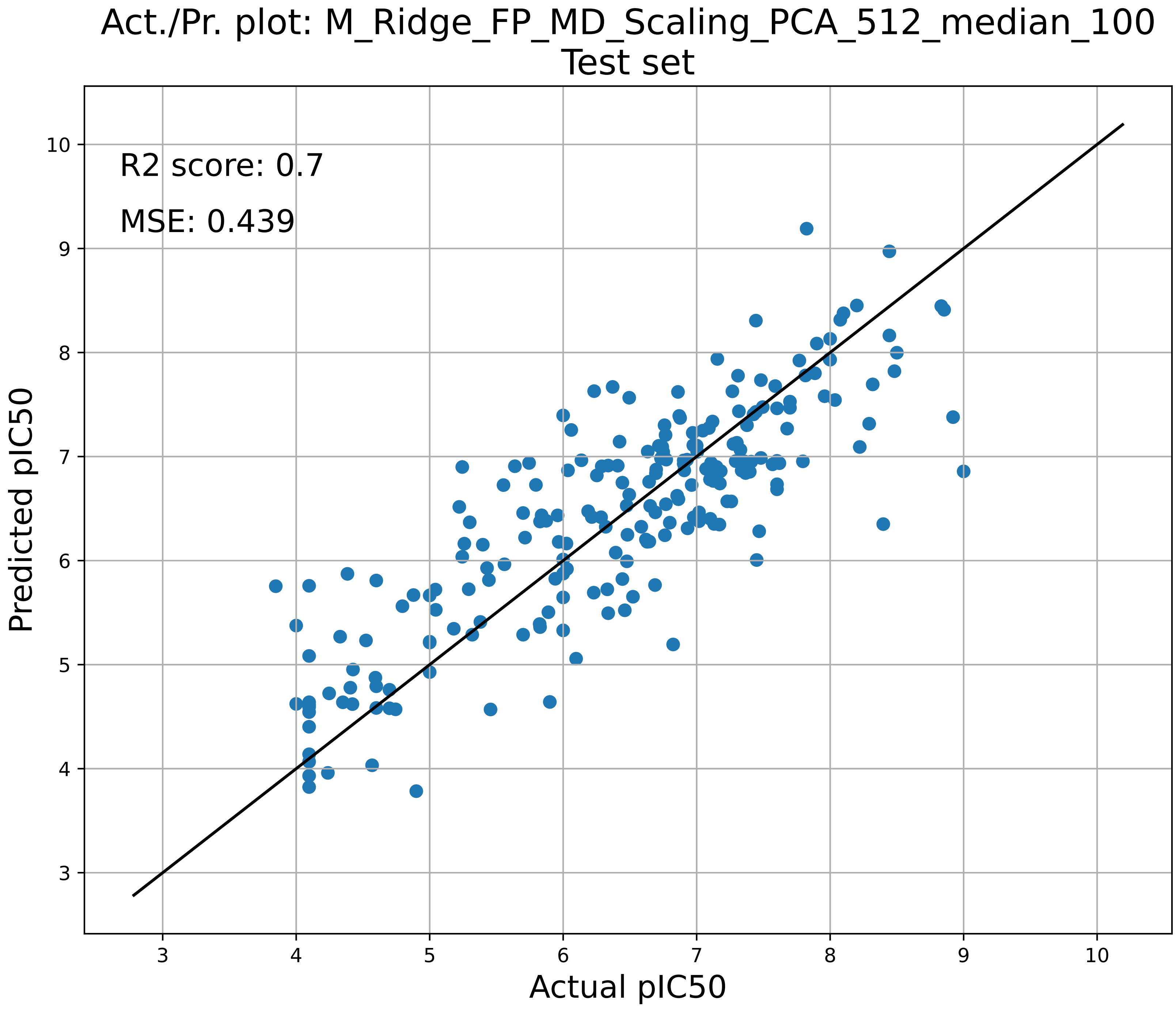

Act./Pr. plot: M_Ridge_FP_MD_Scaling_PCA_512_median_100
Test set
R2 score: 0.7
MSE: 0.439
Predicted pIC50
Actual pIC50
3
4
5
6
7
8
9
10

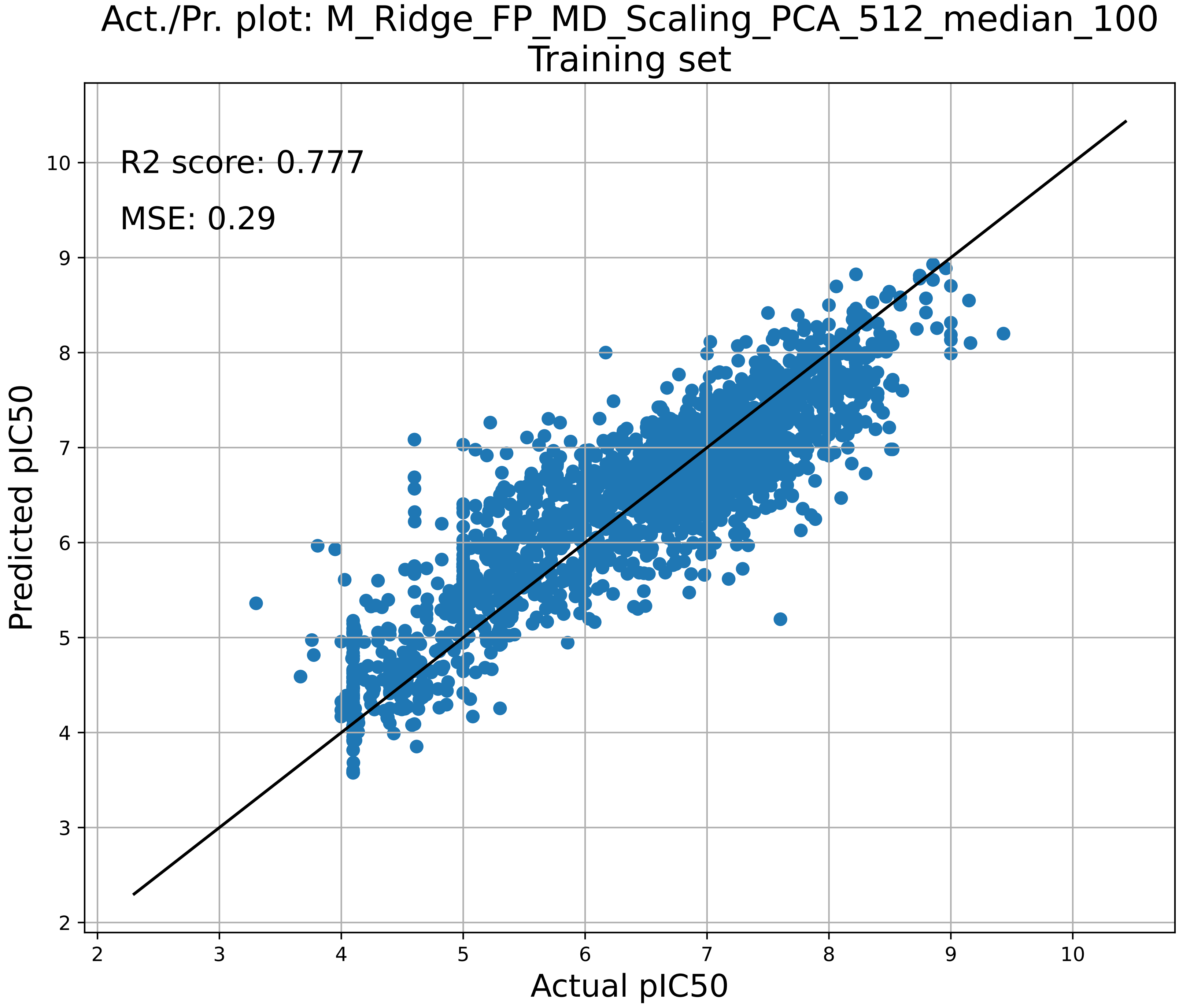

Act./Pr. plot: M_Ridge_FP_MD_Scaling_PCA_512_median_100
Training set
R2 score: 0.777
MSE: 0.29
Predicted pIC50
Actual pIC50
2
3
4
5
6
7
8
9
10

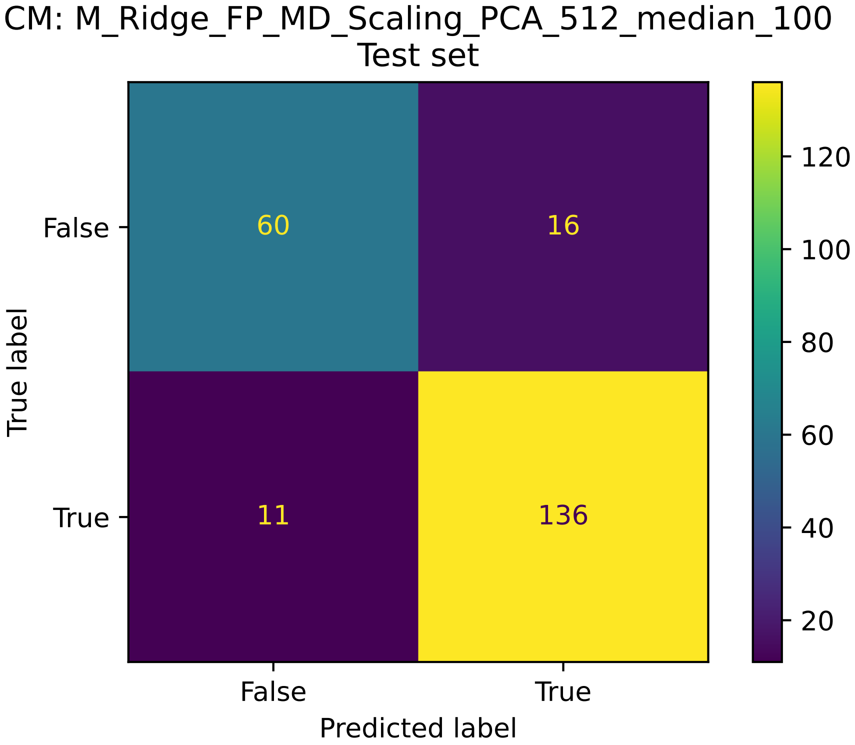
CM: M_Ridge_FP_MD_Scaling_PCA_512_median_100
Test set
True label
False
True
60
16
11
136
Predicted label
False
True
120
100
80
60
40
20

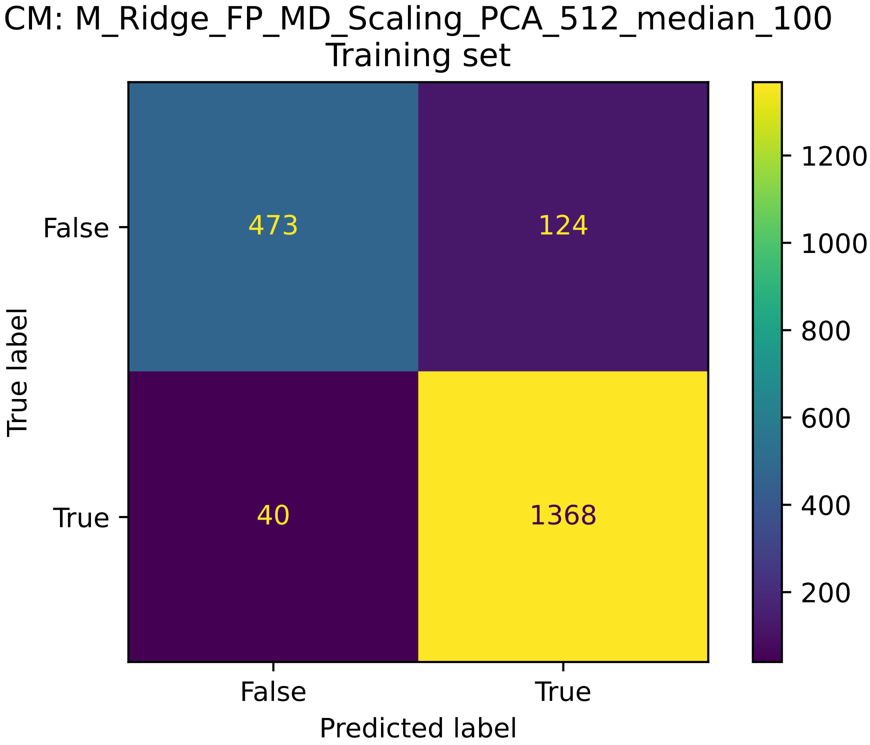
CM: M_Ridge_FP_MD_Scaling_PCA_512_median_100
Training set
True label
False
True
473
124
40
1368
False
True
Predicted label
200
400
600
800
1000
1200

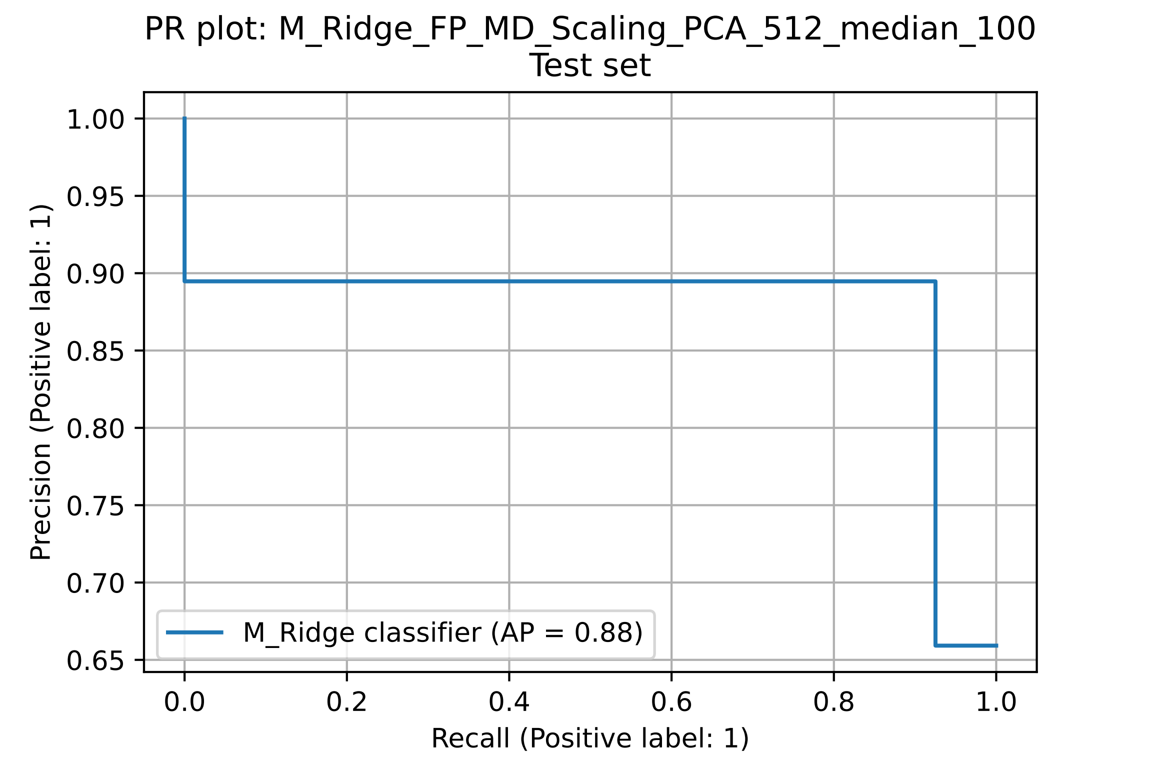
PR plot: M_Ridge_FP_MD_Scaling_PCA_512_median_100
Test set
Precision (Positive label: 1)
Recall (Positive label: 1)
M_Ridge classifier (AP = 0.88)
1.00
0.95
0.90
0.85
0.80
0.75
0.70
0.65
0.0
0.2
0.4
0.6
0.8
1.0

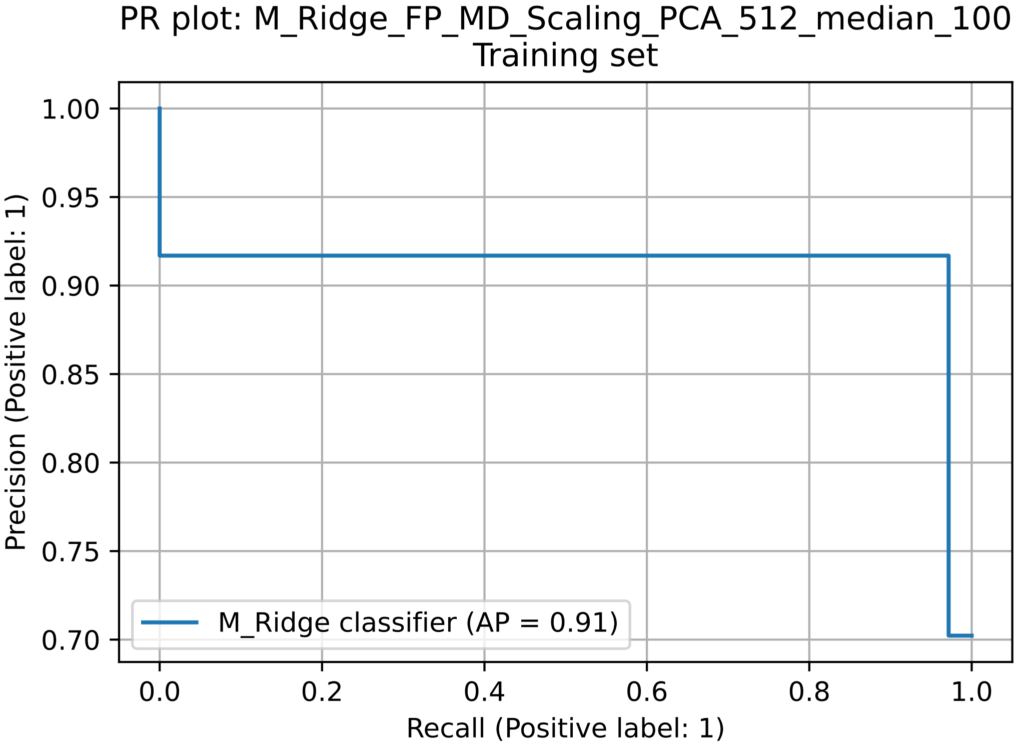
PR plot: M_Ridge_FP_MD_Scaling_PCA_512_median_100
Training set
Precision (Positive label: 1)
Recall (Positive label: 1)
1.00
0.95
0.90
0.85
0.80
0.75
0.70
0.0
0.2
0.4
0.6
0.8
1.0
M_Ridge classifier (AP = 0.91)

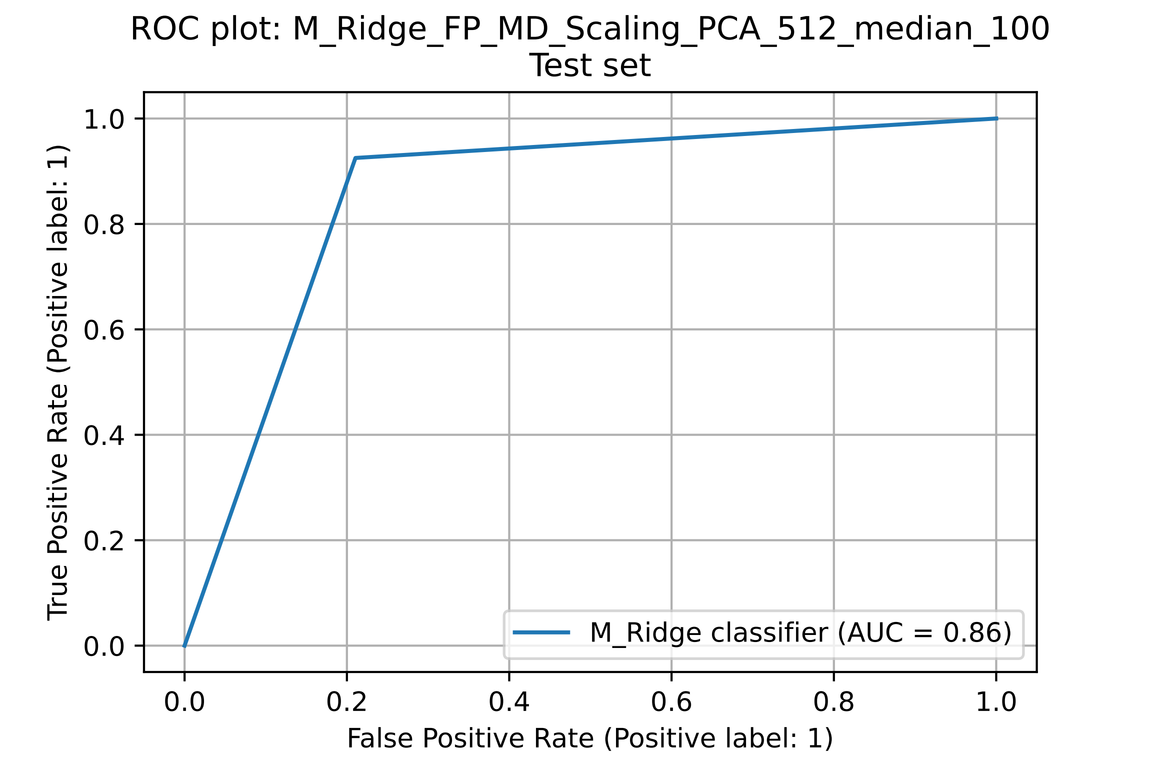
ROC plot: M_Ridge_FP_MD_Scaling_PCA_512_median_100
Test set
True Positive Rate (Positive label: 1)
False Positive Rate (Positive label: 1)
0.0
0.2
0.4
0.6
0.8
1.0
M_Ridge classifier (AUC = 0.86)

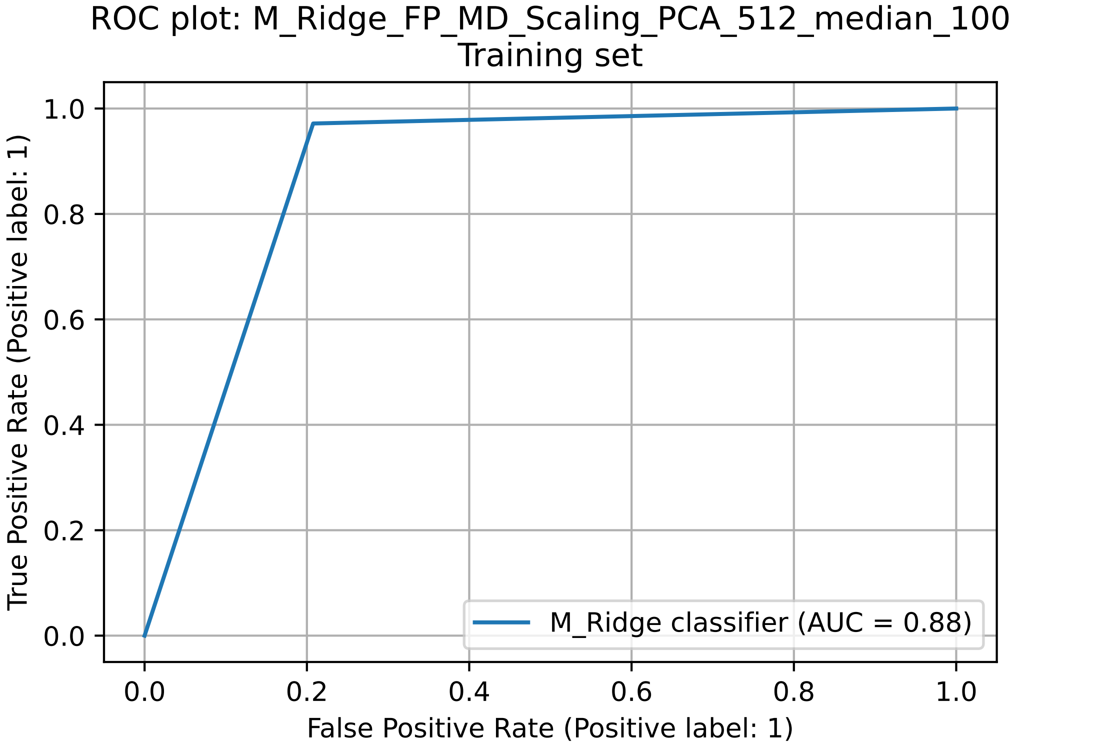
ROC plot: M_Ridge_FP_MD_Scaling_PCA_512_median_100
Training set
True Positive Rate (Positive label: 1)
False Positive Rate (Positive label: 1)
0.0
0.2
0.4
0.6
0.8
1.0
M_Ridge classifier (AUC = 0.88)

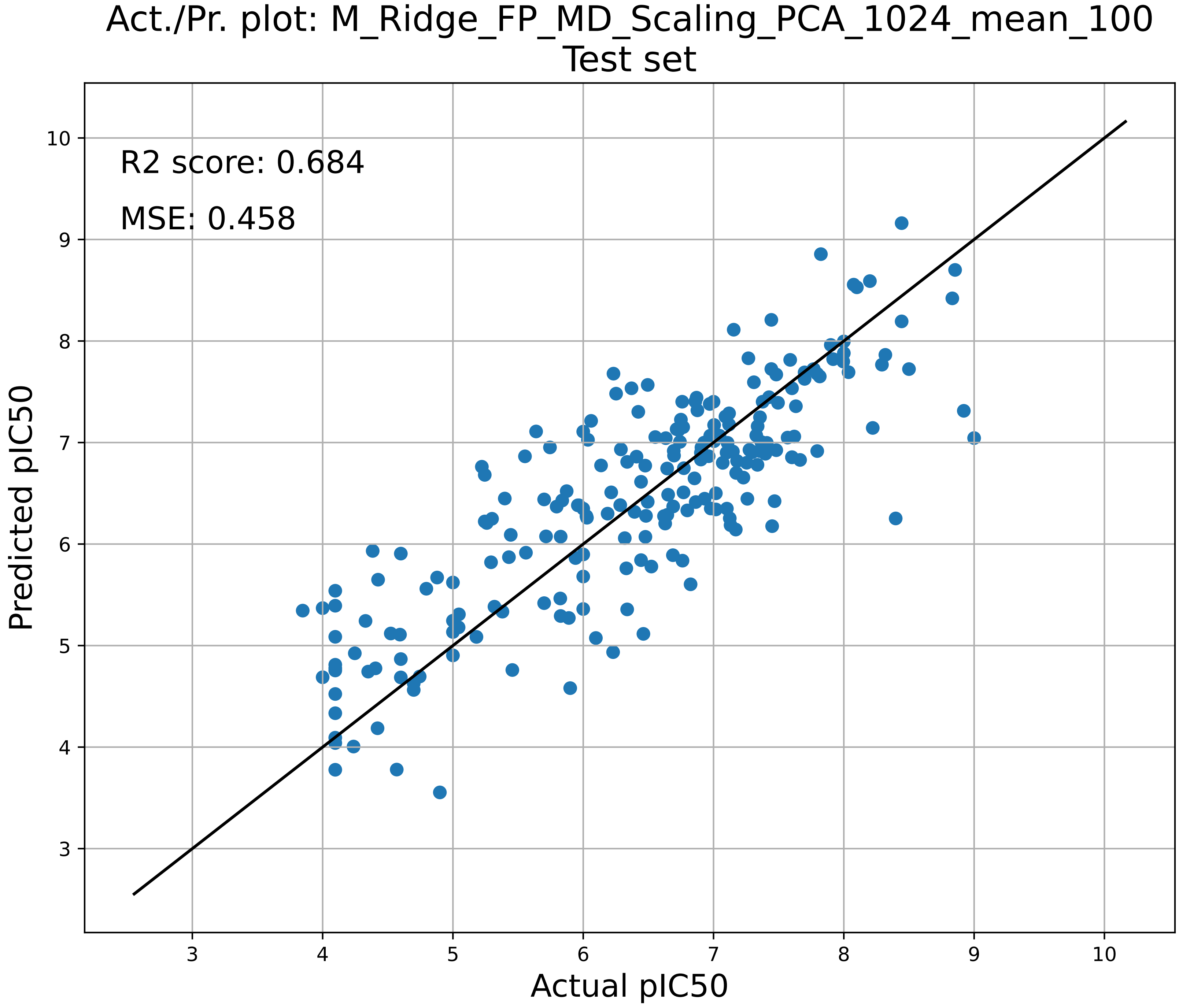

Act./Pr. plot: M_Ridge_FP_MD_Scaling_PCA_1024_mean_100
Test set
R2 score: 0.684
MSE: 0.458
Predicted pIC50
Actual pIC50
3
4
5
6
7
8
9
10

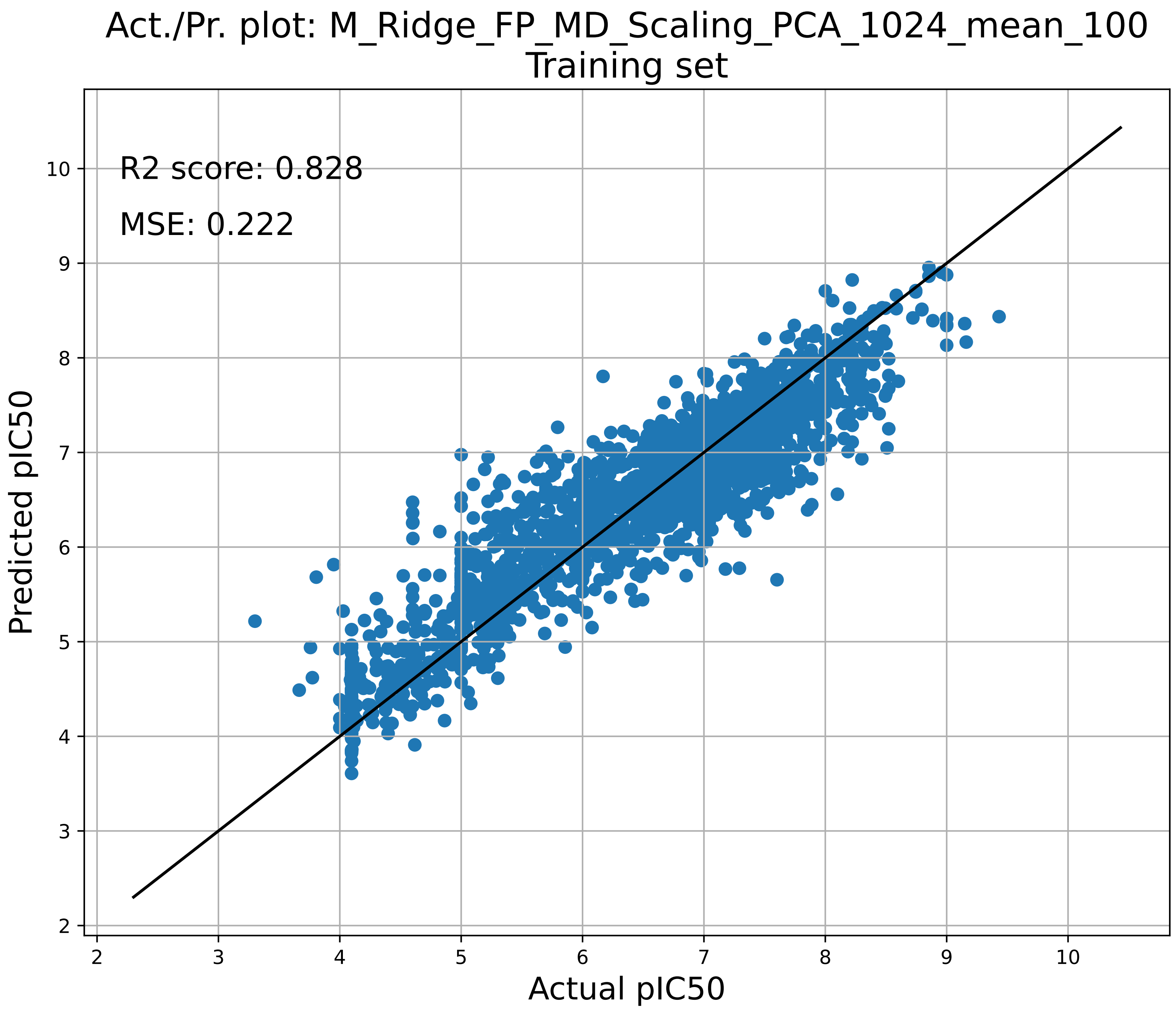
Act./Pr. plot: M_Ridge_FP_MD_Scaling_PCA_1024_mean_100
Training set
R2 score: 0.828
MSE: 0.222
Predicted pIC50
Actual pIC50
2
3
4
5
6
7
8
9
10

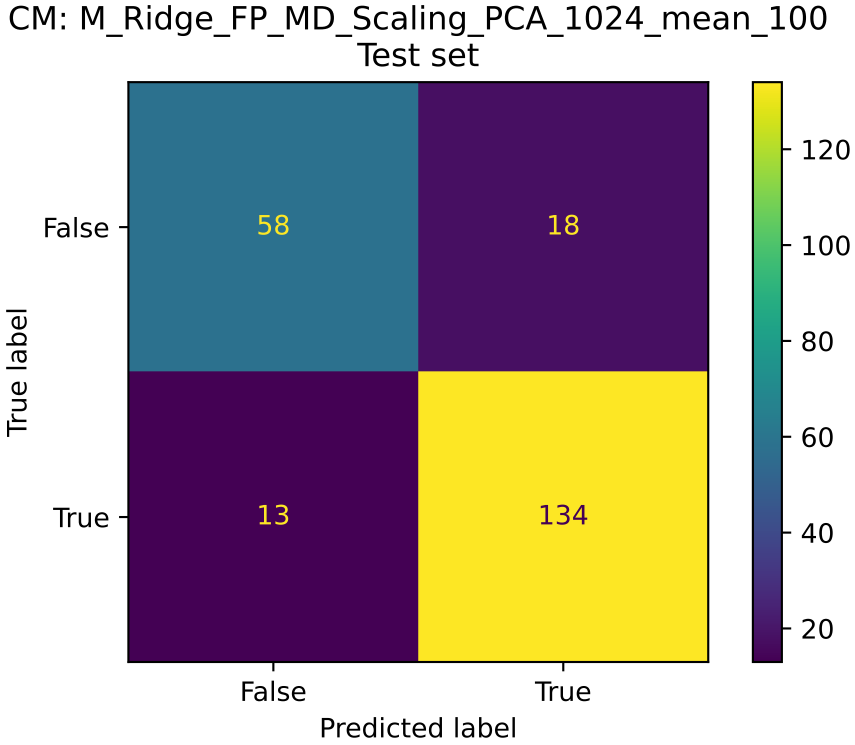
CM: M_Ridge_FP_MD_Scaling_PCA_1024_mean_100
Test set
True label
False
True
58
18
13
134
Predicted label
False
True
120
100
80
60
40
20

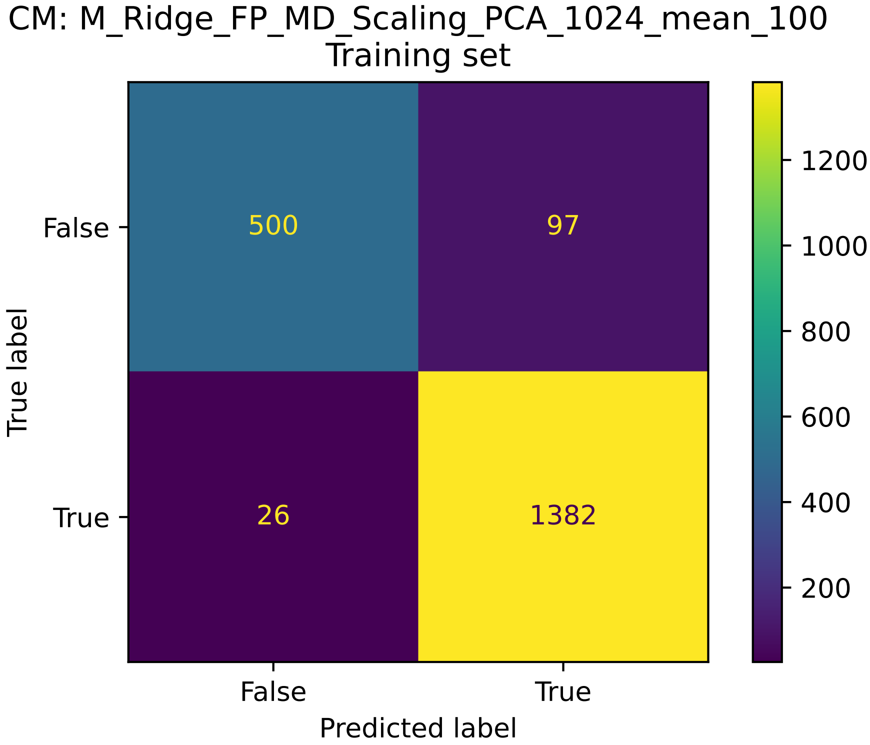

CM: M_Ridge_FP_MD_Scaling_PCA_1024_mean_100
Training set
500
97
26
1382
False
True
True label
False
True
Predicted label
1200
1000
800
600
400
200

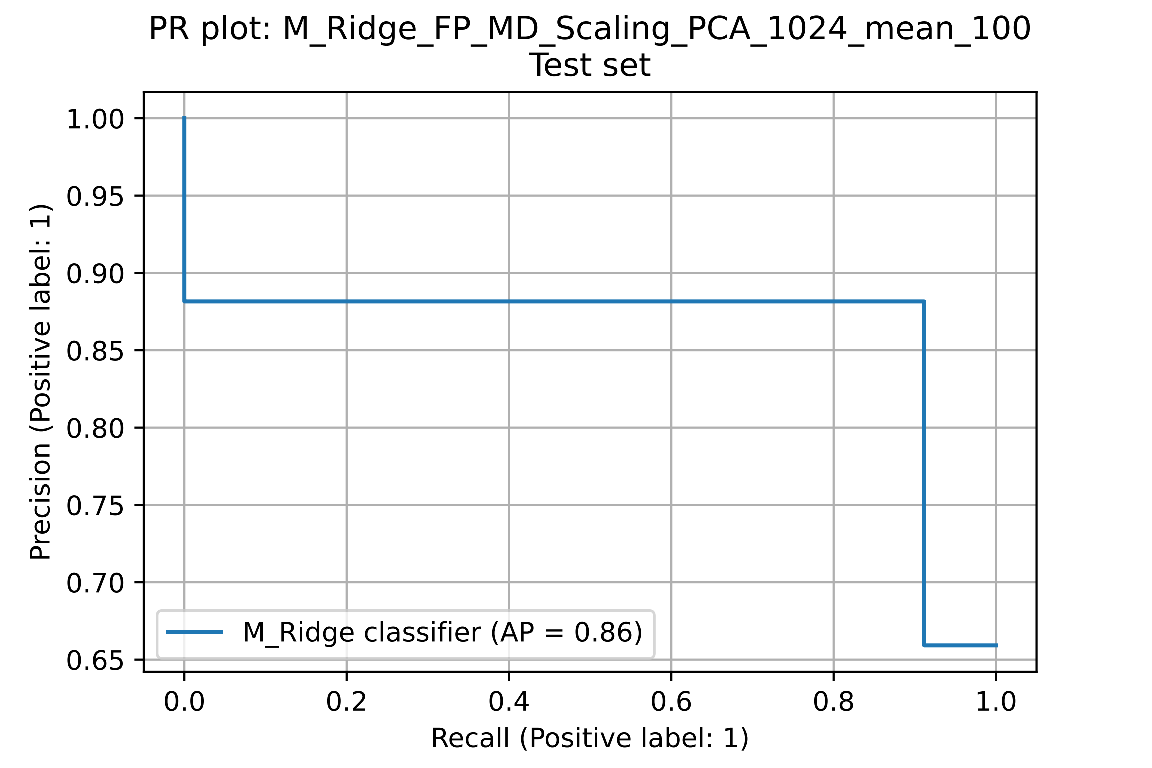
PR plot: M_Ridge_FP_MD_Scaling_PCA_1024_mean_100
Test set
Precision (Positive label: 1)
Recall (Positive label: 1)
1.00
0.95
0.90
0.85
0.80
0.75
0.70
0.65
0.0
0.2
0.4
0.6
0.8
1.0
M_Ridge classifier (AP = 0.86)

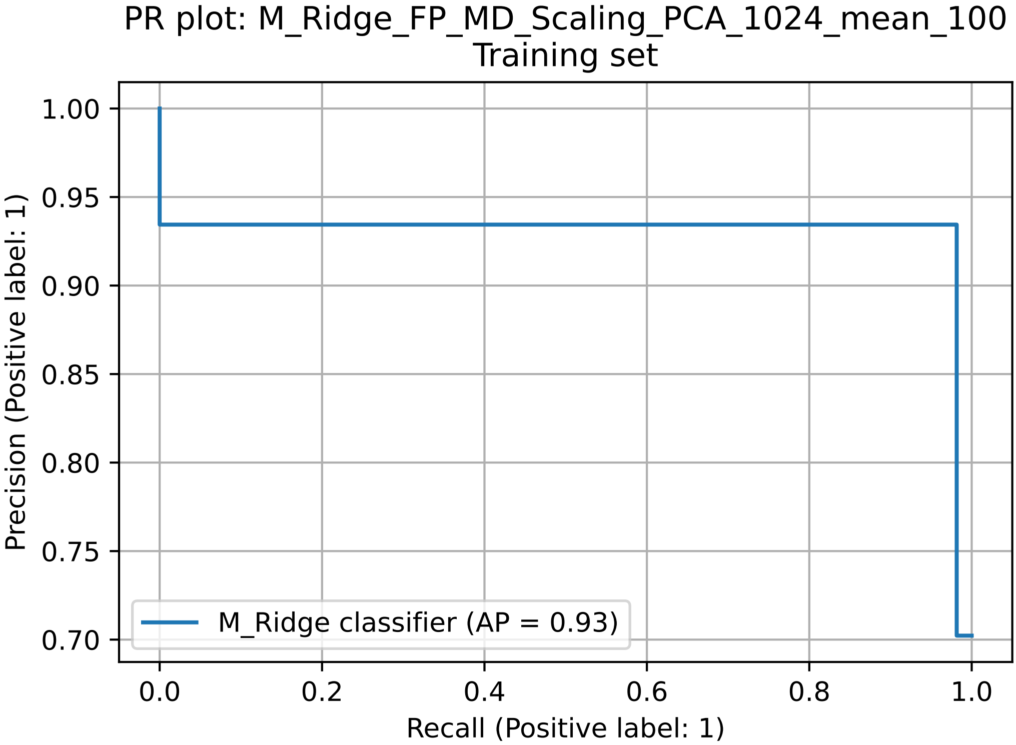
PR plot: M_Ridge_FP_MD_Scaling_PCA_1024_mean_100
Training set
Precision (Positive label: 1)
Recall (Positive label: 1)
M_Ridge classifier (AP = 0.93)
1.00
0.95
0.90
0.85
0.80
0.75
0.70
0.0
0.2
0.4
0.6
0.8
1.0

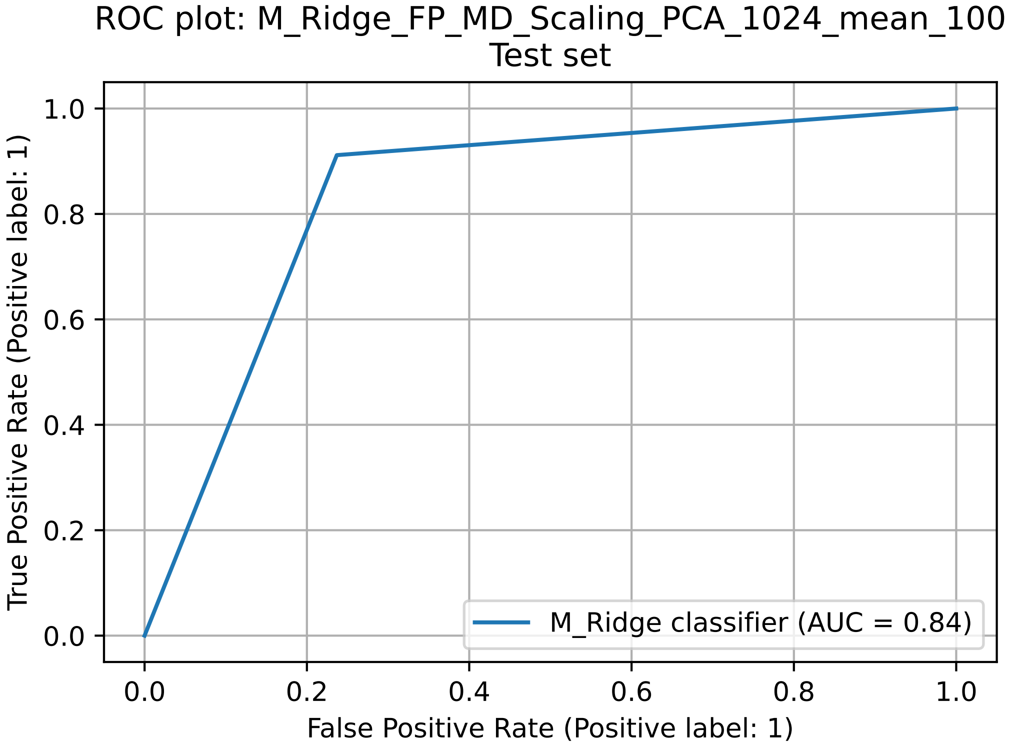
ROC plot: M_Ridge_FP_MD_Scaling_PCA_1024_mean_100
Test set
True Positive Rate (Positive label: 1)
False Positive Rate (Positive label: 1)
0.0
0.2
0.4
0.6
0.8
1.0
M_Ridge classifier (AUC = 0.84)

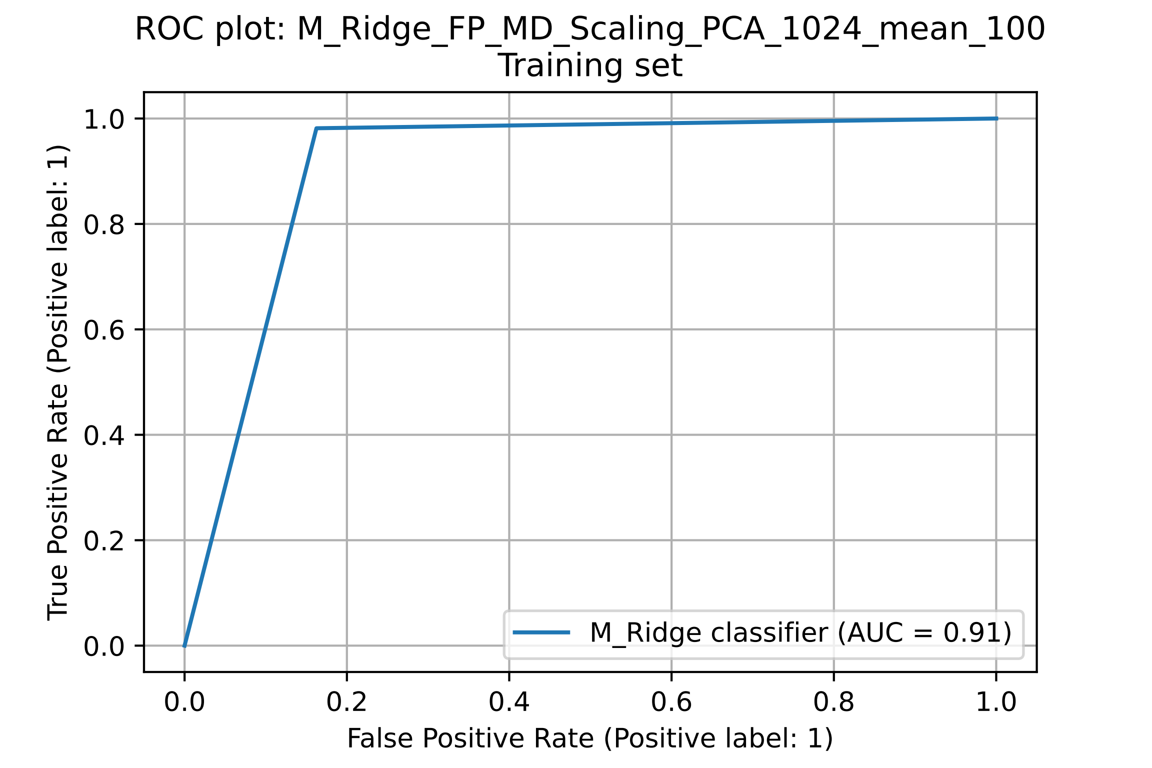
ROC plot: M_Ridge_FP_MD_Scaling_PCA_1024_mean_100
Training set
True Positive Rate (Positive label: 1)
False Positive Rate (Positive label: 1)
0.0
0.2
0.4
0.6
0.8
1.0
M_Ridge classifier (AUC = 0.91)

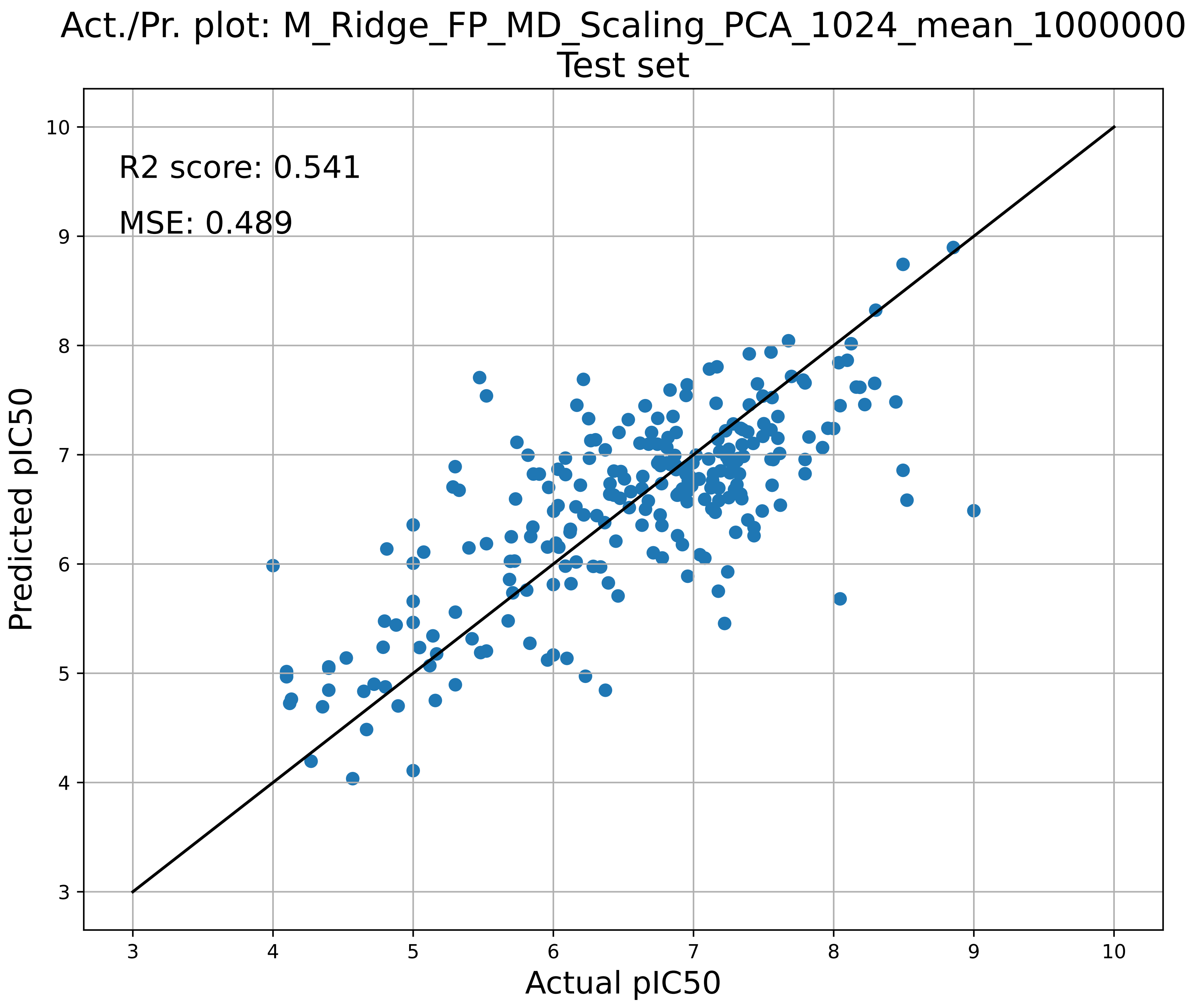

Act./Pr. plot: M_Ridge_FP_MD_Scaling_PCA_1024_mean_1000000
Test set
R2 score: 0.541
MSE: 0.489
Predicted pIC50
Actual pIC50
3
4
5
6
7
8
9
10

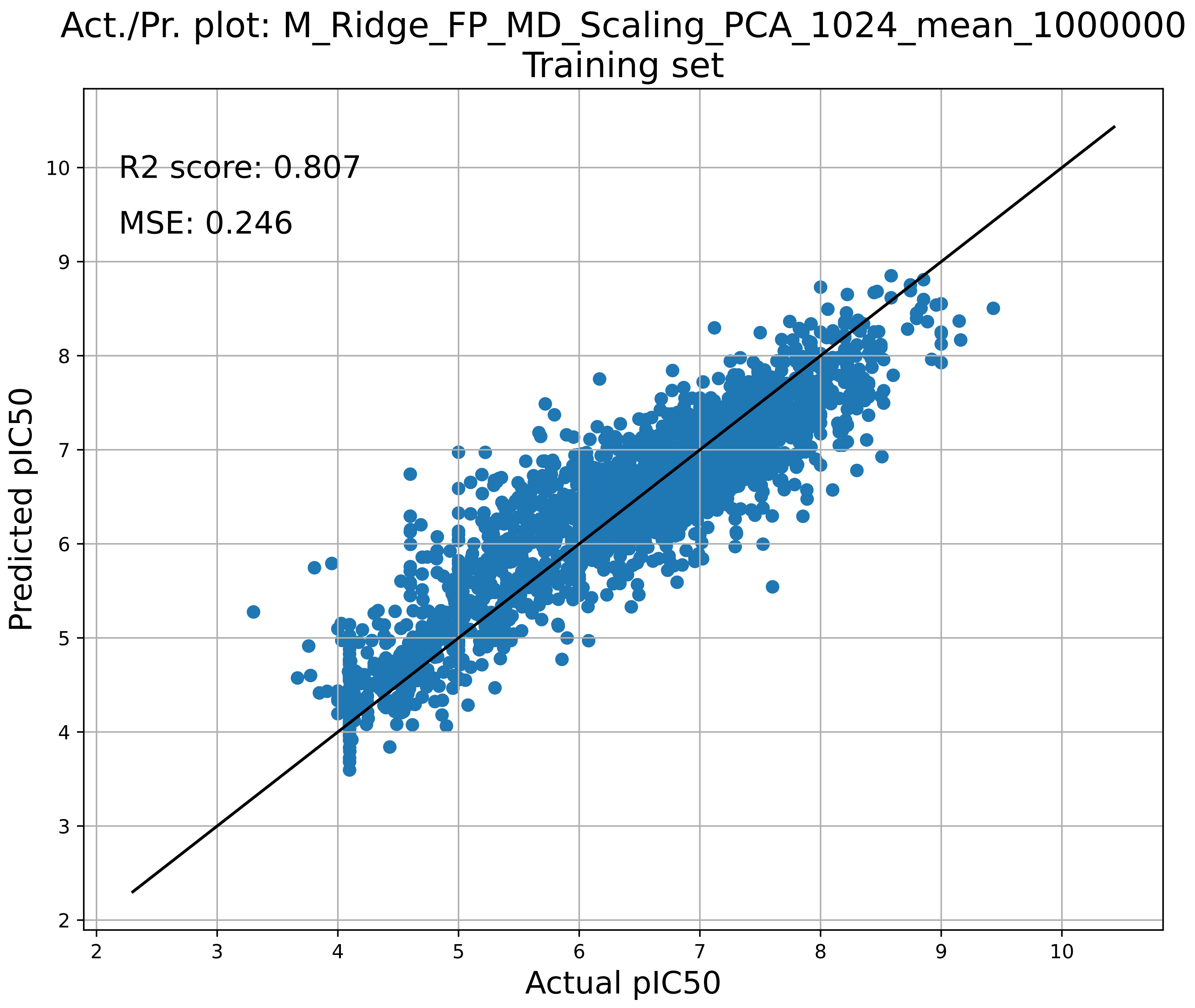
Act./Pr. plot: M_Ridge_FP_MD_Scaling_PCA_1024_mean_1000000
Training set
R2 score: 0.807
MSE: 0.246
Predicted pIC50
Actual pIC50
2
3
4
5
6
7
8
9
10
2
3
4
5
6
7
8
9
10

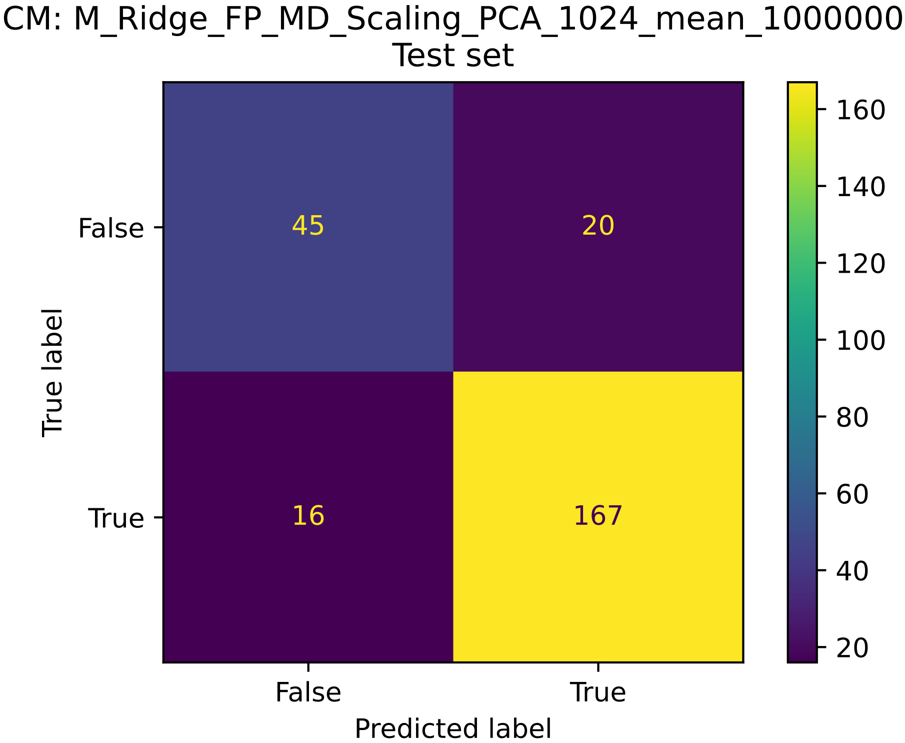
CM: M_Ridge_FP_MD_Scaling_PCA_1024_mean_1000000
Test set
True label
Predicted label
False
True
False
True
45
20
16
167
160
140
120
100
80
60
40
20

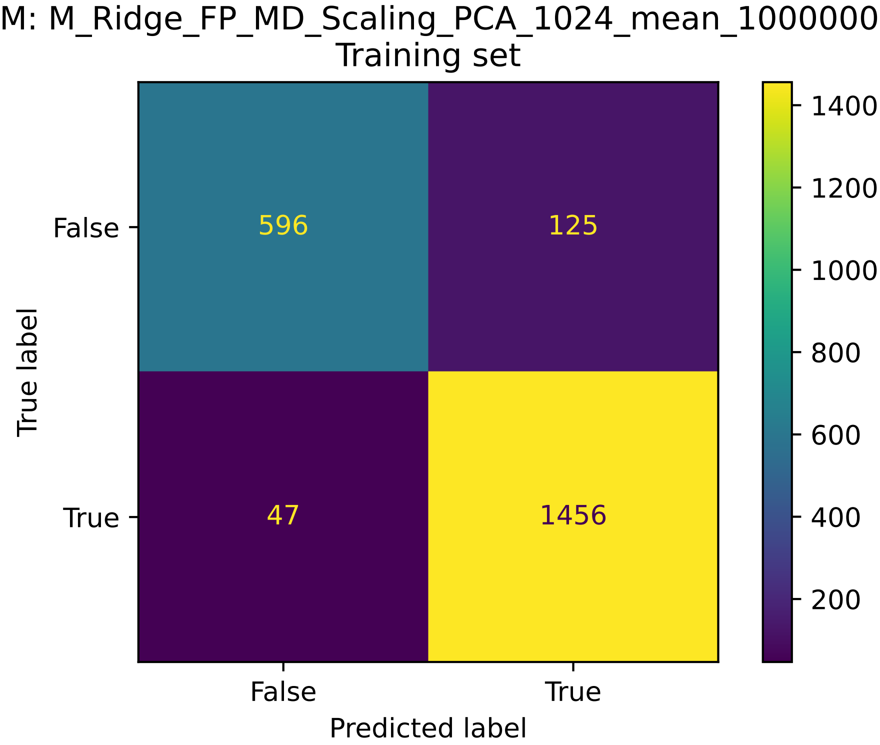
CM: M_Ridge_FP_MD_Scaling_PCA_1024_mean_1000000
Training set
True label
False
True
596
125
47
1456
Predicted label
False
True
1400
1200
1000
800
600
400
200

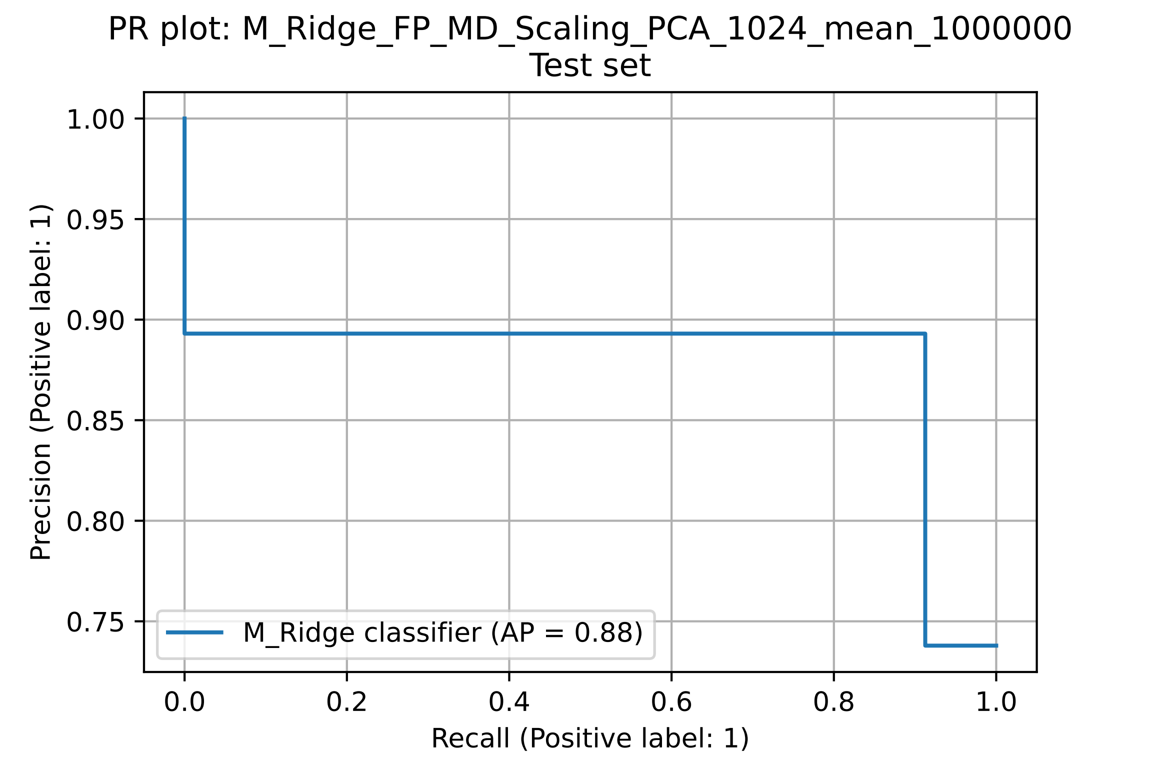

PR plot: M_Ridge_FP_MD_Scaling_PCA_1024_mean_1000000
Test set
Precision (Positive label: 1)
Recall (Positive label: 1)
1.00
0.95
0.90
0.85
0.80
0.75
0.0
0.2
0.4
0.6
0.8
1.0
M_Ridge classifier (AP = 0.88)

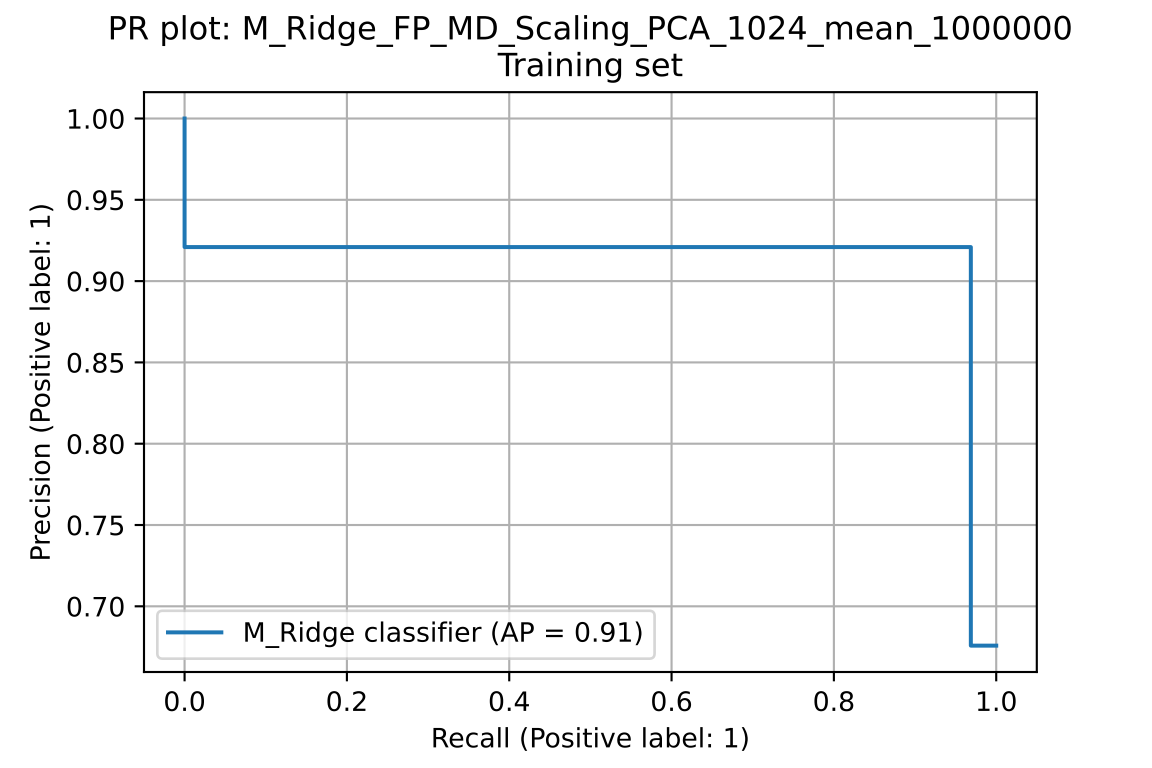
PR plot: M_Ridge_FP_MD_Scaling_PCA_1024_mean_1000000
Training set
Precision (Positive label: 1)
1.00
0.95
0.90
0.85
0.80
0.75
0.70
0.0
0.2
0.4
0.6
0.8
1.0
Recall (Positive label: 1)
M_Ridge classifier (AP = 0.91)

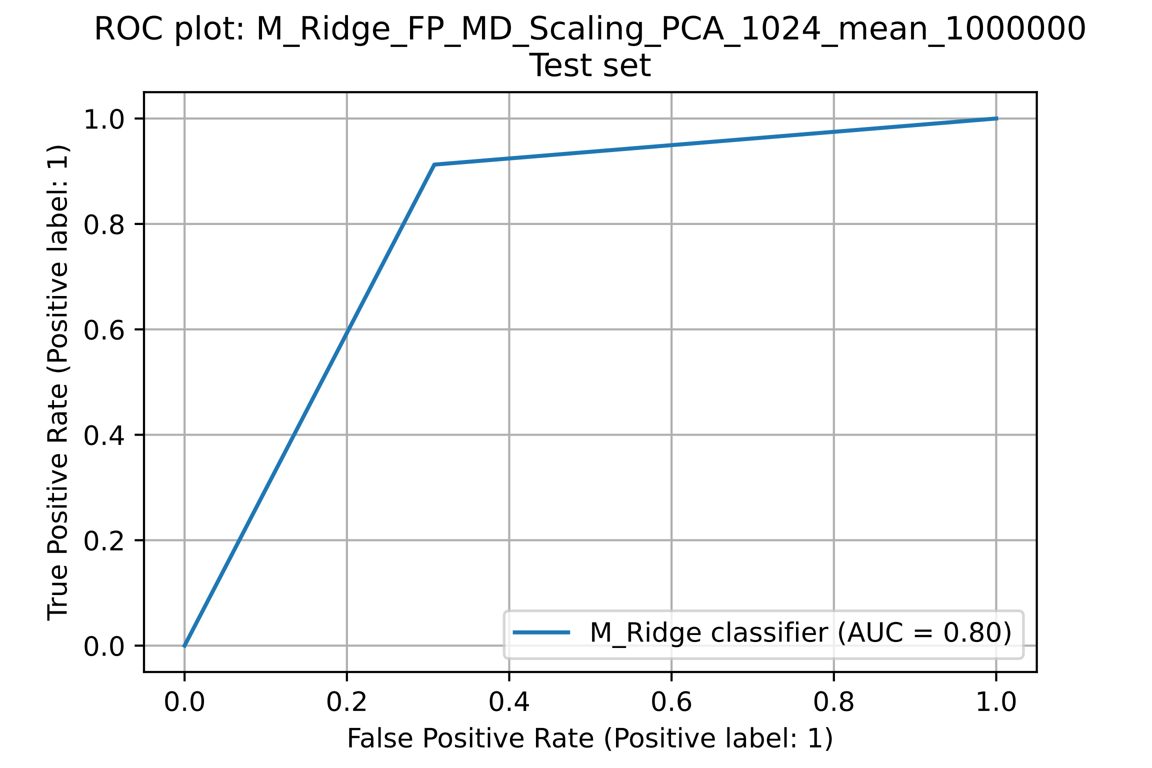
ROC plot: M_Ridge_FP_MD_Scaling_PCA_1024_mean_1000000
Test set
True Positive Rate (Positive label: 1)
False Positive Rate (Positive label: 1)
0.0
0.2
0.4
0.6
0.8
1.0
M_Ridge classifier (AUC = 0.80)

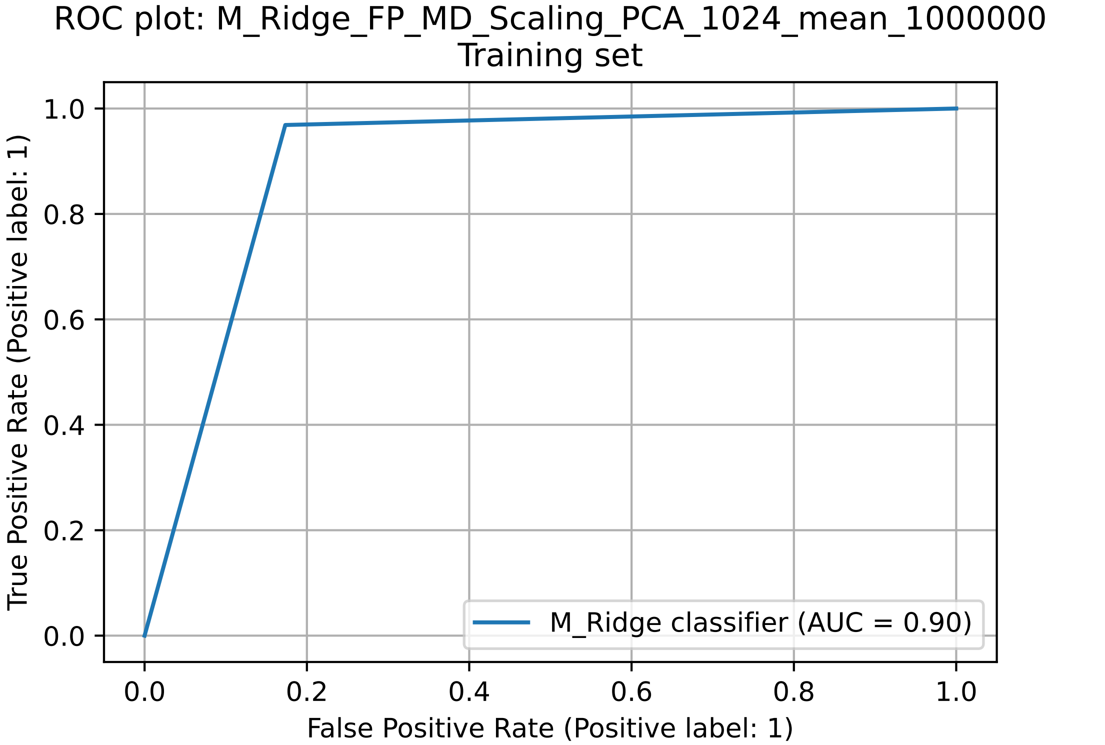

ROC plot: M_Ridge_FP_MD_Scaling_PCA_1024_mean_1000000
Training set
True Positive Rate (Positive label: 1)
False Positive Rate (Positive label: 1)
0.0
0.2
0.4
0.6
0.8
1.0
M_Ridge classifier (AUC = 0.90)

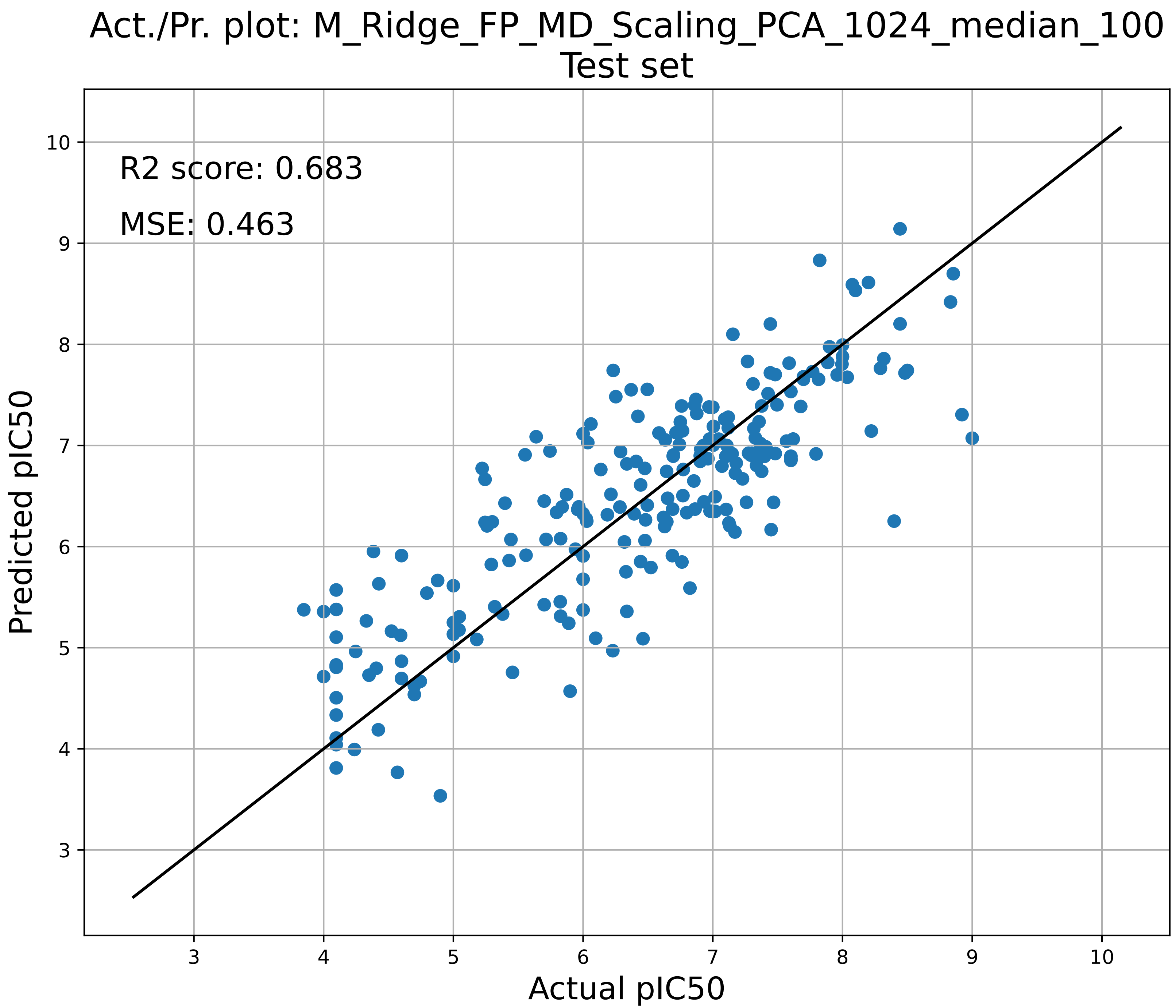

Act./Pr. plot: M_Ridge_FP_MD_Scaling_PCA_1024_median_100
Test set
R2 score: 0.683
MSE: 0.463
Predicted pIC50
Actual pIC50
3
4
5
6
7
8
9
10

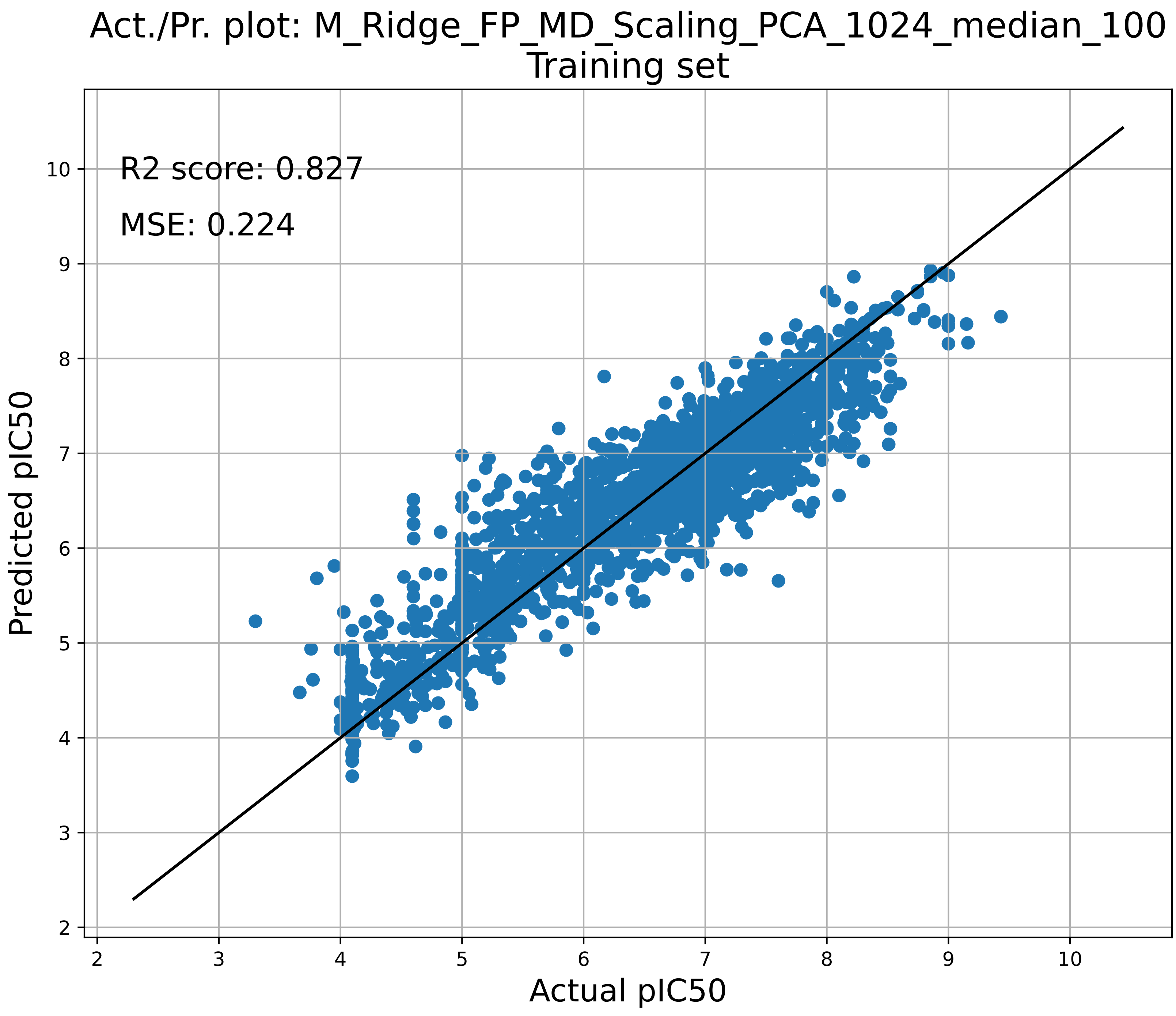

Act./Pr. plot: M_Ridge_FP_MD_Scaling_PCA_1024_median_100
Training set
R2 score: 0.827
MSE: 0.224
Predicted pIC50
Actual pIC50
2
3
4
5
6
7
8
9
10

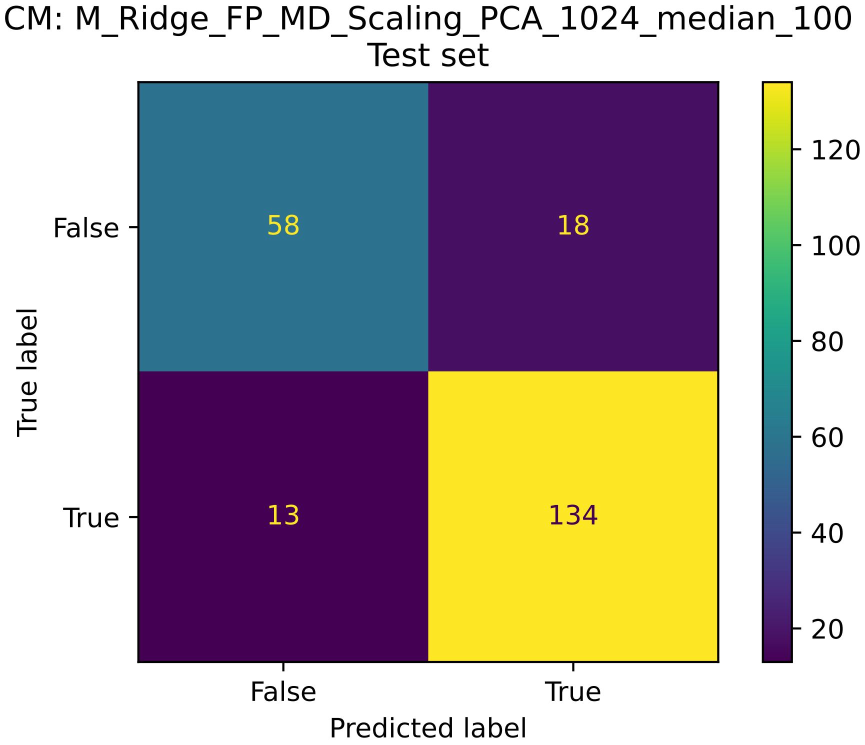
CM: M_Ridge_FP_MD_Scaling_PCA_1024_median_100
Test set
True label
Predicted label
False
True
False
True
58
18
13
134
20
40
60
80
100
120

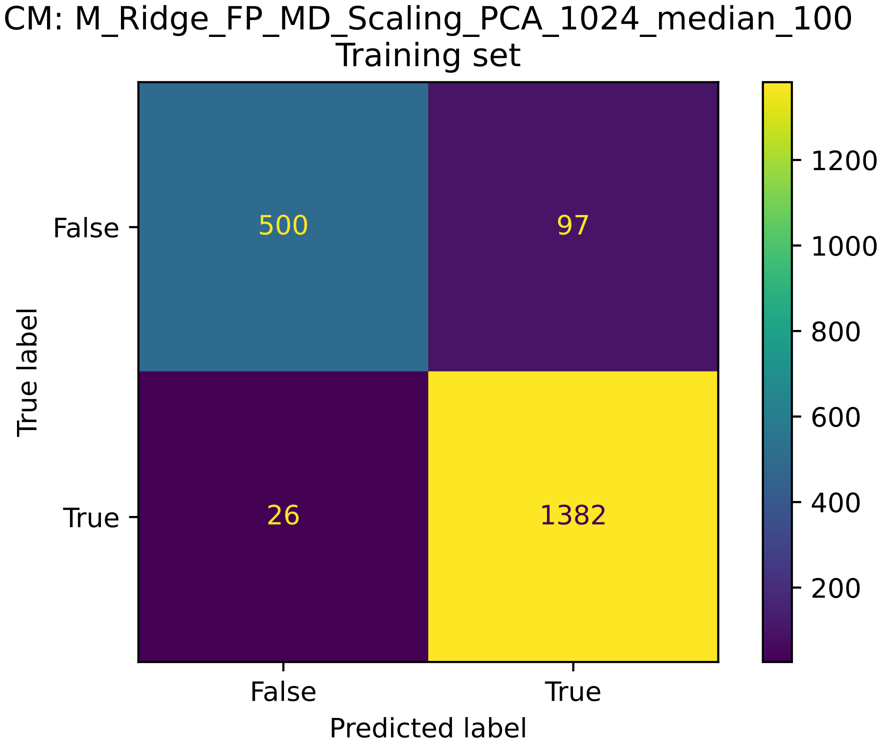
CM: M_Ridge_FP_MD_Scaling_PCA_1024_median_100
Training set
True label
False
True
500
97
26
1382
Predicted label
False
True
1200
1000
800
600
400
200

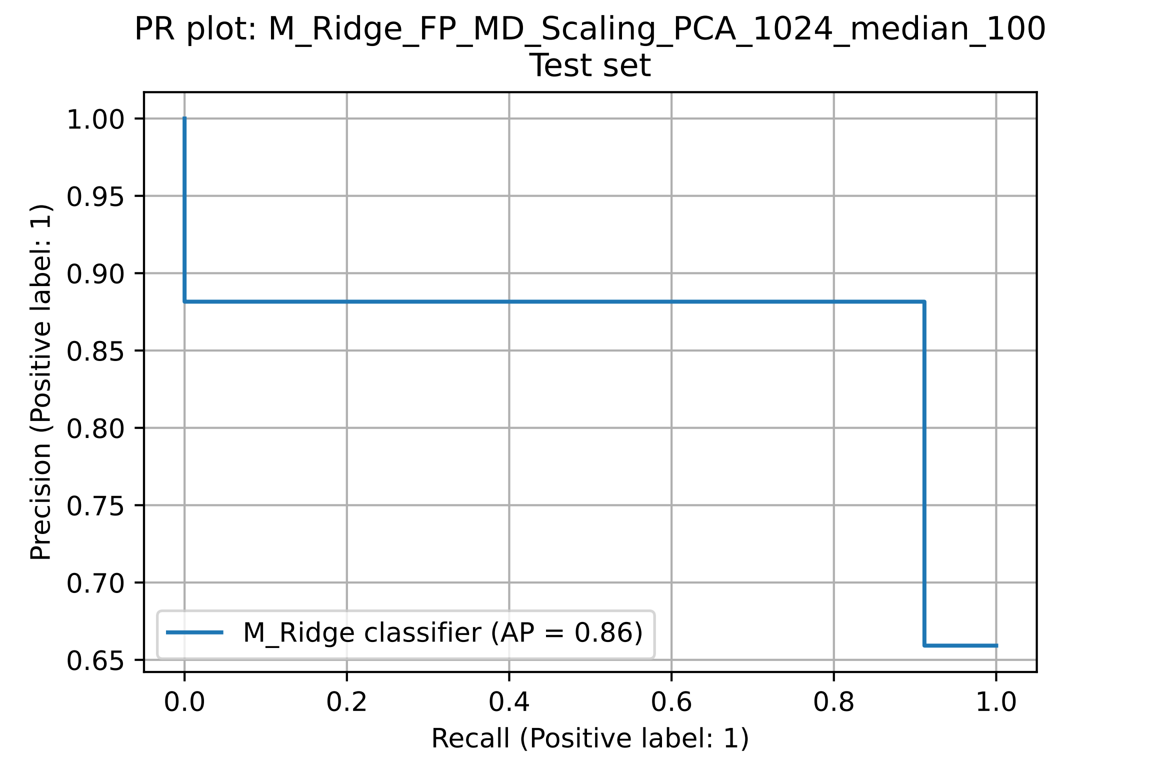
PR plot: M_Ridge_FP_MD_Scaling_PCA_1024_median_100
Test set
Precision (Positive label: 1)
Recall (Positive label: 1)
1.00
0.95
0.90
0.85
0.80
0.75
0.70
0.65
0.0
0.2
0.4
0.6
0.8
1.0
M_Ridge classifier (AP = 0.86)

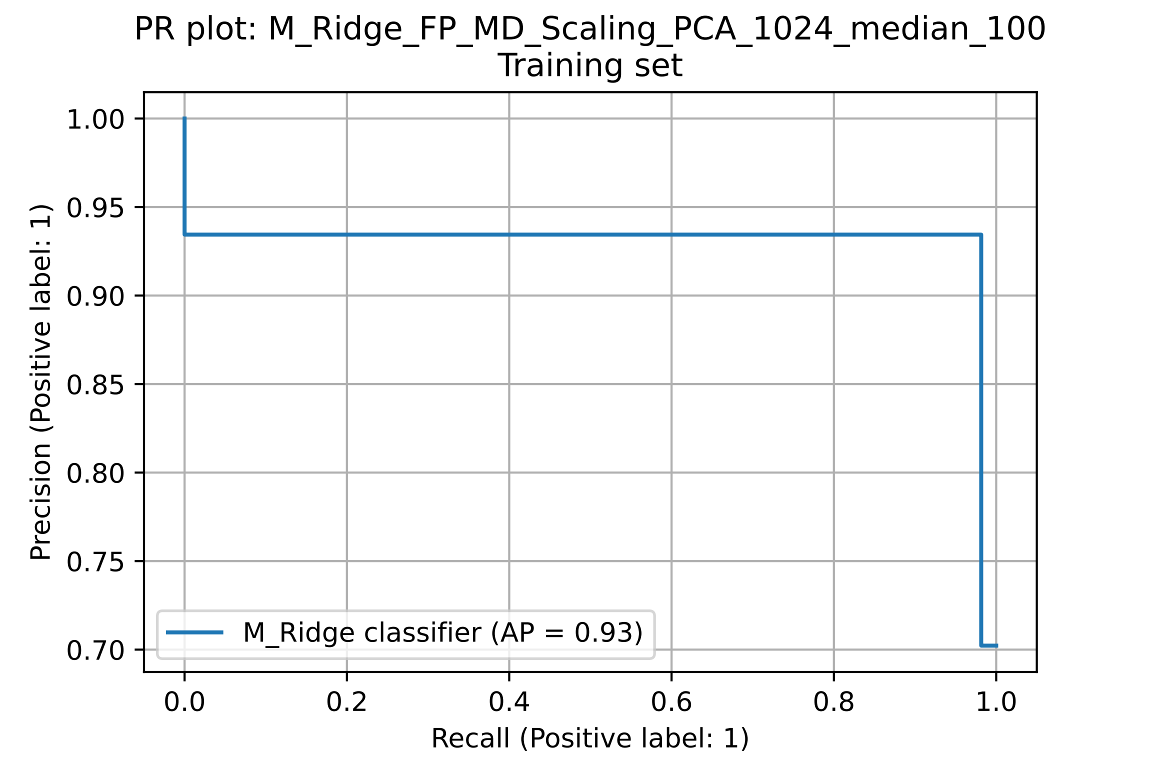
PR plot: M_Ridge_FP_MD_Scaling_PCA_1024_median_100
Training set
1.00
0.95
0.90
0.85
0.80
0.75
0.70
Precision (Positive label: 1)
0.0
0.2
0.4
0.6
0.8
1.0
Recall (Positive label: 1)
M_Ridge classifier (AP = 0.93)

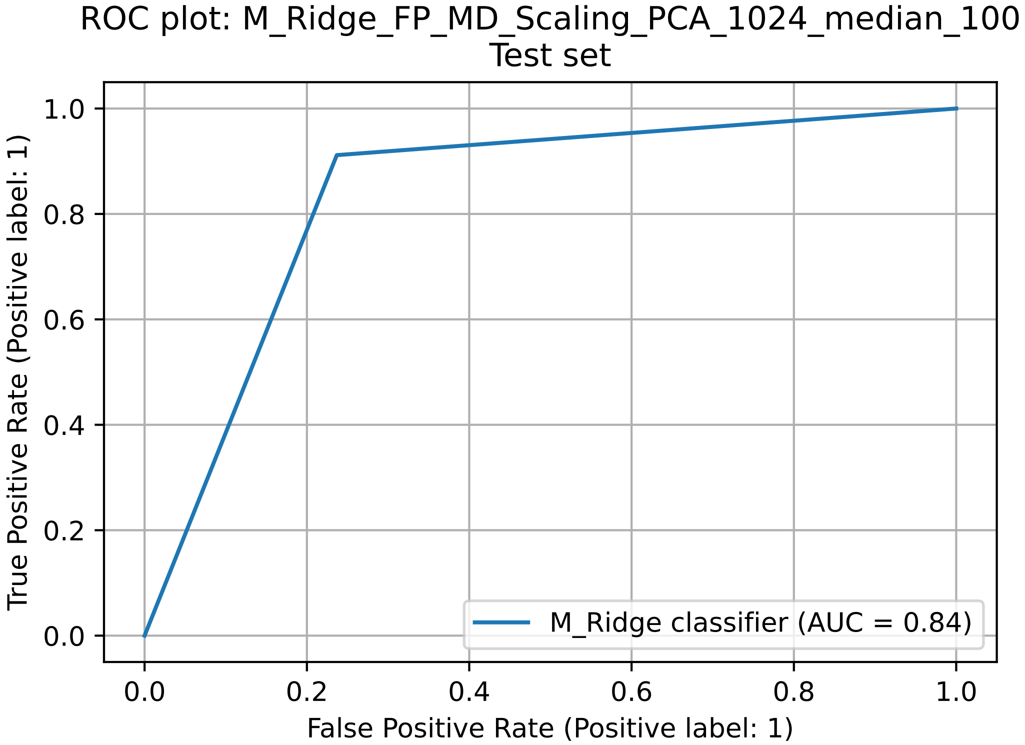
ROC plot: M_Ridge_FP_MD_Scaling_PCA_1024_median_100
Test set
True Positive Rate (Positive label: 1)
False Positive Rate (Positive label: 1)
0.0
0.2
0.4
0.6
0.8
1.0
M_Ridge classifier (AUC = 0.84)

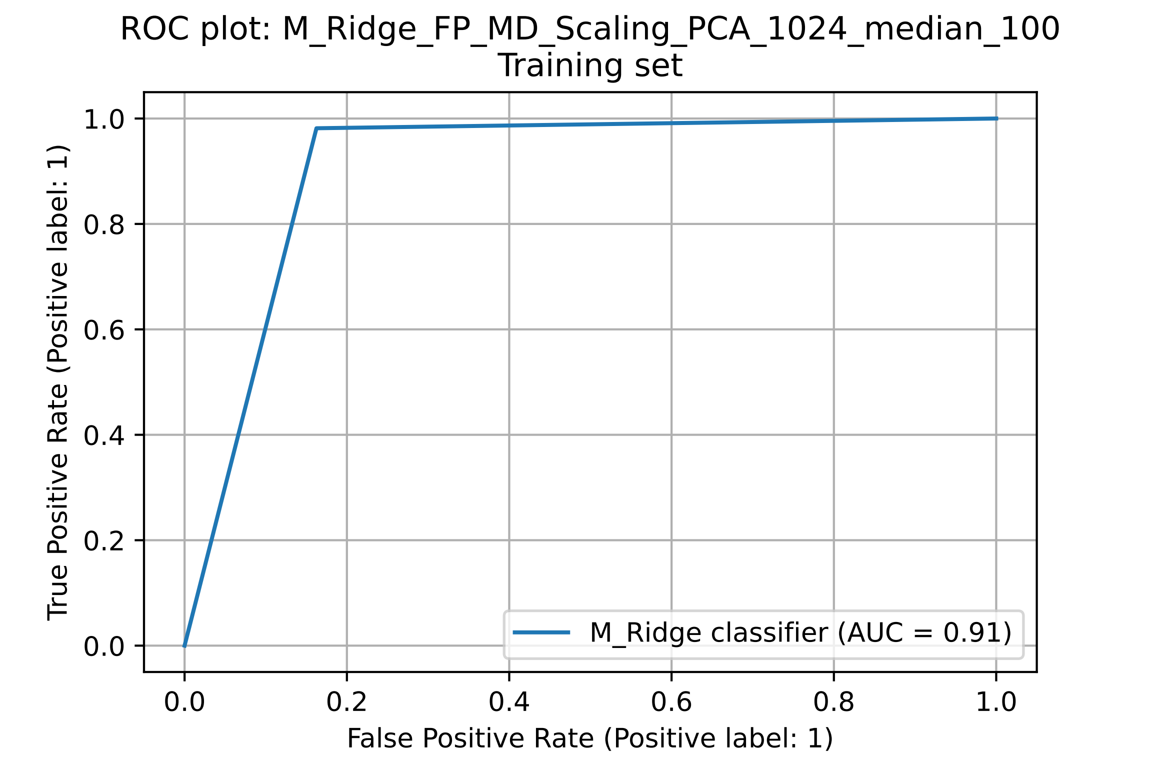
ROC plot: M_Ridge_FP_MD_Scaling_PCA_1024_median_100
Training set
True Positive Rate (Positive label: 1)
False Positive Rate (Positive label: 1)
0.0
0.2
0.4
0.6
0.8
1.0
M_Ridge classifier (AUC = 0.91)

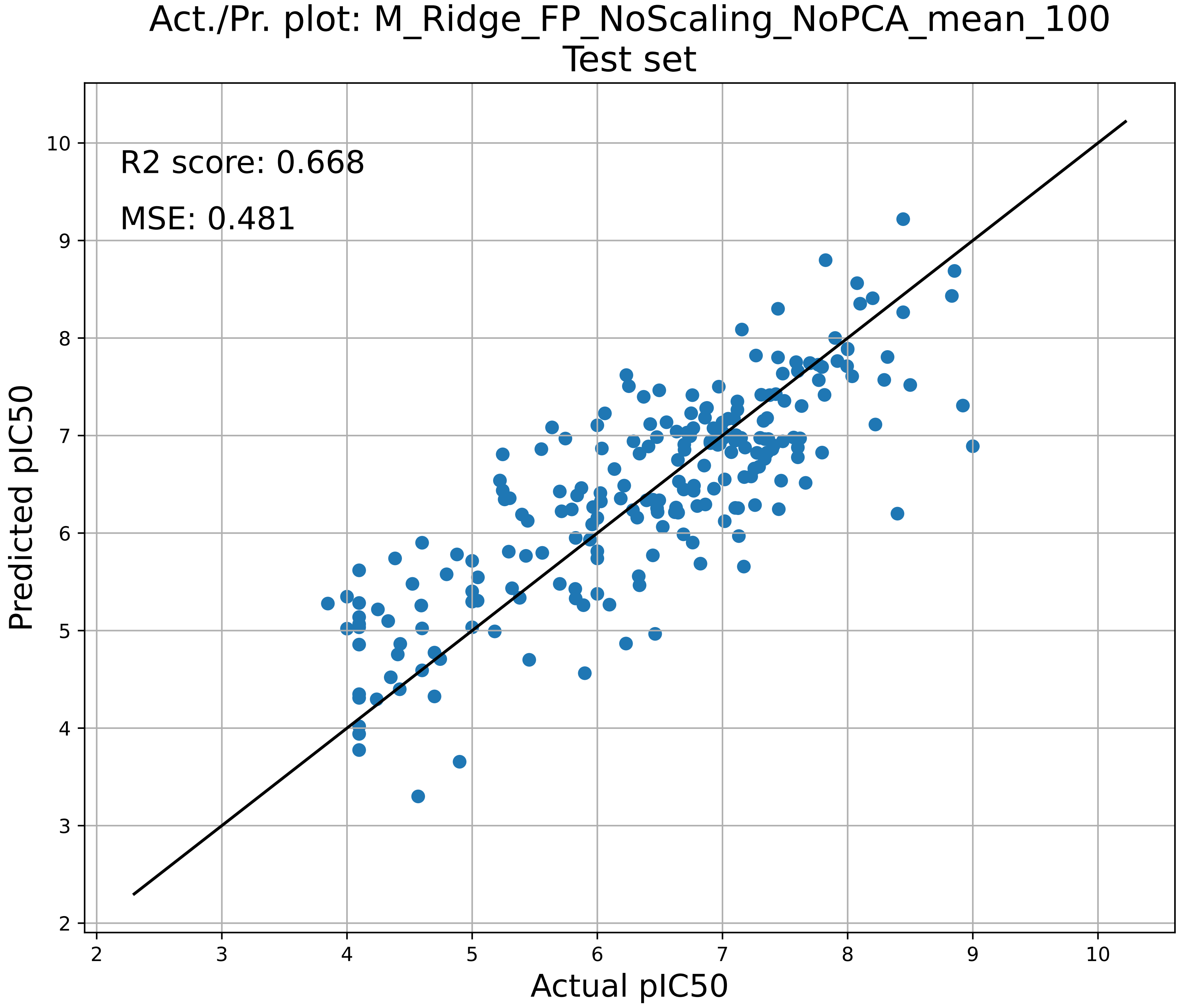

Act./Pr. plot: M_Ridge_FP_NoScaling_NoPCA_mean_100
Test set
R2 score: 0.668
MSE: 0.481
Predicted pIC50
Actual pIC50
2
3
4
5
6
7
8
9
10

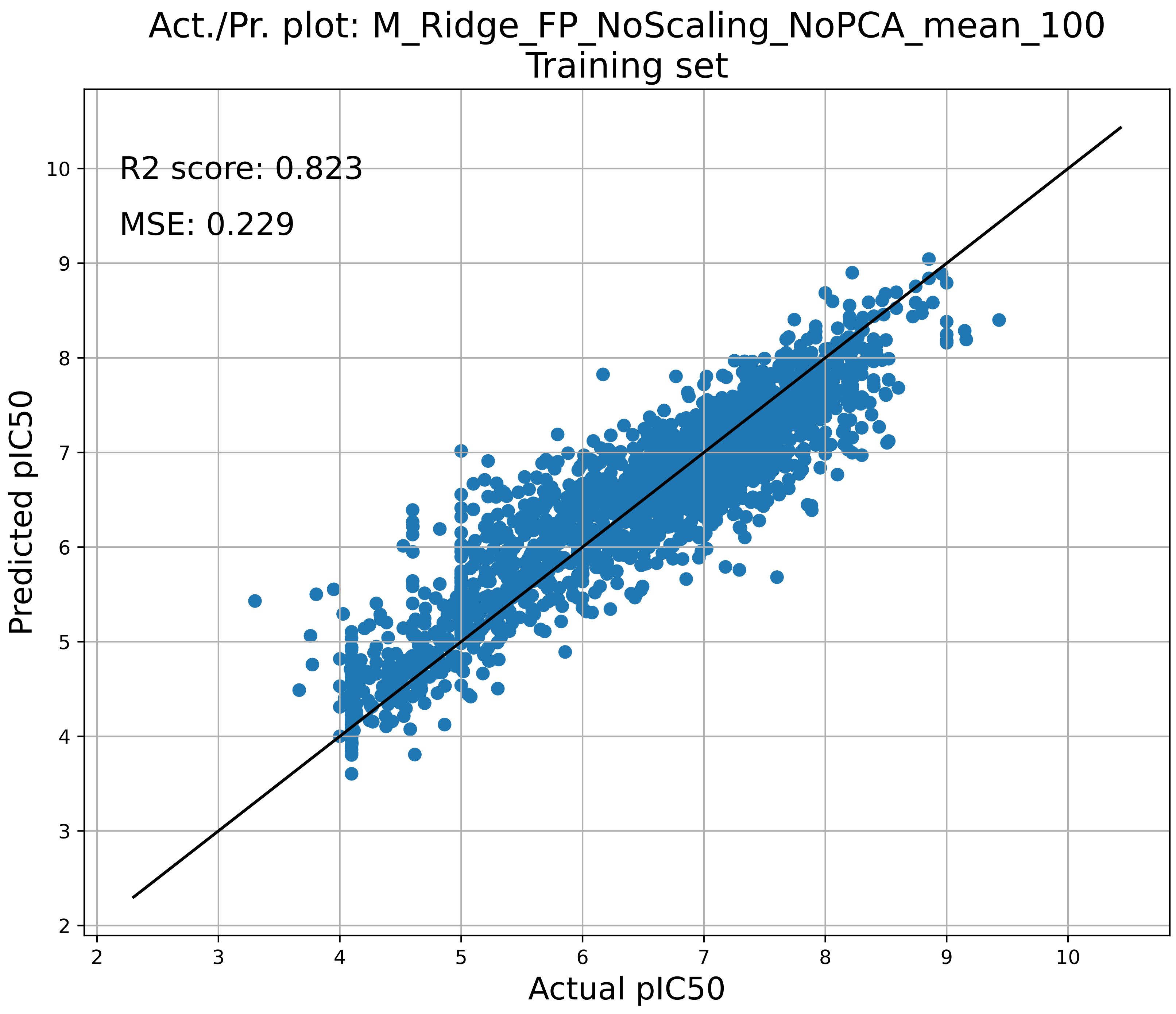
Act./Pr. plot: M_Ridge_FP_NoScaling_NoPCA_mean_100
Training set
R2 score: 0.823
MSE: 0.229
Predicted pIC50
Actual pIC50
2
3
4
5
6
7
8
9
10

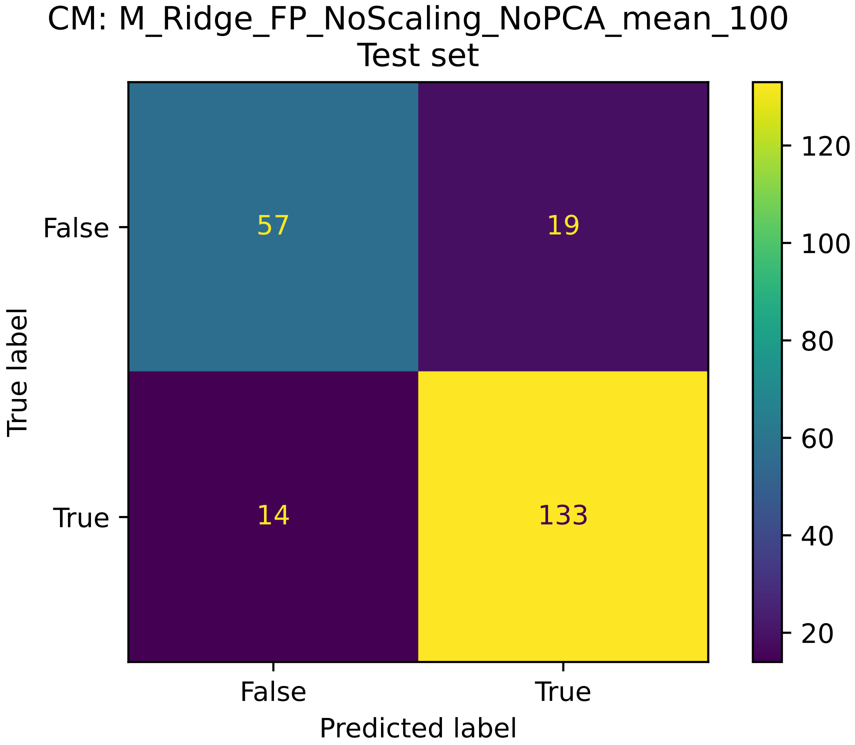

CM: M_Ridge_FP_NoScaling_NoPCA_mean_100
Test set
True label
Predicted label
False
True
False
True
57
19
14
133
20
40
60
80
100
120

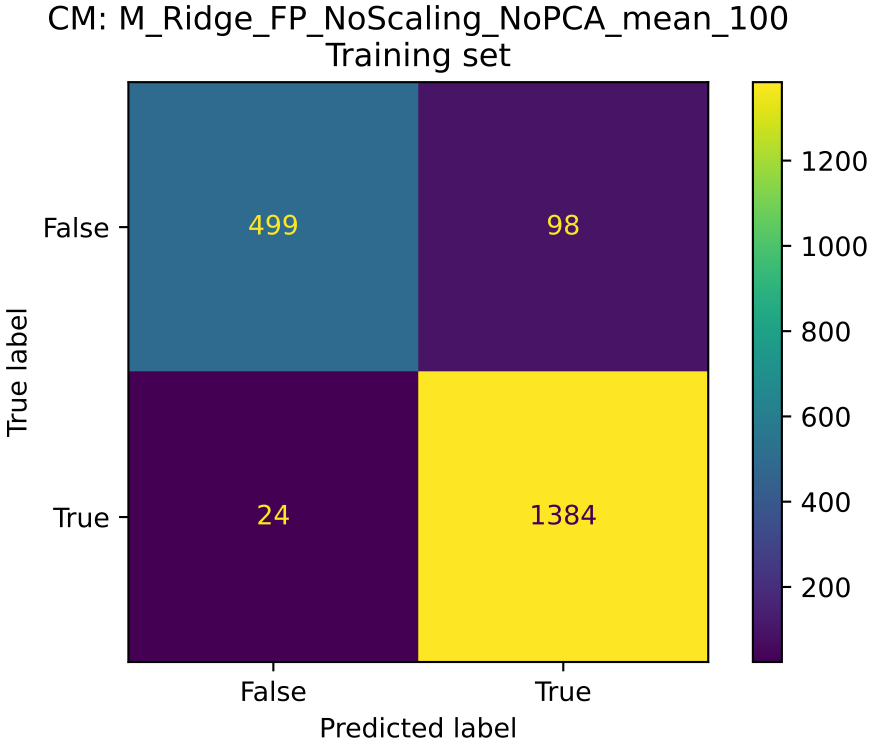
CM: M_Ridge_FP_NoScaling_NoPCA_mean_100
Training set
499
98
24
1384
False
True
True label
False
True
Predicted label
1200
1000
800
600
400
200

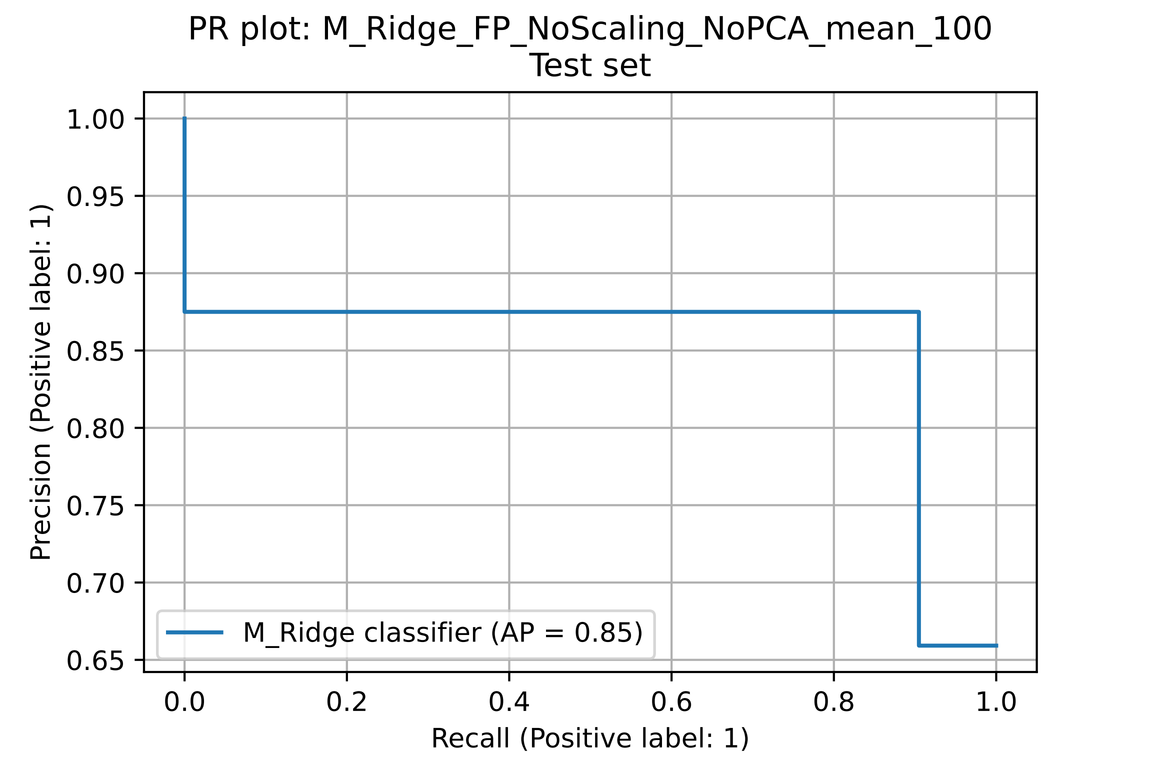
PR plot: M_Ridge_FP_NoScaling_NoPCA_mean_100
Test set
Precision (Positive label: 1)
1.00
0.95
0.90
0.85
0.80
0.75
0.70
0.65
0.0
0.2
0.4
0.6
0.8
1.0
Recall (Positive label: 1)
M_Ridge classifier (AP = 0.85)

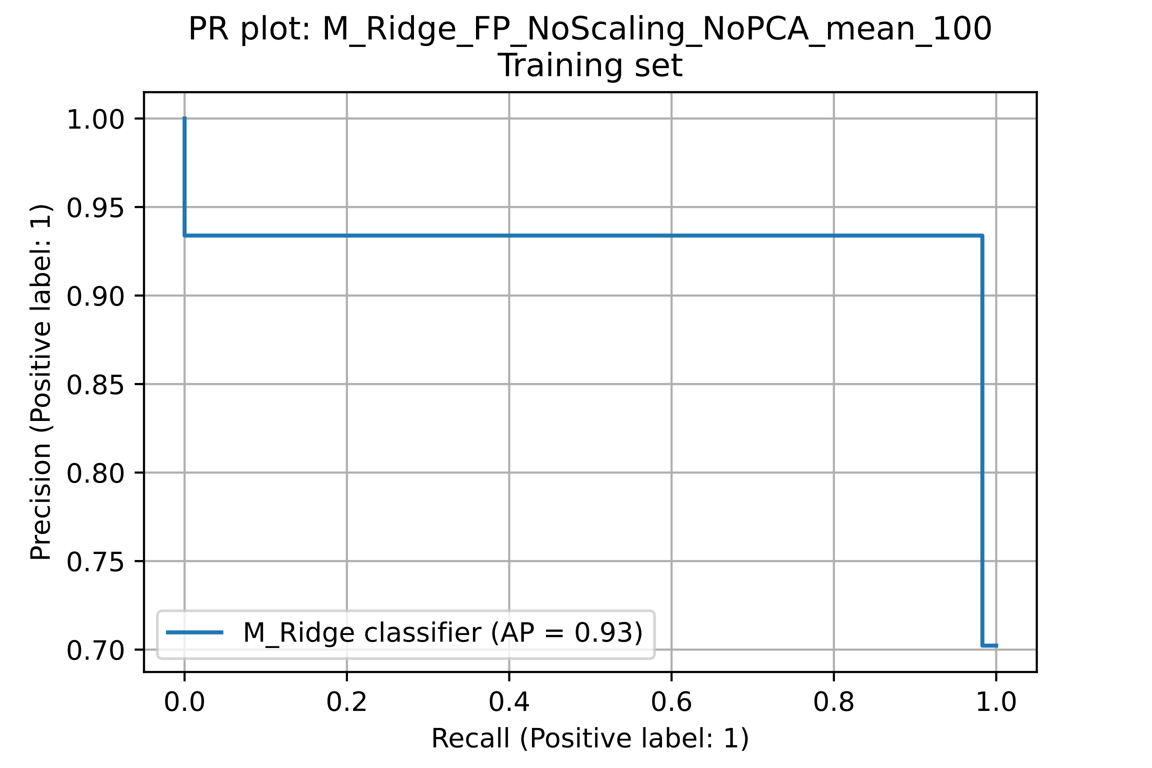
PR plot: M_Ridge_FP_NoScaling_NoPCA_mean_100
Training set
Precision (Positive label: 1)
Recall (Positive label: 1)
1.00
0.95
0.90
0.85
0.80
0.75
0.70
0.0
0.2
0.4
0.6
0.8
1.0
M_Ridge classifier (AP = 0.93)

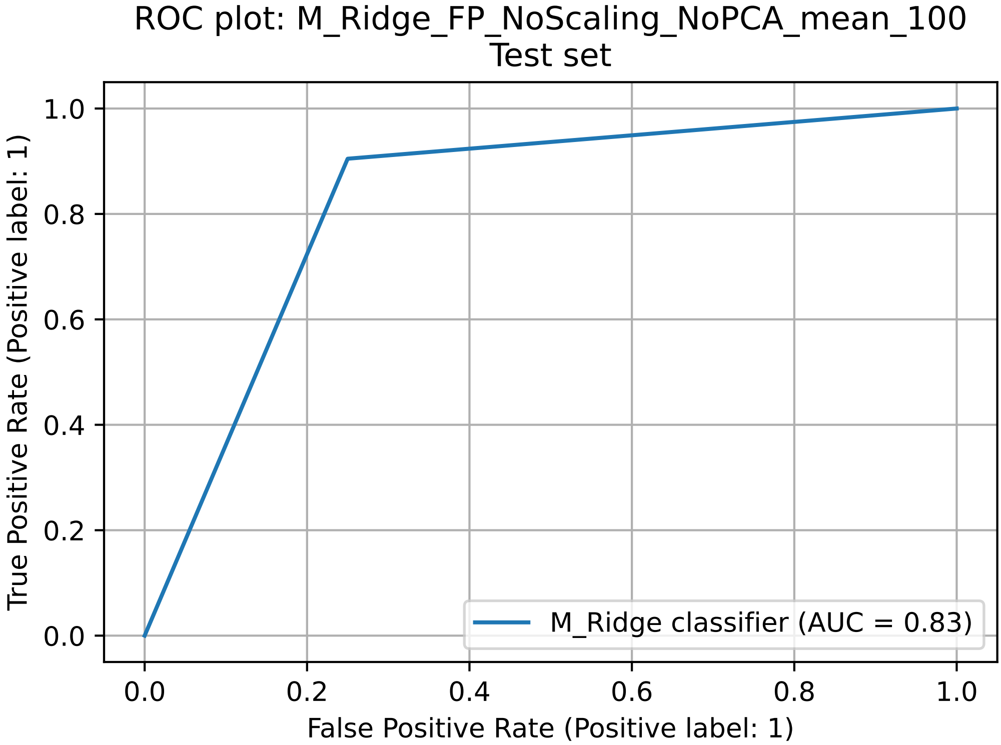
ROC plot: M_Ridge_FP_NoScaling_NoPCA_mean_100
Test set
True Positive Rate (Positive label: 1)
False Positive Rate (Positive label: 1)
0.0
0.2
0.4
0.6
0.8
1.0
M_Ridge classifier (AUC = 0.83)

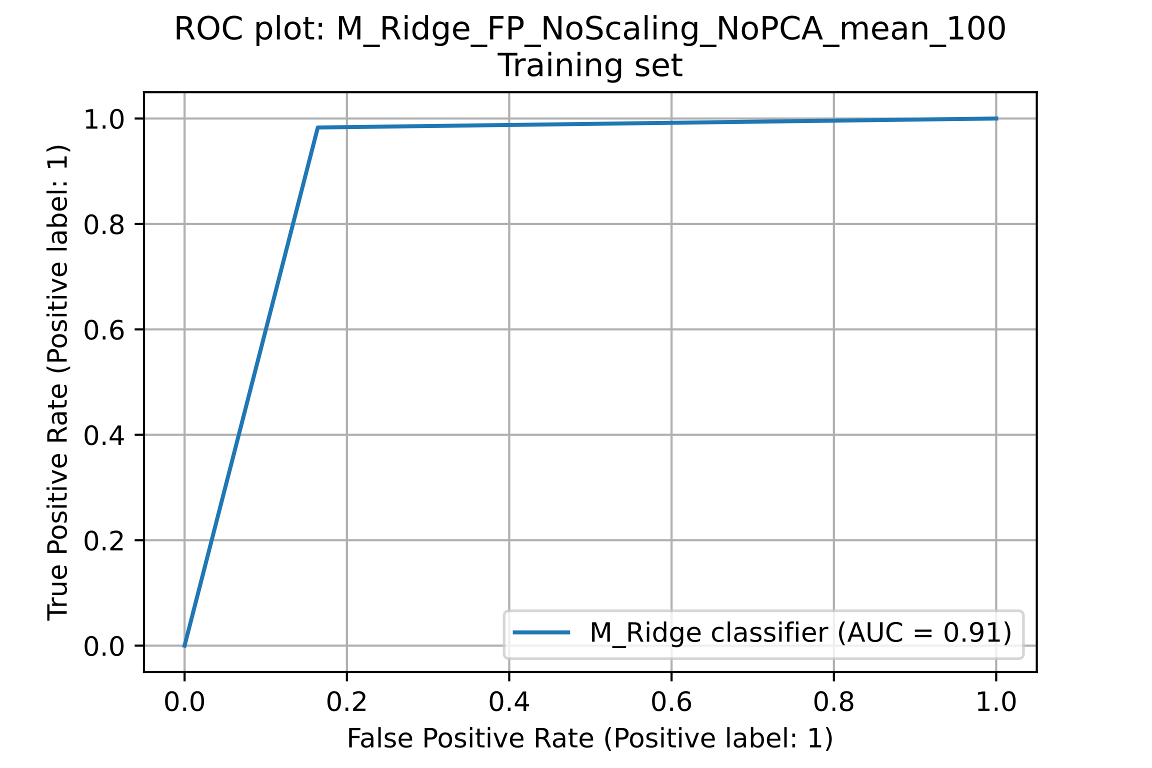
ROC plot: M_Ridge_FP_NoScaling_NoPCA_mean_100
Training set
True Positive Rate (Positive label: 1)
False Positive Rate (Positive label: 1)
0.0
0.2
0.4
0.6
0.8
1.0
M_Ridge classifier (AUC = 0.91)

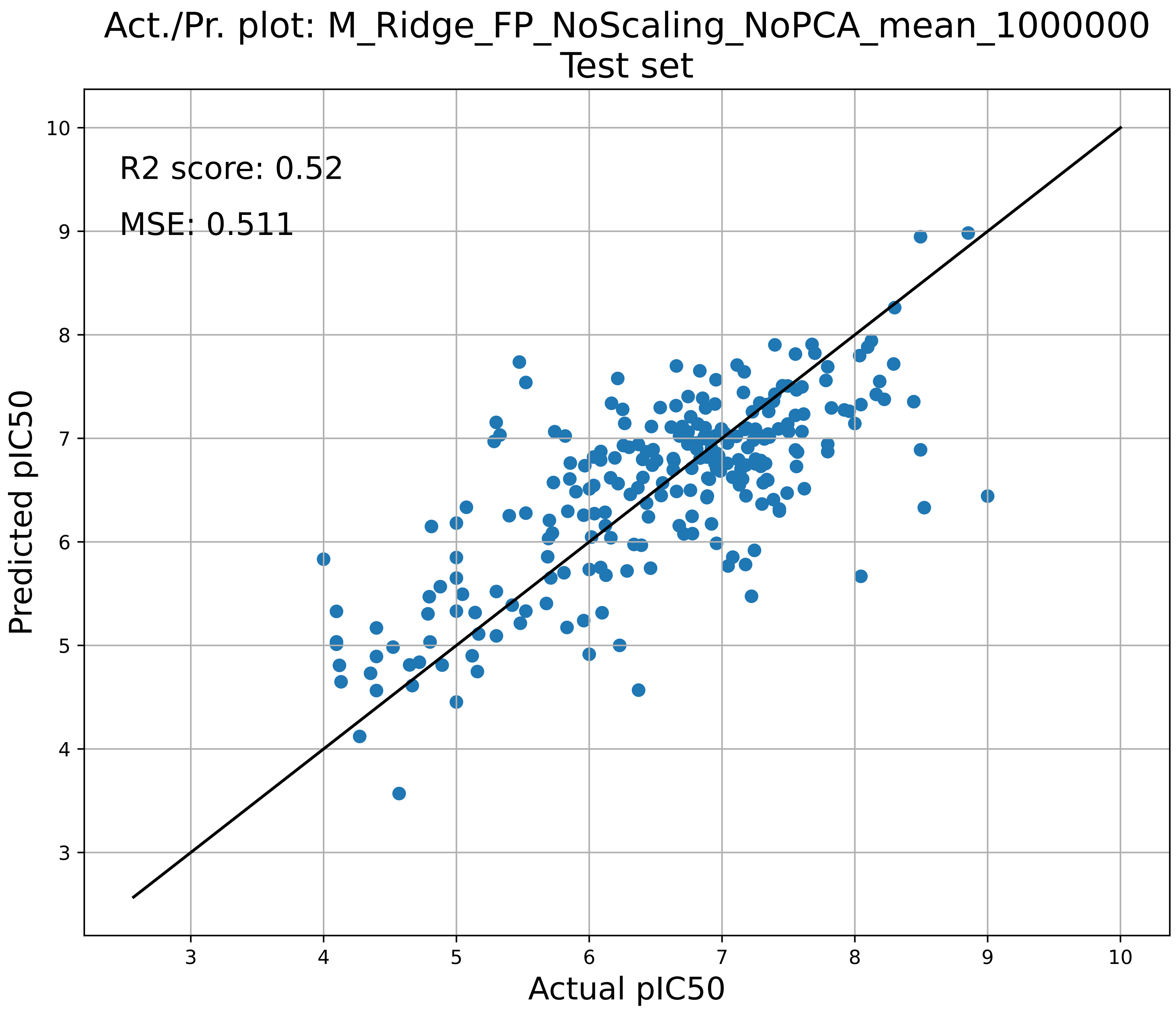

Act./Pr. plot: M_Ridge_FP_NoScaling_NoPCA_mean_1000000
Test set
R2 score: 0.52
MSE: 0.511
Predicted pIC50
Actual pIC50
3
4
5
6
7
8
9
10

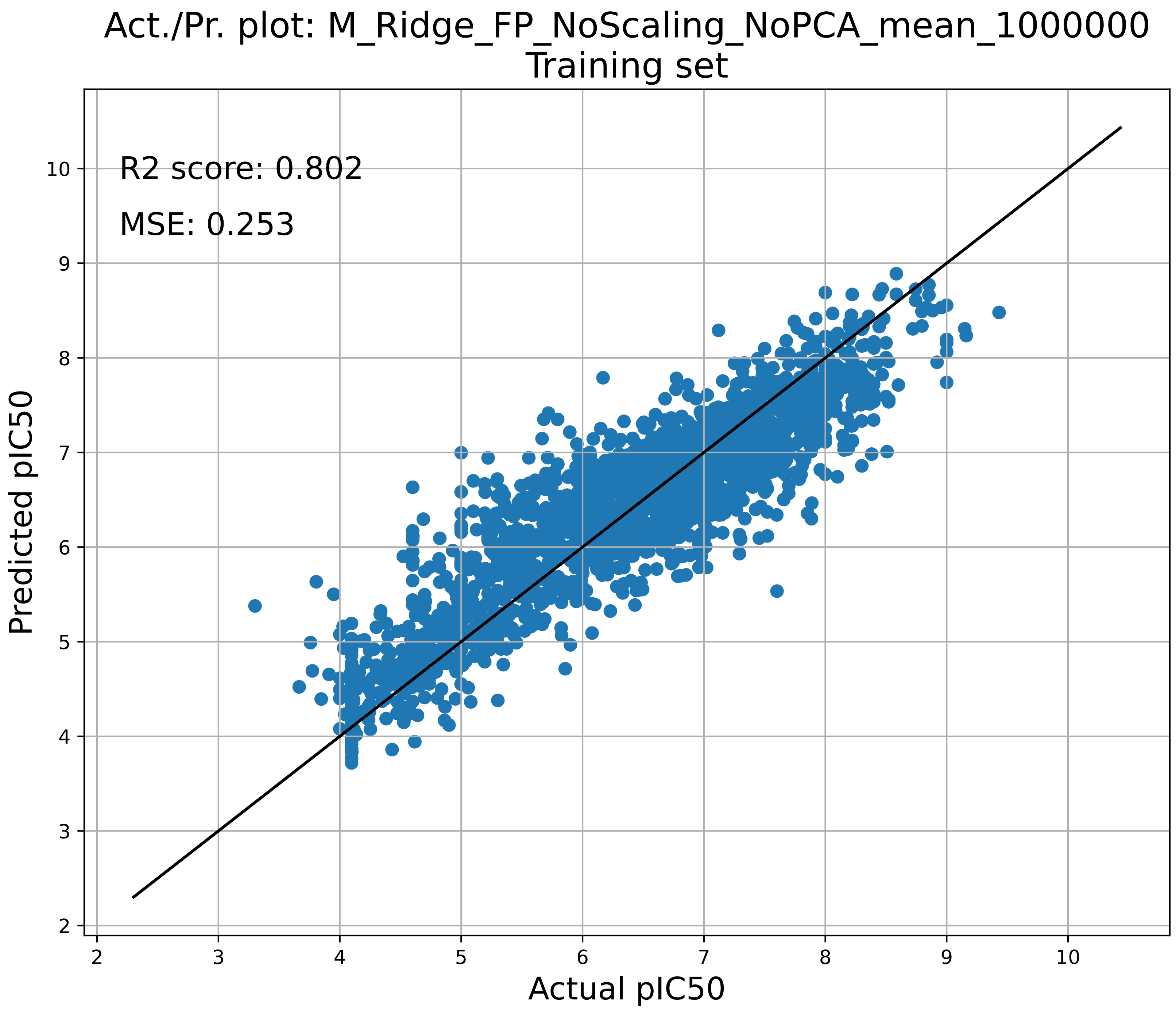

Act./Pr. plot: M_Ridge_FP_NoScaling_NoPCA_mean_1000000
Training set
R2 score: 0.802
MSE: 0.253
Predicted pIC50
Actual pIC50
2
3
4
5
6
7
8
9
10

CM: M_Ridge_FP_NoScaling_NoPCA_mean_1000000
Test set

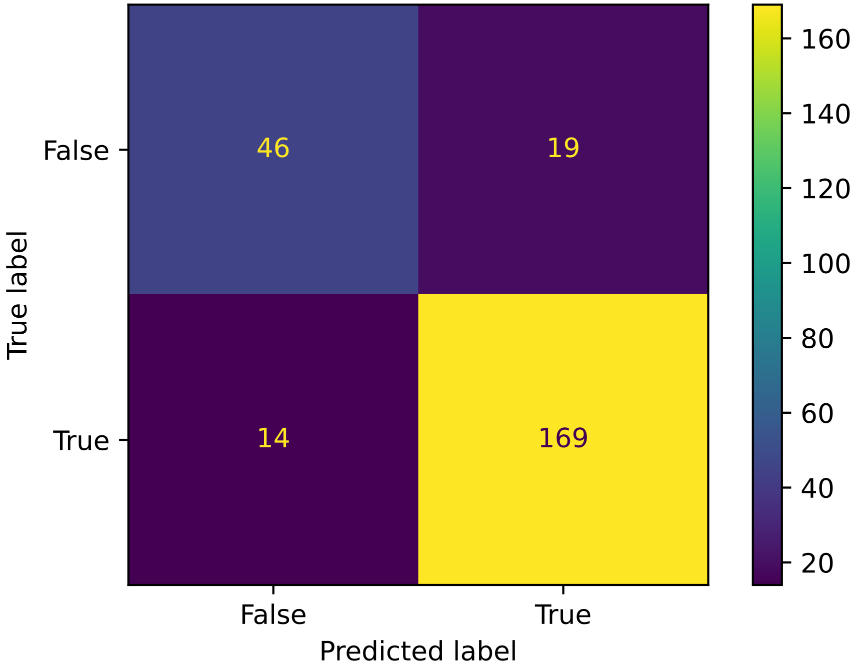

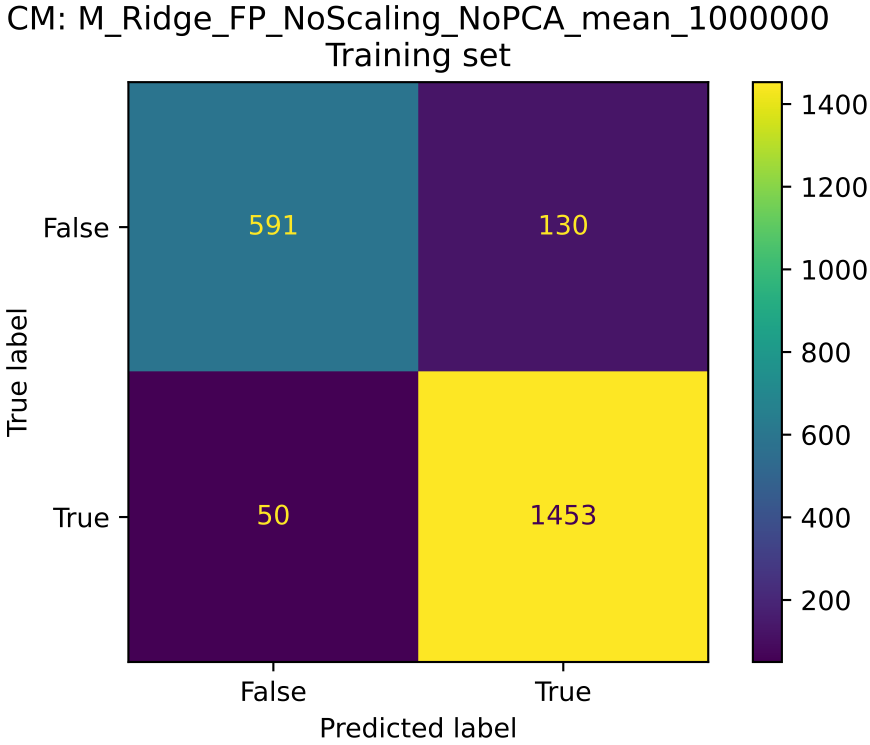
CM: M_Ridge_FP_NoScaling_NoPCA_mean_1000000
Training set
True label
Predicted label
False
True
False
True
591
130
50
1453
1400
1200
1000
800
600
400
200

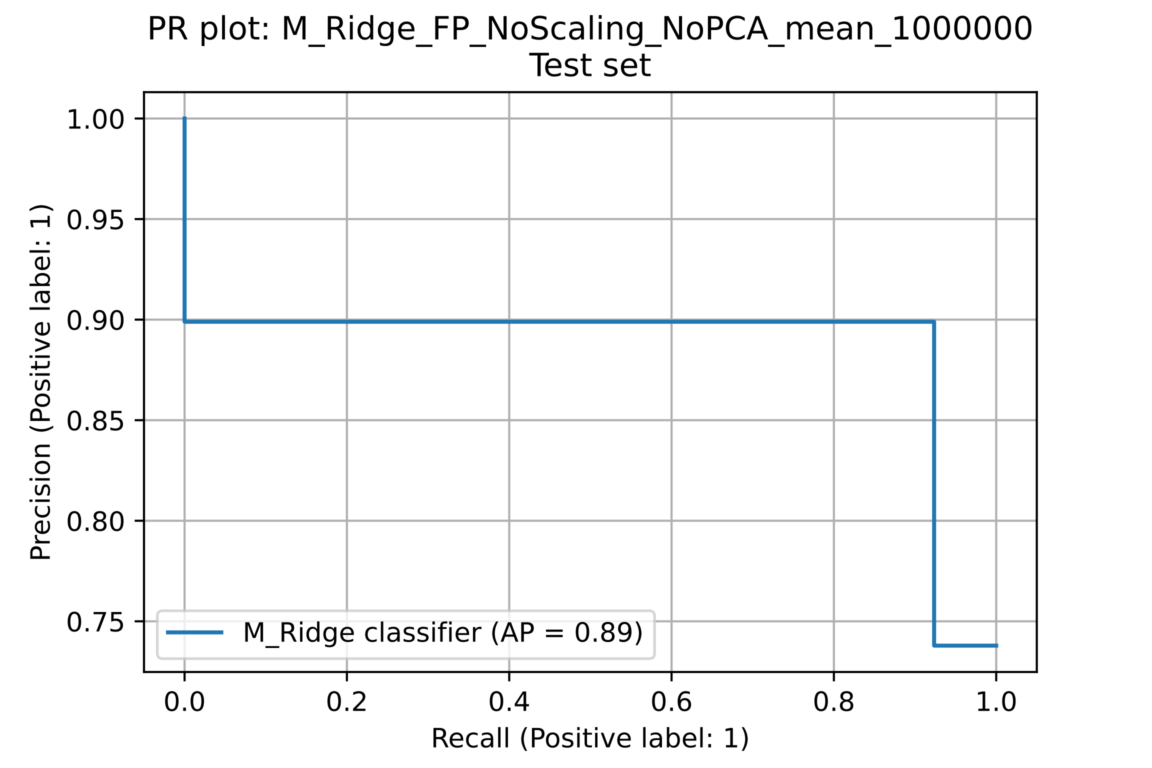

PR plot: M_Ridge_FP_NoScaling_NoPCA_mean_1000000
Test set
Precision (Positive label: 1)
1.00
0.95
0.90
0.85
0.80
0.75
0.0
0.2
0.4
0.6
0.8
1.0
Recall (Positive label: 1)
M_Ridge classifier (AP = 0.89)

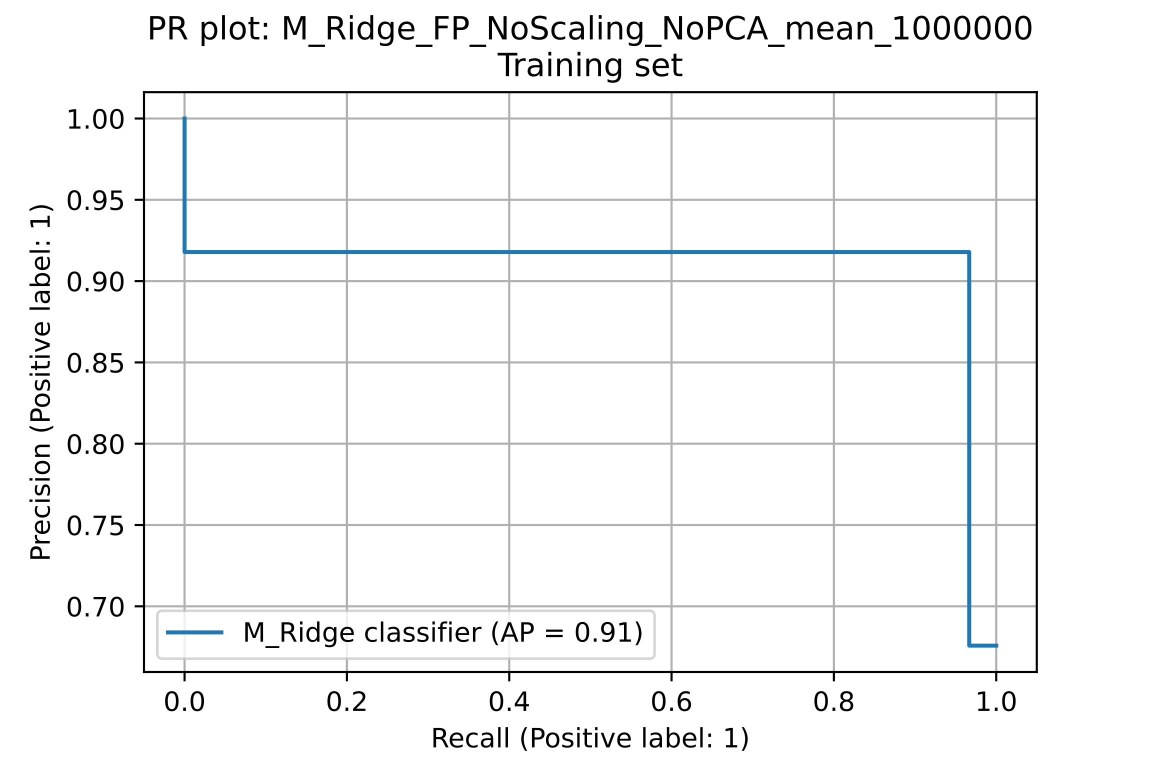

PR plot: M_Ridge_FP_NoScaling_NoPCA_mean_1000000
Training set
Precision (Positive label: 1)
Recall (Positive label: 1)
1.00
0.95
0.90
0.85
0.80
0.75
0.70
0.0
0.2
0.4
0.6
0.8
1.0
M_Ridge classifier (AP = 0.91)

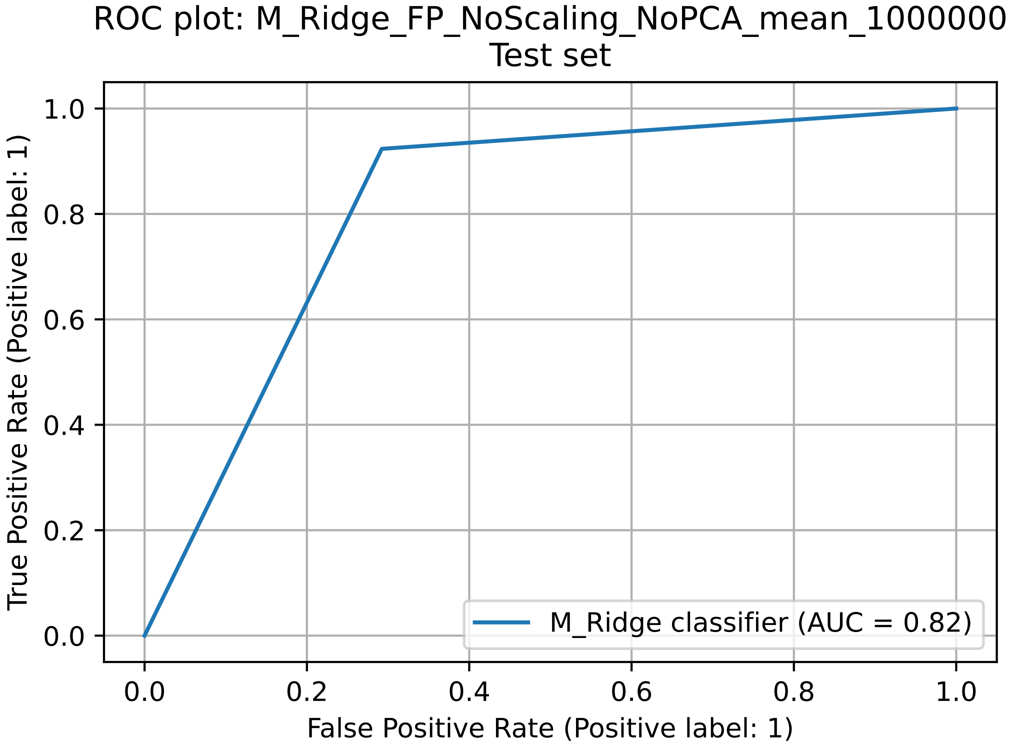
ROC plot: M_Ridge_FP_NoScaling_NoPCA_mean_1000000
Test set
True Positive Rate (Positive label: 1)
False Positive Rate (Positive label: 1)
0.0
0.2
0.4
0.6
0.8
1.0
M_Ridge classifier (AUC = 0.82)

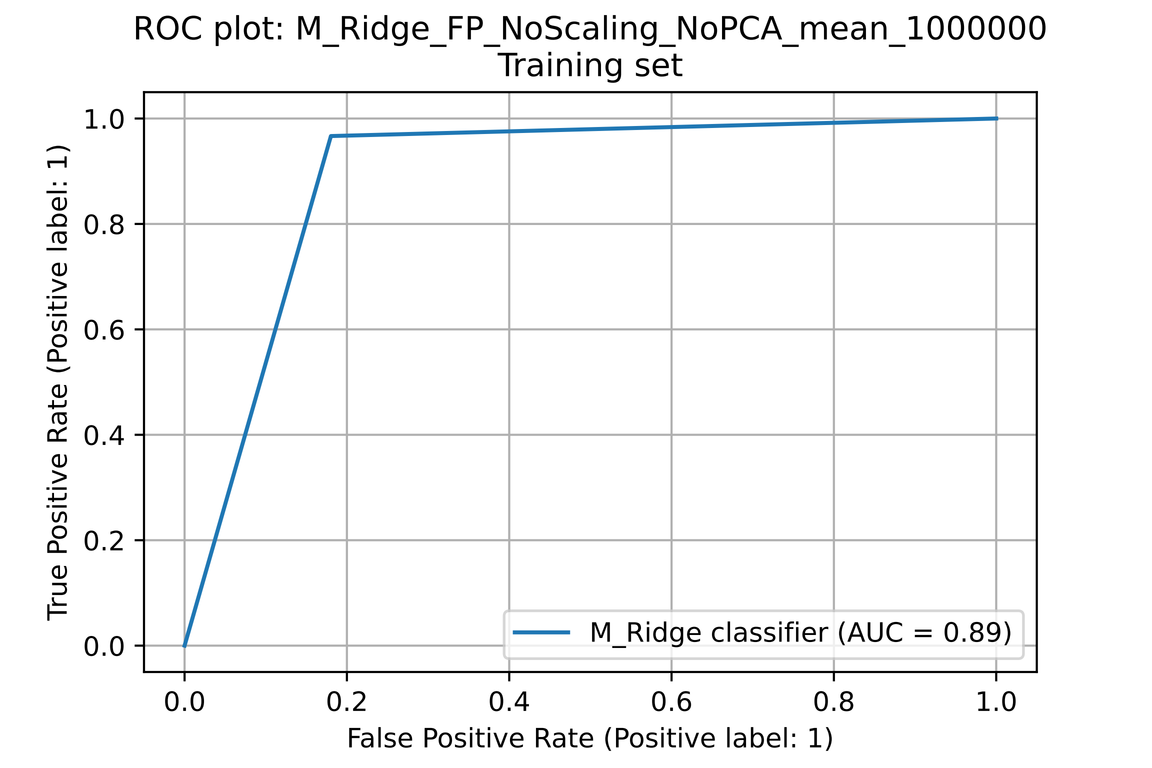
ROC plot: M_Ridge_FP_NoScaling_NoPCA_mean_1000000
Training set
True Positive Rate (Positive label: 1)
False Positive Rate (Positive label: 1)
0.0
0.2
0.4
0.6
0.8
1.0
M_Ridge classifier (AUC = 0.89)

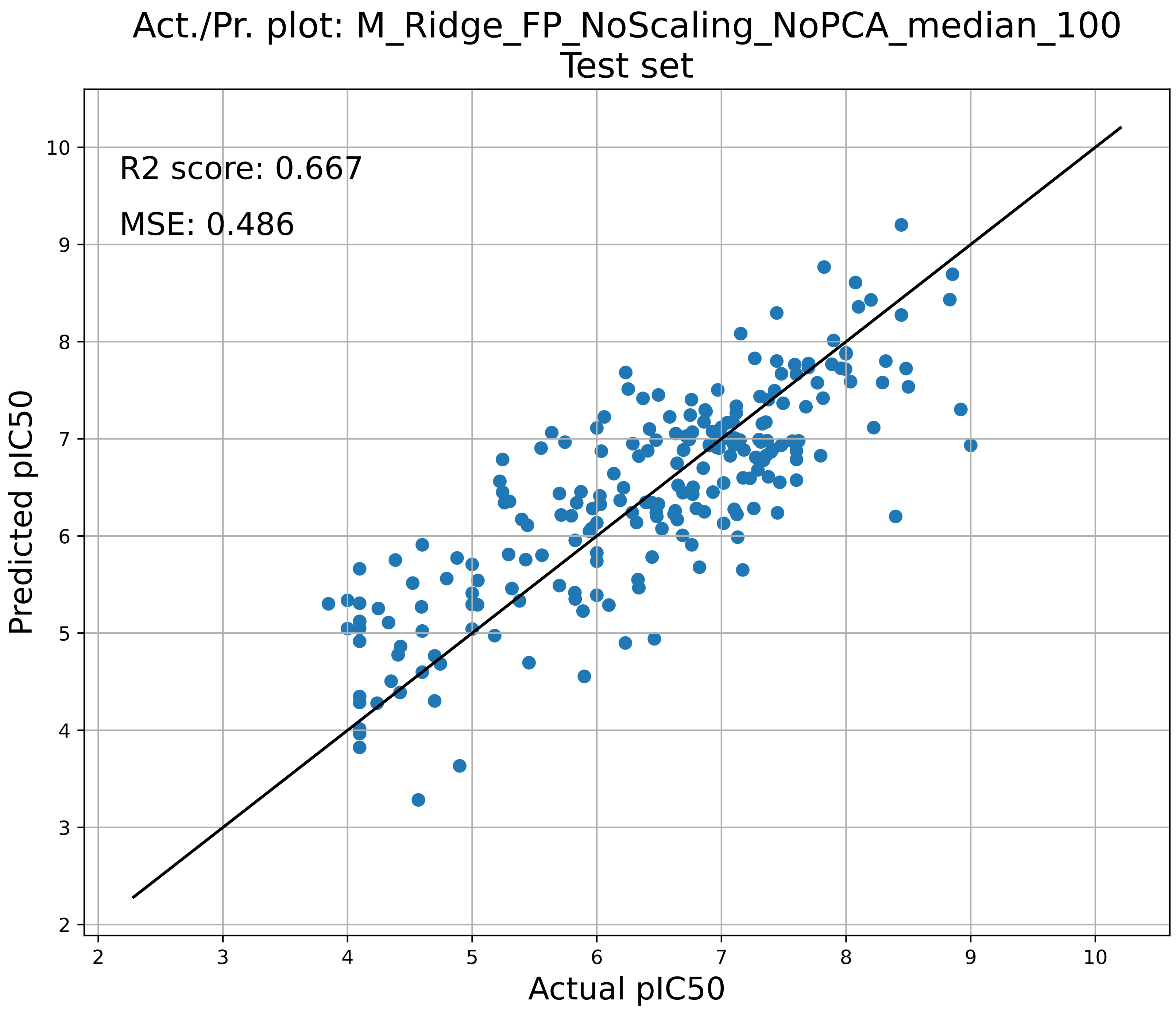
Act./Pr. plot: M_Ridge_FP_NoScaling_NoPCA_median_100
Test set
R2 score: 0.667
MSE: 0.486
Predicted pIC50
Actual pIC50
2
3
4
5
6
7
8
9
10

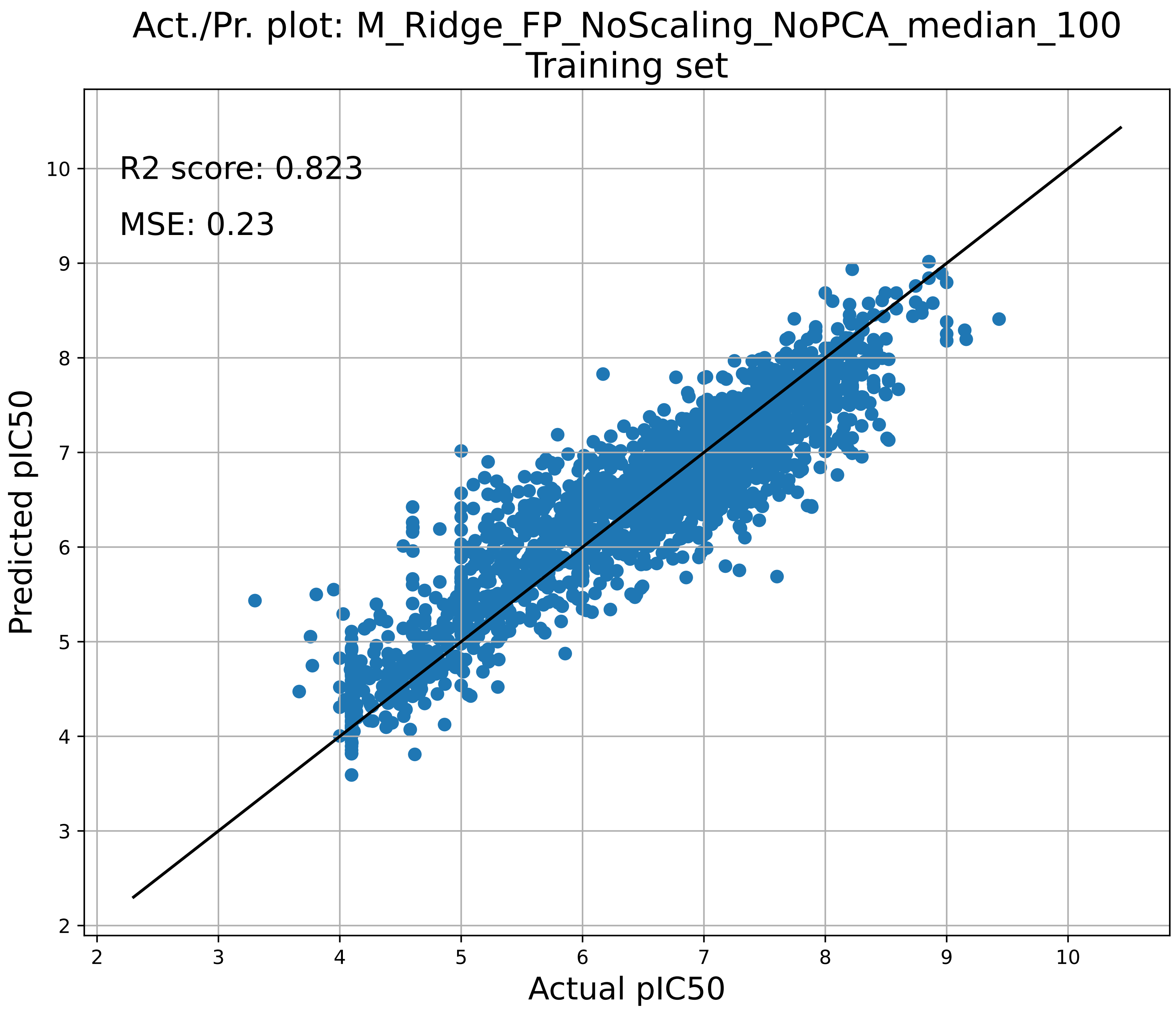

Act./Pr. plot: M_Ridge_FP_NoScaling_NoPCA_median_100
Training set
R2 score: 0.823
MSE: 0.23
Predicted pIC50
Actual pIC50
2
3
4
5
6
7
8
9
10

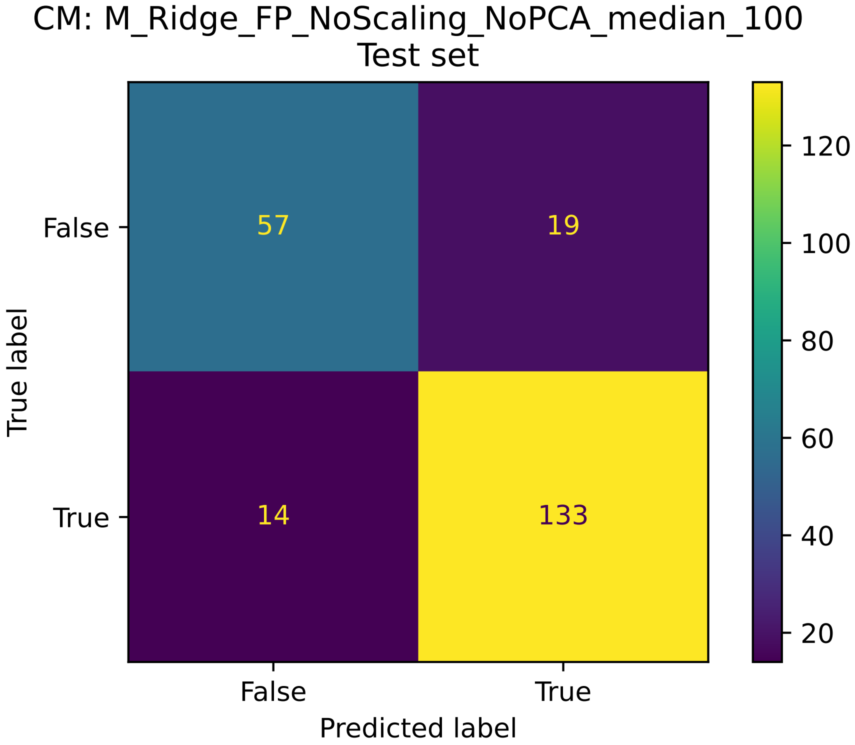

CM: M_Ridge_FP_NoScaling_NoPCA_median_100
Test set
True label
False
True
57
19
14
133
Predicted label
False
True
120
100
80
60
40
20

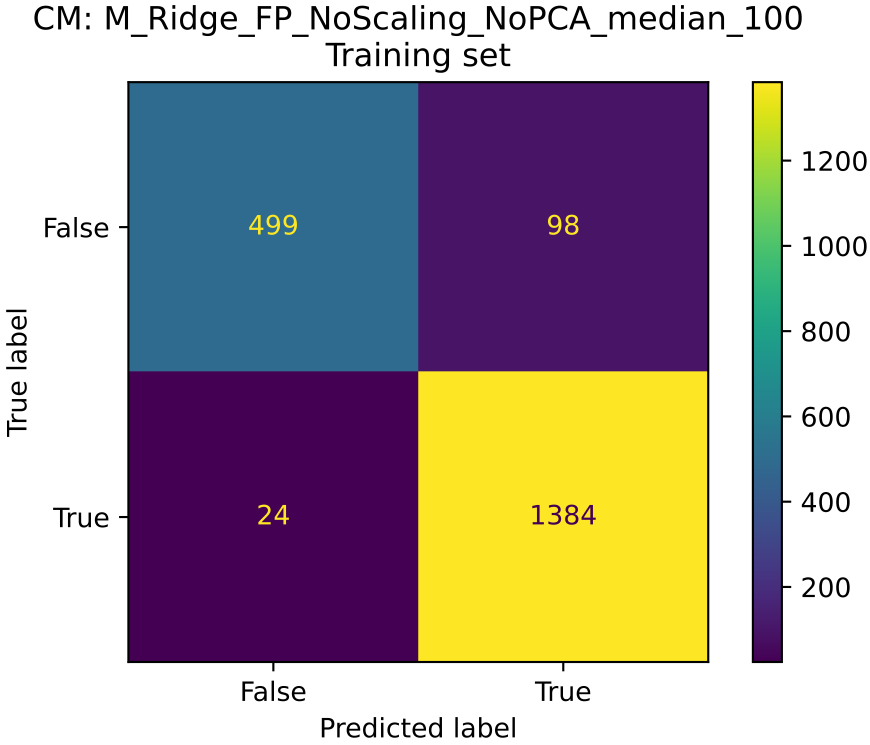
CM: M_Ridge_FP_NoScaling_NoPCA_median_100
Training set
True label
False
True
499
98
24
1384
Predicted label
False
True
1200
1000
800
600
400
200

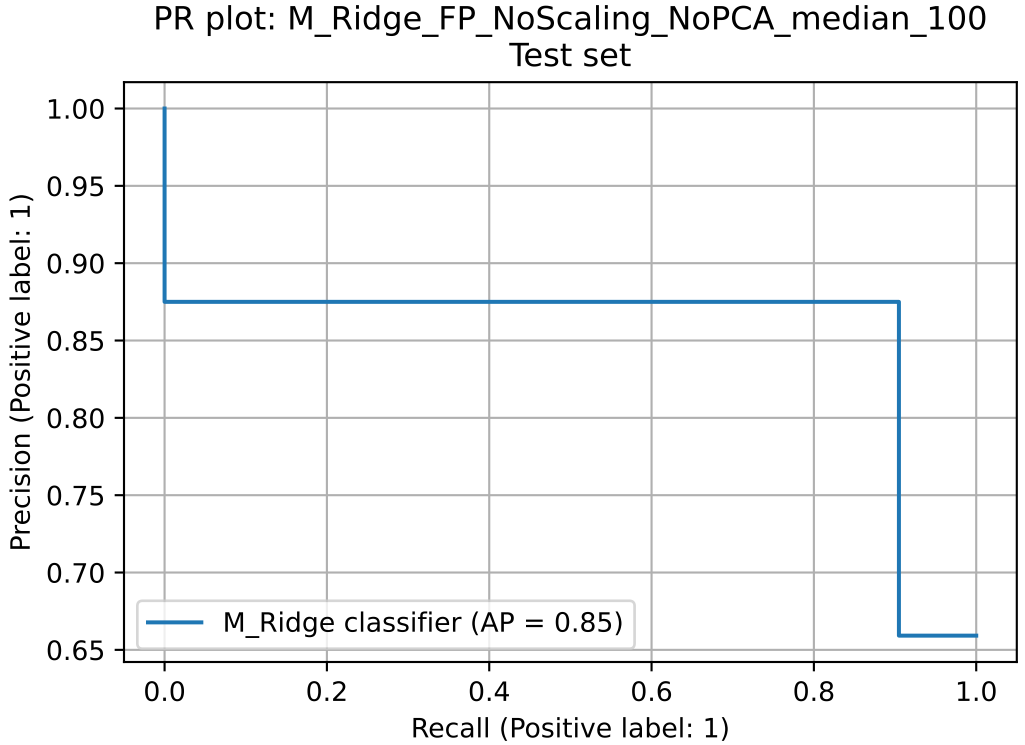
PR plot: M_Ridge_FP_NoScaling_NoPCA_median_100
Test set
Precision (Positive label: 1)
1.00
0.95
0.90
0.85
0.80
0.75
0.70
0.65
0.0
0.2
0.4
0.6
0.8
1.0
Recall (Positive label: 1)
M_Ridge classifier (AP = 0.85)

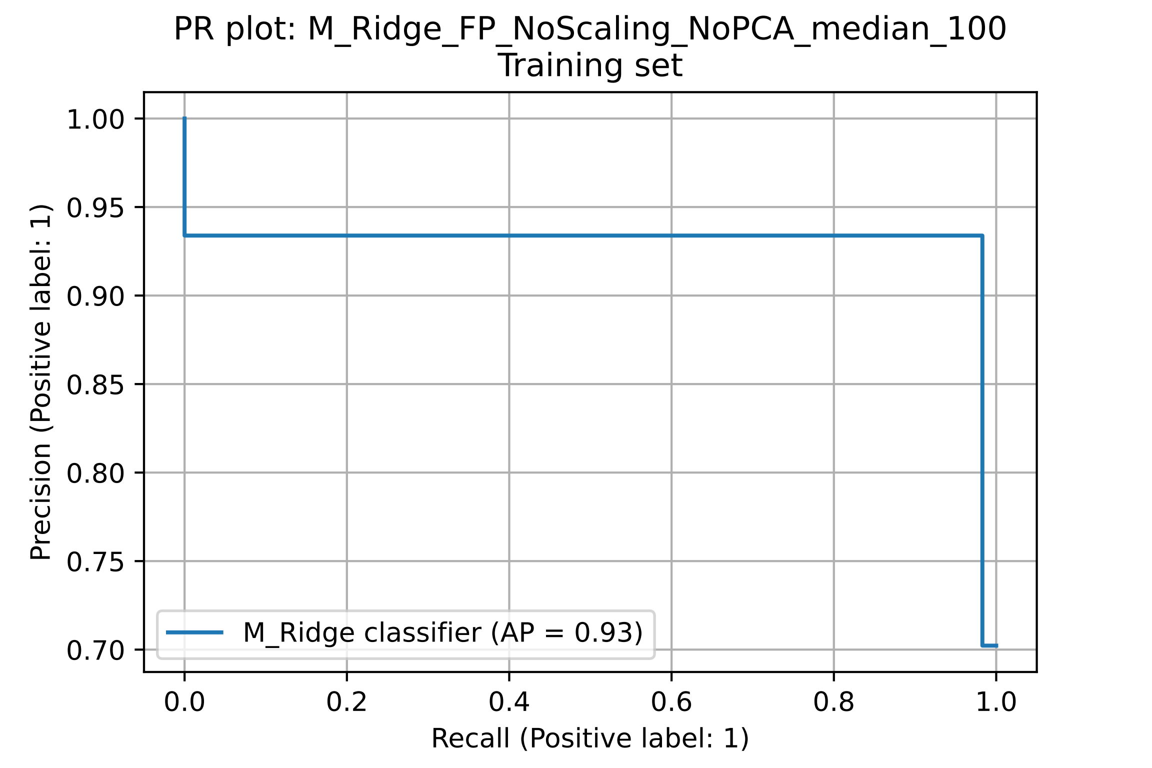

PR plot: M_Ridge_FP_NoScaling_NoPCA_median_100
Training set
Precision (Positive label: 1)
Recall (Positive label: 1)
1.00
0.95
0.90
0.85
0.80
0.75
0.70
0.0
0.2
0.4
0.6
0.8
1.0
M_Ridge classifier (AP = 0.93)

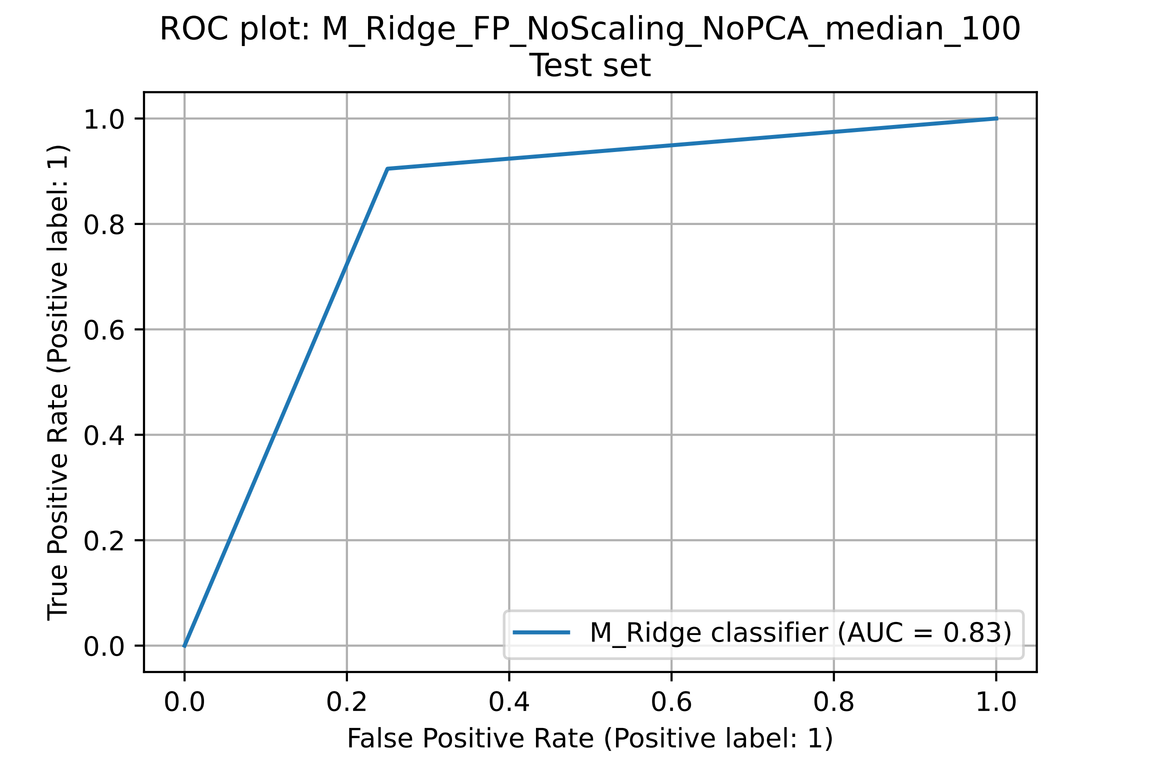
ROC plot: M_Ridge_FP_NoScaling_NoPCA_median_100
Test set
True Positive Rate (Positive label: 1)
False Positive Rate (Positive label: 1)
1.0
0.8
0.6
0.4
0.2
0.0
0.0
0.2
0.4
0.6
0.8
1.0
M_Ridge classifier (AUC = 0.83)

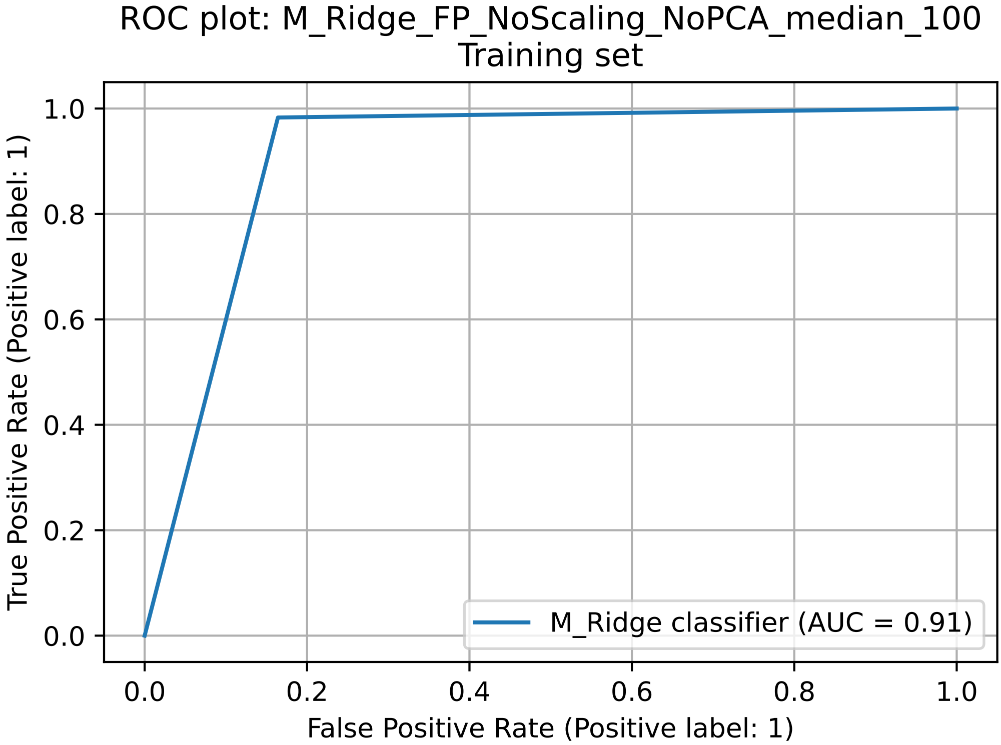
ROC plot: M_Ridge_FP_NoScaling_NoPCA_median_100
Training set
True Positive Rate (Positive label: 1)
False Positive Rate (Positive label: 1)
0.0
0.2
0.4
0.6
0.8
1.0
M_Ridge classifier (AUC = 0.91)

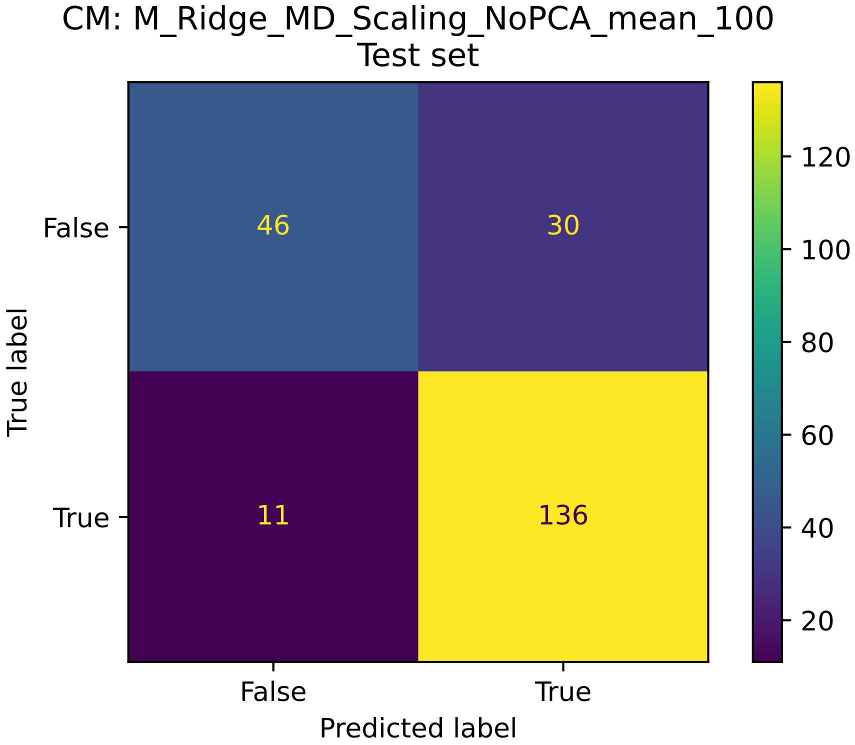
CM: M_Ridge_MD_Scaling_NoPCA_mean_100
Test set
46
30
11
136
False
True
True label
False
True
Predicted label
120
100
80
60
40
20

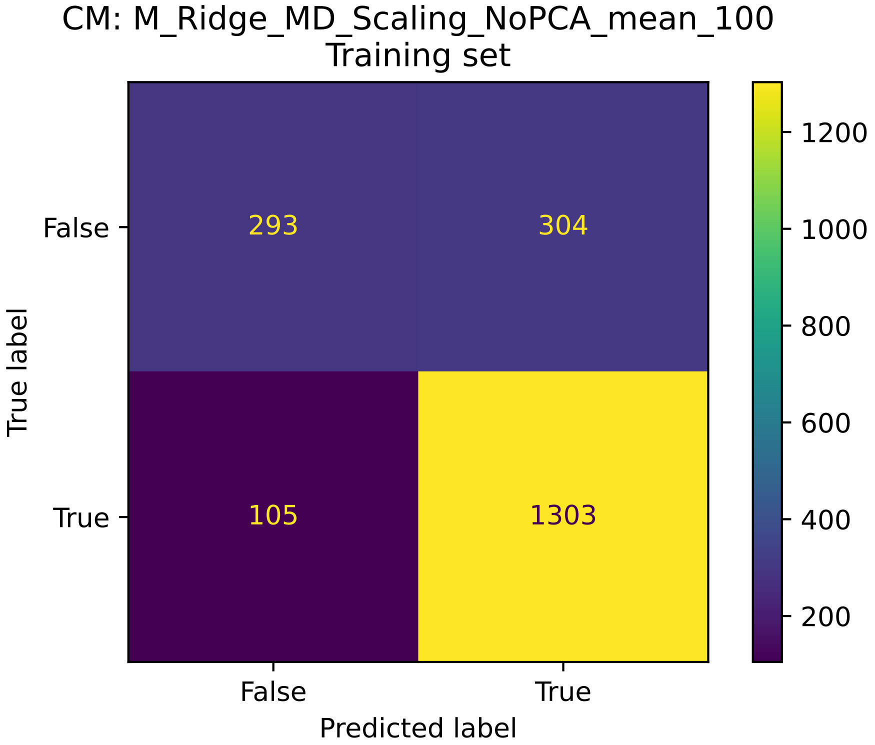

CM: M_Ridge_MD_Scaling_NoPCA_mean_100
Training set
True label
False
True
293
304
105
1303
Predicted label
False
True
1200
1000
800
600
400
200

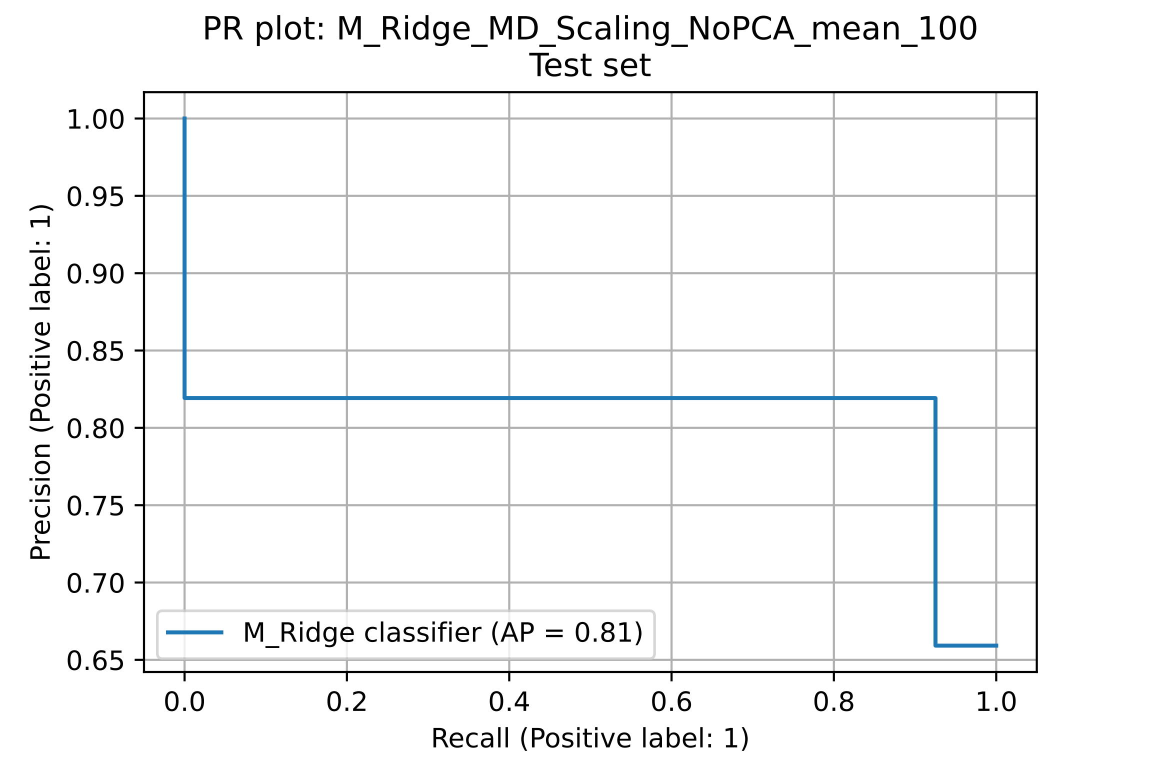
PR plot: M_Ridge_MD_Scaling_NoPCA_mean_100
Test set
Precision (Positive label: 1)
Recall (Positive label: 1)
M_Ridge classifier (AP = 0.81)
1.00
0.95
0.90
0.85
0.80
0.75
0.70
0.65
0.0
0.2
0.4
0.6
0.8
1.0

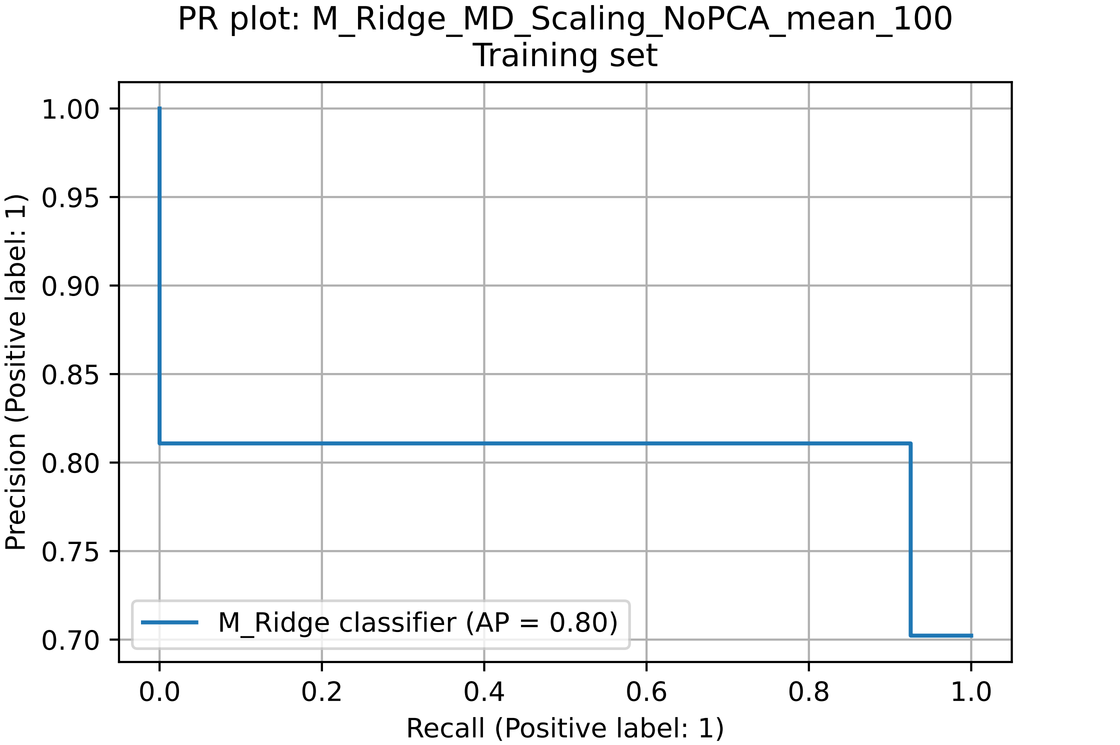

PR plot: M_Ridge_MD_Scaling_NoPCA_mean_100
Training set
Precision (Positive label: 1)
1.00
0.95
0.90
0.85
0.80
0.75
0.70
0.0
0.2
0.4
0.6
0.8
1.0
Recall (Positive label: 1)
M_Ridge classifier (AP = 0.80)

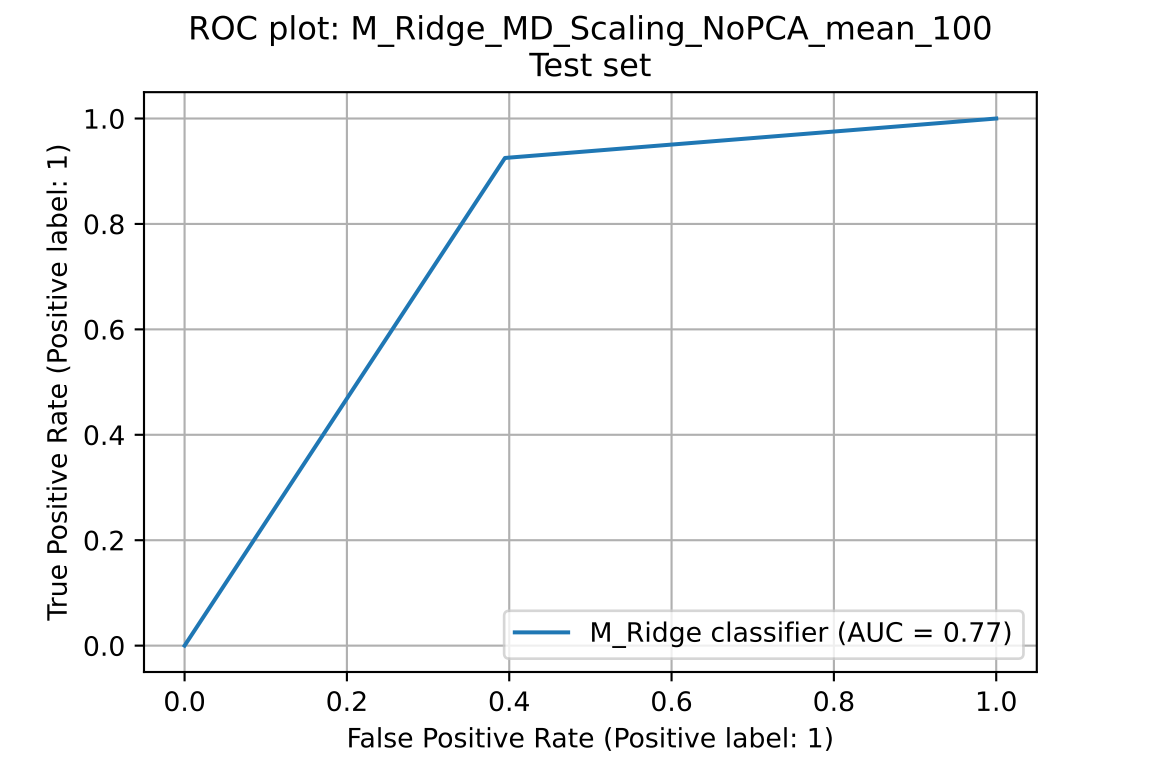

ROC plot: M_Ridge_MD_Scaling_NoPCA_mean_100
Test set
True Positive Rate (Positive label: 1)
False Positive Rate (Positive label: 1)
0.0
0.2
0.4
0.6
0.8
1.0
M_Ridge classifier (AUC = 0.77)

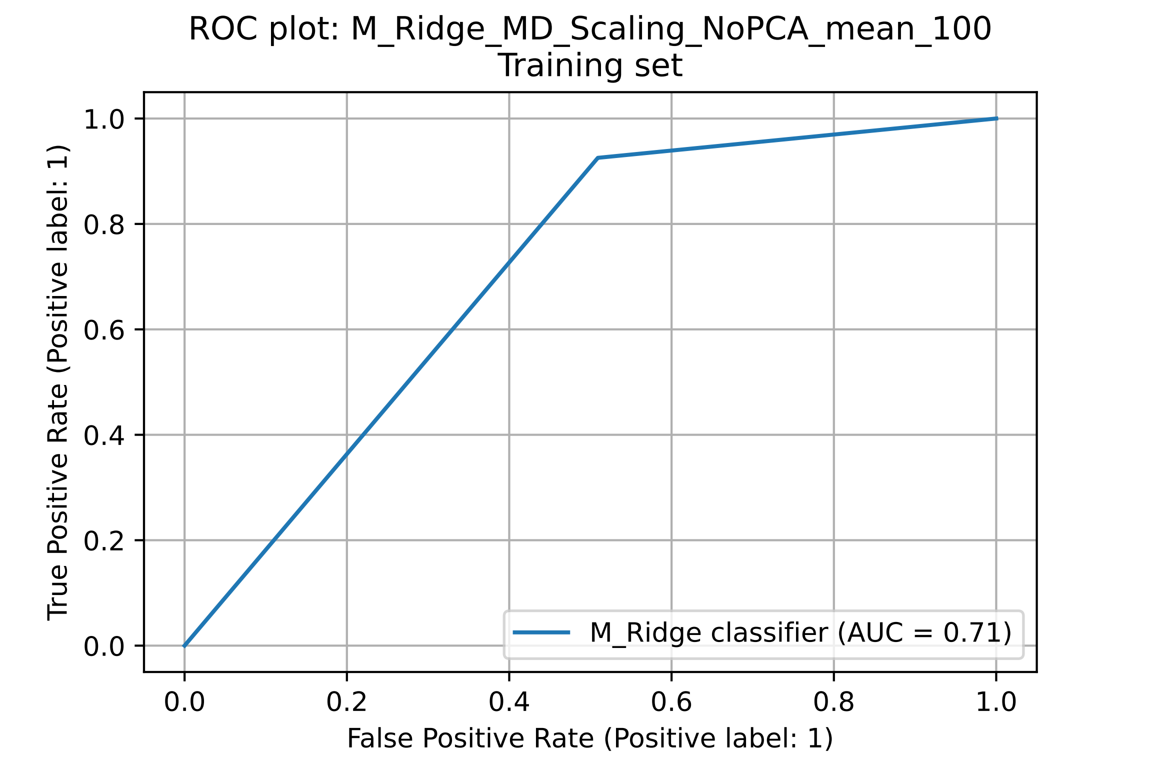

ROC plot: M_Ridge_MD_Scaling_NoPCA_mean_100
Training set
True Positive Rate (Positive label: 1)
False Positive Rate (Positive label: 1)
1.0
0.8
0.6
0.4
0.2
0.0
0.0
0.2
0.4
0.6
0.8
1.0
M_Ridge classifier (AUC = 0.71)

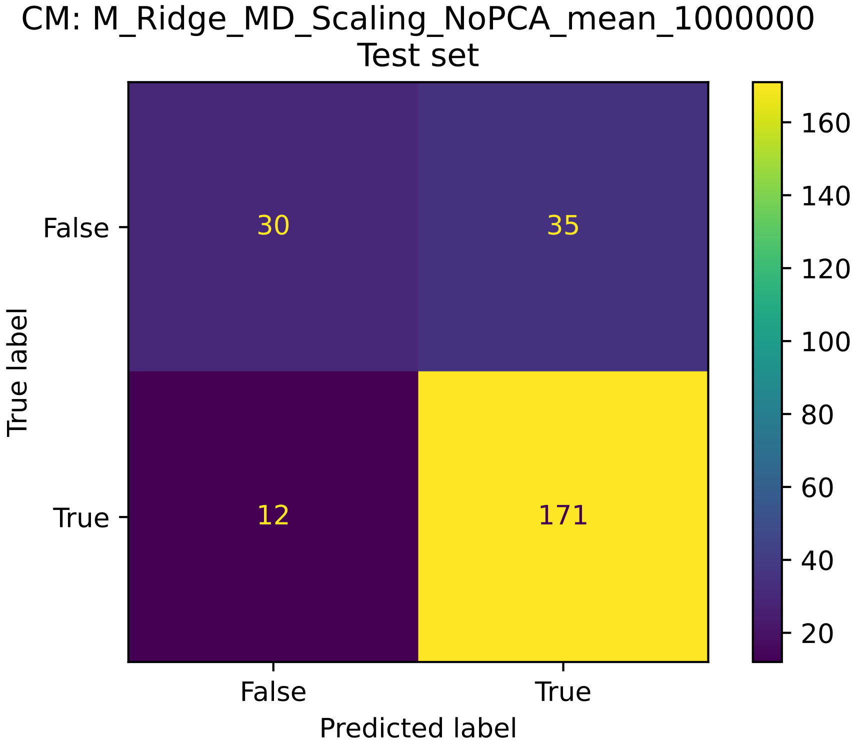
CM: M_Ridge_MD_Scaling_NoPCA_mean_1000000
Test set
True label
False
True
30
35
12
171
False
True
Predicted label
160
140
120
100
80
60
40
20

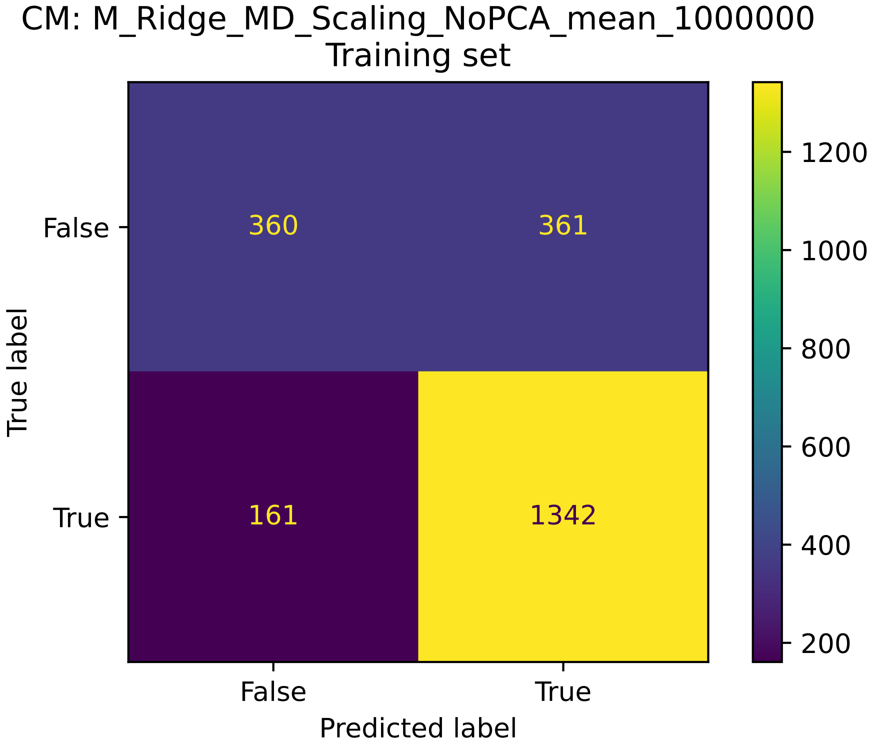
CM: M_Ridge_MD_Scaling_NoPCA_mean_1000000
Training set
True label
False
True
360
361
161
1342
False
True
Predicted label
1200
1000
800
600
400
200

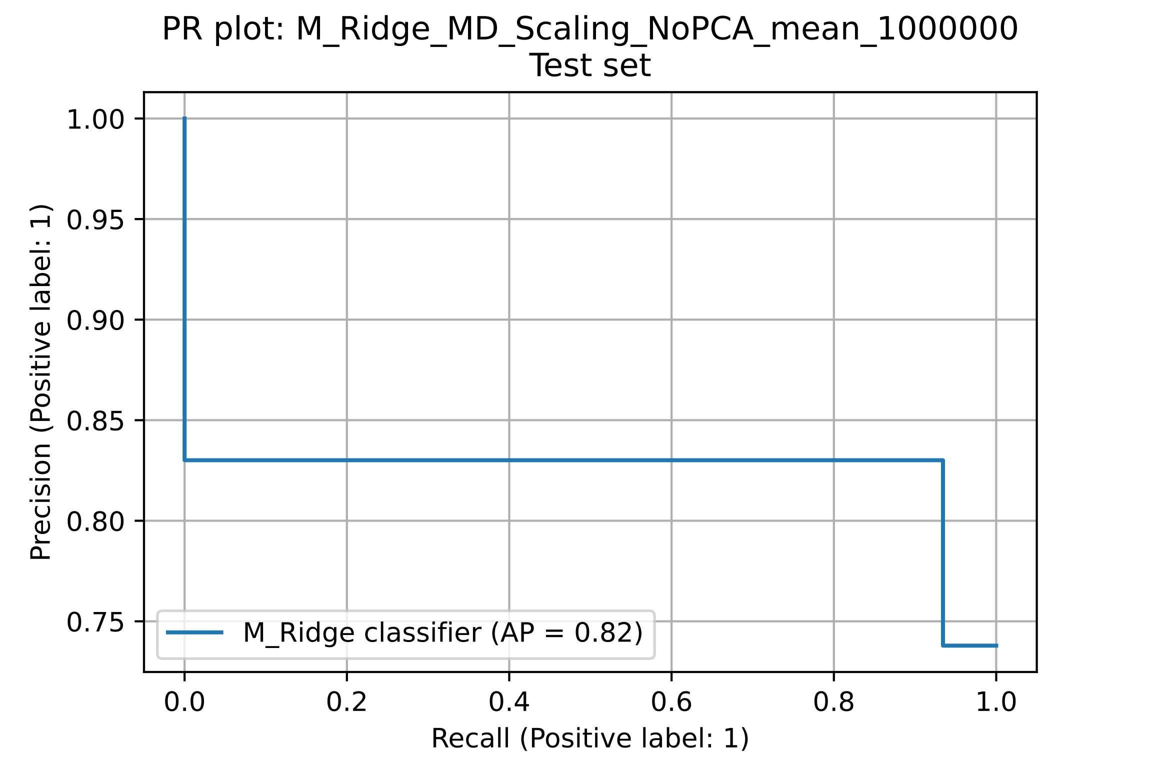
PR plot: M_Ridge_MD_Scaling_NoPCA_mean_1000000
Test set
1.00
0.95
0.90
0.85
0.80
0.75
Precision (Positive label: 1)
0.0
0.2
0.4
0.6
0.8
1.0
Recall (Positive label: 1)
M_Ridge classifier (AP = 0.82)

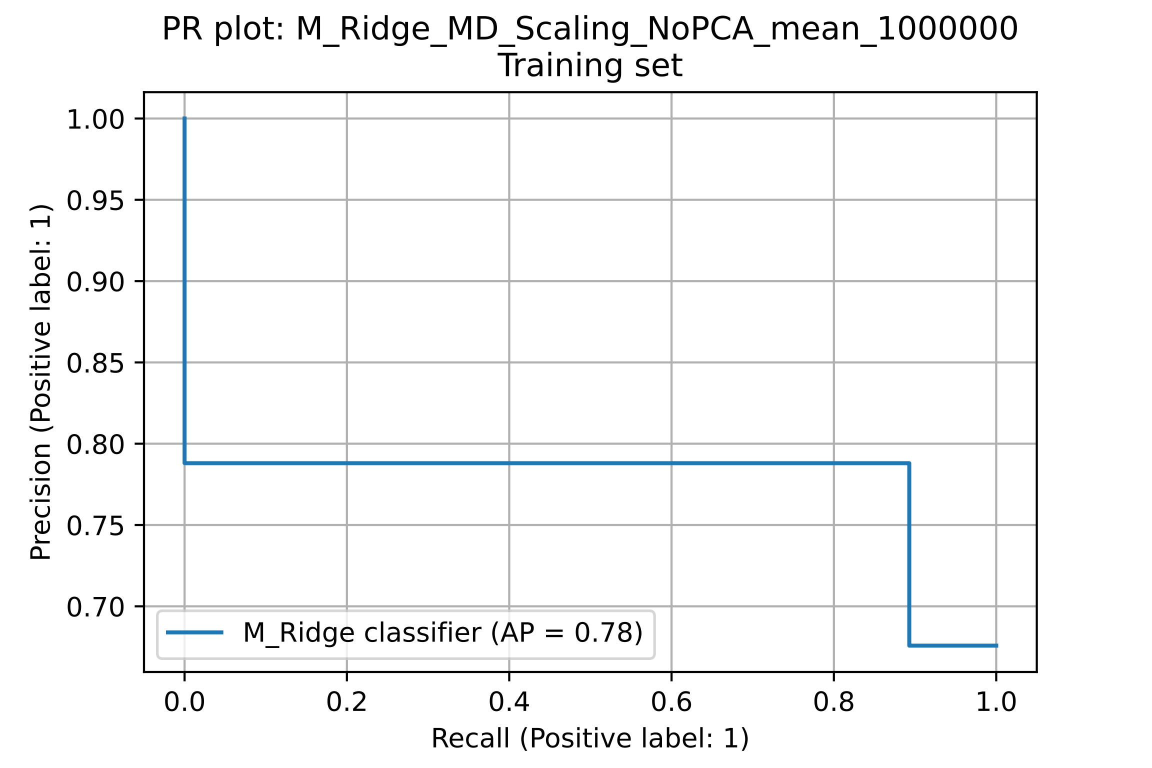
PR plot: M_Ridge_MD_Scaling_NoPCA_mean_1000000
Training set
Precision (Positive label: 1)
1.00
0.95
0.90
0.85
0.80
0.75
0.70
Recall (Positive label: 1)
0.0
0.2
0.4
0.6
0.8
1.0
M_Ridge classifier (AP = 0.78)

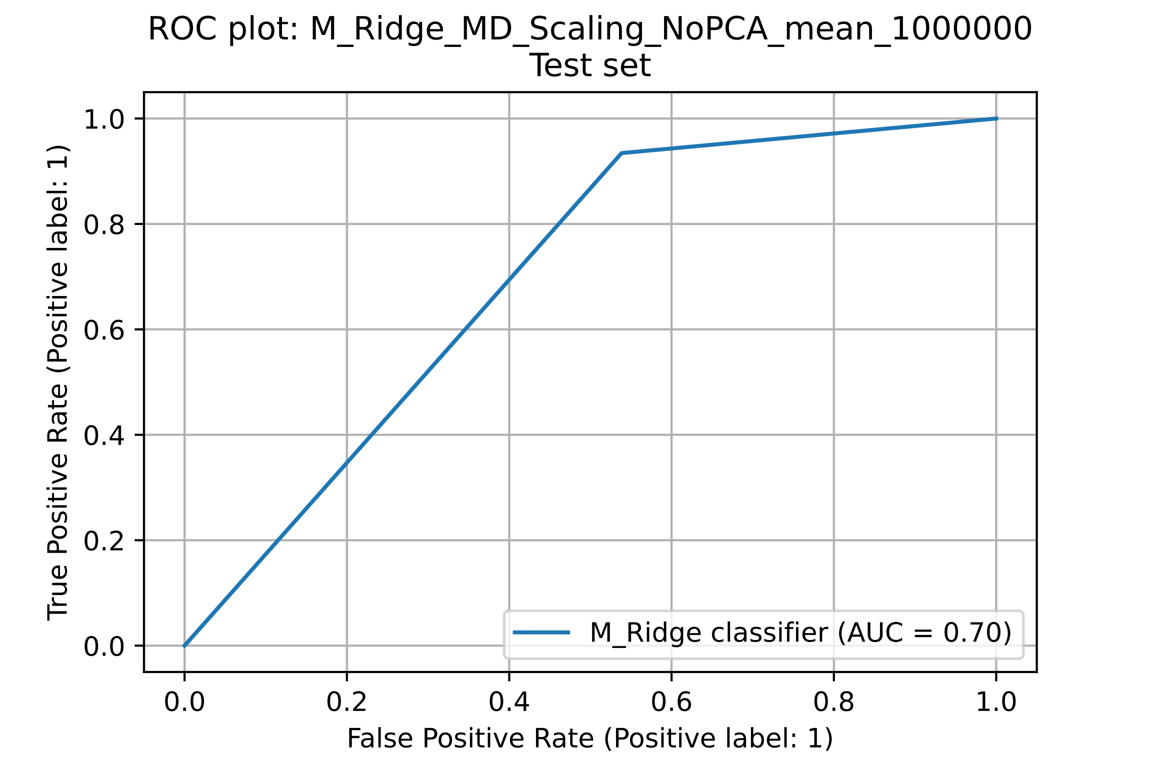

ROC plot: M_Ridge_MD_Scaling_NoPCA_mean_1000000
Test set
True Positive Rate (Positive label: 1)
False Positive Rate (Positive label: 1)
0.0
0.2
0.4
0.6
0.8
1.0
M_Ridge classifier (AUC = 0.70)

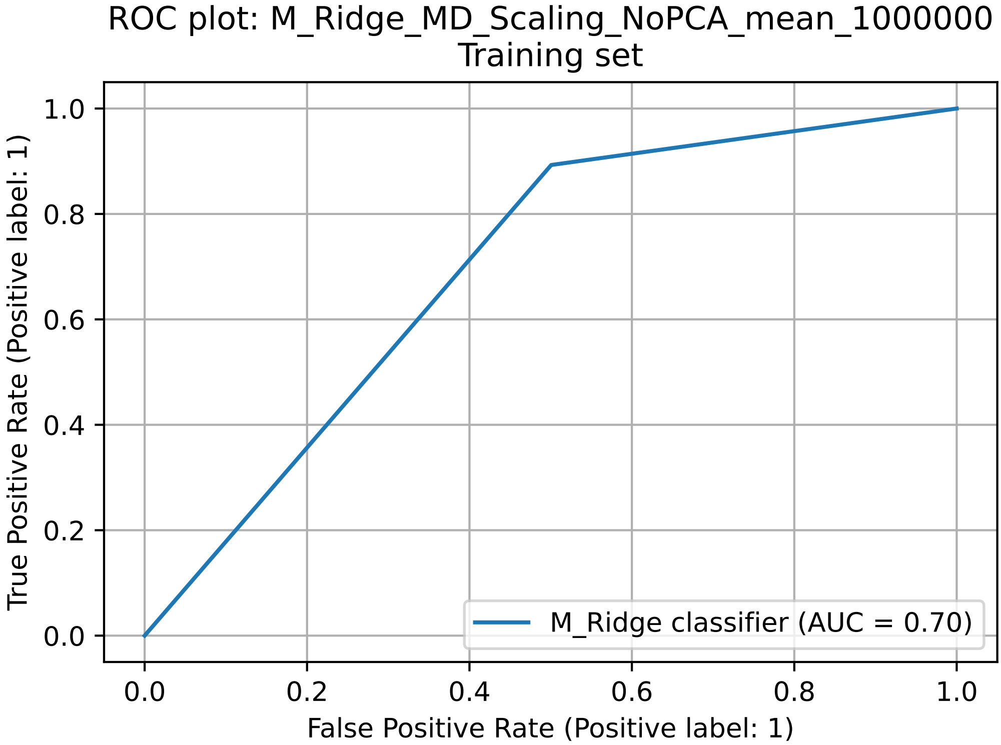
ROC plot: M_Ridge_MD_Scaling_NoPCA_mean_1000000
Training set
True Positive Rate (Positive label: 1)
False Positive Rate (Positive label: 1)
0.0
0.2
0.4
0.6
0.8
1.0
M_Ridge classifier (AUC = 0.70)

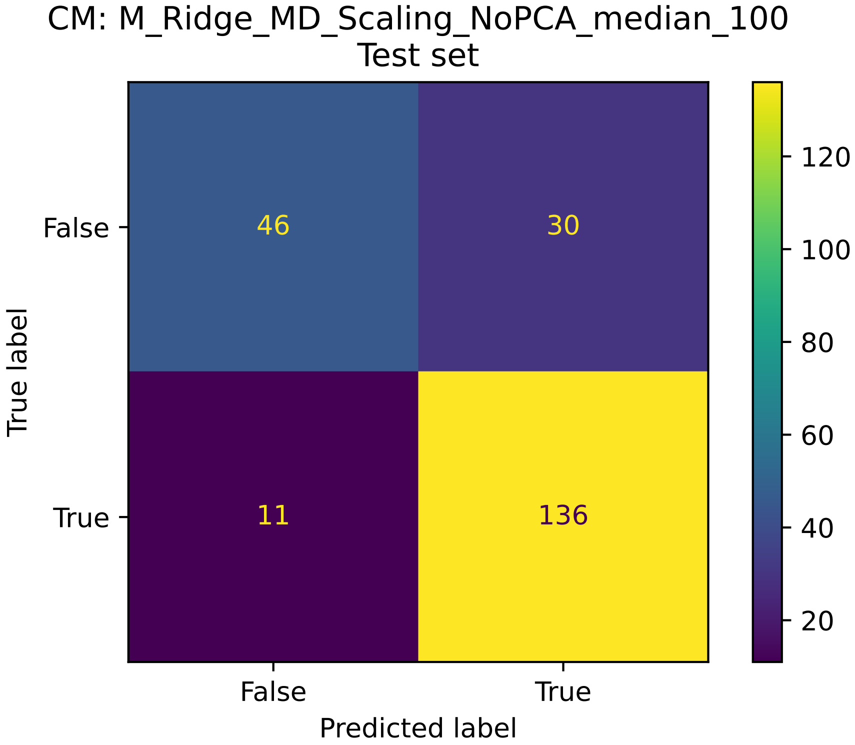

CM: M_Ridge_MD_Scaling_NoPCA_median_100
Test set
True label
Predicted label
False
True
False
True
46
30
11
136
20
40
60
80
100
120

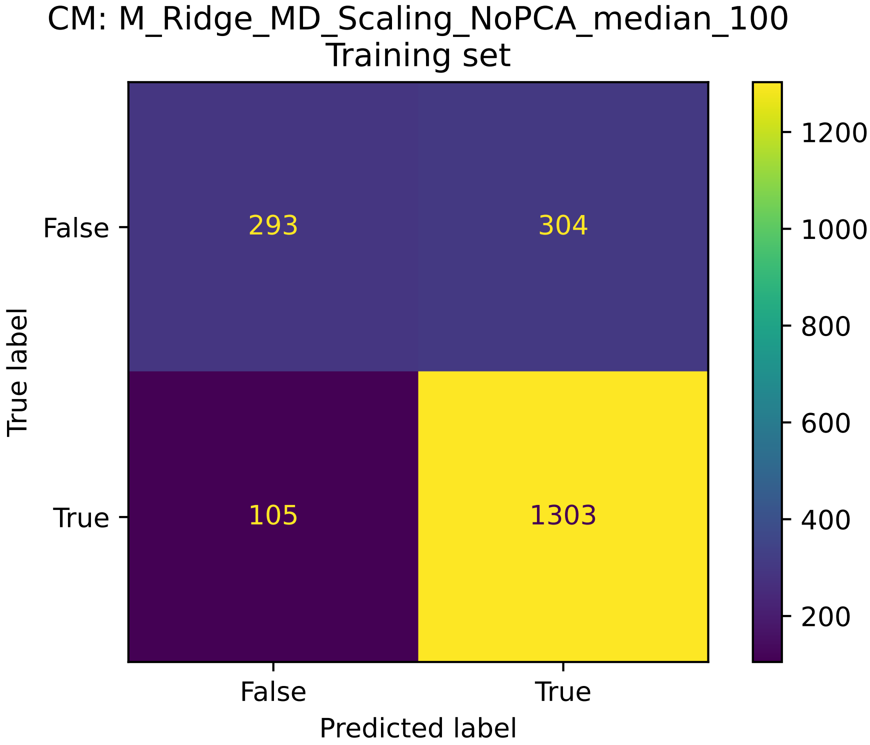
CM: M_Ridge_MD_Scaling_NoPCA_median_100
Training set
True label
Predicted label
False
True
False
True
293
304
105
1303
1200
1000
800
600
400
200

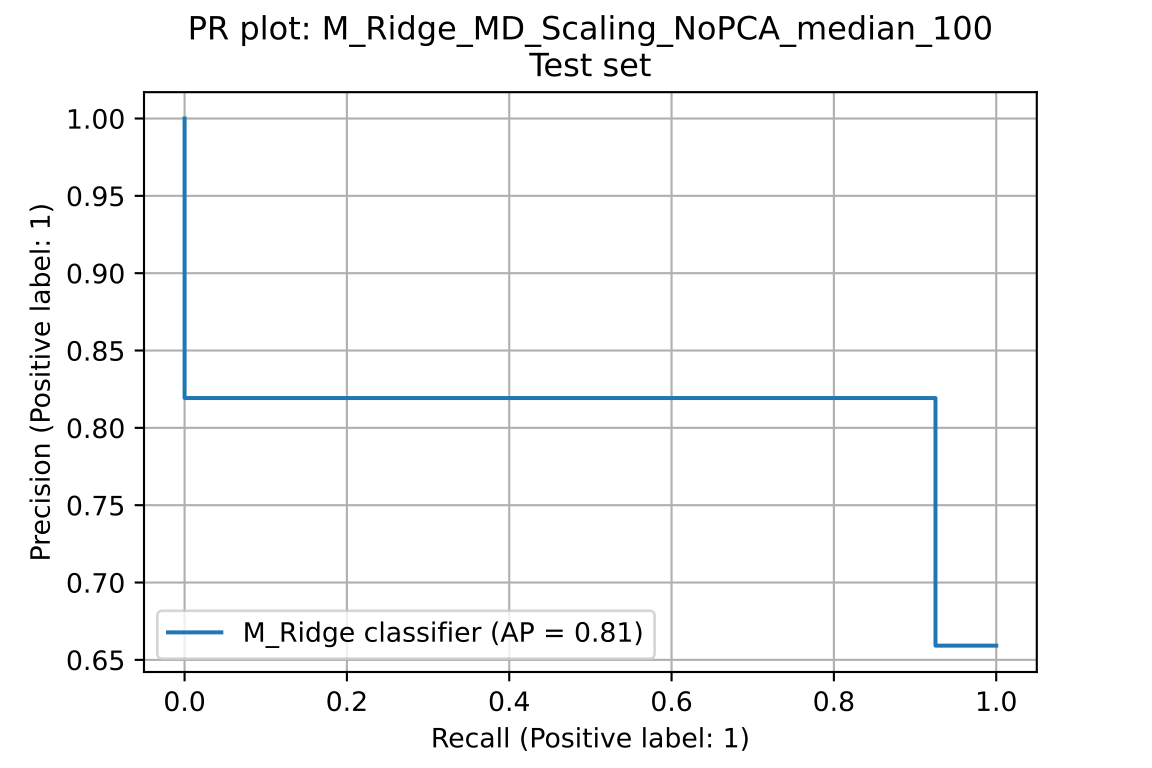
PR plot: M_Ridge_MD_Scaling_NoPCA_median_100
Test set
Precision (Positive label: 1)
Recall (Positive label: 1)
1.00
0.95
0.90
0.85
0.80
0.75
0.70
0.65
0.0
0.2
0.4
0.6
0.8
1.0
M_Ridge classifier (AP = 0.81)

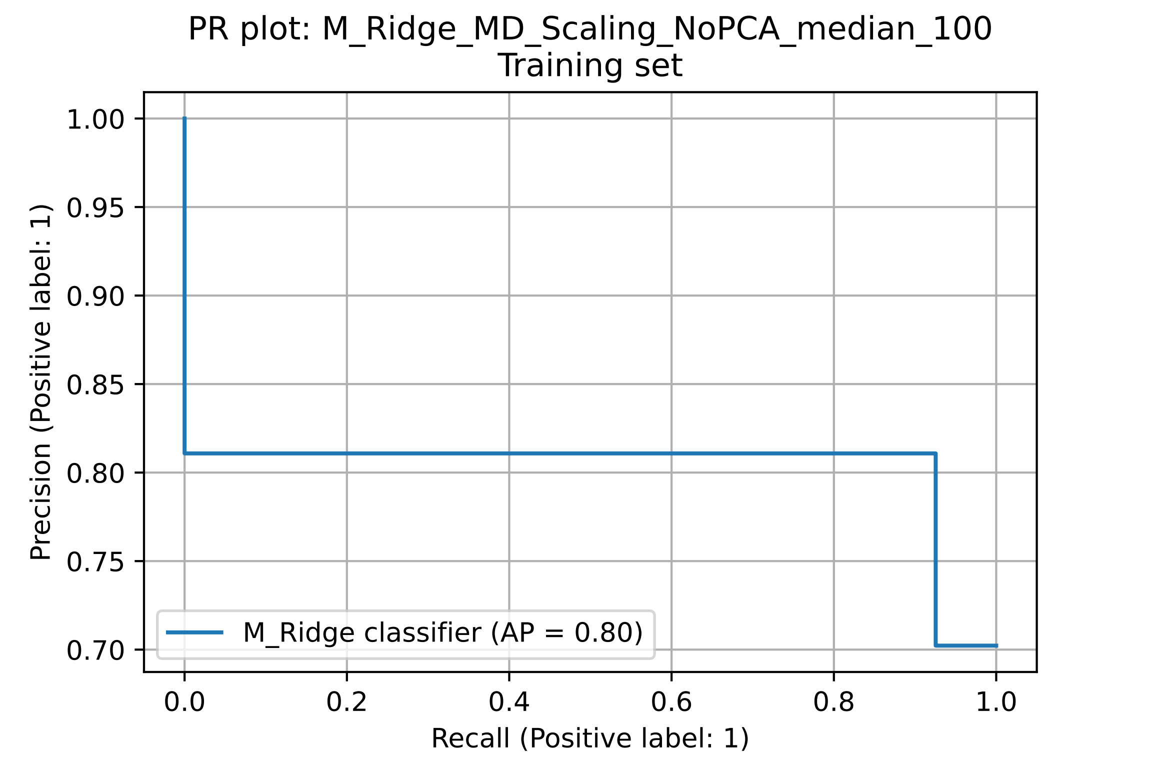
PR plot: M_Ridge_MD_Scaling_NoPCA_median_100
Training set
Precision (Positive label: 1)
Recall (Positive label: 1)
1.00
0.95
0.90
0.85
0.80
0.75
0.70
0.0
0.2
0.4
0.6
0.8
1.0
M_Ridge classifier (AP = 0.80)

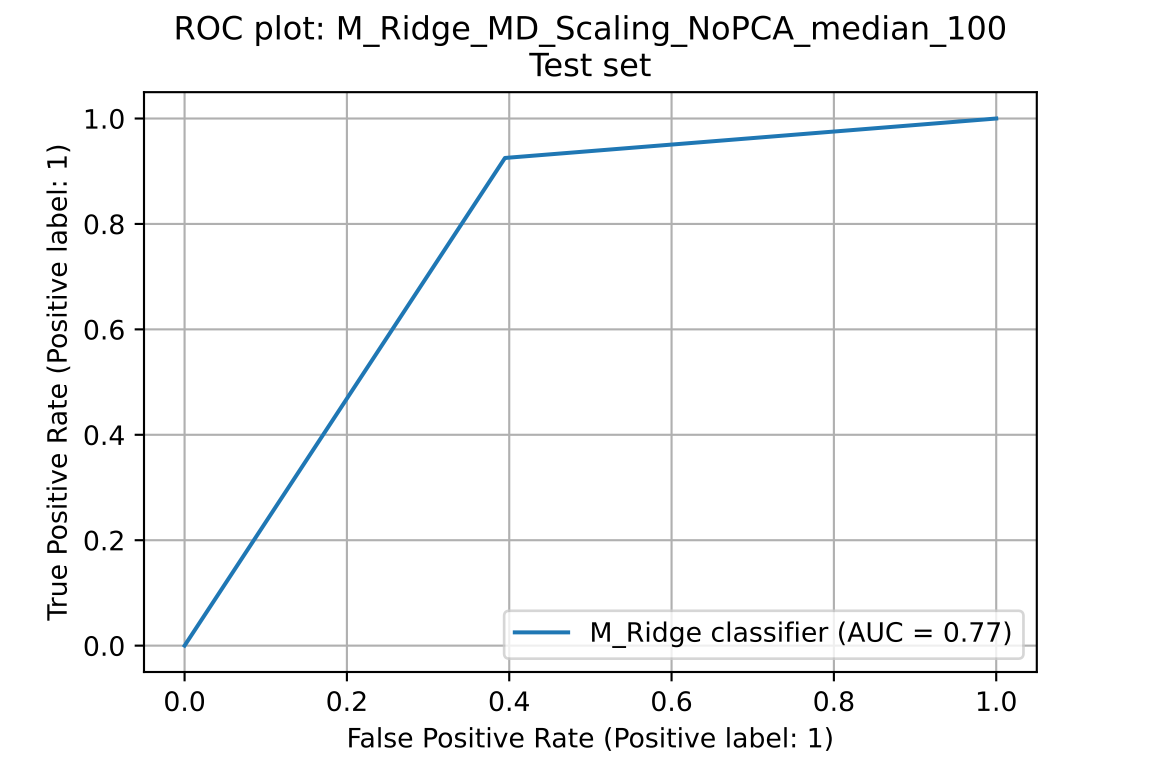
ROC plot: M_Ridge_MD_Scaling_NoPCA_median_100
Test set
True Positive Rate (Positive label: 1)
False Positive Rate (Positive label: 1)
0.0
0.2
0.4
0.6
0.8
1.0
M_Ridge classifier (AUC = 0.77)

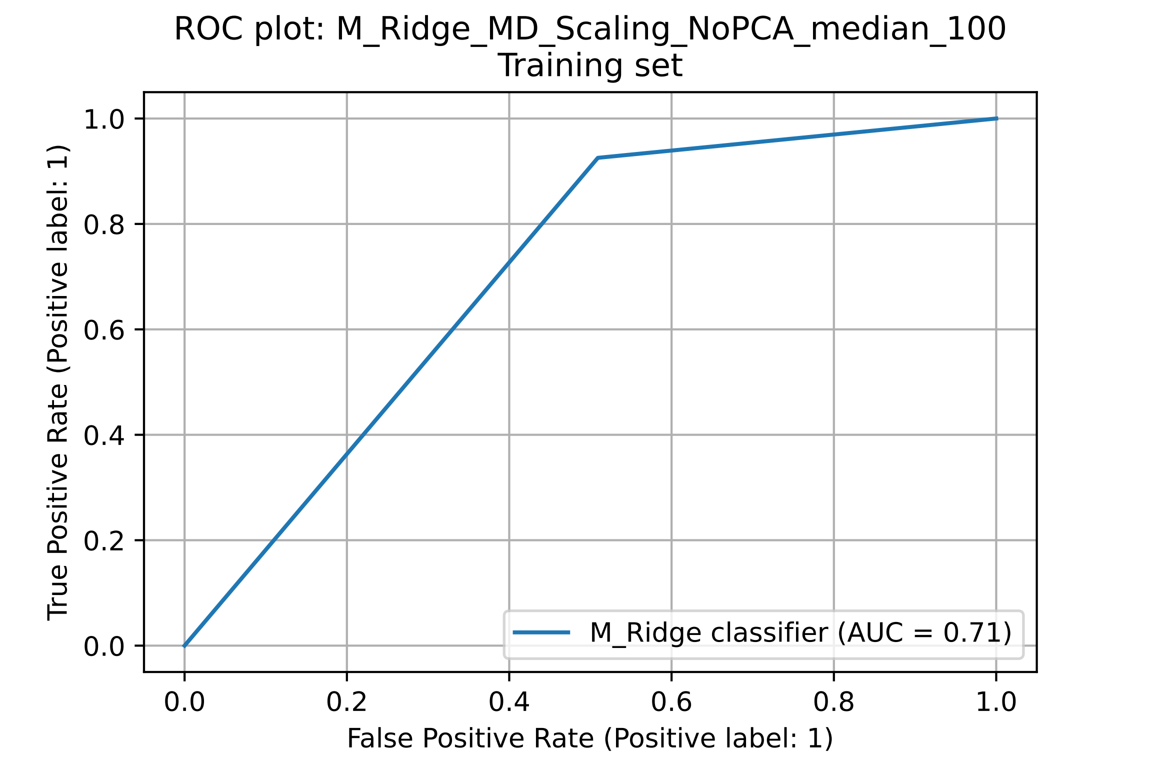

ROC plot: M_Ridge_MD_Scaling_NoPCA_median_100
Training set
1.0
0.8
0.6
0.4
0.2
0.0
True Positive Rate (Positive label: 1)
0.0
0.2
0.4
0.6
0.8
1.0
False Positive Rate (Positive label: 1)
M_Ridge classifier (AUC = 0.71)

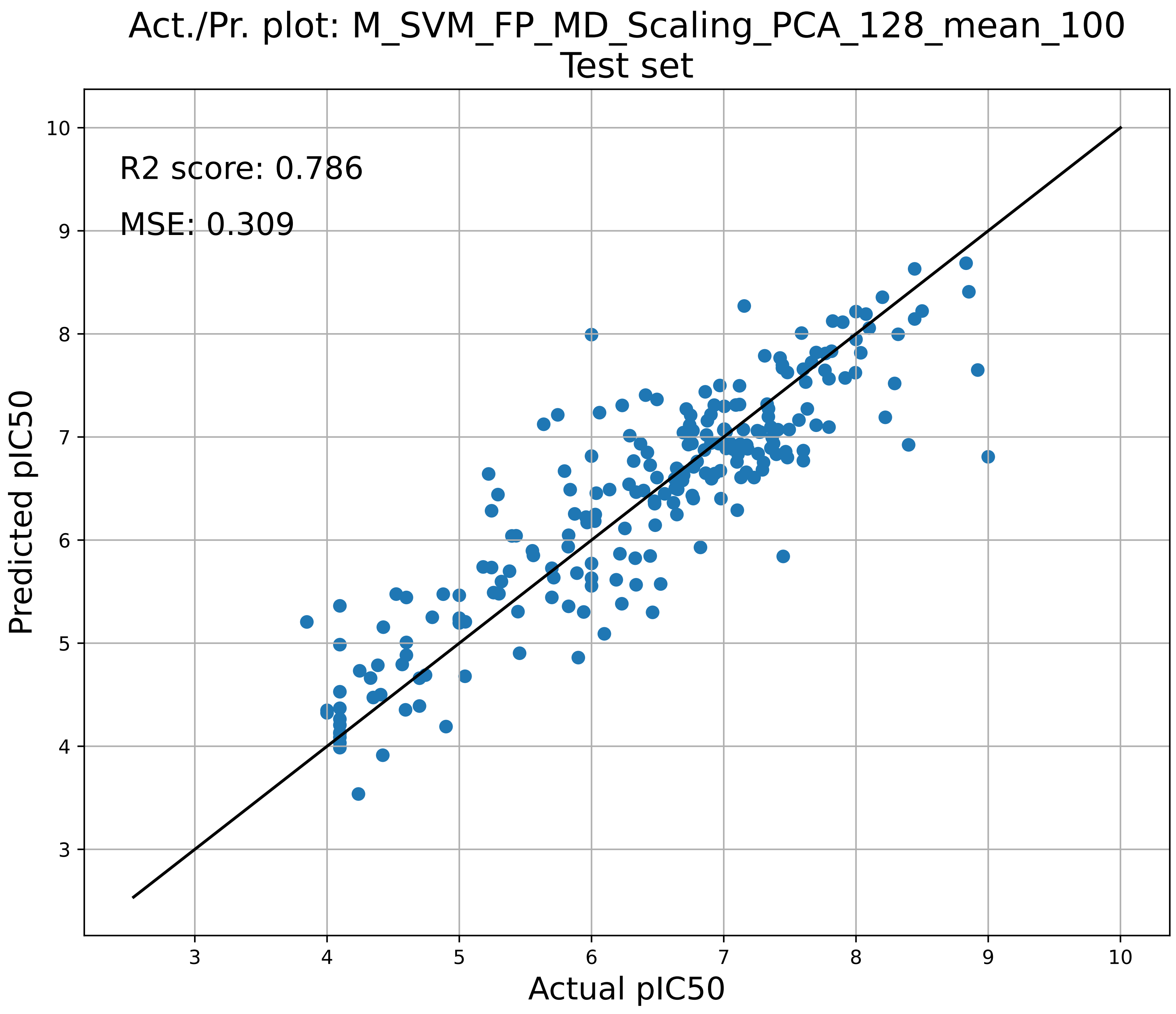

Act./Pr. plot: M_SVM_FP_MD_Scaling_PCA_128_mean_100
Test set
R2 score: 0.786
MSE: 0.309
Predicted pIC50
Actual pIC50
3
4
5
6
7
8
9
10

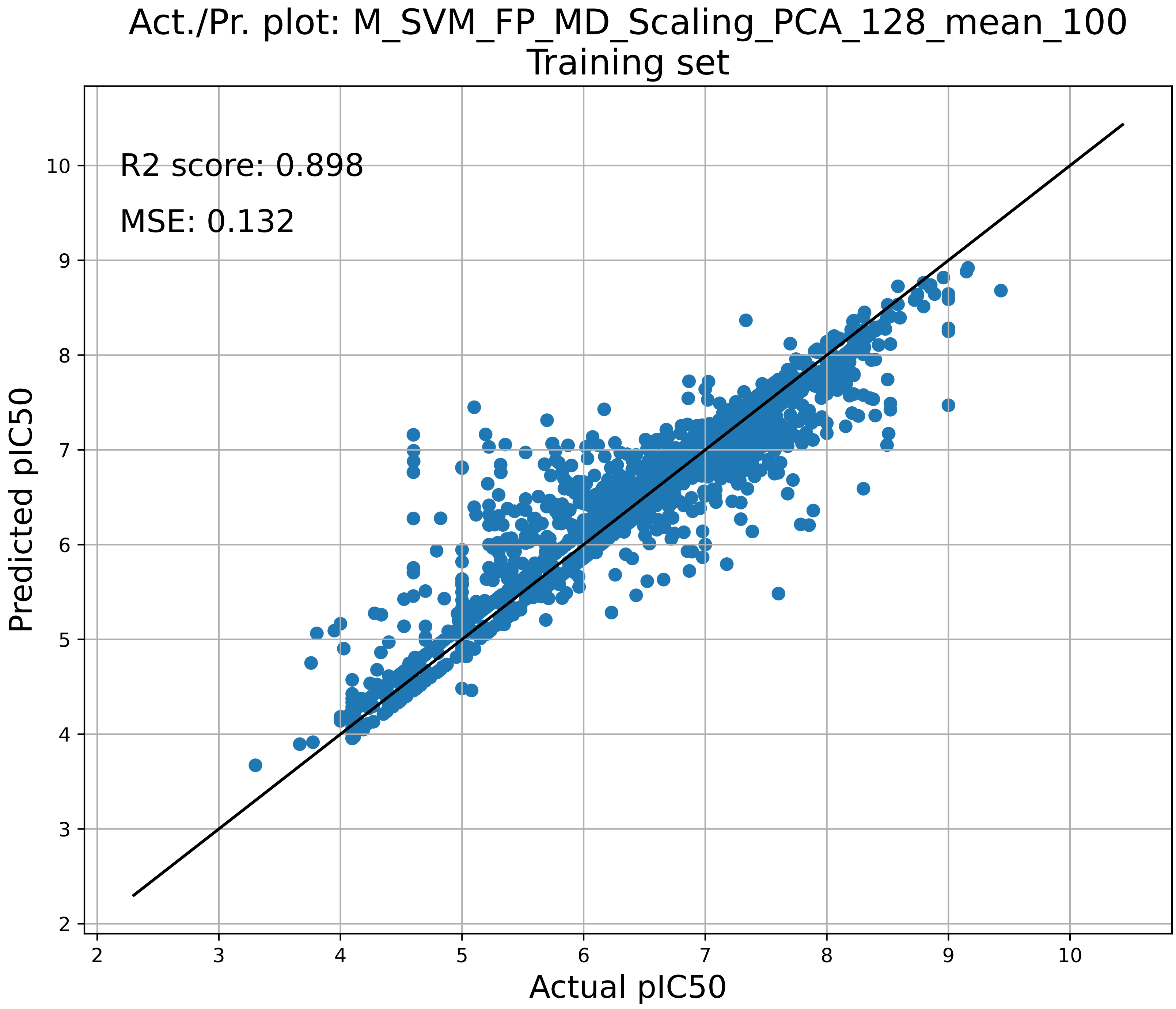
Act./Pr. plot: M_SVM_FP_MD_Scaling_PCA_128_mean_100
Training set
R2 score: 0.898
MSE: 0.132
Predicted pIC50
Actual pIC50
2
3
4
5
6
7
8
9
10

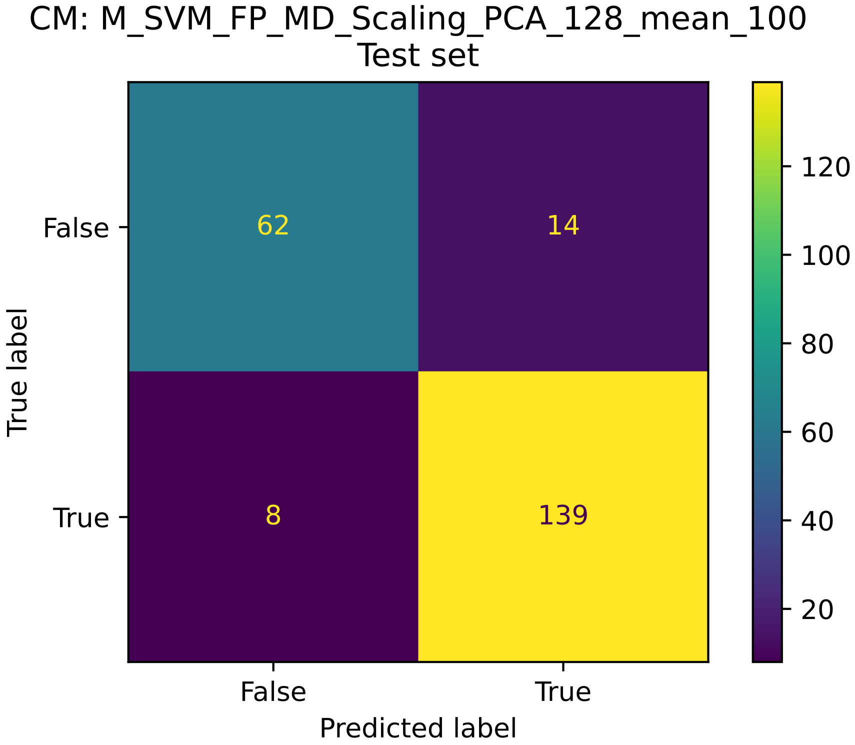
CM: M_SVM_FP_MD_Scaling_PCA_128_mean_100
Test set
True label
False
True
62
14
8
139
Predicted label
False
True
20
40
60
80
100
120

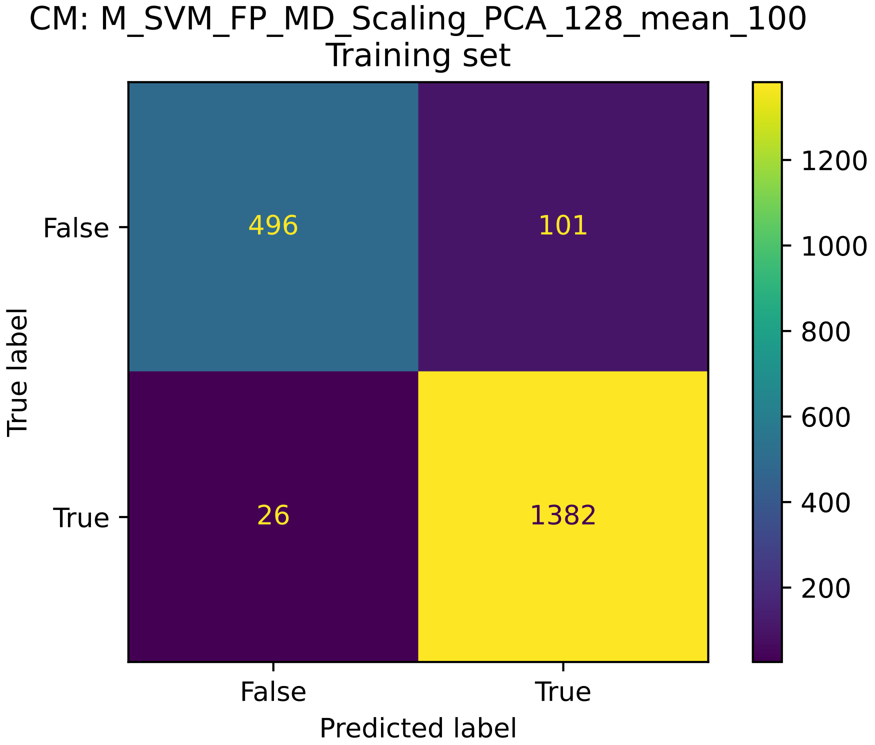
CM: M_SVM_FP_MD_Scaling_PCA_128_mean_100
Training set
True label
False
True
496
101
26
1382
Predicted label
False
True
1200
1000
800
600
400
200

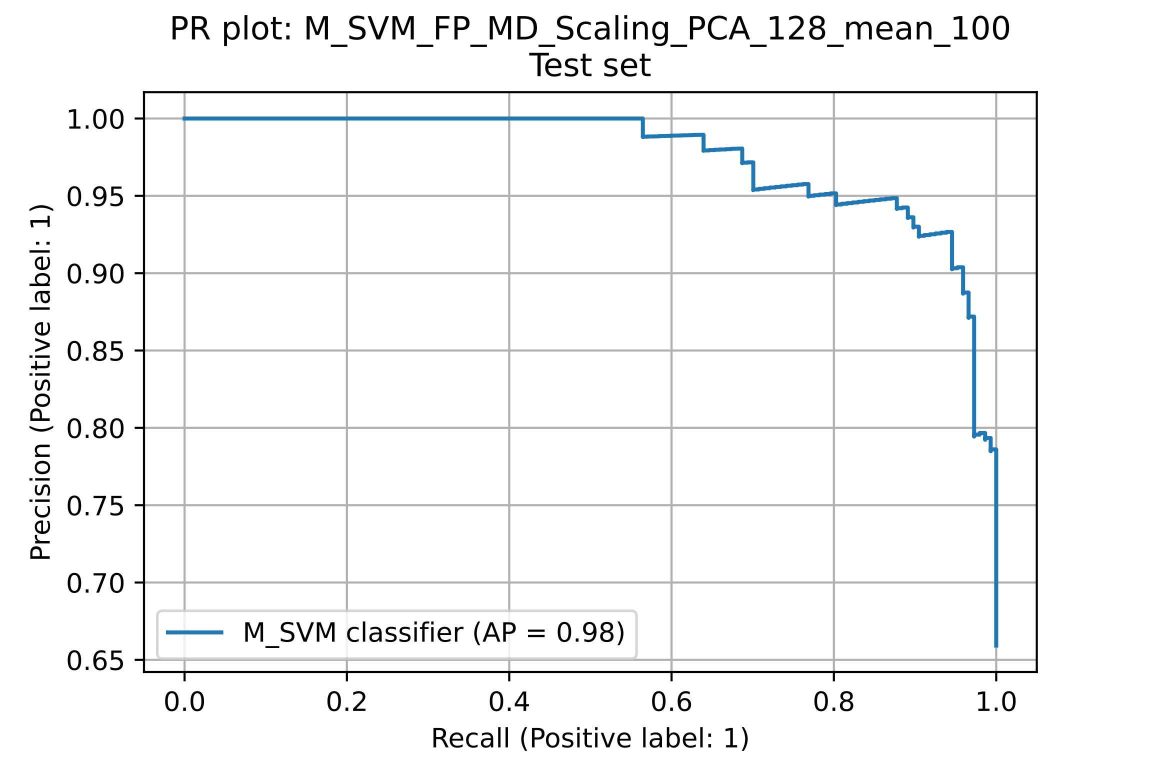

PR plot: M_SVM_FP_MD_Scaling_PCA_128_mean_100
Test set
1.00
0.95
0.90
0.85
0.80
0.75
0.70
0.65
Precision (Positive label: 1)
0.0
0.2
0.4
0.6
0.8
1.0
Recall (Positive label: 1)
M_SVM classifier (AP = 0.98)

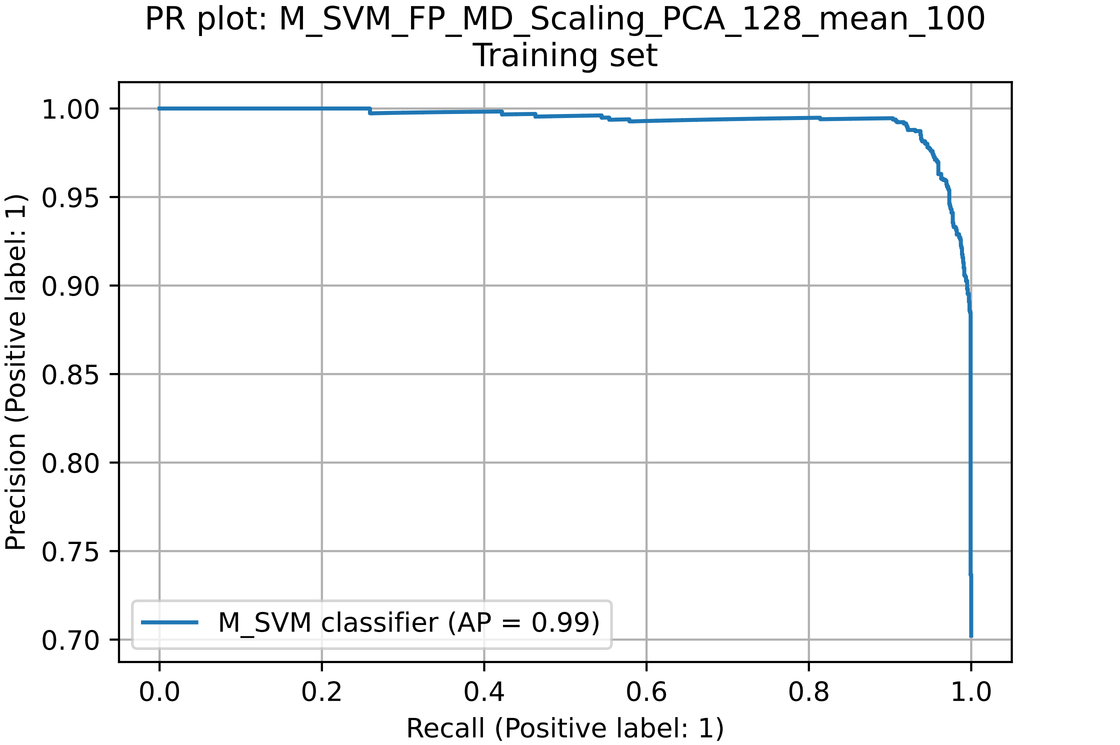
PR plot: M_SVM_FP_MD_Scaling_PCA_128_mean_100
Training set
Precision (Positive label: 1)
Recall (Positive label: 1)
1.00
0.95
0.90
0.85
0.80
0.75
0.70
0.0
0.2
0.4
0.6
0.8
1.0
M_SVM classifier (AP = 0.99)

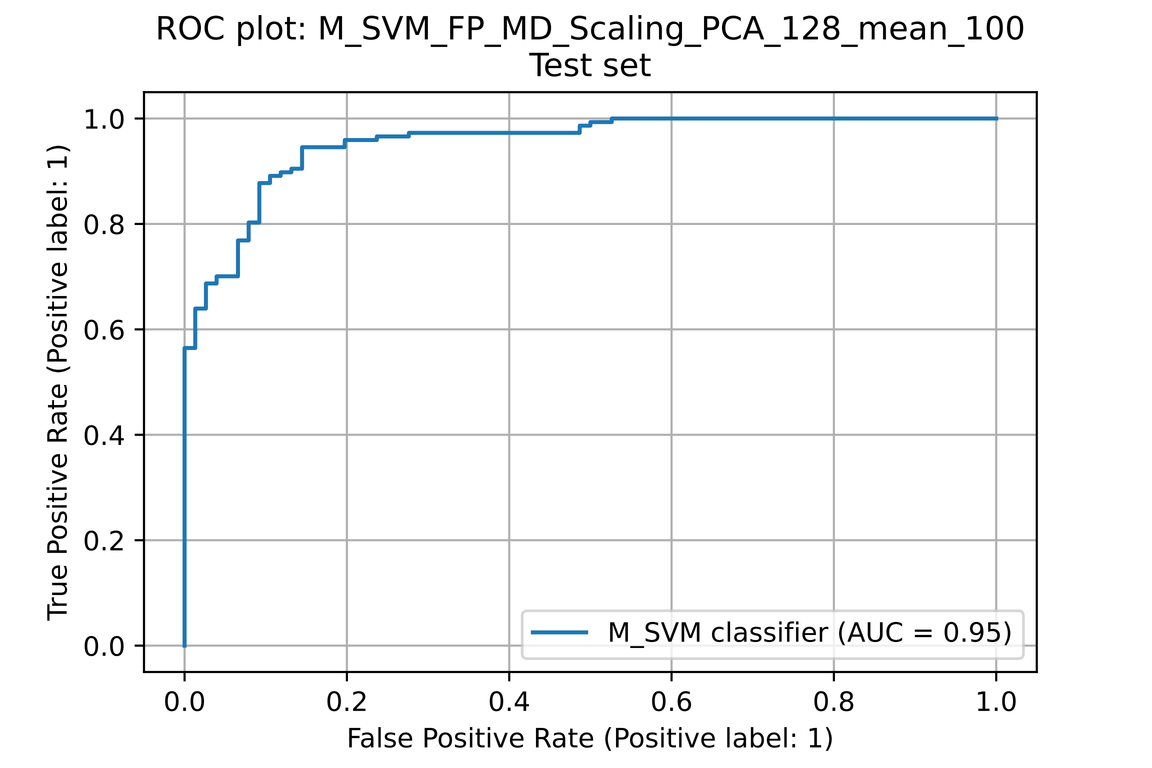
ROC plot: M_SVM_FP_MD_Scaling_PCA_128_mean_100
Test set
True Positive Rate (Positive label: 1)
False Positive Rate (Positive label: 1)
M_SVM classifier (AUC = 0.95)
0.0
0.2
0.4
0.6
0.8
1.0

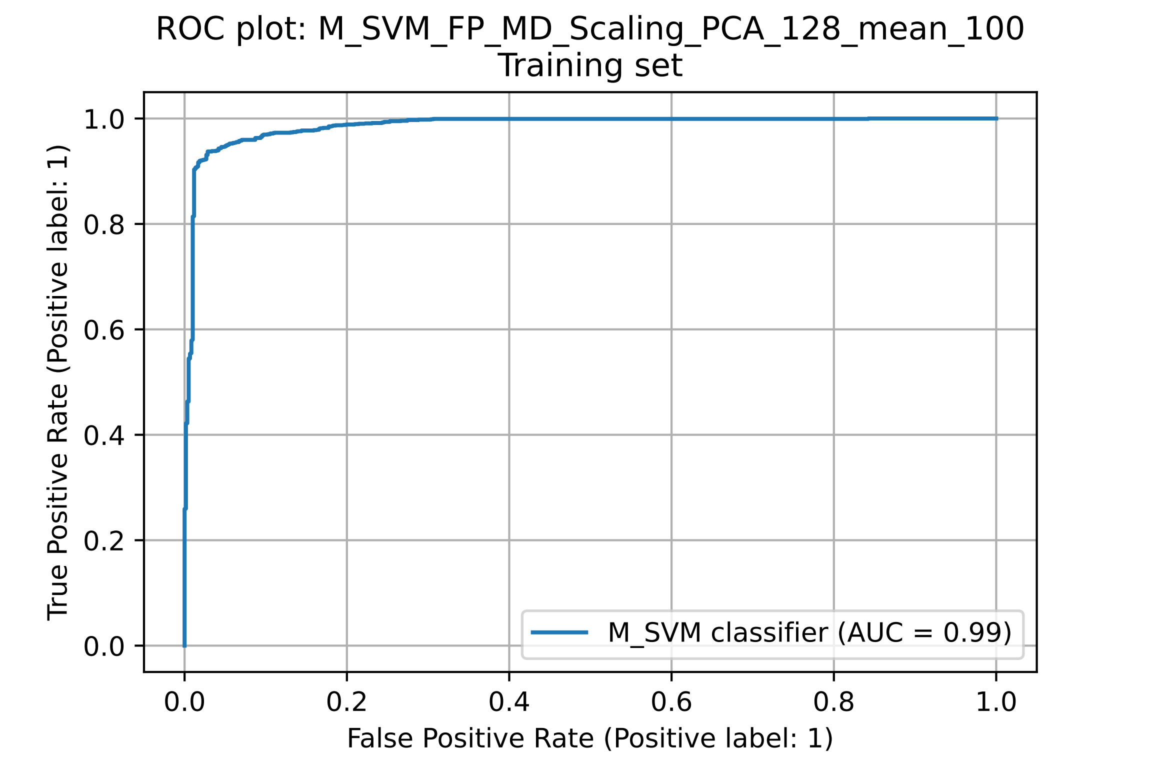

ROC plot: M_SVM_FP_MD_Scaling_PCA_128_mean_100
Training set
True Positive Rate (Positive label: 1)
False Positive Rate (Positive label: 1)
0.0
0.2
0.4
0.6
0.8
1.0
M_SVM classifier (AUC = 0.99)

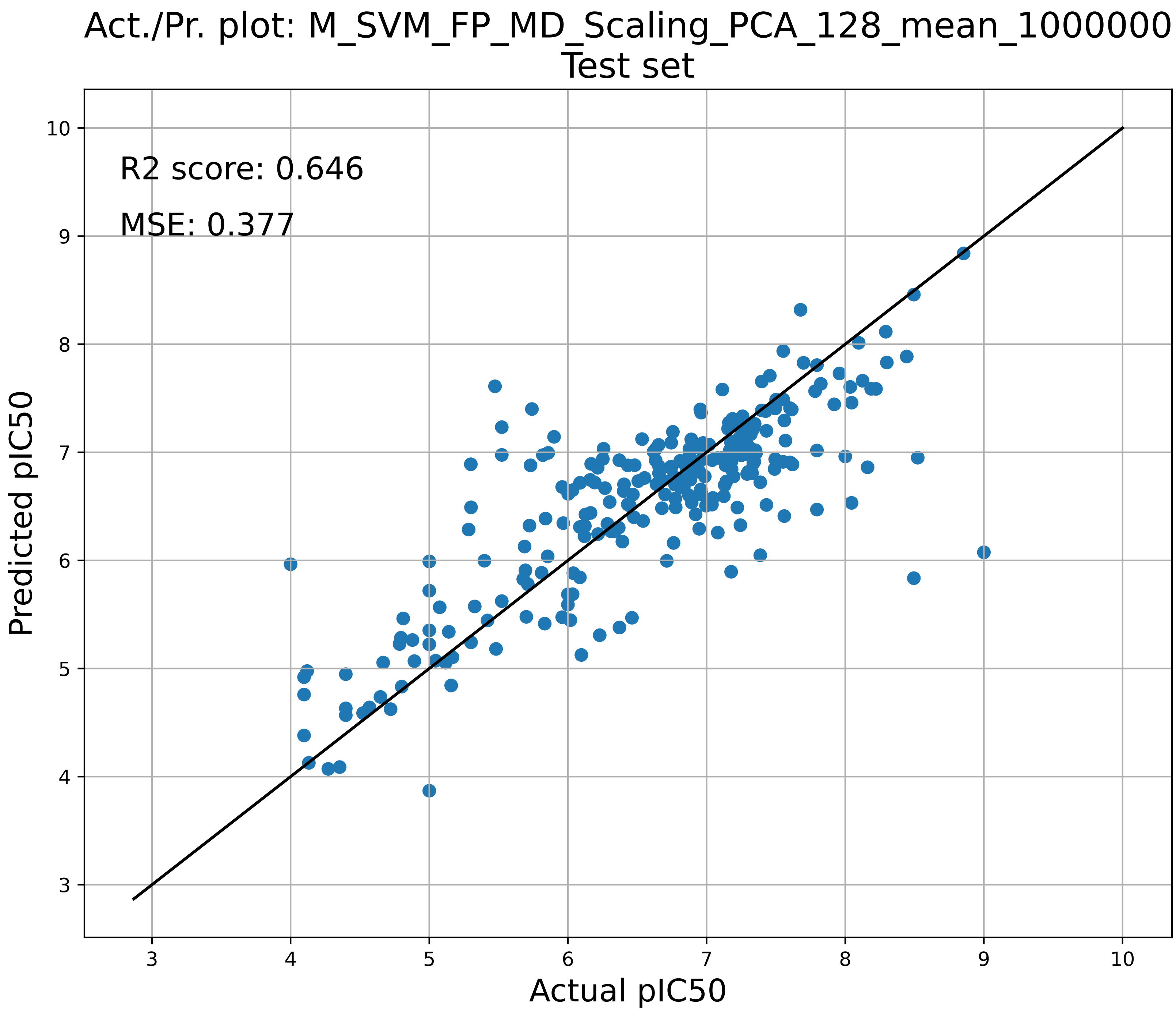
Act./Pr. plot: M_SVM_FP_MD_Scaling_PCA_128_mean_1000000
Test set
R2 score: 0.646
MSE: 0.377
Predicted pIC50
Actual pIC50
3
4
5
6
7
8
9
10

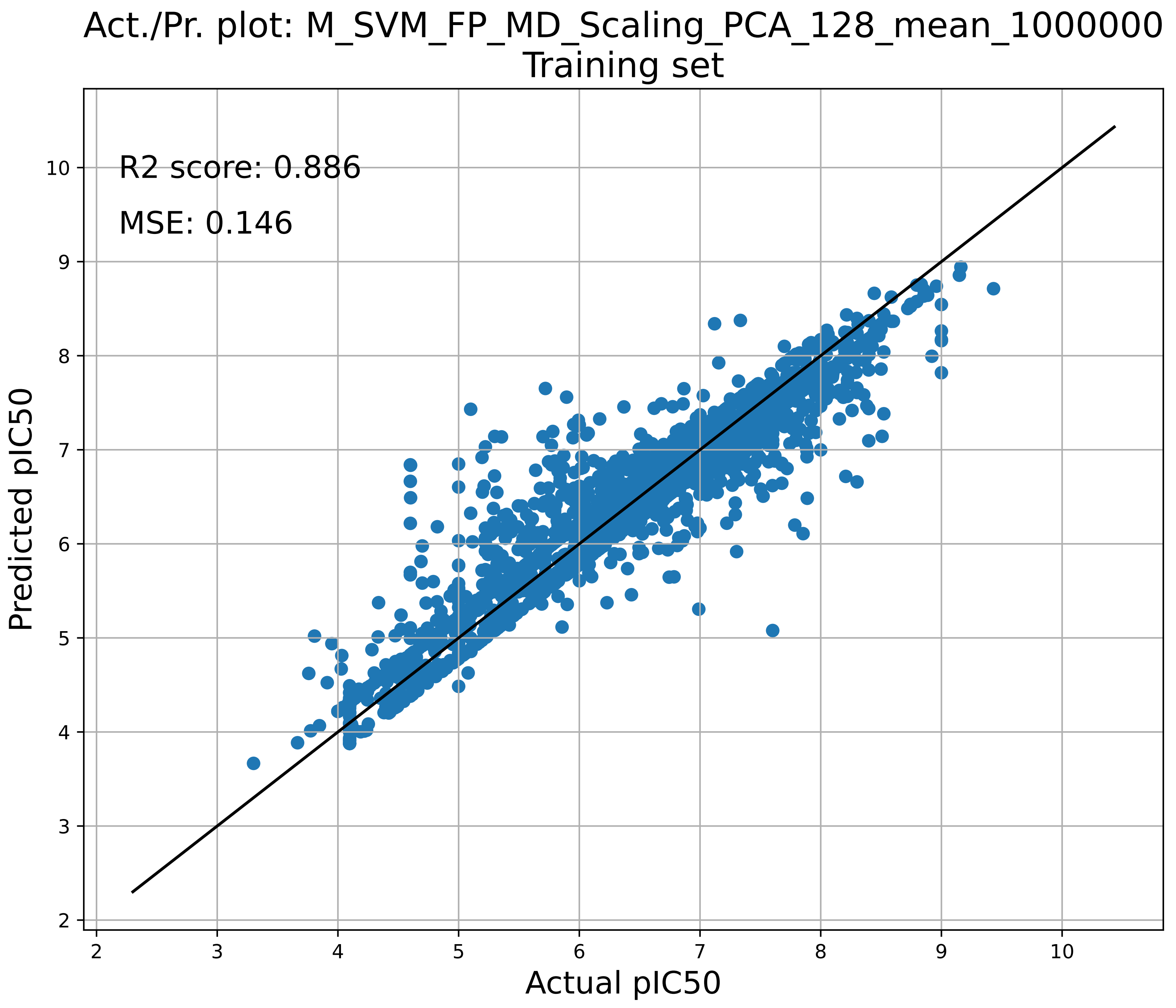

Act./Pr. plot: M_SVM_FP_MD_Scaling_PCA_128_mean_1000000
Training set
R2 score: 0.886
MSE: 0.146
Predicted pIC50
Actual pIC50
2
3
4
5
6
7
8
9
10

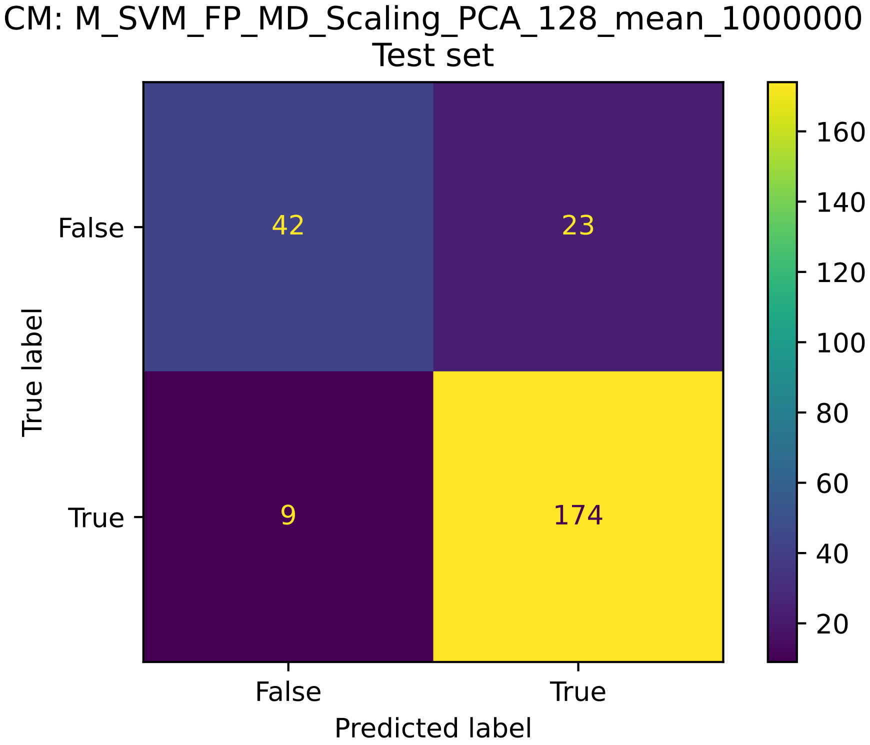
CM: M_SVM_FP_MD_Scaling_PCA_128_mean_1000000
Test set
True label
False
True
42
23
9
174
False
True
Predicted label
160
140
120
100
80
60
40
20

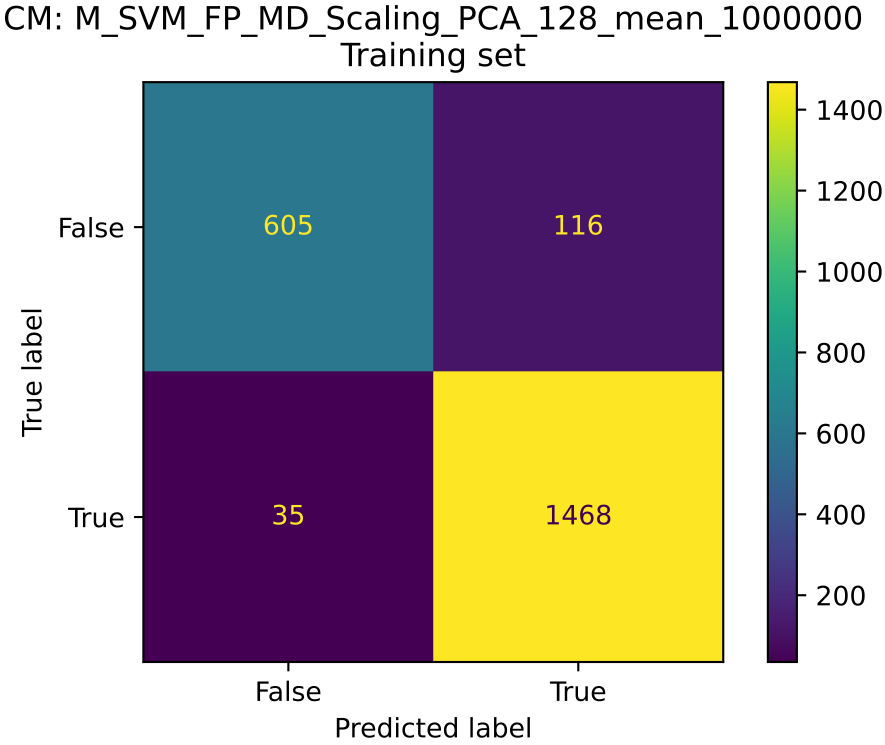

CM: M_SVM_FP_MD_Scaling_PCA_128_mean_1000000
Training set
True label
False
True
605
116
35
1468
Predicted label
False
True
1400
1200
1000
800
600
400
200

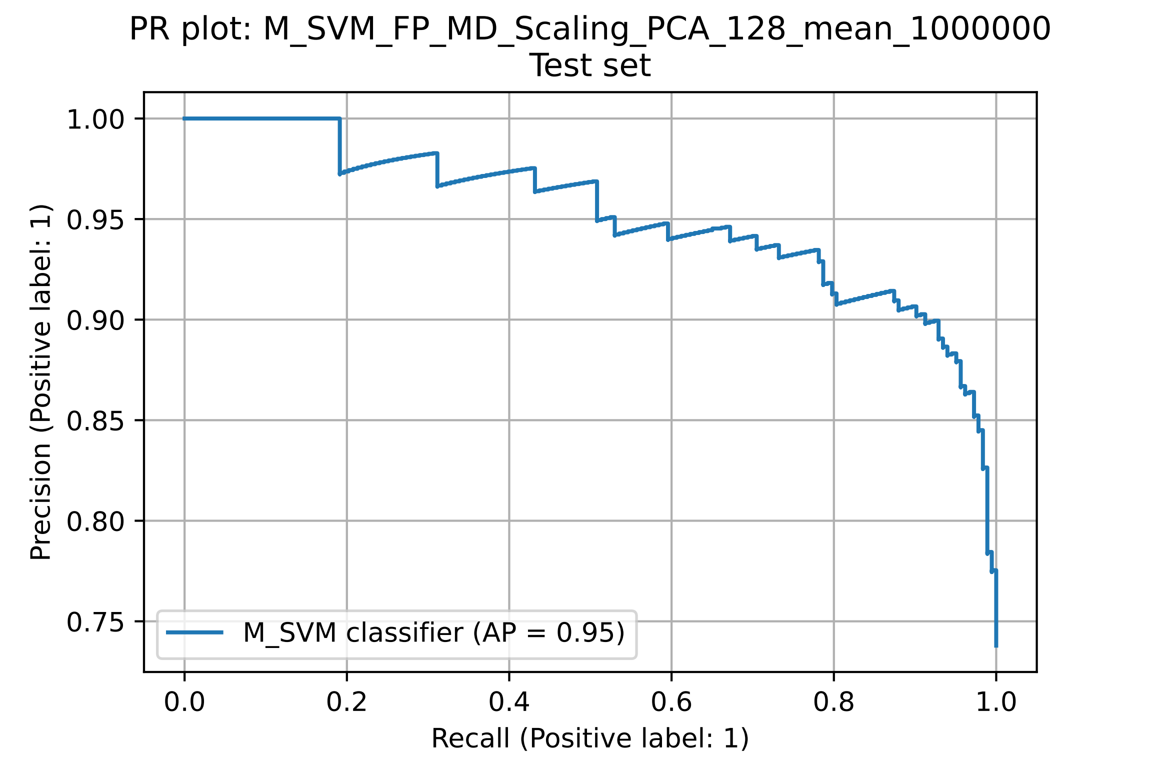

PR plot: M_SVM_FP_MD_Scaling_PCA_128_mean_1000000
Test set
Precision (Positive label: 1)
1.00
0.95
0.90
0.85
0.80
0.75
Recall (Positive label: 1)
0.0
0.2
0.4
0.6
0.8
1.0
M_SVM classifier (AP = 0.95)

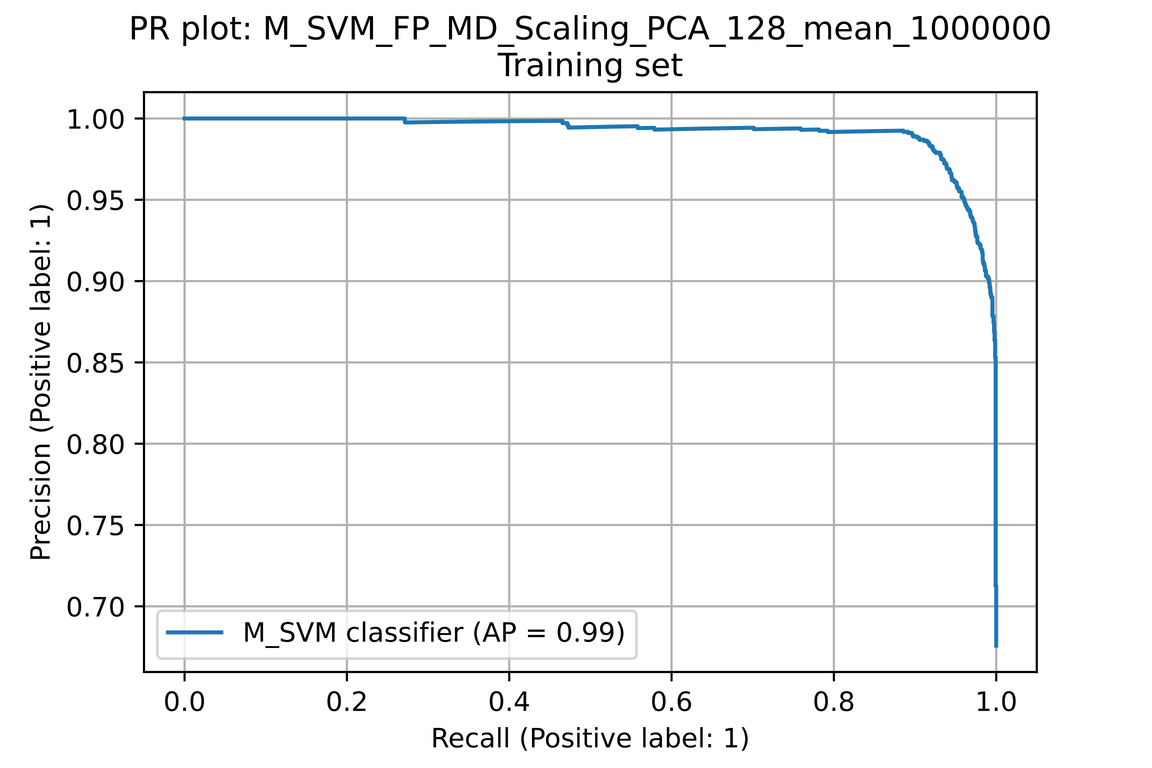
PR plot: M_SVM_FP_MD_Scaling_PCA_128_mean_1000000
Training set
1.00
0.95
0.90
0.85
0.80
0.75
0.70
Precision (Positive label: 1)
0.0
0.2
0.4
0.6
0.8
1.0
Recall (Positive label: 1)
M_SVM classifier (AP = 0.99)

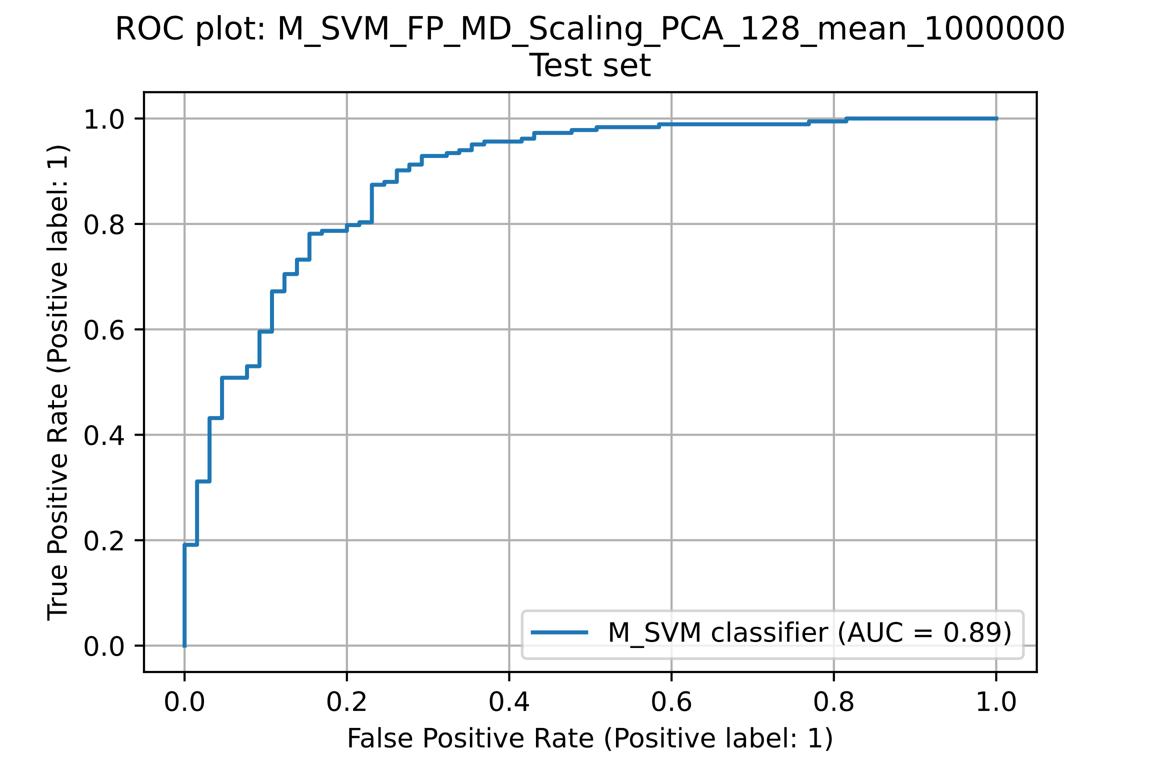

ROC plot: M_SVM_FP_MD_Scaling_PCA_128_mean_1000000
Test set
True Positive Rate (Positive label: 1)
False Positive Rate (Positive label: 1)
0.0
0.2
0.4
0.6
0.8
1.0
M_SVM classifier (AUC = 0.89)

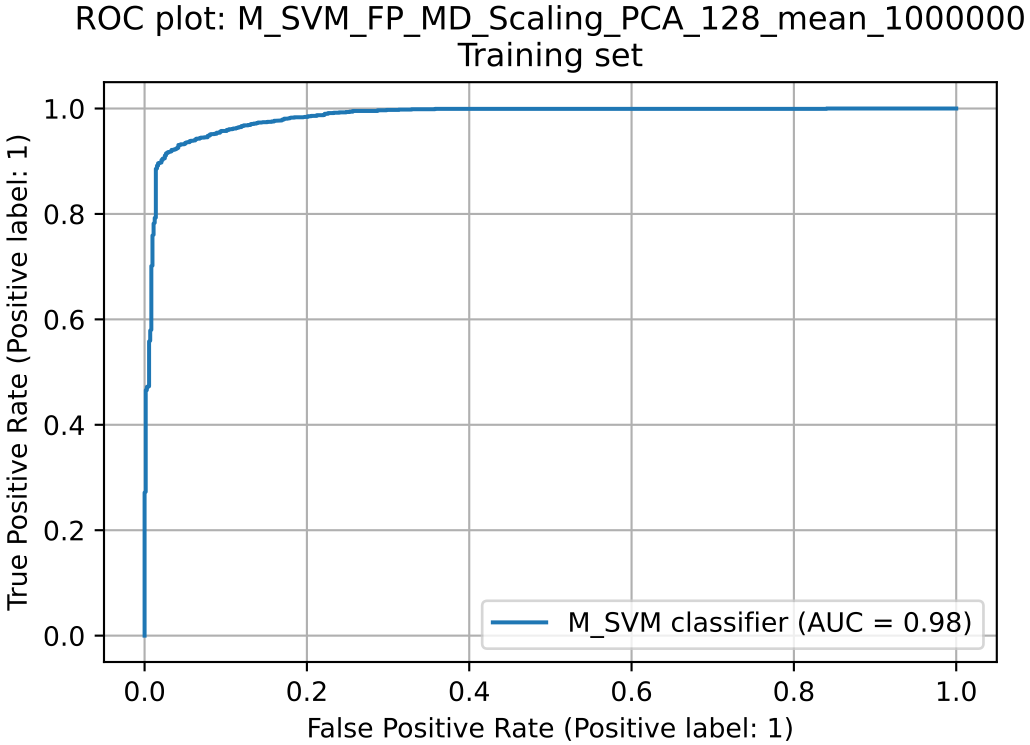
ROC plot: M_SVM_FP_MD_Scaling_PCA_128_mean_1000000
Training set
True Positive Rate (Positive label: 1)
False Positive Rate (Positive label: 1)
0.0
0.2
0.4
0.6
0.8
1.0
M_SVM classifier (AUC = 0.98)

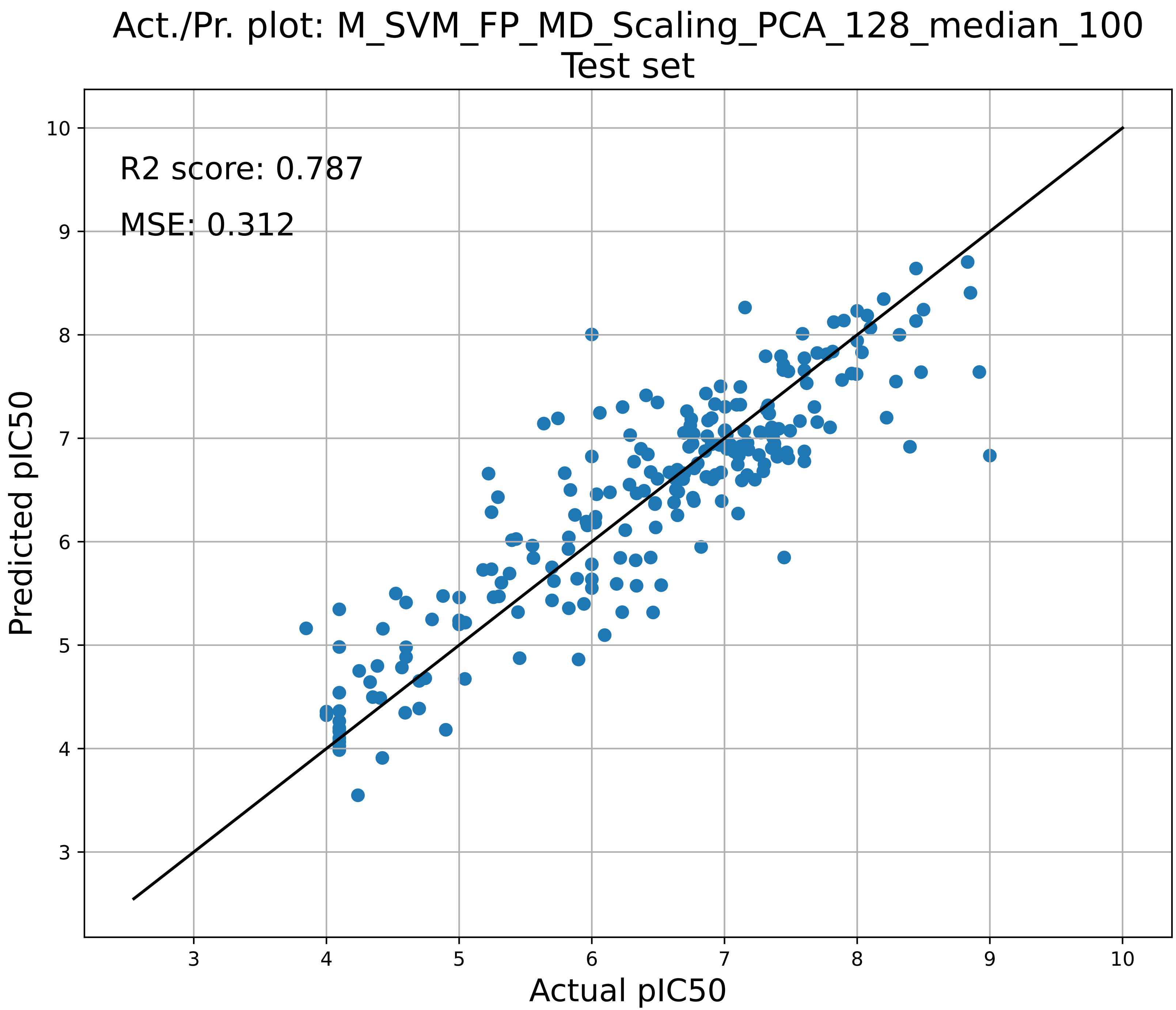

Act./Pr. plot: M_SVM_FP_MD_Scaling_PCA_128_median_100
Test set
R2 score: 0.787
MSE: 0.312
Predicted pIC50
Actual pIC50
3
4
5
6
7
8
9
10

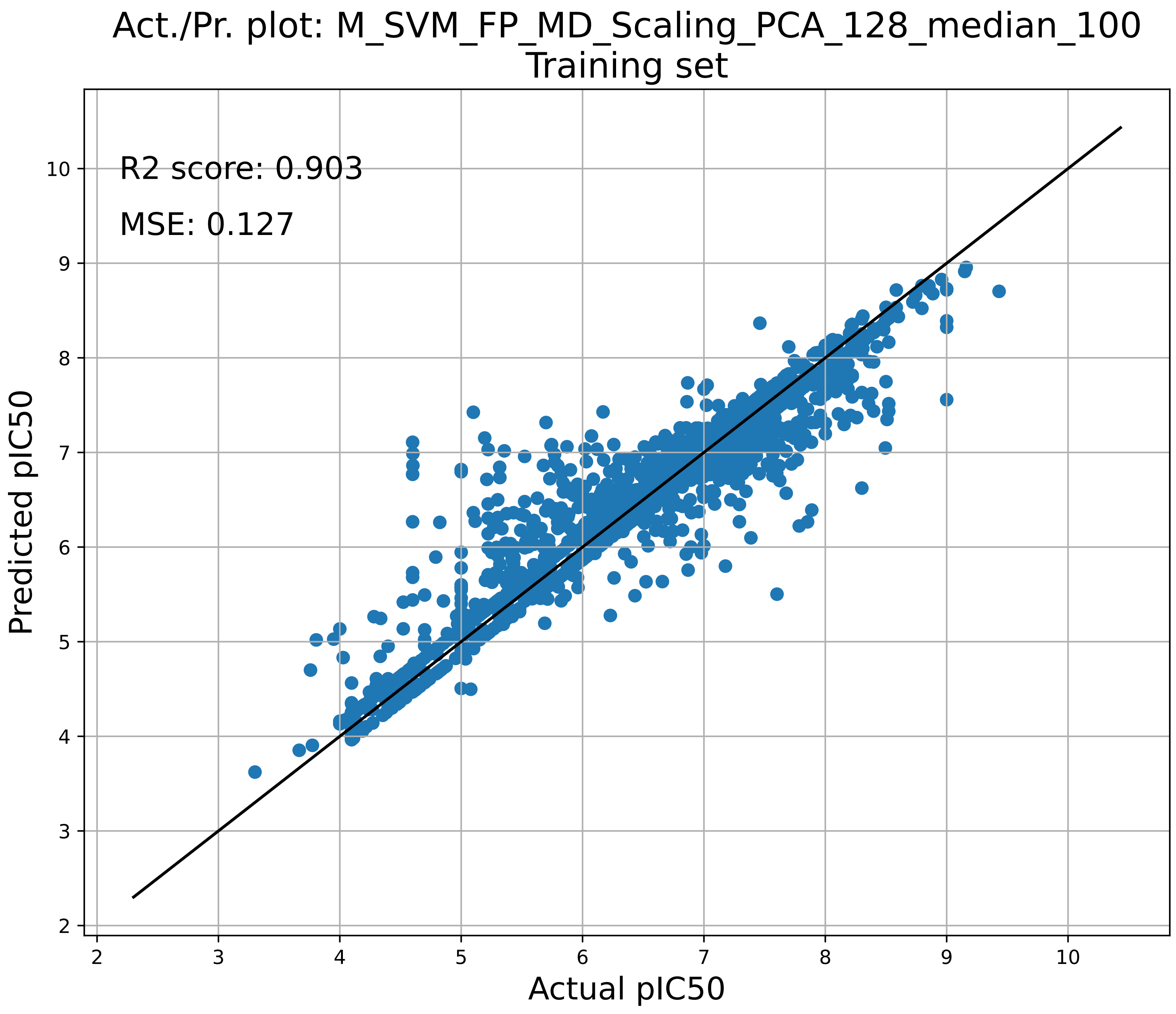

Act./Pr. plot: M_SVM_FP_MD_Scaling_PCA_128_median_100
Training set
R2 score: 0.903
MSE: 0.127
Predicted pIC50
Actual pIC50
2
3
4
5
6
7
8
9
10

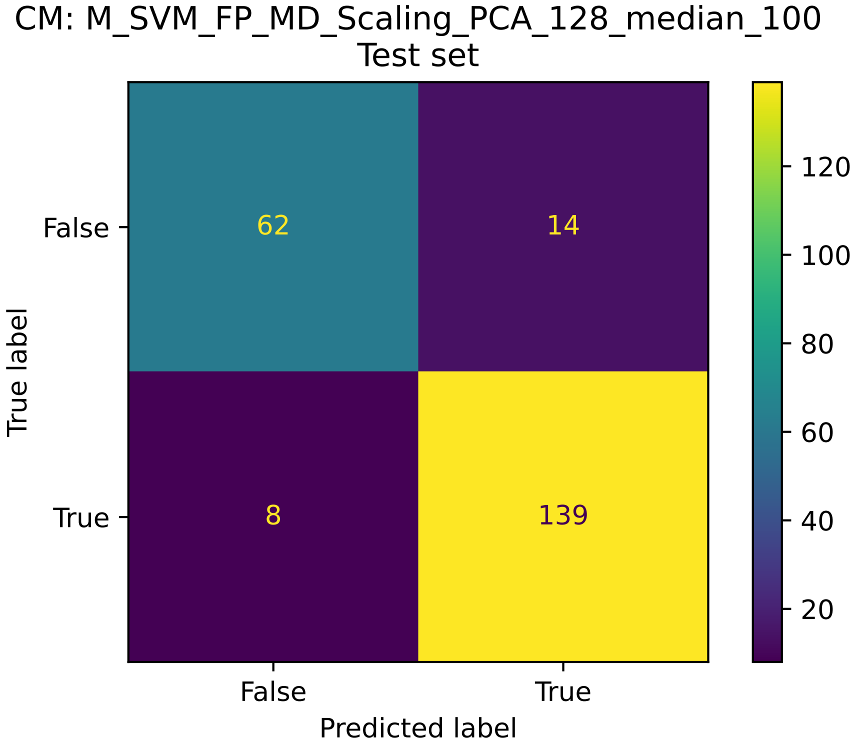

CM: M_SVM_FP_MD_Scaling_PCA_128_median_100
Test set
True label
Predicted label
False
True
62
14
8
139
20
40
60
80
100
120

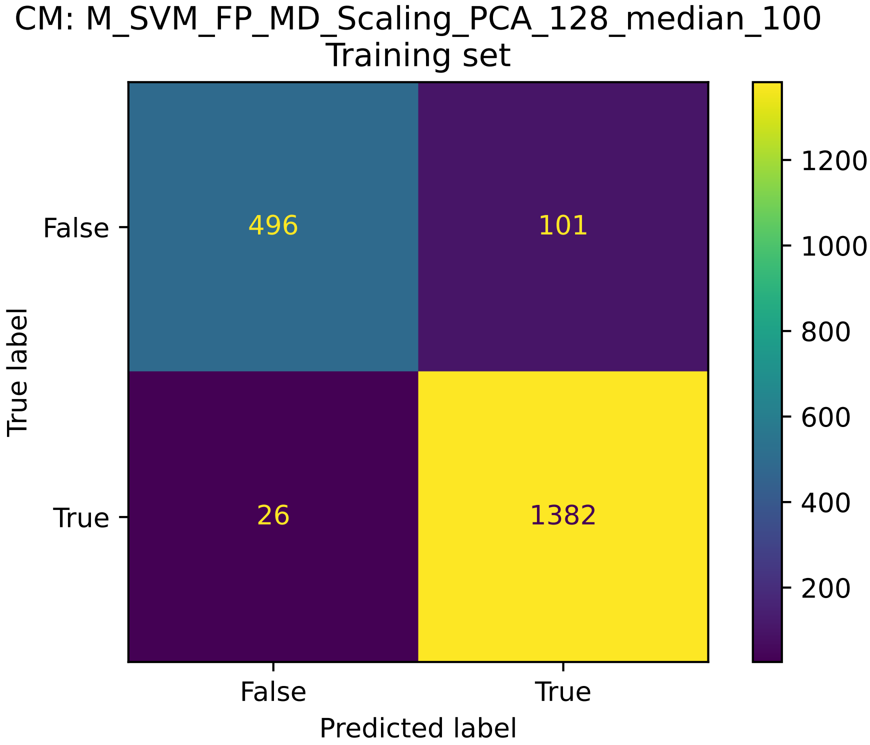
CM: M_SVM_FP_MD_Scaling_PCA_128_median_100
Training set
496
101
26
1382
False
True
True label
False
True
Predicted label
200
400
600
800
1000
1200

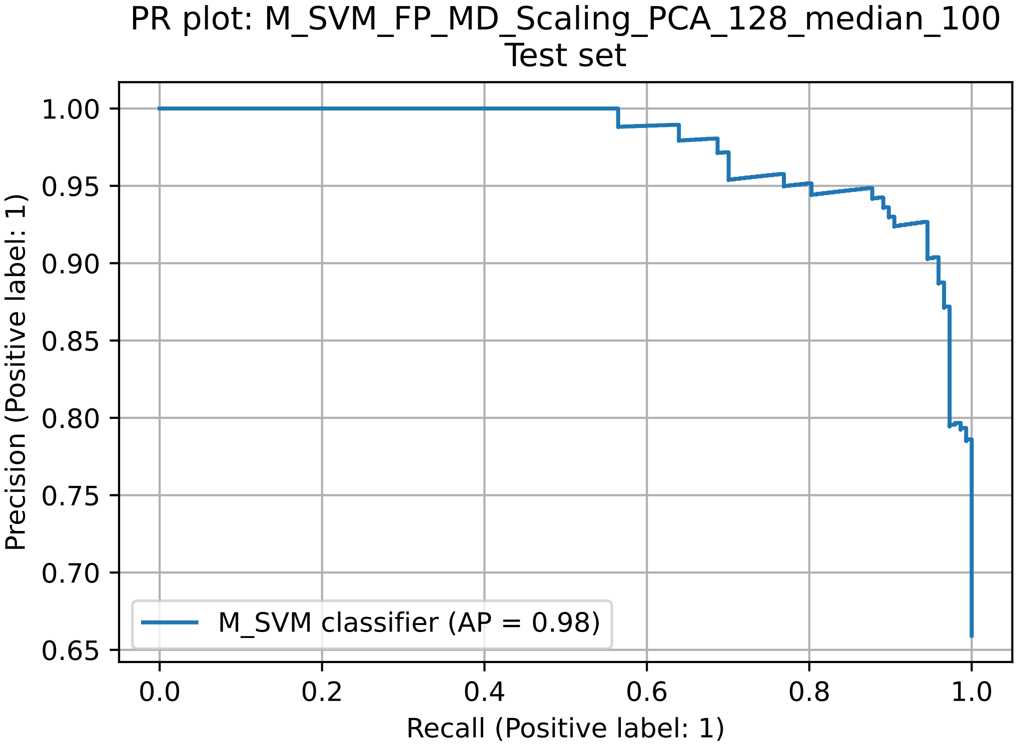

PR plot: M_SVM_FP_MD_Scaling_PCA_128_median_100
Test set
Precision (Positive label: 1)
Recall (Positive label: 1)
1.00
0.95
0.90
0.85
0.80
0.75
0.70
0.65
0.0
0.2
0.4
0.6
0.8
1.0
M_SVM classifier (AP = 0.98)

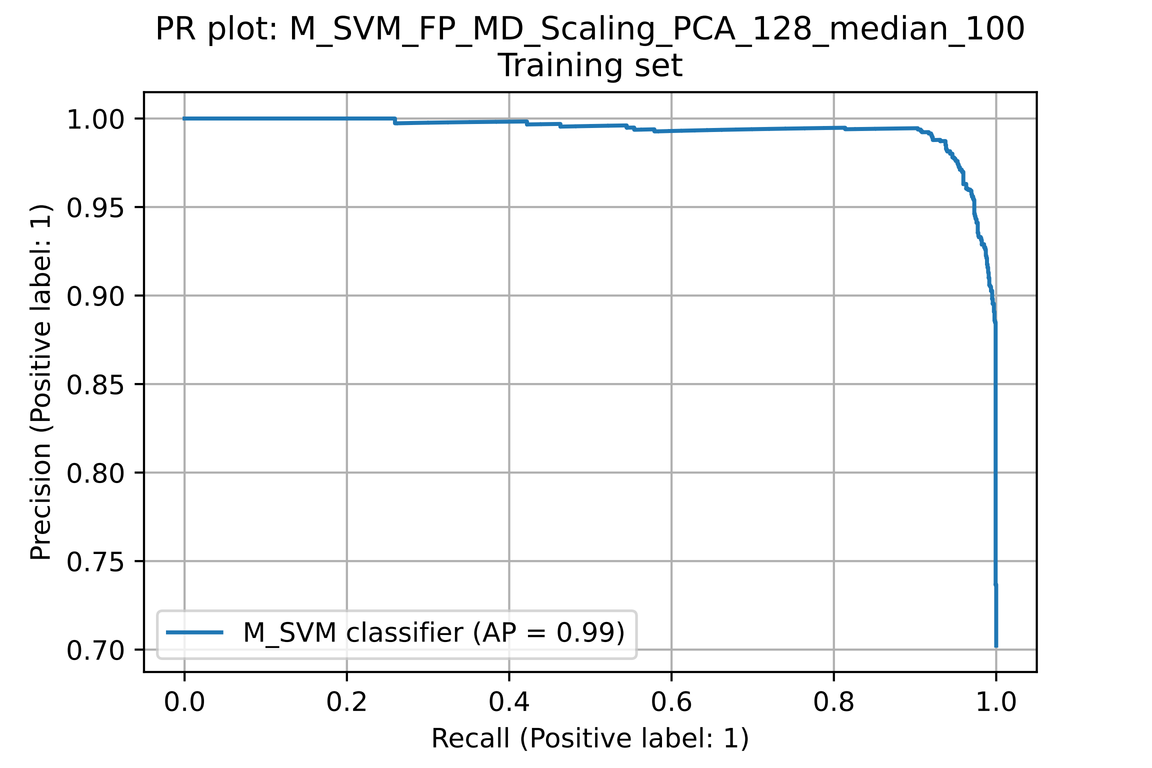
PR plot: M_SVM_FP_MD_Scaling_PCA_128_median_100
Training set
Precision (Positive label: 1)
1.00
0.95
0.90
0.85
0.80
0.75
0.70
0.0
0.2
0.4
0.6
0.8
1.0
Recall (Positive label: 1)
M_SVM classifier (AP = 0.99)

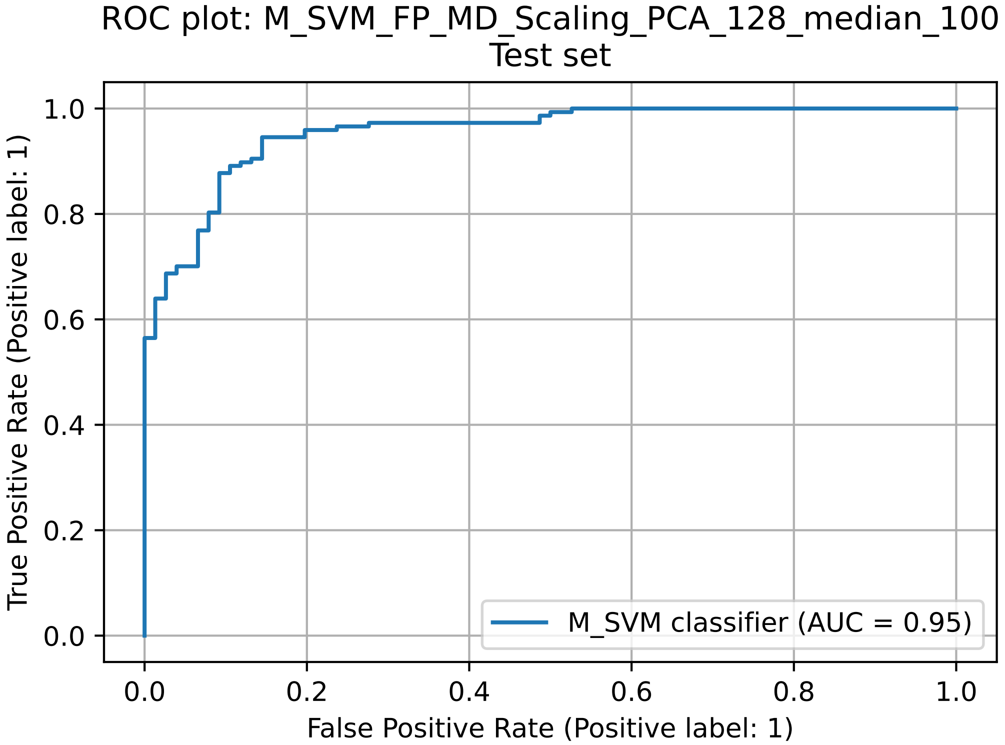
ROC plot: M_SVM_FP_MD_Scaling_PCA_128_median_100
Test set
True Positive Rate (Positive label: 1)
False Positive Rate (Positive label: 1)
0.0
0.2
0.4
0.6
0.8
1.0
M_SVM classifier (AUC = 0.95)

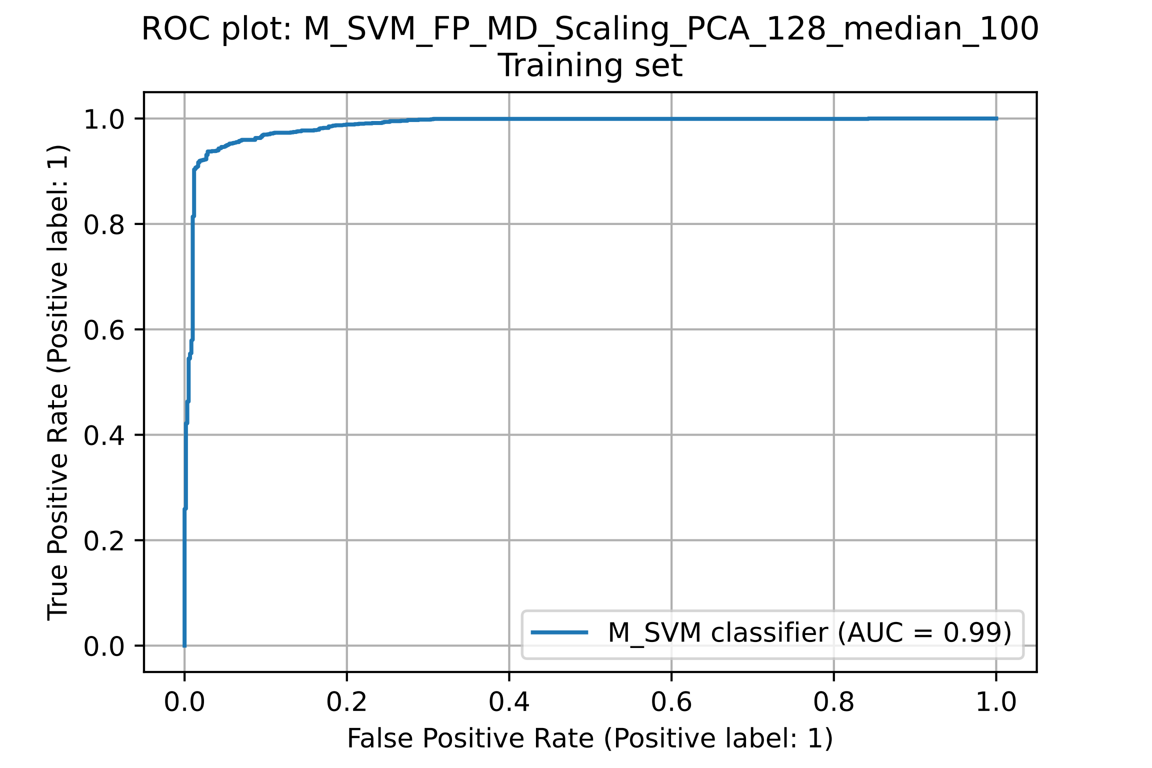
ROC plot: M_SVM_FP_MD_Scaling_PCA_128_median_100
Training set
True Positive Rate (Positive label: 1)
False Positive Rate (Positive label: 1)
0.0
0.2
0.4
0.6
0.8
1.0
M_SVM classifier (AUC = 0.99)

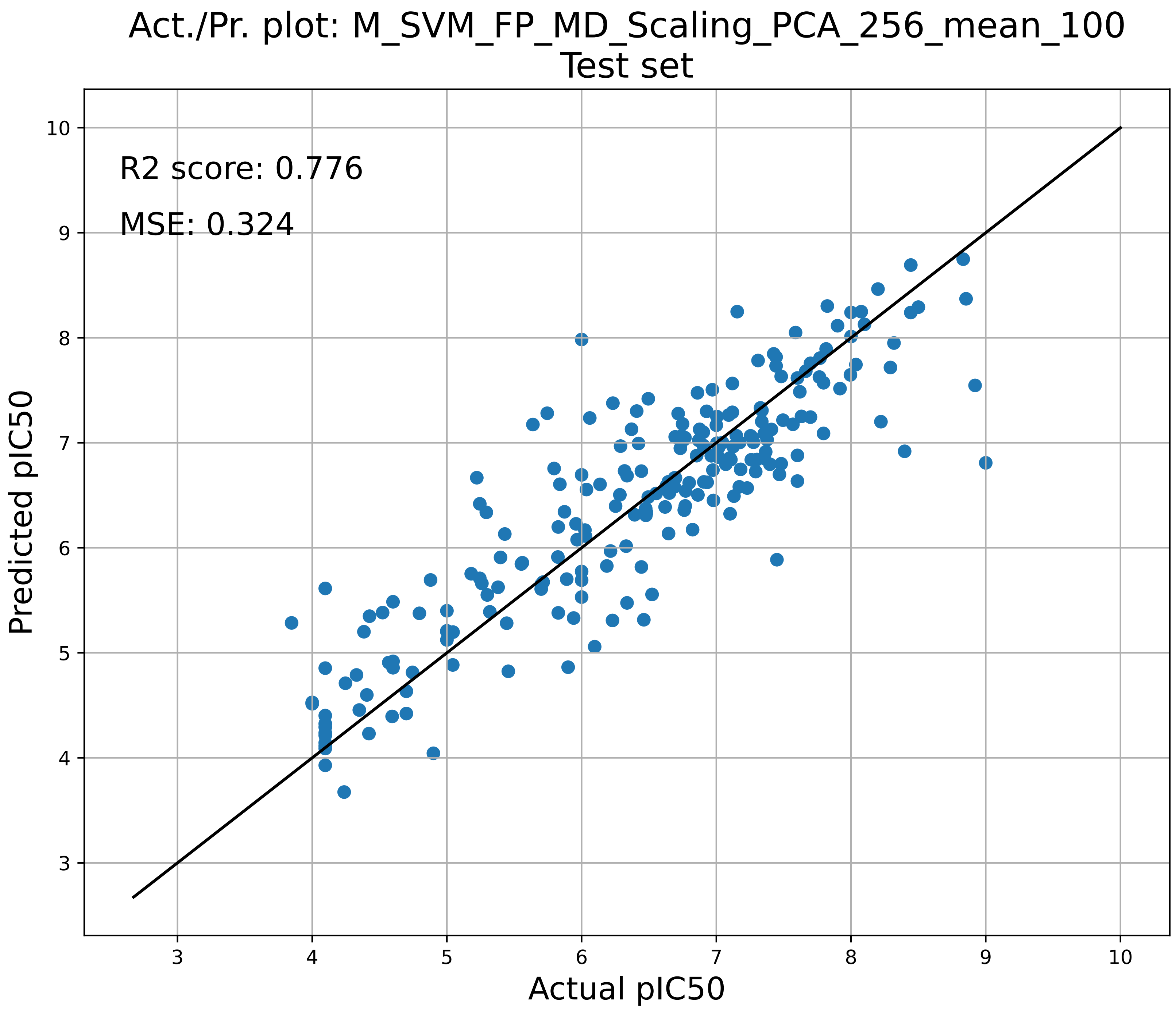
Act./Pr. plot: M_SVM_FP_MD_Scaling_PCA_256_mean_100
Test set
R2 score: 0.776
MSE: 0.324
Predicted pIC50
Actual pIC50
3
4
5
6
7
8
9
10

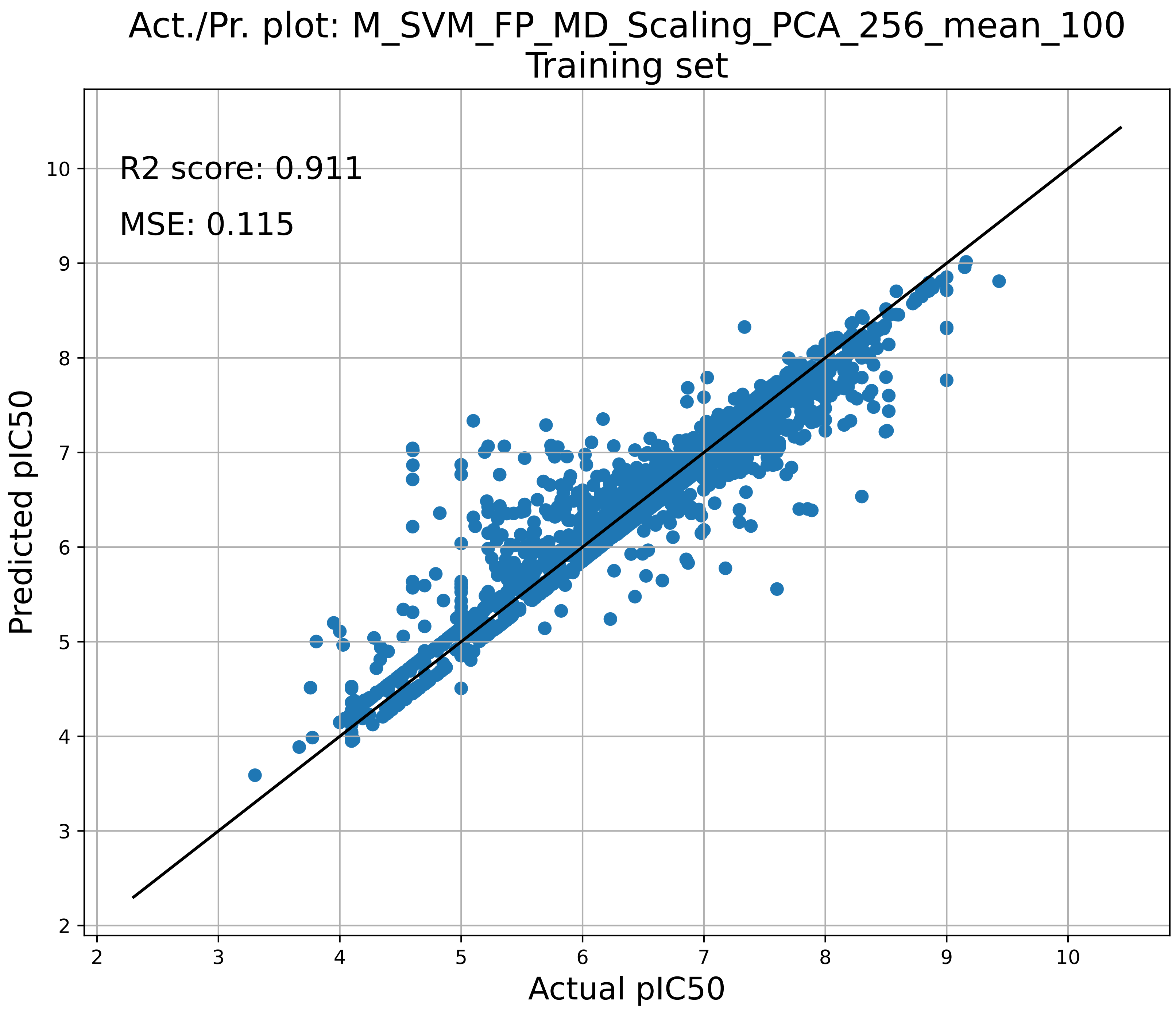
Act./Pr. plot: M_SVM_FP_MD_Scaling_PCA_256_mean_100
Training set
R2 score: 0.911
MSE: 0.115
Predicted pIC50
Actual pIC50
2
3
4
5
6
7
8
9
10

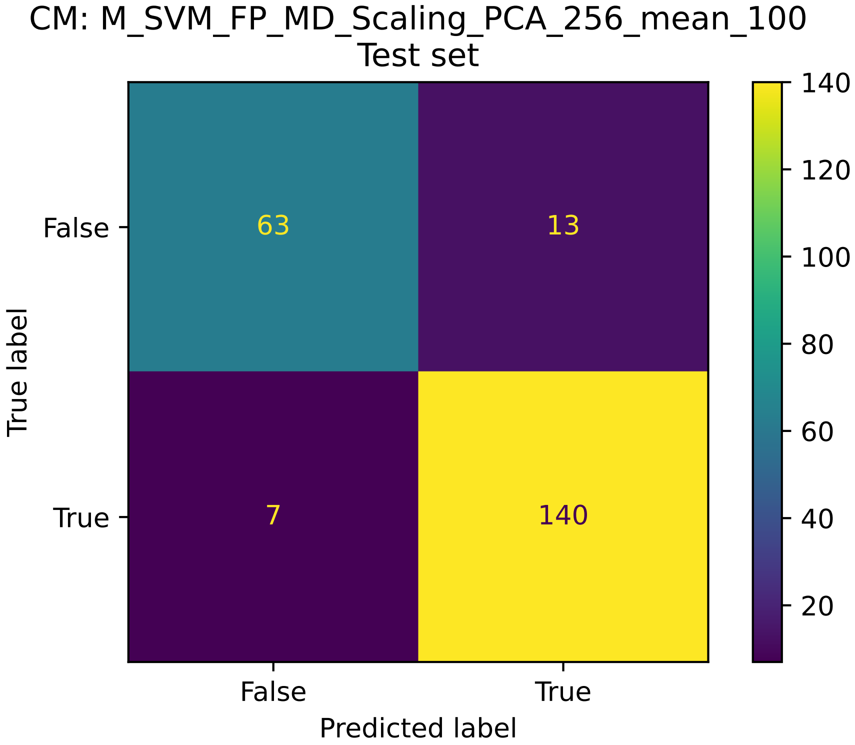
CM: M_SVM_FP_MD_Scaling_PCA_256_mean_100
Test set
True label
Predicted label
False
True
False
True
63
13
7
140
140
120
100
80
60
40
20

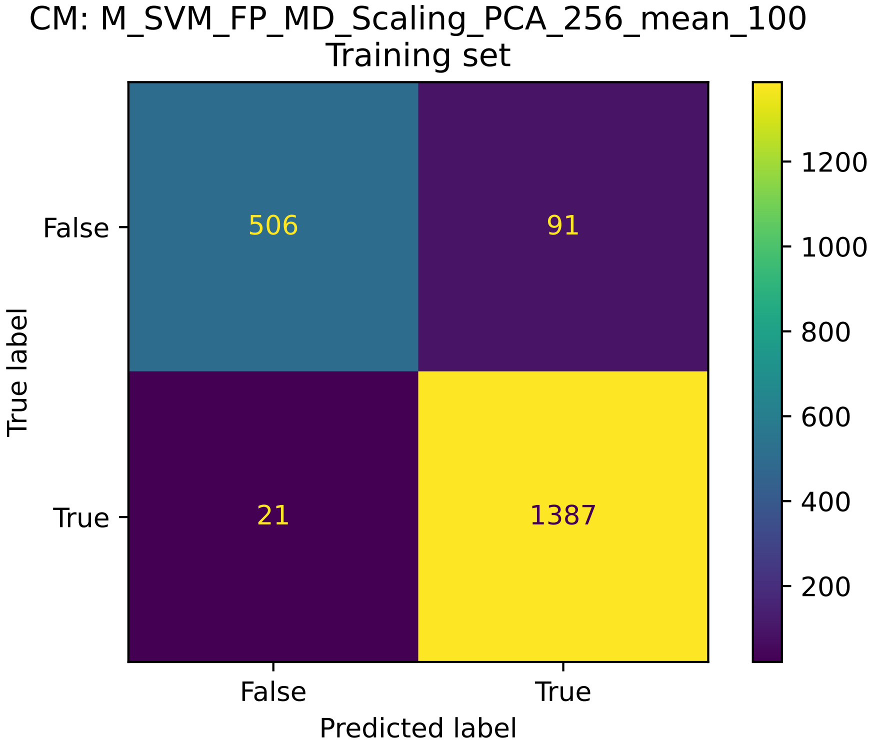
CM: M_SVM_FP_MD_Scaling_PCA_256_mean_100
Training set
True label
False
True
506
91
21
1387
Predicted label
False
True
1200
1000
800
600
400
200

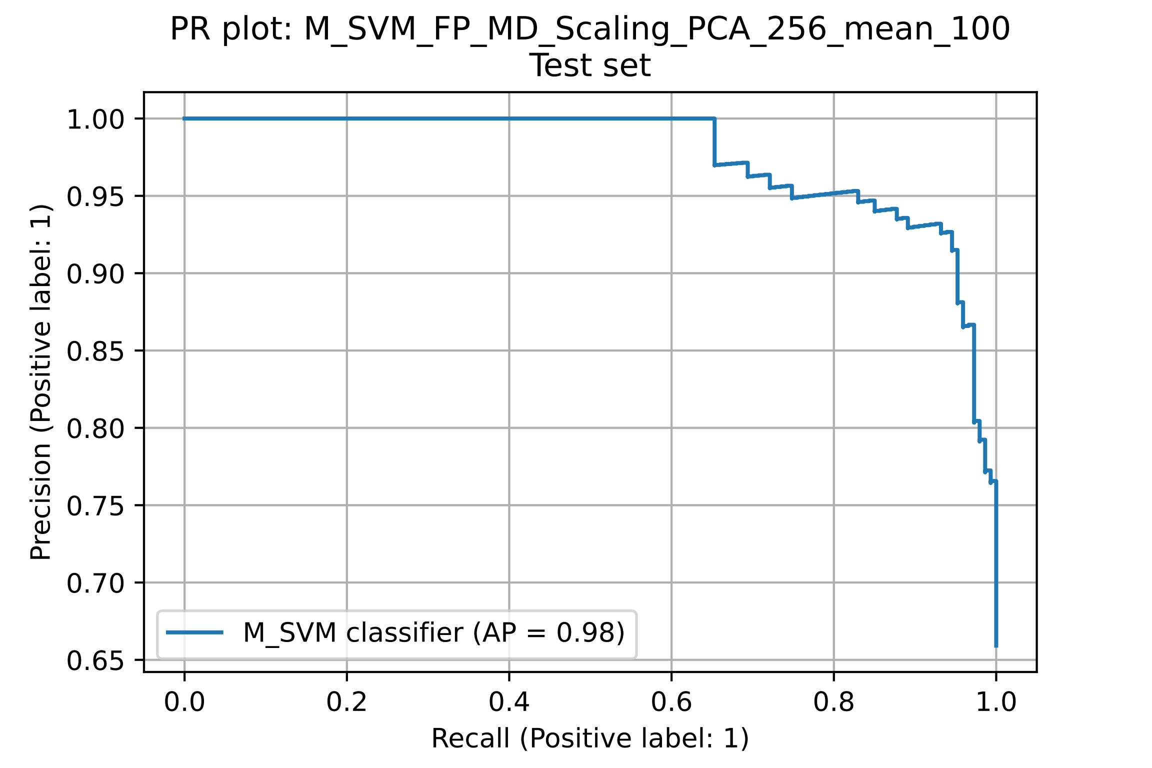
PR plot: M_SVM_FP_MD_Scaling_PCA_256_mean_100
Test set
Precision (Positive label: 1)
Recall (Positive label: 1)
1.00
0.95
0.90
0.85
0.80
0.75
0.70
0.65
0.0
0.2
0.4
0.6
0.8
1.0
M_SVM classifier (AP = 0.98)

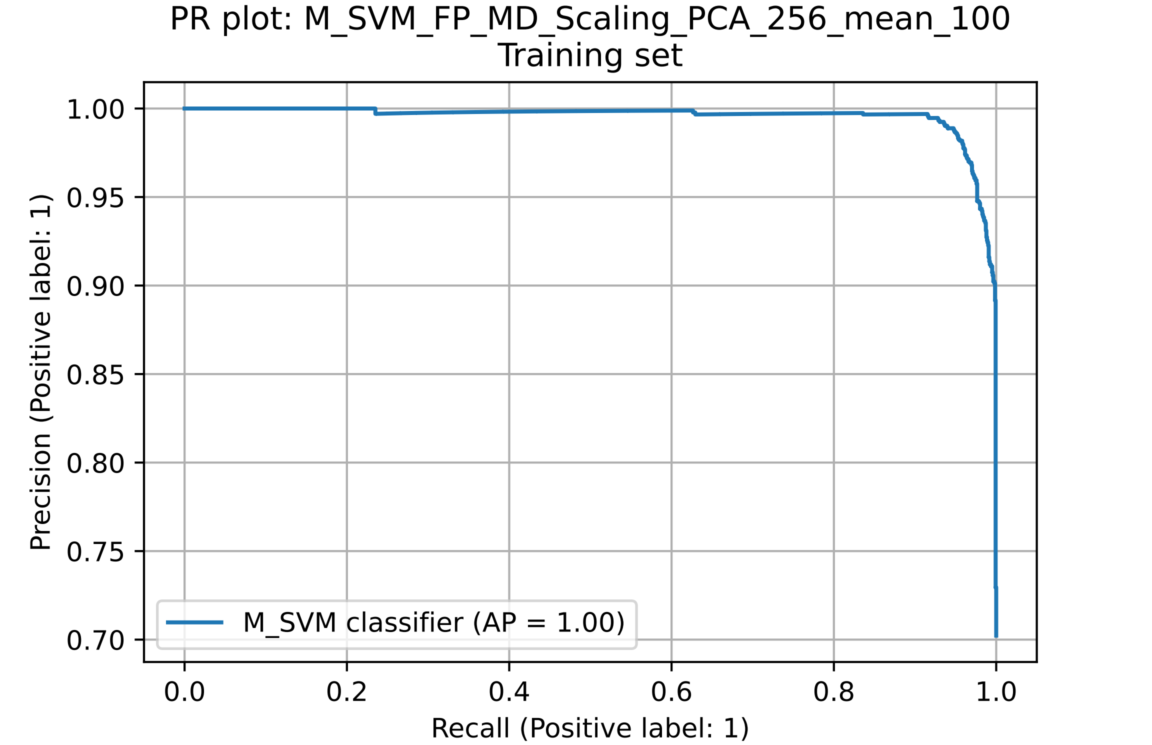
PR plot: M_SVM_FP_MD_Scaling_PCA_256_mean_100
Training set
Precision (Positive label: 1)
1.00
0.95
0.90
0.85
0.80
0.75
0.70
0.0
0.2
0.4
0.6
0.8
1.0
Recall (Positive label: 1)
M_SVM classifier (AP = 1.00)

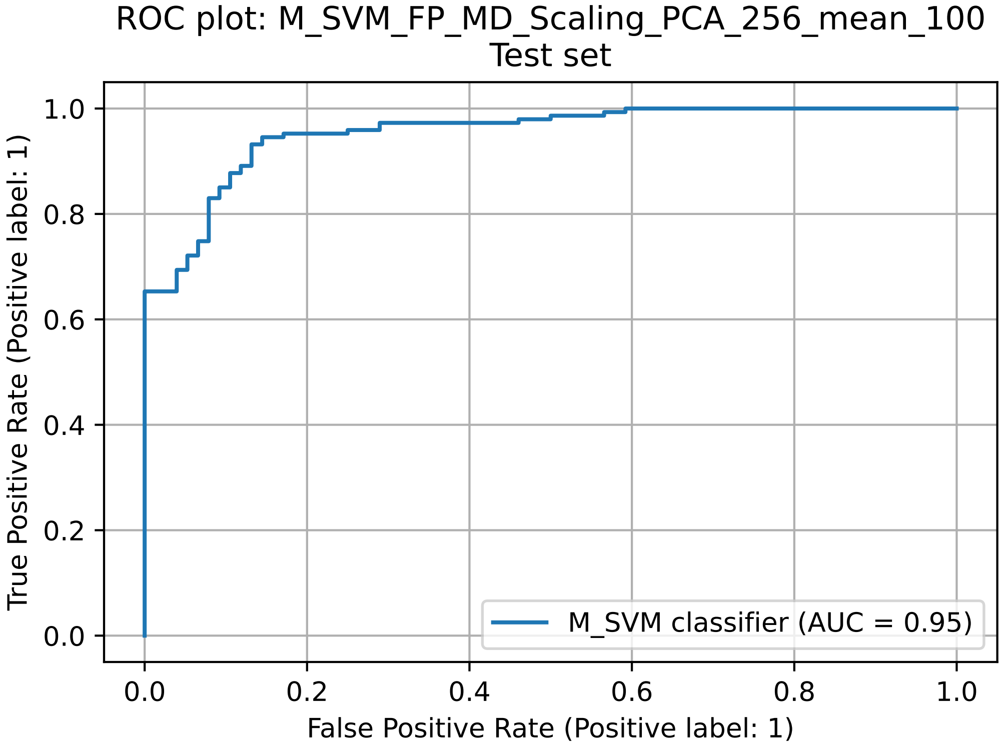
ROC plot: M_SVM_FP_MD_Scaling_PCA_256_mean_100
Test set
True Positive Rate (Positive label: 1)
False Positive Rate (Positive label: 1)
0.0
0.2
0.4
0.6
0.8
1.0
M_SVM classifier (AUC = 0.95)

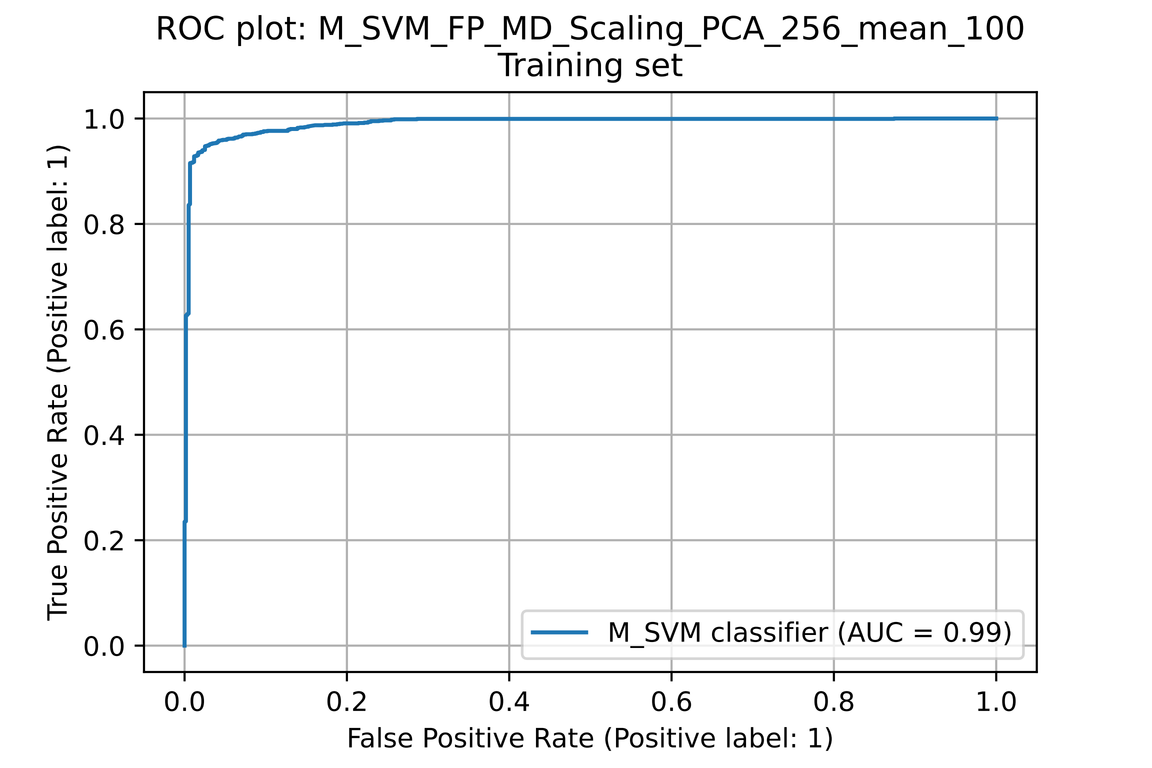
ROC plot: M_SVM_FP_MD_Scaling_PCA_256_mean_100
Training set
True Positive Rate (Positive label: 1)
False Positive Rate (Positive label: 1)
0.0
0.2
0.4
0.6
0.8
1.0
M_SVM classifier (AUC = 0.99)

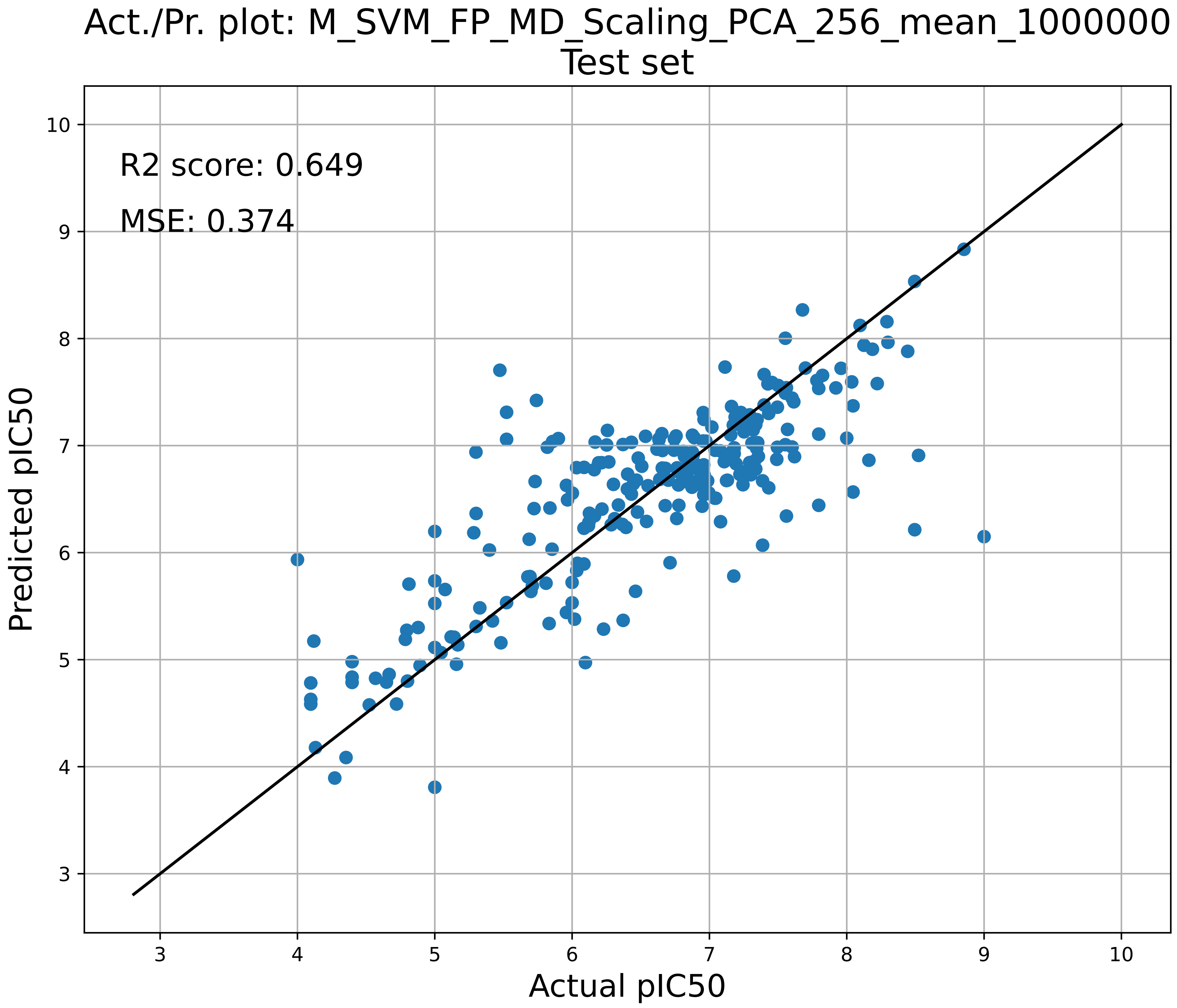
Act./Pr. plot: M_SVM_FP_MD_Scaling_PCA_256_mean_1000000
Test set
R2 score: 0.649
MSE: 0.374
Predicted pIC50
Actual pIC50
3
4
5
6
7
8
9
10

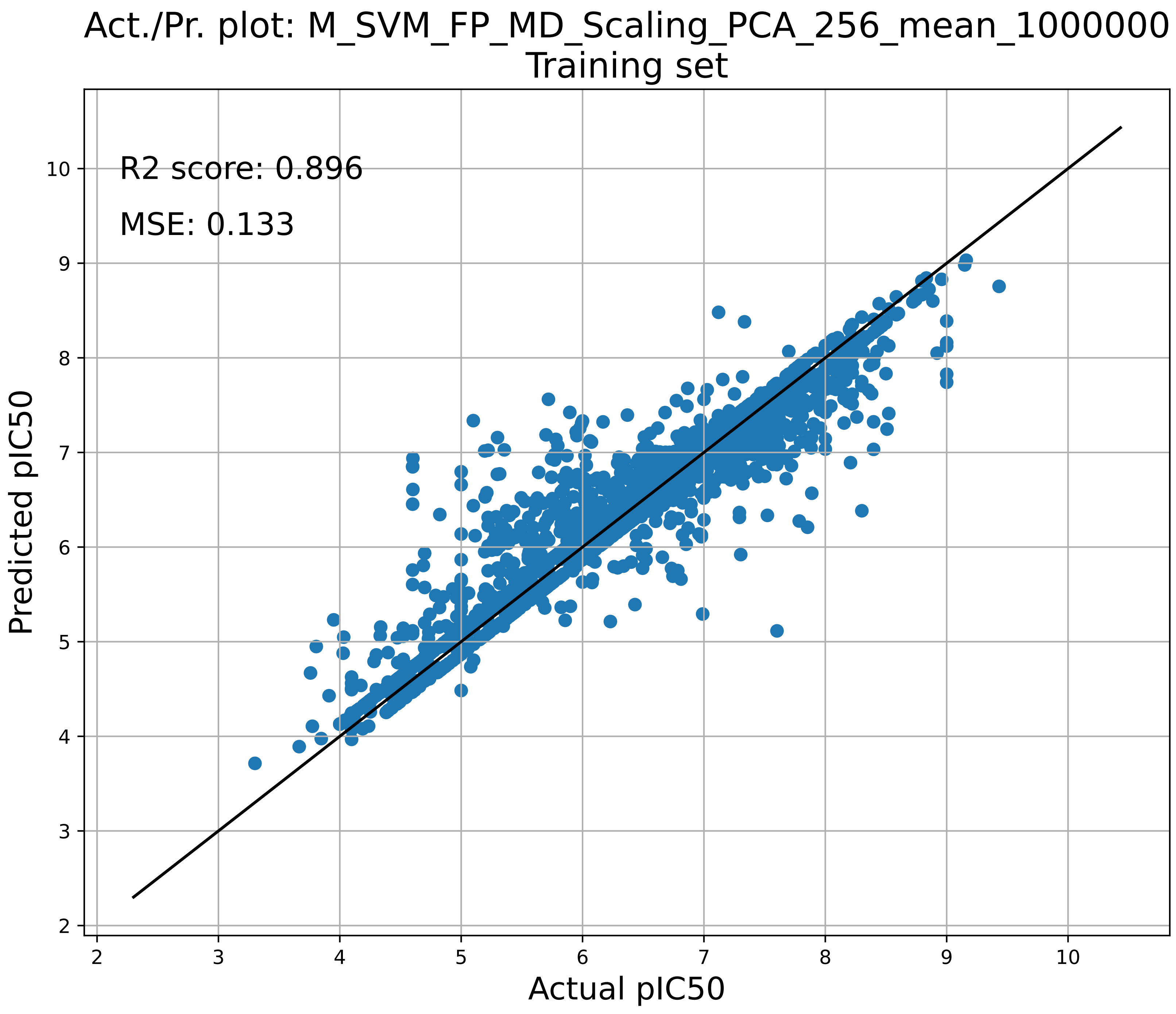

Act./Pr. plot: M_SVM_FP_MD_Scaling_PCA_256_mean_1000000
Training set
R2 score: 0.896
MSE: 0.133
Predicted pIC50
Actual pIC50
2
3
4
5
6
7
8
9
10

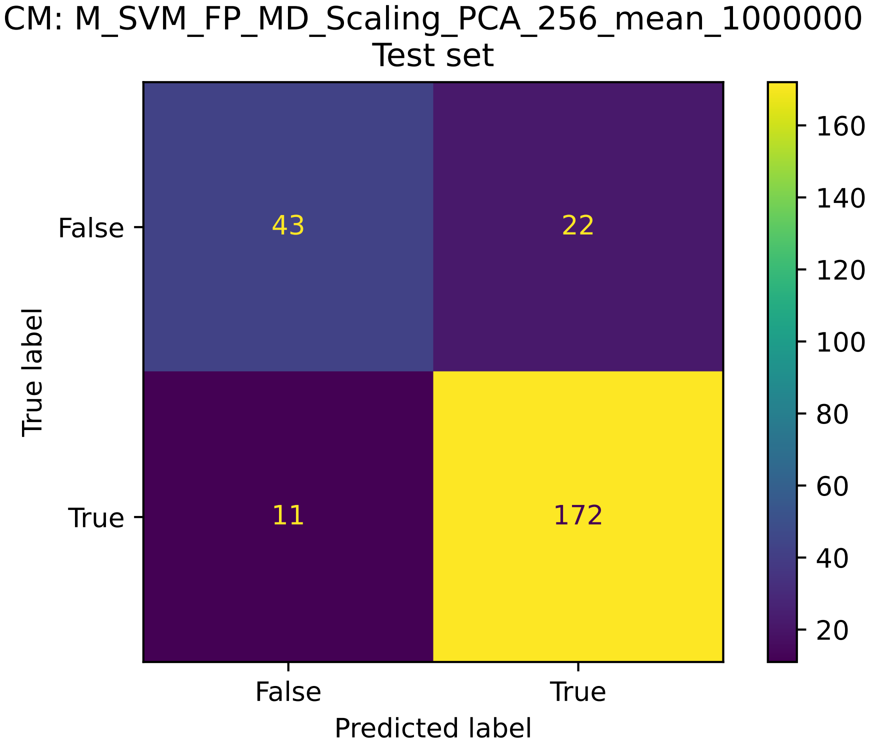
CM: M_SVM_FP_MD_Scaling_PCA_256_mean_1000000
Test set
True label
Predicted label
False
True
False
True
43
22
11
172
20
40
60
80
100
120
140
160

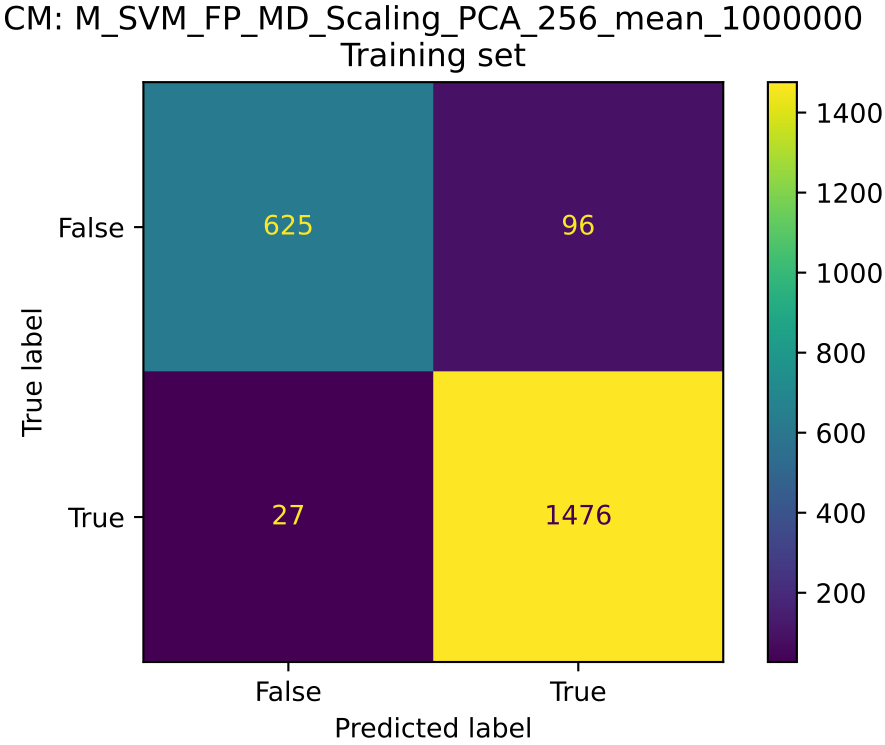
CM: M_SVM_FP_MD_Scaling_PCA_256_mean_1000000
Training set
625
96
27
1476
False
True
True label
False
True
Predicted label
1400
1200
1000
800
600
400
200

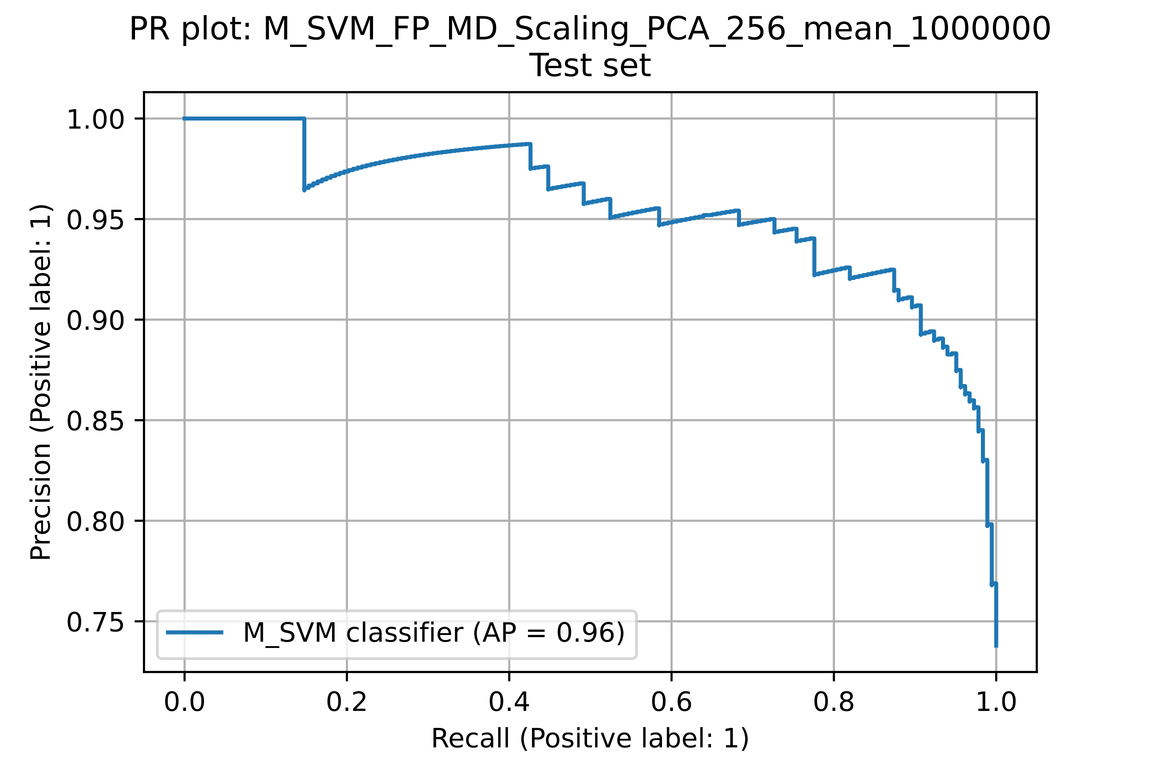
PR plot: M_SVM_FP_MD_Scaling_PCA_256_mean_1000000
Test set
Precision (Positive label: 1)
Recall (Positive label: 1)
1.00
0.95
0.90
0.85
0.80
0.75
0.0
0.2
0.4
0.6
0.8
1.0
M_SVM classifier (AP = 0.96)

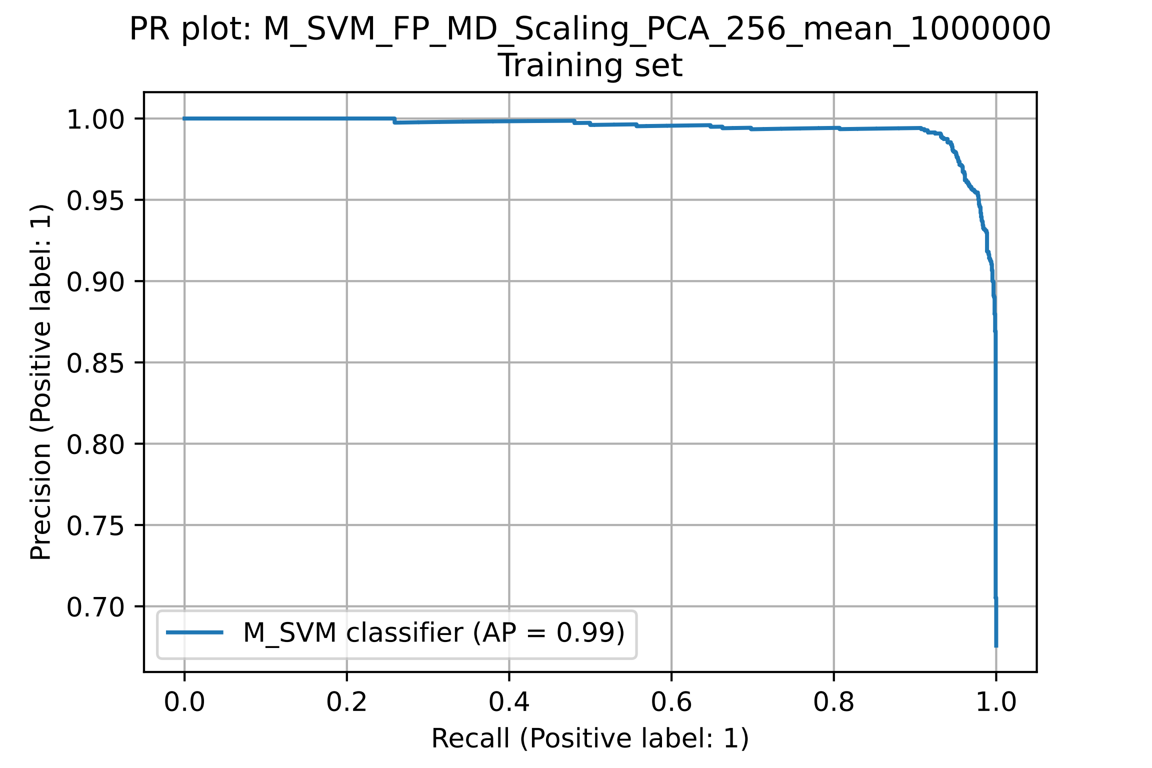
PR plot: M_SVM_FP_MD_Scaling_PCA_256_mean_1000000
Training set
1.00
0.95
0.90
0.85
0.80
0.75
0.70
Precision (Positive label: 1)
0.0
0.2
0.4
0.6
0.8
1.0
Recall (Positive label: 1)
M_SVM classifier (AP = 0.99)

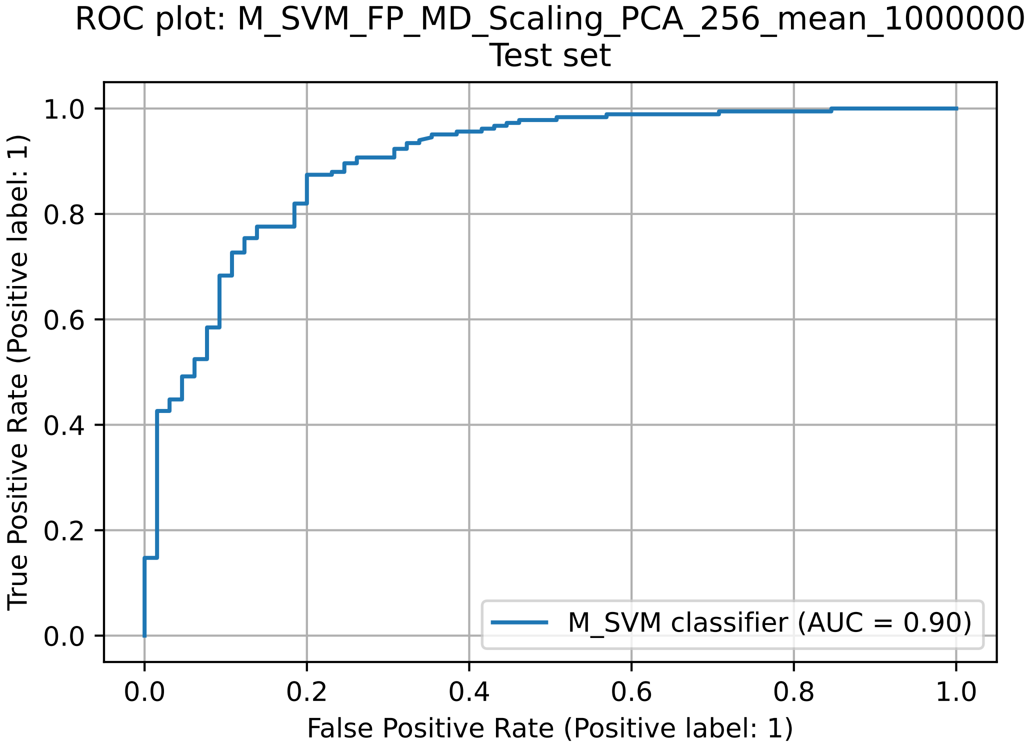
ROC plot: M_SVM_FP_MD_Scaling_PCA_256_mean_1000000
Test set
True Positive Rate (Positive label: 1)
False Positive Rate (Positive label: 1)
0.0
0.2
0.4
0.6
0.8
1.0
M_SVM classifier (AUC = 0.90)

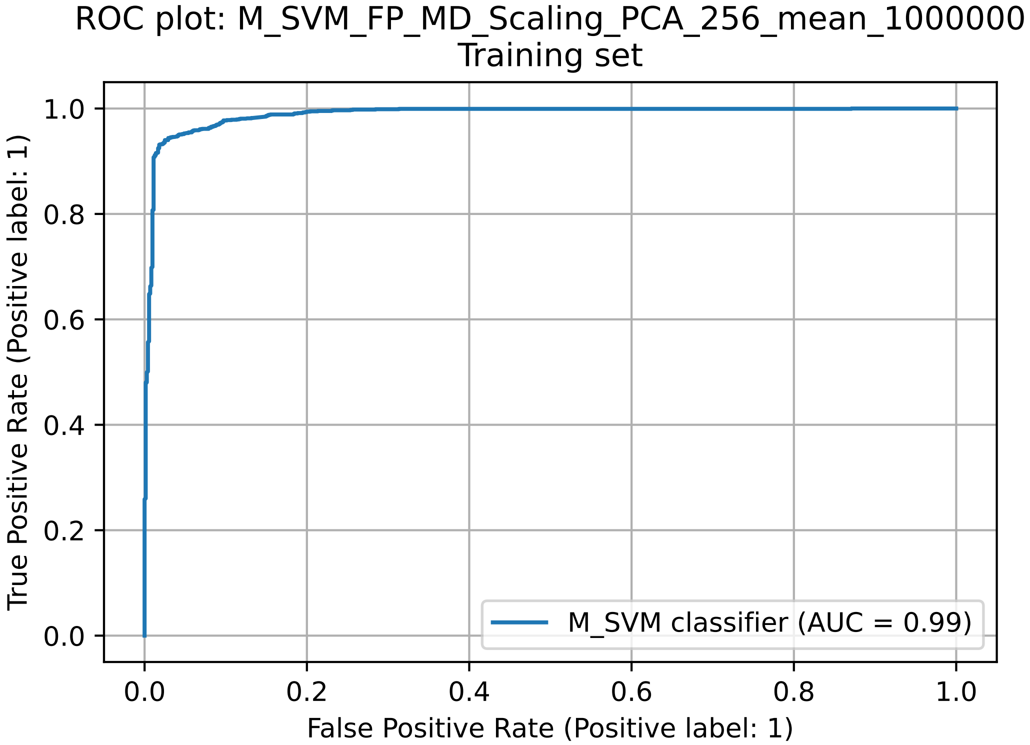
ROC plot: M_SVM_FP_MD_Scaling_PCA_256_mean_1000000
Training set
True Positive Rate (Positive label: 1)
False Positive Rate (Positive label: 1)
0.0
0.2
0.4
0.6
0.8
1.0
M_SVM classifier (AUC = 0.99)

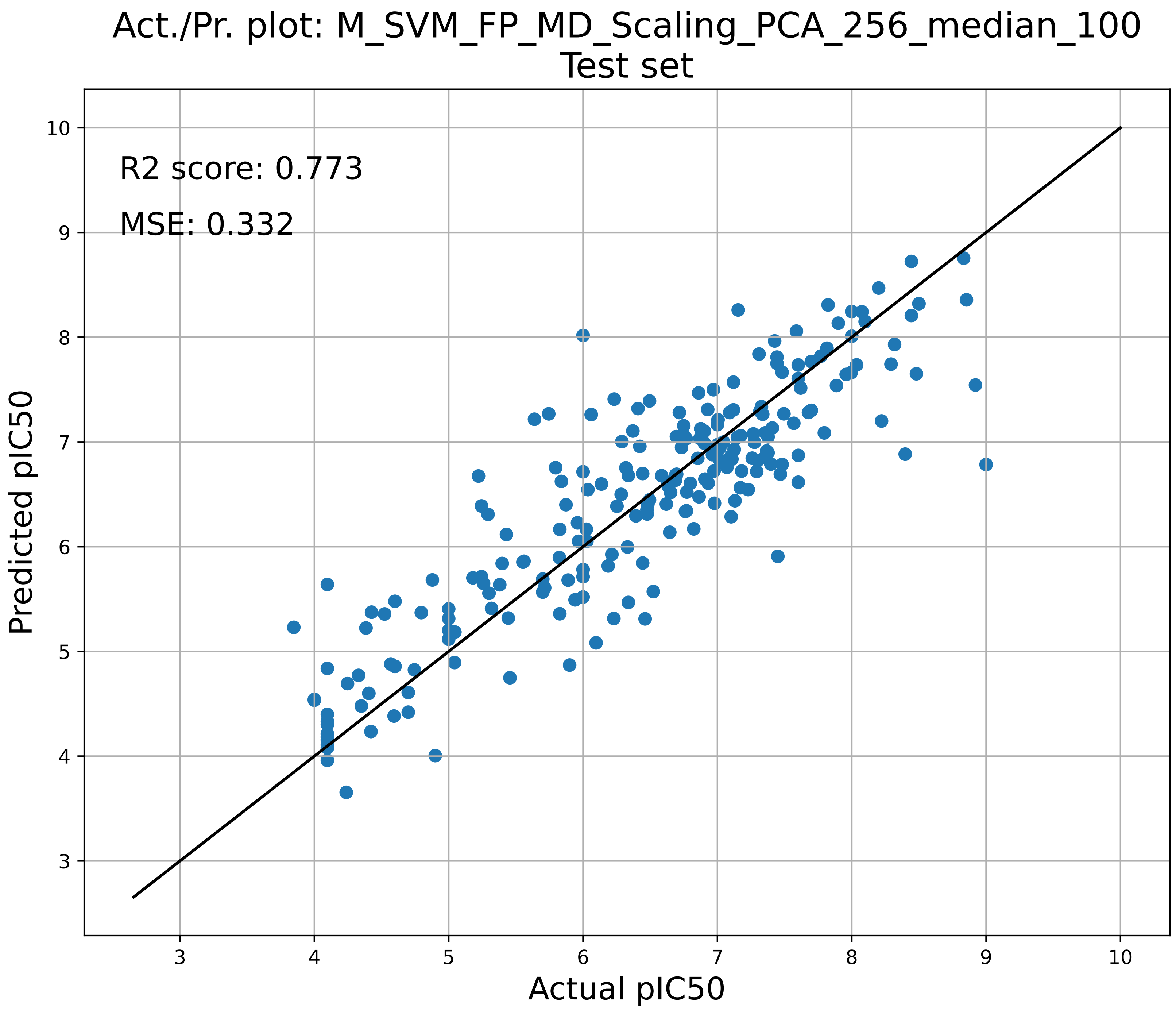

Act./Pr. plot: M_SVM_FP_MD_Scaling_PCA_256_median_100
Test set
R2 score: 0.773
MSE: 0.332
Predicted pIC50
Actual pIC50
3
4
5
6
7
8
9
10

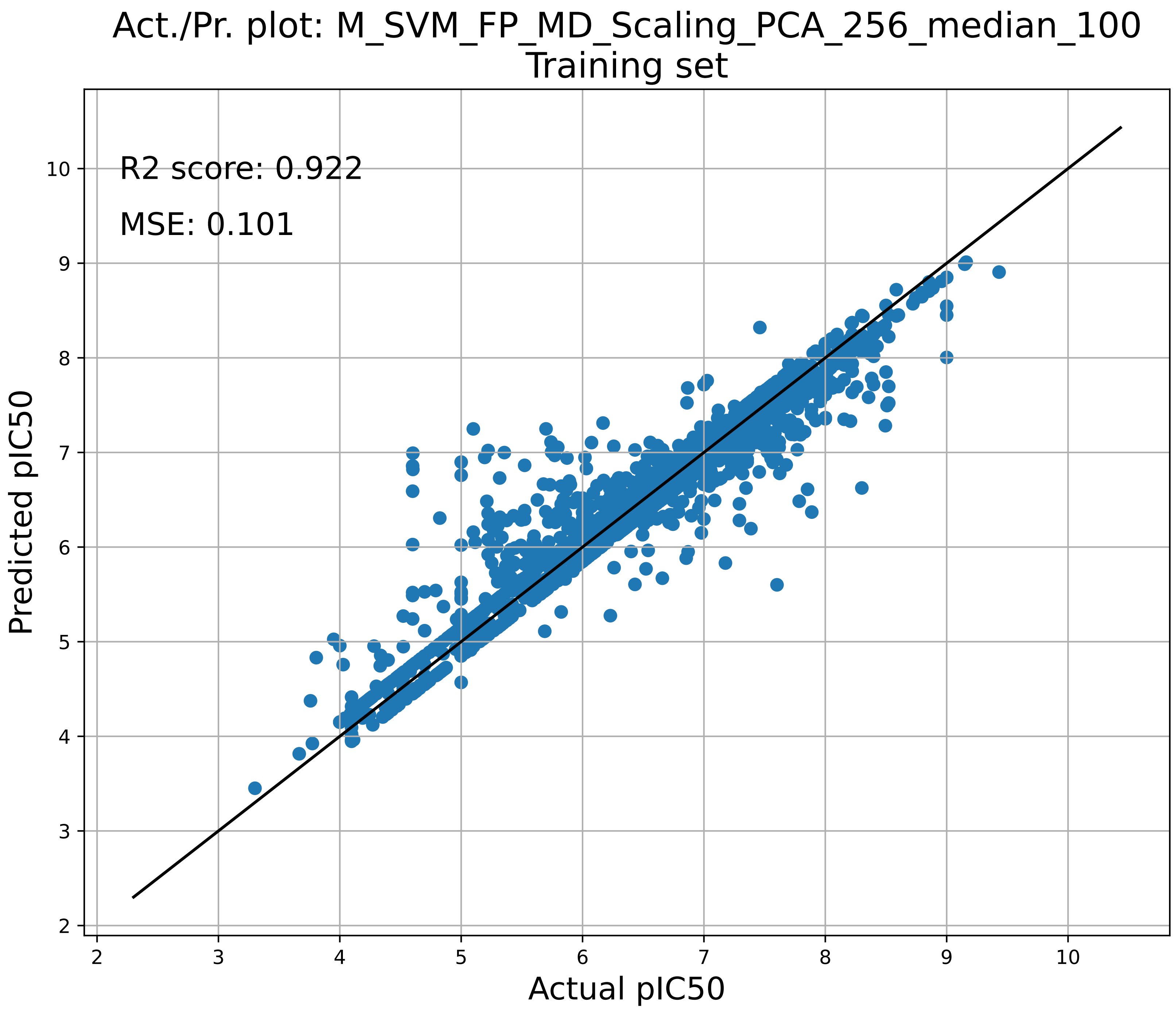

Act./Pr. plot: M_SVM_FP_MD_Scaling_PCA_256_median_100
Training set
R2 score: 0.922
MSE: 0.101
Predicted pIC50
Actual pIC50
2
3
4
5
6
7
8
9
10

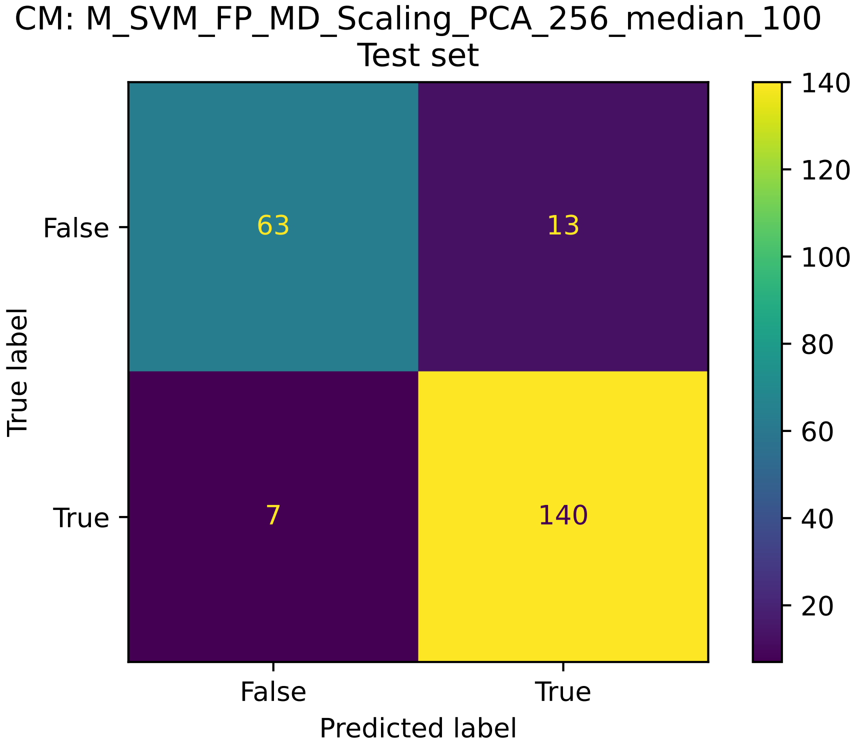
CM: M_SVM_FP_MD_Scaling_PCA_256_median_100
Test set
True label
Predicted label
False
True
False
True
63
13
7
140
140
120
100
80
60
40
20

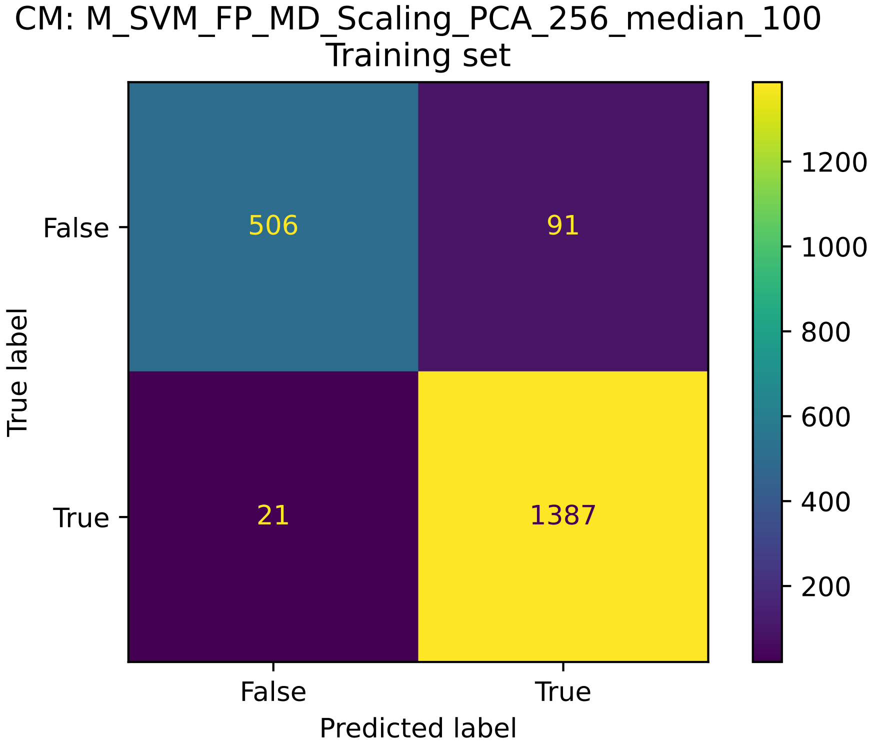
CM: M_SVM_FP_MD_Scaling_PCA_256_median_100
Training set
False
True
506
91
21
1387
True label
Predicted label
200
400
600
800
1000
1200

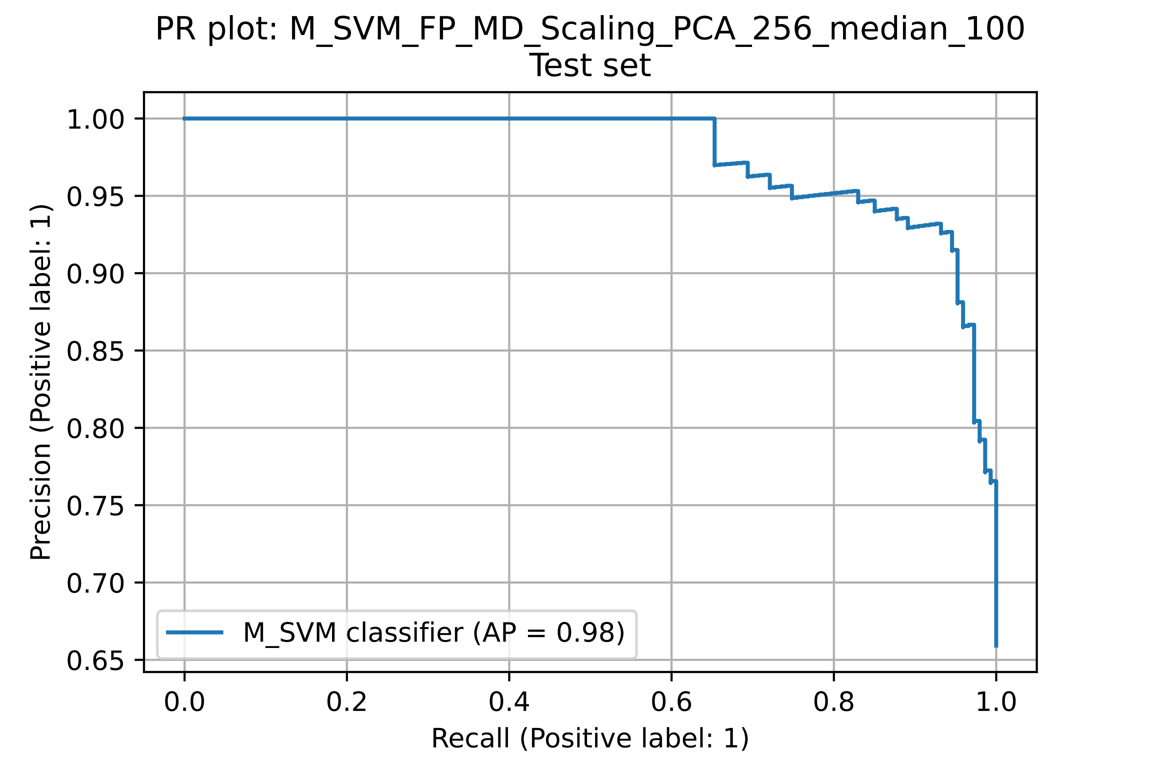
PR plot: M_SVM_FP_MD_Scaling_PCA_256_median_100
Test set
Precision (Positive label: 1)
Recall (Positive label: 1)
1.00
0.95
0.90
0.85
0.80
0.75
0.70
0.65
0.0
0.2
0.4
0.6
0.8
1.0
M_SVM classifier (AP = 0.98)

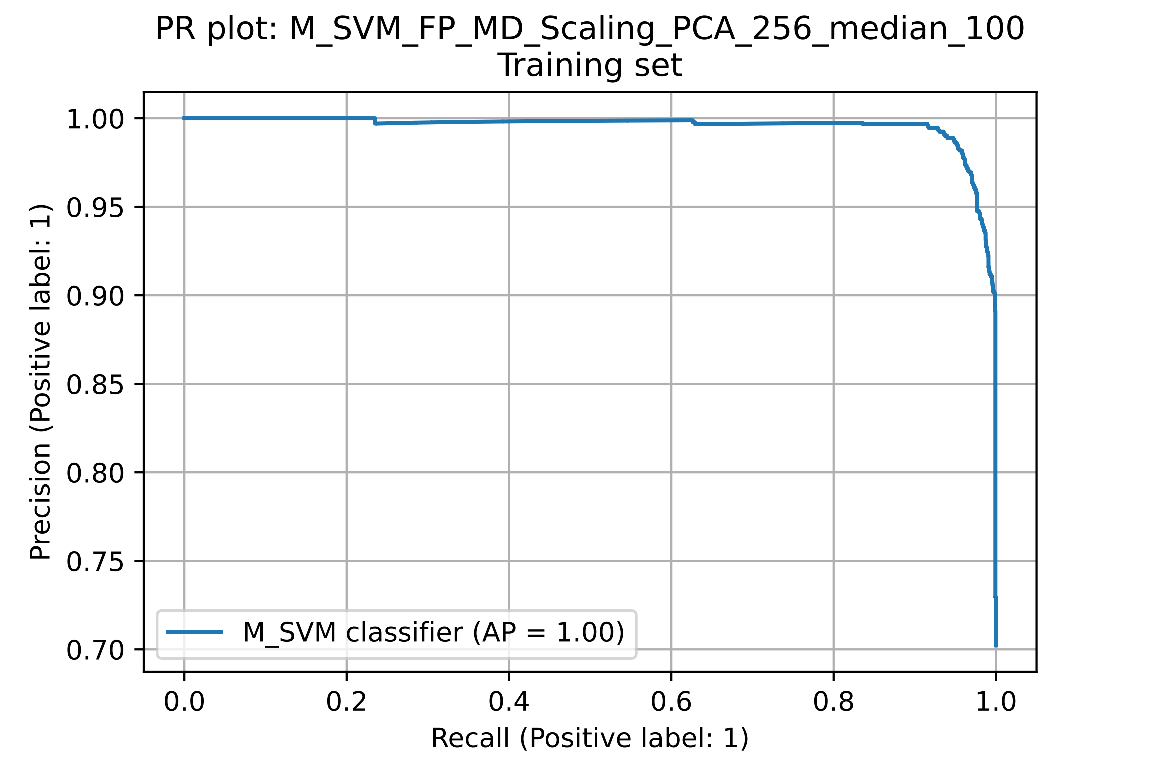
PR plot: M_SVM_FP_MD_Scaling_PCA_256_median_100
Training set
Precision (Positive label: 1)
Recall (Positive label: 1)
M_SVM classifier (AP = 1.00)
1.00
0.95
0.90
0.85
0.80
0.75
0.70
0.0
0.2
0.4
0.6
0.8
1.0

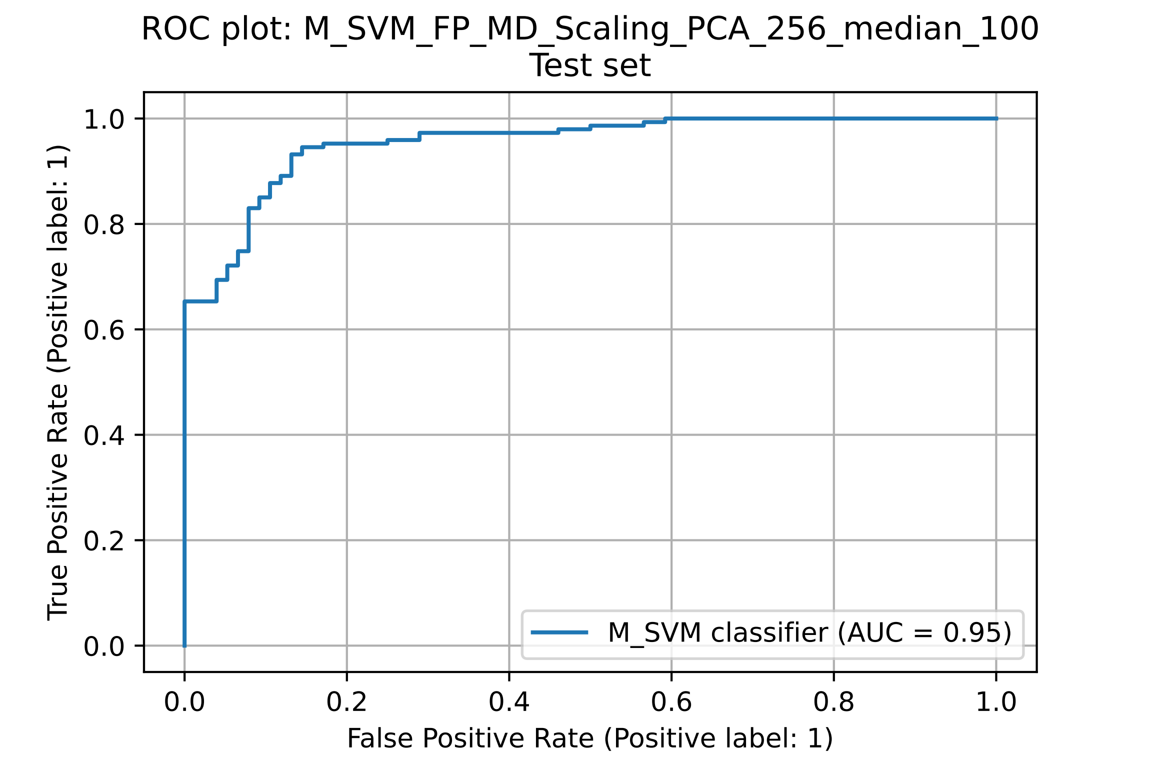

ROC plot: M_SVM_FP_MD_Scaling_PCA_256_median_100
Test set
True Positive Rate (Positive label: 1)
False Positive Rate (Positive label: 1)
0.0
0.2
0.4
0.6
0.8
1.0
M_SVM classifier (AUC = 0.95)

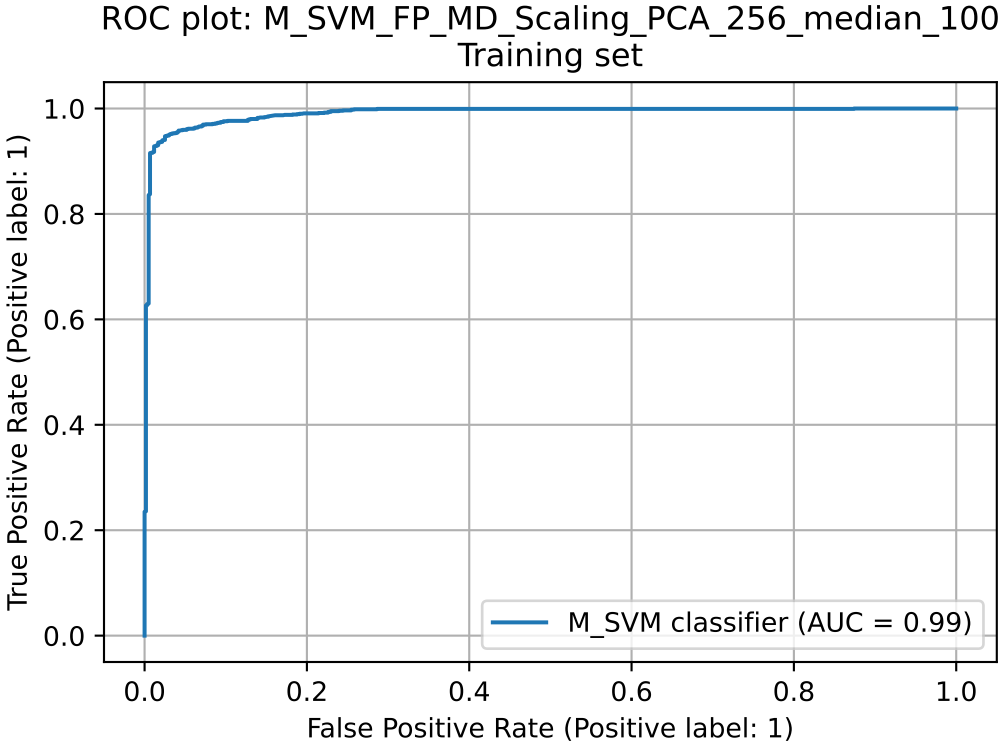

ROC plot: M_SVM_FP_MD_Scaling_PCA_256_median_100
Training set
True Positive Rate (Positive label: 1)
False Positive Rate (Positive label: 1)
0.0
0.2
0.4
0.6
0.8
1.0
M_SVM classifier (AUC = 0.99)

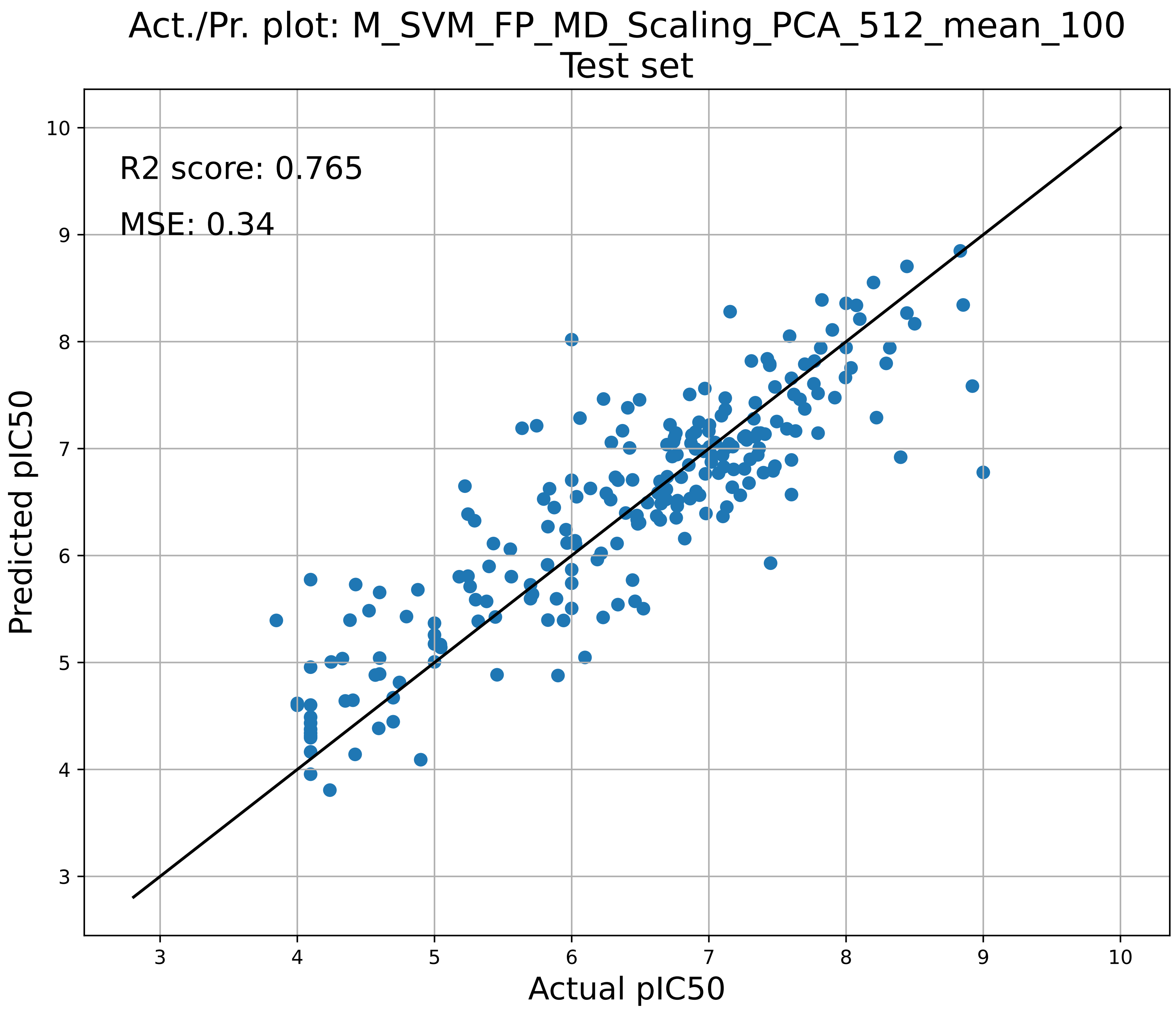
Act./Pr. plot: M_SVM_FP_MD_Scaling_PCA_512_mean_100
Test set
R2 score: 0.765
MSE: 0.34
Predicted pIC50
Actual pIC50
3
4
5
6
7
8
9
10

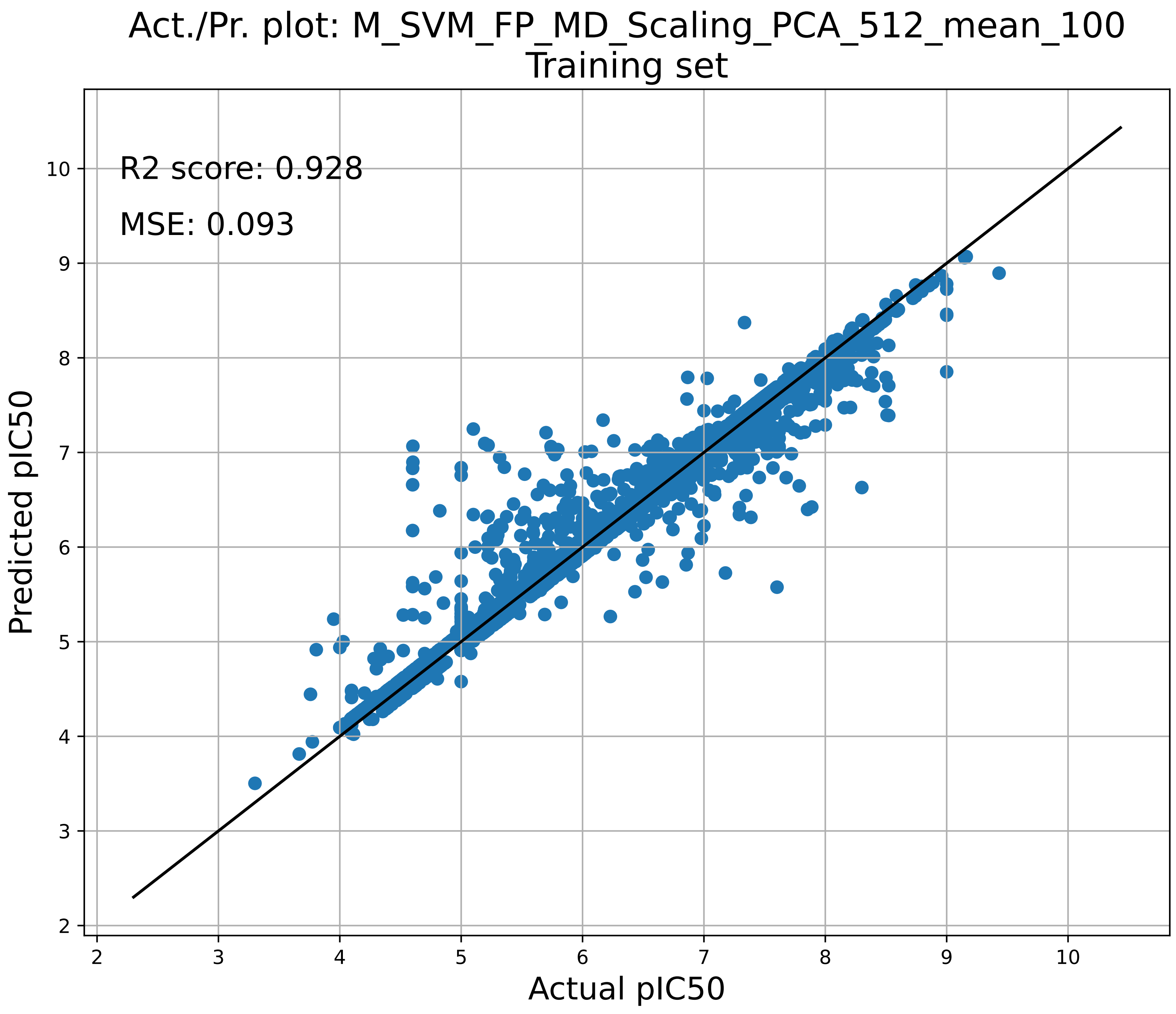

Act./Pr. plot: M_SVM_FP_MD_Scaling_PCA_512_mean_100
Training set
R2 score: 0.928
MSE: 0.093
Predicted pIC50
Actual pIC50
2
3
4
5
6
7
8
9
10

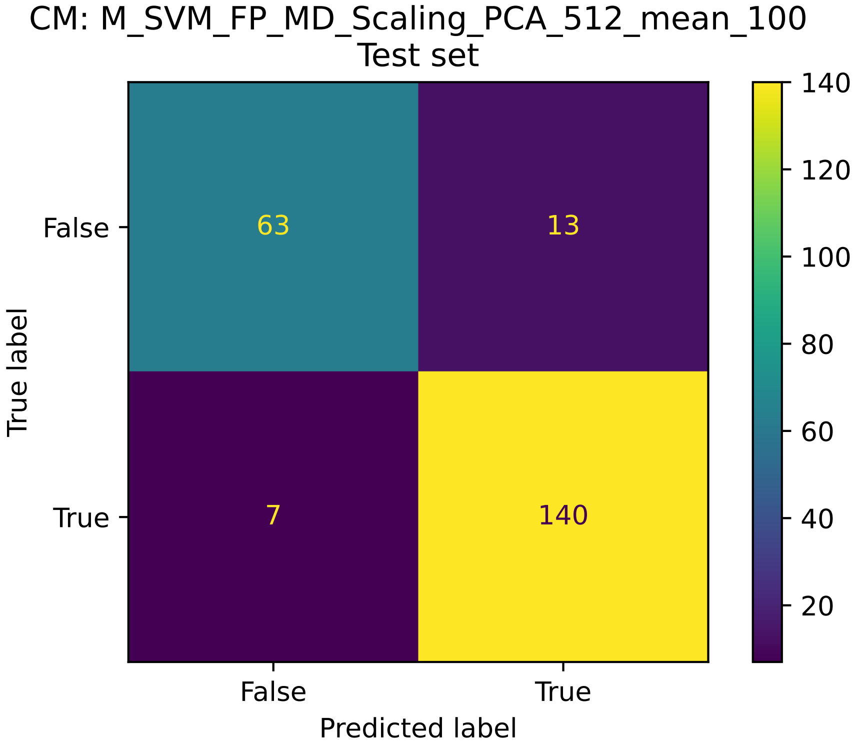
CM: M_SVM_FP_MD_Scaling_PCA_512_mean_100
Test set
63
13
7
140
False
True
True label
False
True
Predicted label
140
120
100
80
60
40
20

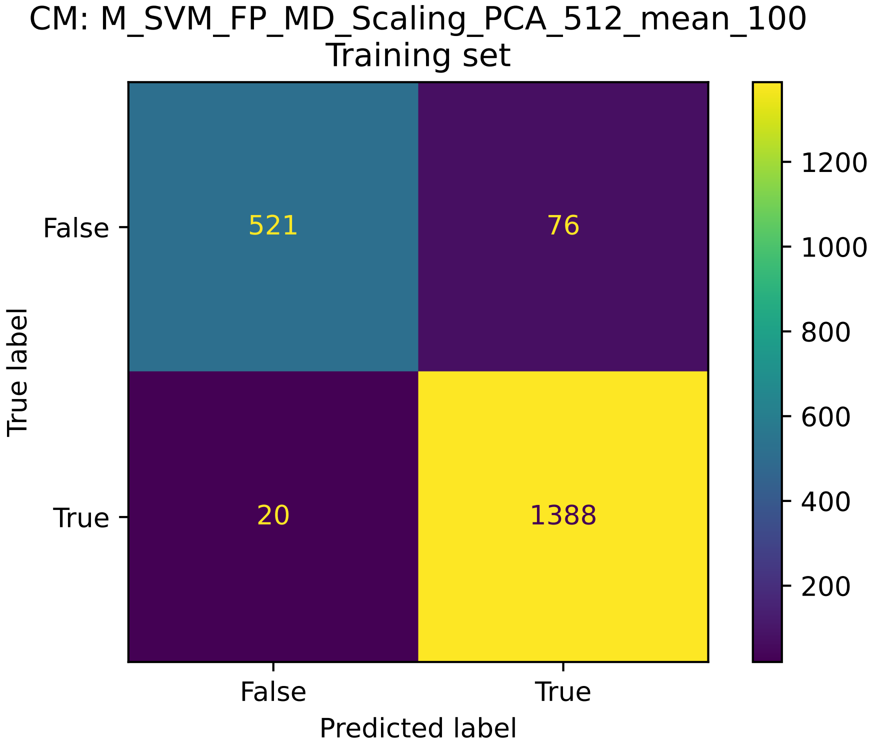
CM: M_SVM_FP_MD_Scaling_PCA_512_mean_100
Training set
True label
Predicted label
False
True
False
True
521
76
20
1388
1200
1000
800
600
400
200

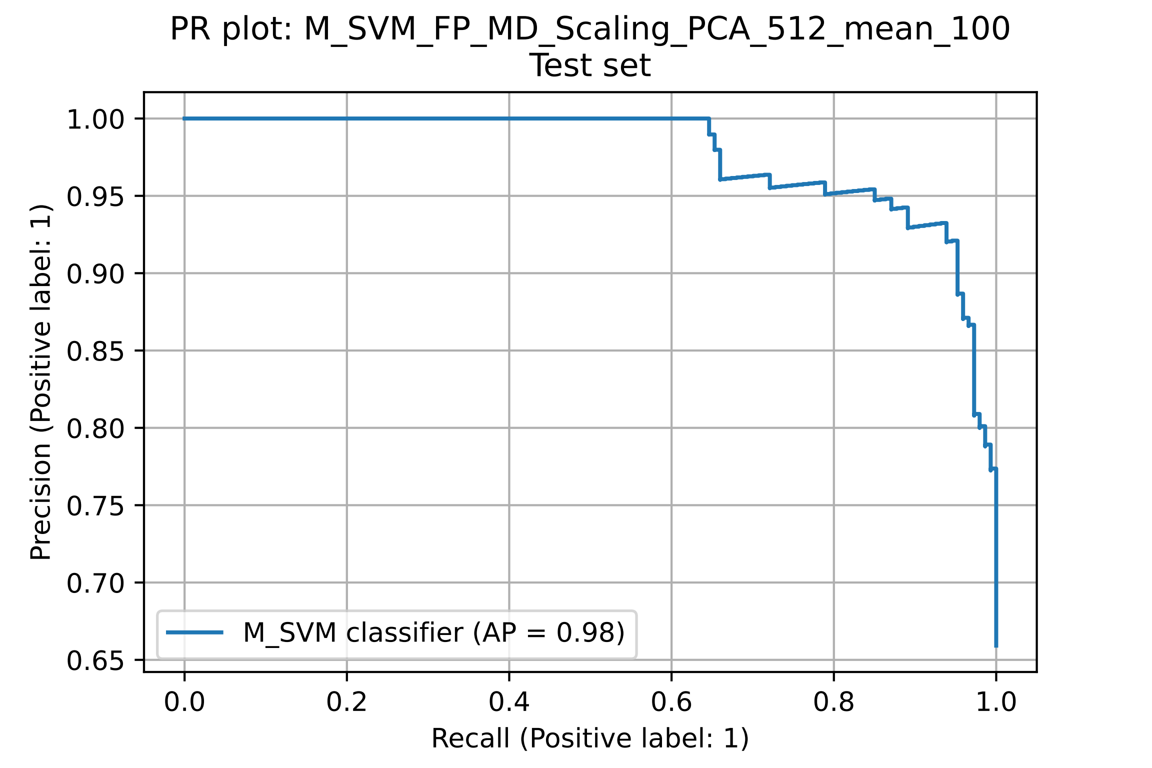
PR plot: M_SVM_FP_MD_Scaling_PCA_512_mean_100
Test set
Precision (Positive label: 1)
Recall (Positive label: 1)
1.00
0.95
0.90
0.85
0.80
0.75
0.70
0.65
0.0
0.2
0.4
0.6
0.8
1.0
M_SVM classifier (AP = 0.98)

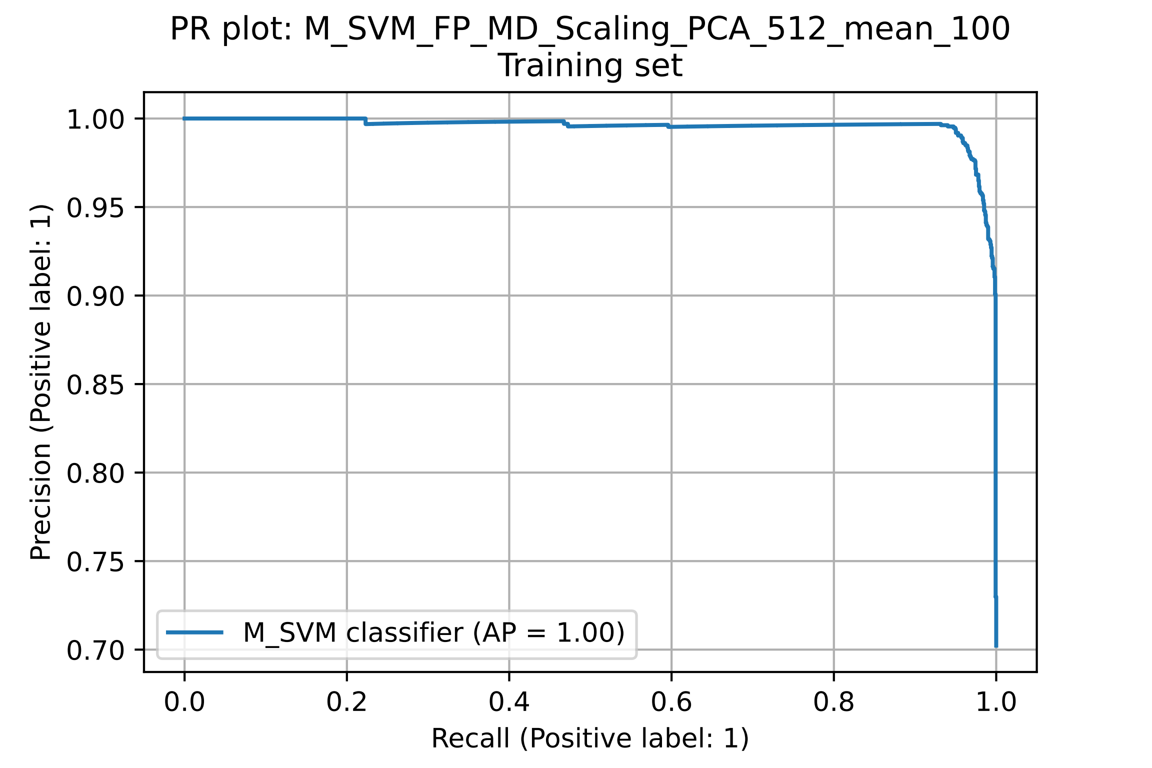
PR plot: M_SVM_FP_MD_Scaling_PCA_512_mean_100
Training set
Precision (Positive label: 1)
1.00
0.95
0.90
0.85
0.80
0.75
0.70
Recall (Positive label: 1)
0.0
0.2
0.4
0.6
0.8
1.0
M_SVM classifier (AP = 1.00)

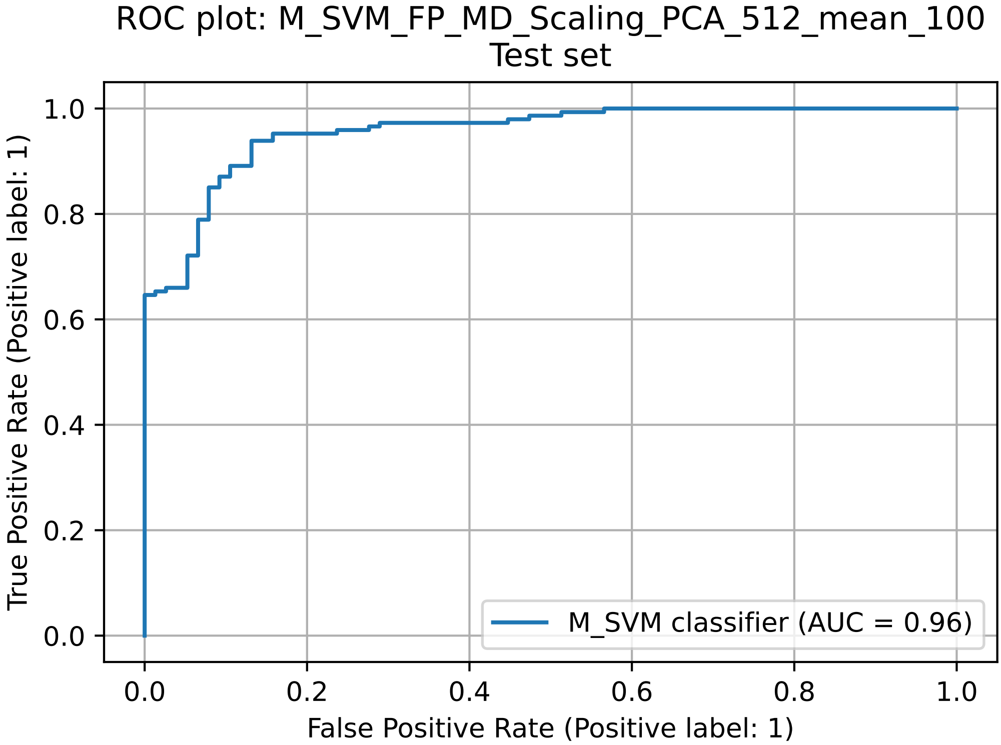

ROC plot: M_SVM_FP_MD_Scaling_PCA_512_mean_100
Test set
True Positive Rate (Positive label: 1)
False Positive Rate (Positive label: 1)
0.0
0.2
0.4
0.6
0.8
1.0
M_SVM classifier (AUC = 0.96)

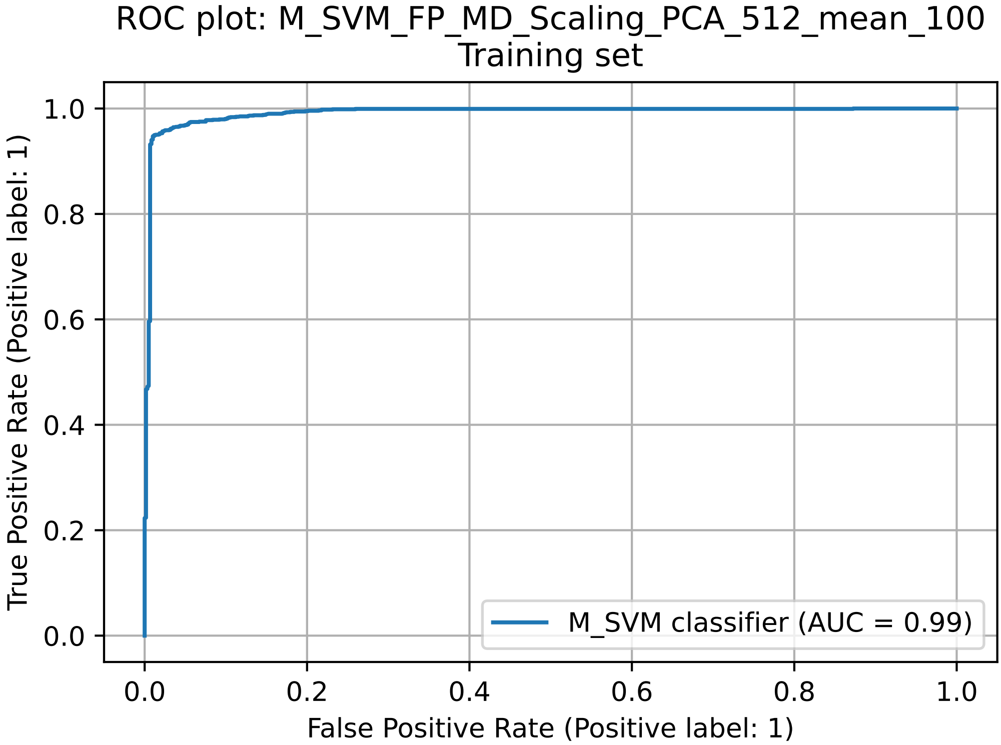
ROC plot: M_SVM_FP_MD_Scaling_PCA_512_mean_100
Training set
True Positive Rate (Positive label: 1)
False Positive Rate (Positive label: 1)
0.0
0.2
0.4
0.6
0.8
1.0
M_SVM classifier (AUC = 0.99)

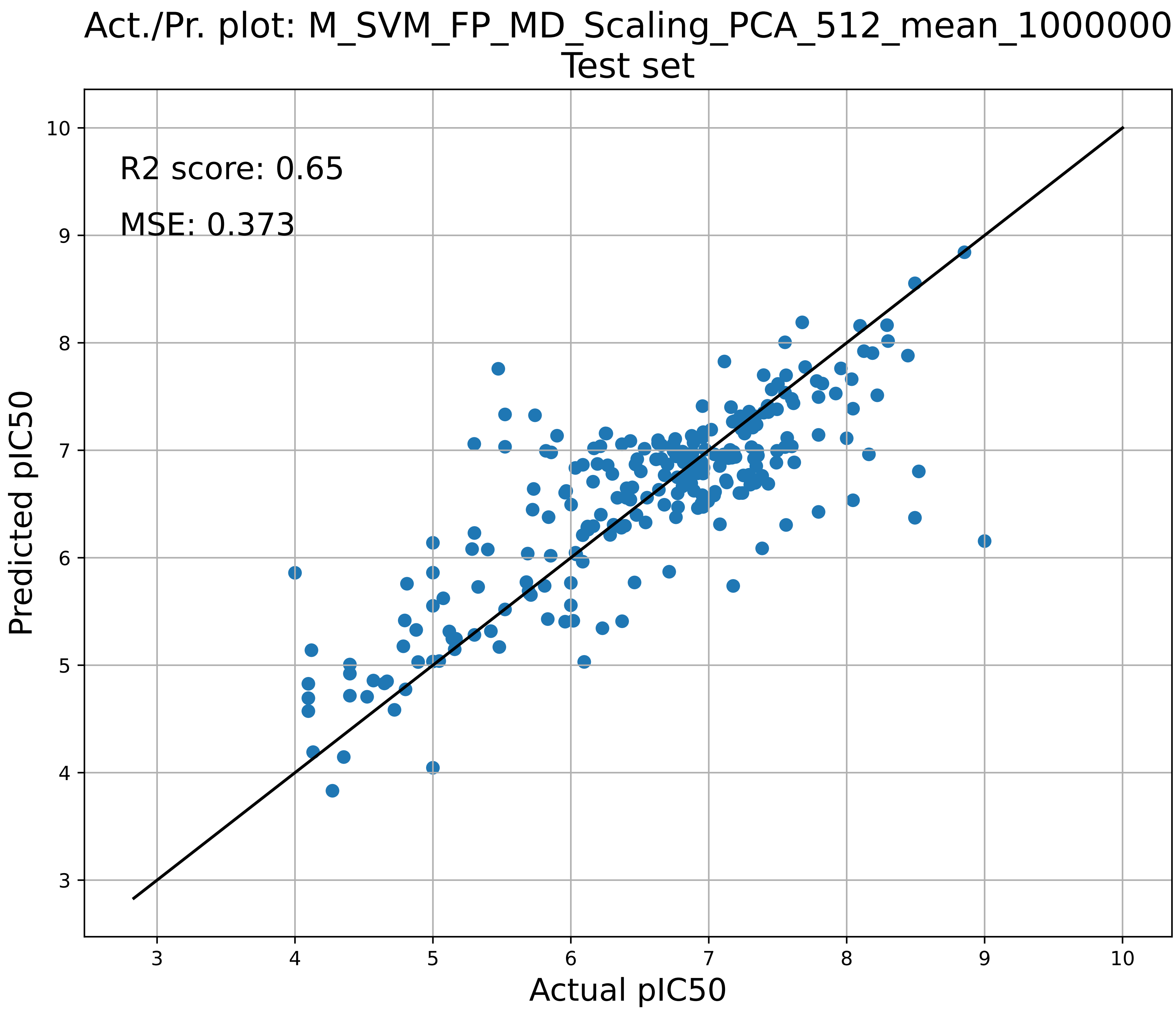

Act./Pr. plot: M_SVM_FP_MD_Scaling_PCA_512_mean_1000000
Test set
R2 score: 0.65
MSE: 0.373
Predicted pIC50
Actual pIC50
3
4
5
6
7
8
9
10

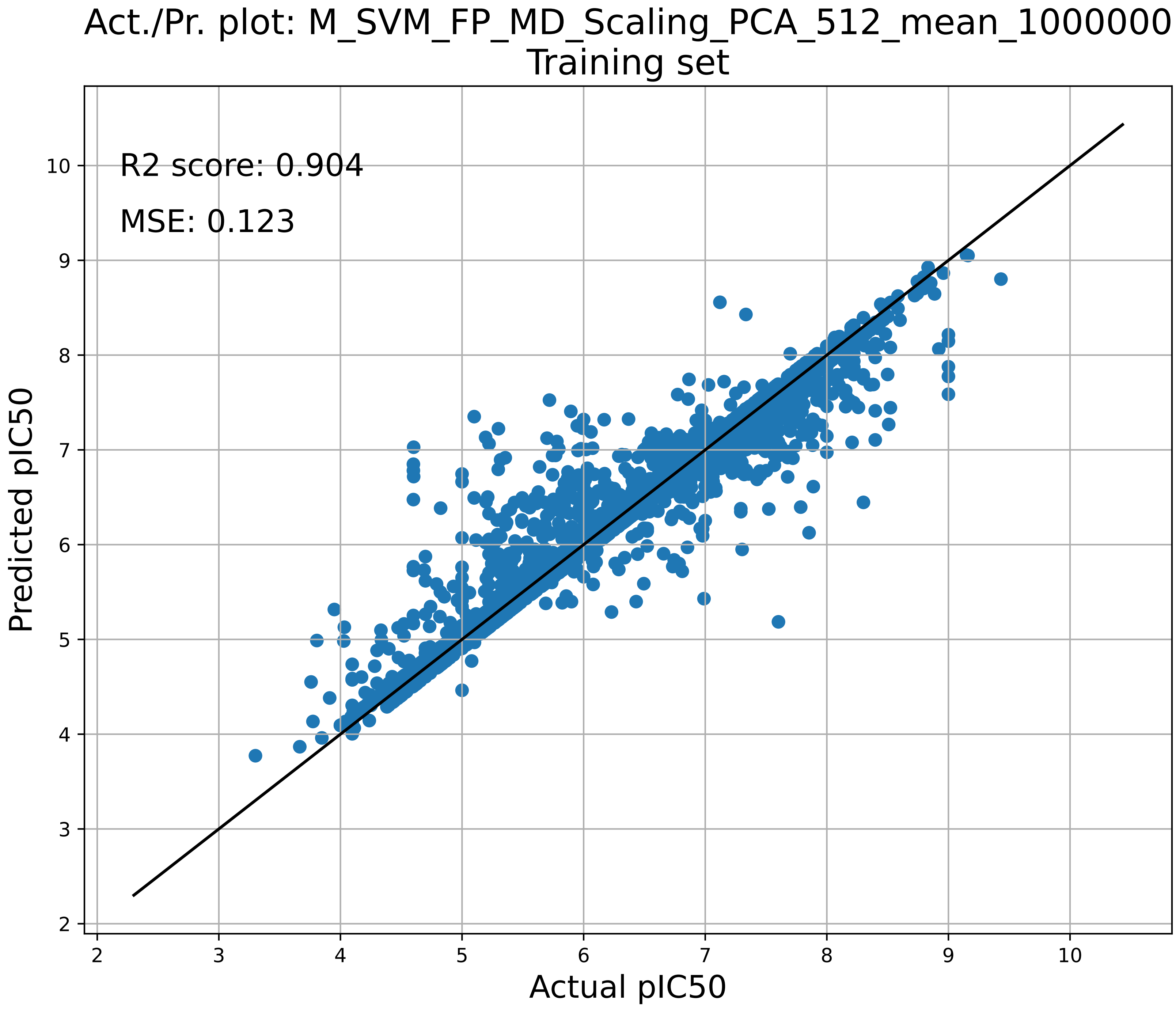
Act./Pr. plot: M_SVM_FP_MD_Scaling_PCA_512_mean_1000000
Training set
R2 score: 0.904
MSE: 0.123
Predicted pIC50
Actual pIC50
2
3
4
5
6
7
8
9
10

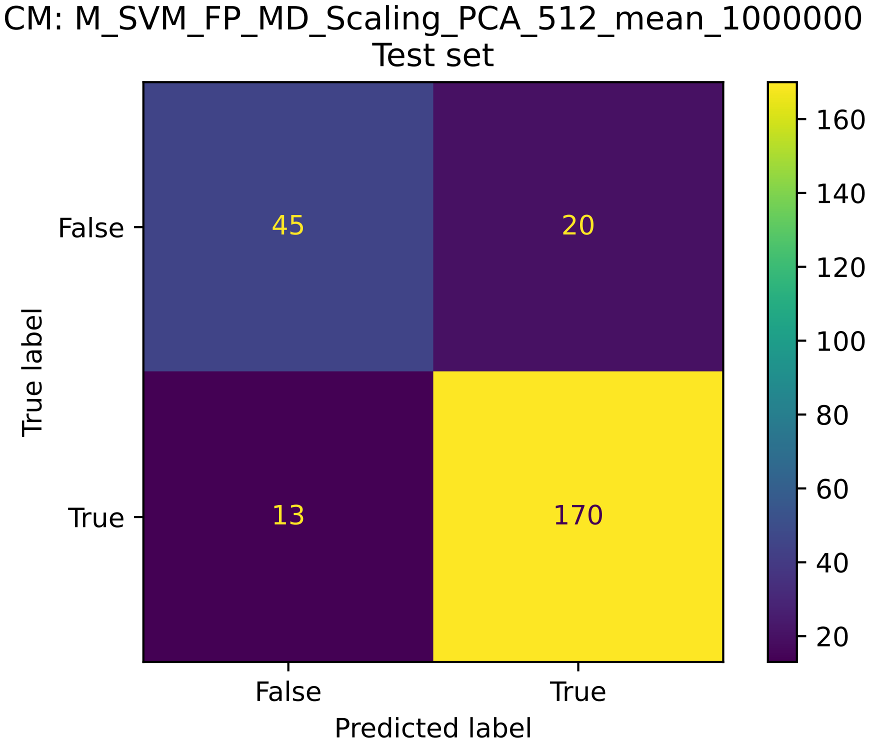
CM: M_SVM_FP_MD_Scaling_PCA_512_mean_1000000
Test set
True label
Predicted label
False
True
False
True
45
20
13
170
160
140
120
100
80
60
40
20

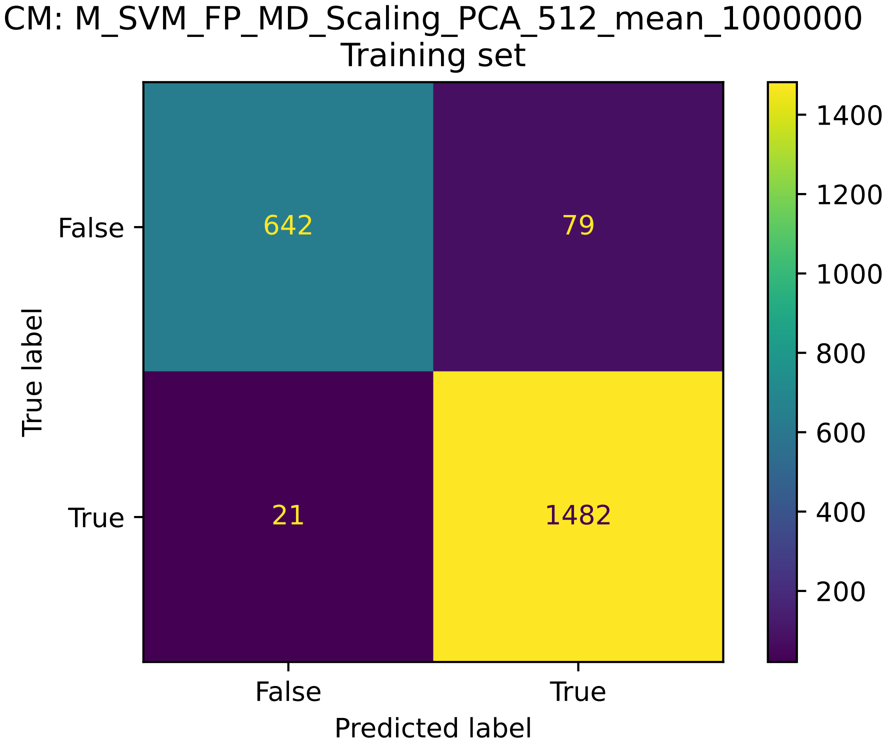
CM: M_SVM_FP_MD_Scaling_PCA_512_mean_1000000
Training set
642
79
21
1482
False
True
True label
False
True
Predicted label
1400
1200
1000
800
600
400
200

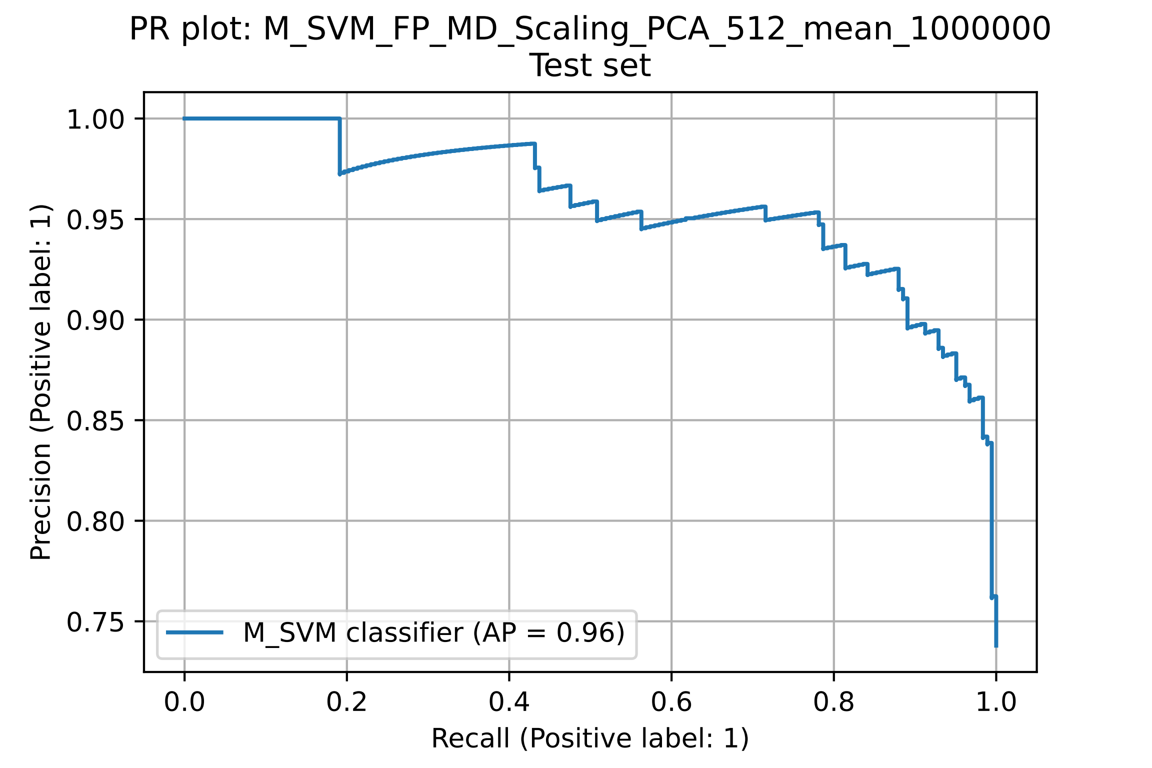

PR plot: M_SVM_FP_MD_Scaling_PCA_512_mean_1000000
Test set
Precision (Positive label: 1)
Recall (Positive label: 1)
1.00
0.95
0.90
0.85
0.80
0.75
0.0
0.2
0.4
0.6
0.8
1.0
M_SVM classifier (AP = 0.96)

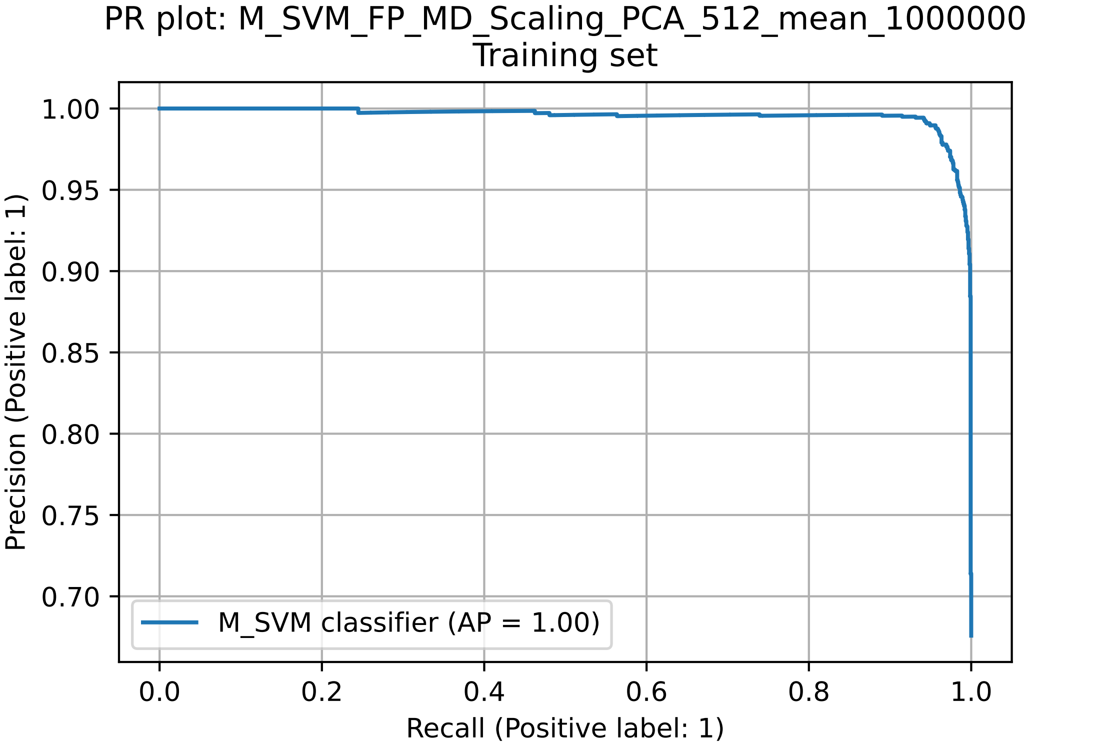

PR plot: M_SVM_FP_MD_Scaling_PCA_512_mean_1000000
Training set
1.00
0.95
0.90
0.85
0.80
0.75
0.70
Precision (Positive label: 1)
0.0
0.2
0.4
0.6
0.8
1.0
Recall (Positive label: 1)
M_SVM classifier (AP = 1.00)

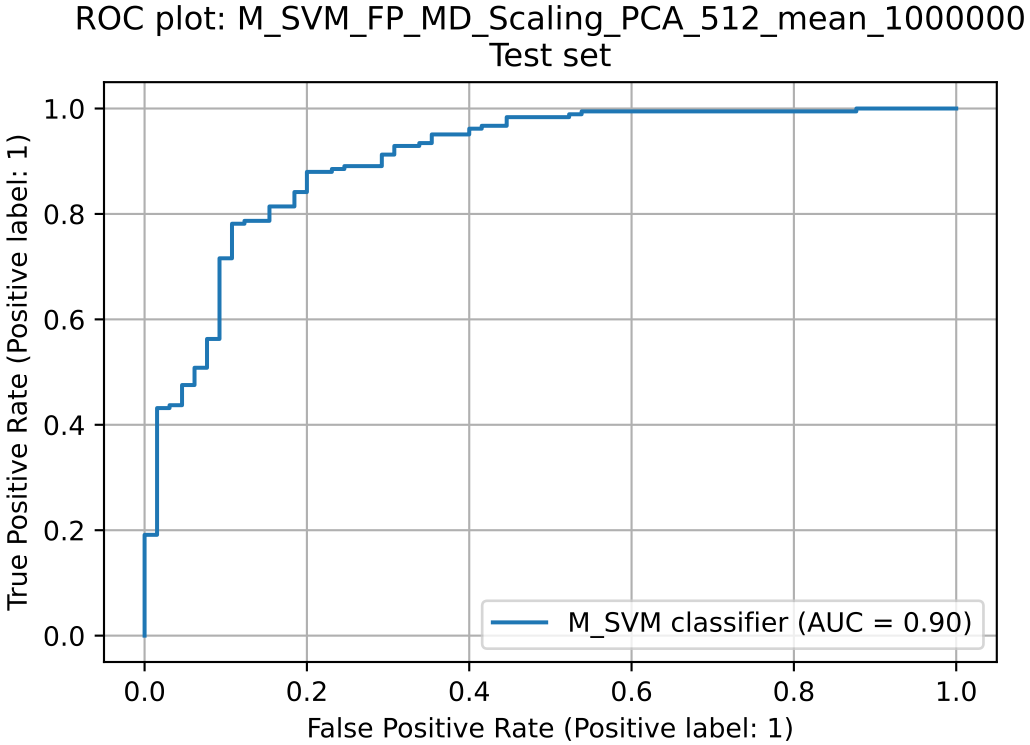
ROC plot: M_SVM_FP_MD_Scaling_PCA_512_mean_1000000
Test set
True Positive Rate (Positive label: 1)
False Positive Rate (Positive label: 1)
0.0
0.2
0.4
0.6
0.8
1.0
M_SVM classifier (AUC = 0.90)

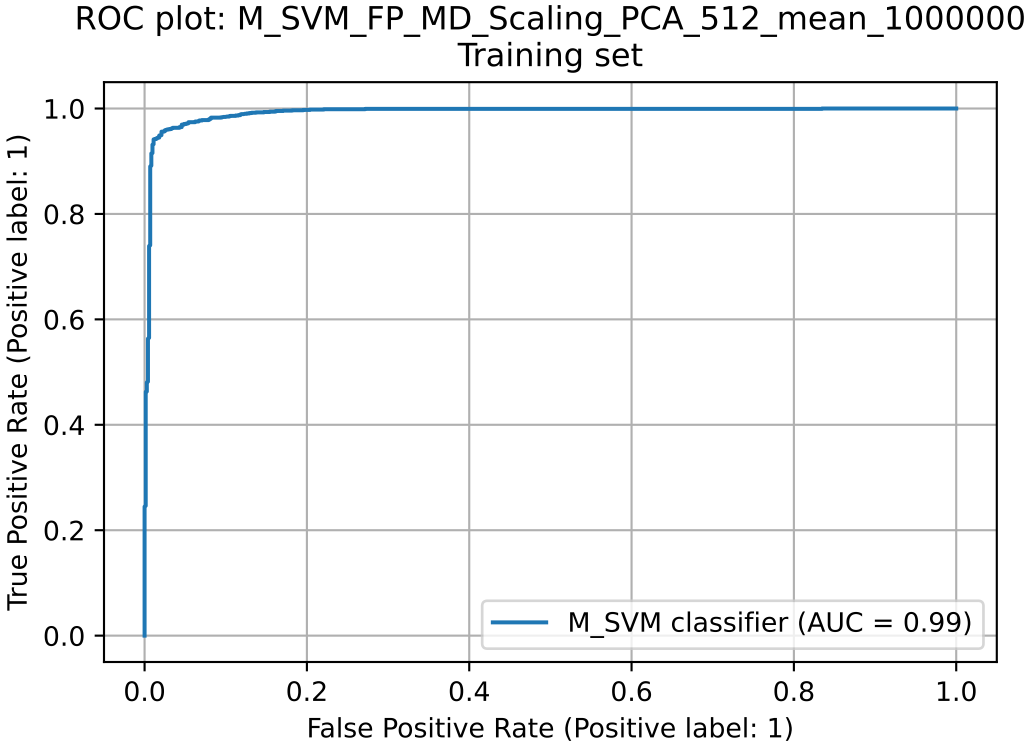
ROC plot: M_SVM_FP_MD_Scaling_PCA_512_mean_1000000
Training set
True Positive Rate (Positive label: 1)
False Positive Rate (Positive label: 1)
0.0
0.2
0.4
0.6
0.8
1.0
M_SVM classifier (AUC = 0.99)

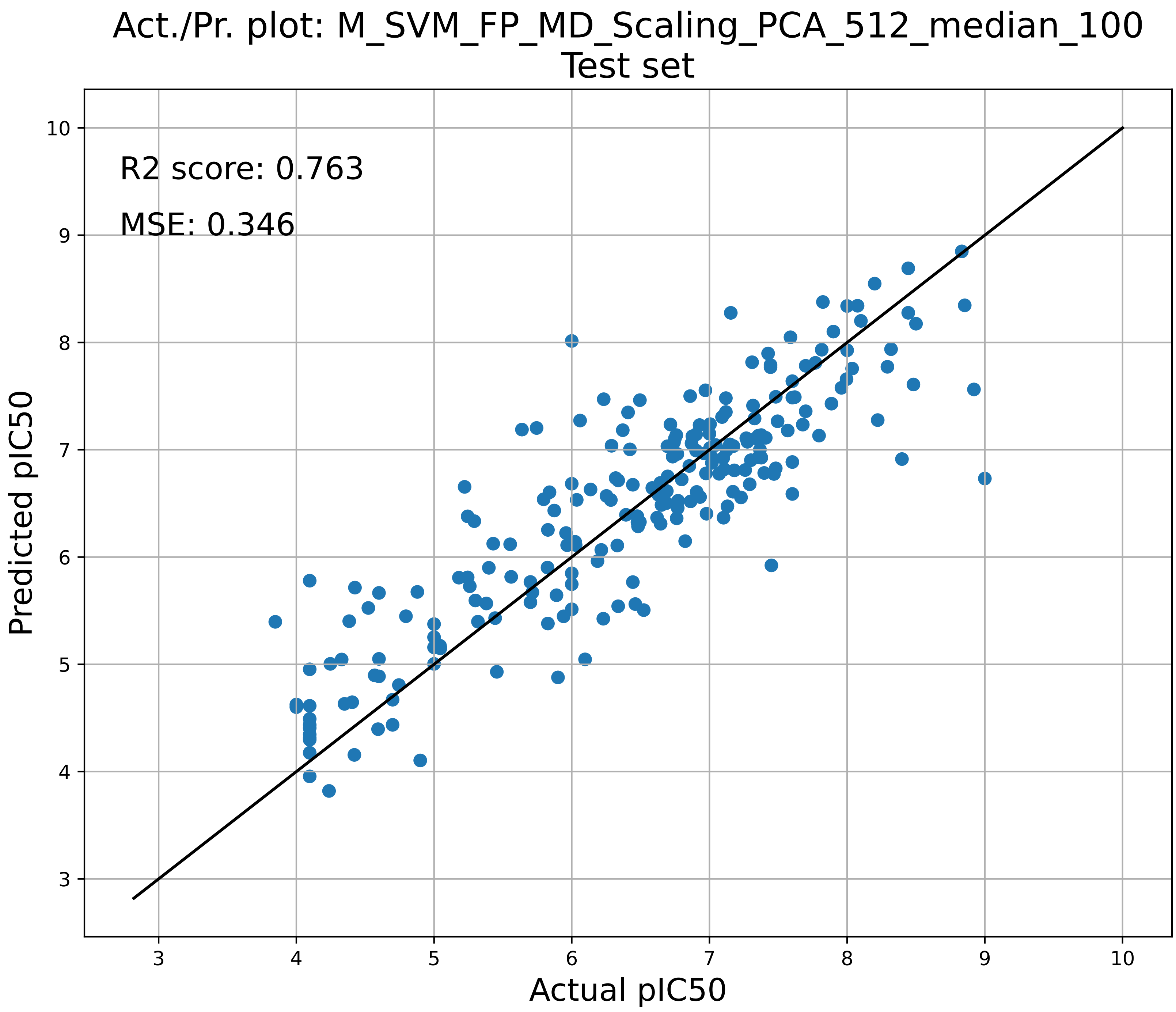

Act./Pr. plot: M_SVM_FP_MD_Scaling_PCA_512_median_100
Test set
R2 score: 0.763
MSE: 0.346
Predicted pIC50
Actual pIC50
3
4
5
6
7
8
9
10

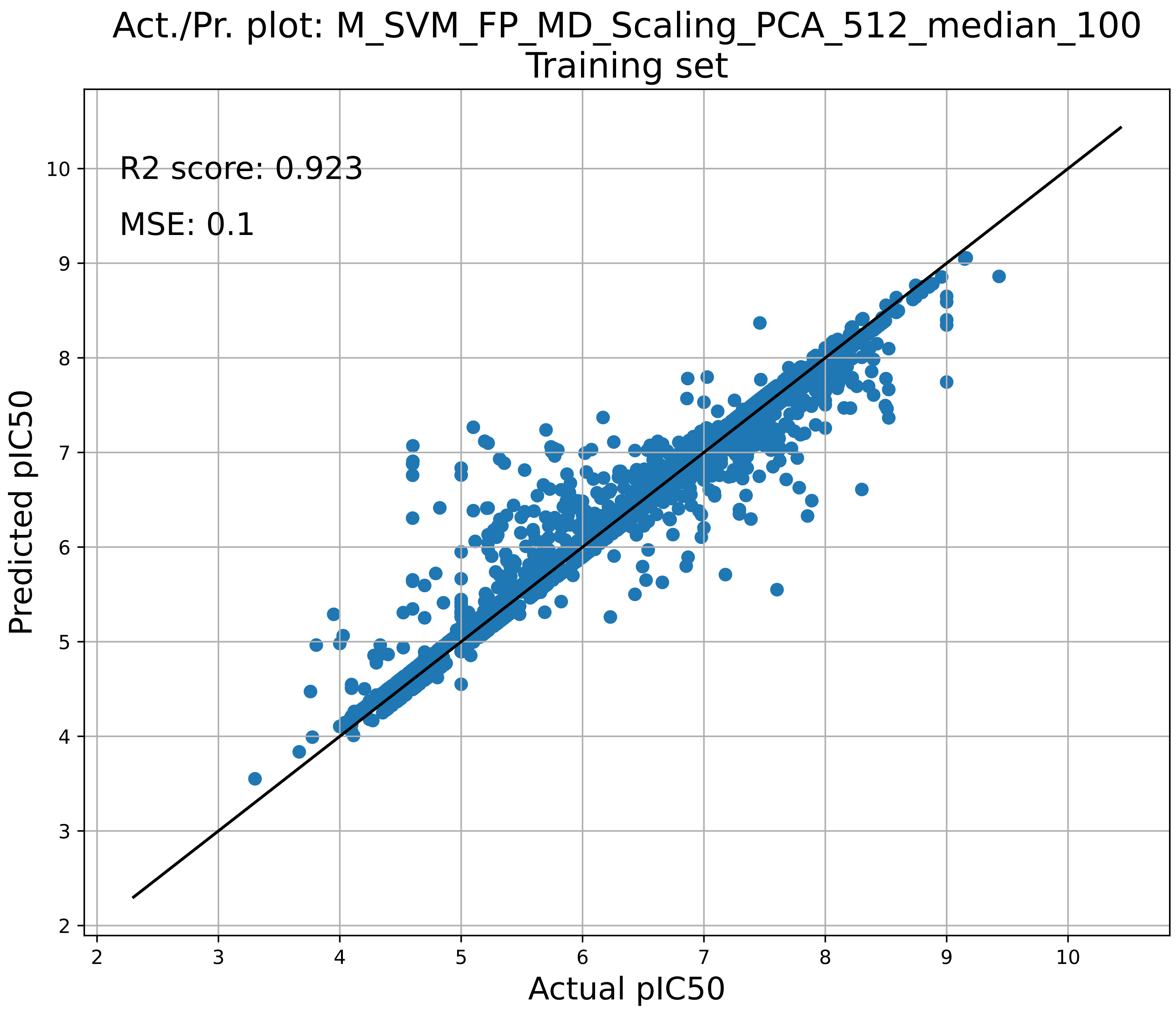

Act./Pr. plot: M_SVM_FP_MD_Scaling_PCA_512_median_100
Training set
R2 score: 0.923
MSE: 0.1
Predicted pIC50
Actual pIC50
2
3
4
5
6
7
8
9
10

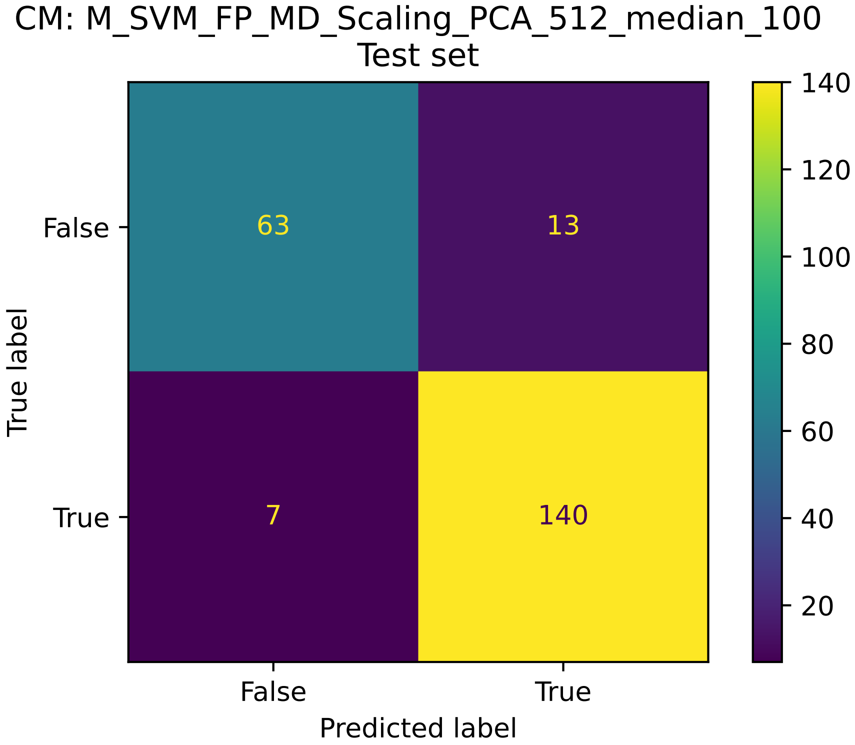

CM: M_SVM_FP_MD_Scaling_PCA_512_median_100
Test set
False
True
63
13
7
140
False
True
Predicted label
True label
140
120
100
80
60
40
20

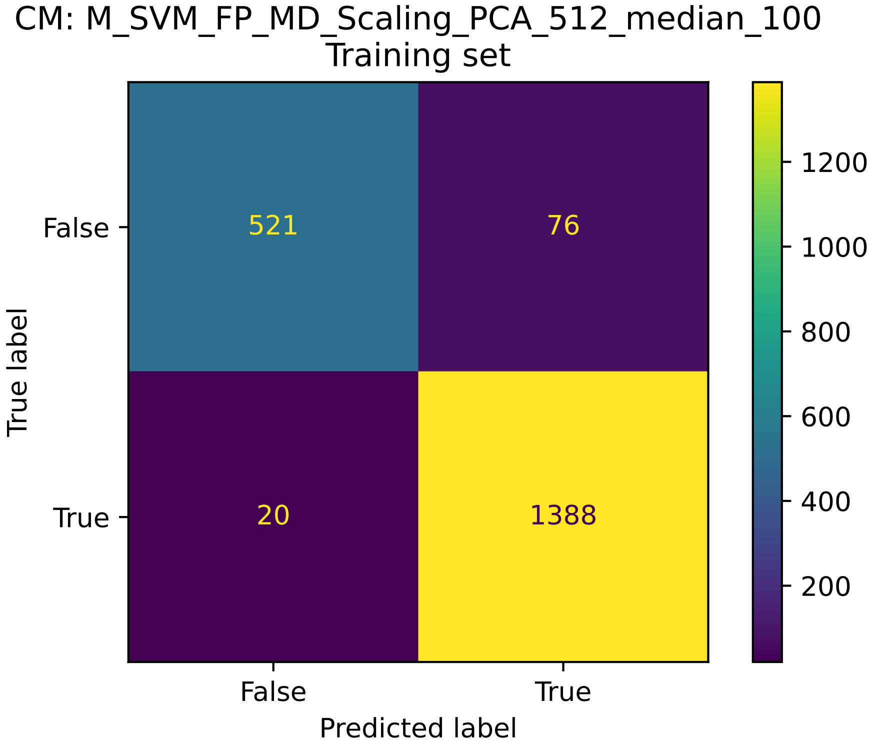
CM: M_SVM_FP_MD_Scaling_PCA_512_median_100
Training set
521
76
20
1388
False
True
True label
False
True
Predicted label
200
400
600
800
1000
1200

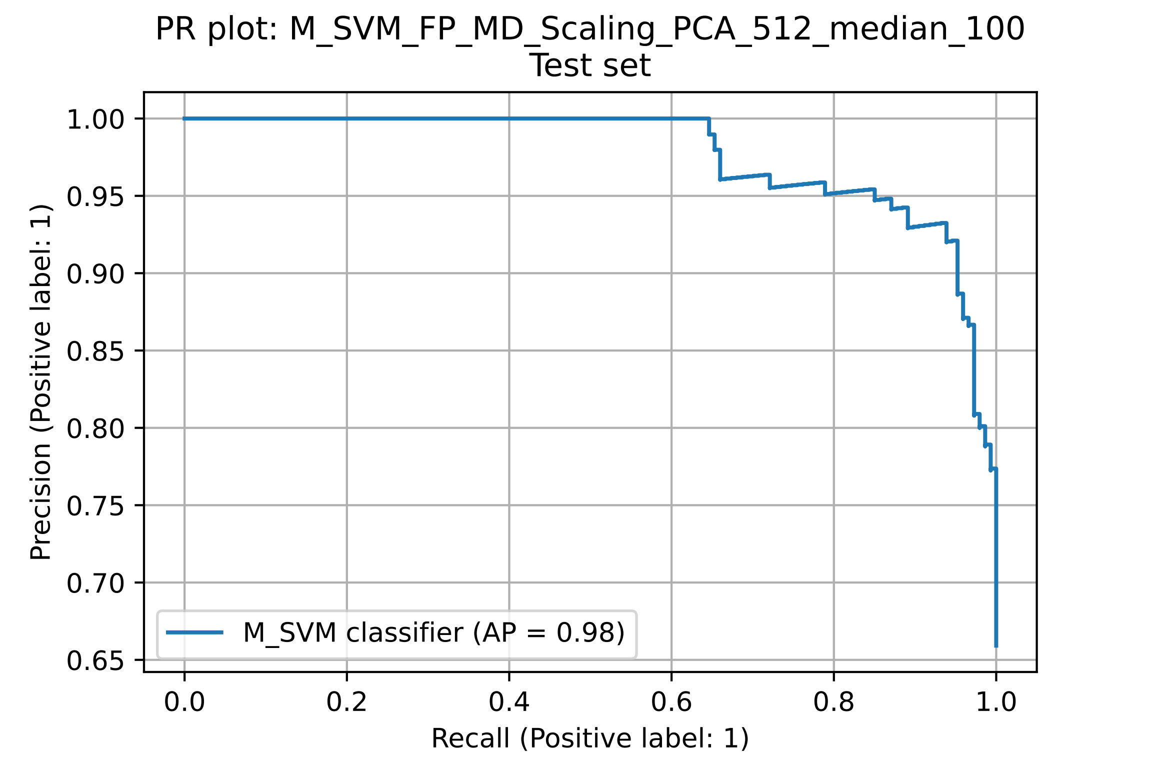
PR plot: M_SVM_FP_MD_Scaling_PCA_512_median_100
Test set
1.00
0.95
0.90
0.85
0.80
0.75
0.70
0.65
Precision (Positive label: 1)
0.0
0.2
0.4
0.6
0.8
1.0
Recall (Positive label: 1)
M_SVM classifier (AP = 0.98)

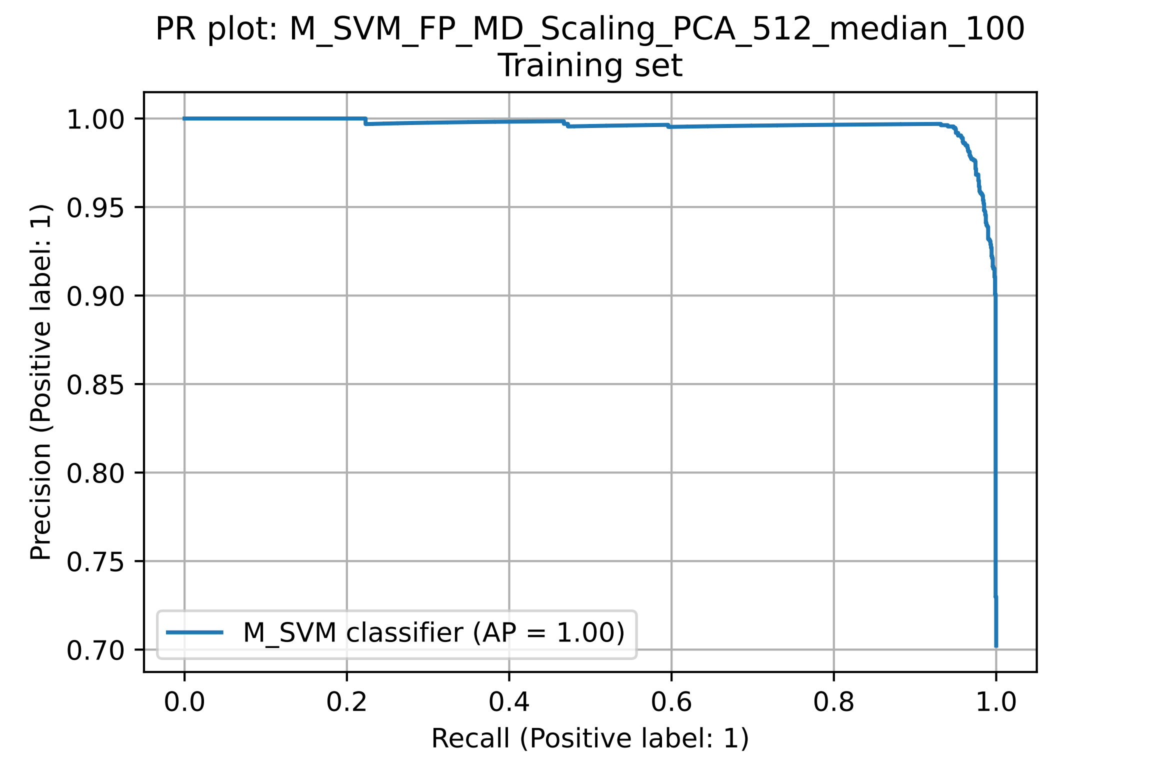
PR plot: M_SVM_FP_MD_Scaling_PCA_512_median_100
Training set
1.00
0.95
0.90
0.85
0.80
0.75
0.70
Precision (Positive label: 1)
0.0
0.2
0.4
0.6
0.8
1.0
Recall (Positive label: 1)
M_SVM classifier (AP = 1.00)

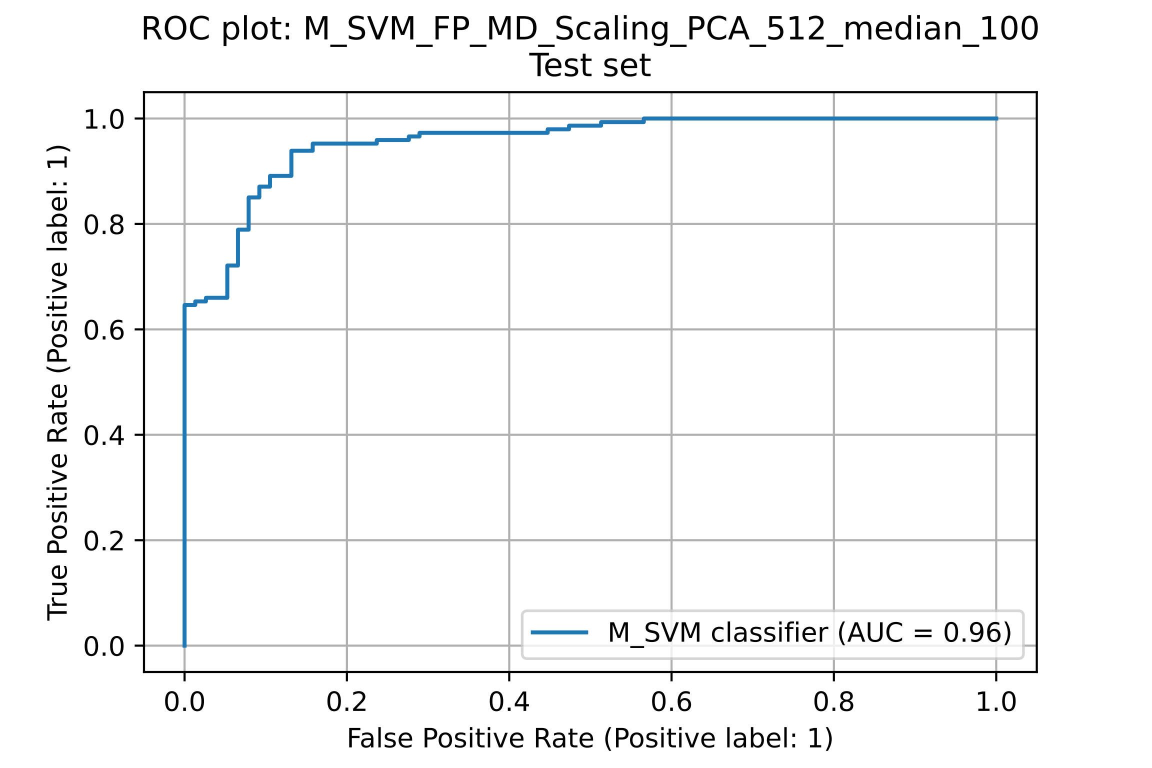

ROC plot: M_SVM_FP_MD_Scaling_PCA_512_median_100
Test set
True Positive Rate (Positive label: 1)
False Positive Rate (Positive label: 1)
0.0
0.2
0.4
0.6
0.8
1.0
M_SVM classifier (AUC = 0.96)

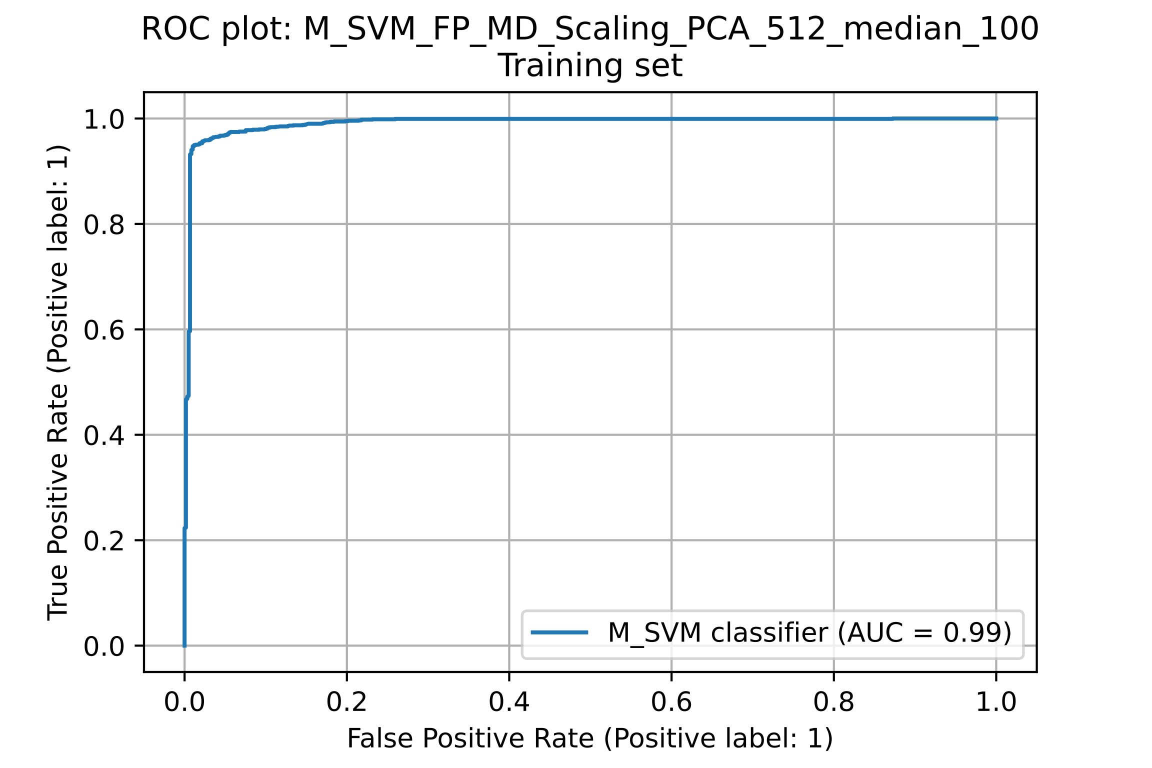
ROC plot: M_SVM_FP_MD_Scaling_PCA_512_median_100
Training set
True Positive Rate (Positive label: 1)
False Positive Rate (Positive label: 1)
0.0
0.2
0.4
0.6
0.8
1.0
M_SVM classifier (AUC = 0.99)

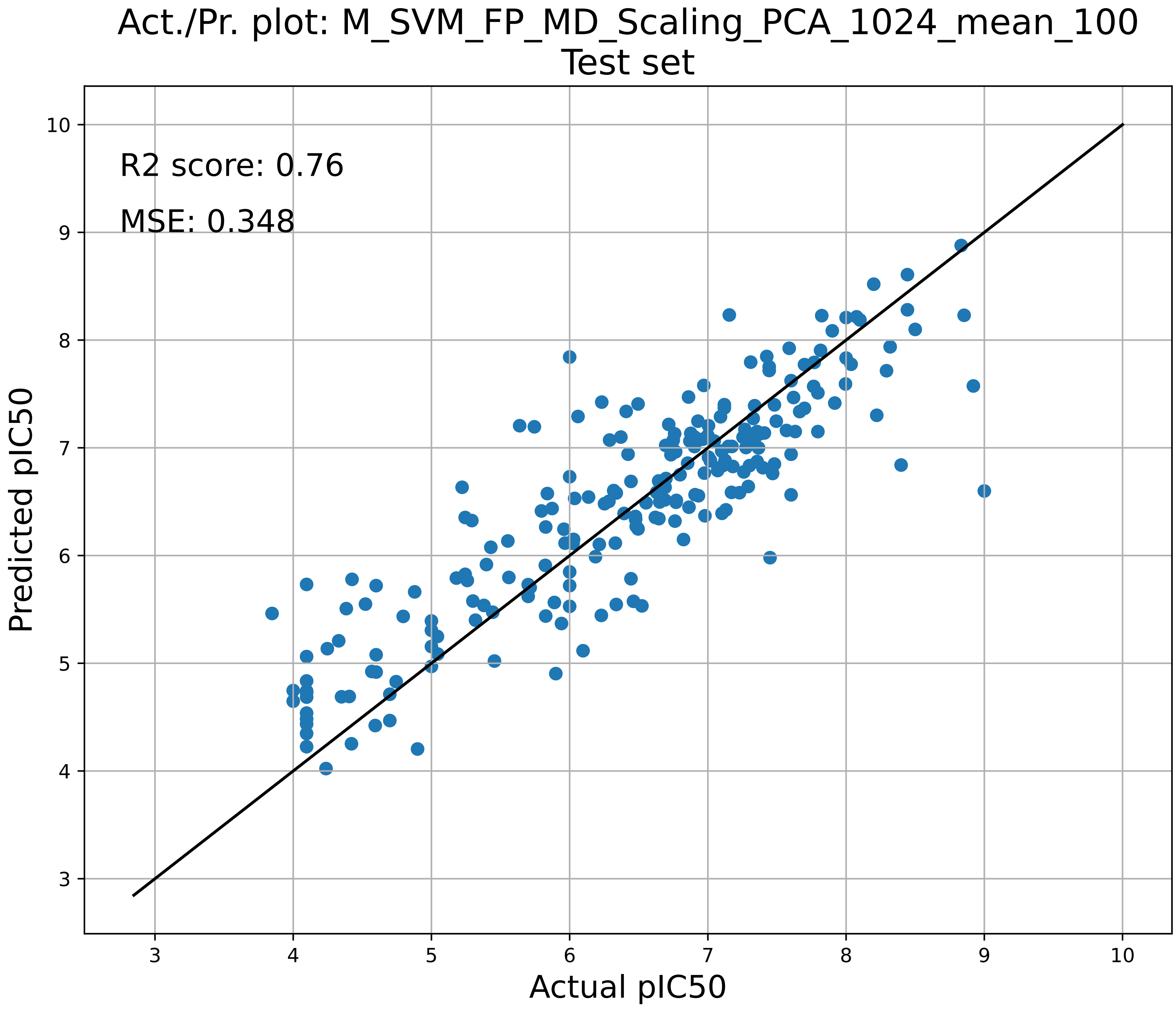

Act./Pr. plot: M_SVM_FP_MD_Scaling_PCA_1024_mean_100
Test set
R2 score: 0.76
MSE: 0.348
Predicted pIC50
Actual pIC50
3
4
5
6
7
8
9
10

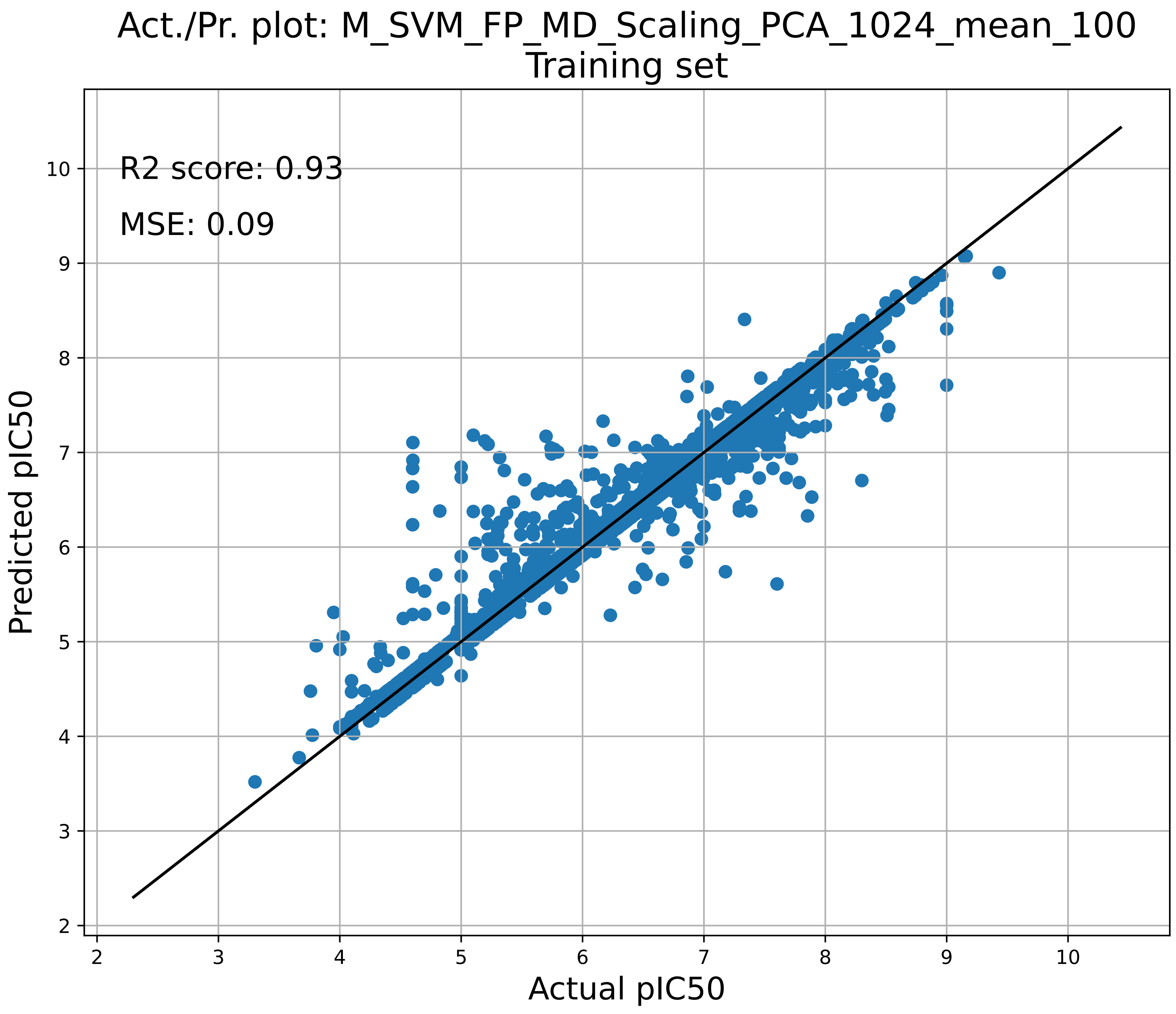

Act./Pr. plot: M_SVM_FP_MD_Scaling_PCA_1024_mean_100
Training set
R2 score: 0.93
MSE: 0.09
Predicted pIC50
Actual pIC50
2
3
4
5
6
7
8
9
10

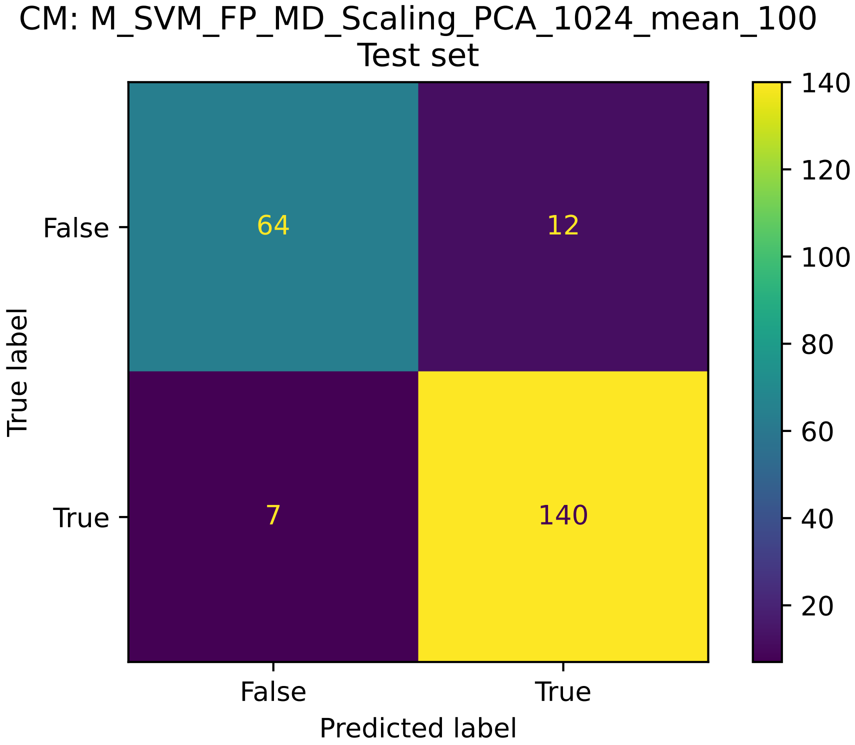
CM: M_SVM_FP_MD_Scaling_PCA_1024_mean_100
Test set
64
12
7
140
False
True
True label
False
True
Predicted label
140
120
100
80
60
40
20

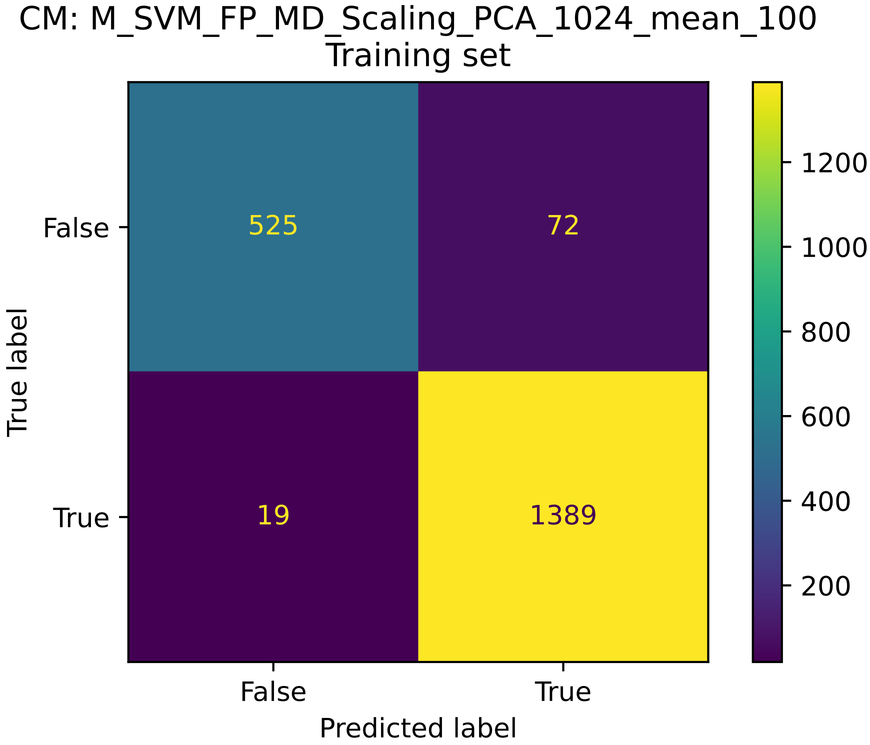

CM: M_SVM_FP_MD_Scaling_PCA_1024_mean_100
Training set
525
72
19
1389
False
True
True label
False
True
Predicted label
1200
1000
800
600
400
200

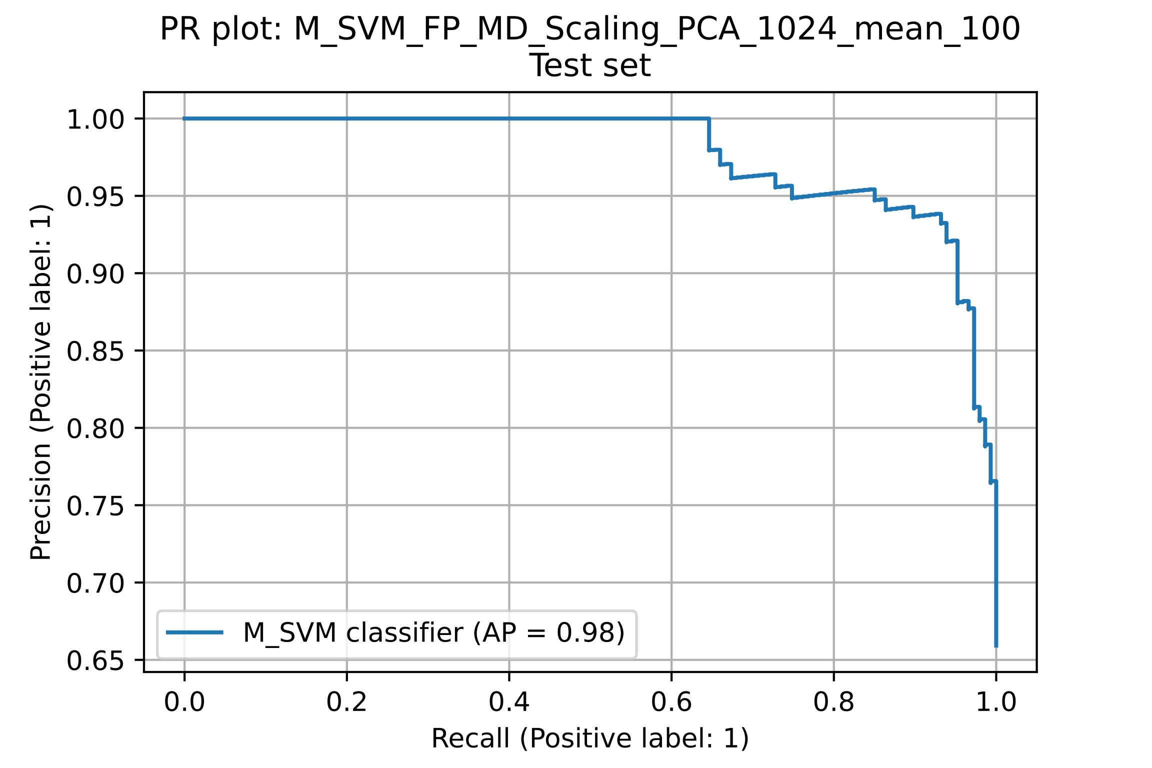

PR plot: M_SVM_FP_MD_Scaling_PCA_1024_mean_100
Test set
Precision (Positive label: 1)
Recall (Positive label: 1)
1.00
0.95
0.90
0.85
0.80
0.75
0.70
0.65
0.0
0.2
0.4
0.6
0.8
1.0
M_SVM classifier (AP = 0.98)

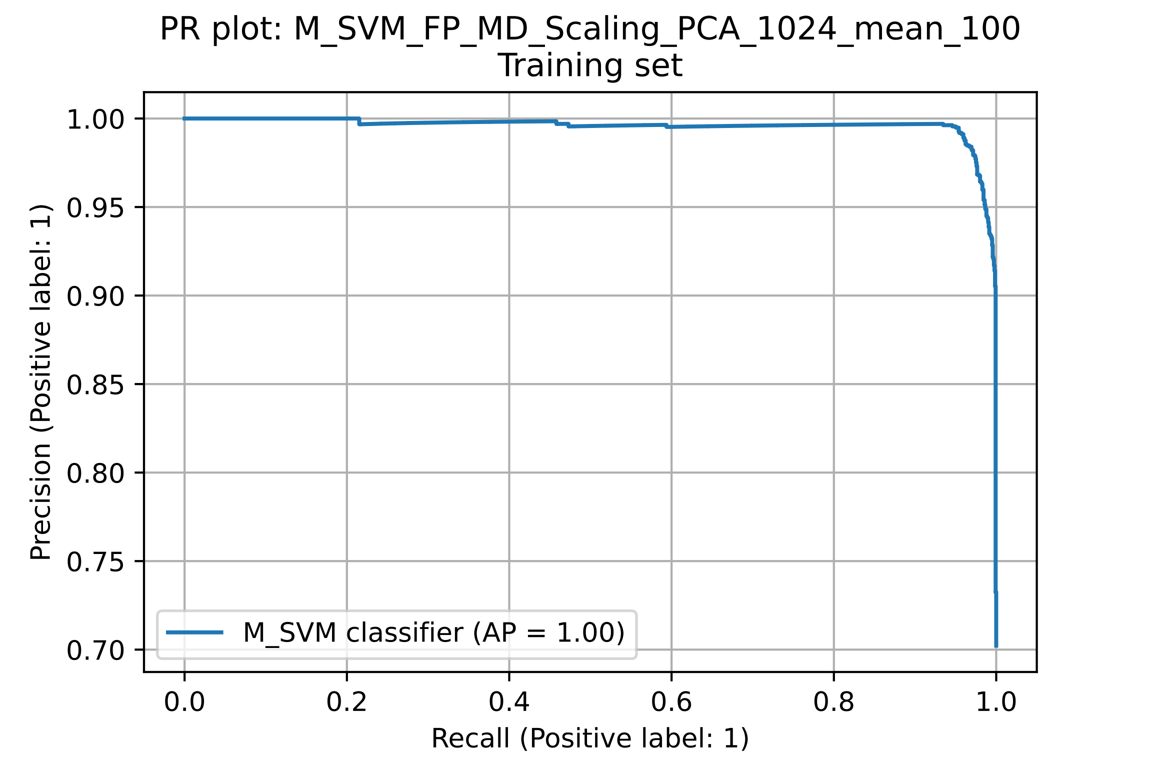

PR plot: M_SVM_FP_MD_Scaling_PCA_1024_mean_100
Training set
Precision (Positive label: 1)
1.00
0.95
0.90
0.85
0.80
0.75
0.70
0.0
0.2
0.4
0.6
0.8
1.0
Recall (Positive label: 1)
M_SVM classifier (AP = 1.00)

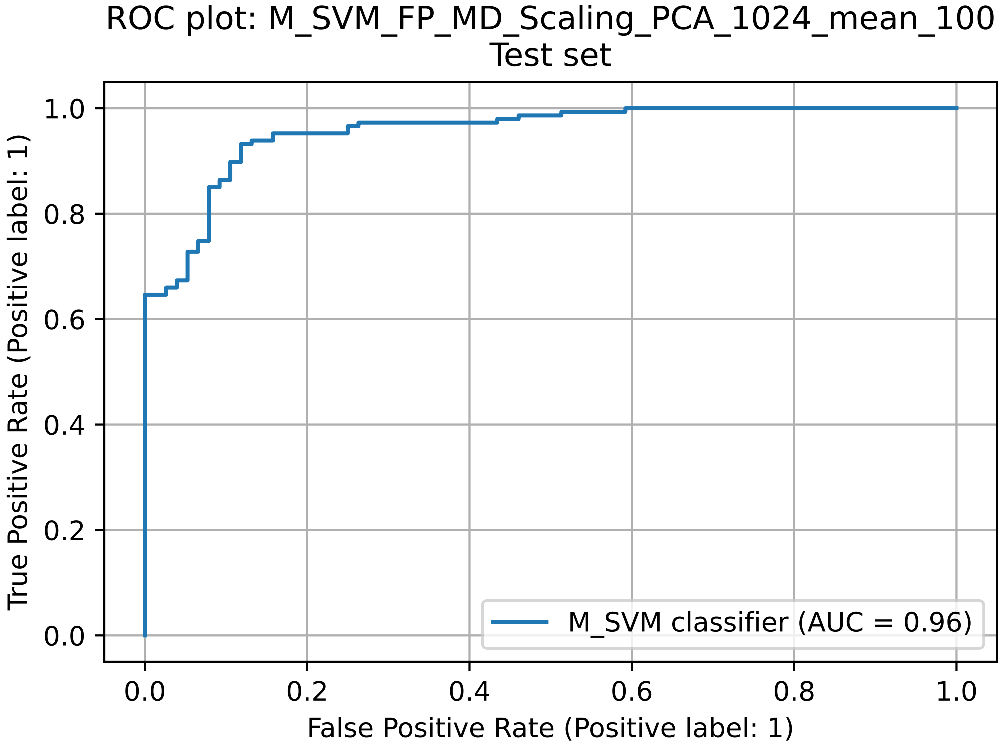
ROC plot: M_SVM_FP_MD_Scaling_PCA_1024_mean_100
Test set
True Positive Rate (Positive label: 1)
False Positive Rate (Positive label: 1)
0.0
0.2
0.4
0.6
0.8
1.0
M_SVM classifier (AUC = 0.96)

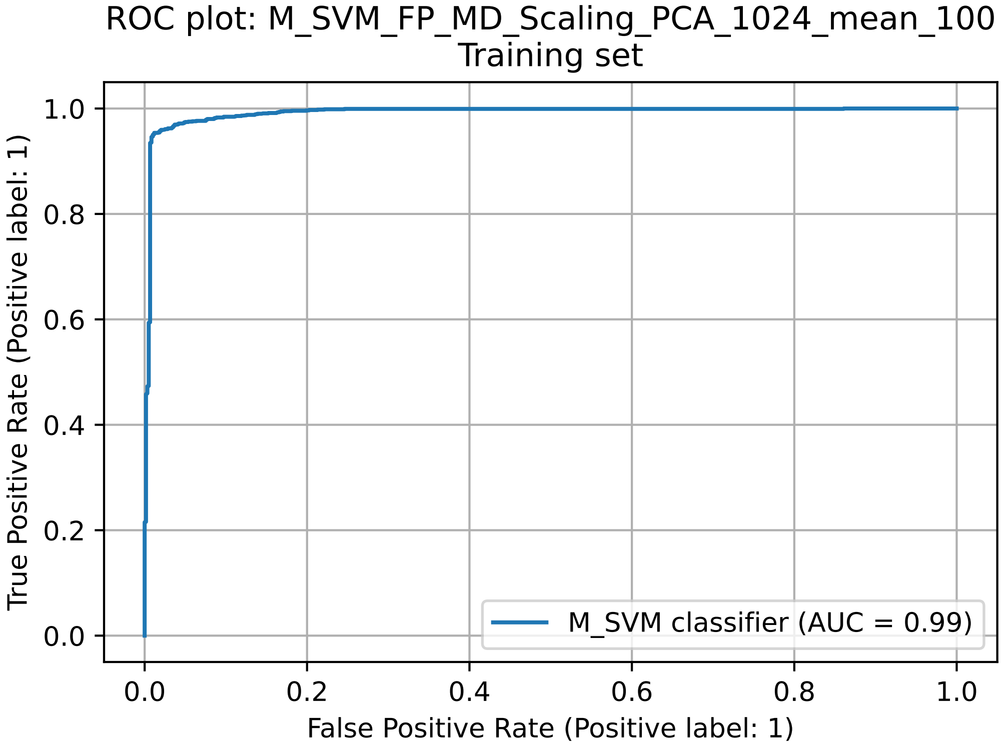
ROC plot: M_SVM_FP_MD_Scaling_PCA_1024_mean_100
Training set
True Positive Rate (Positive label: 1)
False Positive Rate (Positive label: 1)
0.0
0.2
0.4
0.6
0.8
1.0
M_SVM classifier (AUC = 0.99)

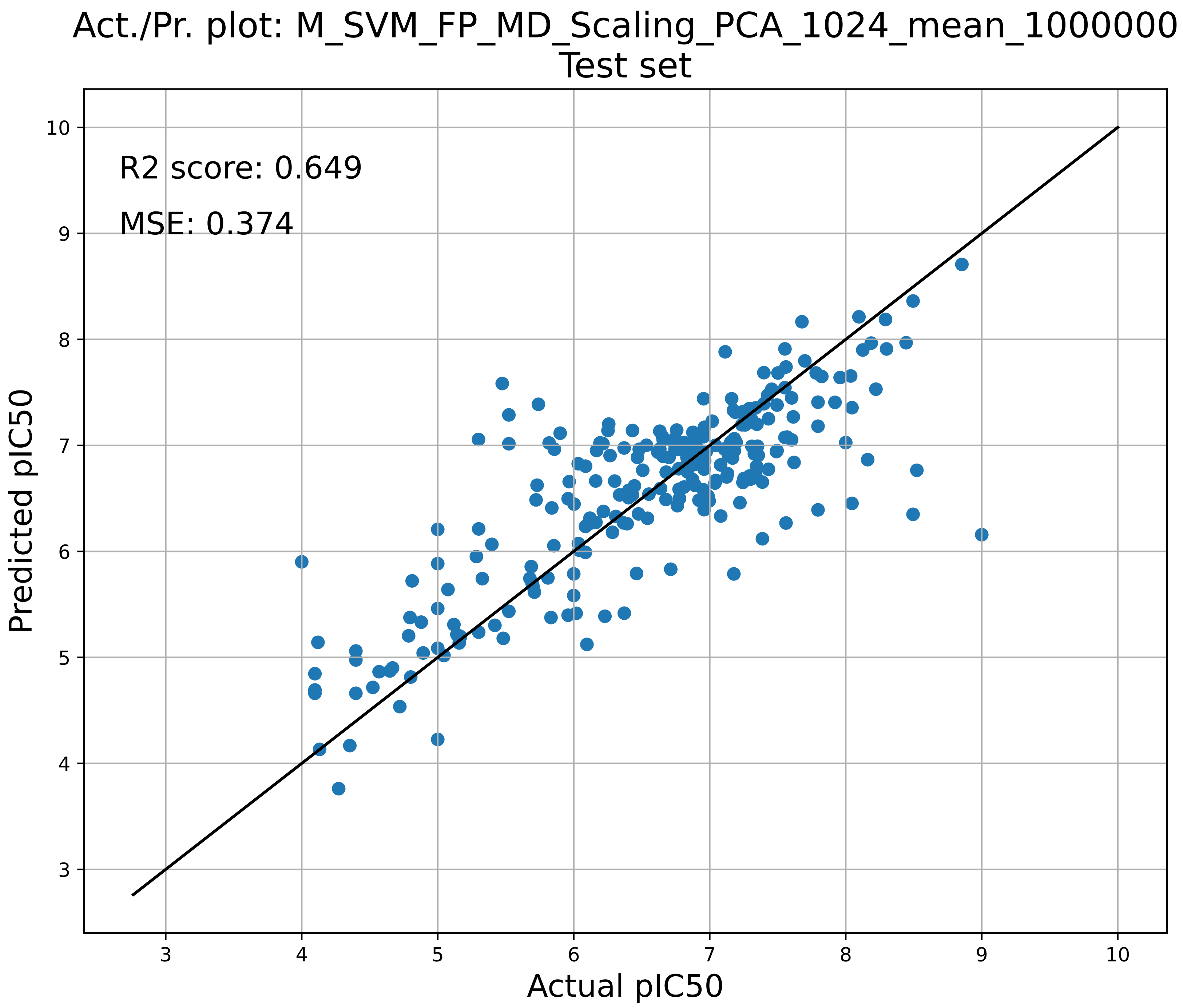
Act./Pr. plot: M_SVM_FP_MD_Scaling_PCA_1024_mean_1000000
Test set
R2 score: 0.649
MSE: 0.374
Predicted pIC50
Actual pIC50
3
4
5
6
7
8
9
10
3
4
5
6
7
8
9
10

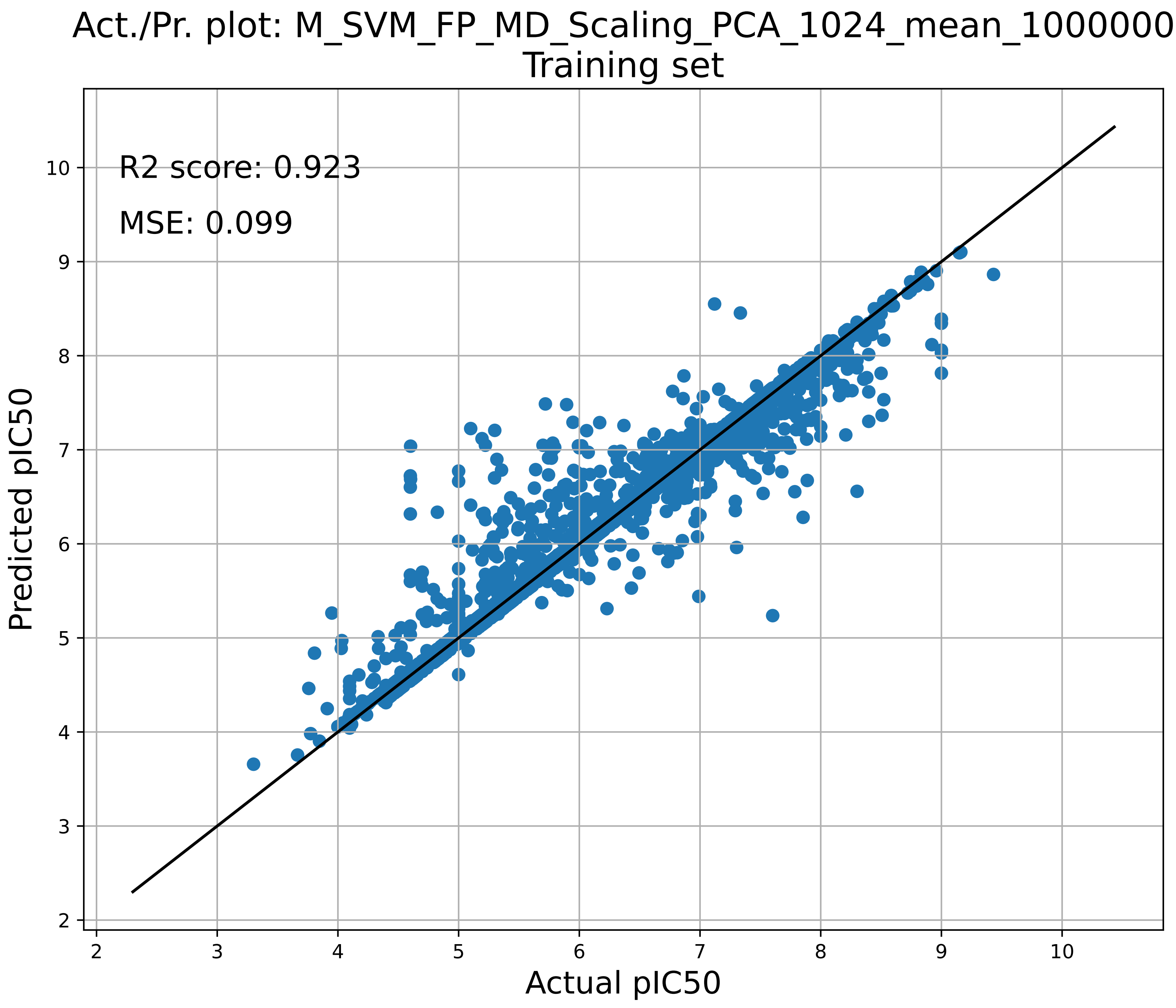

Act./Pr. plot: M_SVM_FP_MD_Scaling_PCA_1024_mean_1000000
Training set
R2 score: 0.923
MSE: 0.099
Predicted pIC50
Actual pIC50
2
3
4
5
6
7
8
9
10

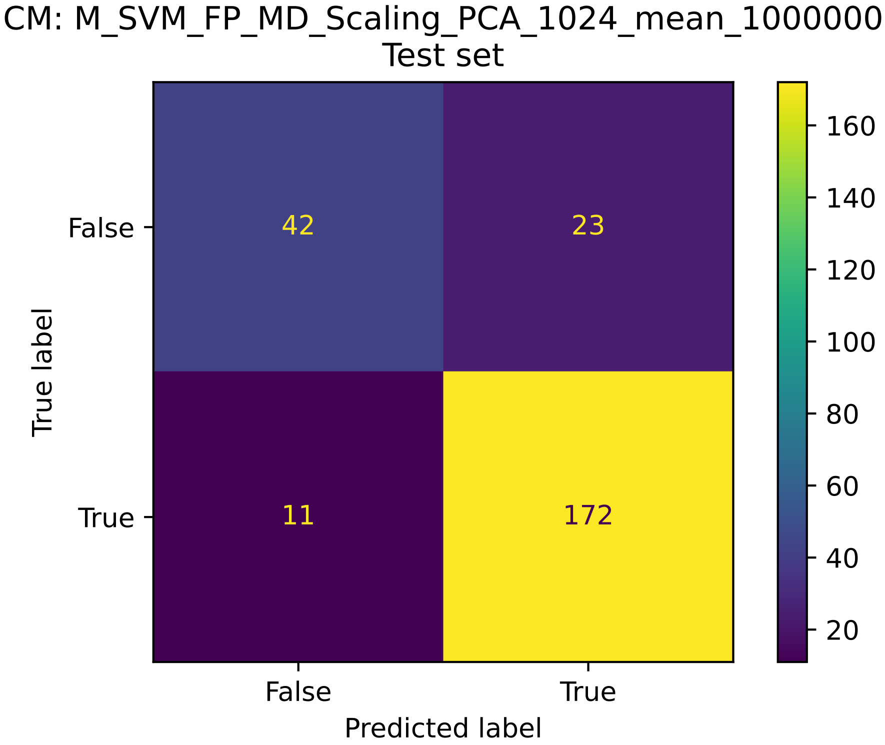
CM: M_SVM_FP_MD_Scaling_PCA_1024_mean_1000000
Test set
True label
Predicted label
False
True
False
True
42
23
11
172
160
140
120
100
80
60
40
20

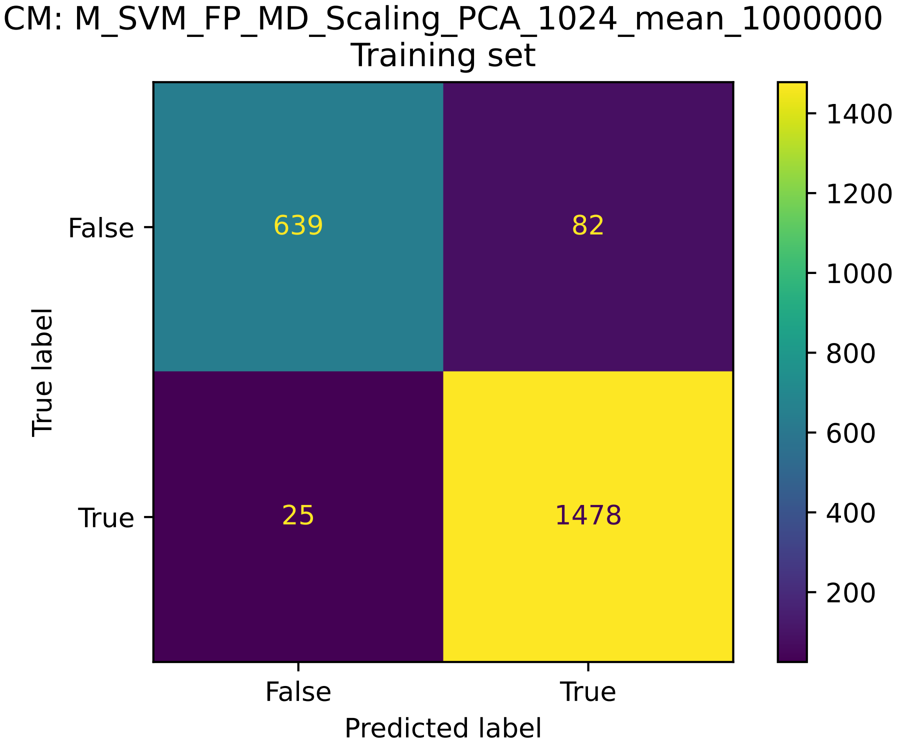

CM: M_SVM_FP_MD_Scaling_PCA_1024_mean_1000000
Training set
True label
False
True
639
82
25
1478
Predicted label
False
True
1400
1200
1000
800
600
400
200

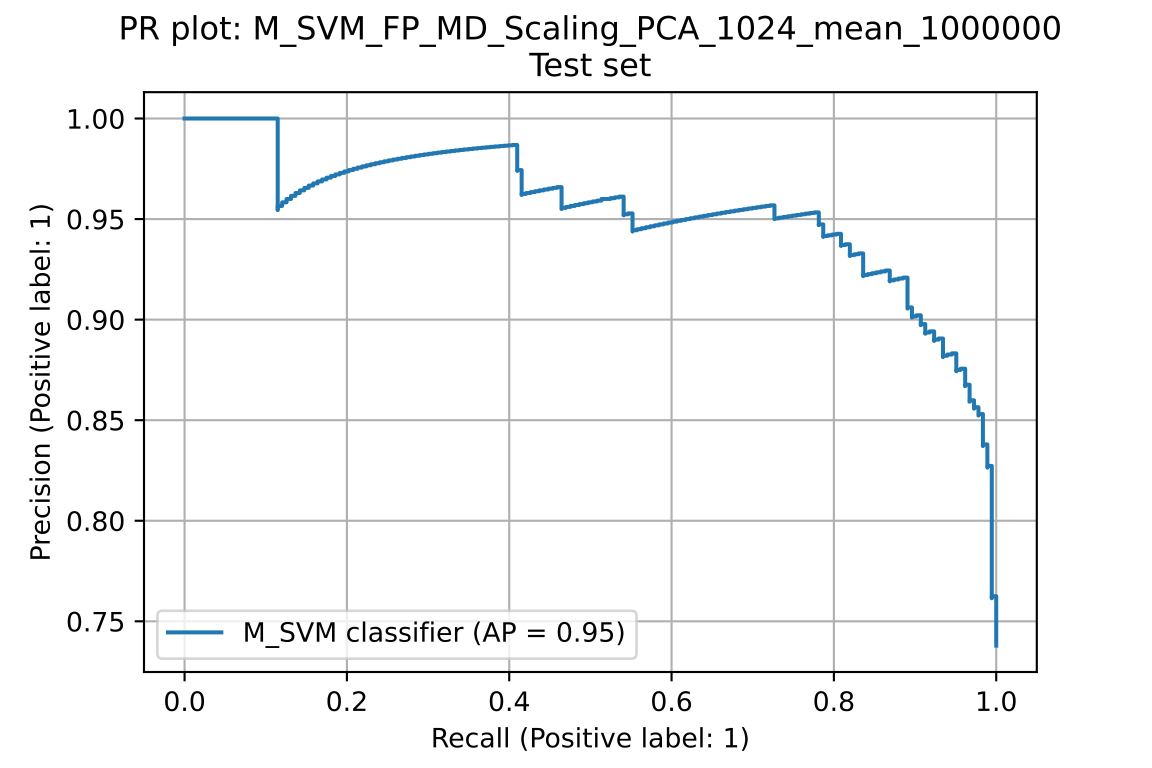

PR plot: M_SVM_FP_MD_Scaling_PCA_1024_mean_1000000
Test set
Precision (Positive label: 1)
1.00
0.95
0.90
0.85
0.80
0.75
Recall (Positive label: 1)
0.0
0.2
0.4
0.6
0.8
1.0
M_SVM classifier (AP = 0.95)

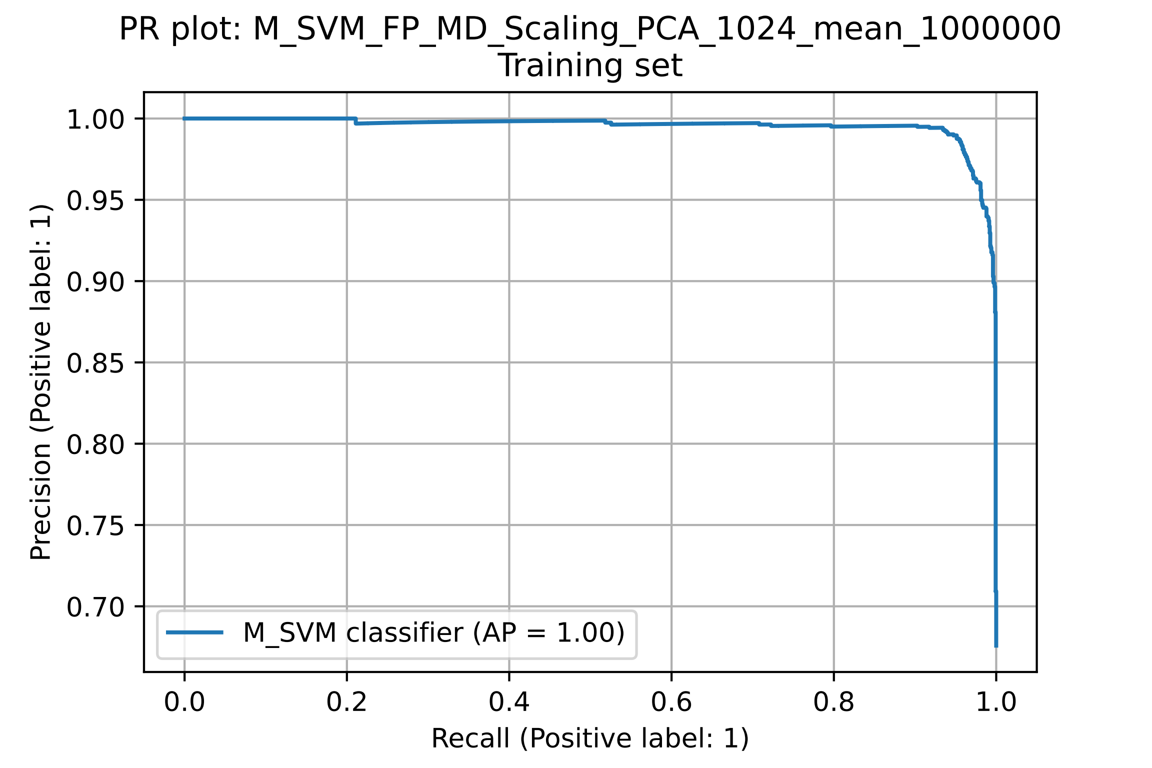
PR plot: M_SVM_FP_MD_Scaling_PCA_1024_mean_1000000
Training set
Precision (Positive label: 1)
Recall (Positive label: 1)
1.00
0.95
0.90
0.85
0.80
0.75
0.70
0.0
0.2
0.4
0.6
0.8
1.0
M_SVM classifier (AP = 1.00)

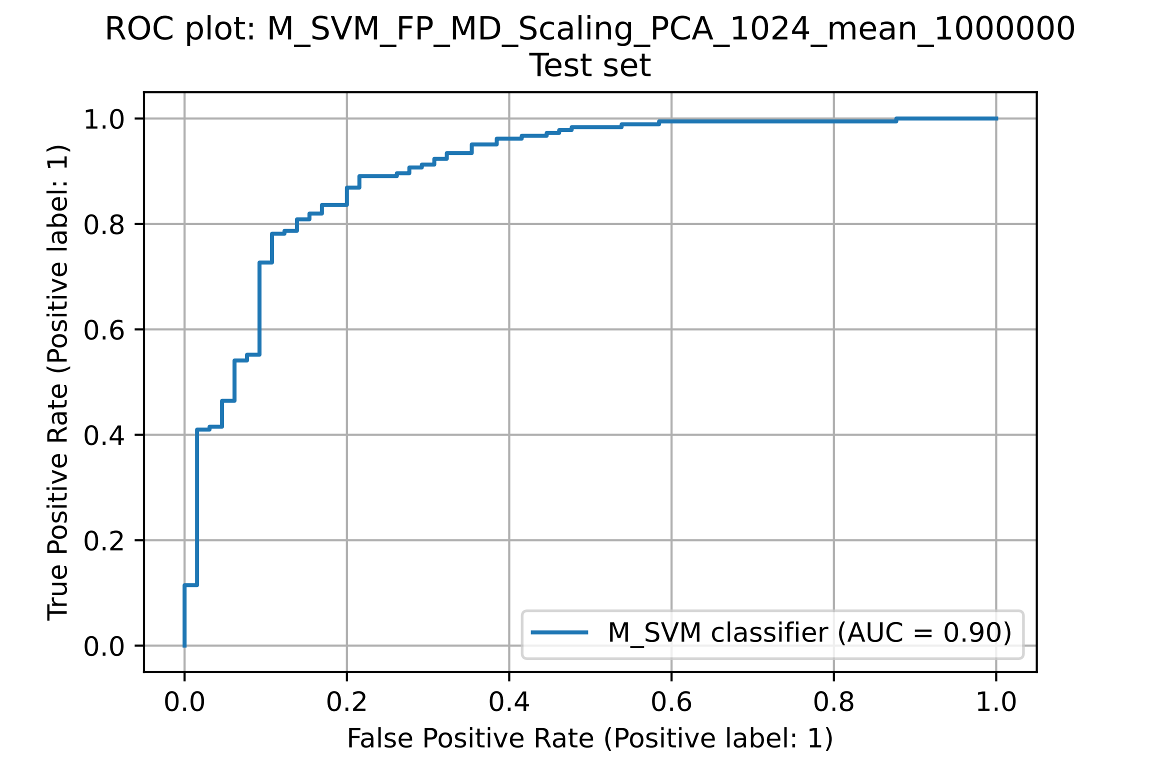

ROC plot: M_SVM_FP_MD_Scaling_PCA_1024_mean_1000000
Test set
True Positive Rate (Positive label: 1)
False Positive Rate (Positive label: 1)
0.0
0.2
0.4
0.6
0.8
1.0
M_SVM classifier (AUC = 0.90)

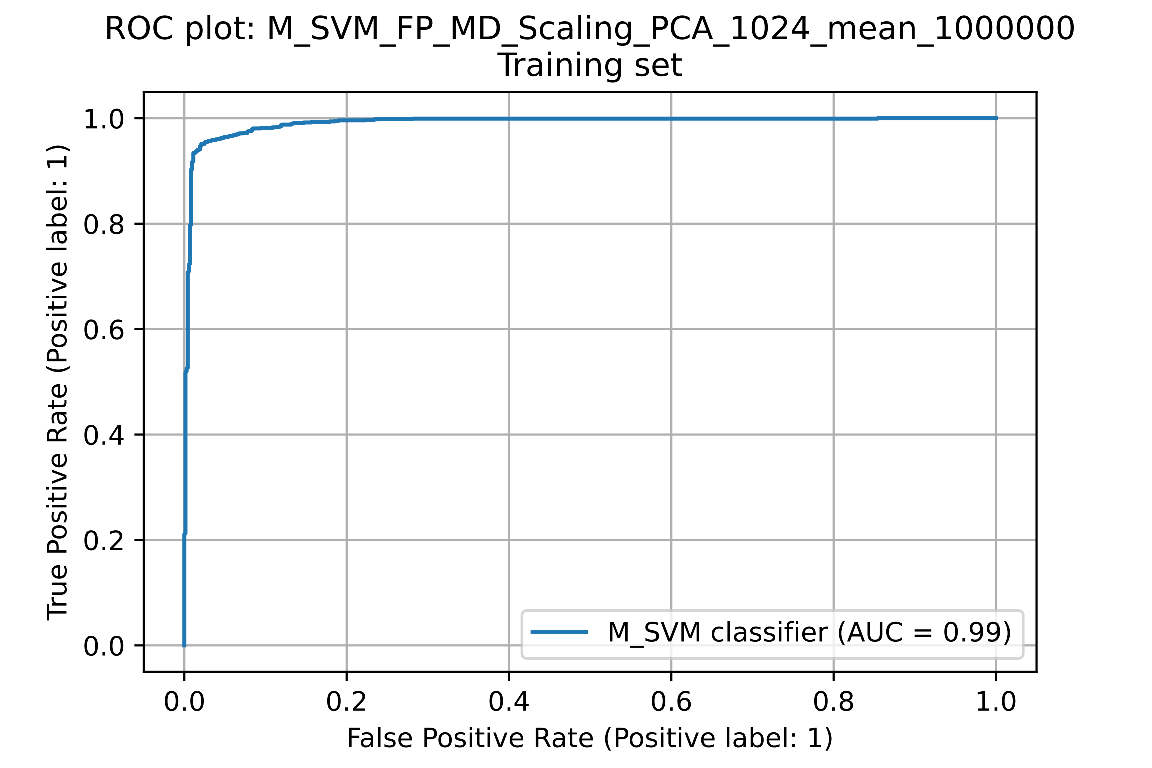
ROC plot: M_SVM_FP_MD_Scaling_PCA_1024_mean_1000000
Training set
True Positive Rate (Positive label: 1)
False Positive Rate (Positive label: 1)
0.0
0.2
0.4
0.6
0.8
1.0
M_SVM classifier (AUC = 0.99)

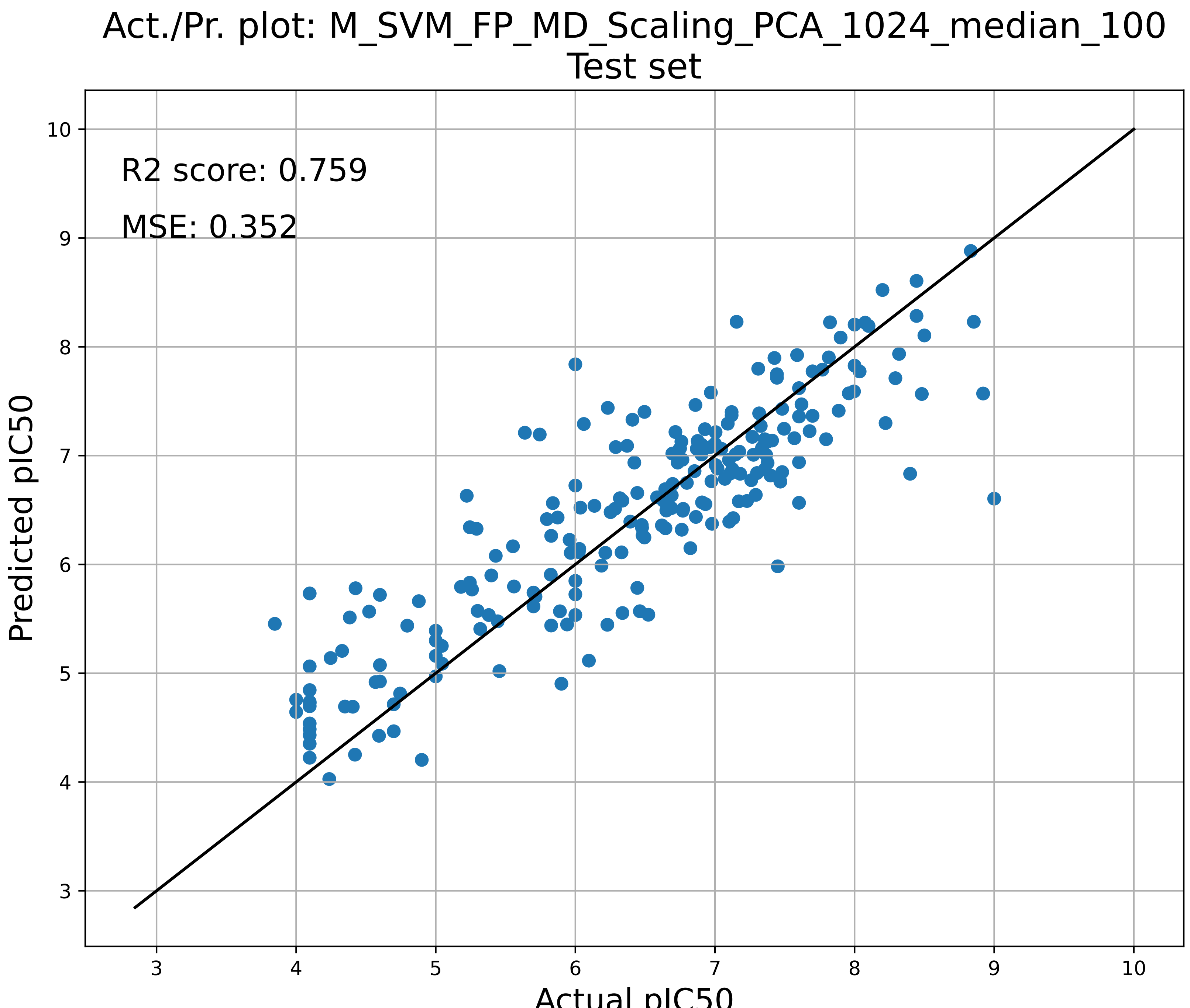

Act./Pr. plot: M_SVM_FP_MD_Scaling_PCA_1024_median_100
Test set
R2 score: 0.759
MSE: 0.352
Predicted pIC50
Actual pIC50
3
4
5
6
7
8
9
10

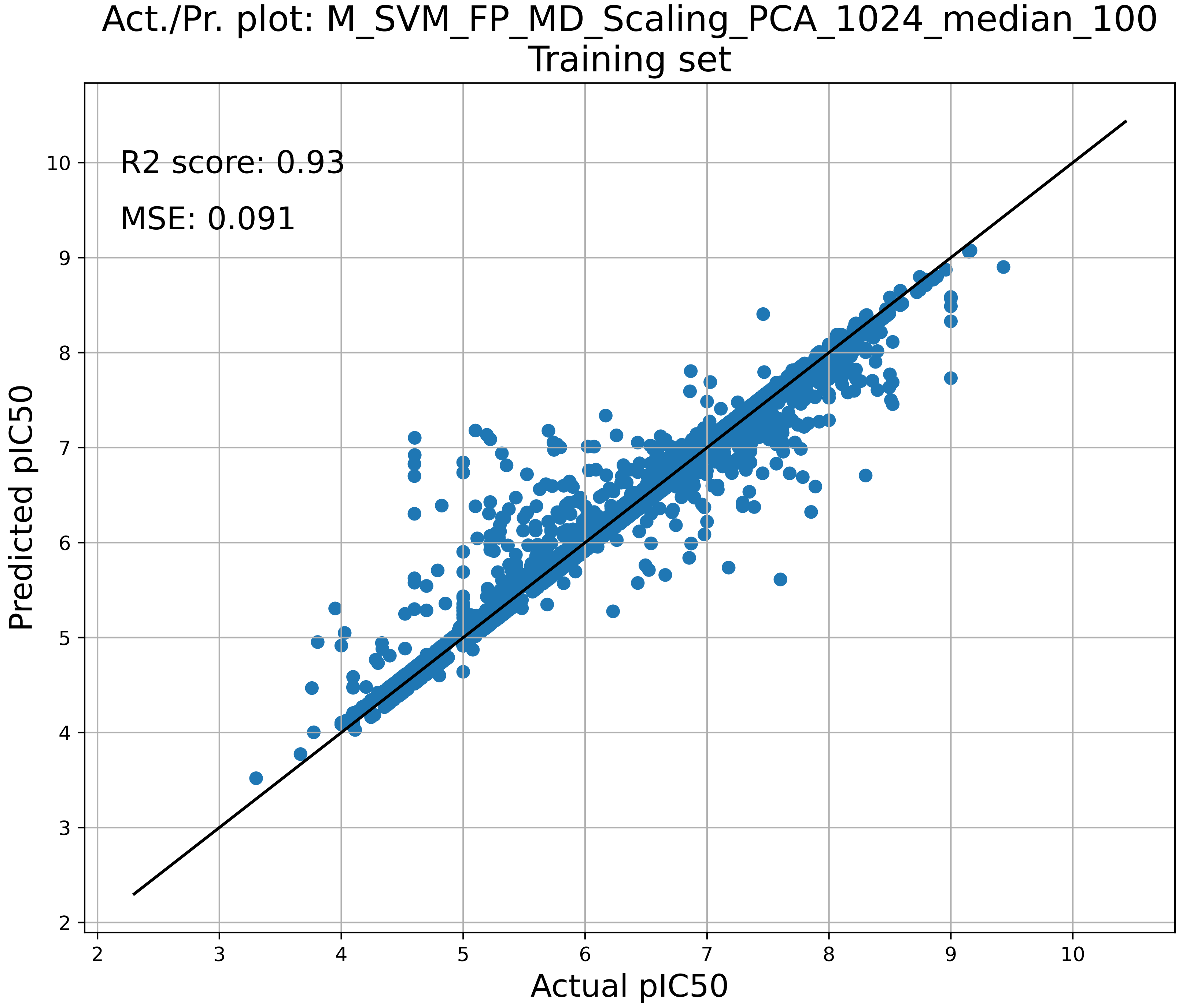

Act./Pr. plot: M_SVM_FP_MD_Scaling_PCA_1024_median_100
Training set
R2 score: 0.93
MSE: 0.091
Predicted pIC50
Actual pIC50
2
3
4
5
6
7
8
9
10

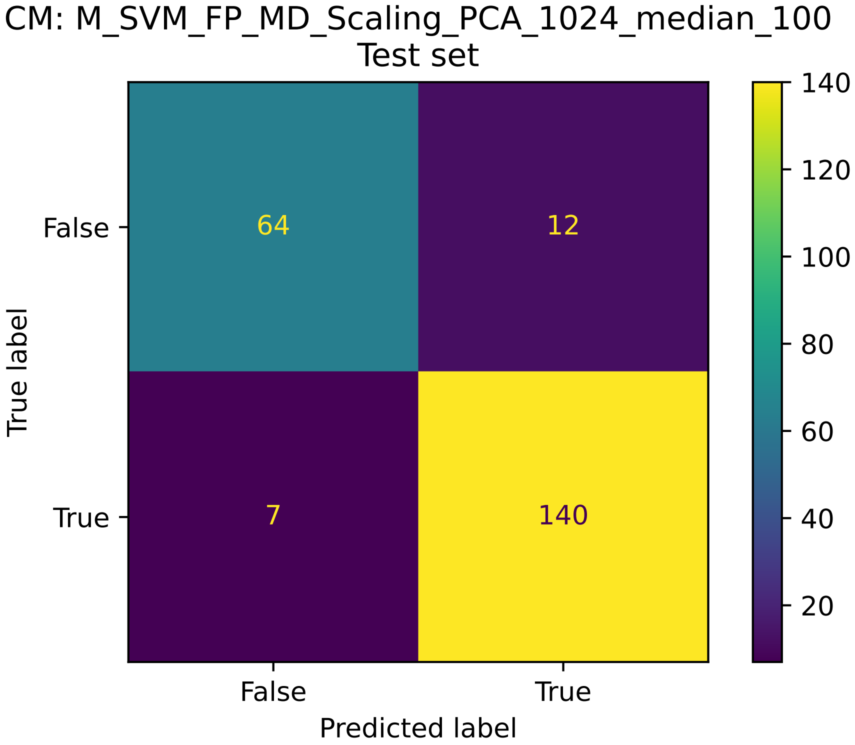
CM: M_SVM_FP_MD_Scaling_PCA_1024_median_100
Test set
True label
False
True
64
12
7
140
Predicted label
False
True
140
120
100
80
60
40
20

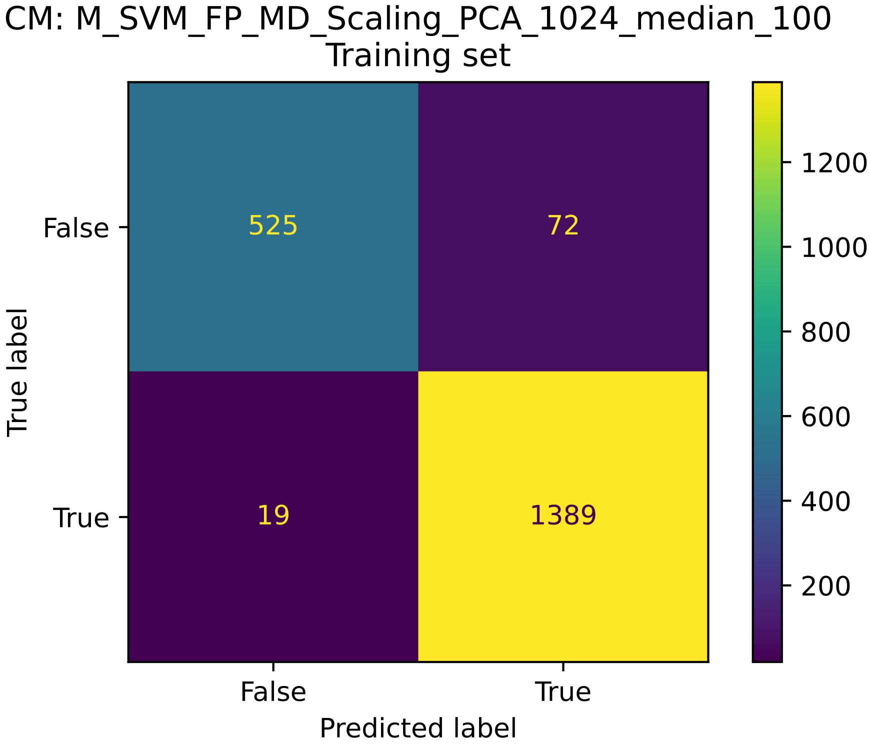
CM: M_SVM_FP_MD_Scaling_PCA_1024_median_100
Training set
True label
False
True
525
72
19
1389
False
True
Predicted label
1200
1000
800
600
400
200

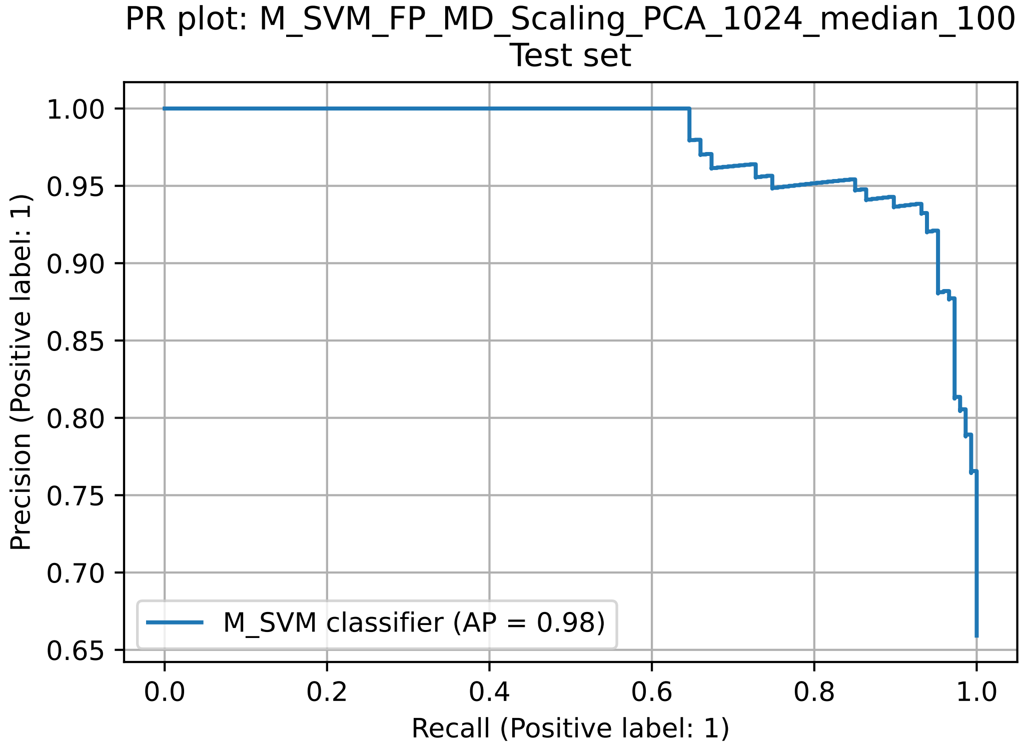
PR plot: M_SVM_FP_MD_Scaling_PCA_1024_median_100
Test set
Precision (Positive label: 1)
Recall (Positive label: 1)
1.00
0.95
0.90
0.85
0.80
0.75
0.70
0.65
0.0
0.2
0.4
0.6
0.8
1.0
M_SVM classifier (AP = 0.98)

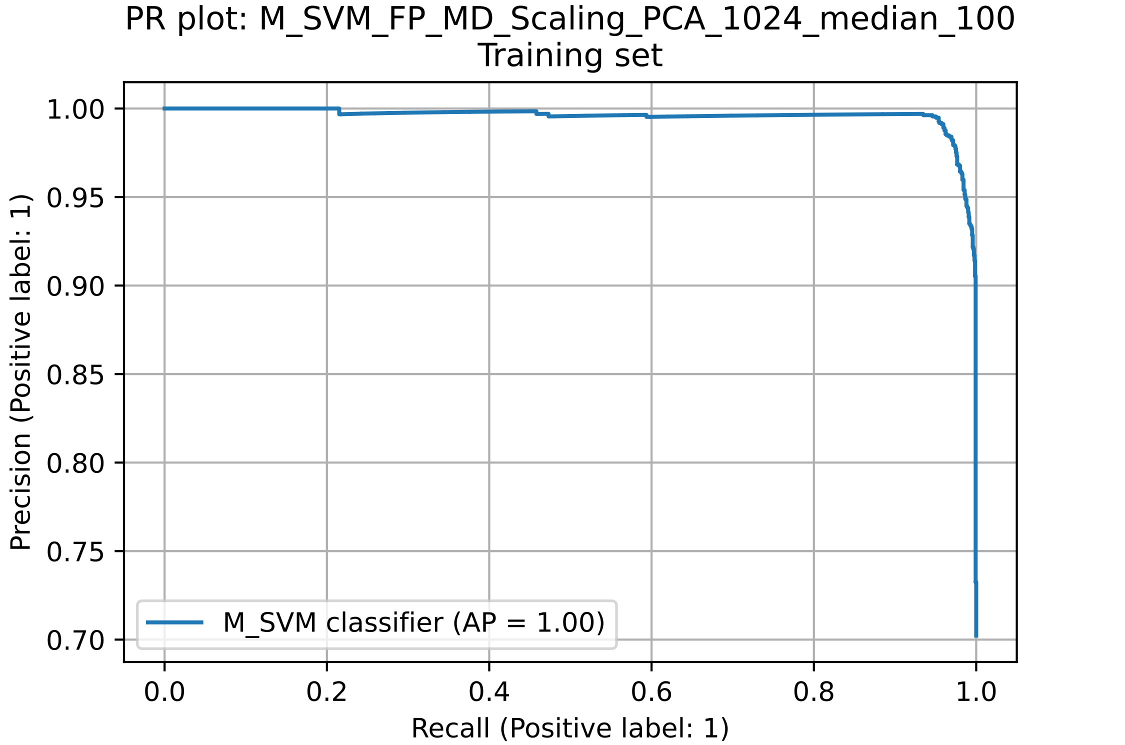

PR plot: M_SVM_FP_MD_Scaling_PCA_1024_median_100
Training set
Precision (Positive label: 1)
1.00
0.95
0.90
0.85
0.80
0.75
0.70
0.0
0.2
0.4
0.6
0.8
1.0
Recall (Positive label: 1)
M_SVM classifier (AP = 1.00)

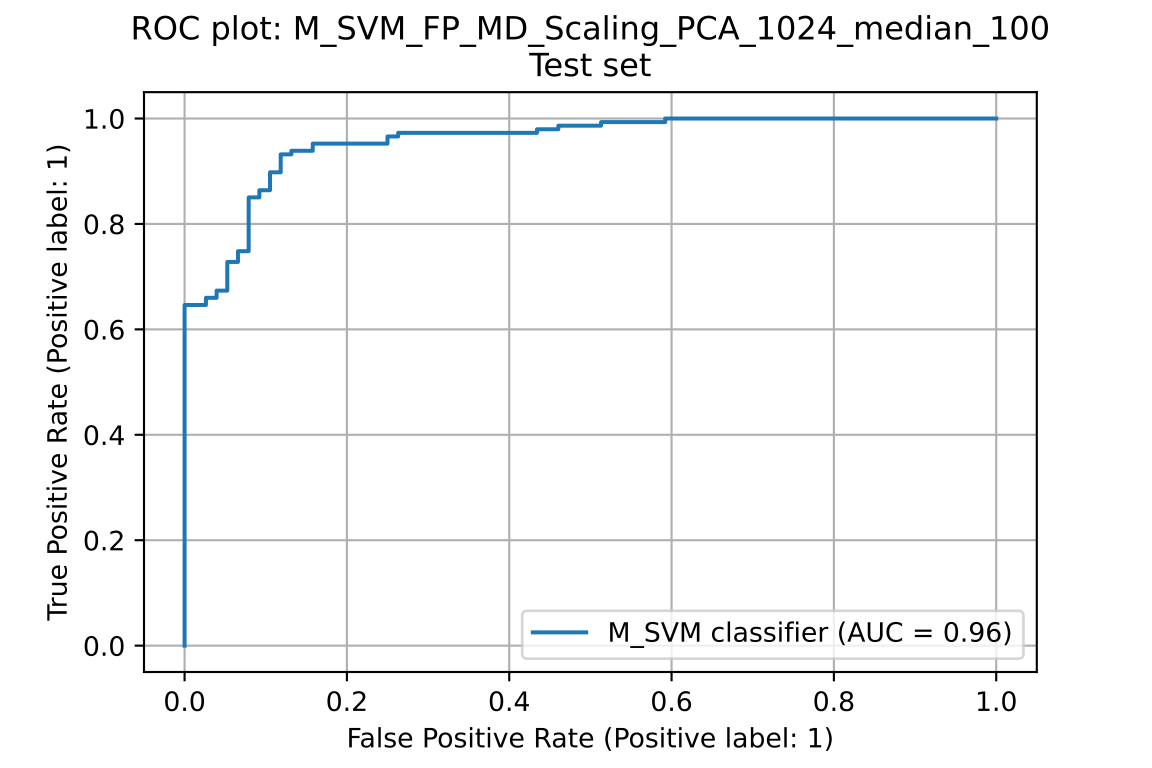

ROC plot: M_SVM_FP_MD_Scaling_PCA_1024_median_100
Test set
True Positive Rate (Positive label: 1)
False Positive Rate (Positive label: 1)
0.0
0.2
0.4
0.6
0.8
1.0
M_SVM classifier (AUC = 0.96)

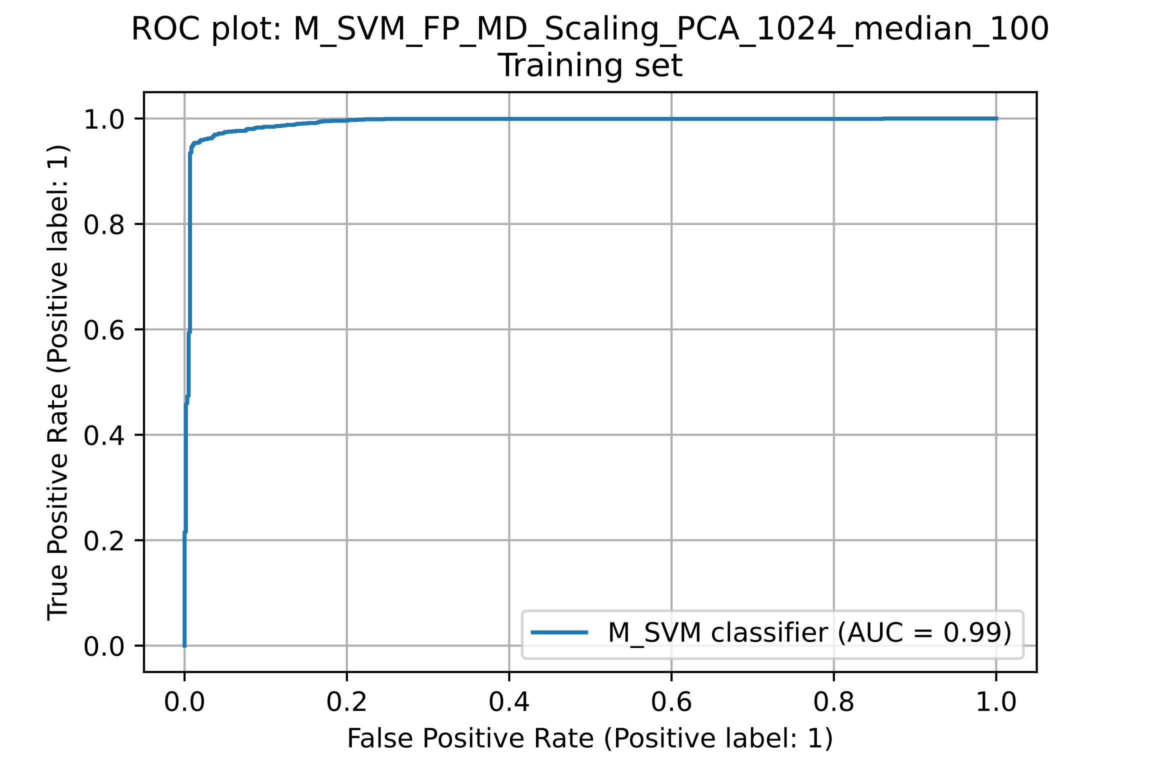
ROC plot: M_SVM_FP_MD_Scaling_PCA_1024_median_100
Training set
True Positive Rate (Positive label: 1)
False Positive Rate (Positive label: 1)
0.0
0.2
0.4
0.6
0.8
1.0
M_SVM classifier (AUC = 0.99)

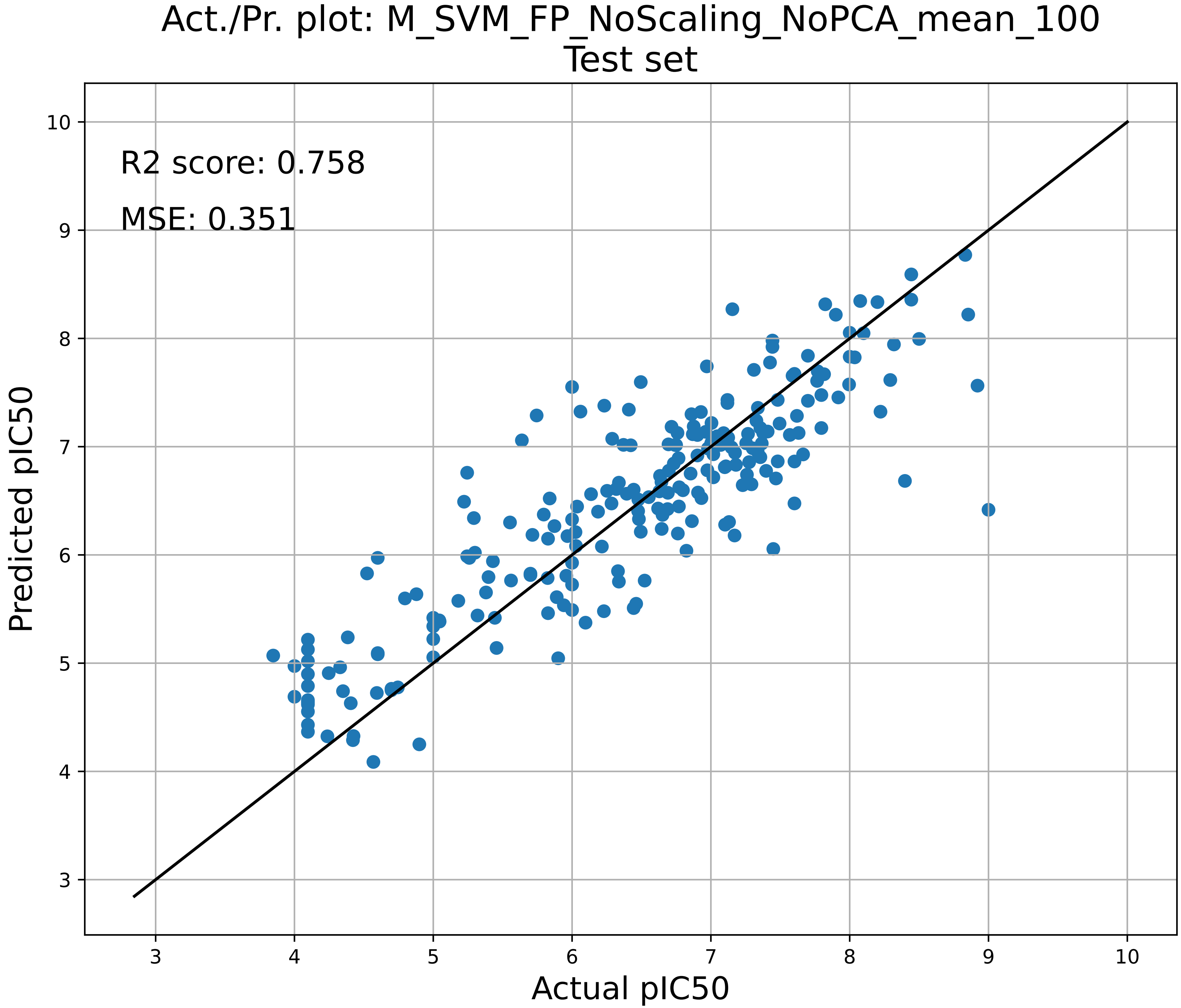

Act./Pr. plot: M_SVM_FP_NoScaling_NoPCA_mean_100
Test set
R2 score: 0.758
MSE: 0.351
Predicted pIC50
Actual pIC50
3
4
5
6
7
8
9
10

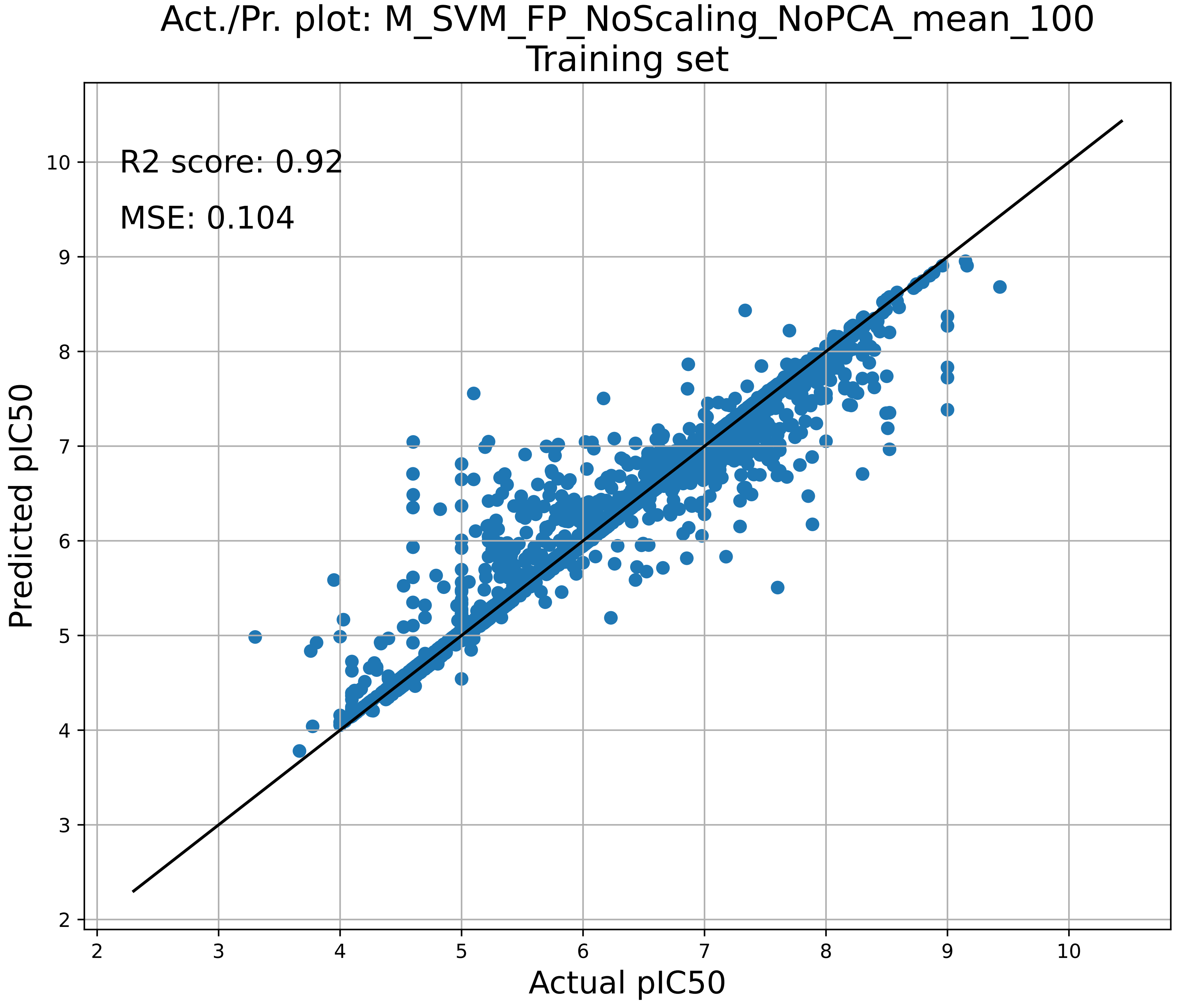
Act./Pr. plot: M_SVM_FP_NoScaling_NoPCA_mean_100
Training set
R2 score: 0.92
MSE: 0.104
Predicted pIC50
Actual pIC50
2
3
4
5
6
7
8
9
10

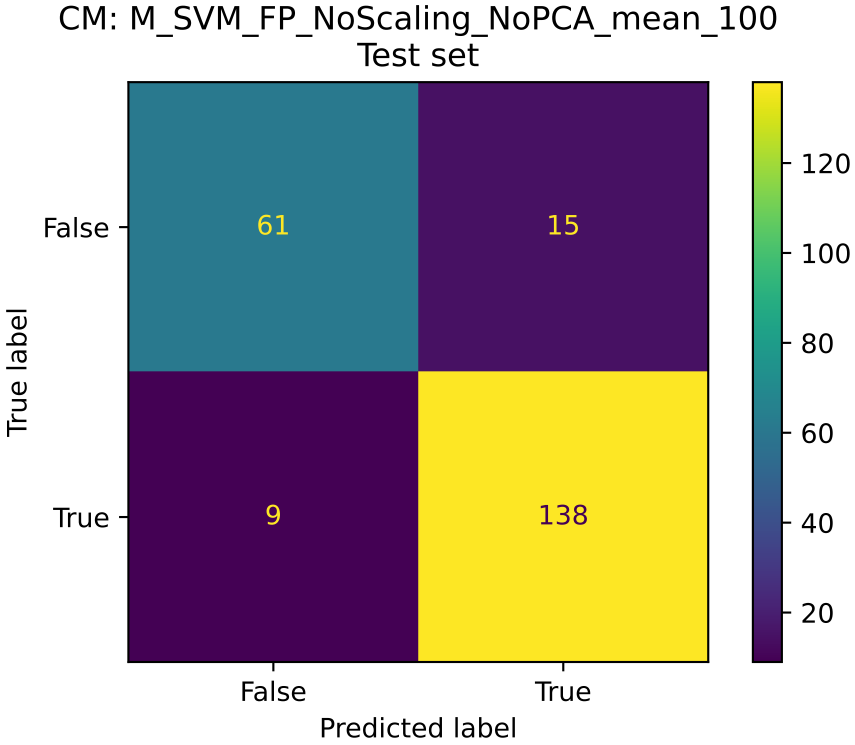
CM: M_SVM_FP_NoScaling_NoPCA_mean_100
Test set
True label
Predicted label
False
True
False
True
61
15
9
138
20
40
60
80
100
120

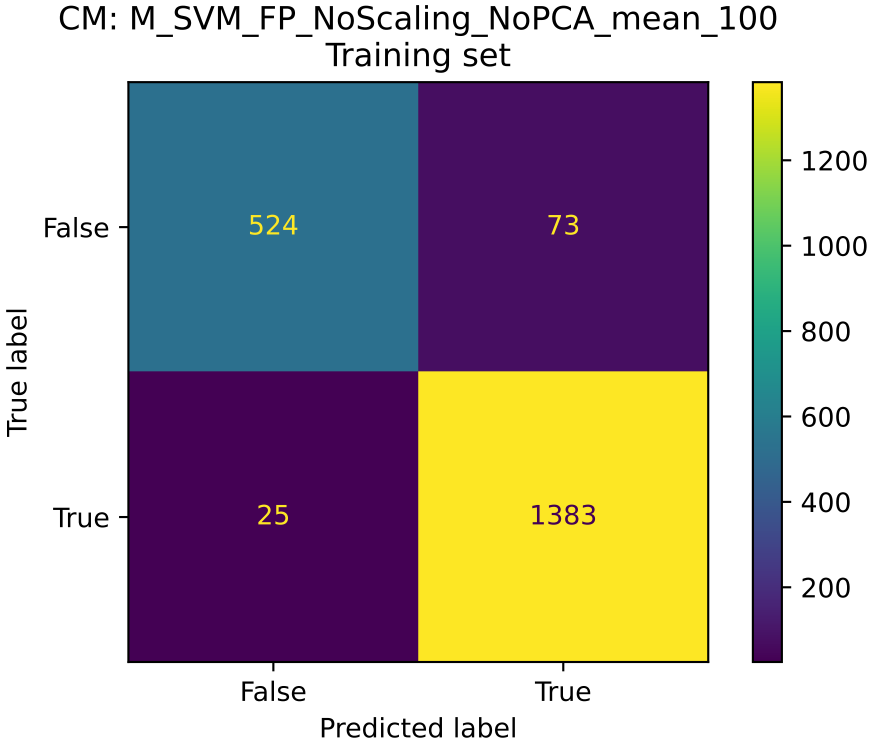
CM: M_SVM_FP_NoScaling_NoPCA_mean_100
Training set
False
True
True label
524
73
25
1383
False
True
Predicted label
1200
1000
800
600
400
200

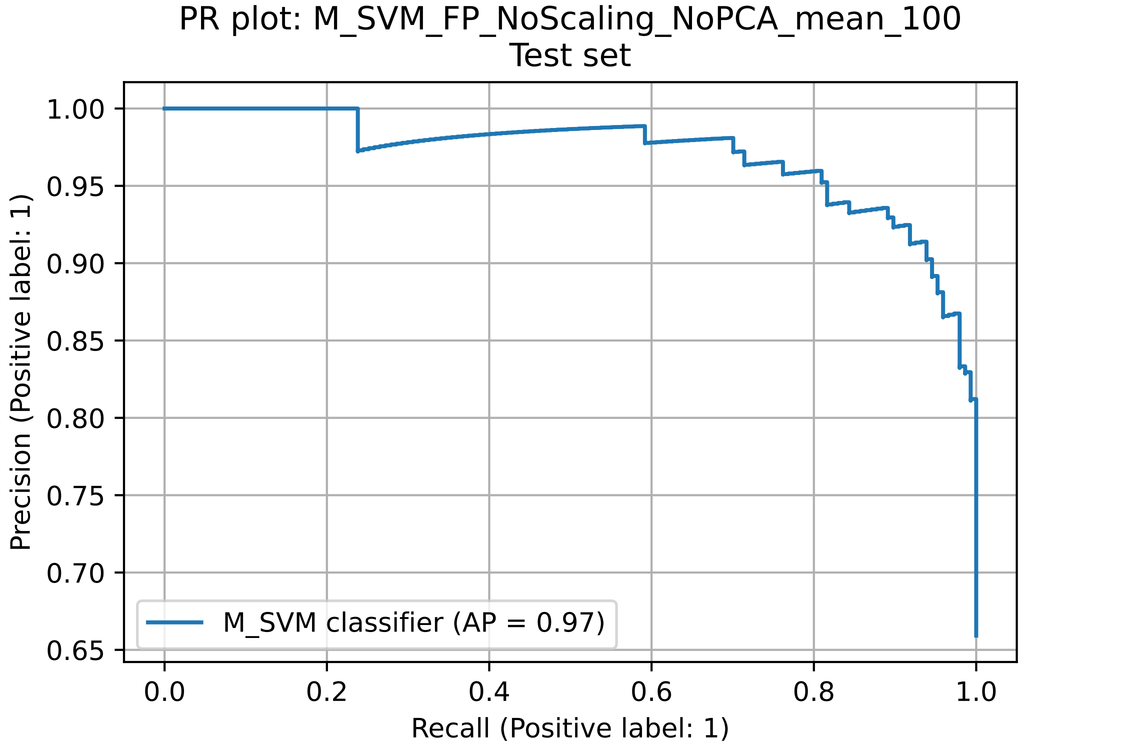
PR plot: M_SVM_FP_NoScaling_NoPCA_mean_100
Test set
Precision (Positive label: 1)
Recall (Positive label: 1)
1.00
0.95
0.90
0.85
0.80
0.75
0.70
0.65
0.0
0.2
0.4
0.6
0.8
1.0
M_SVM classifier (AP = 0.97)

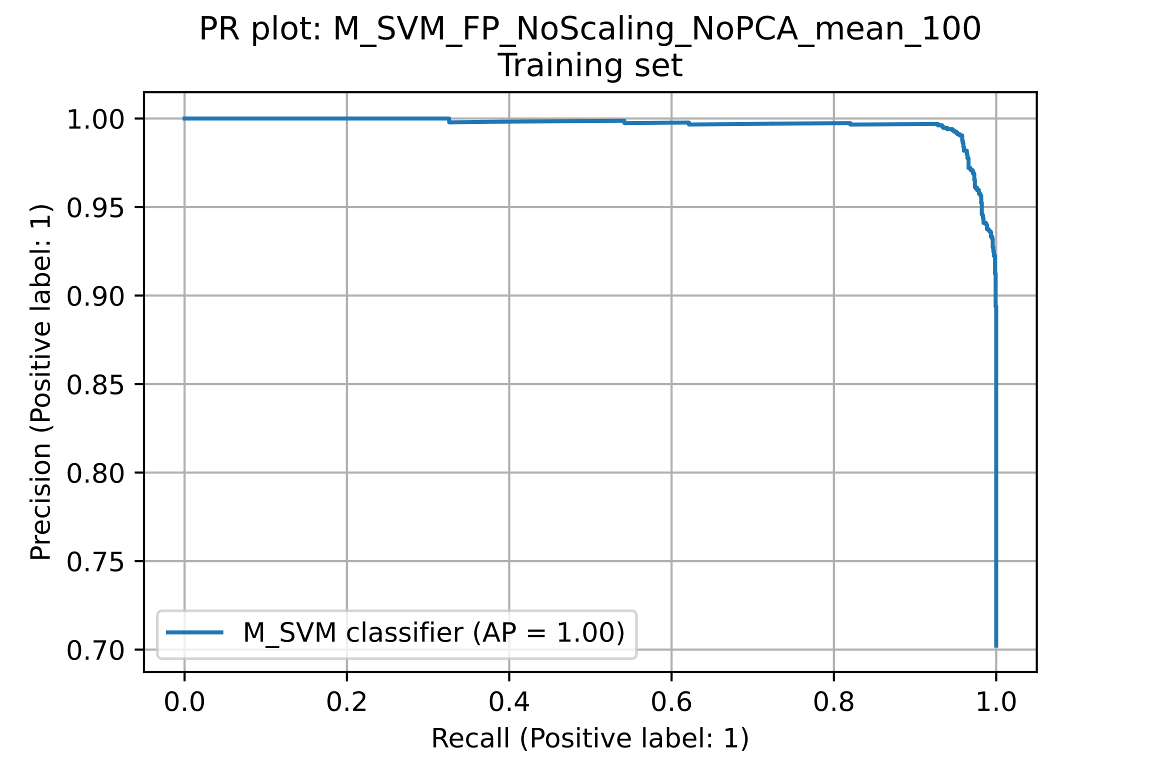

PR plot: M_SVM_FP_NoScaling_NoPCA_mean_100
Training set
Precision (Positive label: 1)
Recall (Positive label: 1)
M_SVM classifier (AP = 1.00)
1.00
0.95
0.90
0.85
0.80
0.75
0.70
0.0
0.2
0.4
0.6
0.8
1.0

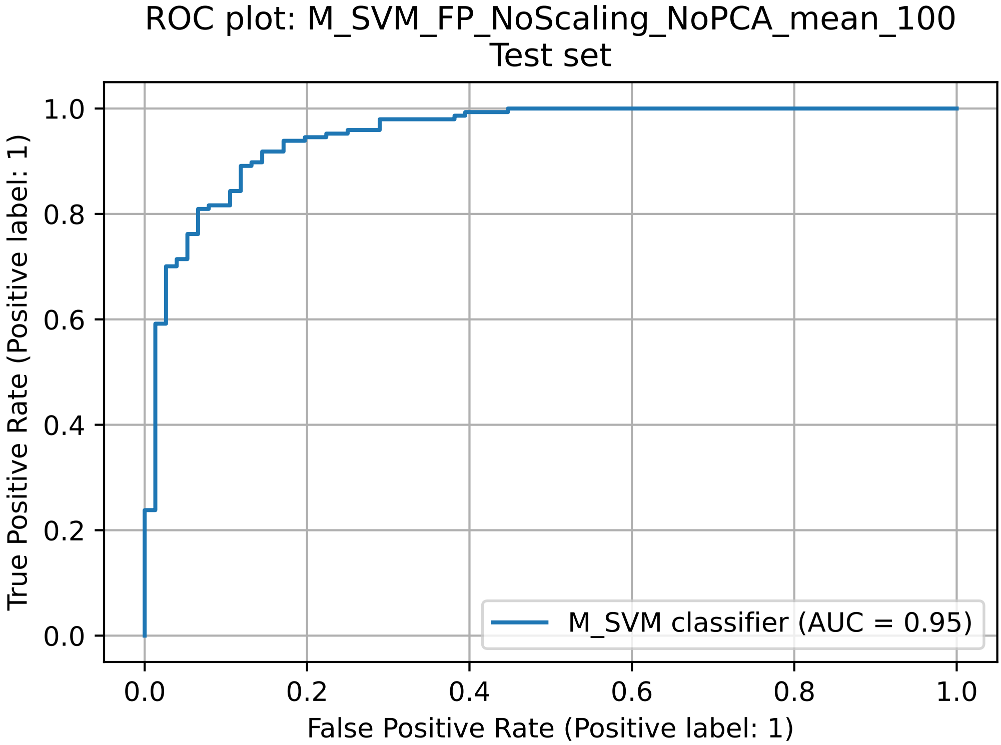
ROC plot: M_SVM_FP_NoScaling_NoPCA_mean_100
Test set
True Positive Rate (Positive label: 1)
False Positive Rate (Positive label: 1)
0.0
0.2
0.4
0.6
0.8
1.0
M_SVM classifier (AUC = 0.95)

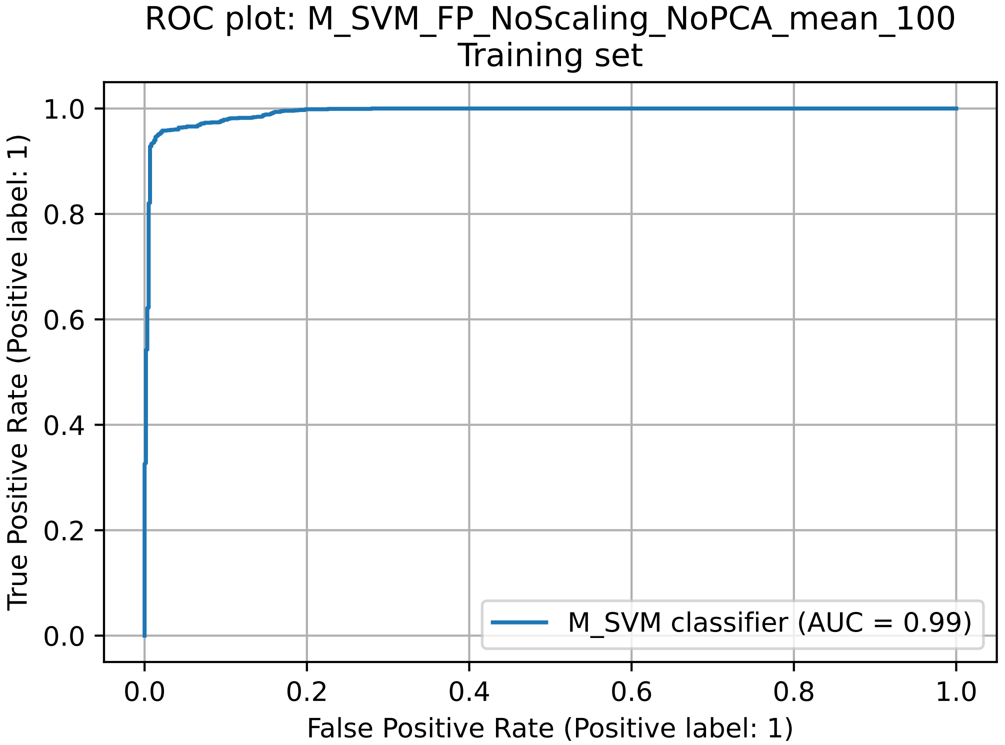
ROC plot: M_SVM_FP_NoScaling_NoPCA_mean_100
Training set
True Positive Rate (Positive label: 1)
False Positive Rate (Positive label: 1)
0.0
0.2
0.4
0.6
0.8
1.0
M_SVM classifier (AUC = 0.99)

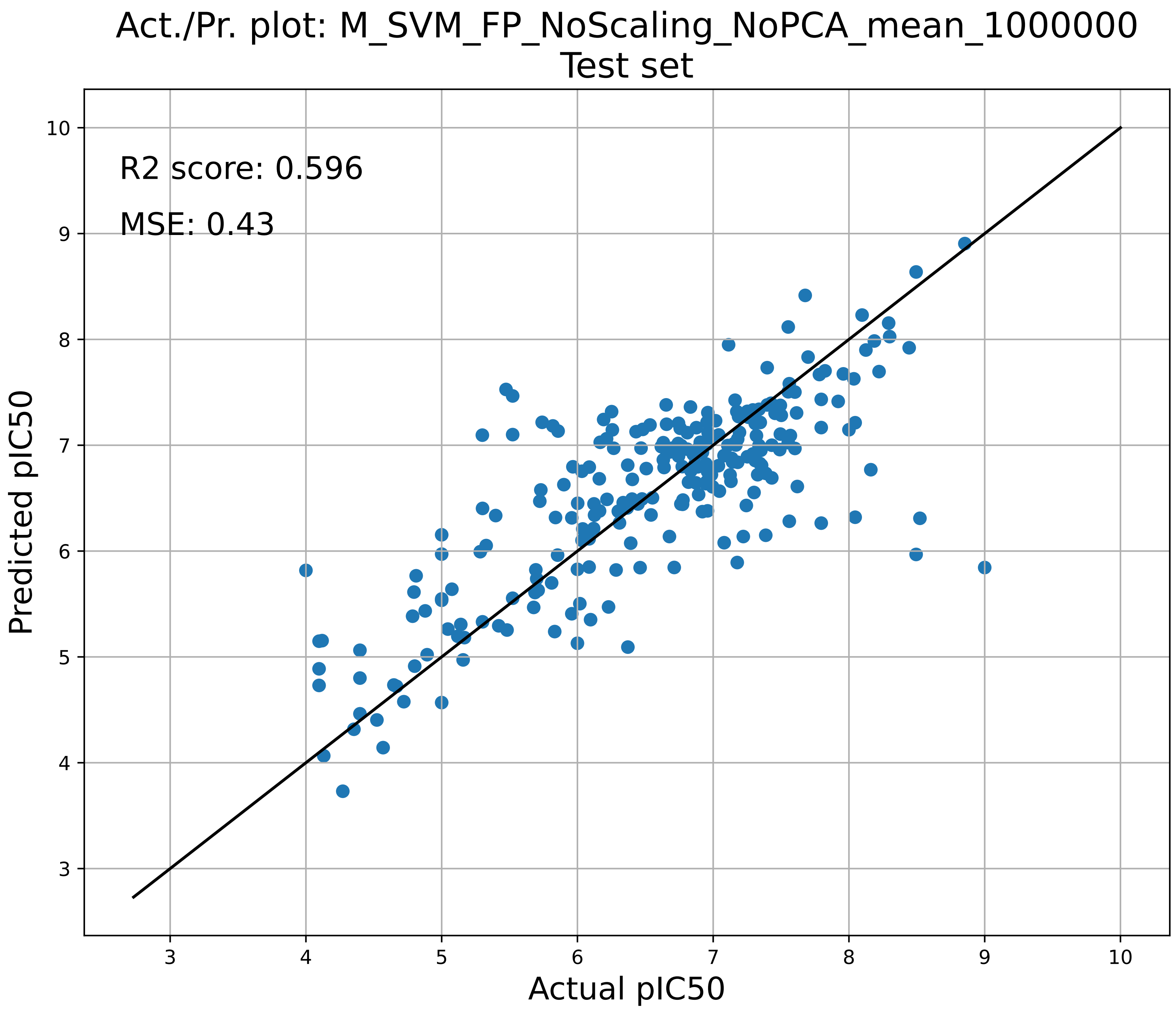
Act./Pr. plot: M_SVM_FP_NoScaling_NoPCA_mean_1000000
Test set
R2 score: 0.596
MSE: 0.43
Predicted pIC50
Actual pIC50
3
4
5
6
7
8
9
10

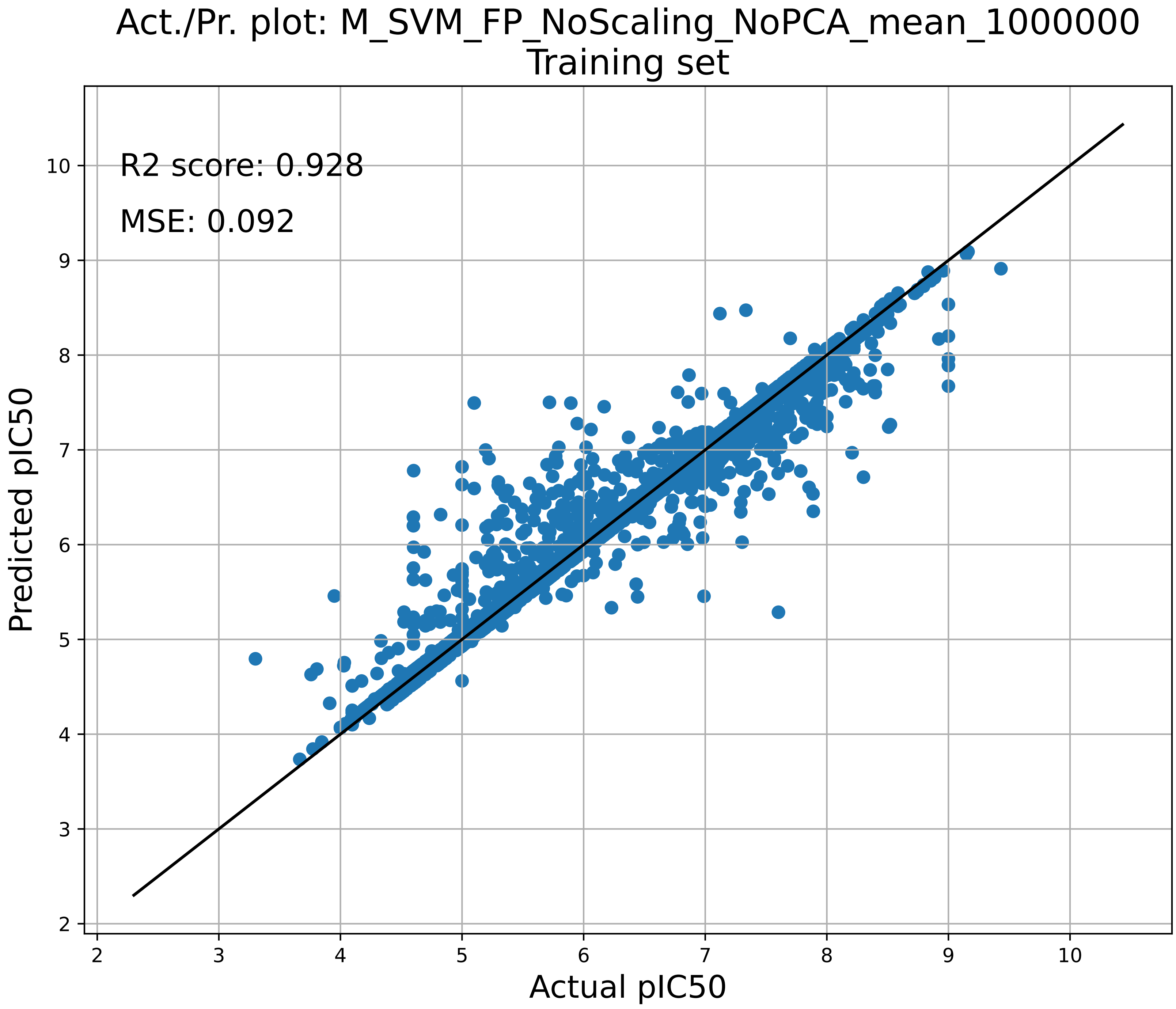

Act./Pr. plot: M_SVM_FP_NoScaling_NoPCA_mean_1000000
Training set
R2 score: 0.928
MSE: 0.092
Predicted pIC50
Actual pIC50
2
3
4
5
6
7
8
9
10

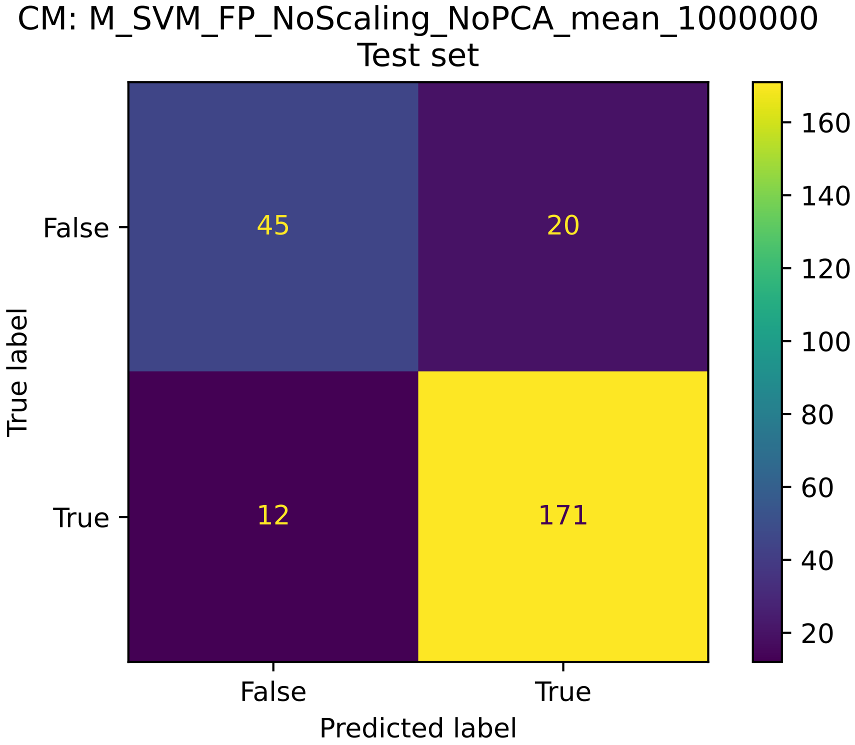

CM: M_SVM_FP_NoScaling_NoPCA_mean_1000000
Test set
True label
Predicted label
False
True
False
True
45
20
12
171
160
140
120
100
80
60
40
20

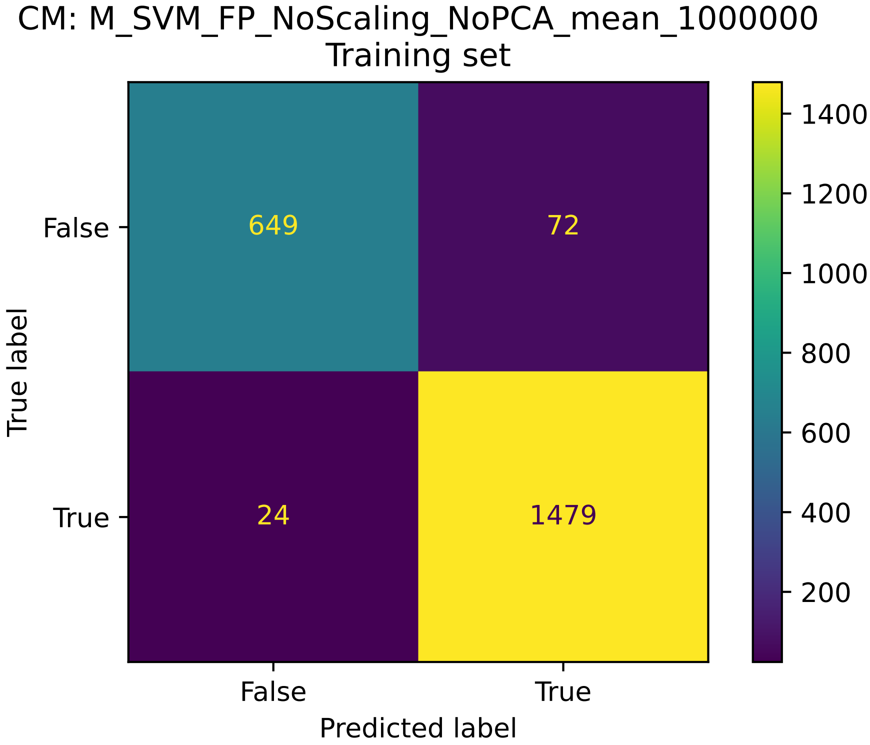
CM: M_SVM_FP_NoScaling_NoPCA_mean_1000000
Training set
True label
False
True
649
72
24
1479
Predicted label
False
True
1400
1200
1000
800
600
400
200

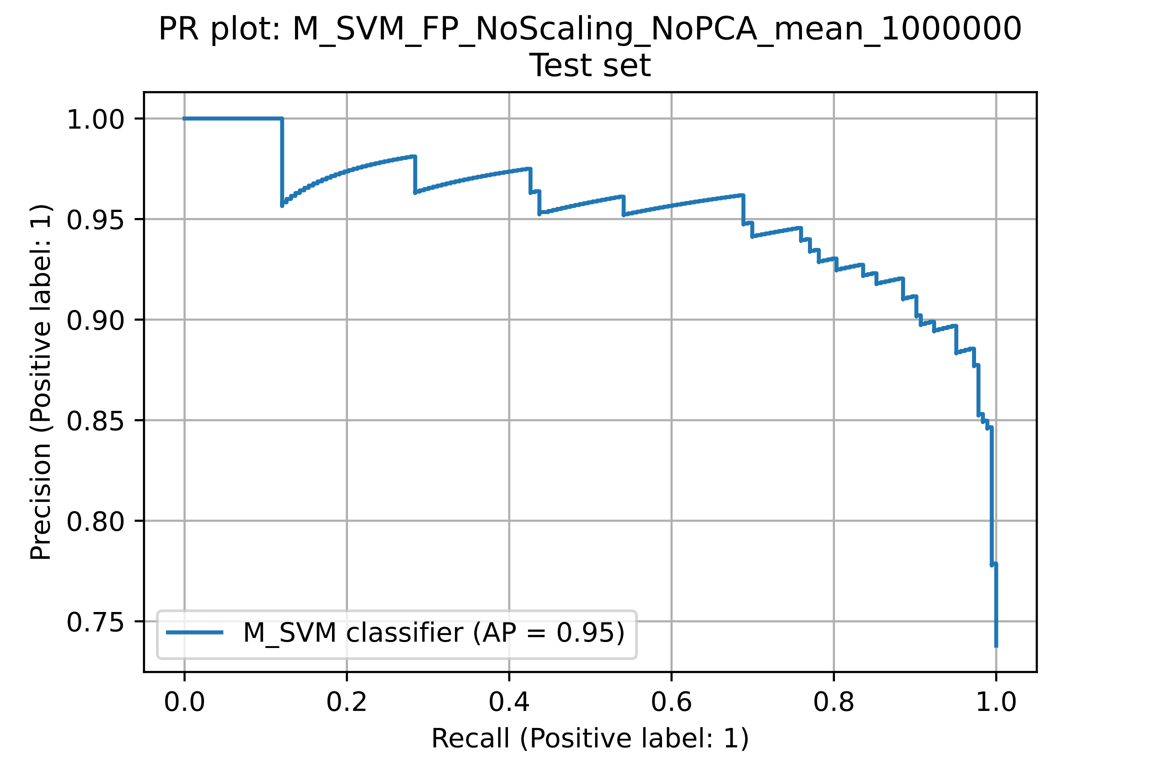
PR plot: M_SVM_FP_NoScaling_NoPCA_mean_1000000
Test set
Precision (Positive label: 1)
Recall (Positive label: 1)
1.00
0.95
0.90
0.85
0.80
0.75
0.0
0.2
0.4
0.6
0.8
1.0
M_SVM classifier (AP = 0.95)

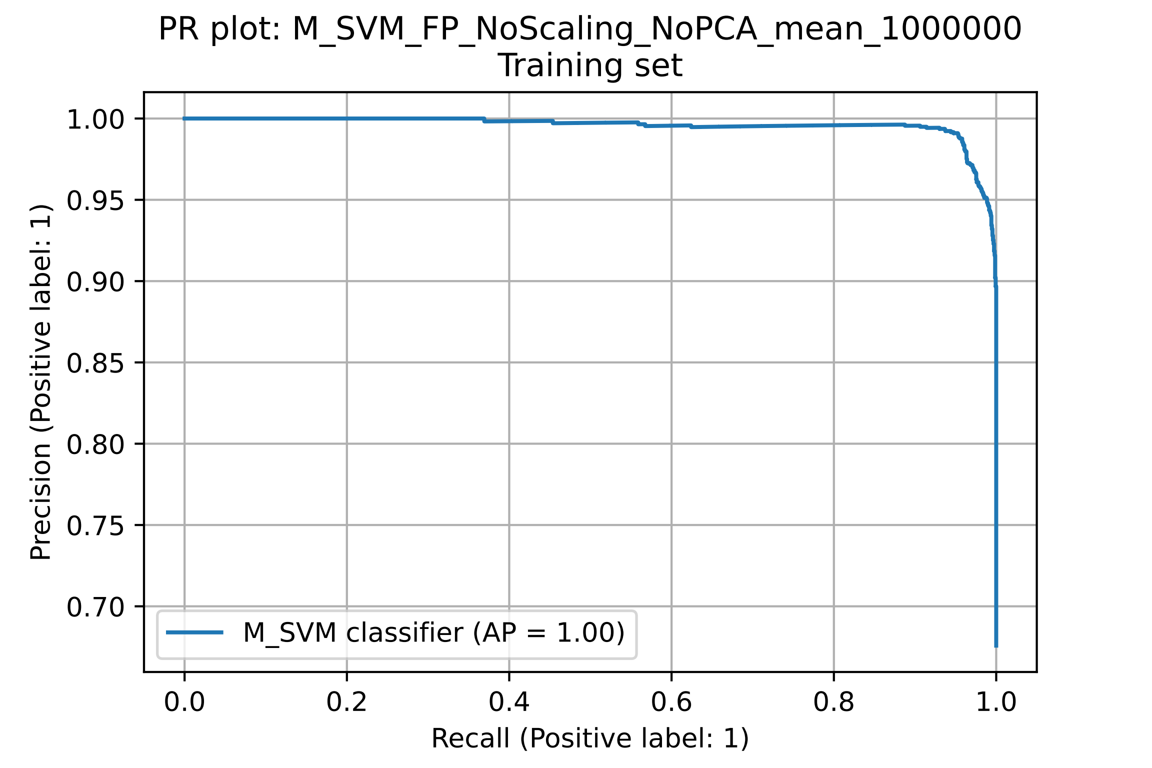

PR plot: M_SVM_FP_NoScaling_NoPCA_mean_1000000
Training set
Precision (Positive label: 1)
Recall (Positive label: 1)
1.00
0.95
0.90
0.85
0.80
0.75
0.70
0.0
0.2
0.4
0.6
0.8
1.0
M_SVM classifier (AP = 1.00)

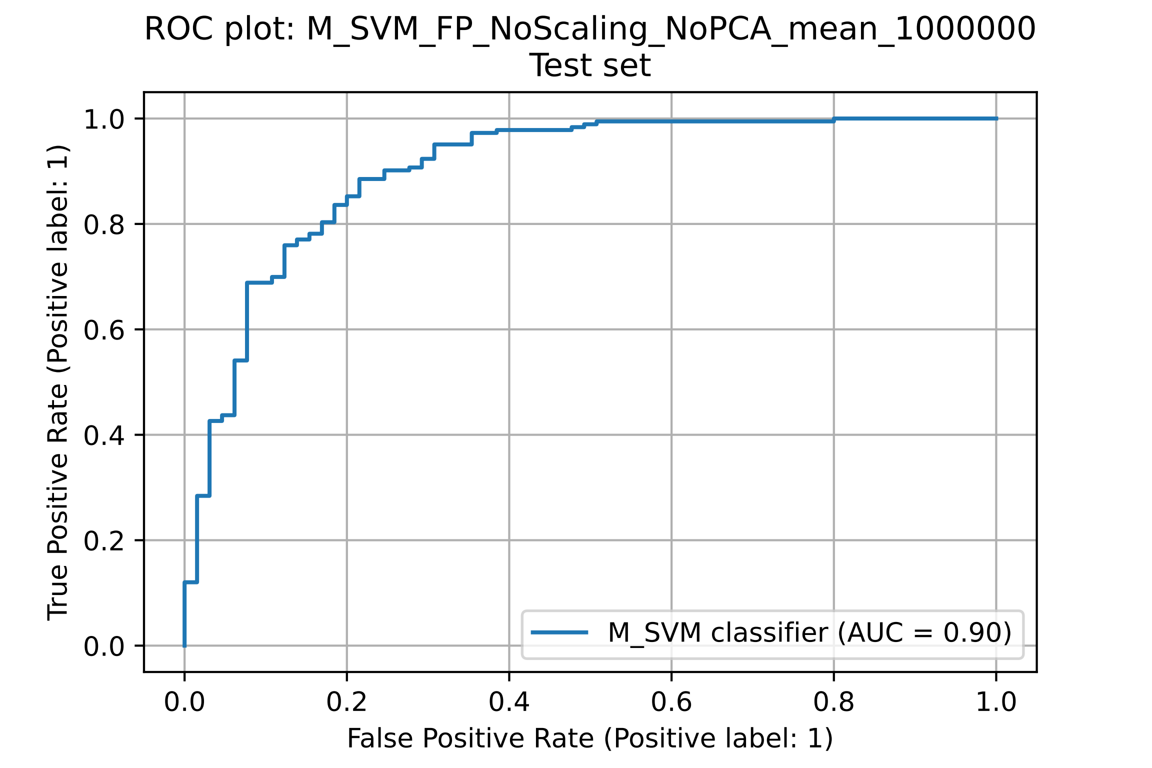
ROC plot: M_SVM_FP_NoScaling_NoPCA_mean_1000000
Test set
True Positive Rate (Positive label: 1)
False Positive Rate (Positive label: 1)
0.0
0.2
0.4
0.6
0.8
1.0
M_SVM classifier (AUC = 0.90)

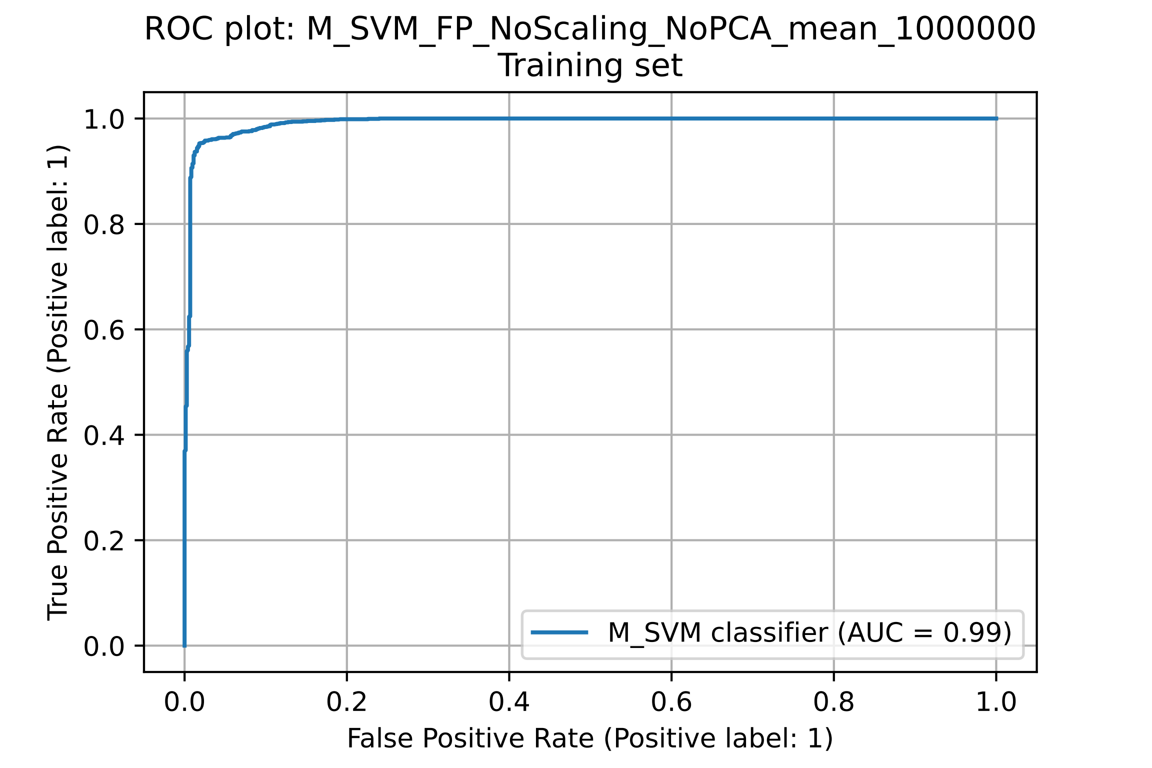
ROC plot: M_SVM_FP_NoScaling_NoPCA_mean_1000000
Training set
True Positive Rate (Positive label: 1)
False Positive Rate (Positive label: 1)
M_SVM classifier (AUC = 0.99)
0.0
0.2
0.4
0.6
0.8
1.0

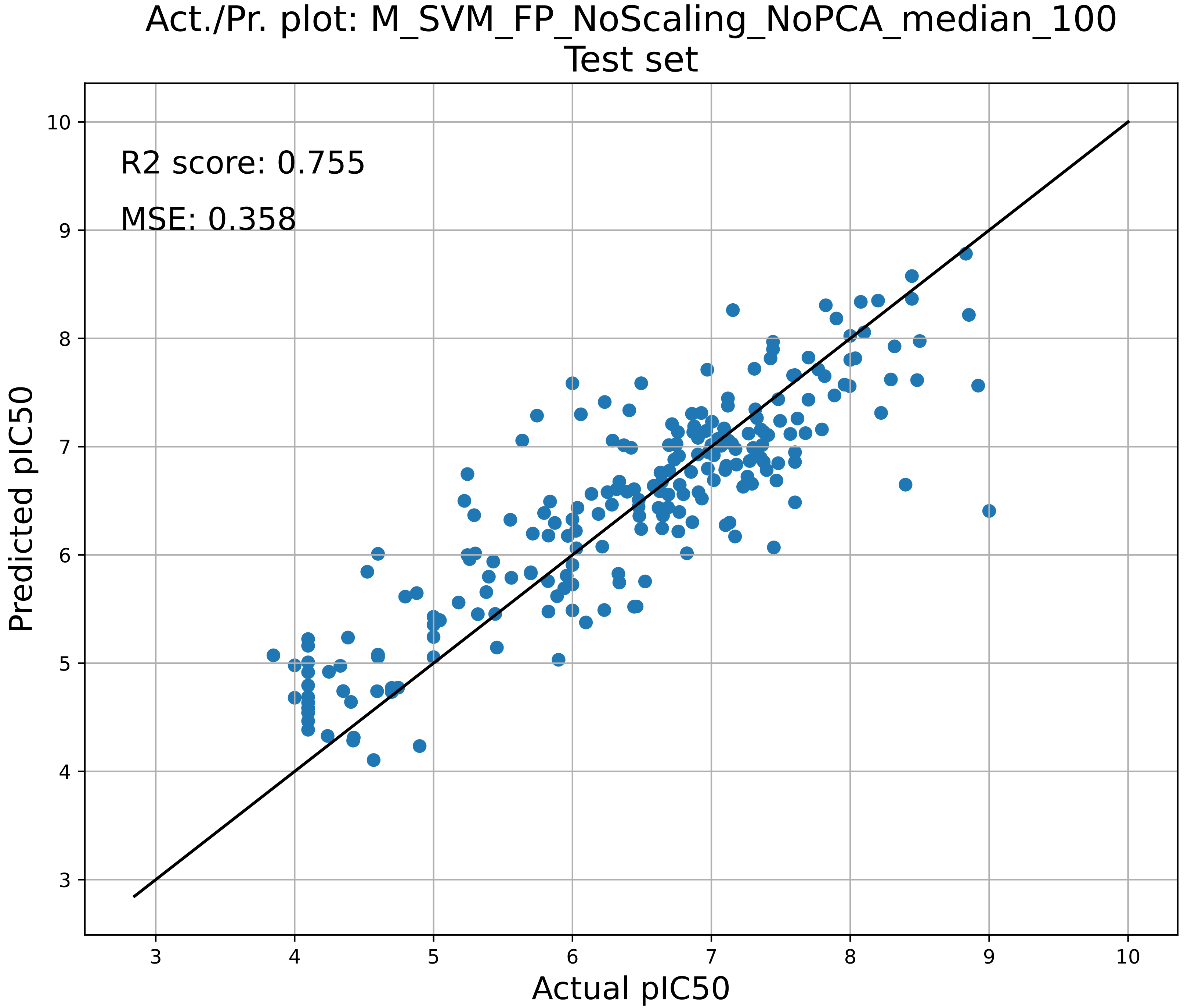
Act./Pr. plot: M_SVM_FP_NoScaling_NoPCA_median_100
Test set
R2 score: 0.755
MSE: 0.358
Predicted pIC50
Actual pIC50
3
4
5
6
7
8
9
10

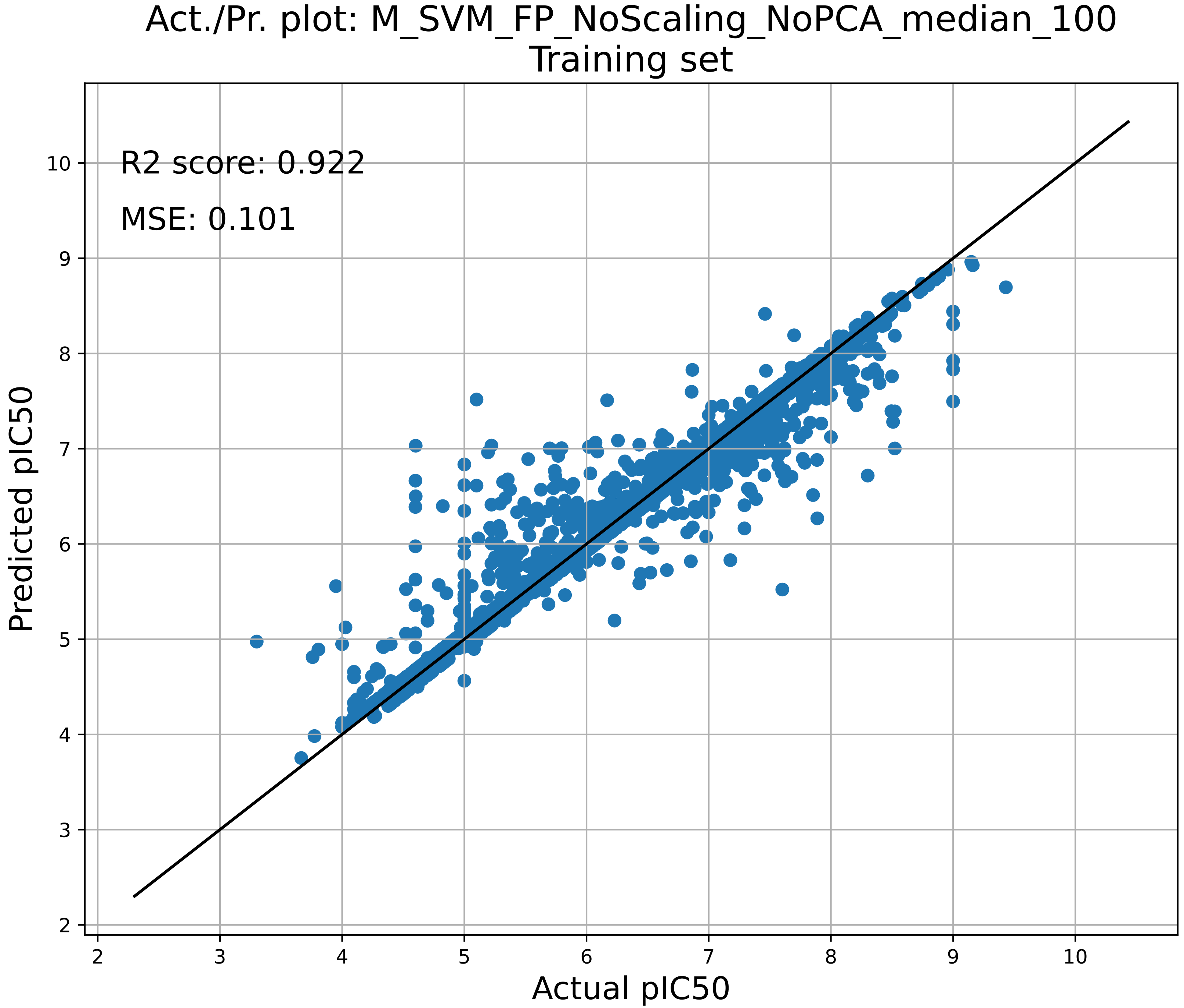

Act./Pr. plot: M_SVM_FP_NoScaling_NoPCA_median_100
Training set
R2 score: 0.922
MSE: 0.101
Predicted pIC50
Actual pIC50
2
3
4
5
6
7
8
9
10

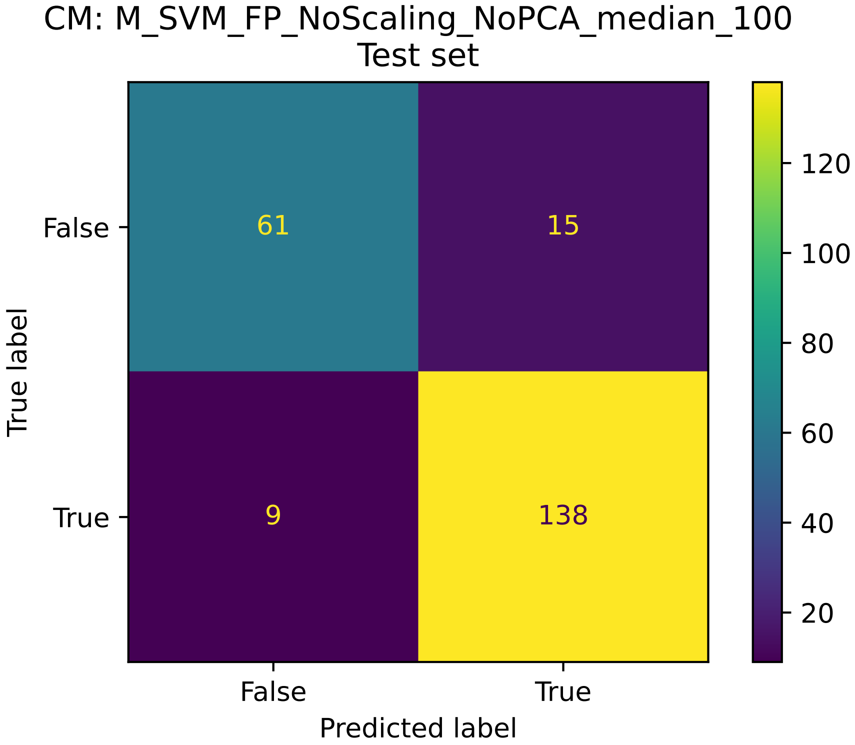

CM: M_SVM_FP_NoScaling_NoPCA_median_100
Test set
True label
Predicted label
False
True
False
True
61
15
9
138
20
40
60
80
100
120

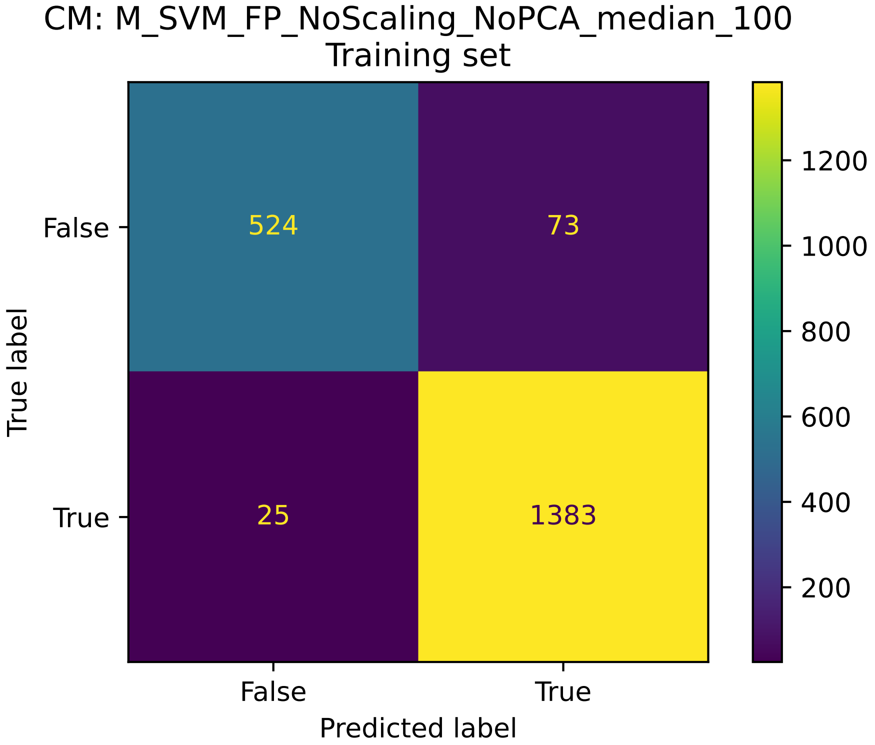
CM: M_SVM_FP_NoScaling_NoPCA_median_100
Training set
True label
False
True
524
73
25
1383
Predicted label
False
True
1200
1000
800
600
400
200

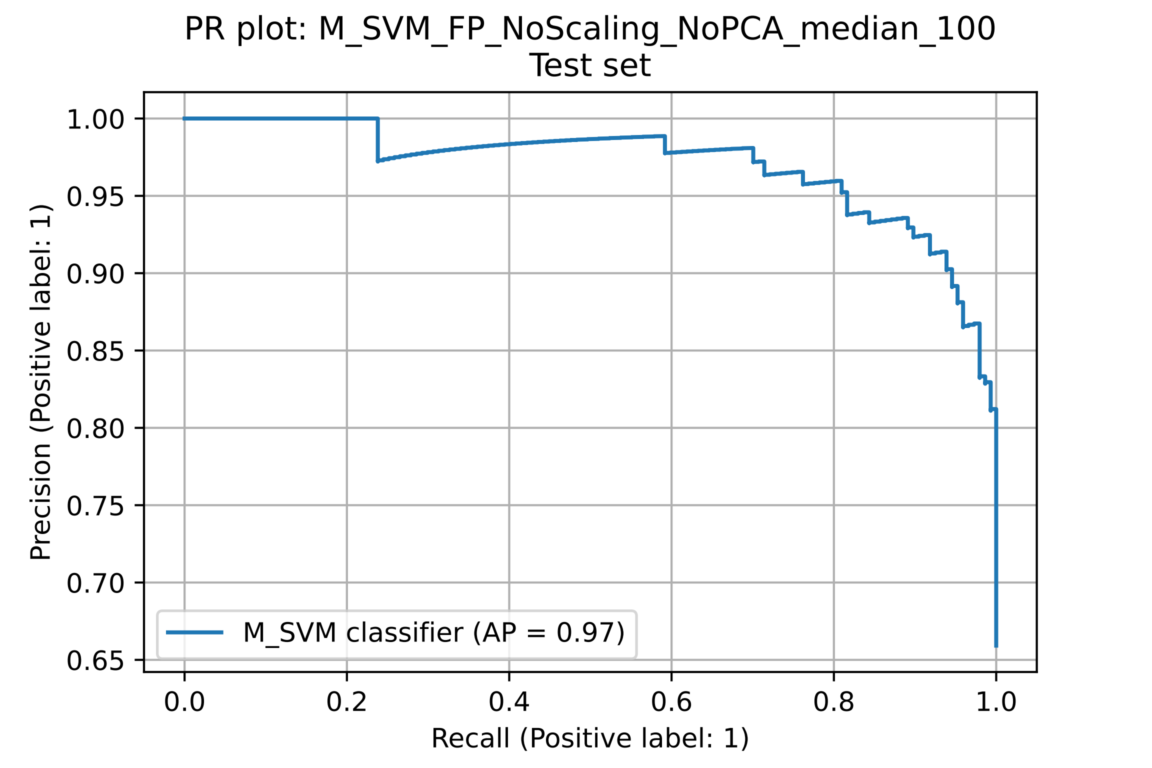
PR plot: M_SVM_FP_NoScaling_NoPCA_median_100
Test set
Precision (Positive label: 1)
Recall (Positive label: 1)
1.00
0.95
0.90
0.85
0.80
0.75
0.70
0.65
0.0
0.2
0.4
0.6
0.8
1.0
M_SVM classifier (AP = 0.97)

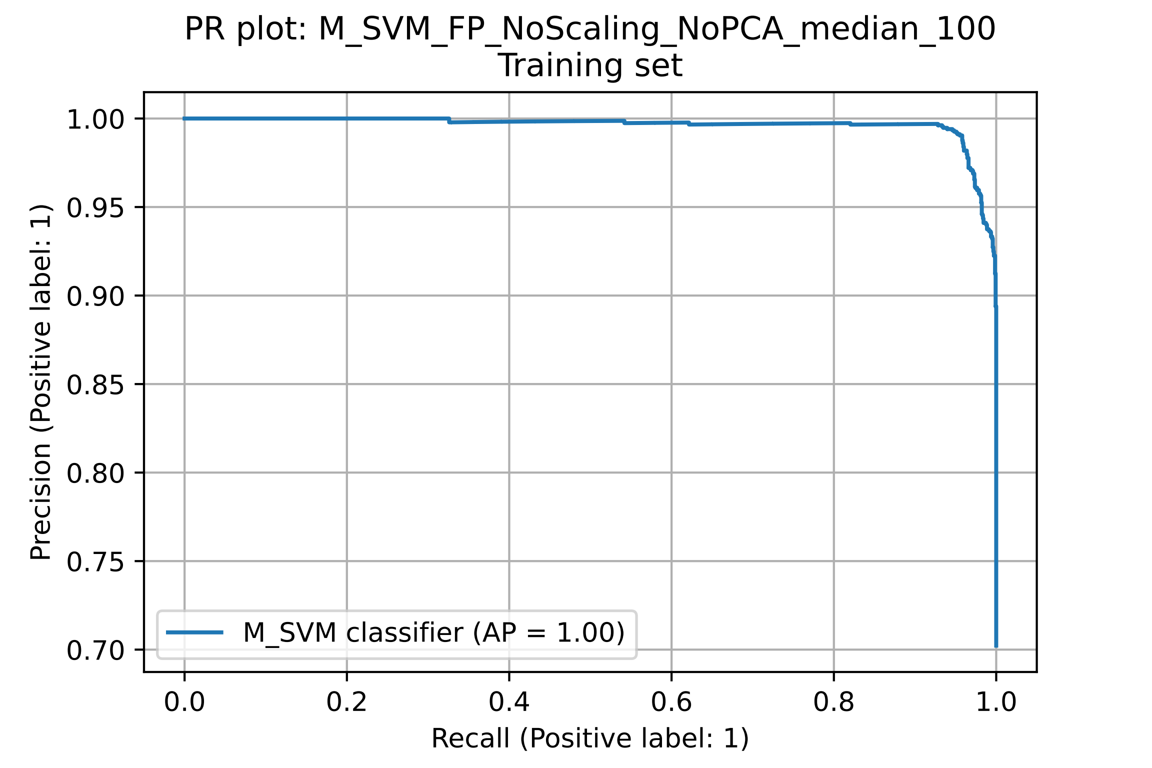
PR plot: M_SVM_FP_NoScaling_NoPCA_median_100
Training set
1.00
0.95
0.90
0.85
0.80
0.75
0.70
Precision (Positive label: 1)
0.0
0.2
0.4
0.6
0.8
1.0
Recall (Positive label: 1)
M_SVM classifier (AP = 1.00)

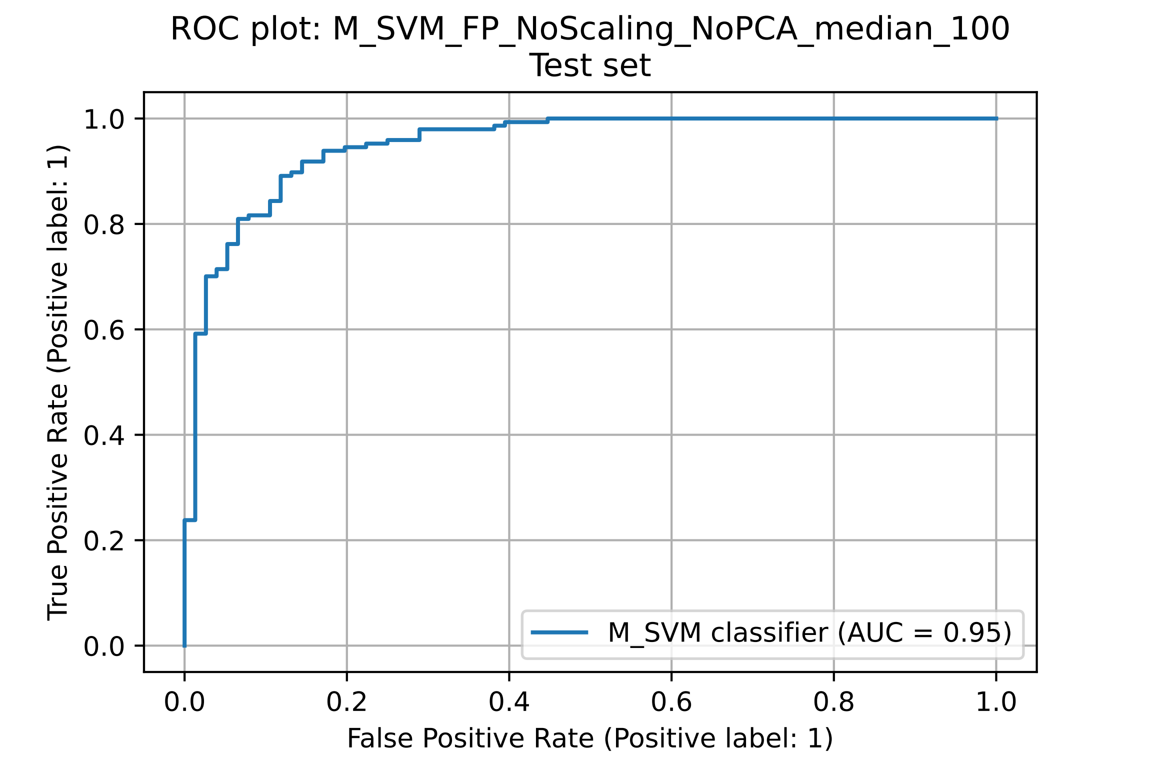
ROC plot: M_SVM_FP_NoScaling_NoPCA_median_100
Test set
True Positive Rate (Positive label: 1)
False Positive Rate (Positive label: 1)
0.0
0.2
0.4
0.6
0.8
1.0
M_SVM classifier (AUC = 0.95)

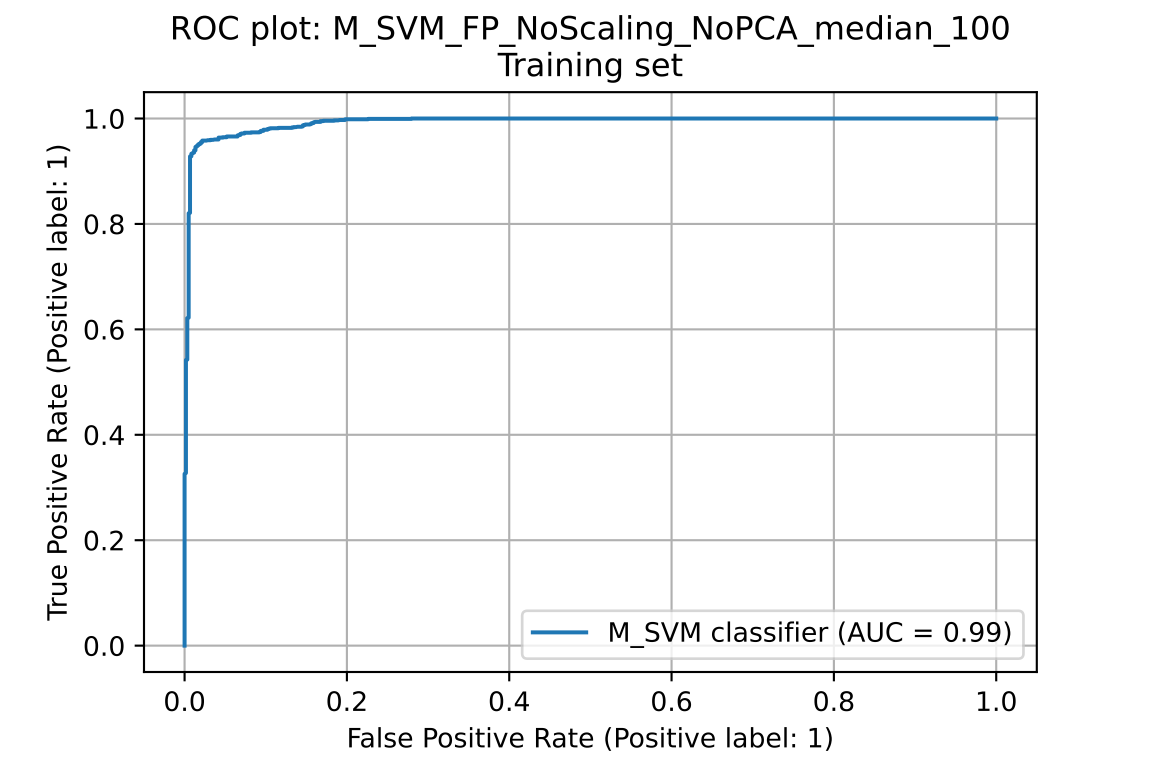
ROC plot: M_SVM_FP_NoScaling_NoPCA_median_100
Training set
True Positive Rate (Positive label: 1)
False Positive Rate (Positive label: 1)
0.0
0.2
0.4
0.6
0.8
1.0
M_SVM classifier (AUC = 0.99)

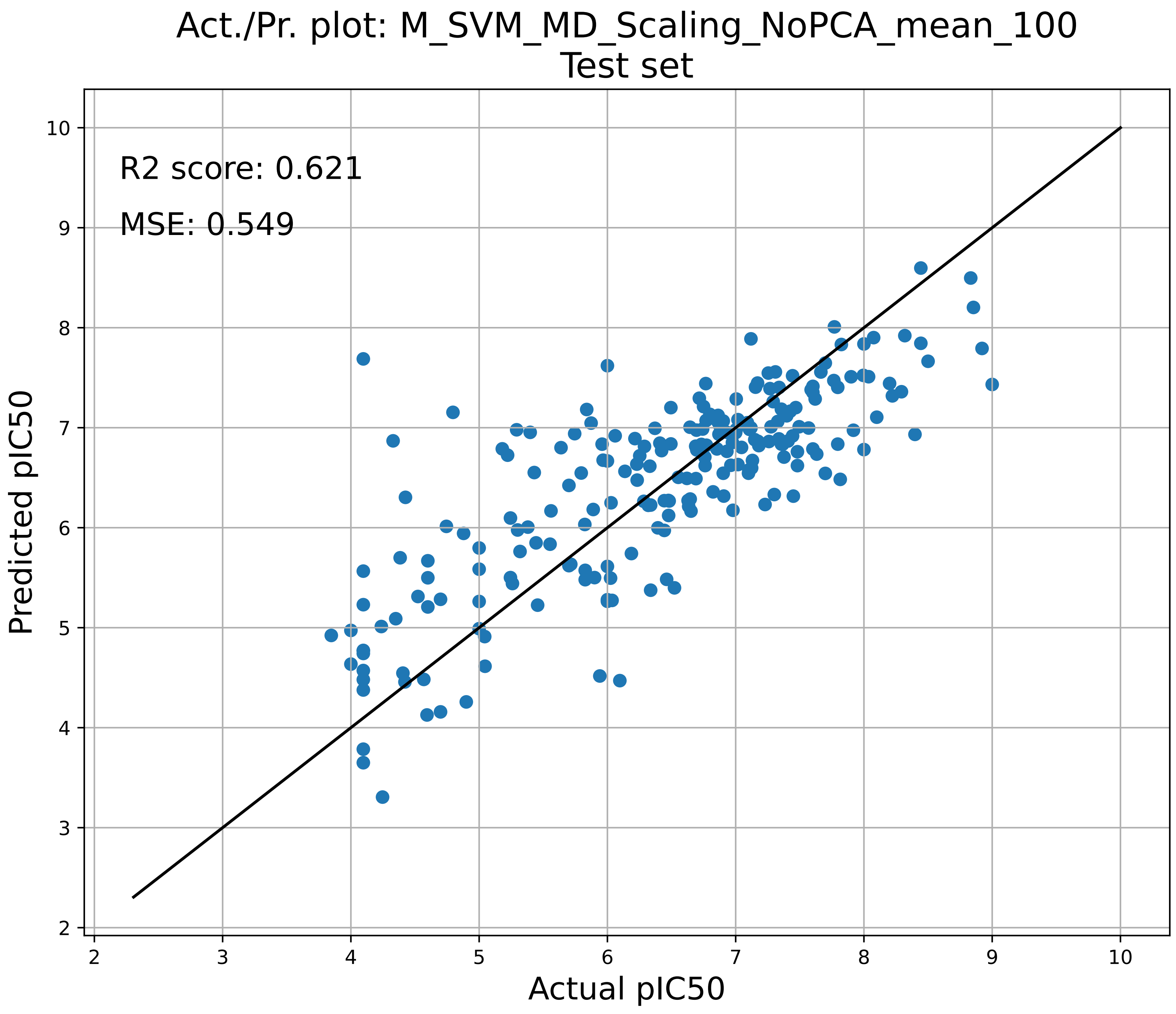

Act./Pr. plot: M_SVM_MD_Scaling_NoPCA_mean_100
Test set
R2 score: 0.621
MSE: 0.549
Predicted pIC50
Actual pIC50
2
3
4
5
6
7
8
9
10

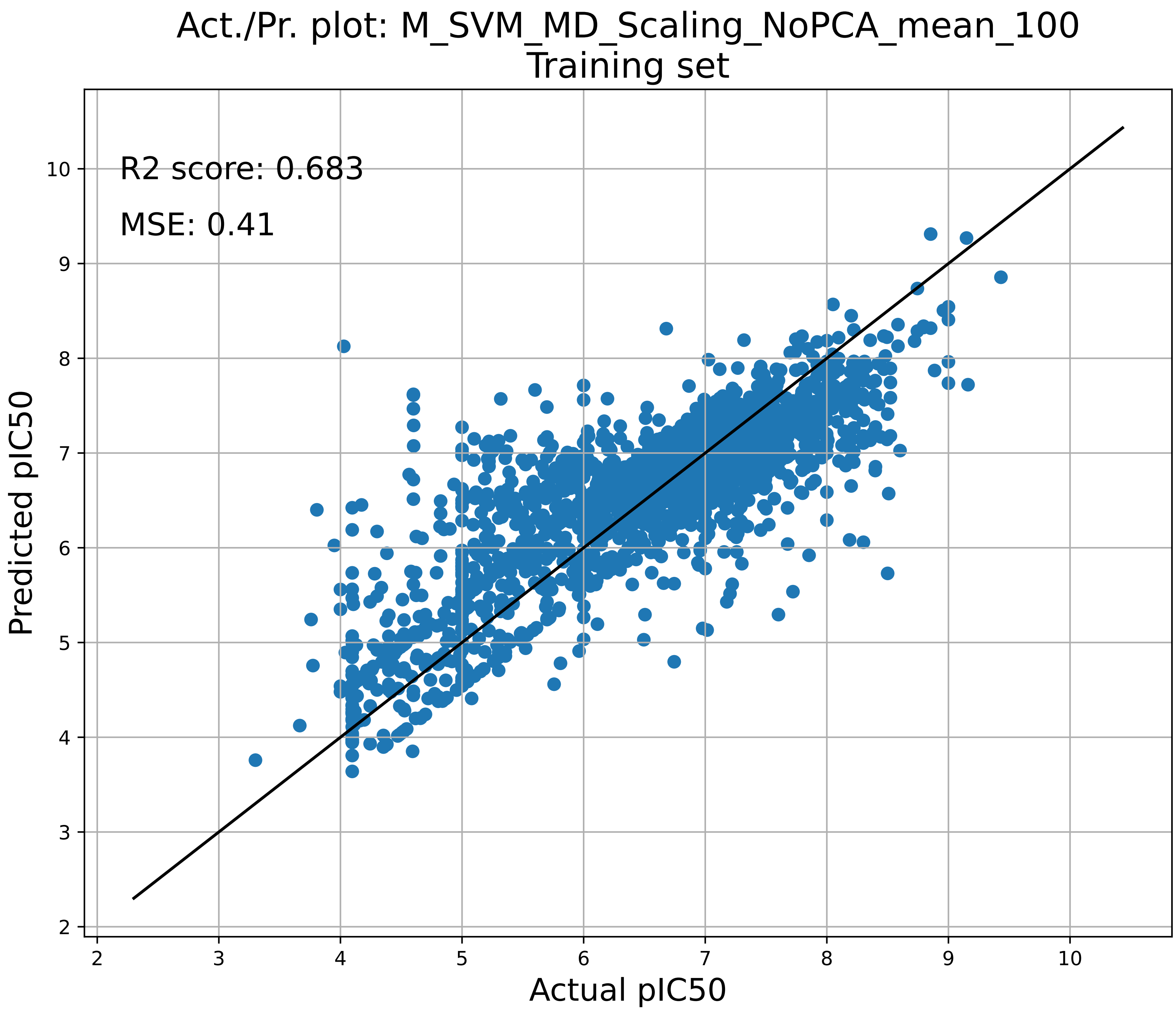
Act./Pr. plot: M_SVM_MD_Scaling_NoPCA_mean_100
Training set
R2 score: 0.683
MSE: 0.41
Predicted pIC50
Actual pIC50
2
3
4
5
6
7
8
9
10

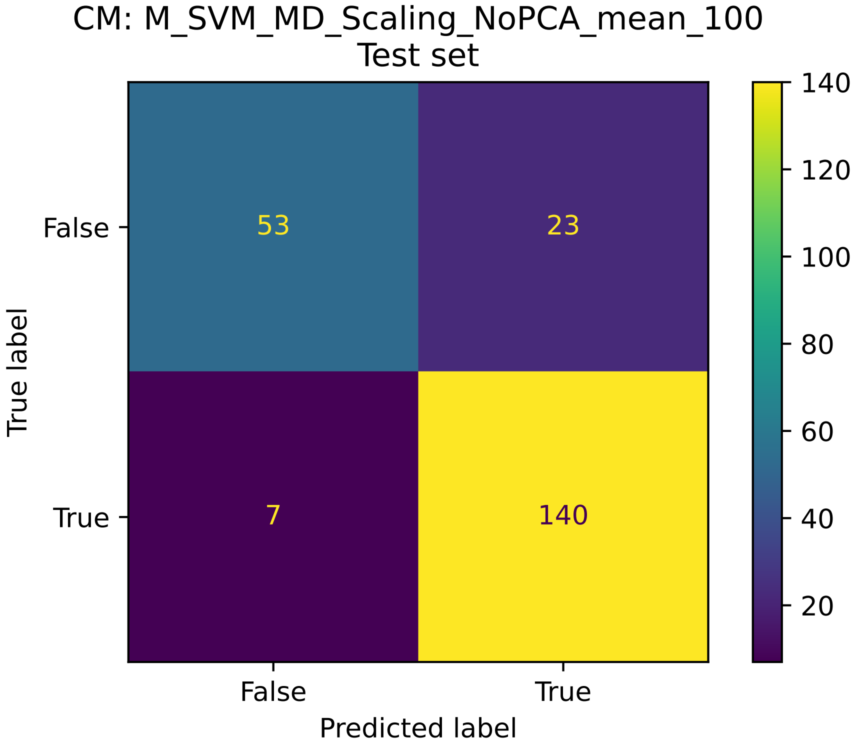
CM: M_SVM_MD_Scaling_NoPCA_mean_100
Test set
True label
False
True
53
23
7
140
False
True
Predicted label
140
120
100
80
60
40
20

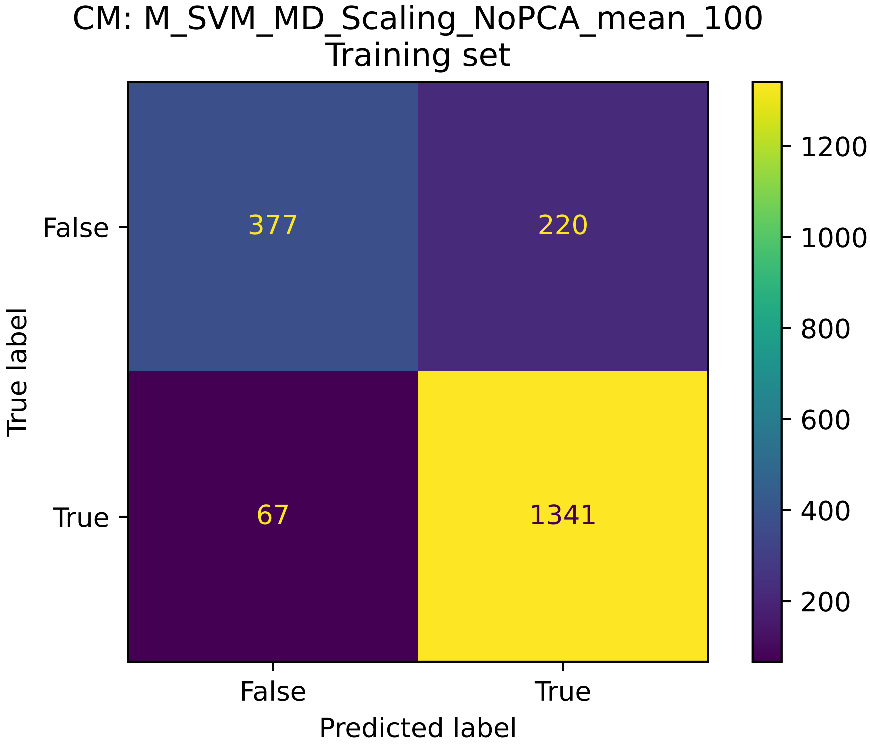
CM: M_SVM_MD_Scaling_NoPCA_mean_100
Training set
True label
Predicted label
False
True
False
True
377
220
67
1341
1200
1000
800
600
400
200

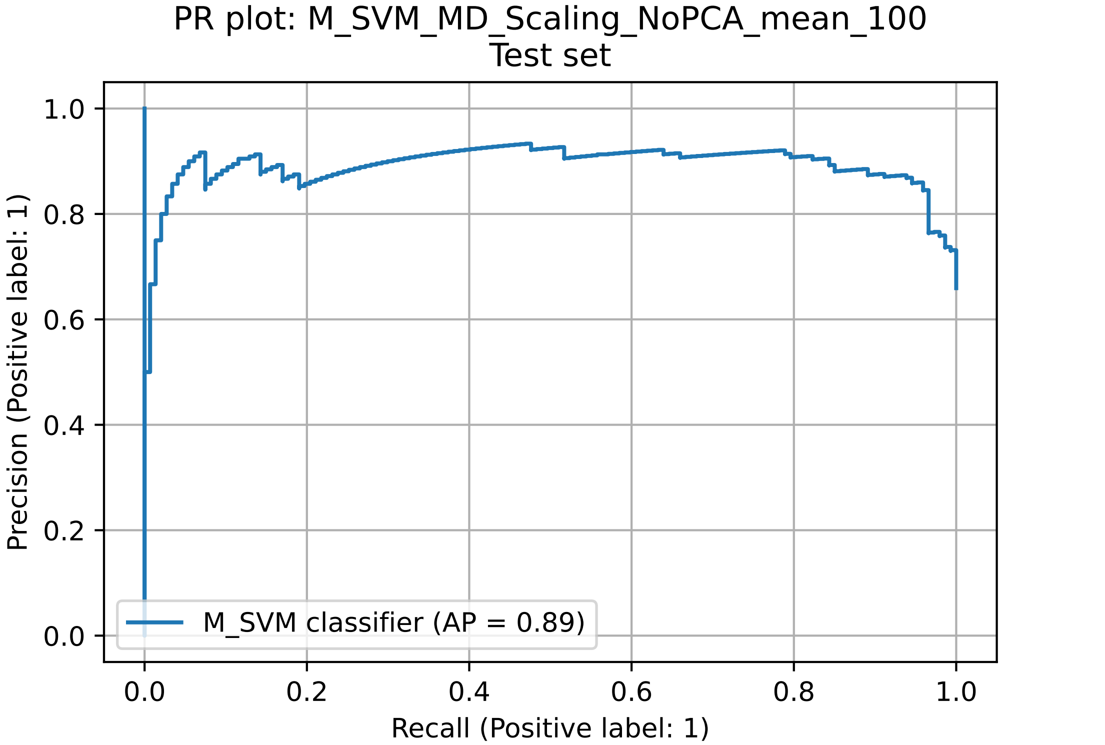
PR plot: M_SVM_MD_Scaling_NoPCA_mean_100
Test set
Precision (Positive label: 1)
Recall (Positive label: 1)
0.0
0.2
0.4
0.6
0.8
1.0
M_SVM classifier (AP = 0.89)

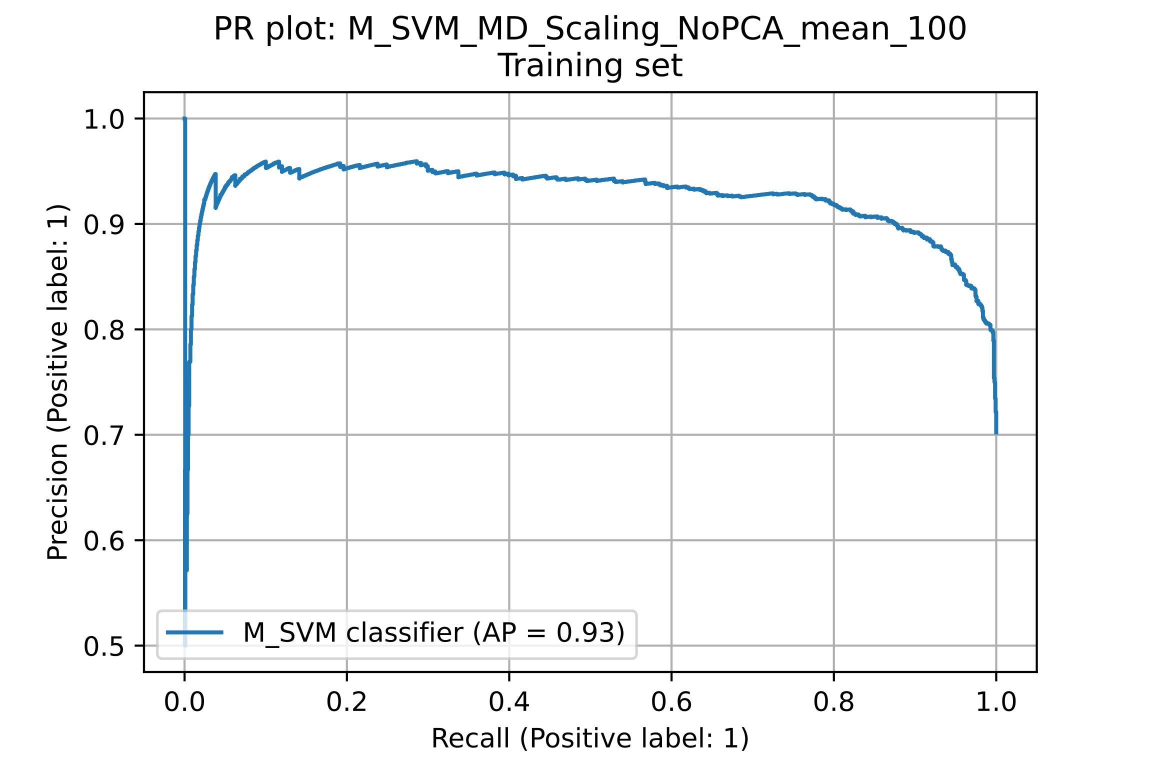

PR plot: M_SVM_MD_Scaling_NoPCA_mean_100
Training set
1.0
0.9
0.8
0.7
0.6
0.5
Precision (Positive label: 1)
0.0
0.2
0.4
0.6
0.8
1.0
Recall (Positive label: 1)
M_SVM classifier (AP = 0.93)

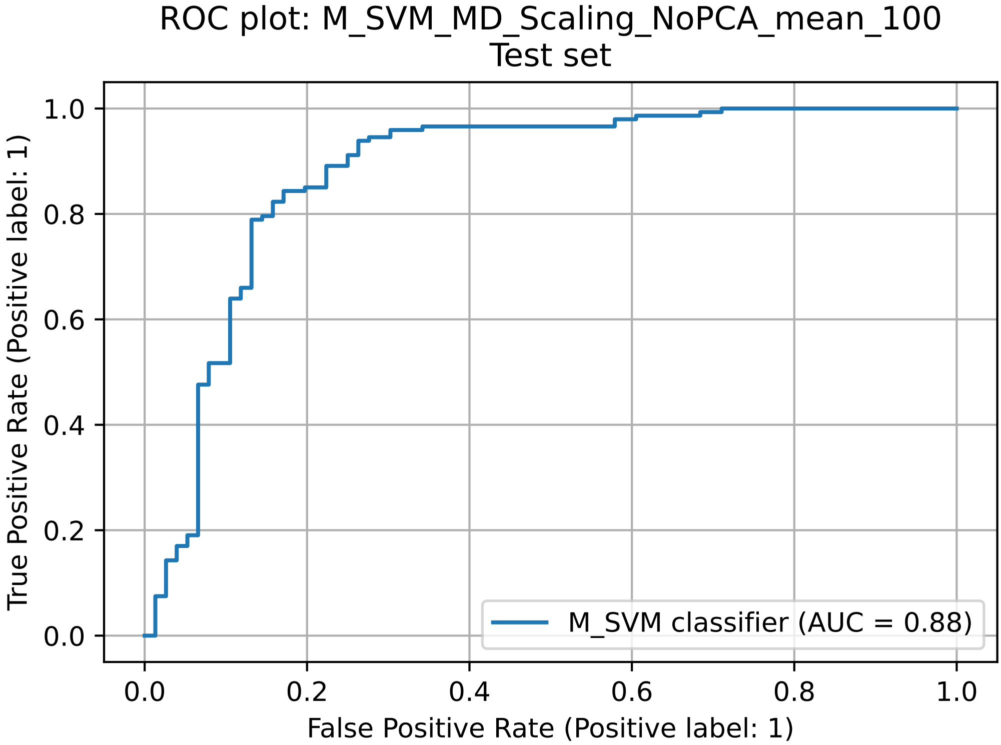
ROC plot: M_SVM_MD_Scaling_NoPCA_mean_100
Test set
True Positive Rate (Positive label: 1)
False Positive Rate (Positive label: 1)
0.0
0.2
0.4
0.6
0.8
1.0
M_SVM classifier (AUC = 0.88)

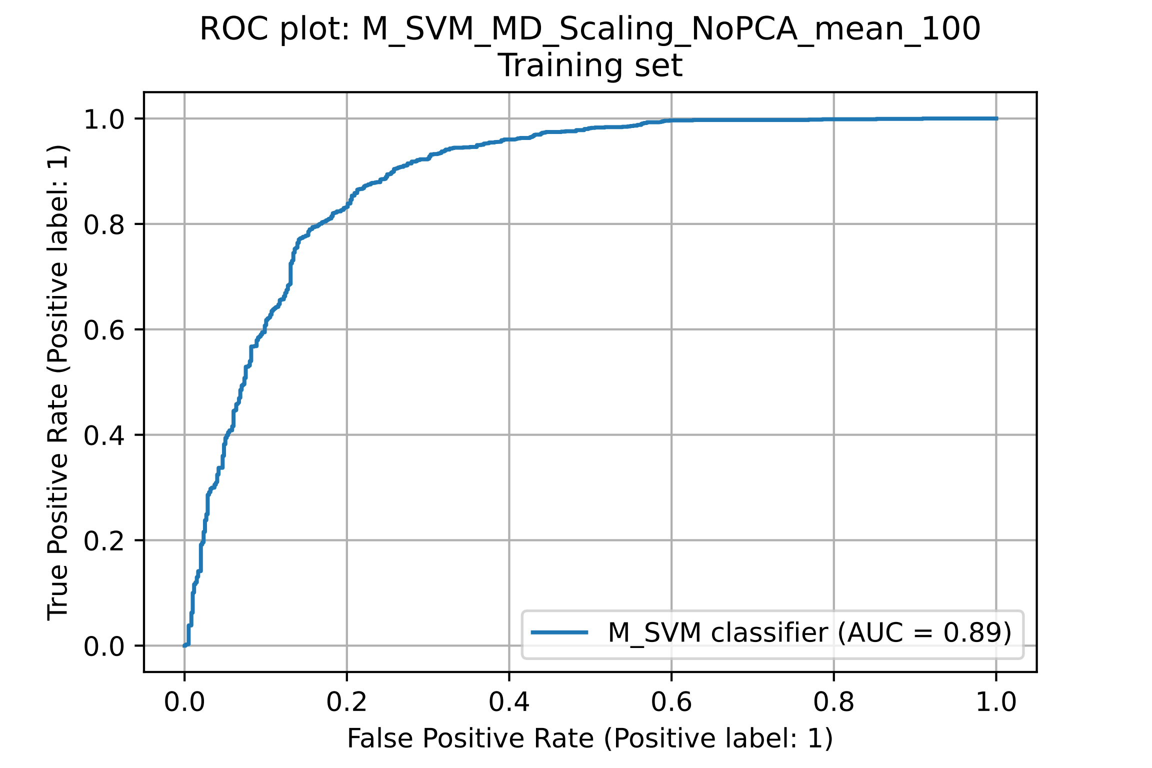
ROC plot: M_SVM_MD_Scaling_NoPCA_mean_100
Training set
True Positive Rate (Positive label: 1)
False Positive Rate (Positive label: 1)
0.0
0.2
0.4
0.6
0.8
1.0
M_SVM classifier (AUC = 0.89)

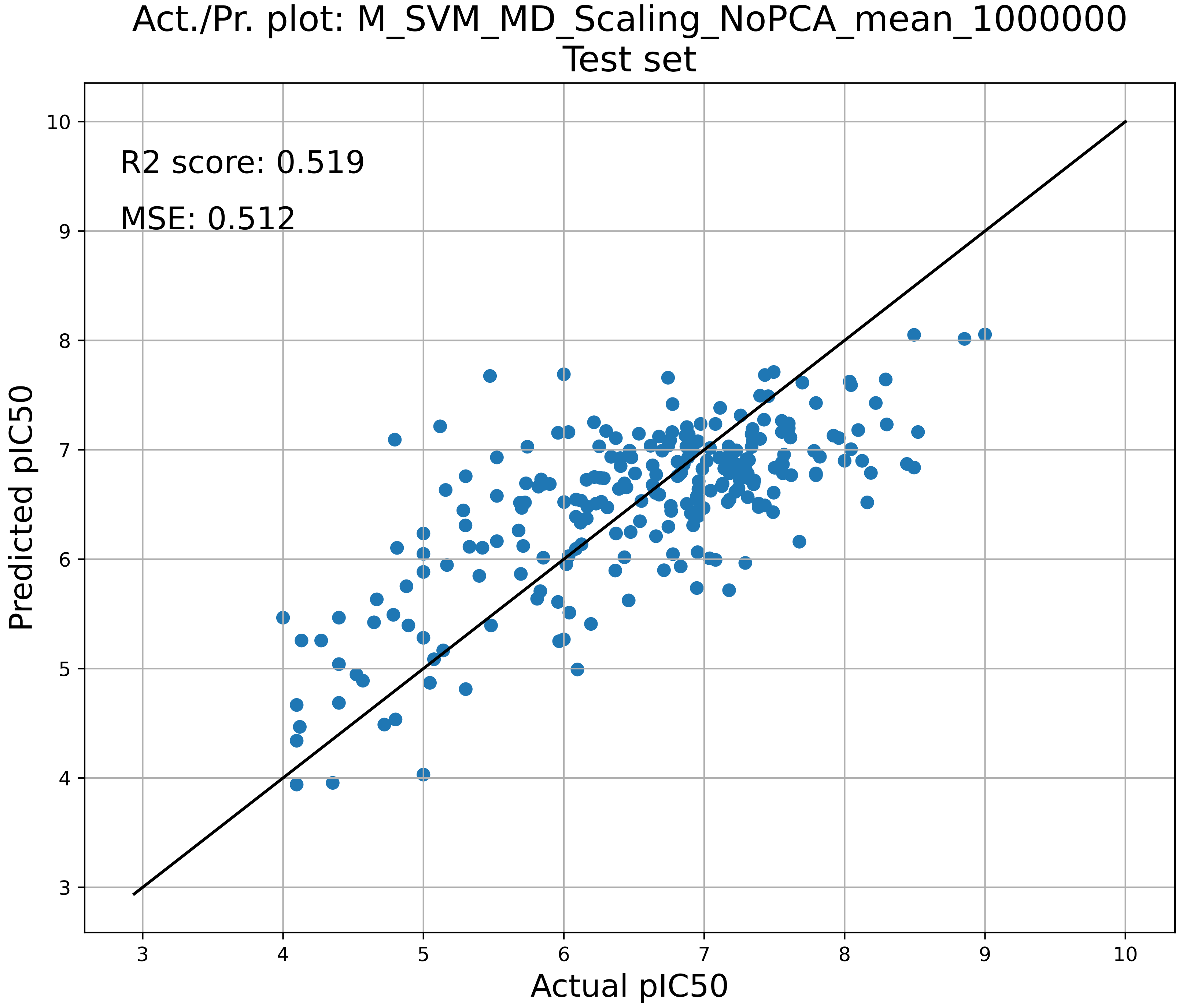
Act./Pr. plot: M_SVM_MD_Scaling_NoPCA_mean_1000000
Test set
R2 score: 0.519
MSE: 0.512
Predicted pIC50
Actual pIC50
3
4
5
6
7
8
9
10

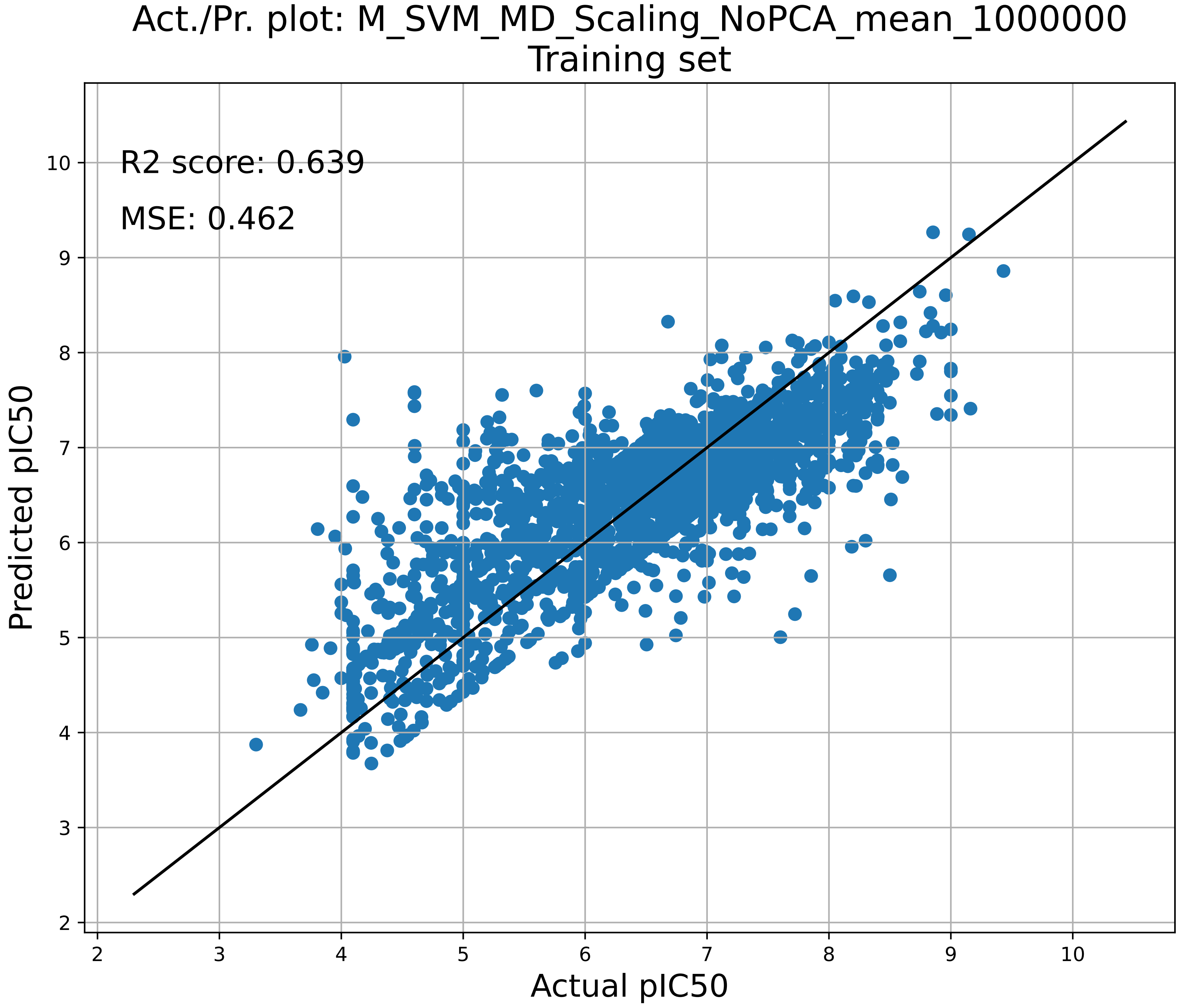
Act./Pr. plot: M_SVM_MD_Scaling_NoPCA_mean_1000000
Training set
R2 score: 0.639
MSE: 0.462
Predicted pIC50
Actual pIC50
2
3
4
5
6
7
8
9
10

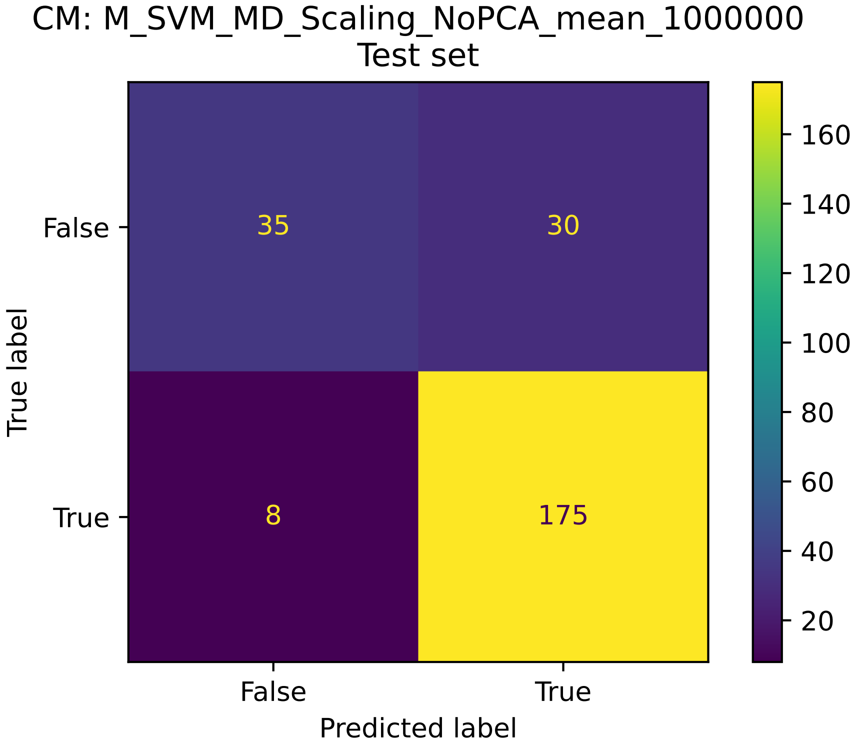
CM: M_SVM_MD_Scaling_NoPCA_mean_1000000
Test set
True label
Predicted label
False
True
False
True
35
30
8
175
160
140
120
100
80
60
40
20

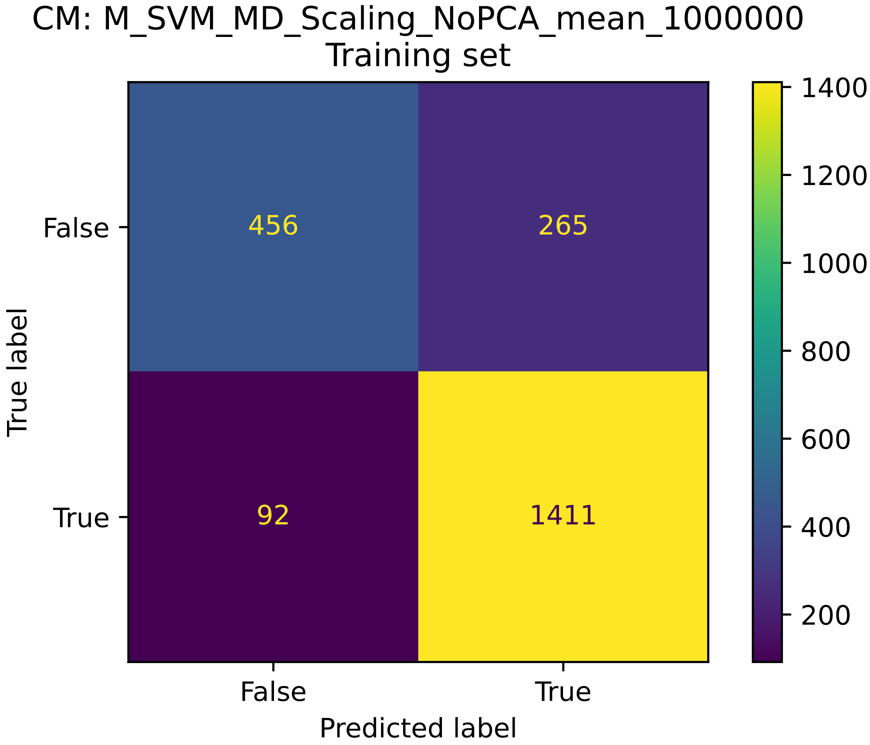
CM: M_SVM_MD_Scaling_NoPCA_mean_1000000
Training set
True label
False
True
456
265
92
1411
Predicted label
False
True
1400
1200
1000
800
600
400
200

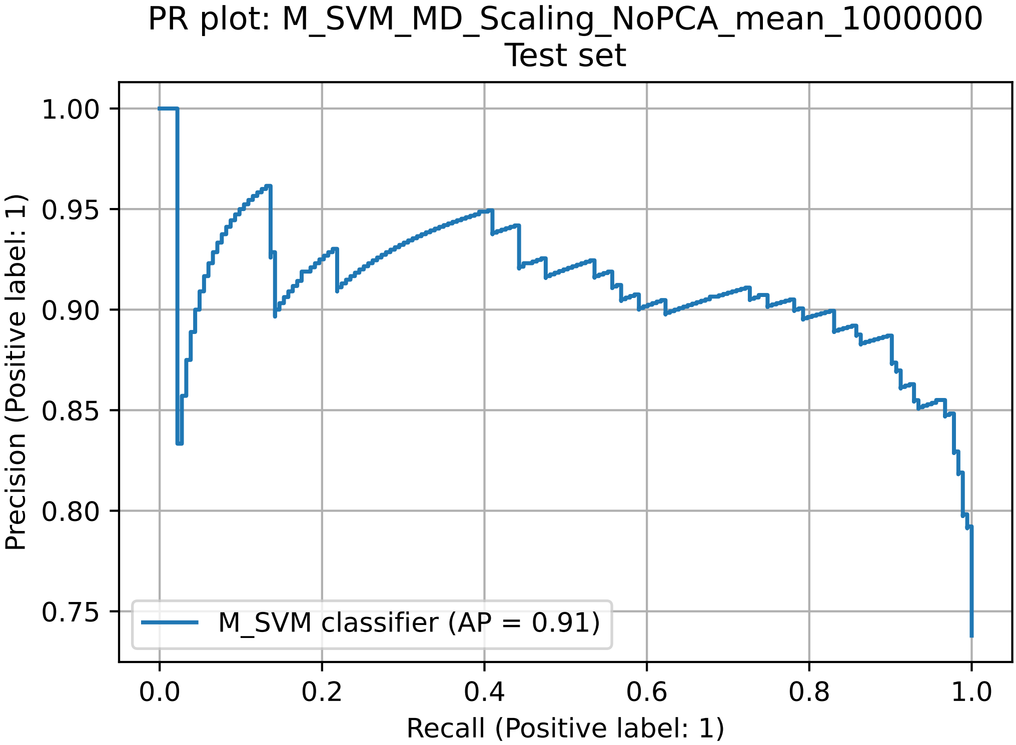
PR plot: M_SVM_MD_Scaling_NoPCA_mean_1000000
Test set
Precision (Positive label: 1)
Recall (Positive label: 1)
1.00
0.95
0.90
0.85
0.80
0.75
0.0
0.2
0.4
0.6
0.8
1.0
M_SVM classifier (AP = 0.91)

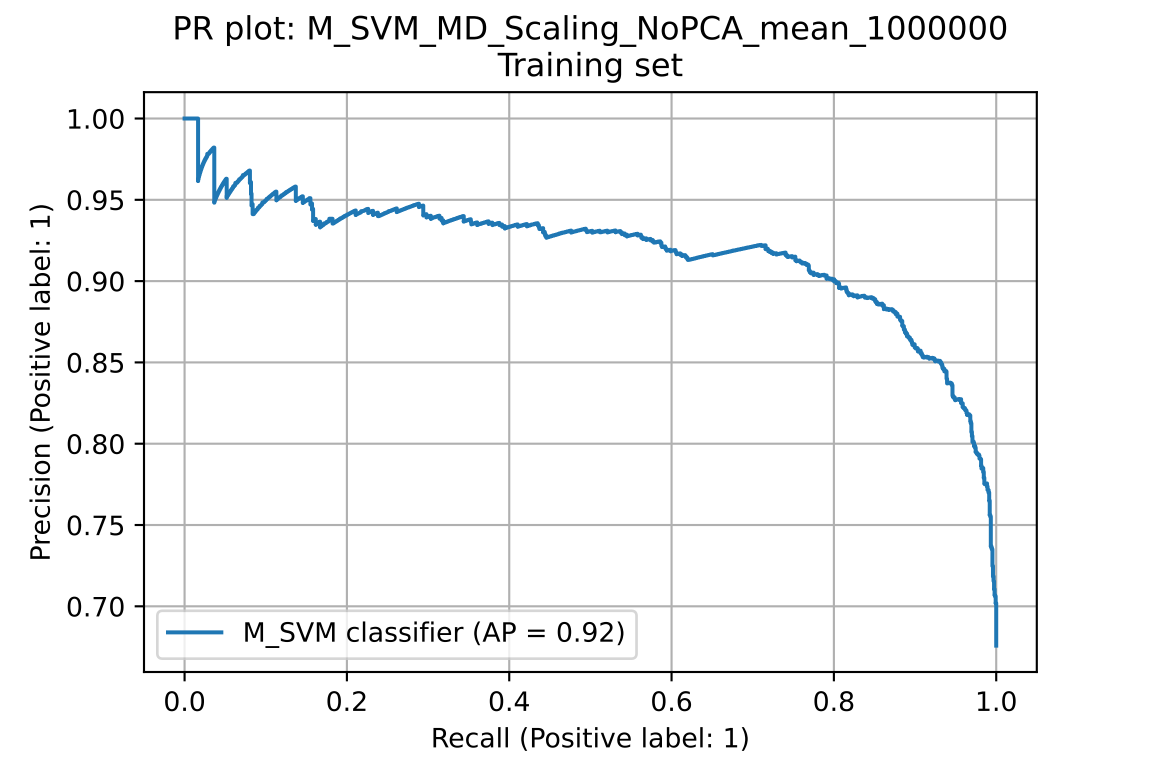
PR plot: M_SVM_MD_Scaling_NoPCA_mean_1000000
Training set
Precision (Positive label: 1)
Recall (Positive label: 1)
1.00
0.95
0.90
0.85
0.80
0.75
0.70
0.0
0.2
0.4
0.6
0.8
1.0
M_SVM classifier (AP = 0.92)

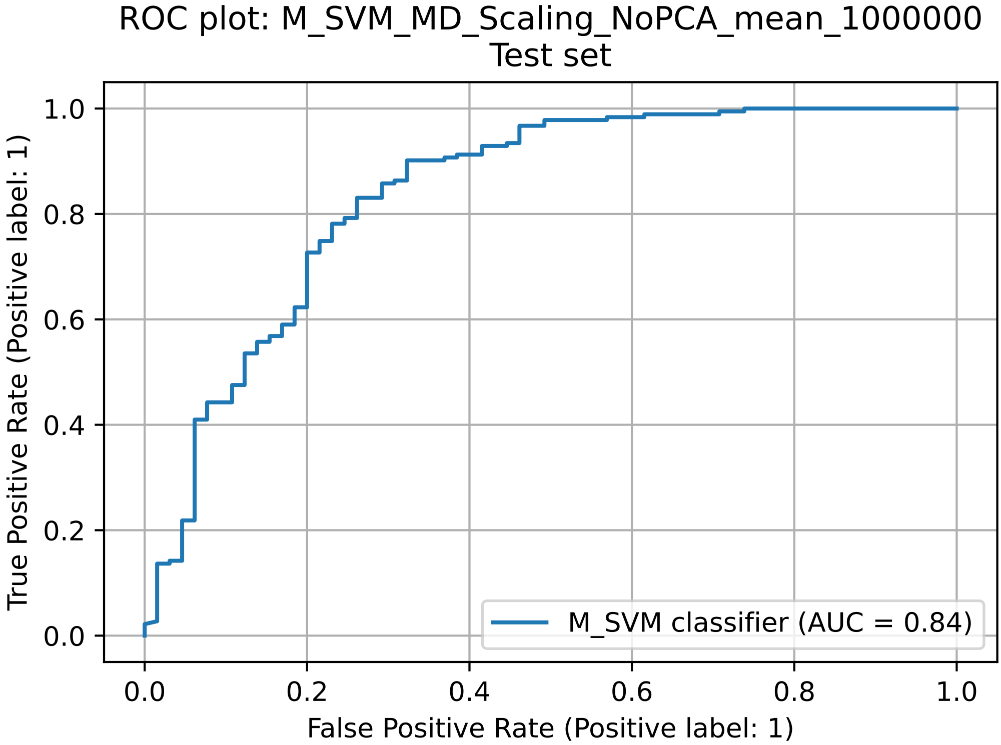

ROC plot: M_SVM_MD_Scaling_NoPCA_mean_1000000
Test set
True Positive Rate (Positive label: 1)
False Positive Rate (Positive label: 1)
1.0
0.8
0.6
0.4
0.2
0.0
0.0
0.2
0.4
0.6
0.8
1.0
M_SVM classifier (AUC = 0.84)

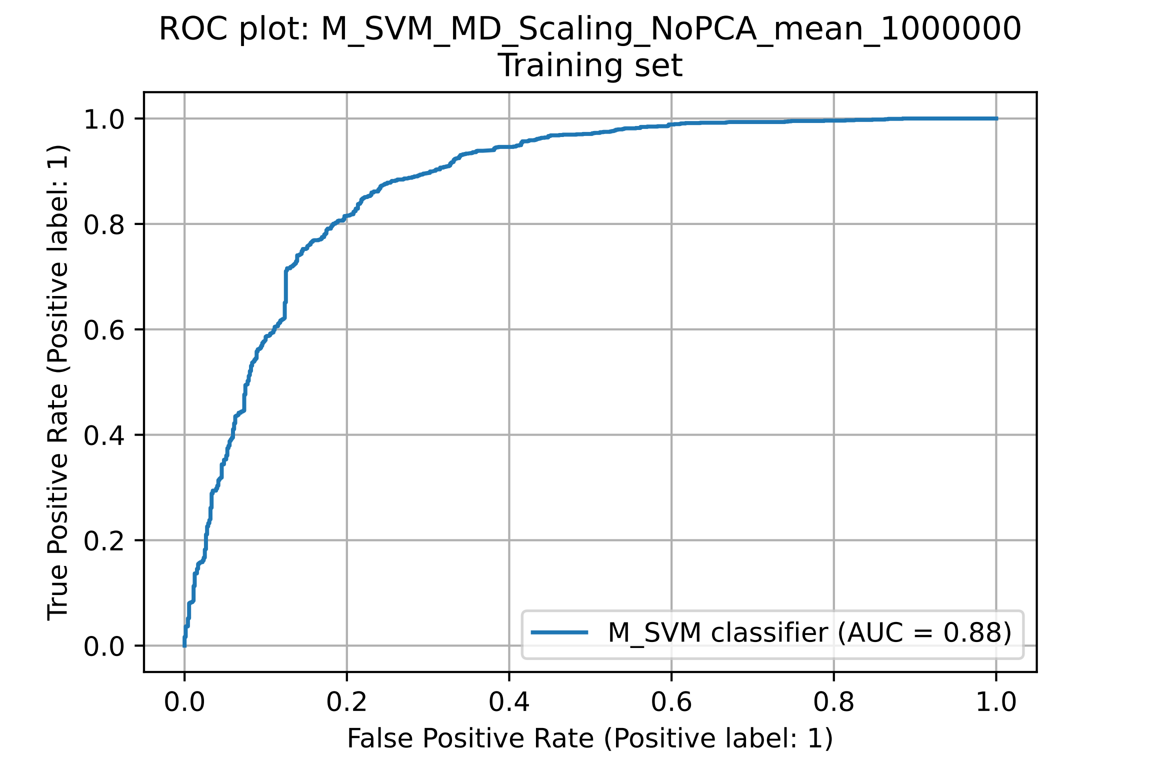
ROC plot: M_SVM_MD_Scaling_NoPCA_mean_1000000
Training set
True Positive Rate (Positive label: 1)
False Positive Rate (Positive label: 1)
0.0
0.2
0.4
0.6
0.8
1.0
M_SVM classifier (AUC = 0.88)

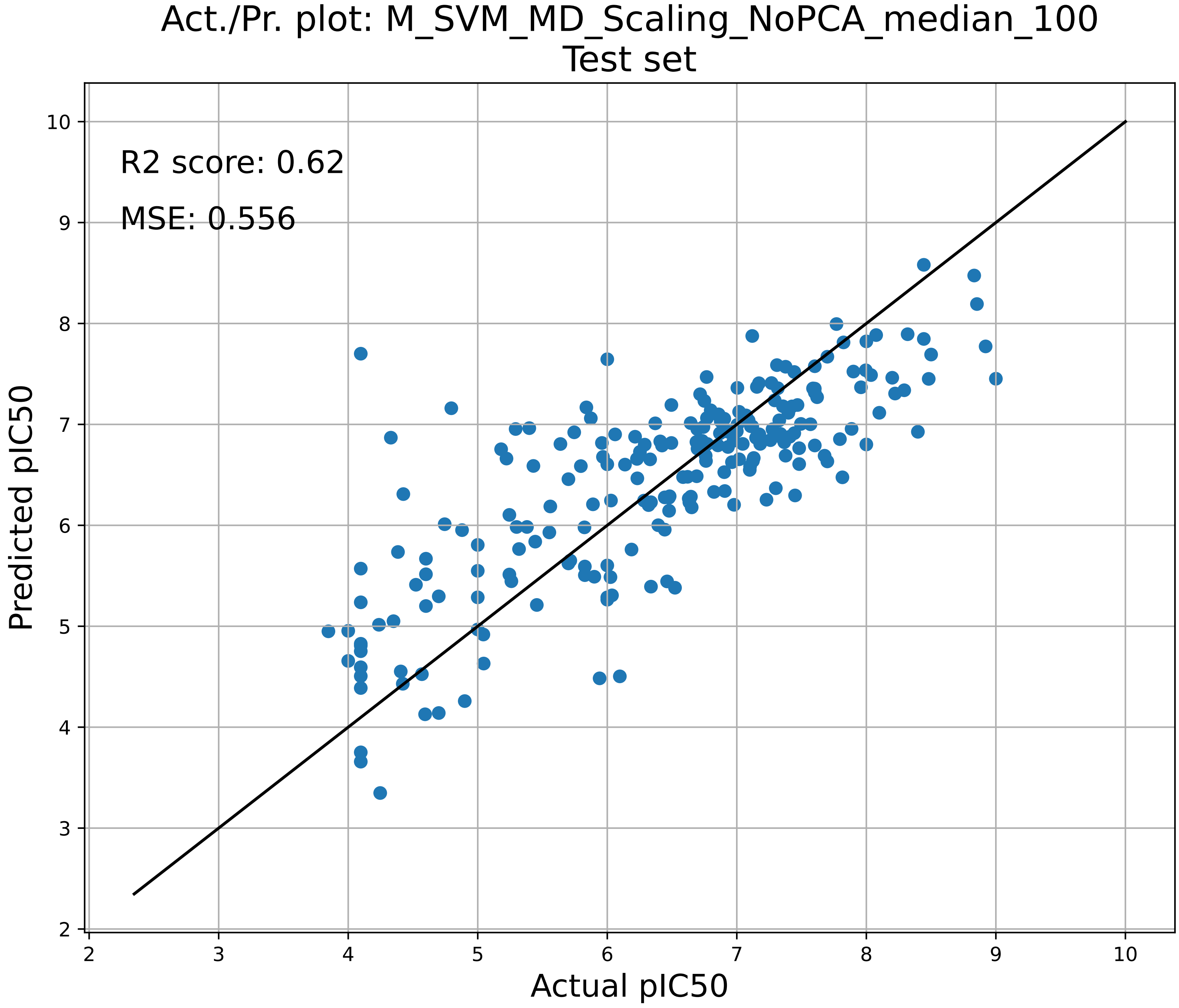
Act./Pr. plot: M_SVM_MD_Scaling_NoPCA_median_100
Test set
R2 score: 0.62
MSE: 0.556
Predicted pIC50
Actual pIC50
2
3
4
5
6
7
8
9
10

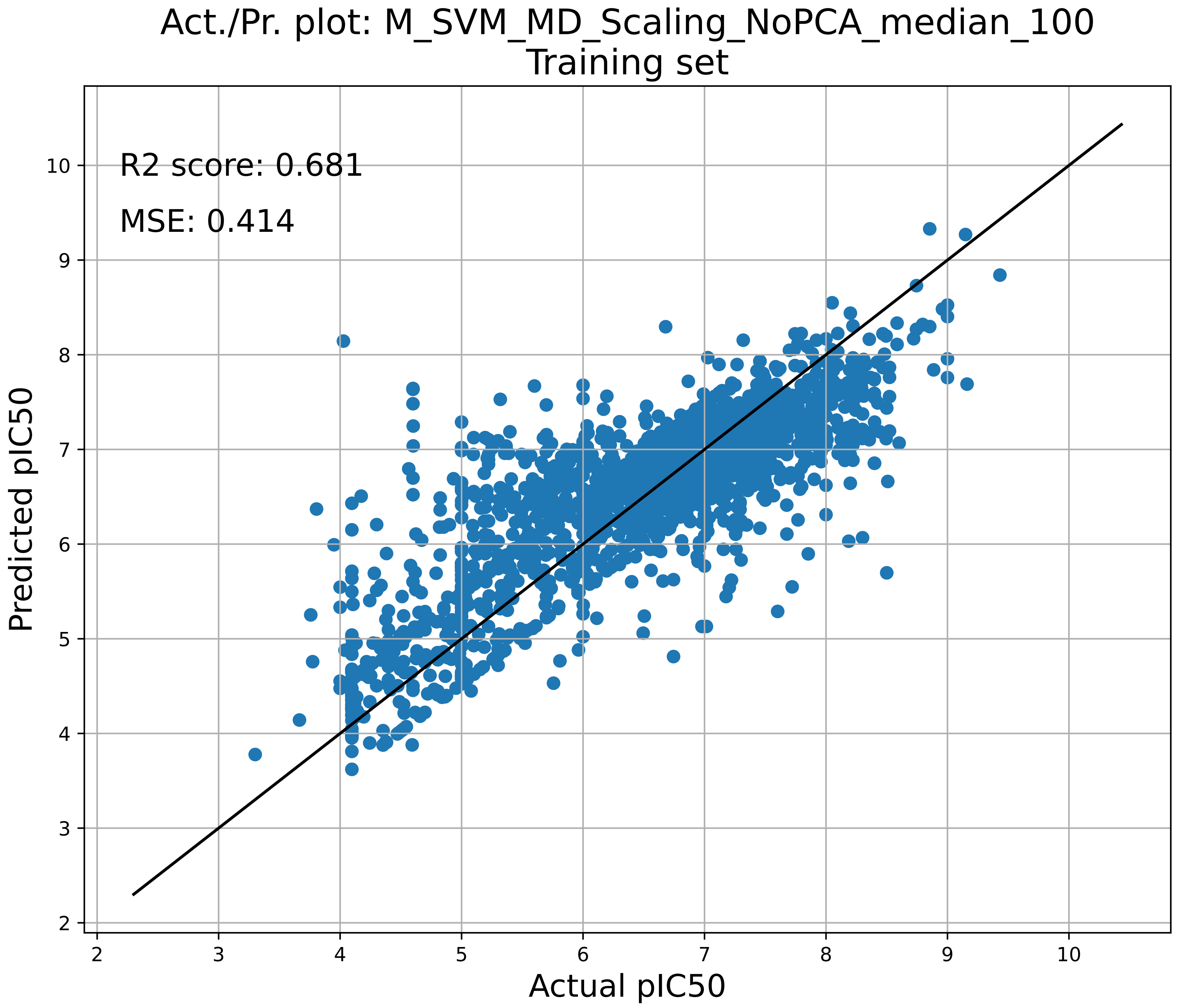
Act./Pr. plot: M_SVM_MD_Scaling_NoPCA_median_100
Training set
R2 score: 0.681
MSE: 0.414
Predicted pIC50
Actual pIC50
2
3
4
5
6
7
8
9
10

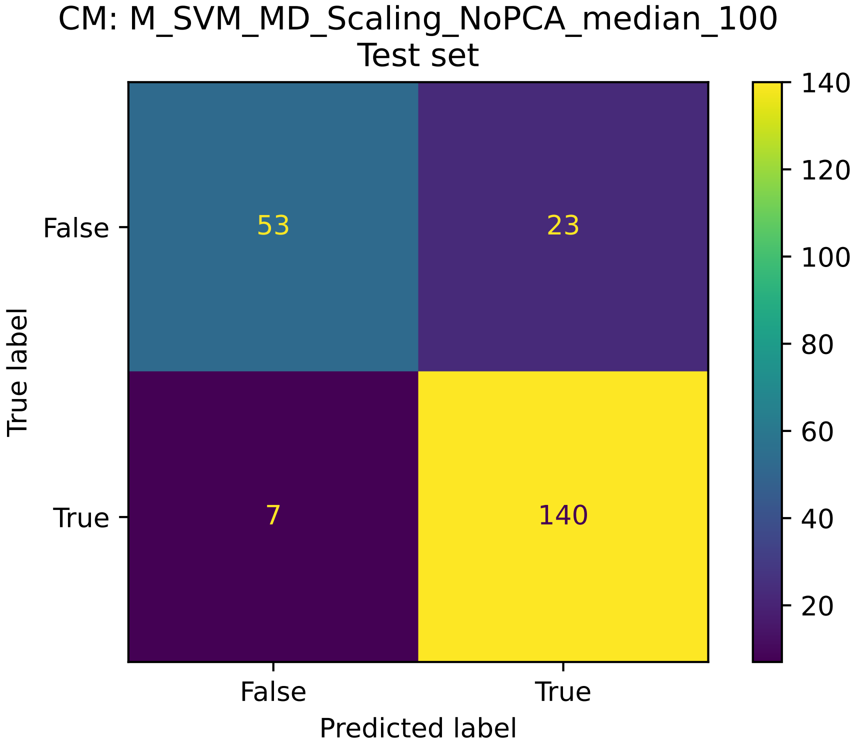
CM: M_SVM_MD_Scaling_NoPCA_median_100
Test set
53
23
7
140
False
True
False
True
True label
Predicted label
140
120
100
80
60
40
20

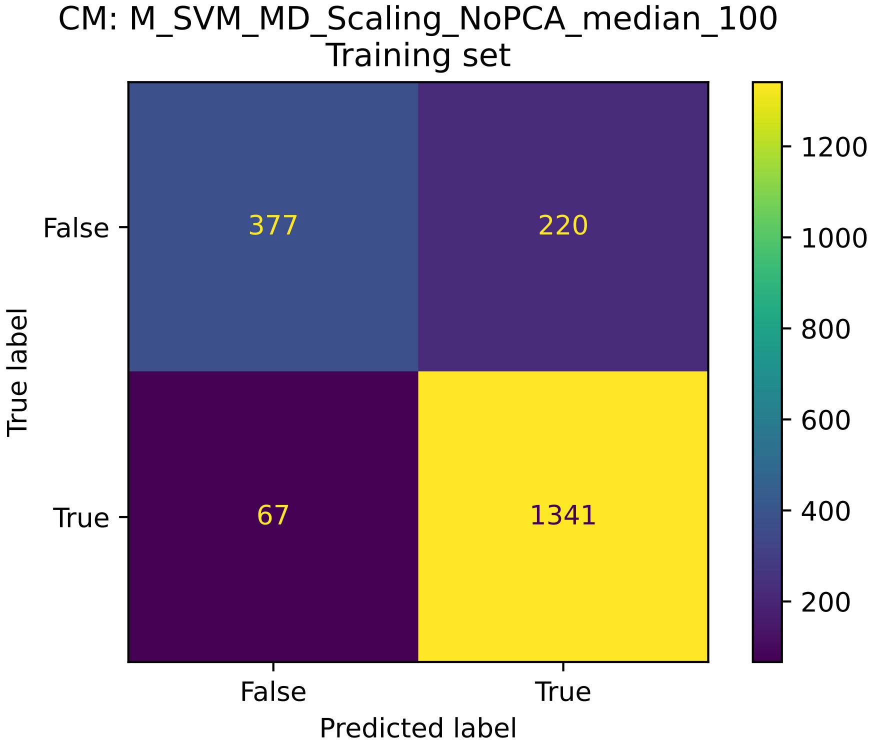

CM: M_SVM_MD_Scaling_NoPCA_median_100
Training set
True label
False
True
377
220
67
1341
False
True
Predicted label
1200
1000
800
600
400
200

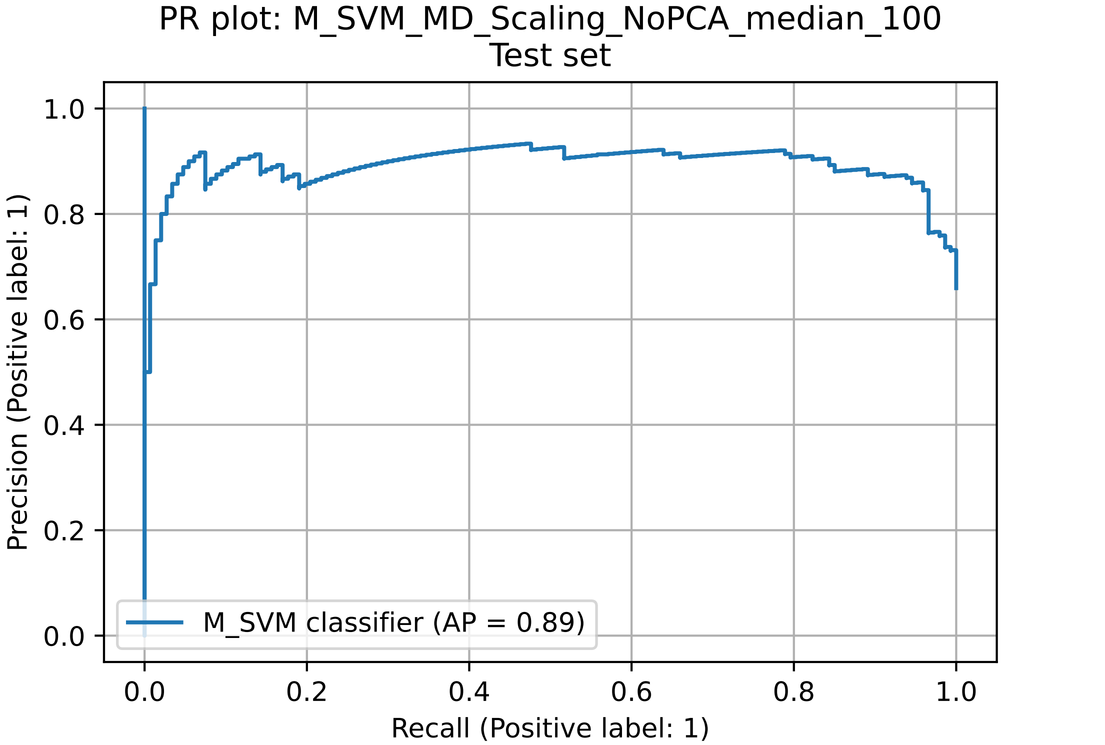
PR plot: M_SVM_MD_Scaling_NoPCA_median_100
Test set
Precision (Positive label: 1)
Recall (Positive label: 1)
0.0
0.2
0.4
0.6
0.8
1.0
M_SVM classifier (AP = 0.89)

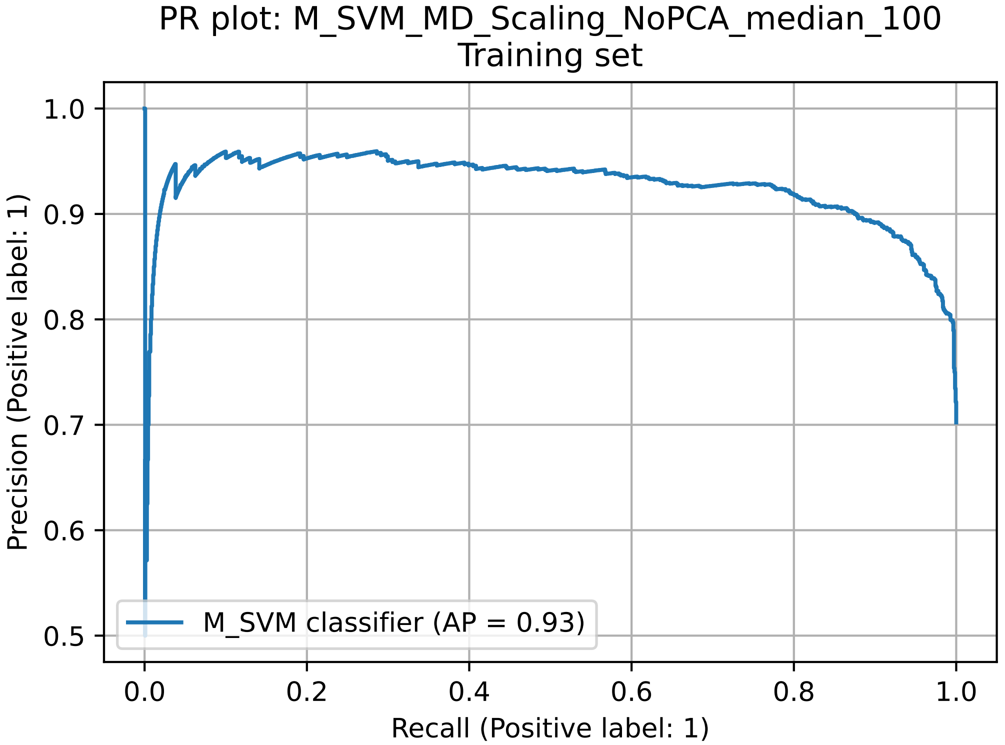
PR plot: M_SVM_MD_Scaling_NoPCA_median_100
Training set
Precision (Positive label: 1)
Recall (Positive label: 1)
1.0
0.9
0.8
0.7
0.6
0.5
0.0
0.2
0.4
0.6
0.8
1.0
M_SVM classifier (AP = 0.93)

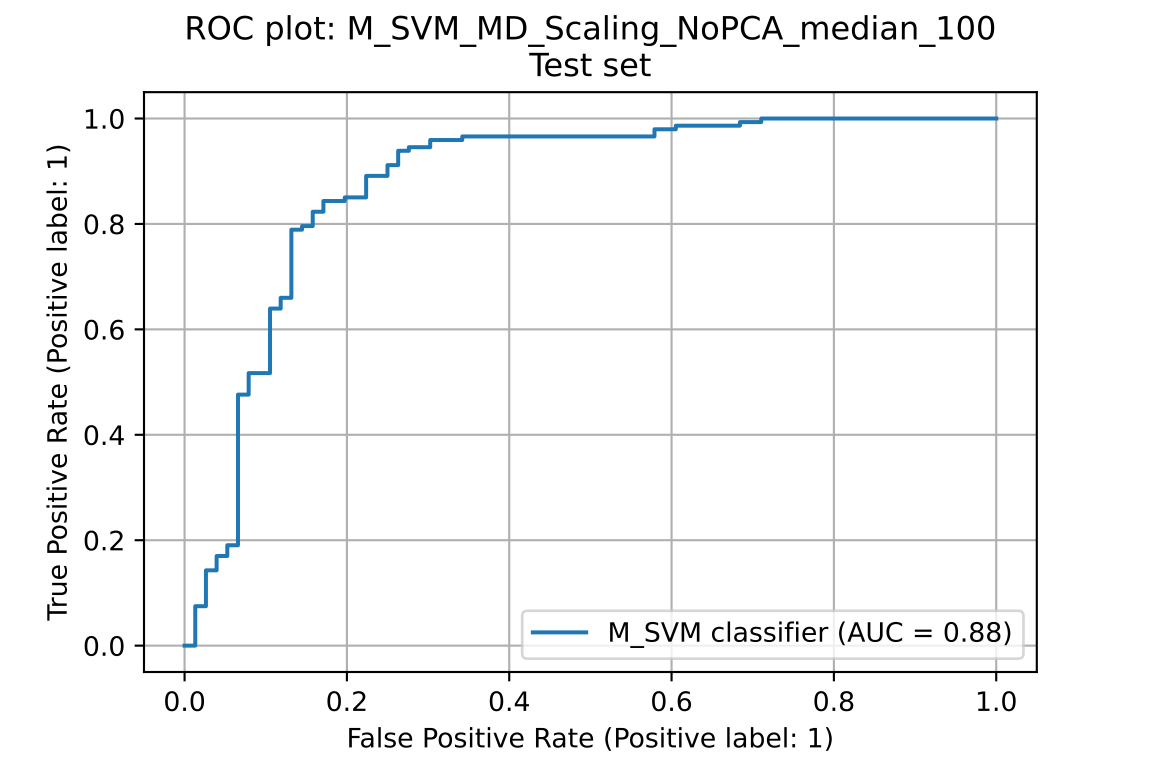
ROC plot: M_SVM_MD_Scaling_NoPCA_median_100
Test set
True Positive Rate (Positive label: 1)
False Positive Rate (Positive label: 1)
0.0
0.2
0.4
0.6
0.8
1.0
M_SVM classifier (AUC = 0.88)

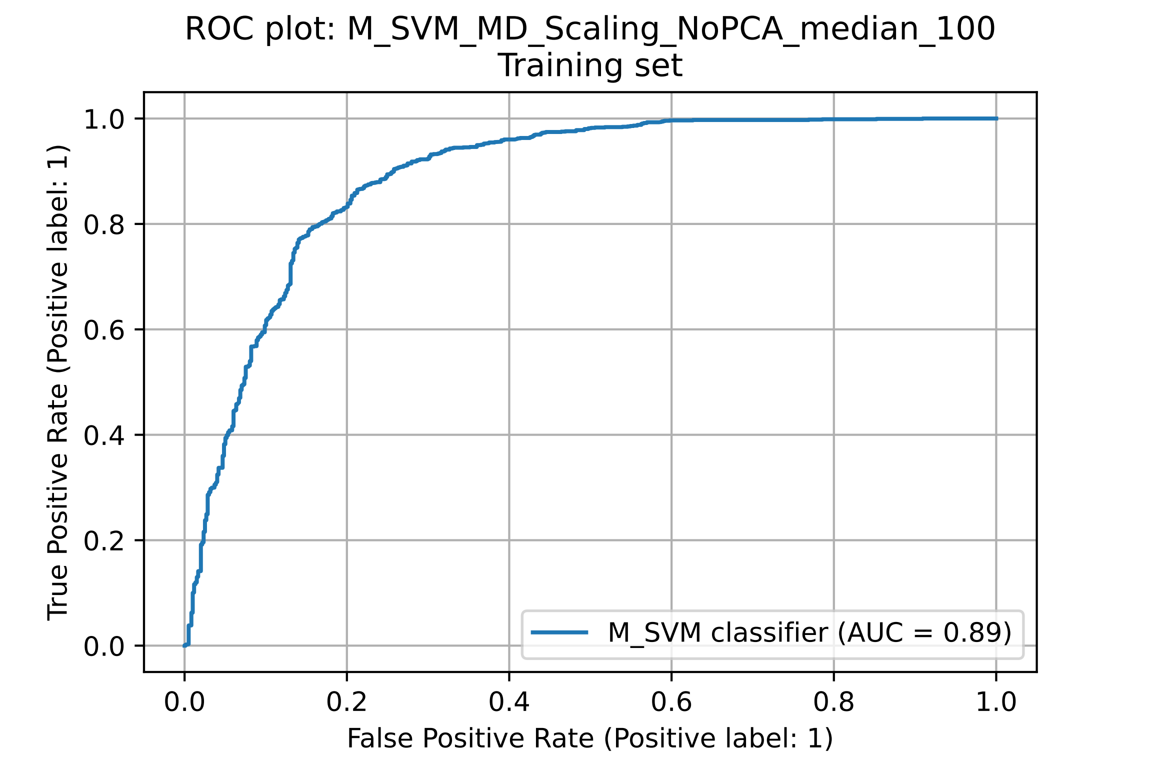

ROC plot: M_SVM_MD_Scaling_NoPCA_median_100
Training set
True Positive Rate (Positive label: 1)
False Positive Rate (Positive label: 1)
0.0
0.2
0.4
0.6
0.8
1.0
M_SVM classifier (AUC = 0.89)

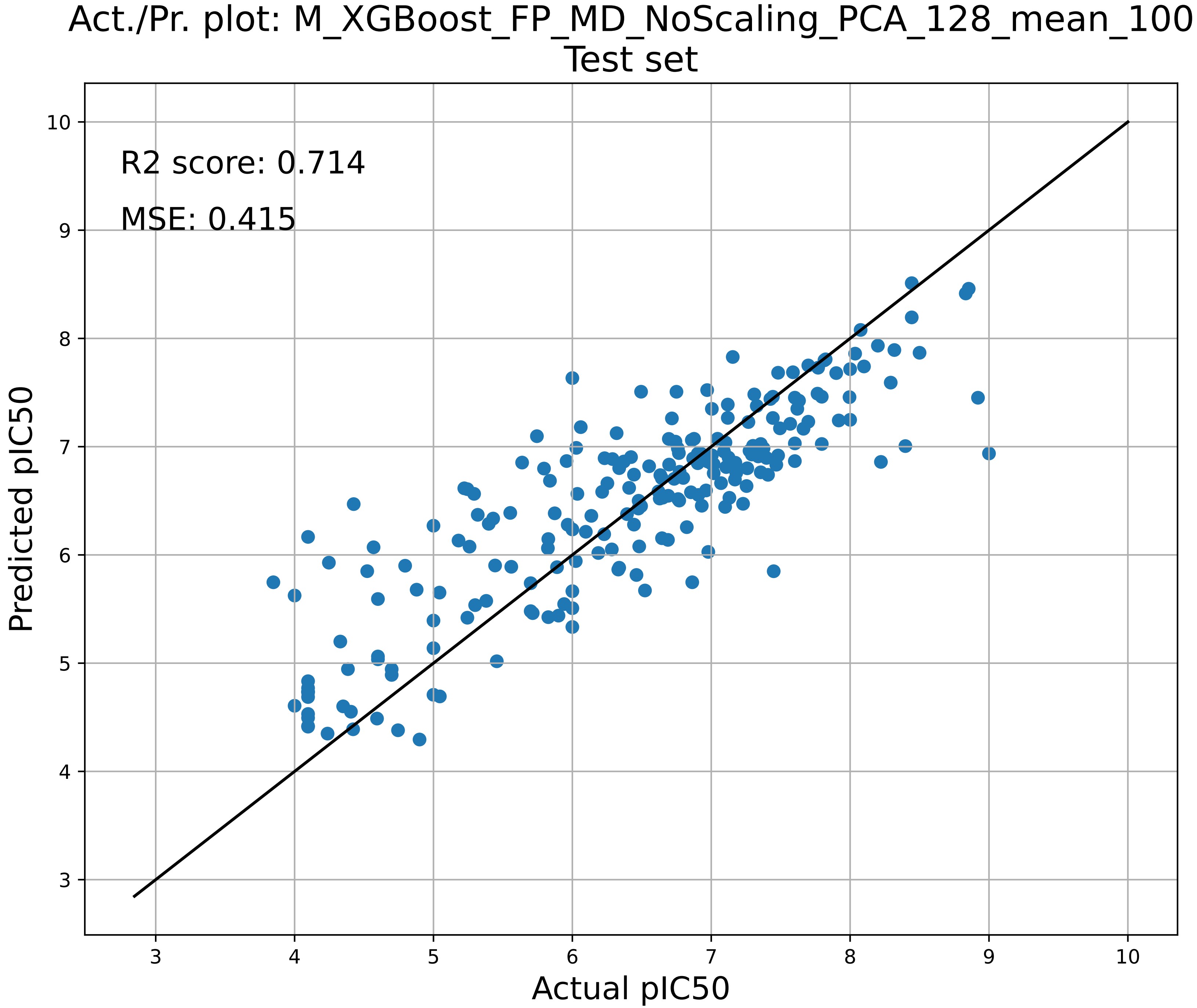
Act./Pr. plot: M_XGBoost_FP_MD_NoScaling_PCA_128_mean_100
Test set
R2 score: 0.714
MSE: 0.415
Predicted pIC50
Actual pIC50
3
4
5
6
7
8
9
10
3
4
5
6
7
8
9
10

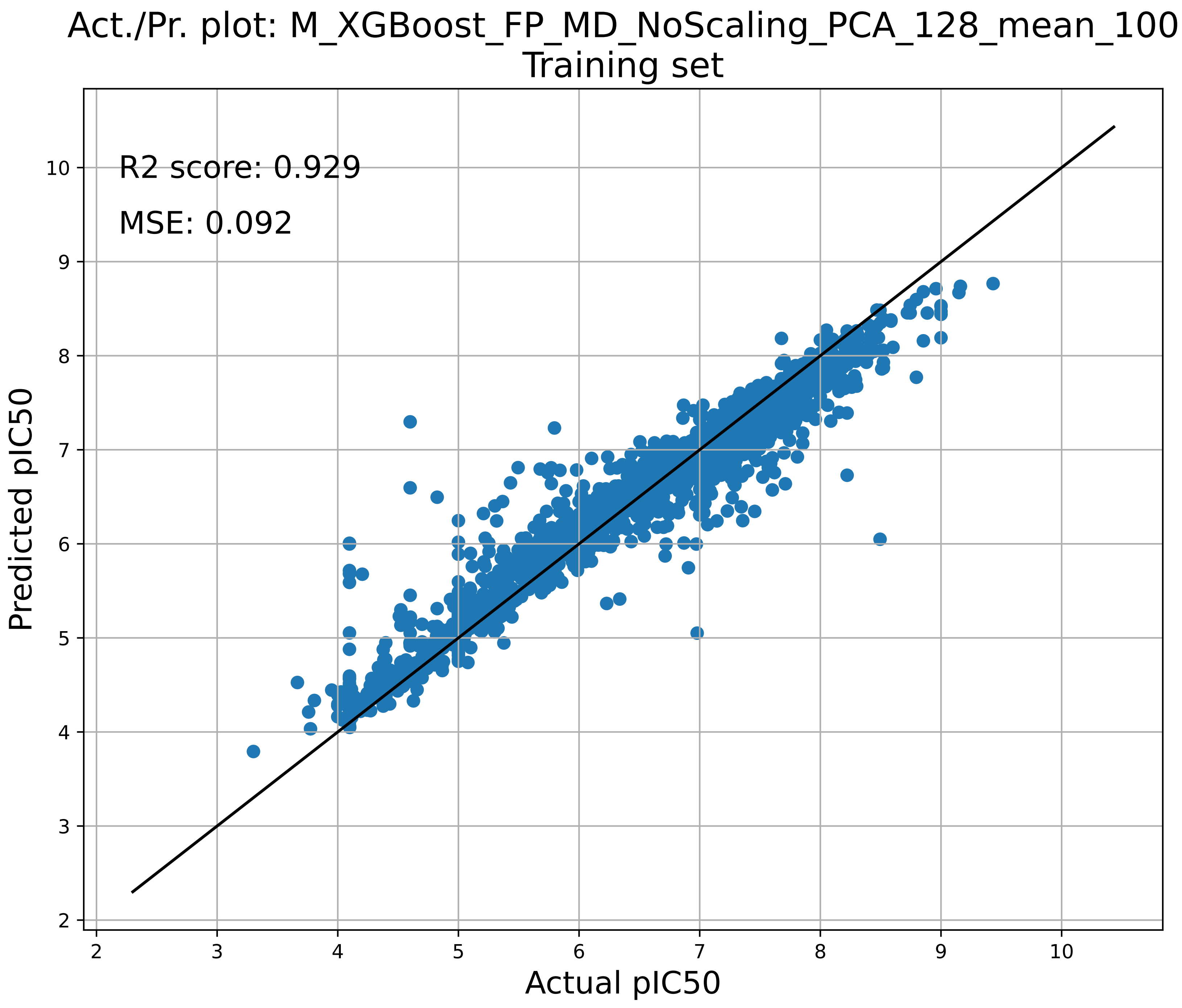
Act./Pr. plot: M_XGBoost_FP_MD_NoScaling_PCA_128_mean_100
Training set
R2 score: 0.929
MSE: 0.092
Predicted pIC50
Actual pIC50
2
3
4
5
6
7
8
9
10

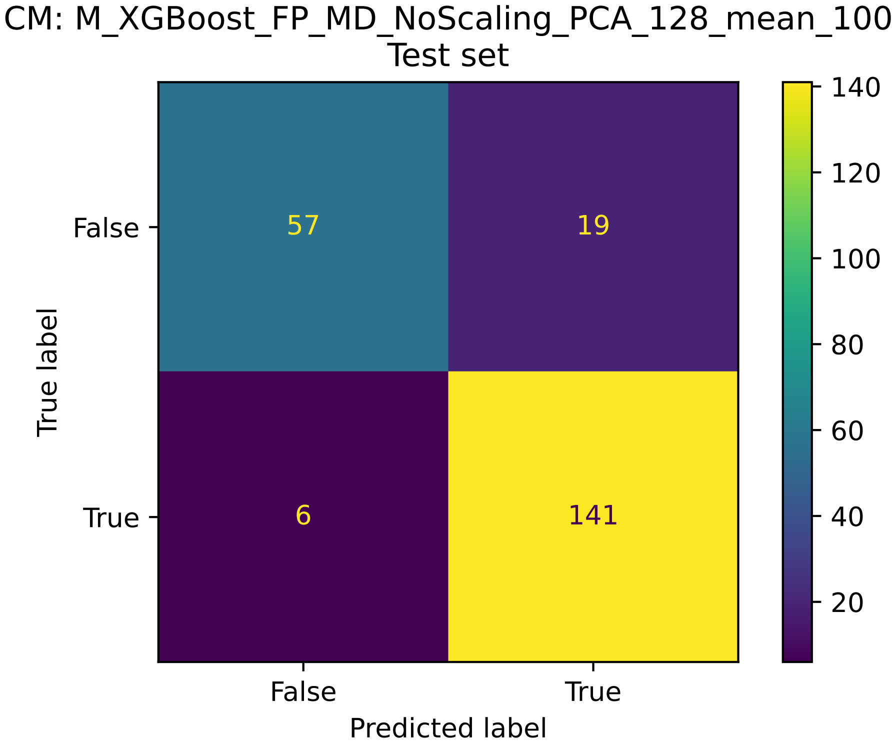
CM: M_XGBoost_FP_MD_NoScaling_PCA_128_mean_100
Test set
True label
False
True
57
19
6
141
Predicted label
False
True
140
120
100
80
60
40
20

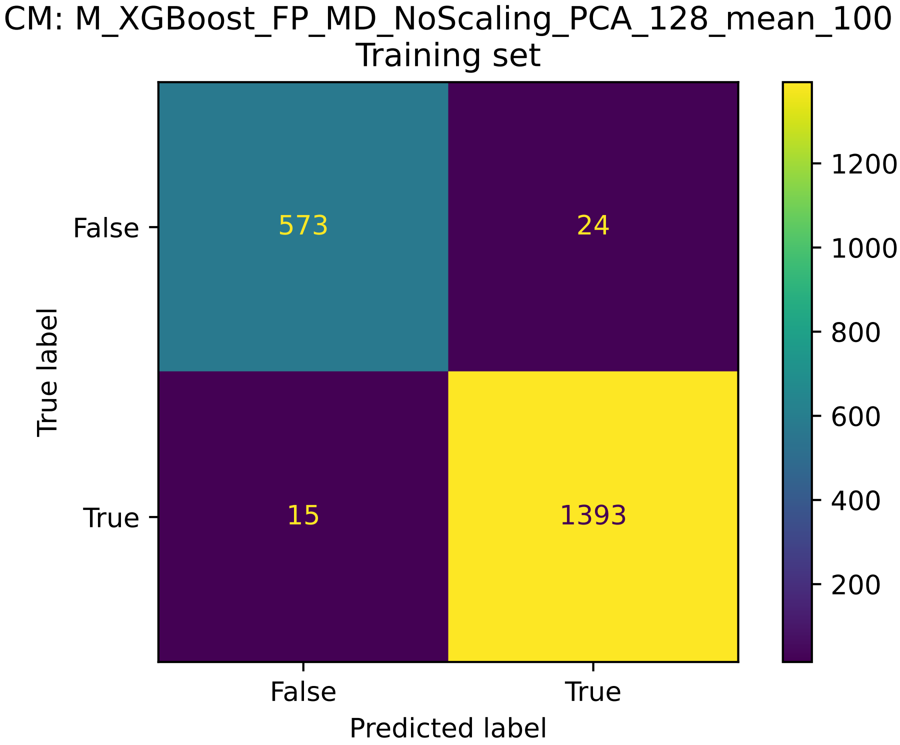
CM: M_XGBoost_FP_MD_NoScaling_PCA_128_mean_100
Training set
True label
False
True
573
24
15
1393
Predicted label
False
True
1200
1000
800
600
400
200

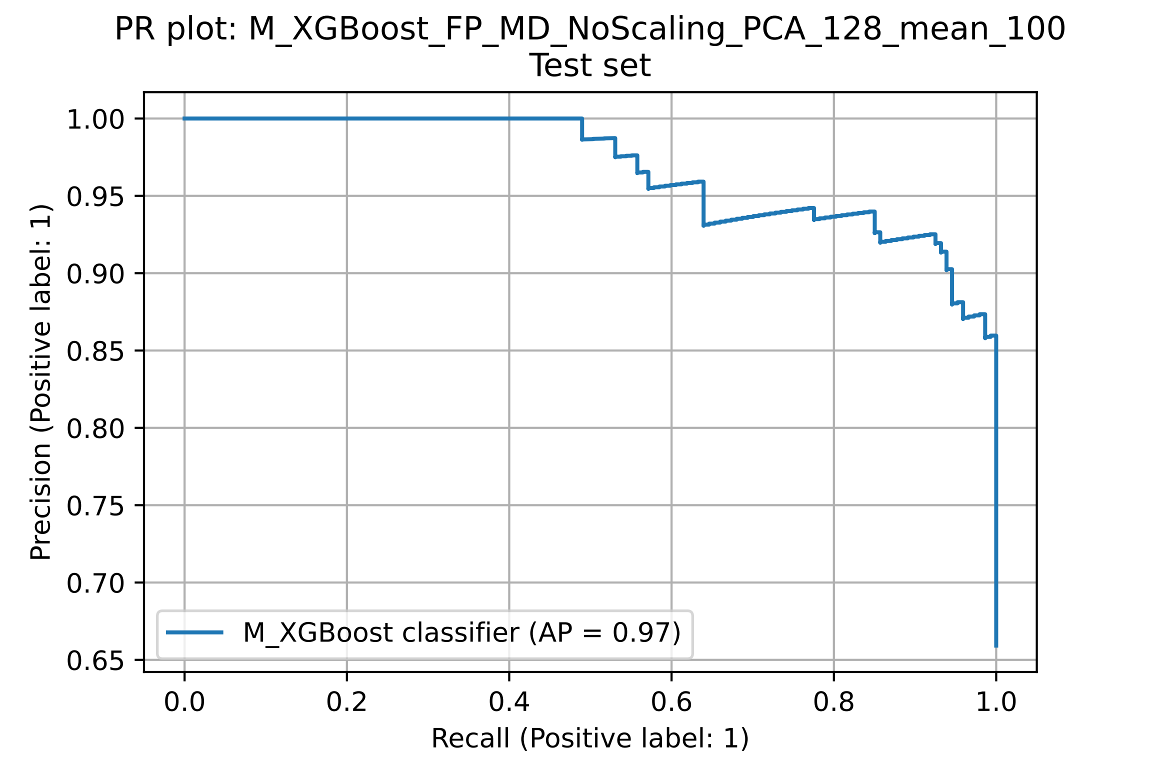
PR plot: M_XGBoost_FP_MD_NoScaling_PCA_128_mean_100
Test set
Precision (Positive label: 1)
Recall (Positive label: 1)
1.00
0.95
0.90
0.85
0.80
0.75
0.70
0.65
0.0
0.2
0.4
0.6
0.8
1.0
M_XGBoost classifier (AP = 0.97)

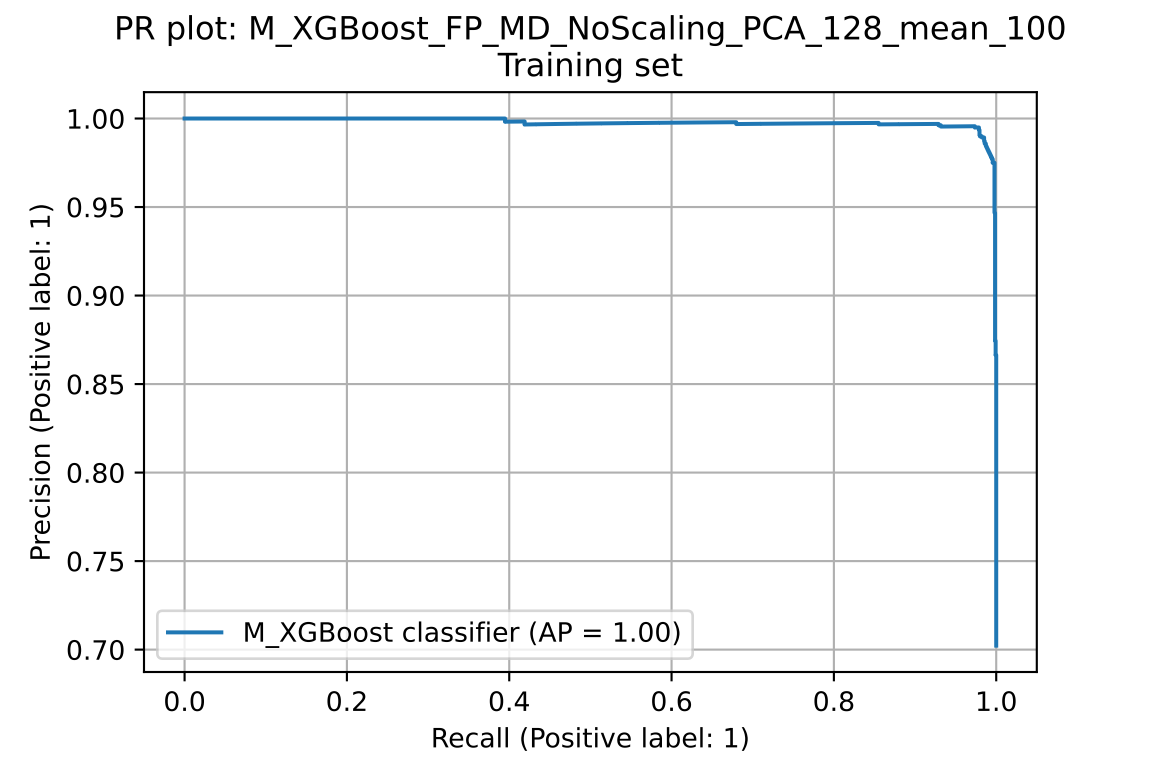
PR plot: M_XGBoost_FP_MD_NoScaling_PCA_128_mean_100
Training set
Precision (Positive label: 1)
Recall (Positive label: 1)
1.00
0.95
0.90
0.85
0.80
0.75
0.70
0.0
0.2
0.4
0.6
0.8
1.0
M_XGBoost classifier (AP = 1.00)

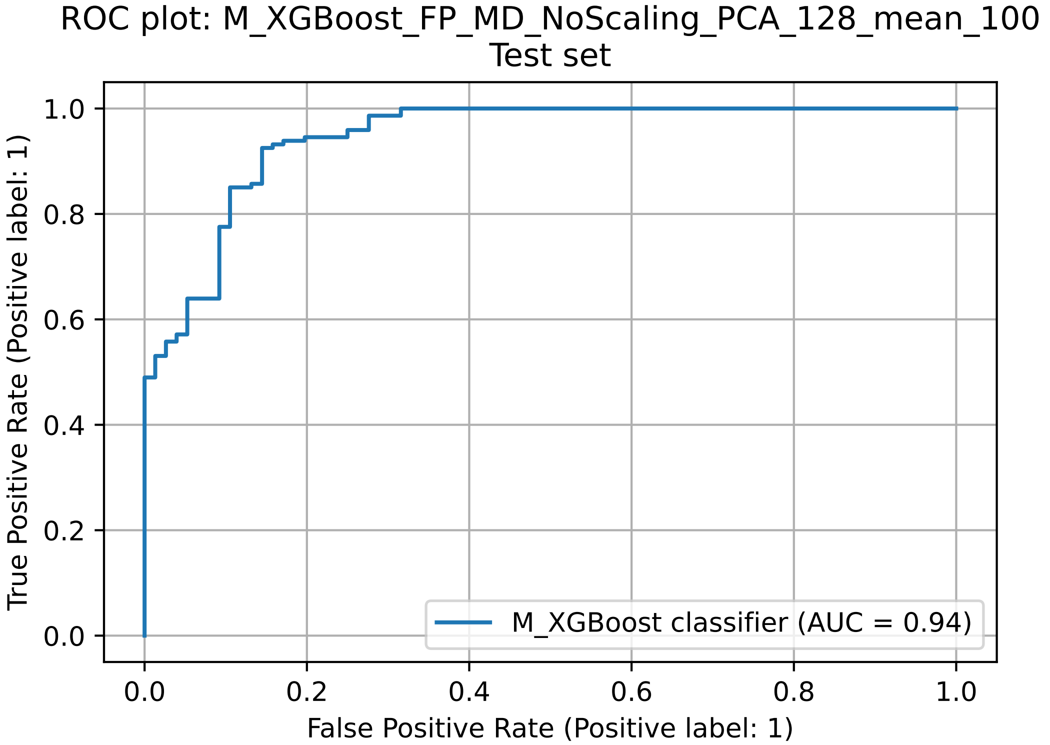

ROC plot: M_XGBoost_FP_MD_NoScaling_PCA_128_mean_100
Test set
True Positive Rate (Positive label: 1)
False Positive Rate (Positive label: 1)
0.0
0.2
0.4
0.6
0.8
1.0
M_XGBoost classifier (AUC = 0.94)

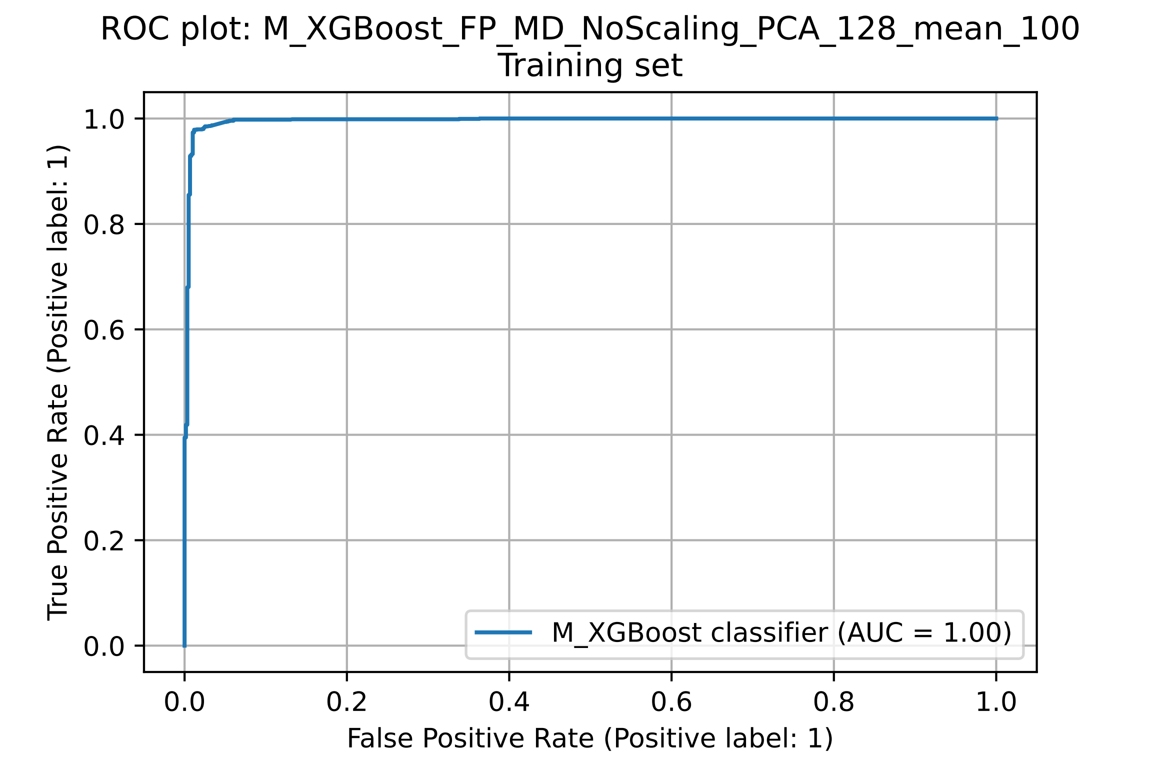

ROC plot: M_XGBoost_FP_MD_NoScaling_PCA_128_mean_100
Training set
True Positive Rate (Positive label: 1)
False Positive Rate (Positive label: 1)
0.0
0.2
0.4
0.6
0.8
1.0
M_XGBoost classifier (AUC = 1.00)

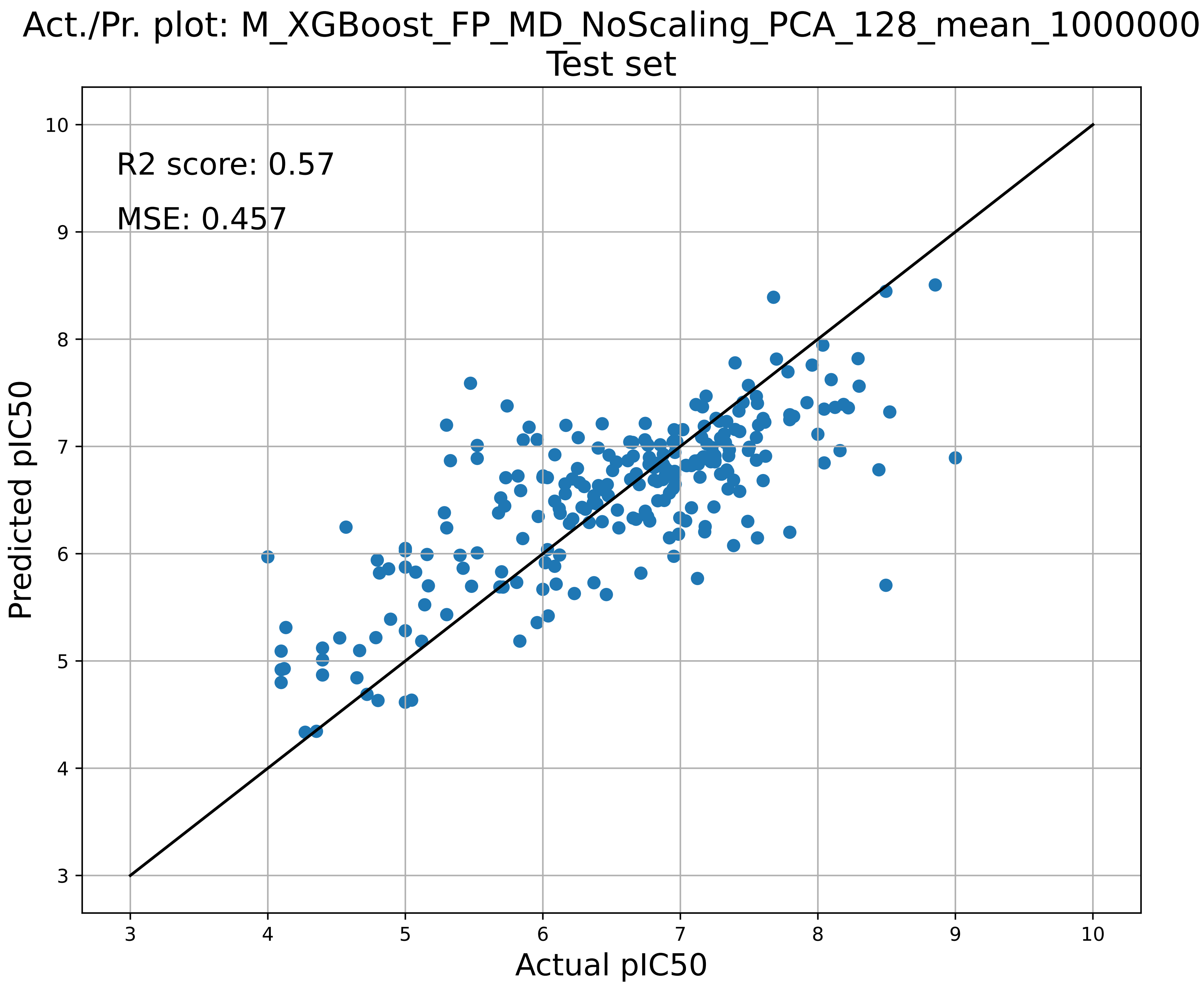

Act./Pr. plot: M_XGBoost_FP_MD_NoScaling_PCA_128_mean_1000000
Test set
R2 score: 0.57
MSE: 0.457
Predicted pIC50
Actual pIC50
3
4
5
6
7
8
9
10

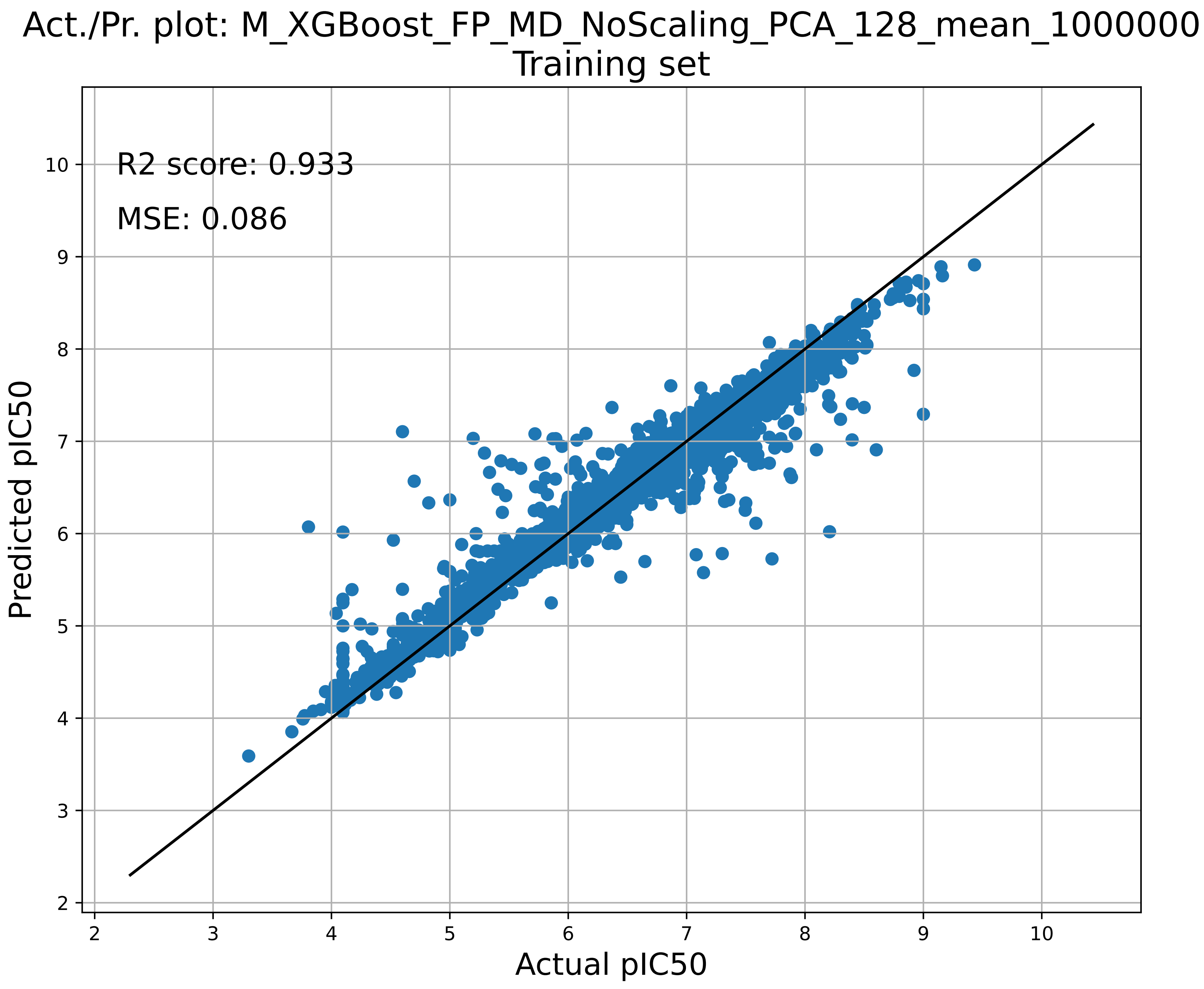

Act./Pr. plot: M_XGBoost_FP_MD_NoScaling_PCA_128_mean_1000000
Training set
R2 score: 0.933
MSE: 0.086
Predicted pIC50
Actual pIC50
2
3
4
5
6
7
8
9
10

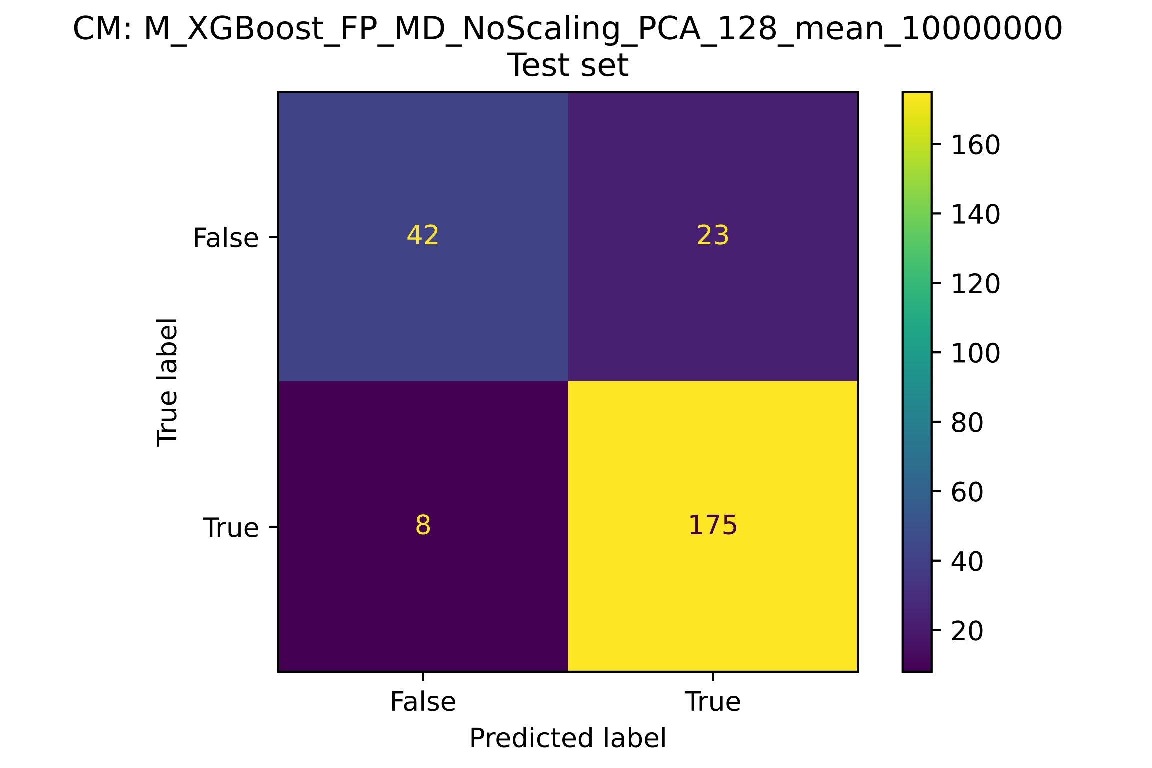
CM: M_XGBoost_FP_MD_NoScaling_PCA_128_mean_10000000
Test set
True label
Predicted label
False
True
False
True
42
23
8
175
160
140
120
100
80
60
40
20

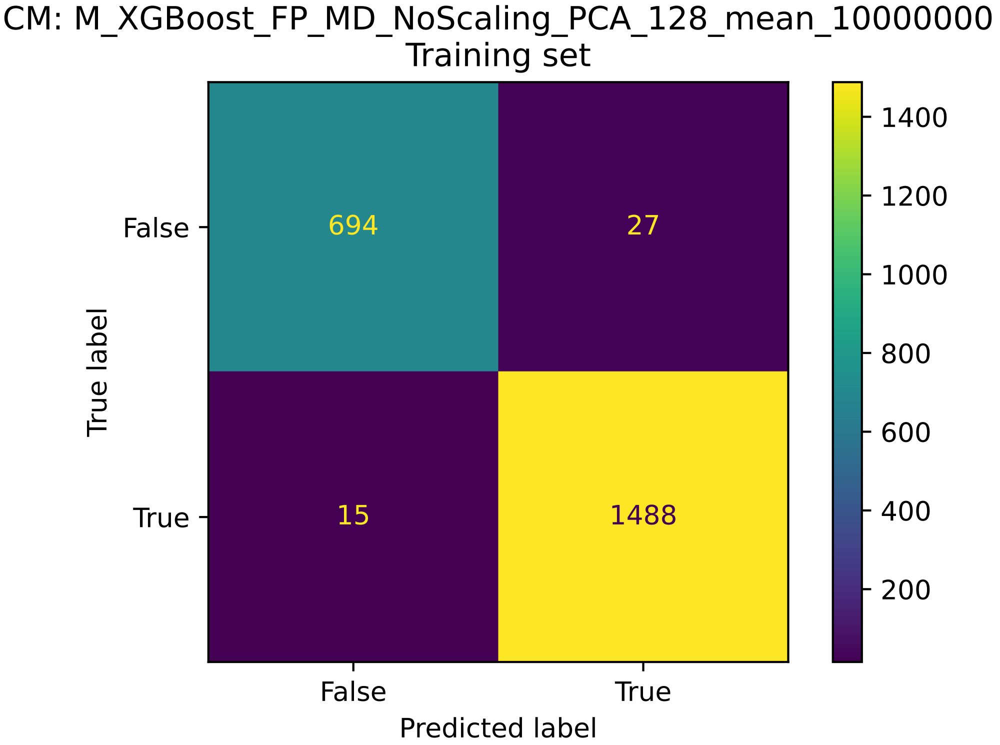
CM: M_XGBoost_FP_MD_NoScaling_PCA_128_mean_10000000
Training set
True label
False
True
694
27
15
1488
Predicted label
False
True
1400
1200
1000
800
600
400
200

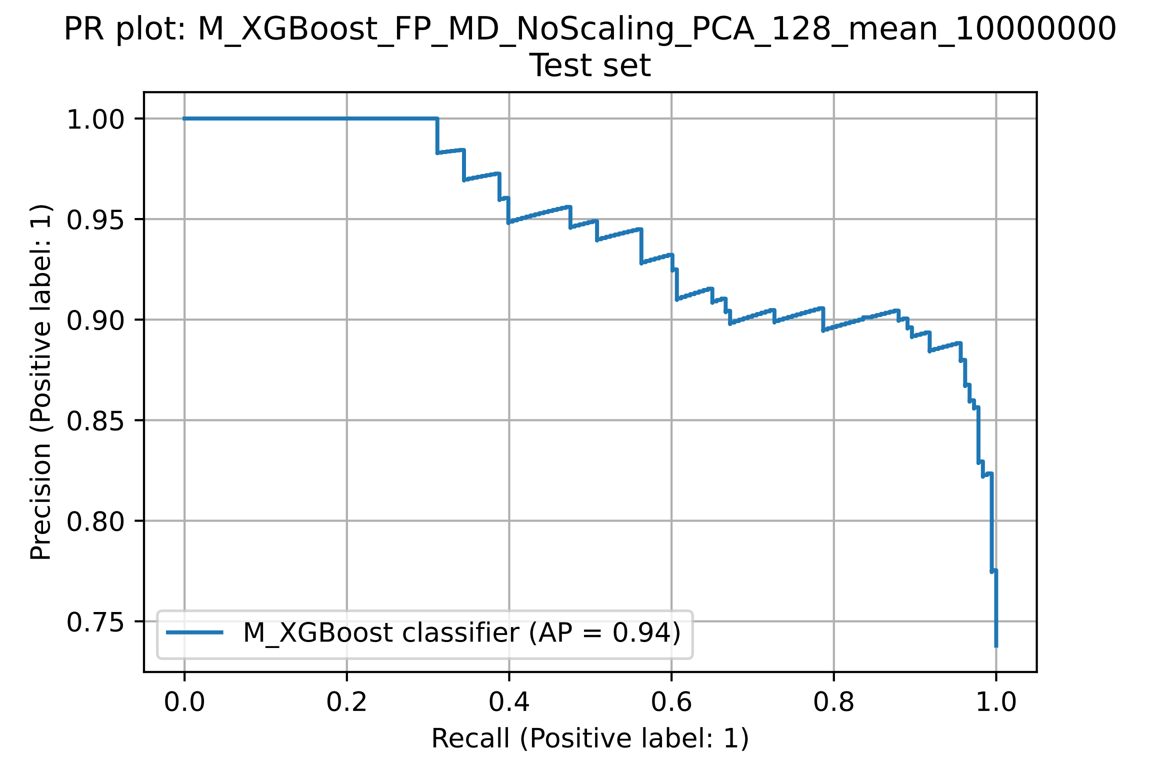

PR plot: M_XGBoost_FP_MD_NoScaling_PCA_128_mean_10000000
Test set
Precision (Positive label: 1)
Recall (Positive label: 1)
1.00
0.95
0.90
0.85
0.80
0.75
0.0
0.2
0.4
0.6
0.8
1.0
M_XGBoost classifier (AP = 0.94)

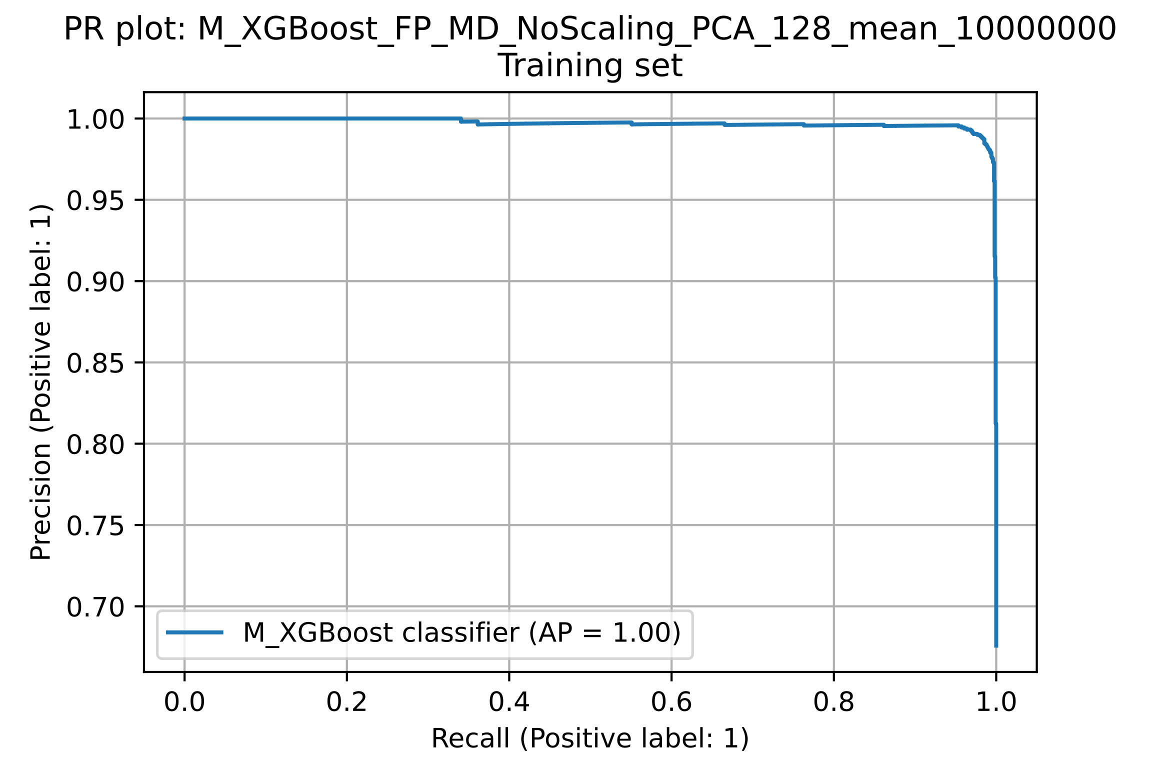

PR plot: M_XGBoost_FP_MD_NoScaling_PCA_128_mean_10000000
Training set
1.00
0.95
0.90
0.85
0.80
0.75
0.70
Precision (Positive label: 1)
0.0
0.2
0.4
0.6
0.8
1.0
Recall (Positive label: 1)
M_XGBoost classifier (AP = 1.00)

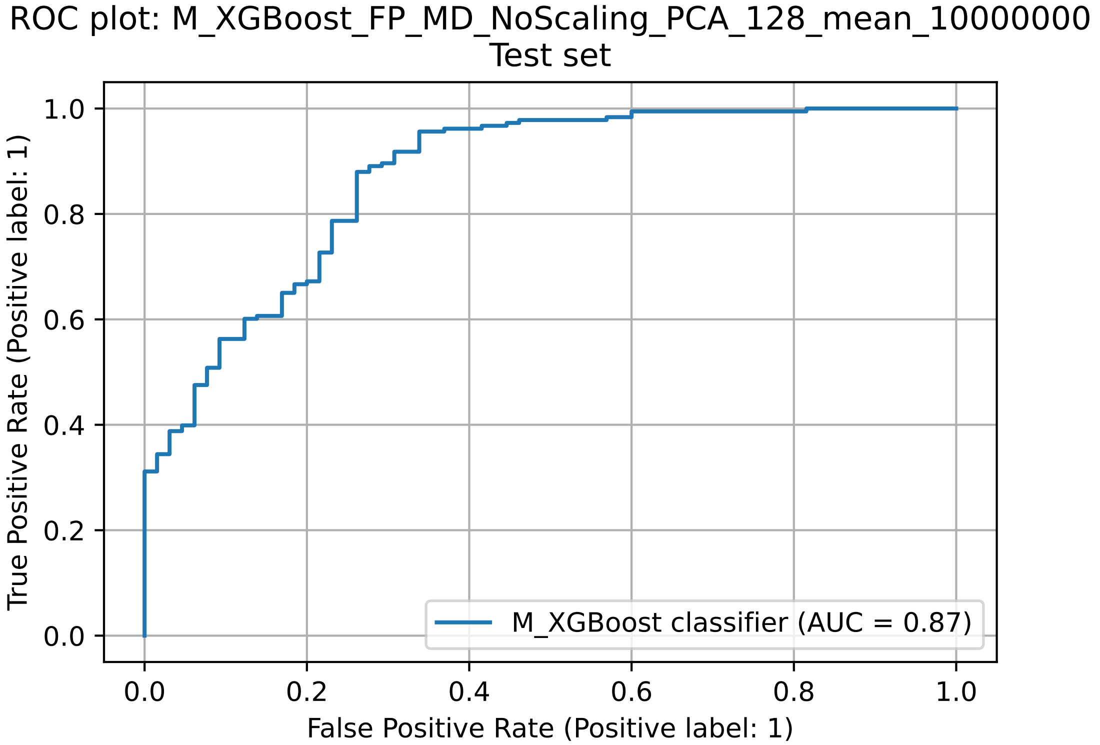
ROC plot: M_XGBoost_FP_MD_NoScaling_PCA_128_mean_10000000
Test set
True Positive Rate (Positive label: 1)
False Positive Rate (Positive label: 1)
0.0
0.2
0.4
0.6
0.8
1.0
M_XGBoost classifier (AUC = 0.87)

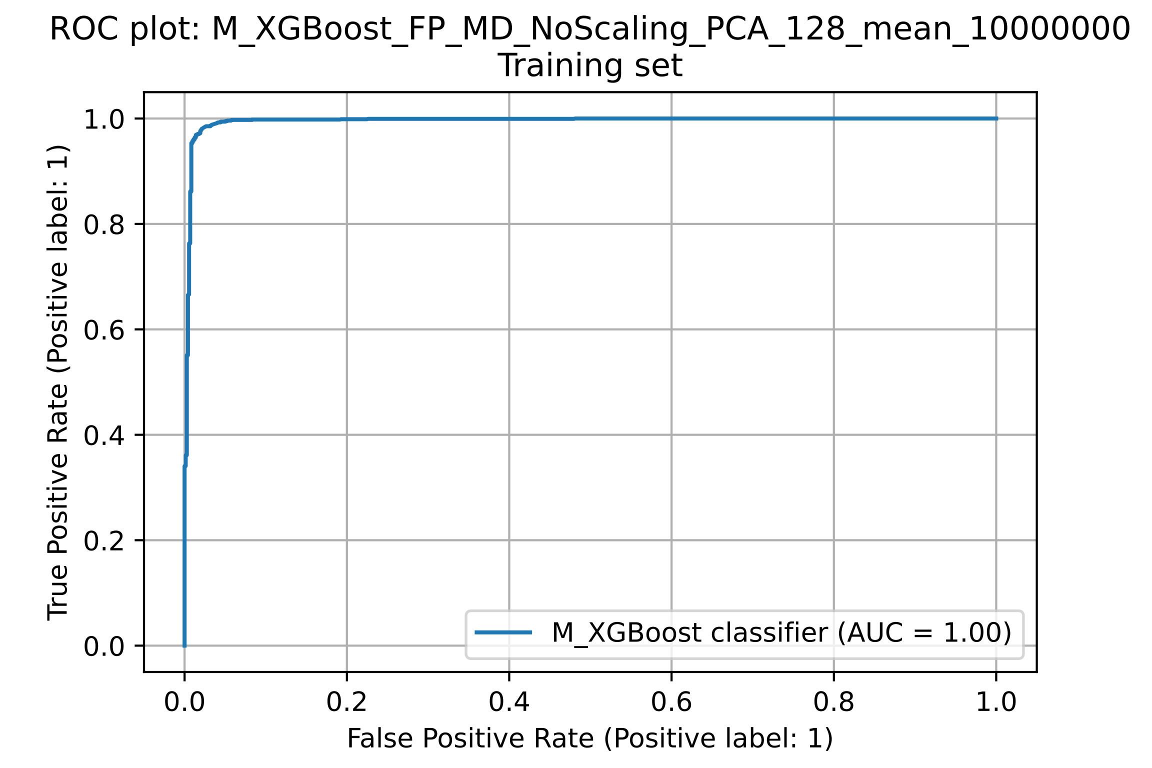
ROC plot: M_XGBoost_FP_MD_NoScaling_PCA_128_mean_10000000
Training set
True Positive Rate (Positive label: 1)
False Positive Rate (Positive label: 1)
0.0
0.2
0.4
0.6
0.8
1.0
M_XGBoost classifier (AUC = 1.00)

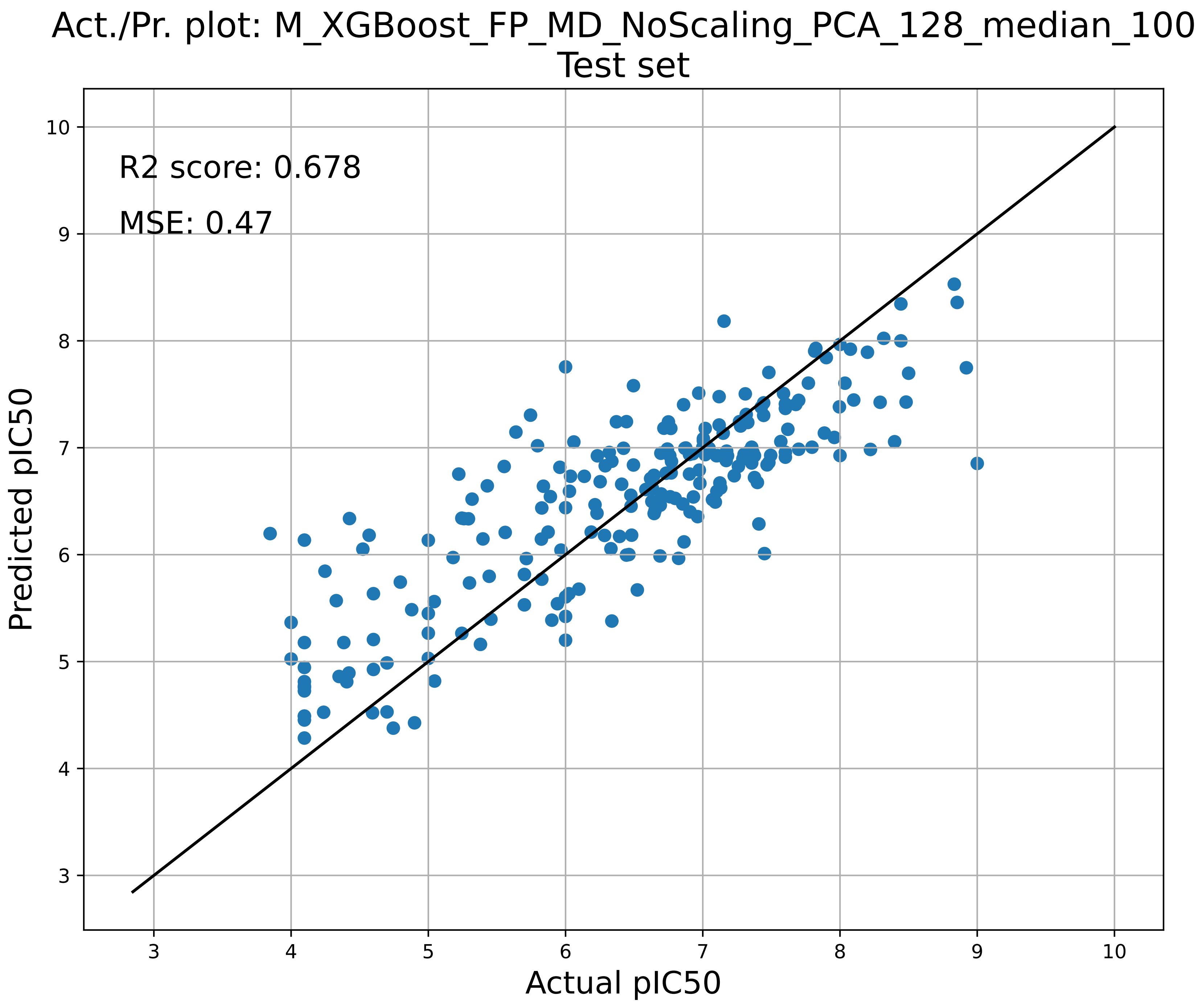

Act./Pr. plot: M_XGBoost_FP_MD_NoScaling_PCA_128_median_100
Test set
R2 score: 0.678
MSE: 0.47
Predicted pIC50
Actual pIC50
3
4
5
6
7
8
9
10

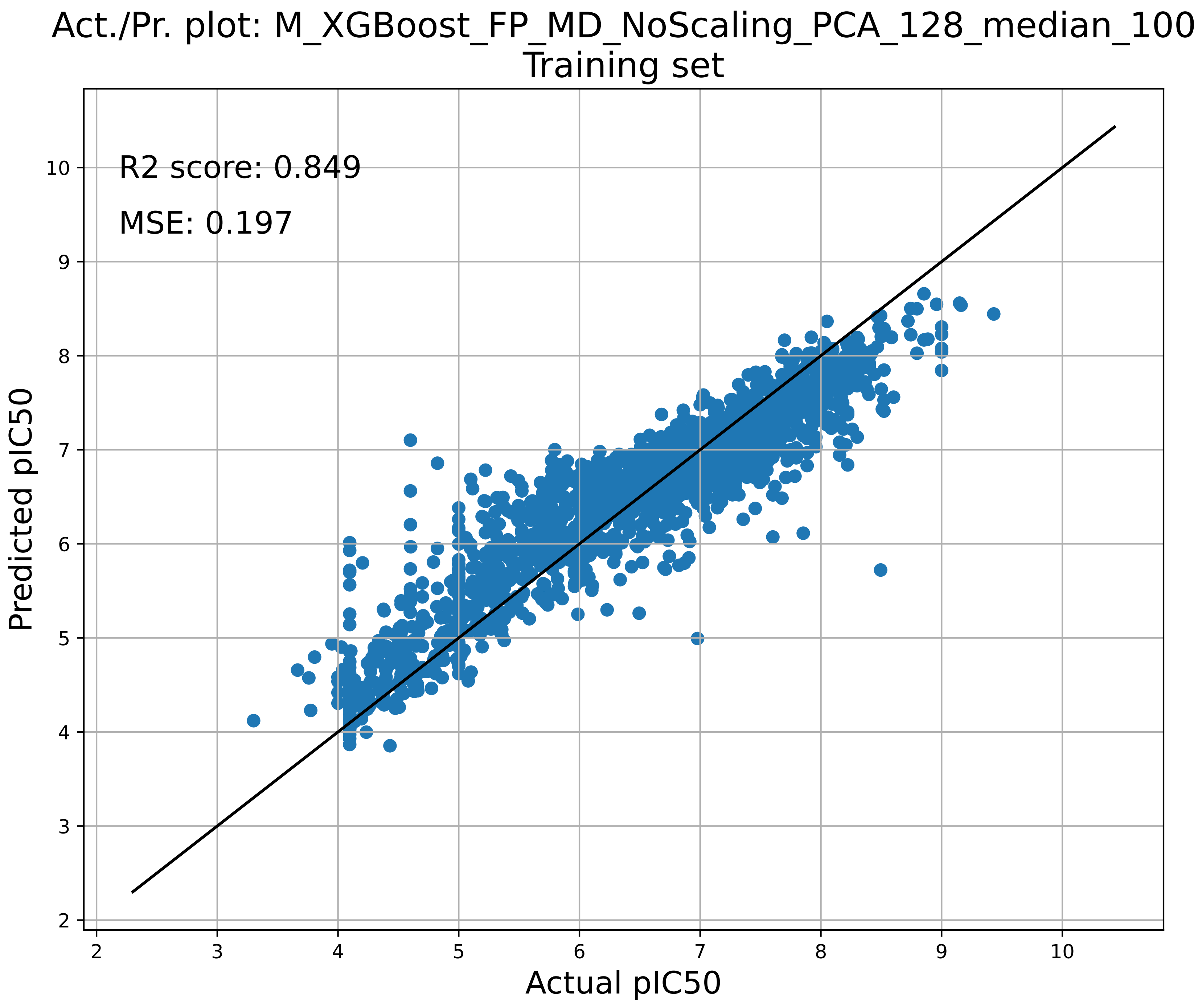

Act./Pr. plot: M_XGBoost_FP_MD_NoScaling_PCA_128_median_100
Training set
R2 score: 0.849
MSE: 0.197
Predicted pIC50
Actual pIC50
2
3
4
5
6
7
8
9
10

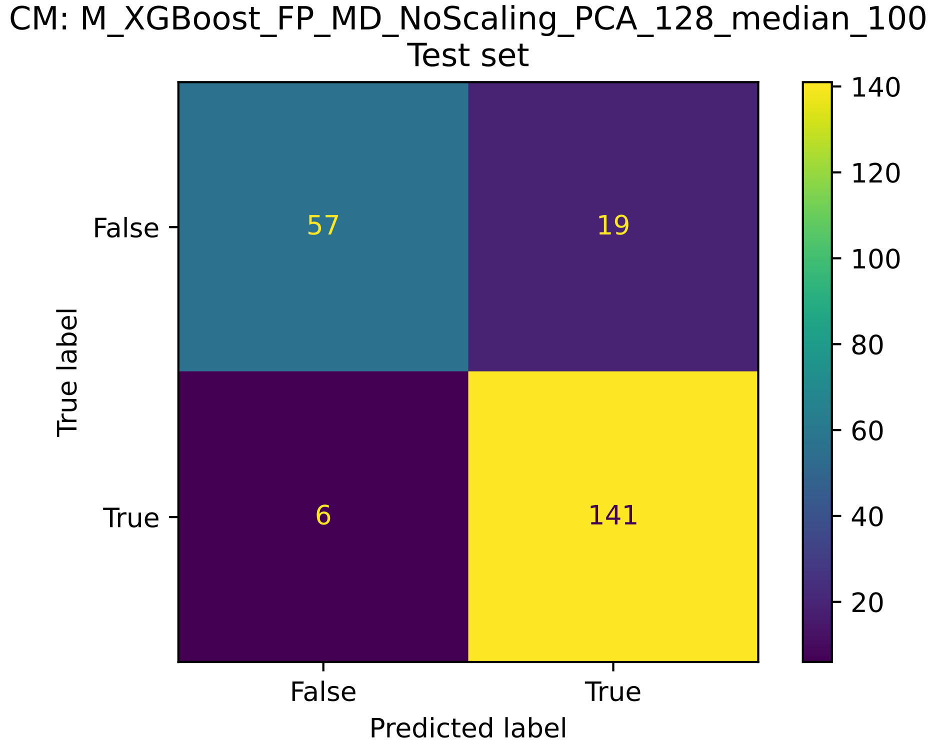
CM: M_XGBoost_FP_MD_NoScaling_PCA_128_median_100
Test set
True label
False
True
57
19
6
141
Predicted label
False
True
140
120
100
80
60
40
20

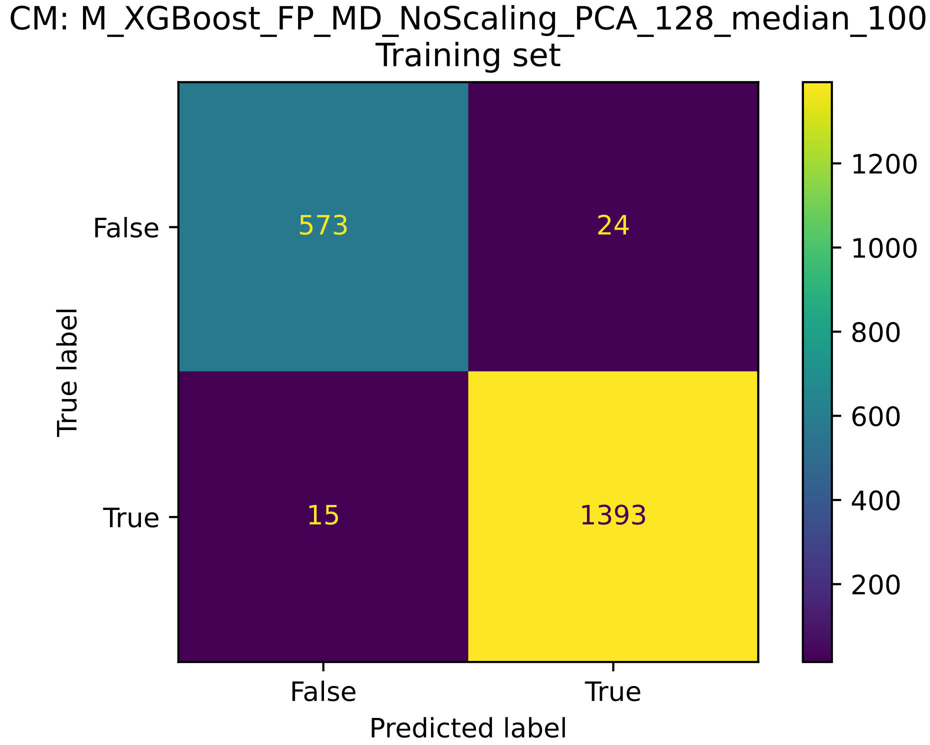

CM: M_XGBoost_FP_MD_NoScaling_PCA_128_median_100
Training set
573
24
15
1393
False
True
True label
False
True
Predicted label
200
400
600
800
1000
1200

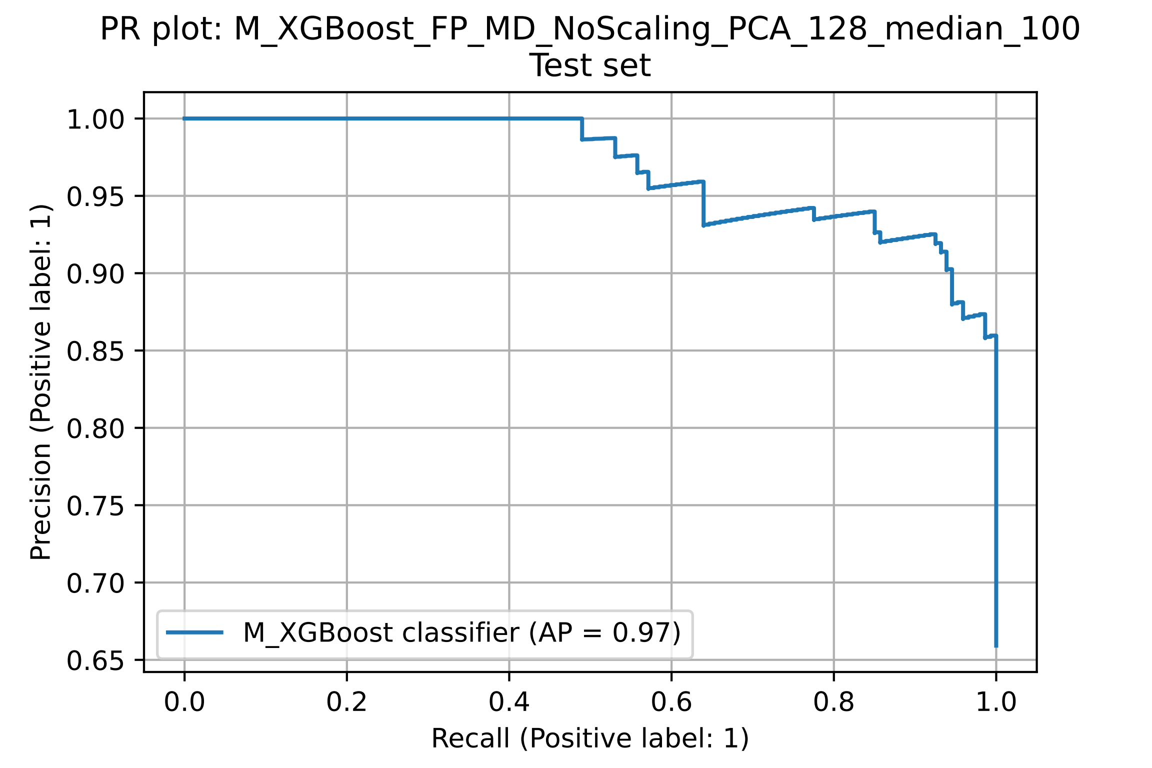

PR plot: M_XGBoost_FP_MD_NoScaling_PCA_128_median_100
Test set
Precision (Positive label: 1)
Recall (Positive label: 1)
1.00
0.95
0.90
0.85
0.80
0.75
0.70
0.65
0.0
0.2
0.4
0.6
0.8
1.0
M_XGBoost classifier (AP = 0.97)

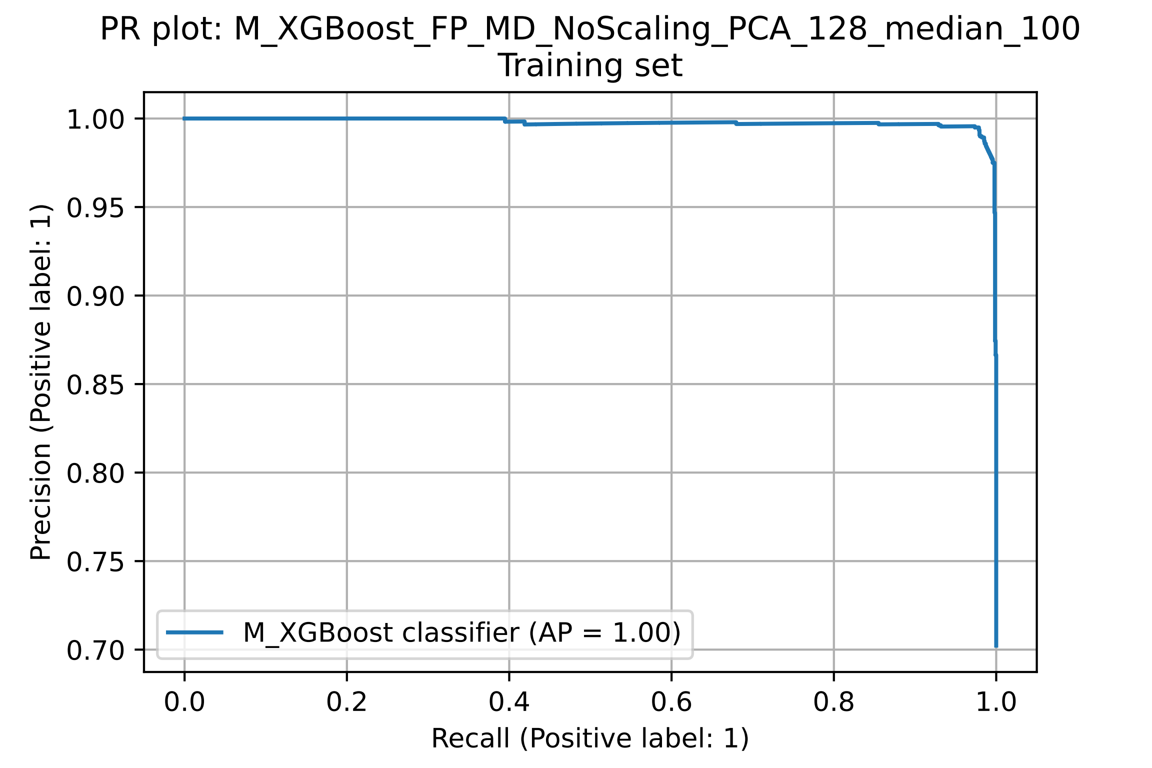

PR plot: M_XGBoost_FP_MD_NoScaling_PCA_128_median_100
Training set
Precision (Positive label: 1)
Recall (Positive label: 1)
1.00
0.95
0.90
0.85
0.80
0.75
0.70
0.0
0.2
0.4
0.6
0.8
1.0
M_XGBoost classifier (AP = 1.00)

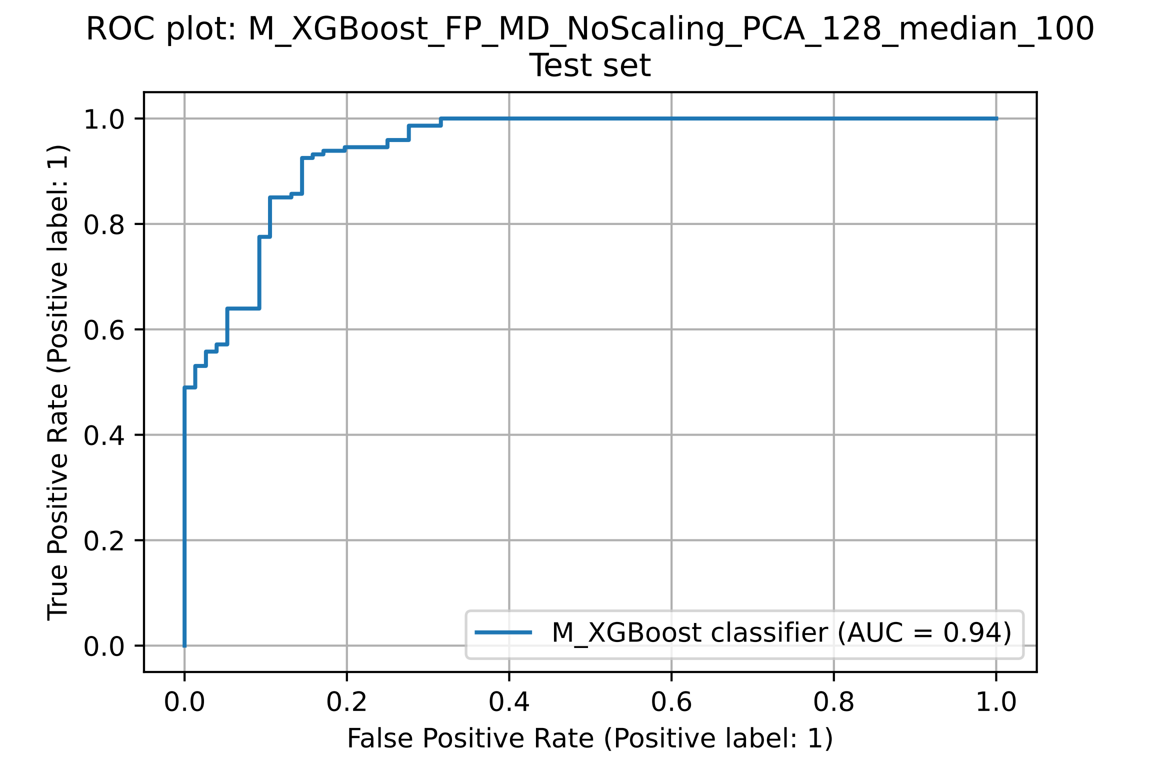

ROC plot: M_XGBoost_FP_MD_NoScaling_PCA_128_median_100
Test set
True Positive Rate (Positive label: 1)
False Positive Rate (Positive label: 1)
0.0
0.2
0.4
0.6
0.8
1.0
M_XGBoost classifier (AUC = 0.94)

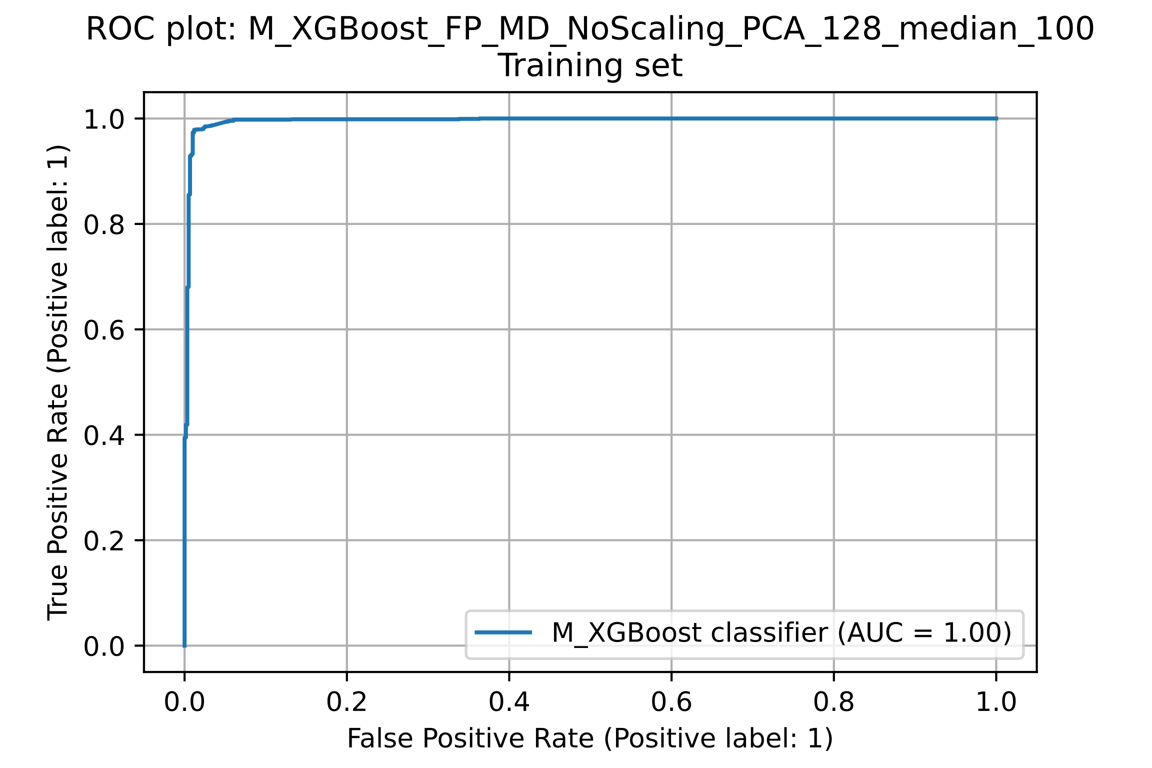
ROC plot: M_XGBoost_FP_MD_NoScaling_PCA_128_median_100
Training set
True Positive Rate (Positive label: 1)
False Positive Rate (Positive label: 1)
0.0
0.2
0.4
0.6
0.8
1.0
M_XGBoost classifier (AUC = 1.00)

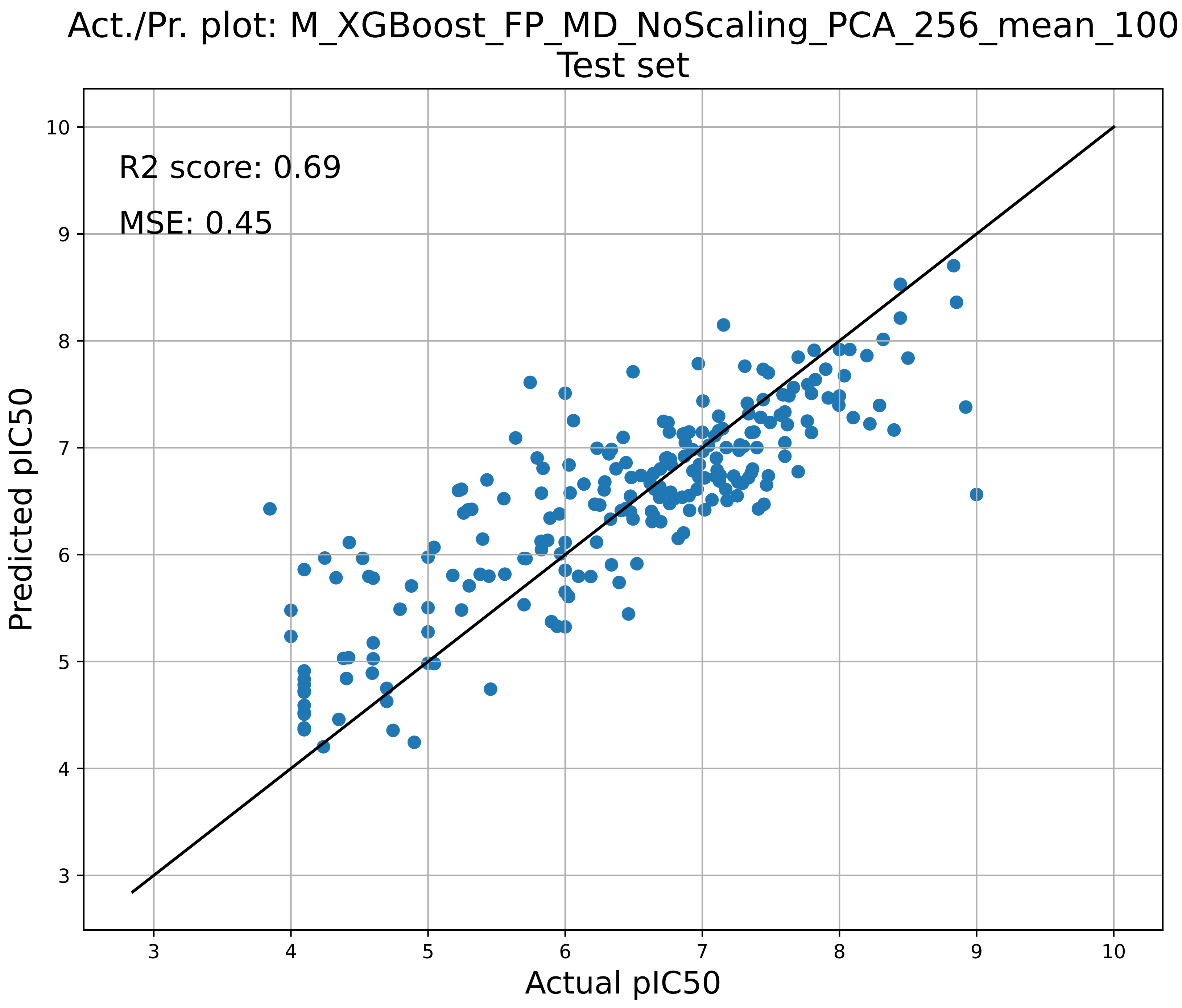

Act./Pr. plot: M_XGBoost_FP_MD_NoScaling_PCA_256_mean_100
Test set
R2 score: 0.69
MSE: 0.45
Predicted pIC50
Actual pIC50
3
4
5
6
7
8
9
10

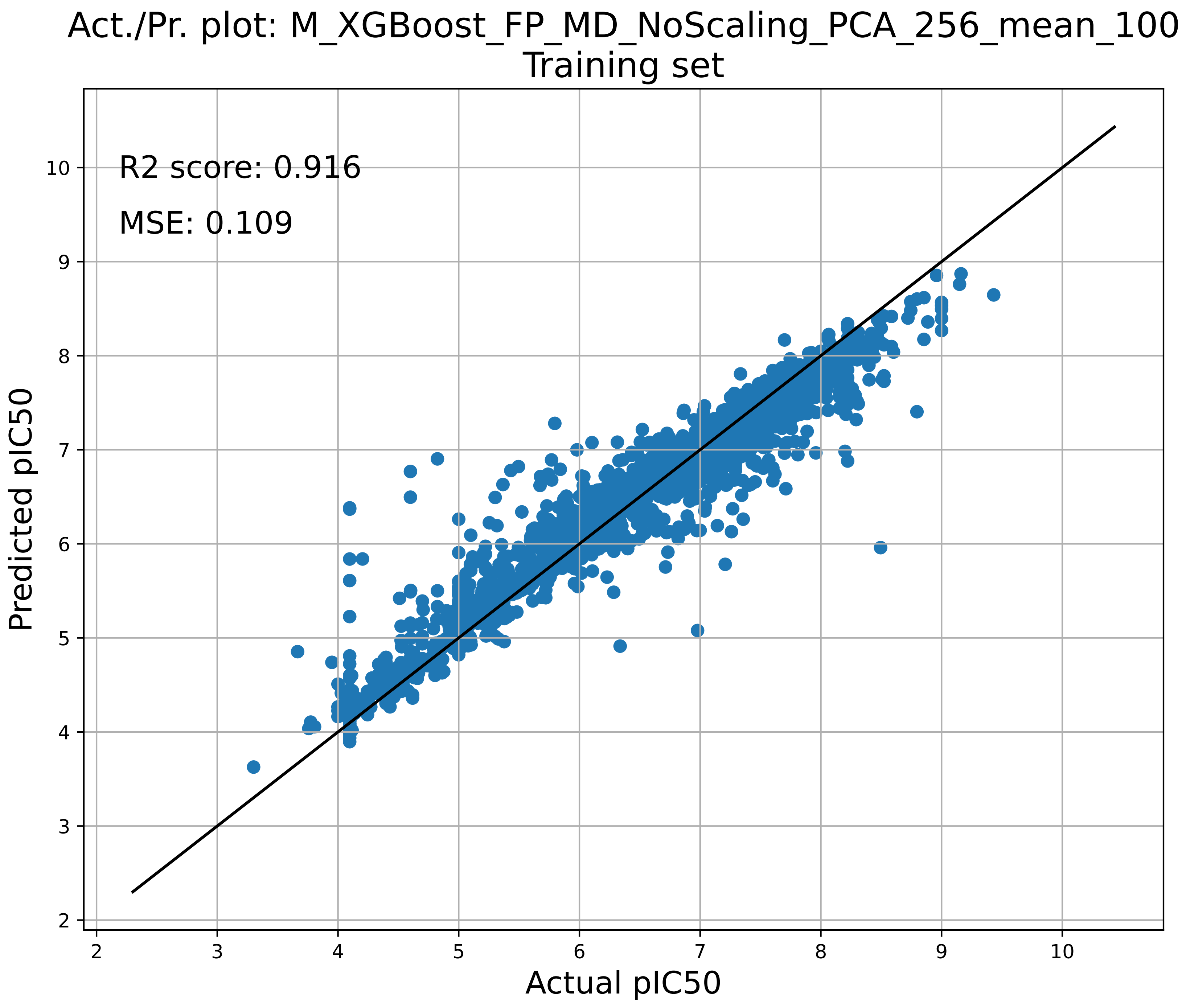

Act./Pr. plot: M_XGBoost_FP_MD_NoScaling_PCA_256_mean_100
Training set
R2 score: 0.916
MSE: 0.109
Predicted pIC50
Actual pIC50
2
3
4
5
6
7
8
9
10

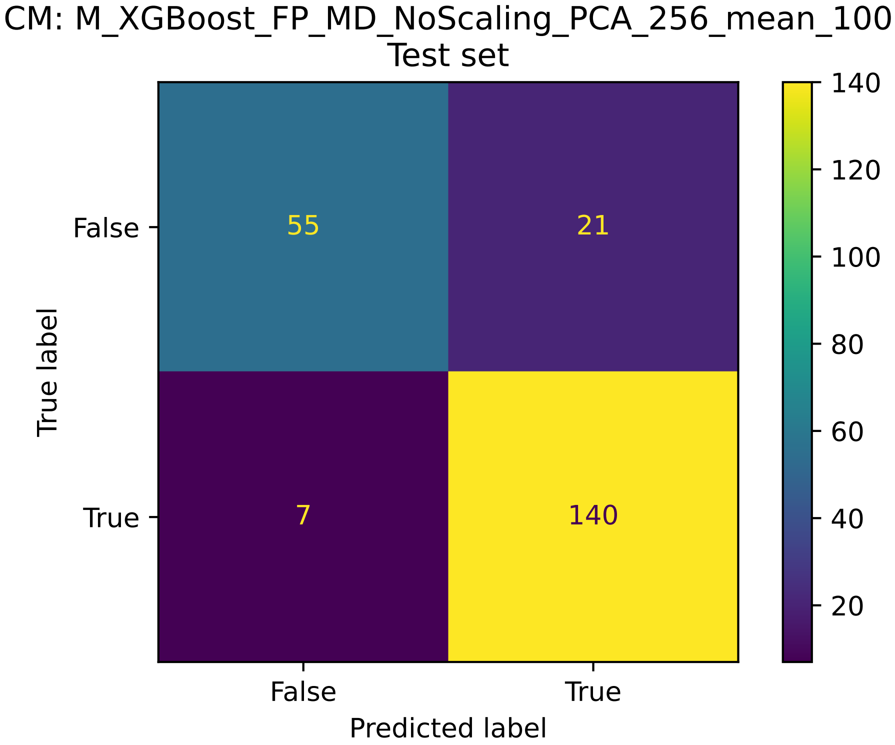
CM: M_XGBoost_FP_MD_NoScaling_PCA_256_mean_100
Test set
True label
Predicted label
False
True
False
True
55
21
7
140
140
120
100
80
60
40
20

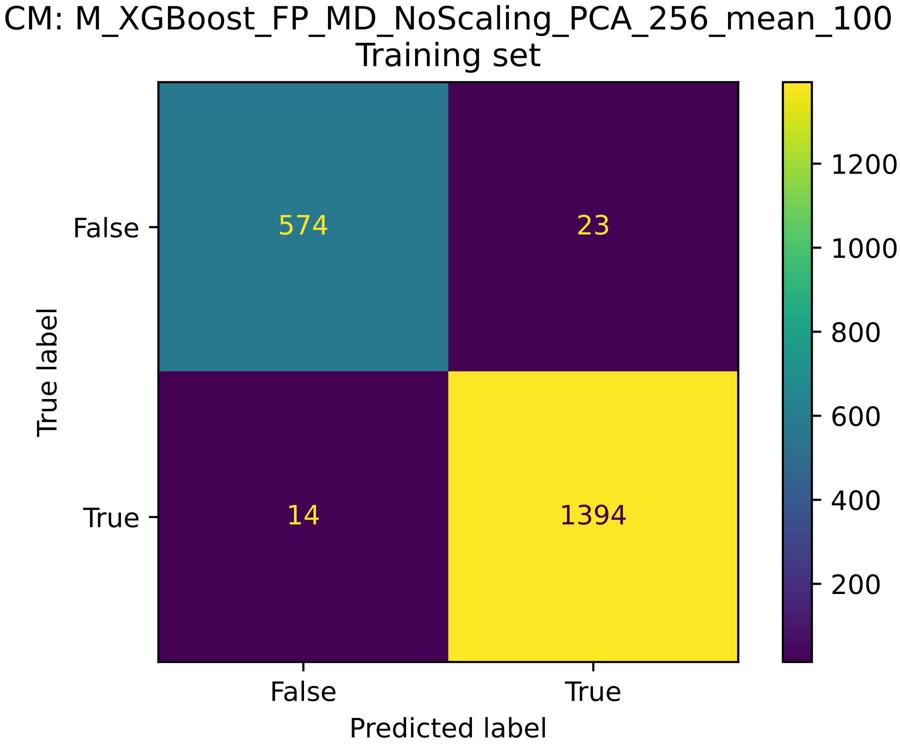

CM: M_XGBoost_FP_MD_NoScaling_PCA_256_mean_100
Training set
574
23
14
1394
False
True
True label
False
True
Predicted label
1200
1000
800
600
400
200

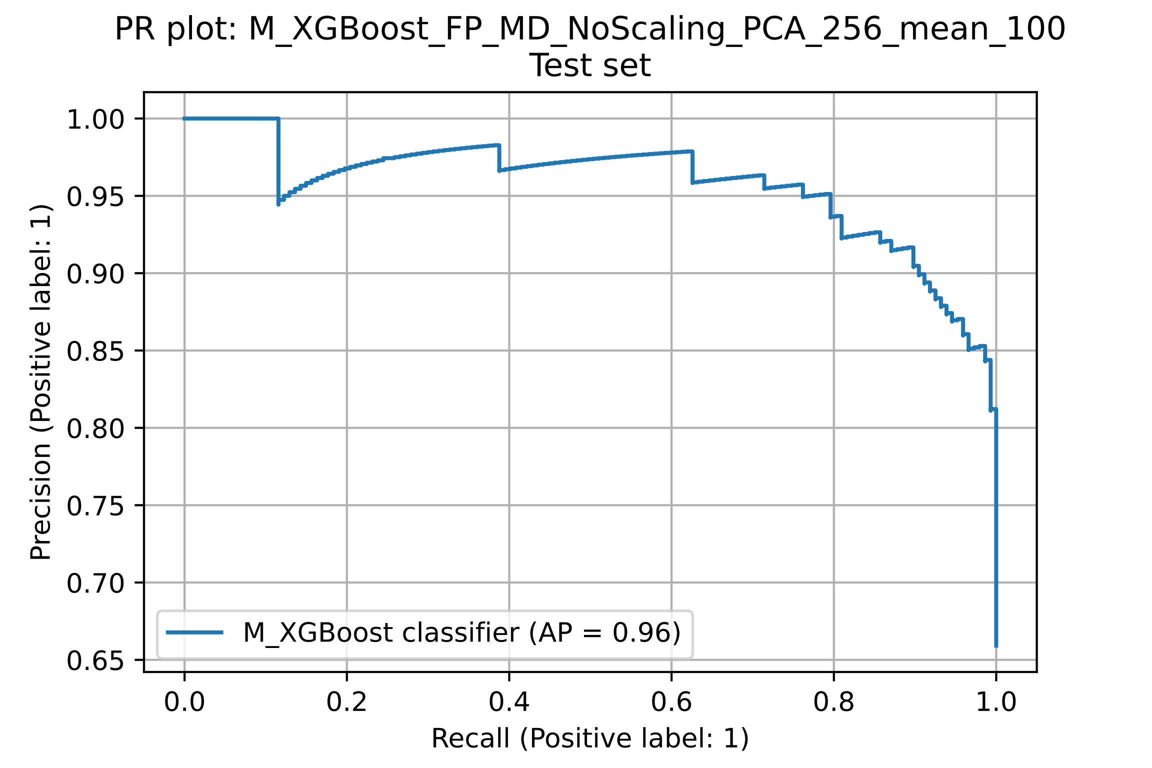
PR plot: M_XGBoost_FP_MD_NoScaling_PCA_256_mean_100
Test set
Precision (Positive label: 1)
Recall (Positive label: 1)
1.00
0.95
0.90
0.85
0.80
0.75
0.70
0.65
0.0
0.2
0.4
0.6
0.8
1.0
M_XGBoost classifier (AP = 0.96)

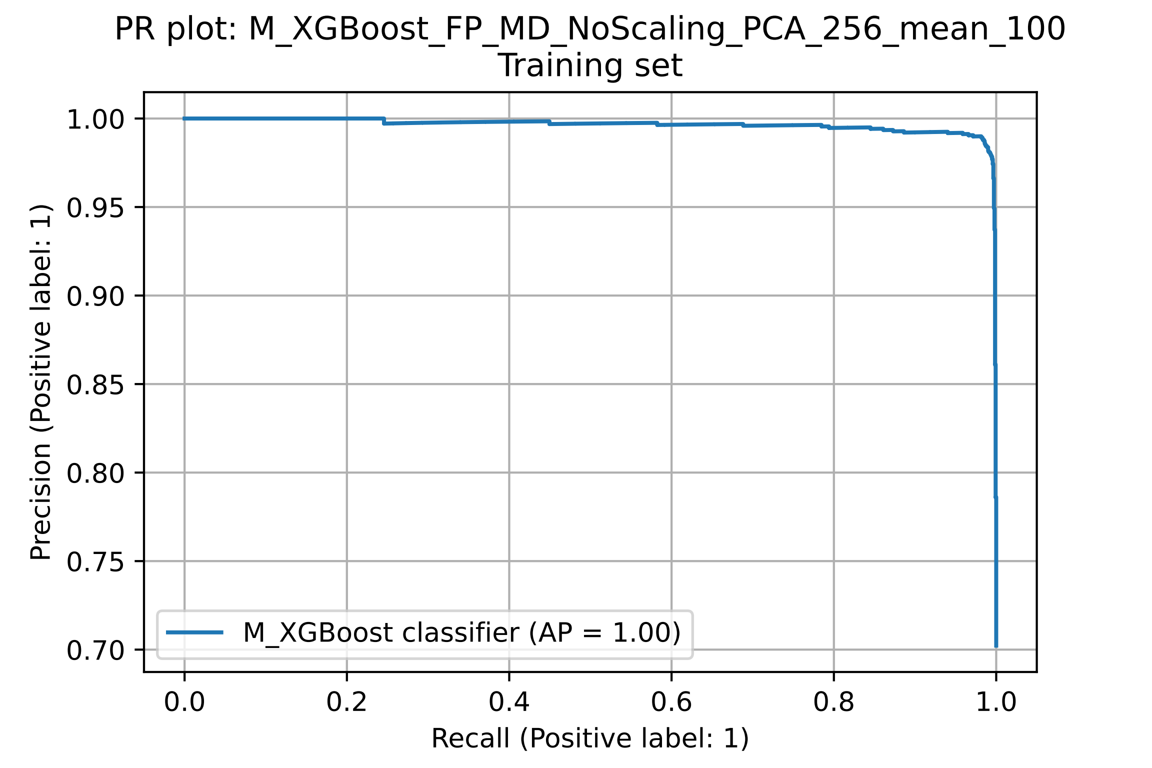

PR plot: M_XGBoost_FP_MD_NoScaling_PCA_256_mean_100
Training set
Precision (Positive label: 1)
1.00
0.95
0.90
0.85
0.80
0.75
0.70
Recall (Positive label: 1)
0.0
0.2
0.4
0.6
0.8
1.0
M_XGBoost classifier (AP = 1.00)

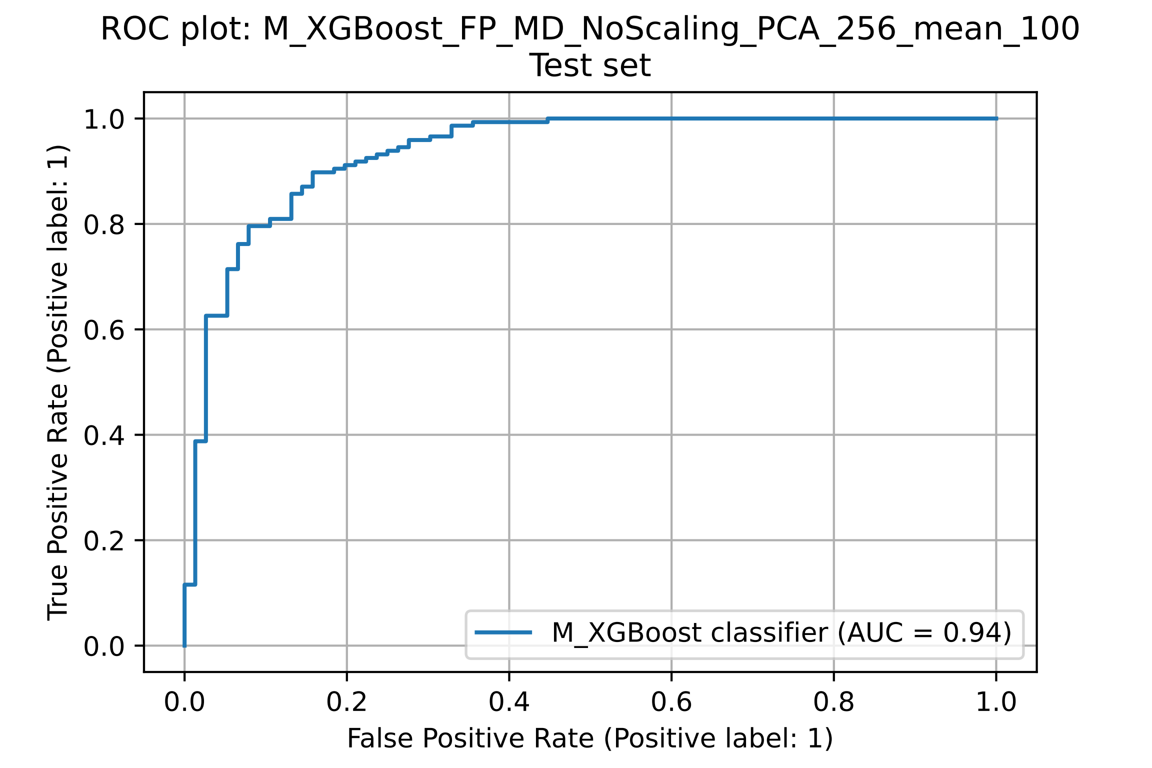
ROC plot: M_XGBoost_FP_MD_NoScaling_PCA_256_mean_100
Test set
True Positive Rate (Positive label: 1)
False Positive Rate (Positive label: 1)
0.0
0.2
0.4
0.6
0.8
1.0
M_XGBoost classifier (AUC = 0.94)

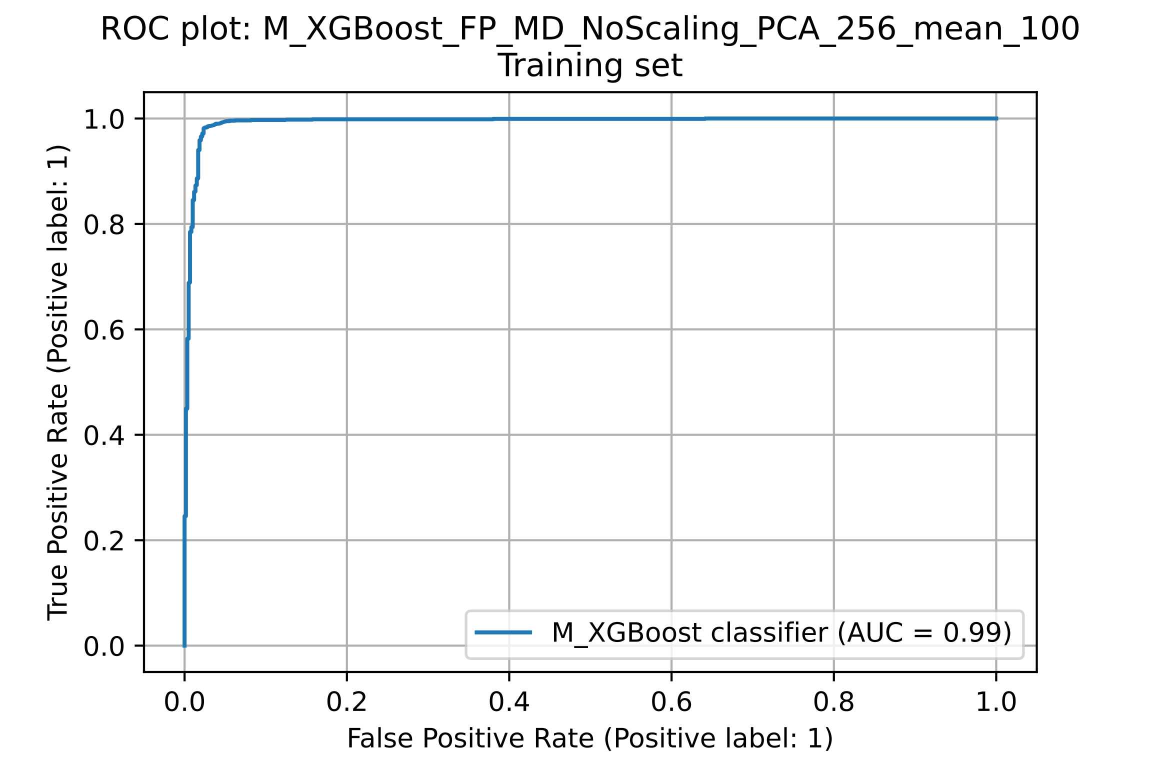
ROC plot: M_XGBoost_FP_MD_NoScaling_PCA_256_mean_100
Training set
True Positive Rate (Positive label: 1)
False Positive Rate (Positive label: 1)
0.0
0.2
0.4
0.6
0.8
1.0
M_XGBoost classifier (AUC = 0.99)

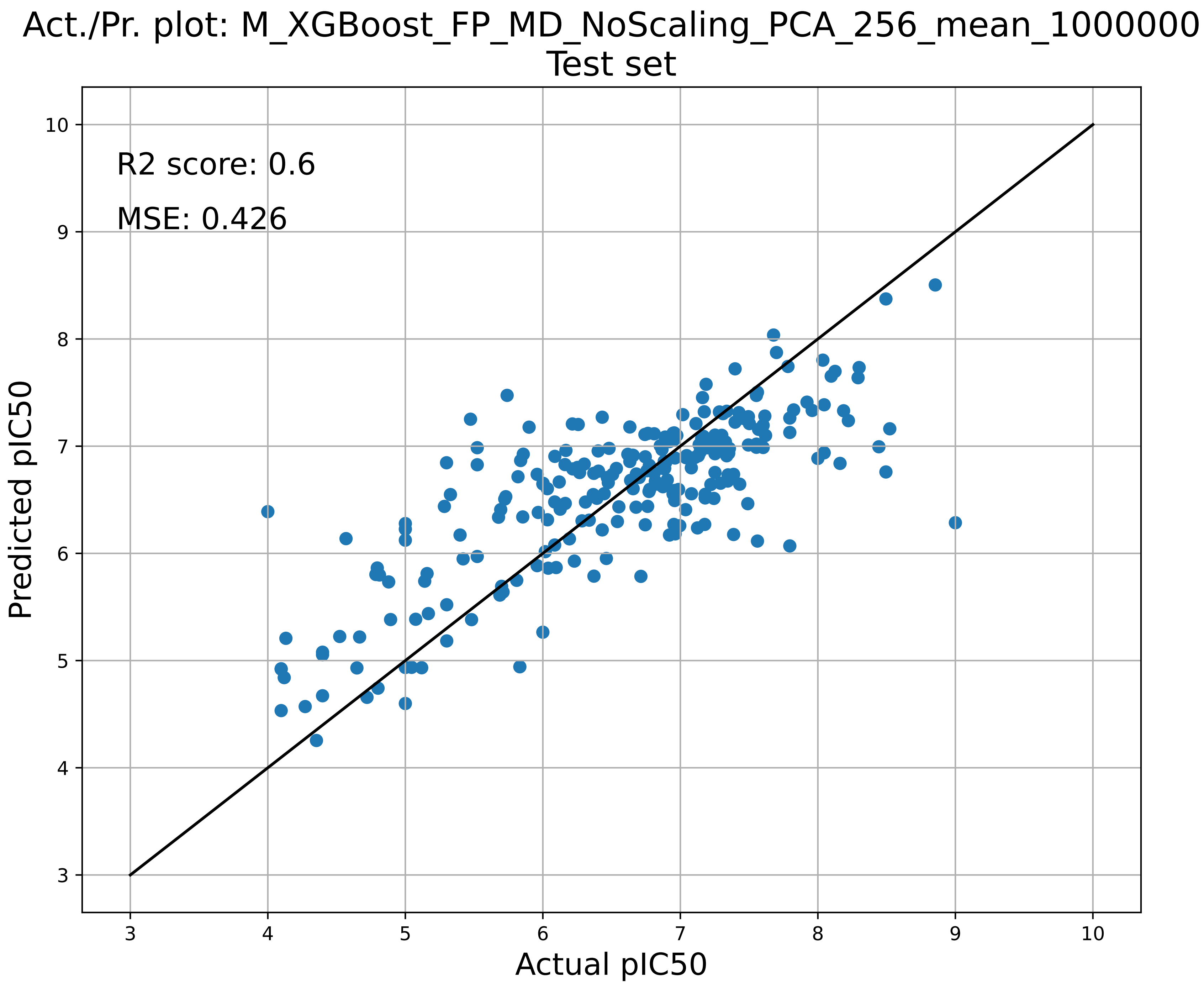
Act./Pr. plot: M_XGBoost_FP_MD_NoScaling_PCA_256_mean_1000000
Test set
R2 score: 0.6
MSE: 0.426
Predicted pIC50
Actual pIC50
3
4
5
6
7
8
9
10

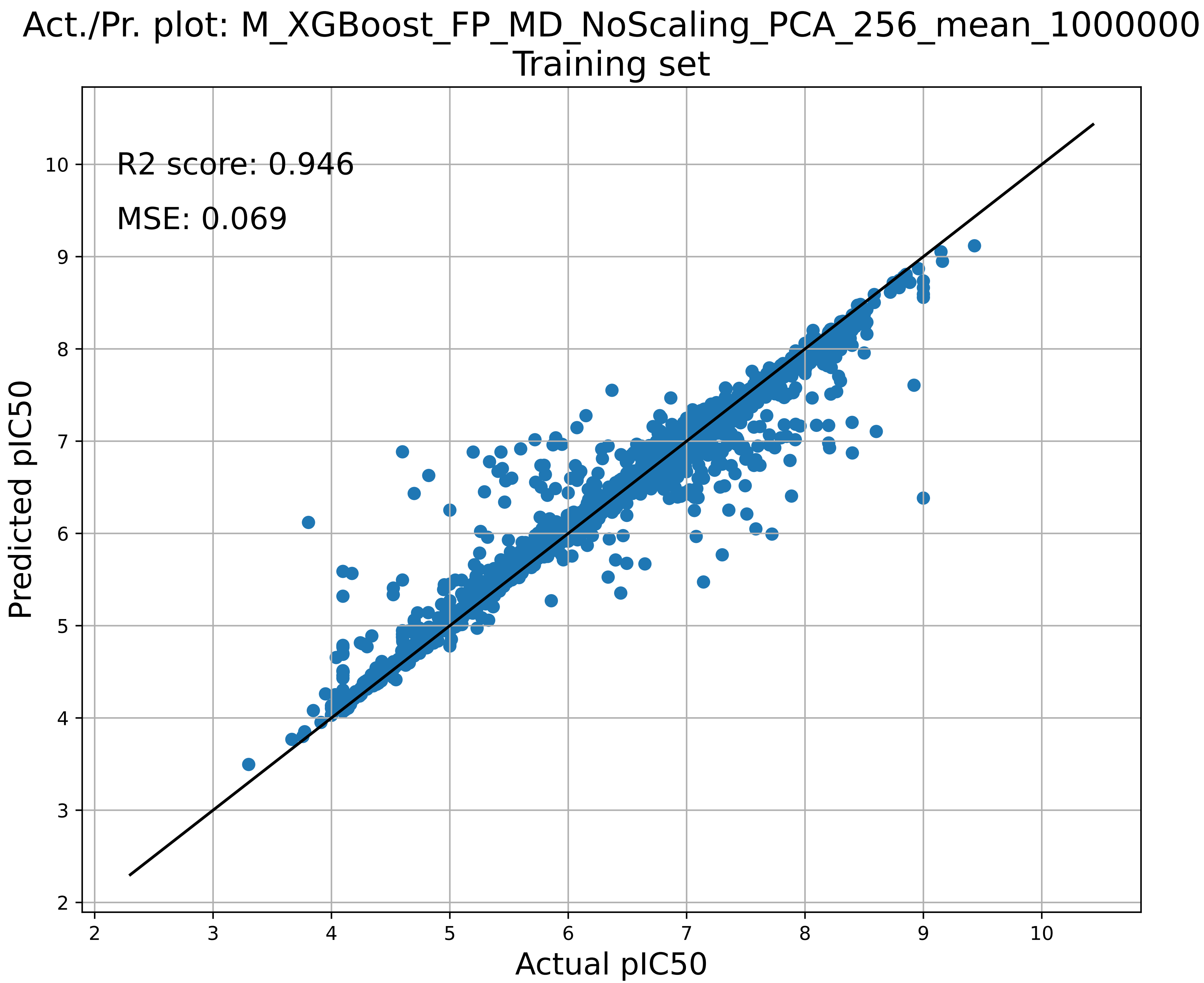

Act./Pr. plot: M_XGBoost_FP_MD_NoScaling_PCA_256_mean_1000000
Training set
R2 score: 0.946
MSE: 0.069
Predicted pIC50
Actual pIC50
2
3
4
5
6
7
8
9
10

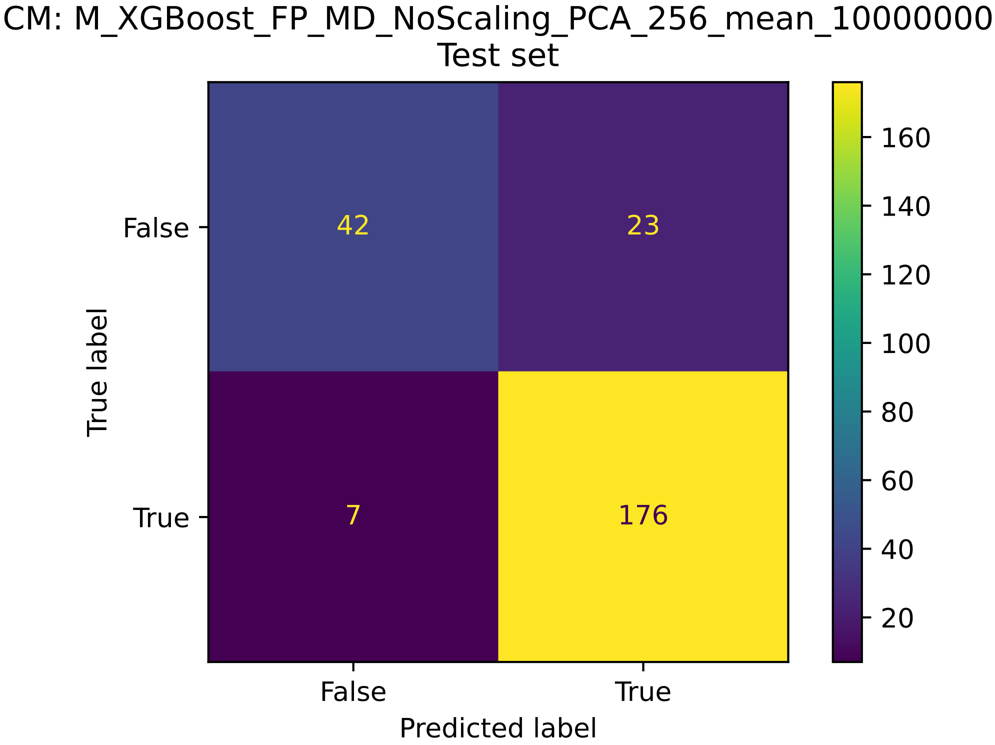
CM: M_XGBoost_FP_MD_NoScaling_PCA_256_mean_10000000
Test set
True label
False
True
42
23
7
176
Predicted label
False
True
160
140
120
100
80
60
40
20

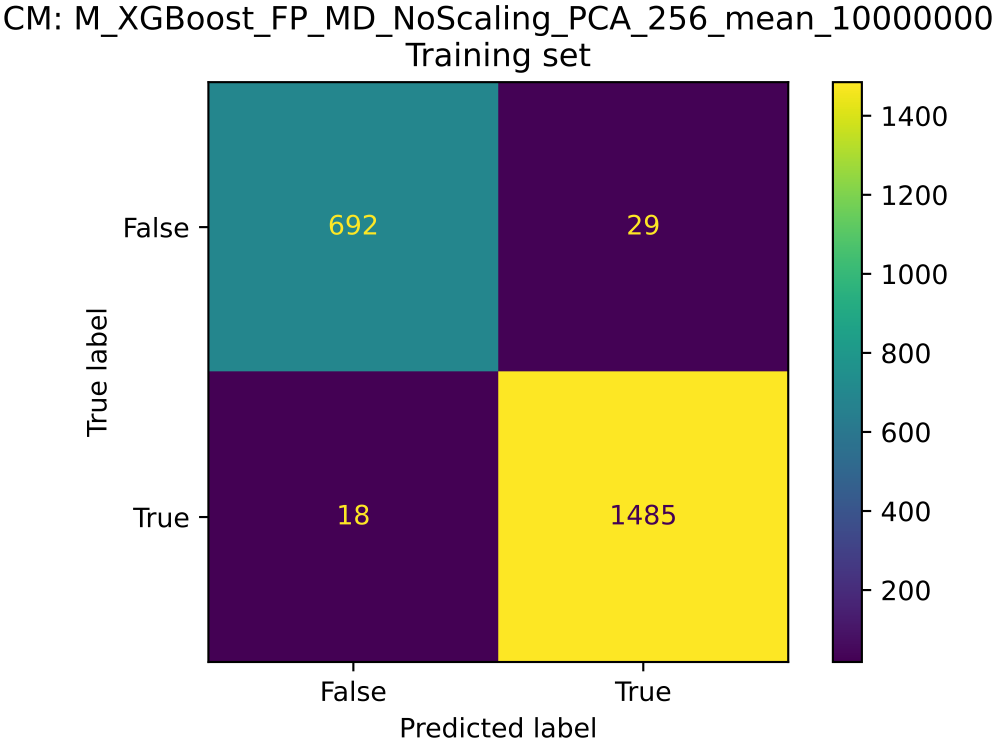

CM: M_XGBoost_FP_MD_NoScaling_PCA_256_mean_10000000
Training set
False
True
692
29
18
1485
True label
Predicted label
False
True
1400
1200
1000
800
600
400
200

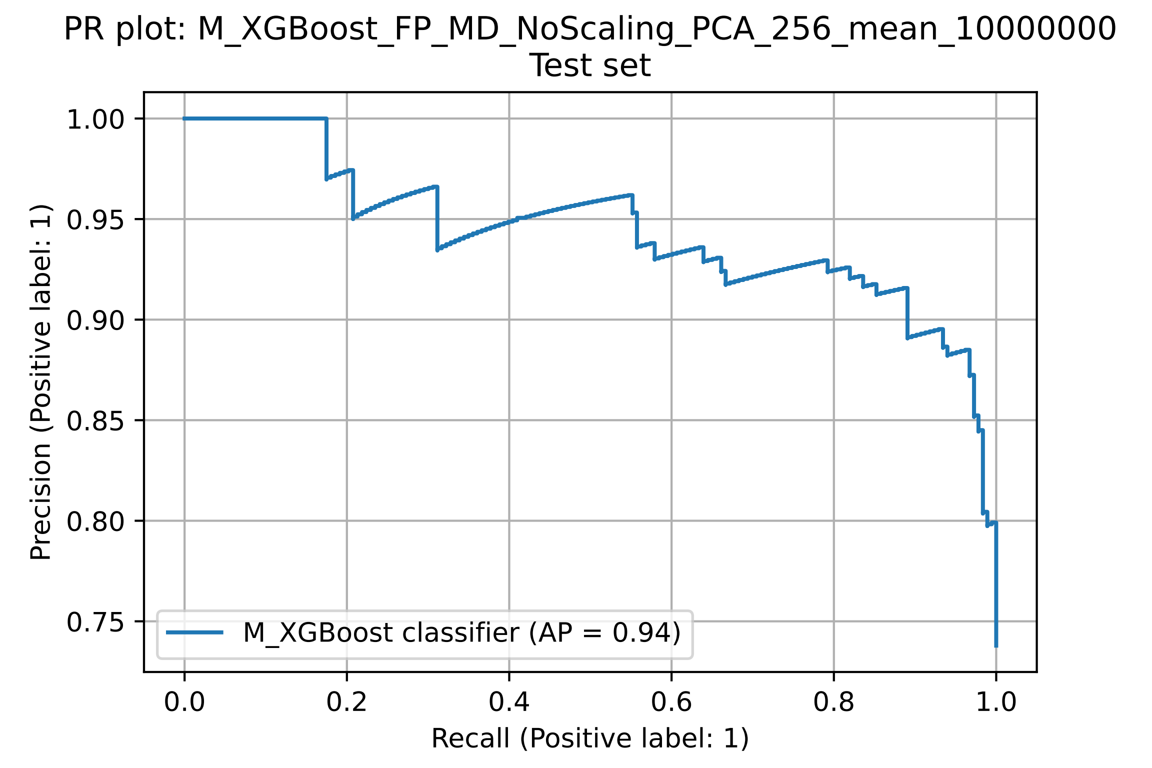

PR plot: M_XGBoost_FP_MD_NoScaling_PCA_256_mean_10000000
Test set
Precision (Positive label: 1)
1.00
0.95
0.90
0.85
0.80
0.75
0.0
0.2
0.4
0.6
0.8
1.0
Recall (Positive label: 1)
M_XGBoost classifier (AP = 0.94)

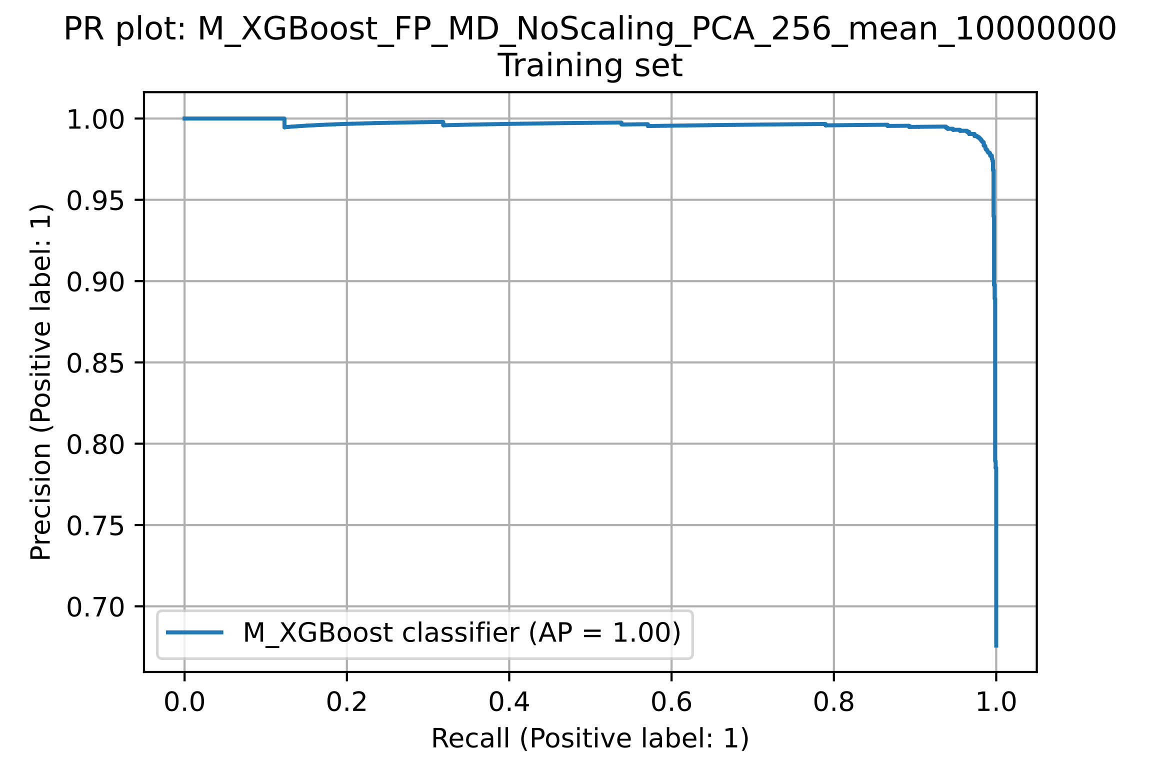
PR plot: M_XGBoost_FP_MD_NoScaling_PCA_256_mean_10000000
Training set
Precision (Positive label: 1)
Recall (Positive label: 1)
1.00
0.95
0.90
0.85
0.80
0.75
0.70
0.0
0.2
0.4
0.6
0.8
1.0
M_XGBoost classifier (AP = 1.00)

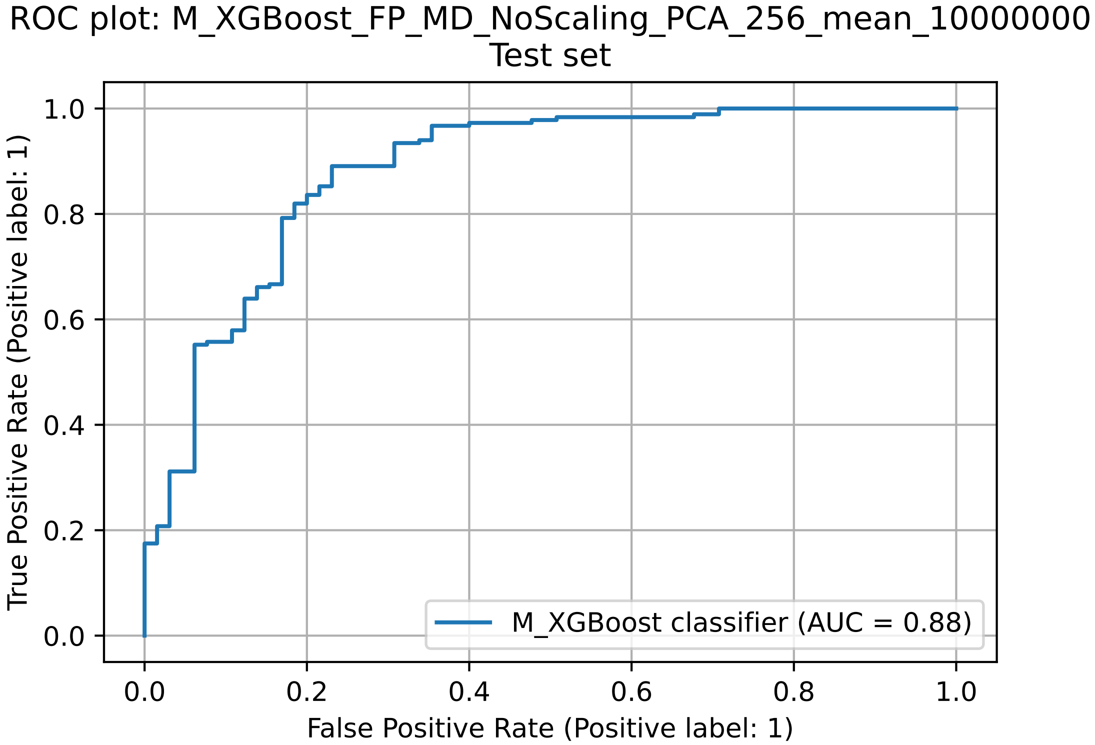
ROC plot: M_XGBoost_FP_MD_NoScaling_PCA_256_mean_10000000
Test set
True Positive Rate (Positive label: 1)
False Positive Rate (Positive label: 1)
0.0
0.2
0.4
0.6
0.8
1.0
M_XGBoost classifier (AUC = 0.88)

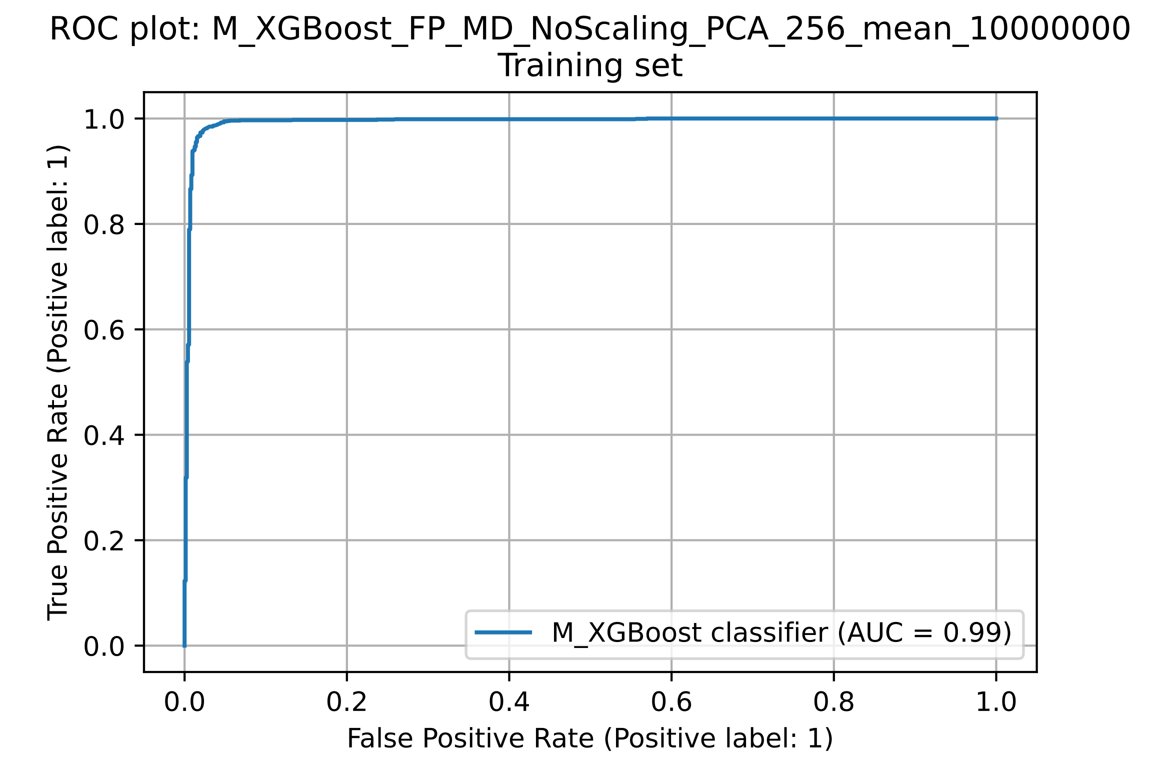

ROC plot: M_XGBoost_FP_MD_NoScaling_PCA_256_mean_10000000
Training set
True Positive Rate (Positive label: 1)
False Positive Rate (Positive label: 1)
0.0
0.2
0.4
0.6
0.8
1.0
M_XGBoost classifier (AUC = 0.99)

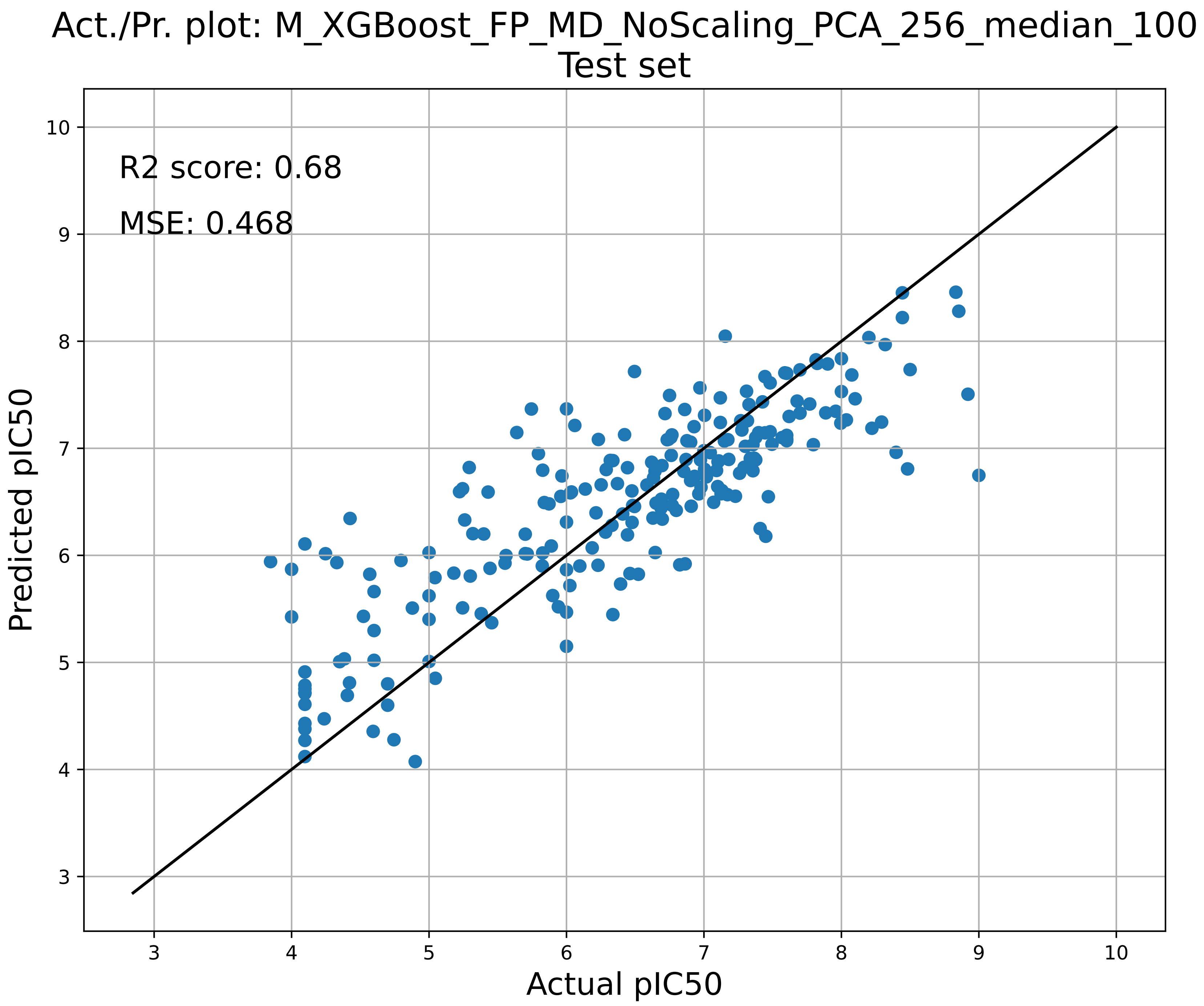
Act./Pr. plot: M_XGBoost_FP_MD_NoScaling_PCA_256_median_100
Test set
R2 score: 0.68
MSE: 0.468
Predicted pIC50
Actual pIC50
3
4
5
6
7
8
9
10

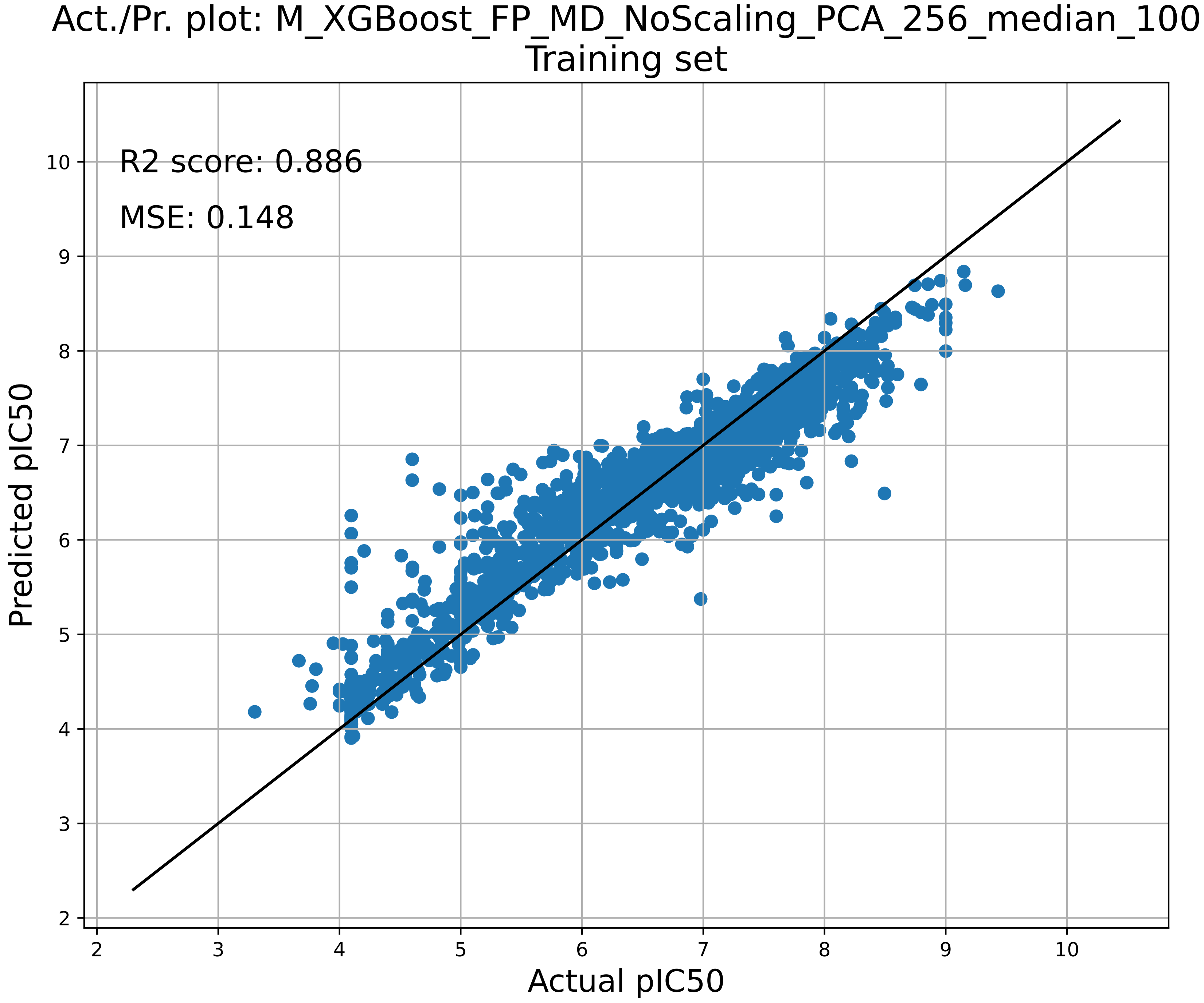
Act./Pr. plot: M_XGBoost_FP_MD_NoScaling_PCA_256_median_100
Training set
R2 score: 0.886
MSE: 0.148
Predicted pIC50
Actual pIC50
2
3
4
5
6
7
8
9
10

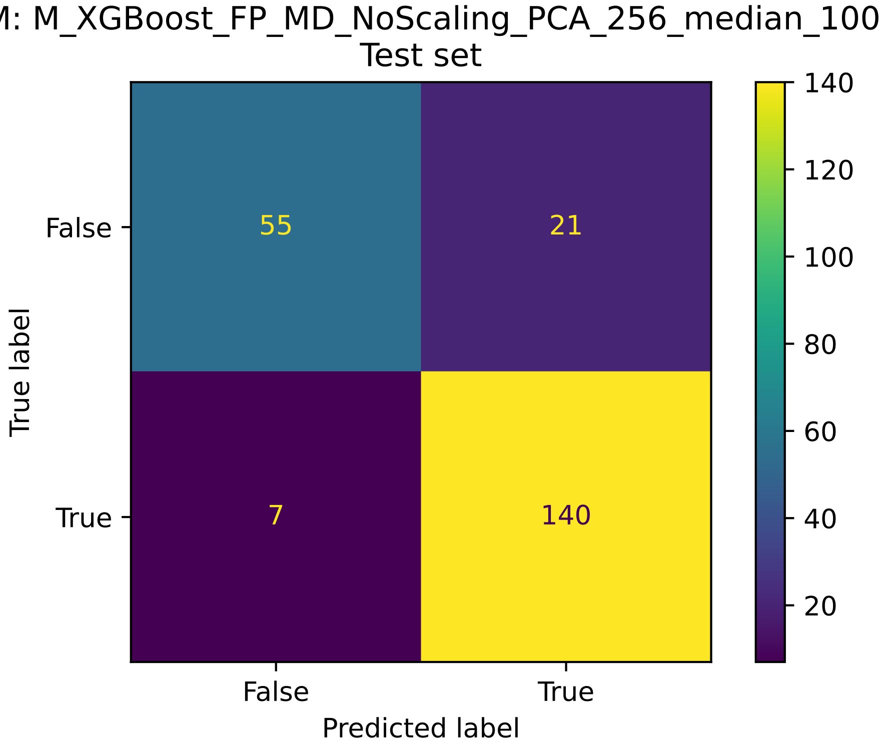

CM: M_XGBoost_FP_MD_NoScaling_PCA_256_median_100
Test set
True label
Predicted label
False
True
False
True
55
21
7
140
140
120
100
80
60
40
20

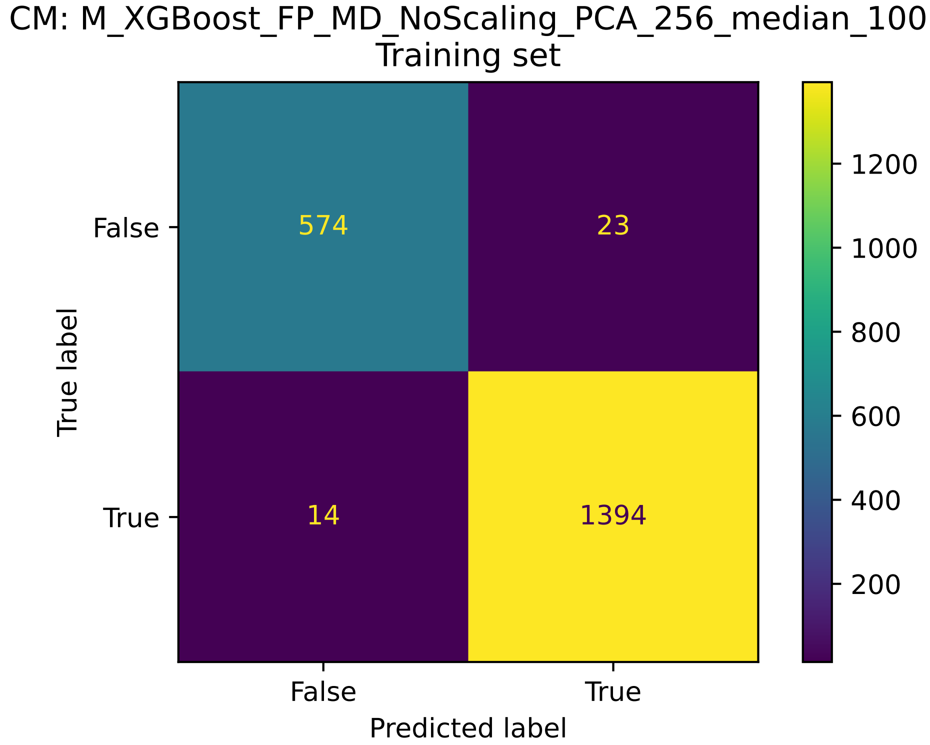
CM: M_XGBoost_FP_MD_NoScaling_PCA_256_median_100
Training set
True label
False
True
574
23
14
1394
Predicted label
False
True
1200
1000
800
600
400
200

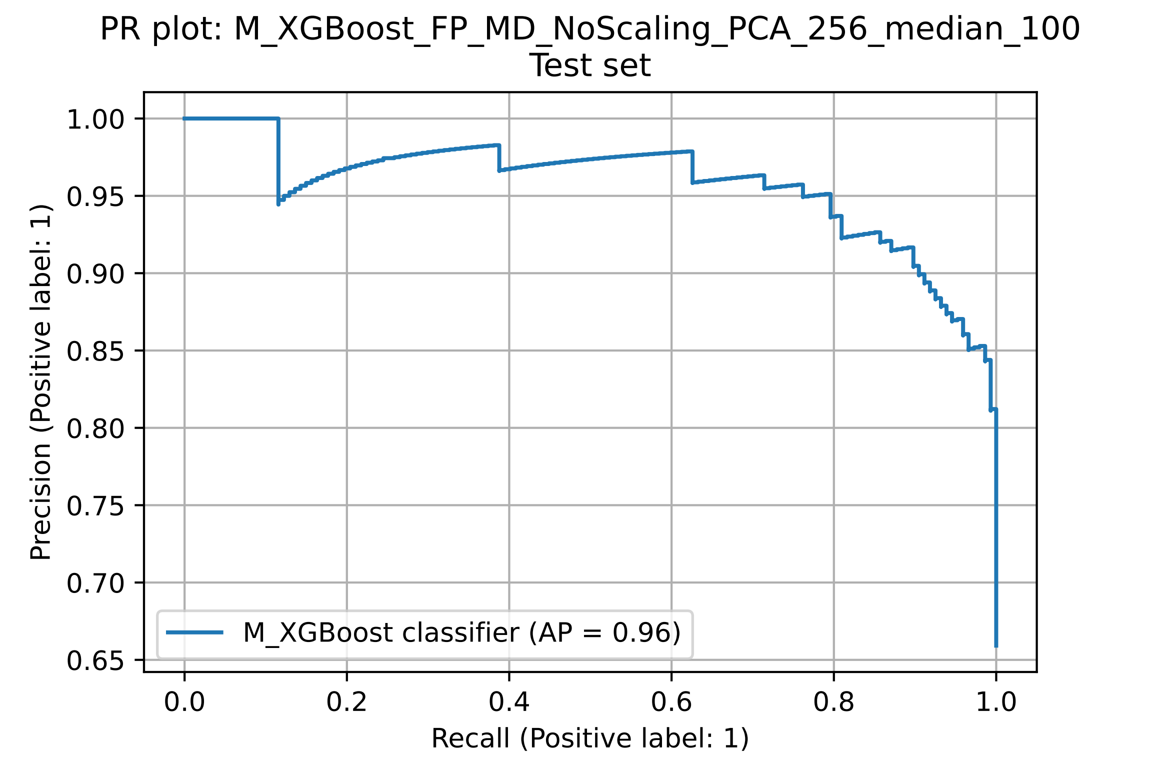
PR plot: M_XGBoost_FP_MD_NoScaling_PCA_256_median_100
Test set
Precision (Positive label: 1)
Recall (Positive label: 1)
1.00
0.95
0.90
0.85
0.80
0.75
0.70
0.65
0.0
0.2
0.4
0.6
0.8
1.0
M_XGBoost classifier (AP = 0.96)

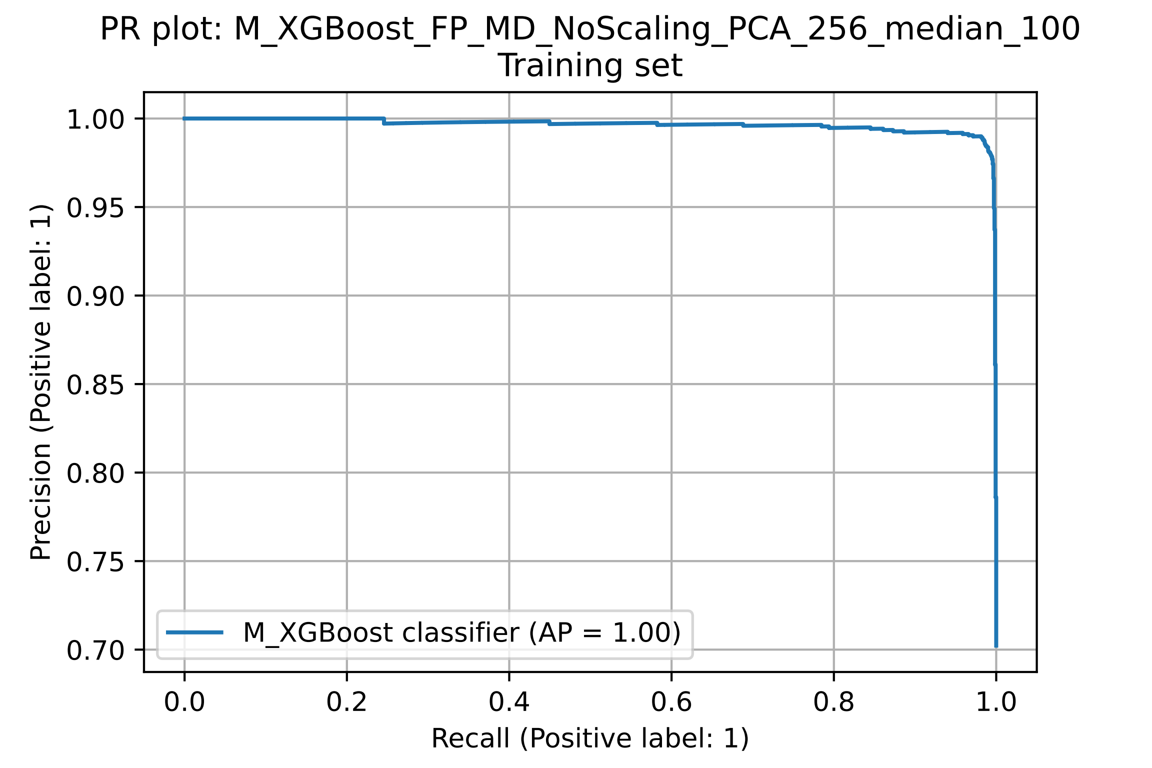

PR plot: M_XGBoost_FP_MD_NoScaling_PCA_256_median_100
Training set
1.00
0.95
0.90
0.85
0.80
0.75
0.70
Precision (Positive label: 1)
0.0
0.2
0.4
0.6
0.8
1.0
Recall (Positive label: 1)
M_XGBoost classifier (AP = 1.00)

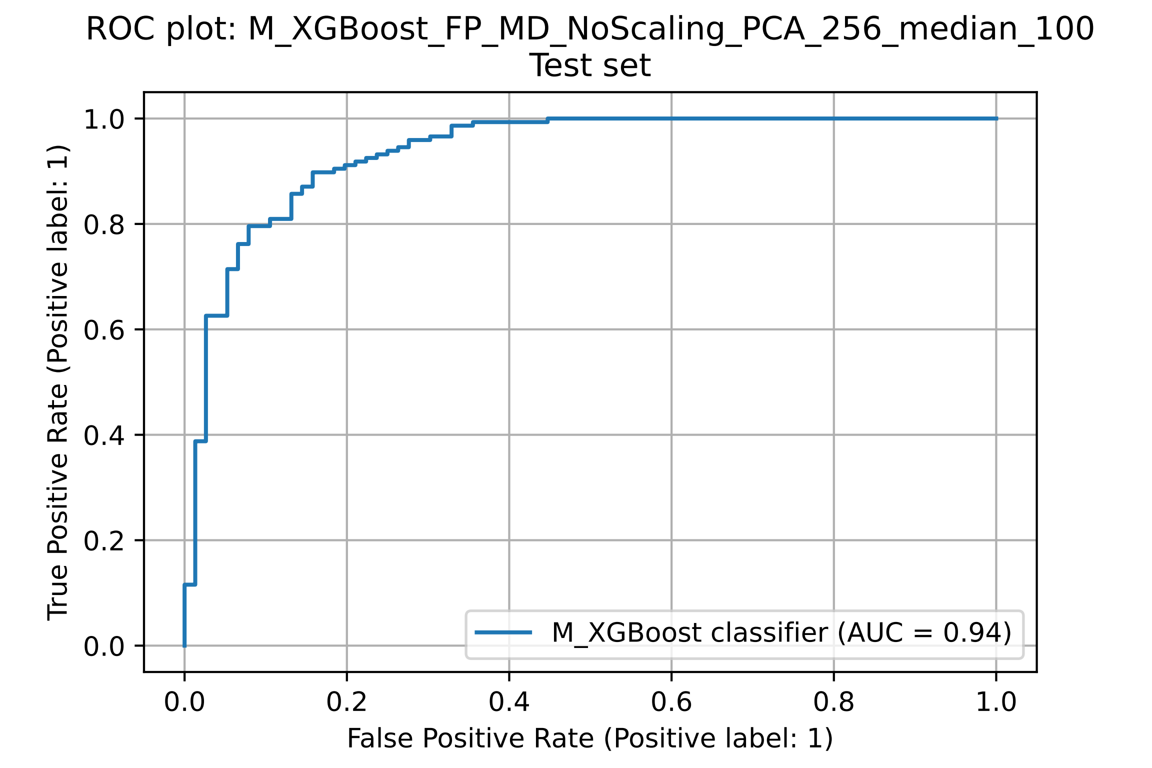
ROC plot: M_XGBoost_FP_MD_NoScaling_PCA_256_median_100
Test set
True Positive Rate (Positive label: 1)
False Positive Rate (Positive label: 1)
0.0
0.2
0.4
0.6
0.8
1.0
M_XGBoost classifier (AUC = 0.94)

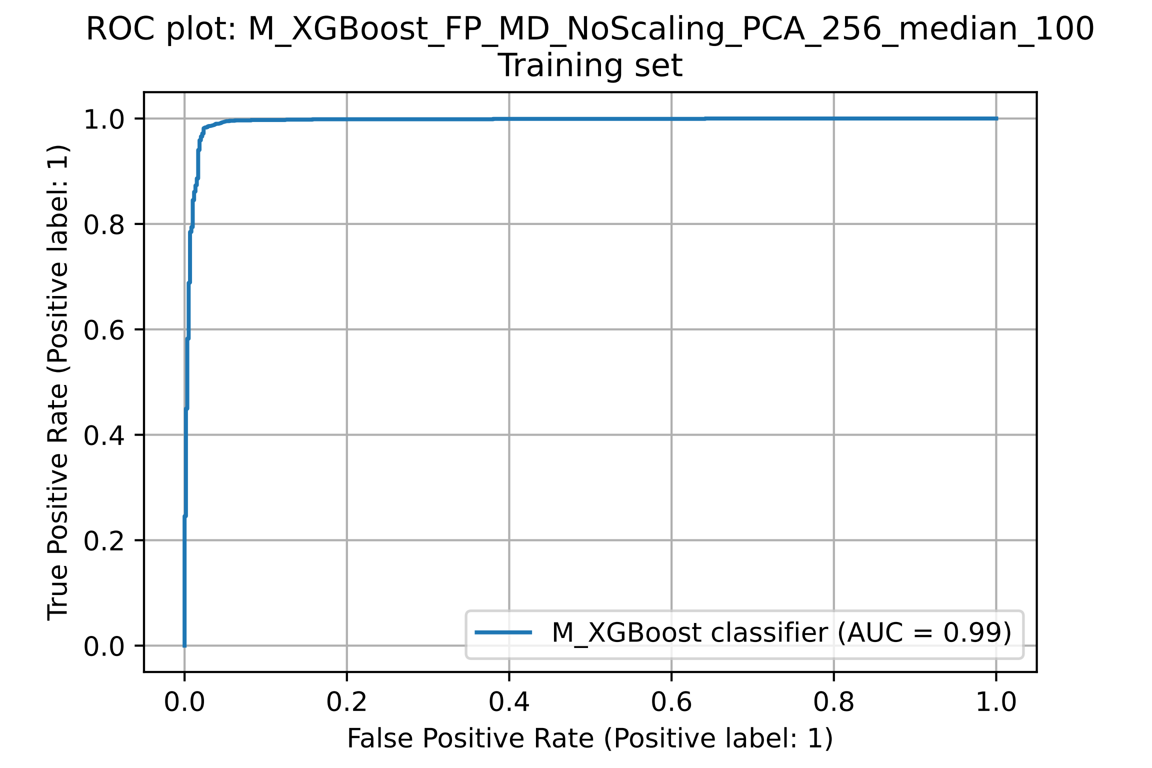
ROC plot: M_XGBoost_FP_MD_NoScaling_PCA_256_median_100
Training set
True Positive Rate (Positive label: 1)
False Positive Rate (Positive label: 1)
0.0
0.2
0.4
0.6
0.8
1.0
M_XGBoost classifier (AUC = 0.99)

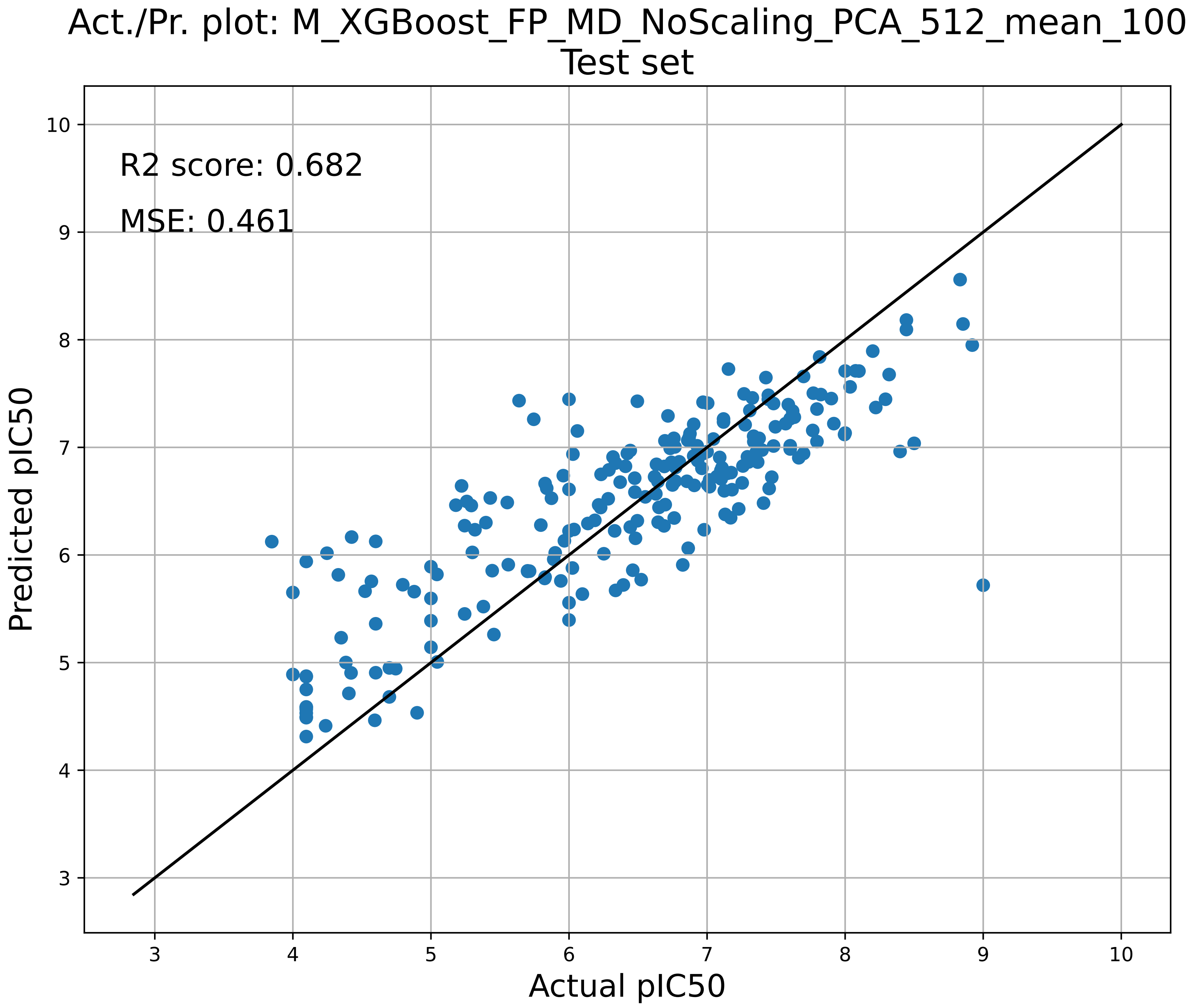
Act./Pr. plot: M_XGBoost_FP_MD_NoScaling_PCA_512_mean_100
Test set
R2 score: 0.682
MSE: 0.461
Predicted pIC50
Actual pIC50
3
4
5
6
7
8
9
10

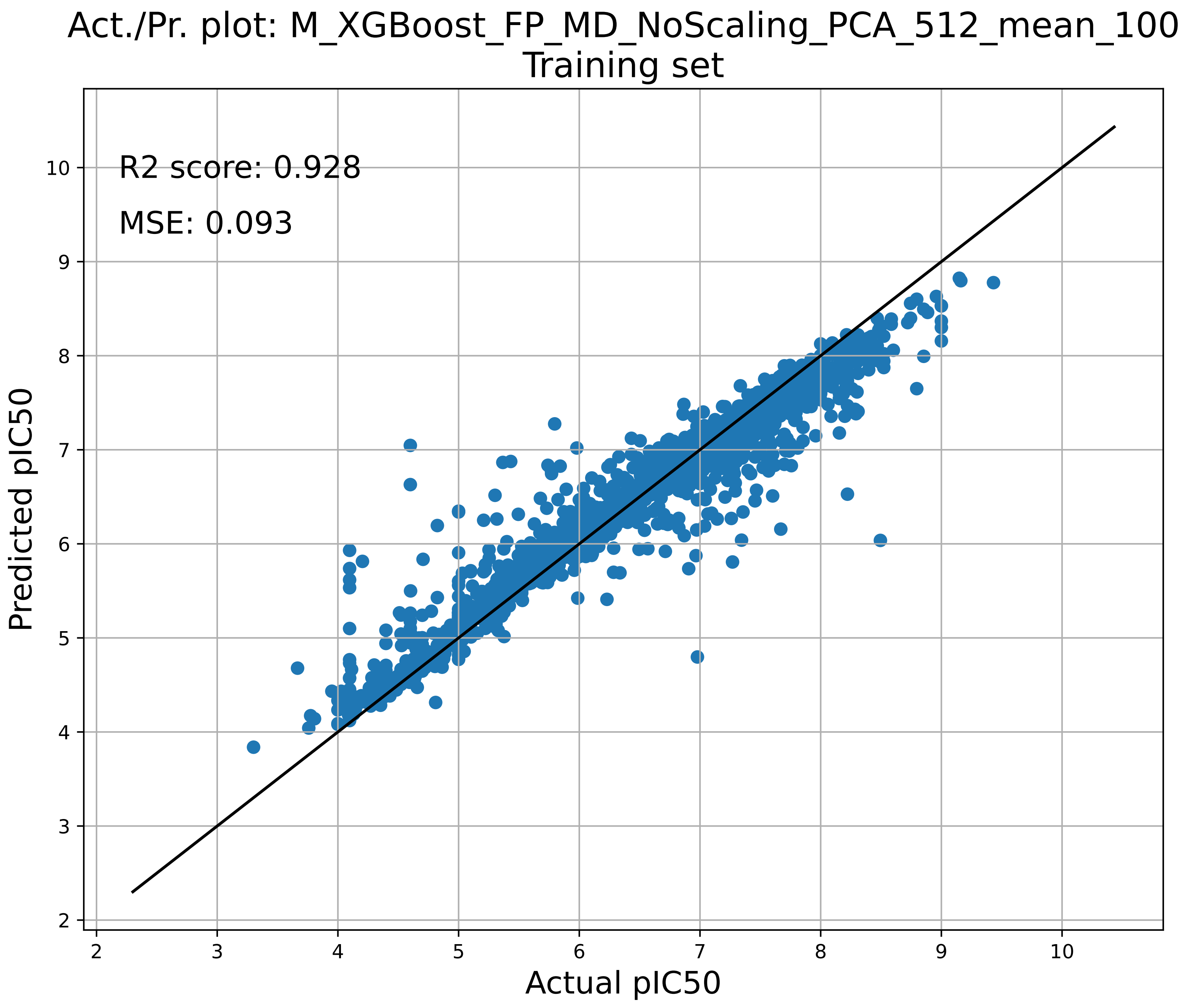

Act./Pr. plot: M_XGBoost_FP_MD_NoScaling_PCA_512_mean_100
Training set
R2 score: 0.928
MSE: 0.093
Predicted pIC50
Actual pIC50
2
3
4
5
6
7
8
9
10

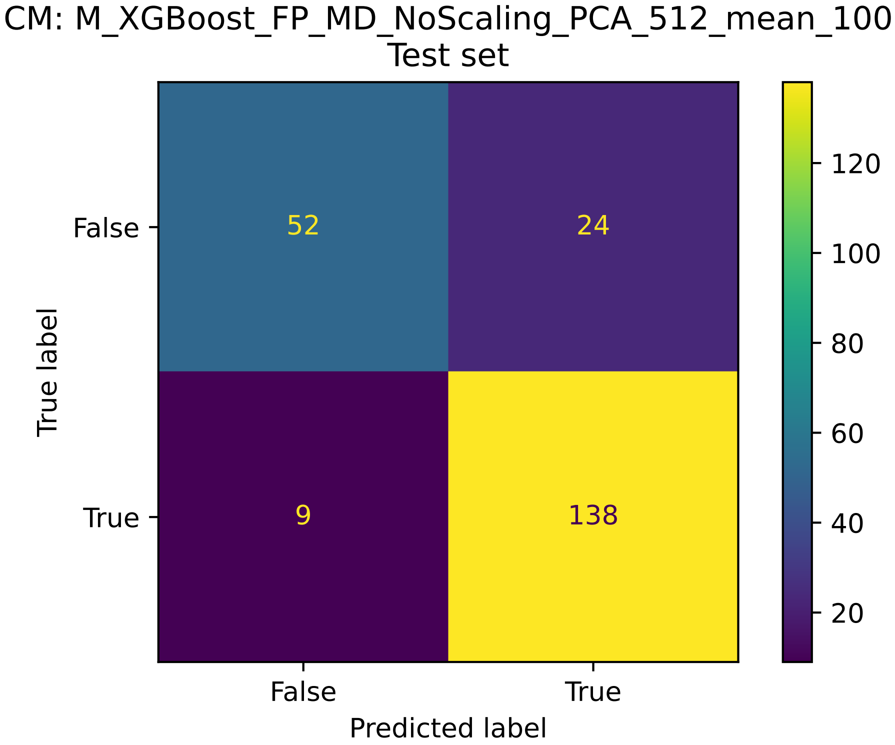
CM: M_XGBoost_FP_MD_NoScaling_PCA_512_mean_100
Test set
True label
False
True
52
24
9
138
False
True
Predicted label
120
100
80
60
40
20

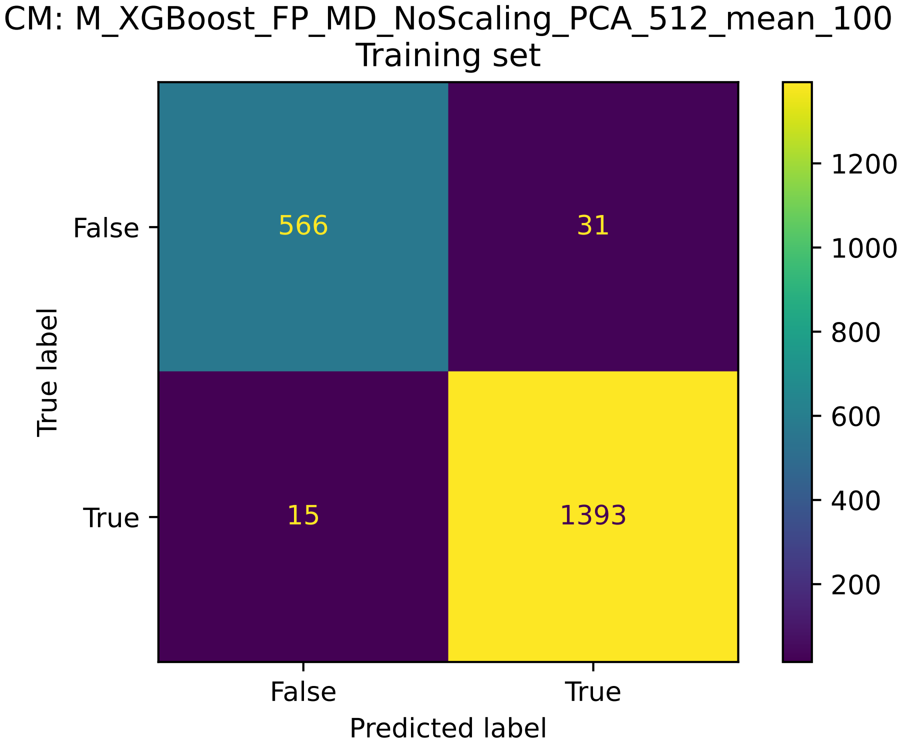
CM: M_XGBoost_FP_MD_NoScaling_PCA_512_mean_100
Training set
566
31
15
1393
False
True
True label
False
True
Predicted label
1200
1000
800
600
400
200

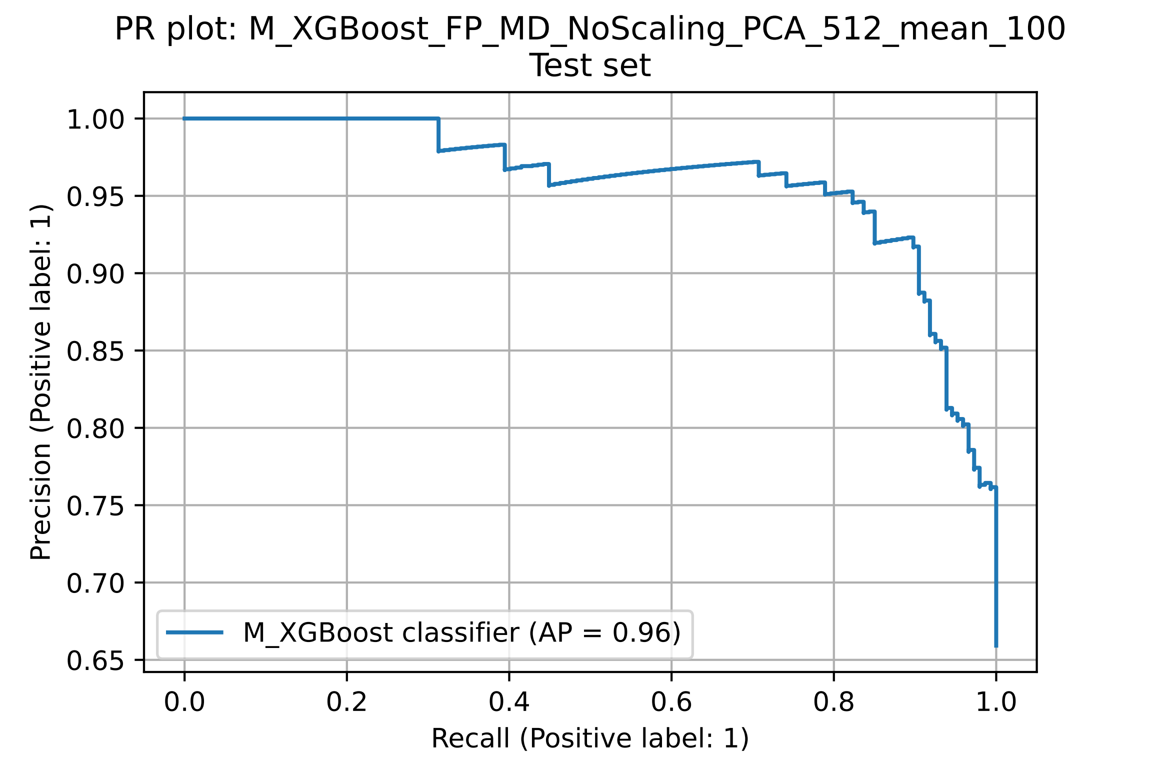
PR plot: M_XGBoost_FP_MD_NoScaling_PCA_512_mean_100
Test set
Precision (Positive label: 1)
Recall (Positive label: 1)
1.00
0.95
0.90
0.85
0.80
0.75
0.70
0.65
0.0
0.2
0.4
0.6
0.8
1.0
M_XGBoost classifier (AP = 0.96)

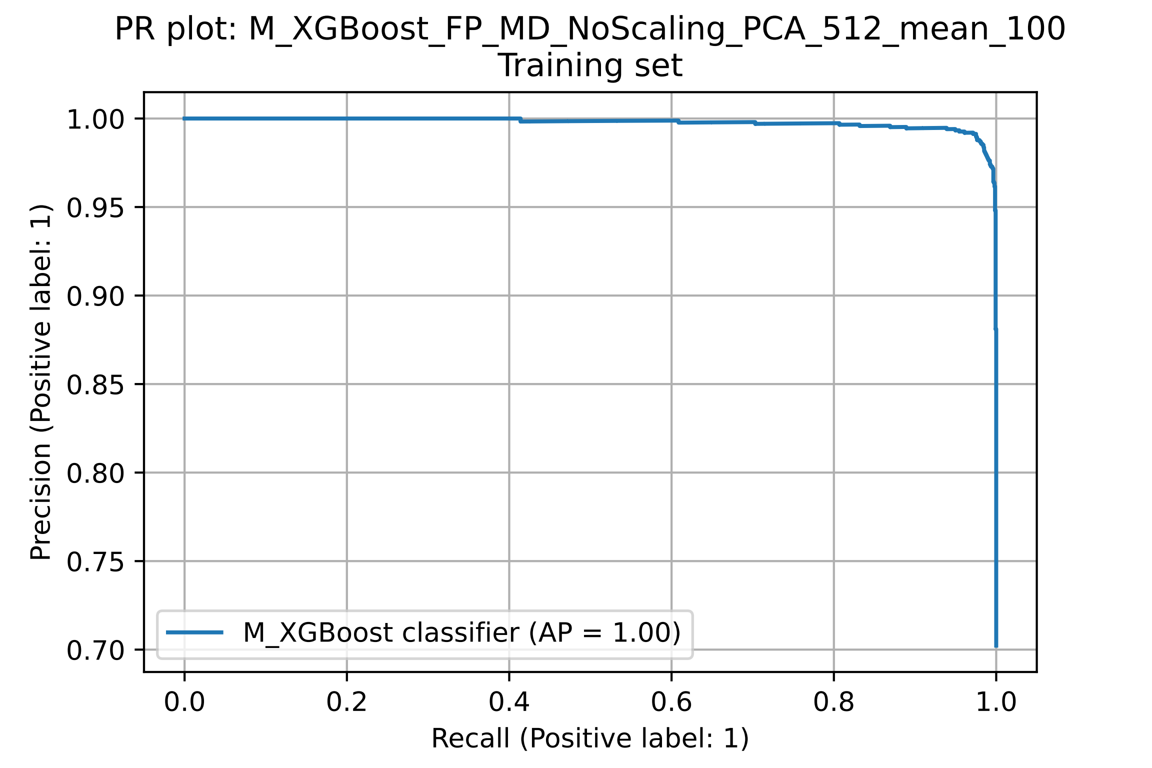
PR plot: M_XGBoost_FP_MD_NoScaling_PCA_512_mean_100
Training set
Precision (Positive label: 1)
Recall (Positive label: 1)
1.00
0.95
0.90
0.85
0.80
0.75
0.70
0.0
0.2
0.4
0.6
0.8
1.0
M_XGBoost classifier (AP = 1.00)

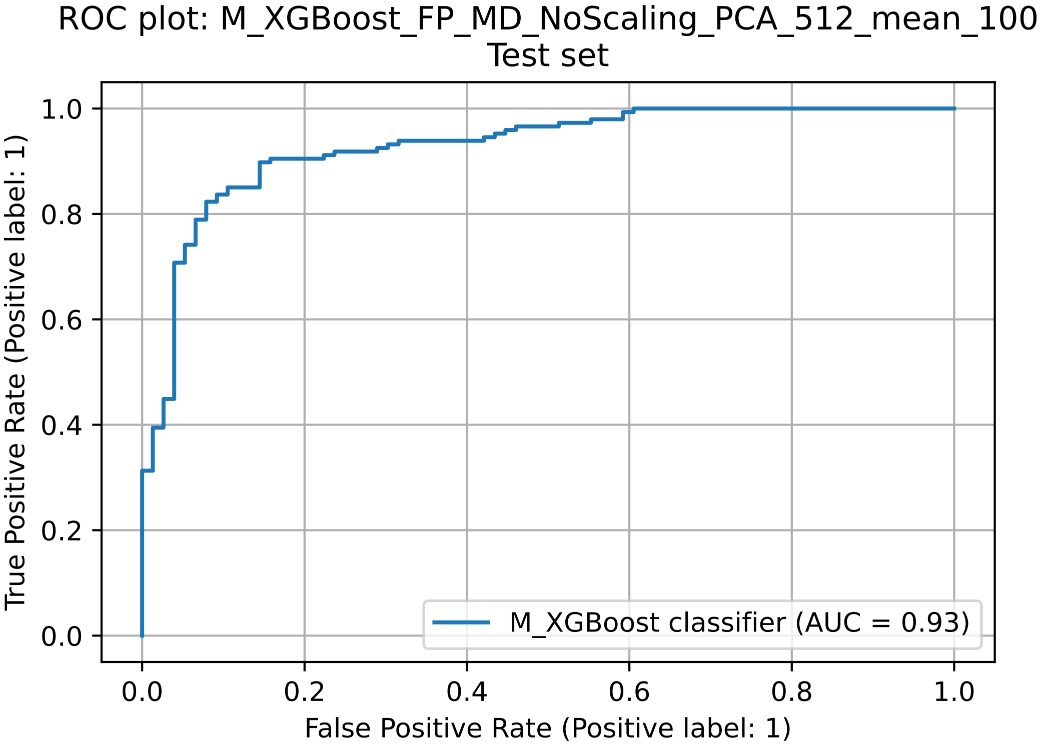
ROC plot: M_XGBoost_FP_MD_NoScaling_PCA_512_mean_100
Test set
True Positive Rate (Positive label: 1)
False Positive Rate (Positive label: 1)
M_XGBoost classifier (AUC = 0.93)
0.0
0.2
0.4
0.6
0.8
1.0

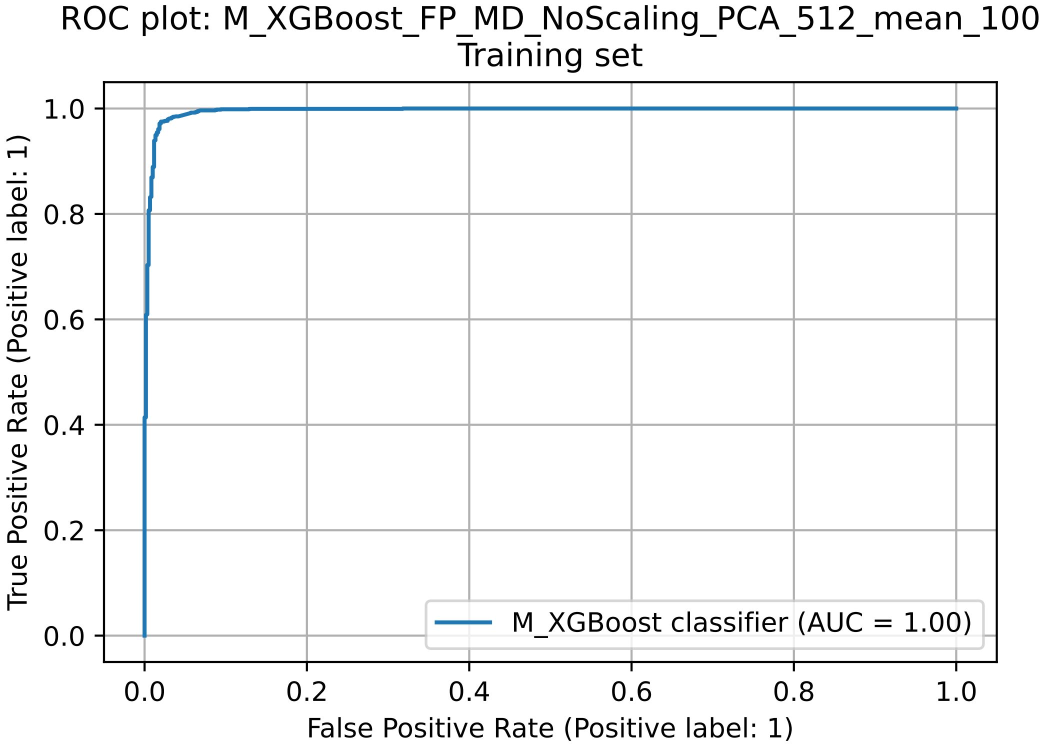
ROC plot: M_XGBoost_FP_MD_NoScaling_PCA_512_mean_100
Training set
True Positive Rate (Positive label: 1)
False Positive Rate (Positive label: 1)
0.0
0.2
0.4
0.6
0.8
1.0
M_XGBoost classifier (AUC = 1.00)

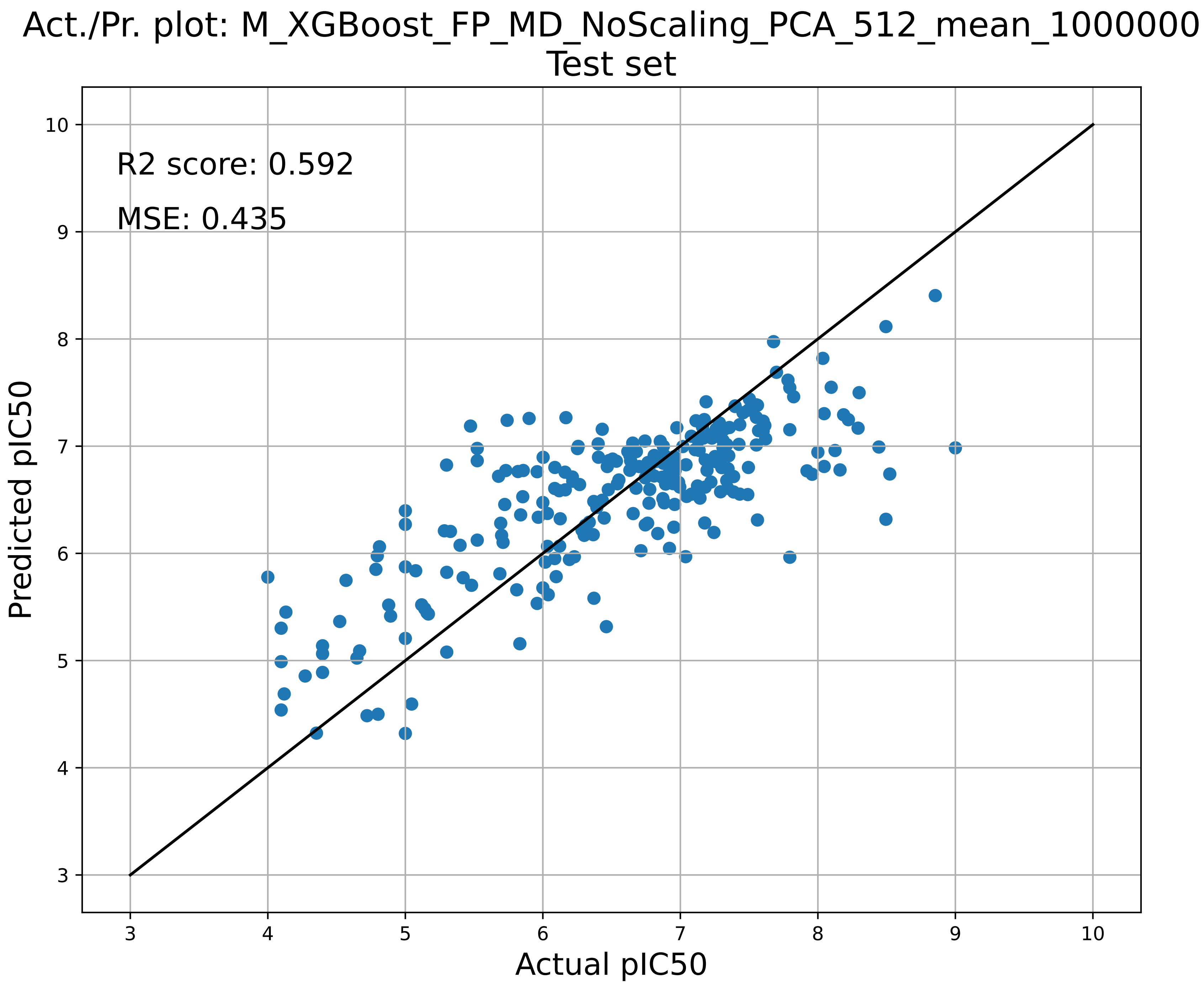
Act./Pr. plot: M_XGBoost_FP_MD_NoScaling_PCA_512_mean_1000000
Test set
R2 score: 0.592
MSE: 0.435
Predicted pIC50
Actual pIC50
3
4
5
6
7
8
9
10

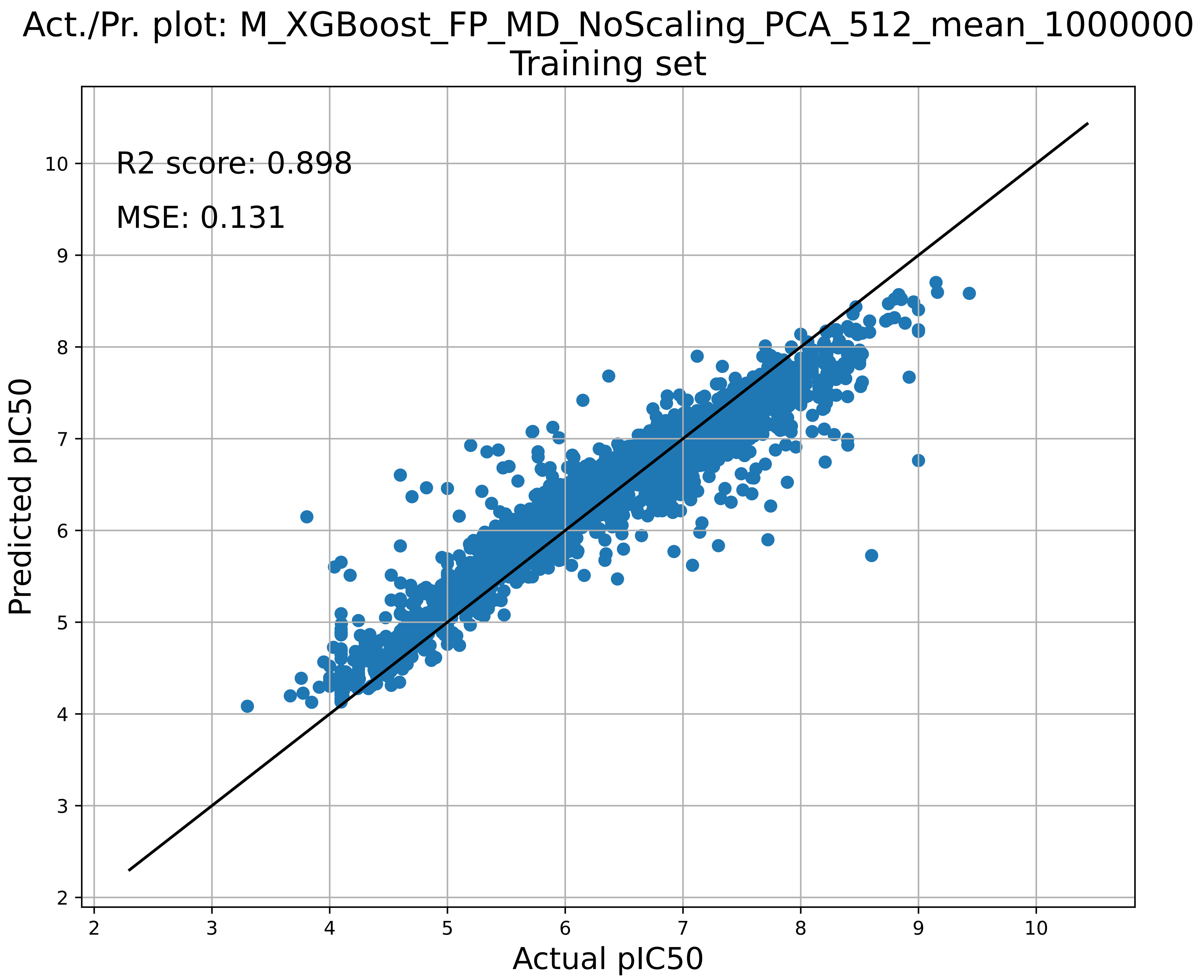
Act./Pr. plot: M_XGBoost_FP_MD_NoScaling_PCA_512_mean_1000000
Training set
R2 score: 0.898
MSE: 0.131
Predicted pIC50
Actual pIC50
2
3
4
5
6
7
8
9
10

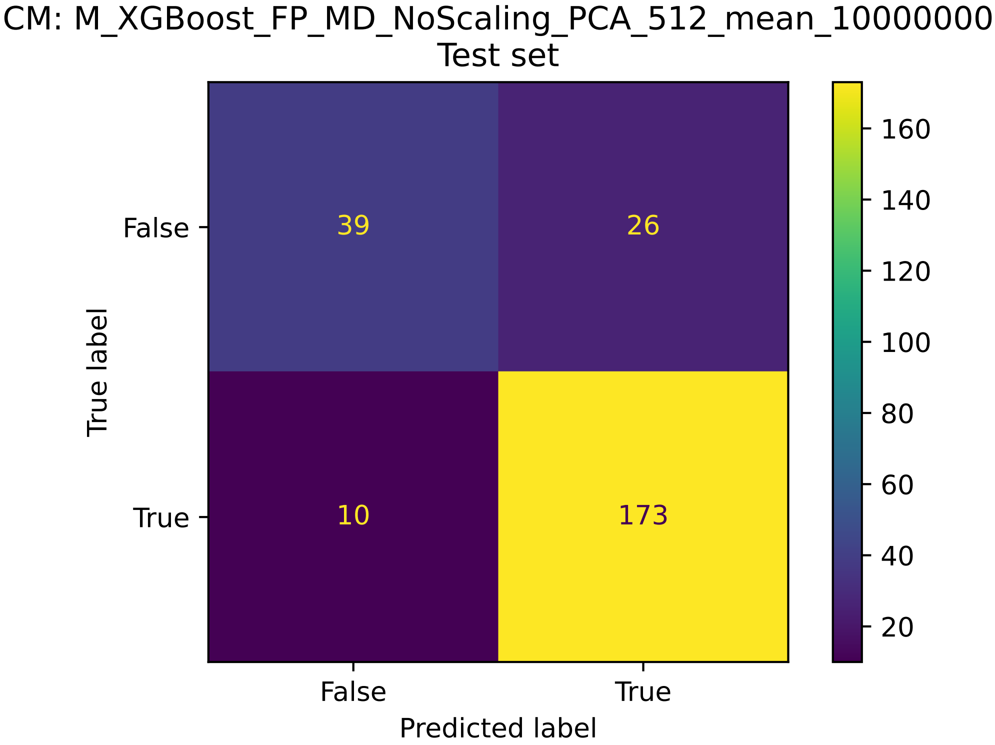

CM: M_XGBoost_FP_MD_NoScaling_PCA_512_mean_10000000
Test set
True label
False
True
39
26
10
173
Predicted label
20
40
60
80
100
120
140
160

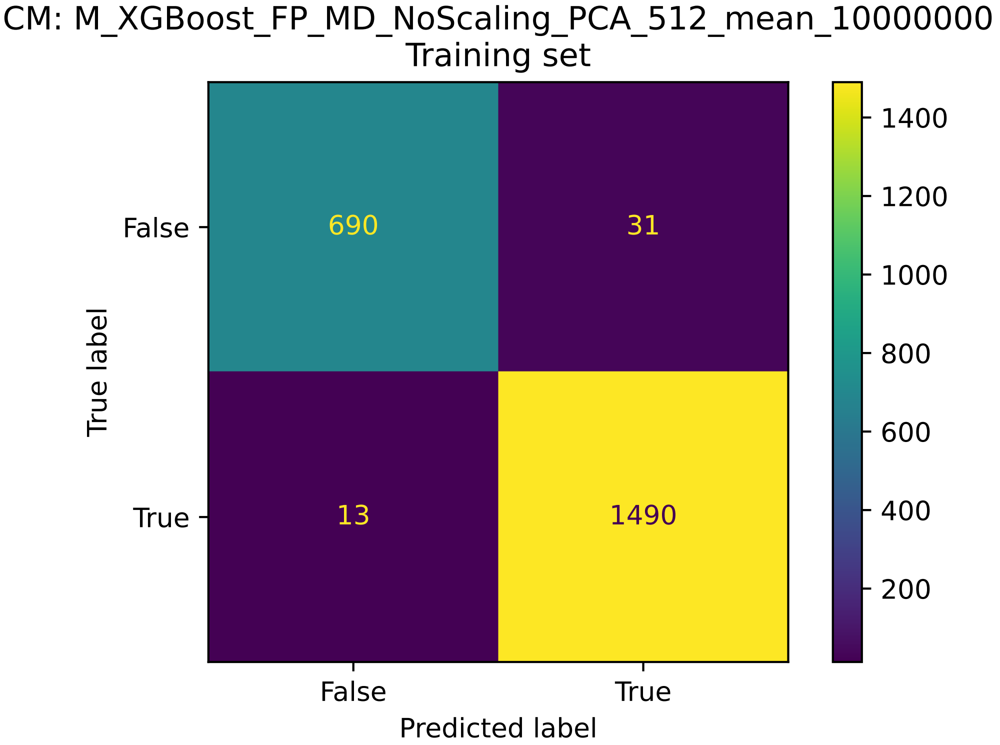
CM: M_XGBoost_FP_MD_NoScaling_PCA_512_mean_10000000
Training set
True label
False
True
690
31
13
1490
Predicted label
False
True
1400
1200
1000
800
600
400
200

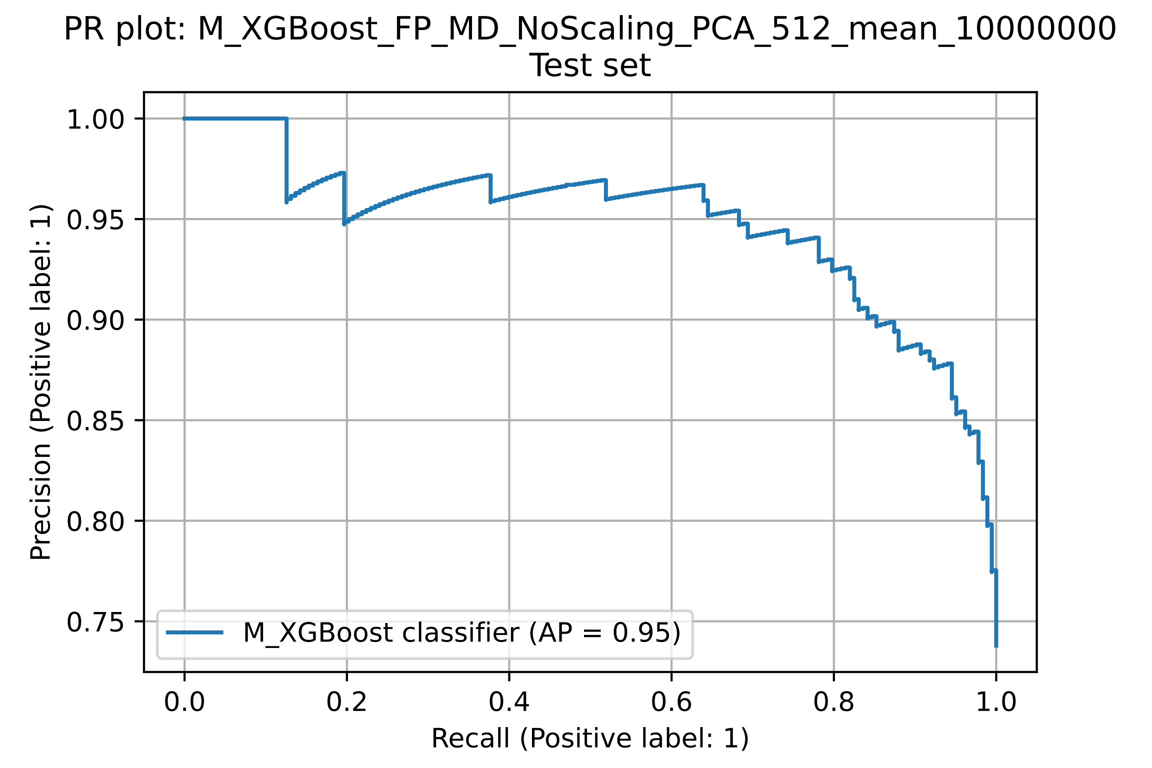

PR plot: M_XGBoost_FP_MD_NoScaling_PCA_512_mean_10000000
Test set
Precision (Positive label: 1)
1.00
0.95
0.90
0.85
0.80
0.75
0.0
0.2
0.4
0.6
0.8
1.0
Recall (Positive label: 1)
M_XGBoost classifier (AP = 0.95)

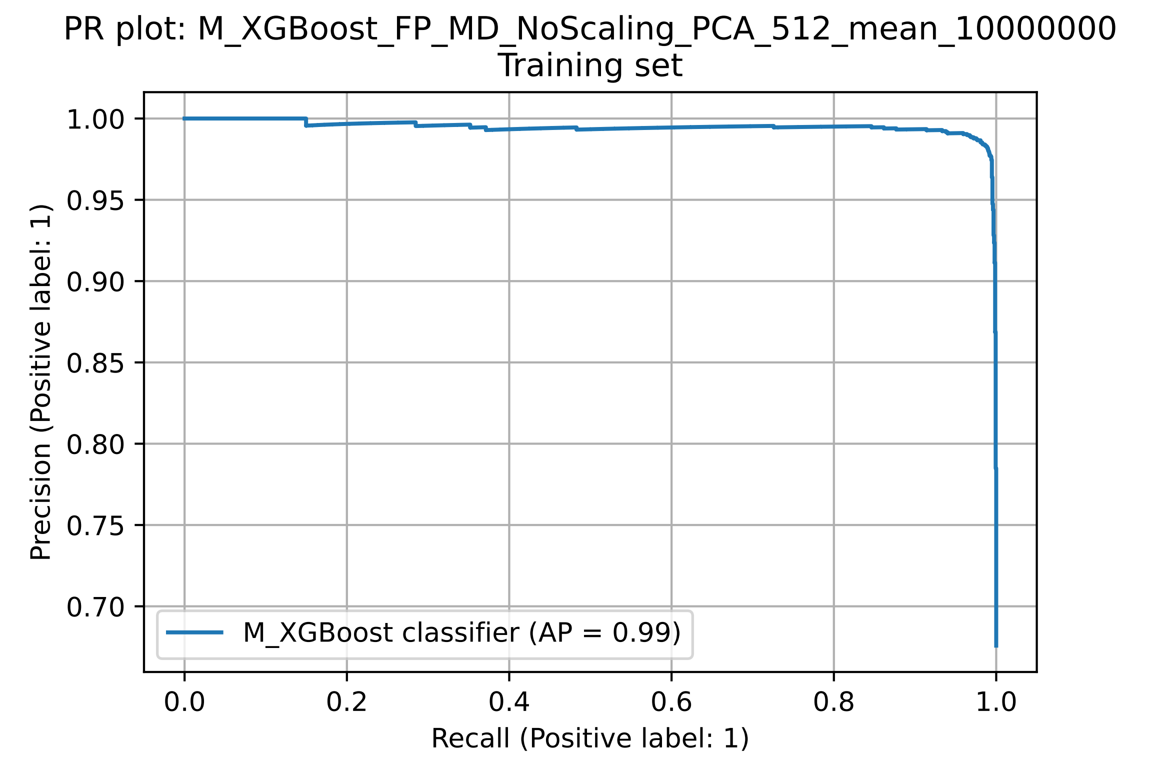

PR plot: M_XGBoost_FP_MD_NoScaling_PCA_512_mean_10000000
Training set
Precision (Positive label: 1)
Recall (Positive label: 1)
1.00
0.95
0.90
0.85
0.80
0.75
0.70
0.0
0.2
0.4
0.6
0.8
1.0
M_XGBoost classifier (AP = 0.99)

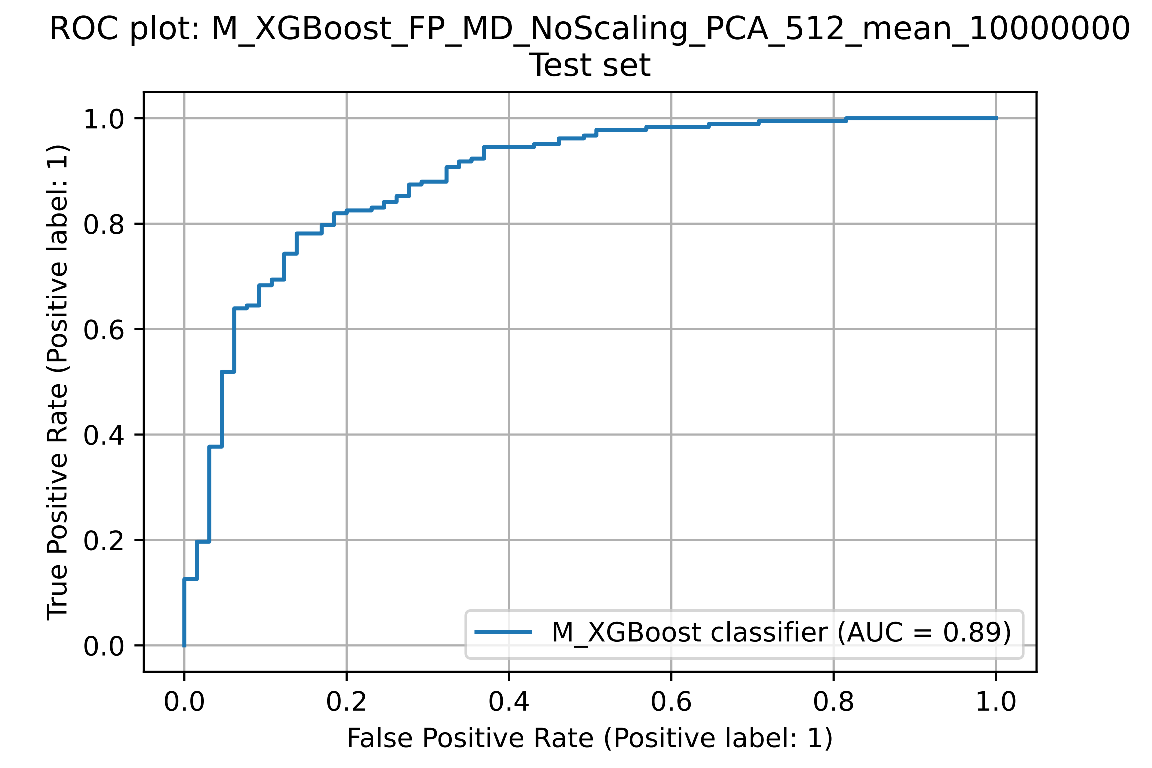
ROC plot: M_XGBoost_FP_MD_NoScaling_PCA_512_mean_10000000
Test set
True Positive Rate (Positive label: 1)
False Positive Rate (Positive label: 1)
0.0
0.2
0.4
0.6
0.8
1.0
M_XGBoost classifier (AUC = 0.89)

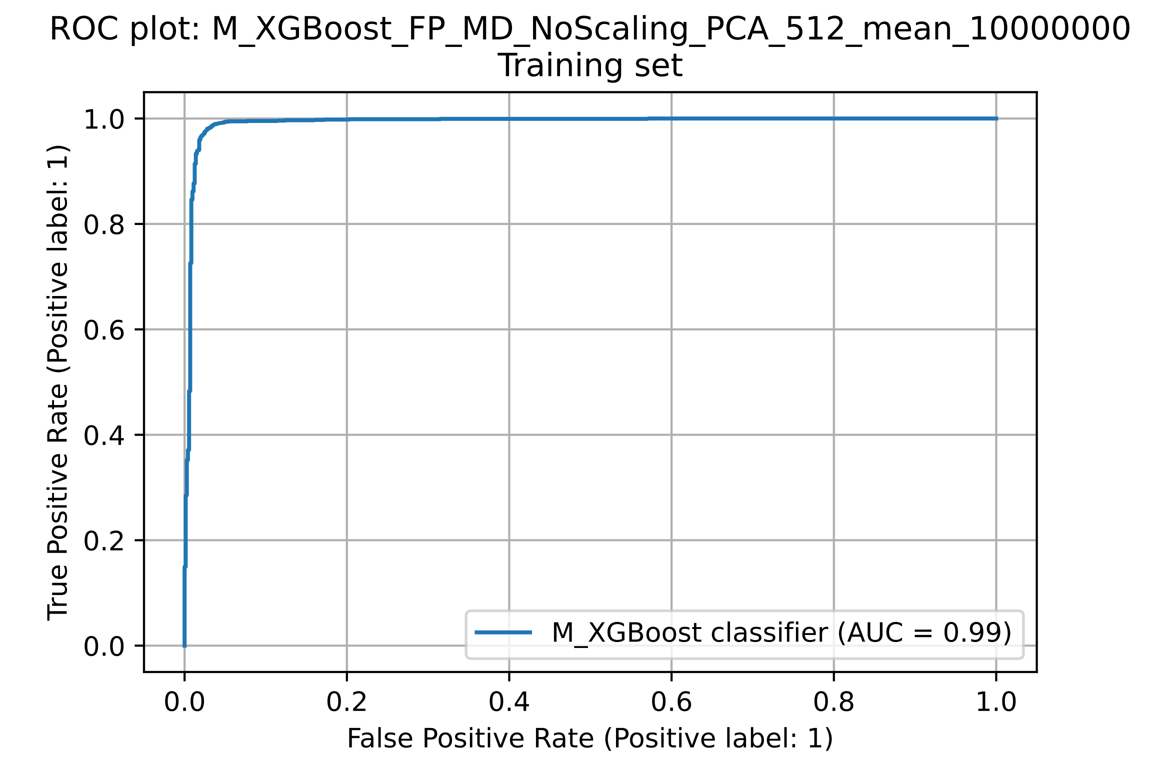
ROC plot: M_XGBoost_FP_MD_NoScaling_PCA_512_mean_10000000
Training set
True Positive Rate (Positive label: 1)
False Positive Rate (Positive label: 1)
0.0
0.2
0.4
0.6
0.8
1.0
M_XGBoost classifier (AUC = 0.99)

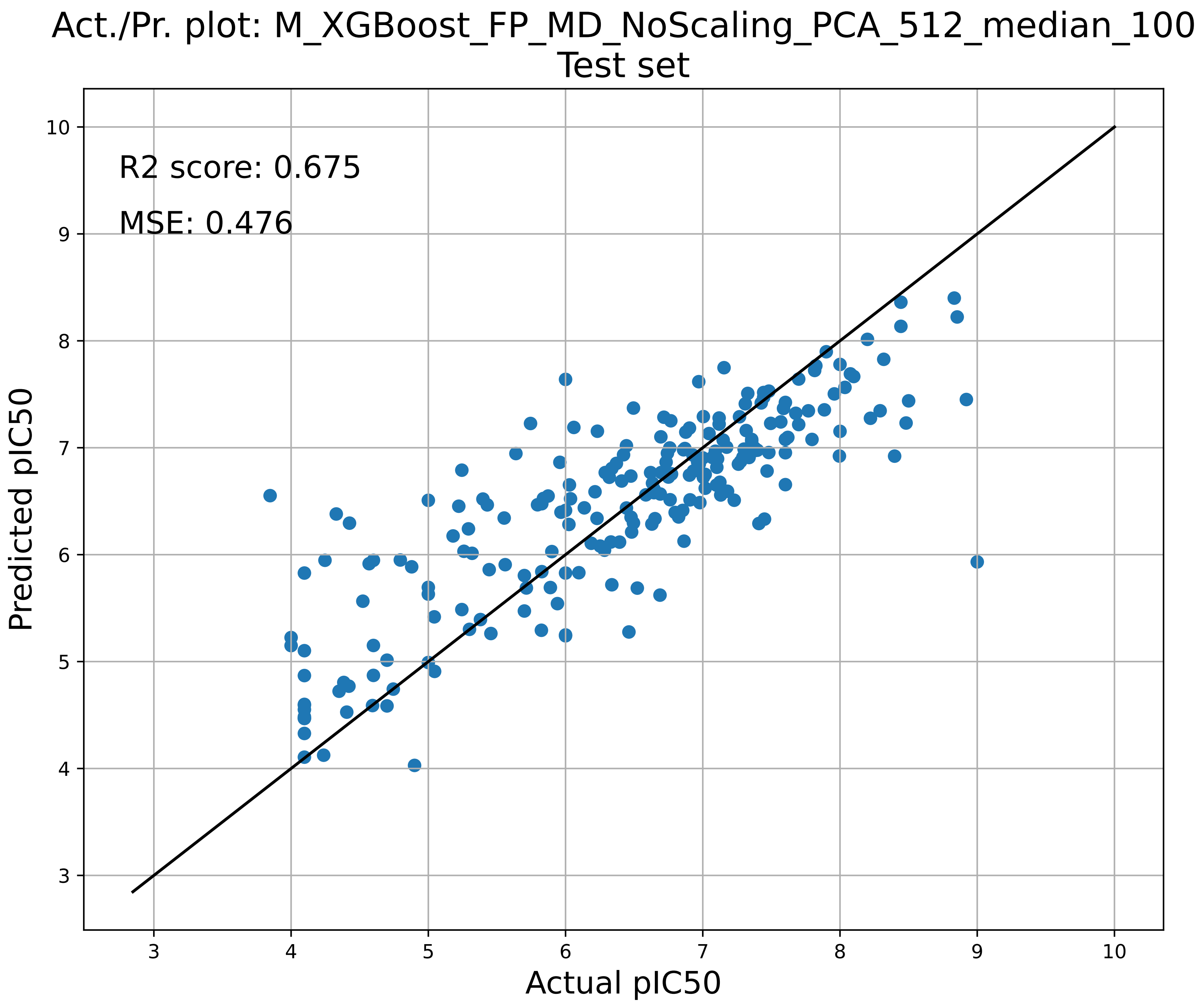

Act./Pr. plot: M_XGBoost_FP_MD_NoScaling_PCA_512_median_100
Test set
R2 score: 0.675
MSE: 0.476
Predicted pIC50
Actual pIC50
3
4
5
6
7
8
9
10

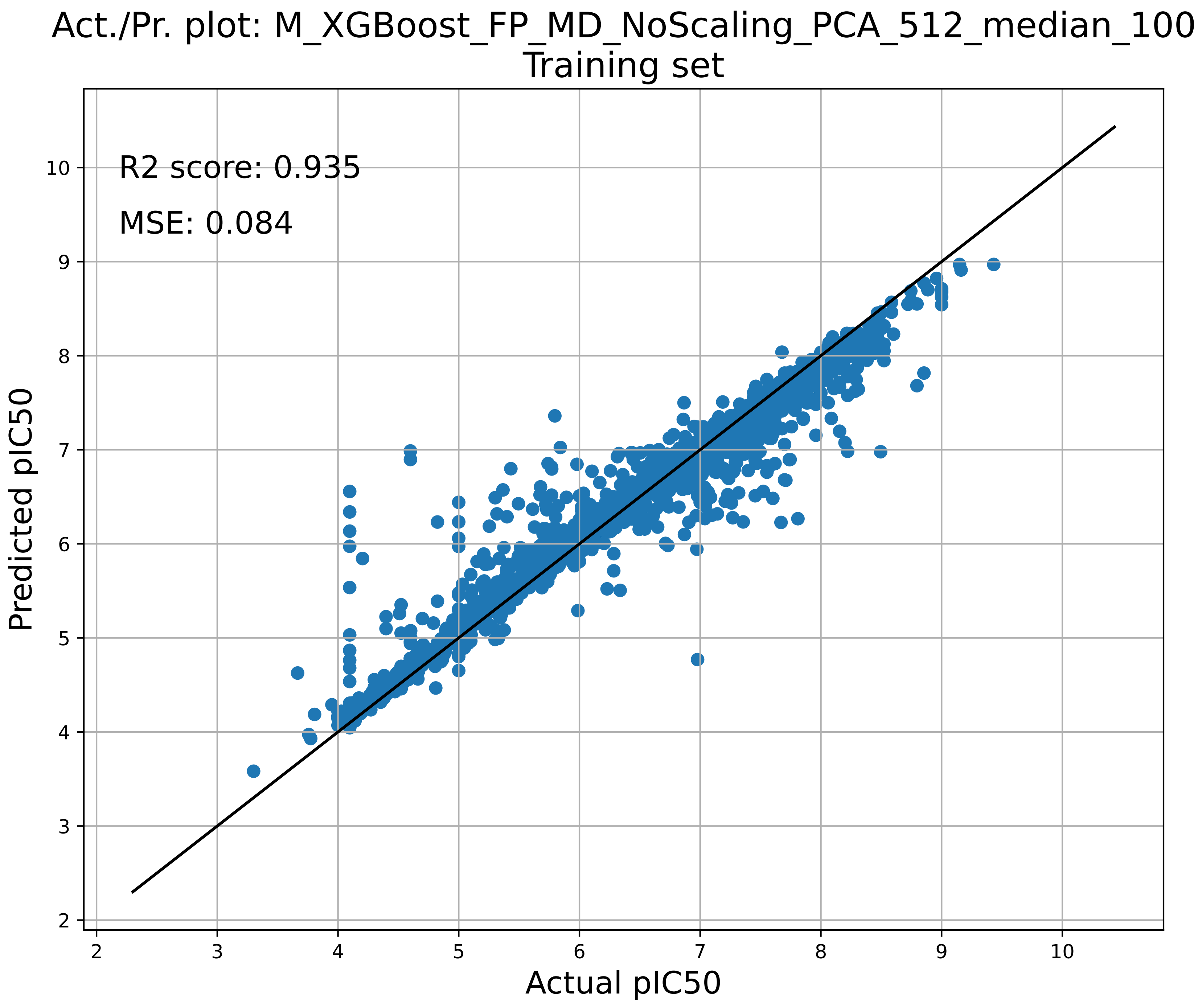
Act./Pr. plot: M_XGBoost_FP_MD_NoScaling_PCA_512_median_100
Training set
R2 score: 0.935
MSE: 0.084
Predicted pIC50
Actual pIC50
2
3
4
5
6
7
8
9
10

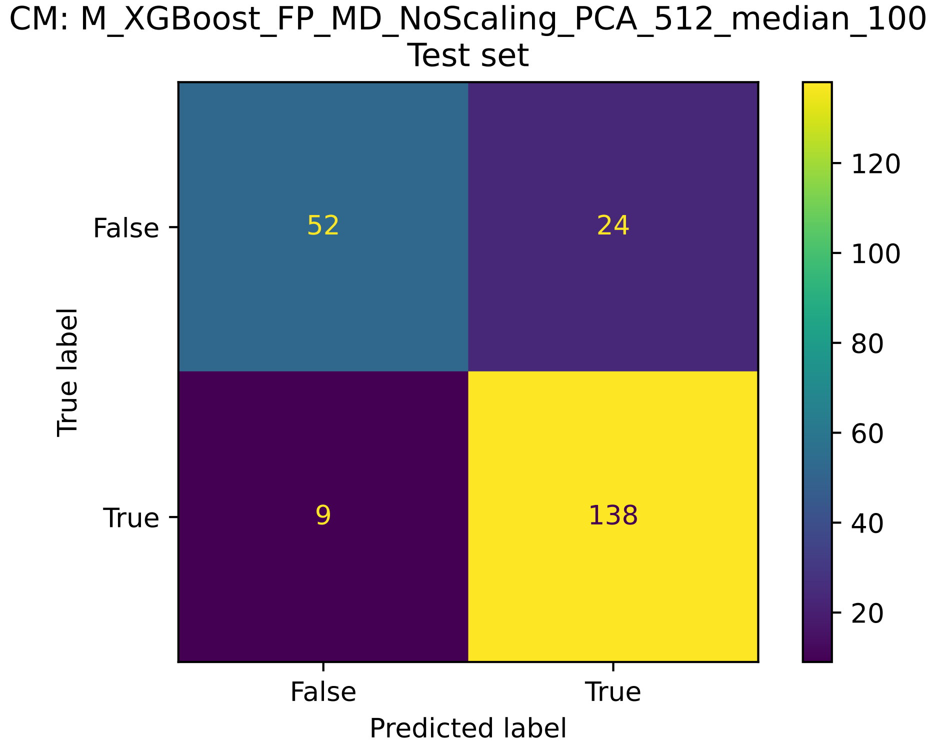
CM: M_XGBoost_FP_MD_NoScaling_PCA_512_median_100
Test set
True label
False
True
52
24
9
138
False
True
Predicted label
120
100
80
60
40
20

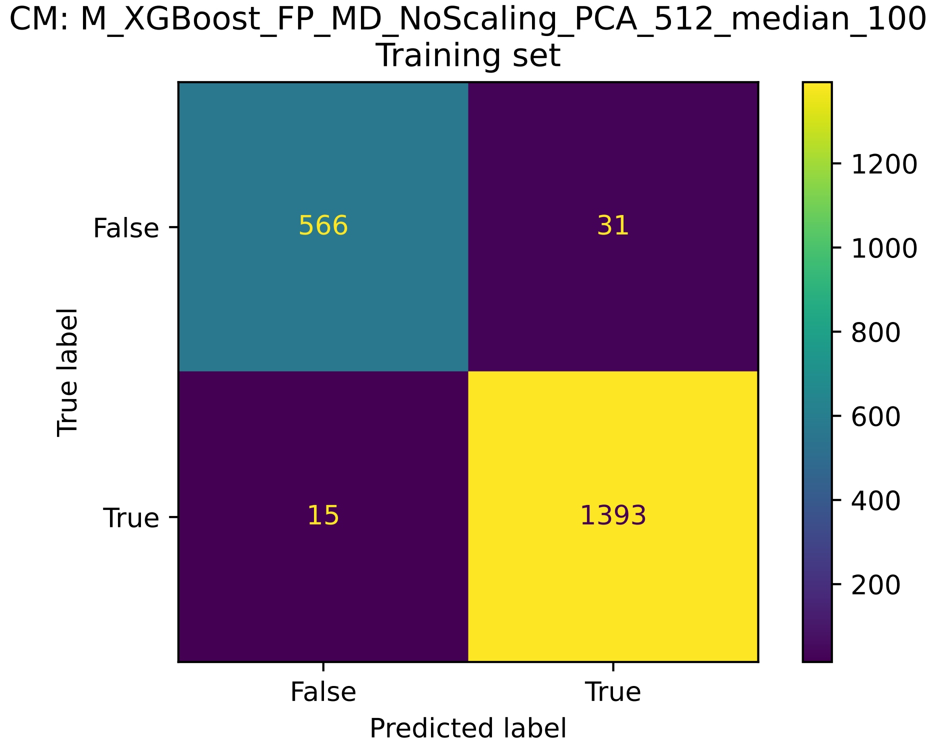

CM: M_XGBoost_FP_MD_NoScaling_PCA_512_median_100
Training set
True label
Predicted label
False
True
False
True
566
31
15
1393
200
400
600
800
1000
1200

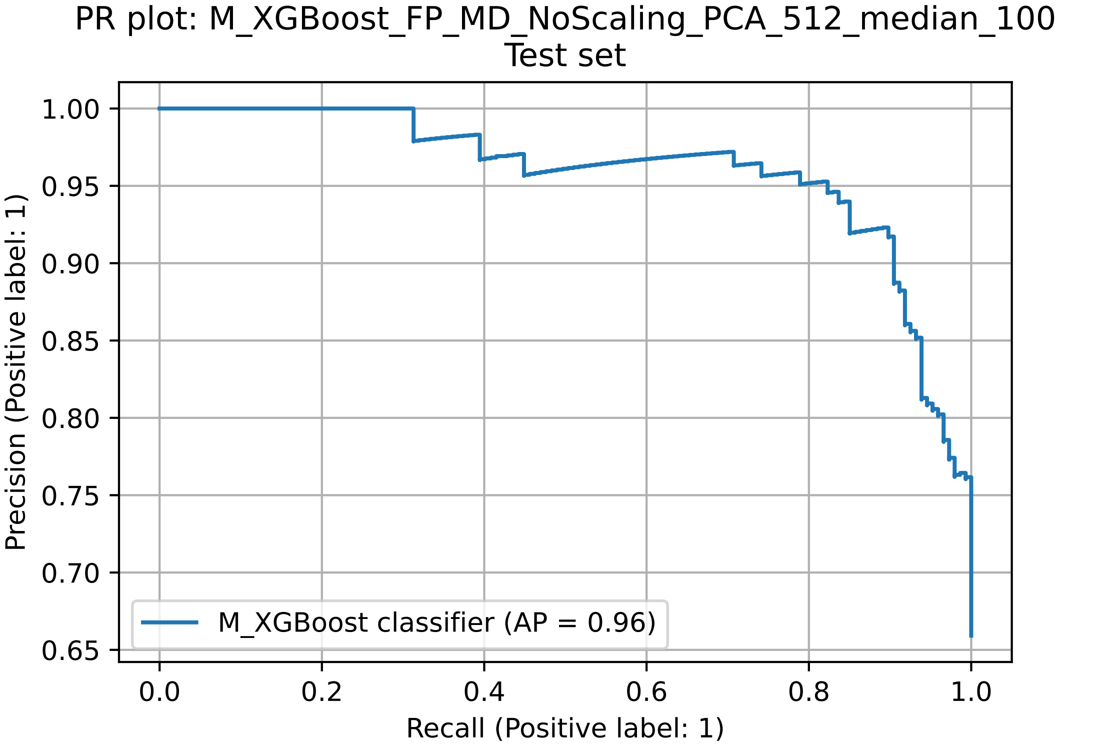
PR plot: M_XGBoost_FP_MD_NoScaling_PCA_512_median_100
Test set
Precision (Positive label: 1)
Recall (Positive label: 1)
1.00
0.95
0.90
0.85
0.80
0.75
0.70
0.65
0.0
0.2
0.4
0.6
0.8
1.0
M_XGBoost classifier (AP = 0.96)

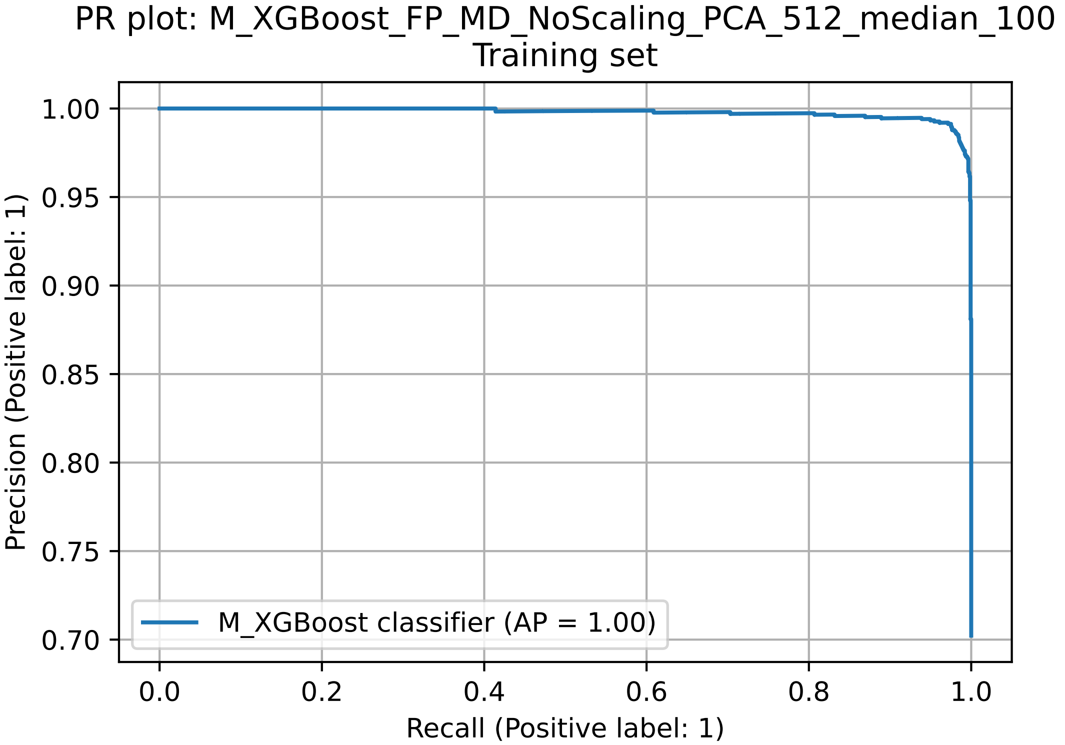
PR plot: M_XGBoost_FP_MD_NoScaling_PCA_512_median_100
Training set
Precision (Positive label: 1)
Recall (Positive label: 1)
1.00
0.95
0.90
0.85
0.80
0.75
0.70
0.0
0.2
0.4
0.6
0.8
1.0
M_XGBoost classifier (AP = 1.00)

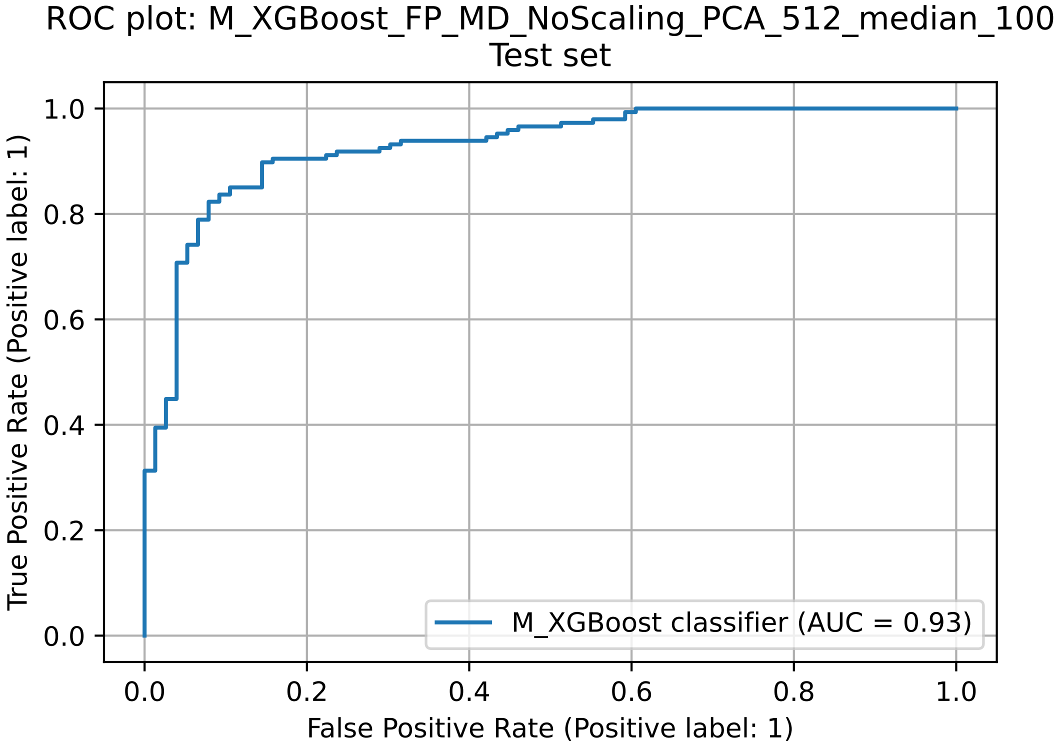

ROC plot: M_XGBoost_FP_MD_NoScaling_PCA_512_median_100
Test set
True Positive Rate (Positive label: 1)
False Positive Rate (Positive label: 1)
0.0
0.2
0.4
0.6
0.8
1.0
M_XGBoost classifier (AUC = 0.93)

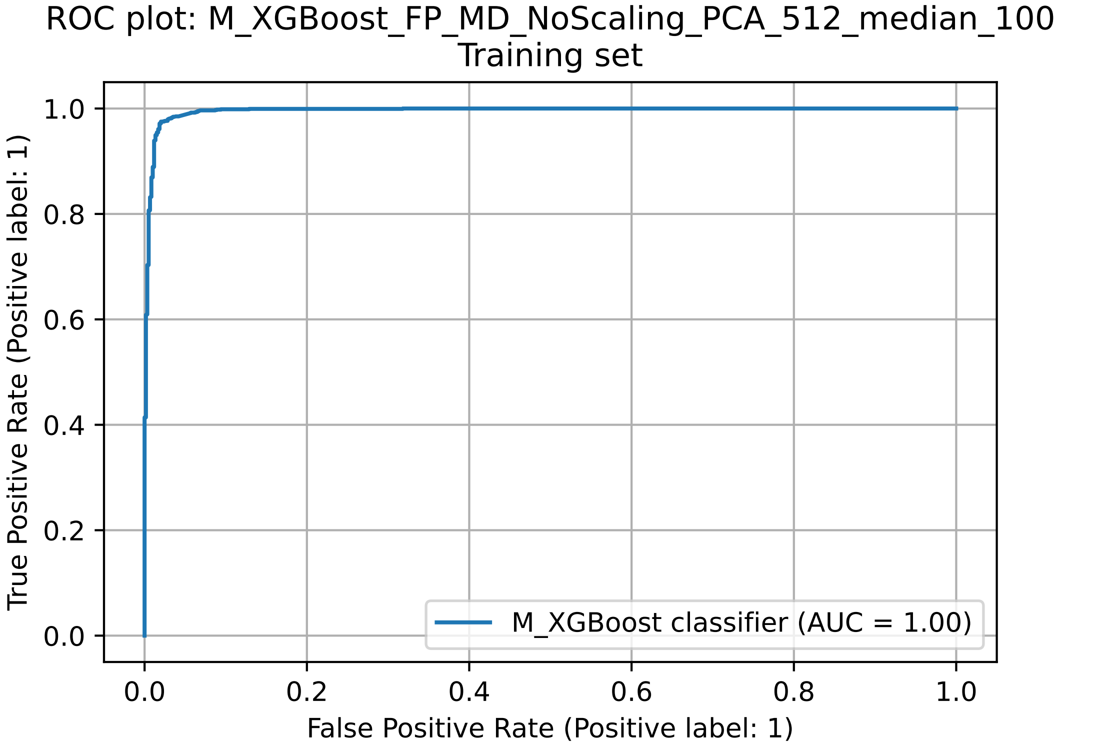
ROC plot: M_XGBoost_FP_MD_NoScaling_PCA_512_median_100
Training set
True Positive Rate (Positive label: 1)
False Positive Rate (Positive label: 1)
0.0
0.2
0.4
0.6
0.8
1.0
M_XGBoost classifier (AUC = 1.00)

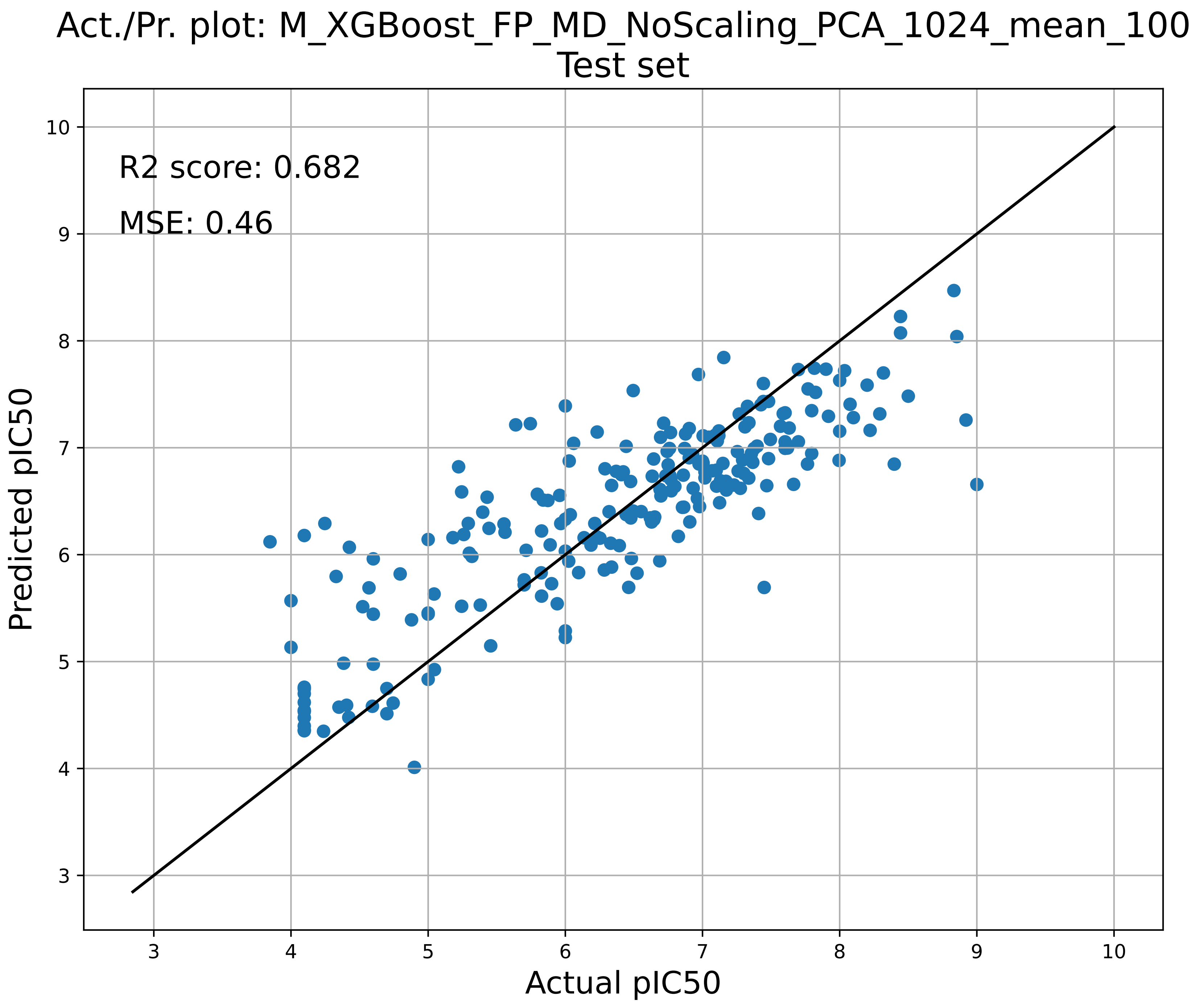

Act./Pr. plot: M_XGBoost_FP_MD_NoScaling_PCA_1024_mean_100
Test set
R2 score: 0.682
MSE: 0.46
Predicted pIC50
Actual pIC50
3
4
5
6
7
8
9
10

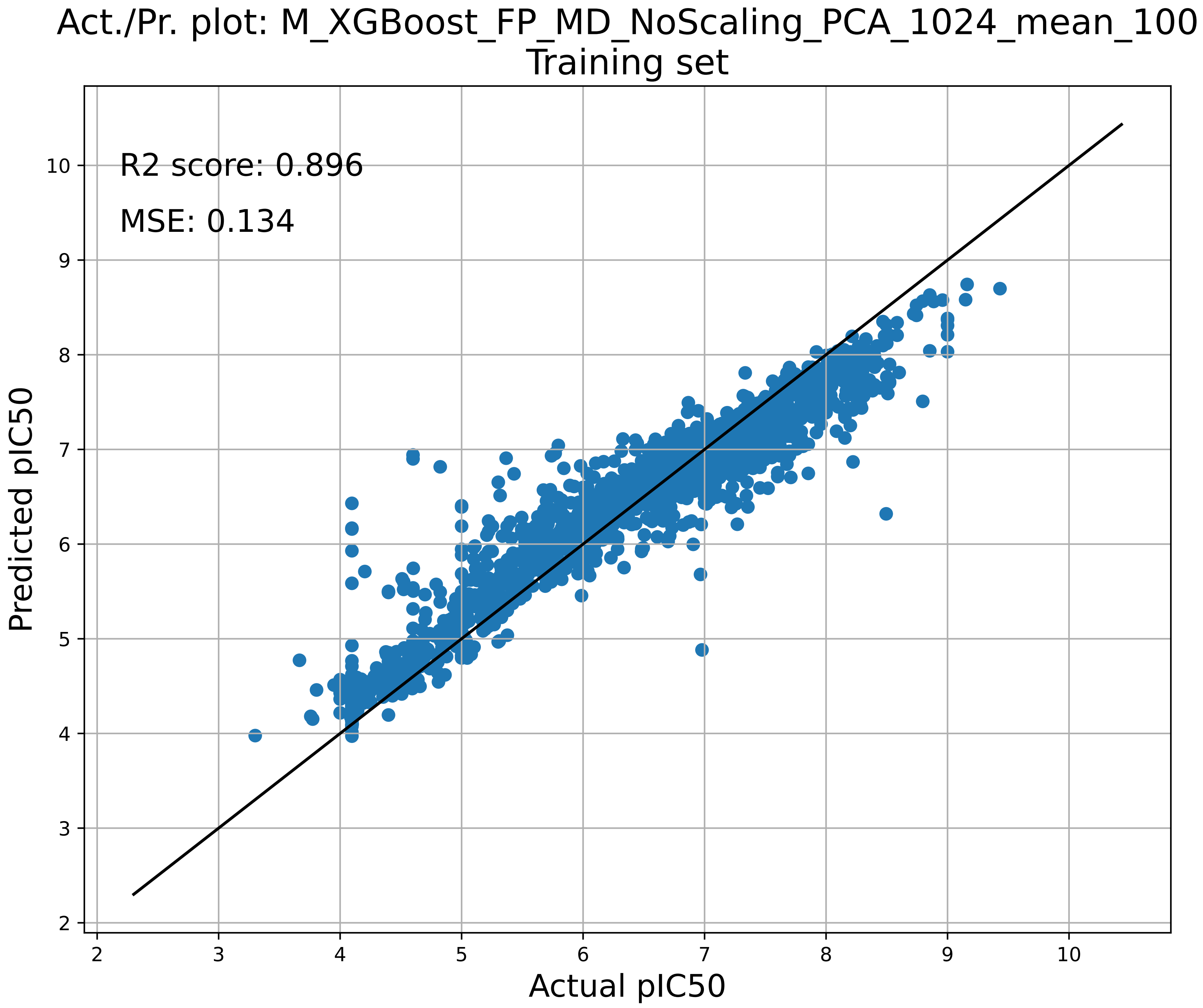

Act./Pr. plot: M_XGBoost_FP_MD_NoScaling_PCA_1024_mean_100
Training set
R2 score: 0.896
MSE: 0.134
Predicted pIC50
Actual pIC50
2
3
4
5
6
7
8
9
10

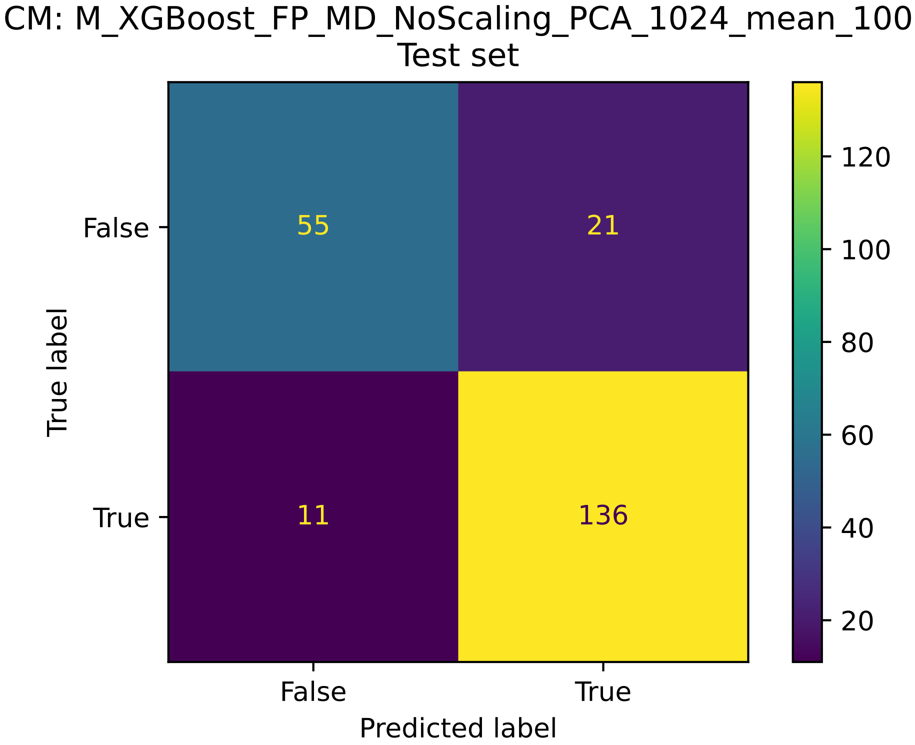

CM: M_XGBoost_FP_MD_NoScaling_PCA_1024_mean_100
Test set
True label
False
True
55
21
11
136
False
True
Predicted label
120
100
80
60
40
20

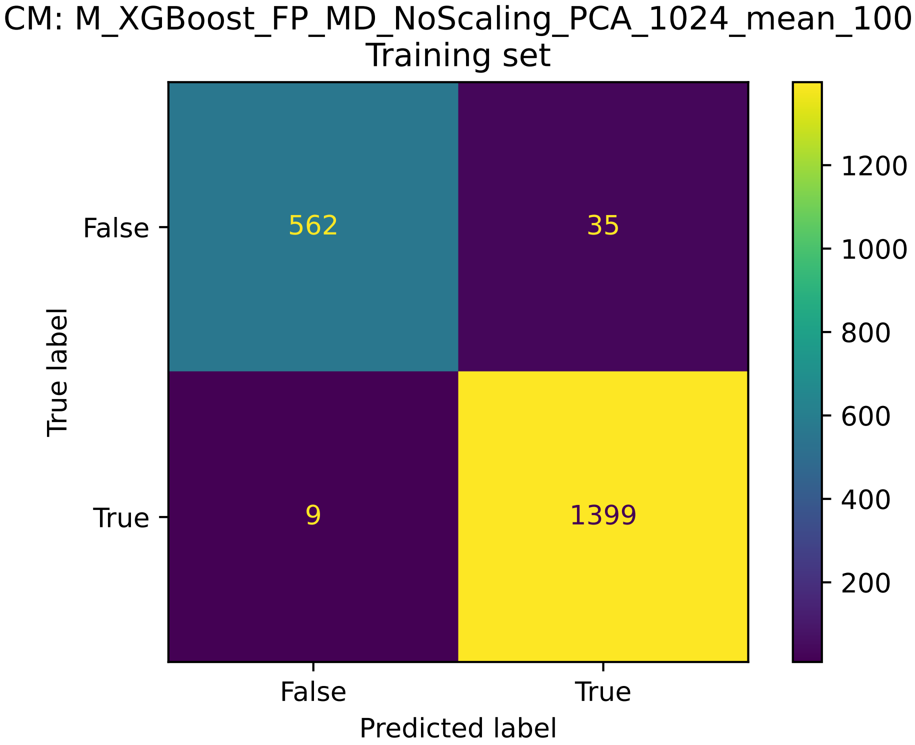

CM: M_XGBoost_FP_MD_NoScaling_PCA_1024_mean_100
Training set
562
35
9
1399
False
True
True label
False
True
Predicted label
1200
1000
800
600
400
200

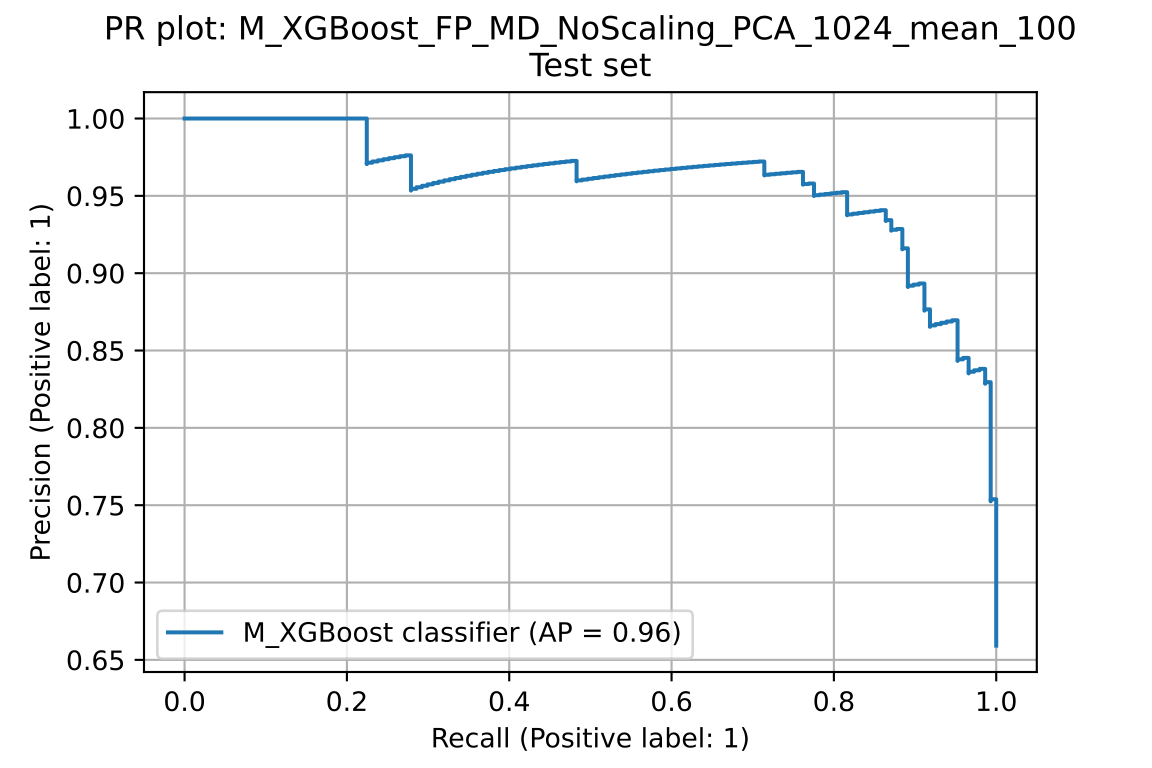
PR plot: M_XGBoost_FP_MD_NoScaling_PCA_1024_mean_100
Test set
Precision (Positive label: 1)
Recall (Positive label: 1)
1.00
0.95
0.90
0.85
0.80
0.75
0.70
0.65
0.0
0.2
0.4
0.6
0.8
1.0
M_XGBoost classifier (AP = 0.96)

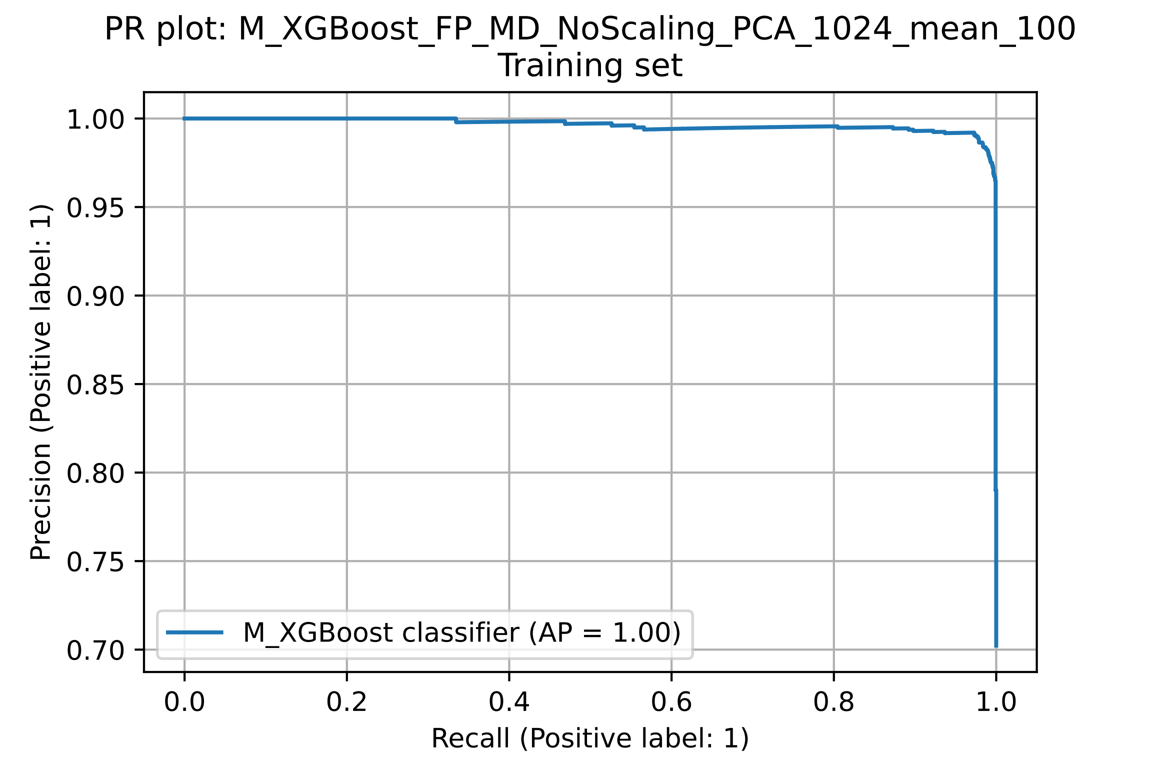
PR plot: M_XGBoost_FP_MD_NoScaling_PCA_1024_mean_100
Training set
Precision (Positive label: 1)
Recall (Positive label: 1)
1.00
0.95
0.90
0.85
0.80
0.75
0.70
0.0
0.2
0.4
0.6
0.8
1.0
M_XGBoost classifier (AP = 1.00)

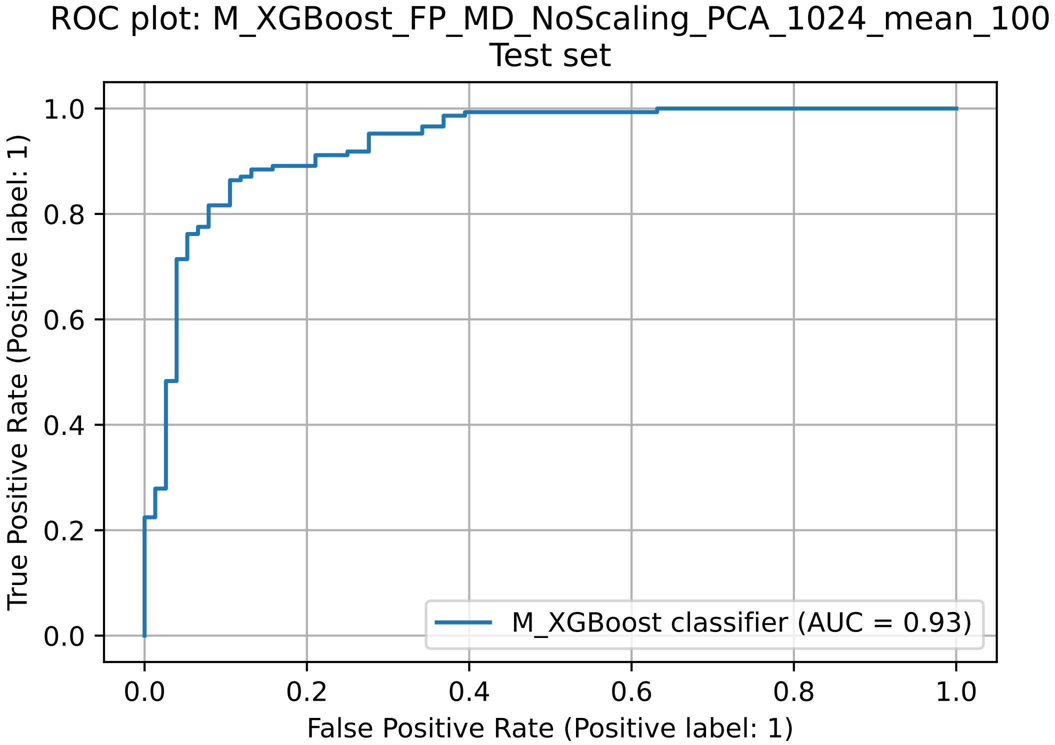
ROC plot: M_XGBoost_FP_MD_NoScaling_PCA_1024_mean_100
Test set
True Positive Rate (Positive label: 1)
False Positive Rate (Positive label: 1)
0.0
0.2
0.4
0.6
0.8
1.0
M_XGBoost classifier (AUC = 0.93)

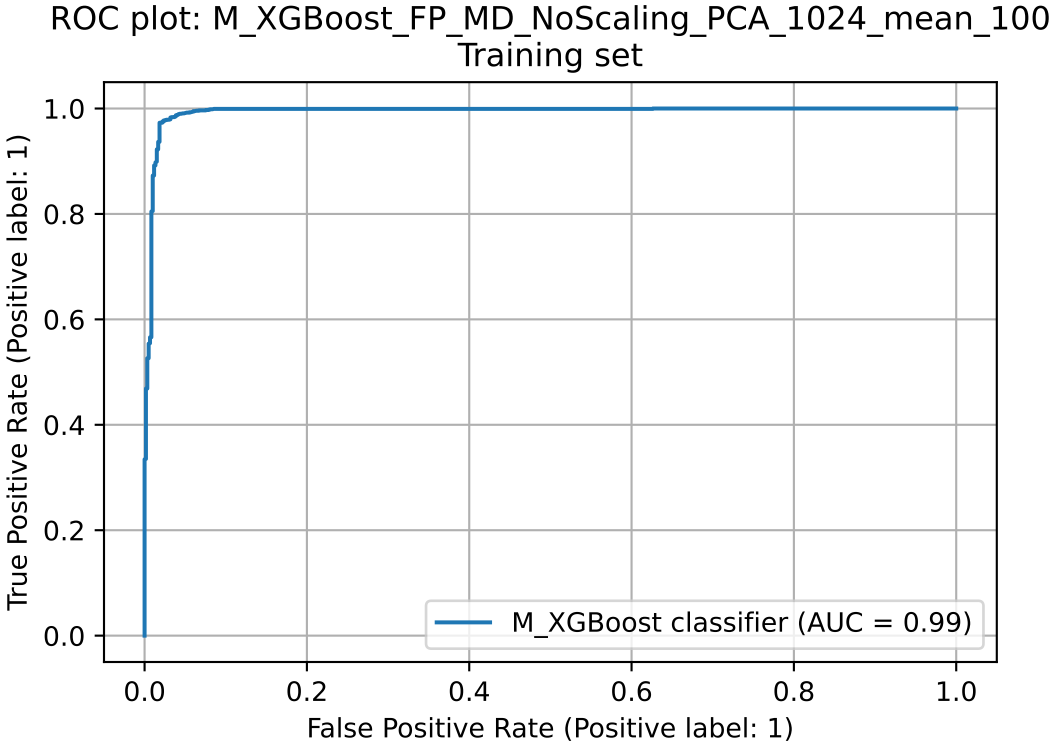
ROC plot: M_XGBoost_FP_MD_NoScaling_PCA_1024_mean_100
Training set
True Positive Rate (Positive label: 1)
False Positive Rate (Positive label: 1)
1.0
0.8
0.6
0.4
0.2
0.0
0.0
0.2
0.4
0.6
0.8
1.0
M_XGBoost classifier (AUC = 0.99)

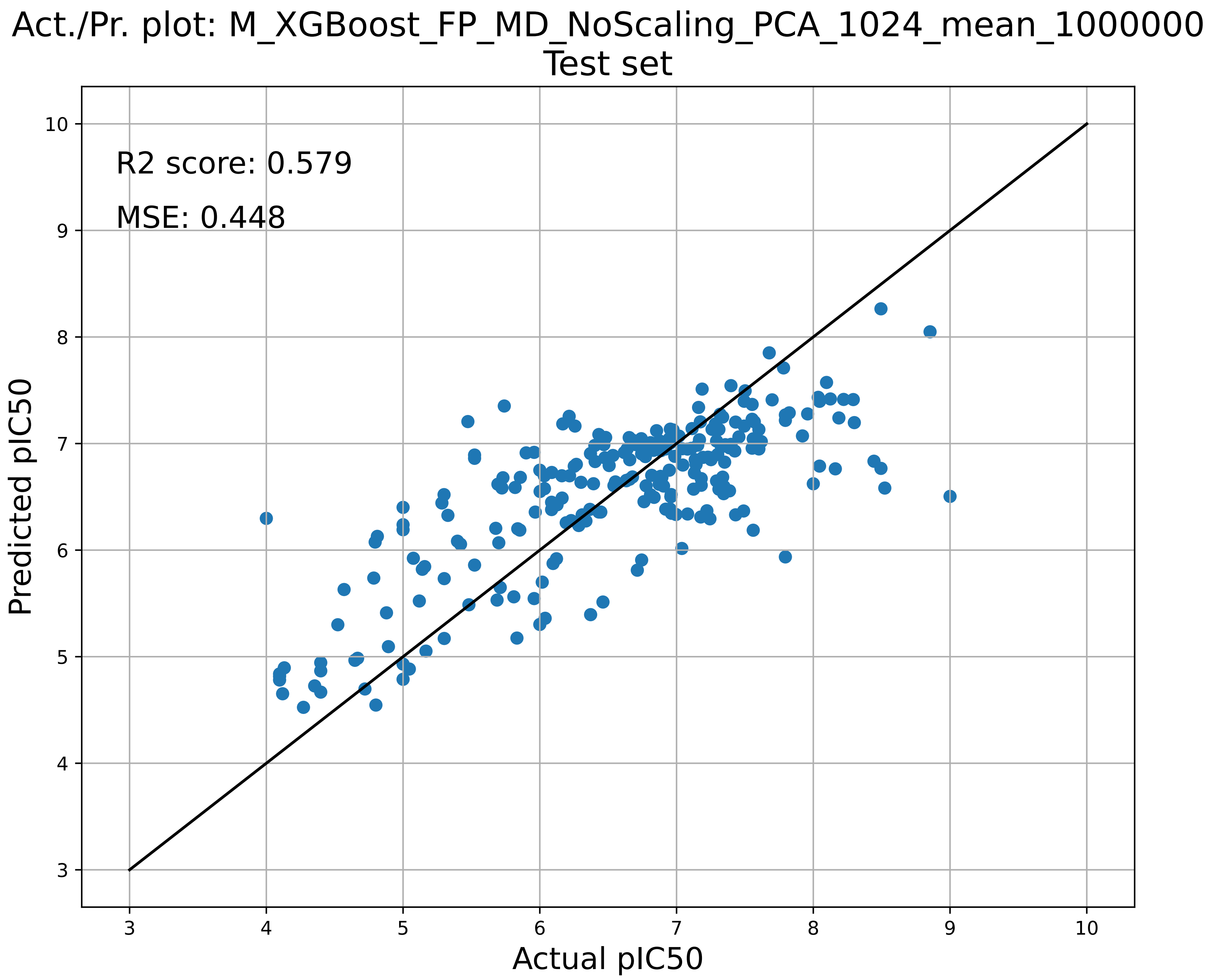

Act./Pr. plot: M_XGBoost_FP_MD_NoScaling_PCA_1024_mean_1000000
Test set
R2 score: 0.579
MSE: 0.448
Predicted pIC50
Actual pIC50
3
4
5
6
7
8
9
10

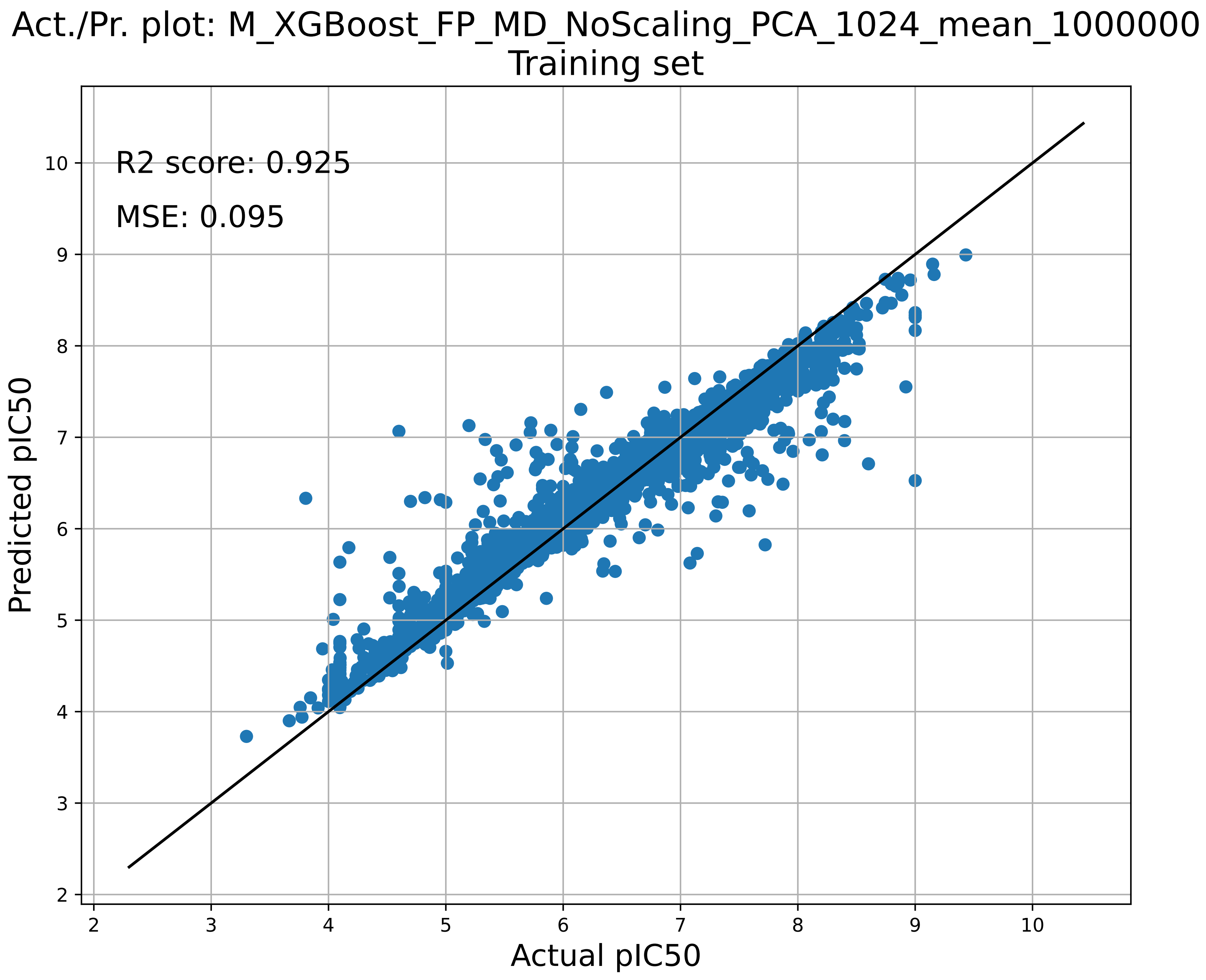
Act./Pr. plot: M_XGBoost_FP_MD_NoScaling_PCA_1024_mean_1000000
Training set
R2 score: 0.925
MSE: 0.095
Predicted pIC50
Actual pIC50
2
3
4
5
6
7
8
9
10

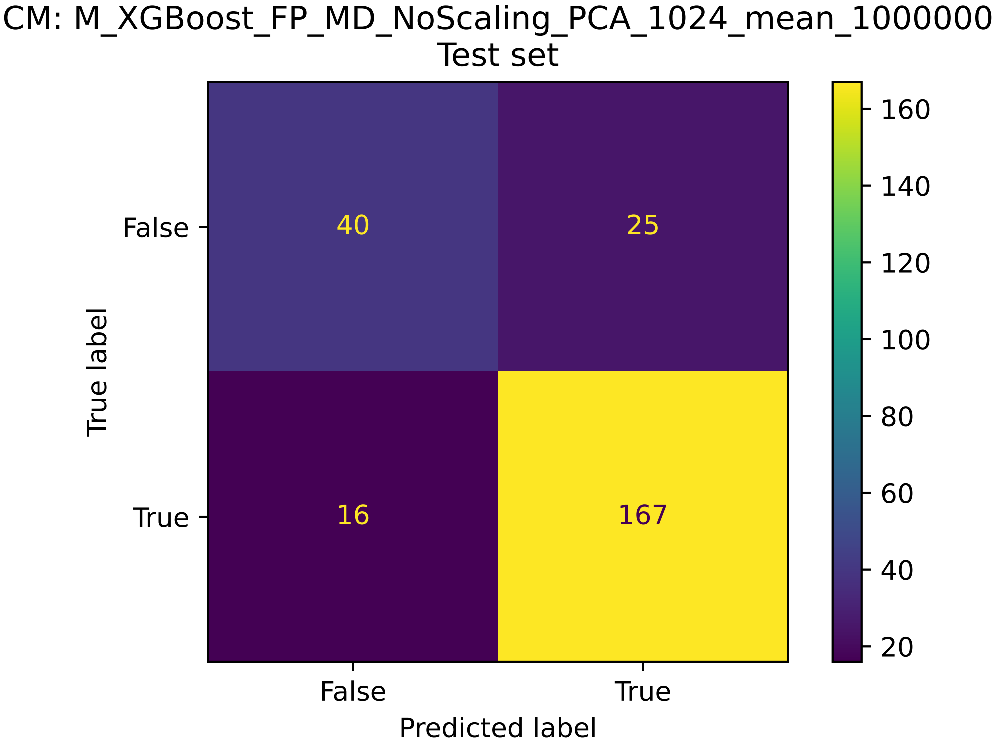
CM: M_XGBoost_FP_MD_NoScaling_PCA_1024_mean_1000000
Test set
True label
Predicted label
False
True
False
True
40
25
16
167
160
140
120
100
80
60
40
20

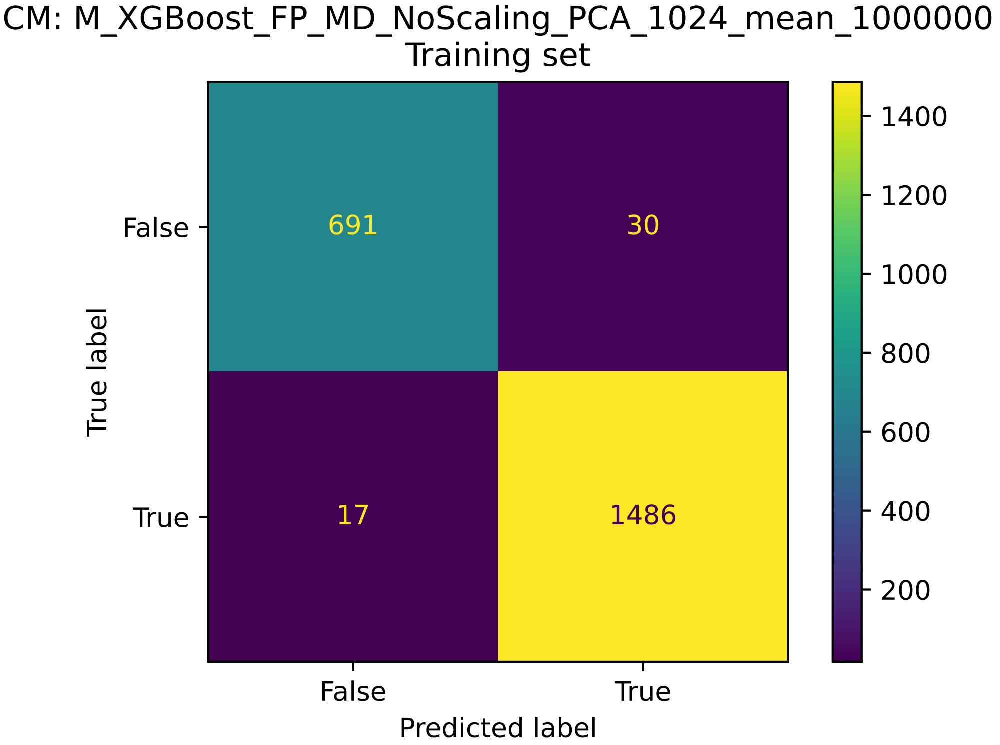

CM: M_XGBoost_FP_MD_NoScaling_PCA_1024_mean_1000000
Training set
True label
Predicted label
False
True
False
True
691
30
17
1486
1400
1200
1000
800
600
400
200

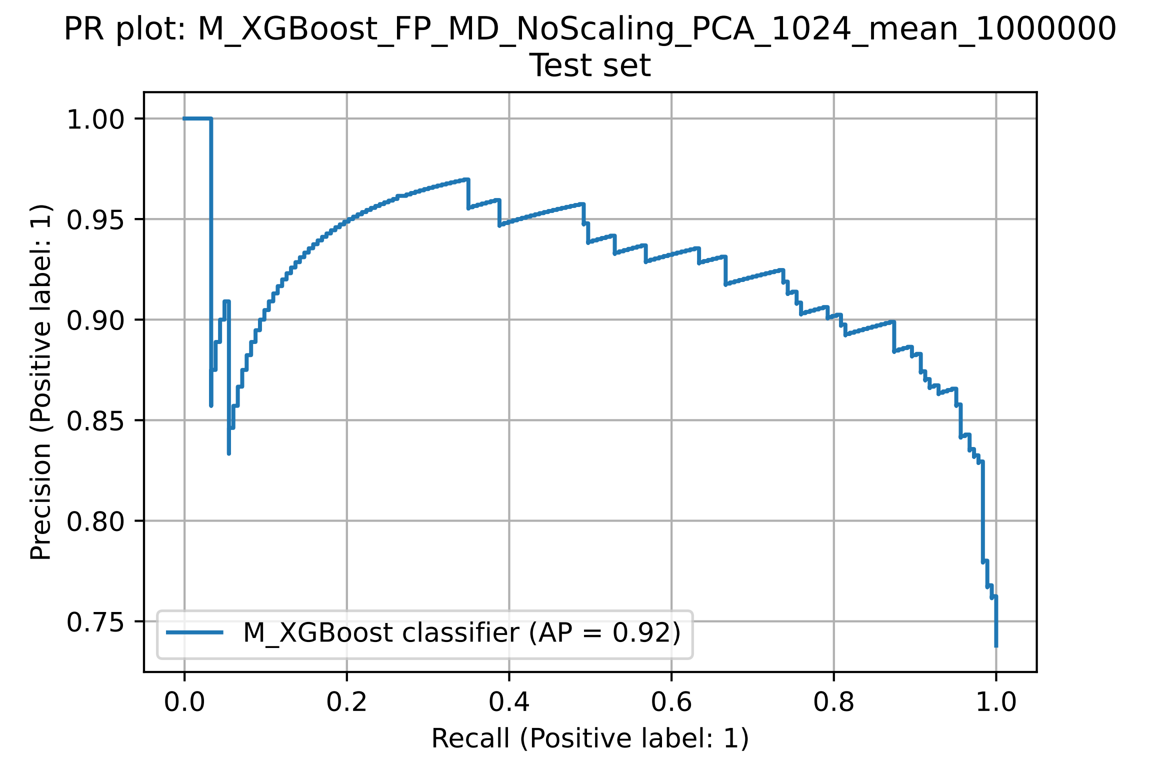
PR plot: M_XGBoost_FP_MD_NoScaling_PCA_1024_mean_1000000
Test set
Precision (Positive label: 1)
Recall (Positive label: 1)
1.00
0.95
0.90
0.85
0.80
0.75
0.0
0.2
0.4
0.6
0.8
1.0
M_XGBoost classifier (AP = 0.92)

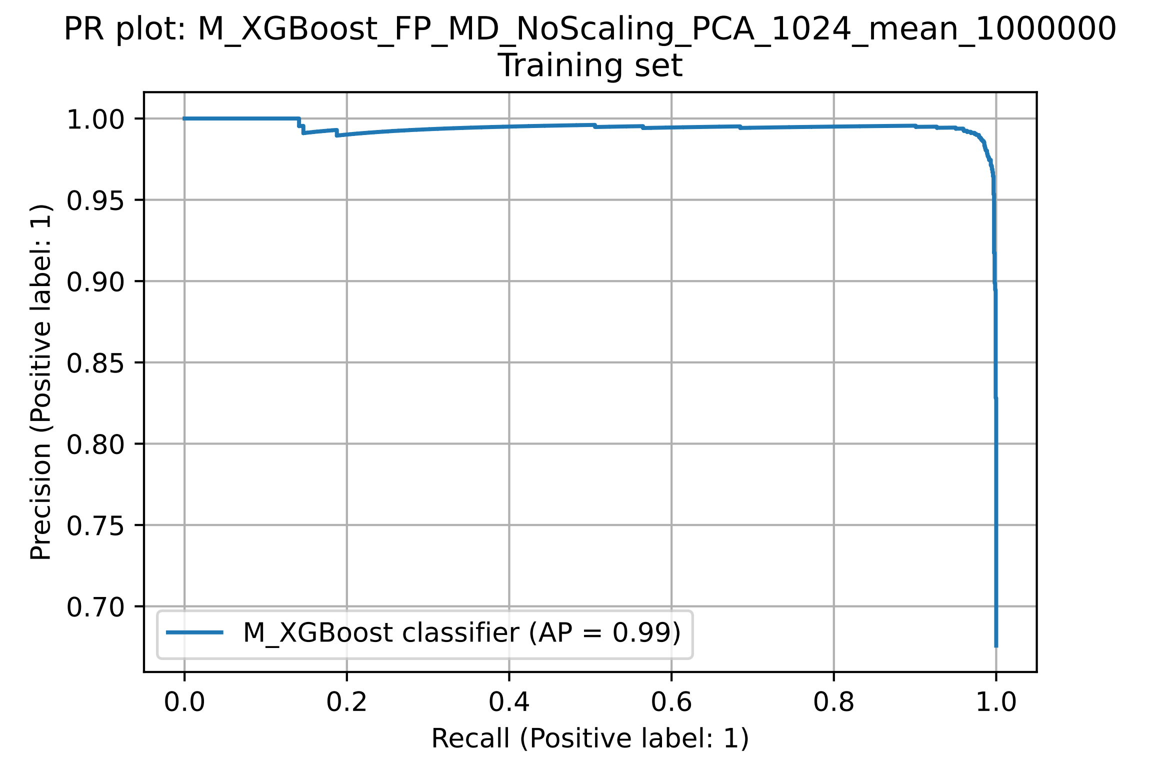

PR plot: M_XGBoost_FP_MD_NoScaling_PCA_1024_mean_1000000
Training set
Precision (Positive label: 1)
Recall (Positive label: 1)
1.00
0.95
0.90
0.85
0.80
0.75
0.70
0.0
0.2
0.4
0.6
0.8
1.0
M_XGBoost classifier (AP = 0.99)

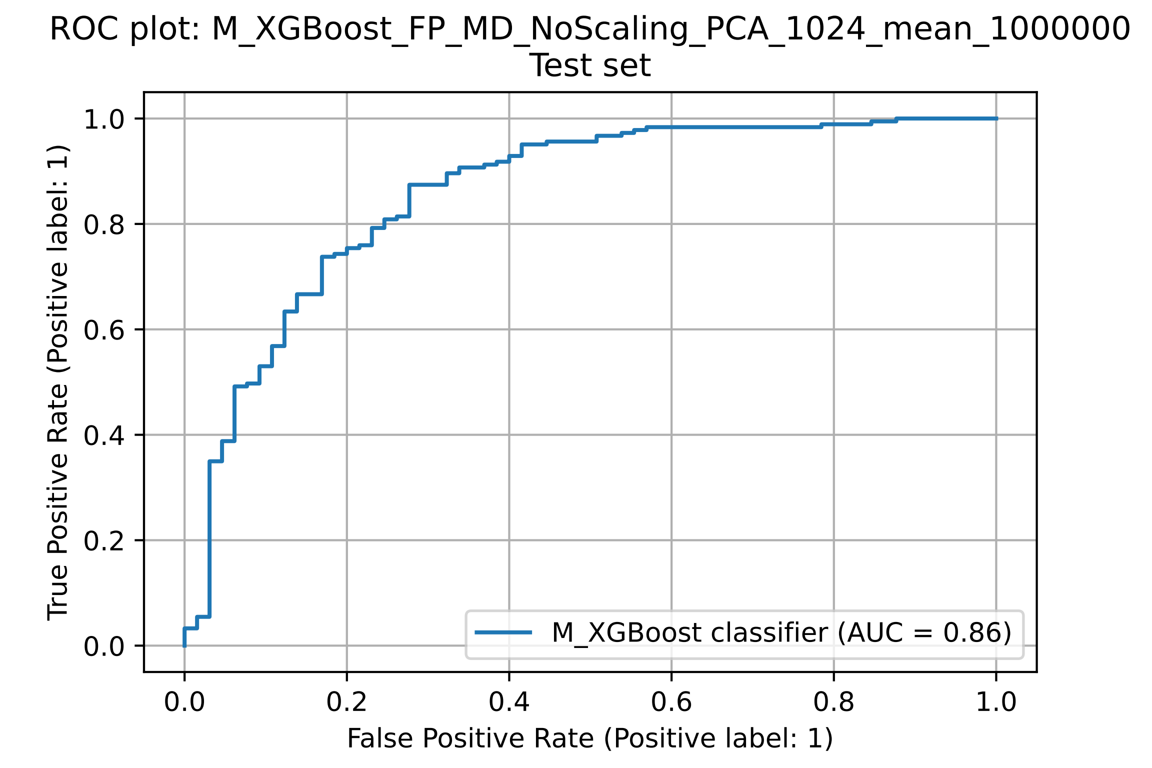
ROC plot: M_XGBoost_FP_MD_NoScaling_PCA_1024_mean_1000000
Test set
True Positive Rate (Positive label: 1)
False Positive Rate (Positive label: 1)
0.0
0.2
0.4
0.6
0.8
1.0
M_XGBoost classifier (AUC = 0.86)

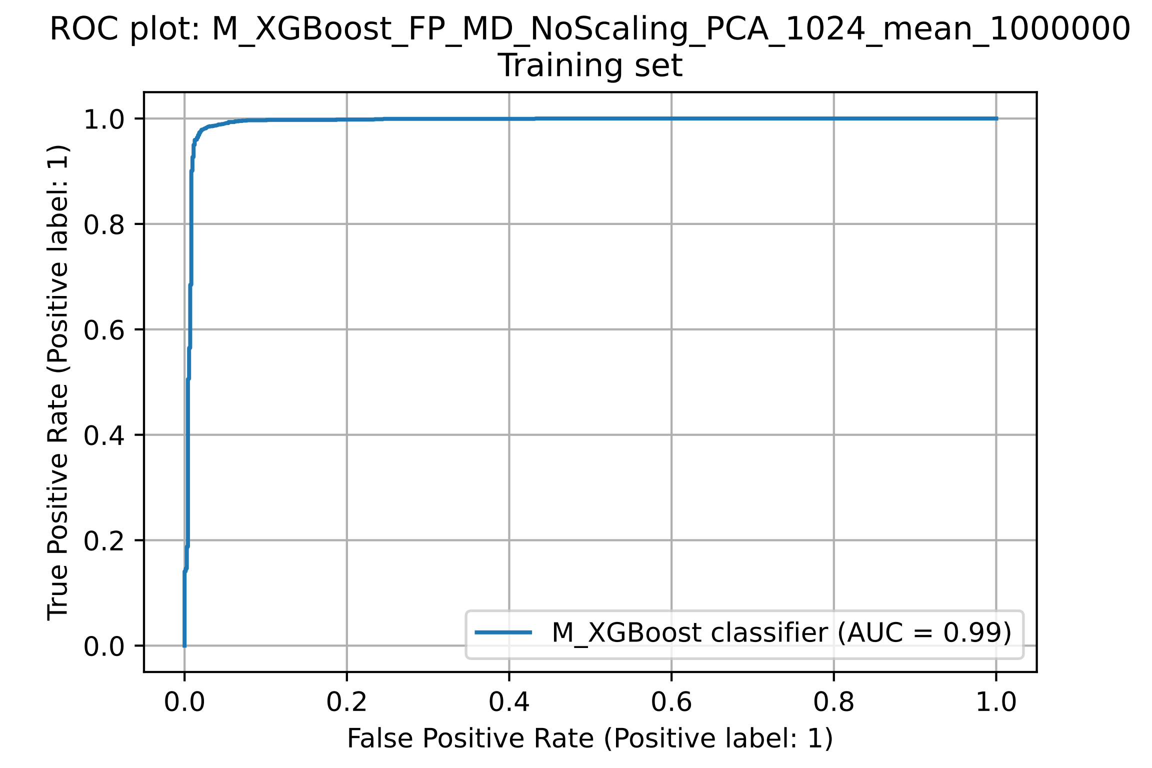

ROC plot: M_XGBoost_FP_MD_NoScaling_PCA_1024_mean_1000000
Training set
True Positive Rate (Positive label: 1)
False Positive Rate (Positive label: 1)
0.0
0.2
0.4
0.6
0.8
1.0
M_XGBoost classifier (AUC = 0.99)

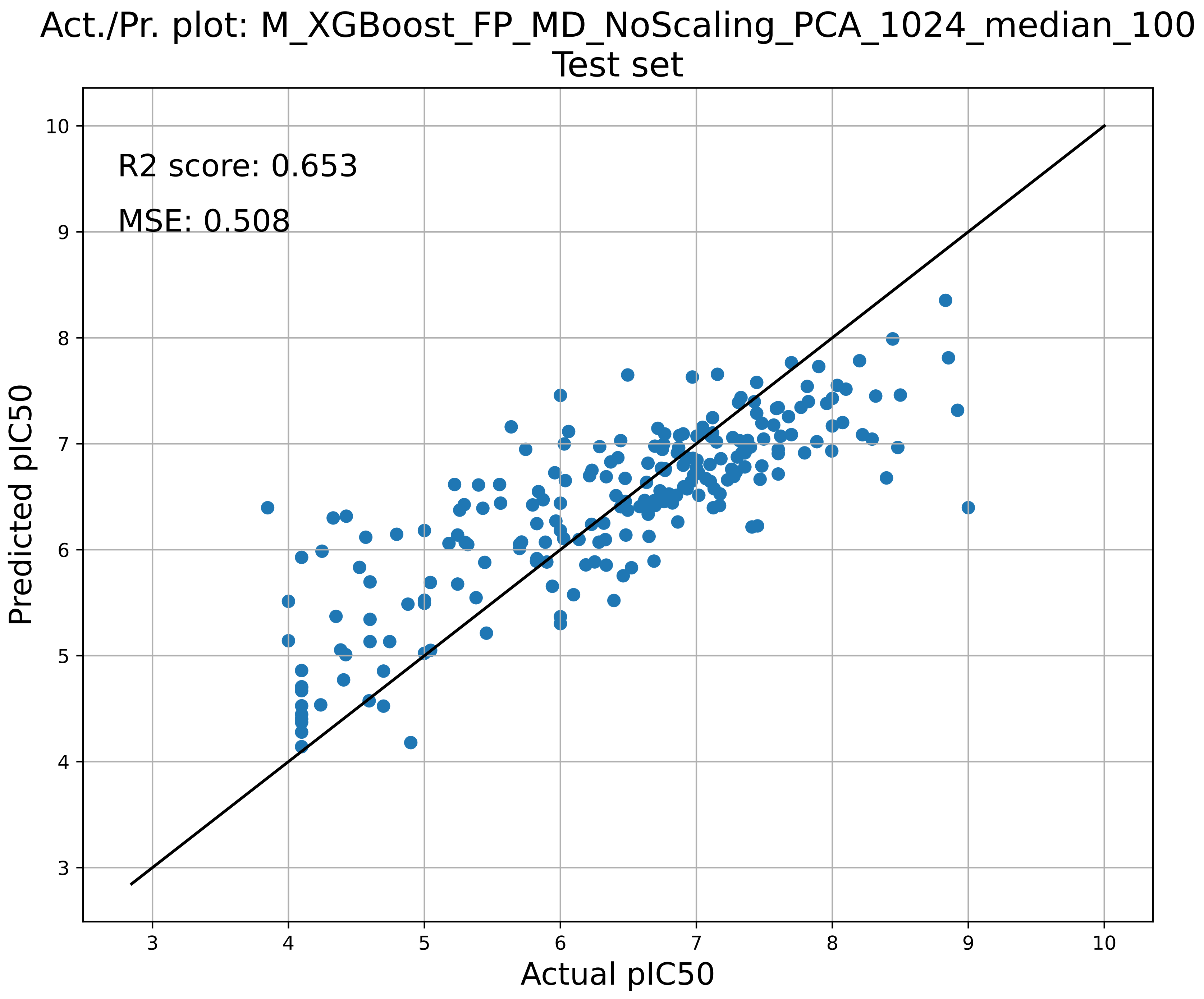

Act./Pr. plot: M_XGBoost_FP_MD_NoScaling_PCA_1024_median_100
Test set
R2 score: 0.653
MSE: 0.508
Predicted pIC50
Actual pIC50
3
4
5
6
7
8
9
10

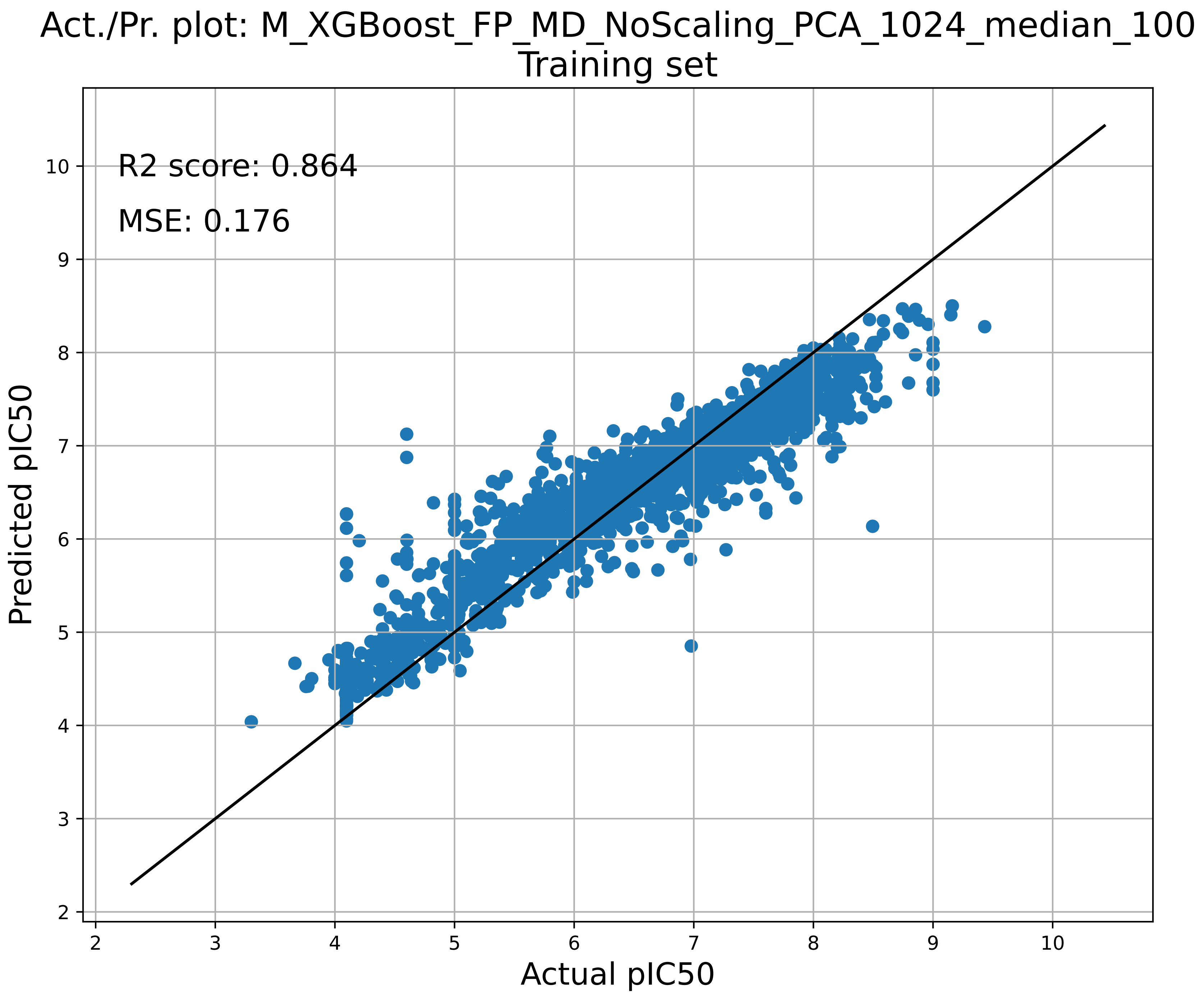
Act./Pr. plot: M_XGBoost_FP_MD_NoScaling_PCA_1024_median_100
Training set
R2 score: 0.864
MSE: 0.176
Predicted pIC50
Actual pIC50
2
3
4
5
6
7
8
9
10

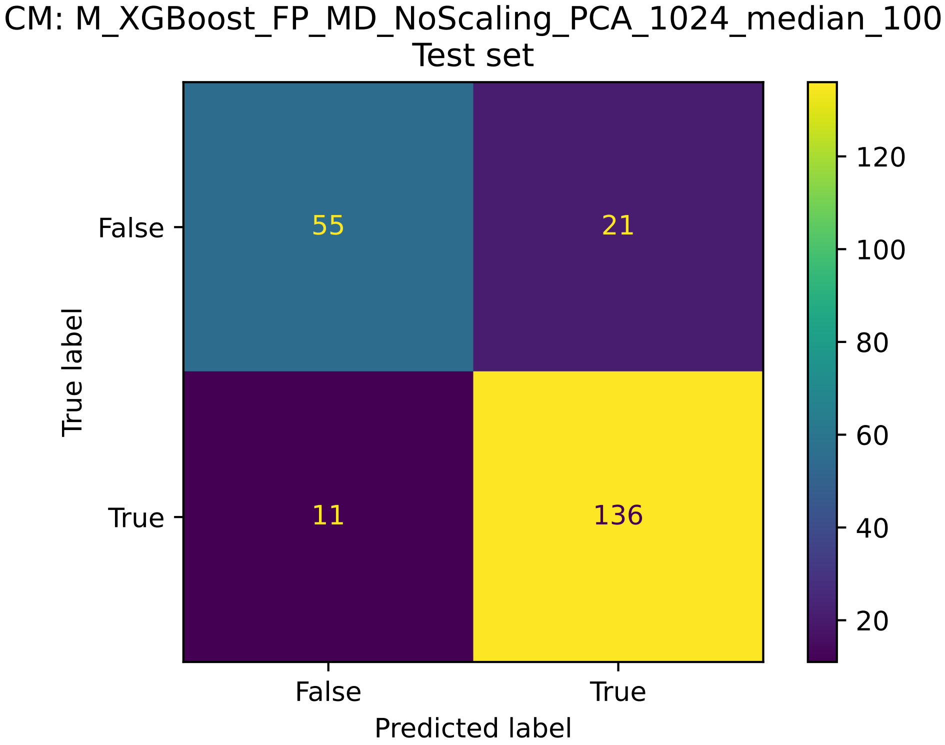
CM: M_XGBoost_FP_MD_NoScaling_PCA_1024_median_100
Test set
True label
False
True
55
21
11
136
Predicted label
False
True
20
40
60
80
100
120

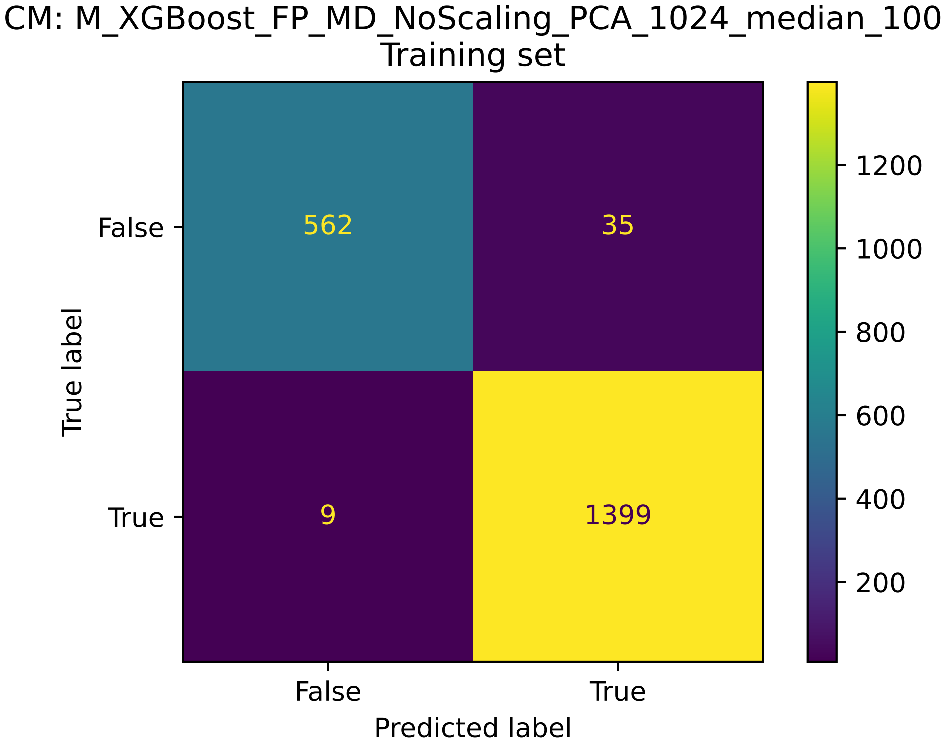

CM: M_XGBoost_FP_MD_NoScaling_PCA_1024_median_100
Training set
562
35
9
1399
False
True
True label
False
True
Predicted label
200
400
600
800
1000
1200

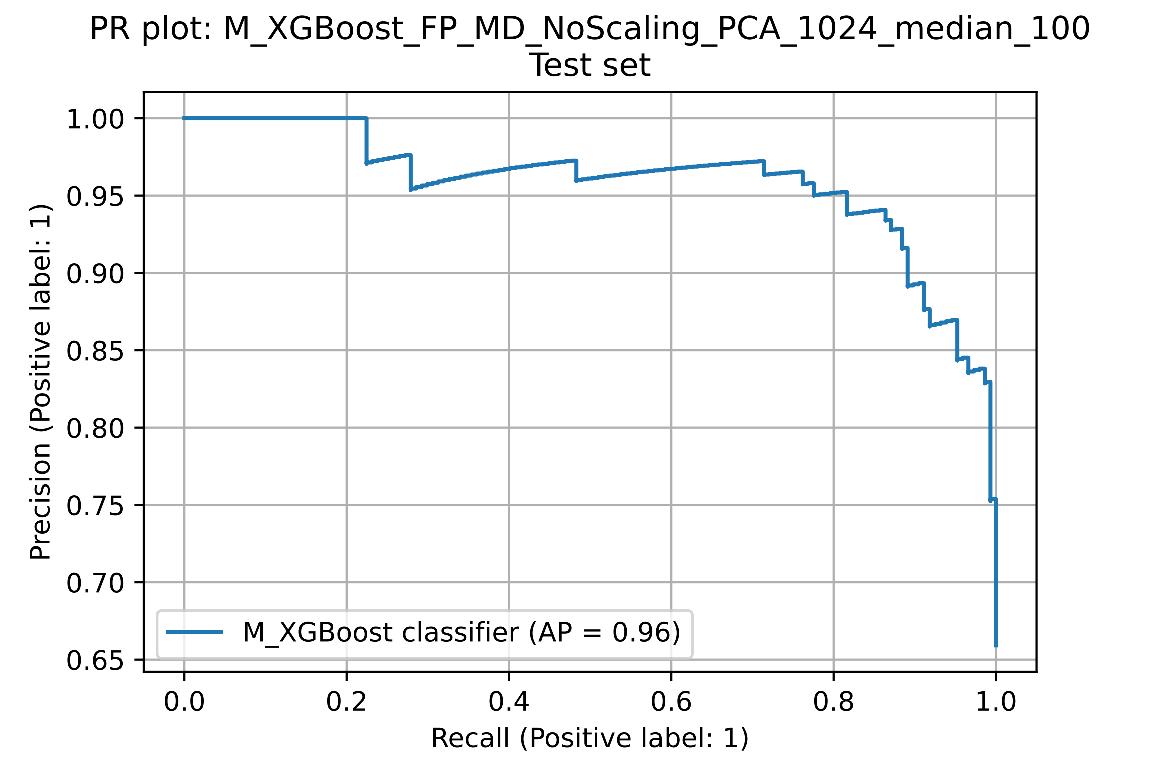
PR plot: M_XGBoost_FP_MD_NoScaling_PCA_1024_median_100
Test set
Precision (Positive label: 1)
Recall (Positive label: 1)
1.00
0.95
0.90
0.85
0.80
0.75
0.70
0.65
0.0
0.2
0.4
0.6
0.8
1.0
M_XGBoost classifier (AP = 0.96)

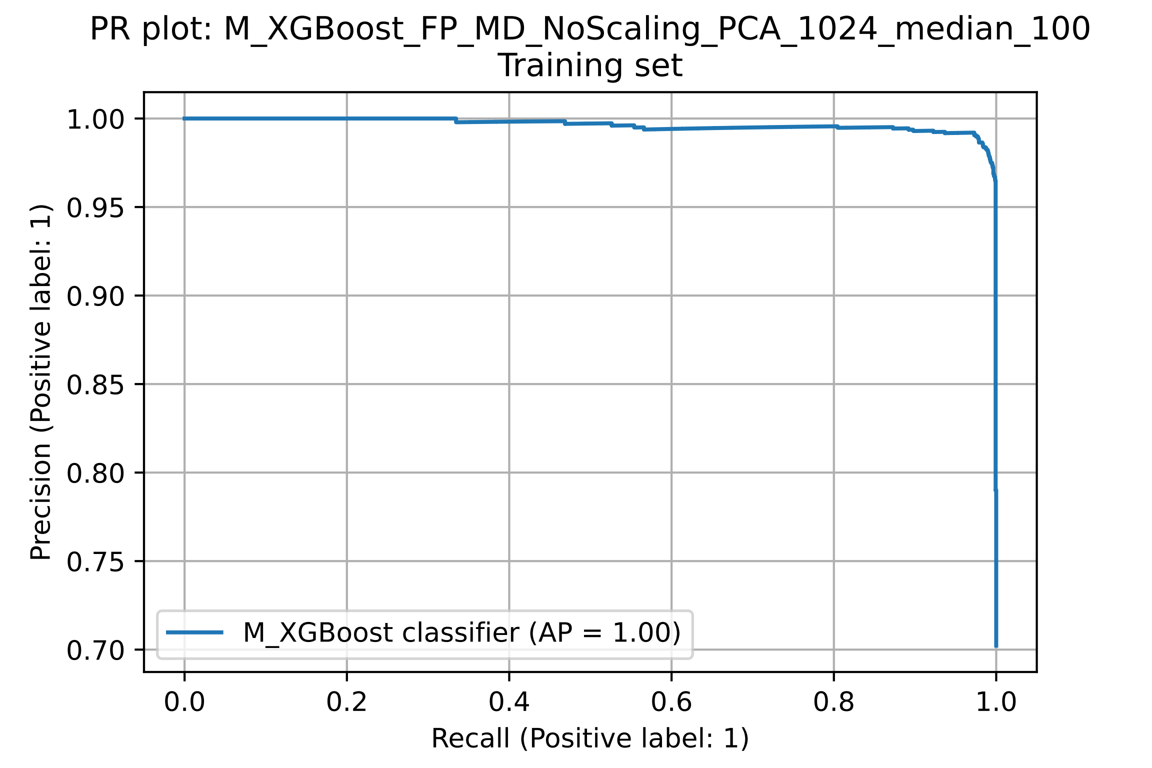
PR plot: M_XGBoost_FP_MD_NoScaling_PCA_1024_median_100
Training set
Precision (Positive label: 1)
Recall (Positive label: 1)
1.00
0.95
0.90
0.85
0.80
0.75
0.70
0.0
0.2
0.4
0.6
0.8
1.0
M_XGBoost classifier (AP = 1.00)

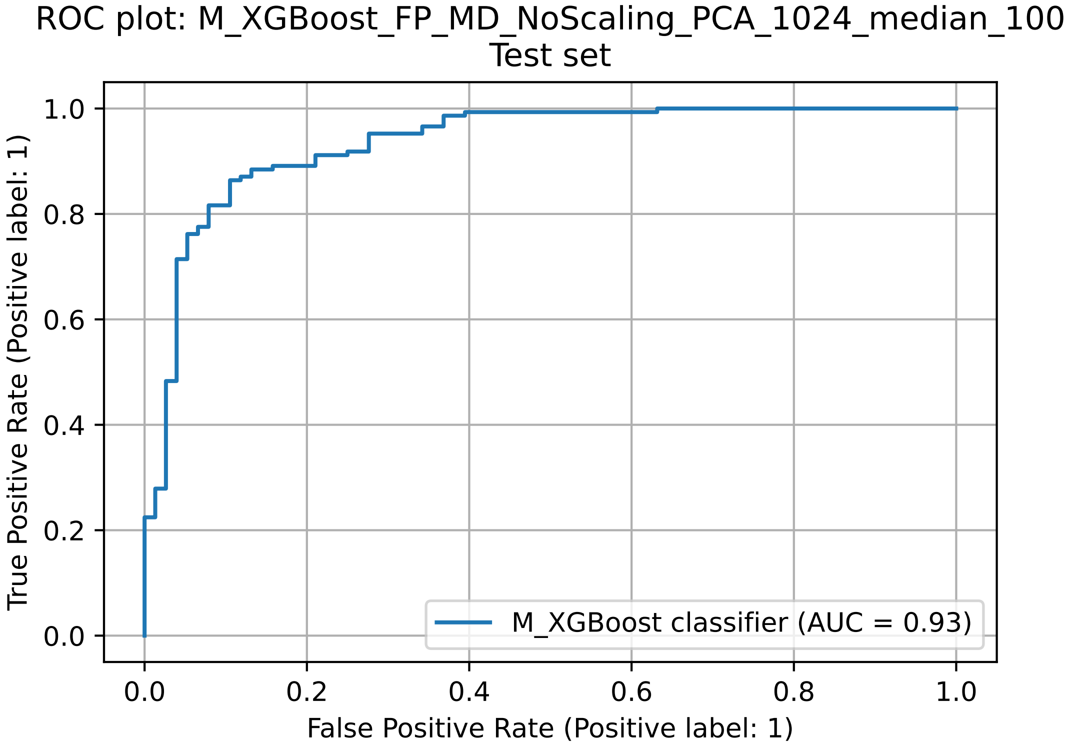
ROC plot: M_XGBoost_FP_MD_NoScaling_PCA_1024_median_100
Test set
True Positive Rate (Positive label: 1)
False Positive Rate (Positive label: 1)
0.0
0.2
0.4
0.6
0.8
1.0
M_XGBoost classifier (AUC = 0.93)

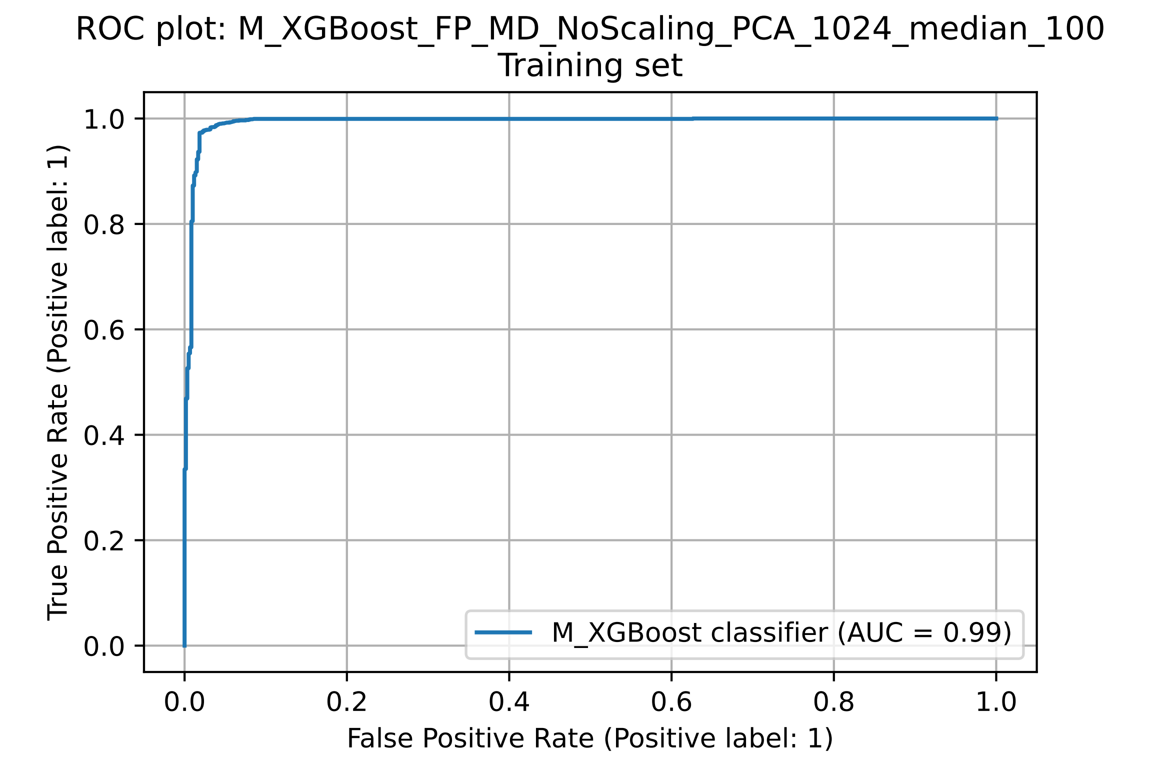
ROC plot: M_XGBoost_FP_MD_NoScaling_PCA_1024_median_100
Training set
True Positive Rate (Positive label: 1)
False Positive Rate (Positive label: 1)
0.0
0.2
0.4
0.6
0.8
1.0
M_XGBoost classifier (AUC = 0.99)

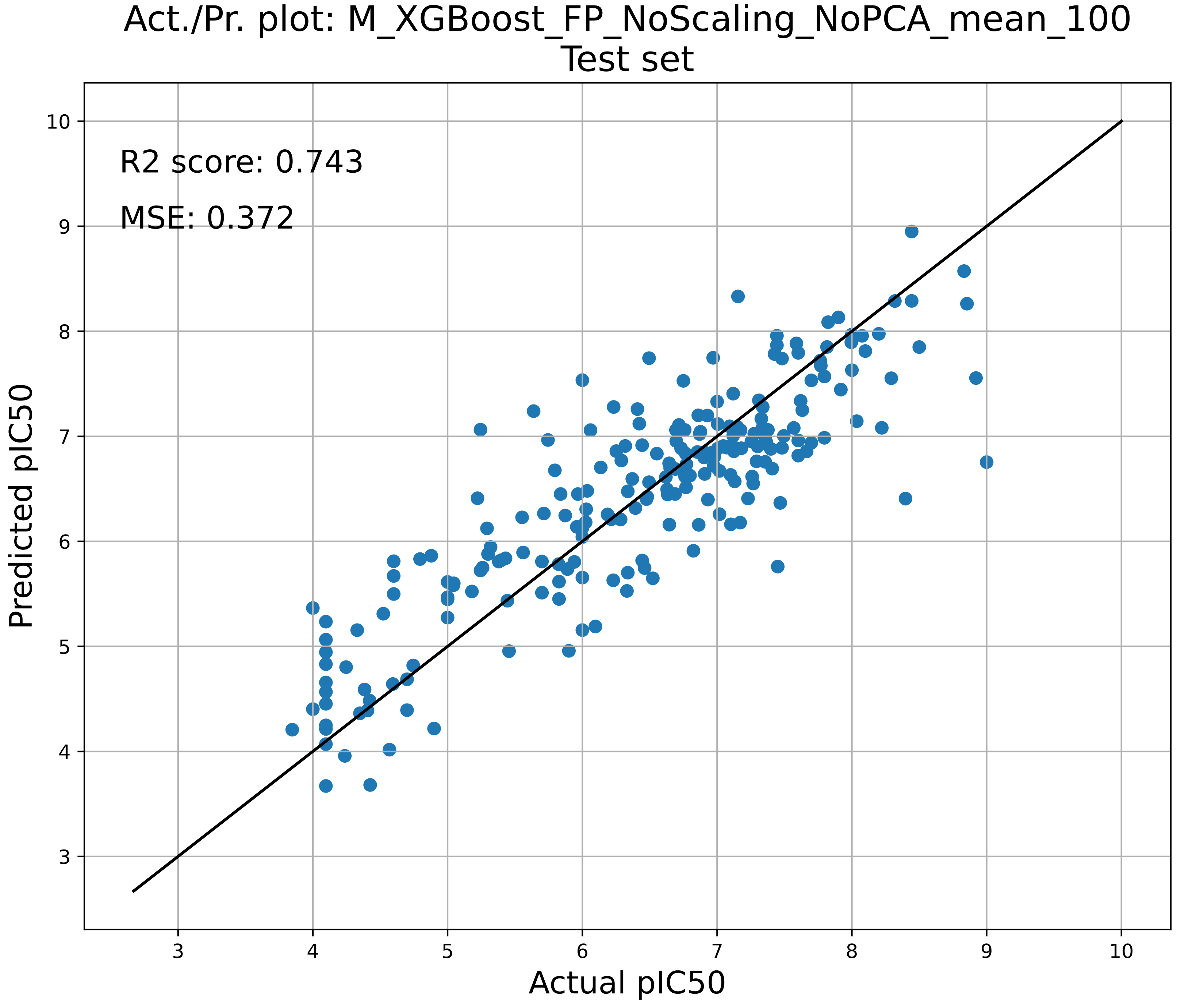
Act./Pr. plot: M_XGBoost_FP_NoScaling_NoPCA_mean_100
Test set
R2 score: 0.743
MSE: 0.372
Predicted pIC50
Actual pIC50
10
9
8
7
6
5
4
3
3
4
5
6
7
8
9
10

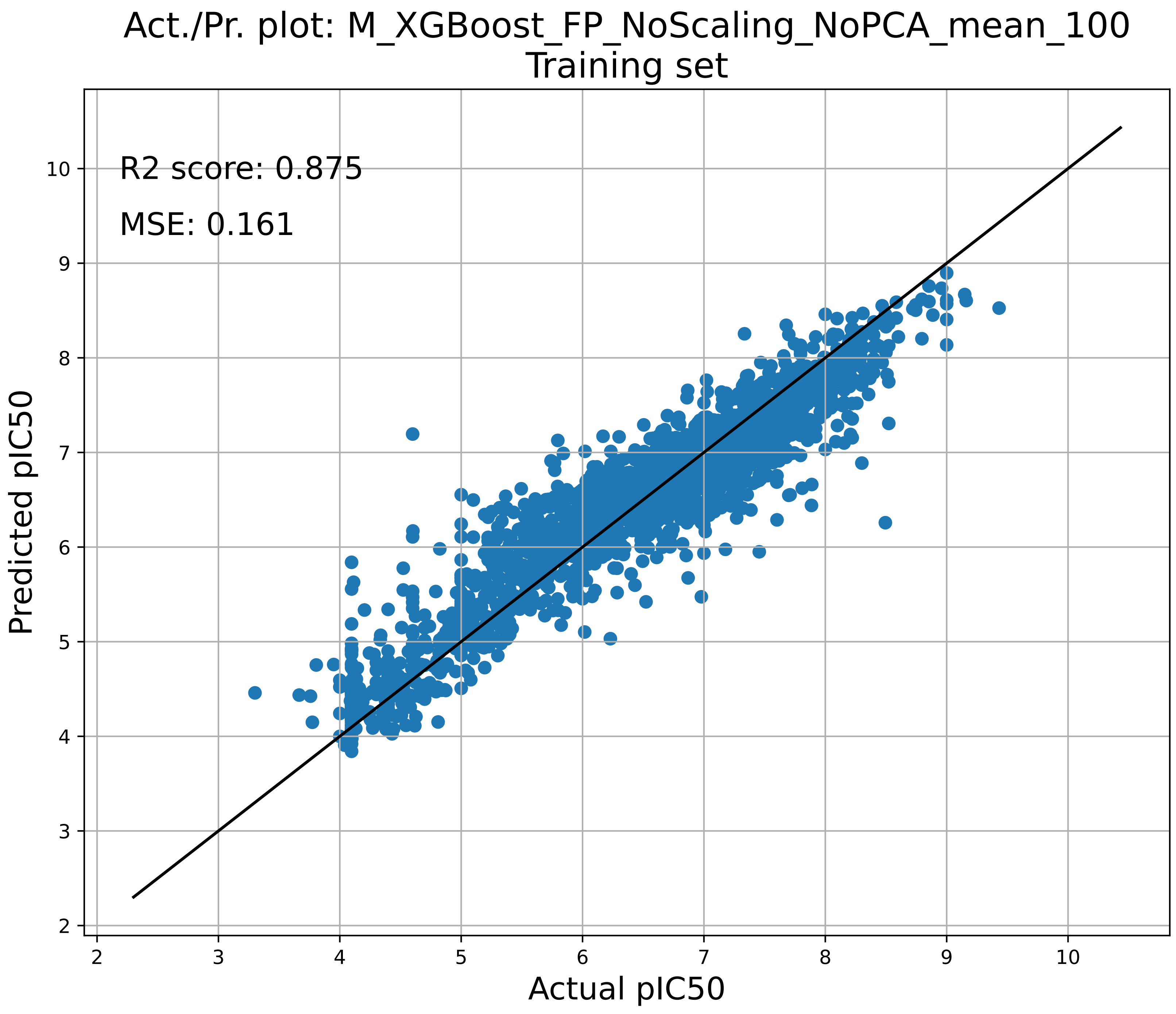
Act./Pr. plot: M_XGBoost_FP_NoScaling_NoPCA_mean_100
Training set
R2 score: 0.875
MSE: 0.161
Predicted pIC50
Actual pIC50
2
3
4
5
6
7
8
9
10

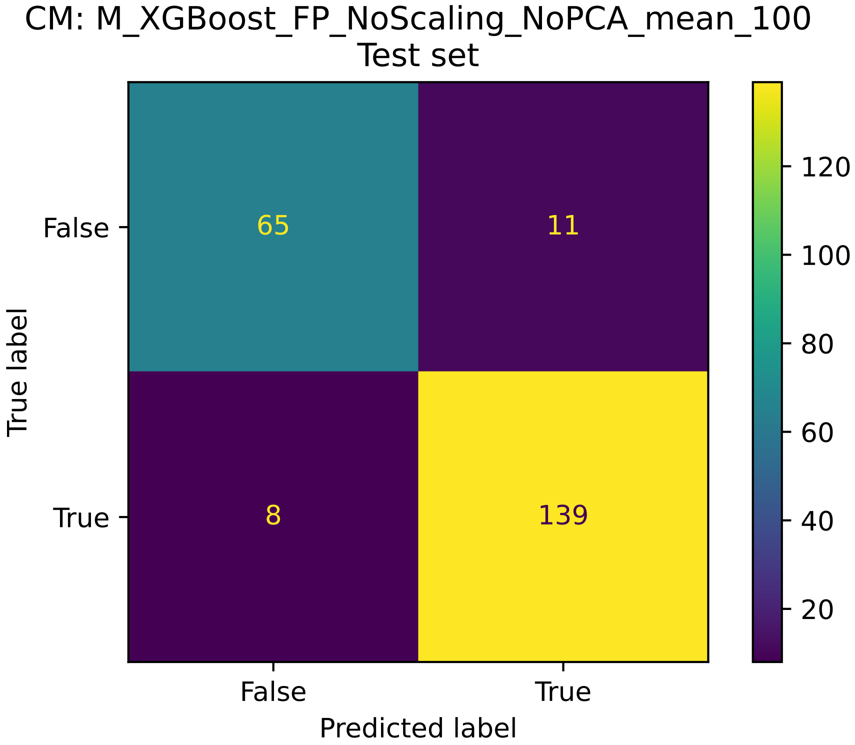
CM: M_XGBoost_FP_NoScaling_NoPCA_mean_100
Test set
True label
Predicted label
False
True
False
True
65
11
8
139
20
40
60
80
100
120

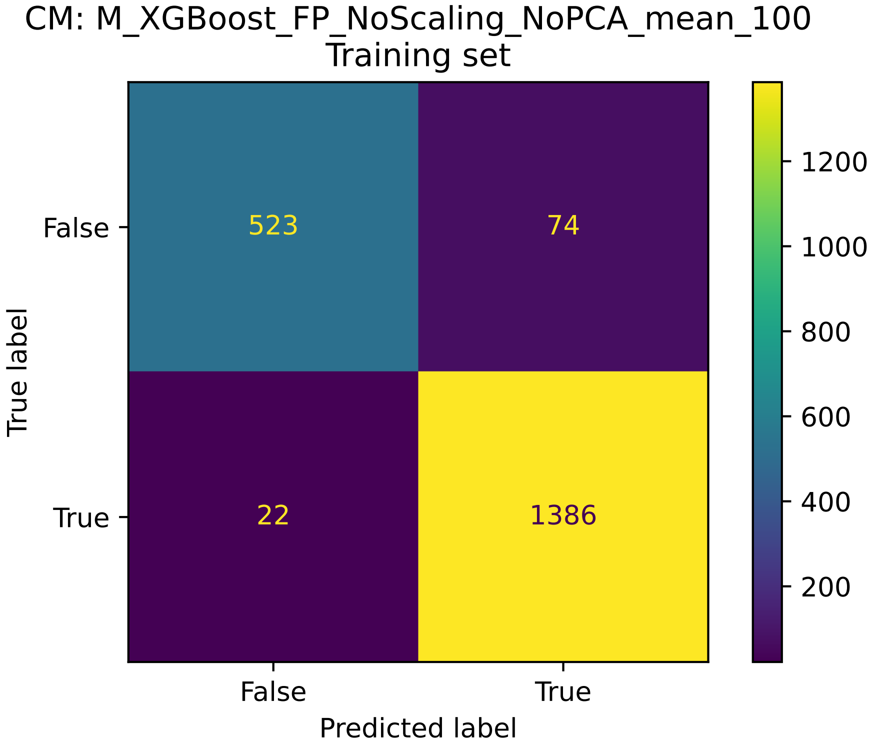

CM: M_XGBoost_FP_NoScaling_NoPCA_mean_100
Training set
True label
False
True
523
74
22
1386
Predicted label
False
True
1200
1000
800
600
400
200

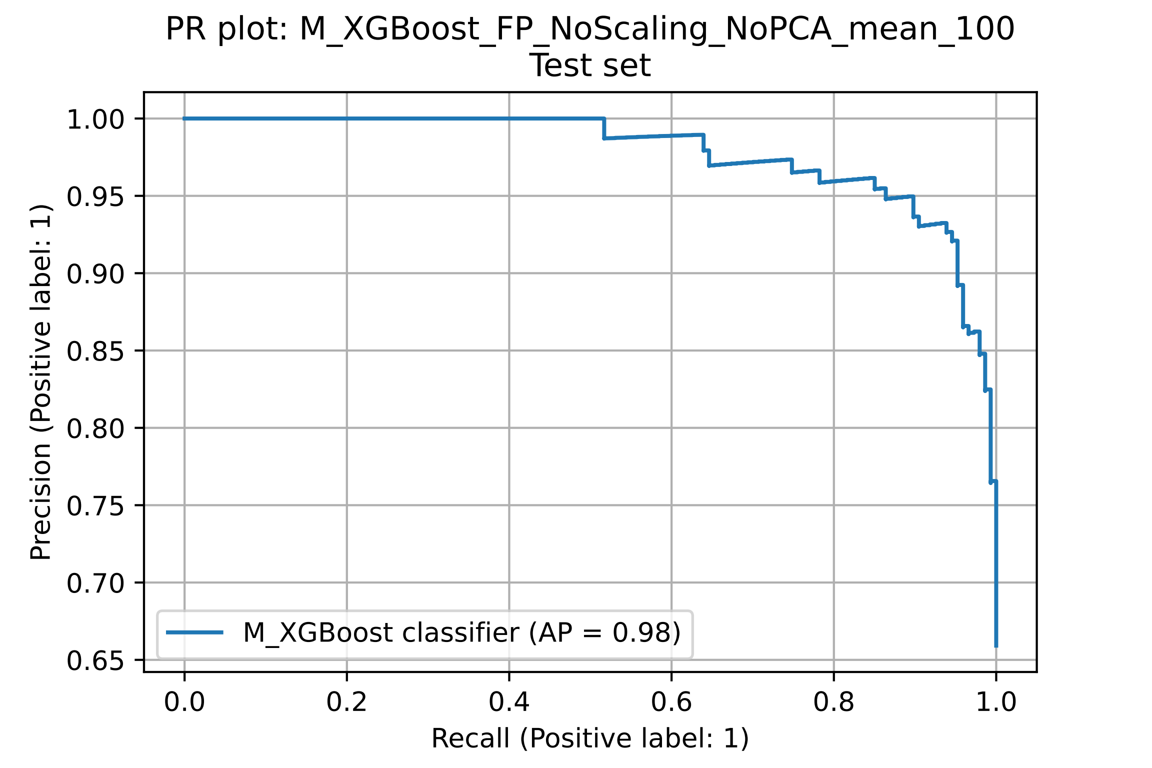
PR plot: M_XGBoost_FP_NoScaling_NoPCA_mean_100
Test set
Precision (Positive label: 1)
Recall (Positive label: 1)
M_XGBoost classifier (AP = 0.98)
1.00
0.95
0.90
0.85
0.80
0.75
0.70
0.65
0.0
0.2
0.4
0.6
0.8
1.0

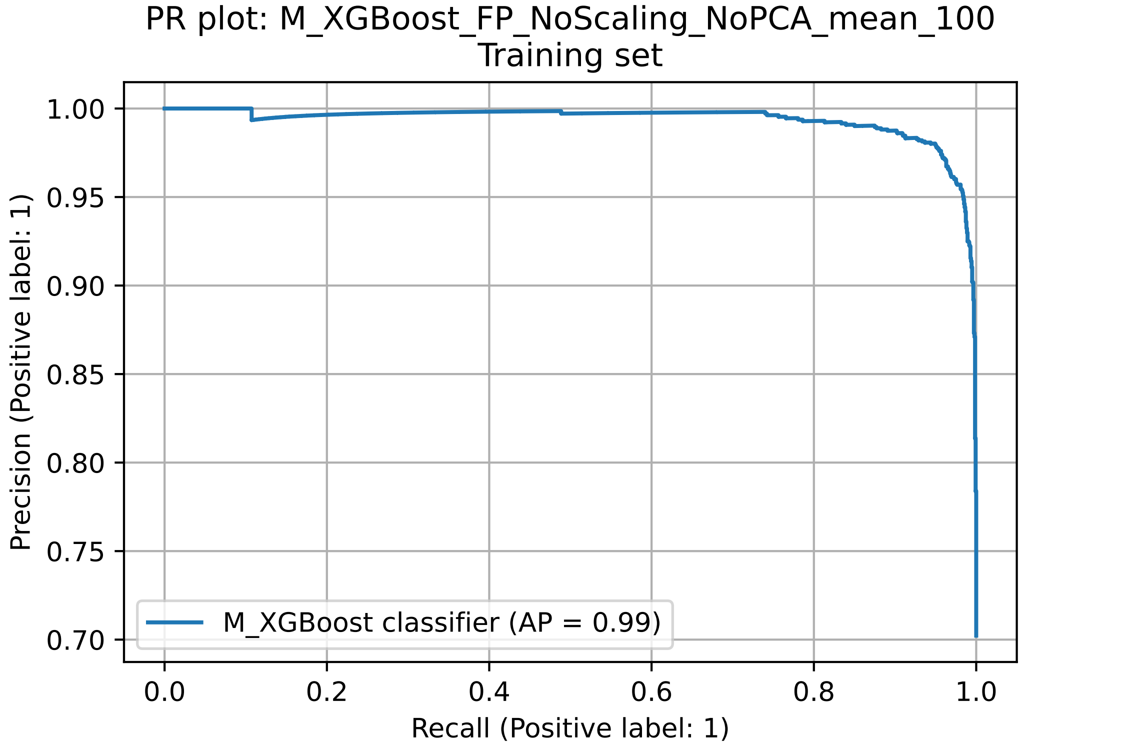

PR plot: M_XGBoost_FP_NoScaling_NoPCA_mean_100
Training set
Precision (Positive label: 1)
Recall (Positive label: 1)
M_XGBoost classifier (AP = 0.99)
1.00
0.95
0.90
0.85
0.80
0.75
0.70
0.0
0.2
0.4
0.6
0.8
1.0

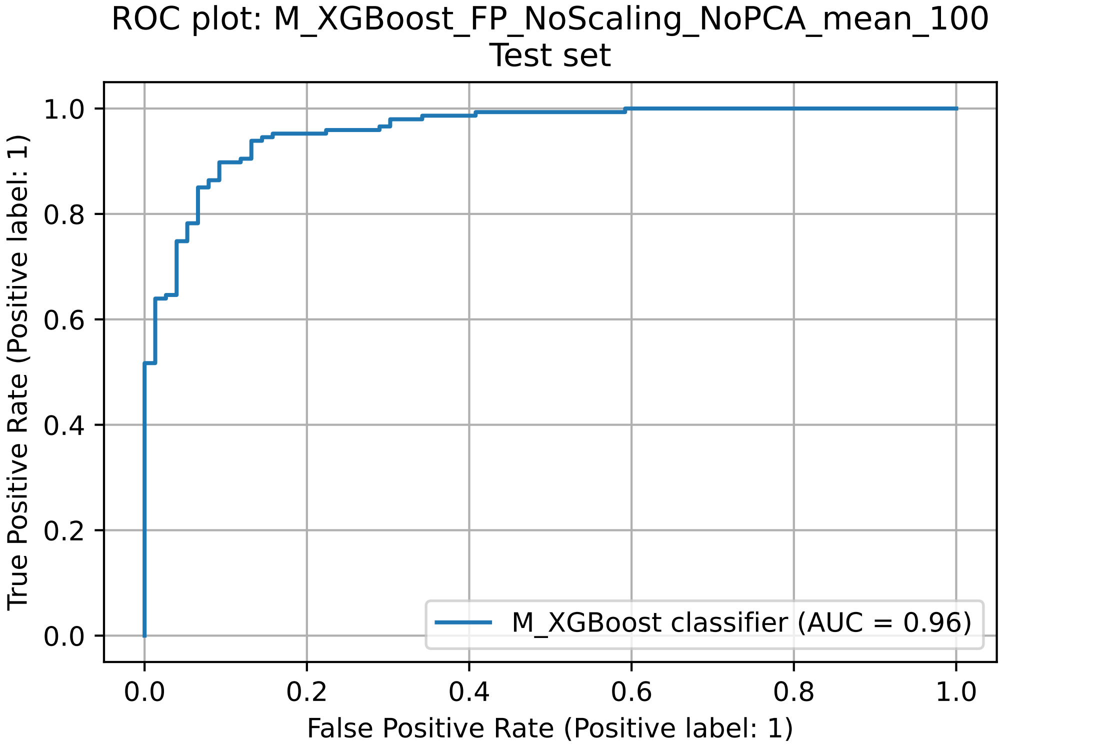
ROC plot: M_XGBoost_FP_NoScaling_NoPCA_mean_100
Test set
True Positive Rate (Positive label: 1)
False Positive Rate (Positive label: 1)
0.0
0.2
0.4
0.6
0.8
1.0
M_XGBoost classifier (AUC = 0.96)

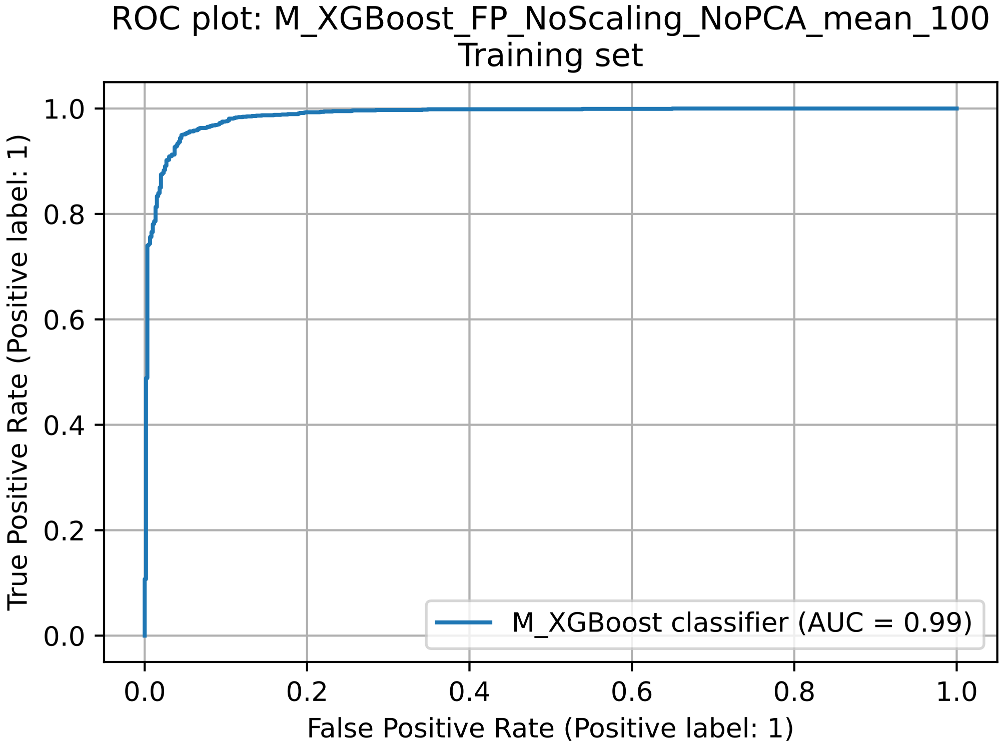
ROC plot: M_XGBoost_FP_NoScaling_NoPCA_mean_100
Training set
True Positive Rate (Positive label: 1)
False Positive Rate (Positive label: 1)
0.0
0.2
0.4
0.6
0.8
1.0
M_XGBoost classifier (AUC = 0.99)

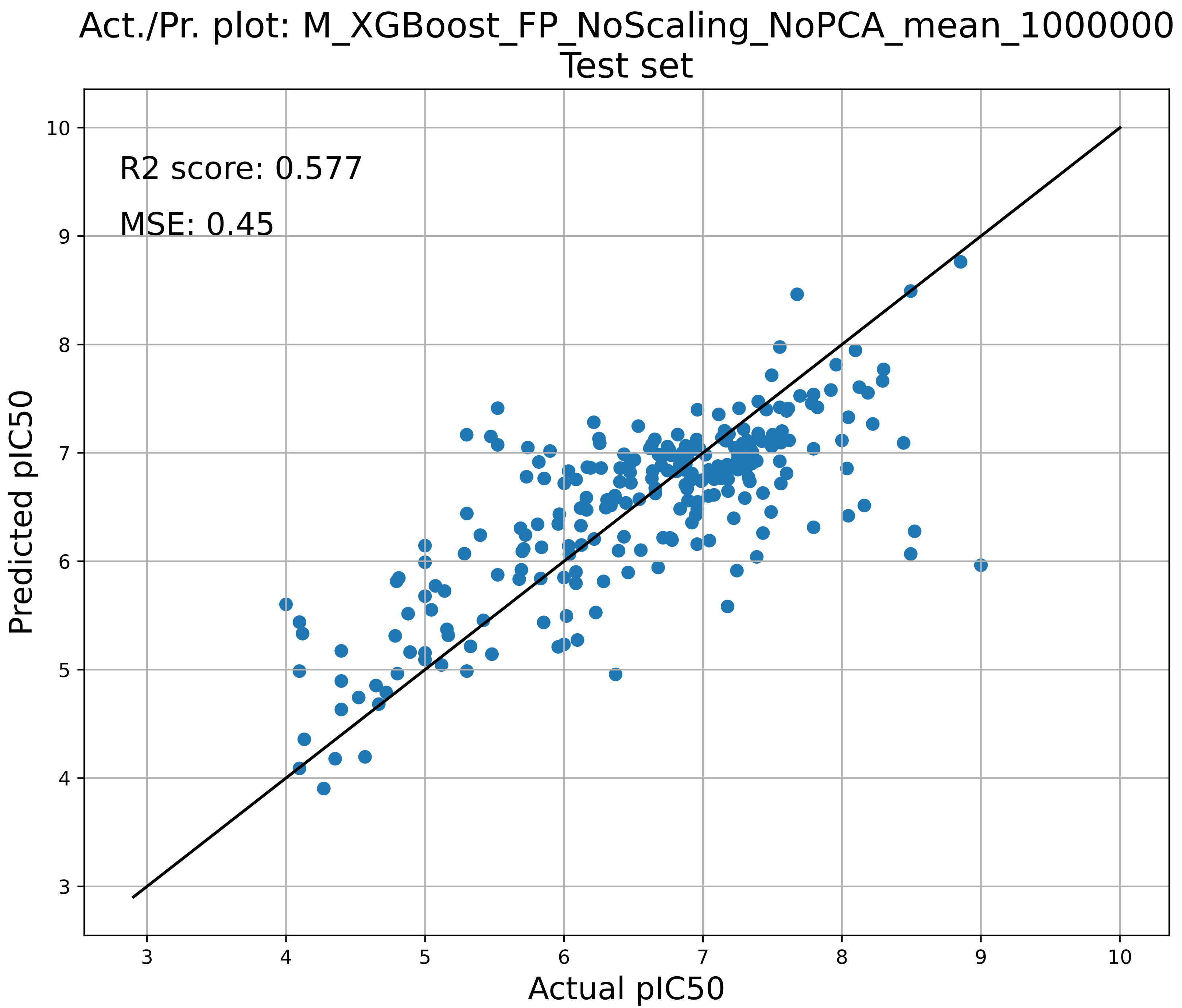

Act./Pr. plot: M_XGBoost_FP_NoScaling_NoPCA_mean_1000000
Test set
R2 score: 0.577
MSE: 0.45
Predicted pIC50
Actual pIC50
3
4
5
6
7
8
9
10

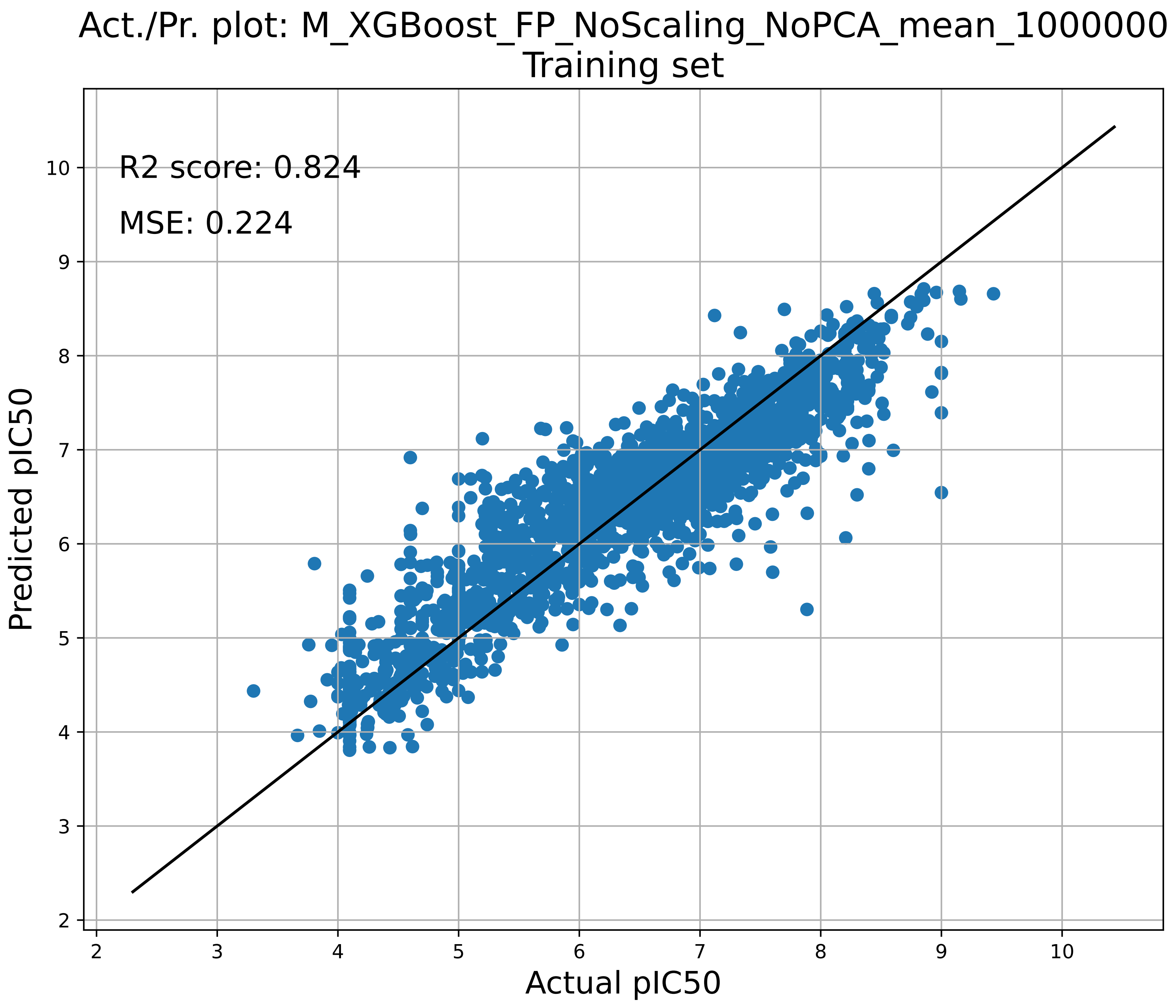
Act./Pr. plot: M_XGBoost_FP_NoScaling_NoPCA_mean_1000000
Training set
R2 score: 0.824
MSE: 0.224
Predicted pIC50
Actual pIC50
2
3
4
5
6
7
8
9
10

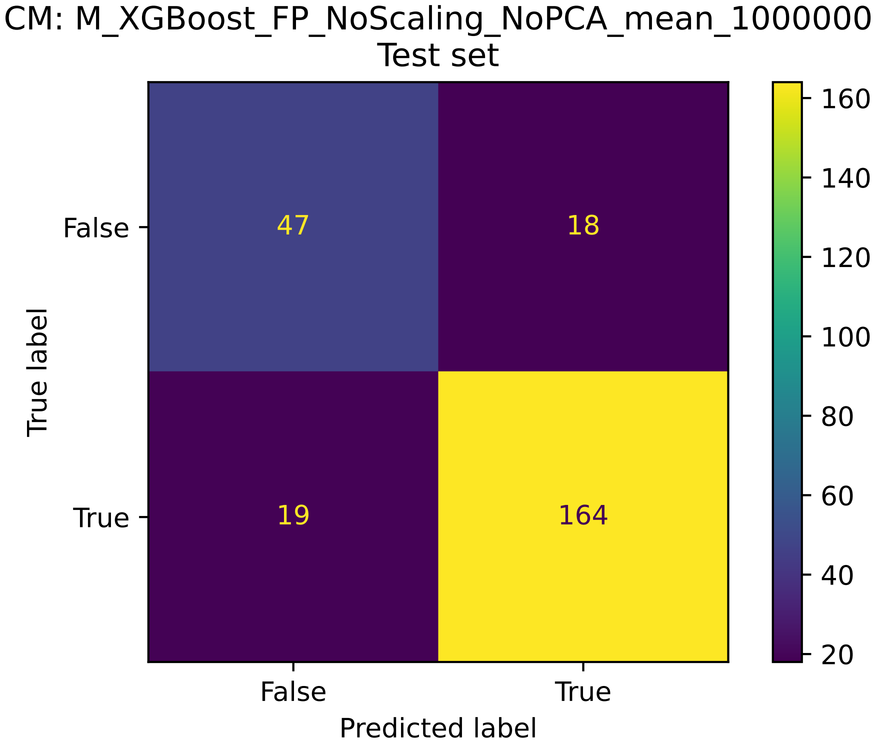
CM: M_XGBoost_FP_NoScaling_NoPCA_mean_1000000
Test set
True label
False
True
47
18
19
164
Predicted label
False
True
160
140
120
100
80
60
40
20

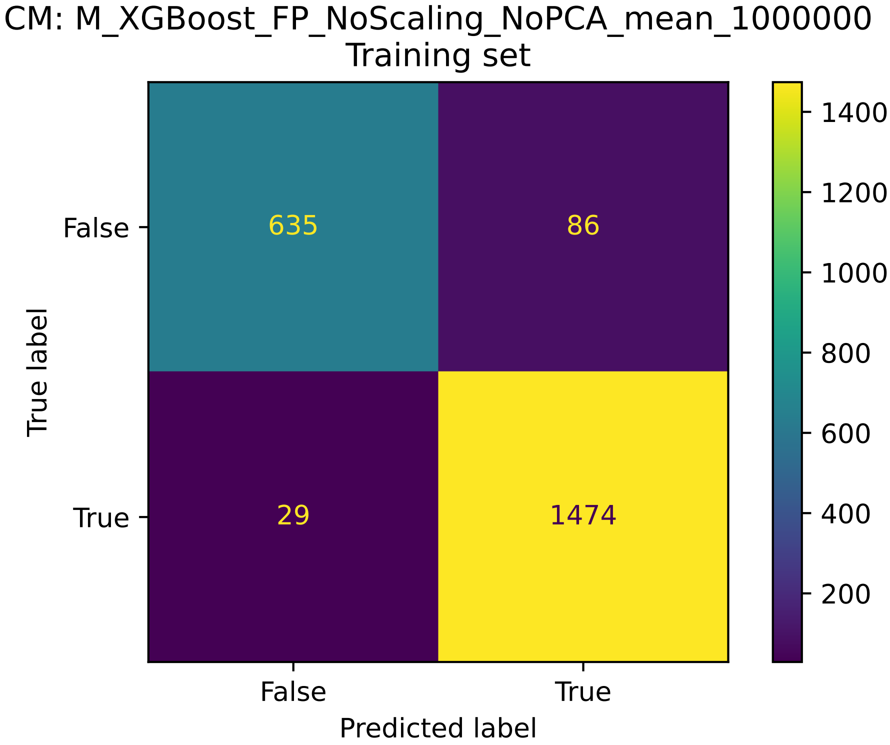
CM: M_XGBoost_FP_NoScaling_NoPCA_mean_1000000
Training set
True label
Predicted label
False
True
False
True
635
86
29
1474
1400
1200
1000
800
600
400
200

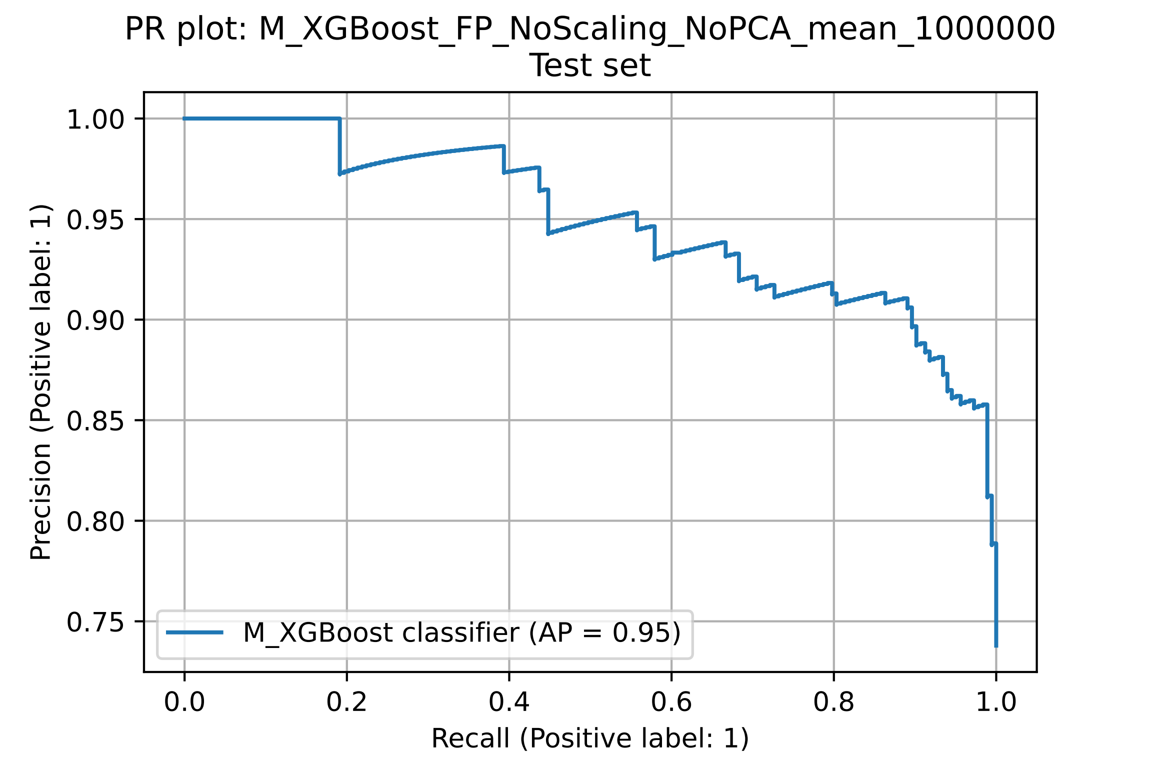

PR plot: M_XGBoost_FP_NoScaling_NoPCA_mean_1000000
Test set
Precision (Positive label: 1)
Recall (Positive label: 1)
1.00
0.95
0.90
0.85
0.80
0.75
0.0
0.2
0.4
0.6
0.8
1.0
M_XGBoost classifier (AP = 0.95)

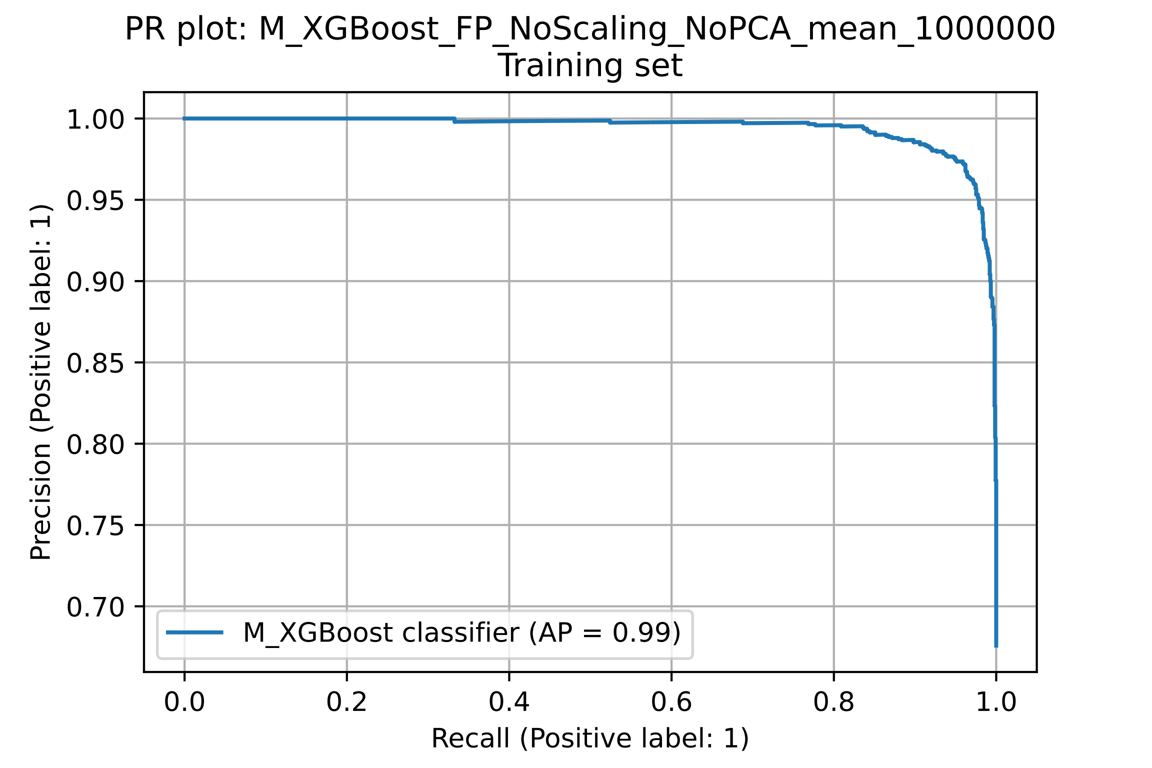
PR plot: M_XGBoost_FP_NoScaling_NoPCA_mean_1000000
Training set
1.00
0.95
0.90
0.85
0.80
0.75
0.70
Precision (Positive label: 1)
0.0
0.2
0.4
0.6
0.8
1.0
Recall (Positive label: 1)
M_XGBoost classifier (AP = 0.99)

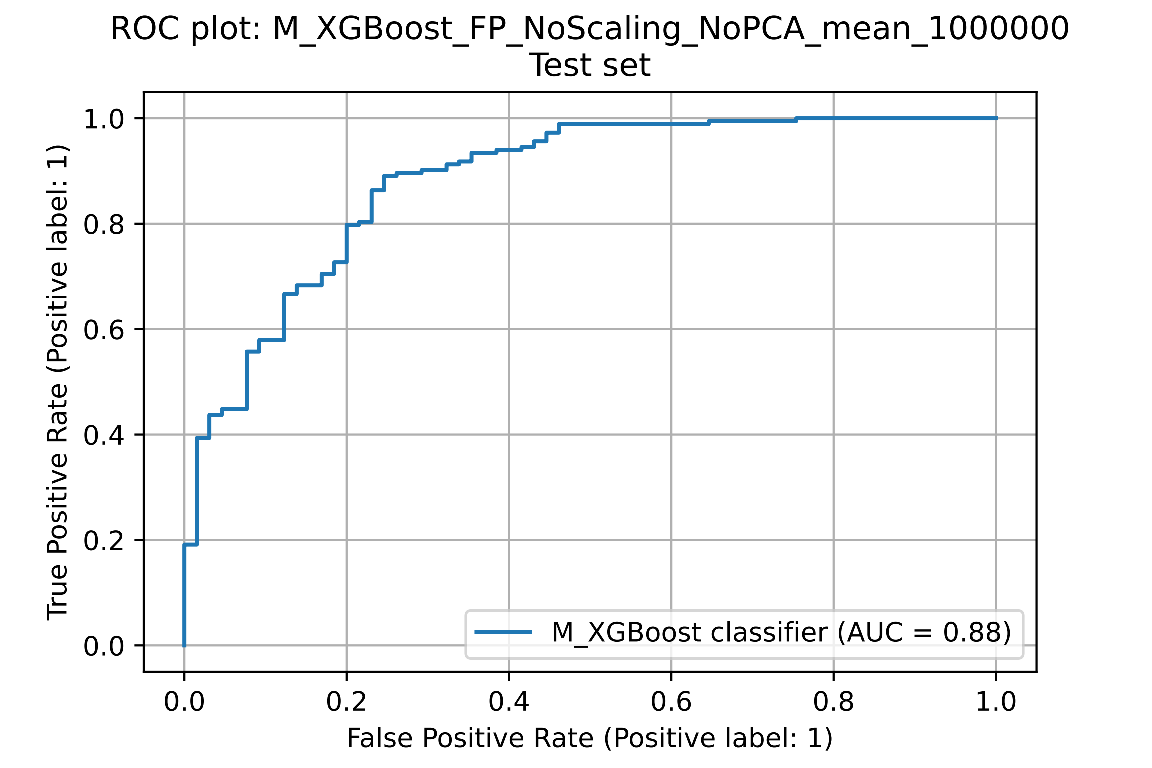
ROC plot: M_XGBoost_FP_NoScaling_NoPCA_mean_1000000
Test set
True Positive Rate (Positive label: 1)
False Positive Rate (Positive label: 1)
0.0
0.2
0.4
0.6
0.8
1.0
M_XGBoost classifier (AUC = 0.88)

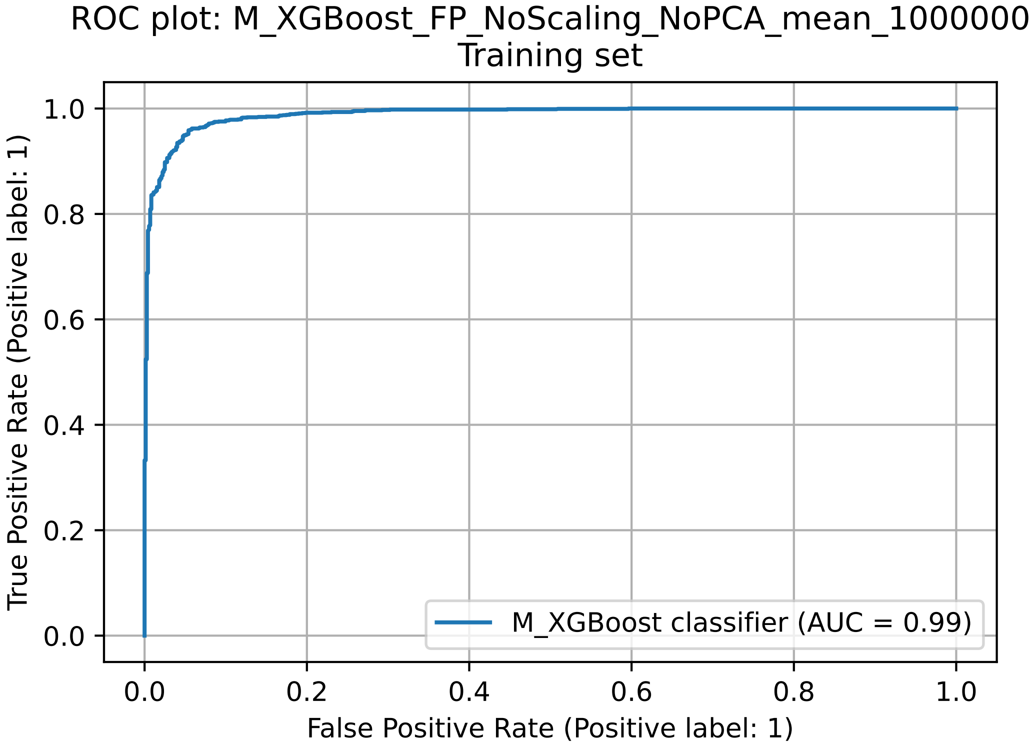
ROC plot: M_XGBoost_FP_NoScaling_NoPCA_mean_1000000
Training set
True Positive Rate (Positive label: 1)
False Positive Rate (Positive label: 1)
0.0
0.2
0.4
0.6
0.8
1.0
M_XGBoost classifier (AUC = 0.99)

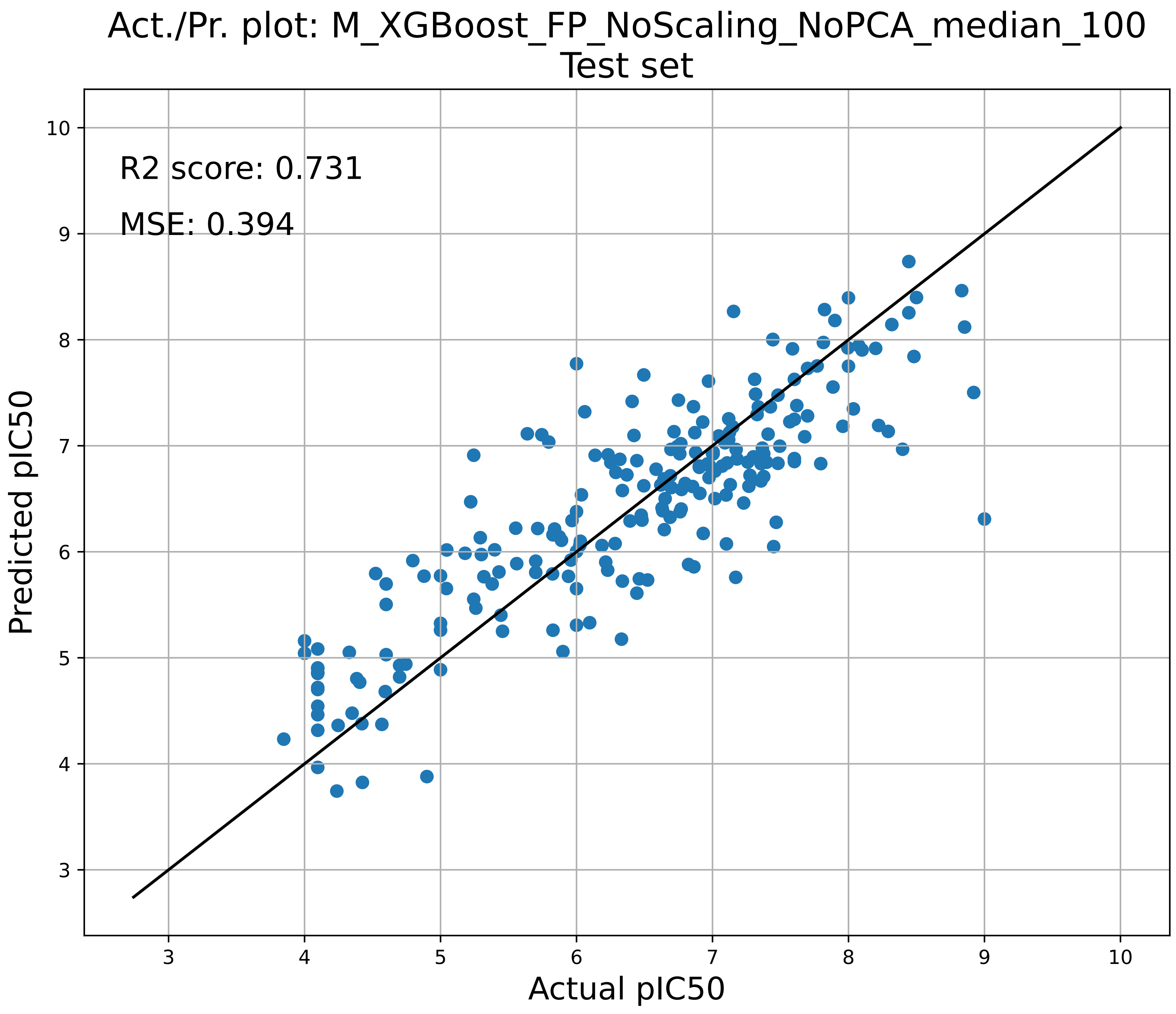

Act./Pr. plot: M_XGBoost_FP_NoScaling_NoPCA_median_100
Test set
R2 score: 0.731
MSE: 0.394
Predicted pIC50
Actual pIC50
10
9
8
7
6
5
4
3
3
4
5
6
7
8
9
10

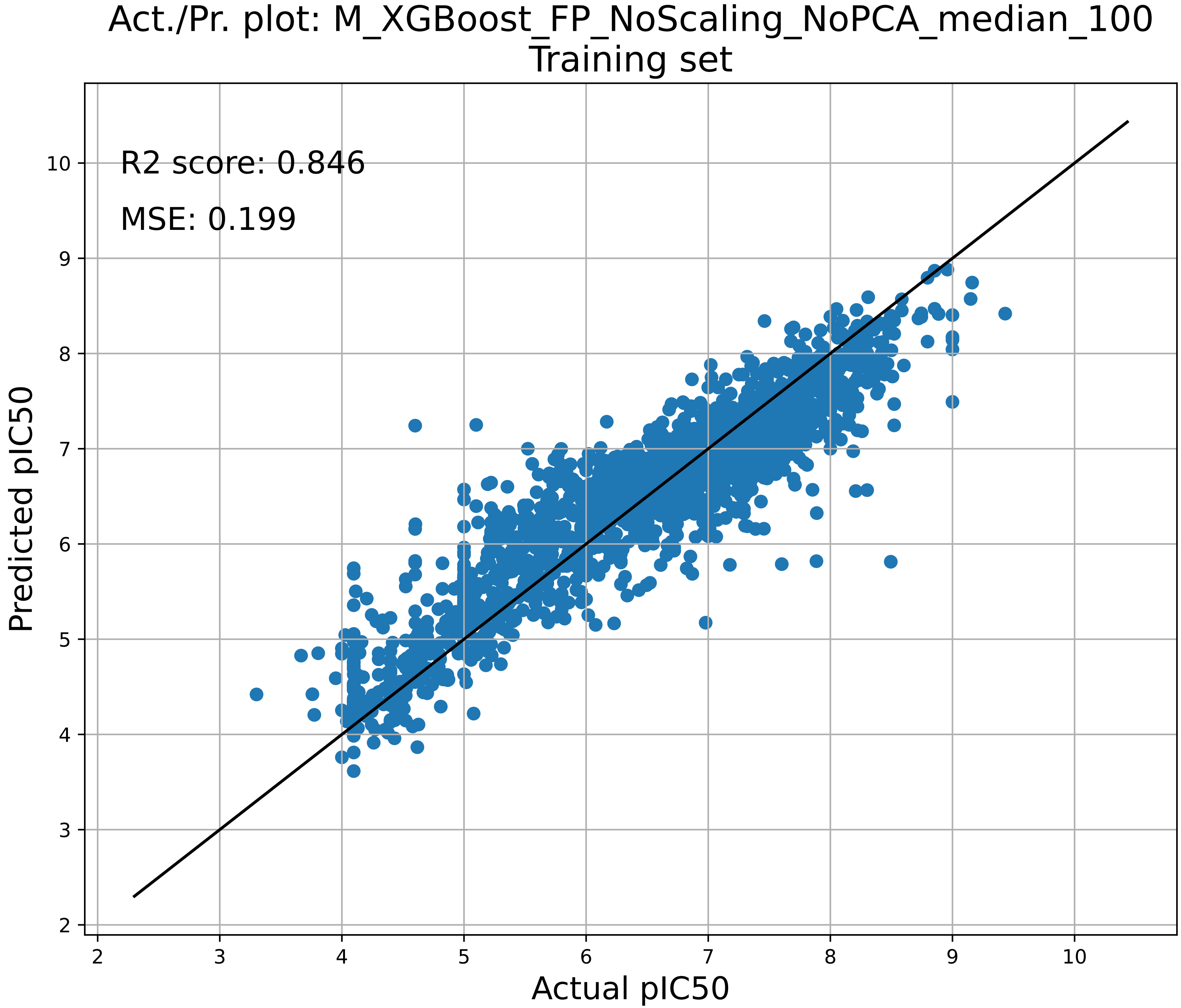

Act./Pr. plot: M_XGBoost_FP_NoScaling_NoPCA_median_100
Training set
R2 score: 0.846
MSE: 0.199
Predicted pIC50
Actual pIC50
2
3
4
5
6
7
8
9
10

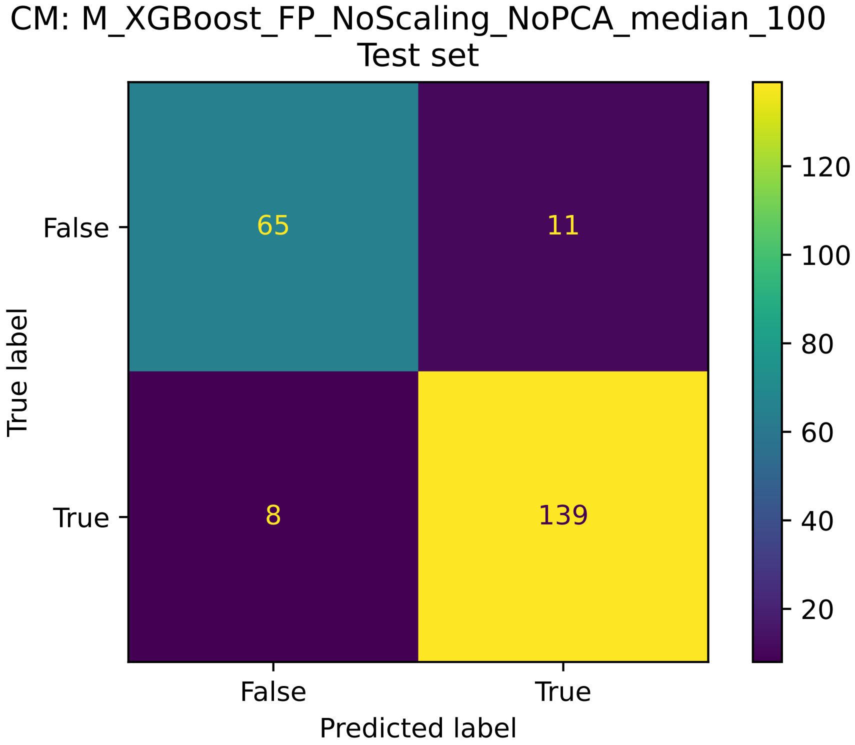
CM: M_XGBoost_FP_NoScaling_NoPCA_median_100
Test set
True label
Predicted label
False
True
False
True
65
11
8
139
20
40
60
80
100
120

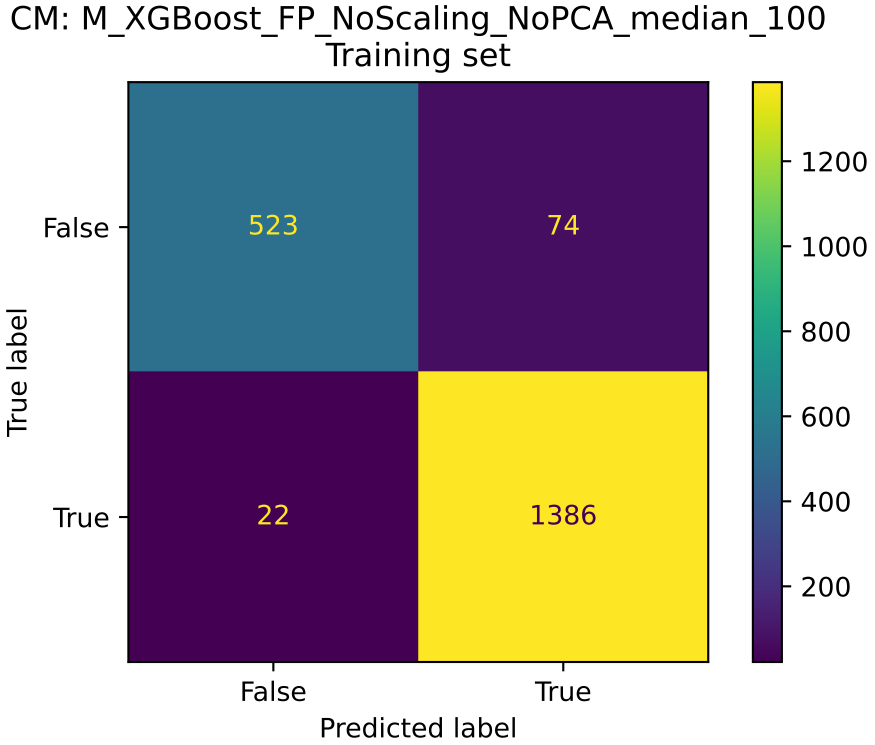
CM: M_XGBoost_FP_NoScaling_NoPCA_median_100
Training set
True label
False
True
523
74
22
1386
Predicted label
False
True
1200
1000
800
600
400
200

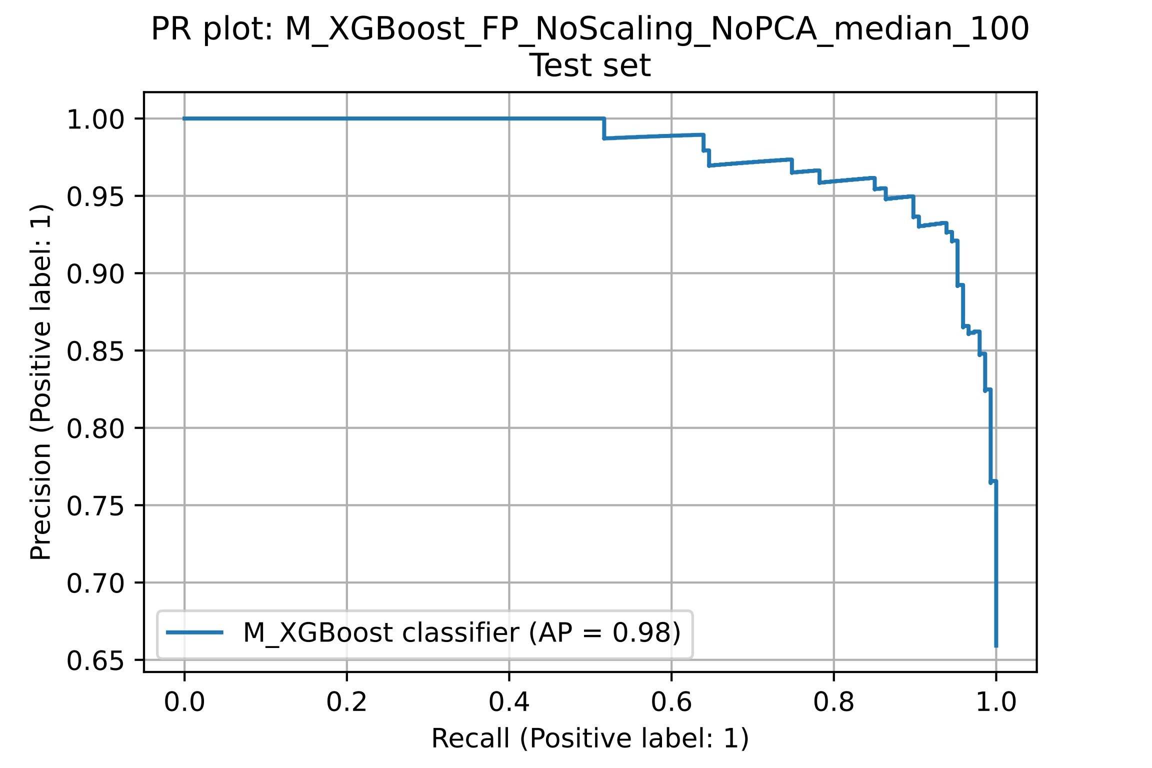
PR plot: M_XGBoost_FP_NoScaling_NoPCA_median_100
Test set
Precision (Positive label: 1)
Recall (Positive label: 1)
1.00
0.95
0.90
0.85
0.80
0.75
0.70
0.65
0.0
0.2
0.4
0.6
0.8
1.0
M_XGBoost classifier (AP = 0.98)

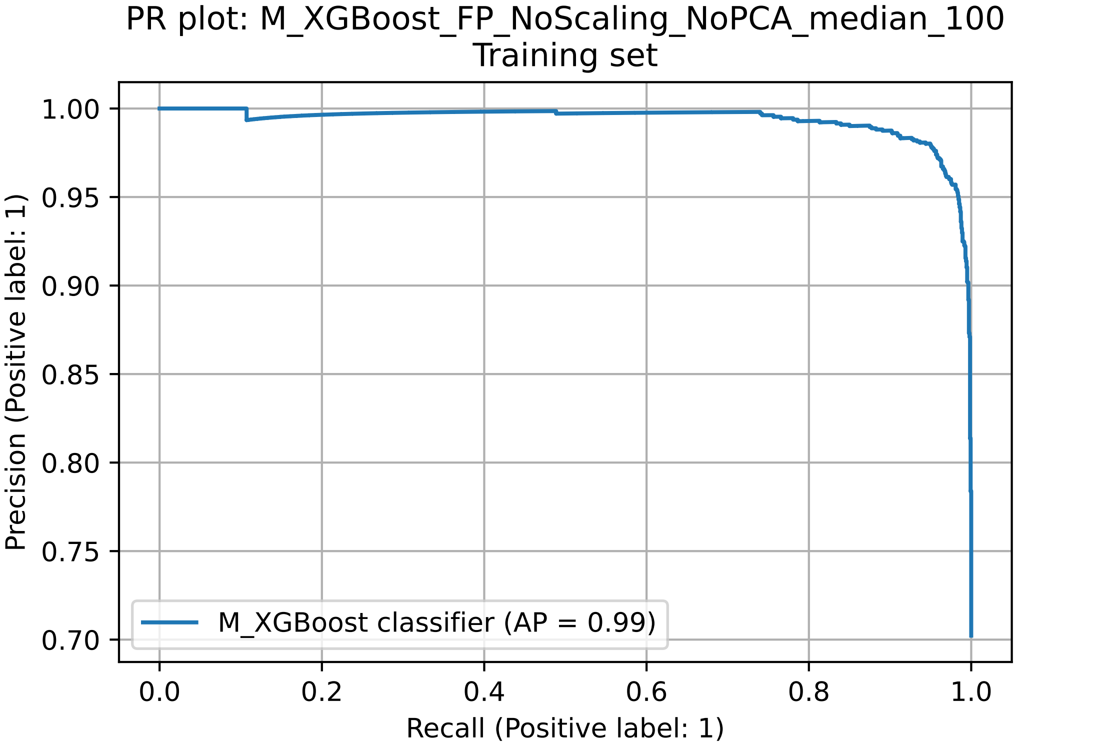

PR plot: M_XGBoost_FP_NoScaling_NoPCA_median_100
Training set
Precision (Positive label: 1)
Recall (Positive label: 1)
1.00
0.95
0.90
0.85
0.80
0.75
0.70
0.0
0.2
0.4
0.6
0.8
1.0
M_XGBoost classifier (AP = 0.99)

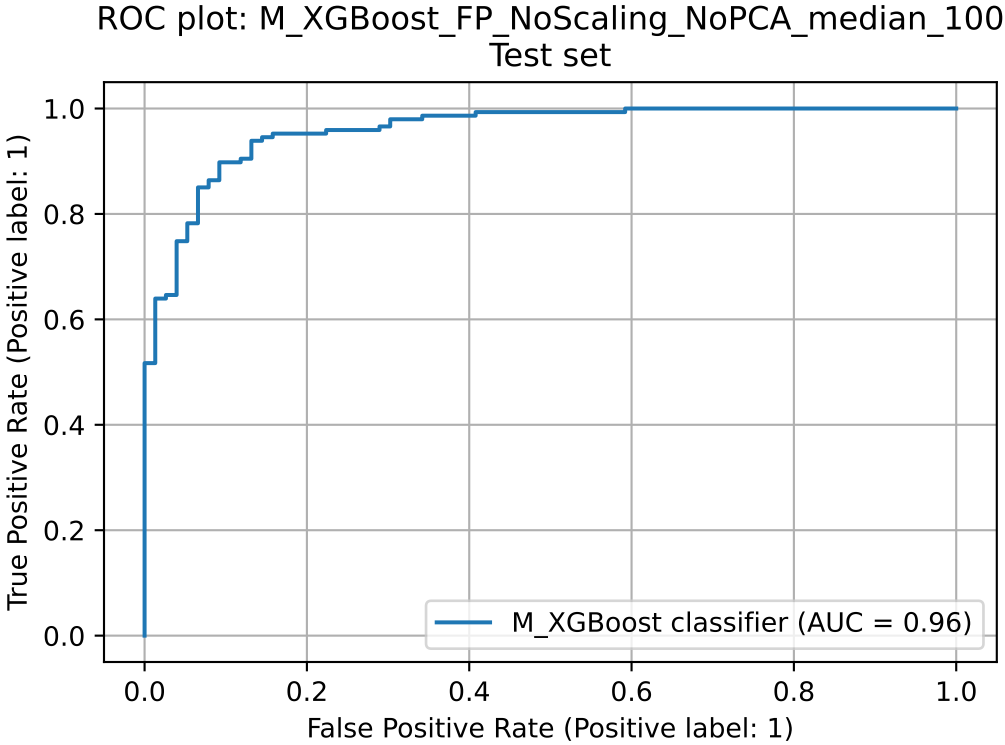
ROC plot: M_XGBoost_FP_NoScaling_NoPCA_median_100
Test set
True Positive Rate (Positive label: 1)
False Positive Rate (Positive label: 1)
0.0
0.2
0.4
0.6
0.8
1.0
M_XGBoost classifier (AUC = 0.96)

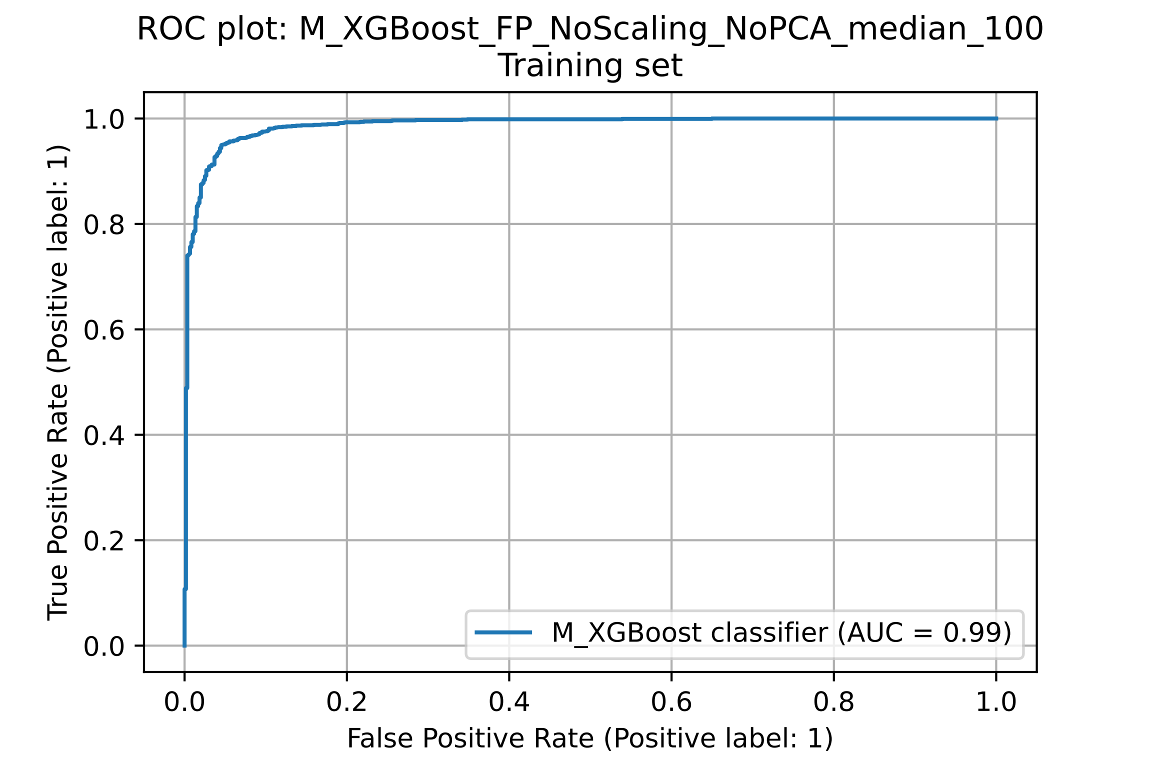
ROC plot: M_XGBoost_FP_NoScaling_NoPCA_median_100
Training set
True Positive Rate (Positive label: 1)
False Positive Rate (Positive label: 1)
0.0
0.2
0.4
0.6
0.8
1.0
M_XGBoost classifier (AUC = 0.99)

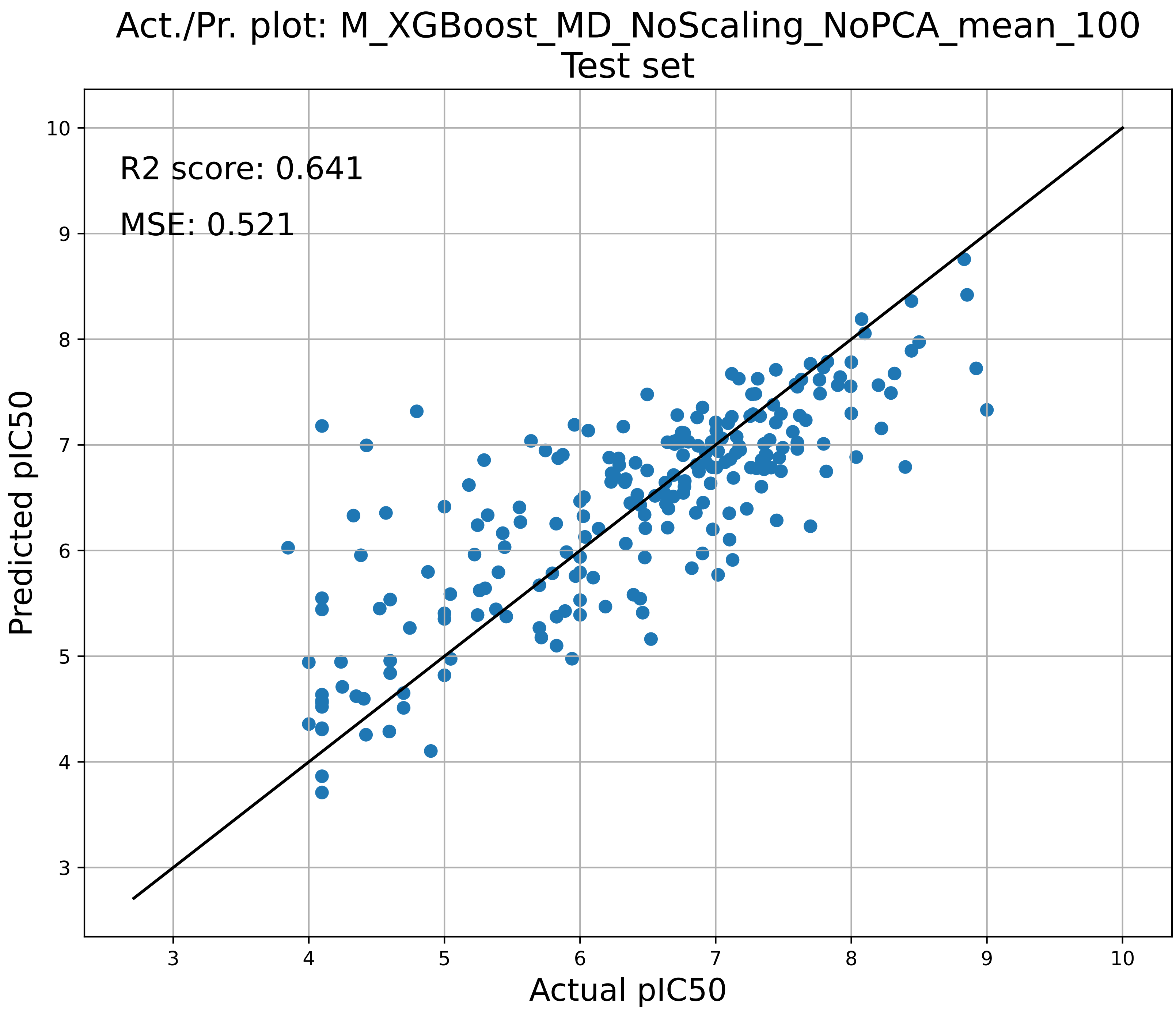

Act./Pr. plot: M_XGBoost_MD_NoScaling_NoPCA_mean_100
Test set
R2 score: 0.641
MSE: 0.521
Predicted pIC50
Actual pIC50
3
4
5
6
7
8
9
10

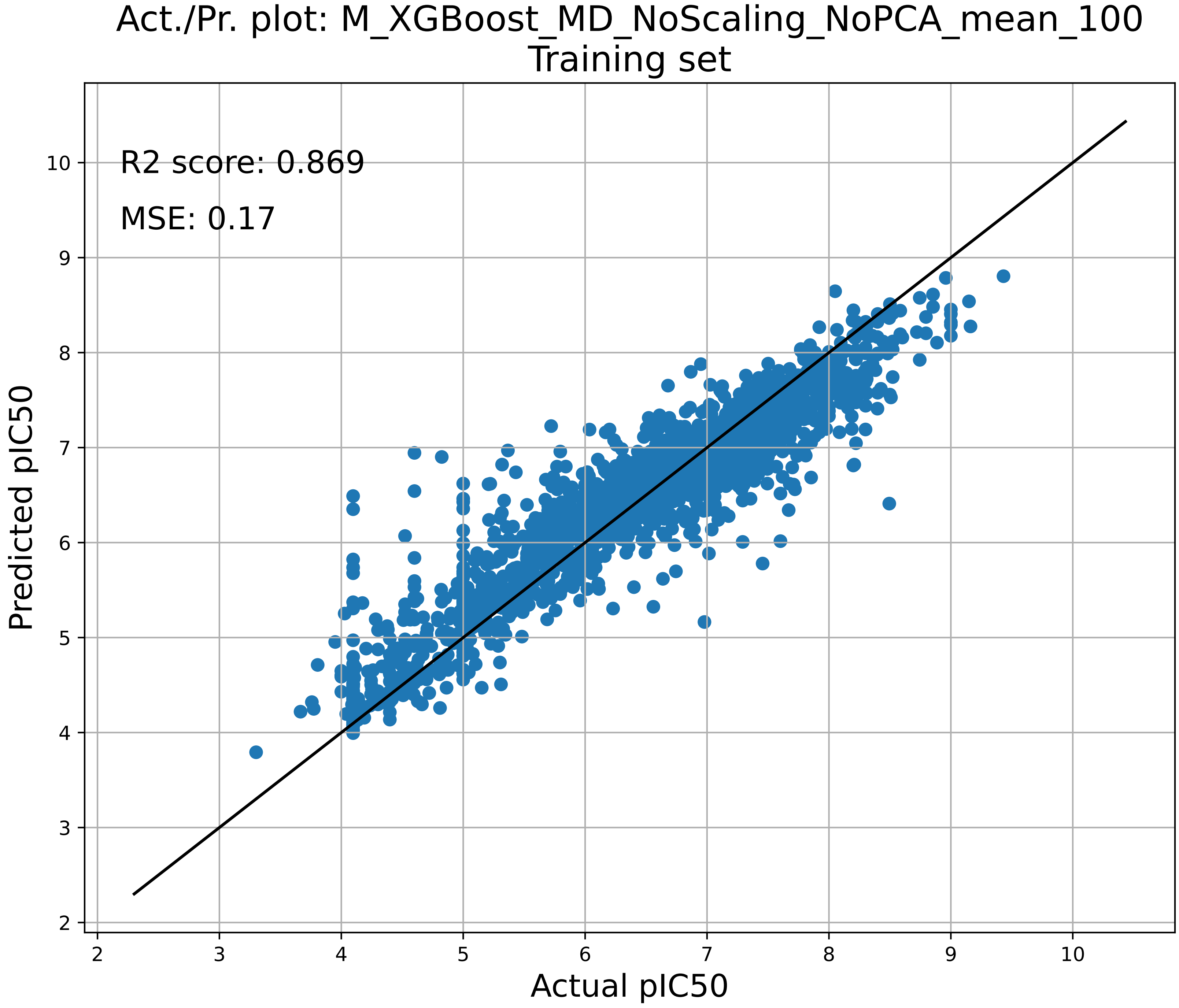

Act./Pr. plot: M_XGBoost_MD_NoScaling_NoPCA_mean_100
Training set
R2 score: 0.869
MSE: 0.17
Predicted pIC50
Actual pIC50
2
3
4
5
6
7
8
9
10

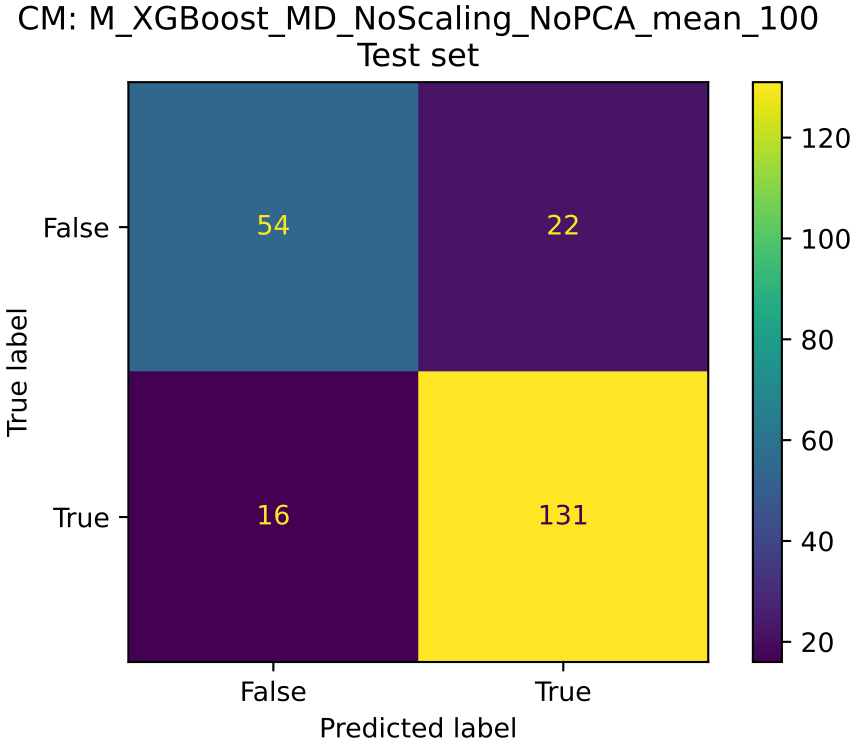
CM: M_XGBoost_MD_NoScaling_NoPCA_mean_100
Test set
True label
Predicted label
False
True
False
True
54
22
16
131
120
100
80
60
40
20

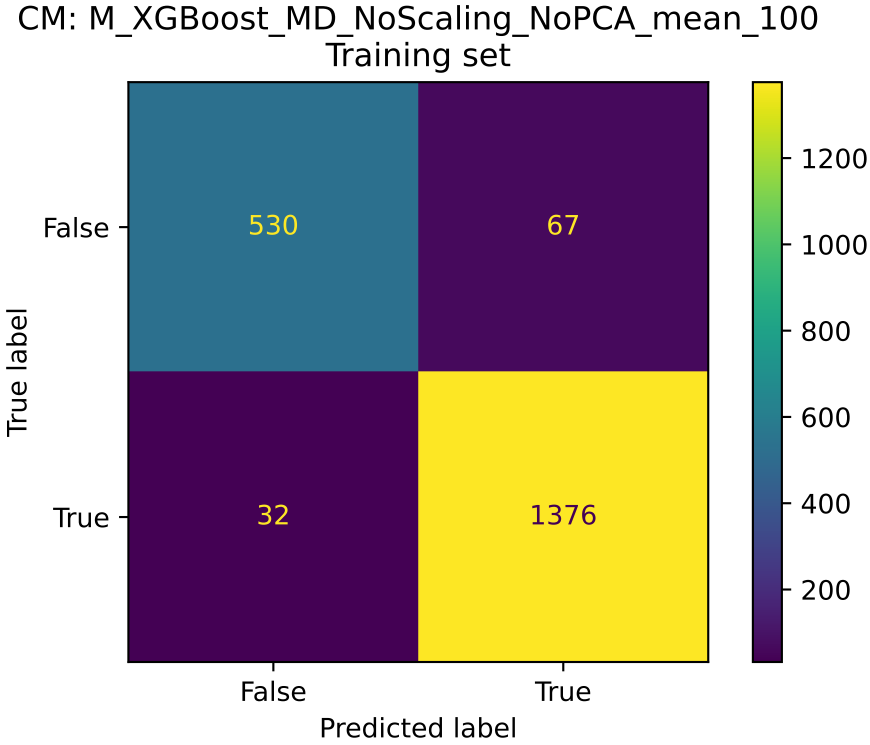
CM: M_XGBoost_MD_NoScaling_NoPCA_mean_100
Training set
False
True
530
67
32
1376
True label
Predicted label
False
True
1200
1000
800
600
400
200

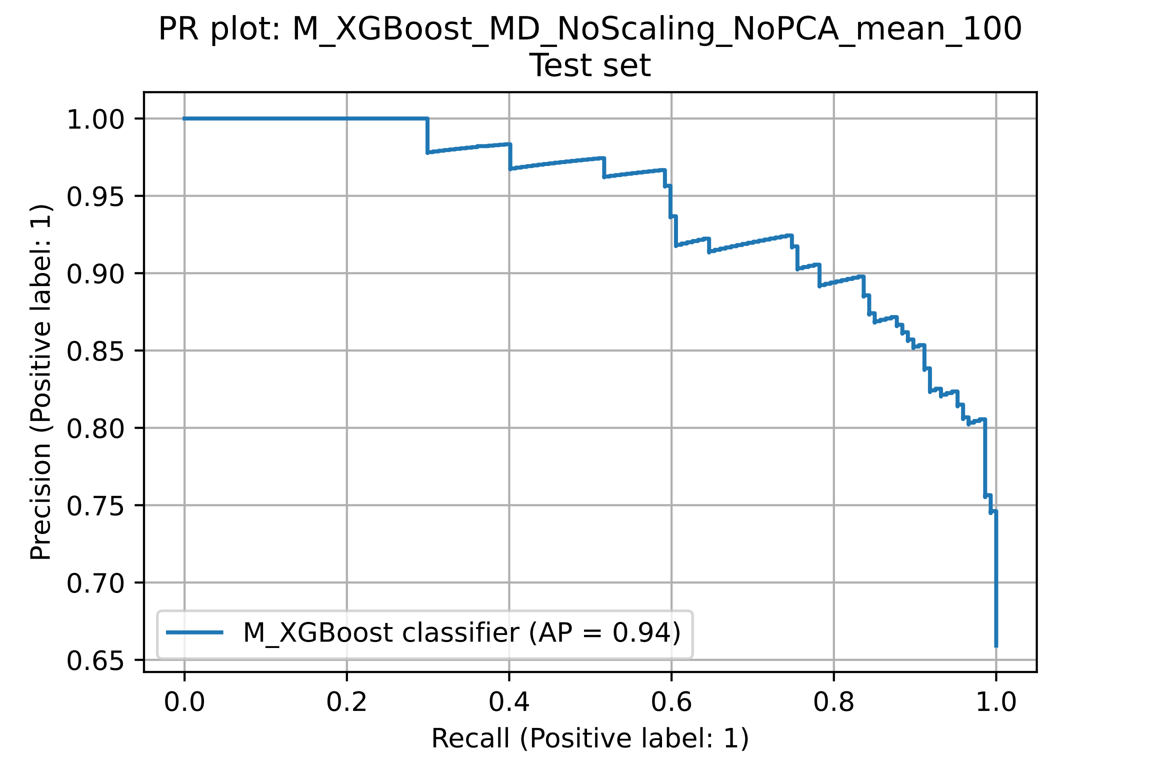
PR plot: M_XGBoost_MD_NoScaling_NoPCA_mean_100
Test set
Precision (Positive label: 1)
1.00
0.95
0.90
0.85
0.80
0.75
0.70
0.65
Recall (Positive label: 1)
0.0
0.2
0.4
0.6
0.8
1.0
M_XGBoost classifier (AP = 0.94)

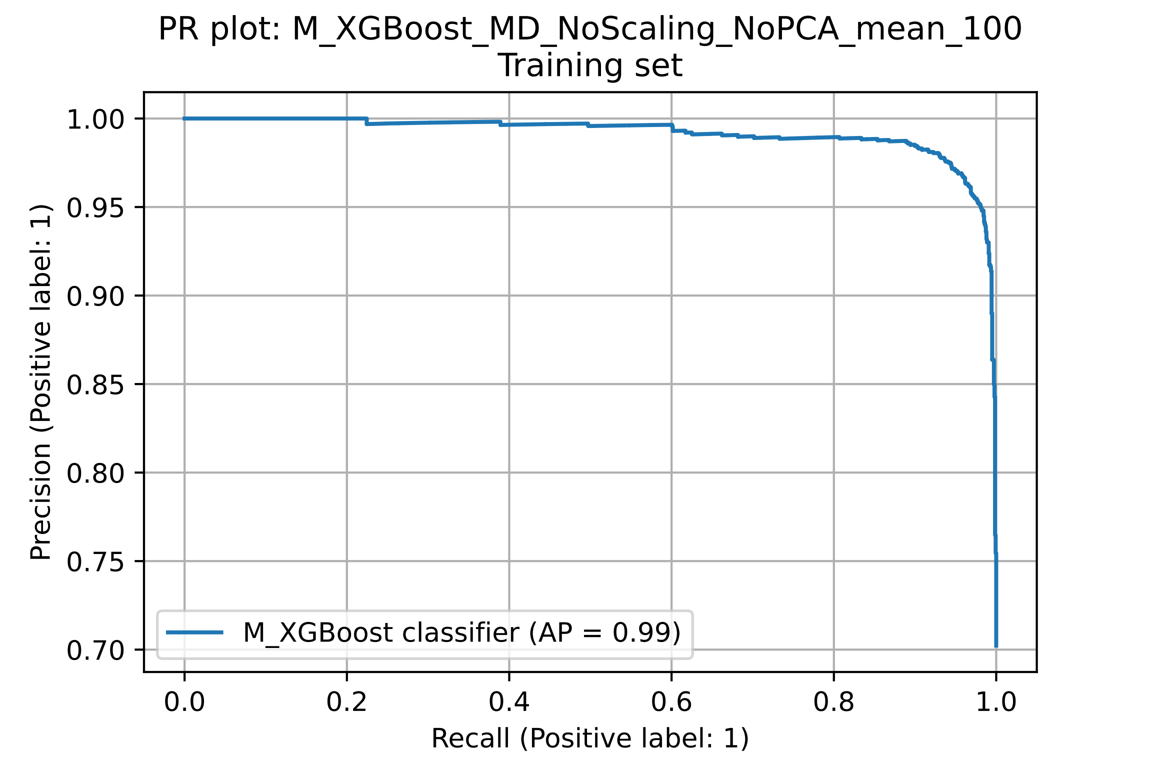
PR plot: M_XGBoost_MD_NoScaling_NoPCA_mean_100
Training set
1.00
0.95
0.90
0.85
0.80
0.75
0.70
Precision (Positive label: 1)
0.0
0.2
0.4
0.6
0.8
1.0
Recall (Positive label: 1)
M_XGBoost classifier (AP = 0.99)

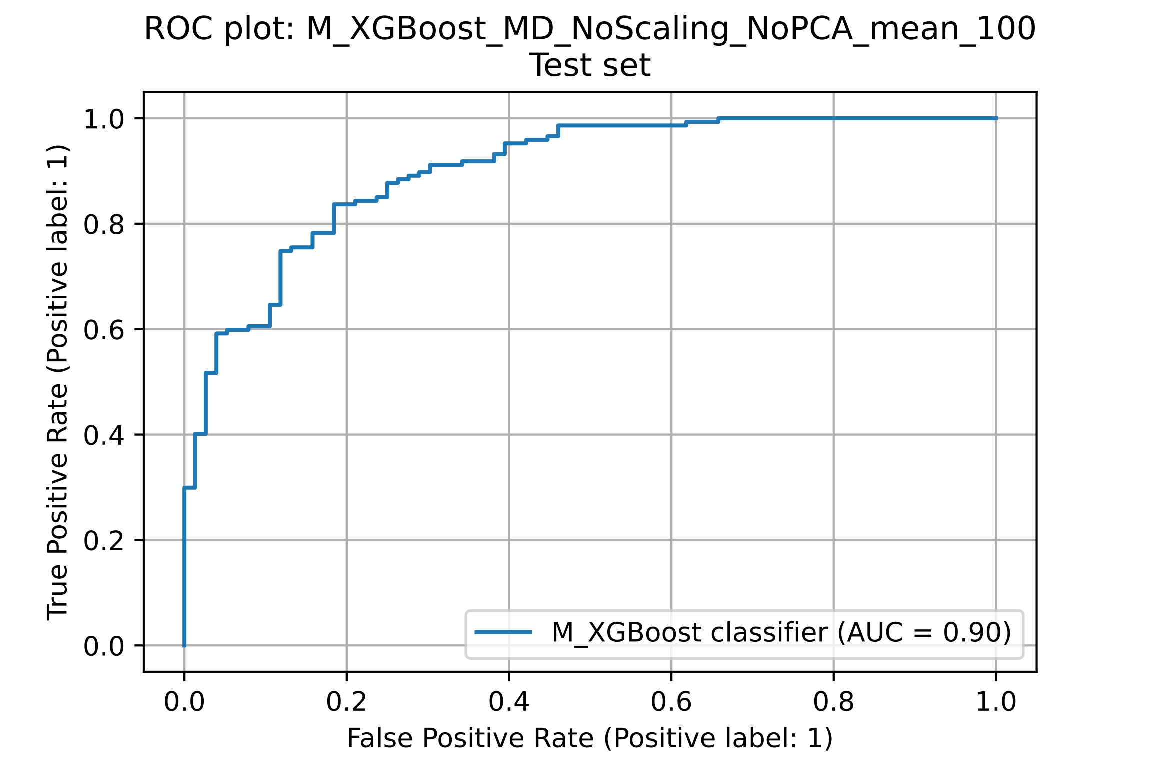
ROC plot: M_XGBoost_MD_NoScaling_NoPCA_mean_100
Test set
True Positive Rate (Positive label: 1)
False Positive Rate (Positive label: 1)
0.0
0.2
0.4
0.6
0.8
1.0
M_XGBoost classifier (AUC = 0.90)

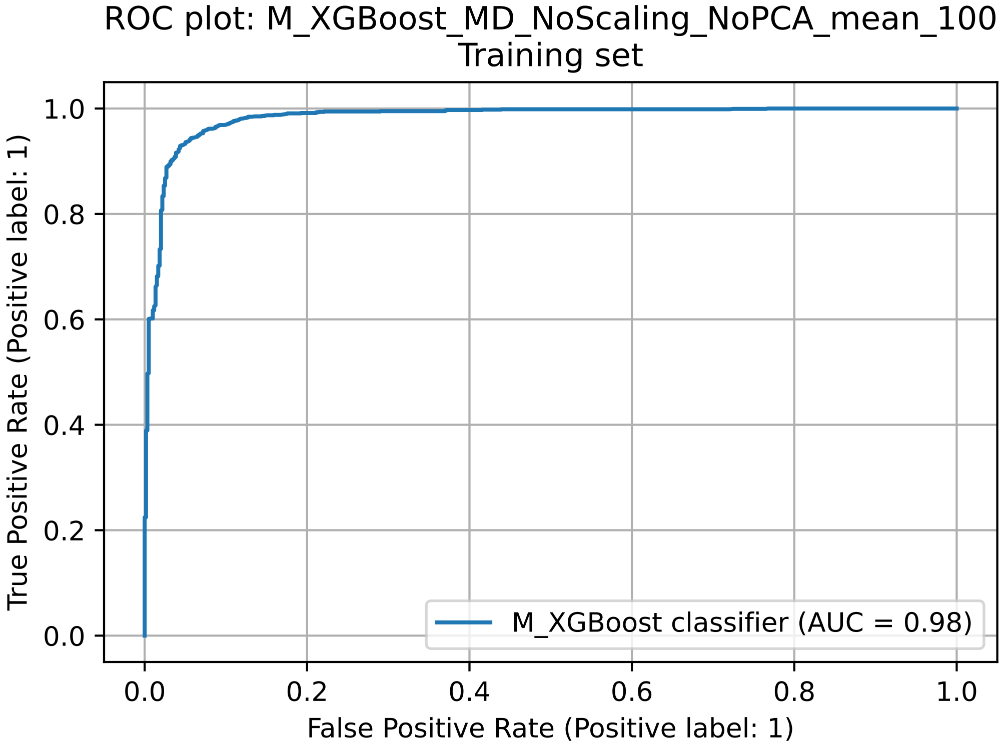

ROC plot: M_XGBoost_MD_NoScaling_NoPCA_mean_100
Training set
True Positive Rate (Positive label: 1)
False Positive Rate (Positive label: 1)
0.0
0.2
0.4
0.6
0.8
1.0
M_XGBoost classifier (AUC = 0.98)

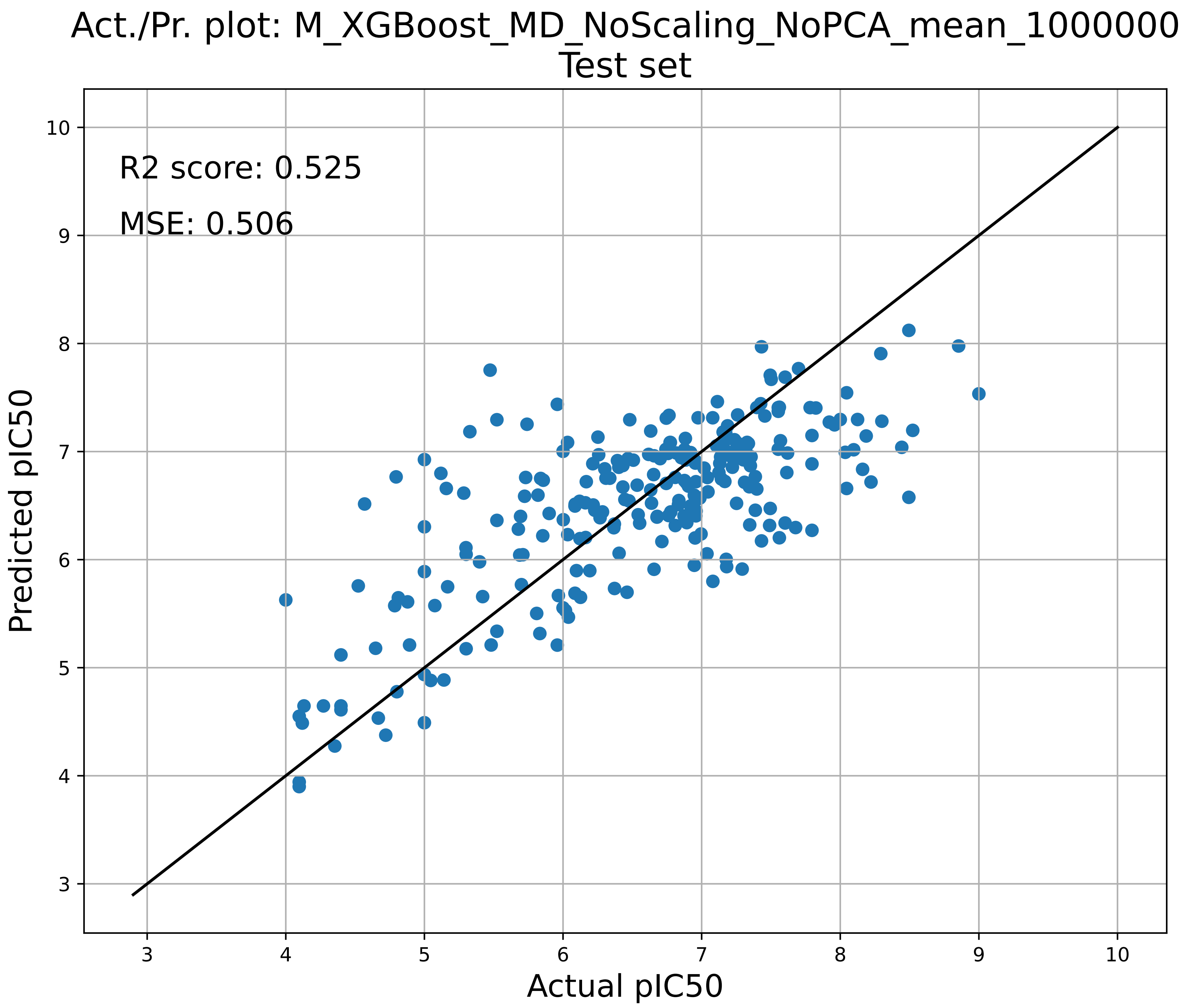
Act./Pr. plot: M_XGBoost_MD_NoScaling_NoPCA_mean_1000000
Test set
R2 score: 0.525
MSE: 0.506
Predicted pIC50
Actual pIC50
3
4
5
6
7
8
9
10
3
4
5
6
7
8
9
10

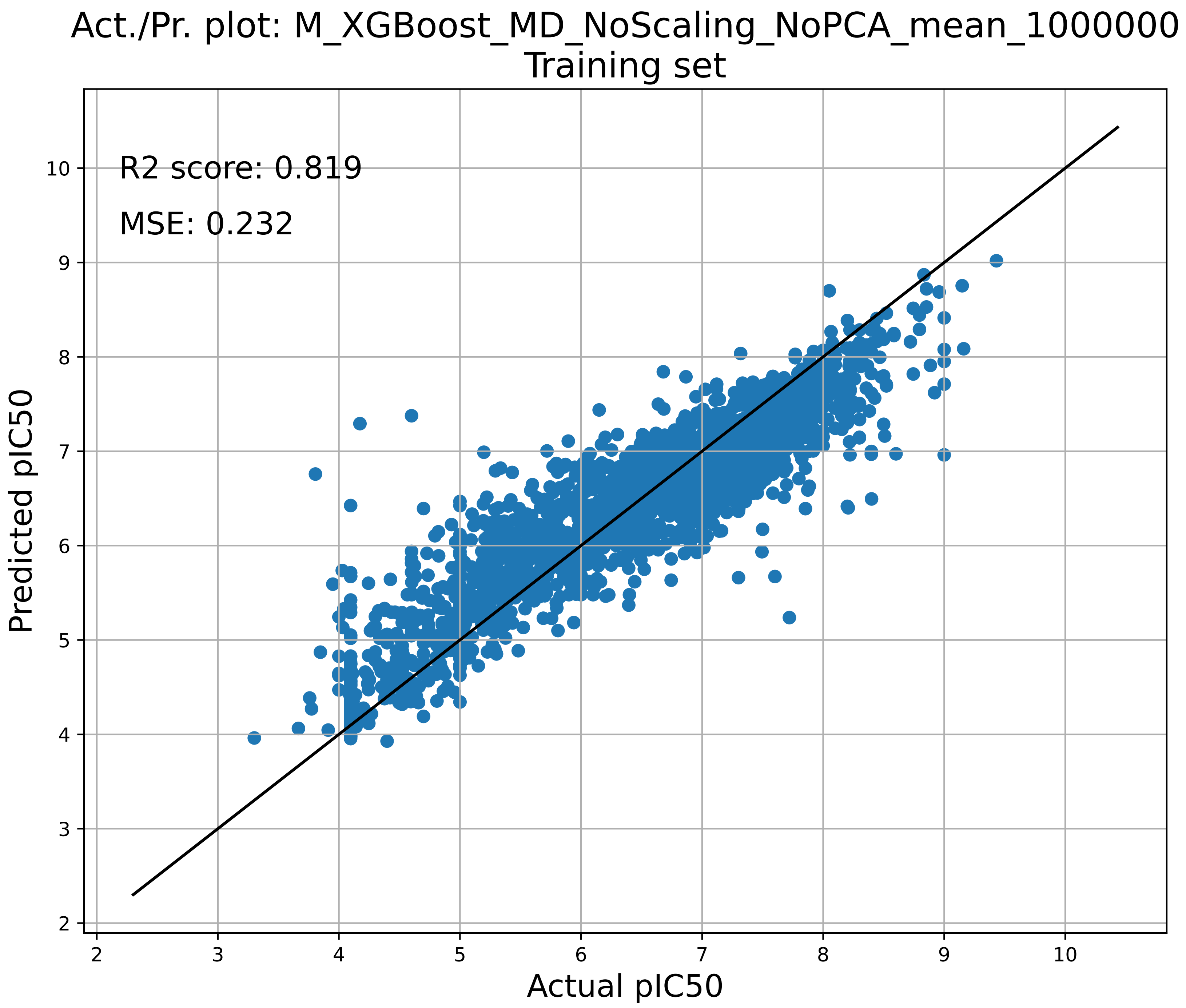
Act./Pr. plot: M_XGBoost_MD_NoScaling_NoPCA_mean_1000000
Training set
R2 score: 0.819
MSE: 0.232
Predicted pIC50
Actual pIC50
2
3
4
5
6
7
8
9
10

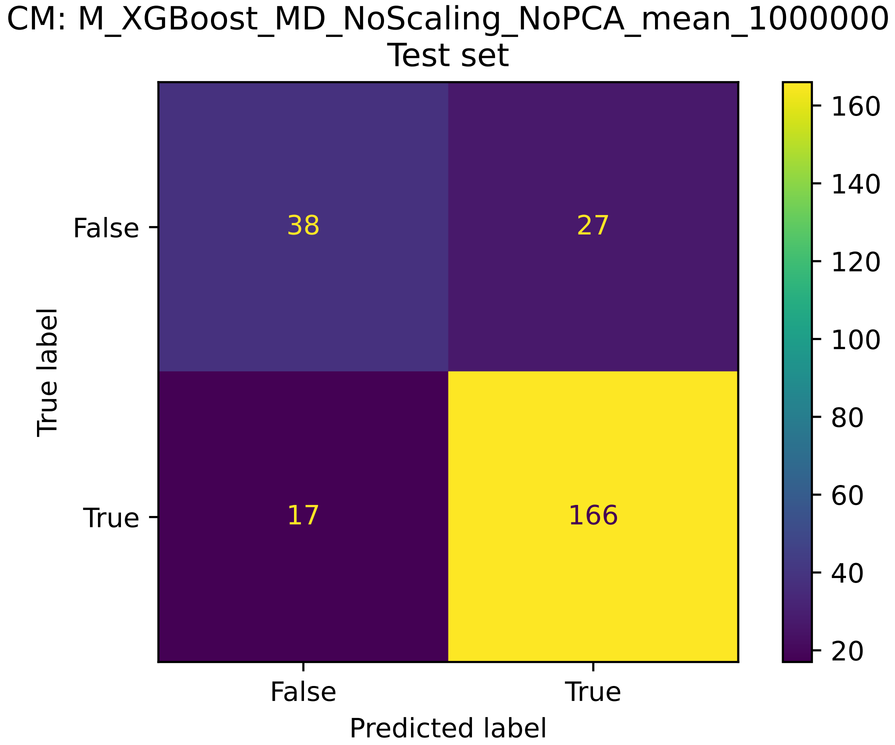

CM: M_XGBoost_MD_NoScaling_NoPCA_mean_1000000
Test set
True label
False
True
38
27
17
166
Predicted label
False
True
160
140
120
100
80
60
40
20

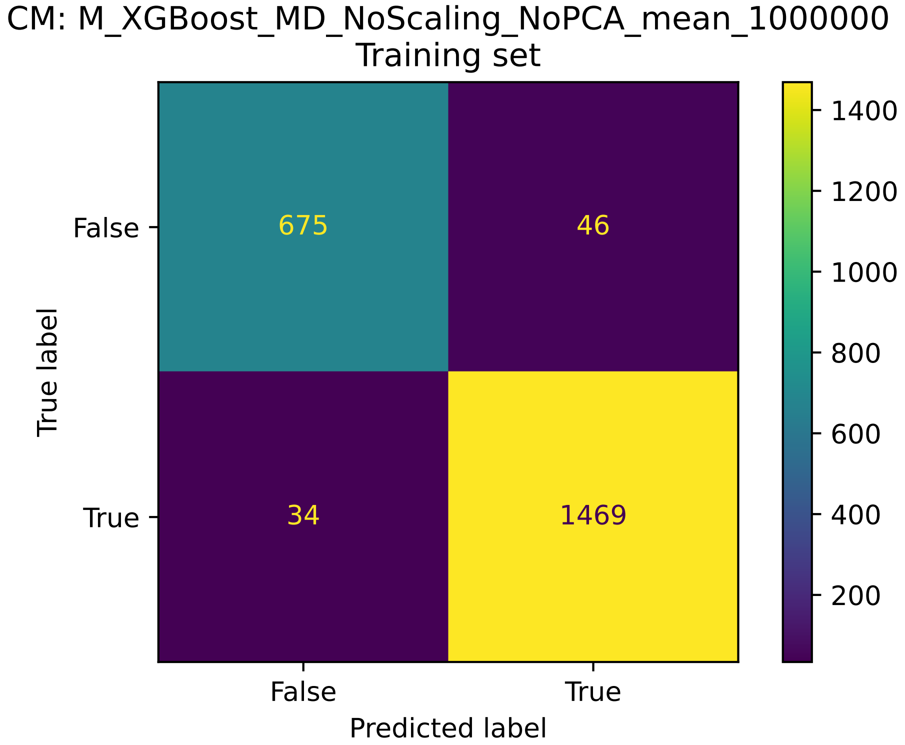
CM: M_XGBoost_MD_NoScaling_NoPCA_mean_1000000
Training set
675
46
34
1469
False
True
True label
False
True
Predicted label
1400
1200
1000
800
600
400
200

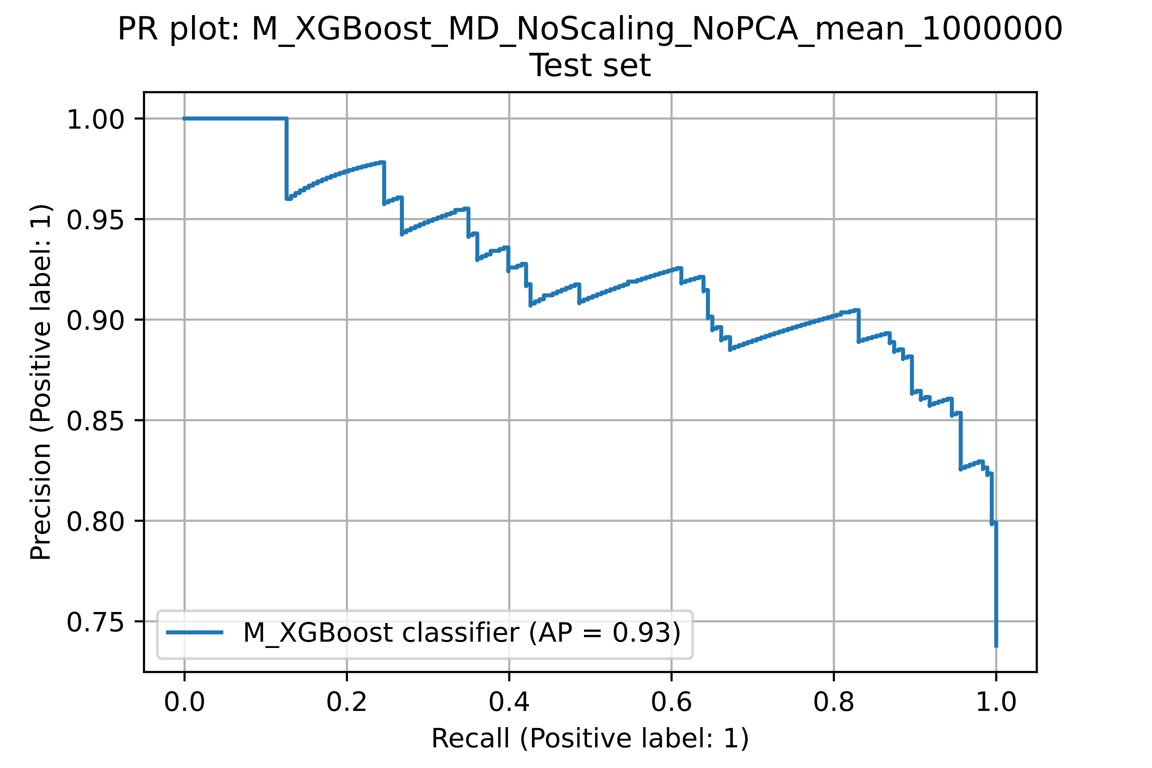
PR plot: M_XGBoost_MD_NoScaling_NoPCA_mean_1000000
Test set
Precision (Positive label: 1)
Recall (Positive label: 1)
1.00
0.95
0.90
0.85
0.80
0.75
0.0
0.2
0.4
0.6
0.8
1.0
M_XGBoost classifier (AP = 0.93)

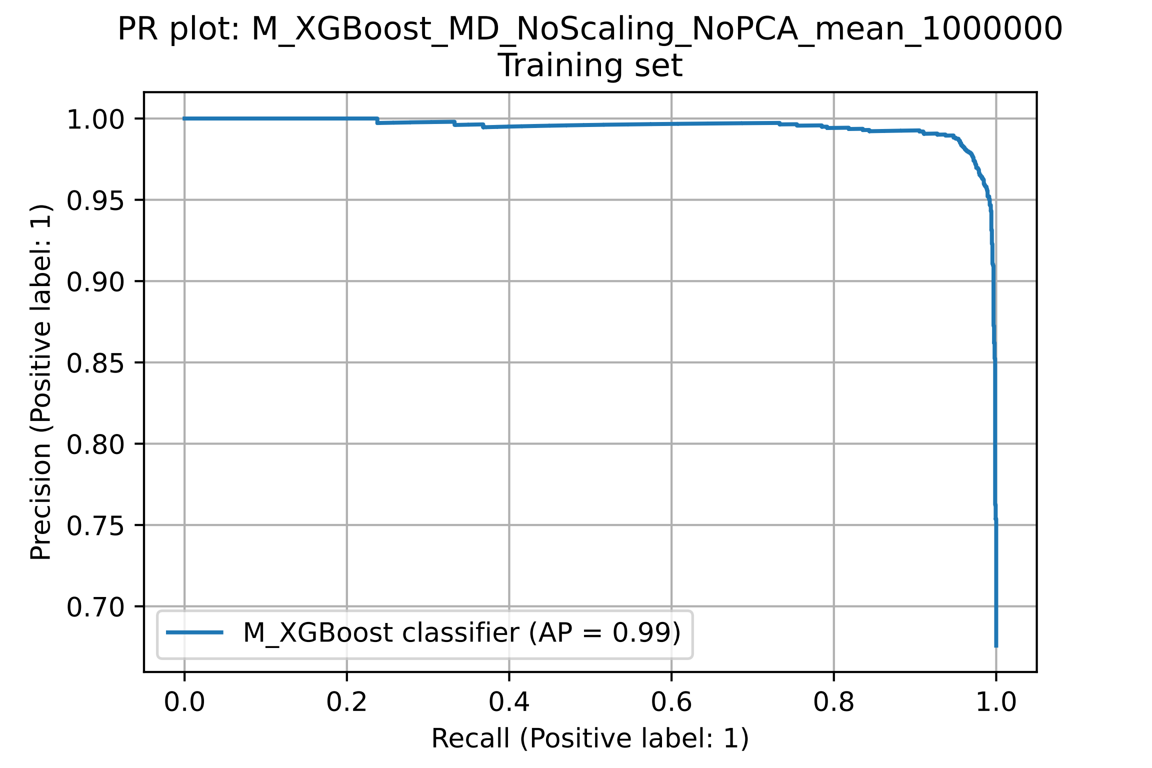
PR plot: M_XGBoost_MD_NoScaling_NoPCA_mean_1000000
Training set
Precision (Positive label: 1)
Recall (Positive label: 1)
1.00
0.95
0.90
0.85
0.80
0.75
0.70
0.0
0.2
0.4
0.6
0.8
1.0
M_XGBoost classifier (AP = 0.99)

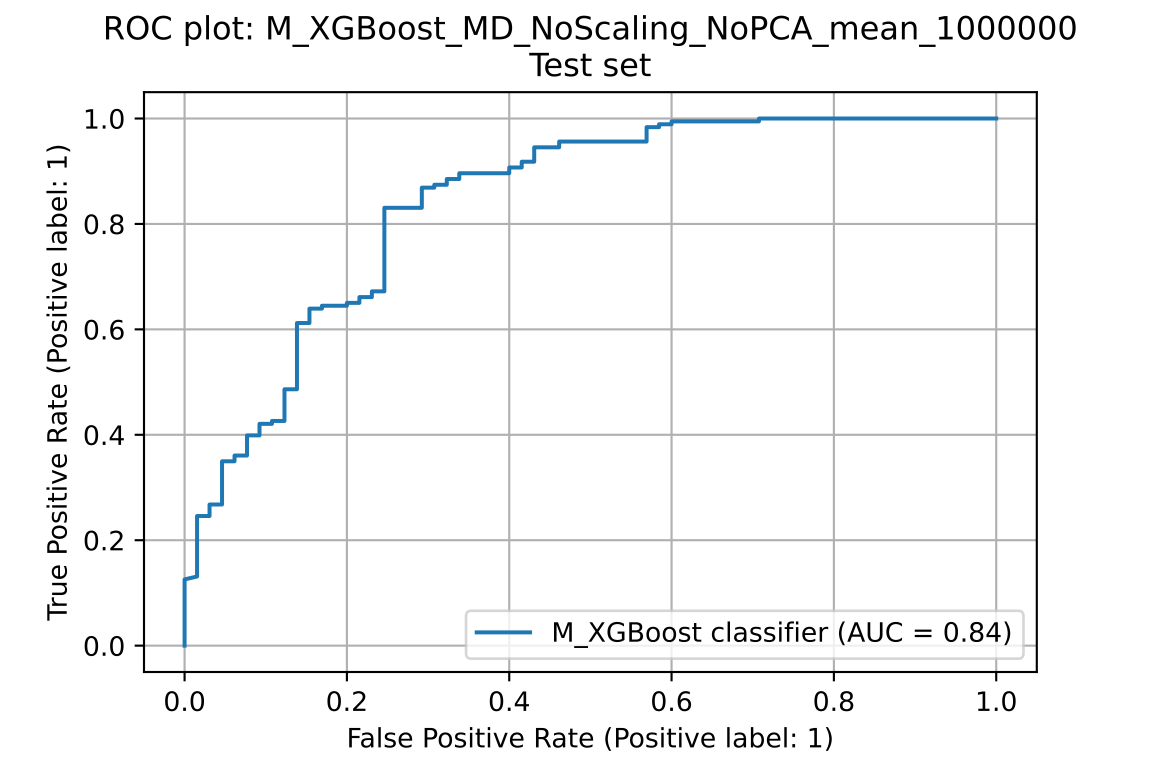
ROC plot: M_XGBoost_MD_NoScaling_NoPCA_mean_1000000
Test set
True Positive Rate (Positive label: 1)
False Positive Rate (Positive label: 1)
0.0
0.2
0.4
0.6
0.8
1.0
M_XGBoost classifier (AUC = 0.84)

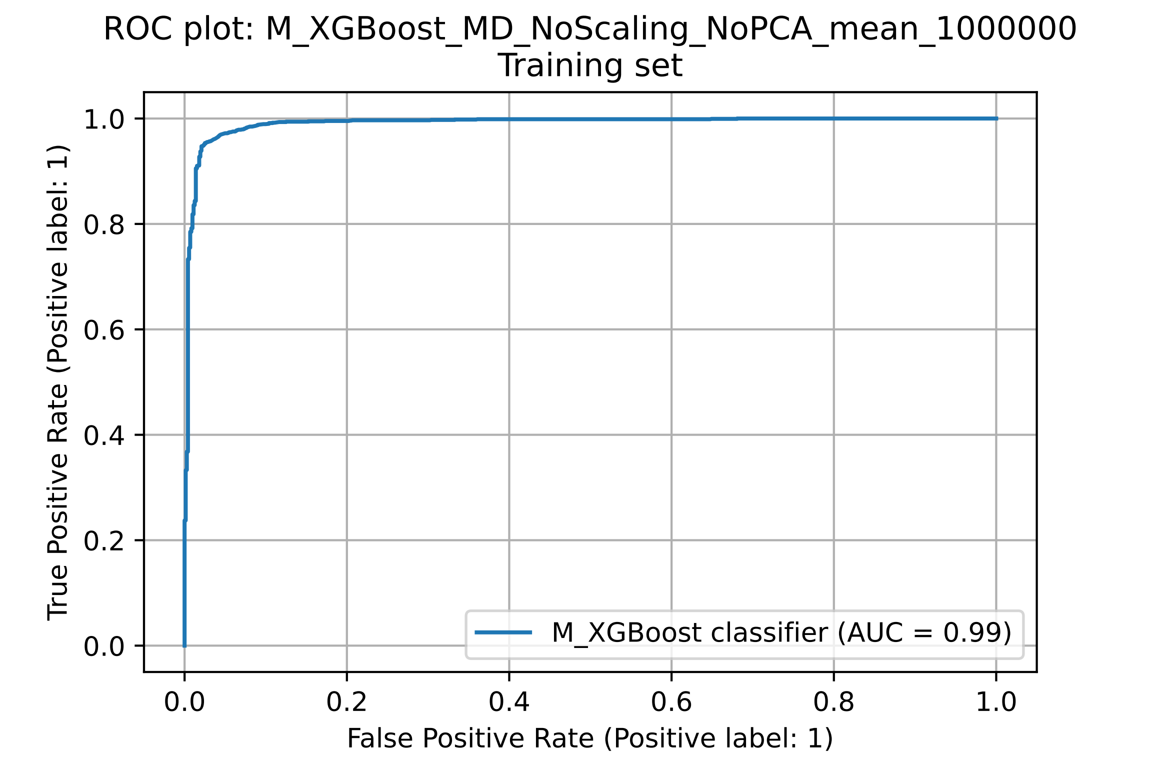
ROC plot: M_XGBoost_MD_NoScaling_NoPCA_mean_1000000
Training set
True Positive Rate (Positive label: 1)
False Positive Rate (Positive label: 1)
0.0
0.2
0.4
0.6
0.8
1.0
M_XGBoost classifier (AUC = 0.99)

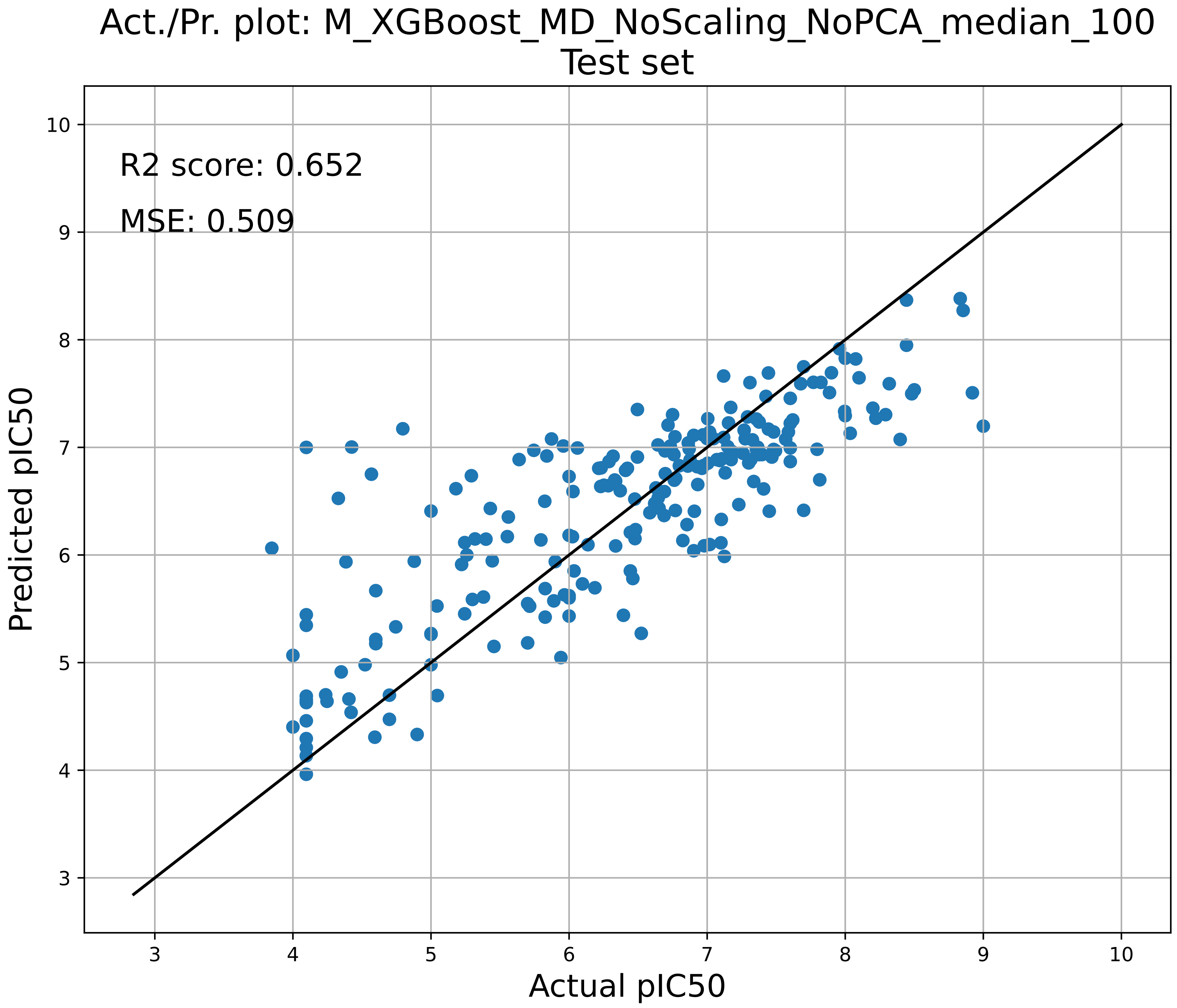
Act./Pr. plot: M_XGBoost_MD_NoScaling_NoPCA_median_100
Test set
R2 score: 0.652
MSE: 0.509
Predicted pIC50
Actual pIC50
3
4
5
6
7
8
9
10

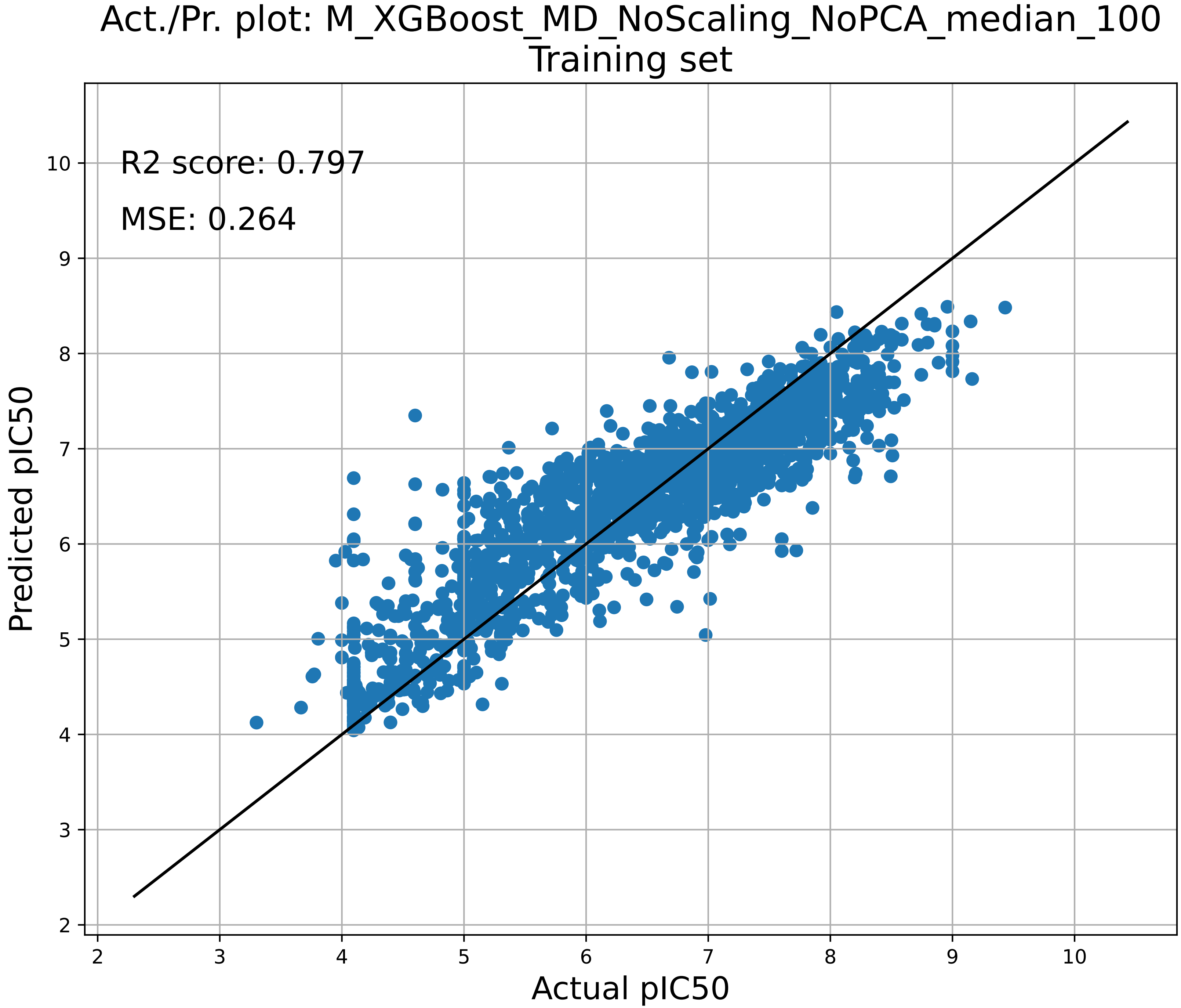
Act./Pr. plot: M_XGBoost_MD_NoScaling_NoPCA_median_100
Training set
R2 score: 0.797
MSE: 0.264
Predicted pIC50
Actual pIC50
2
3
4
5
6
7
8
9
10

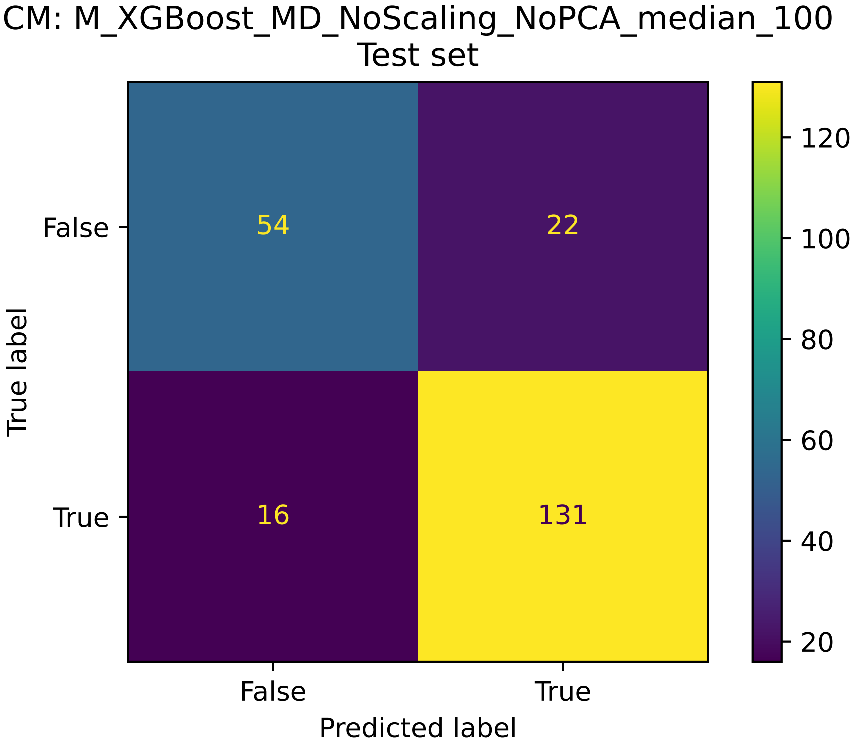
CM: M_XGBoost_MD_NoScaling_NoPCA_median_100
Test set
True label
Predicted label
False
True
False
True
54
22
16
131
20
40
60
80
100
120

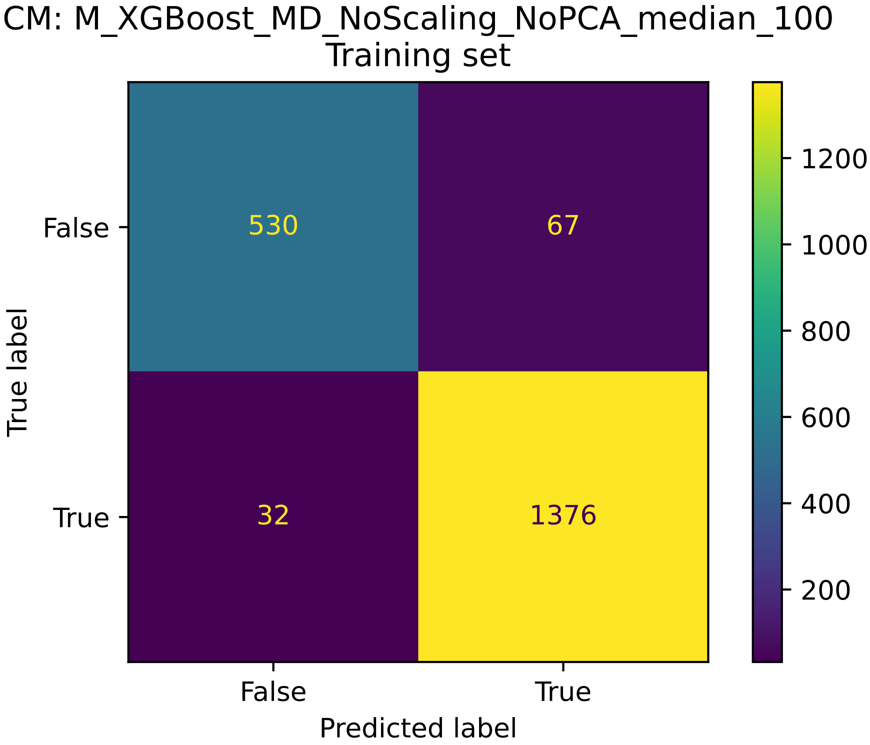
CM: M_XGBoost_MD_NoScaling_NoPCA_median_100
Training set
True label
Predicted label
False
True
False
True
530
67
32
1376
200
400
600
800
1000
1200

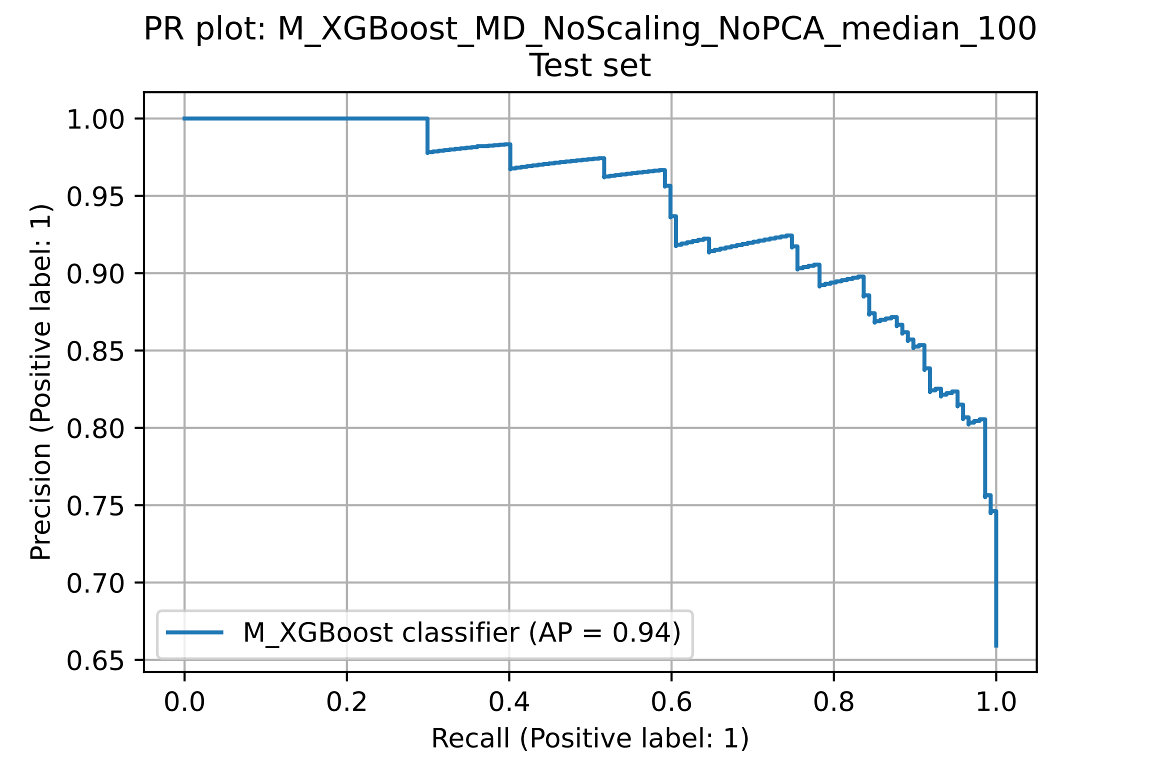
PR plot: M_XGBoost_MD_NoScaling_NoPCA_median_100
Test set
Precision (Positive label: 1)
Recall (Positive label: 1)
1.00
0.95
0.90
0.85
0.80
0.75
0.70
0.65
0.0
0.2
0.4
0.6
0.8
1.0
M_XGBoost classifier (AP = 0.94)

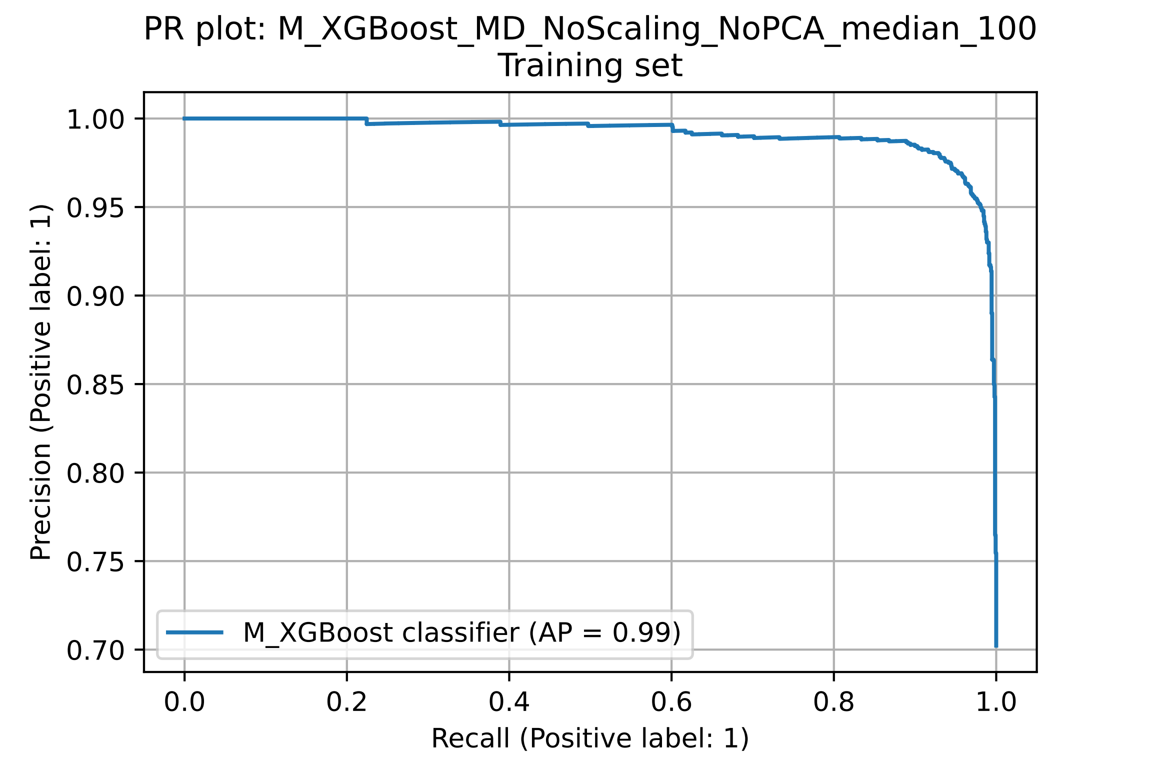
PR plot: M_XGBoost_MD_NoScaling_NoPCA_median_100
Training set
Precision (Positive label: 1)
Recall (Positive label: 1)
1.00
0.95
0.90
0.85
0.80
0.75
0.70
0.0
0.2
0.4
0.6
0.8
1.0
M_XGBoost classifier (AP = 0.99)

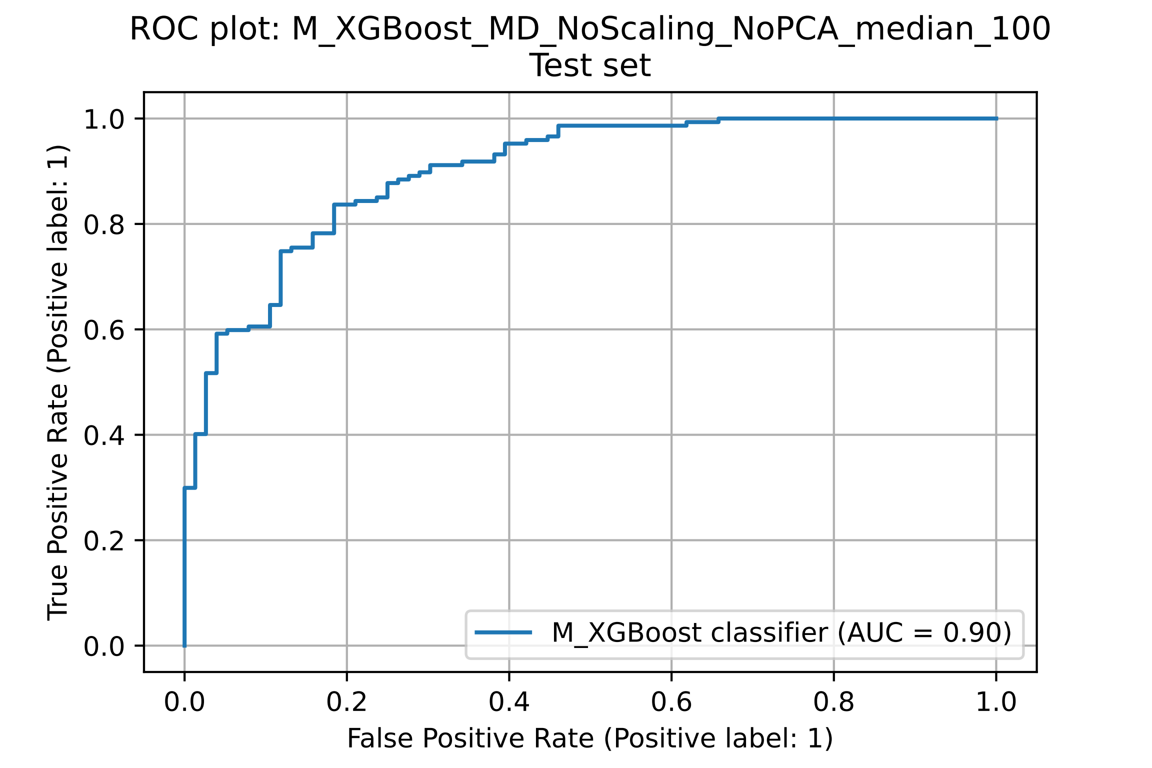
ROC plot: M_XGBoost_MD_NoScaling_NoPCA_median_100
Test set
True Positive Rate (Positive label: 1)
False Positive Rate (Positive label: 1)
0.0
0.2
0.4
0.6
0.8
1.0
M_XGBoost classifier (AUC = 0.90)

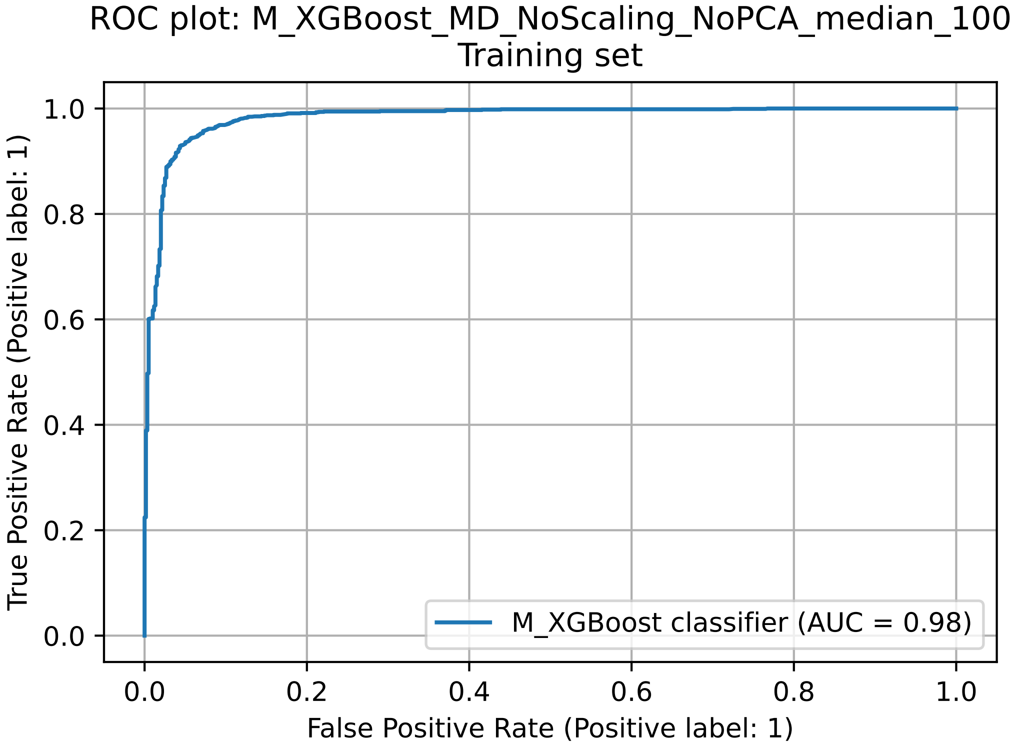
ROC plot: M_XGBoost_MD_NoScaling_NoPCA_median_100
Training set
True Positive Rate (Positive label: 1)
False Positive Rate (Positive label: 1)
0.0
0.2
0.4
0.6
0.8
1.0
M_XGBoost classifier (AUC = 0.98)
